# Standardized Approach for Diversification of Complex Small Molecules via Aryl Thianthrenium Salts

**DOI:** 10.1021/jacs.4c14391

**Published:** 2025-01-22

**Authors:** Dilgam Ahmadli, Sven Müller, Yuanhao Xie, Tomas Smejkal, Simon Jaeckh, Andrei V. Iosub, Simon R. Williams, Tobias Ritter

**Affiliations:** † Max-Planck-Institut für Kohlenforschung, Kaiser-Wilhelm-Platz 1, Mülheim an der Ruhr 45470, Germany; ‡ Institute of Organic Chemistry, RWTH Aachen University, Landoltweg 1, Aachen 152074, Germany; § Research Chemistry, Syngenta Crop Protection AG, Schaffhauserstrasse 101, Stein, AG 4332, Switzerland

## Abstract

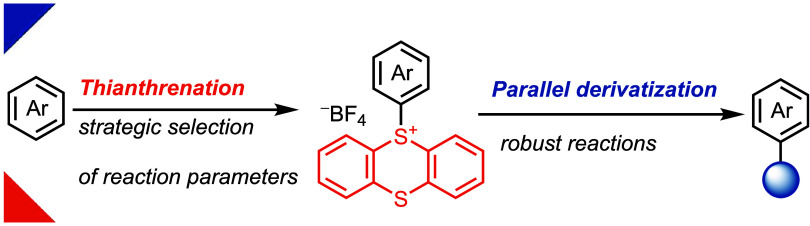

Thianthrenation is a useful strategy for the late-stage diversification of complex small molecules owing to the positional selectivity and the synthetic versatility of thianthrenium salts as electrophilic linchpins. However, substrate-dependent identification of suitable reaction conditions for thianthrenation can be difficult. Reported reaction conditions for the functionalization of thianthrenium salts vary significantly and, in some instances, lack robustness and practicality. Herein, we report a generalized approach for the preparation of thianthrenium salts and two reaction manifolds for practical, robust, and parallel diversification of thianthrenium salts.

## Introduction

Methods for diversification of C–H bonds in complex small molecules provide access to a broad chemical space, bypassing the need to synthesize each analogue de novo.^1−3^ Selective functionalization of C–H bonds in small molecules has been a longstanding challenge due to the presence of multiple C–H bonds and functional groups.^4^ In 2019, our research group introduced thianthrenation as a versatile method for the regioselective functionalization of aromatic C–H bonds in small molecules.^5^ Thianthrenation has grown to be a valuable tool, also because follow-on transformations can be efficient, sometimes outperforming analogous reactions with aryl halides and other pseudohalides.^5−7^ The combination of synthetic flexibility of aryl thianthrenium salts with high-throughput experimentation (HTE) could provide fast access to libraries of small molecules. However, integration of the thianthrenation methodology into automated synthesis systems has been hindered by two significant challenges. First, previously reported conditions offer little guidance for selecting the reaction parameters for new substrates. Additionally, derivatization reactions of aryl thianthrenium salts often lack practical simplicity and robustness for applications in automated settings. The fundamental novelty of this paper lies in the development of a late-stage functionalization platform for strategic selection of reaction parameters for thianthrenation and new reaction conditions for parallel diversification of aryl thianthrenium salts. We anticipate that our platform will find applications in pharmaceutical and agrochemical late-stage chemical space expansion.

In the first part of the study, we evaluate the parameters influencing the efficacy of thianthrenation. We developed a workflow that is easy to follow and demonstrate its applicability across a diverse range of substrates. The approach for selecting the most promising out of a set of three reaction conditions for thianthrenation is based on the electronic structure, functional groups, and connectivity pattern of the arene substrate. Our findings simplify the selection of reaction parameters for thianthrenation, enhance the functional group compatibility, and also provide fundamental insights for troubleshooting. To enable reliable diversification of aryl thianthrenium salts and potential applications to high-throughput experimentation (HTE), we developed a large variety of transformations from arylthianthrenium salts that employ the same reaction parameters. Previously, each follow-on transformation required its individually optimized conditions from different thianthrenium analogs and was thereby not useful for parallel diversification. The new reactions developed here for diversification are more robust and practical than what is currently reported and also include new reactions such as transformations to introduce cyanide, the difluoromethyl group, as well as small alkyl and carbonyl substituents from a single thianthrenium salt. We have paid special attention to only select and develop those reactions that are robust and have a high likelihood of providing the desired product when deviating from the demonstrated substrate scope shown here, as long as the general requirements are conserved. For example, we report a new C–N cross-coupling reaction that can engage primary and secondary amines, anilines, amides, and carbamates under the same reaction conditions, which differentiates it from the reported C–N cross-coupling of aryl thianthrenium salts. The reactions are grouped into only two reaction manifolds to allow for parallel diversification, which is currently not possible with established thianthrene chemistry. Based on the general reaction conditions developed in this work, we showcase the diversification of thianthrenium salts in an automated setting.

Just as C–H functionalization reactions to directly introduce a desired substituent, such as a chloride or a methoxy group, are valuable, thianthrenation is useful because it can selectively functionalize small molecules for a variety of follow-on transformations.^6^ Despite being a two-step process, the comparably reliable second step provides for a simple means to quickly access diverse analogs of an already functionally complex starting material. Thianthrenation proceeds with *para/ortho* and *para/meta* selectivity often exceeding 100:1 ratios for monosubstituted arenes with mesomerically and inductively electron-releasing substituents, such as chloride, methyl, etc.^8^ The resulting thianthrenium salts participate in both conventional cross-coupling and photoredox catalysis processes, as well as transformations that are currently out of scope for other (pseudo)halides.^6,9^ Aryl thianthrenium salts can react via fast oxidative addition to low-valent palladium and nickel catalysts.^10−12^ Owing to the positive charge and stereoelectronic arrangement of the thianthrenium group, they also show reactivity in single electron transfer (SET) reactions such as copper-mediated photoredox processes.^7^ Thianthrenium salts are bench-stable solids that are easy to handle and purify.^5^

For structure–activity relationship (SAR) studies, time- and cost-efficient access to analogs of complex small molecules is valuable.^13^ High-throughput experimentation (HTE) is widely used by companies for new reaction discovery and optimization.^14^ HTE allows chemists to perform multiple reactions in parallel using minimal quantities of starting materials in a time-efficient manner. Thianthrenium salt lynchpins offer the synthetic flexibility to rapidly access structurally and functionally diverse analogs of great value to drug discovery and optimization campaigns. The combination of such synthetic plasticity with HTE would produce a powerful tool for discovery chemists. However, reported thianthrenations and transformations from arylthianthrenium salts do not provide a clear strategy for selecting reaction parameters and hamper the application of thianthrenium salts to an automated setting. Here, we introduce a late-stage functionalization (LSF) platform that consists of a comprehensive and generalized approach for the preparation and parallel diversification of thianthrenium salts.

**Figure 1:**
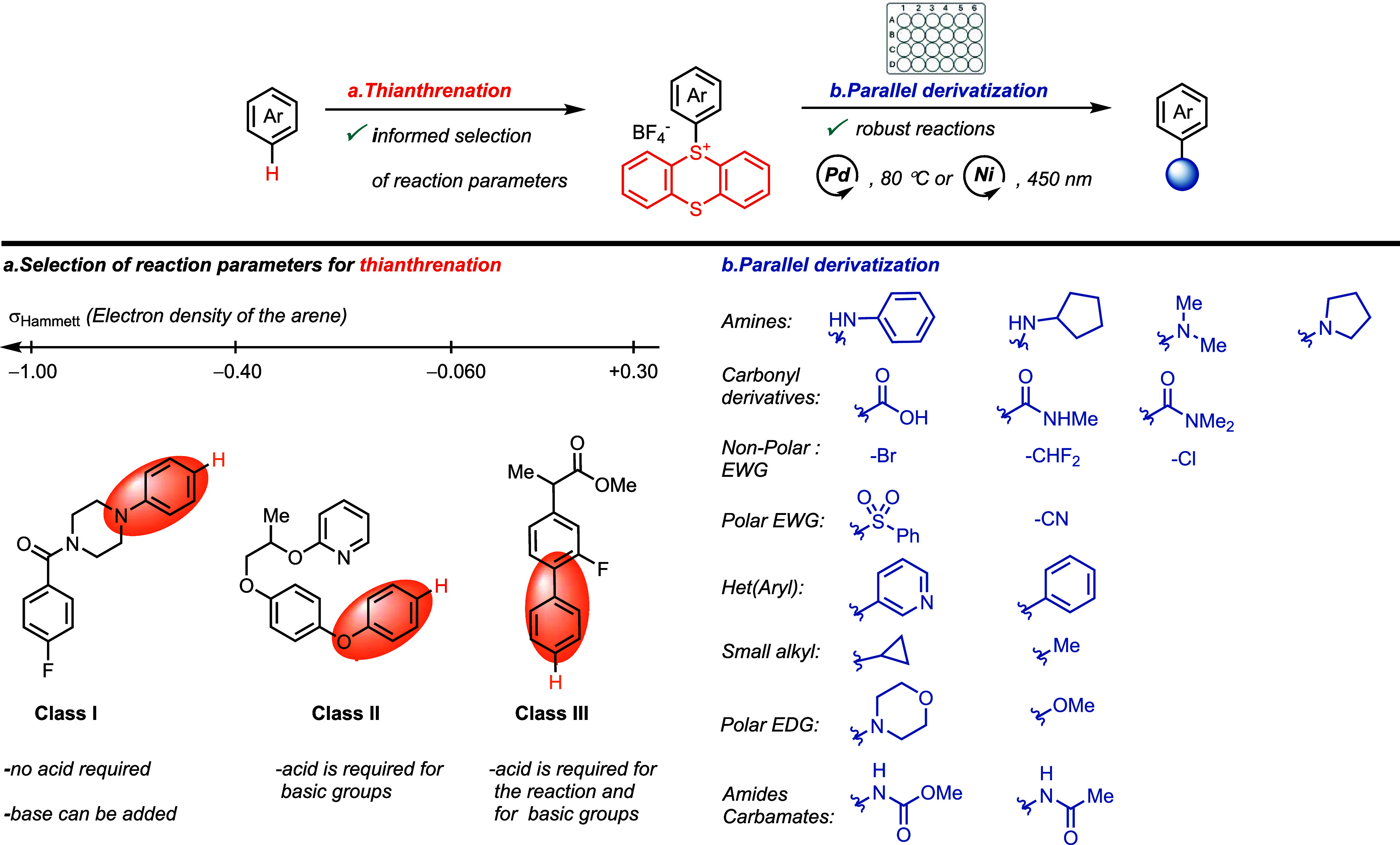
Late-stage functionalization platform for diversification of complex small molecules into valuable analogs. (a) Lynchpin installation by strategic selection of reaction conditions. (b) Unified reaction conditions for parallel diversification of aryl thianthrenium salts into medicinally relevant space. EWG: electron-withdrawing group and EDG: electron-donating group. σ_Hammett_: Sum of the Hammett sigma constant of the substituents.

We found that reaction parameters for thianthrenation can, in general, be predicted based on the electron density of the aromatic ring (Figure 1a). The applicability of this approach was tested and supported across a variety of substrates including complex examples from medicinal and agrochemistry. For the diversification of thianthrenium salts, we developed robust reactions that can be performed in parallel with a simple setup and readily accessible reagents (Figure 1b). Considering the purpose of this article, we did not focus on achieving the lowest possible catalyst loadings and ligand amount for every diversification reaction. We demonstrate that the catalyst and ligand loadings can be reduced for larger scale reactions without significant drops in yields if so desired but remain at a relatively high loading for most reactions to attempt maximum robustness. Some of the previously developed reactions are modified to yield more robust protocols and consolidated conditions for a more expeditious diversification effort.

### Thianthrenation Reaction

**Figure 2:**
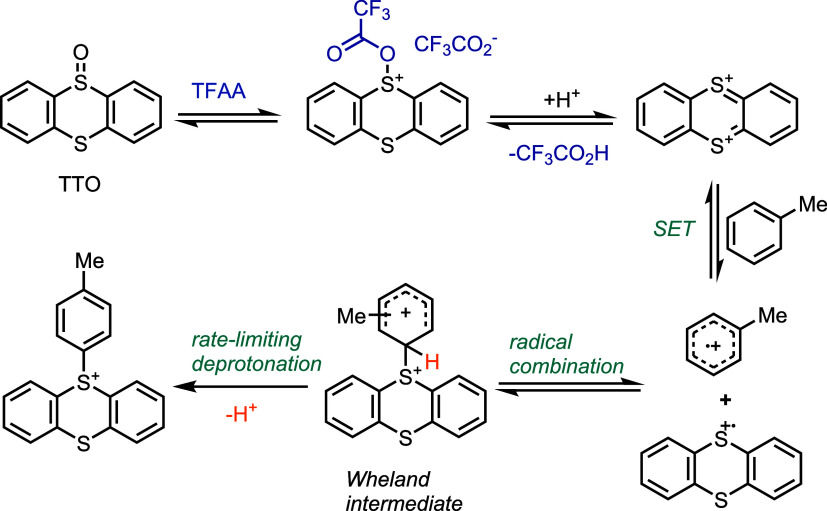
Mechanism of thianthrenation of arenes. TFAA: trifluoroacetic anhydride. SET: single electron transfer. TTO: thinathrene–S–oxide.^8^

Aryl thianthrenium (TT) salts are prepared through the reaction of an arene with thianthrene sulfoxide in the presence of an anhydride and a strong acid. The reaction proceeds via SET between the arene and the electrophilic aromatic thianthrenium dication, followed by radical combination and rate-limiting deprotonation of the Wheland intermediate.^8^ A combination of trifluoroacetic anhydride (TFAA) with a strong acid such as HBF_4_·OEt_2_ or TfOH is commonly used to generate the thianthrenium dication. The acid protonates trifluoroacetate anions and shifts the equilibrium toward the formation of the reactive thianthrenium species also by facilitating the S–O bond dissociation (Figure 2). The alternative approach to activate sulfoxides by trifluoromethanesulfonic anhydride is not as efficient for complex small molecules (see the Supporting Information, Figure S1). To simplify the selection of parameters for thianthrenation and the development of general conditions for diversification reactions, we decided to employ a single sulfoxide. We chose thianthrene–*S*–oxide (TTO) over its tetrafluoro analog for this diversification platform considering its lower cost and ease of preparation, in addition to the superior reactivity of TT salts in subsequent diversification reactions compared to that of tetrafluorothianthrenium (TFT) salts (see the Supporting Information, Figure S2). We accept a slightly smaller substrate scope with regard to electron-poor arenes as a consequence of omitting TFT in favor of simplicity and practicality. When appropriate, however, TFT represents an alternative for optimization in the case of low arene reactivity for less electron-rich arenes.

## Results and Discussion

### Selection of Reaction Parameters Based on the Electronic Structure of the Substrate

**Figure 3:**
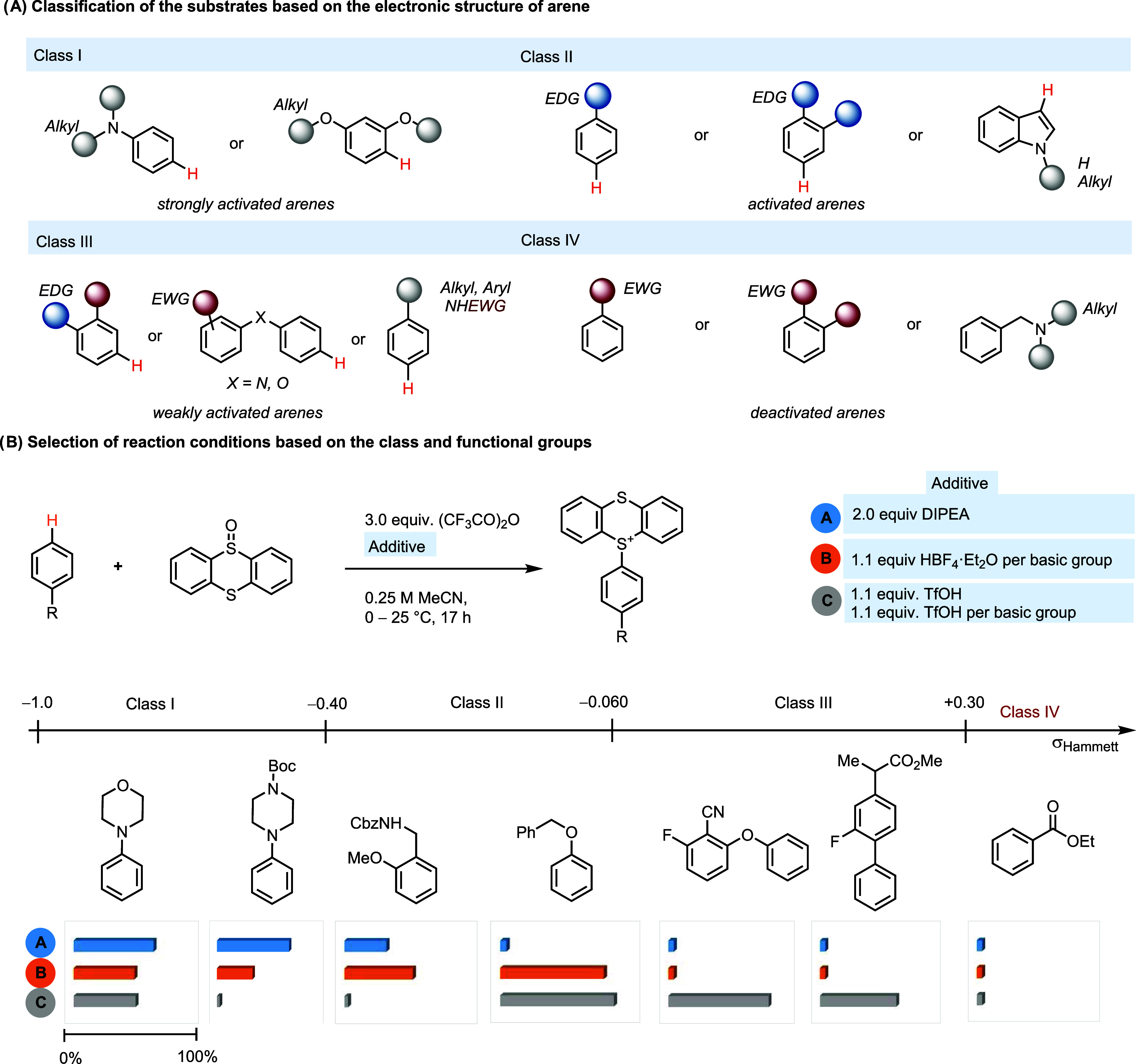
Classification of substrates and selection of reaction parameters based on the electronic structure and functional groups. TTO: thianthrene–S–oxide. DIPEA: diisopropylethylamine. EWG: electron-withdrawing group. Ac: acetyl. MeCN: acetonitrile. TfOH: trifluoromethanesulfonic acid. Cbz: benzyloxycarbonyl.

**Figure 4:**
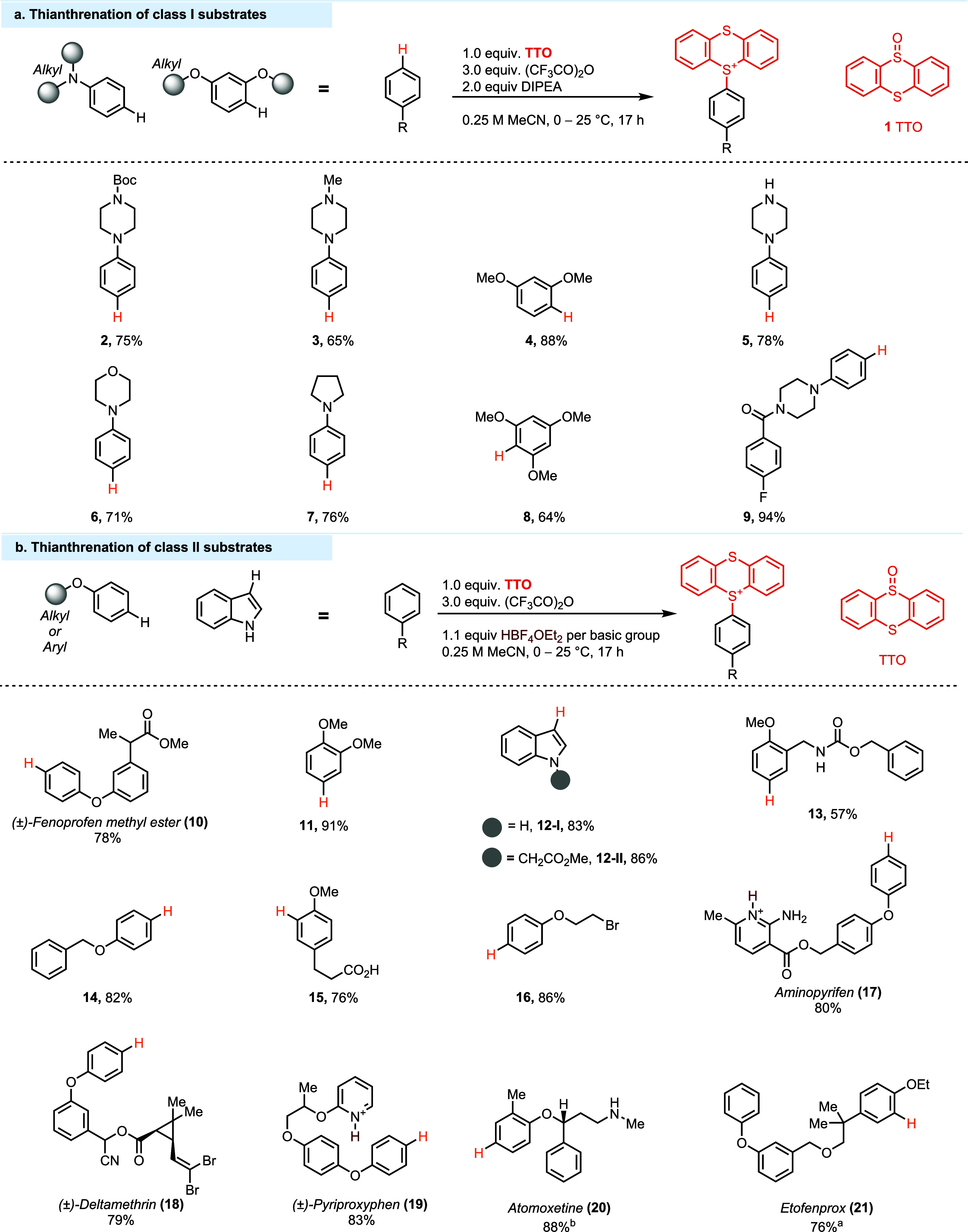
(a) Thianthrenation of Class I substrates. (b) Thianthrenation of Class II substrates. ^a^The reaction was started at −40 °C. ^b^Trifluoroacetylation of the amino group occurs. Basic groups are drawn in their protonated form. Counteranions are omitted for clarity.

**Figure 5:**
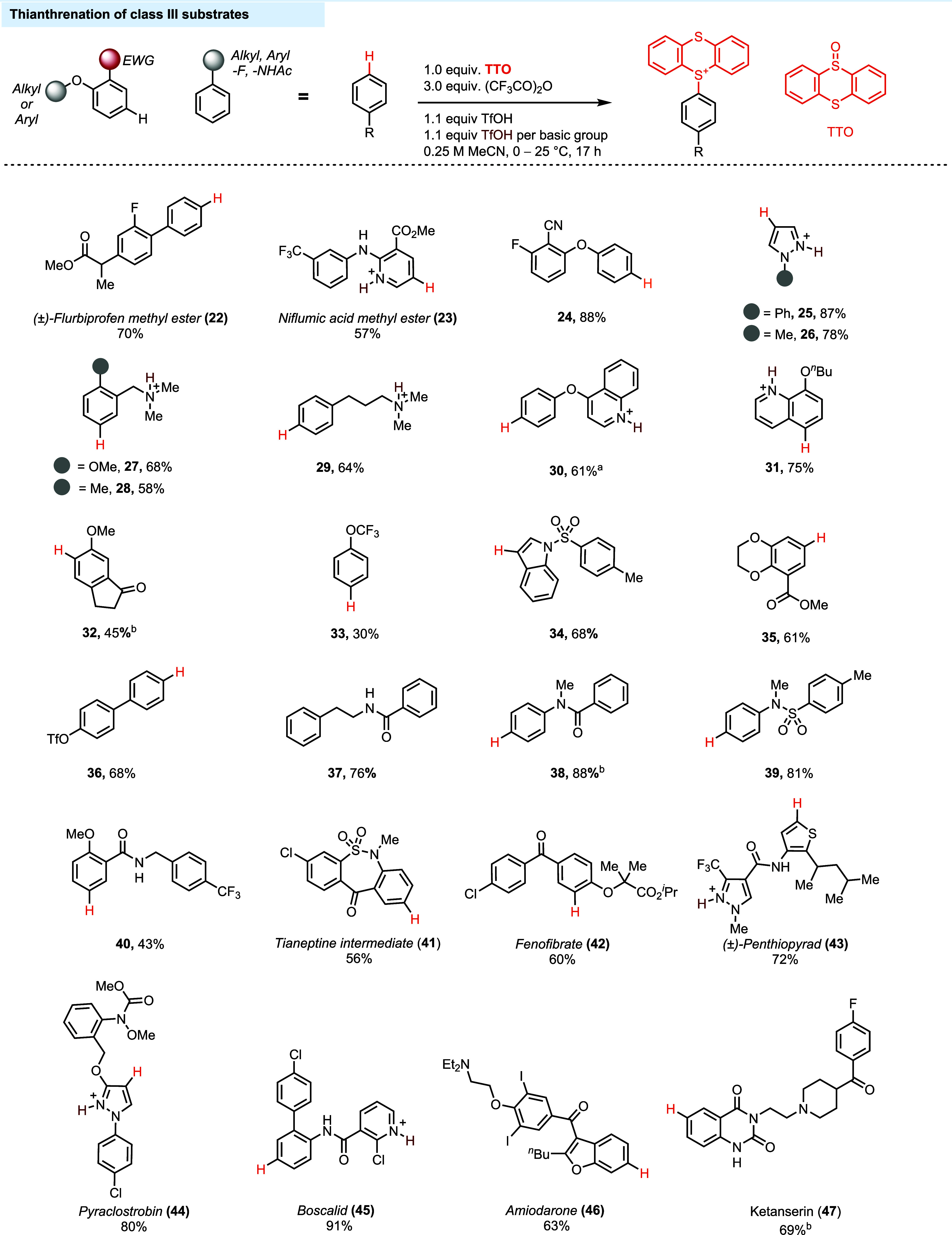
Thianthrenation of Class III substrates. ^a^TMSOTf was used as the acid. ^b^HBF_4_·OEt_2_ was used as the acid. Basic groups are drawn in their protonated/Lewis acid-coordinated form. Counteranions are omitted for clarity.

Arenes are classified into four classes based on their electronic structure, with decreasing electron density and reactivity from classes I to IV. We provide three different reaction conditions, A–C, which increase in severity. In general, class III arenes require the most reactive conditions C, while arenes in class I react in conditions A, B, and C. Although condition C can often be used for class I–III arenes, it does not dominate conditions A and B because several scenarios exist, in which condition C performs worse for class I or class II arenes, as a consequence of side reactions based on the electronic structure or functional groups. Class I substrates encompass arenes containing strongly electron-releasing substituents, such as dialkylamino or two alkoxy groups and no electron-withdrawing groups (Figure 3). Compounds in class I are highly reactive, and thianthrenation of such substrates proceeds by mixing the substrate, TTO, and TFAA in MeCN at 0 °C. The high reactivity of substrates in class I outcompetes the side reactivity of other nucleophilic groups (Figure 4, 3), which are hence tolerated as functional groups. Class I arenes allow the addition of a base such as DIPEA to the reaction mixture. Based on our previous findings, thianthrenation of substrates in class I presumably occurs through a different mechanism compared to the one illustrated in Figure 2 (for a detailed discussion about thianthrenation under basic conditions, see ref (8)). Inclusion of the base is useful to neutralize the trifluoroacetic acid (TFA) liberated during the reaction, which allows the thianthrenation of compounds containing acid-labile groups, such as the Boc group (**2**). Although the addition of a base is not necessary for the successful thianthrenation of electron-rich substrates such as **3**–**9**, it can be used if needed, for example, when a Boc group is present. Base can only be added for arenes of class I. Class II (Figure 3) includes arenes such as alkyl phenyl ethers (Figure 4, **11, 13, 14, 16, 20, 21**), biphenyl ethers (**10**, **17**–**19**), and indoles (**12-I**, **12-II**) without any electron-withdrawing groups on the aromatic ring. Compounds in class II do not require the addition of acid unless there are basic functional groups (**17**, **19**, **21**), in which case 1.1 equiv of HBF_4_·OEt_2_ is required per basic group. Substrates in Class II are not as reactive as Class I compounds, and protonation of basic groups is necessary to suppress competitive pathways such as oxidation of amines and heterocycles with the electrophilic sulfonium species (**17**, **19**). Class III comprises substrates with only weakly electron-rich or slightly electron-deficient arenes (Figure 3). Thianthrenation of compounds in Class III (Figure 5) requires 1 equiv of acid for the reaction and an additional acid equivalent to protonate each basic group in the molecule. Although the use of trifluoromethanesulfonic acid (TfOH) or HBF_4_·OEt_2_ does not make a difference in most cases, the use of trifluoromethanesulfonic acid (TfOH) is advantageous for certain substrates in class III (see the Supporting Information, Figure S3). Alkyl-substituted arenes (**28**, **29**, **37**), biaryls (**22, 36**), arenes with one electron-donating and one electron-withdrawing group (**27**, **35**, **40**, **42)**, pyrazoles (**25**, **26**, **44**), and indoles containing an electron-withdrawing group (**34**) are located in class III. Basic functionalities act as electron-withdrawing groups due to the positive charge after protonation (**27**–**31**). If the basic group is more than 4 σ bonds away from the arene (**29**) or the arene contains electron-releasing substituents, then, the aromatic ring will be reactive enough to react under class III conditions (**27**, **28**). If the basic group is in close proximity ((homo)benzylic position) to the reactive arene and no electron-donating groups are present on the aromatic ring, the σ-withdrawing ability of protonated basic groups may render the arene too electron-poor to react under class III conditions (Supporting Information, **IV-3** and **IV-4** on page S75). Class IV includes deactivated arenes, such as ethylbenzoate and 1,2-dichlorobenzene (Figure 3). Substrates in class IV do not react under the provided reaction conditions. Dichlorobenzene can react with the tetrafluoro analog TFT, and thianthrenation of ethyl benzoate, for example, can be achieved by using liquid SO_2_ as a reaction solvent.^5,15^ However, such conditions are not included in this study in the interest of practicality. Alternatively, thianthrenium salts of electron-deficient (hetero)aryls can be prepared from boronic ester or carboxylic acid precursors by copper-mediated thermal and photochemical strategies.^16,17^Figure 3 summarizes the compatibility of each class under different reaction conditions based on the electron density of the arene and functional groups in the molecule (see the Supporting Information, Figure S5, for more details). The possibility of adding base for substrates in class I and the possibility of avoiding strong acid (TfOH) for class II increase the functional group compatibility. For example, a Boc-protected amine (**2**) can only be tolerated under class I conditions and a Cbz-protected amine (**13**) cannot be tolerated under class III conditions. Calculation of the Hammett σ constants by summing the appropriate σ_p_ and σ_m_ values relative to the expected position of thianthrenation can be readily achieved using a tool developed by Ertl^18^ (Figure 1a, see the Supporting Information, Figure S4 for details). The calculated σ constant serves as a useful and quantitative, predictable descriptor to classify an arene into a class. Although useful in the majority of the cases, the Hammett σ constants cannot always be relied upon as the sole criteria for classifying substrates. The tool cannot be used to locate substrates when thianthrenation occurs on heterocycles (**12**, **23**, **25**, **26**, **31**, **34**, **35**, **43**, **44**, **46**) or on fused arenes (**32**, **35**, **41**, **47**). Additionally, the tool performs poorly when alkyl groups are the only substituents on the aromatic ring (**29**, **37**, **IV-3**, and **IV-4** on page S75)”. While it is not possible to determine an exact threshold value for successful thianthrenation, compounds that do not react under the provided reaction conditions, such as ethylbenzoate 3-phenylpyridine (protonated form) and 1,2-dichlorobenzene, are characterized by σ values higher than +0.40.

### Limitations of the Scope

**Figure 6:**
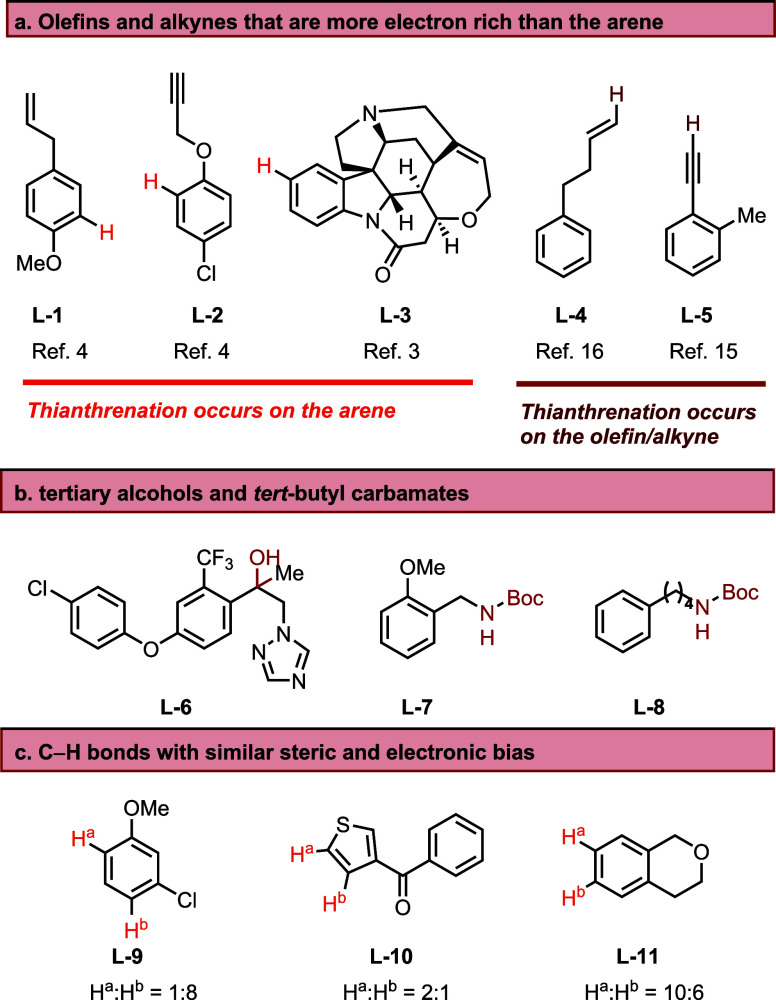
Limitations of the substrate scope for thianthrenation. Boc: tert-butoxycarbamate.

In the absence of electron-releasing substituents on the arene, functional groups such as olefins and alkynes can undergo thianthrenation (Figure 6a).^5,15,19,20^ Additionally, functional groups susceptible to deleterious reactions with strong acids or TFAA, such as tertiary alcohols and *tert*-butyl carbamates, are not tolerated (Figure 6b). Boc groups can be tolerated only if the substrate belongs to class I (**2**). Although rare, certain substitution patterns can result in a low regioselectivity of C–H bond functionalization (Figure 6c). For example, 3-chloroanisole contains two C–H bonds with similar electronic and steric bias. Thianthrenation of such substrates leads to the formation of product mixtures.^19^

### Troubleshooting

#### Case Study: Deactivation of the Arene Due to the Protonation of Basic Groups

4-Phenoxyquinoline (**30**) can be considered a class III substrate where the arene contains an electron-releasing atom (oxygen) connected to an electron-withdrawing substituent (protonated quinoline). The protonated quinoline decreases the electron donation from oxygen and leads to a significant decrease of the electron density on the arene. We found that the use of TMSOTf instead of Brønsted acid eliminates this problem, and thianthrenium salt **30** was prepared in 60% yield (see the Supporting Information, Figure S6). Our finding aligns with selection of TMSOTf as the acid to realize thianthrenation of 2-methoxypyridine in our previous report.^5^

#### Effect of Solvent, Reaction Temperature, and Mode of Addition

For the thianthrenation of ethylbenzene, the reaction yield (98%) did not change when a solvent with 54 ppm water content was used instead of 23 ppm. Addition of 1.5 equiv H_2_O leads to a drop in the yield to 91% (see the Supporting Information, Table S1). A decrease in the reaction yield with an increase in the water content of the solvent can be explained by the hydrolysis of TFAA. Initiating the reaction at 0 °C is generally sufficient to obtain a single constitutional isomer in >100:1 selectivity. Cooling to −40 °C is only required for specific cases, such as for **21** where attack at the most electron-rich site is congested by steric effects; in such cases, a lower temperature provides higher selectivity. The most practical way to set up thianthrenation is to add TFAA to a stirred mixture of TTO and substrate in MeCN at 0 °C followed by addition of an acid (if necessary). If the substrate contains basic groups, then, the appropriate amount of acid has to be added before TFAA to prevent the reaction between basic groups and TFAA.

#### Selection of Important Substituents for Diversification Reactions

**Figure 7:**
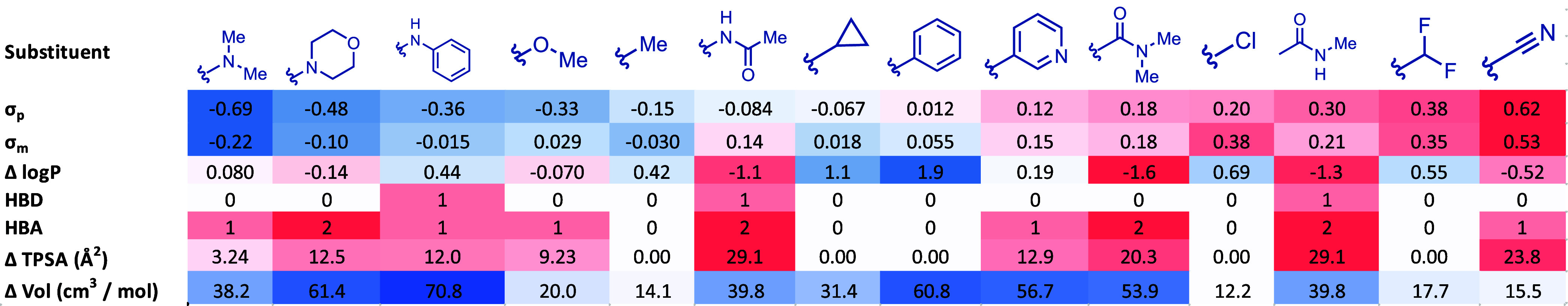
Structural and electronic parameters of substituents in the core set. σ_p_: calculated Hammett constant (para),^18^ σ_m_: calculated Hammett constant (meta),^18^ Δ log *P*: change in the partition coefficient (lipophilicty) of the compound upon addition of the substituent, HBD: hydrogen bond donors, HBA: hydrogen bond acceptors, ΔTPSA: topological polar surface area, and ΔVol: molecular volume calculated in StarDrop and relative to hydrogen in the equivalent position.^21,22^

We selected a core set of common substituents spanning as much diversity in property space as possible with as few examples as possible and focused on small (1–6 heavy atoms) substituents to provide structure–activity relationship (SAR) information (Figure 7). The resulting set of derivatives should be sufficient to guide the design of further analogs. We demonstrate the synthesis of additional derivatives using the same reaction conditions. For example, a bromide substituent could be installed using the conditions for chlorination, or a diversity of amine substituents could be installed using the C–N coupling reaction.

#### Reaction Manifolds for Diversification of Aryl Thianthrenium (TT) Salts

Quick diversification of complex small molecules requires robust reactions that can be carried out in parallel, ideally in an automated setting. We set the following criteria from the start:

- A single thianthrenium salt derivative should be used for each diversification reaction (i.e., same counterion)
- Diversification reactions should give acceptable yields (>30%) across as many substrates as possible with little or no modification required for different substrates.
- Reactions to form each derivative should be performed at the same temperature, with the same reaction time and under the same atmosphere (i.e., they can be performed on the same multiwell plate).
- High catalyst or reagent loadings are acceptable where they do not interfere with product purification.

- Where possible, the same solvent is used for each derivative to facilitate the use of stock solutions and to simplify workup procedures.
- Where possible, the same catalysts/reagents are used for multiple derivatives to simplify reaction preparation.

**Figure 8:**
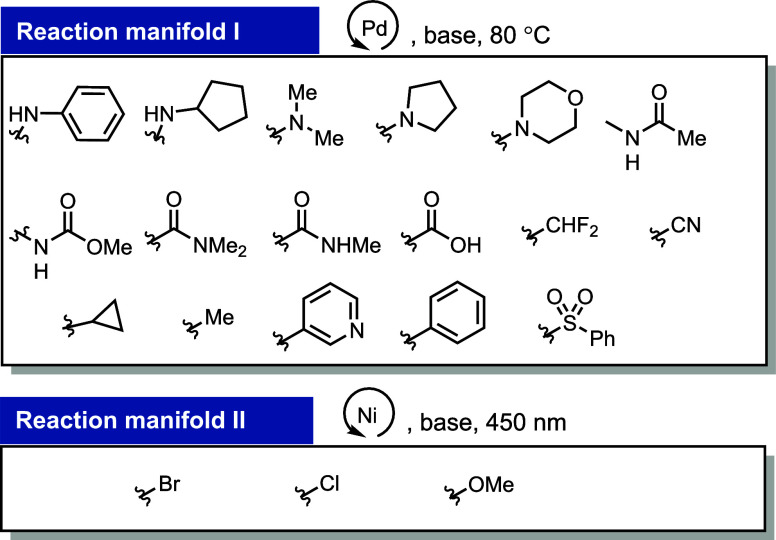
Reaction manifolds for the swift diversification of aryl thianthrenium salts.

We attempted to limit the different reaction manifolds to a minimum to reduce complexity even if it results in lower yields for the introduction of some substituents. This approach resulted in two reaction manifolds to cover the desired substituent classes for diversification (Figure 8). We discovered that a wide range of substituents can be covered with only two orthogonal manifolds, one based on palladium-catalyzed C–C, C–N, and C–S cross-coupling reactions and the other based on nickel-catalyzed photochemical methoxylation and halogenation reactions. Additional manifolds, for example, with other palladium catalysts or copper-based reactions, can, in most cases, improve the yields of products, yet for the sake of simplicity and in the interest of the purpose of this paper, the two manifolds discussed cover the broadest scope while maintaining synthetically useful yields at a minimum of practical complexity. In the following sections, we introduce individual reactions in detail. We start with reactions featuring conceptually new findings and discuss other procedures toward the end.

#### C–N Cross-Couplings

**Figure 9:**
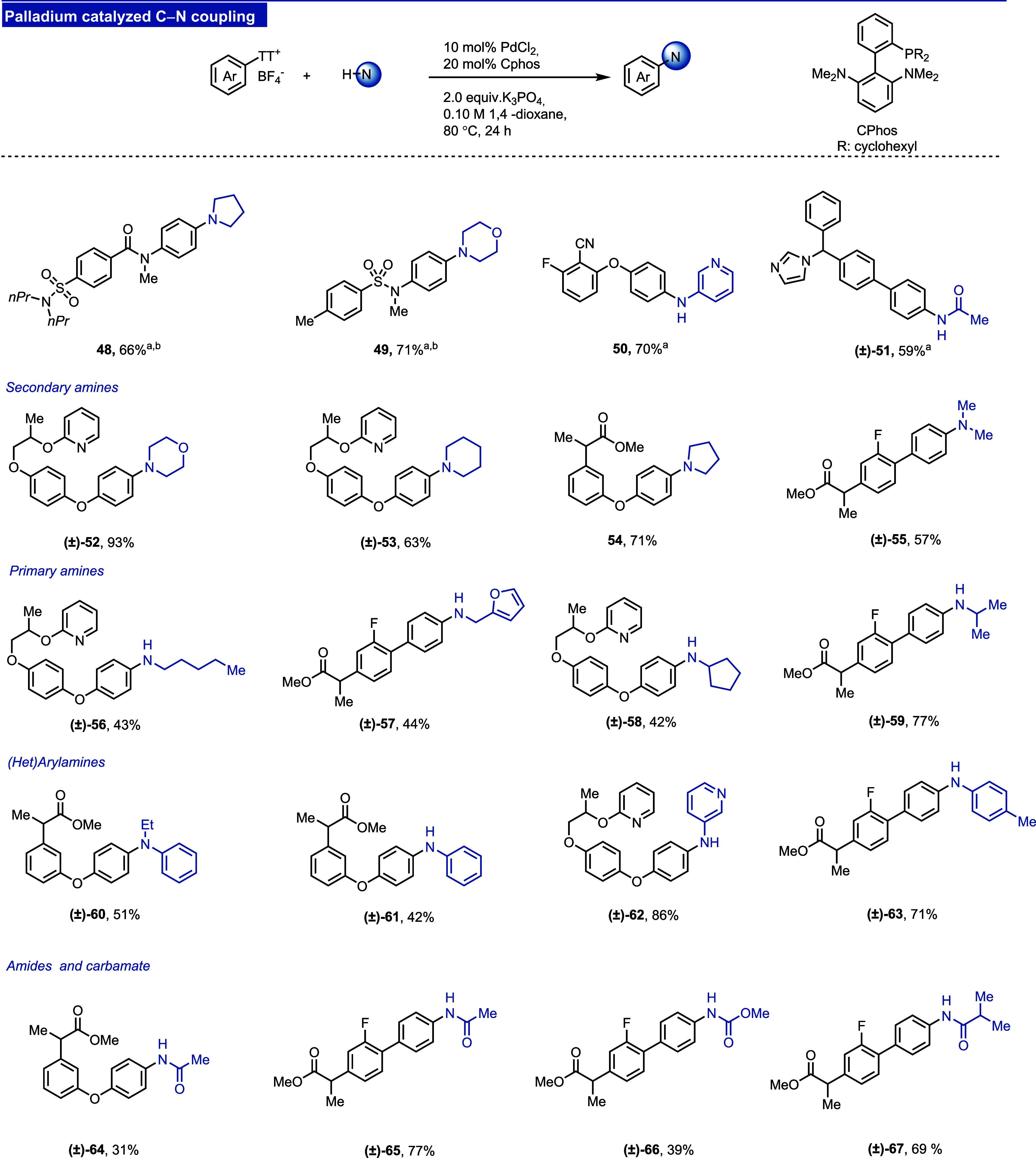
Reaction manifold I: Palladium catalyzed the C–N coupling of aryl thianthrenium salts. ^a^DMF was used as a solvent and Cs_2_CO_3_ was used instead of K_3_PO_4_. ^b^DavePhos was used instead of CPhos.

To date, two different kinds of protocols were developed for C–N coupling of TT salts.^12,23^ Ni-catalyzed C–N bond formation utilizes blue light-emitting diode (LED) irradiation and proceeds through high-valent nickel species. While it is effective over a broad range of amines and thianthrenium salts, we have observed a larger variety of reaction outcomes depending on the different kinds of amines used. Moreover, the reaction is sensitive to the light source and the intensity of light. We sought a more robust transformation that proceeds with a larger scope of different nitrogen sources, including amides, and is more elastic regarding the specific setup. Previously reported palladium-catalyzed aryl thianthrene C–N cross-coupling reactions sometimes give side products that arise from endocyclic ring opening on the thianthrenium core, which complicates purification and reduces yield, sometimes substantially. Furthermore, substantial changes in the reaction conditions and setup are required to accommodate various nitrogen nucleophiles. We show here that the use of CPhos in combination with PdCl_2_ obviates both problems. The palladium/CPhos catalyst is applicable to a wide variety of nitrogen sources, including secondary and primary amines (Figure 9, **52**–**59**), anilines (**60**–**63**), amides, and carbamates (**64**–**67**). Endocyclic ring opening products are observed only for the coupling of amides and carbamates (**54**–**57**), which explains the lower yield in those cases. Most of the reactions were carried out in 1,4-dioxane using K_3_PO_4_ as a base. If the solubility of the thianthrenium salt in 1,4-dioxane is an issue, dimethylformamide (DMF) in combination with Cs_2_CO_3_ can be used alternatively, and DavePhos represents an alternative to CPhos if further optimization is required (see the Supporting Information, Table S2). The use of DMF and Cs_2_CO_3_ does not negatively affect the reactivity and a variety of amines can still be used (**48**–**51**). The major limitation of the reaction is the competitive amination of electron-deficient aryl halides (see the Supporting Information, Figure S7, for more details).

#### Carbonylation

**Figure 10:**
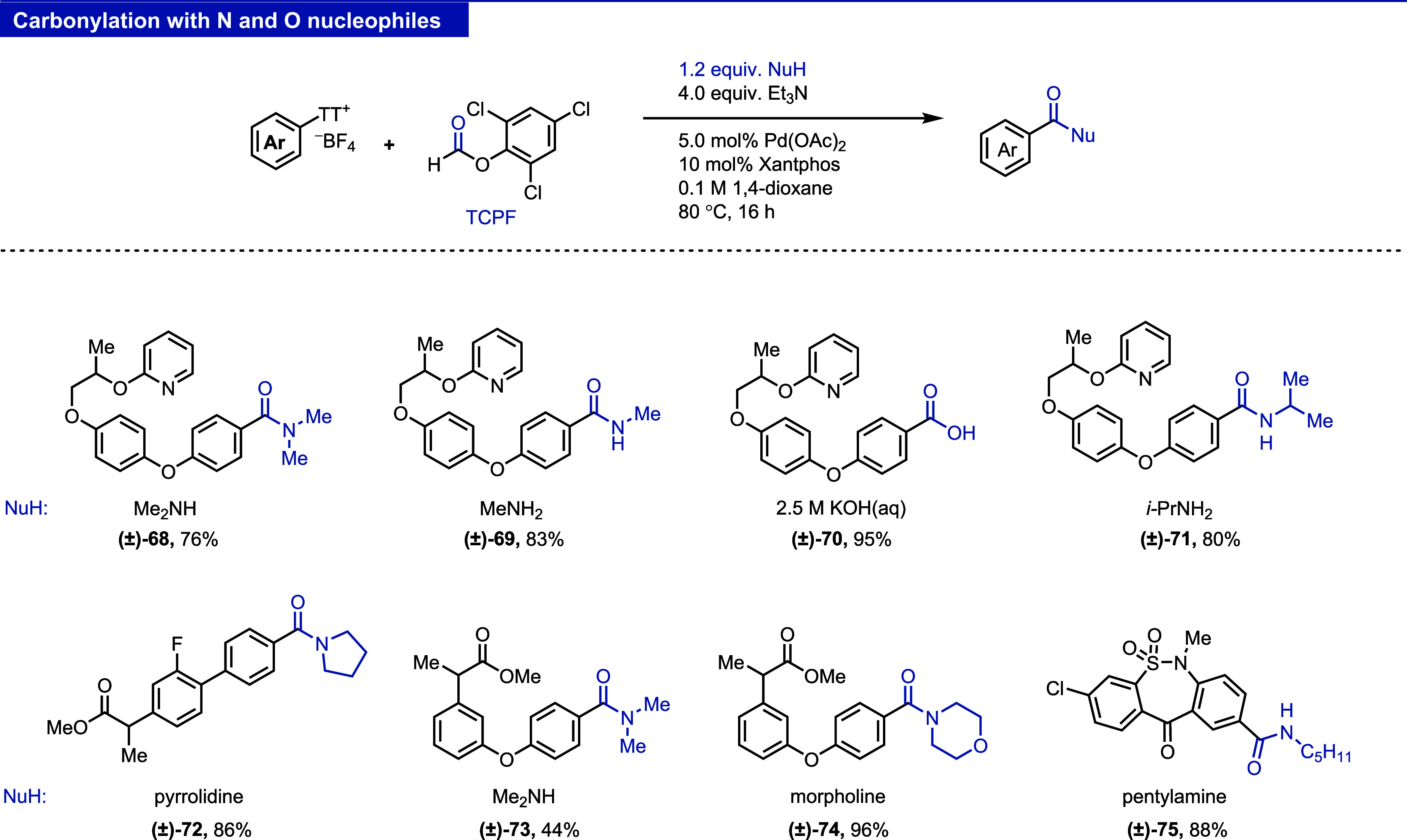
Reaction manifold I: carbonylation of aryl thianthrenium salts with TCPF (2,4,6-trichlorophenyl formate).

The current procedure for the carbonylation of aryl thianthrenium salts employs toxic CO gas, rendering it incompatible for a general screening platform.^5,24^ We evaluated readily available CO surrogates^25,26^ (see the Supporting Information, Table S3) and developed here a one-pot carbonylation procedure with TCPF (2,4,6-trichlorophenyl formate).^27^ TCPF is a commercially available crystalline solid, also accessible in a single step from cheap chemicals.^27,28^ A variety of amines and hydroxides can be used as nucleophiles (Figure 10, **68**–**75**). Use of alcohols such as MeOH and *i*-PrOH as nucleophiles did not yield the desired products. The reaction tolerates various functional groups and heterocycles (see the Supporting Information, Figure S8, for more details).^29^

#### Difluoromethylation

**Figure 11:**
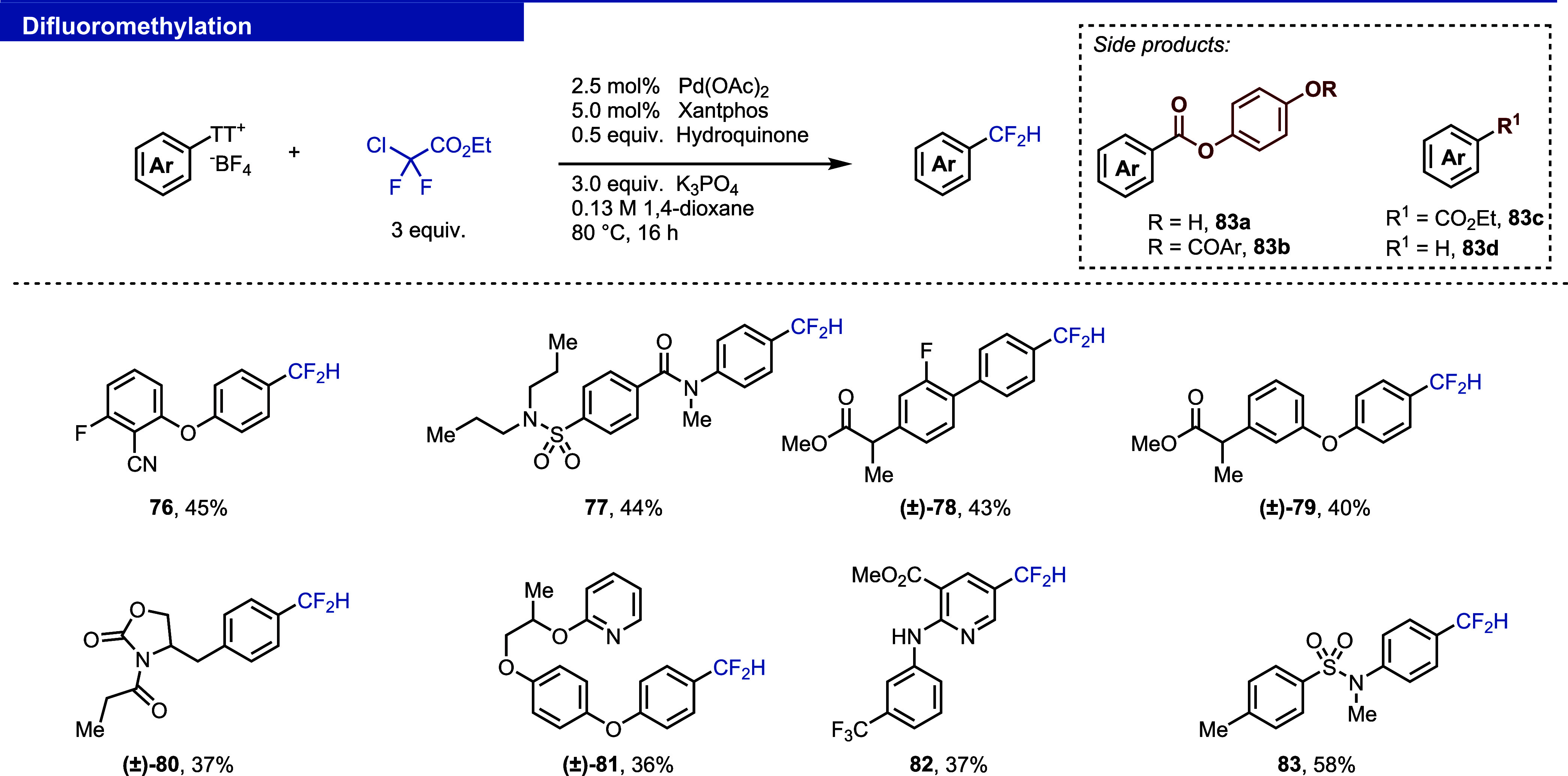
Reaction manifold I: difluoromethylation of aryl thianthrenium salts.

The difluoromethyl group is a nonpolar electron-withdrawing group, which can also act as a hydrogen bond donor and is commonly considered a bioisostere of hydroxyl, thiol, and amino groups.^30,31^ Incorporation of a difluoromethyl substituent to complex small molecules via aryl thianthrenium salts was previously achieved by using stoichiometric copper under photochemical conditions.^32^ The reaction is not general due to the low stability of the [CuCF_2_H] species. During the preparation of this manuscript, Xiaojun and co-workers reported palladium-catalyzed difluoromethylation of aryl thianthrenium salts.^33^ While the reaction is wide in scope, the protocol requires preparation of the difluoromethylating reagent.^34^ Here, we developed a palladium-catalyzed difluoromethylation of thianthrenium salts by using readily available ClCF_2_CO_2_Et.^30,35^ Among the transformations reported here, difluoromethylation is one of the weakest, with rather low yields, and formation of the side products due to competitive carboxylation (see the Supporting Information, Figures S9, S11 and Table S4–S9). We chose to include the transformation, given the importance of the difluoromethyl substituent and the fact that the product is generally obtained for a large variety of thianthrenium salts (Figure 11, **76**–**83**) together with other side products such as **83a**–**d** that are not challenging to separate.

#### Cyanation

**Figure 12:**
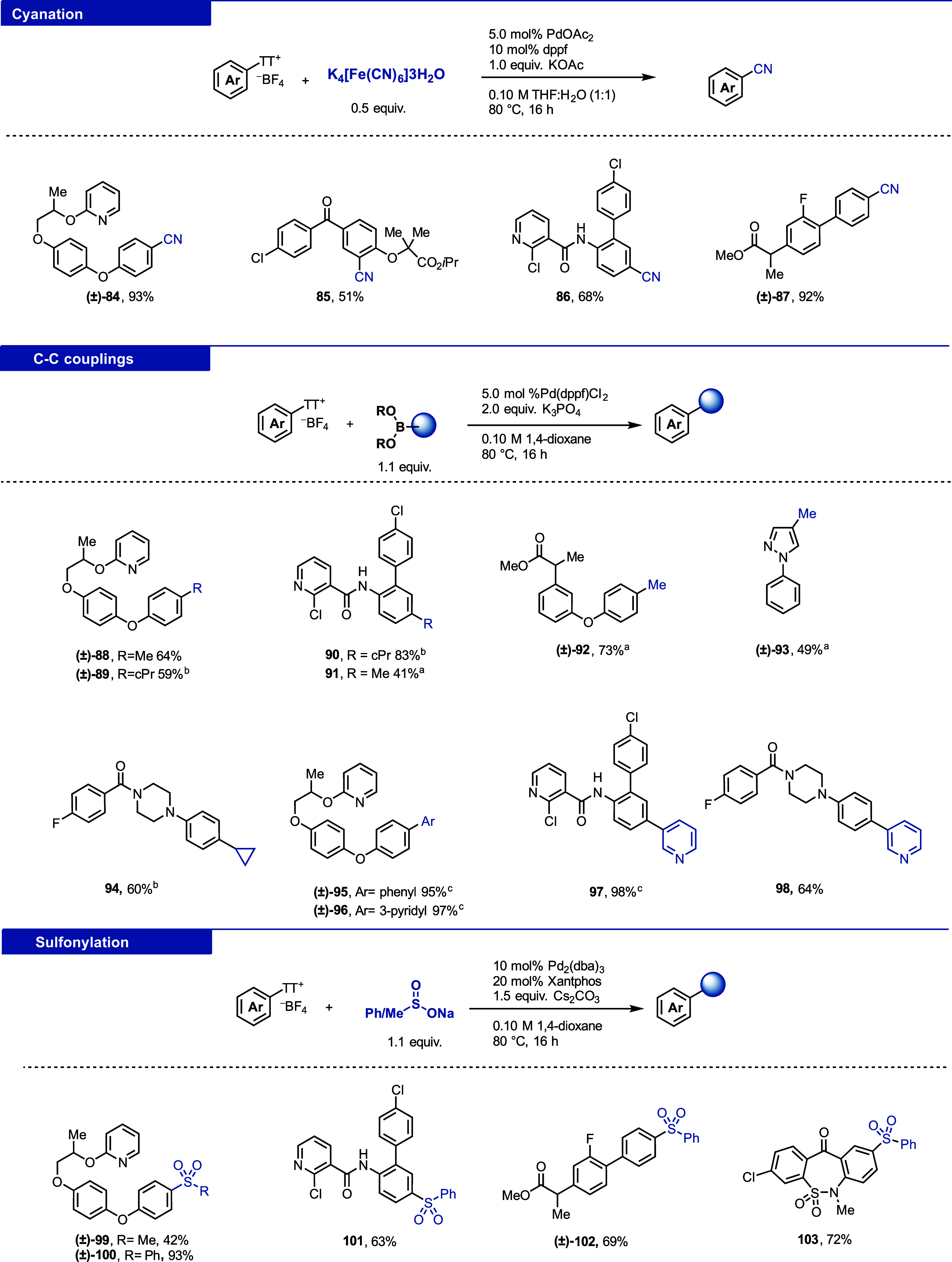
Reaction manifold I: cyanation, sulfonylation, and C–C couplings of aryl thianthrenium salts. ^a^CsF was used instead of K_3_PO_4_. ^b^Pd(amphos)Cl_2_ was used instead of Pd(dppf)Cl_2_. ^c^1,4-dioxane:H_2_O (1:1, v/v) was used as the solvent. cPr: cyclopropyl.

The nitrile group can improve the physicochemical and pharmacokinetic properties of drug molecules such as solubility and bioavailability.^36^ The cyanation of aryl thianthrenium salts was first achieved by using a Cu(I/III) cycle. The report is limited to a single example, utilizing TBACN as a cyanide source and requires a photocatalyst and stoichiometric copper.^5^ The second report employs safer Zn(CN)_2_ as a cyanide source but still requires the use of heterogeneous zinc powder, stoichiometric copper reagent, and elevated temperatures, which make the method incompatible with our platform.^37^ Recently, the copper-mediated photochemical radiocyanation of aryl thianthrenium salts was reported.^38^ We disclose in this work a palladium-catalyzed cyanation that is practical and robust, employs safer K_4_[Fe(CN)_6_]·3H_2_O, and works with a variety of aryl thinathrenium salts (Figure 12, **84**–**87**) The reaction was not affected by the presence of different functional groups and heterocycles (see the Supporting Information, Figure S12, for more details).^29^

#### Methylation and Cyclopropanation

Incorporation of small alkyl groups into complex small molecules can tailor their pharmacological properties.^39,40^ Previously, alkylation of aryl thianthrenium salts with alkylzinc halides was reported.^41^ While robust, few alkylzinc species are available and while they can be prepared, such an approach is not the most practical for a generally available screening platform. Alkyl boronic acids and boronates, on the other hand, are readily available and easy to handle. We developed reaction conditions (see the Supporting Information, Table S10) to introduce methyl and cyclopropyl groups by using trimethylboroxine and cyclopropyl boronic acid, respectively (Figure 12, **88**–**94**).

#### (Hetero)arylation

Csp^2^–Csp^2^ cross-coupling reactions to introduce (hetero)aryls are robust and straightforward with (het)aryl boronic acids.^5^ Challenges for these reactions are rarely observed (Figure 12, **95**–**98**).

#### Sulfonylation

Sulfonylation of aryl thinathrenium salts was previously reported under various reactions conditions.^42−46^ However, only a single example of a palladium-catalyzed coupling between phenylsulfinate and an aryl thianthrenium salt is reported and we present an expanded scope for this reaction.^5^ The reaction is robust and works with a variety of thianthrenium salts (Figure 12, **100**–**103**). The reaction with methylsulfinate would have been a desirable addition to our toolbox of small substituents and was successful in certain cases (**99**); however, the reaction with methylsulfinate is not robust and did not afford the desired products in useful yields with the majority of evaluated thianthrenium salts.

#### Halogenation

**Figure 13:**
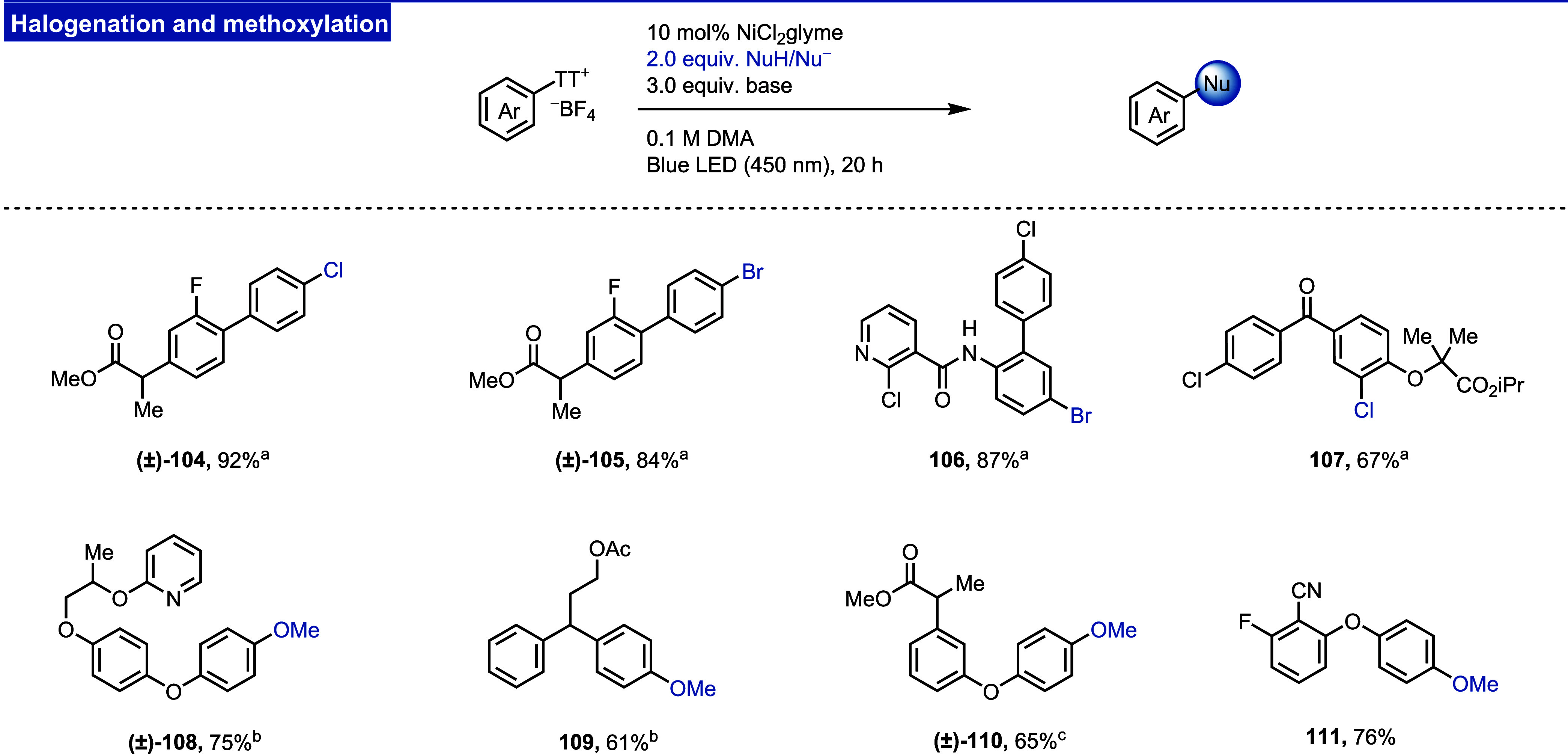
Reaction manifold II: chlorination, bromination, and methoxylation of aryl thianthrenium salts. ^a^Pempidine was used as a base. ^b^ 10 equiv of MeOH was used. Quinuclidine was used as a base.

Aryl thianthrenium salts can be converted to aryl halides either by using stoichiometric copper and light irradiation or thermal nickel-catalyzed cross-coupling.^5,11^ The thermal nickel-catalyzed halogenation is broad in scope. However, we found that the use of heterogeneous zinc powder can sometimes lead to inconsistencies in reaction outcomes. In a separate study, focusing on general nickel-catalyzed coupling reactions of thianthrenium salts under photochemical conditions, halogenation of single thianthrenium salts was reported.^12^ We show here that the reaction is robust and efficient with diverse thianthrenium salts (Figure 13, **104**–**107**).

To ensure the broad applicability of reaction conditions, we investigated the effect of the light source, base, and water. We found that the use of pempidine (1,2,2,6,6-pentamethylpiperidine) as a base is essential to suppress the formation of protodefunctionalized side products, thereby simplifying the purification process. Up to 12% of the protodefunctionalization product was detected when 1.5 equiv. H_2_O was added. The yield of the reaction does not drop when irradiated with various light sources such as blue LED strips, the Penn photoreactor, and Kessil lamps or at different wavelengths (395, 420, 450, 456 nm, see the Supporting Information, Figures S13, S14 and Table S11).

#### Methoxylation

C–O coupling of aryl thianthrenium salts with MeOH was previously achieved for only a few examples, using a combination of stoichiometric copper and a photocatalyst or catalytic nickel under photochemical conditions.^12,47^ We opted to further explore nickel catalysis for the methoxylation of thianthrenium salts to avoid using a photocatalyst or stoichiometric copper. Unlike nickel-catalyzed halogenation, the methoxylation reaction is sensitive to the light source, and the best results were obtained when a Penn photoreactor (450 nm) was used (see the Supporting Information, Figure S14). The reaction is efficient for a variety of aryl thianthrenium salts (Figure 13, **108**–**111**).

#### Application of Findings on an Automated Setting

**Figure 14:**
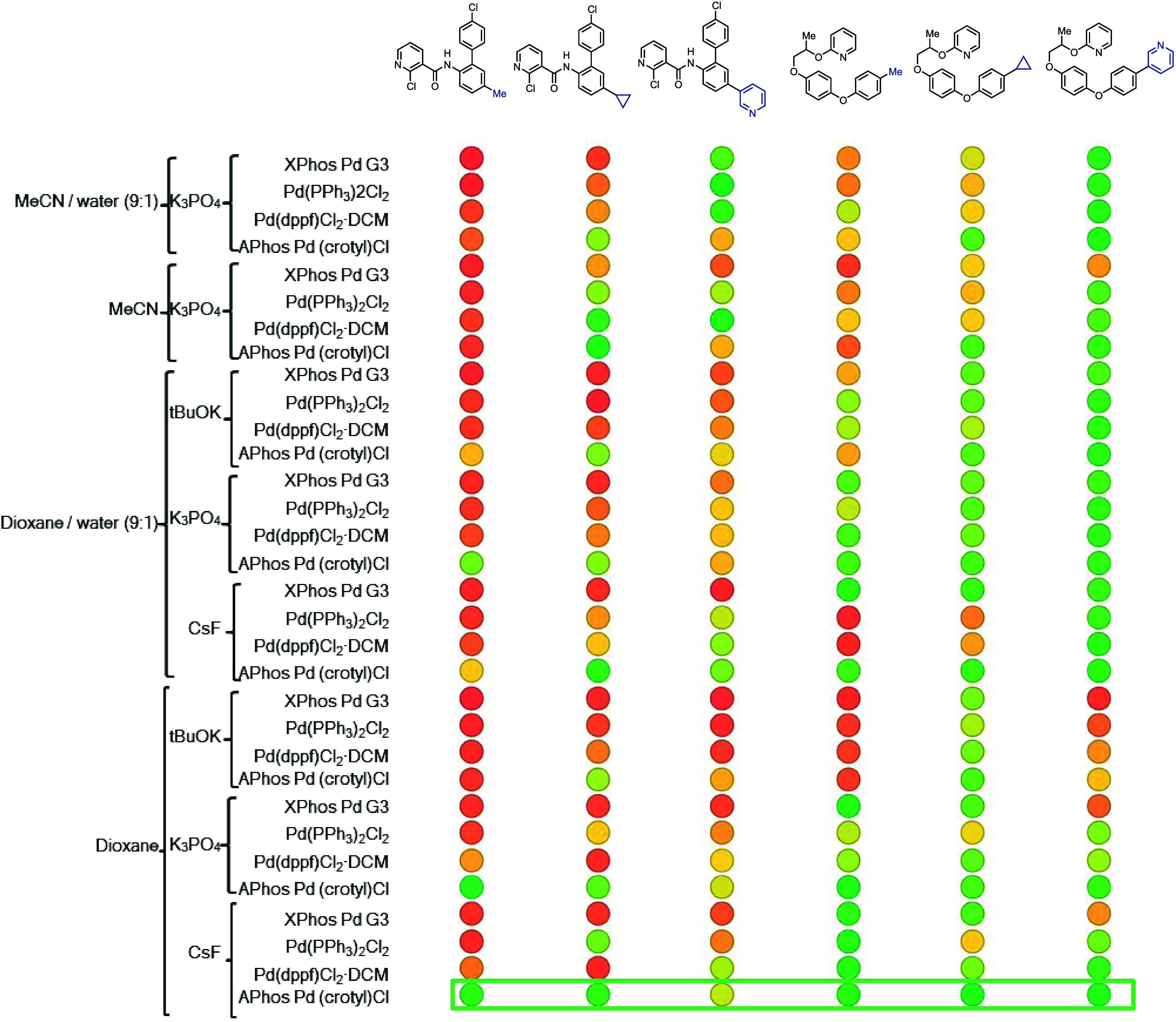
Multiderivative screening of reaction conditions for Suzuki coupling of different boronic acids with boscalid and pyriproxyphen thianthrenium salts. The color represents the LC-area% of the desired product relative to an internal standard and does not represent reaction yield.

As a demonstration of the use of thianthrenium salt diversification in an automated setting and in order to identify the most general set of conditions for Suzuki couplings, we undertook a screening campaign to explore the effect of different bases, catalysts, and solvents across multiple boronic acids and thianthrenium salts. The design of the screen and the results are shown in Figure 14. Evaluating the average performance of all reactions and the lowest-performing reaction revealed that PdCl(crotyl)Amphos was the ideal catalyst, 1,4-dioxane was the best solvent, and CsF was the most effective base. This analysis also showed that K_3_PO_4_ performed well in most reactions and water could be used as a cosolvent, providing flexibility in the reaction setup when needed. Beyond the optimization data, we generated a small Suzuki library of six compounds, albeit without physical isolation. We posit that this proof of concept can be expanded to include the full list of transformations developed in this work, to generate libraries of diversification products from a given thianthrenium salt with only two parallel reaction setups, one for each reactivity manifold.

## Conclusions

Late-stage functionalization and diversification are appealing conceptual approaches for the production of biologically relevant compounds for discovery and optimization campaigns, particularly when combined with high-throughput experimentation and automated synthesis. The practical implementation of such chemistry in the context of highly complex lead-like structures, however, is often nontrivial. In this work, we have developed a late-stage diversification platform based on thianthrenation chemistry that includes both a generalized approach for thianthrenation and two reaction manifolds for parallel diversification of aryl thianthrenium salts to biologically relevant analogs. Classification of the substrates based on the electronic structure of the aromatic ring simplifies the selection of reaction conditions for thianthrenation, and several reaction conditions are presented that should be applicable to the majority of potential substrates. Where possible, we also outline potential pitfalls and troubleshooting options designed to help chemists increase their chances of successfully producing the desired thianthrenium salt derivative without a lengthy optimization campaign. We then present a number of reactions for the diversification of thianthrenium salts into a diverse range of biologically relevant functional groups, focusing on developing and exemplifying reactions that are robust and employ readily available reagents, minimizing practical complexity. Again, we endeavor to present limitations and possible troubleshooting options where possible and note that while we have focused on breadth (i.e., functional diversity) of substituents, depth can also be achieved for a given class using the same reaction conditions, as exemplified for palladium-catalyzed C–N cross-coupling. We recognize the importance of automated synthesis systems in modern discovery chemistry and have attempted to tailor our reaction design to be amenable to that approach wherever possible. Finally, we illustrate our results with numerous examples of lead-like molecules, highlighting the realistic challenges that may be encountered, e.g., solubility, chemoselectivity, reactivity, and how they can be overcome. We anticipate that our research provides a cost- and time-effective yet reliable diversification of complex small molecules to valuable analogs via aryl thianthrenium salts.

## Supporting Information Available

Experimental procedures; spectroscopic data; and NMR spectra of all products (PDF)

## Abbreviations

DIPEA: *N,N*-diisopropylethylamine
DMA: dimethylacetamide
MeCN: acetonitrile
TFAA: trifluoracetic anhydride
TfOH: trifluoromethanesulfonic acid
TTO: thianthrene-*S*-oxide

## References

[ref1] Wencel-Delord J.; Glorius F. C–H bond activation enables the rapid construction and late-stage diversification of functional molecules. Nat. Chem. 2013, 5 (5), 369–375. 10.1038/nchem.1607. 23609086

[ref2] Miller S. J.; Ritter T. Introduction: Remote and Late Stage Functionalization. Chem. Rev. 2023, 123 (24), 13867–13868. 10.1021/acs.chemrev.3c00800. 38148744

[ref3] Börgel J.; Ritter T. Late-Stage Functionalization. Chem 2020, 6 (8), 1877–1887. 10.1016/j.chempr.2020.07.007.

[ref4] Hartwig J. F. Evolution of C–H Bond Functionalization from Methane to Methodology. J. Am. Chem. Soc. 2016, 138 (1), 2–24. 10.1021/jacs.5b08707. 26566092 PMC4809212

[ref5] Berger F.; Plutschack M. B.; Riegger J.; Yu W.; Speicher S.; Ho M.; Frank N.; Ritter T. Site-selective and versatile aromatic C–H functionalization by thianthrenation. Nature 2019, 567 (7747), 223–228. 10.1038/s41586-019-0982-0. 30867606

[ref6] Berger F.; Ritter T. Site-Selective Late-Stage C–H Functionalization via Thianthrenium Salts. Synlett 2022, 33 (04), 339–345. 10.1055/s-0040-1706034.

[ref7] Li J.; Chen J.; Sang R.; Ham W.; Plutschack M. B.; Berger F.; Chabbra S.; Schnegg A.; Genicot C.; Ritter T. Photoredox catalysis with aryl sulfonium salts enables site-selective late-stage fluorination. Nat. Chem. 2020, 12 (1), 56–62. 10.1038/s41557-019-0353-3. 31767996

[ref8] Juliá F.; Shao Q.; Duan M.; Plutschack M. B.; Berger F.; Mateos J.; Lu C.; Xue X.; Houk K. N.; Ritter T. High Site Selectivity in Electrophilic Aromatic Substitutions: Mechanism of C–H Thianthrenation. J. Am. Chem. Soc. 2021, 143 (39), 16041–16054. 10.1021/jacs.1c06281. 34546749 PMC8499029

[ref9] Cai Y.; Chatterjee S.; Ritter T. Photoinduced Copper-Catalyzed Late-Stage Azidoarylation of Alkenes via Arylthianthrenium Salts. J. Am. Chem. Soc. 2023, 145 (25), 13542–13548. 10.1021/jacs.3c04016. 37307146 PMC10311530

[ref10] Zhao D.; Petzold R.; Yan J.; Muri D.; Ritter T. Tritiation of aryl thianthrenium salts with a molecular palladium catalyst. Nature 2021, 600 (7889), 444–449. 10.1038/s41586-021-04007-y. 34912086 PMC8674128

[ref11] Ni S.; Yan J.; Tewari S.; Reijerse E. J.; Ritter T.; Cornella J. Nickel Meets Aryl Thianthrenium Salts: Ni(I)-Catalyzed Halogenation of Arenes. J. Am. Chem. Soc. 2023, 145 (18), 9988–9993. 10.1021/jacs.3c02611. 37126771 PMC10176483

[ref12] Ni S.; Halder R.; Ahmadli D.; Reijerse E. J.; Cornella J.; Ritter T. C–heteroatom coupling with electron-rich aryls enabled by nickel catalysis and light. Nat. Catal. 2024, 7 (6), 733–741. 10.1038/s41929-024-01160-1.

[ref13] Nippa D. F.; Atz K.; Hohler R.; Müller A. T.; Marx A.; Bartelmus C.; Wuitschik G.; Marzuoli I.; Jost V.; Wolfard J.; Binder M.; Stepan A. F.; Konrad D. B.; Grether U.; Martin R. r. E.; Schneider G. Enabling late-stage drug diversification by high-throughput experimentation with geometric deep learning. Nat. Chem. 2024, 16 (2), 239–248. 10.1038/s41557-023-01360-5. 37996732 PMC10849962

[ref14] Buitrago Santanilla A.; Regalado E. L.; Pereira T.; Shevlin M.; Bateman K.; Campeau L.; Schneeweis J.; Berritt S.; Shi Z.; Nantermet P.; Liu Y.; Helmy R.; Welch C. r. J.; Vachal P.; Davies I. W.; Cernak T.; Dreher S. D. Nanomole-scale high-throughput chemistry for the synthesis of complex molecules. Science 2015, 347 (6217), 49–53. 10.1126/science.1259203. 25554781

[ref15] Berger F. Site-selective aromatic C–H functionalization yielding triarylsulfonium salts, and subsequent transformations. Ph.D. Thesis, RWTH Aachen University, 2019.

[ref16] Chen X.-Y.; Li Y.; Wu Y.; Bai J.; Guo Y.; Wang P. Cu-Mediated Thianthrenation and Phenoxathiination of Arylborons. J. Am. Chem. Soc. 2023, 145 (18), 10431–10440. 10.1021/jacs.3c03413. 37099266

[ref17] He Z.; Dydio P. Photoinduced Cu (II)-Mediated Decarboxylative Thianthrenation of Aryl and Heteroaryl Carboxylic Acids. Angew. Chem., Int. Ed. 2024, 136, e202410616 10.1002/ange.202410616. 39012681

[ref18] Ertl P. A Web Tool for Calculating Substituent Descriptors Compatible with Hammett Sigma Constants. Chem. Methods 2022, 2 (12), e202200041 10.1002/cmtd.202200041.

[ref19] Roberts R. A.; Metze B. E.; Nilova A.; Stuart D. R. Synthesis of Arynes via Formal Dehydrogenation of Arenes. J. Am. Chem. Soc. 2023, 145 (6), 3306–3311. 10.1021/jacs.2c13007. 36728842

[ref20] Chen J.; Li J.; Plutschack M. B.; Berger F.; Ritter T. Regio- and Stereoselective Thianthrenation of Olefins To Access Versatile Alkenyl Electrophiles. Angew. Chem., Int. Ed. 2020, 59 (14), 5616–5620. 10.1002/anie.201914215. PMC7154751 31782968

[ref21] Ertl P.; Rohde B.; Selzer P. Fast Calculation of Molecular Polar Surface Area as a Sum of Fragment-Based Contributions and Its Application to the Prediction of Drug Transport Properties. J. Med. Chem. 2000, 43 (20), 3714–3717. 10.1021/jm000942e. 11020286

[ref22] Abraham M. H.; McGowan J. C. The use of characteristic volumes to measure cavity terms in reversed phase liquid chromatography. Chromatographia 1987, 23 (4), 243–246. 10.1007/BF02311772.

[ref23] Engl P. S.; Häring A. P.; Berger F.; Berger G.; Pérez-Bitrián A.; Ritter T. C–N Cross-Couplings for Site-Selective Late-Stage Diversification via Aryl Sulfonium Salts. J. Am. Chem. Soc. 2019, 141 (34), 13346–13351. 10.1021/jacs.9b07323. 31411869

[ref24] Zhao Y.-H.; Gu X.; Wu X. Palladium-Catalyzed Carbonylative Sonogashira Coupling of Aryl Thianthrenium Salts with Arylalkynes. Org. Lett. 2024, 26 (30), 6507–6511. 10.1021/acs.orglett.4c02440. 39023056 PMC11301662

[ref25] Morimoto T.; Kakiuchi K. Evolution of carbonylation catalysis: no need for carbon monoxide. Angew. Chem., Int. Ed. 2004, 43 (42), 5580–5588. 10.1002/anie.200301736. 15372547

[ref26] Larhed M.; Odell L.; Åkerbladh L. Palladium-Catalyzed Molybdenum Hexacarbonyl-Mediated Gas-Free Carbonylative Reactions. Synlett 2019, 30 (02), 141–155. 10.1055/s-0037-1610294.

[ref27] Ueda T.; Konishi H.; Manabe K. Trichlorophenyl Formate: Highly Reactive and Easily Accessible Crystalline CO Surrogate for Palladium-Catalyzed Carbonylation of Aryl/Alkenyl Halides and Triflates. Org. Lett. 2012, 14 (20), 5370–5373. 10.1021/ol302593z. 23020164

[ref28] Wang M.; Zhang X.; Ma M.; Zhao B. Palldium catalyzed Synthesis of Esters from Arenes through C–H Thianthrenation. Org. Lett. 2022, 24 (32), 6031–6036. 10.1021/acs.orglett.2c02330. 35929821

[ref29] Collins K. D.; Glorius F. A robustness screen for the rapid assessment of chemical reactions. Nat. Chem. 2013, 5, 597–601. 10.1038/nchem.1669. 23787750

[ref30] Zhang X. Y.; Sun S. P.; Sang Y. Q.; Xue X. S.; Min Q. Q.; Zhang X. Reductive Catalytic Difluorocarbene Transfer via Palladium Catalysis. Angew. Chem., Int. Ed. 2023, 62 (37), e202306501 10.1002/anie.202306501. 37365143

[ref31] Sap J. B. I.; Meyer C. F.; Straathof N. J. W.; Iwumene N.; Am Ende C. W.; Trabanco A. A.; Gouverneur V. Late-stage difluoromethylation: concepts, developments and perspective. Chem. Soc. Rev. 2021, 50 (14), 8214–8247. 10.1039/D1CS00360G. 34075979

[ref32] Ye F.; Berger F.; Jia H.; Ford J.; Wortman A.; Borgel J.; Genicot C.; Ritter T. Aryl Sulfonium Salts for Site-Selective Late-Stage Trifluoromethylation. Angew. Chem., Int. Ed. 2019, 58 (41), 14615–14619. 10.1002/anie.201906672. PMC7754511 31389649

[ref33] Jiang X.; Gong W.; Li X.; Wang S.; Gu Z.; Yang Y.; Zeng X. Pd-Catalyzed Divergent Site-Selective Difluoromethylation and Difluoromethylcarbonylation of Aryl Sulfonium Salts. ACS Catal. 2024, 14, 13557–13566. 10.1021/acscatal.4c02975.

[ref34] Xu L.; Vicic D. A. Direct Difluoromethylation of Aryl Halides via Base Metal Catalysis at Room Temperature. J. Am. Chem. Soc. 2016, 138 (8), 2536–2539. 10.1021/jacs.6b00053. 26883690

[ref35] Halder R.; Patureau F. W.; Ritter T. Late-stage aromatic C–H difluoroalkylation and amination via arylthianthrenium salts and {18} F-labeling of peptide via ruthenium-mediated deoxyfluorination. Ph.D. Thesis, RWTH Aachen University, 2024.

[ref36] Wang X.; Wang Y.; Li X.; Yu Z.; Song C.; Du Y. Nitrile-containing pharmaceuticals: target, mechanism of action, and their SAR studies. RSC. Med. Chem. 2021, 12 (10), 1650–1671. 10.1039/D1MD00131K. 34778767 PMC8528211

[ref37] Zhang G.; Luo Z.; Guan C.; Zhang X.; Ding C. Nickel-Catalyzed Selective C–H Cyanation via Aromatic Thianthrenium Salts. J. Org. Chem. 2023, 88 (13), 9249–9256. 10.1021/acs.joc.3c00814. 37352468

[ref38] Cheng K.; Webb E. W.; Bowden G. D.; Wright J. S.; Shao X.; Sanford M. S.; Scott P. J. H. Photo- and Cu-Mediated ^11^C Cyanation of (Hetero)Aryl Thianthrenium Salts. Org. Lett. 2024, 26 (16), 3419–3423. 10.1021/acs.orglett.4c00929. 38630573 PMC11099534

[ref39] Nippa D. F.; Atz K.; Müller A. T.; Wolfard J.; Isert C.; Binder M.; Scheidegger O.; Konrad D. B.; Grether U.; Martin R. E.; Schneider G. Identifying opportunities for late-stage C–H alkylation with high-throughput experimentation and in silico reaction screening. Commun. Chem. 2023, 6 (1), 256 10.1038/s42004-023-01047-5. 37985850 PMC10661846

[ref40] Ishikawa M.; Hashimoto Y. Improvement in Aqueous Solubility in Small Molecule Drug Discovery Programs by Disruption of Molecular Planarity and Symmetry. J. Med. Chem. 2011, 54 (6), 1539–1554. 10.1021/jm101356p. 21344906

[ref41] Lansbergen B.; Granatino P.; Ritter T. Site-Selective C–H alkylation of Complex Arenes by a Two-Step Aryl Thianthrenation-Reductive Alkylation Sequence. J. Am. Chem. Soc. 2021, 143 (21), 7909–7914. 10.1021/jacs.1c03459. 34028272 PMC8297726

[ref42] Granados A.; Cabrera-Afonso M. J.; Escolano M.; Badir S. O.; Molander G. A. Thianthrenium-Enabled Sulfonylation via Electron Donor-Acceptor Complex Photoactivation. Chem. Catal. 2022, 2 (4), 898–907. 10.1016/j.checat.2022.03.007. 35846835 PMC9282721

[ref43] Zhang Y.; Xia S.; Shi W.; Lin B.; Su X.; Lu W.; Wu X.; Wang X.; Lu X.; Yan M.; Zhang X. Radical C–H Sulfonation of Arenes: Its Applications on Bioactive and DNA-Encoded Molecules. Org. Lett. 2022, 24 (43), 7961–7966. 10.1021/acs.orglett.2c03077. 36278920

[ref44] He Z.; Li Z.; Lai S.; Li H. Electron Donor–Acceptor Complex Enabled Cyclization/Sulfonylation Cascade of N-Heterocycles with Thianthrenium Salts. Org. Lett. 2024, 26 (31), 6652–6657. 10.1021/acs.orglett.4c02307. 39058904

[ref45] Guo C.; Wang X.; Ding Q.; Wu J. C–H Bond Sulfonylation from Thianthrenium Salts and DABCO·(SO_2_)_2_: Synthesis of 2-Sulfonylindoles. J. Org. Chem. 2024, 89 (13), 9672–9680. 10.1021/acs.joc.4c00827. 38871666

[ref46] Xu H.; Li X.; Wang Y.; Song X.; Shi Y.; Lv J.; Yang D. Arylthianthrenium Salts as the Aryl Sources: Visible Light/Copper Catalysis-Enabled Intermolecular Azidosulfonylation of Alkenes. Org. Lett. 2024, 26 (9), 1845–1850. 10.1021/acs.orglett.4c00017. 38408361

[ref47] Sang R.; Korkis S. E.; Su W.; Ye F.; Engl P. S.; Berger F.; Ritter T. Site-Selective C–H Oxygenation via Aryl Sulfonium Salts. Angew. Chem., Int. Ed. 2019, 58 (45), 16161–16166. 10.1002/anie.201908718. PMC7754133 31475767

