# Supplementary material for: Standardized Approach for Diversification of Complex Small Molecules via Aryl Thianthrenium Salts
Source: J Am Chem Soc. 2025 Jan 22;147(5):4268–83. doi: 10.1021/jacs.4c14391 (PMC11803749; doi:10.1021/jacs.4c14391)
Supplement: Supplementary file 1 — ja4c14391_si_001.pdf [file ja4c14391_si_001.pdf]

SUPPORTING INFORMATION

Standardized Approach for Diversification of Complex Small Molecules via Aryl Thianthrenium Salts

Dilgam Ahmadli,[1,2] Sven Müller,[1,2] Yuanhao Xie,[1,2] Tomas Smejkal,[3] Simon Jaeckh,[3] Andrei V. Iosub[3], Simon R. Williams,[3] and Tobias Ritter*[1]

[1]Max-Planck-Institut für Kohlenforschung, Kaiser-Wilhelm-Platz 1, Mülheim an der Ruhr, 45470, Germany.

[2]Institute of Organic Chemistry, RWTH Aachen University, Landoltweg 1, 52074 Aachen, Germany.

[3]Research Chemistry, Syngenta Crop Protection AG, Schaffhauserstrasse 101, Stein AG 4332, Switzerland.

*E-mail: ritter@kofo.mpg.de

TABLE OF CONTENTS

TABLE OF CONTENTS 2

MATERIALS AND METHODS 15

EXPERIMENTAL DATA 17

General procedure A for thianthrenation of Class I substrates 17

General procedure B for thianthrenation of Class II substrates 17

General procedure C for thianthrenation of Class III substrates 17

Guidelines for precipitation of aryl thinathrenium salts 18

Selection of reaction parameters for thianthrenation 19

Comparison of activators 19

Comparison of sulfoxides 19

Comparison of acids 20

Thianthrenation of arenes 20

Thianthrene–*S*–Oxide 20

tert-Butyloxycarbonyl (-Boc) protected phenylpiperazine derivative **2** 21

tert-Butyloxycarbonyl (-Boc) protected phenylpiperazine-derived thianthrenium salt **2a** 22

1-Methyl-4-phenylpiperazine **3** 22

1-Methyl-4-phenylpiperazine-derived thianthrenium salt **3a** 23

1,3-Dimethoxybenzene-derived thianthrenium salt **4a** 24

4-Phenylpiperazine-derived thianthrenium salt **5a** 25

N-Phenylmorpholine-derived thianthrenium salt **6a** 26

1-Phenylpyrrolidine-derived thianthrenium salt **7a** 27

1,3,5-Trimethoxybenzene-derived thianthrenium salt **8a** 27

N-Phenylpiperazine derivative **9** 28

1-Phenylpiperazine derivative-derived thianthrenium salt **9a** 29

(±)-Fenoprefen methyl ester **10** 30

(±)-Fenoprefen methyl ester-derived thianthrenium salt **10a** 30

1,2-Dimethoxybenzene-derived thianthrenium salt **11a** 31

Indole-derived thianthrenium salt **12-Ia** 32

Indole derivative **12-II** 33

Indole derivative-derived thianthrenium salt **12-IIa** 33

Benzyl 2-methoxybenzylcarbamate **13** 34

Benzyl 2-methoxybenzylcarbamate-derived thianthrenium salt **13a** 35

Benzylphenylether-derived thianthrenium salt **14a** 35

p-Methoxyhydrocinnamic acid-derived thianthrenium salt **15a** 36

2-Phenoxyethyl bromide-derived thianthrenium salt **16a** 37

Aminopyrifen-derived thianthrenium salt **17a** 38

(±)-Deltamethrin-derived thianthrenium salt **18a** 39
(±)-Pyriproxyphen-derived thianthrenium salt **19a** 40
Atomoxetine-derived thianthrenium salt **20a** 40
Etofenprox-derived thianthrenium salt **21a** 41
(±)-Flurbiprofen methyl ester **22-S** 42
(±)-Flurbiprofen methyl ester-derived thianthrenium salt **22a** 43
Niflumic acid methyl ester **23-S** 44
Niflumic acid methyl ester-derived thianthrenium salt **23a** 44
2-Fluoro-6-phenoxybenzonitrile-derived thianthrenium salt **24a** 45
N-Phenyl pyrazole-derived thianthrenium salt **25a** 46
N-Methyl pyrazole-derived thianthrenium salt **26a** 47
1-(2-Methoxyphenyl)-*N,N*-dimethylmethanamine **27** 48
1-(2-Methoxyphenyl)-*N,N*-dimethylmethanamine-derived thianthrenium salt **27a** 48
N,N-Dimethyl-1-(o-tolyl)methanamine **28** 49
N,N-Dimethyl-1-(o-tolyl)methanamine-derived thianthrenium salt **28a** 50
N,N-Dimethyl-2-phenylpropame-1-amine **29** 51
N,N-Dimethyl-2-phenylpropane-1-amine-derived thianthrenium salt **29a** 51
4-Phenoxyquinoline **30** 52
4-Phenoxyquinoline-derived thianthrenium salt **30a** 53
8-Butyloxy quinoline **31** 54
8-Butyloxyquinolone-derived thianthrenium salt **31a** 54
6-Methoxy-2,3-dihydro-1*H*-inden-1-one **32** 55
6-Methoxy-2,3-dihydro-1*H*-inden-1-one derived thianthrenium salt **32a** 56
Trifluoromethoxybenzene-derived thianthrenium salt **33a** 56
N-Tosylindole-derived thianthrenium salt **34a** 57
1,4-Benzodioxane-5-carboxylic acid methyl ester **35** 58
1,4-Benzodioxane-5-carboxylic acid methyl ester-derived thianthrenium salt **35a** 59
Biphenly triflate **36** 59
Biphenyl triflate-derived thianthrenium salt **36a** 60
N-Phenylethylbenzamide-derived thianthrenium salt **37a** 61
N-Methyl-*N*-phenyl-benzamide **38** 62
N-Methyl-*N*-phenyl-benzamide-derived thianthrenium salt **38a** 62
N-Methyl-*N*-phenyltoluenesulfonamide **39** 63
N-Methyl-N-phenyltoluenesulfonamide-derived thianthrenium salt **39a** 64
N-Benzyl-2-methoxybenzamide **40** 64
N-Benzyl-2-methoxybenzamide-derived thianthrenium salt **40a** 65
Tianeptine intermediate-derived thianthrenium salt **41a** 66
Fenofibrate-derived thianthrenium salt **42a** 67
(±)-Penthiopyrad derived thianthrenium salt **43a** 68
Pyraclostrobin derived thianthrenium salt **44a** 69

Boscalid-derived thianthrenium salt **45a** 70
Amiodarone-derived thianthrenium salt **46a** 71
Amiodarone-derived thianthrenium salt **47a** 72
2-Benzoyl thiophene-derived thianthrenium salt **L-10** 72
Isochroman-derived thianthrenium salt **L-11** 73

Calculation of Hammett values for substrates 74

Comparison of different reaction conditions for different classes of arenes 76

Troubleshooting: A Case study for thianthrenation 77

Deactivation of arene due to the protonation of the basic group in close proximity 77

Effect of the water content of the solvent on thianthrenation 77

Follow-on transformations of aryl thianthrenium salts 78

C–N cross-coupling reactions of aryl thianthrenium salts 78

General procedure for C–N cross-coupling reactions of aryl thianthrenium salts 79
Probenecid derivative **48-S-I** 80
Probenecid derivative-derived thianthrenium salt **48-S** 80
Probenecid-derived pyrrolidine derivative **48** 81
N-Phenylbenzenesulfonamide morpholine derivative **49** 82
Phenoxybenzonitrile 3-aminopyridine derivative **50** 82
(±)-Bifonazole derived thianthrenium salt **51-S** 83
(±)-Bifonazole acetamide derivative **51** 84
(±)-Pyriproxyphen morpholine derivative **52** 85
(±)-Pyriproxyphen piperidine derivative **53** 86
(±)-Fenoprofen methyl ester pyrrolidine derivative **54** 86
(±)-Flurbiprofen methyl ester dimethylamine derivative **55** 87
(±)-Pyriproxyphen pentylamine derivative **56** 88
(±)-Flurbiprofen methyl ester furfurylamine derivative **57** 89
(±)-Pyriproxyphen cyclopentylamine derivative **58** 89
(±)-Flurbiprofen methyl ester isopropylamine derivative **59** 90
(±)-Fenoprofen methyl ester *N*-ethylaniline derivative **60** 91
(±)-Fenoprofen methyl ester aniline derivative **61** 92
(±)-Pyriproxyphen 3-aminopyridine derivative **62** 92
(±)-Flurbiprofen methyl ester *para*-methylaniline derivative **63** 93
(±)-Fenoprofen methyl ester acetamide derivative **64** 94
(±)-Flurbiprofen methyl ester acetamide derivative **65** 95
(±)-Flurbiprofen methyl ester carbamate derivative **66** 96
(±)-Flurbiprofen methyl ester isobutyramide derivative **67** 97
Unsuccessful substrate for C–N cross-coupling reactions of aryl thianthrenium salts 98

Carbonylation of aryl thianthrenium salts 98

General procedure for carbonylation of aryl thianthrenium salts 99
(±)-Pyriproxyphen dimethylamine carboamide derivative **68** 99
(±)-Pyriproxyphen methylamine carboamide derivative **69** 100
(±)-Pyriproxyphen carboxylic acid derivative **70** 101
(±)-Pyriproxyphen ispropylamine carboamide derivative **71** 101
(±)-Flurbiprofen methyl ester pyrrolidine carboamide derivative **72** 102
(±)-Fenoprofen methyl ester pyrrolidine carboamide derivative **73** 103
(±)-Fenoprofen methyl ester morpholine carboamide derivative **74** 104
Tianeptine intermediate pentylamine carboamide derivative **75** 104
Additive screening for carboamidation of aryl thianthrenium salts 105

Difluoromethylation of aryl thianthrenium salts 106

General procedure for the optimization of reaction conditions 106
General procedure for difluoromethylation of aryl thianthrenium salts 110
Biphenylether difluormethyl derivative **76** 111
Probenecid amide difluoromethyl derivative **77** 112
(±)-Flurbiprofen methyl ester difluoromethyl derivative **78** 113
(±)-Fenoprofen methyl ester difluoromethyl derivative **79** 114
(±)-Oxazolidinone-derived thianthrenim salt **80-S** 115
(±)-Oxazolidinone difluoromethyl derivative **80** 115
(±)-Pyriproxyphen difluoromethyl derivative **81** 116
Niflumic acid methyl ester difluoromethyl derivative **82** 117
N-Tosylmethylaniline difluoromethyl derivative **83** 118
Analysis of the side products for difluoromethylation of aryl thianthrenium salts 119
Difluoromethylation side product **83a** 120
Difluoromethylation side product **83b** 120
Difluoromethylation side product **83c** 120
Difluoromethylation side product **83d** 121

Cyanation of aryl thianthrenium salts 121

General procedure for cyanation of aryl thianthrenium salts 121
(±)-Pyriproxyphen cyanide derivative **84** 121
Fenofibrate cyanide derivative **85** 122
Boscalid cyanide derivative **86** 123
(±)-Flurbiprofen methyl ester cyanide derivative **87** 124
Additive screening for cyanation of aryl thianthrenium salts 125

C–C bond formation of aryl thianthrenium salts 126

General procedure for C–C bond formation of aryl thianthrenium salts 127
(±)-Pyriproxyphen methyl derivative **88** 128
(±)-Pyriproxyphen cyclopropyl derivative **89** 129
Boscalid cyclopropyl derivative **90** 130

Boscalid methyl derivative **91** ... 130
(±)-Fenoprofen methyl ester methyl derivative **92** ... 131
Phenyl pyrazole methyl derivative **93** ... 132
Phenyl piperazine-derivative cyclopropyl derivative **94** ... 133
Pyriproxyphen phenyl derivative **95** ... 134
(±)-Pyriproxyphen pyridyl derivative **96** ... 134
Boscalid pyridiyl derivative **97** ... 136
Phenyl piperazine-derivative pyridyl derivative **98** ... 136

Sulfonylation of aryl thianthrenium salts ... 137

General procedure for sulfonylation of aryl thianthrenium salts ... 137
(±)-Pyriproxyphen methyl sulfone derivative **99** ... 138
(±)-Pyriproxyphen phenyl sulfone derivative **100** ... 138
Boscalid phenyl sulfone derivative **101** ... 140
(±)-Fluribuprofen methyl ester phenyl sulfone derivative **102** ... 140
Tianeptine intermediate sulfone derivative **103** ... 141

Halogenation and methoxylation of aryl thianthrenium salts ... 142

General procedure for halogenation of aryl thianthrenium salts ... 142
General procedure for methoxylation of aryl thianthrenium salts ... 142
Effect of base on product ratio for chlorination of aryl thianthrenium salts ... 143
Effect of water for chlorination of aryl thianthrenium salts ... 143
Effect of irradiation source ... 144
(±)-Flurbiprofen methyl ester chloride derivative **104** ... 144
(±)-Flurbiprofen methyl ester bromide derivative **105** ... 145
Boscalid bromide derivative **106** ... 146
Fenofibrate chloride derivative **107** ... 147
(±)-Pyriproxyphen methoxide derivative **108** ... 148
3,3-Diphenyl propyl acetate **109-S-I** ... 148
3,3-Diphenyl propyl acetate-derived thianthrenium salt **109-S** ... 149
3,3-Diphenyl propyl acetate methoxide derivative **109** ... 150
(±)-Fenoprofen methyl ester methoxide derivative **110** ... 150
Phenoxybenzonitrile methoxide derivative **111** ... 151
Effect of counterianion on physical properties and reactivity of aryl thianthrenium salts ... 152

SPECTROSCOPIC DATA ... 153

^{1}H NMR of **2** ... 153
^{13}C NMR of **2** ... 154
^{1}H NMR of **2a** ... 155
^{13}C NMR of **2a** ... 156
^{19}F NMR of **2a** ... 157

^{1}H NMR of **3** ... 158
^{13}C NMR of **3** ... 159
^{1}H NMR of **3a** ... 160
^{13}C NMR of **3a** ... 161
^{19}F NMR of **3a** ... 162
^{1}H NMR of **4a** ... 163
^{13}C NMR of **4a** ... 164
^{19}F NMR of **4a** ... 165
^{1}H NMR of **5a** ... 166
^{13}C NMR of **5a** ... 167
^{19}F NMR of **5a** ... 168
^{1}H NMR of **6a** ... 169
^{13}C NMR of **6a** ... 170
^{19}F NMR of **6a** ... 171
^{1}H NMR of **7a** ... 172
^{13}C NMR of **7a** ... 173
^{19}F NMR of **7a** ... 174
^{1}H NMR of **8a** ... 175
^{13}C NMR of **8a** ... 176
^{19}F NMR of **8a** ... 177
^{1}H NMR of **9** ... 178
^{13}C NMR of **9** ... 179
^{19}F NMR of **9** ... 180
^{1}H NMR of **9a** ... 181
^{13}C NMR of **9a** ... 182
^{19}F NMR of **9a** ... 183
^{1}H NMR of **10a** ... 184
^{13}C NMR of **10a** ... 185
^{19}F NMR of **10a** ... 186
^{1}H NMR of **11a** ... 187
^{13}C NMR of **11a** ... 188
^{19}F NMR of 1**1a** ... 189
^{1}H NMR of **12-Ia** ... 190
^{13}C NMR of **12-Ia** ... 191
^{19}F NMR of **12-Ia** ... 192
^{1}H NMR of **12-IIa** ... 193
^{13}C NMR of **12-IIa** ... 194
^{19}F NMR of **12-IIa** ... 195
^{1}H NMR of **13a** ... 196
^{13}C NMR of **13a** ... 197

^{19}F NMR of **13a** 198
^{1}H NMR of **14a** 199
^{13}C NMR of **14a** 200
^{19}F NMR of **14a** 201
^{1}H NMR of **15a** 202
^{13}C NMR of **15a** 203
^{19}F NMR of **15a** 204
^{1}H NMR of **16a** 205
^{13}C NMR of **16a** 206
^{19}F NMR of **16a** 207
^{1}H NMR of **17a** 208
^{13}C NMR of **17a** 209
^{19}F NMR of **17a** 210
^{1}H NMR of **18a** 211
^{13}C NMR of **18a** 212
^{19}F NMR of **18a** 213
^{1}H NMR of **19a** 214
^{13}C NMR of **19a** 215
^{19}F NMR of **19a** 216
^{1}H NMR of **20a** 217
^{13}C NMR of **20a** 218
^{19}F NMR of **20a** 219
^{1}H NMR of **21a** 220
^{13}C NMR of **21a** 221
^{19}F NMR of **21a** 222
^{1}H NMR of **22a** 223
^{13}C NMR of **22a** 224
^{19}F NMR of **22a** 225
^{1}H NMR of **23a** 226
^{13}C NMR of **23a** 227
^{19}F NMR of **23a** 228
^{1}H NMR of **24a** 229
^{13}C NMR of **24a** 230
^{19}F NMR of **24a** 231
^{1}H NMR of **25a** 232
^{13}C NMR of **25a** 233
^{19}F NMR of **25a** 234
^{1}H NMR of **26a** 235
^{13}C NMR of **26a** 236
^{19}F NMR of **26a** 237

^{1}H NMR of **27** 238
^{13}C NMR of **27** 239
^{1}H NMR of **27a** 240
^{13}C NMR of **27a** 241
^{19}F NMR of **27a** 242
^{1}H NMR of **28** 243
^{13}C NMR of **28** 244
^{1}H NMR of **28a** 245
^{13}C NMR of **28a** 246
^{19}F NMR of **28a** 247
^{1}H NMR of **29** 248
^{13}C NMR of **29** 249
^{1}H NMR of **29a** 250
^{13}C NMR of **29a** 251
^{19}F NMR of **29a** 252
^{1}H NMR of **30** 253
^{13}C NMR of **30** 254
^{1}H NMR of **30a** 255
^{13}C NMR of **30a** 256
^{19}F NMR of **30a** 257
^{1}H NMR of **31a** 258
^{13}C NMR of **31a** 259
^{19}F NMR of **31a** 260
^{1}H NMR of **32a** 261
^{13}C NMR of **32a** 262
^{19}F NMR of **32a** 263
^{1}H NMR of **33a** 264
^{13}C NMR of **33a** 265
^{19}F NMR of **33a** 266
^{1}H NMR of **34a** 267
^{13}C NMR of **34a** 268
^{19}F NMR of **34a** 269
^{1}H NMR of **35a** 270
^{13}C NMR of **35a** 271
^{19}F NMR of **35a** 272
^{1}H NMR of **36a** 273
^{13}C NMR of **36a** 274
^{19}F NMR of **36a** 275
^{1}H NMR of **37a** 276
^{13}C NMR of **37a** 277

^{19}F NMR of **37a** 278
^{1}H NMR of **38a** 279
^{13}C NMR of **38a** 280
^{19}F NMR of **38a** 281
^{1}H NMR of **39a** 282
^{13}C NMR of **39a** 283
^{19}F NMR of **39a** 284
^{1}H NMR of **40** 285
^{13}C NMR of **40** 286
^{19}F NMR of **40** 287
^{1}H NMR of **40a** 288
^{13}C NMR of **40a** 289
^{19}F NMR of **40a** 290
^{1}H NMR of **41a** 291
^{13}C NMR of **41a** 292
^{19}F NMR of **41a** 293
^{1}H NMR of **42a** 294
^{13}C NMR of **42a** 295
^{19}F NMR of **42a** 296
^{1}H NMR of **43a** 297
^{13}C NMR of **43a** 298
^{19}F NMR of **43a** 299
^{1}H NMR of **44a** 300
^{13}C NMR of **44a** 301
^{19}F NMR of **44a** 302
^{1}H NMR of **45a** 303
^{13}C NMR of **45a** 304
^{19}F NMR of **45a** 305
^{1}H NMR of **46a** 306
^{13}C NMR of **46a** 307
^{19}F NMR of **46a** 308
^{1}H NMR of **47a** 309
^{13}C NMR of **47a** 310
^{19}F NMR of **47a** 311
^{1}H NMR of **48-S-I** 312
^{13}C NMR of **48-S-I** 313
^{1}H NMR of **48** 314
^{13}C NMR of **48** 315
^{1}H NMR of **49** 316
^{13}C NMR of **49** 317

^{1}H NMR of **50** 318
^{13}C NMR of **50** 319
^{19}F NMR of **50** 320
^{1}H NMR of **51** 321
^{13}C NMR of **51** 322
^{1}H NMR of **52** 323
^{13}C NMR of **52** 324
^{1}H NMR of **53** 325
^{13}C NMR of **53** 326
^{1}H NMR of **54** 327
^{13}C NMR of **54** 328
^{1}H NMR of **55** 329
^{13}C NMR of **55** 330
^{19}F NMR of **55** 331
^{1}H NMR of **56** 332
^{13}C NMR of **56** 333
^{1}H NMR of **57** 334
^{13}C NMR of **57** 335
^{19}F NMR of **57** 336
^{1}H NMR of **58** 337
^{13}C NMR of **58** 338
^{1}H NMR of **59** 339
^{13}C NMR of **59** 340
^{19}F NMR of **59** 341
^{1}H NMR of **60** 342
^{13}C NMR of **60** 343
^{1}H NMR of **61** 344
^{13}C NMR of **61** 345
^{1}H NMR of **62** 346
^{13}C NMR of **62** 347
^{1}H NMR of **63** 348
^{13}C NMR of **63** 349
^{19}F NMR of **63** 350
^{1}H NMR of **64** 351
^{13}C NMR of **64** 352
^{1}H NMR of **65** 353
^{13}C NMR of **65** 354
^{19}F NMR of **65** 355
^{1}H NMR of **66** 356
^{13}C NMR of **66** 357

^{19}F NMR of **66** 358
^{1}H NMR of **67** 359
^{13}C NMR of **67** 360
^{19}F NMR of **67** 361
^{1}H NMR of **68** 362
^{13}C NMR of **68** 363
^{1}H NMR of **69** 364
^{13}C NMR of **69** 365
^{1}H NMR of **70** 366
^{13}C NMR of **70** 367
^{1}H NMR of **71** 368
^{13}C NMR of **71** 369
^{1}H NMR of **72** 370
^{13}C NMR of **72** 371
^{19}F NMR of **72** 372
^{1}H NMR of **73** 373
^{13}C NMR of **73** 374
^{1}H NMR of **74** 375
^{13}C NMR of **74** 376
^{1}H NMR of **75** 377
^{13}C NMR of **75** 378
^{1}H NMR of **76** 379
^{13}C NMR of **76** 380
^{19}F NMR of **76** 381
^{1}H NMR of **77** 382
^{13}C NMR of **77** 383
^{19}F NMR of **77** 384
^{1}H NMR of **78** 385
^{13}C NMR of **78** 386
^{19}F NMR of **78** 387
^{1}H NMR of **79** 388
^{13}C NMR of **79** 389
^{19}F NMR of **79** 390
^{1}H NMR of **80** 391
^{13}C NMR of **80** 392
^{19}F NMR of **80** 393
^{1}H NMR of **81** 394
^{13}C NMR of **81** 395
^{19}F NMR of **81** 396
^{1}H NMR of **82** 397

^{13}C NMR of **82** 398
^{19}F NMR of **82** 399
^{1}H NMR of **83** 400
^{13}C NMR of **83** 401
^{19}F NMR of **83** 402
^{1}H NMR of **83a** 403
^{13}C NMR of **83a** 404
^{1}H NMR of **83b** 405
^{13}C NMR of **83b** 406
^{1}H NMR of **83c** 407
^{13}C NMR of **83c** 408
^{1}H NMR of **83d** 409
^{13}C NMR of **83d** 410
^{1}H NMR of **84** 411
^{13}C NMR of **84** 412
^{1}H NMR of **85** 413
^{13}C NMR of **85** 414
^{1}H NMR of **86** 415
^{13}C NMR of **86** 416
^{1}H NMR of **87** 417
^{13}C NMR of **87** 418
^{19}F NMR of **87** 419
^{1}H NMR of **88** 420
^{13}C NMR of **88** 421
^{1}H NMR of **89** 422
^{13}C NMR of **89** 423
^{1}H NMR of **90** 424
^{13}C NMR of **90** 425
^{1}H NMR of **91** 426
^{13}C NMR of **91** 427
^{1}H NMR of **92** 428
^{13}C NMR of **92** 429
^{1}H NMR of **93** 430
^{13}C NMR of **93** 431
^{1}H NMR of **94** 432
^{13}C NMR of **94** 433
^{19}F NMR of **94** 434
^{1}H NMR of **95** 435
^{13}C NMR of **95** 436
^{1}H NMR of **96** 437

^{13}C NMR of **96** 438
^{1}H NMR of **97** 439
^{13}C NMR of **97** 440
^{1}H NMR of **98** 441
^{13}C NMR of **98** 442
^{19}F NMR of **98** 443
^{1}H NMR of **99** 444
^{13}C NMR of **99** 445
^{1}H NMR of **100** 446
^{13}C NMR of **100** 447
^{1}H NMR of **101** 448
^{13}C NMR of **101** 449
^{1}H NMR of **102** 450
^{13}C NMR of **102** 451
^{19}F NMR of **102** 452
^{1}H NMR of **103** 453
^{13}C NMR of **103** 454
^{1}H NMR of **104** 455
^{13}C NMR of **104** 456
^{19}F NMR of **104** 457
^{1}H NMR of **105** 458
^{13}C NMR of **105** 459
^{19}F NMR of **105** 460
^{1}H NMR of **106** 461
^{13}C NMR of **106** 462
^{1}H NMR of **107** 463
^{13}C NMR of **107** 464
^{1}H NMR of **108** 465
^{13}C NMR of **108** 466
^{1}H NMR of **109** 467
^{13}C NMR of **109** 468
^{1}H NMR of **110** 469
^{13}C NMR of **110** 470
^{1}H NMR of **111** 471
^{13}C NMR of **111** 472
^{19}F NMR of **111** 473

REFERENCES 474

MATERIALS AND METHODS

Concentration under reduced pressure was performed by rotary evaporation at 37–40 °C at an appropriate pressure if not otherwise stated. Purified compounds were further dried under high vacuum (0.01–0.005 mbar). Yields refer to purified and spectroscopically pure compounds. 25 °C is defined as a temperature range from 23–27 °C.

Solvents

Acetonitrile was purchased from *Fischer Chemical* and used as received. Anhydrous DCM, THF and 1,4-dioxane were obtained from *Phoenix Solvent Drying Systems.* DMA (99.5%, Extra Dry) was ordered from *abcr* and stored in a nitrogen-filled Glovebox. All deuterated solvents were purchased from *Euriso-Top*.

Chromatography

Thin layer chromatography (TLC) was performed using Polygram Sil G/UV254 plates from *Macherey-Nagel* pre-coated with 0.20 mm silica and a fluorescence indicator. Plates were visualized by irradiation at 254 nm from a *Herolab* UV-lamp. Flash chromatography was performed using silica gel (40–63 µm particle size) purchased from *Geduran* or Biotage® Sfär Silica columns (100 Å pore size, 60 µm particle size) on an automated purification system (Biotage® Isolera One). The detailed solvent composition of the eluents is given for every compound individually.

Spectroscopy and Instruments

NMR spectra were recorded on a Bruker Ascend TM 500 spectrometer operating at 500 MHz, 471 MHz and 126 MHz, for ^{1}H, ^{19}F, and ^{13}C acquisitions, respectively; a Bruker AVANCE NEO 600 spectrometer equipped with a cryogenically cooled cryoBBO probe operating at 600 MHz, 565 MHz and 151 MHz for ^{1}H, ^{19}F, and ^{13}C acquisitions, respectively. Chemical shifts are reported in ppm with the solvent residual peak as the internal standard.[1] For ^{1}H NMR: $CDCl_3$, 7.26; $(CD_3)_2SO$, 2.50; CD_3CN, 1.94; for ^{13}C NMR: $CDCl_3$, 77.16; $(CD_3)_2SO$, 39.52, CD_3CN, 1.32; ^{19}F NMR spectra were referenced using a unified chemical shift scale based on the ^{1}H resonance of tetramethylsilane (1% v/v solution in the respective solvent).[2] Data is reported as follows: s = singlet, d = doublet, t = triplet, q = quartet, quin = quintet, sext = sextet, sept = septet, m = multiplet, bs = broad singlet; coupling constants in Hz.

Photochemical reactions were performed using a Penn PhD integrated Photoreactor M2. 450 nm light was used as light source for nickel-catalyzed chlorination and methoxylation reactions.

Starting materials

All starting materials were used as received from *Sigma-Aldrich, Chempur, TCI, abcr GmbH, BLD Pharma, Apollo Scientific* and *Alfa Aesar* unless otherwise stated. Starting materials **17**, **19**, **42**, **43**, **44** were obtained from Syngenta Crop Protection AG.

Safety statement

During the preparation of compounds **68**–**75** (Carbonylation of aryl thinathrenium salts), vigorous gas

(CO) evolution starts after the addition of Et_3N. Subsequent addition of other reagents should be performed with caution as pressure build-up may push the liquids outside from the syringe.

EXPERIMENTAL DATA

General procedure A for thianthrenation of Class I substrates

To a solution of an arene (0.250 mmol, 1.00 equiv.) and TTO (thianthrene-*S*-oxide, 58.1 mg, 0.250 mmol, 1.00 equiv.) in MeCN (1.0 mL, *c* = 0.25 M), DIPEA (*N,N*-diisopropylethylamine, 87 µL, 65 mg, 0.50 mmol, 2.0 equiv.) was added under an ambient atmosphere. The suspension was cooled to 0 °C with an ice bath, and trifluoroacetic anhydride (104 µL, 158 mg, 0.750 mmol, 3.00 equiv.) was added dropwise at 0 °C over approximately 30 seconds. The reaction mixture was stirred (400 rpm) at 0 °C for 1 h, and then stirred at 25 °C for 16 h. The solvent was removed under reduced pressure by rotary evaporation and the residue was dissolved in DCM (10 mL). The resulting solution was washed with saturated aqueous $NaHCO_3$ solution (10 mL) and aqueous $NaBF_4$ solution (2 × 10 mL, 10% w/w). The DCM layer was dried over Na_2SO_4, filtered, and the solvent was removed under reduced pressure. The residue was purified by chromatography on silica gel eluting with DCM / *i*-PrOH. Alternatively, the residue was dissolved in DCM (3–5 mL) and ether (MTBE or Et_2O, 60–70 mL) was added to obtain the product as a precipitate after filtration.

General procedure B for thianthrenation of Class II substrates

To a solution of an arene (0.500 mmol, 1.00 equiv.) and TTO (thianthrene-*S*-oxide, 116 mg, 0.500 mmol, 1.00 equiv.) in MeCN (2.0 mL, *c* = 0.25 M), $HBF_4{\cdot}OEt_2$ (89 µL, 81 mg, 0.55 mmol, 1.1 equiv. per basic group in the molecule) was added at 0 °C, under an ambient atmosphere followed by addition of trifluoroacetic anhydride (0.21 mL, 0.32 mg, 1.5 mmol, 3.0 equiv.). The reaction mixture was stirred (400 rpm) at 0 °C for 1 h, and then stirred at 25 °C for 16 h. The solvent was removed under reduced pressure by rotary evaporation and the residue was dissolved in DCM (10 mL). The resulting solution was washed with saturated aqueous $NaHCO_3$ solution (10 mL) and aqueous $NaBF_4$ solution (2 × 10 mL, 10% w/w). The DCM layer was dried over Na_2SO_4, filtered, and the solvent was removed under reduced pressure. The residue was purified by chromatography on silica gel eluting with DCM / *i*-PrOH. Alternatively, the residue was dissolved in DCM (3–5 mL) and ether (MTBE or Et_2O, 60–70 mL) was added to obtain the precipitate.

General procedure C for thianthrenation of Class III substrates

To a solution of an arene (0.500 mmol, 1.00 equiv.) and TTO (thianthrene-*S*-oxide, 116 mg, 0.500 mmol, 1.00 equiv.) in MeCN (2.0 mL, *c* = 0.25 M), TfOH (49 µL, 75 mg, 0.55 mmol, 1.1 equiv. per basic group in the molecule) was added at 0 °C, under an ambient atmosphere. Then, trifluoroacetic anhydride (0.21 mL, 0.32 mg, 1.5 mmol, 3.0 equiv.) was added dropwise over approximately 30 seconds followed by addition of TfOH (49 µL, 83 mg, 0.55 mmol, 1.1 equiv.). The reaction mixture was stirred (400 rpm) at 0 °C for 1 h, and then stirred at 25 °C for 16 h. The solvent was removed under reduced pressure by rotary evaporation and the residue was dissolved in DCM (10 mL). The resulting solution was washed with saturated aqueous $NaHCO_3$ solution (10 mL) and aqueous $NaBF_4$ solution (2 × 10 mL, 10% w/w). The DCM layer was dried over Na_2SO_4, filtered, and the solvent was removed under reduced pressure. The residue was purified by chromatography on silica gel eluting with DCM /

i-PrOH. Alternatively, the residue was dissolved in DCM (3–5 mL) and ether (MTBE or Et_2O, 60–70 mL) was added to obtain the precipitate.

Guidelines for precipitation of aryl thinathrenium salts

In the majority of cases, analytically pure thianthrenium salts can be obtained by precipitation.

Step 1: Confirm full ion exchange to $^{-}BF_4$ (–150 ppm, ^{19}F NMR), $^{-}PF_6$ (–73 ppm, ^{19}F NMR) or ^{-}OTf (–80 ppm, ^{19}F NMR) after aqueous workup. Thianthrenium salts with $CF_3CO_2^-$ (–76 ppm, ^{19}F NMR) counterion are prone to precipitate.

Step 2: Dissolve the residue in the minimum amount of DCM (3–5 mL per 0.5 mmol of the thianthrenium salt) in a 100 mL round-bottomed flask. Slowly add 10–15 mL ether (MTBE or Et_2O) to the flask. Swirl the flask to form a suspension and add 60–70 mL of ether (MTBE or Et_2O) in one portion. Collect the precipitate by filtration.

Troubleshooting:

- If the precipitate does not form in Step 2, try trituration to get an analytically clean sample: Add a stirring bar to the flask and stir vigorously (600–800 rpm) for 3–5 minutes. Then stop the stirring and wait for 3–5minutes. Then, collect the solid by filtration.
- If the precipitate does not form after vigorous stirring: add the solution of thianthrenium salt in DCM (3–5 mL for 0.5 mmol scale) dropwise to ether (70–80 mL, cooled to 0 °C with an ice bath) while stirring vigorously (600–800 rpm).
- If the precipitate becomes sticky during filtration: First remove the solvent by decantation.Then, remove the remaining solvent by a syringe. Concentrate under reduced pressure by rotary evaporation.

Selection of reaction parameters for thianthrenation

Comparison of activators

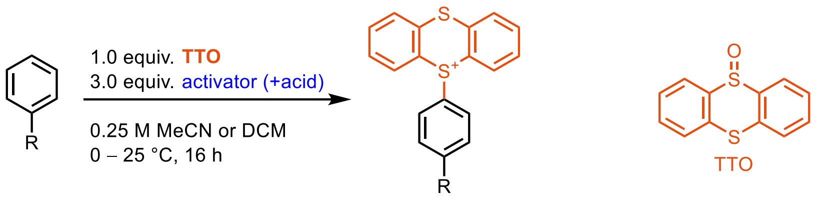

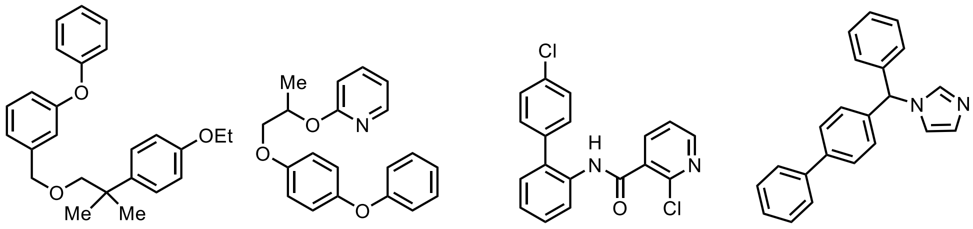

	Etofenprox	Pyriproxyphen	Boscalid	Bifonazole
TFAA + HBF$_4$·OEt$_2$	● (92%)	● (99%)	● (86%)	● (94%)
Tf$_2$O	*n.d. mixture of products*	● (0%)	*n.d. mixture of products*	● (99%)
Tf$_2$O + 1 equiv. TfOH per basic group		● (70%)	● (99%)	● (100%)

● >80% yield, clean reaction

● 40-70% yield, not clean reaction

● 0-10% yield.

Fig S1: Comparison of activators. Reactions were carried out in 0.10 mmol scale in MeCN (0.25 M). Yields were determined by ^{1}H NMR using CH_2Br_2 (1.0 equiv.) as an internal standard. TFAA: Trifluoroacetic anhydride. Tf_2O: Trifluoromethanesulfonic anhydride.

Comparison of sulfoxides

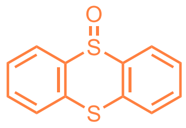

TTO
CAS: 2362-50-7

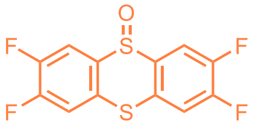

TFTO
CAS: 2362-50-7

- **commercially available: €371/5g**
- **prepared from thianthrene (€94/25g) in one step**
- **Superior reactivity of resulting thianthreniums salts in derivatization reactions**

- **commercially available: €907/5g**
- **prepared from difluorobenzene (€168/50g) in two steps**
- **inferior reactivity of resulting thianthreniums salts reactivity in derivatization reactions**

Fig S2: Comparison of sulfoxides for thianthrenation.

Comparison of acids

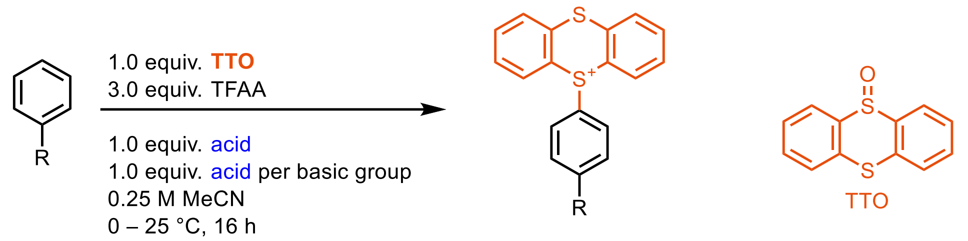

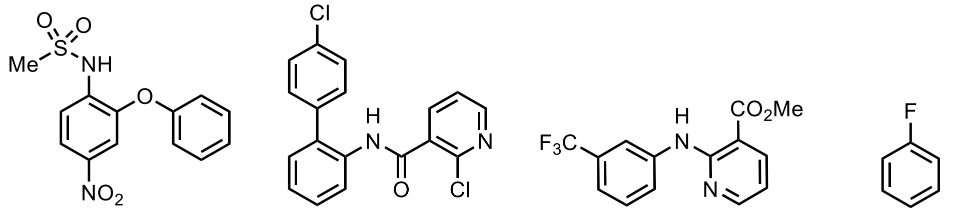

	Nimesulide	Boscalid	Niflumic acid methyl ester	Fluorobenzene
TfOH	🟢 (75%)	🟢 (98%)	🟢 57%[a]	🟢 55%
$HBF_4{\cdot}OEt_2$	🔴 (0%)	🟢 (92%)[a]	🔴 47%	🔴 10%

🟢 **>50% yield, clean reaction**

🔴 **0-50% yield, not clean reaction**

Fig S3: Comparison of acids. Reactions were carried out on a 0.10 mmol scale in MeCN (0.25 M). Yields were determined by ^1H NMR using CH_2Br_2 (1.0 equiv.) as an internal standard. [a]Isolated yield.

Thianthrenation of arenes

Thianthrene–*S*–Oxide

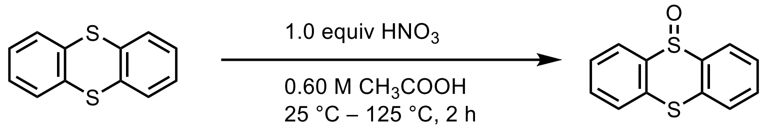

S1 **TTO**, 92%

A 1 L two-neck round-bottomed flask was charged with a Teflon-coated magnetic stirring bar. Thianthrene **S1** (50 g, 0. 23 mol, 1.0 equiv.) and acetic acid (0.60 mol/L, 0.39 L, 14 g, 0.23 mol, 1.0 equiv.) were added to the flask. The central neck of the flask is fitted with a reflux condenser with the outlet passing through a round-bottomed flask before entering a Drecshel bottle containing an aqueous sodium hydroxide solution. The side-neck of the flask was fitted with a 100 mL dropping funnel containing nitric acid (2.7 mol/L, 90 mL, 15 g, 0.24 mol, 1.0 equiv.). The reaction mixture was refluxed at 125 °C (in an oil bath) while the nitric acid was added dropwise over 90min. Afterwards, the mixture was refluxed for additional 15min. The yellow solution was cooled down to room temperature and poured into ice water (750 mL). The suspension was filtered and washed with cold water (3 × 100 mL). The solid was diluted in DCM, poured into a separating funnel and layers were separated. The organic phase was concentrated under reduced pressure by rotary evaporation. Then, the product was recrystallized from ethyl acetate. The resulting solution was filtered, the solid was dissolved in

DCM, precipitated with a large excess of hexanes, and filtered to give **TTO** as colorless solid (40 g, 75% yield). The spectra are in accordance with literature.[3]

NMR Spectroscopy:

^{1}H NMR (600 MHz, CD_3CN, 23 °C, δ): 7.86 (ddd, *J* = 7.8, 1.4, 0.5 Hz, 2H), 7.73 (ddd, *J* = 7.7, 1.1, 0.5 Hz, 2H), 7.62 (dd, *J* = 15.2, 1.1 Hz, 2H), 7.52 (ddd, *J* = 7.8, 7.4, 1.4 Hz, 2H).

^{13}C NMR (151 MHz, CD_3CN, 23 °C, δ): 142.6, 131.2, 130.3, 129.7, 129.3, 125.1.

HRMS-GC-EI(m/z) calc'd for $C_{12}H_{18}OS_2$ $[M]^+$, 232.0011; found 232.0010; deviation: +0.7 ppm.

tert-Butyloxycarbonyl (-Boc) protected phenylpiperazine derivative 2

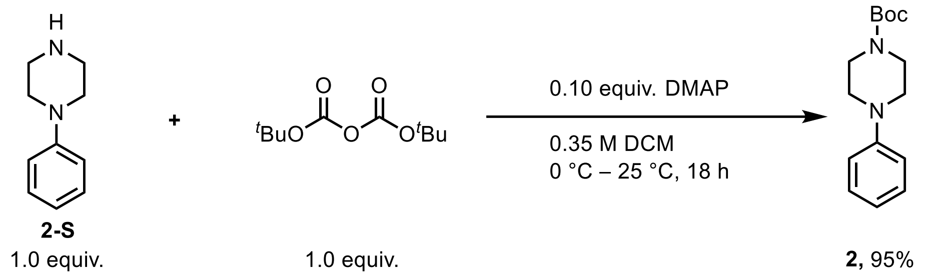

To a mixture of 1-phenylpiperazine **2-S** (0.94 mL, 1.0 g, 6.2 mmol, 1.0 equiv.) and DMAP (4-dimethylaminopyridine, 75 mg, 0.62 mmol, 0.10 equiv.) in DCM (15 mL, *c* = 0.35 M), a solution of Boc_2O (1.4 mL, 1.4 g, 6.2 mmol, 1.0 equiv.) in DCM (3 mL) was added dropwise at 0 °C over approximately 1 minute. The reaction mixture was stirred (400 rpm) at 0 °C for 20 minutes, the ice bath was removed, and the reaction mixture was stirred (400 rpm) at 25 °C for 18 h. Subsequently, the reaction mixture was diluted with DCM (10 mL) and water (10 mL). The mixture was transferred to a separatory funnel. The layers were separated, the organic phase was washed with water (10 mL), dried over Na_2SO_4, filtered and the solvent was removed under reduced pressure by rotary evaporation. The crude mixture was purified by flash column chromatography on silica gel (hexanes / EtOAc = 4:1 (v/v)) to give 1.5 g of the title compound **2** as colorless solid (95% yield).
R*f* = 0.35 (hexanes / EtOAc = 10:1 (v/v))

NMR Spectroscopy:

^{1}H NMR (500 MHz, $CDCl_3$, 23 °C, δ): 7.32 – 7.29 (m, 2H), 6.97 – 6.90 (m, 3H), 3.62 – 3.60 (m, 4H), 3.20 (t, *J* = 5.1 Hz, 4H), 1.52 (s, 9H).

^{13}C NMR (126 MHz, $CDCl_3$, 23 °C, δ): 154.9, 151.4, 129.3, 120.4, 116.8, 80.0, 49.6, 28.6.

HRMS-GC-EI(m/z) calc'd for $C_{15}H_{22}N_2O_2$ $[M]^+$, 262.1676; found 262.1680; deviation: –1.7 ppm.

tert-Butyloxycarbonyl (-Boc) protected phenylpiperazine-derived thianthrenium salt 2a

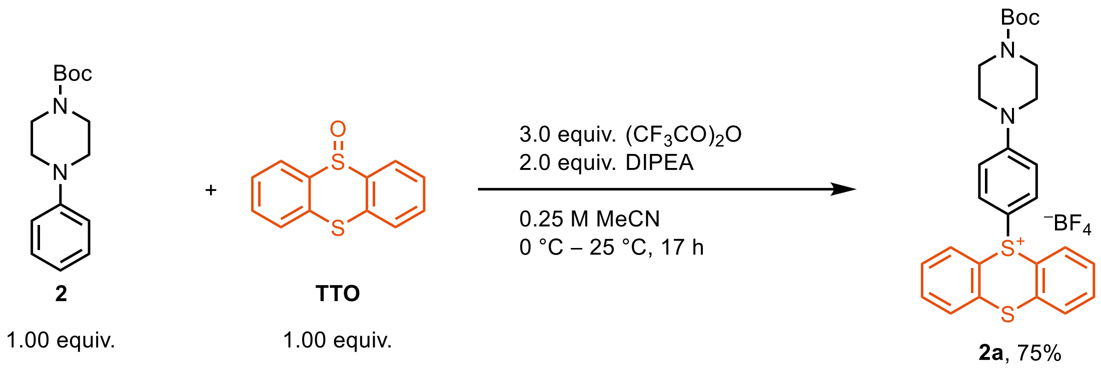

To a solution of *tert*-butyl 4-phenylpiperazine-1-carboxylate **2** (65.6 mg, 0.250 mmol, 1.00 equiv.) and TTO (thianthrene-*S*-oxide, 58.1 mg, 0.250 mmol, 1.00 equiv.) in MeCN (1.0 mL, *c* = 0.25 M), DIPEA (*N,N*-diisopropylethylamine, 87 µL, 65 mg, 0.5 mmol, 2.0 equiv.) was added under an ambient atmosphere. The suspension was cooled to 0 °C with an ice bath, and trifluoroacetic anhydride (104 µL, 158 mg, 0.750 mmol, 3.00 equiv.) was added dropwise over approximately 30 seconds at 0 °C. The reaction mixture was stirred (400 rpm) at 0 °C for 1 h, and then stirred at 25 °C for 16 h. The solvent was removed under reduced pressure by rotary evaporation and the residue was dissolved in DCM (10 mL). The resulting solution was washed with saturated aqueous $NaHCO_3$ solution (10 mL) and aqueous $NaBF_4$ solution (2 × 10 mL, 10% w/w). The DCM layer was dried over Na_2SO_4, filtered, and the solvent was removed under reduced pressure. The crude mixture was dissolved in DCM (3 mL) and Et_2O (30 mL) was added to obtain the product as red precipitate. The precipitate was filtered to give 106 mg of the title compound **2a** as colorless solid (75% yield).

R*f* = 0.43 (DCM / MeOH = 15:1 (v/v))

NMR Spectroscopy:

^{1}H NMR (500 MHz, $CDCl_3$, 23 °C, δ): 8.15 (d, *J* = 7.8 Hz, 2H), 7.79 (d, *J* = 6.6 Hz, 2H), 7.73 (d, *J* = 7.5 Hz, 2H), 7.63 (s, 2H), 7.27 (d, *J* = 9.2 Hz, 2H), 6.88 (d, *J* = 8.9 Hz, 2H), 3.60 – 3.43 (m, 4H), 3.38 – 3.15 (m, 4H), 1.42 (s, 9H).

^{13}C NMR (126 MHz, $CDCl_3$, 23 °C, δ): 154.5, 154.0, 135.4, 135.3, 134.4, 133.2, 131.3, 130.2, 130.2, 120.8, 115.7, 107.2, 80.2, 53.6, 46.5, 28.4.

^{19}F NMR (471 MHz, $CDCl_3$, 23 °C, δ): –151.6 (bs), –151.7 (bs).

HRMS-ESI(m/z) calc'd for $C_{27}H_{29}N_2O_2S_2$ [M–BF_4]$^+$, 477.1665; found, 477.1667; deviation: –0.3 ppm.

1-Methyl-4-phenylpiperazine 3

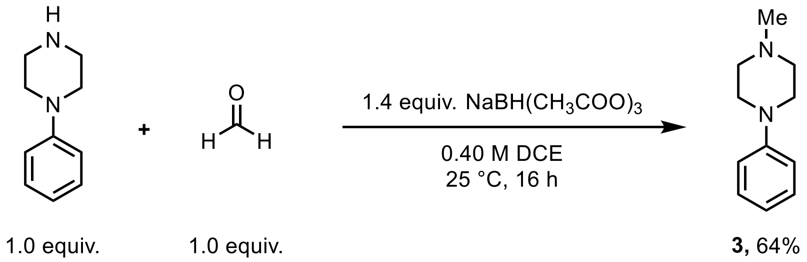

To a solution *N*-phenylpiperazine (0.94 mL, 1.0 g, 6.2 mmol, 1.0 equiv.) in DCE (15 mL, *c* = 0.40 M), 37% aq. solution of formaldehyde (0.46 mL, 0.50 g, 6.2 mmol, 1.0 equiv.) was added dropwise over 1 minute at 25 °C followed by addition of $NaBH(OAC)_3$ (1.8 g, 8.6 mmol, 1.4 equiv.) in one portion. The reaction mixture was stirred (400 rpm) for 16 h at 25 °C and saturated aqueous solution of $NaHCO_3$ (10 mL) was added. The aqueous phase was extracted with EtOAc (3 × 10 mL). Combined organic phases was dried over Na_2SO_4, filtered and concentrated under reduced pressure by rotary evaporation. Purification by flash column chromatography on silica gel (DCM / MeOH = 20:1) afforded 0.7 g of the title compound **3** as colorless oil (64% yield).

R*f* = 0.38 (DCM / MeOH = 20:1 (v/v))

NMR Spectroscopy:

^{1}H NMR (500 MHz, $CDCl_3$, 23 °C, δ): 7.26 (dd, *J* = 8.9, 7.3 Hz, 2H), 6.93 (d, *J* = 7.8 Hz, 2H), 6.85 (t, *J* = 7.2 Hz, 1H), 3.23 – 3.17 (m, 4H), 2.59 – 2.53 (m, 4H), 2.34 (s, 3H).

^{13}C NMR (126 MHz, $CDCl_3$, 23 °C, δ): 151.3, 129.1, 119.7, 116.1, 55.2, 49.1, 46.2.

HRMS-GC-EI(m/z) calc'd for $C_{11}H_{16}N_2$ $[M]^+$, 176.1308; found, 176.1311; deviation: –1.6 ppm.

1-Methyl-4-phenylpiperazine-derived thianthrenium salt 3a

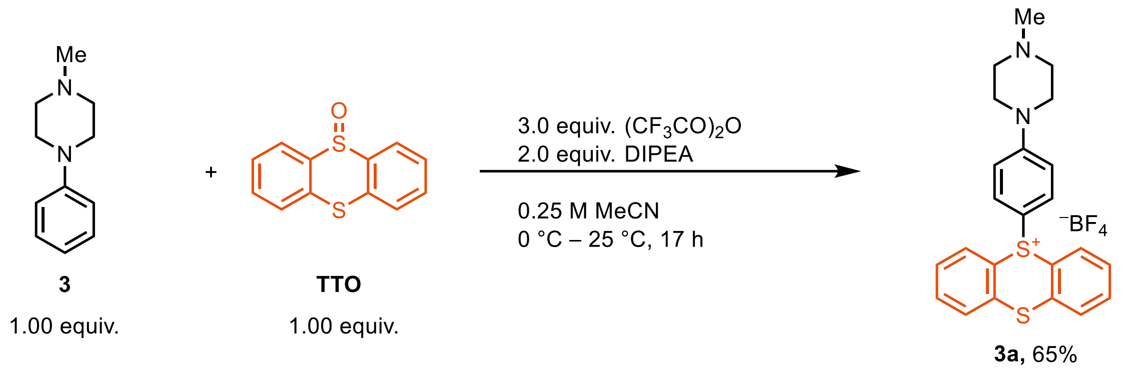

To a solution of 1-methyl-4-phenylpiperazine **3** (44.1 mg, 0.250 mmol, 1.00 equiv.) and TTO (thianthrene-*S*-oxide, 58.1 mg, 0.250 mmol, 1.00 equiv.) in MeCN (1.0 mL, *c* = 0.25 M), DIPEA (*N,N*-diisopropylethylamine, 87 µL, 65 mg, 0.5 mmol, 2.0 equiv.) was added under an ambient atmosphere. The suspension was cooled to 0 °C with an ice bath, and trifluoroacetic anhydride (104 µL, 158 mg, 0.750 mmol, 3.00 equiv.) was added dropwise at 0 °C over approximately 30 seconds. The reaction mixture was stirred (400 rpm) at 0 °C for 1 h, and then stirred at 25 °C for 16 h. The solvent was removed under reduced pressure by rotary evaporation and the residue was dissolved in DCM (10 mL). The resulting solution was washed with saturated aqueous $NaHCO_3$ solution (10 mL) and aqueous $NaBF_4$ solution (2 × 10 mL, 10% w/w). Purification by flash column chromatography on silica gel (DCM / *i*-PrOH / Et_3N = 40:3:0.3 (v/v)) afforded 77.6 mg of the title compound **3a** as colorless solid (65% yield).

R*f* = 0.28 (DCM / MeOH = 10:1 (v/v))

NMR Spectroscopy:

^{1}H NMR (500 MHz, CD_3CN, 23 °C, δ): 8.08 (dd, *J* = 7.9, 1.5 Hz, 2H), 7.93 (dd, *J* = 7.9, 1.4 Hz, 2H), 7.82 (td, *J* = 7.7, 1.4 Hz, 2H), 7.73 (td, *J* = 7.7, 1.4 Hz, 2H), 7.23 – 7.16 (m, 2H), 7.01 – 6.92 (m, 2H), 3.38 – 3.28 (m, 4H), 2.55 – 2.43 (m, 4H), 2.28 (s, 3H)

^{13}C NMR (126 MHz, CD_3CN, 23 °C, δ): 155.3, 136.2, 135.4, 134.1, 132.1, 131.4, 130.9, 122.0, 116.3, 107.5, 55.1, 47.3, 46.0.

^{19}F NMR (471 MHz, $CDCl_3$, 23 °C, δ): –151.6 (bs), –151.7 (bs).

HRMS-ESI(m/z) calc'd for $C_{23}H_{22}N_2S_2$ $[M–BF_4]^+$, 391.1297; found, 391.1295; deviation: +0.5 ppm.

1,3-Dimethoxybenzene-derived thianthrenium salt 4a

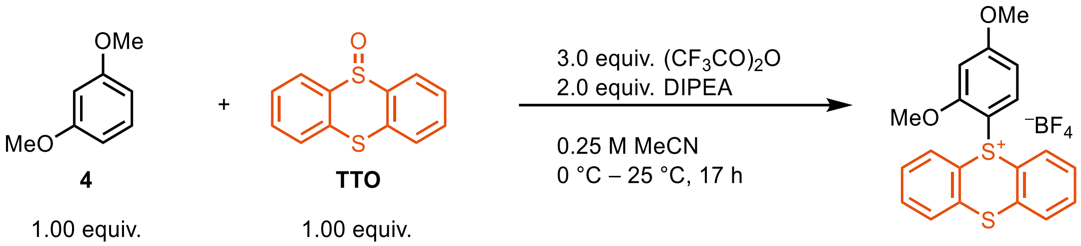

4 1.00 equiv. **TTO** 1.00 equiv. **4a,** 88%

To a solution of 1,3-dimethoxybenzene **4** (69.1 mg, 0.500 mmol, 1.00 equiv.) and TTO (thianthrene-*S*-oxide, 116 mg, 0.500 mmol, 1.00 equiv.) in MeCN (2.0 mL, *c* = 0.25 M), DIPEA (*N*,*N*-diisopropylethylamine, 174 µL, 129 mg, 1.00 mmol, 2.00 equiv.) was added under an ambient atmosphere. The suspension was cooled to 0 °C with an ice bath, and trifluoroacetic anhydride (209 µL, 315 mg, 1.50 mmol, 3.00 equiv.) was added dropwise over approximately 30 seconds at 0 °C. The reaction mixture was stirred (400 rpm) at 0 °C for 1 h, and then stirred at 25 °C for 16 h. The solvent was removed under reduced pressure by rotary evaporation and the residue was dissolved in DCM (20 mL). The resulting solution was washed with saturated aqueous $NaHCO_3$ solution (15 mL) and aqueous $NaBF_4$ solution (2 × 15 mL, 10% w/w). Crude mixture was dissolved in DCM (5 mL) and Et_2O (50 mL) was added to obtain the product as white precipitate. The precipitate was filtered to give 193 mg of the title compound **4a** as colorless solid (88% yield).

R_f = 0.19 (DCM / MeOH = 15:1 (v/v)).

NMR Spectroscopy:

^{1}H NMR (600 MHz, CD_3CN, 23 °C, δ): 8.17 (ddd, *J* = 8.0, 1.4, 0.4 Hz, 2H), 7.94 (ddd, *J* = 7.9, 1.3, 0.4 Hz, 2H), 7.82 (ddd, *J* = 8.0, 7.4, 1.4 Hz, 2H), 7.73 (ddd, *J* = 8.0, 7.4, 1.3 Hz, 2H), 6.72 (d, *J* = 2.5 Hz, 1H), 6.70 (d, *J* = 9.1 Hz, 1H), 6.55 (dd, *J* = 9.1, 2.5 Hz, 1H), 3.92 (s, 3H), 3.81 (s, 3H).

^{13}C NMR (151 MHz, CD_3CN, 23 °C, δ): 167.4, 160.7, 137.8, 135.5, 135.2, 132.1, 131.4, 130.7, 119.0, 108.5, 101.4, 99.8, 57.9, 57.1.

^{19}F NMR (565 MHz, $CDCl_3$, 23 °C, δ): –151.7 (bs), –151.8 (bs).

HRMS-ESI(m/z) calc'd for $C_{20}H_{17}O_2S_2$ $[M–BF_4]^+$, 353.0665; found, 353.0661; deviation: +1.1 ppm.

4-Phenylpiperazine-derived thianthrenium salt 5a

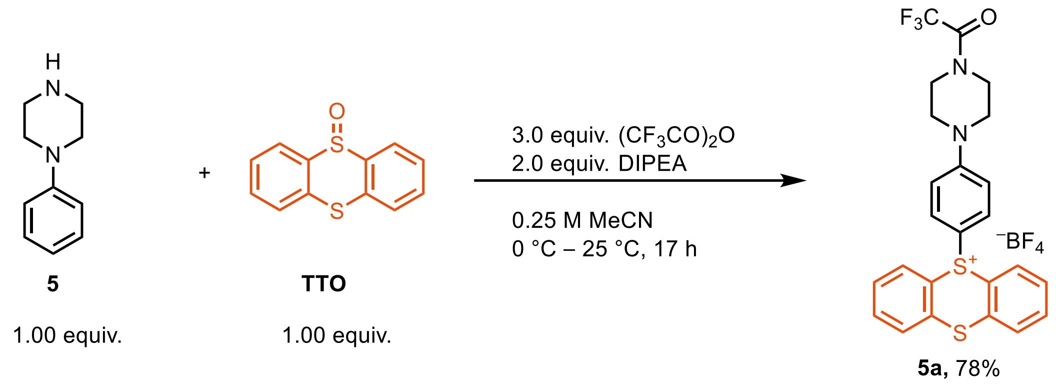

To a solution of 4-phenylpiperazine **5** (40.6 mg, 0.250 mmol, 1.00 equiv.) and TTO (thianthrene-*S*-oxide, 58.1 mg, 0.250 mmol, 1.00 equiv.) in MeCN (1.0 mL, *c* = 0.25 M), DIPEA (*N,N*-diisopropylethylamine, 87 µL, 65 mg, 0.5 mmol, 2.0 equiv.) was added under an ambient atmosphere. The suspension was cooled to 0 °C with an ice bath, and trifluoroacetic anhydride (104 µL, 158 mg, 0.750 mmol, 3.00 equiv.) was added dropwise over approximately 30 seconds at 0 °C. The reaction mixture was stirred (400 rpm) at 0 °C for 1 h, and then stirred at 25 °C for 16 h. The solvent was removed under reduced pressure by rotary evaporation and the residue was dissolved in DCM (10 mL). The resulting solution was washed with saturated aqueous $NaHCO_3$ solution (10 mL) and aqueous $NaBF_4$ solution (2 × 10 mL, 10% w/w). Purification by flash column chromatography on silica gel (DCM / *i*-PrOH = 30:1 (v/v)) afforded 110 mg of the title compound **5a** as colorless solid (78% yield).

R*f* = 0.32 (DCM / MeOH = 15:1 (v/v))

NMR Spectroscopy:

^{1}H NMR (600 MHz, CD_3CN, 23 °C, δ): 8.15 (dd, *J* = 8.0, 1.3 Hz, 2H), 7.92 (dd, *J* = 7.9, 1.3 Hz, 2H), 7.82 (td, *J* = 7.7, 1.4 Hz, 2H), 7.74 (td, *J* = 7.7, 1.3 Hz, 2H), 7.19 (d, *J* = 9.3 Hz, 2H), 6.95 (d, *J* = 9.5 Hz, 2H), 3.70 (dt, *J* = 5.6, 3.4 Hz, 4H), 3.42 – 3.39 (m, 4H).

^{13}C NMR (151 MHz, CD_3CN, 23 °C, δ): 156.1 (q, *J* = 35.6 Hz), 154.6, 136.5, 135.55, 134.5, 131.8, 131.4, 130.9, 121.4, 117.4 (q, *J* = 287.6 Hz), 116.5, 109.1, 47.3, 46.5, 45.5 (q, *J* = 3.5 Hz), 43.5.

^{19}F NMR (565 MHz, CD_3CN, 23 °C, δ): –69.9 (s, 3F), {–151.5 (bs), –151.5 (bs), 4F}.

HRMS-ESI(m/z) calc'd for $C_{24}H_{20}F_3N_2O_1S_2$ $[M–BF_4]^+$, 473.0964; found, 473.0966; deviation: –0.5 ppm.

N-Phenylmorpholine-derived thianthrenium salt 6a

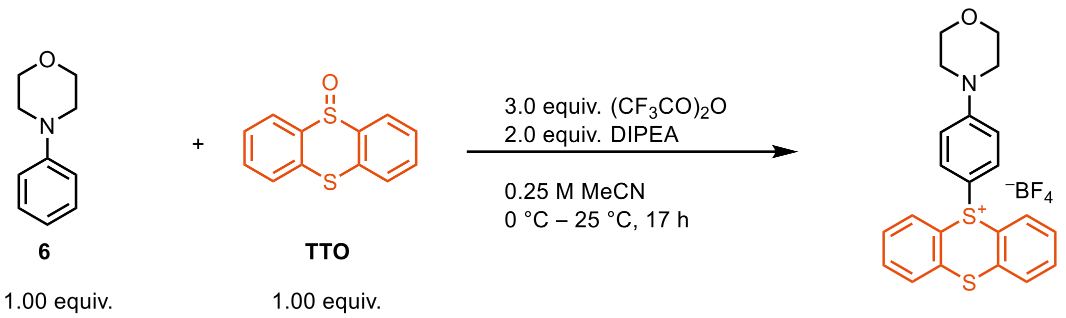

6 1.00 equiv. **TTO** 1.00 equiv. **6a,** 71%

To a solution of *N*-phenylmorpholine **6** (32.6 mg, 0.200 mmol, 1.00 equiv.) and TTO (thianthrene-*S*-oxide, 46.4 mg, 0.200 mmol, 1.00 equiv.) in MeCN (0.80 mL, *c* = 0.25 M), DIPEA (*N,N*-diisopropylethylamine, 70 µL, 52 mg, 0.4 mmol, 2.0 equiv.) was added under an ambient atmosphere. The suspension was cooled to 0 °C with an ice bath, and trifluoroacetic anhydride (83 µL, 0.13 g, 0.60 mmol, 3.0 equiv.) was added dropwise over approximately 30 seconds at 0 °C. The reaction mixture was stirred (400 rpm) at 0 °C for 1 h, and then stirred at 25 °C for 16 h. The solvent was removed under reduced pressure by rotary evaporation and the residue was dissolved in DCM (10 mL). The resulting solution was washed with saturated aqueous $NaHCO_3$ solution (10 mL) and aqueous $NaBF_4$ solution (2 × 10 mL, 10% w/w). The crude mixture was dissolved in DCM (2 mL) and Et_2O (20 mL) was added to obtain the product as white precipitate. The precipitate was filtered to give 65.8 mg of the title compound **6a** as colorless solid (71% yield).

R_f = 0.22 (DCM / MeOH = 15:1 (v/v)).

NMR Spectroscopy:

^{1}H NMR (600 MHz, CD_3CN, 23 °C, δ): 8.10 (dd, *J* = 8.0, 1.4 Hz, 2H), 7.93 (dd, *J* = 8.0, 1.4 Hz, 2H), 7.86 – 7.80 (m, 2H), 7.77 – 7.70 (m, 2H), 7.20 (d, *J* = 9.4 Hz, 2H), 6.96 (d, *J* = 9.4 Hz, 2H), 3.76 – 3.68 (m, 4H), 3.30 – 3.17 (m, 4H).

^{13}C NMR (151 MHz, CD_3CN, 23 °C, δ): 155.5, 136.4, 135.4, 134.2, 131.9, 131.4, 130.9, 121.8, 116.1, 108.2, 66.9, 47.7.

^{19}F NMR (565 MHz, CD_3CN, 23 °C, δ): –151.7 (bs), –151.8 (bs).

HRMS-ESI(m/z) calc'd for $C_{22}H_{20}N_1O_1S_2$ $[M–BF_4]^+$, 378.0981; found, 378.0980; deviation: +0.3 ppm.

1-Phenylpyrrolidine-derived thianthrenium salt 7a

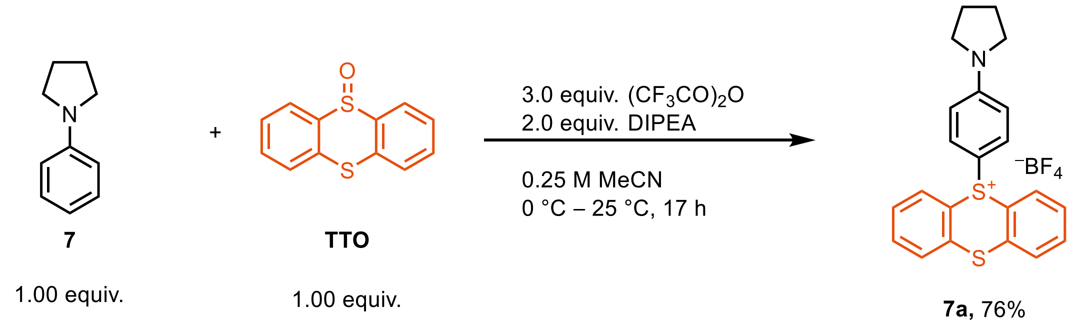

To a solution of 1-phenylpyrrolidine **7** (73.6 mg, 0.500 mmol, 1.00 equiv.) and TTO (thianthrene-*S*-oxide, 116 mg, 0.500 mmol, 1.00 equiv.) in MeCN (2.0 mL, *c* = 0.25 M), DIPEA (*N,N*-diisopropylethylamine, 174 µL, 129 mg, 1.00 mmol, 2.00 equiv.) was added under an ambient atmosphere. The suspension was cooled to 0 °C with an ice bath, and trifluoroacetic anhydride (209 µL, 315 mg, 1.50 mmol, 3.00 equiv.) was added dropwise over approximately 30 seconds at 0 °C. The reaction mixture was stirred (400 rpm) at 0 °C for 1 h, and then stirred at 25 °C for 16 h. The solvent was removed under reduced pressure by rotary evaporation and the residue was dissolved in DCM (20 mL). The resulting solution was washed with saturated aqueous $NaHCO_3$ solution (15 mL) and aqueous $NaBF_4$ solution (2 × 15 mL, 10% w/w). Purification by flash column chromatography on silica gel (DCM / *i*-PrOH / Et_3N = 30:3:0.3 (v/v)) afforded 180 mg of the title compound **7a** as colorless solid (76% yield).

R_f = 0.44 (DCM / MeOH = 15:1 (v/v)).

NMR Spectroscopy:

^{1}H NMR (500 MHz, CD_3CN, 23 °C, δ): 7.93 (dd, *J* = 8.1, 1.4 Hz, 2H), 7.89 (dd, *J* = 7.9, 1.2 Hz, 2H), 7.77 (td, *J* = 7.6, 1.4 Hz, 2H), 7.68 (td, *J* = 7.6, 1.4 Hz, 2H), 7.35 – 7.23 (m, 2H), 6.68 – 6.60 (m, 2H), 3.33 – 3.21 (m, 4H), 1.99 – 1.95 (m, 4H).

^{13}C NMR (126 MHz, CD_3CN, 23 °C, δ): 152.6, 135.5, 134.9, 133.0, 133.0, 131.2, 130.9, 123.5, 114.4, 102.7, 48.6, 26.0.

^{19}F NMR (471 MHz, $CDCl_3$, 23 °C, δ): –151.4 (bs), –151.5 (bs).

HRMS-ESI(m/z) calc'd for $C_{22}H_{20}N_1S_2$ $[M–BF_4]^+$, 362.1032; found, 362.1030; deviation: +0.4 ppm.

1,3,5-Trimethoxybenzene-derived thianthrenium salt 8a

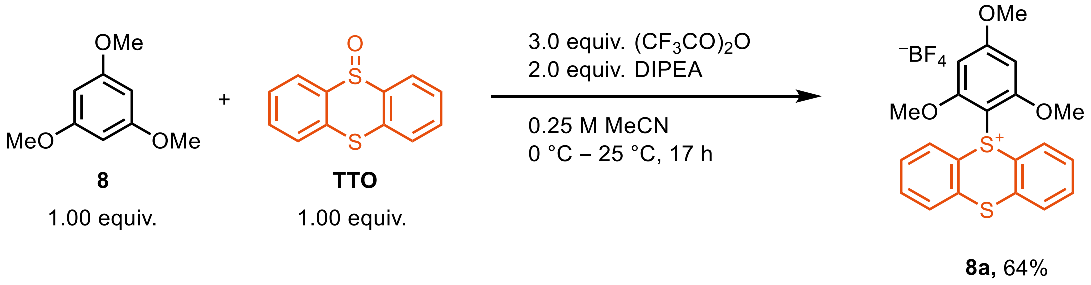

To a solution of 1,3,5-trimethoxybenzene **8** (84.1 mg, 0.500 mmol, 1.00 equiv.) and TTO (thianthrene-*S*-oxide, 116 mg, 0.500 mmol, 1.00 equiv.) in MeCN (2.0 mL, *c* = 0.25 M), DIPEA (*N,N*-diisopropylethylamine, 174 µL, 129 mg, 1.00 mmol, 2.00 equiv.) was added under an ambient atmosphere. The suspension was cooled to 0 °C with an ice bath, and trifluoroacetic anhydride (209 µL, 315 mg, 1.50 mmol, 3.00 equiv.) was added dropwise over approximately 30 seconds at 0 °C. The reaction mixture was stirred (400 rpm) at 0 °C for 1 h, and then stirred at 25 °C for 16 h. The solvent was removed under reduced pressure by rotary evaporation and the residue was dissolved in DCM (10 mL). The resulting solution was washed with saturated aqueous $NaHCO_3$ solution (10 mL) and aqueous $NaBF_4$ solution (2 × 10 mL, 10% w/w). Purification by flash column chromatography on silica gel (DCM / MeOH = 30:1 (v/v)) afforded 150 mg of the title compound **8a** as colorless solid (64% yield).

R*f* = 0.17 (DCM / MeOH = 15:1 (v/v)).

NMR Spectroscopy:

^{1}H NMR (500 MHz, CD_3CN, 23 °C, δ): 7.86 (ddt, *J* = 8.0, 2.3, 1.5 Hz, 2H), 7.68 (tq, *J* = 7.6, 1.1 Hz, 2H), 7.57 – 7.49 (m, 2H), 7.35 (dd, *J* = 8.2, 1.2 Hz, 1H), 6.54 (s, 2H), 4.01 (s, 3H), 3.74 (s, 6H).

^{13}C NMR (126 MHz, CD_3CN, 23 °C, δ): 171.3, 164.7, 133.6, 132.4, 130.8, 130.5, 128.1, 125.5, 94.5, 81.8, 57.9, 57.7.

^{19}F NMR (471 MHz, CD_3CN, 23 °C, δ): –151.8 (bs), –151.9 (bs)

HRMS-ESI(m/z) calc'd for $C_{21}H_{19}O_3S_2$ $[M–BF_4]^+$, 383.0770; found, 383.0771; deviation: –0.2 ppm.

N-Phenylpiperazine derivative 9

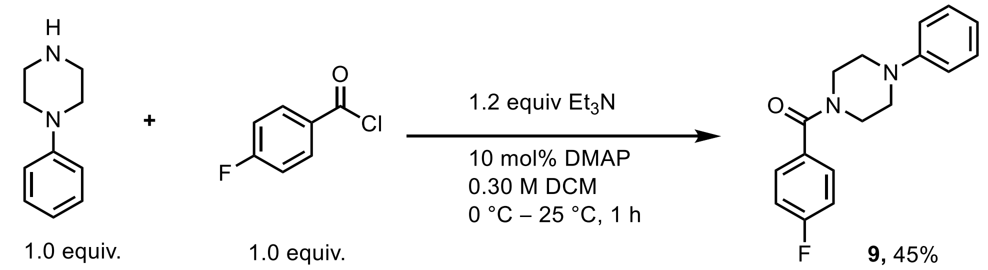

4-fluorobenzoyl chloride (0.36 mL, 0.48 g, 3.0 mmol, 1.0 equiv.) was dissolved in DCM (5 mL) and the solution was cooled to 0 °C with an ice bath. A solution of *N*-phenylpiperizine (0.50 mL, 0.54 g, 3.3 mmol, 1.1 equiv.), triethylamine (0.50 mL, 0.36 g, 3.6 mmol, 1.2 equiv.), and DMAP (3.7 mg, 0.030 mmol, 10 mol%) in DCM (4 mL) was added to the cooled solution of 4-fluorobenzoyl chloride. The reaction mixture was allowed to warm to 25 °C over 1 h. Then the reaction mixture was diluted with DCM (10 mL) and washed with H_2O (10 mL), 1.0 N HCl (2 × 10 mL), and saturated aqueous $NaHCO_3$ solution (10 mL). The organic layer was dried over Na_2SO_4, filtered, and concentrated under reduced pressure by rotary evaporation. Purification by flash column chromatography on silica gel (hexanes / EtOAc = 1:1 to EtOAc only (v/v)) afforded 0.38 g of the title compound **9** as colorless solid (45% yield).

R*f* = 0.45 (hexanes / EtOAc = 1:1 (v/v))

NMR Spectroscopy:

^{1}H NMR (600 MHz, $CDCl_3$, 23 °C, δ): 7.48 – 7.43 (m, 2H), 7.32 – 7.27 (m, 2H), 7.16 – 7.09 (m, 2H), 6.96 – 6.89 (m, 3H), 3.91 (bs, 2H), 3.61 (bs, 2H), 3.19 (bs, 4H).

^{13}C NMR (151 MHz, $CDCl_3$, 23 °C, δ): 169.5, 163.6 (d, *J* = 249.9 Hz), 151.0, 131.7 (d, *J* = 3.5 Hz), 129.6 (d, *J* = 8.3 Hz), 129.4, 120.8, 116.8, 115.8, 115.7, 49.8.

^{19}F NMR (565 MHz, $CDCl_3$, 23 °C, δ): –110.0.

HRMS-GC-EI(m/z) calc'd for $C_{17}H_{17}N_2OF$ $[M]^+$, 284.1319; found, 284.1322; deviation: –1.1 ppm.

1-Phenylpiperazine derivative-derived thianthrenium salt 9a

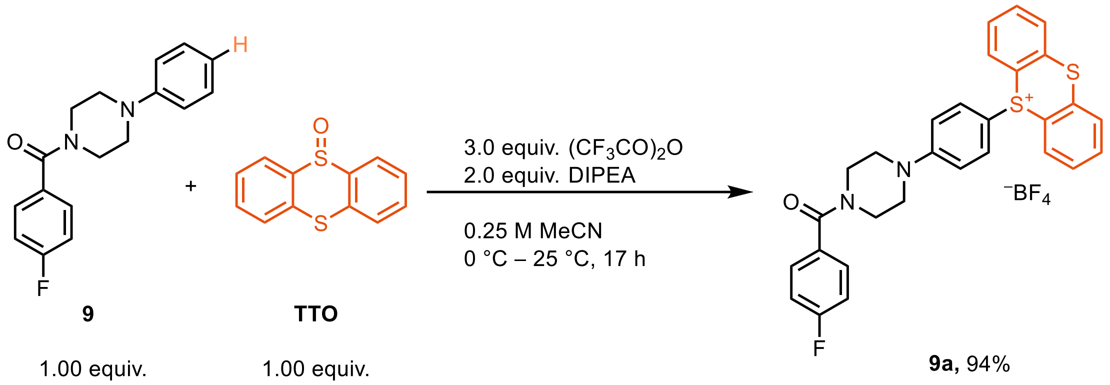

9 1.00 equiv. **TTO** 1.00 equiv. **9a,** 94%

To a solution of phenylpiperazine derivative **9** (71.1 mg, 0.250 mmol, 1.00 equiv.) and TTO (thianthrene-*S*-oxide, 58.1 mg, 0.250 mmol, 1.00 equiv.) in MeCN (1.0 mL, *c* = 0.25 M), DIPEA (*N,N*-diisopropylethylamine, 87 µL, 65 mg, 0.50 mmol, 2.0 equiv.) was added under an ambient atmosphere. The suspension was cooled to 0 °C with an ice bath, and trifluoroacetic anhydride (104 µL, 158 mg, 0.750 mmol, 3.00 equiv.) was added dropwise over approximately 30 seconds at 0 °C. The reaction mixture was stirred (400 rpm) at 0 °C for 1 h, and then stirred at 25 °C for 16 h. The solvent was removed under reduced pressure by rotary evaporation and the residue was dissolved in DCM (10 mL). The resulting solution was washed with saturated aqueous $NaHCO_3$ solution (10 mL) and aqueous $NaBF_4$ solution (2 × 10 mL, 10% w/w). Purification by flash column chromatography on silica gel (DCM / MeOH = 30:1 (v/v)) afforded 138 mg of the title compound **9a** as colorless solid (94% yield).

R_f = 0.36 (DCM / MeOH = 15:1 (v/v)).

NMR Spectroscopy:

^{1}H NMR (600 MHz, $CDCl_3$, 23 °C, δ): 8.13 (dd, *J* = 8.0, 1.3 Hz, 2H), 7.79 (dd, *J* = 7.9, 1.3 Hz, 2H), 7.75 – 7.71 (m, 2H), 7.64 (td, *J* = 7.7, 1.4 Hz, 2H), 7.45 – 7.37 (m, 2H), 7.26 – 7.21 (m, 2H), 7.09 – 7.01 (m, 2H), 6.95 – 6.86 (m, 2H), 4.00 – 3.05 (m, 8H).

^{13}C NMR (151 MHz, $CDCl_3$, 23 °C, δ) 169.7, 163.0 (d, *J* = 250.2 Hz), 153.9, 135.4, 134.4, 133.2, 131.3, 131.1 (d, *J* = 3.2 Hz), 130.3, 130.2, 129.7 (d, *J* = 8.6 Hz), 120.9 (d, *J* = 1.9 Hz), 116.0, 115.8 (d, *J* = 21.6 Hz), 107.5, 55.4, 43.5.

^{19}F NMR (565 MHz, $CDCl_3$, 23 °C, δ): –109.6 (s, 1F), {–151.3 (bs), –151.3 (bs), 4F}.

HRMS-ESI(m/z) calc'd for $C_{29}H_{24}FO_1N_2S_2$ $[M–BF_4]^+$, 499.1309; found, 499.1305; deviation: +0.8 ppm.

(±)-Fenoprefen methyl ester 10

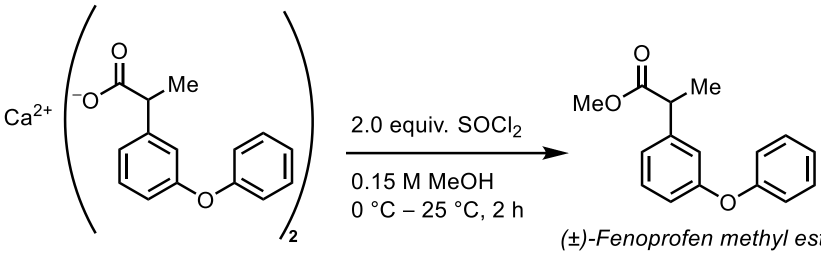

10-S

(±)-Fenoprofen methyl ester
10
95%

Prepared according to the reported procedure and spectra are in accordance with literature.[4] A 100 mL round-bottomed flask equipped with a Teflon-coated magnetic stirring bar was charged with fenoprofen calcium salt (**10-S**) (783 mg, 1.50 mmol, 1.00 equiv.). The solid was dissolved in MeOH (10 mL, *c* = 0.15 M) and the solution was cooled to 0 °C with an ice bath. Thionyl chloride (1.0 mL, 15 mmol, 10 equiv.) was added at 0 °C. The reaction was allowed to warm to 23 °C and stirred for 2 h. Saturated aqueous $NaHCO_3$ solution (5 ml) was added to the reaction mixture, and MeOH was evaporated under reduced pressure by rotary evaporation. The resulting mixture was extracted with EtOAc (3 × 10 mL), the combined organic layers were washed with brine, dried over $MgSO_4$, and concentrated under reduced pressure by rotary evaporation. The residue was washed with diethylether to afford **(±)-10** (731 mg, 95% yield) as colorless solid.

NMR Spectroscopy:

^{1}H NMR (500 MHz, $CDCl_3$, 23 °C, δ): 7.37 – 7.32 (m, 2H), 7.28 (t, *J* = 7.9 Hz, 1H), 7.12 (tt, *J* = 7.3, 1.1 Hz, 1H), 7.06 – 6.98 (m, 4H), 6.89 (ddd, *J* = 8.2, 2.4, 1.0 Hz, 1H), 3.71 (q, *J* = 7.2 Hz, 1H), 3.67 (s, 3H), 1.50 (d, *J* = 7.2 Hz, 3H).

^{13}C NMR (126 MHz, $CDCl_3$, 23 °C, δ) 174.8, 157.6, 157.1, 142.6, 129.9, 129., 123.4, 122.4, 119.11, 118.2, 117.4, 52.2, 45.4, 18.6.

(±)-Fenoprefen methyl ester-derived thianthrenium salt 10a

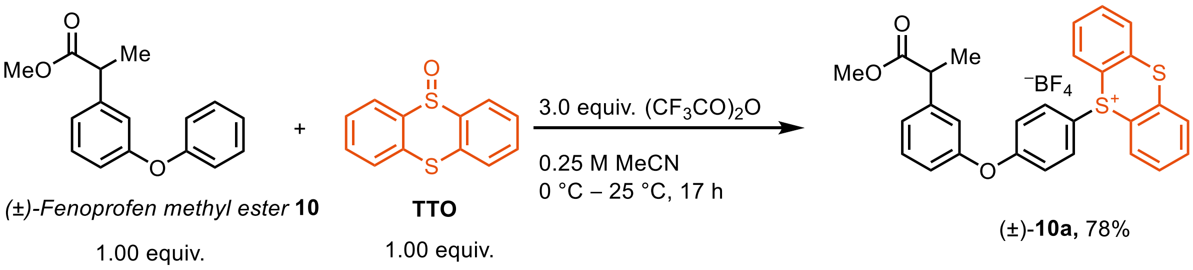

(±)-Fenoprofen methyl ester **10**
1.00 equiv.

TTO
1.00 equiv.

(±)-**10a,** 78%

To a solution of (±)-fenoprofen methyl ester **10** (440 mg, 1.72 mmol, 1.00 equiv.) and TTO (thianthrene-*S*-oxide, 398 mg, 1.72 mmol, 1.00 equiv.) in MeCN (7.0 mL, *c* = 0.25 M), trifluoroacetic

anhydride (0.72 mL, 1.1 g, 5.2 mmol, 3.0 equiv.) was added at 0 °C, under an ambient atmosphere. The reaction mixture was stirred at 0 °C for 1 h, and then stirred (400 rpm) at 25 °C for 16 h. The solvent was removed under reduced pressure by rotary evaporation and the residue was dissolved in DCM (20 mL). The resulting solution was washed with saturated aqueous $NaHCO_3$ solution (15 mL) and aqueous $NaBF_4$ solution (2 × 15 mL, 10% w/w). The DCM layer was dried over Na_2SO_4, filtered, and the solvent was removed under reduced pressure by rotary evaporation. Purification by flash column chromatography on silica gel (DCM to DCM / MeOH = 35:1 (v/v)) afforded 749 mg of the title compound **(±)-10a** as colorless solid (78% yield).

R*f* = 0.37 (DCM / MeOH = 15:1 (v/v)).

NMR Spectroscopy:

^{1}H NMR (500 MHz, $CDCl_3$, 23 ºC, δ): δ 8.32 (dd, *J* = 7.9, 1.4 Hz, 2H), 7.96 (dd, *J* = 7.9, 1.4 Hz, 2H), 7.88 (td, *J* = 7.8, 1.4 Hz, 2H), 7.79 (td, *J* = 7.7, 1.4 Hz, 2H), 7.37 (t, *J* = 7.9 Hz, 1H), 7.16 (dd, *J* = 8.9, 6.7 Hz, 3H), 6.99 (dd, *J* = 9.0, 2.1 Hz, 3H), 6.93 (dd, *J* = 7.6, 3.1 Hz, 1H), 3.75 (q, *J* = 7.2 Hz, 1H), 3.58 (s, 3H), 1.40 (d, *J* = 7.2 Hz, 3H).

^{13}C NMR (126 MHz, $CDCl_3$, 23 °C, δ):175.3, 162.9, 155.8, 144.7, 137.3, 136.0, 135.6, 131.6, 131.6, 131.5, 131.0, 125.6, 120.4, 120.0, 120.0, 119.8, 116.8, 52.6, 45.7, 18.9.

^{19}F NMR (471 MHz, $CDCl_3$, 23 °C, δ): –151.1 (bs), –151.1 (bs)

HRMS-ESI(m/z) calc'd for $C_{28}H_{23}O_3S_2$ $[M–BF_4]^+$, 471.1083; found, 471.1083; deviation: +0.1 ppm.

1,2-Dimethoxybenzene-derived thianthrenium salt 11a

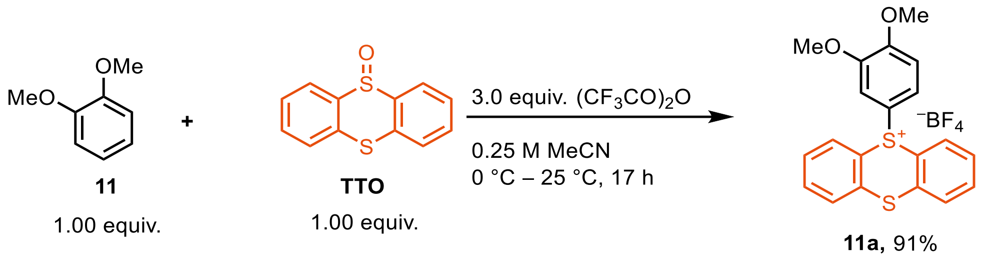

To a solution of 1,2-dimethoxybenzene **11** (69.1 mg, 0.500 mmol, 1.00 equiv.) and TTO (thianthrene-*S*-oxide, 116 mg, 0.500 mmol, 1.00 equiv.) in MeCN (2.0 ml, *c* = 0.25 M) trifluoroacetic anhydride (0.21 mL, 0.32 g, 1.5 mmol, 3.0 equiv.) was added at 0 °C, under an ambient atmosphere. The reaction mixture was stirred (400 rpm) at 0 °C for 1 h, and then stirred at 25 °C for 16 h. The solvent was removed under reduced pressure by rotary evaporation and the residue was dissolved in DCM (10 ml). The resulting solution was washed with saturated aqueous $NaHCO_3$ solution (10 mL) and aqueous $NaBF_4$ solution (2 × 10 mL, 10% w/w). The DCM layer was dried over Na_2SO_4, filtered, and the solvent was removed under reduced pressure. Purification by flash column chromatography on silica gel (DCM / MeOH = 30:1 to 10:1 (v/v)) afforded 200 mg of the title compound **11a** as a colorless solid (91% yield).

R*f* = 0.42 (DCM / MeOH = 15:1 (v/v)).

NMR Spectroscopy:

^{1}H NMR (500 MHz, CD_3CN, 23 °C, δ): 8.22 (d, *J* = 9.5 Hz, 2H), 7.95 (d, *J* = 9.5 Hz, 2H), 7.85 (t, *J* = 6.9 Hz, 2H), 7.80 – 7.73 (m, 2H), 7.01 (d, *J* = 8.9 Hz, 1H), 6.88 (dd, *J* = 8.8, 2.5 Hz, 1H), 6.76 (d, *J* = 2.6 Hz, 1H), 3.82 (s, 3H), 3.65 (s, 3H)

^{13}C NMR (126 MHz, CD_3CN, 23 °C, δ): 154.8, 151.9, 136.7, 135.7, 134.9, 131.5, 130.9, 124.3, 120.8, 113.9, 113.5, 112.0, 57.0, 56.9.

^{19}F NMR (471 MHz, $CDCl_3$, 23 °C, δ): –151.5 (bs), –151.6 (bs).

HRMS-ESI(m/z) calc'd for $C_{20}H_{17}O_2S_2$ $[M–BF_4]^+$, 353.0665; found, 353.0663; deviation: +0.4 ppm.

Indole-derived thianthrenium salt 12-Ia

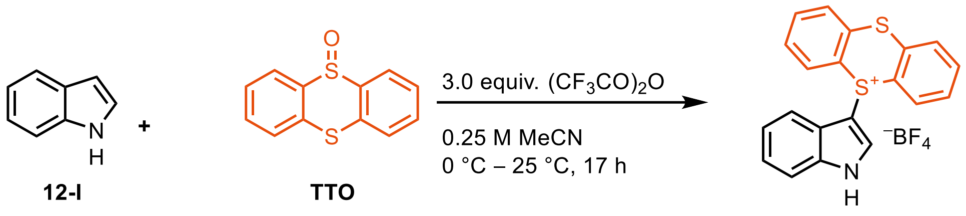

12-I 1.00 equiv. **TTO** 1.00 equiv. **12-Ia,** 83%

To a solution of indole **12-I** (117 mg, 0.500 mmol, 1.00 equiv.) and TTO (thianthrene-*S*-oxide, 116 mg, 0.500 mmol, 1.00 equiv.) in MeCN (2.0 mL, *c* = 0.25 M) trifluoroacetic anhydride (0.21 mL, 0.32 g, 1.5 mmol, 3.0 equiv.) was added at 0 °C, under an ambient atmosphere. The reaction mixture was stirred (400 rpm) at 0 °C for 1 h, and then stirred at 25 °C for 16 h. The solvent was removed under reduced pressure by rotary evaporation and the residue was dissolved in DCM (10 mL). The resulting solution was washed with saturated aqueous $NaHCO_3$ solution (10 mL) and aqueous $NaBF_4$ solution (2 × 10 mL, 10% w/w). The DCM layer was dried over Na_2SO_4, filtered, and the solvent was removed under reduced pressure. Purification by flash silica gel column chromatography (DCM / MeOH = 30:1 (v/v)) afforded 173 mg of the title compound **12-Ia** as a colorless solid (83% yield).

R_f = 0.42 (DCM / MeOH = 15:1 (v/v)).

NMR Spectroscopy:

^{1}H NMR (500 MHz, CD_3CN, 23 °C, δ): 11.1 (bs, 1H), 8.46 (d, *J* = 3.1 Hz, 1H), 7.96 (d, *J* = 7.9 Hz, 2H), 7.84 (d, *J* = 8.4 Hz, 1H), 7.71 (t, *J* = 7.6 Hz, 2H), 7.48 (t, *J* = 7.8 Hz, 2H), 7.42 (t, *J* = 7.8 Hz, 1H), 7.38 (d, *J* = 8.2 Hz, 2H), 7.11 (t, *J* = 7.6 Hz, 1H), 6.86 (d, *J* = 8.2 Hz, 1H).

^{13}C NMR (126 MHz, CD_3CN, 23 °C, δ): 141.1, 139.4, 134.0, 133.3, 131.6, 130.6, 129.4, 126.1, 125.9, 124.1, 123.6, 120.1, 115.6, 83.4.

^{19}F NMR (471 MHz, $CDCl_3$, 23 °C, δ): –151.0 (bs), –151.1 (bs).

HRMS-ESI(m/z) calc'd for $C_{20}H_{14}NS_2$ $[M–BF_4]^+$, 332.0562; found, 332.0561; deviation: +0.4 ppm.

Indole derivative 12-II

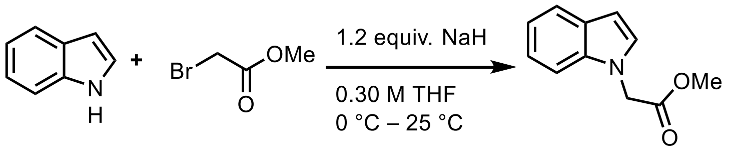

12-I 1.0 equiv. 1.0 equiv. **12-II,** 27%

Prepared according to the reported procedure and spectra are in good accordance with the literature.[5] In a 50 mL two-neck round-bottomed flask, indole **12-I** (0.41 ml, 0.50 g, 4.27 mmol, 1.0 equiv.) was added and dissolved in dry THF (c =0.30 M, 15 mL) and the mixture was cooled to 0 °C with an ice bath. NaH (0.20 g, 5.0 mmol, 60% w/w) was added at 0 °C portion wise and the mixture was allowed to warm to the 25 °C. To the resulting solution was added alkyl bromide(0.47 ml, 0.76 g, 5.0 mmol) dropwise. The reaction mixture was stirred at 25 °C overnight. The mixture was diluted with Et_2O and washed with brine (2 × 25mL). the organic phase was dried over anhydrous Na_2SO_4 and concentrated under reduced pressure. The residue was further purified by flash column chromatography on silica gel (hexanes / Et_2O = 10:1 (v/v)) to afford the product **12-II** as a colorless oil (214 mg, 27% yield)

NMR Spectroscopy:

^{1}H NMR (600 MHz, $CDCl_3$, 23 °C, δ): 7.66 (dt, J = 7.9, 1.0 Hz, 1H), 7.29 – 7.23 (m, 2H), 7.15 (ddd, J = 7.9, 6.4, 1.6 Hz, 1H), 7.10 (d, J = 3.2 Hz, 1H), 6.59 (dd, J = 3.2, 0.8 Hz, 1H), 4.87 (s, 2H), 3.75 (s, 3H).

^{13}C NMR (151 MHz, $CDCl_3$, 23 °C, δ): 169.2, 136.6, 128.7, 128.5, 122.2, 121.3, 120.0, 109.0, 102.7, 52.7, 47.8.

HRMS-GC-EI(m/z) calc'd for $C_{11}H_{11}O_2N$ $[M]^+$, 189.0784; found, 189.0785; deviation: –0.3 ppm.

Indole derivative-derived thianthrenium salt 12-IIa

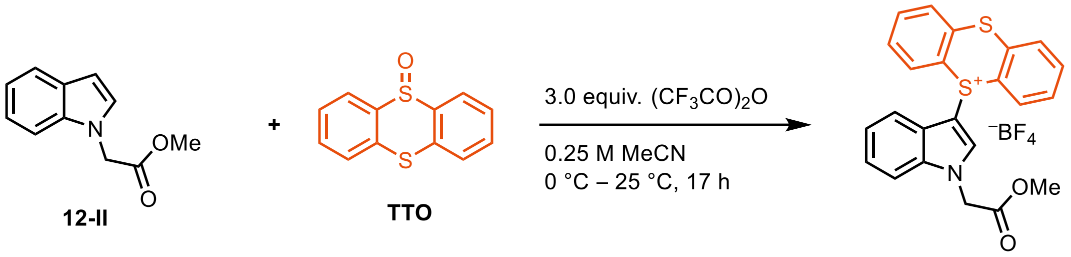

12-II **TTO**

1.00 equiv. 1.00 equiv. **12-IIa,** 86%

To a solution of indole derivative **12-II** (28.0 mg, 0.148 mmol, 1.00 equiv.) and TTO (thianthrene-S-oxide, 34.4 mg, 0.148 mmol, 1.00 equiv.) in MeCN (0.6 mL, c = 0.25 M) trifluoroacetic anhydride (0.062 mL, 93 mg, 0.45 mmol, 3.0 equiv.) was added at 0 °C, under an ambient atmosphere. The reaction mixture was stirred at 0 °C for 1 h, and then stirred (400 rpm) at 25 °C for 16 h. The solvent was removed under reduced pressure by rotary evaporation and the residue was dissolved in DCM (10 mL). The resulting solution was washed with saturated aqueous $NaHCO_3$ solution (10 mL) and aqueous $NaBF_4$ solution (2 × 10 mL, 10% w/w). The DCM layer was dried over Na_2SO_4, filtered, and

the solvent was removed under reduced pressure. Purification by flash column chromatography on silica gel (DCM / MeOH = 30:1 to 15:1 (v/v)) afforded 62.8 mg of the title compound **12-IIa** as a colorless solid (86% yield).

R*f* = 0.33 (DCM / MeOH = 15:1 (v/v))

NMR Spectroscopy:

^{1}H NMR (500 MHz, $CDCl_3$, 23 °C, δ): 8.87 (s, 1H), 7.85 (dd, *J* = 7.9, 1.2 Hz, 2H), 7.63 (td, *J* = 7.6, 1.3 Hz, 2H), 7.55 (d, *J* = 8.4 Hz, 1H), 7.44 – 7.40 (m, 3H), 7.36 (dd, *J* = 8.1, 1.3 Hz, 2H), 7.11 (t, *J* = 7.2 Hz, 1H), 6.77 (d, *J* = 8.1 Hz, 1H), 5.32 (s, 2H), 3.82 (s, 3H).

^{13}C NMR (126 MHz, $CDCl_3$, 23 °C, δ): 168.0, 144.7, 139.1, 133.0, 132.4, 130.6, 129.9, 128.4, 125.6, 125.0, 123.9, 122.8, 119.6, 112.6, 82.1, 53.2, 48.6.

^{19}F NMR (471 MHz, $CDCl_3$, 23 °C, δ): –151.6 (bs), –151.7 (bs).

HRMS-ESI(m/z) calc'd for $C_{23}H_{18}NO_2NS_2$ $[M–BF_4]^+$, 404.0774; found, 404.0771; deviation: +0.7 ppm.

Benzyl 2-methoxybenzylcarbamate 13

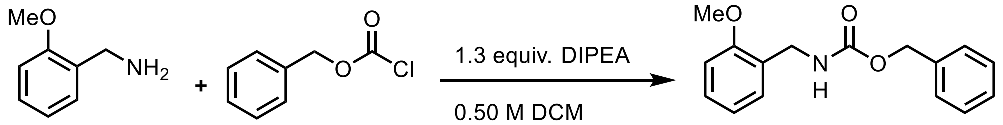

13-S 1.00 equiv. **CbzCl** 1.1 equiv. **13,** 89%

Prepared according to the reported procedure and the spectra are in good accordance with literature.[6] To a solution of 2-methoxybenzylamine **13-S** (0.65 ml, 0.69 g, 5.0 mmol, 1.0 equiv.) in DCM (*c* = 0.50 M, 10 ml) DIPEA (1.1 ml, 0.84 g, 6.5 mmol, 1.3 equiv.). The reaction mixture was cooled to 0 °C with an ice bath and **CbzCl** (0.77 ml, 0.94 g, 5.5 mmol, 1.1 equiv.) was added dropwise over 1 minute. The ice bath was then removed and the reaction mixture was stirred (500 rpm) at 25 °C for 2 h. Then H_2O (10 ml) was added and the mixture was transferred to a separatory funnel. Layers were separated and the organic phase was dried over Na_2SO_4. The residue was purified by flash column chromatography on silica gel (hexanes:EtOAc = 20:1 (v/v) to EtOAc only) to afford **13** (1.2 g, 89% yield).

NMR Spectroscopy:

^{1}H NMR (600 MHz, $CDCl_3$, 23 °C, δ): 7.38 – 7.26 (m, 7H), 6.97 – 6.91 (m, 1H), 6.87 (dd, *J* = 8.2, 1.1 Hz, 1H), 5.34 (t, *J* = 6.5 Hz, 1H), 5.11 (s, 2H), 4.39 (d, *J* = 6.2 Hz, 2H), 3.83 (s, 3H).

^{13}C NMR (151 MHz, $CDCl_3$, 23 °C, δ): 157.6, 156.5, 136.8, 129.6, 129.0, 128.6, 128.3, 128.2, 126.6, 120.7, 110.3, 66.8, 55.3, 41.2.

HRMS-ESI(m/z) calc'd for $C_{16}H_{17}NO_3NNa$ $[M+Na]^+$, 294.1101; found, 294.1101; deviation: –1.0 ppm.

Benzyl 2-methoxybenzylcarbamate-derived thianthrenium salt 13a

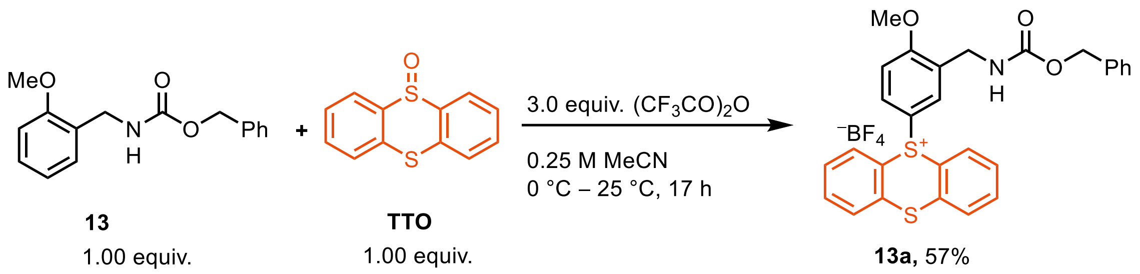

To a solution of benzyl 2-methoxybenzylcarbamate **13** (67.8 mg, 0.250 mmol, 1.00 equiv.) and TTO (thianthrene-*S*-oxide, 58.1 mg, 0.250 mmol, 1.00 equiv.) in MeCN (1.0 mL, *c* = 0.25 M) trifluoroacetic anhydride (104 µL, 157 mg, 0.750 mmol, 3.00 equiv.) was added at 0 °C, under an ambient atmosphere. The reaction mixture was stirred at 0 °C for 1 h, and then stirred at 25 °C for 16 h. The solvent was removed under reduced pressure by rotary evaporation and the residue was dissolved in DCM (10 mL). The resulting solution was washed with saturated aqueous $NaHCO_3$ solution (10 mL) and aqueous $NaBF_4$ solution (2 × 10 mL, 10% w/w). The DCM layer was dried over Na_2SO_4, filtered, and the solvent was removed under reduced pressure. The crude mixture was purified by flash column chromatography on silica gel (DCM only to DCM / MeOH = 12:1 (v/v)) to afford the product **13a** (82.0 mg, 57%) as a colorless solid.

NMR Spectroscopy:

^{1}H NMR (600 MHz, CD_3CN, 23 °C, δ): 8.24 (dd, *J* = 8.0, 1.3 Hz, 2H), 7.89 (dd, *J* = 8.0, 1.2 Hz, 2H), 7.83 (td, *J* = 7.7, 1.5 Hz, 2H), 7.74 (td, *J* = 7.7, 1.4 Hz, 2H), 7.38 (m, 4H), 7.19 (dd, *J* = 8.9, 2.8 Hz, 1H), 7.08 – 6.97 (m, 2H), 5.98 (t, *J* = 6.4 Hz, 1H), 5.01 (s, 2H), 4.12 (d, *J* = 6.3 Hz, 2H), 3.83 (s, 3H).

^{13}C NMR (151 MHz, CD_3CN, 23 °C, δ): 161.9, 157.4, 138.2, 137.0, 135.8, 135.2, 131.8, 131.4, 130.9, 130.8, 129.6, 129.0, 128.8, 128.3, 120.4, 114.0, 113.6, 67.1, 57.0, 40.0.

^{19}F NMR (471 MHz, $CDCl_3$, 23 °C, δ): –151.4 (bs), –151.5 (bs).

HRMS-ESI(m/z) calc'd for $C_{28}H_{24}O_3NS_2$ $[M–BF_4]^+$, 486.1192; found, 486.1194; deviation: –0.4 ppm.

Benzylphenylether-derived thianthrenium salt 14a

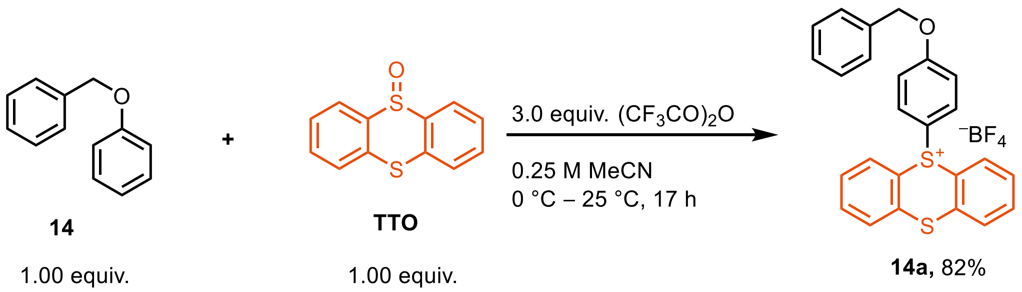

To a solution of benzylphenylether **14** (184 mg, 0.500 mmol, 1.00 equiv.) and TTO (thianthrene-*S*-oxide, 116 mg, 0.500 mmol, 1.00 equiv.) in MeCN (2.0 mL, *c* = 0.25 M) trifluoroacetic anhydride (0.21 mL, 0.32 mg, 1.5 mmol, 3.0 equiv.) was added at 0 °C, under an ambient atmosphere. The reaction mixture was

stirred at 0 °C for 1 h, and then stirred at 25 °C for 16 h. The solvent was removed under reduced pressure by rotary evaporation and the residue was dissolved in DCM (10 mL). The resulting solution was washed with saturated aqueous $NaHCO_3$ solution (10 mL) and aqueous $NaBF_4$ solution (2 × 10 mL, 10% w/w). The DCM layer was dried over Na_2SO_4, filtered, and the solvent was removed under reduced pressure. The crude mixture was dissolved in DCM (5 mL) and Et_2O (50 mL) was added to obtain the product as white precipitate. The precipitate was filtered to give 199 mg of the title compound **14a** as a colorless solid (82% yield).

R*f* = 0.33 (DCM / MeOH = 15:1 (v/v)).

NMR Spectroscopy:

^{1}**H NMR** (500 MHz, CD_3CN, 23 °C, δ): 8.27 (dd, *J* = 7.9, 1.4 Hz, 2H), 7.95 (dd, *J* = 7.9, 1.4 Hz, 2H), 7.87 (td, *J* = 7.7, 1.4 Hz, 2H), 7.78 (td, *J* = 7.7, 1.4 Hz, 2H), 7.45 – 7.29 (m, 5H), 7.21 – 7.13 (m, 2H), 7.13 – 7.04 (m, 2H), 5.10 (s, 2H)

^{13}**C NMR** (126 MHz, CD_3CN, 23 °C, δ): 163.6, 137.1, 137.1, 135.9, 135.4, 131.6, 131.5, 130.9, 129.6, 129.3, 128.8, 120.3, 118.1, 114.5, 71.3.

^{19}**F NMR** (471 MHz, $CDCl_3$, 23 °C, δ): –151.6 (bs), –151.7 (bs).

HRMS-ESI(m/z) calc'd for $C_{25}H_{19}OS_2$ $[M–BF_4]^+$, 399.0872; found, 399.0874; deviation: –0.5 ppm.

p-Methoxyhydrocinnamic acid-derived thianthrenium salt 15a

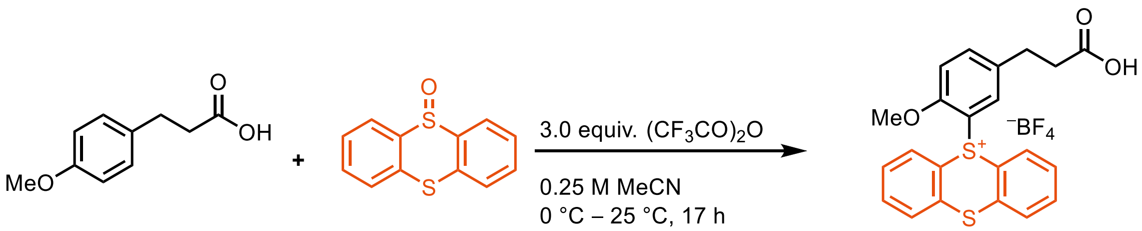

15 **TTO** **15a,** 76%

To a solution of *p*-methoxyhydrocinnamic acid **15** (90.1 mg, 0.500 mmol, 1.00 equiv.) and TTO (thianthrene-*S*-oxide, 116 mg, 0.500 mmol, 1.00 equiv.) in MeCN (2.0 mL, *c* = 0.25 M) trifluoroacetic anhydride (0.21 mL, 0.32 mg, 1.5 mmol, 3.0 equiv.) was added at 0 °C, under an ambient atmosphere. The reaction mixture was stirred at 0 °C for 1 h, and then stirred at 25 °C for 16 h. The solvent was removed under reduced pressure by rotary evaporation and the residue was dissolved in DCM (10 mL). The resulting solution was washed with saturated aqueous $NaHCO_3$ solution (10 mL). The aqueous phase was acidified by 1 N HCl solution and washed with DCM. Combined organic phases was washed by aqueous $NaBF_4$ solution (2 × 10 mL, 10% w/w). The DCM layer was dried over Na_2SO_4, filtered, and the solvent was removed under reduced pressure. Purification by flash column chromatography on silica gel (DCM / MeOH / TFA = 30:1:0.1 (v/v)) afforded 182 mg of the title compound **15a** as a colorless solid (76% yield).

R*f* = 0.12 (DCM / MeOH = 15:1 (v/v)).

NMR Spectroscopy:

^{1}**H NMR** (500 MHz, CD_3CN, 23 °C, δ): 8.24 (ddd, *J* = 8.0, 1.4, 0.5 Hz, 2H), 7.98 – 7.92 (m, 2H), 7.83 (ddd, *J* = 8.0, 7.4, 1.4 Hz, 2H), 7.75 (ddd, *J* = 8.0, 7.4, 1.3 Hz, 2H), 7.48 (dd, *J* = 8.5, 2.1 Hz, 1H), 7.13 (d, *J* = 8.5 Hz, 1H), 6.52 (d, *J* = 2.1 Hz, 1H), 3.90 (s, 3H), 2.70 (t, *J* = 7.2 Hz, 2H), 2.40 (t, *J* = 7.2 Hz, 2H).

^{13}**C NMR** (126 MHz, CD_3CN, 23 °C, δ): 174.0, 157.3, 138.4, 137.0, 136.0, 135.9, 135.7, 131.6, 130.7, 129.6, 118.1, 115.0, 109.5, 57.7, 35.4, 30.0.

^{19}**F NMR** (471 MHz, $CDCl_3$, 23 °C, δ): –151.7 (bs), –151.7 (bs).

HRMS-ESI(m/z) calc'd for $C_{22}H_{19}O_3S_2$ $[M–BF_4]^+$, 395.0770; found, 395.0772; deviation: –0.6 ppm.

2-Phenoxyethyl bromide-derived thianthrenium salt 16a

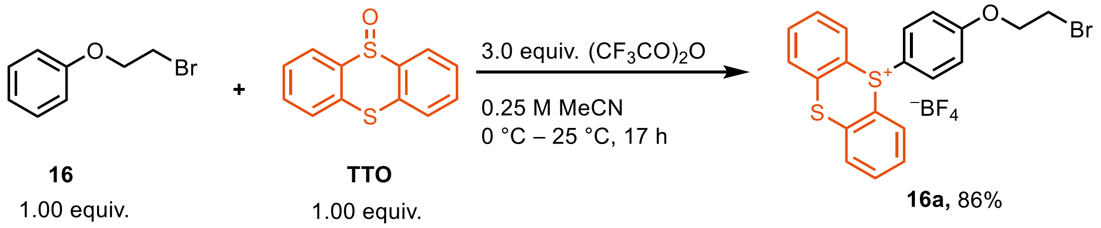

To a solution of 2-phenoxyethyl bromide **16** (100 mg, 0.500 mmol, 1.00 equiv.) and TTO (thianthrene-*S*-oxide, 116 mg, 0.500 mmol, 1.00 equiv.) in MeCN (2.0 mL, *c* = 0.25 M) trifluoroacetic anhydride (0.21 mL, 0.32 mg, 1.5 mmol, 3.0 equiv.) was added dropwise at 0 °C, under an ambient atmosphere. The reaction mixture was allowed to warm to 25 °C over 1 h and then stirred (400 rpm) at this temperature for 16 h. The solvent was removed under reduced pressure by rotary evaporation and the residue was dissolved in DCM (15 mL). The resulting solution was washed with saturated aqueous $NaHCO_3$ solution (15 mL) and aqueous $NaBF_4$ solution (2 × 15 mL, 10% w/w). The DCM layer was dried over Na_2SO_4, filtered, and the solvent was removed under reduced pressure. The crude mixture was dissolved in DCM (5 mL) and Et_2O (50 mL) was added to obtain the product as a white precipitate. The precipitate was filtered to give 216 mg of the title compound **16a** as colorless solid (86% yield).

R_f = 0.27 (DCM / MeOH = 30:1).

NMR Spectroscopy:

^{1}**H NMR** (500 MHz, CD_3CN, 23 °C, δ): 8.29 (dd, *J* = 7.9, 1.5 Hz, 2H), 7.92 (dd, *J* = 7.9, 1.5 Hz, 2H), 7.85 (td, *J* = 7.7, 1.4 Hz, 2H), 7.78 (td, *J* = 7.6, 1.4 Hz, 2H), 7.19 – 7.12 (m, 2H), 7.04 – 6.96 (m, 2H), 4.33 – 4.28 (m, 2H), 3.69 – 3.61 (m, 2H).

^{13}**C NMR** (126 MHz, CD_3CN, 23 °C, δ): 163.1, 137.0, 135.9, 135.4, 131.5, 130.9, 120.2, 117.8, 114.9, 69.6, 30.7.

^{19}**F NMR** (471 MHz, $CDCl_3$, 23 °C, δ): –151.1 (bs), –151.1 (bs).

HRMS-EI(m/z) calc'd for $C_{20}H_{16}OS_2Br$ $[M–BF_4]^+$, 414.9821; found, 414.9826; deviation: –1.3 ppm.

Aminopyrifen-derived thianthrenium salt 17a

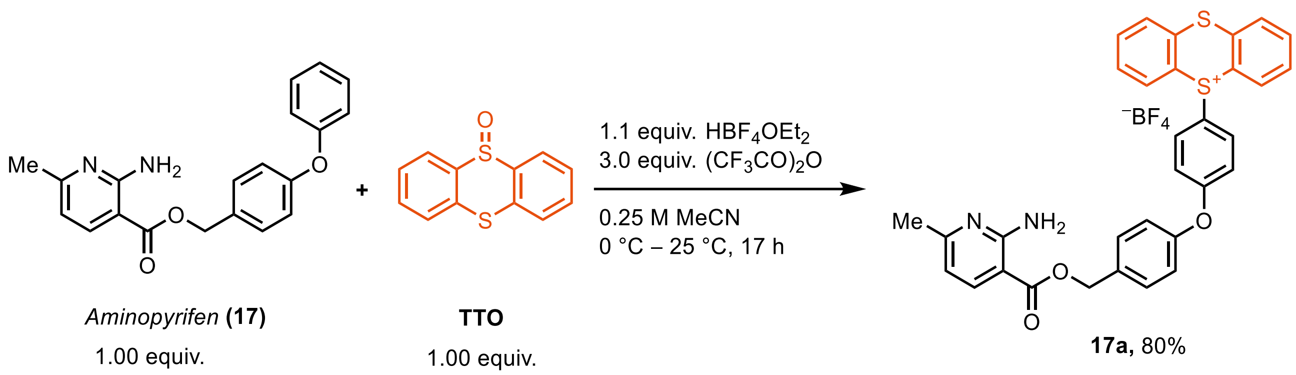

To a solution of aminopyrifen **17**(16.7 mg, 0.0500 mmol, 1.00 equiv.) and TTO (thianthrene-*S*-oxide, 11.6 mg, 0.0500 mmol, 1.00 equiv.) in MeCN (0.20 mL, *c* = 0.25 M), $HBF_4{\cdot}OEt_2$ (8 µL, 9 mg, 6 µmol, 1 equiv.) and trifluoroacetic anhydride (21 µL, 32 mg, 0.15 mmol, 3.0 equiv.) was added at 0 °C, under an ambient atmosphere. The reaction mixture was stirred (400 rpm) at 0 °C for 1 h, and then stirred at 25 °C for 16 h. The solvent was removed under reduced pressure by rotary evaporation and the residue was dissolved in DCM (10 mL). The resulting solution was washed with saturated aqueous $NaHCO_3$ solution (10 mL) and aqueous $NaBF_4$ solution (2 × 10 mL, 10% w/w). The DCM layer was dried over Na_2SO_4, filtered, and the solvent was removed under reduced pressure. The residue was flash column chromatography on silica gel (DCM / MeOH = 30:1 to 15:1 (v/v)) to afford 25.6 mg of the title compound **17a** (80% yield)

R*f* = 0.31 (DCM / MeOH = 15:1 (v/v)).

NMR Spectroscopy:

^{1}H NMR (400 MHz, CD_3CN, 23 °C, δ): 8.53 (d, *J* = 9.3 Hz, 2H), 8.12 (d, *J* = 7.9 Hz, 1H), 7.89 – 7.65 (m, 6H), 7.42 (d, *J* = 8.7 Hz, 2H), 7.25 – 7.19 (m, 2H), 6.98 (dd, *J* = 13.4, 8.9 Hz, 4H), 6.51 (d, *J* = 8.0 Hz, 1H), 5.27 (s, 2H), 3.64 (bs, 2H), 2.43 (s, 3H).

^{13}C NMR (101 MHz, $CDCl_3$, 23 °C, δ): 166.4, 162.2, 162.1, 158.5, 154.5, 141.8, 136.4, 135.3, 134.9, 133.2, 130.6, 130.6, 130.5, 130.3, 120.8, 119.4, 119.3, 116.2, 112.6, 104.3, 65.9, 23.7.

^{19}F NMR (377 MHz, $CDCl_3$, 23 °C, δ): –151.9 (bs), –150.9 (bs).

HRMS-FIA(m/z) calc'd for $C_{32}H_{25}O_4N_3S_2$ $[M–BF_4]^+$, 549.1301; found, 549.1303; deviation: –0.8 ppm.

(±)-Deltamethrin-derived thianthrenium salt 18a

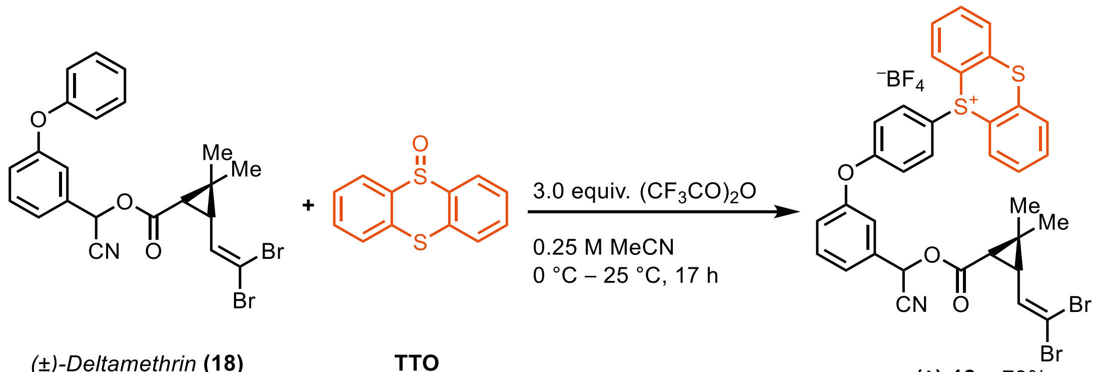

(±)-Deltamethrin **(18)** 1.00 equiv. **TTO** 1.00 equiv. **(±)-18a,** 79%

To a solution of (±)-deltamethrin (**18**) (126 mg, 0.250 mmol, 1.00 equiv.) and TTO (thianthrene-*S*-oxide, 58.1 mg, 0.250 mmol, 1.00 equiv.) in MeCN (1.0 mL, *c* = 0.25 M) trifluoroacetic anhydride (104 µL, 158 mg, 0.750 mmol, 3.00 equiv.) was added at 0 °C, under an ambient atmosphere. The reaction mixture was stirred at 0 °C for 1 h, and then stirred (400 rpm) at 25 °C for 16 h. The solvent was removed under reduced pressure by rotary evaporation and the residue was dissolved in DCM (10 mL). The resulting solution was washed with saturated aqueous $NaHCO_3$ solution (10 mL) and aqueous $NaBF_4$ solution (2 × 10 mL, 10% w/w). The DCM layer was dried over Na_2SO_4, filtered, and the solvent was removed under reduced pressure. The crude mixture was dissolved in DCM (5 mL) and MTBE (50 mL) was added to obtain the product as white precipitate. The precipitate was filtered to give 160 mg of the title compound **(±)-18a** as a colorless solid (79% yield).

R*f* = 0.42 (DCM / MeOH = 15:1 (v/v)).

NMR Spectroscopy:

^{1}**H NMR** (500 MHz, $CDCl_3$, 23 °C, δ): 8.50 (dt, *J* = 8.0, 1.8 Hz, 2H), 7.87 – 7.78 (m, 4H), 7.74 (tt, *J* = 7.5, 1.3 Hz, 2H), 7.46 (t, *J* = 8.0 Hz, 1H), 7.35 (d, *J* = 7.9 Hz, 1H), 7.28 – 7.21 (m, 2H), 7.17 (t, *J* = 2.1 Hz, 1H), 7.07 (dd, *J* = 7.7, 1.9 Hz, 1H), 7.00 – 6.94 (m, 2H), 6.65 (d, *J* = 8.4 Hz, 1H), 6.34 (s, 1H), 2.06 (t, *J* = 8.3 Hz, 1H), 1.91 (d, *J* = 8.3 Hz, 1H), 1.21 (d, *J* = 26.4 Hz, 6H).

^{13}**C NMR** (126 MHz, $CDCl_3$, 23 °C, δ): 168.6, 161.5, 155.1, 136.4, 136.4, 135.1, 135.1, 134.4, 132.4, 131.4, 130.7, 130.5, 130.4, 124.6, 122.2, 119.8, 119.5, 119.0, 119.0, 116.8, 115.8, 91.0, 62.2, 36.6, 31.0, 29.0, 28.2, 15.0.

^{19}**F NMR** (471 MHz, $CDCl_3$, 23 °C, δ): –150.8 (bs), –150.9 (bs).

HRMS-ESI(m/z) calc'd for $C_{34}H_{26}NO_3S_2Br_2$ $[M–BF_4]^+$, 717.9716; found, 717.9719; deviation: –0.5 ppm.

(±)-Pyriproxyphen-derived thianthrenium salt 19a

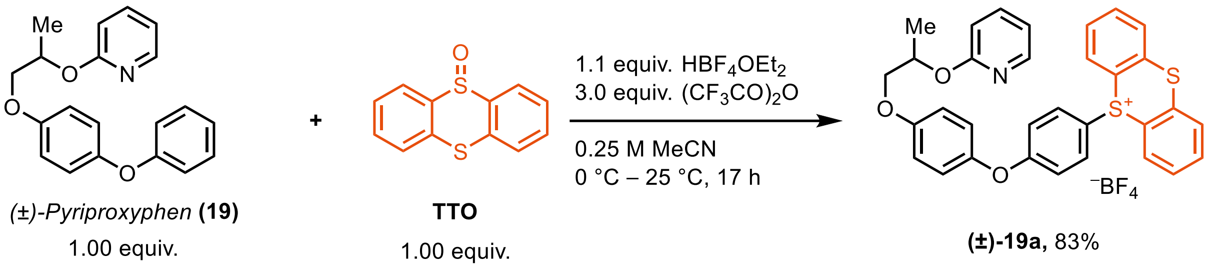

To a solution of (±)-pyriproxyphen (**19**) (1.61 g, 5.00 mmol, 1.00 equiv.) and TTO (thianthrene-*S*-oxide, 1.16 g, 5.00 mmol, 1.00 equiv.) in MeCN (20 mL, *c* = 0.25 M), $HBF_4 \cdot OEt_2$ (0.75 mL, 0.89 g, 5.5 mmol, 1.1 equiv.) and trifluoroacetic anhydride (2.1 mL, 3.2 g, 15 mmol, 3.0 equiv.) were added at 0 °C, under an ambient atmosphere. The reaction mixture was stirred (400 rpm) at 0 °C for 1 h, and then stirred at 25 °C for 16 h. The solvent was removed under reduced pressure by rotary evaporation and the residue was dissolved in DCM (50 mL). The resulting solution was washed with saturated aqueous $NaHCO_3$ solution (25 mL) and aqueous $NaBF_4$ solution (2 × 25 mL, 10% w/w). The DCM layer was dried over Na_2SO_4, filtered, and the solvent was removed under reduced pressure. Purification by flash silica gel column chromatography (DCM to DCM / MeOH = 10:1 (v/v)) afforded 2.58 g of the title compound **(±)-19a** as a colorless solid (83% yield).

R_f = 0.28 (DCM / MeOH = 15:1 (v/v)).

NMR Spectroscopy:

^{1}H NMR (500 MHz, CD_3CN, 23 °C, δ): 8.30 (dd, *J* = 7.9, 1.5 Hz, 2H), 8.09 (ddd, *J* = 5.0, 2.0, 0.8 Hz, 1H), 7.91 (dd, *J* = 7.9, 1.4 Hz, 2H), 7.84 (td, *J* = 7.7, 1.4 Hz, 2H), 7.80 – 7.72 (m, 2H), 7.60 (d, *J* = 0.8 Hz, 1H), 7.16 – 7.07 (m, 2H), 6.95 – 6.84 (m, 7H), 6.67 (dt, *J* = 8.4, 0.9 Hz, 1H), 5.52 (m, 1H), 4.13 (dd, *J* = 10.3, 6.0 Hz, 1H), 4.08 (dd, *J* = 10.3, 4.0 Hz, 1H), 1.37 (d, *J* = 6.4 Hz, 3H).

^{13}C NMR (126 MHz, CD_3CN, 23 °C, δ): 164.1, 163.8, 157.3, 148.7, 147.8, 140.1, 137.1, 135.9, 135.51, 131.54, 131.52, 131.0, 122.8, 120.0, 119.3, 117.9, 117.0, 116.1, 112.2, 71.9, 70.2, 17.0.

^{19}F NMR (471 MHz, $CDCl_3$, 23 °C, δ): –151.7 (bs), –151.8 (bs).

HRMS-ESI(m/z) calc'd for $C_{32}H_{26}NO_3S_2$ $[M+H]^+$, 536.1349; found, 536.1346; deviation: +0.4 ppm.

Atomoxetine-derived thianthrenium salt 20a

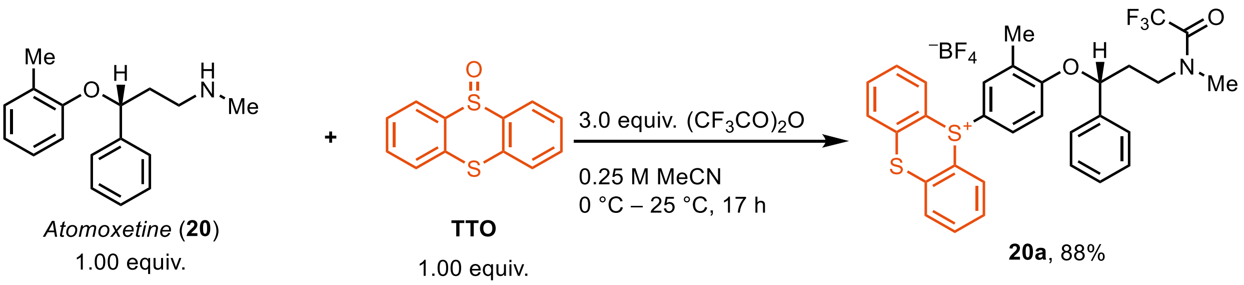

Atomoxetine hydrochloride **20·HCl** (146 mg, 0.500 mmol, 1.00 equiv.) was dissolved in EtOAc (5 mL)

and washed with saturated aquesous Na_2CO_3 solution. The organic layer was dried over Na_2SO_4, filtered, and concentrated under reduced pressure. The residue was dissolved in MeCN (2.0 mL, *c* = 0.25 M) and added to a 20-mL glass vial containing TTO (thianthrene-*S*-oxide, 116 mg, 0.500 mmol, 1.00 equiv.). The mixture was cooled to 0 °C with an ice bath and trifluoroacetic anhydride (0.21 mL, 0.32 g, 1.5 mmol, 3.0 equiv.) was added at 0 °C, under an ambient atmosphere. The reaction mixture was stirred (400 rpm) at 0 °C for 1 h, and then stirred at 25 °C for 16 h. The solvent was removed under reduced pressure by rotary evaporation and the residue was dissolved in DCM (10 mL). The resulting solution was washed with saturated aqueous $NaHCO_3$ solution (10 mL) and aqueous $NaBF_4$ solution (2 × 10 mL, 10% w/w). The DCM layer was dried over Na_2SO_4, filtered, and the solvent was removed under reduced pressure. Purification by flash column chromatography on silica gel (DCM / MeOH = 30:1 (v/v)) afforded 207 mg of the title compound **20a** as a colorless solid (88% yield).

R_f = 0.53 (DCM / MeOH = 15:1 (v/v)).

NMR Spectroscopy:

^{1}H NMR (600 MHz, $CDCl_3$, 23 °C, δ): 8.37 – 8.27 (m, 2H), 7.84 – 7.62 (m, 6H), 7.32 – 7.27 (m, 2H), 7.26 – 7.22 (m, 3H), 7.13 (ddd, *J* = 8.2, 2.8, 0.8 Hz, 1H), 6.92 – 6.83 (m, 1H), 6.72 – 6.60 (m, 1H), 5.27, 5.21 (dd, *J* = 8.6, 4.1 Hz, 1H), 3.83 – 3.43 (m, 2H), 3.08 (t, *J* = 1.6 Hz, 2H), 2.98 (s, 1H), 2.36 – 2.00 (m, 5H).

^{13}C NMR (151 MHz, CD_3CN, 23 °C, δ): 160.1, 160.0, 157.01 (q, *J* = 35.9 Hz), 156.8 (q, *J* = 35.9 Hz), 139.5, 139.2, 136.19, 136.17, 136.0, 134.76, 134.73, 134.70, 134.68, 134.6, 134.5, 131.4, 131.2, 130.6, 130.32, 130.30, 130.27, 129.24, 129.17, 128.6, 128.5, 128.4, 128.3, 125.7, 125.5, 11967, 199.65, 119.39, 119.35, 116.61 (q, *J* = 287.5 Hz), 116.46 (q, *J* = 287.7 Hz), 114.51, 114.43, 113.22, 113.13, 78.6, 78.1, 46.49 (d, *J* = 3.2 Hz), 35.7, 35.5, 35.3 (q, *J* = 3.8 Hz), 34.8, 16.8, 16.7.

^{19}F NMR (565 MHz, $CDCl_3$, 23 °C, δ): {–68.9, (s); –69.9, (s), 3F}, {–151.2 (bs), –151.3 (bs), 4F}.

HRMS-ESI(m/z) calc'd for $C_{31}H_{27}F_3O_2N_1S_2$ $[M–BF_4]^+$, 566.1430; found, 566.1425; deviation: +0.9 ppm.

Etofenprox-derived thianthrenium salt 21a

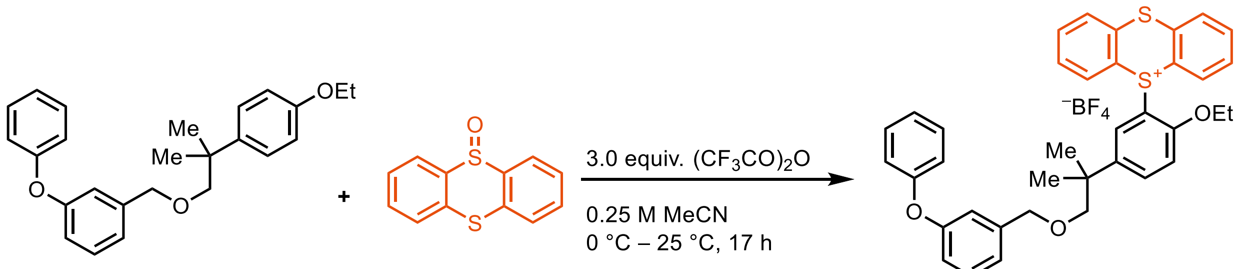

Etofenprox **(21)** 1.00 equiv. **TTO** 1.00 equiv. **21a,** 76%

To a solution of etofenprox (**21**) (188 mg, 0.500 mmol, 1.00 equiv.) and TTO (thianthrene-*S*-oxide, 116 mg, 0.500 mmol, 1.00 equiv.) in MeCN (2.0 mL, *c* = 0.25 M) trifluoroacetic anhydride (0.21 mL, 0.32 mg, 1.5 mmol, 3.0 equiv.) was added at –40 °C, under an ambient atmosphere. The reaction mixture

was allowed to warm to 25 °C over 2 h and then stirred (400 rpm) at this temperature for 15 h. The solvent was removed under reduced pressure by rotary evaporation and the residue was dissolved in DCM (15 mL). The resulting solution was washed with saturated aqueous $NaHCO_3$ solution (15 mL) and aqueous $NaBF_4$ solution (2 × 15 mL, 10% w/w). The DCM layer was dried over Na_2SO_4, filtered, and the solvent was removed under reduced pressure. Purification by flash column chromatography on silica gel (DCM to DCM / MeOH = 10:1 (v/v)) afforded 276 mg of the title compound **21a** as a colorless solid (76% yield).

R_f = 0.38 (DCM / MeOH = 15:1 (v/v)).

NMR Spectroscopy:

^{1}H NMR (600 MHz, CD_3CN, 23 °C, δ): 8.20 (dd, *J* = 8.0, 1.4 Hz, 2H), 7.86 (dd, *J* = 7.9, 1.4 Hz, 2H), 7.80 (td, *J* = 7.6, 1.4 Hz, 2H), 7.72 (td, *J* = 7.7, 1.4 Hz, 2H), 7.58 (dd, *J* = 8.7, 2.3 Hz, 1H), 7.37 (dd, *J* = 8.7, 7.4 Hz, 2H), 7.30 (t, *J* = 7.9 Hz, 1H), 7.15 (tt, *J* = 7.4, 1.1 Hz, 1H), 7.07 (d, *J* = 8.8 Hz, 1H), 6.98 (dd, *J* = 8.7, 1.1 Hz, 2H), 6.93 – 6.87 (m, 2H), 6.73 – 6.68 (m, 1H), 6.58 (d, *J* = 2.3 Hz, 1H), 4.27 (s, 2H), 4.17 (q, *J* = 7.0 Hz, 2H), 3.24 (s, 2H), 1.41 (t, *J* = 7.0 Hz, 3H), 1.06 (s, 6H).

^{13}C NMR (151 MHz, CD_3CN, 23 °C, δ): 158.2, 158.1, 156.4, 142.2, 142.0, 138.1, 136.1, 135.7, 134.8, 131.3, 130.8, 130.8, 130.7, 128.0, 124.5, 123.1, 119.8, 118.7, 118.2, 118.2, 115.0, 108.5, 79.9, 73.0, 67.1, 39.4, 26.1, 14.8.

^{19}F NMR (565 MHz, $CDCl_3$, 23 °C, δ): –151.7 (bs), –151.7 (bs).

HRMS-ESI(m/z) calc'd for $C_{37}H_{35}O_3S_2$ $[M–BF_4]^+$, 591.2022; found, 591.2029; deviation: +0.5 ppm.

(±)-Flurbiprofen methyl ester 22-S

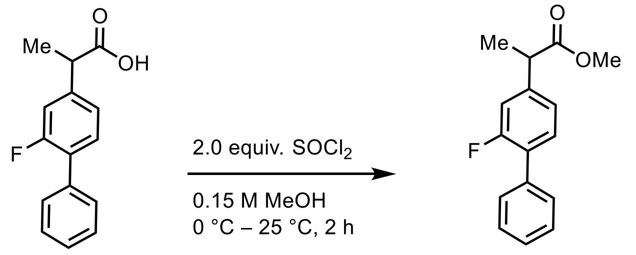

Flurbiprofen **22-S** *(±)-Flurbiprofen methyl ester* **22** 90%

Prepared according to the reported procedure and spectra are in good accordance with literature.[7] A 100 mL round-bottomed flask equipped with a Teflon-coated magnetic stirring bar was charged with flurbiprofen (**22-S**) (1.22 g, 5.00 mmol, 1.00 equiv.). The solid was dissolved in MeOH (33 mL, *c* = 0.15 M) and the solution was cooled to 0 °C with an ice bath. Thionyl chloride (0.73 mL, 10 mmol, 2.0 equiv.) was added at 0 °C. The reaction was allowed to warm to 25 °C and stirred for 2 h at 25 °C. Saturated aqueous $NaHCO_3$ solution (10 ml) was added to the reaction mixture, and MeOH was evaporated under reduced pressure. The resulting mixture was extracted with EtOAc (3 × 20 mL), the combined organic layers were washed with brine, dried over $MgSO_4$, and concentrated under reduced pressure. The residue was washed with diethylether to afford **(±)-22** (1.16 g, 90% yield) of the title

compound as a colorless oil.

NMR Spectroscopy:

^{1}H NMR (500 MHz, $CDCl_3$, 23 °C, δ): 7.54 (dt, *J* = 8.1, 1.5 Hz, 2H), 7.46 – 7.43 (m, 2H), 7.42 – 7.35 (m, 2H), 7.17 – 7.11 (m, 2H), 3.77 (q, *J* = 7.2 Hz, 1H), 3.71 (s, 3H), 1.55 (d, *J* = 7.2 Hz, 3H).

^{13}C NMR (126 MHz, $CDCl_3$, 23 °C, δ) 174.6, 159.8 (d, *J* = 248 Hz), 141.9 (d, *J* = 7.8 Hz), 135.6, 130.9 (d, *J* = 3.8 Hz), 129.1 (d, *J* = 2.9 Hz), 128.6, 128.0 (d, *J* = 13.3 Hz), 127.8, 123.6 (d, *J* = 3.1 Hz), 115.4 (d, *J* = 23.2 Hz), 52.3, 45.0 (d, *J* = 1.5 Hz), 18.6.

(±)-Flurbiprofen methyl ester-derived thianthrenium salt 22a

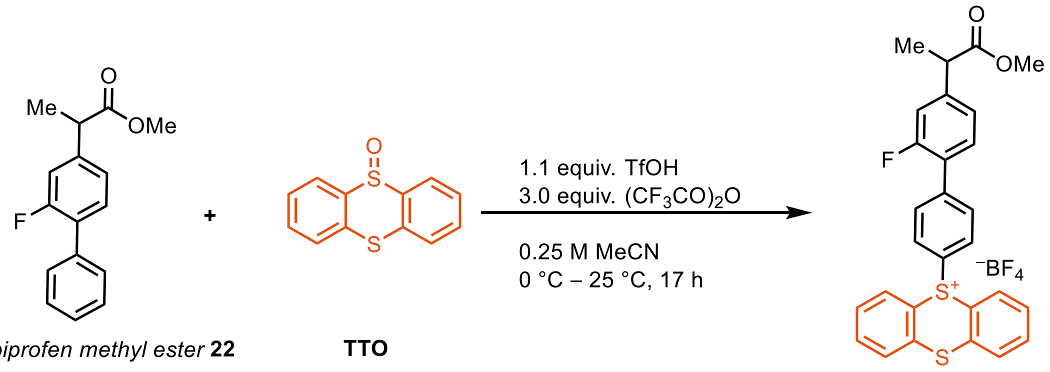

(±)-Flurbiprofen methyl ester **22** — 1.00 equiv.; **TTO** — 1.00 equiv.; **(±)-22a,** 70%

To a solution of (±)-flurbiprofen methyl ester **22** (129 mg, 0.500 mmol, 1.00 equiv.) and TTO (thianthrene-*S*-oxide, 116 mg, 0.500 mmol, 1.00 equiv.) in MeCN (2.0 mL, *c* = 0.25 M), trifluoroacetic anhydride (0.21 mL, 0.32 g, 1.5 mmol, 3.0 equiv.) was added at 0 °C, under an ambient atmosphere followed by addition of TfOH (49 µL, 83 mg, 0.55 mmol, 1.1 equiv.). The reaction mixture was stirred (400 rpm) at 0 °C for 1 h, and then stirred at 25 °C for 16 h. The solvent was removed under reduced pressure by rotary evaporation and the residue was dissolved in DCM (10 mL). The resulting solution was washed with saturated aqueous $NaHCO_3$ solution (10 mL) and aqueous $NaBF_4$ solution (2 × 10 mL, 10% w/w). The DCM layer was dried over Na_2SO_4, filtered, and the solvent was removed under reduced pressure. The crude mixture was dissolved in DCM (5 mL) and Et_2O (30 mL) was added to obtain the product as white precipitate. The precipitate was filtered to give 213 mg of the title compound **(±)-22a** as a colorless solid (70% yield).

R_f = 0.24 (DCM / MeOH = 15:1 (v/v)).

NMR Spectroscopy:

^{1}H NMR (500 MHz, $(CD_3)_2SO$, 23 °C, δ): 8.63 (d, *J* = 7.8 Hz, 2H), 8.09 (d, *J* = 7.9 Hz, 2H), 7.95 (t, *J* = 7.6 Hz, 2H), 7.89 (t, *J* = 7.6 Hz, 2H), 7.73 (d, *J* = 8.4 Hz, 2H), 7.47 (t, *J* = 8.2 Hz, 1H), 7.32 (d, *J* = 8.7 Hz, 2H), 7.25 (dd, *J* = 18.5, 10.1 Hz, 2H), 3.91 (q, *J* = 7.2 Hz, 1H), 3.60 (s, 3H), 1.41 (d, *J* = 7.2 Hz, 3H).

^{13}C NMR (126 MHz, $(CD_3)_2SO$, 23 °C, δ): 173.6, 159.8, 157.8, 143.9 (d, *J* = 7.7 Hz), 139.0, 135.7, 135.5, 134.9, 130.9 (d, *J* = 3.0 Hz), 130.6 (d, *J* = 3.0 Hz), 130.3, 129.7, 128.4, 124.6 (d, *J* = 13.1

Hz), 124.3 (d, J = 3.6 Hz), 119.2, 115.4 (d, J = 23.2 Hz), 52.0, 43.8, 18.2.

^{19}F NMR (471 MHz, $CDCl_3$, 23 °C, δ): –117.8 (s, 1F), {–148.2 (bs), –148.2 (bs), 4F}.

HRMS-GC-EI(m/z) calc'd for $C_{16}H_{15}O_2F$ $[M]^+$, 258.1051; found, 258.1055; deviation: +1.6ppm.

Niflumic acid methyl ester 23-S

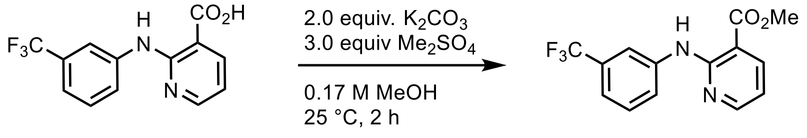

Niflumic acid (**23-S**)

Niflumic acid methyl ester **23**
86%

Prepared according to the reported procedure and spectra are in good accordance with literature.[8,9] To a solution of niflumic acid (**23-S**) (1.41 g, 5.00 mmol, 1.00 equiv.) in methanol (30 mL) was added K_2CO_3 (1.38 g, 10.0 mmol, 2.00 equiv.), and dimethyl sulfate (1.4 mL, 1.9 g, 15 mmol, 3.0 equiv.). The mixture was stirred at 25 °C for 2 h. The resulting yellow suspension was filtered. The filtrate was extracted with ethyl acetate (75 mL), washed with water (25 mL) and brine (50 mL). The combined organic phases was dried over $MgSO_4$, and concentrated under reduced pressure by rotary evaporation to give a yellow oil which was purified by flash column chromatography on silica gel (hexanes / EtOAc = 10:1 (v/v) to EtOAc only) to give the desired ester **23-S** (1.3 g, 86% yield)

NMR Spectroscopy:

^{1}H NMR (500 MHz, $CDCl_3$, 23 °C, δ): 10.40 (s, 1H), 8.44 (dd, J = 4.8, 2.0 Hz, 1H), 8.29 (dd, J = 7.8, 2.0 Hz, 1H), 8.11 (t, J = 2.0 Hz, 1H), 7.90 (dd, J = 8.2, 2.2 Hz, 1H), 7.46 (t, J = 7.9 Hz, 1H), 7.31 (ddt, J = 7.7, 1.6, 0.9 Hz, 1H), 6.82 (dd, J = 7.8, 4.7 Hz, 1H), 3.97 (s, 3H).

^{13}C NMR (151 MHz, $CDCl_3$, 23 °C, δ) 168.1, 155.9, 140.5, 140.4, 131.3 (q, J = 31.9 Hz), 124.3 (q, J = 272.2 Hz), 123.6, 119.1 (q, J = 3.8 Hz), 117.2 (q, J = 3.8 Hz), 114.2, 107.6, 52.5.

^{19}F NMR (471 MHz, $CDCl_3$, 23 °C, δ): –62.5.

HRMS-GC-EI(m/z) calc'd for $C_{14}H_{11}O_2N_2F_3$ $[M]^+$, 296.0767; found, 296.0766; deviation: +0.5 ppm.

Niflumic acid methyl ester-derived thianthrenium salt 23a

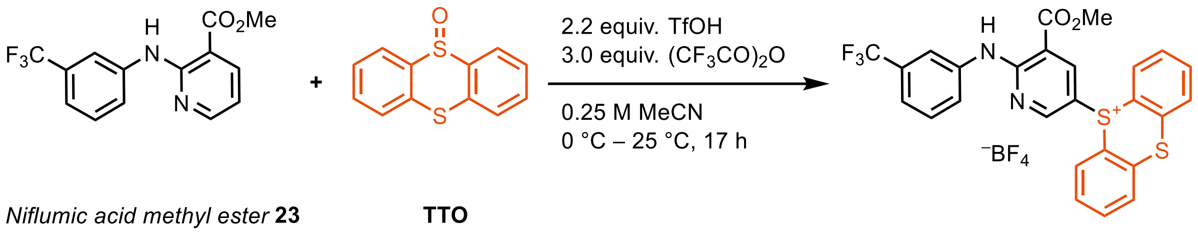

Niflumic acid methyl ester **23**
1.00 equiv.

TTO
1.00 equiv.

23a, 57%

To a solution of niflumic acid methyl ester **23** (148 mg, 0.500 mmol, 1.00 equiv.) and TTO (thianthrene-*S*-oxide, 116 mg, 0.500 mmol, 1.00 equiv.) in MeCN (2.0 mL, c = 0.25 M), TfOH (49 µL, 83 mg, 0.55 mmol, 1.1 equiv.) and trifluoroacetic anhydride (0.21 mL, 0.32 g, 1.5 mmol, 3.0 equiv.)

were added sequentially at 0 °C, under an ambient atmosphere followed by addition of TfOH (49 µL, 83 mg, 0.55 mmol, 1.1 equiv.). The reaction mixture was stirred (400 rpm) at 0 °C for 1 h, and then stirred at 25 °C for 16 h. The solvent was removed under reduced pressure by rotary evaporation and the residue was dissolved in DCM (10 mL). The resulting solution was washed with saturated aqueous $NaHCO_3$ solution (10 mL) and aqueous $NaBF_4$ solution (2 × 10 mL, 10% w/w). The DCM layer was dried over Na_2SO_4, filtered, and the solvent was removed under reduced pressure. The crude mixture was dissolved in DCM (5 mL) and MTBE (50 mL) was added to obtain the product as a white precipitate. The precipitate was filtered to give 170 mg of the title compound **22a** as a colorless solid (57% yield).

R_f = 0.34 (DCM / MeOH = 15:1 (v/v)).

NMR Spectroscopy:

^{1}H NMR (600 MHz, CD_3CN, 23 °C, δ): 10.53 (s, 1H), 8.26 (dd, *J* = 8.0, 1.3 Hz, 2H), 8.18 (d, *J* = 2.8 Hz, 1H), 8.15 (d, *J* = 2.7 Hz, 1H), 8.12 (d, *J* = 2.2 Hz, 1H), 7.99 (ddd, *J* = 8.0, 1.3, 0.4 Hz, 2H), 7.88 (ddd, *J* = 8.0, 7.5, 1.3 Hz, 2H), 7.79 (ddd, *J* = 8.0, 7.5, 1.3 Hz, 2H), 7.75 – 7.72 (m, 1H), 7.53 (t, *J* = 8.0 Hz, 1H), 7.47 – 7.43 (m, 1H), 3.87 (s, 3H).

^{13}C NMR (151 MHz, CD_3CN, 23 °C, δ): 166.9, 158.1, 153.7, 141.7, 139.7, 137.0, 136.0, 135.0, 131.8, 131.5 (q, *J* = 32.1 Hz), 131.1, 130.8, 126.7, 125.2 (q, *J* = 271.5 Hz), 122.0 (q, *J* = 3.9 Hz), 120.1, 119.6 (q, *J* = 4.0 Hz), 110.4, 108.7, 54.0

^{19}F NMR (471 MHz, $CDCl_3$, 23 °C, δ): –63.2 (s, 3F), {–151.7 (bs), –151.7 (bs), 4F}.

HRMS-ESI(m/z) calc'd for $C_{26}H_{18}F_3N_2O_2S_2$ $[M–BF_4]^+$, 511.0756; found, 511.0754; deviation: +0.4 ppm.

2-Fluoro-6-phenoxybenzonitrile-derived thianthrenium salt 24a

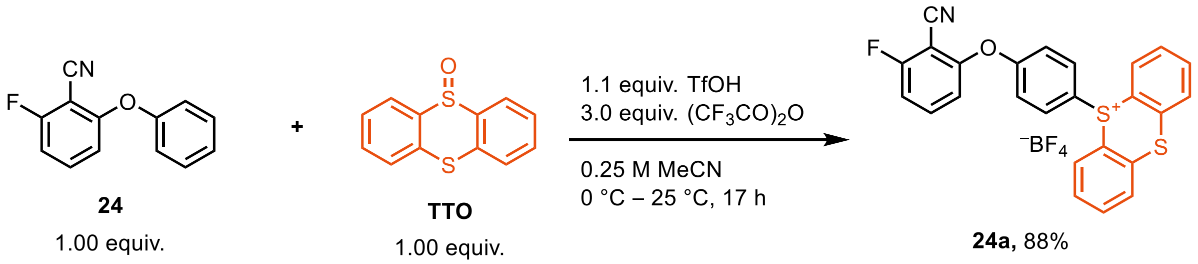

To a solution of 2-fluoro-6-phenoxybenzonitrile **24** (107 mg, 0.500 mmol, 1.00 equiv.) and TTO (thianthrene-*S*-oxide, 116 mg, 0.500 mmol, 1.00 equiv.) in MeCN (2.0 mL, *c* = 0.25 M), trifluoroacetic anhydride (0.21 mL, 0.32 g, 1.5 mmol, 3.0 equiv.) was added at 0 °C, under an ambient atmosphere followed by addition of TfOH (49 µl, 83 mg, 0.55 mmol, 1.1 equiv.). The reaction mixture was stirred at 0 °C for 1 h, and then stirred (400 rpm) at 25 °C for 16 h. The solvent was removed under reduced pressure by rotary evaporation and the residue was dissolved in DCM (10 mL). The resulting solution was washed with saturated aqueous $NaHCO_3$ solution (10 mL) and aqueous $NaBF_4$ solution (2 × 10 mL, 10% w/w). The DCM layer was dried over Na_2SO_4, filtered, and the solvent was removed under reduced pressure. The crude mixture was dissolved in DCM (5 mL) and Et_2O (50 mL) was added to obtain the product as white precipitate. The precipitate was filtered to give 227 mg of the title

compound **24a** as a colorless solid (88% yield).

R*f* = 0.28 (DCM / MeOH = 15:1 (v/v)).

NMR Spectroscopy:

^{1}H NMR (500 MHz, CD_3CN, 23 °C, δ): 8.37 (dd, *J* = 8.0, 1.4 Hz, 2H), 7.98 (dd, *J* = 8.0, 0.9 Hz, 2H), 7.90 (ddd, *J* = 7.9, 7.4, 1.4 Hz, 2H), 7.82 (ddd, *J* = 8.0, 7.4, 1.3 Hz, 2H), 7.66 (td, *J* = 8.6, 6.6 Hz, 1H), 7.25 – 7.11 (m, 5H), 6.85 (dt, *J* = 8.5, 0.9 Hz, 1H).

^{13}C NMR (126 MHz, CD_3CN, 23 °C, δ): 164.9 (d, *J* = 257.5 Hz), 160.3, 158.8 (d, *J* = 3.8 Hz), 137.6, 137.4 (d, *J* = 10.5 Hz), 136.2, 135.9, 131.8, 131.8, 131.0, 121.5, 119.8, 119.7, 116.2 (d, *J* = 3.5 Hz), 113.2 (d, *J* = 19.7 Hz), 111.7, 96.1 (d, *J* = 18.1 Hz).

^{19}F NMR (471 MHz, $CDCl_3$, 23 °C, δ): –103.3 (s, 1F), {–150.9 (bs), –150.9 (bs), 4F}.

HRMS-FIA(m/z) calc'd for $C_{25}H_{15}FNOS_2$ $[M–BF_4]^+$, 428.0574; found, 428.0573; deviation: +0.1 ppm.

N-Phenyl pyrazole-derived thianthrenium salt 25a

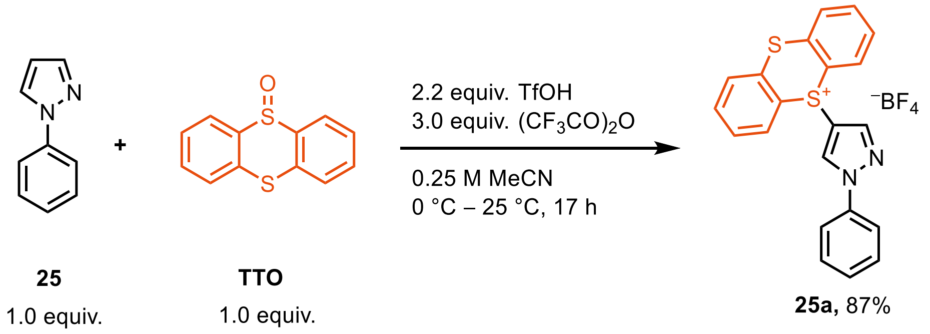

To a solution of *N*-phenylpyrazole **25** (66 µL, 72 mg, 0.5 mmol, 1.0 equiv.) and TTO (thianthrene-*S*-oxide, 0.12 g, 1.0 mmol, 1.0 equiv.) in MeCN (2.0 mL, *c* = 0.25 M), TfOH (49 µL, 83 mg, 0.55 mmol, 1.1 equiv.) and trifluoroacetic anhydride (0.21 mL, 0.31 g, 3.0 mmol, 3.0 equiv.) were added sequentially at 0 °C, under an ambient atmosphere followed by addition of TfOH (49 µL, 83 mg, 0.55 mmol, 1.1 equiv.). The reaction mixture was stirred (400 rpm) at 0 °C for 1 h, and then stirred at 25 °C for 16 h. The solvent was removed under reduced pressure by rotary evaporation and the residue was dissolved in DCM (15 mL). The resulting solution was washed with saturated aqueous $NaHCO_3$ solution (10 mL) and aqueous $NaBF_4$ solution (2 × 10 mL, 10% w/w). The DCM layer was dried over Na_2SO_4, filtered, and the solvent was removed under reduced pressure. Purification by flash silica gel column chromatography (EtOAc to DCM / MeOH = 20:1 (v/v)) afforded 410 mg of the title compound **25a** as a colorless solid (87% yield).

R*f* = 0.33 (DCM / MeOH = 15:1 (v/v)).

NMR Spectroscopy:

^{1}H NMR (500 MHz, $CDCl_3$, 23 °C, δ): 9.14 (s, 1H), 8.35 (d, *J* = 7.9 Hz, 2H), 7.87 (s, 1H), 7.84 (d, *J* = 7.9 Hz, 2H), 7.75 (t, *J* = 7.8 Hz, 2H), 7.63 (t, *J* = 8.5 Hz, 4H), 7.37 (t, *J* = 7.9 Hz, 2H), 7.29 (t, *J* = 7.4 Hz, 1H).

^{13}**C NMR** (126 MHz, $CDCl_3$, 23 °C, δ): 140.4, 138.5, 135.8, 134.7, 133.6, 133.3, 130.4, 130.3, 129.7, 128.5, 120.6, 119.7, 103.3.

^{19}**F NMR** (471 MHz, $CDCl_3$, 23 °C, δ): –149.6 (bs), –149.6 (bs).

HRMS-FIA(m/z) calc'd for $C_{21}H_{15}N_2S_2$ $[M–BF_4]^+$, 359.0671; found, 359.0673; deviation: –0.4 ppm.

N-Methyl pyrazole-derived thianthrenium salt 26a

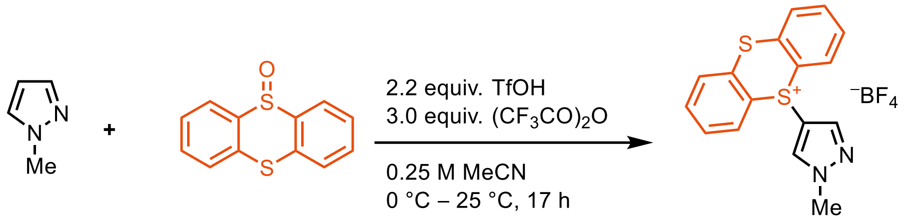

26 1.0 equiv. **TTO** 1.0 equiv. **26a,** 78%

To a solution of *N*-methylpyrazole **26** (42 µL, 41 mg, 0.50 mmol, 1.0 equiv.) and TTO (thianthrene-*S*-oxide, 0.12 g, 0.50 mmol, 1.0 equiv.) in MeCN (2.0 mL, c = 0.25 M), TfOH (49 µL, 83 mg, 0.55 mmol, 1.1 equiv.) and trifluoroacetic anhydride (0.21 mL, 0.32 g, 1.5 mmol, 3.0 equiv.) were added sequentially at 0 °C, under an ambient atmosphere followed by addition of TfOH (49 µL, 83 mg, 0.55 mmol, 1.1 equiv.). The reaction mixture was stirred at 0 °C for 1 h, and then stirred (400 rpm) at 25 °C for 16 h. The solvent was removed under reduced pressure by rotary evaporation and the residue was dissolved in DCM (10 mL). The resulting solution was washed with saturated aqueous $NaHCO_3$ solution (10 mL) and aqueous $NaBF_4$ solution (2 × 10 mL, 10% w/w). The DCM layer was dried over Na_2SO_4, filtered, and the solvent was removed under reduced pressure. The crude mixture was dissolved in DCM (5 mL) and MTBE (50 mL) was added to obtain the product as white precipitate. The precipitate was filtered to afford 151 mg of the title compound **26a** as a colorless solid (78% yield).

$\mathbf{R}_f$ = 0.36 (DCM / MeOH = 15:1 (v/v)).

NMR Spectroscopy:

^{1}**H NMR** (500 MHz, CD_3CN, 23 °C, δ): 8.13 (s, 1H), 8.09 (dd, J = 8.1, 1.4 Hz, 2H), 7.92 (dd, J = 7.9, 1.4 Hz, 2H), 7.79 (td, J = 7.6, 1.4 Hz, 2H), 7.75 (s, 1H), 7.69 (td, J = 7.7, 1.4 Hz, 2H), 3.85 (s, 3H).

^{13}**C NMR** (151 MHz, CD_3CN, 23 °C, δ): 141.3, 136.6, 136.1, 135.3, 133.3, 131.4, 130.9, 122.6, 118.4, 40.7.

^{19}**F NMR** (471 MHz, $CDCl_3$, 23 °C, δ): –151.1 (bs), –151.1 (bs).

HRMS-ESI(m/z) calc'd for $C_{16}H_{13}N_2S_2$ $[M–BF_4]^+$, 297.0515; found, 297.0512; deviation: +0.9 ppm.

1-(2-Methoxyphenyl)-*N,N*-dimethylmethanamine 27

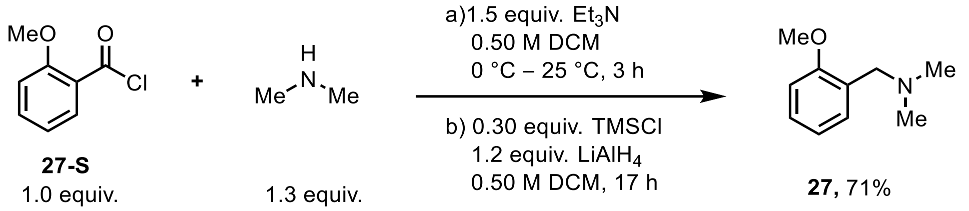

The compound **27** was prepared according to the modified versions of the reported procedures and the NMR spectra are in good accordance with reported data.[10-12] To a solution of dimethylamine (2.0 M in THF, 4.6 mL, 3.1 g, 9.1 mmol, 1.3 equiv.) and Et_3N (1.5 mL, 1.1 g, 11 mmol, 1.5 equiv.) in dry DCM (14 mL, *c* = 0.50 M) , acid chloride (1.0 mL, 1.2 g, 7.0 mmol, 1.0 equiv.) was added dropwise at 0 °C and the reaction mixture was stirred (400 rpm) at 25 °C for 3 h. Then, water (10 mL) was added, and the organic layer was separated. The aqueous layer was extracted with DCM (3 × 15 mL). The combined organic layer was washed with saturated aqueous $NaHCO_3$ solution (15 mL) followed by water (15 mL). After that, the organic layer was dried over Na_2SO_4 and concentrated under reduced pressure by rotary evaporation. The residue was transferred to a 25 mL 2-neck round-bottomed flask and dissolved in dry DCM (14 mL) under Ar atmosphere. The mixture was cooled to 0 °C and TMSCl (0.27 mL, 0.23 g, 2.1 mmol, 0.30 equiv.) was added slowly at 0 °C. Then $LiAlH_4$ (0.32 g, 8.4 mmol, 1.2 equiv.) was added portion wise at 0 °C. The reaction mixture was allowed to warm to 25 °C over 1 h and stirred for 16 h at 25 °C. Then 2 M sodium hydroxide (ca. 10 mL) was added and the mixture was stirred for 10 minutes. The mixture was transferred to a separatory funnel and layers were separated. The aqueous layer was washed with DCM (3 × 20 mL). The combined organic layers were dried over Na_2SO_4, filtered and concentrated under reduced pressure. Purification by flash column chromatography on silica gel (EtOAc only) afforded 820 mg of the title compound **27** as a colorless oil (71% yield)

NMR Spectroscopy:

^{1}H NMR (500 MHz, $CDCl_3$, 23 °C, δ): 7.30 (dd, *J* = 7.5, 1.8 Hz, 1H), 7.28 – 7.22 (m, 1H), 6.94 (td, *J* = 7.4, 1.1 Hz, 1H), 6.87 (dd, *J* = 8.2, 1.1 Hz, 1H), 3.83 (s, 3H), 3.47 (s, 2H), 2.29 (s, 6H).

^{13}C NMR (126 MHz, $CDCl_3$, 23 °C, δ): 157.8, 130.9, 128.1, 126.8, 120.1, 110.3, 57.9, 55.3, 45.4.

1-(2-Methoxyphenyl)-*N,N*-dimethylmethanamine-derived thianthrenium salt 27a

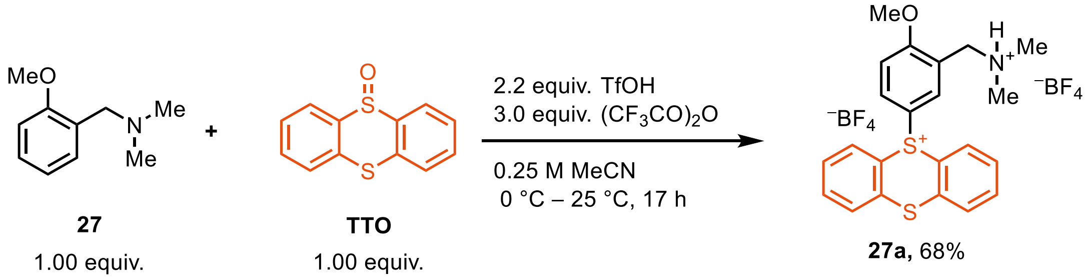

To a solution of 1-(2-methoxyphenyl)-*N,N*-dimethylmethanamine **27** (41 mg, 0.25 mmol, 1.0 equiv.)

and TTO (thianthrene-*S*-oxide, 58 mg, 0.25 mmol, 1.0 equiv.) in MeCN (1.0 mL, *c* = 0.25 M), TfOH (25 µL, 42 mg, 0.27 mmol, 1.1 equiv.) and trifluoroacetic anhydride (0.10 mL, 0.16 g, 0.75 mmol, 3.0 equiv.) were added sequentially at 0 °C, under an ambient atmosphere followed by addition of TfOH (25 µL, 42 mg, 0.27 mmol, 1.1 equiv.). The reaction mixture was stirred (400 rpm) at 0 °C for 1 h, and then stirred at 25 °C for 16 h. The solvent was removed under reduced pressure by rotary evaporation and the residue was dissolved in DCM (10 mL). The resulting solution was washed with saturated aqueous $NaHCO_3$ solution (10 mL) and aqueous $NaBF_4$ solution (2 × 10 mL, 10% w/w). The DCM layer was dried over Na_2SO_4, filtered, and the solvent was removed under reduced pressure. The residue was dissolved in MeCN (1 mL), and $HBF_4 \cdot OEt_2$ (37 µL, 45 mg, 0.27 mmol, 1.1 equiv.) was added. The solution was added dropwise to MTBE (40 mL) over 3 minutes with vigorous stirring (800 rpm) to obtain white precipitate. Then the solvent was removed by decantation, the precipitate was collected and dried in vacuo to afford 94 mg of the title compound **27a** as a colorless solid (68% yield).

R*f* = 0.14 (DCM / MeOH = 15:1 (v/v)).

NMR Spectroscopy:

^{1}H NMR (500 MHz, CD_3CN, 23 °C, δ): 8.33 (dd, *J* = 7.9, 1.5 Hz, 2H), 7.98 (dd, *J* = 7.9, 1.5 Hz, 2H), 7.90 (td, *J* = 7.6, 1.4 Hz, 2H), 7.82 (td, *J* = 7.7, 1.4 Hz, 2H), 7.32 (dd, *J* = 9.1, 2.7 Hz, 1H), 7.18 – 7.09 (m, 2H), 4.11 (s, 2H), 3.92 (s, 3H), 2.68 (s, 6H).

^{13}C NMR (126 MHz, CD_3CN, 23 °C, δ): 162.8, 137.4, 136.1, 135.7, 133.7, 133.2, 131.8, 131.0, 121.5, 120.1, 115.1, 114.5, 57.5, 57.3, 43.9, 41.3.

^{19}F NMR (471 MHz, $CDCl_3$, 23 °C, δ): –151.7 (bs), –151.8 (bs).

HRMS-ESI(m/z) calc'd for $C_{22}H_{23}NOS_2$ $[M–BF_4]^{2+}$, 190.5605; found, 190.5603; deviation: +1.2 ppm.

N,N-Dimethyl-1-(o-tolyl)methanamine 28

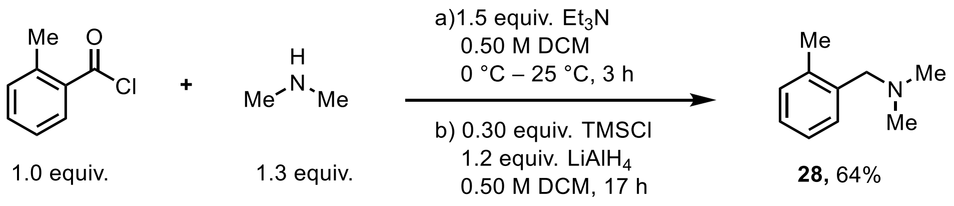

The compound **28** was prepared according to the modified versions of the reported procedures and the NMR spectra are in good accordance with reported data.[11,12] To a solution of dimethylamine (2.0 M in THF, 5.2 mL, 3.4 g, 10 mmol, 1.3 equiv.) and Et_3N (1.7 mL, 1.2 g, 12 mmol, 1.5 equiv.) in dry DCM (16 mL, *c* = 0.50 M), acid chloride (1.1 mL, 1.2 g, 8.0 mmol, 1.0 equiv.) was added dropwise at 0 °C and the reaction mixture was stirred (400 rpm) at 25 °C for 3 h. Then, water (10 mL) was added, and the organic layer was separated. The aqueous layer was extracted with DCM (3 × 15 mL). The combined organic layers was washed with saturated aqueous $NaHCO_3$ solution (15 mL) followed by water (15 mL). After that, the organic layer was dried over Na_2SO_4 and concentrated under reduced pressure by rotary evaporation. The residue was transferred to a 25 mL 2-neck round-bottomed flask and dissolved in dry DCM (14 mL) under Ar atmosphere. The mixture was cooled to 0 °C and TMSCl

(0.31 mL, 0.26 g, 2.4 mmol, 0.30 equiv.) was added at 0 °C over approximately 30 seconds. Then $LiAlH_4$ (0.37 g, 9.6 mmol, 1.2 equiv.) was added portion wise at 0 °C. The reaction mixture was allowed to warm to 25 °C over 1 h and stirred (400 rpm) for 16 h at 25 °C. Then 2 M sodium hydroxide (ca. 10 mL) was added and the mixture was stirred for 10 minutes. The mixture was transferred to a separatory funnel and layers were separated. The aqueous layer was washed with DCM (3 × 20 mL). The combined organic layers were dried over Na_2SO_4, filtered and concentrated under reduced pressure. Purification by flash column chromatography on silica gel (EtOAc only) afforded 766 mg of the title compound **28** as colorless oil (64% yield)

NMR Spectroscopy:

^{1}H NMR (500 MHz, $CDCl_3$, 23 °C, δ): 7.31 – 7.25 (m, 1H), 7.22 – 7.11 (m, 3H), 3.40 (s, 2H), 2.39 (s, 3H), 2.27 (s, 6H)

^{13}C NMR (126 MHz, CD_3CN, 23 °C, δ): 137.4, 137.2, 130.3, 130.0, 127.1, 125.6, 62.2, 45.7, 19.2.

N,N-Dimethyl-1-(o-tolyl)methanamine-derived thianthrenium salt 28a

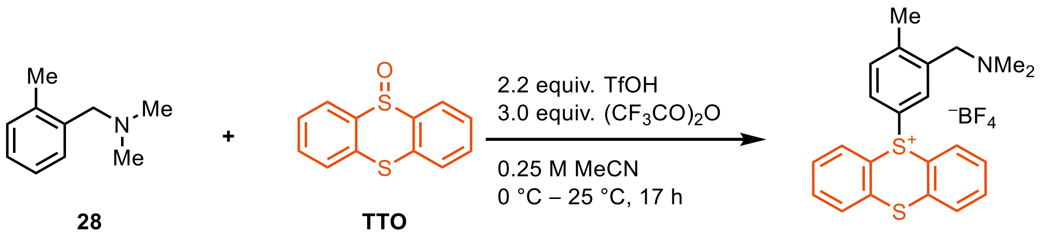

28 1.00 equiv. **TTO** 1.00 equiv. **28a**, 58%

To a solution of *N,N*-dimethyl-1-(o-tolyl)methanamine **28** (74.6 mg, 0.500 mmol, 1.00 equiv.) and TTO (thianthrene-*S*-oxide, 116 mg, 0.500 mmol, 1.00equiv.) in MeCN (2.0 mL, *c* = 0.25 M), TfOH (49 µL, 83 mg, 0.55 mmol, 1.1 equiv.) and trifluoroacetic anhydride (0.21 mL, 0.32 g, 1.5 mmol, 3.0 equiv.) were added sequentially at 0 °C, under an ambient atmosphere followed by addition of TfOH (49 µL, 83 mg, 0.55 mmol, 1.1 equiv.). The reaction mixture was stirred at 0 °C for 1 h, and then stirred (400 rpm) at 25 °C for 16 h. The solvent was removed under reduced pressure by rotary evaporation and the residue was dissolved in DCM (10 mL). The resulting solution was washed with saturated aqueous $NaHCO_3$ solution (10 mL) and aqueous $NaBF_4$ solution (2 × 10 mL, 10% w/w). The DCM layer was dried over Na_2SO_4, filtered, and the solvent was removed under reduced pressure. Purification by flash column chromatography on silica gel (DCM to DCM / MeOH / Et_3N = 10:1:0.1 (v/v)) afforded 132 mg of the title compound **28a** as a colorless solid (58% yield).

R_f = 0.12 (DCM / MeOH = 15:1 (v/v)).

NMR Spectroscopy:

^{1}H NMR (500 MHz, $CDCl_3$, 23 °C, δ): 8.36 (d, *J* = 9.2 Hz, 2H), 7.95 (d, *J* = 6.6 Hz, 2H), 7.88 (t, *J* = 7.6 Hz, 2H), 7.81 (t, *J* = 8.4 Hz, 2H), 7.29 (d, *J* = 8.2 Hz, 1H), 7.12 (s, 1H), 6.94 (dd, *J* = 8.3, 2.5 Hz, 1H), 3.42 (s, 2H), 2.32 (s, 3H), 2.14 (s, 6H).

^{13}C NMR (126 MHz, $CDCl_3$, 23 °C, δ): 143.9, 138.9, 136.5, 135.4, 135.0, 132.7, 130.4, 130.3,

129.0, 127.0, 120.9, 119.1, 60.1, 44.9, 27.1, 19.3.

^{19}F NMR (471 MHz, $CDCl_3$, 23 °C, δ): –151.4 (bs), –151.5 (bs).

HRMS-ESI(m/z) calc'd for $C_{22}H_{22}NS_2$ [M–BF_4]$^+$, 364.1188; found, 364.1187; deviation: +0.3 ppm.

N,N-Dimethyl-2-phenylpropame-1-amine 29

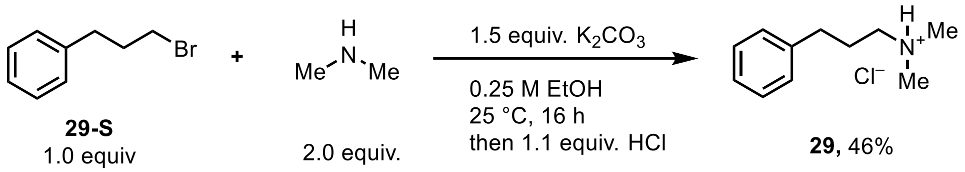

29-S 1.0 equiv; 2.0 equiv.; **29,** 46%

The compound **29** was prepared according to the modified versions of the reported procedures and the NMR spectra are in good accordance with reported data.[13,14] To a solution of 3-phenylpropylbromide **29-S** (0.76 mL, 0.10 g, 5.0 mmol, 1.0 equiv.) in EtOH (20 mL, c = 0.25 M), Me_2NH (40% in H_2O (w/w), 1.7 mL, 1.1 g, 10 mmol, 2.0 equiv.) was added at 25 °C followed by addition of K_2CO_3 (1.1 g, 7.5 mmol, 1.5 equiv.). The reaction mixture was stirred at 25 °C for 16 h. The solvent was removed under reduced pressure by rotary evaporation. The residue was dissolved in DCM (15 mL) and washed with H_2O (15 mL). The organic phase was dried over Na_2SO_4, filtered and concentrated under reduced pressure. The residue was purified by flash column chromatography on silica gel (hexanes / EtOAc = 1:1(v/v)) and then dissolved in DCM (2 mL).Concentrated HCl solution (0.40 mL, 12 M, 0.60 g, 5.5 mmol, 1.1 equiv.) was added to afford 460 mg of the title compound **29·HCl** as a colorless solid (46%).

NMR Spectroscopy:

^{1}H NMR (500 MHz, CD_3CN, 23 ºC, δ): 7.34 – 7.17 (m, 5H), 3.03 – 2.91 (m, 2H), 2.69 (t, *J* = 7.8 Hz, 2H), 2.67 (s, 6H), 2.16 – 2.00 (m, 2H).

^{13}C NMR (126 MHz, CD_3CN, 23 ºC, δ): 141.7, 129.5, 129.4, 127.2, 57.6, 42.9, 33.1, 26.6.

N,N-Dimethyl-2-phenylpropane-1-amine-derived thianthrenium salt 29a

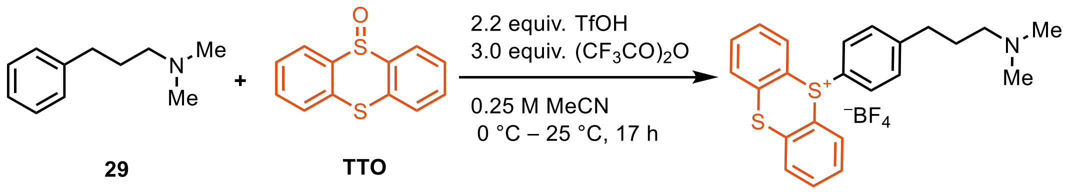

29 1.00 equiv.; **TTO** 1.00 equiv.; **29a,** 64%

A solution of **29·HCl** (232 mg, 0.250 mmol, 1.00 equiv.) in EtOAc (3 mL) was washed with saturated aquesous Na_2CO_3 solution (3 mL). The organic layer was dried over Na_2SO_4, filtered, and concentrated under reduced pressure by rotary evaporation. The residue was dissolved in MeCN (1.0 mL, *c* = 0.25 M) and added to a 20-mL glass vial containing TTO (thianthrene-*S*-oxide, 58.1 mg, 0.250 mmol, 1.00 equiv.). The mixture was cooled to 0 °C with an ice bath and trifluoroacetic anhydride (104 µL, 157 mg, 0.750 mmol, 3.00 equiv.) was added at 0 °C, under an ambient atmosphere. The reaction

mixture was stirred (400 rpm) at 0 °C for 1 h, and then stirred at 25 °C for 16 h. The solvent was removed under reduced pressure by rotary evaporation and the residue was dissolved in DCM (10 mL). The resulting solution was washed with saturated aqueous $NaHCO_3$ solution (10 mL) and aqueous $NaBF_4$ solution (2 × 10 mL, 10% w/w). The DCM layer was dried over Na_2SO_4, filtered, and the solvent was removed under reduced pressure. Purification by flash column chromatography on silica gel (DCM / MeOH / Et_3N = 30:1:0.1 (v/v)) afforded 74.0 mg of the title compound **29a** as a colorless solid (64% yield).

R_f = 0.11 (DCM / MeOH = 15:1 (v/v)).

NMR Spectroscopy:

^{1}H NMR (500 MHz, CD_3CN, 23 ºC, δ): 8.37 (dd, *J* = 7.9, 1.4 Hz, 2H), 7.96 (dd, *J* = 8.0, 1.4 Hz, 2H), 7.89 (td, *J* = 7.7, 1.4 Hz, 2H), 7.82 (td, *J* = 7.7, 1.4 Hz, 2H), 7.36 (d, *J* = 8.9 Hz, 2H), 7.09 (d, *J* = 8.7 Hz, 2H), 3.06 (dt, *J* = 9.0, 4.3 Hz, 2H), 2.79 (s, 6H), 2.72 – 2.60 (m, 2H), 1.98 – 1.84 (m, 2H).

^{13}C NMR (126 MHz, CD_3CN, 23 ºC, δ): 147.6, 137.5, 136.2, 136.0, 131.7, 131.6, 131.0, 129.3, 122.5, 119.6, 58.4, 44.1, 32.3, 26.3.

^{19}F NMR (471 MHz, $CDCl_3$, 23 ºC, δ): –151.5 (bs), –151.5 (bs).

HRMS-API (m/z) calc'd for $C_{23}H_{24}NS_2$ $[M–BF_4]^+$, 378.1345; found, 378.1344; deviation: –0.1 ppm.

4-Phenoxyquinoline 30

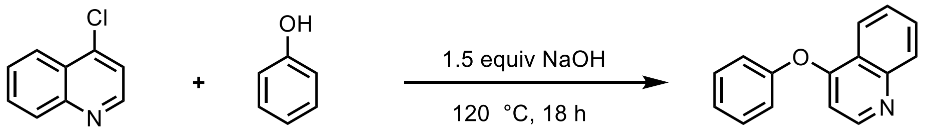

30-S 1.0 equiv. 5.0 equiv **30,** 83%

The compound **30** was prepared according to the reported procedure and the NMR spectra are in good accordance with reported data.[15] A mixture of the 4-chloroquinoline substrate **30-S** (0.26 mL, 0.33 g, 2.0 mmol, 1.0 equiv.), the phenol (0.94 g, 10 mmol, 5.0 equiv.), and NaOH (crushed pellets) (0.12 g, 3.0 mmol, 1.5 equiv.) was stirred (600 rpm) at 120 °C for 18 h. Then the reaction mixture was cooled to 25 °C and diluted with 10% aqueous NaOH solution (ca. 5 mL) and stirred at 25 °C for 1 h. The aqueous phase was then extracted with DCM (3 × 20 mL). The combined organic layers were washed with brine, dried over Na_2SO_4, filtered and concentrated under reduced pressure by rotary evaporation. Purified by flash column chromatography on silica gel (hexanes / EtOAc = 4:1 (v/v)) to afford 365 mg of title compound **30** as colorless oil (83% yield)

R_f = 0.34 (hexanes / EtOAc = 2:1 (v/v))

NMR Spectroscopy:

^{1}H NMR (500 MHz, $CDCl_3$, 23 °C, δ): 8.66 (d, *J* = 5.2 Hz, 1H), 8.36 (d, *J* = 8.2 Hz, 1H), 8.11 (d, *J* = 8.5 Hz, 1H), 7.84 – 7.69 (m, 1H), 7.64 – 7.52 (m, 1H), 7.51 – 7.38 (m, 2H), 7.35 – 7.24 (m, 1H),

7.22 – 7.13 (m, 2H), 6.54 (dd, *J* = 5.6, 2.5 Hz, 1H).

^{13}C NMR (126 MHz, $CDCl_3$, 23 °C, δ): 162.0, 154.5, 151.1, 149.8, 130.4, 130.3, 129.1, 126.2, 125.7, 121.9, 121.6, 121.2, 104.4.

HRMS-GC-EI(m/z) calc'd for $C_{15}H_{11}NO$ $[M]^+$, 221.0835; found, 221.0838; deviation: –1.3 ppm.

4-Phenoxyquinoline-derived thianthrenium salt 30a

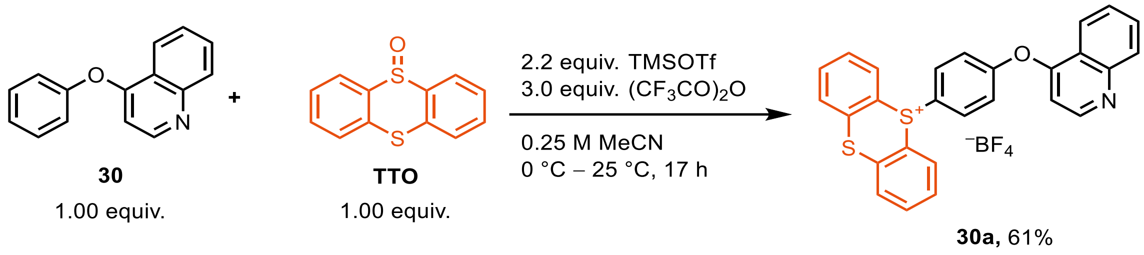

To a solution of 4-phenoxyquinoline **30** (44.3 mg, 0.200 mmol, 1.00 equiv.) and TTO (thianthrene-*S*-oxide, 46.5 mg, 0.200 mmol, 1.00 equiv.) in MeCN (0.8 mL, *c* = 0.25 M), TMSOTf (45 µL, 56 mg, 0.25 mmol, 1.1 equiv.) and atmosphere trifluoroacetic anhydride (0.21 mL, 0.32 g, 1.5 mmol, 3.0 equiv.) were added sequentially at 0 °C, under an ambient was added at 0 °C, followed by addition of TMSOTf (45 µL, 56 mg, 0.25 mmol, 1.1 equiv.). The reaction mixture was stirred (400 rpm) at 0 °C for 1 h, and then stirred at 25 °C for 16 h. The solvent was removed under reduced pressure by rotary evaporation and the residue was dissolved in DCM (10 mL). The resulting solution was washed with saturated aqueous $NaHCO_3$ solution (10 mL) and aqueous $NaBF_4$ solution (2 × 10 mL, 10% w/w). The DCM layer was dried over Na_2SO_4, filtered, and the solvent was removed under reduced pressure. Purification by flash silica gel column chromatography (DCM to DCM / MeOH = 10:1 (v/v)) afforded 64 mg of the title **30a** compound as a colorless solid (61% yield).

R_f = 0.37 (DCM / MeOH = 15:1 (v/v)).

NMR Spectroscopy:

^{1}H NMR (600 MHz, CD_3CN, 23 °C, δ): 8.68 (d, *J* = 5.1 Hz, 1H), 8.49 (dd, *J* = 8.0, 1.4 Hz, 2H), 8.07 – 8.02 (m, 2H), 7.84 (dd, *J* = 7.9, 1.4 Hz, 2H), 7.80 (td, *J* = 7.6, 1.4 Hz, 2H), 7.75 – 7.69 (m, 3H), 7.51 (ddd, *J* = 8.2, 6.9, 1.2 Hz, 1H), 7.29 – 7.25 (m, 2H), 7.18 – 7.14 (m, 2H), 6.68 (d, *J* = 5.1 Hz, 1H).

^{13}C NMR (151 MHz, CD_3CN, 23 °C, δ): 159.9, 158.9, 150.8, 149.3, 136.5, 135.3, 135.2, 130.9, 130.8, 130.6, 130.5, 128.7, 127.1, 122.0, 121.6, 121.4, 119.4, 118.6, 106.8.

^{19}F NMR (565 MHz, $CDCl_3$, 23 °C, δ): –151.5 (bs), –151.6 (bs).

HRMS-ESI(m/z) calc'd for $C_{27}H_{18}ONS_2$ $[M–BF_4]^+$, 436.0824; found, 436.0824; deviation: +0.2 ppm.

8-Butyloxy quinoline 31

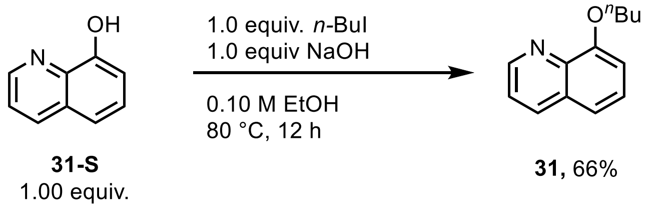

To a solution of 8-quinolinol **31-S** (73 mg, 0.50 mmol, 1.0 equiv.) in EtOH (c = 0.10 M, 5.0 ml) NaOH (20 mg, 0.50 mmol, 1.0 equiv.) and butyliodide (57 µL, 92 mg, 0.50 mmol, 1.0 equiv) were added at 25 °C. The reaction mixture was stirred at 80 °C for 12 h Then the reaction mixture was cooled to 25 °C and the solvent was removed by rotary evaporation. The residue was dissolved in DCM and washed with H_2O. Purification by flash column chromatography on silica gel (hexanes / EtOAc = 10:1 (v/v)) afforded product **31** (66 mg, 65% yield). The spectra are in accordance with the literature.[16]

NMR Spectroscopy:

^{1}H NMR (500 MHz, $CDCl_3$, 23 °C, δ): 8.96 (dd, J = 4.3, 1.8 Hz, 1H), 8.13 (dd, J = 8.3, 1.8 Hz, 1H), 7.65 – 7.34 (m, 3H), 7.07 (dd, J = 7.7, 1.3 Hz, 1H), 4.25 (t, J = 7.0 Hz, 2H), 2.01 (p, J = 7.1 Hz, 2H), 1.72 – 1.49 (m, 2H), 1.01 (t, J = 7.4 Hz, 3H)

^{13}C NMR (126 MHz, $CDCl_3$, 23 °C, δ): 154.8, 149.2, 140.3, 136.0, 129.5, 126.7, 121.5, 119.4, 108.7, 68.7, 31.0, 19.3, 13.9.

HRMS-ESI(m/z) calc'd for $C_{13}H_{15}NO$ [M–BF_4]$^+$, 211.1148; found, 211.1148; deviation: 0.0 ppm.

8-Butyloxyquinolone-derived thianthrenium salt 31a

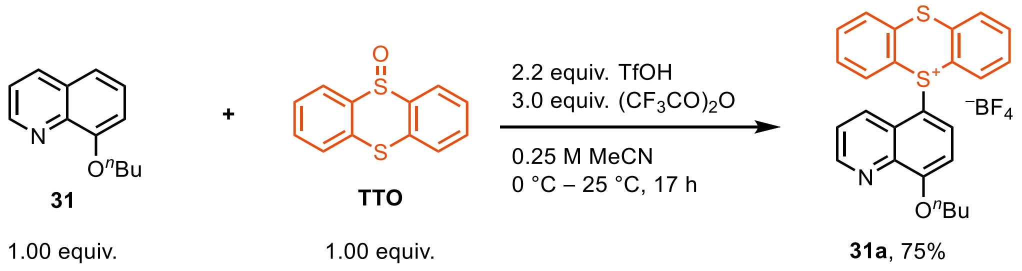

To a solution of 8-butyloxyquinoline **31** (116 mg, 0.579 mmol, 1.00 equiv.) and TTO (thianthrene-*S*-oxide, 134 mg, 0.579 mmol, 1.00 equiv.) in MeCN (2.2 mL, c = 0.25 M), TfOH (56 µL, 95 mg, 0.63 mmol, 1.1 equiv.) and trifluoroacetic anhydride (241 µL, 365 mg, 1.74 mmol, 3.00 equiv.) were added at 0 °C, under an ambient atmosphere followed by addition of TfOH (56 µL, 95 mg, 0.63 mmol, 1.1 equiv.). The reaction mixture was stirred at 0 °C for 1 h, and then stirred at 25 °C for 16 h. The solvent was removed under reduced pressure by rotary evaporation and the residue was dissolved in DCM (10 mL). The resulting solution was washed with saturated aqueous $NaHCO_3$ solution (10 mL) and aqueous $NaBF_4$ solution (2 × 10 mL, 10% w/w). The DCM layer was dried over Na_2SO_4, filtered, and the solvent was removed under reduced pressure. The residue was purified by flash column chromatography on silica gel (DCM / MeOH = 50:1 (v/v)) to afford 219 mg of the title compound **31a** (75% yield) as a yellow solid.

R*f* = 0.13 (DCM / MeOH = 50:1 (v/v)).

NMR Spectroscopy:

^{1}H NMR (500 MHz, $(CD_3)_2SO$, 23 °C, δ): 9.05 (dd, *J* = 4.1, 1.5 Hz, 1H), 8.91 (dd, *J* = 8.7, 1.5 Hz, 1H), 8.36 (dd, *J* = 8.2, 1.3 Hz, 2H), 8.10 (dd, *J* = 7.9, 1.3 Hz, 2H), 7.86 (td, *J* = 7.7, 1.3 Hz, 2H), 7.81 (dd, *J* = 8.7, 4.2 Hz, 1H), 7.74 (ddd, *J* = 8.5, 7.4, 1.3 Hz, 3H), 7.70 (d, *J* = 8.7 Hz, 1H), 4.25 (t, *J* = 6.4 Hz, 2H), 1.83 (dq, *J* = 8.6, 6.4 Hz, 2H), 1.60 – 1.27 (m, 2H), 0.94 (t, *J* = 7.4 Hz, 3H).

^{13}C NMR (126 MHz, $(CD_3)_2SO$, 23 °C, δ): 160.4, 151.0, 140.9, 135.2, 134.8, 134.7, 133.8, 131.9, 131.0, 130.4, 126.8, 124.5, 120.3, 109.7, 106.6, 69.5, 30.8, 19.2, 14.1.

^{19}F NMR (470 MHz, $(CD_3)_2SO$, 23 °C, δ): –148.2 (bs), –148.3 (bs).

HRMS-ESI(m/z) calc'd for $C_{25}H_{22}ONS_2$ $[M–BF_4]^+$, 416.1137; found,416.1137; deviation: +0.2 ppm.

6-Methoxy-2,3-dihydro-1*H*-inden-1-one 32

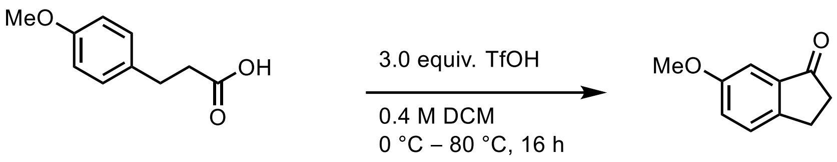

32-S
1.00 equiv.

32, 33%

The compound **32** was prepared according to the modified version of the reported procedure.[17] Under N_2 atmosphere, a 25-mL pressure tube was charged with *para*-hydroxyhydrocinnamate **32-S** (360 mg, 2.00 mmol, 1.00 equiv.) and dissolved in DCM (5.0 mL, *c* = 0.40 M). TfOH (0.53 mL, 0.90 g, 6.0 mmol, 3.0 equiv.) was added at 0 °C. Then the reaction mixture was allowed to warm to 25 °C, and subsequently heated to 80 °C in an oil bath. The reaction mixture was stirred at 80 °C for 16 h. Then the reaction mixture was poured into ice water (ca. 10 mL) and extracted with DCM (3 × 10 mL). The DCM layer were collected and dried over Na_2SO_4, filtered, and the solvent was removed under reduced pressure. The residue was purified by chromatography on silica gel (hexanes / EtOAc = 10:1 to 5:1 (v/v)) to afford 107 mg (33% yield) of the title compound **32** as a colorless solid.

R*f* = 0.36 (hexanes / EtOAc = 5:1 (v/v)).

NMR Spectroscopy:

^{1}H NMR (500 MHz, $CDCl_3$, 23 °C, δ): 7.36 (d, *J* = 8.3 Hz, 1H), 7.21 – 7.17 (m, 2H), 3.83 (s, 3H). 3.08 – 3.05 (m, 2H), 2.73 – 2.70 (m, 2H).

^{13}C NMR (126 MHz, $CDCl_3$, 23 °C, δ): 207.2, 159.5, 148.1, 138.4, 127.5, 124.2, 105.0, 55.7, 37.1, 25.3.

HRMSGC-EI(m/z) calc'd for $C_{10}H_{10}O_2$ $[M]^+$,162.0675; found, 162.0677; deviation: –1.2 ppm.

6-Methoxy-2,3-dihydro-1*H*-inden-1-one derived thianthrenium salt 32a

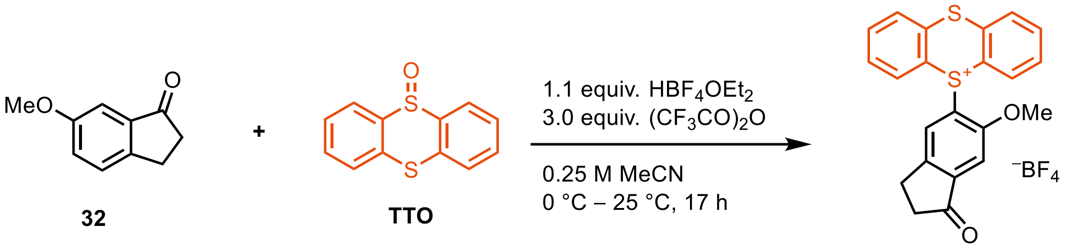

32 1.00 equiv. **TTO** 1.00 equiv. **32a**, 45%

To a solution of 6-methoxy-2,3-dihydro-1*H*-inden-1-one **32** (81.1 mg, 0.500 mmol, 1.00 equiv.) and TTO (thianthrene-*S*-oxide, 116 mg, 0.500 mmol, 1.00 equiv.) in MeCN (2.0 mL, *c* = 0.25 M), $HBF_4 \cdot OEt_2$ (75 µL, 89 mg, 0.55 mmol, 1.1 equiv.) and trifluoroacetic anhydride (208 µL, 315 mg, 1.50 mmol, 3.00 equiv.) were added at 0 °C, under an ambient atmosphere. The reaction mixture was stirred (400 rpm) at 0 °C for 1 h, and then stirred at 25 °C for 16 h. The solvent was removed under reduced pressure by rotary evaporation and the residue was dissolved in DCM (10 mL). The resulting solution was washed with saturated aqueous $NaHCO_3$ solution (10 mL) and aqueous $NaBF_4$ solution (2 × 10 mL, 10% w/w). The DCM layer was dried over Na_2SO_4, filtered, and the solvent was removed under reduced pressure. The residue was purified by flash column chromatography on silica gel (DCM / MeOH = 50:1 to 20:1 (v/v)) to afford 105 mg of the title compound **32a** (45% yield) as a yellow solid.

R*f* = 0.30 (DCM / MeOH = 10:1 (v/v)).

NMR Spectroscopy:

^{1}H NMR (500 MHz, CD_3CN, 23 °C, δ): 8.11 (d, *J* = 9.5 Hz, 1H), 7.82 (d, *J* = 8.0, 1.2 Hz, 2H), 7.71 – 7.64 (m, 4H), 7.49 (td, *J* = 8.3, 1.4 Hz, 2H), 7.42 (dd, *J* = 8.3, 1.1 Hz, 2H), 3.67 (s, 3H), 3.24 – 3.20 (m, 2H), 2.85 – 2.81 (m, 2H).

^{13}C NMR (126 MHz, CD_3CN, 23 °C, δ): 205.2, 161.4, 152.2, 139.5, 137.9, 134.0, 133.0, 130.3, 130.2, 129.5, 122.3, 122.2, 101.9, 55.1, 38.1, 25.8.

^{19}F NMR (470 MHz, CD_3CN, 23 °C, δ): –151.8 (bs), –151.9 (bs).

HRMS-ESI(m/z) calc'd for $C_{22}H_{17}O_2S_2$ $[M–BF_4]^+$, 377.0665; found,377.0663; deviation: +0.4 ppm.

Trifluoromethoxybenzene-derived thianthrenium salt 33a

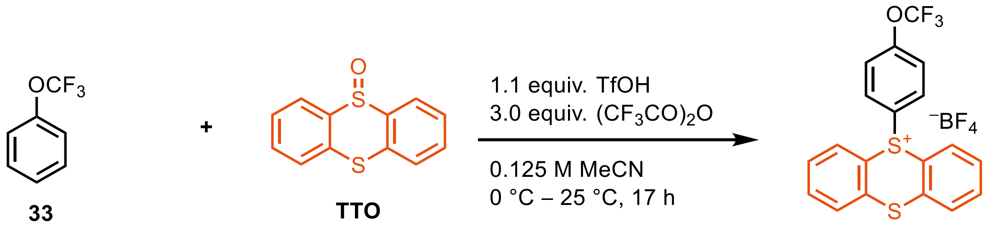

33 1.00 equiv. **TTO** 1.00 equiv. **33a**, 33%

Under ambient atmosphere, a 100 mL round-bottomed flask was charged with (trifluoromethoxy)benzol **33** (162 mg, 1.00 mmol, 1.00 equiv.), TTO (thianthrene-*S*-oxide, 232 mg, 1.00 mmol, 1.00 equiv.) and

dry MeCN (8.0 mL, *c* = 0.125 M). After cooling to 0 °C, trifluoroacetic anhydride (0.21 mL, 0.32 g, 0.15 mmol, 3.0 equiv.) was added at 0 °C dropwise, followed by addition of TfOH (49 µL, 0.6 mmol, 1.1 equiv.) in one portion at 0 °C. The mixture was stirred at 0 °C for 3 h and then warmed to 25 °C. After stirring at 25 °C for 14 h, the reaction mixture was concentrated under reduced pressure by rotary evaporation, and diluted with DCM (5 ml). The DCM phase was poured onto a saturated aqueous $NaHCO_3$ solution (10 mL). The mixture was poured into a separatory funnel, and the layers were separated. The DCM layer was collected, and the aqueous layer was further extracted with DCM (2 × 50 mL). The combined organic phases was washed with aqueous $NaBF_4$ solution (2 × 20 mL, 10 % w/w) and H_2O (20 ml). The organic layer was dried over Na_2SO_4, filtered, and the solvent was removed under reduced pressure. The residue was purified by chromatography on silica gel (DCM / MeOH (20:1 (v/v)). The product was collected and dried in vacuo to afford **33a** (153 mg, 33 %) as a colorless solid.

NMR Spectroscopy:

^{1}H NMR (600 MHz, CD_3CN, 23 °C, δ): 8.40 (ddd, *J* = 7.9, 1.4, 0.4 Hz, 2H), 7.98 (ddd, *J* = 8.0, 1.4, 0.4 Hz, 2H), 7.91 (ddd, *J* = 8.0, 7.5, 1.4 Hz, 2H), 7.84 (ddd, *J* = 8.0, 7.5, 1.4 Hz, 2H), 7.39 – 7.34 (m, 2H), 7.25 – 7.18 (m, 2H).

^{13}C NMR (151 MHz, CD_3CN, 23 °C, δ): 137.8, 136.4, 136.2, 132.5, 132.5, 131.9, 131.5, 131.0, 123.5, 123.4, 122.0, 121.1(q, *J* = 258.1 Hz).120.3, 119.4, 118.6.

^{19}F NMR (565 MHz, CD_3CN, 23 °C, δ): –58.8 (s, 3F), {(–151.7 (bs), –151.8 (bs), 4F}.

HRMS-ESI(m/z) calc'd for $C_{19}H_{12}OF_3S_2$ $[M–BF_4]^+$, 377.0276; found, 377.0277; deviation: –0.3 ppm.

N-Tosylindole-derived thianthrenium salt 34a

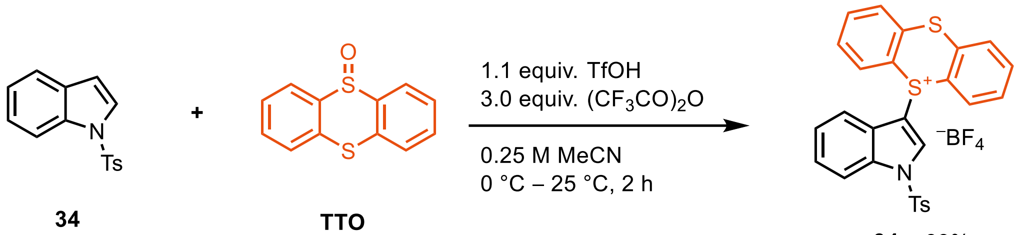

34 1.00 equiv. **TTO** 1.00 equiv. **34a,** 68%

To a solution of *N*-tosylindole **34** (136 mg, 0.500 mmol, 1.00 equiv.) and TTO (thianthrene-*S*-oxide, 116 mg, 0.500 mmol, 1.00 equiv.) in MeCN (2.0 mL, *c* = 0.25 M), trifluoroacetic anhydride (0.21 mL, 0.32 g, 1.5 mmol, 3.0 equiv.) was added at 0 °C, under an ambient atmosphere followed by addition of TfOH (49 µl, 83 mg, 0.55 mmol, 1.1 equiv.). The reaction mixture was stirred at 0 °C for 1 h, and then stirred (400 rpm) at 25 °C for 1 h. The solvent was removed under reduced pressure by rotary evaporation and the residue was dissolved in DCM (10 mL). The resulting solution was washed with saturated aqueous $NaHCO_3$ solution (10 mL) and aqueous $NaBF_4$ solution (2 × 10 mL, 10% w/w). The DCM layer was dried over Na_2SO_4, filtered, and the solvent was removed under reduced pressure. The crude mixture was dissolved in DCM (5 mL) and Et_2O (50 mL) was added to obtain the product as white precipitate. The precipitate was filtered to give 194 mg of the title compound **34a** as a colorless solid (68% yield).

R$_f$ = 0.40 (DCM / MeOH = 10:1 (v/v)).

NMR Spectroscopy:

^{1}H NMR (300 MHz, CD_3CN, 23 °C, δ): 8.17 (s, 1H), 8.08 (d, *J* = 8.7 Hz, 1H), 8.01 – 7.87 (m, 6H), 7.81 (t, *J* = 7.9 Hz, 2H), 7.64 (t, *J* = 7.9 Hz, 2H), 7.50 (t, *J* = 8.4 Hz, 1H), 7.44 (d, *J* = 8.2 Hz, 2H), 7.25 (t, *J* = 7.6 Hz, 1H), 7.10 (t, *J* = 7.6 Hz, 1H), 2.40 (s, 3H).

^{13}C NMR (76 MHz, DMSO-d_6, 23 °C, δ): 147.1, 134.4, 134.3, 134.2, 134.0, 133.0,132.6, 130.7, 130.4, 129.7, 127.6, 126.9, 125.2, 124.6, 120.0, 119.4, 114.3, 98.7, 21.1.

^{19}F NMR (282 MHz, CD_3CN, 23 °C, δ): –151.7 (bs), –151.8 (bs).

HRMS-ESI(m/z) calc'd for $C_{27}H_{20}N_1O_2S_3$ $[M–BF_4]^+$, 486.0651; found, 486.0653; deviation: –0.5 ppm.

1,4-Benzodioxane-5-carboxylic acid methyl ester 35

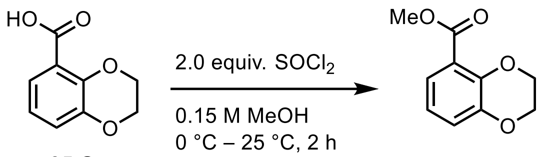

35-S
1.00 equiv.

35, 70%

100 mL round-bottomed flask equipped with a Teflon-coated magnetic stirring bar was charged with 1,4-Benzodioxane-5-carboxylic acid **35-S** (1.08 g, 5.00 mmol, 1.00 equiv.). The solid was dissolved in MeOH (40 mL, *c* = 0.15 M) and the solution was cooled to 0 °C. Thionyl chloride (0.87 mL, 1.4 g, 12 mmol, 2.0 equiv.) was added at 0 °C. Then the reaction mixture was allowed to warm to 25 °C and stirred for 2 h at 25 °C. Saturated aqueous $NaHCO_3$ solution (10 ml) was added to the reaction mixture, and MeOH was evaporated under reduced pressure by rotary evaporation. The resulting mixture was extracted with EtOAc (3 × 20 mL), the combined organic layers were washed with brine, dried over $MgSO_4$, filtered and concentrated under reduced pressure. The residue was washed with diethylether to afford **35** (810 mg, 70% yield) of the title compound as a colorless solid. The spectra are in accordance with literature.[18]

NMR Spectroscopy:

^{1}H NMR (300 MHz, $CDCl_3$, 23 °C, δ): 7.41 (dd, *J* = 7.8, 1.7 Hz, 1H), 7.04 (dd, *J* = 8.1, 1.7 Hz, 1H), 6.87 (t, *J* = 7.9 Hz, 1H), 4.47 – 4.36 (m, 1H), 4.35 – 4.22 (m, 1H), 3.91 (s, 3H).

^{13}C NMR (75 MHz, $CDCl_3$, 23 °C, δ): 166.4, 144.4, 124.0, 121.6, 120.7, 120.3, 64.9, 64.2, 52.4.

HRMS-GC-MS(m/z) calc'd for $C_{10}H_{10}O_4$ $[M]^+$, 194.0574; found, 194.0575; deviation: +0.9 ppm.

1,4-Benzodioxane-5-carboxylic acid methyl ester-derived thianthrenium salt 35a

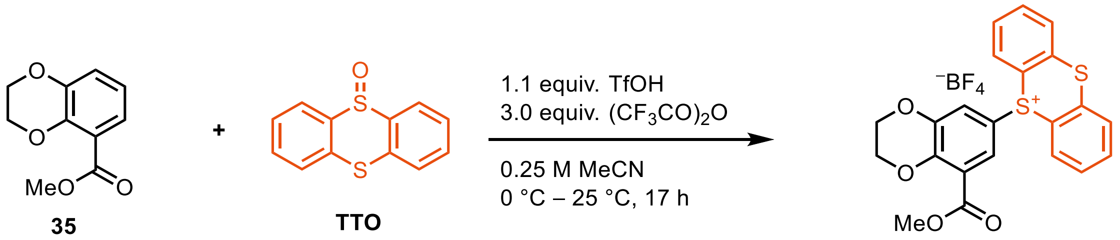

35 1.00 equiv. **TTO** 1.00 equiv. **35a**, 61%

To a solution of **35** (97.1 mg, 0.500 mmol, 1.00 equiv.) and TTO (thianthrene-*S*-oxide, 116 mg, 0.500 mmol, 1.00 equiv.) in MeCN (2.0 mL, *c* = 0.25 M), trifluoroacetic anhydride (0.21 mL, 0.32 mg, 1.5 mmol, 3.0 equiv.) was added at 0 °C, under an ambient atmosphere followed by addition of TfOH (49 µL, 90 mg, 0.60 mmol, 1.10 equiv.). The reaction mixture was stirred at 0 °C for 1 h, and then stirred at 25 °C for 16 h. The solvent was removed under reduced pressure by rotary evaporation and the residue was dissolved in DCM (10 mL). The resulting solution was washed with saturated aqueous $NaHCO_3$ solution (10 mL) and aqueous $NaBF_4$ solution (2 × 10 mL, 10% w/w). The DCM layer was dried over Na_2SO_4, filtered, and the solvent was removed under reduced pressure. Purification by flash column chromatography on silica gel (DCM to DCM / MeOH = 20:1 (v/v)) afforded 151 mg of the title compound **35a** as a colorless solid (61% yield).

NMR Spectroscopy:

^{1}H NMR (600 MHz, CD_3CN, 23 °C, δ): 8.32 (ddd, *J* = 8.0, 1.4, 0.4 Hz, 2H), 7.94 (dd, *J* = 7.9, 1.4 Hz, 2H), 7.87 (dd, *J* = 7.4, 1.4 Hz, 2H), 7.79 (ddd, *J* = 8.0, 7.5, 1.3 Hz, 2H), 7.06 (d, *J* = 2.7 Hz, 1H), 6.80 (d, *J* = 2.6 Hz, 1H), 4.34 – 4.29 (m, 2H), 4.27 – 4.22 (m, 2H), 3.75 (s, 3H).

^{13}C NMR (151 MHz, CD_3CN, 23 °C, δ): 164.9, 148.9, 147.1, 137.2, 136.2, 135.7, 131.6, 131.1, 123.1, 124.1, 120.4, 119.7, 115.0, 65.9, 65.0, 53.3.

^{19}F NMR (565 MHz, CD_3CN, 23 °C, δ):, –151.2 (bs), –151.3 (bs).

HRMS-ESI(m/z) calc'd for $C_{22}H_{17}O_4S_2$ $[M–BF_4]^+$, 409.0563; found, 409.0564; deviation: –0.3 ppm.

Biphenly triflate 36

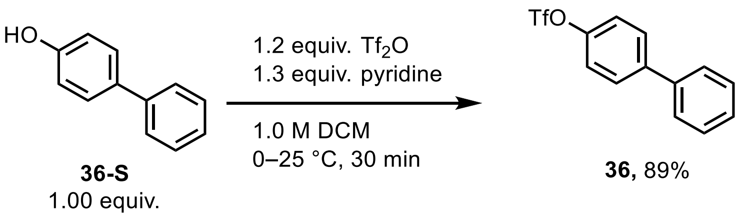

36-S 1.00 equiv. **36,** 89%

Prepared according to the reported procedure and spectra are in accordance with literature.[3] Under an ambient atmosphere, a 20 ml glass-vial was charged with 4-phenylphenol **36-S** (425 mg, 2.50 mmol, 1.00 equiv.), and DCM (2.5 mL, *c* = 1.0 M). After cooling to 0 °C, pyridine (0.26 mL, 0.26 g, 3.3 mmol, 1.3 equiv.), and triflic anhydride (0.51 mL, 0.84 g, 3.0 mmol, 1.2 equiv.) were added a 0 °C. The mixture was stirred at 0 °C for 20 min, then it was allowed to warm to 25 °C. The mixture was diluted

with 10 mL EtOAc, and subsequently washed with 30 ml water. The organic layer was dried over $MgSO_4$, filtered, and the solvent was removed under reduced pressure. The residue was purified by chromatography on silica gel (hexanes / EtOAc = 1:0 to 10:1 (v/v)) to afford 670 mg (89 %) of **36** as a colorless solid.

NMR Spectroscopy:

^{1}H NMR (300 MHz, CD_3CN, 23 °C, δ): 7.65 (d, *J* = 9.0 Hz, 2H), 7.57 (d, *J* = 6.7 Hz, 2H), 7.45 (m, 3H), 7.35 (d, *J* = 8.8 Hz, 2H)

^{13}C NMR (75 MHz, CD_3CN, 23 °C, δ): 149.1, 141.9, 139.4, 129.1, 129.0, 128.2, 127.3, 121.1, 118.9 (d, *J* = 320.9 Hz):

^{19}F NMR (282 MHz, CD_3CN, 23 °C, δ): –72.8.

HRMS-ESI-TOF(m/z) calc'd for $C_{13}H_9O_3F_3S_2$ $[M–BF_4]^+$: 302.0219, found: 302.0221; deviation: –0.9 ppm

Biphenyl triflate-derived thianthrenium salt 36a

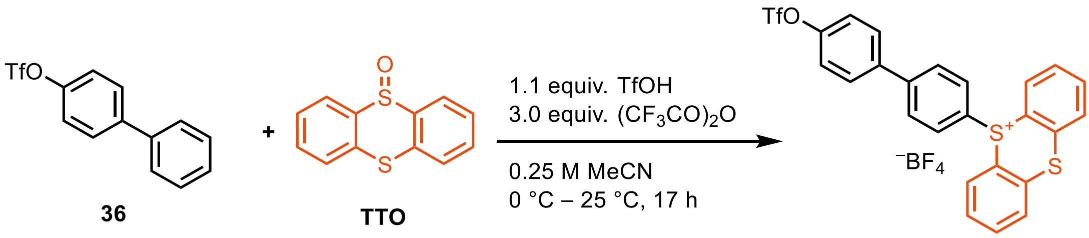

36 1.00 equiv. **TTO** 1.00 equiv. **36a,** 68%

To a solution of biphenyl triflate **36** (151 mg, 0.500 mmol, 1.00 equiv.) and TTO (thianthrene-*S*-oxide, 116 mg, 0.500 mmol, 1.00 equiv.) in MeCN (2.0 mL, *c* = 0.25 M) trifluoroacetic anhydride (0.21 mL, 0.32 g, 0.15 mmol, 3.0 equiv.) was added at 0 °C dropwise, followed by addition of TfOH (49 µL, 0.6 mmol, 1.1 equiv.). The mixture was stirred at 0 °C for 1 h, followed by warming the reaction mixture to 25 °C. After stirring at 25 °C for 16 h, the reaction mixture was concentrated under reduced pressure, and diluted with 10 mL DCM. The DCM phase was poured onto a saturated aqueous $NaHCO_3$ solution (10 mL). The mixture was poured into a separatory funnel, and the layers were separated. The DCM layer was collected, and the aqueous layer was further extracted with DCM (2 × 10 mL). The combined organic phases was washed with aqueous $NaBF_4$ solution (2 × 10 mL, 10 % w/w). The organic layer was dried over Na_2SO_4, filtered, and the solvent was removed under reduced pressure. The residue was purified by chromatography on silica gel (DCM / MeOH = 50:1 (v/v)). The product was collected and dried in vacuo to afford **36a** (205 mg, 68 %) as a colorless solid.

NMR Spectroscopy:

^{1}H NMR (600 MHz, CD_3CN, 23 °C, δ): 8.42 (dd, *J* = 7.9, 1.4 Hz, 2H), 7.95 (dd, *J* = 8.0, 1.4 Hz, 2H), 7.90 (td, *J* = 7.7, 1.3 Hz, 2H), 7.84 (td, *J* = 7.7, 1.4 Hz, 2H), 7.73 – 7.66 (m, 4H), 7.47 – 7.42 (m, 2H), 7.27 – 7.13 (m, 2H).

^{13}C NMR (151 MHz, CD_3CN, 23 °C, δ): 150.9, 144.5, 139.7, 137.6, 136.3, 136.2, 131.7, 131.1,

130.5, 130.1, 129.7, 124.4, 123.2, 120.8, 119.8 (d, J = 319.8 Hz), 118.7.

^{19}F NMR (565 MHz, CD_3CN, 23 °C, δ): –73.8 (s, 3F), {–151.3 (bs), –151.4 (bs), 4F}.

HRMS-ESI-TOF(m/z) calc'd for $C_{25}H_{16}O_3F_3S_3$ [M–BF_4]+: 517.0208, found: 517.0212; deviation: –0.8 ppm.

N-Phenylethylbenzamide-derived thianthrenium salt 37a

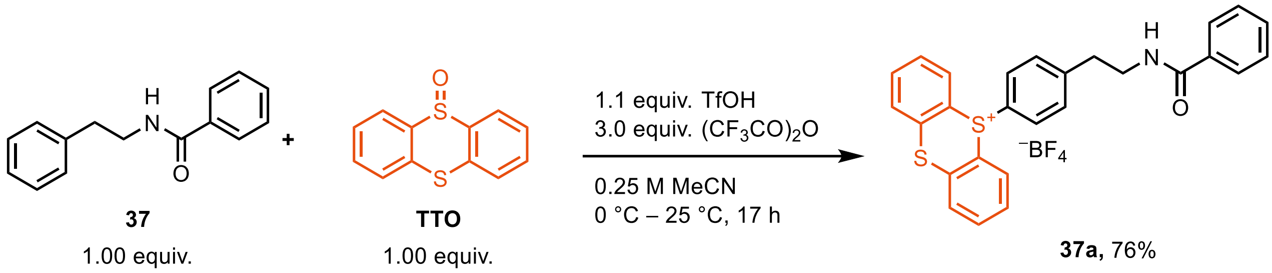

To a solution of *N*-phenethylbenzamide **37** (116 mg, 0.500 mmol, 1.00 equiv.) and TTO (thianthrene-*S*-oxide, 116 mg, 0.500 mmol, 1.00 equiv.) in MeCN (2.0 mL, c = 0.25 M), trifluoroacetic anhydride (0.21 mL, 0.32 g, 1.5 mmol, 3.0 equiv.) was added at 0 °C, under an ambient atmosphere followed by addition of TfOH (49 µl, 83 mg, 0.55 mmol, 1.1 equiv.). The reaction mixture was stirred (400 rpm) at 0 °C for 1 h, and then stirred at 25 °C for 16 h. The solvent was removed under reduced pressure by rotary evaporation and the residue was dissolved in DCM (10 mL). The resulting solution was washed with saturated aqueous $NaHCO_3$ solution (10 mL) and aqueous $NaBF_4$ solution (2 × 10 mL, 10% w/w). Purification by flash silica gel column chromatography (DCM / MeOH = 50:1 to 20:1 (v/v)) afforded 200 mg of the title compound **37a** as colorless solid (76% yield).

R_f = 0.21 (DCM / MeOH = 15:1 (v/v)).

NMR Spectroscopy:

^{1}H NMR (600 MHz, CD_3CN, 23 °C, δ): 8.33 (dd, J = 8.2, 1.2 Hz, 2H), 7.92 (dd, J = 8.2, 1.2 Hz, 2H), 7.87 (ddd, J = 7.9, 7.4, 1.4 Hz, 2H), 7.79 (ddd, J = 8.0, 7.4, 1.4 Hz, 2H), 7.65 – 7.61 (m, 2H), 7.51 – 7.47 (m, 1H), 7.43 – 7.38 (m, 2H), 7.37 – 7.34 (m, 2H), 7.10 (s, 1H), 7.04 (d, J = 2.1 Hz, 2H), 3.56 – 3.51 (m, 2H), 2.90 (t, J = 6.9 Hz, 2H).

^{13}C NMR (151 MHz, CD_3CN, 23 °C, δ): 167.8, 147.0, 137.5, 136.1, 135.9, 135.7, 132.2, 132.2, 131.6, 130.9, 129.4, 129.0, 127.8, 122.2, 119.8, 41.0, 35.8.

^{19}F NMR (565 MHz, $CDCl_3$, 23 °C, δ): –151.4 (bs), –151.5(bs).

HRMS-FIA(m/z) calc'd for $C_{27}H_{22}NOS_2$ [M–BF_4]$^+$, 440.1137; found, 440.1138; deviation: –0.1 ppm.

N-Methyl-*N*-phenyl-benzamide 38

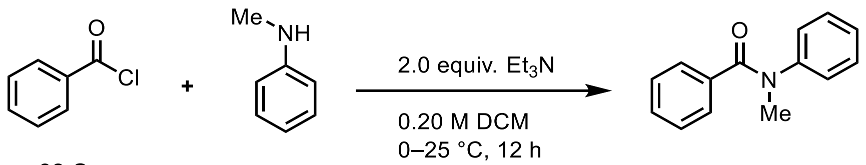

38-S 1.00 equiv. 1.2 equiv. **38,** 90%

Prepared according to the reported procedure and spectra are in accordance with the literature.[19] Under an ambient atmosphere, a 100 ml round-bottomed flask was charged with *N*-methylaniline (0.64 g, 6.0 mmol, 1.2 equiv.), and DCM (25 mL, *c* = 0.20 M). After cooling to 0 °C with an ice bath, BzCl **38-S** (0.58 ml, 0.70 g, 5.0 mmol, 1.0 equiv.) and Et_3N (1.4 mL, 1.0 g, 10 mmol, 2.0 equiv.) were added while stirring the reaction mixture. Then, the reaction mixture was allowed to warm up to 25°C. After stirring for 12 h, the solution was diluted with DCM (mL). The mixture was poured into a separatory funnel, and washed with HCl solution (ca. 100 mL, 1.0 M), and with saturated NaCl solution (ca. 100 ml). The organic layer was dried over Na_2SO_4, filtered, and the solvent was removed under reduced pressure. The residue was purified by chromatography on silica gel (pentane / EtOAc = 5:1 (v/v)) to afford 0.95 g (90% yield) of product **38** as a colorless oil.

NMR Spectroscopy:

^{1}**H NMR** (300 MHz, $CDCl_3$, 23 °C, δ): 7.36 – 7.28 (m, 2H), 7.27 – 7.13 (m, 6H), 7.09 – 7.02 (m, 2H), 3.51 (s, 3H).

^{13}**C NMR** (75 MHz, $CDCl_3$, 23 °C, δ): 170.7, 145.0, 136.0, 129.6, 129.2, 128.8, 127.8, 127.0, 126.5, 38.4.

HRMS-GC-EI(m/z) calc'd for $C_{14}H_{13}NO$ $[M]^+$, 211.0992; found, 211.0992; deviation: –0.3 ppm.

N-Methyl-*N*-phenyl-benzamide-derived thianthrenium salt 38a

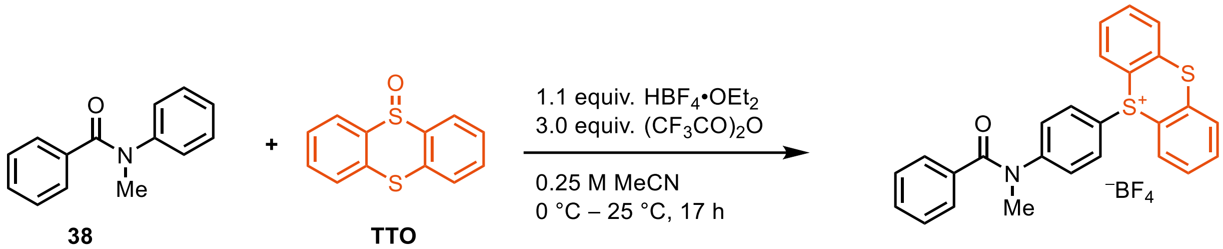

38 1.00 equiv. **TTO** 1.00 equiv. **38a,** 88%

Under ambient atmosphere, a 100 mL round-bottomed flask was charged with *N*-methyl-*N*-phenylbenzamide **38** (1.06 g, 5.00 mmol, 1.00 equiv.), TTO (thianthrene-*S*-oxide, 1.16 g, 5.00 mmol, 1.00 equiv.) and dry MeCN (20 mL, c = 0.25 M). After cooling to 0 °C, trifluoroacetic anhydride (2.1 mL, 15 mmol, 3.0 equiv.) was added at 0 °C dropwise, followed by $HBF_4·OEt_2$ (0.83 mL, 6.0 mmol, 1.2 equiv.) was added in one portion at 0 °C. The mixture was stirred at 0 °C for 1 h, followed by warming the reaction mixture to 25 °C. After stirring at 25 °C for 16 h, the reaction mixture was concentrated under reduced pressure, and diluted with DCM (50 mL). The DCM phase was poured onto a saturated aqueous

$NaHCO_3$ solution (50 mL). The mixture was poured into a separatory funnel, and the layers were separated. The DCM layer was collected, and the aqueous layer was further extracted with DCM (2 × 50 mL). The combined DCM solution was washed with aqueous $NaBF_4$ solution (2 × 100 mL, 10 % w/w). The organic layer was dried over Na_2SO_4, filtered, and the solvent was removed under reduced pressure. The residue was purified by chromatography on silica gel (DCM / MeOH = 50:1 (v/v)). The product was collected and dried in vacuo to afford **38a** (2.3 g, 88 %) as a colorless solid.

Rf = 0.47 (20:1, DCM:MeOH (v/v)).

NMR Spectroscopy:

^{1}**H NMR** (600 MHz, CD_3CN, 23 °C, δ): 8.54 – 8.45 (m, 2H), 7.86 – 7.68 (m, 6H), 7.34 – 7.25 (m, 1H), 7.23 – 7.17 (m, 4H), 7.15 – 7.09 (m, 2H), 7.03 – 6.94 (m, 2H), 3.40 (s, 3H).

^{13}**C NMR** (151 MHz, CD_3CN, 23 °C, δ): 170.7, 149.1, 136.6, 135.5, 135.2, 134.8, 130.7, 130.5, 130.5, 128.9, 128.7, 128.4, 128.0, 120.3, 118.8, 38.2

^{19}**F NMR** (565 MHz, CD_3CN, 23 °C, δ): –150.9 (bs), –150.9 (bs).

HRMS-ESI-TOF(m/z) calc'd for $C_{26}H_{20}NOS_2$ [M–BF_4]+: 426.0981, found: 426.0982; deviation: –0.3 ppm.

N-Methyl-*N*-phenyltoluenesulfonamide 39

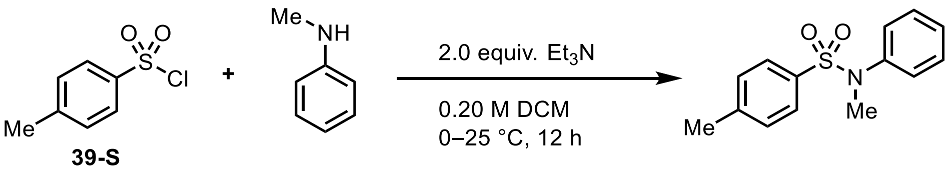

39-S 1.00 equiv. | 1.2 equiv. | **39,** 86%

Prepared according to the modified version of the reported procedure and spectra are in good accordance with the literature.[20] Under an ambient atmosphere, a 100 mL round-bottomed flask was charged with *N*-methylaniline (0.64 g, 6.0 mmol, 1.2 equiv.), and DCM (25 mL, c = 0.20 M). After cooling to 0 °C, TsCl **39-S** (0.95 g, 5.0 mmol, 1.0 equiv.) and Et_3N (1.4 mL, 1.0 g, 10 mmol, 2.0 equiv.) were added sequentially while stirring the reaction mixture. Then, the reaction mixture was allowed to warm up to 25°C. After stirring for 12 h, the solution was diluted with DCM (50 ml). The mixture was poured into a separatory funnel, and washed with HCl solution (ca. 100 ml, 1.0 M), and with saturated NaCl solution (ca. 100 ml). The organic layer was dried over Na_2SO_4, filtered, and the solvent was removed under reduced pressure. The residue was purified by chromatography on silica gel (pentane / EtOAc = 5:1 (v/v)) to afford 1.1 g (86 %) **39** as a colorless solid.

NMR Spectroscopy:

^{1}**H NMR** (300 MHz, $CDCl_3$, 23 °C, δ): 7.49 – 7.41 (m, 2H), 7.37 – 7.23 (m, 5H), 7.17 – 7.08 (m, 2H), 3.19 (s, 3H), 2.44 (s, 3H).

^{13}**C NMR** (126 MHz, $CDCl_3$, 23 °C, δ): 143.7, 141.8, 133.7, 129.5, 129.0, 128.1, 127.4, 126.8, 38.2, 21.7.

HRMS-ESI(m/z) calc'd for $C_{14}H_{15}NO_2S$ $[M]^+$, 261.0818; found, 261.0819; deviation: –0.5 ppm.

N-Methyl-N-phenyltoluenesulfonamide-derived thianthrenium salt 39a

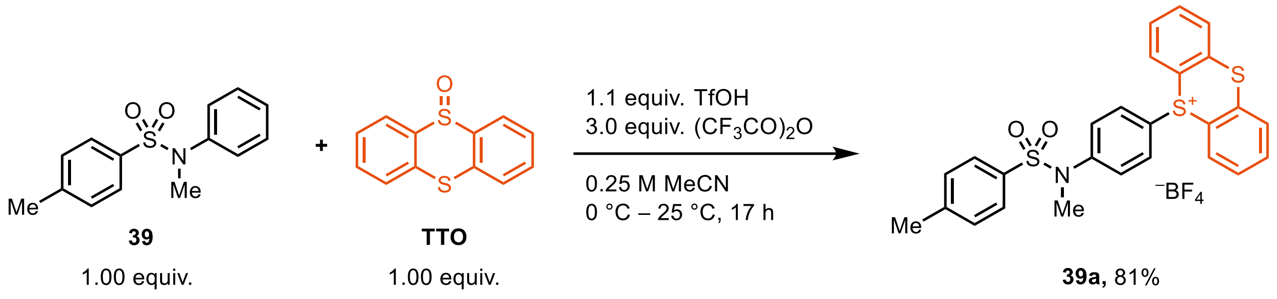

To a solution of **39** (653 mg, 2.50 mmol, 1.00 equiv.) and TTO (thianthrene-*S*-oxide, 581 mg, 2.50 mmol, 1.00 equiv.) in MeCN (10 mL, *c* = 0.25 M), trifluoroacetic anhydride (1.0 mL, 1.6 g, 7.5 mmol, 3.0 equiv.) was added at 0 °C, under an ambient atmosphere followed by addition of TfOH (243 µL, 413 mg, 2.75 mmol, 1.10 equiv.). The reaction mixture was stirred (400 rpm) at 0 °C for 1 h, and then stirred at 25 °C for 16 h. The solvent was removed under reduced pressure by rotary evaporation and the residue was dissolved in DCM (10 mL). The resulting solution was washed with saturated aqueous $NaHCO_3$ solution (10 mL) and aqueous $NaBF_4$ solution (2 × 10 mL, 10% w/w). The DCM layer was dried over Na_2SO_4, filtered, and the solvent was removed under reduced pressure. The crude mixture was dissolved in DCM (5 mL) and MTBE (50 mL) was added to obtain the product as a white precipitate. The precipitate was filtered to afford 241 mg of the title compound **39a** as a colorless solid (81% yield).

R*f* = 0.21 (DCM / MeOH = 30:1 (v/v)).

NMR Spectroscopy:

> **^{1}H NMR** (500 MHz, $CDCl_3$, 23 °C, δ): 8.36 (dd, *J* = 7.9, 1.5 Hz, 2H), 7.99 (dd, *J* = 7.9, 1.4 Hz, 2H), 7.90 (td, *J* = 7.8, 1.5 Hz, 2H), 7.82 (td, *J* = 7.7, 1.4 Hz, 2H), 7.34 (d, *J* = 8.4 Hz, 2H), 7.29 (d, *J* = 8.1 Hz, 2H), 7.27 – 7.22 (m, 2H), 7.04 (dd, *J* = 9.6, 2.6 Hz, 2H), 3.10 (s, 3H), 2.39 (s, 3H).
>
> **^{13}C NMR** (126 MHz, $CDCl_3$, 23 °C, δ): 146.9, 145.8, 137.6, 136.2, 136.1, 134.0, 131.8, 131.0, 130.7, 129.7, 128.5, 127.7, 121.9, 119.6, 38.0, 21.6.

HRMS-ESI(m/z) calc'd for $C_{26}H_{22}F_3NO_2S_3$ $[M–BF_4]^+$, 476.0807; found, 476.0804; deviation: +0.7 ppm.

N-Benzyl-2-methoxybenzamide 40

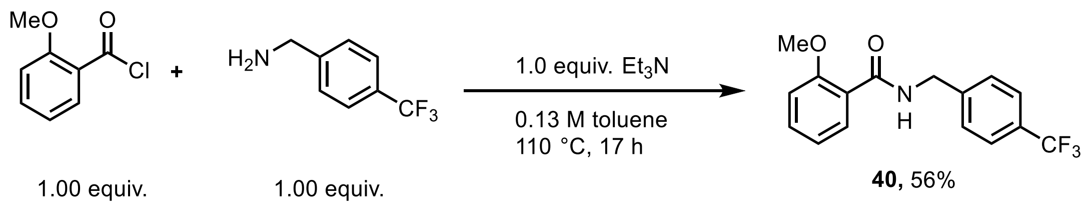

Prepared according to the reported procedure and the spectra are in good accordance with the literature.[21] To a solution of *para*-trifluoromethylbenzylamine (0.71 ml, 0.88 g, 5.0 mmol, 1.0 equiv.) and trimethylamine (0.70 ml, 0.51g, 5.0 mmol, 1.0 equiv.) in toluene (20 ml), 2-

methoxybenzoylchloride (0.74 ml, 0.85 g, 5.0 mmol, 1.0 equiv.) in toluene (20 ml) was added dropwise over 2 min. The reaction mixture was stirred (500 rpm) at 110 °C for 17 h. Then, the reaction mixture was cooled to room temperature, diluted with H_2O (ca. 50 ml) and EtOAc (ca. 50 ml), and transferred to a separatory funnel. Layers were separated and the organic phase was dried over anhydrous Na_2SO_4. The residue was purified by flash column chromatography on silica (hexanes / EtOAc = 20:1 (v/v) to EtOAc only) to afford **40** (0.86 g, 56% yield) as a colorless solid.

NMR Spectroscopy:

^{1}H NMR (300 MHz, $CDCl_3$, 23 °C, δ): 8.42 – 8.21 (m, 2H), 7.62 (d, *J* = 8.1 Hz, 2H), 7.56 – 7.45 (m, 3H), 7.14 (dd, *J* = 7.7, 1.0 Hz, 1H), 7.02 (dd, *J* = 8.4, 1.0 Hz, 1H), 4.77 (d, *J* = 5.9 Hz, 2H), 3.98 (s, 3H).

^{13}C NMR (151 MHz, $CDCl_3$, 23 °C, δ): 165.9, 157.8, 143.4, 143.4, 133.4, 132.8, 130.0, 129.6, 127.9, 125.9 (q, *J* = 3.9 Hz), 124.5 (d, *J* = 272.1 Hz).121.8, 111.7, 56.3, 43.6.

^{19}F NMR (282 MHz, $CDCl_3$, 23 °C, δ): –62.5.

HRMS-GC-EI(m/z) calc'd for $C_{16}H_{14}NO_2F_3$ $[M]^+$, 309.0971; found, 309.0975; deviation: –1.1 ppm.

N-Benzyl-2-methoxybenzamide-derived thianthrenium salt 40a

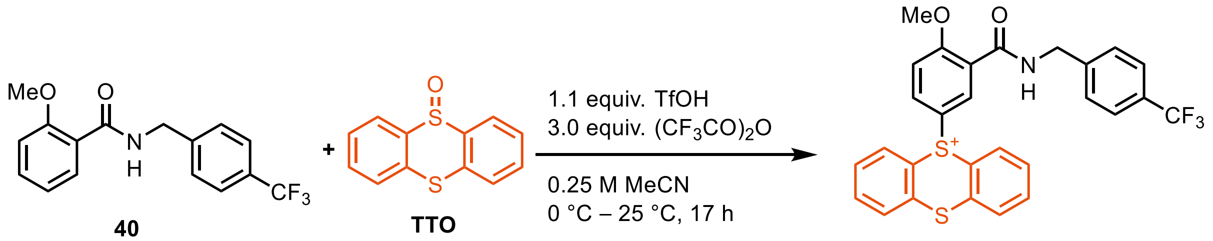

40 1.00 equiv. | **TTO** 1.00 equiv. | **40a,** 43%

To a solution of benzamide derivative **40** (154 mg, 0.500 mmol, 1.00 equiv.) and TTO (thianthrene-*S*-oxide, 116 mg, 0.500 mmol, 1.00 equiv.) in MeCN (2.0 mL, *c* = 0.25 M), trifluoroacetic anhydride (0.21 mL, 0.32 g, 1.5 mmol, 3.0 equiv.) was added at 0 °C, under an ambient atmosphere followed by addition of TfOH (49 µl, 83 mg, 0.55 mmol, 1.1 equiv.). The reaction mixture was stirred at 0 °C for 1 h, and then stirred (400 rpm) at 25 °C for 16 h. The solvent was removed under reduced pressure by rotary evaporation and the residue was dissolved in DCM (10 mL). The resulting solution was washed with saturated aqueous $NaHCO_3$ solution (10 mL) and aqueous $NaBF_4$ solution (2 × 10 mL, 10% w/w). The DCM layer was dried over Na_2SO_4, filtered, and the solvent was removed under reduced pressure. The crude mixture was dissolved in DCM (5 mL) and Et_2O (50 mL) was added to obtain the product as white precipitate. The precipitate was filtered to give 130 mg of the title compound **40a** as colorless solid (43% yield).

NMR Spectroscopy:

^{1}H NMR (600 MHz, CD_3CN, 23 °C, δ): 8.44 – 8.37 (m, 1H), 8.34 (ddd, *J* = 8.0, 1.4, 0.4 Hz, 2H), 7.94 (ddd, *J* = 7.9, 1.3, 0.4 Hz, 2H), 7.87 (ddd, *J* = 7.9, 7.4, 1.4 Hz, 2H), 7.82 – 7.74 (m, 3H), 7.62 – 7.56 (m, 2H), 7.46 (ddt, *J* = 8.1, 1.6, 0.8 Hz, 2H), 7.29 (dd, *J* = 9.1, 2.9 Hz, 1H), 7.22 (d, *J* = 9.1

Hz, 1H), 4.56 (dd, J = 6.1, 1.0 Hz, 2H), 3.97 (s, 3H).

^{13}C NMR (151 MHz, CD_3CN, 23 °C, δ): 163.7, 162.3, 145.1, 137.3, 136.1, 135.7, 133.8, 132.7, 131.6, 131.0, 129.4 (q, J = 31.9 Hz), 128.9, 126.2 (q, J = 3.8 Hz), 125.5 (q, J = 271.2 Hz), 119.9, 115.7, 115.5, 57.9, 43.9.

^{19}F NMR (565 MHz, $CDCl_3$, 23 °C, δ): –62.9 (3F), {–151.5 (bs), –151.6(bs), 4F}.

HRMS-ESI(m/z) calc'd for $C_{28}H_{21}NO_2F_3S_2$ $[M–BF_4]^+$, 524.0960; found, 524.0962; deviation: –0.4 ppm.

Tianeptine intermediate-derived thianthrenium salt 41a

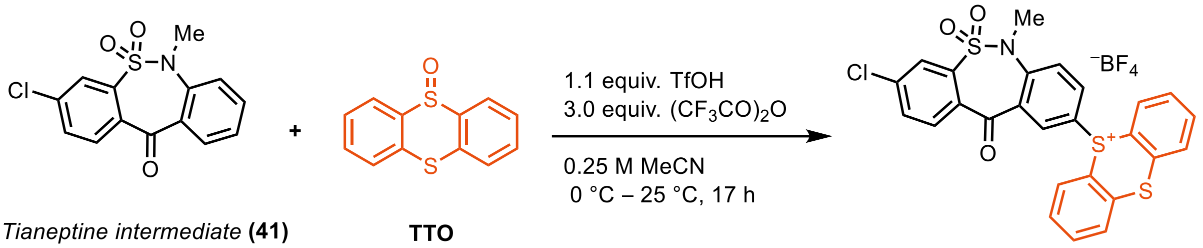

Tianeptine intermediate **(41)** **TTO**

1.00 equiv. 1.00 equiv. **41a**, 56%

To a solution of tianeptine intermediate **41** (307 mg, 1.00 mmol, 1.00 equiv.) and TTO (thianthrene-*S*-oxide, 232 mg, 1.00 mmol, 1.00 equiv.) in MeCN (4.0 mL, c = 0.25 M), trifluoroacetic anhydride (0.42 mL, 0.63 g, 3.0 mmol, 3.0 equiv.) was added at 0 °C, under an ambient atmosphere followed by addition of TfOH (97 µl, 0.17 g, 1.1 mmol, 1.1 equiv.). The reaction mixture was stirred (400 rpm) at 0 °C for 1 h, and then stirred at 25 °C for 16 h. The solvent was removed under reduced pressure by rotary evaporation and the residue was dissolved in DCM (10 mL). The resulting solution was washed with saturated aqueous $NaHCO_3$ solution (10 mL) and aqueous $NaBF_4$ solution (2 × 10 mL, 10% w/w).Purification by flash silica gel column chromatography (DCM / MeOH = 20:1 (v/v)) afforded 340 mg of the title compound **41a** as a colorless solid (56% yield).

R_f = 0.30 (DCM / MeOH = 15:1 (v/v)).

NMR Spectroscopy:

^{1}H NMR (600 MHz, CD_3CN, 23 °C, δ): 8.44 (ddd, J = 7.9, 1.4, 0.5 Hz, 2H), 8.00 (ddd, J = 7.9, 1.4, 0.5 Hz, 2H), 7.93 (ddd, J = 8.0, 7.5, 1.5 Hz, 2H), 7.91 (dd, J = 2.1, 0.5 Hz, 1H), 7.89 – 7.84 (m, 3H), 7.79 (dd, J = 8.3, 2.0 Hz, 1H), 7.74 (dd, J = 8.4, 0.5 Hz, 1H), 7.51 (d, J = 8.6 Hz, 1H), 7.38 (dd, J = 9.0, 2.7 Hz, 1H), 3.36 (s, 3H).

^{13}C NMR (151 MHz, CD_3CN, 23 °C, δ): 189.6, 146.0, 139.7, 138.7, 137.7, 136.4, 136.3, 135.2, 134.7, 134.3, 133.9, 132.3, 131.9, 131.6, 131.1, 126.6, 125.8, 121.2, 119.2, 39.1.

^{19}F NMR (565 MHz, $CDCl_3$, 23 °C, δ): –151.6 (bs), –151.6 (bs).

HRMS-ESI(m/z) calc'd for $C_{26}H_{17}NClO_3S_3$ $[M–BF_4]^+$, 522.0054; found, 522.0052; deviation: +0.4 ppm.

Fenofibrate-derived thianthrenium salt 42a

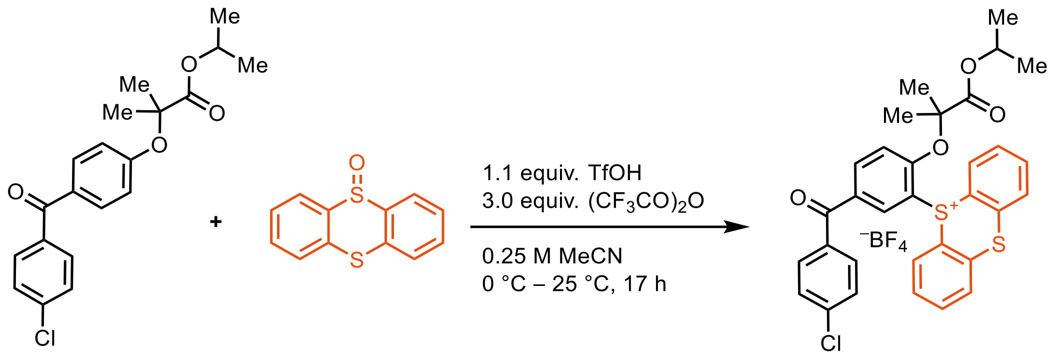

Fenofibrate **(42)** 1.00 equiv. **TTO** 1.00 equiv. **42a,** 60%

To a solution of fenofibrate (**42**) (180 mg, 0.500 mmol, 1.00 equiv.) and TTO (thianthrene-*S*-oxide, 116 mg, 0.500 mmol, 1.00 equiv.) in MeCN (2.0 mL, *c* = 0.25 M), trifluoroacetic anhydride (0.21 mL, 0.32 g, 1.5 mmol, 3.0 equiv.) was added at 0 °C, under an ambient atmosphere followed by addition of TfOH (49 µl, 83 mg, 0.55 mmol, 1.1 equiv.). The reaction mixture was stirred (400 rpm) at 0 °C for 1 h, and then stirred at 25 °C for 16 h. The solvent was removed under reduced pressure by rotary evaporation and the residue was dissolved in DCM (10 mL). The resulting solution was washed with saturated aqueous $NaHCO_3$ solution (10 mL) and aqueous $NaBF_4$ solution (2 × 10 mL, 10% w/w). The residue was dissolved in DCM (5 mL) and MTBE (50 mL) was added to obtain the product as white precipitate. The precipitate was filtered to afford 197 mg of the title compound **42a** as a colorless solid (60% yield).

R*f* = 0.34 (DCM / MeOH = 15:1 (v/v)).

NMR Spectroscopy:

^{1}H NMR (600 MHz, CD_3CN, 23 °C, δ): 8.27 (dd, *J* = 8.0, 1.3 Hz, 2H), 7.99 (dd, *J* = 8.8, 2.1 Hz, 1H), 7.94 (dd, *J* = 7.8, 1.4 Hz, 2H), 7.83 (td, *J* = 7.7, 1.5 Hz, 2H), 7.78 (td, *J* = 7.7, 1.4 Hz, 2H), 7.56 – 7.47 (m, 4H), 6.99 (d, *J* = 8.7 Hz, 1H), 6.95 (d, *J* = 2.1 Hz, 1H), 4.99 – 4.83 (m, 1H), 1.74 (s, 6H), 1.03 (d, *J* = 6.3 Hz, 6H).

^{13}C NMR (151 MHz, CD_3CN, 23 °C, δ): 192.7, 171.8, 158.71, 139.4, 138.2, 137.6, 136.3, 136.2, 135.9, 133.3, 131.9, 131.6, 131.1, 130.9, 129.7, 117.9, 110.6, 84.4, 71.1, 27.3, 25.6, 21.6.

^{19}F NMR (471 MHz, $CDCl_3$, 23 °C, δ): –151.7 (bs), –151.7 (bs).

HRMS-ESI(m/z) calc'd for $C_{32}H_{28}ClO_4S_2$ $[M–BF_4]^+$, 575.1112; found, 575.1112; deviation: –0.1 ppm.

(±)-Penthiopyrad derived thianthrenium salt 43a

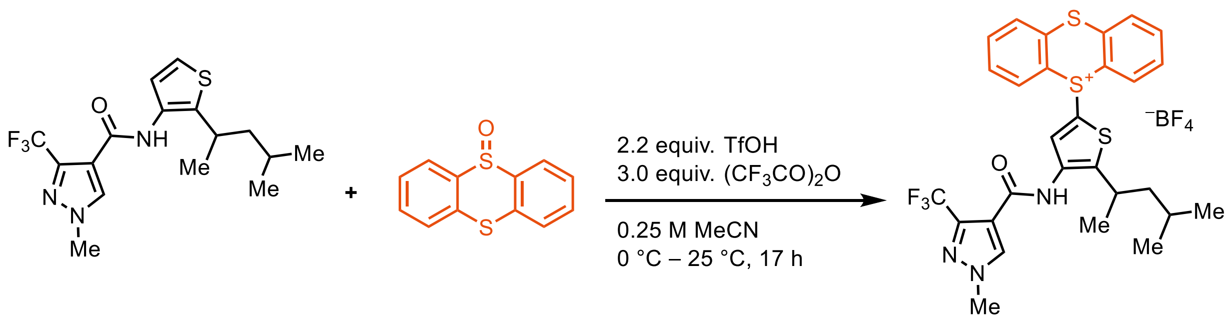

(±)-Penthiopyrad **(43)** 1.00 equiv. **TTO** 1.00 equiv. **(±)-43a,** 72%

To a solution of (±)-penthiopyrad (**43**) (89.9 mg, 0.250 mmol, 1.00 equiv.) and TTO (thianthrene-*S*-oxide, 58.1 mg, 0.250 mmol, 1.00 equiv.) in MeCN (1.0 mL, *c* = 0.25 M), TfOH (25 µl, 45 mg, 0.27 mmol, 1.1 equiv.), and trifluoroacetic anhydride (104 µL, 157 mg, 0.750 mmol, 3.00 equiv.) were added sequentially at 0 °C, under an ambient atmosphere followed by addition of TfOH (25 µl, 45 mg, 0.27 mmol, 1.1 equiv.). The reaction mixture was stirred (400 rpm) at 0 °C for 1 h, and then stirred at 25 °C for 16 h. The solvent was removed under reduced pressure by rotary evaporation and the residue was dissolved in DCM (10 mL). The resulting solution was washed with saturated aqueous $NaHCO_3$ solution (10 mL) and aqueous $NaBF_4$ solution (2 × 10 mL, 10% w/w). Purification by flash silica gel column chromatography (DCM to DCM / MeOH =20:1 (v/v)) afforded 120 mg of the title compound **(±)-43a** as a colorless solid (72% yield).

R*f* = 0.24 (DCM / MeOH = 15:1 (v/v)).

NMR Spectroscopy:

^{1}H NMR (600 MHz, CD_3CN, 23 °C, δ): 8.28 (dddd, *J* = 8.0, 5.1, 1.4, 0.4 Hz, 2H), 8.25 (s, 1H), 8.09 (s, 1H), 7.98 (ddd, *J* = 8.0, 2.5, 1.3 Hz, 2H), 7.91 (s, 1H), 7.86 (dddd, *J* = 8.0, 7.5, 3.0, 1.3 Hz, 2H), 7.75 (dddd, *J* = 8.0, 7.5, 2.8, 1.3 Hz, 2H), 3.94 (s, 3H), 3.36 – 3.18 (m, 1H), 1.45 – 1.30 (m, 3H), 1.14 (d, *J* = 6.7 Hz, 3H), 0.77 (t, *J* = 6.2 Hz, 6H).

^{13}C NMR (151 MHz, CD_3CN, 23 °C, δ): 160.4, 155.4, 140.9 (d, *J* = 37.8 Hz), 137.2, 136.8, 136.1, 135.0, 134.7, 132.7, 131.4, 131.1, 122.9, 121.1 (d, *J* = 9.2 Hz), 117.4, 116.3, 48.7, 40.4, 32.1, 26.4, 22.9, 22.7, 22.5.

^{19}F NMR (471 MHz, $CDCl_3$, 23 °C, δ): –61.2 (s, 3F), {–150.1 (bs), –150.2 (bs), 4F}.

HRMS-ESI(m/z) calc'd for $C_{28}H_{27}ON_3F_3S_2$ [M–BF_4]$^+$, 574.1263; found, 574.1264; deviation: –0.3 ppm.

Pyraclostrobin derived thianthrenium salt 44a

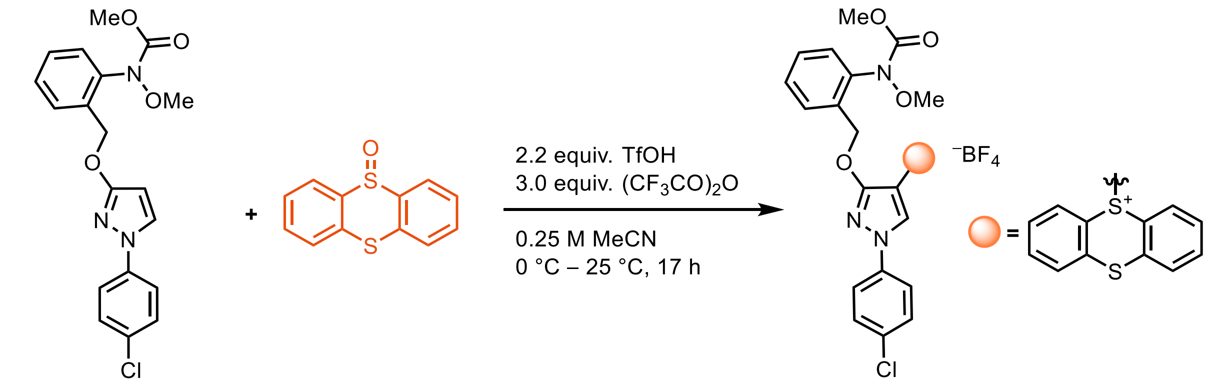

Pyraclostrobin (**44**) 1.00 equiv. **TTO** 1.00 equiv. **44a,** 80%

To a solution of pyraclostrobin (**44**) (96.9 mg, 0.250 mmol, 1.00 equiv.) and TTO (thianthrene-*S*-oxide, 58.1 mg, 0.250 mmol, 1.00 equiv.) in MeCN (1.0 mL, *c* = 0.25 M), TfOH (25 µl, 45 mg, 0.27 mmol, 1.1 equiv.), and trifluoroacetic anhydride (104 µL, 157 mg, 0.750 mmol, 3.00 equiv.) were added sequentially at 0 °C, under an ambient atmosphere followed by addition of TfOH (25 µl, 45 mg, 0.27 mmol, 1.1 equiv.). The reaction mixture was stirred (400 rpm) at 0 °C for 1 h, and then stirred at 25 °C for 16 h. The solvent was removed under reduced pressure by rotary evaporation and the residue was dissolved in DCM (10 mL). The resulting solution was washed with saturated aqueous $NaHCO_3$ solution (10 mL) and aqueous $NaBF_4$ solution (2 × 10 mL, 10% w/w).Purification by flash silica gel column chromatography (DCM to DCM / MeOH = 10:1) afforded 138 mg of the title compound **44a** as a colorless solid (80% yield).

R_f = 0.30 (DCM / MeOH = 15:1 (v/v)).

NMR Spectroscopy:

^{1}H NMR (600 MHz, $CDCl_3$, 23 °C, δ): 8.98 (s, 1H), 7.98 (dd, *J* = 8.1, 1.3 Hz, 2H), 7.74 – 7.67 (m, 4H), 7.64 (ddd, *J* = 8.0, 7.4, 1.3 Hz, 2H), 7.55 (ddd, *J* = 8.1, 7.4, 1.3 Hz, 2H), 7.41 – 7.30 (m, 4H), 7.22 (ddd, *J* = 7.7, 7.2, 1.5 Hz, 1H), 6.91 (dd, *J* = 8.0, 1.3 Hz, 1H), 5.28 (s, 2H), 3.75 (s, 3H), 3.58 (s, 3H).

^{13}C NMR (151 MHz, CD_3CN, 23 °C, δ): 160.7, 155.8, 137.4, 137.1, 136.0, 134.9, 133.8, 133.7, 132.9, 131.4, 130.0, 129.9, 129.9, 129.2, 128.8, 128.6, 127.1, 120.8, 120.6, 85.0, 68.0, 62.2, 53.8.

^{19}F NMR (565 MHz, $CDCl_3$, 23 °C, δ): –151.5 (bs), –151.5 (bs).

HRMS-ESI(m/z) calc'd for $C_{31}H_{25}N_3ClO_4S_2$ $[M–BF_4]^+$, 602.0970; found, 602.0974; deviation: –0.8 ppm.

Boscalid-derived thianthrenium salt 45a

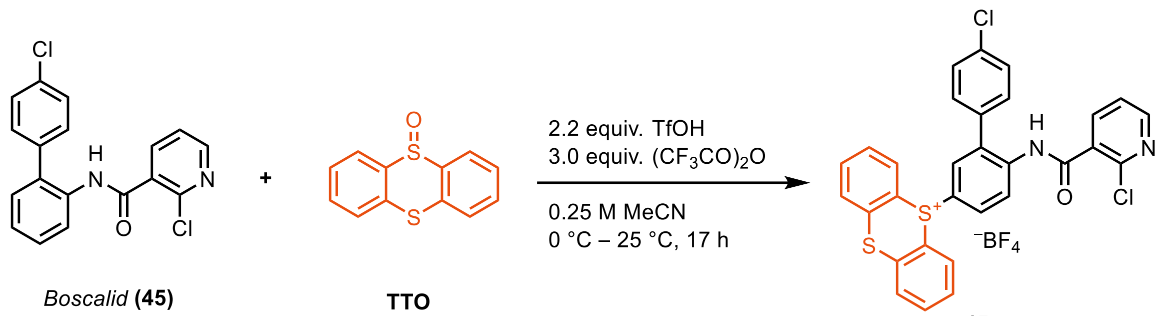

Boscalid (**45**)
1.00 equiv.

TTO
1.00 equiv.

45a, 91%

To a solution of boscalid (**45**) (2059 mg, 6.000 mmol, 1.000 equiv.) and TTO (thianthrene-*S*-oxide, 1393 mg, 6.000 mmol, 1.000 equiv.) in MeCN (24 mL, *c* = 0.25 M), TfOH (0.6 mL, 1.0 g, 6.6 mmol, 1.1 equiv.), and trifluoroacetic anhydride (2.5 mL, 3.8 g, 18 mmol, 3.0 equiv.) were added sequentially at 0 °C, under an ambient atmosphere followed by addition of TfOH (0.6 mL, 1.0 g, 6.6 mmol, 1.1 equiv.). The reaction mixture was stirred (400 rpm) at 0 °C for 1 h, and then stirred at 25 °C for 16 h. The solvent was removed under reduced pressure by rotary evaporation and the residue was dissolved in DCM (10 mL). The resulting solution was washed with saturated aqueous $NaHCO_3$ solution (10 mL) and aqueous $NaBF_4$ solution (2 × 10 mL, 10% w/w).Purification by flash silica gel column chromatography (DCM / MeOH = 30:1 to 10:1 (v/v)) afforded 3572 mg of the title compound **45a** as colorless solid (91% yield).

R*f* = 0.23 (DCM / MeOH = 15:1 (v/v)).

NMR Spectroscopy:

^{1}H NMR (500 MHz, CD_3CN, 23 °C, δ): 8.45 (s, 1H), 8.40 (dd, *J* = 4.8, 1.9 Hz, 1H), 8.36 (ddd, *J* = 7.9, 1.4, 0.4 Hz, 2H), 8.26 (d, *J* = 9.0 Hz, 1H), 7.98 (ddd, *J* = 8.0, 1.4, 0.4 Hz, 2H), 7.88 (ddd, *J* = 8.0, 7.5, 1.4 Hz, 1H), 7.84 – 7.77 (m, 6H), 7.45 – 7.42 (m, 2H), 7.37 (dd, *J* = 7.7, 4.8 Hz, 1H), 7.29 – 7.24 (m, 2H), 7.18 (dd, *J* = 9.0, 2.6 Hz, 1H), 7.07 (d, *J* = 2.6 Hz, 1H).

^{13}C NMR (126 MHz, CD_3CN, 23 °C, δ): 165.2, 152.1, 147.6, 140.1, 139.1, 137.6, 136.4, 136.2, 136.0, 135.5, 135.5, 132.8, 132.0, 131.8, 131.1, 131.0, 130.1, 129.4, 125.8, 123.9, 120.2, 119.7.

^{19}F NMR (565 MHz, CD_3CN, 23 °C, δ): –151.6 (bs), –151.7 (bs).

HRMS-ESI(m/z) calc'd for $C_{30}H_{19}ON_2S_2Cl_2$ $[M–BF_4]^+$, 557.0310; found, 557.0312; deviation: –0.3 ppm.

Amiodarone-derived thianthrenium salt 46a

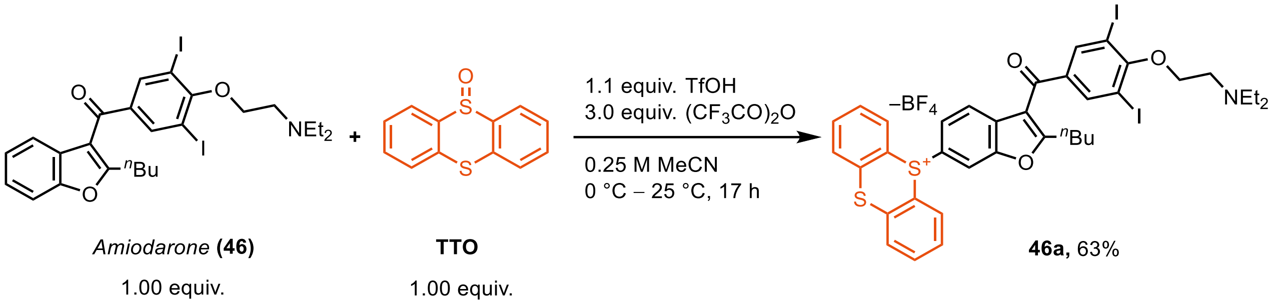

Amiodarone hydrochloride **46·HCl** (341 mg, 0.500 mmol, 1.00 equiv.) was dissolved in EtOAc (5 mL) and washed with saturated aqueous Na_2CO_3 solution (5 ml). The organic layer was dried over Na_2SO_4, filtered, and concentrated under reduced pressure by rotary evaporation. The residue was dissolved in MeCN (2.0 mL, *c* = 0.25 M) and added to a 20-mL glass vial containing TTO (thianthrene-*S*-oxide, 116 mg, 0.500 mmol, 1.00 equiv.). The reaction mixture was cooled to 0 °C with an ice bath, TfOH (49 µl, 83 mg, 0.55 mmol, 1.1 equiv.) and trifluoroacetic anhydride (0.21 mL, 0.32 g, 1.5 mmol, 3.0 equiv.) were added at 0 °C, under an ambient atmosphere followed by addition of TfOH (49 µl, 83 mg, 0.55 mmol, 1.1 equiv.). The reaction mixture was stirred (400 rpm) at 0 °C for 1 h, and then stirred at 25 °C for 16 h. The solvent was removed under reduced pressure by rotary evaporation and the residue was dissolved in DCM (10 mL). The resulting solution was washed with saturated aqueous $NaHCO_3$ solution (10 mL) and aqueous $NaBF_4$ solution (2 × 10 mL, 10% w/w). The residue was dissolved in DCM (5 mL) and MTBE (50 mL) was added to obtain the product as white precipitate. The precipitate was filtered to afford 197 mg of the title compound **46a** as colorless solid (63% yield).

NMR Spectroscopy:

^{1}H NMR (600 MHz, CD_3CN, 23 °C, δ): 8.40 (dd, *J* = 8.1, 1.3 Hz, 2H), 8.16 (s, 2H), 8.00 – 7.95 (m, 2H), 7.91 (td, *J* = 7.7, 1.4 Hz, 2H), 7.86 – 7.81 (m, 2H), 7.62 (d, *J* = 8.1 Hz, 2H), 7.36 (d, *J* = 2.0 Hz, 1H), 7.06 (dd, *J* = 8.6, 2.0 Hz, 1H), 4.41 – 4.29 (m, 2H), 3.72 (t, *J* = 5.0 Hz, 2H), 3.54 – 3.39 (m, 4H), 2.79 – 2.59 (m, 2H), 1.73 – 1.59 (m, 2H), 1.38 (t, *J* = 7.3 Hz, 6H), 1.33 – 1.20 (m, 2H), 0.83 (t, *J* = 7.4 Hz, 3H).

^{13}C NMR (151 MHz, CD_3CN, 23 °C, δ): 187.8, 170.6, 161.2, 154.1, 141.5, 139.7, 137.6, 136.3, 136.0, 132.4, 131.8, 131.1, 124.4, 124.1, 120.5, 119.9, 116.9, 112.8, 91.5, 67.4, 52.6, 49.7, 30.3, 29.0, 23.1, 13.9, 9.3.

^{19}F NMR (471 MHz, $CDCl_3$, 23 °C, δ): –151.5 (bs), –151.5 (bs).

HRMS-ESI(m/z) calc'd for $C_{37}H_{36}I_2NO_3S_2$ $[M–BF_4]^+$, 860.0221; found, 860.0220; deviation: +0.0 ppm.

Amiodarone-derived thianthrenium salt 47a

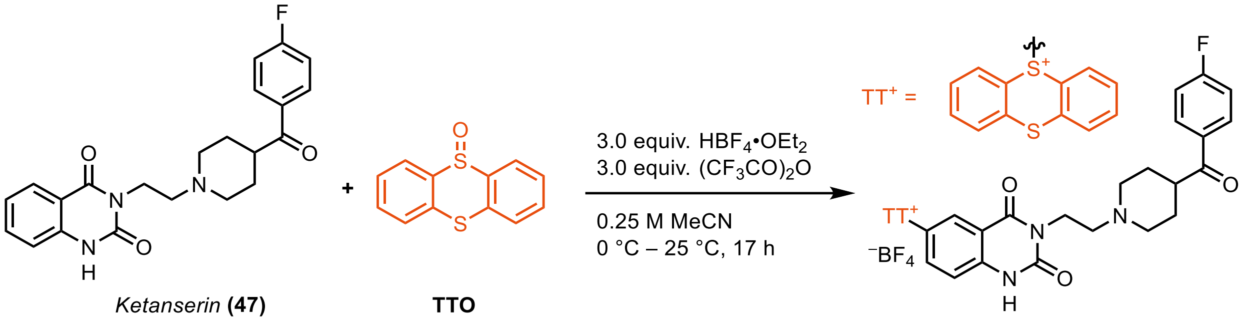

Ketanserin **(47)** 1.00 equiv. **TTO** 1.00 equiv. **47a**, 69%

To a solution of ketanserin (**47**) (326 mg, 0.824 mmol, 1.00 equiv.) and TTO (thianthrene-*S*-oxide, 192 mg, 0.824 mmol, 1.00 equiv.) in MeCN (2.0 mL, *c* = 0.25 M), $HBF_4{\cdot}OEt_2$ (0.34 mL, 0.40 g, 2.5 mmol, 3.0 equiv.) was added at 0 °C, under an ambient atmosphere followed by addition of trifluoroacetic anhydride (0.34 mL, 0.52 g, 2.5 mmol, 3.0 equiv.). The reaction mixture was stirred (400 rpm) at 0 °C for 1 h, and then stirred at 25 °C for 16 h. The solvent was removed under reduced pressure by rotary evaporation and the residue was dissolved in DCM (10 mL). The resulting solution was washed with saturated aqueous $NaHCO_3$ solution (10 mL) and aqueous $NaBF_4$ solution (2 × 10 mL, 10% w/w). The residue was dissolved in DCM (5 mL) and diethylether (50 mL) was added to obtain the product as white precipitate. The precipitate was filtered to afford 636 mg of the title compound **47a** as a colorless solid (69% yield).

NMR Spectroscopy:

^{1}H NMR (300 MHz, CD_3CN, 23 °C, δ): 8.41 (dd, *J* = 7.7, 1.6 Hz, 2H), 8.05 – 7.80 (m, 8H), 7.69 (d, *J* = 2.3 Hz, 1H), 7.28 (dq, *J* = 14.8, 8.8 Hz, 4H), 4.16 (t, *J* = 6.0 Hz, 2H), 3.61 – 3.30 (m, 3H), 3.07 (t, *J* = 6.0 Hz, 2H), 2.81 (t, *J* = 11.9 Hz, 2H), 2.06 – 1.89 (m, 2H), 1.83 – 1.62 (m, 2H).

^{13}C NMR (75 MHz, CD_3CN, 23 °C, δ): 201.0, 166.7 (d, *J* = 252.6 Hz), 150.6, 142.8, 136.8, 135.7, 135.4, 134.1, 132.6, 132.5, 131.6 (d, *J* = 9.4 Hz), 131.1, 130.4, 128.9, 118.9, 118.0, 116.1 (d, *J* = 22.1 Hz), 56.2, 53.6, 41.7, 37.7, 27.7

^{19}F NMR (282 MHz, $CDCl_3$, 23 °C, δ): –107.3 (s, 1F), {–151.7 (bs), –151.7 (bs), 4F}.

HRMS-ESI(m/z) calc'd for $C_{34}H_{29}FNO_3S_2$ $[M–BF_4]^+$, 610.1629; found, 616.1634; deviation: –0.8 ppm.

2-Benzoyl thiophene-derived thianthrenium salt L-10

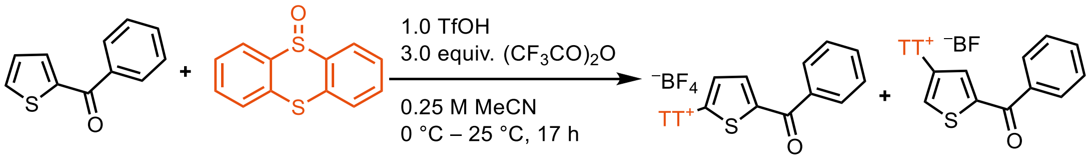

L-10 1.00 equiv. **TTO** 1.00 equiv. **L-10a**, 40% **L-10b**, 20%

To a solution of fenofibrate **L-10** (94.1 mg, 0.500 mmol, 1.00 equiv.) and TTO (thianthrene-*S*-oxide, 116 mg, 0.500 mmol, 1.00 equiv.) in MeCN (2.0 mL, *c* = 0.25 M), trifluoroacetic anhydride (0.21 mL,

0.32 g, 1.5 mmol, 3.0 equiv.) was added at 0 °C, under an ambient atmosphere followed by addition of TfOH (49 µl, 83 mg, 0.55 mmol, 1.1 equiv.). The reaction mixture was stirred (400 rpm) at 0 °C for 1 h, and then stirred at 25 °C for 16 h. The solvent was removed under reduced pressure by rotary evaporation and the residue was dissolved in DCM (10 mL). The resulting solution was washed with saturated aqueous $NaHCO_3$ solution (10 mL) and aqueous $NaBF_4$ solution (2 × 10 mL, 10% w/w). The residue was purified by flash column chromatography (DCM / MeOH = 20:1 (v/v)) to afford 145 mg of white solid (60% yield). Analysis of the reaction mixture by NMR spectroscopy showed the formation of two isomers **L-10a** and **L-10b** in 2:1 ratio.

Isochroman-derived thianthrenium salt L-11

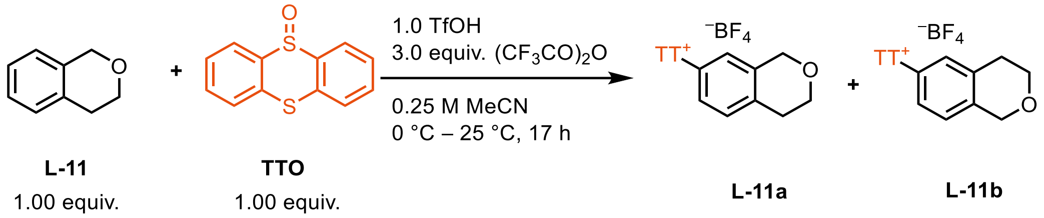

To a solution of fenofibrate **L-11** (67.1 mg, 0.500 mmol, 1.00 equiv.) and TTO (thianthrene-*S*-oxide, 116 mg, 0.500 mmol, 1.00 equiv.) in MeCN (2.0 mL, c = 0.25 M), trifluoroacetic anhydride (0.21 mL, 0.32 g, 1.5 mmol, 3.0 equiv.) was added at 0 °C, under an ambient atmosphere followed by addition of TfOH (49 µl, 83 mg, 0.55 mmol, 1.1 equiv.). The reaction mixture was stirred (400 rpm) at 0 °C for 1 h, and then stirred at 25 °C for 16 h. The solvent was removed under reduced pressure by rotary evaporation and the residue was dissolved in DCM (10 mL). The resulting solution was washed with saturated aqueous $NaHCO_3$ solution (10 mL) and aqueous $NaBF_4$ solution (2 × 10 mL, 10% w/w). The residue was purified by flash column chromatography (DCM / MeOH = 20:1 (v/v)) to afford 104 mg of white solid. Analysis of the reaction mixture showed the formation of two isomers **L-11a** and **L-11b** in 10:6 ratio together with unknown impurities.

Calculation of Hammett values for substrates

Class I substrates

σp: –0.457

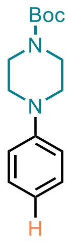

σp: –0.476

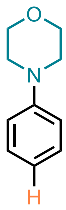

σp: –0.331
σp: –0.331
σ = –0.662

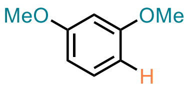

σp: –0.358

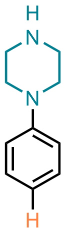

σp: –0.631

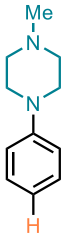

σp: –0.724

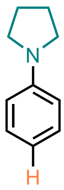

σp: –0.331
σp: –0.331
σp: –0.331
σ = –0.993

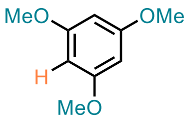

σp: –0.378

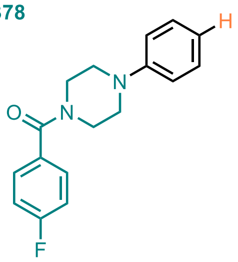

Class II substrates

σp: –0.122

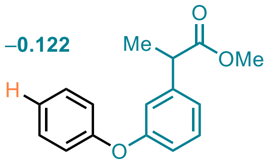

σp: –0.331
σm: +0.029
σ = –0.302

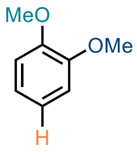

σp: –0.331
σm: +0.135
σ = –0.196

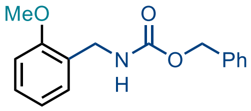

σp: –0.304

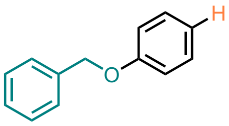

σp: –0.331
σm: +0.005
σ = –0.226

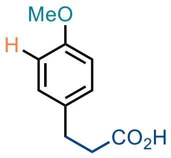

σp: –0.193

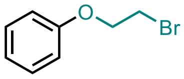

σp: –0.122

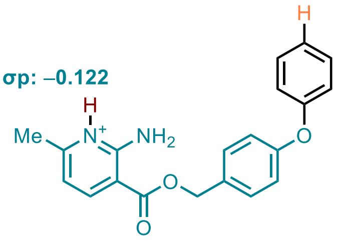

σp: –0.06

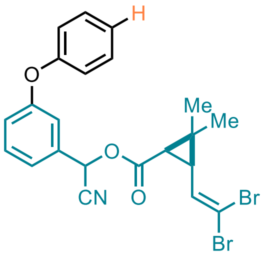

σp: –0.179

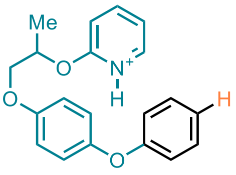

σp: –0.293
σm: –0.030
σ = –0.323

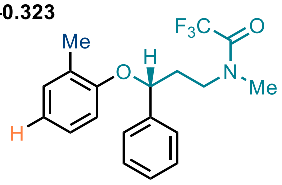

σm: 0.012
σp: -0.332
σ = -0.320

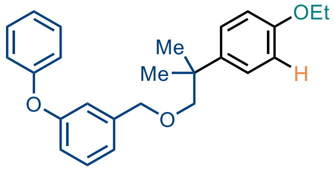

Class III

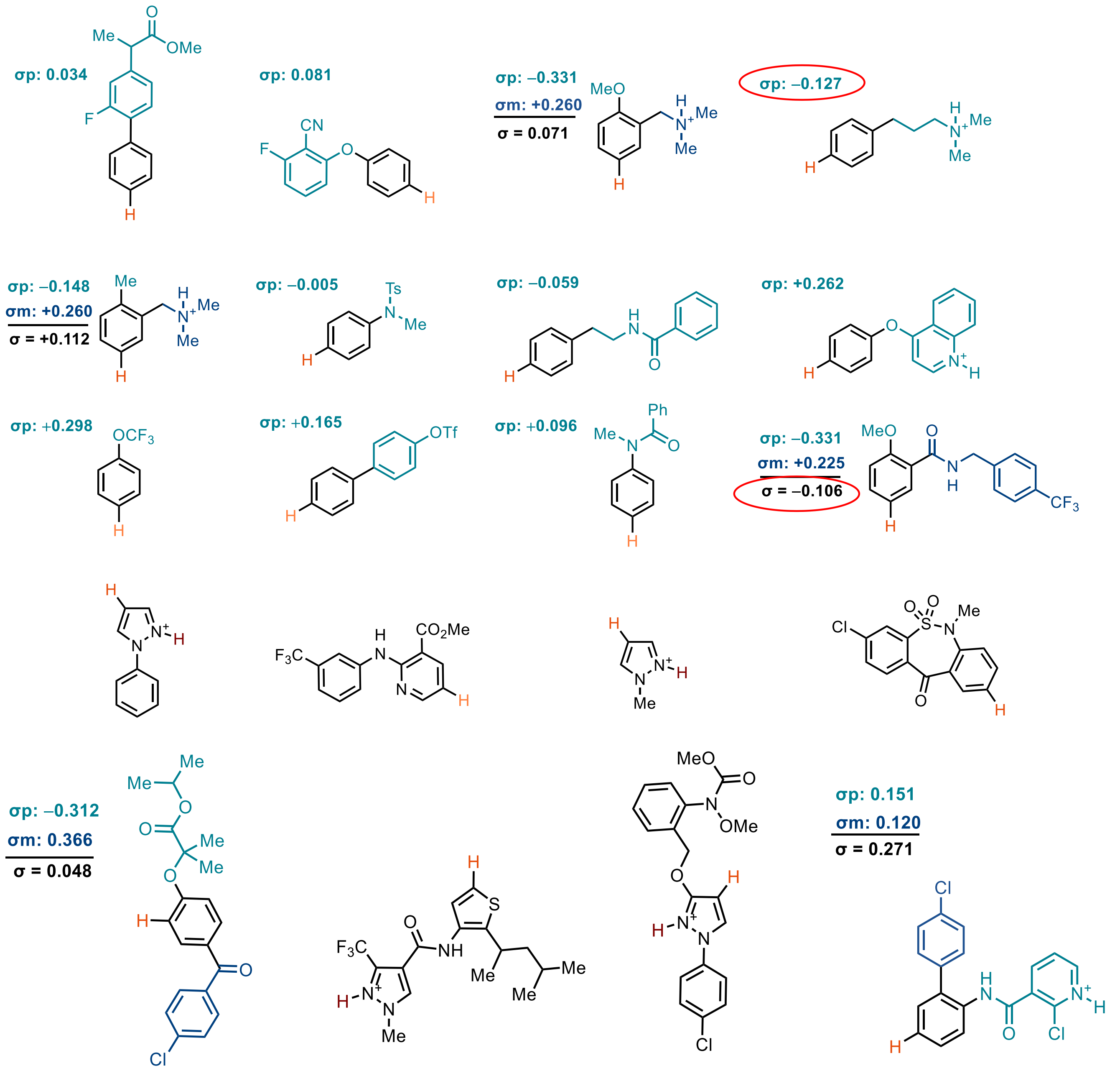

Class IV

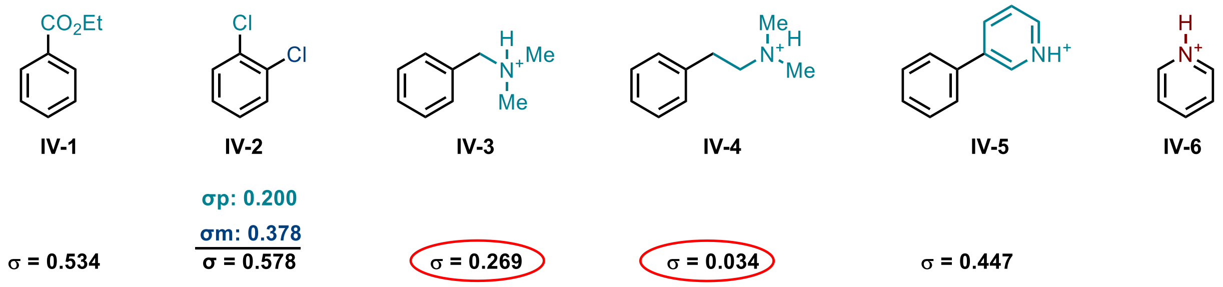

Fig S4: Calculation of the Hammett parameters for complex molecules.[22] Substituents were drawn in their protonated forms. The σ values circled in red are exceptions where Hammett sigma constant fail to assign the substrate correctly. For *ortho* substituents σ_p values were used.

Comparison of different reaction conditions for different classes of arenes

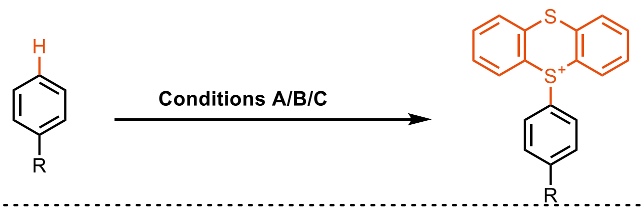

A

1.0 equiv. TTO
3.0 equiv. $(CF_3CO)_2O$
2.0 equiv DIPEA
MeCN, 0 – 25 °C

B

1.0 equiv. TTO
3.0 equiv. $(CF_3CO)_2O$
1.1 equiv HBF_4·Et_2O per basic group
MeCN, 0 – 25 °C

C

1.0 equiv. TTO
3.0 equiv. $(CF_3CO)_2O$
1.1 equiv. TfOH
1.1 equiv. TfOH per basic group
MeCN, 0 – 25 °C

Class I

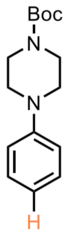

✓ A: 75%
X B: 37%
X C: 0%

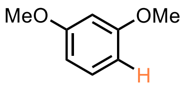

✓ A: 88%
✓ B: 99%
✓ C: 100%

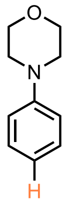

✓ A: 71%
✓ B: 54%
✓ C: 55%

Class II

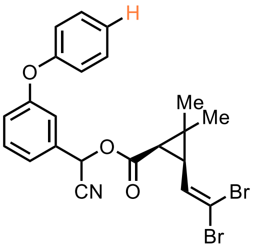

X A: 0%
✓ B: 79%
✓ C: 60%

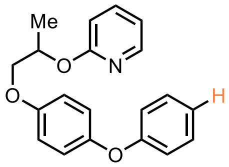

X A: 0%
✓ B: 83%
✓ C: 66%

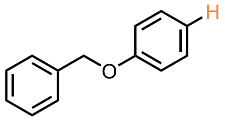

X A: 5%
✓ B: 82%
✓ C: 90%

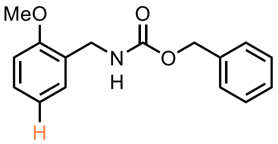

X A: 35%
✓ B: 57%
X C: 0%[a]

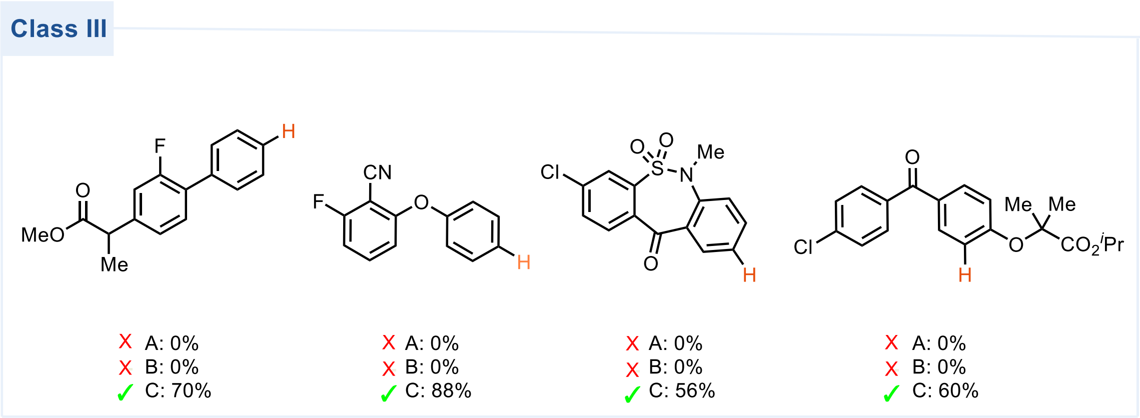

Fig S5: Comparison of reaction conditions for different classes of arenes. Yields were determined by ^1H NMR using CH_2Br_2 as an internal standard.a ^1H NMR analysis of the crude reaction mixture showed a mixture of products. The signal corresponding to the product was not detected in LCMS.

Troubleshooting: A Case study for thianthrenation

Deactivation of arene due to the protonation of the basic group in close proximity

Protonation of a basic functionality in close proximity to the arene makes the arene too electron deficient. Screening of different acids show that, TMSOTf gives the best result.

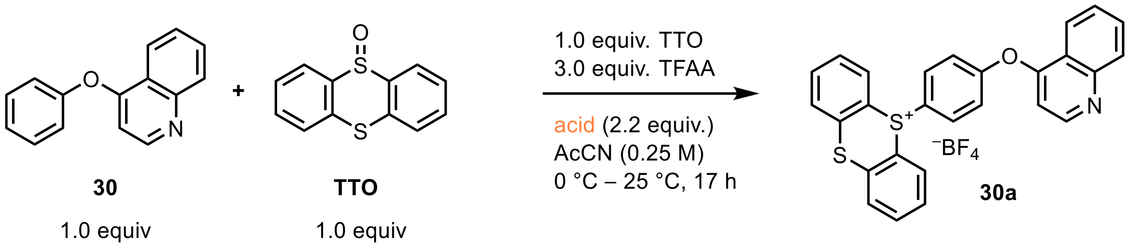

acid	Yield (%)
TfOH	0
HBF_4OEt_2	0
**TMSOTf**	**60**

Fig S6 : Yields were determined by ^1H NMR using CH_2Br_2 as an internal standard.

Effect of the water content of the solvent on thianthrenation

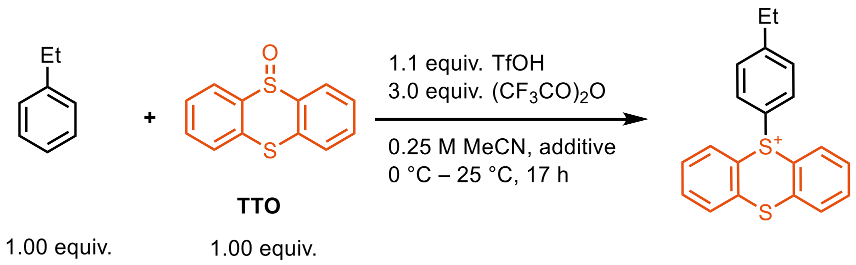

Table S1 Effect of water on thianthrenation:

Entry	Water content of the solvent	Yield (%)
**1**	23 ppm	98
**2**	54 ppm	98
**3**	0.5 equiv. water added	94
**4**	1.5 equiv. water added	91

Reactions were carried out on a 0.25 M scale. Yields were determined by ^{1}H NMR in $CDCl_3$ using CH_2Br_2 (1.0 equiv.) as an internal standard.

Follow-on transformations of aryl thianthrenium salts

The reaction vials were introduced to a nitrogen-filled glovebox to facilitate convenient exchange of the atmosphere from oxygen to nitrogen. However, the reactions do not require glovebox. Additional procedures by using Schlenk line are provided for substrates **52** (C–N cross-coupling), **68** (carboamidation), **83** (difluoromethylation), **87** (cyanation), **96** (Suzuki coupling), **100** (sulfonylation) and **105** (nickel-catalyzed bromination).

C–N cross-coupling reactions of aryl thianthrenium salts

Table S2 Optimization of reaction conditions for C–N couplings of aryl thianthrenium salts:

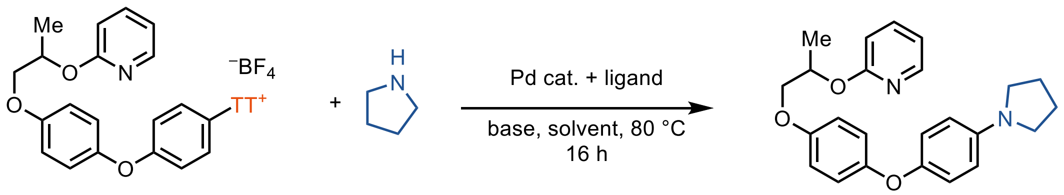

Entry	Pd cat. + ligand	base	solvent	Yield (%)
**1**	Pd_2dba_3	Cs_2CO_3	DMF	5
**2**	$PdCl_2$amphos	Cs_2CO_3	1,4-dioxane	36
**3**	$PdCl_2$amphos	K_3PO_4	1,4-dioxane	25
**4**	PddppfCl$_2$	K_3PO_4	1,4-dioxane	15
**5**	Pd_2dba_3+ Xphos	NaOtBu	THF	22
**6**	Pd_2dba_3 + Sphos	NaOtBu	THF	31
**7**	$PdCl_2$amphos	K_3PO_4	toluene	14
**8**	$PdCl_2$amphos	K_3PO_4	THF	15
**9**	$PdCl_2$amphos	NaOtBu	THF	30
**10**	$PdCl_2$amphos	NaOtBu	1,4-dioxane	25

**11**	$PdCl_2$ + CycAphos	K_3PO_4	1,4-dioxane	12
**12**	**$PdCl_2$ + CPhos**	**K_3PO_4**	**1,4-dioxane**	**75**
**13**	$PdCl_2$ + JackiePhos	K_3PO_4	1,4-dioxane	25
**14**	**$PdCl_2$ + DavePhos**	**K_3PO_4**	**1,4-dioxane**	**65**
**15**	$PdCl_2$ + *t*-BuDavePhos	K_3PO_4	1,4-dioxane	25
**16**	$PdCl_2$ + *t*-BuXPhos	K_3PO_4	1,4-dioxane	2
**17**	$PdCl_2$ + DavePhos	K_3PO_4	1,4-dioxane:H_2O (1:1)	70
**18**	$PdCl_2$ + DavePhos	K_3PO_4	1,4-dioxane:*i*-PrOH (1:1)	0
**19**	$PdCl_2$ + DavePhos	K_3PO_4	1,4-dioxane:*n*-BuOH (1:1)	0
**20**	$PdCl_2$ + DavePhos	K_3PO_4	1,4-dioxane:MeCN (1:1)	80
**21**[a]	**$PdCl_2$ + DavePhos**	**K_3PO_4**	**DMF**	**80**
**22**[a]	**$PdCl_2$ + DavePhos**	**K_3PO_4**	**DMSO**	**64**
**23**[a]	$PdCl_2$ + DavePhos	K_3PO_4	DCE	0
**24**[a]	$PdCl_2$ + DavePhos	K_3PO_4	NMP	81
**25**[a]	$PdCl_2$ + DavePhos	K_3PO_4	DME	0

Reactions were carried out on a 0.10 mmol scale. Yields were determined by ^{1}H NMR using 1,3,5-trimethoxybenzene as an internal standard. [a]Flurbiprofen methyl ester-derived thianthrenium salt (±)-**22a** was used.

General procedure for C–N cross-coupling reactions of aryl thianthrenium salts

A 4-mL borosilicate vial containing a Teflon-coated magnetic stirring bar was used. Arylthianthrenium salt (0.100 mmol, 1.00 equiv.), $PdCl_2$ (1.8 mg, 10 µmol, 10 mol%), CPhos (2-dicyclohexylphosphino-2′,6′-bis(*N,N*-dimethylamino)biphenyl, 8.7 mg, 20 µmol, 20 mol%), K_3PO_4 (42.5 mg, 0.200 mmol, 2.00 equiv.) and nitrogen nucleophile (1.5 equiv., if solid) were introduced into the vial. The vial was introduced into a glovebox, 1,4-dioxane (1.0 mL, *c* = 0.10 M) was added using a syringe. The reaction vial was taken out of the glovebox, nitrogen nucleophile (1.5 equiv., if liquid) was added. The mixture was stirred (600 rpm) at 80 °C. After 16 h, the mixture was diluted with EtOAc (2 mL) washed with water (2 mL), and dried over Na_2SO_4. Upon filtration, the organic layer was concentrated under reduced pressure by rotary evaporation and purified by flash column chromatography on silica gel to afford the desired product.

Probenecid derivative 48-S-I

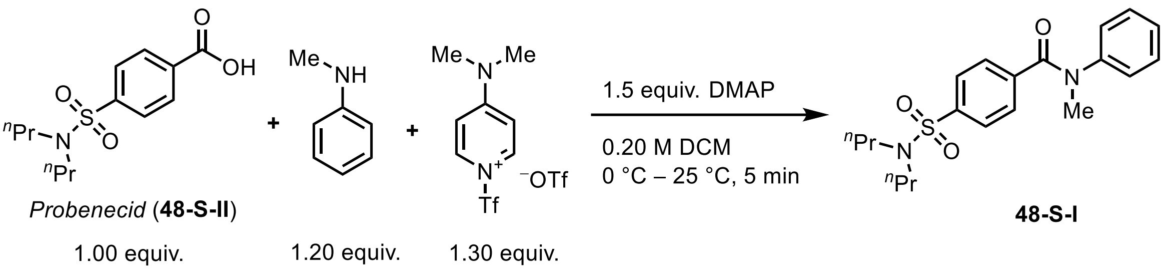

Prepared according to the reported procedure.[23] To a 25 mL flame-dried reaction tube equipped with a magnetic stirring bar, probenecid (**48-S-II)** (1.14 g, 4.00 mmol, 1.00 equiv.), DMAP (733 mg, 6.00 mmol, 1.50 equiv.), *N*-methylaniline (0.52 ml, 4.8 mmol, 1.2 equiv.) and DCM (20 mL) were added at 25 °C. Then the Tf-DMAP (2.1 g, 5.2 mmol, 1.3 equiv.) was added to reaction mixture. The reaction mixture was stirred for 5 min at 25 °C. Then the crude mixture was diluted with H_2O and extracted by DCM. The combined organic layers were dried over anhydrous Na_2SO_4. Then the volatiles were removed under reduced pressure and the resulting residue was purified by flash column chromatography on silica gel and eluted with ether / ethyl acetate to afford the desired amide **48-S-I** (1.16 g, 77% yield).

NMR Spectroscopy:

^{1}H NMR (500 MHz, $CDCl_3$, 23 ºC, δ): 7.58 (d, *J* = 8.2 Hz, 2H), 7.37 (d, *J* = 8.1 Hz, 2H), 7.20 (t, *J* = 7.6 Hz, 2H), 7.14 (t, *J* = 7.4 Hz, 1H), 6.99 (d, *J* = 9.2 Hz, 2H), 3.49 (s, 3H), 3.06 – 2.92 (m, 4H), 1.54 – 1.31 (m, 4H), 0.80 (t, *J* = 7.4 Hz, 6H).

^{13}C NMR (126 MHz, $CDCl_3$, 23 ºC, δ): 169.2, 144.1, 141.0, 139.9, 129.5, 129.2, 127.2, 127.0, 126.6, 49.8, 38.3, 21.8, 11.2.

HRMS-GC-EI (m/z) calc'd for $C_{20}H_{26}N_2O_3S$ $[M–BF_4]^+$, 374.1659; found, 374.1660; deviation: –0.4

Probenecid derivative-derived thianthrenium salt 48-S

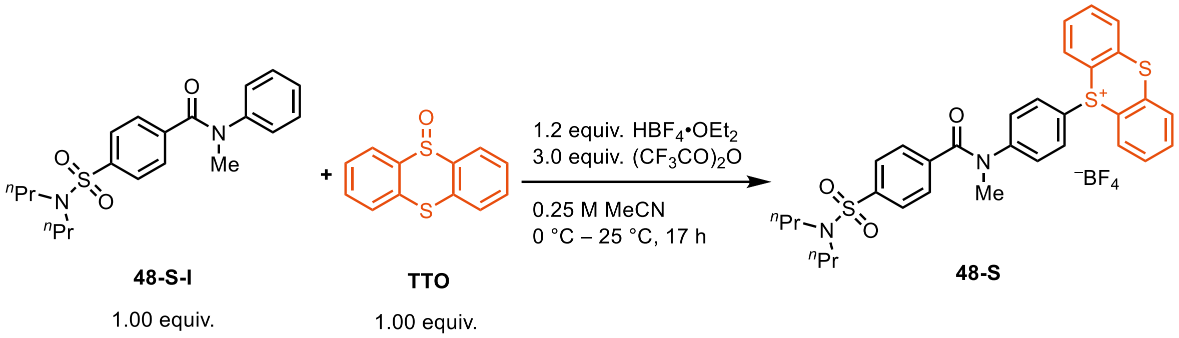

Prepared according to the reported procedure and the spectra are in good accordance with the literature.[24] Under an ambient atmosphere, a 100 mL round-bottomed flask was charged with probenecid amide **48-S-I** (1.5 g, 4.0 mmol, 1.0 equiv.), thianthrene-*S*-oxide (929 mg, 4.00 mmol, 1.00 equiv.) and MeCN (16 mL, c = 0.25 M) and placed in ice-water bath. TFAA (1.67 mL, 2.52 g, 12.0 mmol, 3.00 equiv.) was added to the solution at 0 °C followed by HBF_4 Et_2O (653 µL, 777 mg, 4.80 mmol, 1.20 equiv.). The reaction mixture was stirred at 0 °C for 1 h, then gradually warmed up to

25 °C and stirred at 25 °C for 16 h. Volatiles were evaporated, the residue was dissolved in DCM (40 mL) and the resulting solution was washed with water (20 mL) and aqueous $NaBF_4$ solution (3 × 25 mL, 10 % w/w). The organic layer was dried over Na_2SO_4, filtered and concentrated under reduced pressure. The resulting residue was purified by column chromatography on silica gel eluting with a solvent mixture of (DCM / MeOH 100:0 to 15:1 (v/v)) to afford 1.56 g (57%) of desired product **48-S** as an off-white solid.

R*f* = 0.33 (DCM / MeOH = 94:6 (v/v)).

NMR Spectroscopy:

^{1}**H NMR** (500 MHz, CD_3CN, 23 ºC, δ): 8.34 (dd, *J* = 7.9, 1.5 Hz, 2H), 7.93 (dd, *J* = 7.9, 1.4 Hz, 2H), 7.86 (td, *J* = 7.7, 1.4 Hz, 2H), 7.78 (td, *J* = 7.7, 1.4 Hz, 2H), 7.62 (d, *J* = 8.5 Hz, 2H), 7.32 (d, *J* = 8.5 Hz, 2H), 7.20 (d, *J* = 8.9 Hz, 2H), 6.94 (d, *J* = 8.9 Hz, 2H), 3.35 (s, 3H), 3.08 – 2.97 (m, 4H), 1.46 (h, *J* = 7.4 Hz, 4H), 0.79 (t, *J* = 7.4 Hz, 6H).

^{13}**C NMR** (126 MHz, CD_3CN, 23 ºC, δ): 169.5, 149.1, 142.0, 140.4, 137.4, 136.1, 136.0, 131.6, 130.8, 129.8, 129.7, 129.3, 127.5, 121.8, 119.5, 50.5, 38.0, 22.5, 11.3.

^{19}**F NMR** (471 MHz, CD_3CN, 23 ºC, δ): – 151.21.

HRMS-ESIpos (m/z) calc'd for $C_{32}H_{33}N_2O_3S_3$ $[M–BF_4]^+$, 589.1648; found, 589.1651; deviation: –0.5 ppm.

Probenecid-derived pyrrolidine derivative 48

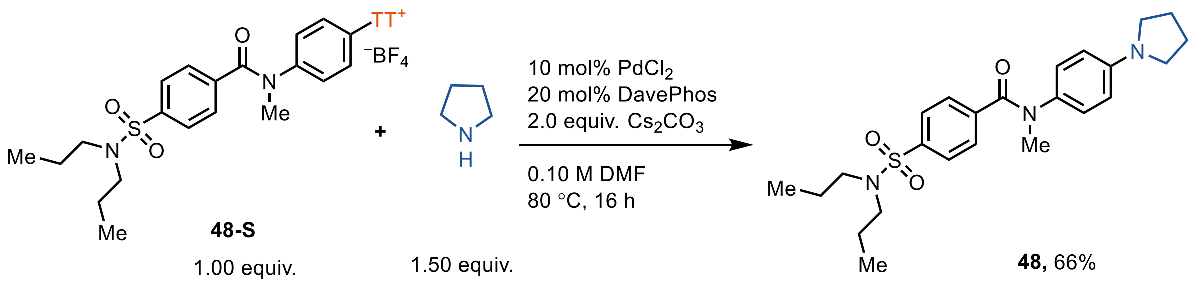

A 4-mL borosilicate vial containing a Teflon-coated magnetic stirring bar was used. Probenecid thianthrenium salt **48-S** (33.8 mg, 0.0500 mmol, 1.00 equiv.), $PdCl_2$ (0.9 mg, 15 µmol, 10 mol%), DavePhos (3.9 mg, 20 µmol, 20 mol%) and Cs_2CO_3 (32.6 mg, 0.200 mmol 2.00 equiv) were introduced into the vial. The vial was introduced into a glovebox and DMF (0.50 mL, *c* = 0.10 M) was added using a syringe. The reaction vial was taken out of the glovebox, pyrrolidine (6 µL, 5 mg, 0.08 mmol, 1.5 equiv.) was added. The mixture was stirred (600 rpm) at 80 °C. After 16 h, the mixture was diluted with EtOAc (2 mL), washed with brine (2 mL), and dried over Na_2SO_4. Upon filtration, the organic layer was concentrated and purified by flash column chromatography on silica gel (hexanes / EtOAc = 10:1 to 5:1 (v/v)) to afford the desired product **48** (14.6 mg, 66%).

R*f* = 0.17 (hexanes / EtOAc = 5:1 (v/v)).

NMR Spectroscopy:

^{1}**H NMR** (500 MHz, $CDCl_3$, 23 °C, δ): 7.58 (d, *J* = 8.1 Hz, 2H), 7.39 (d, *J* = 8.1 Hz, 2H), 6.81 (d, *J* =

8.4 Hz, 2H), 6.33 (d, *J* = 8.4 Hz, 2H), 3.44 (s, 3H), 3.21 (t, *J* = 6.6 Hz, 4H), 3.06 – 2.91 (m, 4H), 2.03 – 1.95 (m, 4H), 1.48 (h, *J* = 7.4 Hz, 4H), 0.82 (t, *J* = 7.4 Hz, 6H).

^{13}C NMR (151 MHz, $CDCl_3$, 23 °C, δ): 169.5, 146.6, 140.8, 140.2, 129.2, 128.0, 126.6, 112.0, 50.0, 47.8, 38.6, 25.6, 22.0, 11.3.

HRMS-ESI(m/z) calc'd for $C_{24}H_{33}N_3O_3SNa$ $[M+Na]^+$, 466.2135; found, 466.2141; deviation: –1.4 ppm

N-Phenylbenzenesulfonamide morpholine derivative 49

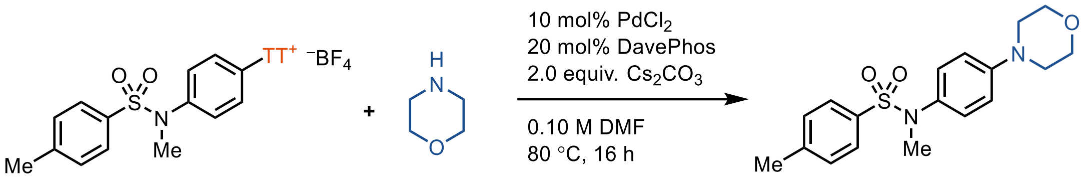

39a 1.00 equiv. 1.50 equiv. **49,** 71%

A 4-mL borosilicate vial containing a Teflon-coated magnetic stirring bar was used. *N*-phenylbenzenesulfonamide thianthrenium salt **39a** (54.9 mg, 0.100 mmol, 1.00 equiv.), $PdCl_2$ (1.8 mg, 10 µmol, 10 mol%), DavePhos (7.8 mg, 20.0 µmol, 20.0 mol%) and Cs_2CO_3 (65.2 mg, 0.200 mmol 2.00 equiv.) were introduced into the vial. The vial was introduced into a glovebox and DMF (1.0 mL, *c* = 0.10 M) was added using a syringe. The reaction vial was taken out of the glovebox, morpholine (13 µL, 13 mg, 0.15 mmol, 1.5 equiv.) was added. The mixture was stirred (600 rpm) at 80 °C. After 16 h, the mixture was diluted with EtOAc (2 mL), washed with brine (2 mL), and dried over Na_2SO_4. Upon filtration, the organic layer was concentrated and purified by flash column chromatography on silica gel (hexanes / EtOAc = 10:1 to 5:1 (v/v)) to afford the desired product **49** (24.5 mg, 71%).

R*f* = 0.19 (hexanes / EtOAc = 5:1 (v/v)).

NMR Spectroscopy:

^{1}H NMR (600 MHz, $CDCl_3$, 23 °C, δ): 7.49 – 7.41 (m, 2H), 7.24 (d, *J* = 8.0 Hz, 2H), 6.99 – 6.92 (m, 2H), 6.79 (d, *J* = 9.2 Hz, 2H), 3.87 – 3.82 (m, 4H), 3.17 – 3.13 (m, 4H), 3.12 (s, 3H), 2.42 (s, 3H).

^{13}C NMR (151 MHz, $CDCl_3$, 23 °C, δ): 150.3, 143.5, 133.8, 133.5, 129.4, 128.1, 127.9, 115.5, 66.9, 49.1, 38.5, 21.7.

HRMS-EI(m/z) calc'd for $C_{18}H_{22}N_2O_3S$ $[M]^+$, 346.1346; found, 346.1347; deviation: –0.3 ppm

Phenoxybenzonitrile 3-aminopyridine derivative 50

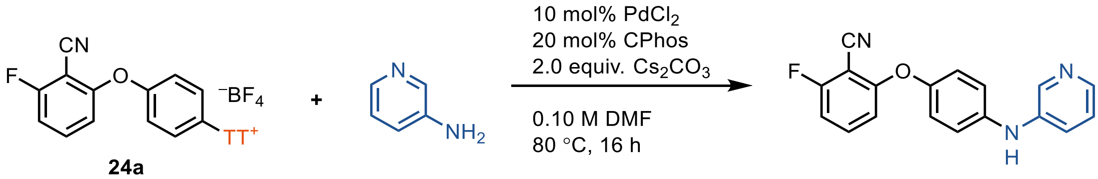

24a 1.00 equiv. 1.50 equiv. **50,** 70%

A 4-mL borosilicate vial containing a Teflon-coated magnetic stirring bar was used. Phenoxybenzonitrile-derived thianthrenium salt **24a** (51.5 mg, 0.100 mmol, 1.00 equiv.), $PdCl_2$ (1.8

mg, 10 µmol, 10 mol%), CPhos (8.7 mg, 20.0 µmol, 20.0 mol%) 3-aminopyridine (14.1 mg, 0.150 mmol, 1.50 equiv.) and Cs_2CO_3 (65.2 mg, 0.200 mmol 2.00 equiv.) were introduced into the vial. The vial was introduced into a glovebox and DMF (1.0 mL, *c* = 0.10 M) was added using a syringe. The reaction vial was taken out of the glovebox. The mixture was stirred (600 rpm) at 80 °C. After 16 h, the mixture was diluted with EtOAc (2 mL), washed with brine (2 mL), and dried over Na_2SO_4. Upon filtration, the organic layer was concentrated and purified by flash column chromatography on silica gel (hexanes / EtOAc = 10:1 to 5:1 The reaction vial was taken out of the glovebox) to afford the desired product **50** (21.3 mg, 70%).

R_f = 0.10 (hexanes / EtOAc = 5:1 The reaction vial was taken out of the glovebox).

NMR Spectroscopy:

^{1}H NMR (500 MHz, $(CD_3)_2SO$, 23 °C, δ): 8.49 (s, 1H), 8.36 (d, *J* = 2.7 Hz, 1H), 8.06 (dd, *J* = 4.7, 1.3 Hz, 1H), 7.68 (td, *J* = 8.5, 6.8 Hz, 1H), 7.49 (dd, *J* = 8.3, 4.3 Hz, 1H), 7.30 – 7.14 (m, 6H), 6.72 (d, *J* = 8.6 Hz, 1H).

^{13}C NMR (151 MHz, $(CD_3)_2SO$, 23 °C, δ): 163.3 (d, *J* = 255.7 Hz), 161.2, 147.2, 140.2, 140.4, 140.0, 139.0, 136.5 (d, *J* = 10.7 Hz), 123.9, 122.4, 121.6, 118.7, 111.8, 111.4, 109.6 (d, *J* = 19.1 Hz), 91.6.

^{19}F NMR (282 MHz, $(CD_3)_2SO$, 23 ºC, δ): –107.0.

HRMS-EI(m/z) calc'd for $C_{18}H_{12}N_3OF$ $[M]^+$, 305.0959; found, 305.0959; deviation: –1.5 ppm

(±)-Bifonazole derived thianthrenium salt 51-S

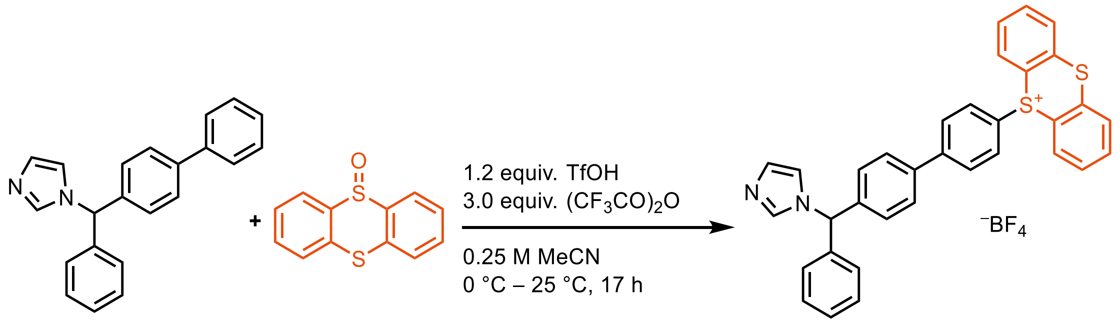

(±)-*Bifonazole* (**51-S-II**) 1.00 equiv. **TTO** 1.00 equiv. **(±)-51-S,** 55%

To a solution of (±)-bifonazole (**51-S-II**) (116 mg, 0.500 mmol, 1.00 equiv.) and TTO (thianthrene-*S*-oxide, 116 mg, 1.00 mmol, 1.00 equiv.) in MeCN (2.0 mL, *c* = 0.25 M), TfOH (49 µL, 83 mg, 0.55 mmol, 1.1 equiv.) and trifluoroacetic anhydride (0.21 mL, 0.31 g, 3.0 mmol, 3.0 equiv.) were added sequentially at 0 °C, under an ambient atmosphere followed by addition of TfOH (49 µL, 83 mg, 0.55 mmol, 1.1 equiv.). The reaction mixture was stirred (400 rpm) at 0 °C for 1 h, and then stirred at 25 °C for 16 h. The solvent was removed under reduced pressure by rotary evaporation and the residue was dissolved in DCM (15 mL). The resulting solution was washed with saturated aqueous $NaHCO_3$ solution (10 mL) and aqueous $NaBF_4$ solution (2 × 10 mL, 10% w/w). The DCM layer was dried over Na_2SO_4, filtered, and the solvent was removed under reduced pressure. Purification by flash silica gel column chromatography (EtOAc to DCM / MeOH = 20:1 (v/v)) afforded 167 mg of the title compound

(±)-51-S as a colorless solid (55% yield).

NMR Spectroscopy:

^{1}H NMR (600 MHz, CD_3CN, 23 °C, δ): 8.41 (dd, *J* = 8.1, 1.2 Hz, 2H), 8.12 (s, 1H), 7.98 (dd, *J* = 7.9, 1.3 Hz, 2H), 7.91 (td, *J* = 7.7, 1.4 Hz, 2H), 7.84 (td, *J* = 7.7, 1.3 Hz, 2H), 7.76 – 7.69 (m, 2H), 7.67 – 7.63 (m, 2H), 7.47 – 7.40 (m, 3H), 7.34 (s, 1H), 7.29 (d, *J* = 8.8 Hz, 2H), 7.26 – 7.18 (m, 5H), 6.89 (s, 1H).

^{13}C NMR (151 MHz, CD_3CN, 23 °C, δ): 145.5, 139.6, 139.4, 138.3, 137.7, 137.0, 136.3, 136.1, 131.8, 131.0, 130.3, 130.1, 129.9, 129.9, 129.7, 129.2, 129.0, 124.2, 123.9, 122.2, 119.6, 66.7.

^{19}F NMR (565 MHz, CD_3CN, 23 °C, δ): –151.6 (bs), –151.6 (bs).

(±)-Bifonazole acetamide derivative 51

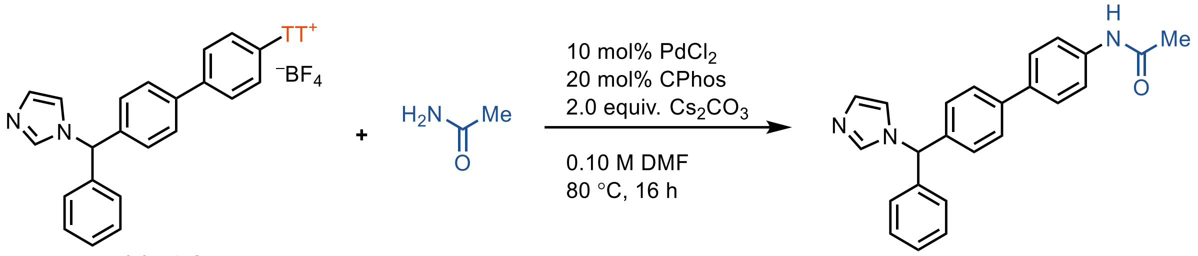

(±)-51-S 1.00 equiv. 1.50 equiv. **(±)-51,** 59%

A 4-mL borosilicate vial containing a Teflon-coated magnetic stirring bar was used. (±)-Bifonazole-derived thianthrenium salt **(±)-51-S** (61.3 mg, 0.100 mmol, 1.00 equiv.), $PdCl_2$ (1.8 mg, 10 µmol, 10 mol%), CPhos (8.7 mg, 20 µmol, 20 mol%), acetamide (8.9 mg, 0.15 mmol, 1.5 equiv.), and Cs_2CO_3 (65.2 mg, 0.200 mmol 2.00 equiv.) were introduced into the vial. The vial was introduced into a glovebox and DMF (1.0 mL, *c* = 0.10 M) was added using a syringe. The mixture was stirred (600 rpm) at 80 °C. After 16 h, the mixture was diluted with EtOAc (2 mL), washed with brine (2 mL), and dried over Na_2SO_4. Upon filtration, the organic layer was concentrated and purified by flash column chromatography on silica gel (hexanes / EtOAc = 10:1 (v/v) to EtOAc only) to afford the desired product **(±)-51** (21.7 mg, 59%).

R_f = 0.19 (EtOAc only).

NMR Spectroscopy:

^{1}H NMR (500 MHz, $CDCl_3$, 23 °C, δ): 8.10 (bs, 1H), 7.60 (d, *J* = 8.5 Hz, 2H), 7.52 (dd, *J* = 14.8, 8.5 Hz, 5H), 7.37 (d, *J* = 7.6 Hz, 3H), 7.14 (d, *J* = 8.1 Hz, 5H), 6.91 (bs, 1H), 6.56 (bs, 1H), 2.20 (s, 3H).

^{13}C NMR (151 MHz, $CDCl_3$, 23 °C, δ): 168.8, 140.8, 139.0, 138.1, 137.8, 135.9, 129.2, 129.1, 128.6, 128.6, 128.2, 127.6, 127.5, 127.3, 120.3, 65.0, 24.7.

HRMS-ESI(m/z) calc'd for $C_{24}H_{22}N_3O$ $[M+H]^+$, 368.1757; found, 368.1758; deviation: –0.1 ppm

(±)-Pyriproxyphen morpholine derivative 52

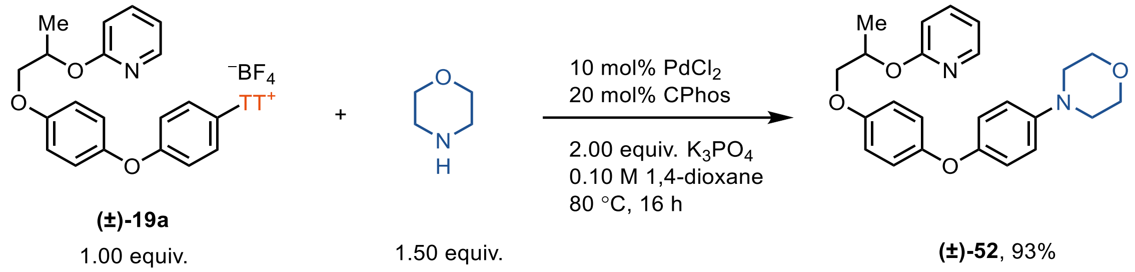

A 4-mL borosilicate vial containing a Teflon-coated magnetic stirring bar was used. (±)-Pyriproxyphen-derived thianthrenium salt **19a** (93.5 mg, 0.150 mmol, 1.00 equiv.), $PdCl_2$ (2.7 mg, 15 µmol, 10 mol%), CPhos (13.1 mg, 30.0 µmol, 20.0 mol%) and K_3PO_4 (63.7 mg, 0.300 mmol, 2.00 equiv.) were introduced into the vial. The vial was introduced into a glovebox and 1,4-dioxane (1.5 mL, *c* = 0.10 M) was added using a syringe. The reaction vial was taken out of the glovebox, morpholine (20 µL, 0.23 mmol, 1.5 equiv.) was added. The mixture was stirred (600 rpm) at 80 °C. After 16 h, the mixture was diluted with EtOAc (2 mL), washed with water (2 mL), and dried over Na_2SO_4. Upon filtration, the organic layer was concentrated and purified by flash column chromatography on silica gel (hexanes / EtOAc = 10:1 to 3:1 (v/v)) to afford the desired product **(±)-52** (56.7 mg, 93%).

R*f* = 0.22 (hexanes / EtOAc = 10:1).

NMR Spectroscopy:

^{1}H NMR (600 MHz, $CDCl_3$, 23 °C, δ): 8.15 (ddd, *J* = 5.1, 2.1, 0.9 Hz, 1H), 7.56 (ddd, *J* = 8.4, 7.1, 2.0 Hz, 1H), 6.94 – 6.81 (m, 9H), 6.74 (dt, *J* = 8.4, 0.9 Hz, 1H), 5.58 (dt, *J* = 6.4, 5.1 Hz, 1H), 4.17 (dd, *J* = 9.9, 5.3 Hz, 1H), 4.05 (dd, *J* = 9.9, 4.9 Hz, 1H), 3.88 – 3.83 (m, 4H), 3.12 – 3.06 (m, 4H), 1.47 (d, *J* = 6.4 Hz, 3H).

^{13}C NMR (151 MHz, $CDCl_3$, 23 °C, δ): 163.3, 154.8, 151.8, 151.7, 147.4, 146.1, 138.8, 119.7, 119.4, 117.5, 116.9, 115.8, 111.8, 71.3, 69.4, 67.0, 50.4, 17.1.

HRMS-ESI(m/z) calc'd for $C_{24}H_{27}N_2O_4$ $[M+H]^+$, 407.1965; found, 407.1962; deviation: +0.9 ppm

Procedure by using the Schlenk line: A 4-mL borosilicate vial containing a Teflon-coated magnetic stirring bar was used. (±)-Pyriproxyphen-derived thianthrenium salt **19a** (62.3 mg, 0.100 mmol, 1.00 equiv.), $PdCl_2$ (1.8 mg, 10 µmol, 10 mol%), CPhos (8.7 mg, 30 µmol, 20 mol%), K_3PO_4 (42.5 mg, 0.200 mmol, 2.00 equiv.) were introduced into the vial under an ambient atmosphere. After sealing with a Schlenk-line adapter, the vial was evacuated and backfilled with argon three times using a Schlenk line. 1,4-dioxane (1.0 mL, *c* = 0.10 M) was added using a syringe under Ar flow. The vial was sealed with a septum cap and morpholine (13 µL, 13 mg, 0.15 mmol, 1.5 equiv.) was added. The mixture was stirred (600 rpm) at 80 °C. After 16 h, the mixture was cooled to 25 °C. 1,3,5-trimethoxybenzene (16.8 mg, 0.100 mmol, 1.00 equiv.) was added into the reaction mixture as an internal standard, followed by EtOAc (2.0 mL) and H_2O (2 mL). Layers were separated and an aliquot (ca. 1 mL) from the EtOAc phase was taken and the concentrated by rotary evaporation. The residue was dissolved in $CDCl_3$ (0.6 mL), and the ^{1}H NMR was recorded. The yield (56%) was determined from the ^{1}H NMR spectrum by integrating the signal of the internal standard at 6.11 ppm (s, 3H) and

the product **52** at 3.88 – 3.83 ppm (m, 4H).

(±)-Pyriproxyphen piperidine derivative 53

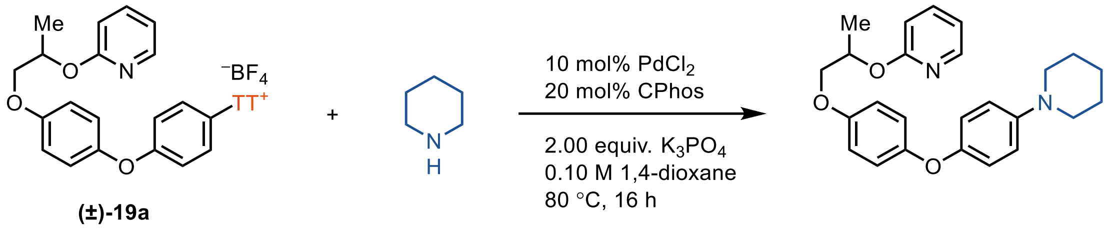

(±)-19a 1.00 equiv. 1.5 equiv. **(±)-53**, 63%

A 4-mL borosilicate vial containing a Teflon-coated magnetic stirring bar was used. (±)-Pyriproxyphen-derived thianthrenium salt **19a** (93.5 mg, 0.150 mmol, 1.00 equiv.), $PdCl_2$ (2.7 mg, 15 µmol, 10 mol%), CPhos (13.1 mg, 30.0 µmol, 20.0 mol%), K_3PO_4 (63.7 mg, 0.300 mmol, 2.00 equiv.) were introduced into the vial. The vial was introduced into a glovebox, 1,4-dioxane (1.5 mL, *c* = 0.10 M) was added using a syringe. The reaction vial was taken out of the glovebox, piperidine (22 µL, 19 mg, 0.23 mmol, 1.5 equiv.) was added. The mixture was stirred (600 rpm) at 80 °C. After 16 h, the mixture was diluted with EtOAc (2 mL), washed with water (2 mL), and dried over Na_2SO_4. Upon filtration, the organic layer was concentrated and purified by flash column chromatography on silica gel (EtOAc / hexanes = 1:20 to EtOAc / hexanes = 1:10 (v/v)) to afford the desired product **(±)-53** (38.4 mg, 63%).

R*f* = 0.40 (hexanes / EtOAc = 10:1 (v/v)).

NMR Spectroscopy:

^{1}H NMR (600 MHz, $CDCl_3$, 23 °C, δ): 8.14 (ddd, *J* = 5.0, 2.0, 0.8 Hz, 1H), 7.56 (ddd, *J* = 8.4, 7.1, 2.0 Hz, 1H), 6.94 – 6.82 (m, 9H), 6.74 (dt, *J* = 8.4, 0.9 Hz, 1H), 5.62 – 5.53 (m, 1H), 4.17 (dd, *J* = 9.9, 5.3 Hz, 1H), 4.05 (dd, *J* = 9.9, 4.8 Hz, 1H), 3.10 – 3.04 (m, 4H), 1.72 (q, *J* = 5.6 Hz, 4H), 1.56 (d, *J* = 5.7 Hz, 2H), 1.47 (d, *J* = 6.4 Hz, 3H).

^{13}C NMR (126 MHz, $CDCl_3$, 23 °C, δ): 163.3, 154.7, 151.9, 151.3, 148.4, 146.9, 138.8, 119.6, 119.3, 118.3, 116.9, 115.8, 111.8, 71.23, 69.4, 51.9, 26.1, 24.3, 17.1.

HRMS-ESI(m/z) calc'd for $C_{25}H_{29}N_2O_3$ $[M+H]^+$, 405.2173; found, 405.2169; deviation: +0.9 ppm.

(±)-Fenoprofen methyl ester pyrrolidine derivative 54

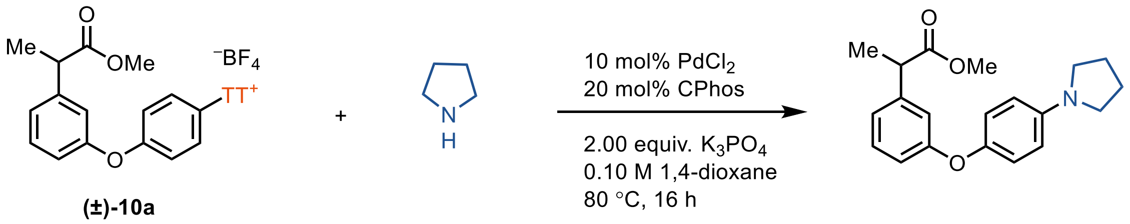

(±)-10a 1.00 equiv. 1.5 equiv. **(±)-54**, 71%

A 4-mL borosilicate vial containing a Teflon-coated magnetic stirring bar was used. (±)Fenoprofen methyl ester derived thianthrenium salt **10a** (83.7 mg, 0.150 mmol, 1.00 equiv.), $PdCl_2$ (2.7 mg, 15 µmol, 10 mol%), CPhos (13.1 mg, 30.0 µmol, 20.0 mol%), K_3PO_4 (63.7 mg, 0.300 mmol, 2.00 equiv.)

were introduced into the vial. The vial was introduced into a glovebox, 1,4-dioxane (1.5 mL, *c* = 0.10 M) was added using a syringe. The reaction vial was taken out of the glovebox, pyrrolidine (19 µL, 16 mg, 0.23 mmol, 1.5 equiv.) was added. The mixture was stirred (600 rpm) at 80 °C. After 16 h, the mixture was diluted with EtOAc (2 mL), washed with water (2 mL), and dried over Na_2SO_4. Upon filtration, the organic layer was concentrated and purified by flash column chromatography on silica gel (hexanes / EtOAc = 1:30 (v/v)) to afford the desired product **(±)-54** (23.1 mg, 71%).

R*f* = 0.45 (hexanes / EtOAc = 10:1)

NMR Spectroscopy:

^{1}H NMR (600 MHz, $CDCl_3$, 23 °C, δ): 7.19 (t, *J* = 7.9 Hz, 1H), 6.96 (d, *J* = 8.9 Hz, 2H), 6.92 (d, *J* = 7.7 Hz, 1H), 6.90 (t, *J* = 2.1 Hz, 1H), 6.76 (ddd, *J* = 8.3, 2.5, 0.9 Hz, 1H), 6.55 (d, *J* = 8.9 Hz, 2H), 3.66 (m, 4H), 3.31 – 3.27 (m, 4H), 2.04 – 2.00 (m, 4H), 1.47 (d, *J* = 7.2 Hz, 3H).

^{13}C NMR (151 MHz, $CDCl_3$, 23 °C, δ): 174.9, 159.8, 145.9, 145.3, 142.3, 129.7, 121.5, 120.8, 116.4, 115.3, 112.4, 52.2, 48.1, 45.5, 25.6, 18.7.

HRMS-EI(m/z) calc'd for $C_{20}H_{24}NO_3$ $[M+H]^+$, 326.1751; found, 326.1751; deviation: –0.1 ppm.

(±)-Flurbiprofen methyl ester dimethylamine derivative 55

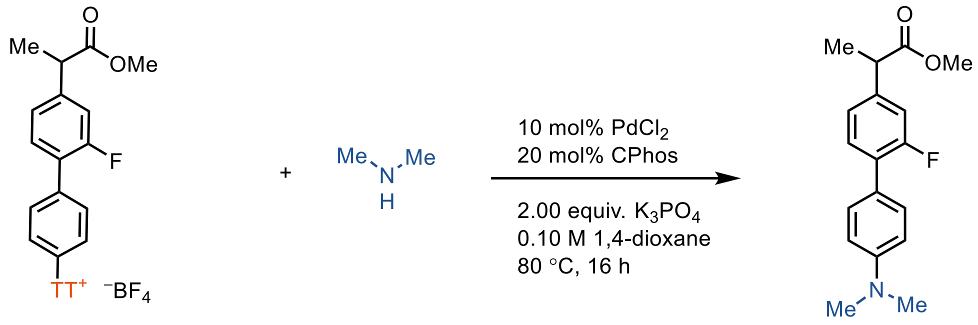

(±)-22a

1.00 equiv. 1.5 equiv. **(±)-55**, 57%

A 4-mL borosilicate vial containing a Teflon-coated magnetic stirring bar was used. (±)-Flurbiprofen methyl ester-derived thianthrenium salt **22a** (28.2 mg, 50.0 µmol, 1.00 equiv.), $PdCl_2$ (0.9 mg, 5 µmol, 10 mol%), CPhos (4.3 mg, 10 µmol, 20 mol%), K_3PO_4 (21.3 mg, 0.100 mmol, 2.00 equiv.) were introduced into the vial. The vial was introduced into a glovebox, 1,4-dioxane (0.50 mL, *c* = 0.10 M) was added using a syringe. The reaction vial was taken out of the glovebox, dimethylamine (2.0 M in THF, 38 µL, 26 mg, 75 µmol, 1.5 equiv.) was added. The mixture was stirred (600 rpm) at 80 °C. After 16 h, the mixture was diluted with EtOAc (2 mL), washed with water (2 mL), and dried over Na_2SO_4. Upon filtration, the organic layer was concentrated and purified by flash column chromatography on silica gel (EtOAc / hexanes = 1:30 (v/v)) to afford the desired product **(±)-55** (17.0 mg, 57%).

R*f* = 0.38 (hexanes / EtOAc = 10:1 (v/v)).

NMR Spectroscopy:

^{1}H NMR (600 MHz, CD_3CN, 23 °C, δ): 7.50 – 7.40 (m, 2H), 7.37 (t, *J* = 8.2 Hz, 1H), 7.14 – 7.03 (m, 2H), 6.82 – 6.76 (m, 2H), 3.74 (q, *J* = 7.4 Hz, 1H), 3.70 (s, 3H), 3.00 (s, 6H), 1.52 (d, *J* = 7.2

Hz, 3H).

^{13}C NMR (151 MHz, CD_3CN, 23C, δ): 174.8, 160.7, 159.1, 150.1, 140.5 (d, J = 7.6 Hz), 130.4 (d, J = 4.5 Hz), 129.7 (d, J = 3.5 Hz), 128.1 (d, J = 13.4 Hz), 123.5 (d, J = 3.5 Hz), 123.4, 115.3 (d, J = 24.2 Hz), 112.4, 52.3, 45.0, 40.6, 18.6.

^{19}F NMR (282 MHz, $CDCl_3$, 23 °C, δ): –117.9

HRMS-GC-EI(m/z) calc'd for $C_{18}H_{20}FNO_2$ $[M]^+$, 301.1472; found, 301.1475; deviation: –0.9 ppm.

(±)-Pyriproxyphen pentylamine derivative 56

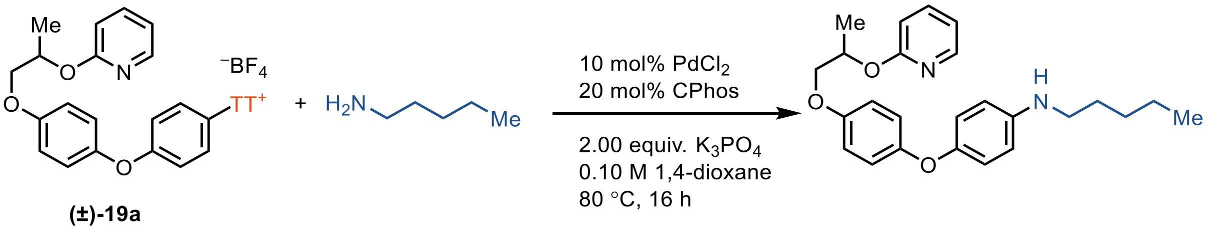

(±)-19a
1.00 equiv. 1.5 equiv. **(±)-56**, 43%

A 4-mL borosilicate vial containing a Teflon-coated magnetic stirring bar was used. (±)-Pyriproxyphen-derived thianthrenium salt **19a** (93.5 mg, 0.150 mmol, 1.00 equiv.), $PdCl_2$ (2.7 mg, 15 µmol, 10 mol%), CPhos (13.1 mg, 30.0 µmol, 20.0 mol%), K_3PO_4 (63.7 mg, 0.300 mmol, 2.00 equiv.) were introduced into the vial. The vial was introduced into a glovebox, 1,4-dioxane (1.5 mL, c = 0.10 M) was added using a syringe. The reaction vial was taken out of the glovebox, pentylamine (26 µL, 20 mg, 0.23 mmol, 1.5 equiv.) was added. The mixture was stirred (600 rpm) at 80 °C. After 16 h, the mixture was diluted with EtOAc (2 mL), washed with water (2 mL), and dried over Na_2SO_4. Upon filtration, the organic layer was concentrated and purified by flash column chromatography (EtOAc / hexanes = 1:30 (v/v)) on silica gel to afford the desired product **(±)-56** (26 mg, 43%).

R_f = 0.33 (hexanes / EtOAc = 10:1 (v/v))

NMR Spectroscopy:

^{1}H NMR (600 MHz, $CDCl_3$, 23 °C, δ): 8.14 (ddd, J = 5.0, 2.0, 0.8 Hz, 1H), 7.56 (ddd, J = 8.3, 7.1, 2.1 Hz, 1H), 6.93 – 6.80 (m, 7H), 6.73 (dt, J = 8.3, 0.9 Hz, 1H), 6.58 – 6.55 (m, 2H), 5.56 (dt, J = 6.4, 5.1 Hz, 1H), 4.16 (dd, J = 9.9, 5.3 Hz, 1H), 4.04 (dd, J = 9.9, 4.9 Hz, 1H), 3.15 – 2.97 (m, 2H), 1.69 – 1.57 (m, 2H), 1.47 (d, J = 6.3 Hz, 3H), 1.42 – 1.32 (m, 4H), 0.96 – 0.87 (m, 3H).

^{13}C NMR (151 MHz, $CDCl_3$, 23 °C, δ): 163.3, 154.4, 152.7, 149.0, 146.9, 144.9, 138.8, 120.4, 118.9, 116.9, 115.8, 113.8, 111.8, 71.3, 69.5, 44.8, 29.50, 29.47, 22.7, 17.1, 14.2.

HRMS-ESI(m/z) calc'd for $C_{25}H_{31}N_2O_3$ $[M+H]^+$, 407.2329; found, 407.2329; deviation: +0.1 ppm.

(±)-Flurbiprofen methyl ester furfurylamine derivative 57

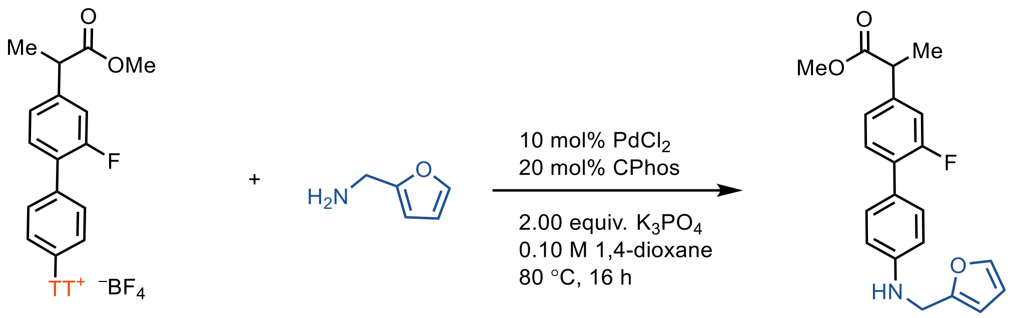

(±)-22a 1.00 equiv. 1.5 equiv. **(±)-57**, 44%

A 4-mL borosilicate vial containing a Teflon-coated magnetic stirring bar was used. (±)-Flurbiprofen methyl ester-derived thianthrenium salt **22a** (84.1 mg, 0.150 mmol, 1.00 equiv.), $PdCl_2$ (2.7 mg, 15 µmol, 10 mol%), CPhos (13.1 mg, 30.0 µmol, 20.0 mol%), K_3PO_4 (63.7 mg, 0.300 mmol, 2.00 equiv.) were introduced into the vial. The vial was introduced into a glovebox, 1,4-dioxane (1.5 mL, *c* = 0.10 M) was added using a syringe. The reaction vial was taken out of the glovebox, furfurylamine (20 µL, 22 mg, 0.23 mmol, 1.5 equiv.) was added. The mixture was stirred (600 rpm) at 80 °C. After 16 h, the mixture was diluted with EtOAc (2 mL), washed with water (2 mL), and dried over Na_2SO_4. Upon filtration, the organic layer was concentrated and purified by flash column chromatography (hexanes / EtOAc = 30:1 (v/v)) on silica gel to afford the desired product **(±)-57** (23.0 mg, 44%).

R*f* = 0.25 (hexanes / EtOAc = 10:1 (v/v)).

NMR Spectroscopy:

^{1}H NMR (600 MHz, $CDCl_3$, 23 °C, δ): 7.42 – 7.31 (m, 4H), 7.12 – 7.03 (m, 2H), 6.77 – 6.68 (m, 2H), 6.33 (dd, *J* = 3.2, 1.8 Hz, 1H), 6.26 (dq, *J* = 3.3, 0.8 Hz, 1H), 4.36 (s, 2H), 3.76 – 3.71 (m, 4H), 1.52 (d, *J* = 7.2 Hz, 3H).

^{13}C NMR (151 MHz, $CDCl_3$, 23 °C, δ): 174.7, 159.8 (d, *J* = 247.3 Hz), 152.7, 147.3, 142.1, 140.7 (d, *J* = 7.6 Hz), 130.4 (d, *J* = 4.4 Hz), 130.0 (d, *J* = 3.5 Hz), 128.0 (d, *J* = 13.4 Hz), 125.1, 123.5 (d, *J* = 3.2 Hz), 115.3 (d, *J* = 23.8 Hz), 113.1, 110.5, 107.2, 52.3, 45.0, 41.5, 18.6.

^{19}F NMR (471 MHz, $CDCl_3$, 23 °C, δ): –117.8

HRMS-ESI(m/z) calc'd for $C_{21}H_{21}FNO_3$ $[M+H]^+$, 354.1500; found, 354.1496; deviation: +1.1 ppm.

(±)-Pyriproxyphen cyclopentylamine derivative 58

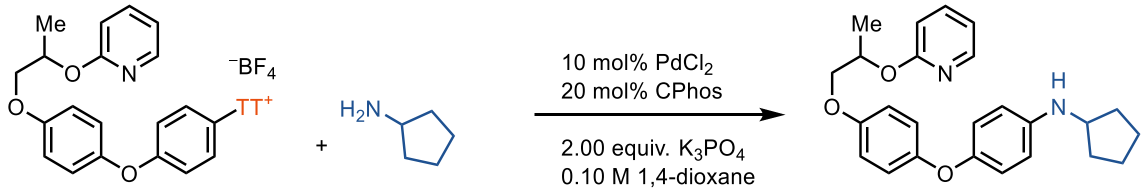

(±)-19a 1.00 equiv. 1.5 equiv. **(±)-58**, 42%

A 4-mL borosilicate vial containing a Teflon-coated magnetic stirring bar was used. (±)-Pyriproxyphen-

derived thianthrenium salt **19a** (93.5 mg, 0.150 mmol, 1.00 equiv.), $PdCl_2$ (2.7 mg, 15 µmol, 10 mol%), CPhos (13.1 mg, 30.0 µmol, 20.0 mol%), K_3PO_4 (63.7 mg, 0.300 mmol, 2.00 equiv.) were introduced into the vial. The vial was introduced into a glovebox, 1,4-dioxane (1.5 mL, *c* = 0.10 M) was added using a syringe. The reaction vial was taken out of the glovebox, cyclopentylamine (22 µL, 19 mg, 0.23 mmol, 1.5 equiv.) was added. The mixture was stirred (600 rpm) at 80 °C. After 16 h, the mixture was diluted with EtOAc (2 mL), washed with water (2 mL), and dried over Na_2SO_4. Upon filtration, the organic layer was concentrated and purified by flash column chromatography (hexanes / EtOAc= 30:1) on silica gel to afford the desired product **(±)-58** (24 mg, 42%).

R*f* = 0.30 (hexanes / EtOAc = 10:1 (v/v)).

NMR Spectroscopy:

^{1}H NMR (600 MHz, $CDCl_3$, 23 °C, δ): 8.14 (ddd, *J* = 5.0, 2.0, 0.8 Hz, 1H), 7.56 (ddd, *J* = 8.4, 7.1, 2.1 Hz, 1H), 6.91 – 6.79 (m, 7H), 6.74 (dt, *J* = 8.4, 0.9 Hz, 1H), 6.62 – 6.50 (m, 2H), 5.61 – 5.49 (m, 1H), 4.16 (dd, *J* = 9.9, 5.3 Hz, 1H), 4.04 (dd, *J* = 9.9, 4.8 Hz, 1H), 3.78 – 3.69 (m, 1H), 2.06 – 1.96 (m, 2H), 1.77 – 1.68 (m, 2H), 1.67 – 1.57 (m, 2H), 1.52 – 1.42 (m, 5H).

^{13}C NMR (151 MHz, $CDCl_3$, 23 °C, δ): 163.3, 154.4, 152.7, 148.9, 146.9, 144.4, 138.8, 120.3, 118.9, 116.9, 115.8, 114.3, 111.8, 71.3, 69.5, 55.4, 33.8, 24.2, 17.1.

HRMS-ESI(m/z) calc'd for $C_{25}H_{29}N_2O_3$ $[M+H]^+$, 405.2173; found, 405.2169; deviation: +0.8 ppm.

(±)-Flurbiprofen methyl ester isopropylamine derivative 59

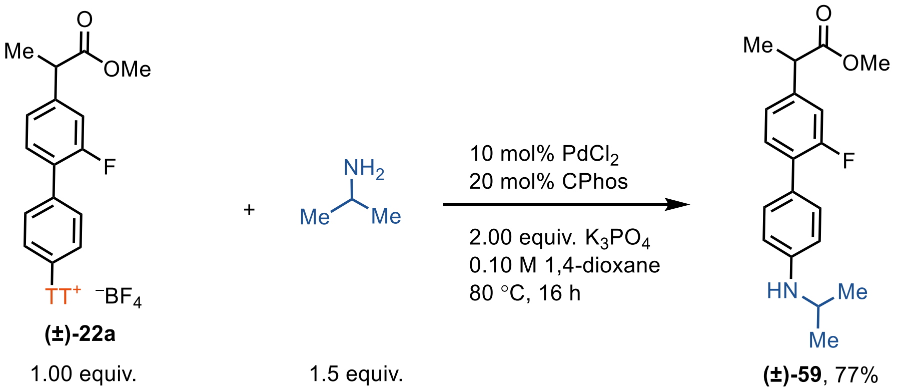

A 4-mL borosilicate vial containing a Teflon-coated magnetic stirring bar was used. (±)-Flurbiprofen methyl ester-derived thianthrenium salt **22a** (56.0 mg, 0.100 mmol, 1.00 equiv.), $PdCl_2$ (1.8 mg, 10 µmol, 10 mol%), CPhos (8.7 mg, 20 µmol, 20 mol%), K_3PO_4 (42.5 mg, 0.200 mmol, 2.00 equiv.) were introduced into the vial. The vial was introduced into a glovebox, 1,4-dioxane (1.0 mL, *c* = 0.10 M) was added using a syringe. The reaction vial was taken out of the glovebox, isopropylamine (13 µL, 8.9 mg, 0.15 mmol, 1.5 equiv.) was added. The mixture was stirred (600 rpm) at 80 °C. After 16 h, the mixture was diluted with EtOAc (2 ml) washed with water (2 mL), and dried over Na_2SO_4. Upon filtration, the organic layer was concentrated and purified by flash column chromatography (hexanes / EtOAc =20:1 to 10:1 (v/v)) on silica gel to afford the desired product **(±)-59** (24.3 mg, 77%).

R*f* = 0.33 (hexanes / EtOAc = 5:1 (v/v)).

NMR Spectroscopy:

^{1}H NMR (600 MHz, $CDCl_3$, 23 °C, δ): 7.40 – 7.32 (m, 3H), 7.12 – 7.03 (m, 2H), 6.68 – 6.57 (m, 2H), 3.78 – 3.64 (m, 5H), 3.60 (s, 1H), 1.52 (d, J = 7.2 Hz, 3H), 1.24 (d, J = 6.3 Hz, 6H).

^{13}C NMR (151 MHz, $CDCl_3$, 23 °C, δ): 174.8, 159.8 (d, J = 247.3 Hz), 147.2, 140.4 (d, J = 7.6 Hz), 130.3 (d, J = 4.2 Hz), 130.0 (d, J = 3.3 Hz), 128.2 (d, J = 13.3 Hz), 123.5 (d, J = 3.2 Hz), 115.2 (d, J = 23.9 Hz), 113.0, 52.3, 45.0, 44.3, 23.2, 18.6.

^{19}F NMR (565 MHz, $CDCl_3$, 23 °C, δ): –117.8

HRMS-FIA(m/z) calc'd for $C_{19}H_{22}NO_2F$ $[M]^+$, 315.1629; found, 315.1631; deviation: –0.7 ppm.

(±)-Fenoprofen methyl ester *N*-ethylaniline derivative 60

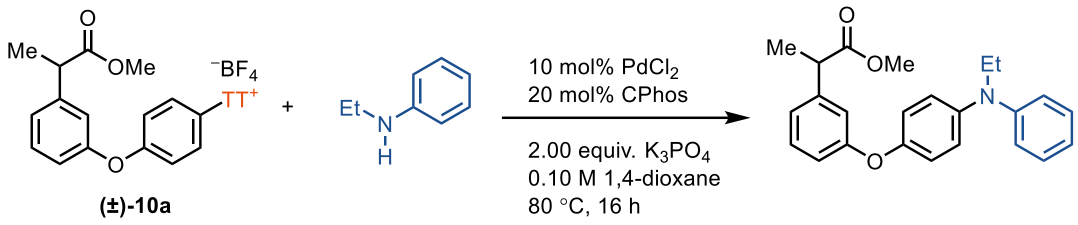

(±)-10a

1.00 equiv. 1.5 equiv. **(±)-60**, 51%

A 4-mL borosilicate vial containing a Teflon-coated magnetic stirring bar was used. (±)-Fenoprofen methyl ester-derived thianthrenium salt **10a** (83.7 mg, 0.150 mmol, 1.00 equiv.), $PdCl_2$ (2.7 mg, 15 μmol, 10 mol%), CPhos (13.1 mg, 30.0 μmol, 20.0 mol%), K_3PO_4 (63.7 mg, 0.300 mmol, 2.00 equiv.) were introduced into the vial. The vial was introduced into a glovebox, 1,4-dioxane (1.5 mL, c = 0.10 M) was added using a syringe. The reaction vial was taken out of the glovebox, *N*-ethylaniline (28 µL, 27 mg, 0.23 mmol, 1.5 equiv.) was added. The mixture was stirred (600 rpm) at 80 °C. After 16 h, the mixture was diluted with EtOAc (2 mL) washed with water (2 mL), and dried over Na_2SO_4. Upon filtration, the organic layer was concentrated and purified by flash column chromatography (EtOAc / hexanes = 1:30 (v/v)) on silica gel to afford the desired product **(±)-60** (28.8 mg, 51%).

R_f = 0.45 (hexanes / EtOAc = 10:1).

NMR Spectroscopy:

^{1}H NMR (600 MHz, $CDCl_3$, 23 ºC, δ): 7.31 – 7.17 (m, 3H), 7.07 – 6.95 (m, 6H), 6.91 – 6.81 (m, 4H), 3.80 – 3.70 (m, 3H), 3.68 (s, 3H), 1.50 (d, J = 7.2 Hz, 3H), 1.24 (t, J = 7.1 Hz, 3H).

^{13}C NMR (151 MHz, $CDCl_3$, 23 ºC, δ): 174.8, 158.1, 152.1, 148.2, 143.5, 142.6, 129.9, 129.3, 124.9, 122.1, 120.3, 119.8, 118.4, 117.8, 116.8, 52.2, 46.7, 45.4, 18.7, 12.8.

HRMS-ESI(m/z) calc'd for $C_{24}H_{25}O_3NNa$ $[M+Na^+]^+$, 398.1727; found, 398.1726; deviation: +0.1 ppm.

(±)-Fenoprofen methyl ester aniline derivative 61

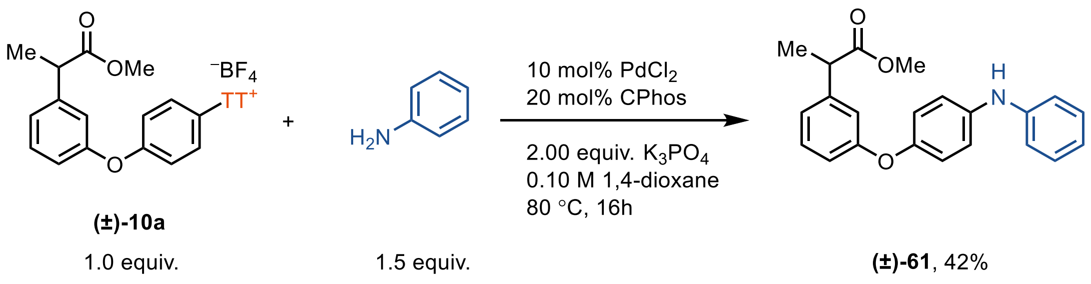

A 4-mL borosilicate vial containing a Teflon-coated magnetic stirring bar was used. (±)-Fenoprofen methyl ester-derived thianthrenium salt **10a** (27.9 mg, 0.0500 mmol, 1.00 equiv.), $PdCl_2$ (0.9 mg, 5.0 µmol, 10 mol%), CPhos (4.4 mg, 10 µmol, 20 mol%), K_3PO_4 (21.2 mg, 0.100 mmol, 2.00 equiv.) were introduced into the vial. The vial was introduced into a glovebox, dioxane (0.50 mL, *c* = 0.10 M) was added using a syringe. The reaction vial was taken out of the glovebox, phenylamine (7 µL, 7 mg, 8 µmol, 2 equiv.) was added. The mixture was stirred (600 rpm) at 80 °C. After 16 h, the mixture was diluted with EtOAc (2 mL) washed with water (2 mL), and dried over Na_2SO_4. Upon filtration, the organic layer was concentrated and purified by flash column chromatography (EtOAc / hexanes = 1:10 (v/v)) on silica gel to afford the desired product **(±)-61** (7.2 mg, 42%).

R*f* = 0.27 (hexanes / EtOAc = 10:1)

NMR Spectroscopy:

^{1}H NMR (600 MHz, $CDCl_3$, 23 ºC, δ): 7.28 – 7.23 (m, 3H), 7.10 – 7.05 (m, 2H), 7.04 – 6.95 (m, 6H), 6.90 (tt, *J* = 7.4, 1.2 Hz, 1H), 6.85 (ddd, *J* = 8.2, 2.5, 1.0 Hz, 1H), 5.63 (bs, 1H), 3.70 (q, *J* = 7.2 Hz, 1H), 3.67 (s, 3H), 1.49 (d, *J* = 7.2 Hz, 3H).

^{13}C NMR (151 MHz, $CDCl_3$, 23 ºC, δ): 174.9, 158.5, 151.8, 144.0, 142.5, 129.9, 129.5, 121.8, 120.7, 120.4, 117.4, 117.1, 116.5, 52.2, 45.5, 29.9, 24.5, 18.7.

HRMS-GC-EI(m/z) calc'd for $C_{22}H_{21}NO_3$ $[M]^+$, 347.1516; found, 347.1522; deviation: –1.6 ppm.

(±)-Pyriproxyphen 3-aminopyridine derivative 62

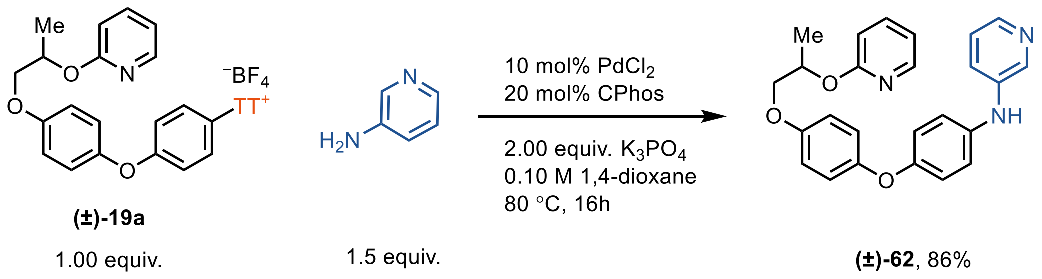

A 4-mL borosilicate vial containing a Teflon-coated magnetic stirring bar was used. (±)-Pyriproxyphen-derived thianthrenium salt **19a** (62.3 mg, 0.100 mmol, 1.00 equiv.), $PdCl_2$ (1.8 mg, 10 µmol, 10 mol%), CPhos (8.7 mg, 20 µmol, 20.0 mol%), 3-aminopyridine (14.1 mg, 0.150 mmol, 1.50 equiv.) and K_3PO_4 (42.5 mg, 0.200 mmol, 2.00 equiv.) were introduced into the vial. The vial was introduced into a glovebox, 1,4-dioxane (1.0 mL, *c* = 0.10 M) was added using a syringe. The reaction vial was taken

out of the glovebox. The mixture was stirred (600 rpm) at 80 °C. After 16 h, the mixture was diluted with EtOAc (2 mL) washed with water (2 mL), and dried over Na_2SO_4. Upon filtration, the organic layer was concentrated and purified by flash column chromatography (EtOAc / hexanes = 1:30 (v/v)) on silica gel to afford the desired product **(±)-62** (35.7 mg, 86%).

R*f* = 0.21 (hexanes / EtOAc = 1:1 (v/v))

NMR Spectroscopy:

^{1}H NMR (600 MHz, $CDCl_3$, 23 °C, δ): 8.30 (s, 1H), 8.14 (ddd, *J* = 5.0, 2.1, 0.9 Hz, 1H), 8.10 (s, 1H), 7.56 (ddd, *J* = 8.3, 7.1, 2.0 Hz, 1H), 7.28 (ddd, *J* = 8.3, 2.8, 1.3 Hz, 1H), 7.13 (dd, *J* = 8.3, 4.6 Hz, 1H), 7.08 – 7.03 (m, 2H), 6.97 – 6.89 (m, 7H), 6.85 (ddd, *J* = 7.1, 5.0, 1.0 Hz, 1H), 6.74 (dt, *J* = 8.3, 0.9 Hz, 1H), 5.78 (bs, 1H), 5.66 – 5.50 (m, 1H), 4.18 (dd, *J* = 9.9, 5.3 Hz, 1H), 4.07 (dd, *J* = 9.9, 4.8 Hz, 1H), 1.48 (d, *J* = 6.4 Hz, 3H).

^{13}C NMR (151 MHz, $CDCl_3$, 23 °C, δ): 163.3, 155.2, 153.9, 151.0, 146.9, 141.2, 139.0, 138.8, 136.6, 123.9, 122.1, 121.6, 120.2, 119.3, 116.9, 116.1, 111.8, 71.2, 69.4, 44.8, 17.1.

HRMS-ESI(m/z) calc'd for $C_{25}H_{24}O_3N_3$ $[M+H]^+$, 414.1812; found, 414.1812; deviation: 0.0 ppm.

(±)-Flurbiprofen methyl ester *para*-methylaniline derivative 63

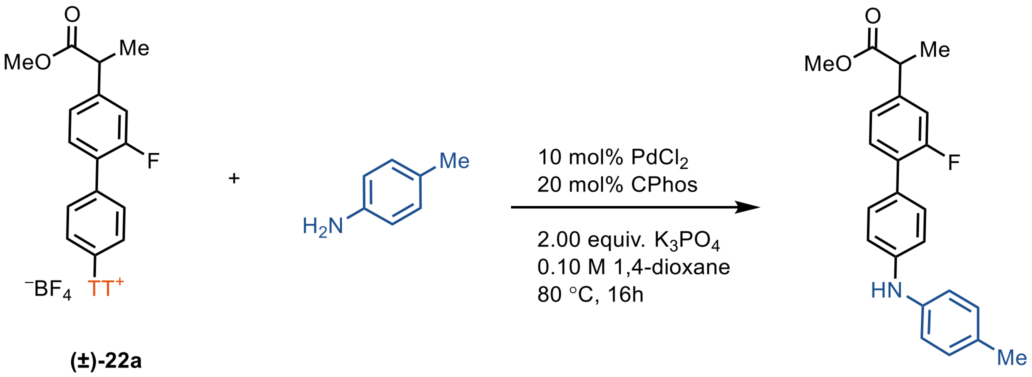

(±)-22a 1.00 equiv. 1.5 equiv. **(±)-63**, 71%

A 4-mL borosilicate vial containing a Teflon-coated magnetic stirring bar was used. (±)-Flurbiprofen methyl ester-derived thianthrenium salt **22a** (56.0 mg, 0.100 mmol, 1.00 equiv.), $PdCl_2$ (1.8 mg, 10 µmol, 10 mol%), CPhos (8.7 mg, 20 µmol, 20 mol%), K_3PO_4 (42.5 mg, 0.200 mmol, 2.00 equiv.) were introduced into the vial. The vial was introduced into a glovebox, 1,4-dioxane (1.0 mL, *c* = 0.10 M) was added using a syringe. The reaction vial was taken out of the glovebox, *para*-methylaniline (15 µL, 16 mg, 0.15 mmol, 1.5 equiv.) was added. The mixture was stirred at 80 °C. After 16 h, the mixture was diluted with EtOAc (2 mL) washed with water (2 mL), and dried over Na_2SO_4. Upon filtration, the organic layer was concentrated and purified by flash column chromatography (hexanes / EtOAc = 30:1 (v/v)) on silica gel to afford the desired product **(±)-63** (25.7 mg, 71%).

R*f* = 0.43 (hexanes / EtOAc = 10:1 (v/v)).

NMR Spectroscopy:

^{1}H NMR (600 MHz, $CDCl_3$, 23 °C, δ): 7.45 – 7.41 (m, 2H), 7.38 (t, *J* = 8.1 Hz, 1H), 7.15 – 7.08 (m,

4H), 7.08 – 7.04 (m, 4H), 5.73 (bs, 1H), 3.76 (q, J = 7.2 Hz, 1H), 3.71 (s, 3H), 2.33 (s, 3H), 1.54 (d, J = 7.2 Hz, 3H).

^{13}C NMR (151 MHz, $CDCl_3$, 23 °C, δ): 174.7, 149.8 (d, J = 247.3 Hz), 143.9, 141.0 (d, J = 7.9 Hz), 139.9, 131.6, 130.4 (d, J = 4.1 Hz), 130.0, 129.9 (d, J = 3.1 Hz), 127.8 (d, J = 13.4 Hz), 127.1, 123.6 (d, J = 3.2 Hz), 119.6, 116.3, 115.3 (d, J = 23.8 Hz), 52.3, 45.0, 20.9, 18.6.

^{19}F NMR (565 MHz, $CDCl_3$, 23 °C, δ): –117.7

HRMS-FIA(m/z) calc'd for $C_{23}H_{22}NO_2NaF$ $[M+Na]^+$,386.1527; found, 386.1526; deviation: +0.2 ppm.

(±)-Fenoprofen methyl ester acetamide derivative 64

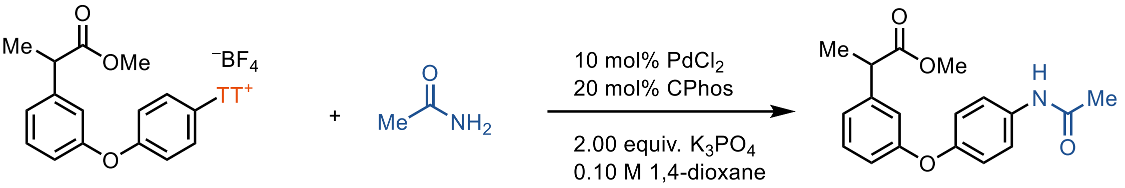

(±)-10a 1.00 equiv. 1.5 equiv. **(±)-64**, 31%

A 4-mL borosilicate vial containing a Teflon-coated magnetic stirring bar was used. (±)-Fenoprofen methyl ester-derived thianthrenium salt **10a** (27.9 mg, 0.0500 mmol, 1.00 equiv.), $PdCl_2$ (0.9 mg, 5 µmol, 10 mol%), CPhos (4.4 mg, 10 µmol, 20 mol%), K_3PO_4 (21.2 mg, 0.100 mmol, 2.00 equiv.) and acetamide (4.4 mg, 75 µmol, 1.5 equiv.) were introduced into the vial. The vial was introduced into a glovebox, 1,4-dioxane (0.50 mL, c = 0.10 M) was added using a syringe. The reaction vial was taken out of the glovebox. The mixture was stirred (600 rpm) at 80 °C. After 16 h, the mixture was diluted with EtOAc (2 mL) washed with water (2 mL), and dried over Na_2SO_4. Upon filtration, the organic layer was concentrated and purified by flash column chromatography (hexanes / EtOAc = 10:1 to 1:1 (v/v)) on silica gel to afford the desired product **(±)-64** (4.9 mg, 31%).

R_f = 0.29 (hexanes / EtOAc = 1:1)

NMR Spectroscopy:

^{1}H NMR (600 MHz, $CDCl_3$, 23 °C, δ): 7.48 – 7.43 (m, 2H), 7.25 (t, J = 7.9 Hz, 1H), 7.16 (bs, 1H), 7.03 – 6.91 (m, 4H), 6.84 (dd, J = 8.2, 3.5 Hz, 1H), 3.69 (q, J = 6.9 Hz, 1H), 3.66 (s, 3H), 2.18 (s, 3H), 1.48 (d, J = 7.2 Hz, 3H).

^{13}C NMR (151 MHz, $CDCl_3$, 23 °C, δ): 174.8, 168.3, 157.8, 153.4, 142.6, 133.6, 130.0, 122.3, 121.8, 119.8, 117.8, 117.0, 52.3, 45.4, 24.6, 18.6.

HRMS-GC-EI (m/z) calc'd for $C_{18}H_{19}NO_4$ $[M]^+$, 313.1309; found, 313.1315; deviation: –2.0 ppm.

(±)-Flurbiprofen methyl ester acetamide derivative 65

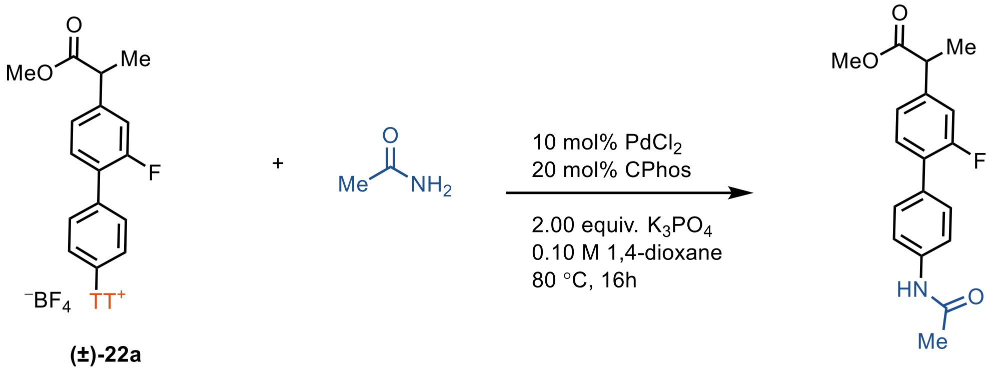

(±)-22a 1.00 equiv. 1.5 equiv. **(±)-65**, 77%

A 4-mL borosilicate vial containing a Teflon-coated magnetic stirring bar was used. (±)-Flurbiprofen methyl ester-derived thianthrenium salt **22a** (28.0 mg, 0.0500 mmol, 1.00 equiv.), $PdCl_2$ (0.9 mg, 5 µmol, 10 mol%), CPhos (4.4 mg, 10 µmol, 20 mol%), K_3PO_4 (21.2 mg, 0.100 mmol, 2.00 equiv.) and acetamide (4.4 mg, 75 µmol, 1.5 equiv.) were introduced into the vial. The vial was introduced into a glovebox, 1,4-dioxane (0.50 mL, *c* = 0.10 M) was added using a syringe. The reaction vial was taken out of the glovebox. The mixture was stirred (600 rpm) at 80 °C. After 16 h, the mixture was diluted with EtOAc (2 mL) washed with water (2 mL), and dried over Na_2SO_4. Upon filtration, the organic layer was concentrated and purified by flash column chromatography (hexanes / EtOAc = 1:1 (v/v)) on silica gel to afford the desired product **(±)-65** (12.1 mg, 77%).

R*f* = 0.38 (hexanes / EtOAc = 1:1 (v/v))

NMR Spectroscopy:

^{1}H NMR (600 MHz, $CDCl_3$, 23 °C, δ):7.57 (d, *J* = 8.7 Hz, 2H), 7.49 (d, *J* = 7.0 Hz, 2H), 7.40 (bs, 1H), 7.36 (t, *J* = 8.0 Hz, 1H), 7.17 – 7.08 (m, 2H), 3.75 (q, *J* = 6.9 Hz, 1H), 3.70 (s, 3H), 2.19 (s, 3H), 1.53 (d, *J* = 7.2 Hz, 3H).

^{13}C NMR (151 MHz, CD_3CN, 23 °C, δ): 174.6, 168.5, 159.8 (d, *J* = 248.0 Hz), 141.8 (d, *J* = 7.6 Hz), 137.6, 131.5, 130.7 (d, *J* = 3.8 Hz), 129.7 (d, *J* = 3.2 Hz), 127.3 (d, *J* = 13.4 Hz), 123.7 (d, *J* = 3.5 Hz), 119.9, 115.4 (d, *J* = 23.5 Hz), 52.4, 45.0 (d, *J* = 1.4 Hz), 24.8, 18.6.

^{19}F NMR (565 MHz, $CDCl_3$, 23 °C, δ): –117.6

HRMS-GC-EI(m/z) calc'd for $C_{18}H_{18}NO_3F$ $[M]^+$, 315.1265; found, 315.1271; deviation: –1.9 ppm.

(±)-Flurbiprofen methyl ester carbamate derivative 66

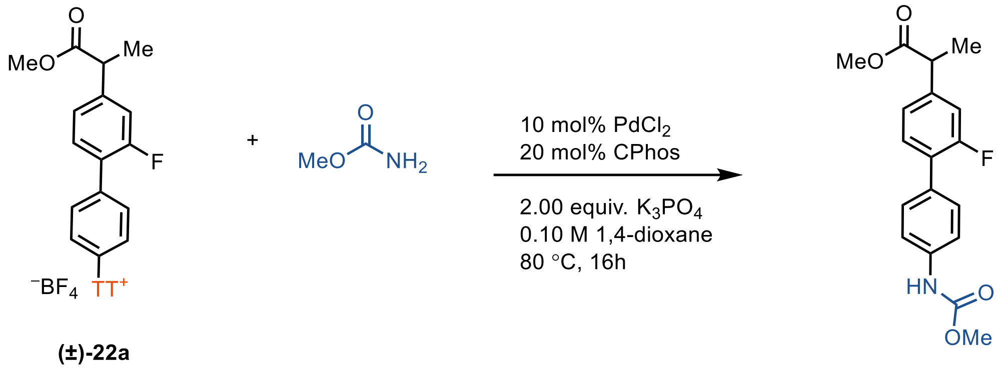

(±)-22a 1.00 equiv.   1.5 equiv.   **(±)-66**, 39%

A 4-mL borosilicate vial containing a Teflon-coated magnetic stirring bar was used. (±)-Fluribiprofen methyl ester-derived thianthrenium salt **22a** (28.0 mg, 0.0500 mmol, 1.00 equiv.), $PdCl_2$ (0.9 mg, 5 µmol, 10 mol%), CPhos (4.4 mg, 10 µmol, 20 mol%), K_3PO_4 (21.2 mg, 0.100 mmol, 2.00 equiv.) and methyl carbamate (5.6 mg, 75 µmol, 1.5 equiv.) were introduced into the vial. The vial was introduced into a glovebox, 1,4-dioxane (0.50 mL, c = 0.10 M) was added using a syringe. The reaction vial was taken out of the glovebox. The mixture was stirred (600 rpm) at 80 °C. After 16 h, the mixture was diluted with EtOAc (2 mL) washed with water (2 mL), and dried over Na_2SO_4. Upon filtration, the organic layer was concentrated and purified by flash column chromatography (hexanes / EtOAc = 1:1 (v/v)) on silica gel to afford the desired product **(±)-66** (6.4 mg, 39%).

R*f* = 0.13 (hexanes / EtOAc = 10:1 (v/v)).

NMR Spectroscopy:

^{1}H NMR (600 MHz, $CDCl_3$, 23 ºC, δ): 7.51 – 7.43 (m, 4H), 7.37 (t, J = 8.1 Hz, 1H), 7.15 – 7.08 (m, 2H), 6.67 (bs, 1H), 3.80 (s, 3H), 3.75 (q, J = 7.2 Hz, 1H), 3.70 (s, 3H), 1.53 (d, J = 7.3 Hz, 3H).

^{13}C NMR (151 MHz, $CDCl_3$, 23 ºC, δ): 174.6, 168.5, 159.8 (d, J = 248.0 Hz), 141.8 (d, J = 7.6 Hz), 137.6, 131.5, 130.7 (d, J = 3.8 Hz), 129.7 (d, J = 3.2 Hz), 127.3 (d, J = 13.4 Hz), 123.7 (d, J = 3.3 Hz), 119.9, 115.4 (d, J = 23.5 Hz), 52.4, 45.0 (d, J = 1.4 Hz), 24.8, 18.6.

^{19}F NMR (565 MHz, $CDCl_3$, 23 ºC, δ): –117.6

HRMS-ESI(m/z) calc'd for $C_{18}H_{18}FNO_4Na$ $[M+Na]^+$, 354.1112; found, 354.1113; deviation: –0.3 ppm.

(±)-Flurbiprofen methyl ester isobutyramide derivative 67

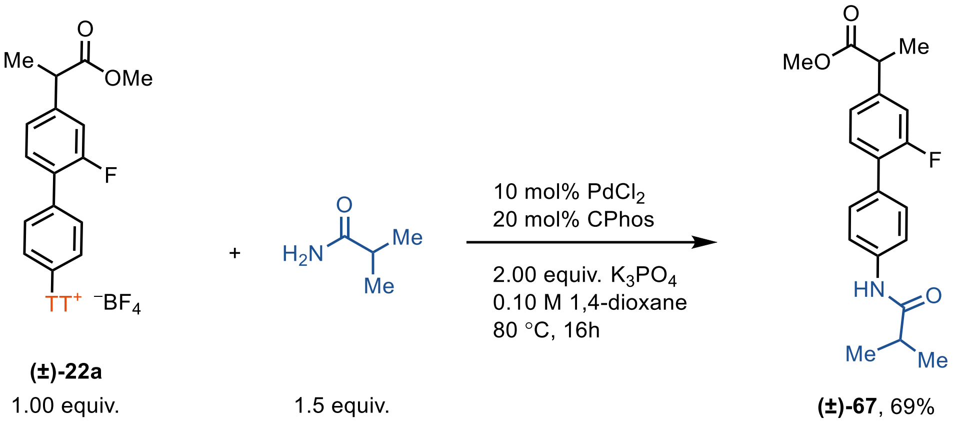

(±)-22a 1.00 equiv. 1.5 equiv. **(±)-67**, 69%

A 4-mL borosilicate vial containing a Teflon-coated magnetic stirring bar was used. (±)-Flurbiprofen methyl ester-derived thianthrenium salt **22a** (56.0 mg, 0.100 mmol, 1.00 equiv.), $PdCl_2$ (1.8 mg, 10 µmol, 10 mol%), CPhos (8.7 mg, 20 µmol, 20 mol%), K_3PO_4 (42.5 mg, 0.200 mmol, 2.00 equiv.) and isobutyramide (13.1 mg, 0.150 mmol, 1.50 equiv.) were introduced into the vial. The vial was introduced into a glovebox, 1,4-dioxane (1.5 mL, *c* = 0.10 M) was added using a syringe. The reaction vial was taken out of the glovebox. The mixture was stirred (600 rpm) at 80 °C. After 16 h, the mixture was diluted with EtOAc (2 mL) washed with water (2 mL), and dried over Na_2SO_4. Upon filtration, the organic layer was concentrated and purified by flash column chromatography (hexanes / EtOAc = 1:30 (v/v)) on silica gel to afford the desired product **(±)-67** (23.6 mg, 69%).

R_f = 0.52 (hexanes / EtOAc = 10:1 (v/v))

NMR Spectroscopy:

^{1}H NMR (600 MHz, $CDCl_3$, 23 °C, δ): 7.61 (d, *J* = 8.7 Hz, 2H), 7.49 (dd, *J* = 8.8, 1.6 Hz, 2H), 7.41 – 7.31 (m, 2H), 7.17 – 7.01 (m, 2H), 3.75 (q, *J* = 7.2 Hz, 1H), 3.69 (s, 3H), 2.53 (p, *J* = 6.8 Hz, 1H), 1.53 (d, *J* = 7.2 Hz, 3H), 1.26 (d, *J* = 6.9 Hz, 6H).

^{13}C NMR (151 MHz, $CDCl_3$, 23 °C, δ): 175.5, 174.6, 159.8 (d, *J* = 248.0 Hz), 141.7 (d, *J* = 7.6 Hz), 137.8, 131.3, 130.7 (d, *J* = 3.8 Hz), 129.6 (d, *J* = 3.1 Hz), 127.4 (d, *J* = 13.4 Hz), 123.7 (d, *J* = 3.5 Hz), 119.8, 115.4 (d, *J* = 23.5 Hz), 52.4, 45.0 (d, *J* = 1.5 Hz), 36.9, 27.0, 19.8, 18.5.

^{19}F NMR (565 MHz, $CDCl_3$, 23 °C, δ): –117.5

HRMS-ESI(m/z) calc'd for $C_{20}H_{23}FNO_3$ $[M+H]^+$, 344.1657; found, 344.1661; deviation: –1.3 ppm.

Unsuccessful substrate for C–N cross-coupling reactions of aryl thianthrenium salts

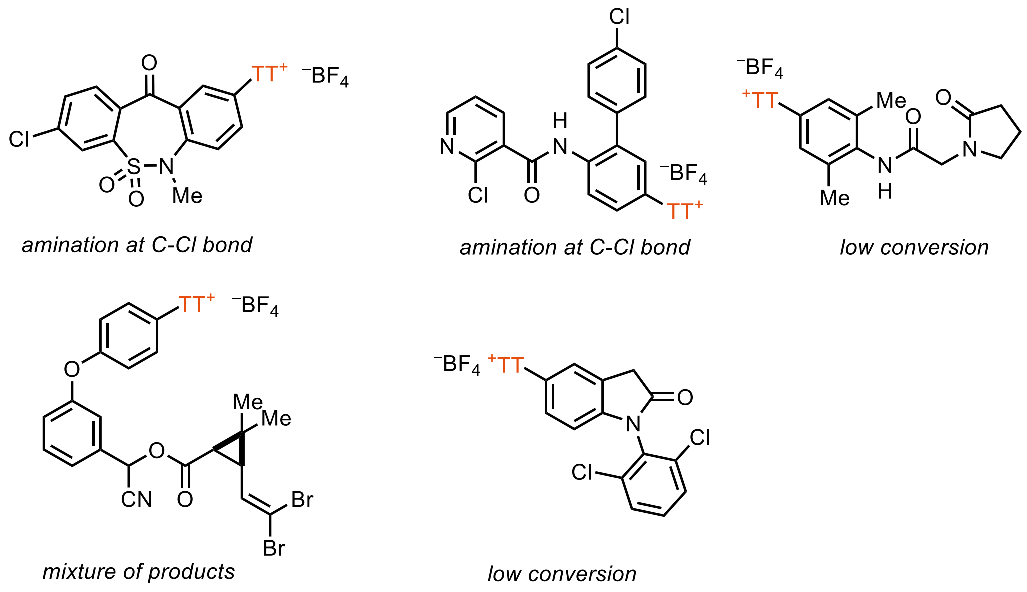

Fig S7: Unsuccessful substrates for amination reaction. Reaction were carried out on a 0.05 mmol scale. MeCN:H_2O (1:1, 0.1 M), DMF (0.1 M), 1,4-dioxane (0.1 M) were tested as solvents.

Carbonylation of aryl thianthrenium salts

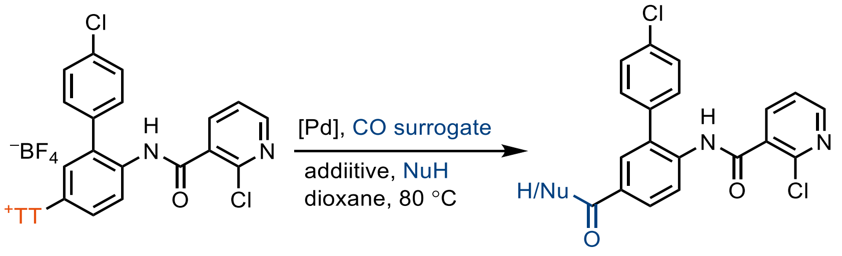

Table S3 Screening of CO surrogates for carbonylation of ary thianthrenium salts:

Entry	CO surrogate	Additive	Nucleophile	Yield (%)
**1**	$CHCl_3$	KOH	Morpholine	0
**2**	$Mo(CO)_6$	DBU	Me_2NH	n.d.
**3**	HCO_2Me	NaOMe	None	0
**4**	DMF	$POCl_3$	None	0
**5**	$Fe(CO)_5$	None	morpholine	n.d.
**6**	Phenyl formate	Et_3N	None	n.d.
**7**	Methyl formate	Et_3N	None	n.d.
**8**[b]	Trichlorophenyl formate	Et_3N	None	98

Reactions were carried out on a 0.100 mmol scale. Reactions were analyzed by LCMS and ^1H NMR. [b]Pyriproxyphen-derived thinathrenium salt **(±)-19a** was used. The yields were determined by ^1H NMR using 1,3,5-trimethoxybenzene as an internal standard.

General procedure for carbonylation of aryl thianthrenium salts

A 4-mL borosilicate vial containing a Teflon-coated magnetic stirring bar was used. Aryl thianthrenium salt (0.100 mmol, 1.00 equiv.), $PdOAc_2$ (1.4 mg, 6.0 µmol, 6.0 mol%), Xantphos (6.9 mg, 12 µmol, 12 mol%), 2,4,6-trichlorophenylformate (27.1 mg, 0.120 mmol, 1.20 equiv.) were introduced into the vial. The vial was introduced into a nitrogen-filled glovebox, 1,4-dioxane (1.0 mL, *c* = 0.10 M) was added using a syringe. The reaction vial was taken out of the glovebox, triethylamine (56 µL, 20 mg, 0.40 mmol, 4.0 equiv.), was added followed by addition of nucleophile (0.15 mmol, 1.5 equiv.). The mixture was stirred (600 rpm) at 80 °C. After 16 h, the mixture was diluted with EtOAc (2 mL) washed with water (2 mL), and dried over Na_2SO_4. Upon filtration, the organic layer was concentrated under reduced pressure and purified by flash column chromatography on silica gel.

(±)-Pyriproxyphen dimethylamine carboamide derivative 68

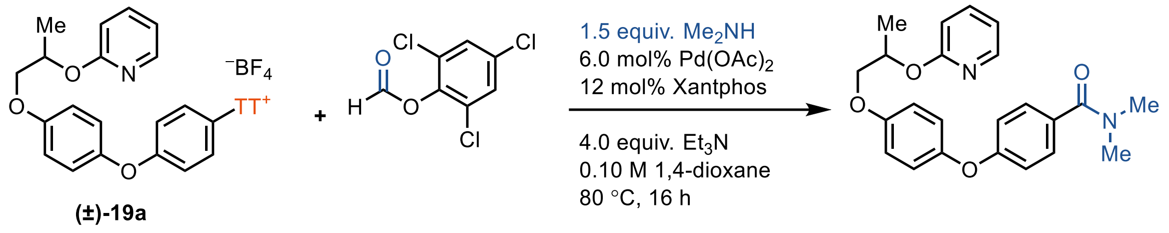

(±)-19a
1.00 equiv. 1.20 equiv. **(±)-68**, 76%

A 4-mL borosilicate vial containing a Teflon-coated magnetic stirring bar was used. (±)-Pyriproxyphen-derived thianthrenium salt **19a** (62.3 mg, 0.100 mmol, 1.00 equiv.), $PdOAc_2$ (1.4 mg, 6.0 µmol, 6.0 mol%), Xantphos (6.9 mg, 12 µmol, 12 mol%), 2,4,6-trichlorophenylformate (27.1 mg, 0.120 mmol, 1.20 equiv.) were introduced into the vial. The vial was introduced into a glovebox, 1,4-dioxane (1.0 mL, *c* = 0.10 M) was added using a syringe. The reaction vial was taken out of the glovebox, triethylamine (56 µL, 20 mg, 0.40 mmol, 4.0 equiv.), was added followed by addition of dimethylamine (2.0 M in THF, 75 µL, 51 mg, 0.15 mmol, 1.5 equiv.). The mixture was stirred (600 rpm) at 80 °C. After 16 h, the mixture was diluted with EtOAc (2 mL) washed with water (2 mL), and dried over Na_2SO_4. Upon filtration, the organic layer was concentrated under reduced pressure and purified by flash column chromatography (hexanes / EtOAc = 20:1 to 10:1 to 1:1 (v/v)) on silica gel to afford the desired product **(±)-68** (30.0 mg, 76%)

R_f = 0.24 (hexanes / EtOAc = 1:1 (v/v))

NMR Spectroscopy:

^{1}H NMR (500 MHz, $CDCl_3$, 23 °C, δ): 8.14 (dd, *J* = 5.1, 2.1 Hz, 1H), 7.56 (ddd, *J* = 9.2, 7.1, 2.1 Hz, 1H), 7.40 – 7.34 (m, 2H), 7.00 – 6.89 (m, 6H), 6.89 – 6.82 (m, 1H), 6.74 (d, *J* = 8.4 Hz, 1H), 5.58 (dt, *J* = 6.4, 5.1 Hz, 1H), 4.19 (dd, *J* = 9.9, 5.3 Hz, 1H), 4.08 (dd, *J* = 9.9, 4.8 Hz, 1H), 3.08 (bs, 3H), 3.08 (bs, 3H), 1.48 (d, *J* = 6.4 Hz, 3H).

^{13}C NMR (126 MHz, $CDCl_3$, 23 °C, δ): 171.4, 163.3, 159.9, 155.8, 149.6, 146.9, 138.8, 130.2, 129.2, 121.3, 117.0, 116.9, 116.0, 111.8, 71.2, 69.4, 17.1.

HRMS-ESI(m/z) calc'd for $C_{23}H_{25}N_2O_4$ $[M+H]^+$, 393.1809; found, 393.1812; deviation: –0.8 ppm.

Procedure by using the Schlenk line: A 4-mL borosilicate vial containing a Teflon-coated magnetic stirring bar was used. (±)-Pyriproxyphen-derived thianthrenium salt **19a** (62.3 mg, 0.100 mmol, 1.00 equiv.), $PdOAc_2$ (1.4 mg, 6.0 µmol, 6.0 mol%), Xantphos (6.9 mg, 12 µmol, 12 mol%), 2,4,6-trichlorophenylformate (27.1 mg, 0.120 mmol, 1.20 equiv.) were introduced into the vial. After sealing with a Schlenk-line adapter, the vial was evacuated and backfilled with argon three times using a Schlenk line. Then, 1,4-dioxane (1.0 mL, *c* = 0.10 M) was added using a syringe under Ar flow. The vial was sealed with a septum cap and triethylamine (56 µL, 20 mg, 0.40 mmol, 4.0 equiv.), was added followed by addition of dimethyamine (2.0 M in THF, 75 µL, 51 mg, 0.15 mmol, 1.5 equiv.). The mixture was stirred (600 rpm) at 80 °C. After 16 h, the mixture was cooled to 25 °C. 1,3,5-trimethoxybenzene (16.8 mg, 0.100 mmol, 1.00 equiv.) was added into the reaction mixture as an internal standard, followed by EtOAc (2.0 mL) and H_2O (2 mL). Layers were separated and an aliquot (ca. 1 mL) from the EtOAc phase was taken and the concentrated by rotary evaporation. The residue was dissolved in $CDCl_3$ (0.6 mL), and the ^1H NMR was recorded. The yield (60%) was determined from the ^1H NMR spectrum by integrating the signal of the internal standard at 6.11 ppm (s, 3H) and the product **68** at 3.08 ppm (6H, bs).

(±)-Pyriproxyphen methylamine carboamide derivative 69

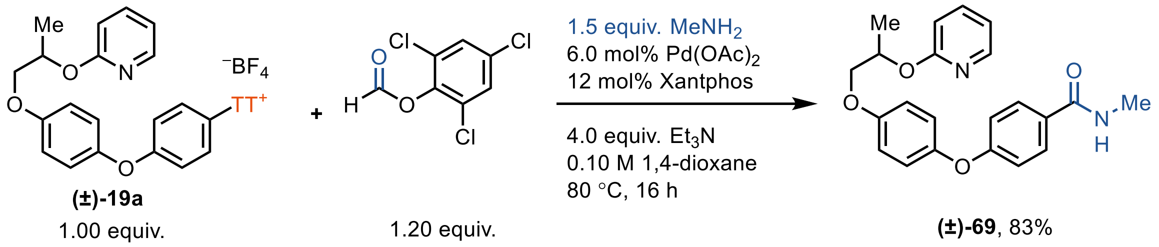

A 4-mL borosilicate vial containing a Teflon-coated magnetic stirring bar was used. (±)-Pyriproxyphen-derived thianthrenium salt **19a** (62.3 mg, 0.100 mmol, 1.00 equiv.), $PdOAc_2$ (1.4 mg, 6.0 µmol, 6.0 mol%), Xantphos (6.9 mg, 12 µmol, 12 mol%), 2,4,6-trichlorophenylformate (27.1 mg, 0.120 mmol, 1.20 equiv.) were introduced into the vial. The vial was introduced into a glovebox, 1,4-dioxane (1.0 mL, *c* = 0.10 M) was added using a syringe. The reaction vial was taken out of the glovebox, triethylamine (56 µL, 20 mg, 0.40 mmol, 4.0 equiv.), was added followed by addition of methylamine (2.0 M in THF, 75 µL, 53 mg, 0.15 mmol, 1.5 equiv.). The mixture was stirred (600 rpm) at 80 °C. After 16 h, the mixture was diluted with EtOAc (2 mL) washed with water (2 mL), and dried over Na_2SO_4. Upon filtration, the organic layer was concentrated under reduced pressure and purified by flash column chromatography (hexanes / EtOAc = 20:1 to 10:1 to 1:1 (v/v)) on silica gel to afford the desired product **(±)-69** (30.0 mg, 83%)

R*f* = 0.19 (hexanes / EtOAc = 1:1 (v/v))

NMR Spectroscopy:

^{1}H NMR (600 MHz, $CDCl_3$, 23 °C, δ): 8.15 (ddd, *J* = 5.0, 2.0, 0.8 Hz, 1H), 7.72 – 7.67 (m, 2H), 7.57 (ddd, *J* = 8.3, 7.1, 2.0 Hz, 1H), 6.99 – 6.90 (m, 6H), 6.86 (ddd, *J* = 7.1, 5.0, 1.0 Hz, 1H), 6.74 (dt, *J* = 8.3, 0.9 Hz, 1H), 6.06 (bs, 1H), 5.63 – 5.54 (m, 1H), 4.20 (dd, *J* = 9.9, 5.3 Hz, 1H), 4.08 (dd, *J* = 9.9, 4.8 Hz, 1H), 3.00 (d, *J* = 4.8 Hz, 3H), 1.48 (d, *J* = 6.4 Hz, 3H).

^{13}C NMR (151 MHz, $CDCl_3$, 23 ºC, δ): 167.7, 163.3, 161.5, 156.0, 149.2, 146.9, 138.9, 128.8, 128.6, 121.5, 116.9, 116.9, 116.1, 111.8, 71.2, 69.4, 29.8, 27.0, 17.1.

HRMS-ESI(m/z) calc'd for $C_{22}H_{23}N_2O_4$ $[M+H]^+$, 379.1652; found, 379.1657; deviation: –1.2 ppm.

(±)-Pyriproxyphen carboxylic acid derivative 70

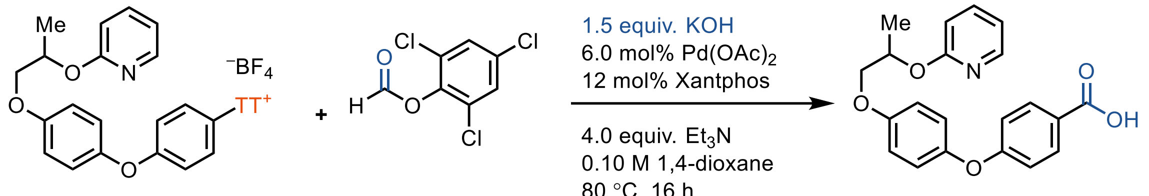

(±)-19a 1.00 equiv. 1.20 equiv. **(±)-70**, 95%

A 4-mL borosilicate vial containing a Teflon-coated magnetic stirring bar was used. (±)-Pyriproxyphen-derived thianthrenium salt **19a** (124.7 mg, 0.200 mmol, 1.00 equiv.), $PdOAc_2$ (2.8 mg, 12 µmol, 6.0 mol%), Xantphos (13.9 mg, 24.0 µmol, 12.0 mol%), 2,4,6-trichlorophenylformate (45.1 mg, 0.240 mmol, 1.20 equiv.) were introduced into the vial. The vial was introduced into a glovebox, 1,4-dioxane (2.0 mL, *c* = 0.10 M) was added using a syringe. The reaction vial was taken out of the glovebox, triethylamine (112 µL, 81 mg, 0.800 mmol, 4.00 equiv.), was added via septum followed by addition of KOH (2.5 M in water, 1.0 mL, 17 mg, 0.3 mmol, 1.5 equiv.),. The mixture was stirred (600 rpm) at 80 °C. After 16 h, the mixture was diluted with EtOAc (2 mL) washed with water (2 mL), and dried over Na_2SO_4. Upon filtration, the organic layer was concentrated under reduced pressure and purified by flash column chromatography (EtOAc / hexanes, 1:10 to 1:1 (v/v)) on silica gel to afford the desired product **(±)-70** (69.7 mg, 95%)

R*f* = 0.75 (EtOAc only).

NMR Spectroscopy:

^1H NMR (500 MHz, $CDCl_3$, 23 ºC, δ): 8.19 – 8.13 (m, 1H), 8.07 – 7.99 (m, 2H), 7.61 – 7.55 (m, 1H), 7.02 – 6.92 (m, 6H), 6.90 – 6.84 (m, 1H), 6.75 (d, *J* = 8.4 Hz, 1H), 5.65 – 5.55 (m, 1H), 4.21 (dd, *J* = 9.9, 5.3 Hz, 1H), 4.10 (dd, *J* = 9.9, 4.8 Hz, 1H), 1.49 (d, *J* = 6.4 Hz, 3H).

^{13}C NMR (126 MHz, $CDCl_3$, 23 ºC, δ): 171.1, 163.7, 163.3, 156.2, 148.7, 146.9, 139.0, 132.5, 123.0, 121.8, 117.0, 116.5, 116.2, 111.9, 71.2, 69.4, 17.2.

HRMS-ESI(m/z) calc'd for $C_{21}H_{20}NO_5$ $[M+H]^+$, 366.1336; found, 366.1336; deviation: +0.5 ppm.

(±)-Pyriproxyphen ispropylamine carboamide derivative 71

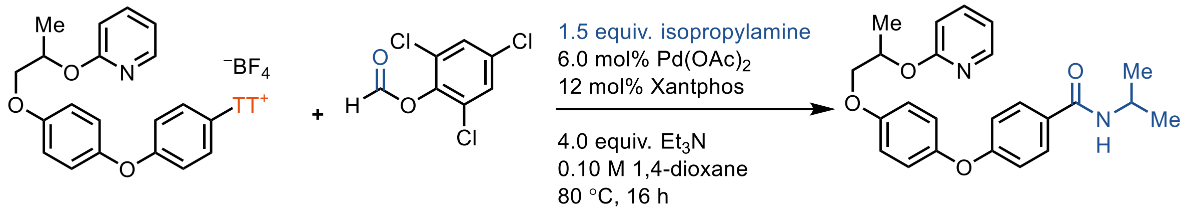

(±)-19a 1.00 equiv. 1.20 equiv. **(±)-71**, 80%

A 4-mL borosilicate vial containing a Teflon-coated magnetic stirring bar was used. (±)-Pyriproxyphen-derived thianthrenium salt **19a** (62.4 mg, 0.100 mmol, 1.00 equiv.), PdOAc$_2$ (1.4 mg, 6.0 µmol, 6.0 mol%), Xantphos (6.9 mg, 12 µmol, 12 mol%), 2,4,6-trichlorophenylformate (27.1 mg, 0.120 mmol, 1.20 equiv.) were introduced into the vial. The vial was introduced into a glovebox, 1,4-dioxane (1.0 mL, *c* = 0.10 M) was added using a syringe. The reaction vial was taken out of the glovebox, triethylamine (56 µL, 20 mg, 0.40 mmol, 4.0 equiv.), was added followed by addition of ispropylamine (10 µL, 7.1 mg, 0.12 mmol, 1.2 equiv.). The mixture was stirred (600 rpm) at 80 °C. After 16 h, the mixture was diluted with EtOAc (2 mL) washed with water (2 mL), and dried over Na$_2$SO$_4$. Upon filtration, the organic layer was concentrated under reduced pressure and purified by flash column chromatography (hexanes / EtOAc = 10:1 to 2:1 (v/v)) on silica gel to afford the desired product **(±)-71** (32.6 mg, 80%)

R*f* = 0.18 (hexanes / EtOAc = 5:1 (v/v)).

NMR Spectroscopy:

^{1}H NMR (500 MHz, CDCl$_3$, 23 ºC, δ): 8.15 (d, *J* = 5.2 Hz, 1H), 7.69 (d, *J* = 8.4 Hz, 2H), 7.57 (t, *J* = 7.6 Hz, 1H), 7.02 – 6.66 (m, 8H), 5.83 (d, *J* = 7.9 Hz, 1H), 5.59 (d, *J* = 5.2 Hz, 1H), 4.27 (q, *J* = 6.6 Hz, 1H), 4.20 (dd, *J* = 9.9, 4.5 Hz, 1H), 1.49 (d, *J* = 5.6 Hz, 3H), 1.25 (d, *J* = 6.1 Hz, 6H).

^{13}C NMR (126 MHz, CDCl$_3$, 23 ºC, δ): 166.2, 163.3, 161.4, 155.9, 149.3, 146.9, 138.9, 128.8, 121.4, 117.0, 117.0, 116.0, 111.9, 71.2, 69.4, 42.0, 23.1, 17.2.

HRMS-ESI(m/z) calc'd for $C_{24}H_{27}O_4N_2$ $[M+H]^+$, 407.1939; found, 407.1963; deviation: +0.5 ppm.

(±)-Flurbiprofen methyl ester pyrrolidine carboamide derivative 72

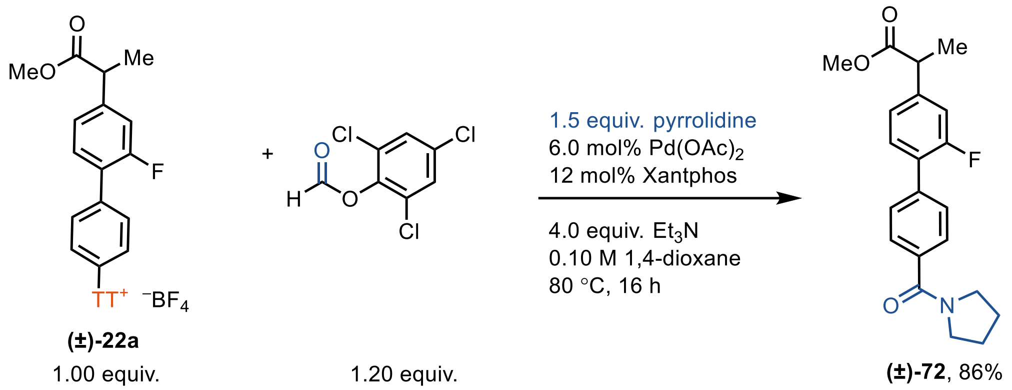

(±)-22a 1.00 equiv. | 1.20 equiv. | **(±)-72**, 86%

A 4-mL borosilicate vial containing a Teflon-coated magnetic stirring bar was used. (±)-Flurbiprofen methyl ester-derived thianthrenium salt **22a** (56.0 mg, 0.100 mmol, 1.00 equiv.), PdOAc$_2$ (1.4 mg, 6.0 µmol, 6.0 mol%), Xantphos (6.9 mg, 12 µmol, 12 mol%), 2,4,6-trichlorophenylformate (27.1 mg, 0.120 mmol, 1.20 equiv.) were introduced into the vial. The vial was introduced into a glovebox, 1,4-dioxane (1.0 mL, *c* = 0.10 M) was added using a syringe. The reaction vial was taken out of the glovebox, triethylamine (56 µL, 20 mg, 0.40 mmol, 4.0 equiv.), was added via septum followed by addition of pyrrolidine (10 µL, 8.5 mg, 0.15 mmol, 1.5 equiv.). The mixture was stirred (600 rpm) at 80 °C. After 16 h, the mixture was diluted with EtOAc (2 mL), washed with water (2mL), and dried over Na$_2$SO$_4$. Upon filtration, the organic layer was concentrated under reduced pressure and purified by flash column

chromatography (hexanes / EtOAc = 10:1 to 1:1 (v/v)) on silica gel to afford the desired product **(±)-72** (30.5 mg, 86%)

R*f* = 0.24 (hexanes / EtOAc = 1:1 (v/v)).

NMR Spectroscopy:

^{1}H NMR (600 MHz, $CDCl_3$, 23 °C, δ): 7.63 – 7.49 (m, 4H), 7.38 (t, *J* = 8.0 Hz, 1H), 7.19 – 7.10 (m, 2H), 3.76 (q, *J* = 7.2 Hz, 1H), 3.69 (s, 3H), 3.66 (t, *J* = 7.0 Hz, 2H), 3.48 (t, *J* = 6.7 Hz, 2H), 1.97 (p, *J* = 7.1 Hz, 2H), 1.89 (p, *J* = 6.4 Hz, 2H), 1.53 (d, *J* = 7.2 Hz, 6H).

^{13}C NMR (151 MHz, $CDCl_3$, 23 °C, δ): 174.5, 169.5, 159.8 (d, *J* = 248.9 Hz), 142.5 (d, *J* = 7.6 Hz), 137.1, 136.5, 130.8 (d, *J* = 3.8 Hz), 128.9 (d, *J* = 3.2 Hz), 127.4, 127.2 (d, *J* = 13.4 Hz), 115.5 (d, *J* = 23.5 Hz), 52.4, 49.8, 46.4, 46.0, 45.0, 24.6, 18.5, 8.7.

^{19}F NMR (565 MHz, $CDCl_3$, 23 °C, δ): –117.3 (bs)

HRMS-FIA(m/z) calc'd for $C_{21}H_{23}FNO_3$ $[M+H]^+$, 356.1657; found, 356.1652; deviation: +1.2 ppm.

(±)-Fenoprofen methyl ester pyrrolidine carboamide derivative 73

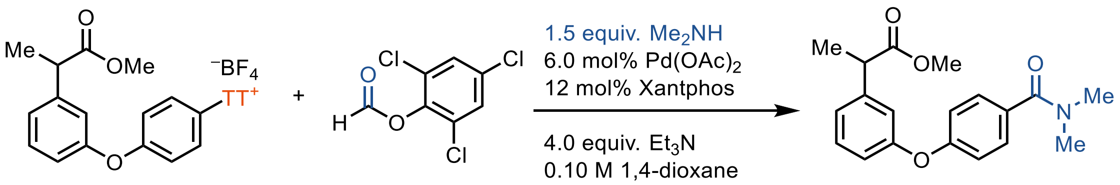

(±)-10a 1.00 equiv. 1.20 equiv. **(±)-73**, 44%

A 4-mL borosilicate vial containing a Teflon-coated magnetic stirring bar was used. (±)-Fenoprofen methyl ester-derived thianthrenium salt **10a** (140 mg, 0.250 mmol, 1.00 equiv.), $PdOAc_2$ (3.4 mg, 15 µmol, 6.0 mol%), Xantphos (6.9 mg, 30 µmol, 12 mol%), 2,4,6-trichlorophenylformate (67.6 mg, 0.300 mmol, 1.20 equiv.) were introduced into the vial. The vial was introduced into a glovebox, 1,4-dioxane (2.5 mL, *c* = 0.10 M) was added using a syringe. The reaction vial was taken out of the glovebox, triethylamine (139 µL, 101 mg, 1.00 mmol, 4.00 equiv.), was added followed by addition of dimethylamine (2.0 M in THF, 0.15 mL, 0.10 g, 0.30 mmol, 1.5 equiv.). The mixture was stirred (600 rpm) at 80 °C. After 16 h, the mixture was diluted with EtOAc (4 mL), washed with water (4 mL), and dried over Na_2SO_4. Upon filtration, the organic layer was concentrated under reduced pressure and purified by flash column chromatography (hexanes / EtOAc = 10:1 to 1:1 (v/v)) on silica gel to afford the desired product **(±)-73** (35.6 mg, 44%)

R*f* = 0.19 (hexanes / EtOAc = 1:1 (v/v))

NMR Spectroscopy:

^{1}H NMR (600 MHz, $CDCl_3$, 23 °C, δ): 7.45 – 7.36 (m, 2H), 7.30 (t, *J* = 7.9 Hz, 1H), 7.11 – 7.04 (m, 1H), 7.03 – 6.96 (m, 3H), 6.90 (ddd, *J* = 8.2, 2.5, 1.0 Hz, 1H), 3.71 (q, *J* = 7.2 Hz, 1H), 3.67 (s, 3H), 3.10 (bs, 3H), 3.03 (bs, 3H), 1.49 (d, *J* = 7.2 Hz, 3H)..

^{13}C NMR (151 MHz, CDCl, 23 °C, δ): 174.7, 171.3, 158.6, 156.7, 142.8, 131.0, 130.1, 129.3,

128.2, 123.1, 118.8, 118.0, 118.10, 52.3, 45.4, 39.9, 35.7, 18.6.

HRMS-GC-EI(m/z) calc'd for $C_{19}H_{21}NO_4$ $[M+H]^+$, 327.1465; found, 327.1467; deviation: –0.5 ppm.

(±)-Fenoprofen methyl ester morpholine carboamide derivative 74

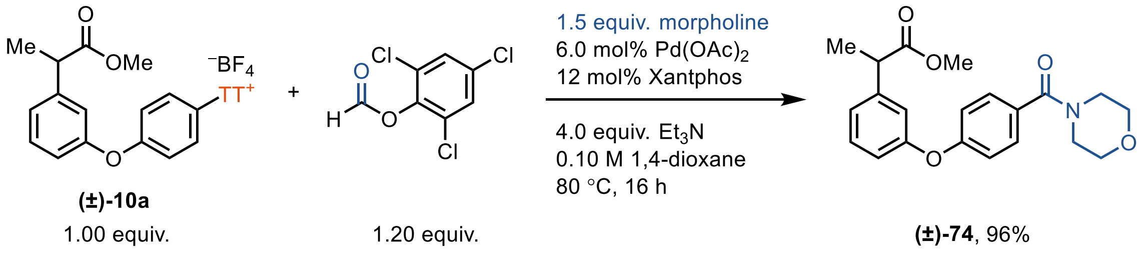

A 4-mL borosilicate vial containing a Teflon-coated magnetic stirring bar was used. (±)-Fenoprofen methyl ester-derived thianthrenium salt **10a** (55.8 mg, 0.100 mmol, 1.00 equiv.), $PdOAc_2$ (1.4 mg, 6.0 µmol, 6.0 mol%), Xantphos (6.9 mg, 12 µmol, 12 mol%), 2,4,6-trichlorophenylformate (27.1 mg, 0.120 mmol, 1.20 equiv.) were introduced into the vial. The vial was introduced into a glovebox, 1,4-dioxane (1.0 mL, *c* = 0.10 M) was added using a syringe. The reaction vial was taken out of the glovebox, triethylamine (56 µL, 20 mg, 0.40 mmol, 4.0 equiv.), was added by using a microsyringe via septum followed by addition of morpholine (11 µL, 11 mg, 0.12 mmol, 1.2 equiv.). The mixture was stirred (600 rpm) at 80 °C. After 16 h, the mixture was diluted with EtOAc (2 mL), washed with water (2 mL), and dried over Na_2SO_4. Upon filtration, the organic layer was concentrated under reduced pressure and purified by flash column chromatography (hexanes / EtOAc = 10:1 to 1:1 (v/v)) on silica gel to afford the desired product **(±)-74** (35.4 mg, 96%)

R*f* = 0.35 (hexanes / EtOAc = 1:1 (v/v))

NMR Spectroscopy:

^{1}H NMR (500 MHz, $CDCl_3$, 23 °C, δ): 7.40 (m, 3H), 7.30 (t, *J* = 8.0 Hz, 1H), 7.10 – 7.07 (m, 1H), 7.02 – 6.98 (m, 3H), 3.74 – 3.63 (m, 12H), 1.49 (d, *J* = 7.2 Hz, 3H).

^{13}C NMR (151 MHz, $CDCl_3$, 23 °C, δ): 174.7, 170.2, 159.0, 156.4, 142.89, 130.2, 130.0, 129.8, 129.4, 128.7, 127.2, 123.3, 119.0, 118.2, 67.0, 52.3, 45.4, 18.6.

HRMS-ESI(m/z) calc'd for $C_{21}H_{23}NO_5Na$ $[M+Na]^+$, 392.1468; found, 392.1469; deviation: –0.3 ppm.

Tianeptine intermediate pentylamine carboamide derivative 75

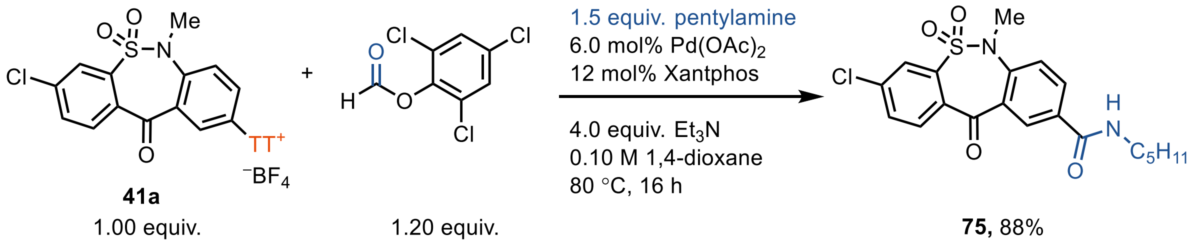

A 4-mL borosilicate vial containing a Teflon-coated magnetic stirring bar was used. Tianeptine intermediate-derived thianthrenium salt **41a** (60.9 mg, 0.100 mmol, 1.00 equiv.), $PdOAc_2$ (1.4 mg, 6.0

μmol, 6.0 mol%), Xantphos (6.9 mg, 12 μmol, 12 mol%), 2,4,6-trichlorophenylformate (27.1 mg, 0.120 mmol, 1.20 equiv.) were introduced into the vial. The vial was introduced into a glovebox, 1,4-dioxane (1.0 mL, *c* = 0.10 M) was added using a syringe. The reaction vial was taken out of the glovebox, triethylamine (56 µL, 20 mg, 0.40 mmol, 4.0 equiv.), was added followed by addition of pentylamine (14 µL, 11 mg, 0.15 mmol, 1.5 equiv.). The mixture was stirred (600 rpm) at 80 °C. After 16 h, the mixture was diluted with EtOAc (2 mL), washed with water (2 mL), and dried over Na_2SO_4. Upon filtration, the organic layer was concentrated under reduced pressure and purified by flash column chromatography (hexanes / EtOAc = 10:1 to 1:1 (v/v)) on silica gel to afford the desired product **(±)-75** (37.2 mg, 88%)

R*f* = 0.52 (hexanes / EtOAc = 1:1 (v/v))

NMR Spectroscopy:

^{1}**H NMR** (600 MHz, $CDCl_3$, 23 ºC, δ): 8.55 (d, *J* = 2.2 Hz, 1H), 8.12 (dd, *J* = 8.4, 2.3 Hz, 1H), 7.93 (d, *J* = 2.1 Hz, 1H), 7.85 (d, *J* = 8.3 Hz, 1H), 7.68 (dd, *J* = 8.3, 2.1 Hz, 1H), 7.35 (d, *J* = 8.4 Hz, 1H), 6.50 (t, *J* = 5.7 Hz, 1H), 3.48 – 3.42 (m, 2H), 3.40 (s, 3H), 1.70 – 1.57 (m, 2H), 1.40 – 1.32 (m, 4H), 1.04 – 0.67 (m, 3H).

^{13}**C NMR** (151 MHz, $CDCl_3$, 23 ºC, δ): 189.4, 165.7, 143.6, 139.0, 138.4, 134.4, 134.0, 133.7, 133.2, 132.2, 129.7, 125.0, 124.0, 40.4, 38.7, 29.4, 29.3, 22.5, 14.1.

HRMS-ESI(m/z) calc'd for $C_{20}H_{21}N_2O_4SClNa$ $[M+Na]^+$, 443.0803; found, 443.0805; deviation: –0.4 ppm.

Additive screening for carboamidation of aryl thianthrenium salts

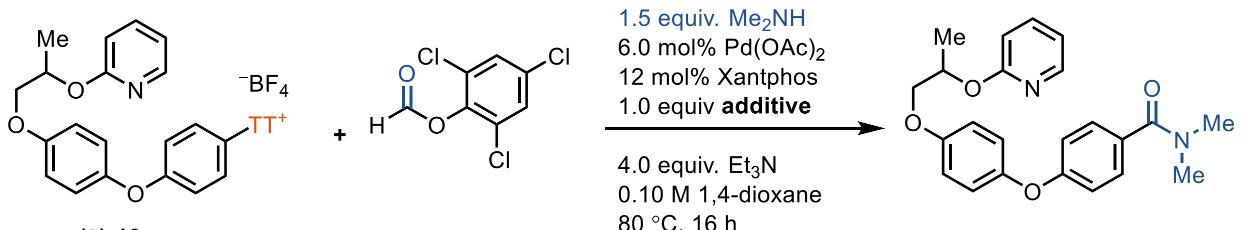

(±)-19a 1.00 equiv.   1.20 equiv.   **(±)-68**, 76%

Additive	Yield of 68 (%)	Remaing additive (%)	Additive	Yield of 68 (%)	Remaing additive (%)
None					
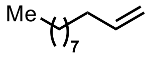	99 ✓	58 ✓	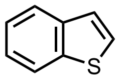	60 ✓	82 ✓
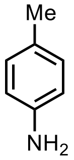	54 ✓	44 ✓	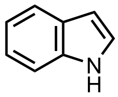	84 ✓	94 ✓
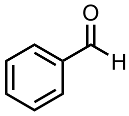	94 ✓	100 ✓	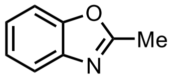	95 ✓	83 ✓
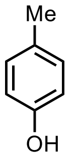	54 ✓	94 ✓	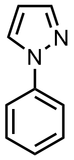	76 ✓	70 ✓
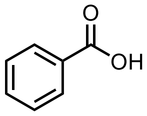	99 ✓	5% X	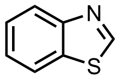	75 ✓	60 ✓

Fig S8: Additive screening for carboamidation of aryl thianthrenium salts. Reactions were carried out on a 0.10 mmol scale and yields were determined by ^{1}H NMR by using 1,3,5-trimethoxybenzene (1.0 equiv.) as an internal standard

Difluoromethylation of aryl thianthrenium salts

General procedure for the optimization of reaction conditions

To a 4-mL borosilicate vial containing a Teflon-coated magnetic stirring bar were added thianthrenium salt (0.100 mmol, 1.00 equiv.) and base (0.400 mmol, 4.00 equiv.) The vial was transferred into a nitrogen-filled glovebox. An orange stock solution of dry 1,4-dioxane (0.75 mL, *c* = 0.13 M) containing $Pd(OAc)_2$ (0.6 mg, 3 µmol, 3 mol%), Xantphos (2.9 mg, 5.0 µmol, 5.0 mol%), and hydroquinone (5.5 mg, 0.50 mmol, 0.50 equiv.) were added followed by the addition of ethyl chlorodifluoroacetate (38 µL, 0.30 mmol, 3.0 equiv.) via a Hamilton syringe. The vial was sealed with a Teflon-lined screw cap and parafilm, removed from the glovebox, and transferred to a heating block preheated to 80 °C where the reaction mixture was stirred (500 rpm) for 16 h. Then, the mixture was cooled to 25 °C, MTBE (1.5 mL) and water (1.5 mL) were added, and the resulting layers were separated using a pipette. The aqueous layer was extracted with MTBE (2 × 2 mL), the organic layers were combined, and all volatiles were evaporated under reduced pressure. The resulting residue was dissolved in $CDCl_3$ (0.4 mL), fluorobenzene (19 µL, 0.2 mmol) and CH_2Br_2 (7 µL, 0.1 mmol) were added as internal standards

and the mixture mixed thoroughly before an aliquot was taken and submitted for ^{19}F and ^{1}H NMR analysis. The yield of the difluoromethylated product was determined by relative integrals of the product (δ = –110.7 ppm) with respect to the signal of fluorobenzene (δ = –113.1 ppm).

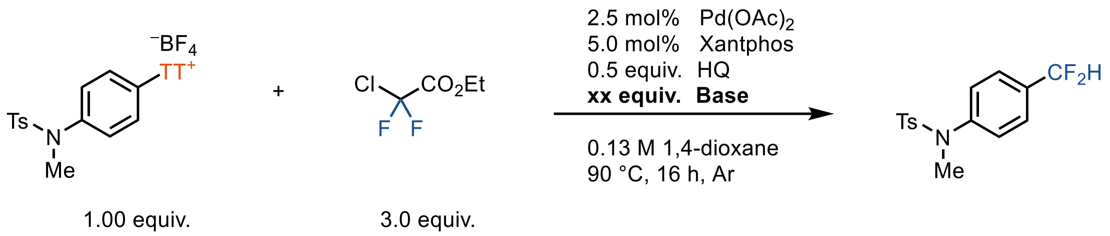

Table S4 Screening of bases:

Entry	Base	Yield
2	LiOH (3 equiv.)	<5%
3	^{t}BuOK (3 equiv.)	<5%
5	K_2CO_3 (3 equiv.)	21%
6	Cs_2CO_3 (3 equiv.)	<5%
**7**	**K_3PO_4 (3 equiv.)**	**55%**
8	KH_2PO_4 (3 equiv.)	<5%
9	Li_3PO_4 (3 equiv.)	<5%
10	Na_3PO_4 (3 equiv.)	<5%
11	Ag_3PO_4 (3 equiv.)	<5%
12	K_3PO_4 (4 equiv.)	50%
13	K_3PO_4 (2 equiv.)	37%
14	K_3PO_4 (1 equiv.)	11%

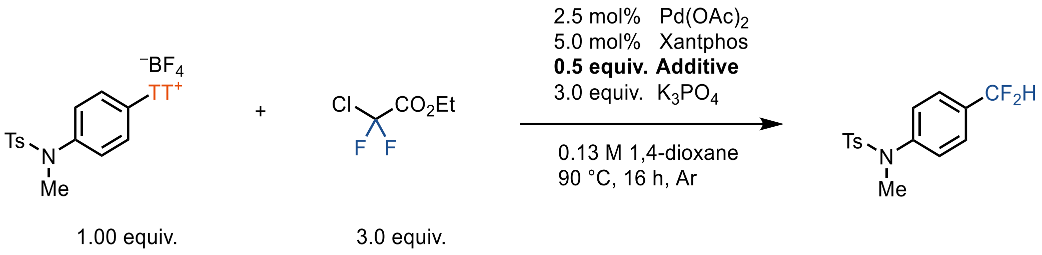

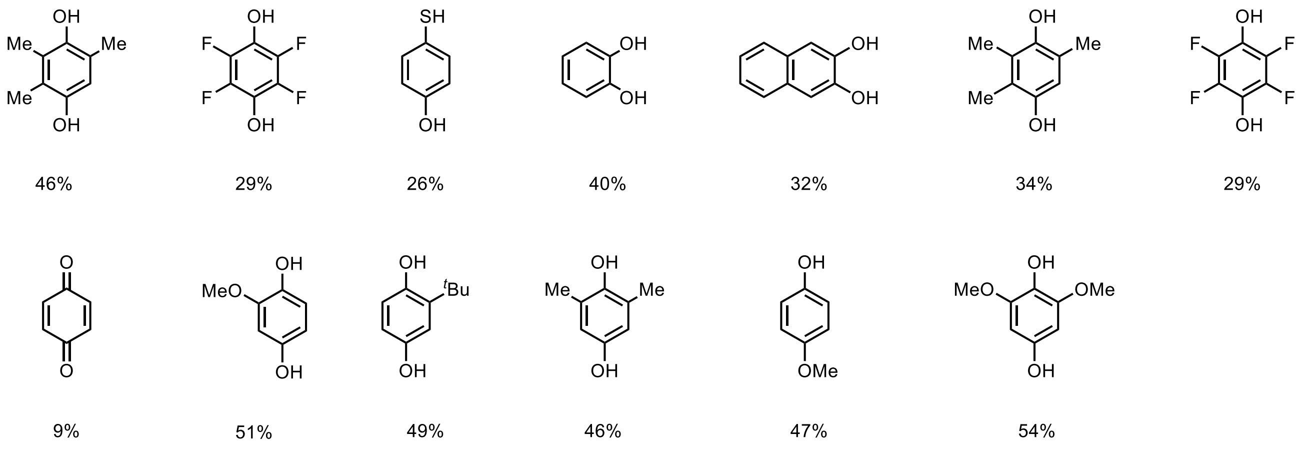

Fig S9: Screening of additives.

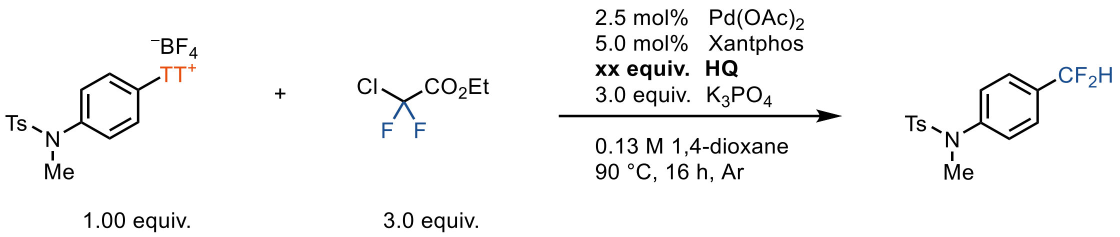

Table S5 Screening of hydroquinone amount:

Entry	HQ (x equiv.)	Yield
1	none	35%
2	HQ (1.0 equiv.)	56%
3	HQ (0.75 equiv.)	54%
4	HQ (1.5 equiv.)	35%

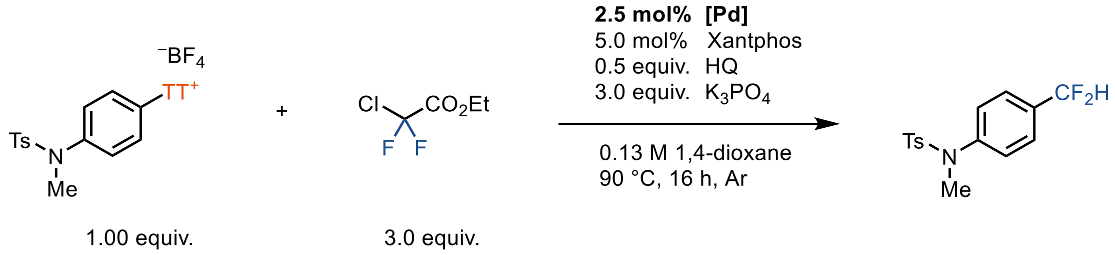

Table S6 Screening of Pd catalysts:

Entry	Pd-source	Yield
1	$PdSO_4$	6%
2	$Pd(OPiv)_2$	48%
3	$Pd(O_2CCF_3)_2$	31%

4	$Pd(O_2CCH_2CH_3)_2$	51%
5	Xantphos Pd G3 OMs (without additional Xantphos)	18%

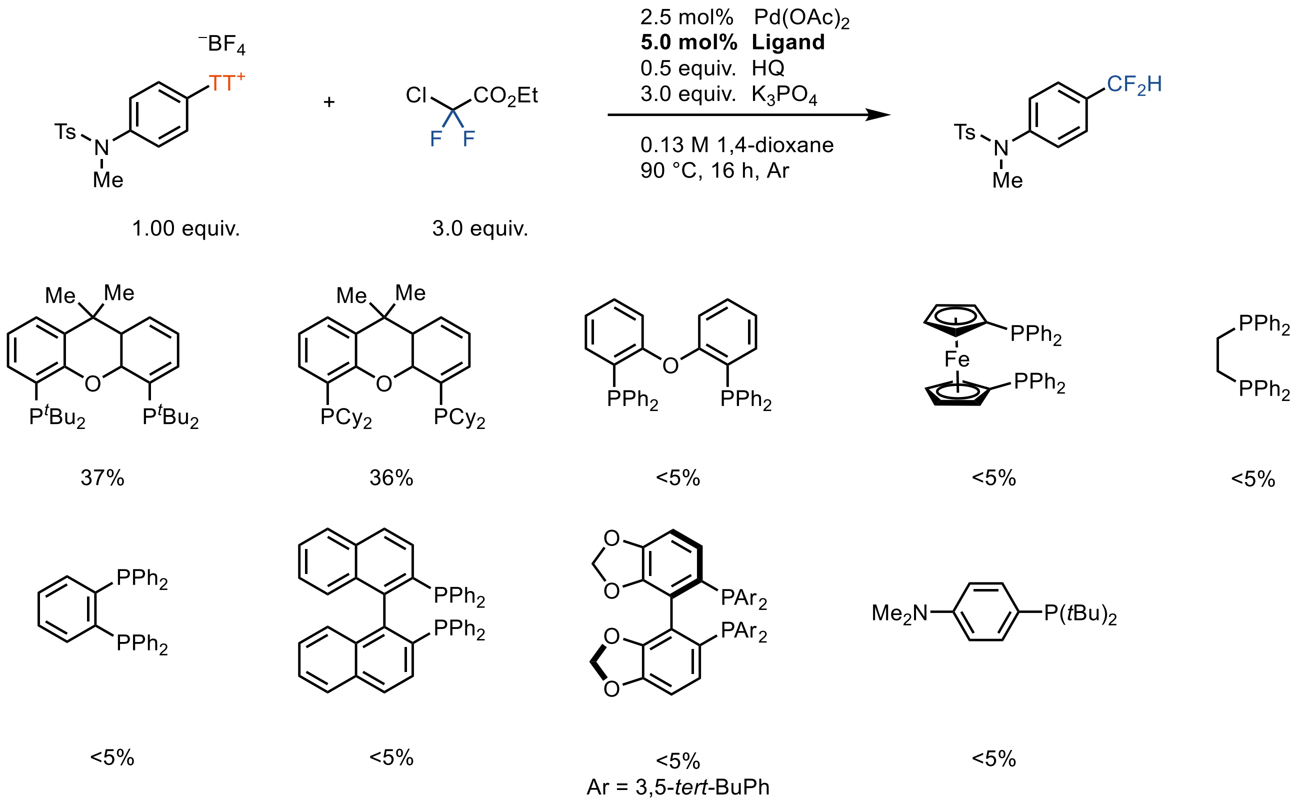

Fig S10: Screening of ligands

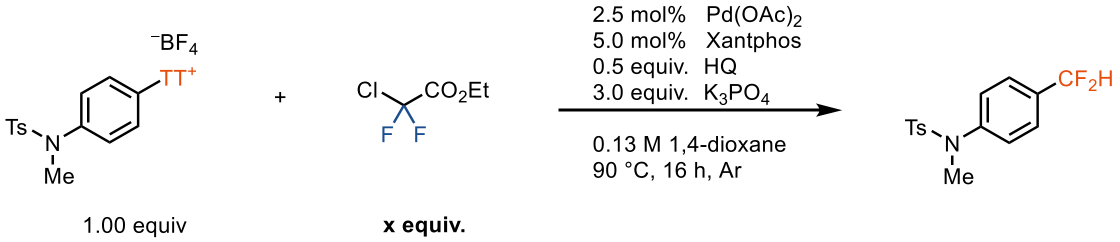

Table S7 Screening ethyl chlorodifluoroacetate amount:

Entry	$ClCF_2CO_2Et$ (x equiv.)	Yield
1	$ClCF_2CO_2Et$ (10 equiv.)	27%
2	$ClCF_2CO_2Et$ (5 equiv.)	42%
3	$ClCF_2CO_2Et$ (2 equiv.)	49%

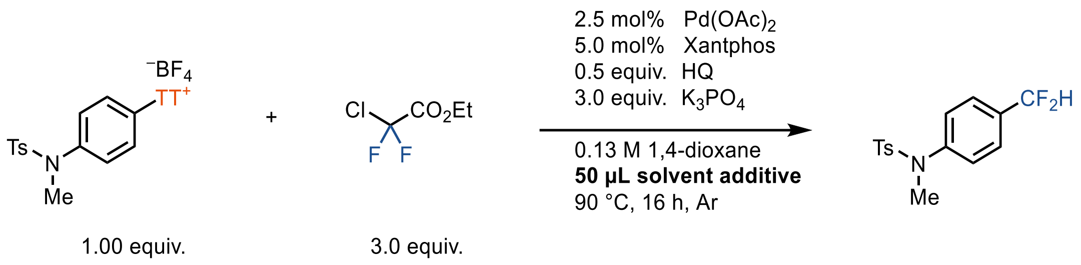

Table S8 Screening of solvent additives:

Entry	Solvent additive (50 µL)	Yield
1	DMSO	<5%
2	DMA	<5%
3	DMF	<5%
4	DMPU	<5%
5	NMP	<5%

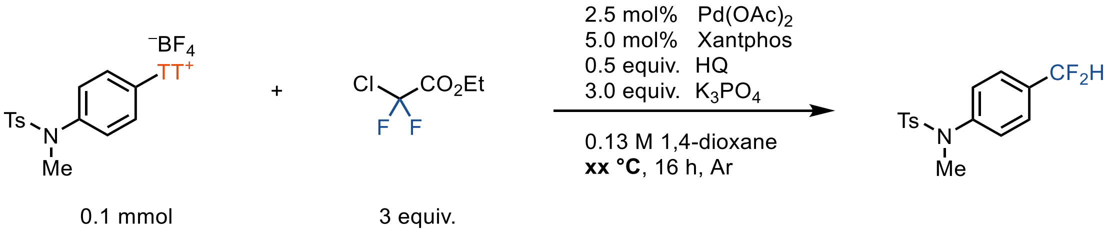

Table S9 Temperature screening:

Entry	Temperature	Yield
**1**	**80 °C**	**54%**
2	100 °C	52%
3	110 °C	25%

General procedure for difluoromethylation of aryl thianthrenium salts

To a 4-mL borosilicate vial containing a Teflon-coated magnetic stirring bar were added thianthrenium salt (1.00 equiv.) and K_3PO_4 (3.00 equiv.) The vial was transferred into a nitrogen-filled glovebox. An orange stock solution of dry 1,4-dioxane (*c* = 0.13 M) containing $Pd(OAc)_2$ (3 mol%), Xantphos (5.0 mol%), and hydroquinone (0.5 equiv.) were added followed by the addition of ethyl chlorodifluoroacetate (3.0 equiv.) via a Hamilton syringe. The vial was sealed with a Teflon-lined screw cap and parafilm, removed from the glovebox, and transferred to a heating block preheated to 80 °C, where the reaction mixture was stirred (500 rpm) for 16 h. Then, the mixture was cooled to 25 °C and transferred to a 20 mL borosilicate vial. MTBE and water were added and the resulting layers were

separated using a pipette. The aqueous layer was extracted with MTBE, the organic layers were combined, and all volatiles were evaporated under reduced pressure. The resulting residue was purified by flash column chromatography on silica gel to afford desired product.

Biphenylether difluormethyl derivative 76

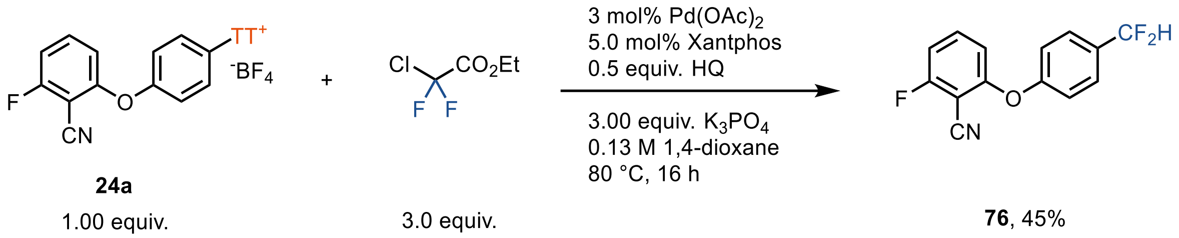

24a 1.00 equiv. 3.0 equiv. **76**, 45%

To a 4-mL borosilicate vial containing a Teflon-coated magnetic stirring bar were added biphenylether-derived thianthrenium salt **24a** (51.5 mg, 0.100 mmol, 1.00 equiv.) and K_3PO_4 (63.7 mg, 0.300 mmol, 3.00 equiv.) The vial was transferred into a nitrogen-filled glovebox. An orange stock solution of dry 1,4-dioxane (0.75 mL, *c* = 0.13 M) containing $Pd(OAc)_2$ (0.6 mg, 3 µmol, 3 mol%), Xantphos (2.9 mg, 15 µmol, 5.0 mol%), and hydroquinone (5.5 mg, 0.50 mmol, 0.50 equiv.) were added followed by the addition of ethyl chlorodifluoroacetate (38 µL, 0.30 mmol, 3.0 equiv.) via a Hamilton syringe. The vial was sealed with a Teflon-lined screw cap and parafilm, removed from the glovebox, and transferred to a heating block preheated to 80 °C, where the reaction mixture was stirred (500 rpm) for 16 h. Then, the mixture was cooled to 25 °C, MTBE (1.5 mL) and water (1.5 mL) were added and the resulting layers were separated using a pipette. The aqueous layer was extracted with MTBE (2 × 1.5 mL), the organic layers were combined, and all volatiles were evaporated under reduced pressure. The resulting residue was purified by flash column chromatography on silica gel (eluting with EtOAc / hexanes = 1:15 (v/v)) to afford 11.9 mg of biphenylether derivative **76** as a colorless oil (45%).

R_f = 0.20 (EtOAc / hexanes 1:9 (v/v))

NMR Spectroscopy:

^{1}H NMR (500 MHz, $CDCl_3$, 25 ºC, δ): 7.58 (d, *J* = 8.3 Hz, 2H), 7.50 – 7.45 (m, 1H), 7.18 (d, *J* = 8.3 Hz, 2H), 6.95 (t, *J* = 8.3 Hz, 1H), 6.68 (d, *J* = 2.3 Hz, 1H), 6.66 (t, *J* = 50.1 Hz, 1H).

^{13}C NMR (126 MHz, $CDCl_3$, 25 ºC, δ): 165.3, 163.2, 160.2 (d, *J* = 3.9 Hz), 156.6 (t, *J* = 2.1 Hz), 135.1 (d, *J* = 10.2 Hz), 131.7 (t, *J* = 22.9 Hz), 128.0 (t, *J* = 6.0 Hz), 120.4, 115.8, 114.2, 112.7 (d, *J* = 3.5 Hz), 111.0, 110.6 (d, *J* = 19.5 Hz), 94.5 (d, *J* = 18.1 Hz).

^{19}F NMR (471 MHz, $CDCl_3$, 25 ºC, δ): –104.1 (t, *J* = 6.8 Hz, 1F), –110.1 (d, *J* = 56.4 Hz, 2F).

HRMS-ESI (m/z) calc'd for $C_{14}H_8OF_3$ $[M]^+$, 263.0553; found, 263.0556; deviation: –1.1 ppm.

Probenecid amide difluoromethyl derivative 77

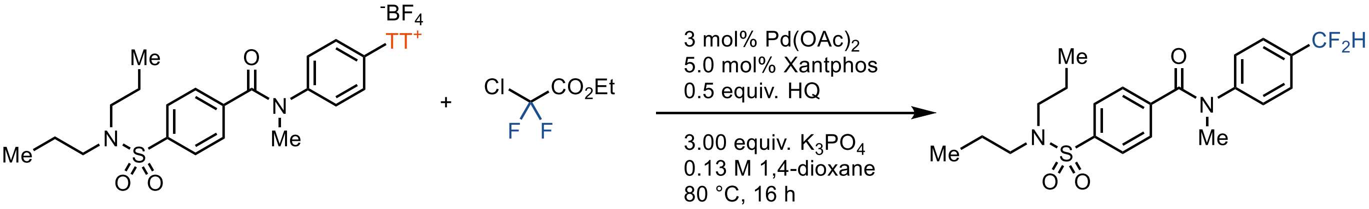

48-S 1.00 equiv. 3.0 equiv. **77**, 44%

To a 4-mL borosilicate vial containing a Teflon-coated magnetic stirring bar were added probenecid amide-derived thianthrenium salt **48-S** (67.7 mg, 0.100 mmol, 1.00 equiv.) and K_3PO_4 (63.7 mg, 0.300 mmol, 3.00 equiv.) The vial was transferred into a nitrogen-filled glovebox. An orange stock solution of dry 1,4-dioxane (0.75 mL, *c* = 0.13 M) containing $Pd(OAc)_2$ (0.6 mg, 3 µmol, 3 mol%), Xantphos (2.9 mg, 5.0 µmol, 5.0 mol%), and hydroquinone (5.5 mg, 0.50 mmol, 0.50 equiv.) were added followed by the addition of ethyl chlorodifluoroacetate (38 µL, 0.30 mmol, 3.0 equiv.) via a Hamilton syringe. The vial was sealed with a Teflon-lined screw cap and parafilm, removed from the glovebox, and transferred to a heating block preheated to 80 °C, where the reaction mixture was stirred (500 rpm) for 16 h. Then, the mixture was cooled to 25 °C and transferred to a 20 mL borosilicate vial. MTBE (4 mL) and water (4 mL) were added and the resulting layers were separated using a pipette. The aqueous layer was extracted with MTBE (2 × 4 mL), the organic layers were combined, and all volatiles were evaporated under reduced pressure. The resulting residue was purified by flash column chromatography on silica gel (hexanes / EtOAc = 3:1 to 1:1 (v/v)) to afford a mixture of **77** and the analogous ethyl ester product. Further purification by HPLC (150 mm Eclipse plus C18, 5 µm, 21.2 mm, MeCN / H_2O = 50:50 (v/v), flow rate = 20 mL/min, 25 °C, retention time = 8.29 min) afforded 18.8 mg of probenecid derivative **(±)-77** as a yellow oil (44%).

R_f = 0.14 (hexanes / EtOAc = 3:1 (v/v))

NMR Spectroscopy:

^{1}H NMR (500 MHz, $CDCl_3$, 25 ºC, δ): 7.67 – 7.59 (m, 2H), 7.43 – 7.33 (m, 6H), 7.10 (d, *J* = 8.1 Hz, 2H), 6.57 (t, *J* = 56.3 Hz, 1H), 3.52 (s, 3H), 3.05 – 2.98 (m, 4H), 1.53 – 1.42 (m, 4H), 0.82 (t, *J* = 7.4 Hz, 6H).

^{13}C NMR (151 MHz, $CDCl_3$, 25 ºC, δ): 169.3, 146.3, 141.5, 139.4, 133.2 (t, *J* = 22.8 Hz), 129.3, 127.2, 127.0 (t, *J* = 6.0 Hz), 126.9, 114.0 (t, *J* = 239.4 Hz), 49.9, 38.4, 21.9, 11.3.

^{19}F NMR (565 MHz, $CDCl_3$, 25 ºC, δ): –111.2 (d, *J* = 56.1 Hz).

HRMS-ESI (m/z) calc'd for $C_{21}H_{26}N_2O_3SF_2Na$ $[M+Na]^+$, 447.1524; found, 447.1525; deviation: 0.0 ppm.

(±)-Flurbiprofen methyl ester difluoromethyl derivative 78

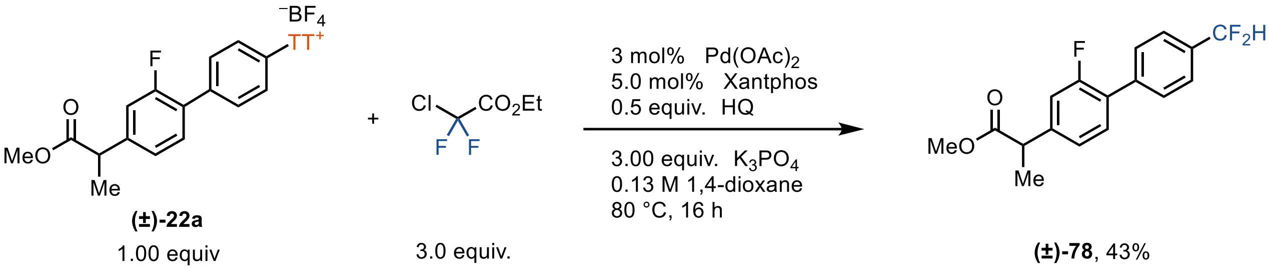

To a 4-mL borosilicate vial containing a Teflon-coated magnetic stirring bar were added (±)-flurbiprofen methyl ester-derived thianthrenium salt **22a** (56.0 mg, 0.100 mmol, 1.00 equiv.) and K_3PO_4 (63.7 mg, 0.300 mmol, 3.00 equiv.) The vial was transferred into a nitrogen-filled glovebox. An orange stock solution of dry 1,4-dioxane (0.75 mL, *c* = 0.13 M) containing $Pd(OAc)_2$ (0.6 mg, 3 µmol, 3 mol%), Xantphos (2.9 mg, 15 µmol, 5.0 mol%), and hydroquinone (5.5 mg, 0.50 mmol, 0.50 equiv.) were added followed by the addition of ethyl chlorodifluoroacetate (38 µL, 0.30 mmol, 3.0 equiv.) via a Hamilton syringe. The vial was sealed with a Teflon-lined screw cap and parafilm, removed from the glovebox, and transferred to a heating block preheated to 80 °C, where the reaction mixture was stirred (500 rpm) for 16 h. Then, the mixture was cooled to 25 °C, MTBE (1.5 mL) and water (1.5 mL) were added and the resulting layers were separated using a pipette. The aqueous layer was extracted with MTBE (2 × 1.5 mL), the organic layers were combined, and all volatiles were evaporated under reduced pressure. The resulting residue was purified by flash column chromatography on silica gel (hexanes / EtOAc = 15:1 (v/v)) to afford 13.2 mg of (±)-flurbiprofen derivative **(±)-78** as a colorless oil (43%).

R_f = 0.51 (hexanes / EtOAc = 9:1 (v/v))

NMR Spectroscopy:

^{1}H NMR (500 MHz, $CDCl_3$, 25 °C, δ): 7.65 – 7.61 (m, 2H), 7.61 – 7.56 (m, 2H), 7.40 (t, *J* = 8.0 Hz, 1H), 7.20 – 7.12 (m, 2H), 6.69 (t, *J* = 56.5 Hz, 1H), 3.78 (q, *J* = 7.2 Hz, 1H), 3.71 (s, 3H), 1.55 (d, *J* = 7.2 Hz, 3H).

^{13}C NMR (126 MHz, $CDCl_3$, 25 °C, δ): 174.5, 159.8 (d, *J* = 249.1 Hz), 142.7 (d, *J* = 7.7 Hz), 138.1, 133.7 (t, *J* = 22.5 Hz), 130.9 (d, *J* = 3.8 Hz), 129.4 (d, *J* = 3.0 Hz), 127.0 (d, *J* = 13.3 Hz), 125.8 (t, *J* = 6.0 Hz), 123.9 (d, *J* = 3.5 Hz), 115.5 (d, *J* = 23.7 Hz), 114.7 (t, *J* = 238.4 Hz), 52.4, 45.1, 18.5.

^{19}F NMR (471 MHz, $CDCl_3$, 25 °C, δ): –110.7 (d, *J* = 56.4 Hz, 2F), –117.4 (dd, *J* = 11.2, 8.1 Hz, 1F).

HRMS-ESI (m/z) calc'd for $C_{17}H_{15}O_2F_3$ $[M]^+$, 308.1019; found, 308.1024; deviation: –1.6 ppm.

(±)-Fenoprofen methyl ester difluoromethyl derivative 79

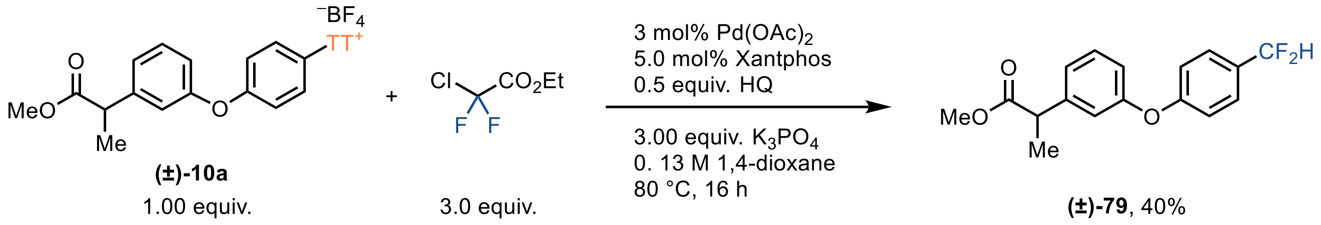

To a 4-mL borosilicate vial containing a Teflon-coated magnetic stirring bar were added (±)-fenoprofen methyl ester-derived thianthrenium salt **10a** (55.8 mg, 0.100 mmol, 1.00 equiv.) and K_3PO_4 (63.7 mg, 0.300 mmol, 3.00 equiv.) The vial was transferred into a nitrogen-filled glovebox. An orange stock solution of dry 1,4-dioxane (0.75 mL, *c* = 0.13 M) containing $Pd(OAc)_2$ (0.6 mg, 3 µmol, 3 mol%), Xantphos (2.9 mg, 15 µmol, 5.0 mol%), and hydroquinone (5.5 mg, 0.50 mmol, 0.50 equiv.) were added followed by the addition of ethyl chlorodifluoroacetate (38 µL, 0.30 mmol, 3.0 equiv.) via a Hamilton syringe. The vial was sealed with a Teflon-lined screw cap and parafilm, removed from the glovebox, and transferred to a heating block preheated to 80 °C, where the reaction mixture was stirred (500 rpm) for 16 h. Then, the mixture was cooled to 25 °C, MTBE (1.5 mL) and water (1.5 mL) were added and the resulting layers were separated using a pipette. The aqueous layer was extracted with MTBE (2 × 1.5 mL), the organic layers were combined, and all volatiles were evaporated under reduced pressure. The resulting residue was purified by flash column chromatography on silica gel (hexanes / EtOAc = 15:1 (v/v)) to afford 12.4 mg of (±)-fenoprofen derivative **(±)-79** as a colorless oil (40%).

R*f* = 0.49 (hexanes / EtOAc = 9:1 (v/v))

NMR Spectroscopy:

^{1}H NMR (500 MHz, $CDCl_3$, 25 ºC, δ): 7.47 (dt, *J* = 8.8, 1.2 Hz, 2H), 7.31 (t, *J* = 7.9 Hz, 1H), 7.10 (dt, *J* = 7.6, 1.3 Hz, 1H), 7.06 – 7.03 (m, 2H), 7.01 (t, *J* = 2.1 Hz, 1H), 6.91 (dd, *J* = 8.2, 1.0 Hz, 1H), 6.63 (t, *J* = 56.6 Hz, 1H), 3.72 (q, *J* = 7.2 Hz, 1H), 3.67 (s, 3H), 1.50 (d, *J* = 7.2 Hz, 3H).

^{13}C NMR (126 MHz, $CDCl_3$, 25 ºC, δ): 174.7, 159.5, 156.6, 142.9, 130.2, 129.2 (t, *J* = 22.7 Hz), 128.9, 127.8, 127.5 (t, *J* = 6.0 Hz), 123.3, 118.9, 118.5, 118.2, 114.7 (t, *J* = 238.4 Hz), 52.3, 45.4, 18.7.

^{19}F NMR (471 MHz, $CDCl_3$, 25 ºC, δ): –109.1 (d, *J* = 56.6 Hz).

HRMS-ESI (m/z) calc'd for $C_{17}H_{16}O_3F_2$ $[M]^+$, 306.1062; found, 306.1066; deviation: –1.3 ppm.

(±)-Oxazolidinone-derived thianthrenim salt 80-S

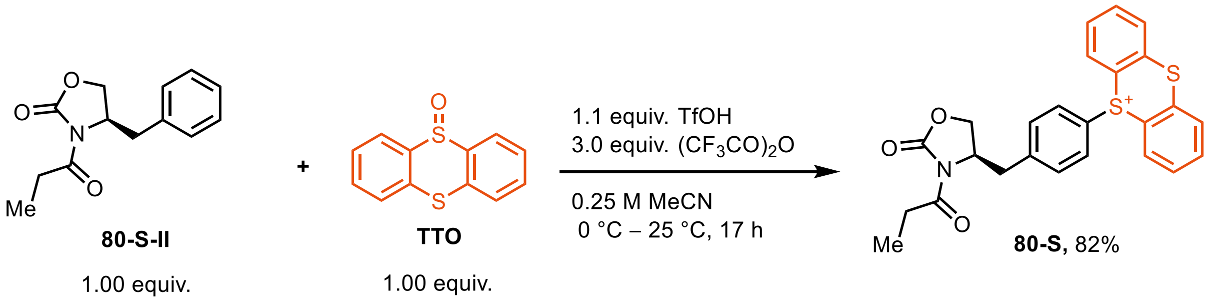

To a solution of oxazalidinone derivative **80-S-II** (602 mg, 2.58 mmol, 1.00 equiv.) and TTO (thianthrene-*S*-oxide, 600 mg, 2.58 mmol, 1.00 equiv.) in MeCN (6.0 mL, c = 0.25 M), trifluoroacetic anhydride (1.1 mL, 1.6 g, 7.8 mmol, 3.0 equiv.) was added at 0 °C, under an ambient atmosphere followed by addition of TfOH (0.20 mL, 0.34 g, 2.2 mmol, 1.1 equiv.). The reaction mixture was stirred (400 rpm) at 0 °C for 1 h, and then stirred at 25 °C for 16 h. The solvent was removed under reduced pressure by rotary evaporation and the residue was dissolved in DCM (10 mL). The resulting solution was washed with saturated aqueous $NaHCO_3$ solution (10 mL) and aqueous $NaBF_4$ solution (2 × 10 mL, 10% w/w). The crude mixture was dissolved in DCM (5 mL) and Et_2O (50 mL) was added to obtain the product as white precipitate. The precipitate was filtered to give 1.1 g of the title compound **80-S** as a colorless solid (82% yield). The spectra are in good accordance with the literature.[25]

NMR Spectroscopy:

^{1}H NMR (500 MHz, $(CD_3)_2SO$, 23 °C, δ): 8.59 (dq, J = 6.6, 1.9 Hz, 1H), 8.07 (d, J = 7.6 Hz, 1H), 7.93 (ddd, J = 7.8, 5.6, 2.2 Hz, 1H), 7.86 (t, J = 7.7 Hz, 1H), 7.39 (d, J = 8.7 Hz, 1H), 7.17 (d, J = 8.5 Hz, 1H), 4.64 (dt, J = 8.2, 2.8 Hz, 0H), 4.30 (t, J = 8.6 Hz, 0H), 4.11 (dd, J = 9.0, 2.7 Hz, 0H), 3.00 (d, J = 5.4 Hz, 1H), 2.76 (dd, J = 7.6, 5.9 Hz, 0H), 1.00 (t, J = 7.3 Hz, 1H).

^{13}C NMR (126 MHz, $(CD_3)_2SO$, 23 °C, δ): 173.7, 153.7, 141.7, 136.1, 135.9, 135.3, 132.0, 130.7, 130.1, 128.7, 123.7, 120.0, 119.9, 66.5, 54.2, 36.8, 28.8, 8.8.

^{19}F NMR (565 MHz, $(CD_3)_2SO$, 23 °C, δ): –148.2 (bs), –148.3 (bs).

HRMS-ESI(m/z) calc'd for $C_{25}H_{22}O_3NS_2$ $[M–BF_4]^+$, 448.1036; found, 448.1039; deviation: –0.7 ppm

(±)-Oxazolidinone difluoromethyl derivative 80

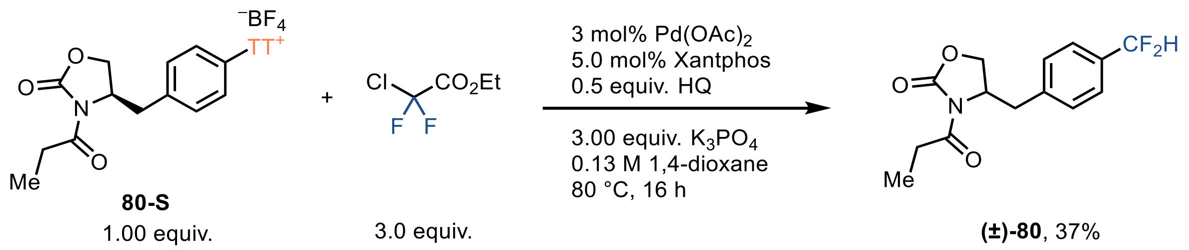

To a 4-mL borosilicate vial containing a Teflon-coated magnetic stirring bar were added oxazolidinone-derived thianthrenium salt **80-S** (55.8 mg, 0.100 mmol, 1.00 equiv.) and K_3PO_4 (63.7 mg, 0.300 mmol, 3.00 equiv.) The vial was transferred into a nitrogen-filled glovebox. An orange stock solution of dry

1,4-dioxane (0.75 mL, c = 0.13 M) containing $Pd(OAc)_2$ (0.6 mg, 3 µmol, 3 mol%), Xantphos (2.9 mg, 15 µmol, 5.0 mol%), and hydroquinone (5.5 mg, 0.50 mmol, 0.50 equiv.) were added followed by the addition of ethyl chlorodifluoroacetate (38 µL, 0.30 mmol, 3.0 equiv.) via a Hamilton syringe. The vial was sealed with a Teflon-lined screw cap and parafilm, removed from the glovebox, and transferred to a heating block preheated to 80 °C, where the reaction mixture was stirred (500 rpm) for 16 h. Then, the mixture was cooled to 25 °C, MTBE (1.5 mL) and water (1.5 mL) were added and the resulting layers were separated using a pipette. The aqueous layer was extracted with MTBE (2 × 1.5 mL), the organic layers were combined, and all volatiles were evaporated under reduced pressure. The resulting residue was purified by flash column chromatography on silica gel (hexanes / EtOAc = 3:1 (v/v)) to afford a mixture of **80** and the analogous ethyl ester product. Further purification by HPLC (150 mm Eclipse plus C18, 5 µm, 21.2 mm, MeCN / H_2O = 50:50 (v/v), flow rate = 20 mL/min, 25 °C, retention time = 6.95 min) afforded 10.5 mg of (±)-oxazolidinone derivative **80** as a colorless oil (37%).

R*f* = 0.37 (hexanes / EtOAc = 3:1 (v/v))

NMR Spectroscopy:

^{1}H NMR (500 MHz, $CDCl_3$, 25 ºC, δ): 7.49 (d, J = 7.8 Hz, 2H), 7.31 (d, J = 7.9 Hz, 2H), 6.63 (t, J = 56.4 Hz, 1H), 4.74 – 4.63 (m, 1H), 4.22 (t, J = 8.5 Hz, 1H), 4.14 (dd, J = 9.1, 2.7 Hz, 1H), 3.35 (dd, J = 13.5, 3.4 Hz, 1H), 3.04 – 2.88 (m, 2H), 2.83 (dd, J = 13.4, 9.6 Hz, 1H), 1.21 (t, J = 7.3 Hz, 3H).

^{13}C NMR (151 MHz, $CDCl_3$, 25 ºC, δ): 174.3, 153.5, 138.4 (t, J = 2.0 Hz), 133.7 (t, J = 22.6 Hz), 129.9, 126.4 (t, J = 6.0 Hz), 114.6 (t, J = 238.8 Hz), 66.3, 55.2, 38.0, 29.3, 8.4.

^{19}F NMR (471 MHz, $CDCl_3$, 25 ºC, δ): –110.7 (d, J = 56.4 Hz, 2F), –110.7 (d, J = 56.4 Hz, 2F).

HRMS-ESI (m/z) calc'd for $C_{14}H_{15}NO_3F_2$ $[M]^+$, 283.1015; found, 283.1013; deviation: +0.4 ppm.

(±)-Pyriproxyphen difluoromethyl derivative 81

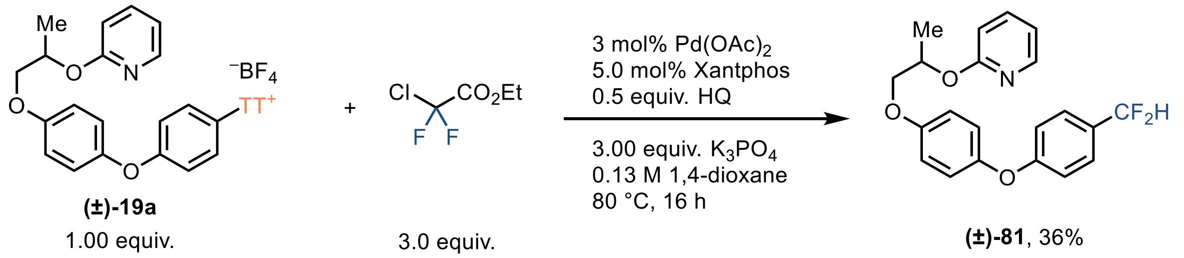

To a 4-mL borosilicate vial containing a Teflon-coated magnetic stirring bar were added (±)-pyriproxyphen-derived thianthrenium salt **19a** (62.3 mg, 0.100 mmol, 1.00 equiv.) and K_3PO_4 (63.7 mg, 0.300 mmol, 3.00 equiv.) The vial was transferred into a nitrogen-filled glovebox. An orange stock solution of dry 1,4-dioxane (0.75 mL, c = 0.13 M) containing $Pd(OAc)_2$ (0.6 mg, 3 µmol, 3 mol%), Xantphos (2.9 mg, 15 µmol, 5.0 mol%), and hydroquinone (5.5 mg, 0.50 mmol, 0.50 equiv.) were added followed by the addition of ethyl chlorodifluoroacetate (38 µL, 0.30 mmol, 3.0 equiv.) via a Hamilton syringe. The vial was sealed with a Teflon-lined screw cap and parafilm, removed from the glovebox, and transferred to a heating block preheated to 80 °C, where the reaction mixture was

stirred (500 rpm) for 16 h. Then, the mixture was cooled to 25 °C, MTBE (1.5 mL) and water (1.5 mL) were added and the resulting layers were separated using a pipette. The aqueous layer was extracted with MTBE (2 × 1.5 mL), the organic layers were combined, and all volatiles were evaporated under reduced pressure. The resulting residue was purified by flash column chromatography on silica gel (hexanes / EtOAc = 15:1 (v/v)) to afford 13.3 mg of (±)-pyriproxyphen derivative **81** as a colorless oil (36%).

R*f* = 0.38 (hexanes / EtOAc = 7:1 (v/v))

NMR Spectroscopy:

> **^{1}H NMR** (600 MHz, $CDCl_3$, 25 °C, δ): 8.15 (ddd, *J* = 5.0, 2.0, 0.8 Hz, 1H), 7.57 (ddd, *J* = 8.3, 7.1, 2.0 Hz, 1H), 7.44 – 7.41 (m, 2H), 7.00 – 6.93 (m, 6H), 6.86 (ddd, *J* = 7.1, 5.0, 1.0 Hz, 1H), 6.75 (dt, *J* = 8.3, 0.9 Hz, 1H), 6.61 (t, *J* = 56.5 Hz, 1H), 5.61 – 5.58 (m, 1H), 4.20 (dd, *J* = 9.9, 5.3 Hz, 1H), 4.09 (dd, *J* = 9.9, 4.9 Hz, 1H), 1.49 (d, *J* = 6.4 Hz, 3H).
>
> **^{13}C NMR** (151 MHz, $CDCl_3$, 25 °C, δ): 163.3, 160.8, 155.9, 149.4, 146.9, 138.9, 128.4 (t, *J* = 22.7 Hz), 127.4 (t, *J* = 6.0 Hz), 121.4, 117.3, 116.9, 116.4, 116.1, 114.8 (t, *J* = 237.8 Hz), 71.2, 69.4, 17.2.
>
> **^{19}F NMR** (471 MHz, $CDCl_3$, 25 °C, δ): –108.8 (d, *J* = 56.5 Hz).

HRMS-ESI (m/z) calc'd for $C_{21}H_{19}NO_3F_2Na$ $[M+Na]^+$, 394.1225; found, 394.1222; deviation: +0.9 ppm.

Niflumic acid methyl ester difluoromethyl derivative 82

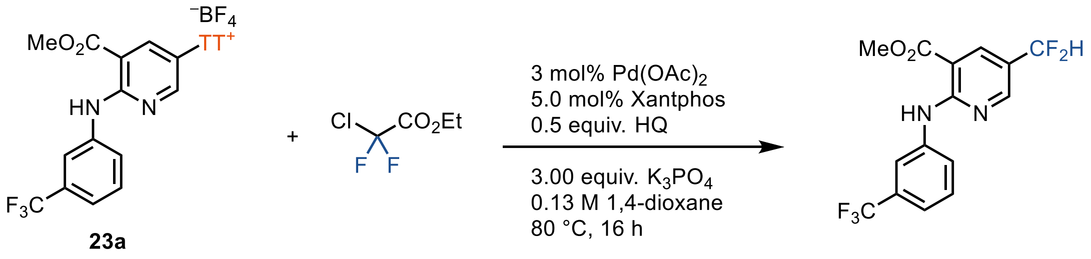

23a 1.00 equiv. 3.0 equiv. **82**, 37%

To a 4-mL borosilicate vial containing a Teflon-coated magnetic stirring bar were added niflumic acid methyl ester-derived thianthrenium salt **23a** (59.8 mg, 0.100 mmol, 1.00 equiv.) and K_3PO_4 (63.7 mg, 0.300 mmol, 3.00 equiv.) The vial was transferred into a nitrogen-filled glovebox. An orange stock solution of dry 1,4-dioxane (0.75 mL, *c* = 0.13 M) containing $Pd(OAc)_2$ (0.6 mg, 3 µmol, 3 mol%), Xantphos (2.9 mg, 15 µmol, 5.0 mol%), and hydroquinone (5.5 mg, 0.50 mmol, 0.50 equiv.) were added followed by the addition of ethyl chlorodifluoroacetate (38 µL, 0.30 mmol, 3.0 equiv.) via a Hamilton syringe. The vial was sealed with a Teflon-lined screw cap and parafilm, removed from the glovebox, and transferred to a heating block preheated to 80 °C where the reaction mixture was stirred (500 rpm) for 16 h. Then, the mixture was cooled to 25 °C, MTBE (1.5 mL) and water (1.5 mL) were added and the resulting layers were separated using a pipette. The aqueous layer was extracted with MTBE (2 × 1.5 mL), the organic layers were combined, and all volatiles were evaporated under reduced pressure. The resulting residue was purified by flash column chromatography on silica gel

(hexanes / EtOAc = 50:1 (v/v)) to afford 12.9 mg of niflumic acid derivative **82** as a colorless oil (37%).

R*f* = 0.13 (hexanes / EtOAc = 50:1 (v/v))

NMR Spectroscopy:

^{1}H NMR (500 MHz, $CDCl_3$, 25 ºC, δ): 10.53 (s, 1H), 8.57 – 8.48 (m, 1H), 8.46 – 8.40 (m, 1H), 8.09 (d, *J* = 1.9 Hz, 1H), 7.86 (d, *J* = 1.4 Hz, 1H), 7.46 (t, *J* = 7.9 Hz, 1H), 7.36 – 7.32 (m, 1H), 6.65 (t, *J* = 56.1 Hz, 1H), 3.98 (s, 3H).

^{13}C NMR (151 MHz, $CDCl_3$, 25 ºC, δ): 167.5, 156.9, 151.0 (t, *J* = 6.4 Hz), 139.7, 138.1 (t, *J* = 5.1 Hz), 131.4 (q, *J* = 32.3 Hz), 129.5, 125.2 – 123.2 (m), 120.7 (t, *J* = 24.0 Hz), 120.1 (q, *J* = 3.9 Hz), 117.9 (q, *J* = 3.9 Hz), 113.5 (t, *J* = 238.1 Hz), 107.1, 52.8.

^{19}F NMR (471 MHz, $CDCl_3$, 25 °C, δ): –62.7 (s, 3F), –110.0 (d, *J* = 56.2 Hz, 2F).

HRMS-ESI (m/z) calc'd for $C_{15}H_{12}N_2O_2F_5$ $[M+H]^+$, 347.0814; found, 347.0812; deviation: +0.4 ppm.

N-Tosylmethylaniline difluoromethyl derivative 83

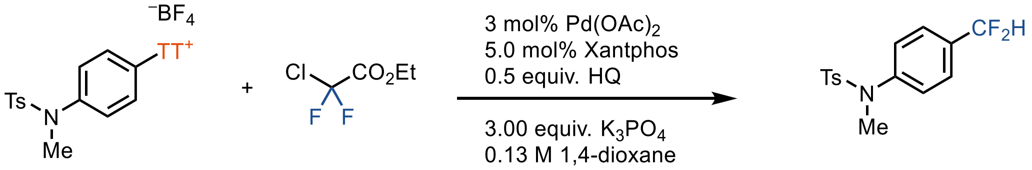

39a 1.00 equiv. 3.0 equiv. **83**, 58%

To a 4-mL borosilicate vial containing a Teflon-coated magnetic stirring bar were added *N*-tosylmethylaniline-derived thianthrenium salt **39a** (56.3 mg, 0.100 mmol, 1.00 equiv.) and K_3PO_4 (63.7 mg, 0.300 mmol, 3.00 equiv.) The vial was transferred into a nitrogen-filled glovebox. An orange stock solution of dry 1,4-dioxane (0.75 mL ml, *c* = 0.13 M) containing $Pd(OAc)_2$ (0.6 mg, 3 µmol, 3 mol%), Xantphos (2.9 mg, 5.0 µmol, 5.0 mol%), and hydroquinone (5.5 mg, 0.50 mmol, 0.50 equiv.) were added followed by the addition of ethyl chlorodifluoroacetate (38 µL, 0.30 mmol, 3.0 equiv.) via a Hamilton syringe. The vial was sealed with a Teflon-lined screw cap and parafilm, removed from the glovebox, and transferred to a heating block preheated to 80 °C, where the reaction mixture was stirred (500 rpm) for 16 h. Then, the mixture was cooled to 25 °C and transferred to a 20 mL borosilicate vial. MTBE (4 mL) and water (4 mL) were added and the resulting layers were separated using a pipette. The aqueous layer was extracted with MTBE (2 × 4 mL), the organic layers were combined, and all volatiles were evaporated under reduced pressure. The resulting residue was purified by flash column chromatography on silica gel (hexanes / EtOAc = 12:1 (v/v)) to afford 17.9 mg of *N*-tosylmethylaniline derivative **83** as a colorless oil (58%).

R*f* = 0.16 (hexanes / EtOAc = 9:1 (v/v))

NMR Spectroscopy:

^{1}H NMR (500 MHz, $CDCl_3$, 25 ºC, δ): 7.43 (dd, *J* = 15.1, 8.2 Hz, 4H), 7.23 (dd, *J* = 17.7, 8.1 Hz, 4H), 6.63 (t, *J* = 56.4 Hz, 1H), 3.17 (s, 3H), 2.42 (s, 3H).

^{13}C NMR (126 MHz, $CDCl_3$, 25 ºC, δ): 144.0, 144.0, 133.4, 133.1 (t, *J* = 22.7 Hz), 129.6, 127.9, 126.6, 126.3 (t, *J* = 6.0 Hz), 114.3 (t, *J* = 239.0 Hz), 38.0, 21.7.

^{19}F NMR (471 MHz, $CDCl_3$, 25 ºC, δ): –110.8 (d, *J* = 56.4 Hz).

HRMS-ESI (m/z) calc'd for $C_{15}H_{15}NO_2SF_2$ $[M]^+$, 311.0786; found, 311.0789; deviation: –1.1 ppm.

Procedure by using the Schlenk line: To a 4-mL borosilicate vial containing a Teflon-coated magnetic stirring bar were added *N*-tosylmethylaniline derived thianthrenium salt (56.3 mg, 0.100 mmol, 1.00 equiv.), K_3PO_4 (63.7 mg, 0.300 mmol, 3.00 equiv.), $Pd(OAc)_2$ (0.6 mg, 3 µmol, 3 mol%), Xantphos (2.9 mg, 5.0 µmol, 5.0 mol%), and hydroquinone (5.5 mg, 0.50 mmol, 0.50 equiv.). The vial was sealed with a septum cap. The septum was pierced with a needle connected to a Schlenk line. The vial was evacuated and backfilled with argon three times. Under a positive pressure of argon, ethyl chlorodifluoroacetate (38 µL, 0.30 mmol, 3.0 equiv.) was added via a Hamilton syringe followed by dry 1,4-dioxane (0.13 M, 0.75 mL). Vacuum grease was applied to pierced parts of the septum and the septum cap was wrapped with parafilm. The vial was then transferred to a heating block preheated at 80 °C where the reaction mixture was stirred (500 rpm) for 24 h. Then, the mixture was cooled to 25 °C and transferred to a 20 mL borosilicate vial. MTBE (4 mL) and water (4 mL) were added and the resulting layers were separated using a pipette. The aqueous layer was extracted with MTBE (2 × 4 mL), the organic layers were combined, and all volatiles were evaporated under reduced pressure. The residue was dissolved in $CDCl_3$ (0.3 mL) and fluorobenzene (19.2 mg, 18.6 µL, 2.00 equiv.) was added via a Hamilton syringe. After gentle agitation for 1 min, the solution was transferred to an NMR tube and a $^{19}F\{^1H\}$ NMR spectrum was recorded. The yield (50%) was determined by the relative integrals of the signals of the internal standard at –113.1 ppm(s, 1F) and the product **83** at –110.8 ppm (s, 2F).

Analysis of the side products for difluoromethylation of aryl thianthrenium salts

During the optimization of the reaction conditions several side products were detected which diminishes the yield of the desired product. Despite low yields and formation of multiple side products, desired product can be purified by flash column chromatography without any challenges in most of the cases. All side products are also purified by flash column chromatography and were characterized by ^{1}H NMR spectroscopy, ^{13}C NMR spectroscopy, and HRMS. Due to the spectral overlap, the yield of side product **83c** cannot be reliably determined via ^{1}H NMR but upon isolation was found to be in the range of 5–10%.

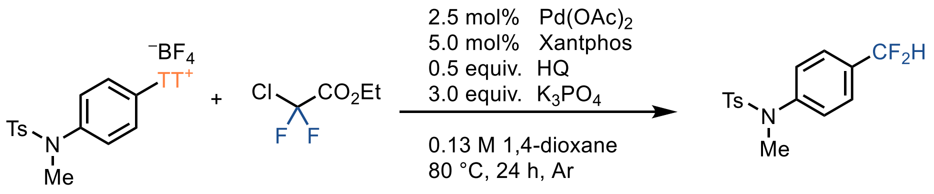

39a 1.00 equiv. 3.0 equiv. **83,** 51%[a]

Side Products

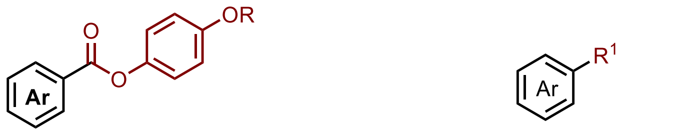

R = H, **83a,** 17%[b]
R = COAr, **83b,** 18%[b]

R^1 = CO_2Et, **83c,** 4%[b]
R^1 = H, **83d,** n.d.

Fig S11: Reactions are carrid out on a 0.100 mmol scale. [a]Yield is determined by ^{1}H NMR. [b]Yield is determined by ^{19}F NMR.

Difluoromethylation side product 83a

R*f* = 0.50 (EtOAc / hexanes = 1:1 (v/v))

NMR Spectroscopy:

^{1}H NMR (500 MHz, $CDCl_3$, 25 ºC, δ): 8.12 (d, *J* = 8.7 Hz, 2H), 7.44 (d, *J* = 8.4 Hz, 2H), 7.31 – 7.27 (m, 2H), 7.08 – 7.03 (m, 2H), 6.89 – 6.82 (m, 2H), 4.90 (s, 1H), 3.22 (s, 3H), 2.42 (s, 3H).

^{13}C NMR (151 MHz, $CDCl_3$, 25 ºC, δ): 165.0, 153.5, 146.5, 144.5, 144.2, 133.4, 131.0, 129.7, 127.9, 127.8, 125.7, 122.7, 116.2, 37.8, 21.7.

HRMS-ESI (m/z) calc'd for $C_{21}H_{19}NO_5SNa$ $[M+Na]^+$, 420.0876; found, 420.0877; deviation: –0.3 ppm.

Difluoromethylation side product 83b

R*f* = 0.53 (EtOAc / hexanes = 1:1 (v/v))

NMR Spectroscopy:

^{1}H NMR (500 MHz, $CDCl_3$, 25 ºC, δ): 8.14 (d, *J* = 8.5 Hz, 4H), 7.45 (d, *J* = 8.1 Hz, 4H), 7.34 – 7.26 (m, 12H), 3.23 (s, 6H), 2.43 (s, 6H).

^{13}C NMR (151 MHz, $CDCl_3$, 25 ºC, δ): 164.5, 148.5, 146.7, 144.2, 133.4, 131.0, 129.7, 127.9, 127.6, 125.8, 122.8, 116.2, 37.8, 21.7.

HRMS-ESI (m/z) calc'd for $C_{36}H_{32}N_2O_8S_2Na$ $[M+Na]^+$, 707.1492; found, 707.1494; deviation: –0.3 ppm.

Difluoromethylation side product 83c

R*f* = 0.15 (EtOAc / hexanes = 1:9 (v/v))

NMR Spectroscopy:

^{1}H NMR (500 MHz, $CDCl_3$, 25 ºC, δ): 7.97 (d, *J* = 8.7 Hz, 2H), 7.43 – 7.38 (m, 2H), 7.23 (d, *J* =

8.0 Hz, 2H), 7.20 (d, *J* = 8.6 Hz, 2H), 4.37 (q, *J* = 7.1 Hz, 2H), 3.19 (s, 3H), 2.41 (s, 3H), 1.39 (t, *J* = 7.1 Hz, 3H).

^{13}C NMR (151 MHz, $CDCl_3$, 25 ºC, δ): 166.1, 145.8, 144.1, 133.4, 130.3, 129.6, 128.9, 127.9, 125.7, 61.3, 37.8, 21.7, 14.5.

HRMS-ESI (m/z) calc'd for $C_{17}H_{19}NO_4S$ $[M]^+$, 333.1029; found, 333.1032; deviation: –0.7 ppm.

Difluoromethylation side product 83d

R*f* = 0.27 (EtOAc / hexanes = 1:9 (v/v))

NMR Spectroscopy:

^{1}H NMR (500 MHz, $CDCl_3$, 25 ºC, δ): 7.44 (d, *J* = 8.3 Hz, 2H), 7.33 – 7.27 (m, 3H), 7.25 – 7.23 (m, 1H), 7.13 – 7.09 (m, 2H), 3.18 (s, 3H), 2.43 (s, 3H).

^{13}C NMR (126 MHz, $CDCl_3$, 25 ºC, δ): 143.6, 141.7, 133.6, 129.4, 128.9, 128.0, 127.3, 126.7, 38.2, 21.6.

HRMS-ESI (m/z) calc'd for $C_{14}H_{15}NO_2S$ $[M]^+$, 261.0818; found, 261.0820; deviation: –0.7 ppm.

Cyanation of aryl thianthrenium salts

General procedure for cyanation of aryl thianthrenium salts

Under an ambient atmosphere, aryl thianthrenium salt (0.500 mmol, 1.00 equiv.), $K_4[FeCN_6]\cdot 3H_2O$ (106 mg, 0.250 mmol, 0.500 equiv.), KOAc (49 mg, 0.50 mmol, 1.0 equiv.), $Pd(OAc)_2$ (5.6 mg, 25 μmol, 5.0 mol%) and dppf (27.7 mg, 50.0 μmol, 10.0 mol%) were added to a 20-mL borosilicate vial containing a Teflon-coated magnetic stirring bar. The vial was transferred to a nitrogen-filled glovebox and THF (2.5 mL) was added. The vial was removed from the glovebox and H_2O (2.5 mL, deoxygenated for 1 minute with Ar flow) was added under Ar atmosphere. The vial was sealed with a cap, and the reaction mixture was stirred (600 rpm) vigorously at 80 °C for 16 h on a heating block. After that, the reaction mixture was cooled to room temperature and transferred to a separatory funnel using ethyl acetate (15 mL) and brine (15 mL). The organic layer was separated from the aqueous layer, and the aqueous layer was further extracted with ethyl acetate (2 × 15 mL). The combined organic layers were dried over Na_2SO_4, filtered, and concentrated by rotary evaporation. The residue was purified by chromatography on silica gel.

(±)-Pyriproxyphen cyanide derivative 84

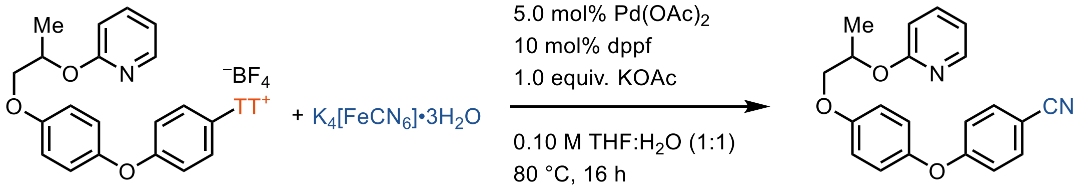

(±)-19a

1.00 equiv. 0.500 equiv. **(±)-84**, 93%

Under an ambient atmosphere, (±)-pyriproxyphen-derived thianthrenium salt **19a** (311 mg, 0.500 mmol, 1.00 equiv.), $K_4[FeCN_6]\cdot 3H_2O$ (106 mg, 0.250 mmol, 0.500 equiv.), KOAc (49 mg, 0.50 mmol, 1.0 equiv.), $Pd(OAc)_2$ (5.6 mg, 25 μmol, 5.0 mol%) and dppf (27.7 mg, 50.0 μmol, 10.0 mol%) were added to a 20-mL borosilicate vial containing a Teflon-coated magnetic stirring bar. The vial was transferred to a nitrogen-filled glovebox and THF (2.5 mL) was added. The vial was removed from the glovebox and H_2O (2.5 mL, deoxygenated for 1 minute with Ar flow) was added under Ar atmosphere. The vial was sealed with a cap, and the reaction mixture was stirred (600 rpm) vigorously at 80 °C for 16 h on a heating block. After that, the reaction mixture was cooled to room temperature and transferred to a separatory funnel using ethyl acetate (15 mL) and brine (15 mL). The organic layer was separated from the aqueous layer, and the aqueous layer was further extracted with ethyl acetate (2 × 15 mL). The combined organic layers were dried over Na_2SO_4, filtered, and concentrated by rotary evaporation. The residue was purified by chromatography on silica gel, eluting with (hexanes / EtOAc = 10:1 (v/v)) to afford 161 mg (93% yield) of the title compound **(±)-84** as a colorless oil.

R_f = 0.23 (hexanes/ EtOAc = 10:1 (v/v))

NMR Spectroscopy:

^{1}H NMR (600 MHz, $CDCl_3$, 25 °C, δ): 8.14 (ddd, J = 5.0, 2.0, 0.8 Hz, 1H), 7.58 – 7.53 (m, 3H), 6.97 (d, J = 0.5 Hz, 4H), 6.95 – 6.90 (m, 2H), 6.85 (ddd, J = 7.1, 5.0, 1.0 Hz, 1H), 6.74 (dt, J = 8.3, 0.9 Hz, 1H), 5.62 – 5.57 (m, 1H), 4.20 (dd, J = 9.9, 5.3 Hz, 1H), 4.09 (dd, J = 9.9, 4.8 Hz, 1H), 1.48 (d, J = 6.4 Hz, 3H).

^{13}C NMR (151 MHz, $CDCl_3$, 25 °C, δ): 163.2, 162.5, 156.4, 148.1, 146.8, 138.8, 134.1, 121.8, 119.0, 117.2, 116.9, 116.2, 111.7, 105.3, 71.1, 69.2, 17.1.

HRMS ESI (m/z) calc'd for $C_{21}H_{18}N_2O_3Na$ $[M+Na]^+$, 369.1210; found, 369.1207. deviation: +0.7 ppm.

Fenofibrate cyanide derivative 85

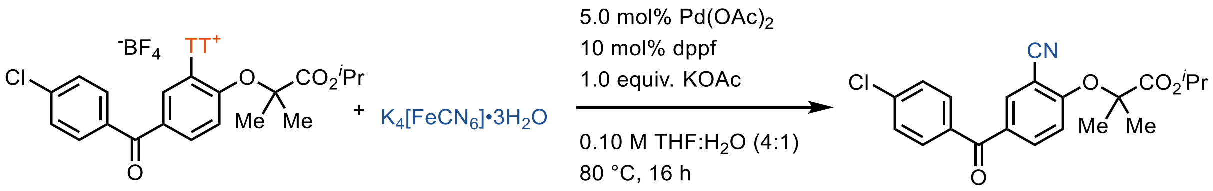

42a 1.00 equiv. 0.500 equiv. **85**, 51%

Under an ambient atmosphere, fenofibrate-derived thianthrenium salt **42a** (332 mg, 0.500 mmol, 1.00 equiv.), $K_4[FeCN_6]\cdot 3H_2O$ (106 mg, 0.250 mmol, 0.500 equiv.), KOAc (49 mg, 0.50 mmol, 1.0 equiv.), $Pd(OAc)_2$ (5.6 mg, 25 μmol, 5.0 mol%) and dppf (27.7 mg, 50.0 μmol, 10.0 mol%) were added to a 20-mL borosilicate vial containing a Teflon-coated magnetic stirring bar. The vial was transferred to a nitrogen-filled glovebox and THF (2.5 mL) was added. The vial was removed from the glovebox and H_2O (2.5 mL, deoxygenated for 1 minute with Ar flow) was added under Ar atmosphere. The vial was sealed with a cap, and the reaction mixture was stirred (600 rpm) vigorously at 80 °C for 16 h on a heating block. After that, the reaction mixture was cooled to room temperature and transferred to a

separatory funnel using ethyl acetate (15 mL) and brine (15 mL). The organic layer was separated from the aqueous layer, and the aqueous layer was further extracted with ethyl acetate (2 × 15 mL). The combined organic layers were dried over Na_2SO_4, filtered, and concentrated by rotary evaporation. The residue was purified by chromatography on silica gel (hexanes / EtOAc = 50:1 to 10:1 (v/v)) to afford 98.1 mg (51% yield) of the title compound **85** as a colorless solid.

R*f* = 0.20 (hexanes / EtOAc = 10:1 (v/v))

NMR Spectroscopy:

^{1}H NMR (500 MHz, $CDCl_3$, 25 °C, δ): 8.02 (d, *J* = 2.2 Hz, 1H), 7.91 (dd, *J* = 8.9, 2.2 Hz, 1H), 7.70 – 7.65 (m, 2H), 7.51 – 7.45 (m, 2H), 6.84 (d, *J* = 8.8 Hz, 1H), 5.08 (h, *J* = 6.3 Hz, 1H), 1.73 (s, 6H), 1.20 (d, *J* = 6.3 Hz, 6H).

^{13}C NMR (126 MHz, $CDCl_3$, 25 °C, δ): 192.4, 172.1, 161.1, 139.4, 136.3, 135.3, 135.2, 131.2, 130.4, 129.1, 115.6, 115.5, 104.5, 81.7, 70.0, 25.4, 21.6.

HRMS ESIpos (m/z) calc'd for $C_{21}H_{20}NO_4ClNa$ $[M+Na]^+$, 408.0973; found, 408.0974. deviation: –0.2 ppm.

Boscalid cyanide derivative 86

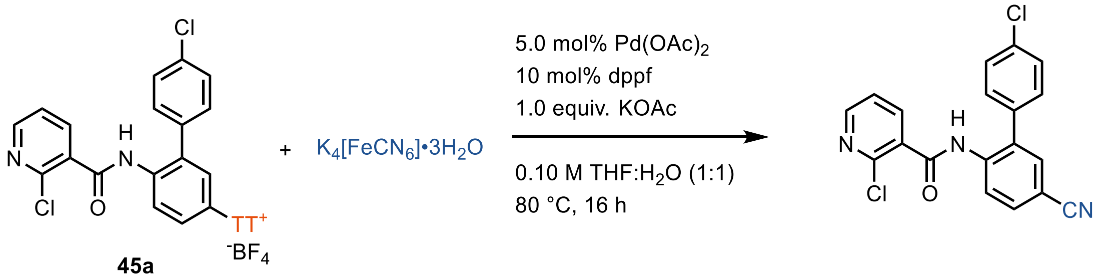

45a
1.00 equiv. 0.500 equiv. **86**, 68%

Under an ambient atmosphere, boscalid-derived thianthrenium salt **45a** (323 mg, 0.500 mmol, 1.00 equiv.), $K_4[FeCN_6]·3H_2O$ (106 mg, 0.250 mmol, 0.500 equiv.), KOAc (49 mg, 0.50 mmol, 1.0 equiv.), $Pd(OAc)_2$ (5.6 mg, 25 μmol, 5.0 mol%) and dppf (27.7 mg, 50.0 μmol, 10.0 mol%) were added to a 20-mL borosilicate vial containing a Teflon-coated magnetic stirring bar. The vial was transferred to a nitrogen-filled glovebox and THF (2.5 mL) was added. The vial was removed from the glovebox and H_2O (2.5 mL, deoxygenated for 1 minute with Ar flow) was added under Ar atmosphere. The vial was sealed with a cap, and the reaction mixture was stirred (600 rpm) vigorously at 80 °C for 16 h on a heating block. After that, the reaction mixture was cooled to room temperature and transferred to a separatory funnel using ethyl acetate (15 mL) and brine (15 mL). The organic layer was separated from the aqueous layer, and the aqueous layer was further extracted with ethyl acetate (2 × 15 mL). The combined organic layers were dried over Na_2SO_4, filtered, and concentrated by rotary evaporation. The residue was purified by chromatography on silica gel (hexanes / EtOAc = 10:1 to 2:1 (v/v)) to afford 125 mg (68% yield) of the title compound **86** as a colorless solid.

R*f* = 0.30 (hexanes / EtOAc = 1:1 (v/v))

NMR Spectroscopy:

^{1}H NMR (500 MHz, $CDCl_3$, 25 °C, δ): 8.75 (d, *J* = 8.7 Hz, 1H), 8.48 (dd, *J* = 4.7, 2.0 Hz, 2H), 8.22 (dd, *J* = 7.7, 2.0 Hz, 1H), 7.74 (dd, *J* = 8.7, 2.0 Hz, 1H), 7.55 (d, *J* = 2.0 Hz, 1H), 7.52 – 7.49 (m, 2H), 7.39 (dd, *J* = 7.7, 4.7 Hz, 1H), 7.35 – 7.30 (m, 2H).

^{13}C NMR (126 MHz, $CDCl_3$, 25 °C, δ): 162.6, 152.0, 146.6, 141.0, 138.9, 135.9, 133.89, 133.90, 133.1, 132.2, 130.8, 130.3, 130.0, 123.3, 121.5, 118.5, 108.3.

HRMS GC-EI (m/z) calc'd for $C_{19}H_{11}N_3OCl_2$ $[M]^+$, 367.0274; found, 367.0277. deviation: –0.8 ppm.

(±)-Flurbiprofen methyl ester cyanide derivative 87

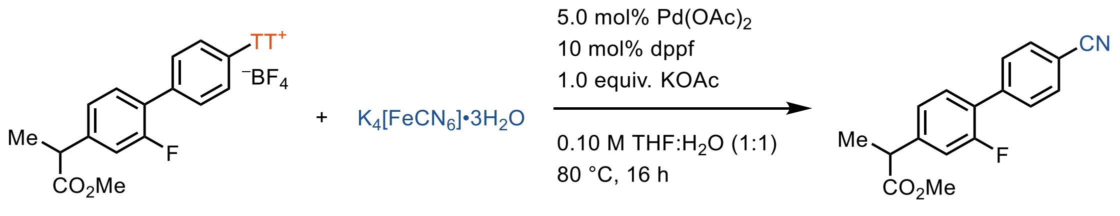

(±)-22a 1.00 equiv. 0.500 equiv. **(±)-87**, 92%

Under an ambient atmosphere, (±)-flurbiprofen methyl ester-derived thianthrenium salt **22a** (56.0 mg, 0.100 mmol, 1.00 equiv.), $K_4[FeCN_6]\cdot 3H_2O$ (21.1 mg, 50.0 µmol, 50.0 mol%), KOAc (9.8 mg, 0.10 mmol, 1.0 equiv.), $Pd(OAc)_2$ (1.1 mg, 5.0 µmol, 5.0 mol%) and dppf (5.5 mg, 10 µmol, 10 mol%) were added to a 4-mL borosilicate vial containing a Teflon-coated magnetic stirring bar. The vial was transferred to a nitrogen-filled glovebox and THF (0.5 mL) was added. The vial was removed from the glovebox and H_2O (0.5 mL, deoxygenated for 1 minute with Ar flow) was added under Ar atmosphere. The vial was sealed with a cap, and the reaction mixture was stirred vigorously at 80 °C for 16 h on a heating block. After that, the reaction mixture was cooled to room temperature and transferred to a separatory funnel using ethyl acetate (5 mL) and brine (5 mL). The organic layer was separated from the aqueous layer, and the aqueous layer was further extracted with ethyl acetate (2 × 5 mL). The combined organic layers were dried over Na_2SO_4, filtered, and concentrated by rotary evaporation. The residue was purified by chromatography on silica gel (hexanes / EtOAc = 50:1 to 10:1 (v/v)) to afford 26.0 mg (92% yield) of the title compound **(±)-87** as a colorless solid.

R*f* = 0.30 (hexanes / EtOAc = 5:1 (v/v))

NMR Spectroscopy:

^{1}H NMR (500 MHz, $CDCl_3$, 25 °C, δ): 7.72 (d, *J* = 8,4 Hz, 2H), 7.64 (d, *J* = 7.9 Hz, 2H), 7.38 (t, *J* = 8.0 Hz, 1H), 7.19 (d, *J* = 8.0 Hz, 1H), 7.16 (d, *J* = 11.6 Hz, 1H), 3.81 (q, *J* = 7.2 Hz, 1H), 3.70 (s, 3H), 1.54 (d, *J* = 7.2 Hz, 3H).

^{13}C NMR (126 MHz, $CDCl_3$, 25 °C, δ): 174.3, 159.7 (d, *J* = 248.8 Hz), 143.6 (d, *J* = 8.2 Hz), 140.3, 132.4, 130.7 (d, *J* = 3.6 Hz), 129.7 (d, *J* = 3.4 Hz), 126.1 (d, *J* = 12.8z Hz), 124.1 (d, *J* = 3.2 Hz), 118.9, 115.7 (d, *J* = 23.7 Hz), 111.5, 52.4, 45.1, 18.5.

^{19}F NMR (471 MHz, $CDCl_3$, 25 °C, δ): –117.0.

HRMS GC-EI (m/z) calc'd for $C_{17}H_{14}NO_2F$ $[M]^+$, 283.1003; found, 283.1008. deviation: –1.7 ppm.

Procedure by using Schlenk line: To a a 4-mL borosilicate vial containing a Teflon-coated magnetic stirring bar were added flurbiprofen methyl ester-derived thianthrenium salt (56.0 mg, 0.100 mmol, 1.00 equiv.), $K_4[FeCN_6]·3H_2O$ (21.1 mg, 50.0 µmol, 0.500 equiv.), KOAc (9.8 mg, 0.10 mmol, 1.0 equiv.), $Pd(OAc)_2$ (1.1 mg, 5.0 µmol, 5.0 mol%) and dppf (5.5 mg, 10 µmol, 10 mol%. After attaching with a Schlenk-line adapter, the vial was evacuated and backfilled with argon three times using a Schlenk line. Degassed THF (0.5 mL) was then added to the reaction mixture, followed by degassed H_2O (0.5 mL) under Ar flow. The vial was sealed, and the reaction mixture was stirred vigorously at 80 °C for 16 h on a heating block. After that, the reaction mixture was cooled to room temperature. 1,3,5-trimethoxybenzene (16.8 mg, 0.100 mmol, 1.00 equiv.) was added into the reaction mixture as an internal standard, followed by EtOAc (2.0 mL). An aliquot (ca. 0.5 mL) from the EtOAc phase was taken and the concentrated by rotary evaporation. The residue was dissolved in $CDCl_3$ (0.6 mL), and the ^{1}H NMR was recorded. The yield (95%) was determined from the ^{1}H NMR spectrum by integrating the signal of the internal standard at 6.11 ppm (s, 3H) and the product **87** at 7.72 ppm (d, J = 8,4 Hz, 2H).

Additive screening for cyanation of aryl thianthrenium salts

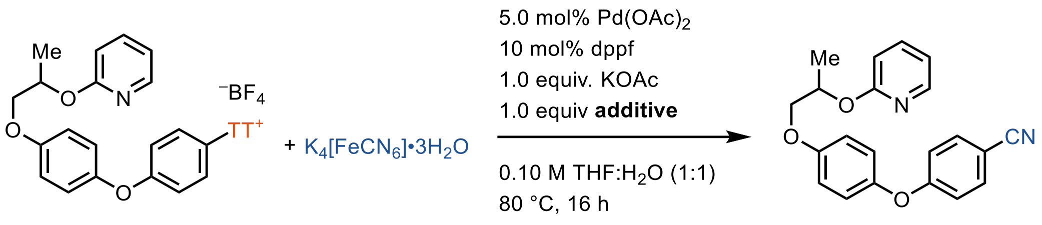

(±)-19a
1.00 equiv.　　0.500 equiv.　　**(±)-84**

Additive	Yield of 84 (%)	Remaing additive (%)	Additive	Yield of 84 (%)	Remaing additive (%)
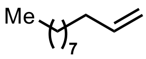	96 ✓	75 ✓	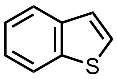	98 ✓	88 ✓
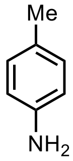	69 ✓	96 ✓	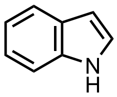	100 ✓	100 ✓
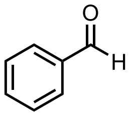	93 ✓	60 ✓	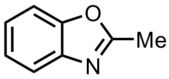	98 ✓	84 ✓
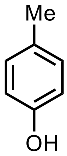	94 ✓	85 ✓	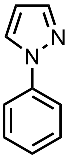	93 ✓	93 ✓
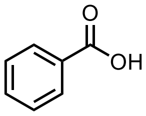	96 ✓	100 ✓	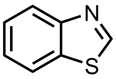	89 ✓	72 ✓

Fig S12: Additive screening for cyanation of aryl thianthrenium salts. Reactions were carried out on a 0.100 mmol scale and yields were determined by ^{1}H NMR by using 1,3,5-trimethoxybenzene as an internal standard.

C–C bond formation of aryl thianthrenium salts

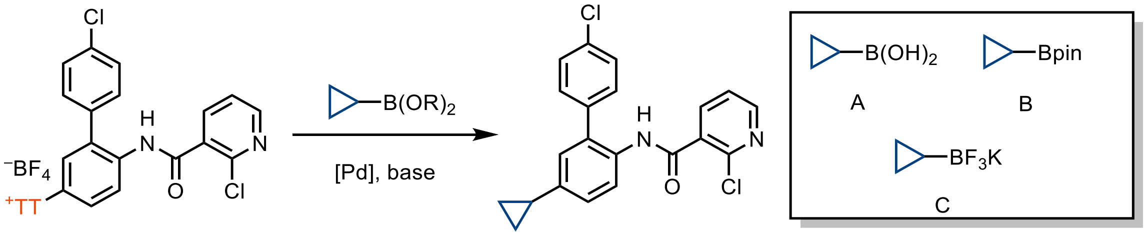

Table S10 Optimization of reaction conditions for cyclopropylation of aryl thianthrenium salts:

Entry	Cyclopropyl source	Pd catalyst and ligand	Temperature (°C)	Base (2.00 equiv.)	Solvent (0.10 M)	Yield (%)
**1**	A	$PddppfCl_2$ (2 mol%)	50	K_3PO_4	1,4-dioxane:*i*PrOH (1:1)	0
**2**	B	$PddppfCl_2$ (2 mol%)	50	CsF	1,4-dioxane:*i*PrOH	0

					(1:1)	
**3**	A	PddppfCl$_2$ (4 mol%)	50	K_3PO_4	1,4-dioxane:*i*PrOH (1:1)	0
**4**	A	PddppfCl$_2$ (4 mol%)	80	K_3PO_4	1,4-dioxane	<5%
**5**	A	Pd(PPh$_3$)$_4$ (10 mol%)	50	K_3PO_4	1,4-dioxane:*i*PrOH (1:1)	0
**6**	B	Pd(PPh$_3$)$_4$ (10 mol%)	50	CsF	1,4-dioxane:*i*PrOH (1:1)	0
**7**	A	Pd(PPh$_3$)$_4$ (10 mol%)	80	K_3PO_4	1,4-dioxane	30%
**8**	A	Pd(PPh$_3$)$_4$ (10 mol%)	80	K_3PO_4	1,4-dioxane:H_2O (1:1)	0
**9**	A	Pd(PPh$_3$)$_4$ (10 mol%)	80	KOAc	1,4-dioxane:H_2O (1:1)	<5%
**10**	A	Pd(PPh$_3$)$_4$ (10 mol%)	80	K_2CO_3	1,4-dioxane:H_2O (1:1)	<5%
**11**	A	Pd(OAc)$_2$ (10 mol%), dppf (10 mol%)	80	K_3PO_4	1,4-dioxane	<5%
**12**	A	Pd(OAc)$_2$ (10 mol%), PPh$_3$ (10 mol%)	80	K_3PO_4	1,4-dioxane	<5%
**13**	**A**	**PdCl$_2$amphos** (5 mol%)	**80**	**K_3PO_4**	**1,4-dioxane**	**82%**
**14**	C	PdCl$_2$amphos (5 mol%)	80	K_3PO_4	1,4-dioxane	0

Reactions were carried out on a 0.10 mmol scale. Yields were determined by ^{1}H NMR using 1,3,5-trimethoxybenzene (1.0 equiv.) as an internal standard.

General procedure for C–C bond formation of aryl thianthrenium salts

Under an ambient atmosphere, aryl thianthrenium salt (0.500 mmol, 1.00 equiv.), K_3PO_4 (212 mg, 1.00 mmol, 2.00 equiv.) and Pd catalyst (5.00 mol%) and boronic acid/ester (if solid, 0.550 mmol, 1.10 equiv.) were added to a 20-mL vial containing a Teflon-coated magnetic stirring bar. The vial was transferred to a nitrogen-filled glovebox and 1,4-dioxane (5.0 mL, *c* = 0.10 M) was added. The reaction vial was taken out of the glovebox, boronic acid/ester (if liquid, 0.550 mmol, 1.10 equiv.) was added by using a microsyringe under Ar atmosphere. The vial was sealed with a septum cap, and the reaction mixture was stirred (600 rpm) at 80 °C for 16 h on a heating block. After that, the reaction mixture was cooled to room temperature and transferred to a separatory funnel using ethyl acetate (15 mL) and

water (15 mL). The organic layer was separated from the aqueous layer, and the aqueous layer was further extracted with ethyl acetate (2 × 15 mL). The combined organic layers were dried over Na_2SO_4, filtered, and concentrated by rotary evaporation. The residue was purified by flash column chromatography on silica gel

(±)-Pyriproxyphen methyl derivative 88

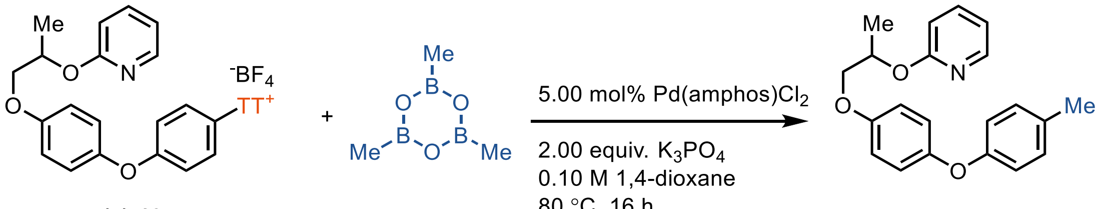

(±)-19a 1.00 equiv. 1.10 equiv. **(±)-88**, 64%

Under an ambient atmosphere, (±)-pyriproxyphen-derived thianthrenium salt **88a** (311 mg, 0.500 mmol, 1.00 equiv.), K_3PO_4 (212 mg, 1.00 mmol, 2.00 equiv.) and Pd(amphos)Cl_2 (17.7 mg, 25.0 µmol, 5.00 mol%) were added to a 20-mL vial containing a Teflon-coated magnetic stirring bar. The vial was transferred to a nitrogen-filled glovebox and 1,4-dioxane (5.0 mL, *c* = 0.10 M) was added. The reaction vial was taken out of the glovebox, trimethylboroxine (50% in THF, 154 µL, 138 mg, 0.550 mmol, 1.10 equiv.) was added by using a microsyringe under Ar atmosphere. The vial was sealed with a septum cap, and the reaction mixture was stirred (600 rpm) at 80 °C for 16 h on a heating block. After that, the reaction mixture was cooled to room temperature and transferred to a separatory funnel using ethyl acetate (15 mL) and water (15 mL). The organic layer was separated from the aqueous layer, and the aqueous layer was further extracted with ethyl acetate (2 × 15 mL). The combined organic layers were dried over Na_2SO_4, filtered, and concentrated by rotary evaporation. The residue was purified by flash column chromatography on silica gel, (hexanes / EtOAc = 10:1 to 5:1) to afford a mixture of **88** and pyriproxyphen (**19**). Further purification by HPLC (150 mm Eclipse plus C18, 5 µm, 4.6 mm, MeOH / H_2O = 80:50, flow rate = 1 mL/min, 308 K, retention time = 10.56 min) afforded 102 mg (64% yield) of the title compound **(±)-88** as a colorless oil.

R_f = 0.74 (hexanes / EtOAc = 1:1 (v/v))

NMR Spectroscopy:

^{1}H NMR (600 MHz, $CDCl_3$, 25 °C, δ): 8.17 (ddd, *J* = 5.0, 2.0, 0.8 Hz, 1H), 7.57 (ddd, *J* = 8.3, 7.1, 2.0 Hz, 1H), 7.18 – 7.06 (m, 2H), 6.99 – 6.90 (m, 4H), 6.90 – 6.83 (m, 3H), 6.76 (dt, *J* = 8.4, 0.9 Hz, 1H), 5.61 (ddd, *J* = 6.4, 5.3, 4.9 Hz, 1H), 4.20 (dd, *J* = 9.9, 5.3 Hz, 1H), 4.08 (dd, *J* = 9.9, 4.9 Hz, 1H), 2.33 (s, 3H), 1.50 (d, *J* = 6.4 Hz, 3H).

^{13}C NMR (151 MHz, $CDCl_3$, 25 °C, δ): 163.3, 156.2, 155.1, 151.0, 147.0, 138.8, 132.1, 130.2, 120.4, 117.9, 116.9, 115.8, 111.8, 71.2, 69.4, 20.7, 17.1.

HRMS ESI (m/z) calc'd for $C_{21}H_{22}NO_3$ $[M+H]^+$, 336.1594; found, 336.1590. deviation: –1.1 ppm.

(±)-Pyriproxyphen cyclopropyl derivative 89

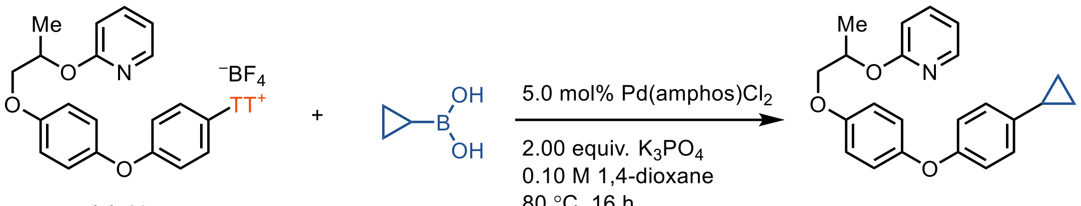

(±)-19a 1.00 equiv. 1.10 equiv. **(±)-89**, 59%

Under an ambient atmosphere, (±)-pyriproxyphen-derived thianthrenium salt **19a** (311 mg, 0.500 mmol, 1.00 equiv.), K_3PO_4 (212 mg, 1.00 mmol, 1.00 equiv.), Pd(amphos)Cl_2 (17.7 mg, 25.0 µmol, 5.00 mol%) and cyclopropyl boronic acid (47.2 mg, 0.550 mmol, 1.10 equiv) were added to a 20-mL vial containing a Teflon-coated magnetic stirring bar. The vial was transferred to a nitrogen-filled glovebox and 1,4-dioxane (5.0 mL, *c* = 0.10 M) was added. The vial was sealed with a cap, and removed from the glovebox. The reaction mixture was stirred (600 rpm) at 80 °C for 16 h on a heating block. After that, the reaction mixture was cooled to room temperature and transferred to a separatory funnel using ethyl acetate (15 mL) and water (15 mL). The organic layer was separated from the aqueous layer, and the aqueous layer was further extracted with ethyl acetate (2 × 15 mL). The combined organic layers were dried over Na_2SO_4, filtered, and concentrated by rotary evaporation. The residue was purified by flash column chromatography on silica gel (hexanes / EtOAc = 20:1 (v/v)) to afford a mixture of **89** and pyriproxyphen (**19**). Further purification by HPLC (150 mm Eclipse plus C18, 5 µm, 4.6 mm, MeOH / H_2O = 80:50 (v/v), flow rate = 1 mL/min, 308 K, retention time = 14.28 min) afforded 106 mg (59% yield) of the title compound **(±)-89** as colorless oil.

R_f = 0.79 (hexanes / EtOAc = 1:1 (v/v))

NMR Spectroscopy:

^{1}H NMR (600 MHz, $CDCl_3$, 25 °C, δ): 8.15 (ddd, *J* = 5.0, 2.0, 0.8 Hz, 1H), 7.57 (ddd, *J* = 8.4, 7.1, 2.0 Hz, 1H), 7.03 – 7.00 (m, 2H), 6.95 – 6.89 (m, 4H), 6.88 – 6.83 (m, 3H), 6.75 (dt, *J* = 8.4, 0.9 Hz, 1H), 5.63 – 5.54 (m, 1H), 4.18 (dd, *J* = 9.9, 5.3 Hz, 1H), 4.07 (dd, *J* = 9.9, 4.9 Hz, 1H), 1.87 (tt, *J* = 8.4, 5.1 Hz, 1H), 1.48 (d, *J* = 6.4 Hz, 3H), 0.95 – 0.90 (m, 2H), 0.66 – 0.62 (m, 2H).

^{13}C NMR (151 MHz, $CDCl_3$, 25 °C, δ): 163.3, 156.2, 155.1, 151.0, 146.9, 138.8, 138.2, 127.0, 120.4, 118.0, 116.9, 115.9, 111.8, 71.2, 69.4, 17.1, 14.9, 8.9.

HRMS GC-EI (m/z) calc'd for $C_{23}H_{24}NO_3$ $[M+H]^+$, 362.1751; found, 362.1747. deviation: +1.0 ppm.

Boscalid cyclopropyl derivative 90

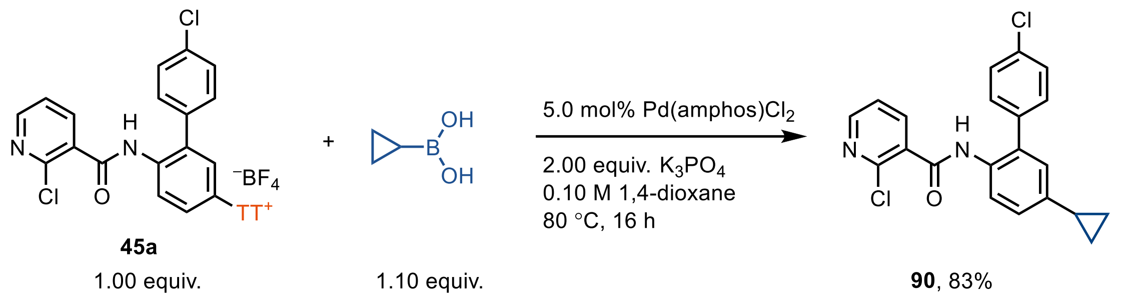

Under a nitrogen atmosphere, boscalid-derived thianthrenium salt **45a** (193 mg, 0.300 mmol, 1.00 equiv.), K_3PO_4 (127 mg, 2.00 mmol, 2.00 equiv.), Pd(amphos)Cl_2 (10.6 mg, 15.0 µmol, 5.00 mol%) and cyclopropyl boronic acid (30.9 mg, 0.330 mmol, 1.10 equiv.) were added to a 20-mL vial containing a Teflon-coated magnetic stirring bar. The vial was transferred to a nitrogen-filled glovebox and 1,4-dioxane (3.0 mL, *c* = 0.10 M) was added. The vial was sealed with a cap, and removed from the glovebox. The reaction mixture was stirred (600 rpm) at 80 °C for 16 h on a heating block. After that, the reaction mixture was cooled to room temperature and transferred to a separatory funnel using ethyl acetate (15 mL) and water (15 mL). The organic layer was separated from the aqueous layer, and the aqueous layer was further extracted with ethyl acetate (2 × 15 mL). The combined organic layers were dried over Na_2SO_4, filtered, and concentrated by rotary evaporation. The residue was purified by flash column chromatography on silica gel (hexanes / EtOAc = 15:1 to 1:1 (v/v)) to afford 95.0 mg (83% yield) of the title compound **90** as a colorless oil.

R_f = 0.48 (hexanes / EtOAc = 1:1 (v/v))

NMR Spectroscopy:

^{1}H NMR (500 MHz, $CDCl_3$, 25 °C, δ): 8.35 (dd, *J* = 4.7, 2.0 Hz, 1H), 8.15 (d, *J* = 8.5 Hz, 1H), 8.04 (dd, *J* = 7.7, 2.0 Hz, 1H), 7.99 (s, 1H), 7.38 – 7.31 (m, 2H), 7.29 – 7.22 (m, 3H), 7.06 (dd, *J* = 8.5, 2.2 Hz, 1H), 6.91 (d, *J* = 2.2 Hz, 1H), 1.85 (tt, *J* = 8.4, 5.1 Hz, 1H), 0.96 – 0.88 (m, 2H), 0.68 – 0.60 (m, 2H).

^{13}C NMR (126 MHz, $CDCl_3$, 25 °C, δ): 162.5, 151.4, 146.9, 141.5, 140.2, 136.7, 134.5, 132.6, 131.8, 131.3, 130.9, 129.4, 127.7, 126.0, 123.0, 122.5, 15.2, 9.4.

HRMS GC-EI (m/z) calc'd for $C_{21}H_{16}N_2OCl_2$ $[M]^+$, 382.0634; found, 382.0640. deviation: –1.6 ppm.

Boscalid methyl derivative 91

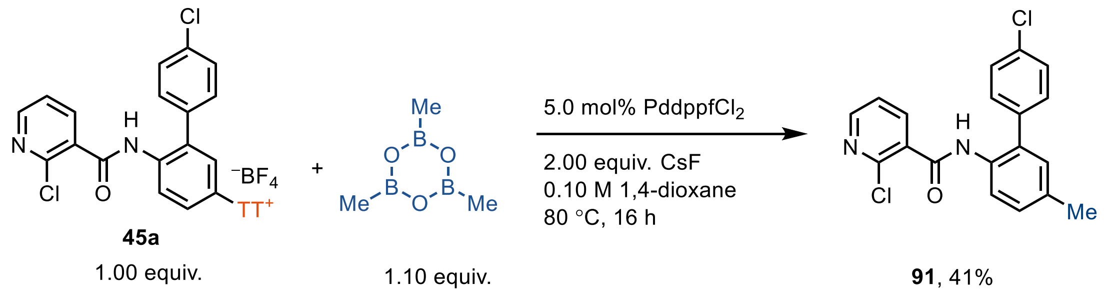

Under an ambient atmosphere, boscalid-derived thianthrenium salt **45a** (129 mg, 0.200 mmol, 1.00 equiv.), CsF (60.8 mg, 0.400 mmol, 2.00 equiv.) and $PddppfCl_2$ (7.3 mg, 10 μmol, 5.0 mol%) were added to a 4-mL vial containing a Teflon-coated magnetic stirring bar. The vial was transferred to a nitrogen-filled glovebox and 1,4-dioxane (2.0 mL, *c* = 0.10 M) was added. The reaction vial was taken out of the glovebox, trimethylboroxine (50% in THF, 62 μL, 55 mg, 0.22 mmol, 1.10 equiv.), was added by using a microsyringe. The reaction mixture was stirred (600 rpm) at 80 °C for 16 h on a heating block. After that, the reaction mixture was cooled to room temperature and transferred to a separatory funnel using ethyl acetate (10 mL) and water (10 mL). The organic layer was separated from the aqueous layer, and the aqueous layer was further extracted with ethyl acetate (2 × 10 mL). The combined organic layers were dried over Na_2SO_4, filtered, and concentrated by rotary evaporation. The residue was purified by flash column chromatography on silica gel, (hexanes / EtOAc = 10:1 to 1:1 (v/v)) to afford 29.3 mg of the title compound **91** as a colorless oil (41% yield).

R_f = 0.53 (hexanes / EtOAc = 1:1 (v/v)).

NMR Spectroscopy:

^{1}H NMR (500 MHz, $CDCl_3$, 25 °C, δ): δ 8.61 (dd, *J* = 4.8, 2.0 Hz, 1H), 8.41 (d, *J* = 8.3 Hz, 1H), 8.29 (dd, *J* = 7.7, 2.0 Hz, 1H), 8.24 (bs, 1H), 7.64 – 7.56 (m, 2H), 7.54 – 7.47 (m, 3H), 7.44 (q, *J* = 2.9 Hz, 2H), 2.56 (s, 3H).

^{13}C NMR (126 MHz, $CDCl_3$, 25 °C, δ): 162.6, 151.4, 146.9, 140.2, 136.6, 135.4, 134.4, 132.6, 131.9, 131.3, 130.9, 130.9, 129.5, 129.3, 123.0, 122.5, 21.1.

HRMS GC-EI (m/z) calc'd for $C_{19}H_{14}N_2OCl_2$ $[M]^+$, 356.0478; found, 356.0485. deviation: –1.9 ppm.

(±)-Fenoprofen methyl ester methyl derivative 92

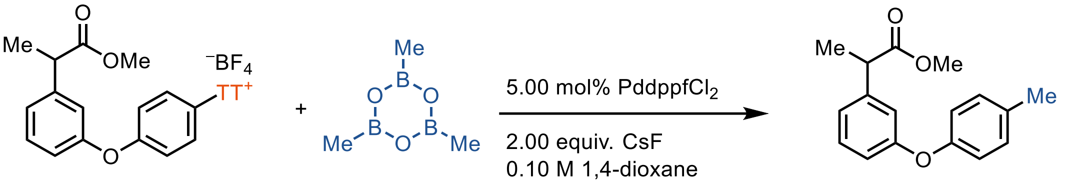

(±)-10a 1.00 equiv. | 1.10 equiv. | (±)-92, 73%

Under an ambient atmosphere, (±)-fenoprofen methyl ester-derived thianthrenium salt **(±)-10a** (229 mg, 0.500 mmol, 1.00 equiv.), CsF (152 mg, 1.00 mmol, 2.00 equiv.) and $Pd(dppf)Cl_2$ (18.3 mg, 25.0 μmol, 5.00 mol%) were added to a 20-mL vial containing a Teflon-coated magnetic stirring bar. The vial was transferred to a nitrogen-filled glovebox and 1,4-dioxane (5.0 mL, *c* = 0.10 M) was added. The reaction vial was taken out of the glovebox, trimethylboroxine (50% in THF, 154 µL, 138 mg, 0.550 mmol, 1.10 equiv.) was added by using a microsyringe under Ar atmosphere. The vial was sealed with a cap, and the reaction mixture was stirred (600 rpm) at 80 °C for 16 h on a heating block. After that, the reaction mixture was cooled to room temperature and transferred to a separatory funnel using ethyl acetate (15 mL) and water (15 mL). The organic layer was separated from the aqueous layer, and the aqueous layer was further extracted with ethyl acetate (2 × 15 mL). The combined organic layers were dried over Na_2SO_4, filtered, and concentrated by rotary evaporation. The residue was

purified by flash column chromatography on silica gel (hexanes / EtOAc = 50:1 to 20:1 to 10:1 (v/v)) to afford 98.0 mg (73% yield) of the title compound **(±)-92** as colorless oil.

R*f* = 0.43 (hexanes / EtOAc = 10:1).

NMR Spectroscopy:

^{1}H NMR (500 MHz, $CDCl_3$, 25 °C, δ): 7.28 (t, *J* = 7.9 Hz, 1H), 7.21 – 7.14 (m, 2H), 7.04 (dt, *J* = 7.7, 1.3 Hz, 1H), 6.99 (t, *J* = 2.1 Hz, 1H), 6.98 – 6.93 (m, 2H), 6.87 (ddd, *J* = 8.2, 2.5, 1.0 Hz, 1H), 3.75 – 3.69 (m, 4H), 2.37 (s, 3H), 1.51 (d, *J* = 7.2 Hz, 3H).

^{13}C NMR (126 MHz, $CDCl_3$, 25 °C, δ): 174.8, 158.1, 154.6, 142.5, 133.1, 130.4, 129.9, 121.9, 119.3, 117.7, 116.9, 52.2, 45.4, 20.8, 18.6.

HRMS GC-EI (m/z) calc'd for $C_{17}H_{18}O_3$ $[M]^+$, 270.1251; found, 270.1255. deviation: –1.7 ppm.

Phenyl pyrazole methyl derivative 93

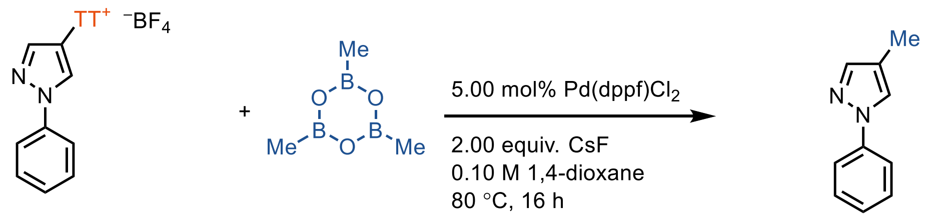

25a 1.00 equiv. 1.10 equiv. **93**, 49%

Under an ambient atmosphere, phenylpyrazole-derived thianthrenium salt **25a** (156 mg, 0.350 mmol, 1.00 equiv.), CsF (106 mg, 0.700 mmol, 2.00 equiv.) and $Pd(dppf)Cl_2$ (12.3 mg, 17.5 µmol, 5.00 mol%) were added to a 20-mL vial containing a Teflon-coated magnetic stirring bar. The vial was transferred to a nitrogen-filled glovebox and 1,4-dioxane (3.5 mL, *c* = 0.10 M) was added. The reaction vial was taken out of the glovebox, trimethylboroxine (50% in THF, 108 µL, 96.6 mg, 0.385 mmol, 1.10 equiv.), was added by using a microsyringe under Ar atmosphere. The vial was sealed with a cap, and the reaction mixture was stirred vigorously at 80 °C for 16 h on a heating block. After that, the reaction mixture was cooled to room temperature and transferred to a separatory funnel using ethyl acetate (15 mL) and water (15 mL). The organic layer was separated from the aqueous layer, and the aqueous layer was further extracted with ethyl acetate (2 × 15 mL). The combined organic layers were dried over Na_2SO_4, filtered, and concentrated by rotary evaporation. The residue was purified by chromatography on silica gel (hexanes / EtOAc (v/v)) to afford a mixture of **93** and protodefunctionalization product (**25**, phenyl pyrazole). Further purification by HPLC (150 mm Eclipse plus C18, 1.8 µm, 3.20 mm, MeOH in H_2O 70% to 90%, flow rate = 0.5 mL/min, 308 K, retention time = 1.32 min) afforded 27.3 mg (49% yield) of the title compound **93** as a colorless oil.

R*f* = 0.55 (hexanes / EtOAc = 10:1 (v/v))

NMR Spectroscopy:

^{1}H NMR (600 MHz, $CDCl_3$, 25 °C, δ): 7.72 – 7.68 (m, 1H), 7.66 – 7.63 (m, 2H), 7.53 (s, 1H), 7.45 – 7.41 (m, 2H), 7.27 – 7.23 (m, 1H), 2.16 (t, *J* = 0.8 Hz, 3H).

^{13}C NMR (151 MHz, $CDCl_3$, 25 °C, δ): 141.9, 140.4, 129.5, 126.1, 125.5, 118.9, 118.3, 9.1.

HRMS GC-EI (m/z) calc'd for $C_{10}H_{10}N_2$ $[M]^+$, 158.0838; found, 158.0839. deviation: –0.3 ppm.

Phenyl piperazine-derivative cyclopropyl derivative 94

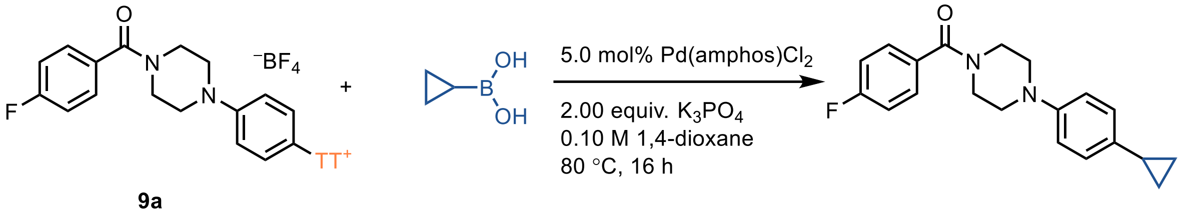

9a 1.00 equiv. 1.10 equiv. **94**, 60%

Under an ambient atmosphere, thianthrenium salt **9a** (293 mg, 0.500 mmol, 1.00 equiv.), K_3PO_4 (212 mg, 1.00 mmol, 2.00 equiv.), Pd(amphos)Cl_2 (35.4 mg, 50.0 μmol, 5.00 mol%) and cyclopropyl boronic acid (51.5 mg, 0.600 mmol, 1.10 equiv.) were added to a 20-mL vial containing a Teflon-coated magnetic stirring bar. The vial was transferred to a nitrogen-filled glovebox and 1,4-dioxane (5.0 mL, *c* = 0.10 M) was added. The vial was sealed with a cap, and removed from the glovebox. The reaction mixture was stirred (600 rpm) at 80 °C for 16 h on a heating block. After that, the reaction mixture was cooled to room temperature and transferred to a separatory funnel using ethyl acetate (15 mL) and water (15 mL). The organic layer was separated from the aqueous layer, and the aqueous layer was further extracted with ethyl acetate (2 × 15 mL). The combined organic layers were dried over Na_2SO_4, filtered, and concentrated by rotary evaporation. Purification by HPLC (50 mm Eclipse plus C18, 1.8 μm, 4.60 mm, MeOH in H_2O 70%, flow rate = 1.0 mL/min, 308 K, retention time = 2.97 min) afforded 98.0 mg (60% yield) of the title compound **94** as a colorless solid.

NMR Spectroscopy:

^{1}H NMR (600 MHz, $CDCl_3$, 25 °C, δ): 7.47 – 7.42 (m, 2H), 7.14 – 7.08 (m, 2H), 7.04 – 6.99 (m, 2H), 6.87 – 6.83 (m, 2H), 3.37 (m, 8H), 1.84 (tt, *J* = 8.4, 5.1 Hz, 1H), 1.00 – 0.78 (m, 2H), 0.69 – 0.48 (m, 2H).

^{13}C NMR (151 MHz, $CDCl_3$, 25 °C, δ): 169.6, 163.6 (d, *J* = 250.2 Hz), 149.0, 136.5, 131.8 (d, *J* = 3.5 Hz), 129.6 (d, *J* = 8.5 Hz), 126.7, 115.7 (d, *J* = 21.8 Hz), 50.4, 47.9, 42.5, 14.7, 8.7.

^{19}F NMR (565 MHz, $CDCl_3$, 25 °C, δ): –110.1.

HRMS GC-EI (m/z) calc'd for $C_{20}H_{21}N_2OF$ $[M]^+$, 324.1632; found, 324.1635. deviation: –0.8 ppm.

Pyriproxyphen phenyl derivative 95

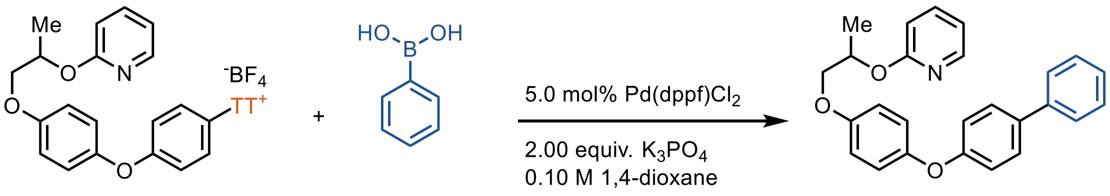

(±)-19a 1.00 equiv.; 1.10 equiv.; **(±)-95**, 95%

Under an ambient atmosphere, pyriproxyphen-derived thianthrenium salt **(±)-19a** (62.3 mg, 0.100 mmol, 1.00 equiv.), K_3PO_4 (42.5 mg, 2.00 mmol, 2.00 equiv.), $Pd(dppf)Cl_2$ (3.5 mg, 5.0 µmol, 5.0 mol%) and phenyl boronic acid (13.4 mg, 0.110 mmol, 1.10 equiv.) were added to a 4-mL vial containing a Teflon-coated magnetic stirring bar. The vial was transferred to a nitrogen-filled glovebox and 1,4-dioxane (1.0 mL, *c* = 0.10 M) was added. The vial was sealed with a septum cap, and removed from the glovebox. The reaction mixture was stirred (600 rpm) vigorously at 80 °C for 16 h on a heating block. After that, the reaction mixture was cooled to room temperature and transferred to a separatory funnel using ethyl acetate (10 mL) and water (10 mL). The organic layer was separated from the aqueous layer, and the aqueous layer was further extracted with ethyl acetate (2 × 10 mL). The combined organic layers were dried over Na_2SO_4, filtered, and concentrated by rotary evaporation. The residue was purified by flash column chromatography on silica gel (hexanes / EtOAc = 20:1 to 10:1 (v/v)) to afford 37.6 mg (95% yield) of the title compound **(±)-95** as a colorless oil.

R*f* = 0.41 (hexanes / EtOAc = 10:1 (v/v)).

NMR Spectroscopy:

^{1}H NMR (600 MHz, $CDCl_3$, 25 °C, δ): 8.17 (ddd, *J* = 5.0, 2.0, 0.8 Hz, 1H), 7.60 – 7.51 (m, 5H), 7.47 – 7.39 (m, 2H), 7.36 – 7.30 (m, 1H), 7.05 – 6.99 (m, 4H), 6.99 – 6.93 (m, 2H), 6.87 (ddd, *J* = 7.1, 5.0, 1.0 Hz, 1H), 6.76 (dt, *J* = 8.4, 0.9 Hz, 1H), 5.61 (qt, *J* = 6.4, 5.1 Hz, 1H), 1.51 (d, *J* = 6.4 Hz, 3H).

^{13}C NMR (151 MHz, $CDCl_3$, 25 °C, δ): 163.3, 158.2, 155.5, 150.3, 146.9, 140.8, 138.8, 135.6, 128.9, 128.4, 127.0, 128.0, 121.0, 117.9, 116.9, 116.0, 111.8, 71.2, 69.4, 17.2.

HRMS ESI (m/z) calc'd for $C_{26}H_{24}NO_3$ $[M+H]^+$, 398.1751; found, 398.1748. deviation: +0.6 ppm.

(±)-Pyriproxyphen pyridyl derivative 96

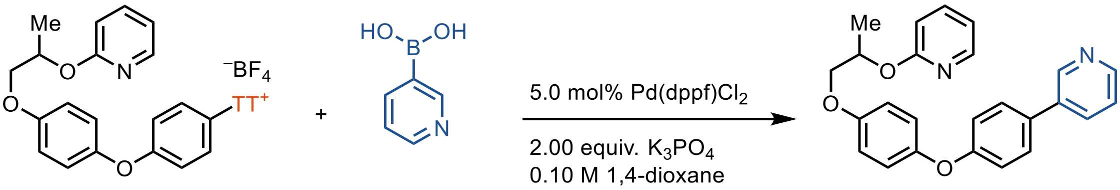

(±)-19a 1.00 equiv.; 1.10 equiv.; **(±)-96**, 97%

Under an ambient atmosphere, (±)-pyriproxyphen-derived thianthrenium salt **(±)-19a** (187 mg, 0.300

mmol, 1.00 equiv.), K_3PO_4 (127 mg, 0.600 mmol, 2.00 equiv.), Pd(dppf)Cl_2 (11 mg, 15 μmol, 5.0 mol%) and pyridine-3-boronic acid (40.6 mg, 0.330 mmol, 1.10 equiv.) were added to a 20-mL vial containing a Teflon-coated magnetic stirring bar. The vial was transferred to a nitrogen-filled glovebox and 1,4-dioxane (3.0 mL, *c* = 0.10 M) was added. The vial was sealed with a septum cap, and removed from the glovebox. The reaction mixture was stirred (600 rpm) at 80 °C for 16 h on a heating block. After that, the reaction mixture was cooled to room temperature and transferred to a separatory funnel using ethyl acetate (15 mL) and water (15 mL). The organic layer was separated from the aqueous layer, and the aqueous layer was further extracted with ethyl acetate (2 × 15 mL). The combined organic layers were dried over Na_2SO_4, filtered, and concentrated by rotary evaporation. The residue was purified by flash column chromatography on silica gel, (hexanes / EtOAc = 15:1 to 1:1 (v/v)) to afford 116 mg (97% yield) of the title compound **(±)-96** as a colorless oil.

Note: When the reaction was performed on 1.0 mmol scale with 1 mol% of Pd(dppf)Cl_2, the reaction yield was found to be 90%. The yield was determined by ^{1}H NMR after aqueous workup by using 1,3,5-trimethoxybenzene as an internal standard.

R*f* = 0.49 (hexanes / EtOAc = 1:1 (v/v))

NMR Spectroscopy:

^{1}H NMR (600 MHz, $CDCl_3$, 25 °C, δ): 8.83 (bs, 1H), 8.57 (bs, 1H), 8.15 (ddd, *J* = 5.0, 2.0, 0.8 Hz, 1H), 7.83 (d, *J* = 7.9 Hz, 1H), 7.55 (ddd, *J* = 8.3, 7.1, 2.0 Hz, 1H), 7.49 (d, *J* = 8.7 Hz, 2H), 7.35 (bs, 1H), 7.05 – 6.98 (m, 4H), 6.97 – 6.92 (m, 2H), 6.85 (ddd, *J* = 7.1, 5.0, 1.0 Hz, 1H), 6.74 (dt, *J* = 8.3, 0.9 Hz, 1H), 5.64 – 5.56 (m, 1H), 4.20 (dd, *J* = 9.9, 5.3 Hz, 1H), 4.08 (dd, *J* = 9.9, 4.9 Hz, 1H), 1.48 (d, 3H)

^{13}C NMR (151 MHz, $CDCl_3$, 25 °C, δ): 163.2, 159.0, 155.6, 149.8, 147.9 (2 peaks overlapping), 146.9, 146.9, 138.8, 136.3, 134.2, 131.9, 128.5, 121.1, 118.0, 116.9, 116.0, 111.7, 71.1, 69.3, 17.1.

HRMS ESI (m/z) calc'd for $C_{25}H_{23}N_2O_3$ $[M+H]^+$, 399.1703; found, 399.1704. deviation: –0.3 ppm.

Procedure by using Schlenk line: To a a 4-mL borosilicate vial containing a Teflon-coated magnetic stirring bar were added (±)-pyriproxyphen-derived thianthrenium salt **(±)-19a** (62.3 mg, 0.100 mmol, 1.00 equiv.), K_3PO_4 (42.5 mg, 0.200 mmol, 2.00 equiv.), Pd(dppf)Cl_2 (0.7 mg, 1 μmol, 5 mol%) and pyridine-3-boronic acid (14.7 mg, 0.110 mmol, 1.10 equiv.). After attaching a Schlenk-line adapter, the vial was evacuated and backfilled with argon three times using a Schlenk line. Degassed 1,4-dioxane (1.0 mL, *c* = 0.10 M) was then added under Ar flow. The vial was sealed, and the reaction mixture was stirred vigorously (600 rpm) at 80 °C for 16 h on a heating block. After that, the reaction mixture was cooled to room temperature. 1,3,5-trimethoxybenzene (16.8 mg, 0.100 mmol, 1.00 equiv.) was added into the reaction mixture as an internal standard, followed by EtOAc (2.0 mL). An aliquot (ca. 0.5 mL) from the EtOAc phase was taken and the concentrated by rotary evaporation. The residue was dissolved in $CDCl_3$ (0.6 mL), and the ^{1}H NMR was recorded. The yield (96%) was determined from the ^{1}H NMR spectrum by integrating the peaks of the internal standard (6.11 ppm) and the product (8.15 ppm).

Boscalid pyridiyl derivative 97

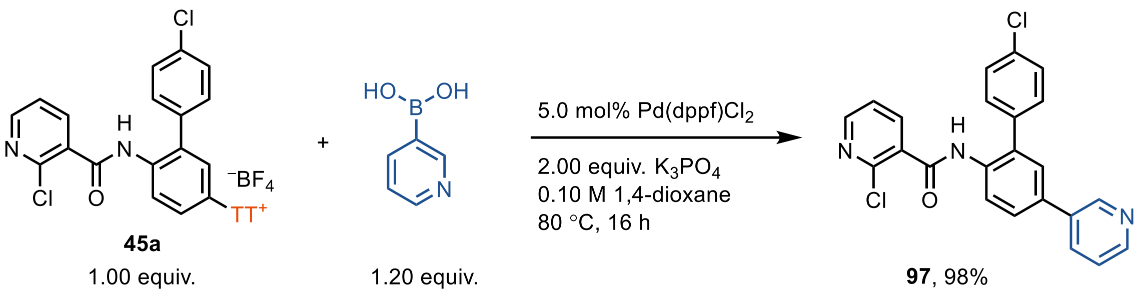

Under an ambient atmosphere, boscalid-derived thianthrenium salt **45a** (64.5 mg, 0.100 mmol, 1.00 equiv.), K_3PO_4 (42.5 mg, 0.200 mmol, 2.00 equiv.), Pd(dppf)Cl_2 (3.7 mg, 5.0 µmol, 5.0 mol%) and pridine-3-boronic acid (14.8 mg, 0.120 mmol, 1.20 equiv.) were added to a 4-mL vial containing a Teflon-coated magnetic stirring bar. The vial was transferred to a nitrogen-filled glovebox and 1,4-dioxane (1.0 mL, *c* = 0.10 M) was added. The vial was sealed with a septum cap, and removed from the glovebox. The reaction mixture was stirred (600 rpm) at 80 °C for 16 h on a heating block. After that, the reaction mixture was cooled to room temperature and transferred to a separatory funnel using ethyl acetate (10 mL) and water (10 mL). The organic layer was separated from the aqueous layer, and the aqueous layer was further extracted with ethyl acetate (2 × 10 mL). The combined organic layers were dried over Na_2SO_4, filtered, and concentrated by rotary evaporation. The residue was purified by flash column chromatography on silica gel (hexanes / EtOAc = 1:1 to 1:4 (v/v)) to afford 40.1 mg (98% yield) of the title compound **97** as a colorless oil.

R*f* = 0.41 (EtOAc only)

NMR Spectroscopy:

^{1}H NMR (500 MHz, $CDCl_3$, 25 °C, δ): 8.86 (s, 1H), 8.59 (d, *J* = 8.2 Hz, 2H), 8.47 (dd, *J* = 4.7, 2.0 Hz, 1H), 8.41 (s, 1H), 8.19 (dd, *J* = 7.6, 2.0 Hz, 1H), 7.92 (dt, *J* = 7.9, 1.9 Hz, 1H), 7.69 (dd, *J* = 8.5, 2.2 Hz, 1H), 7.52 – 7.46 (m, 3H), 7.44 – 7.36 (m, 4H).

^{13}C NMR (126 MHz, $CDCl_3$, 25 °C, δ): 162.7, 151.6, 148.7, 148.2, 146.8, 140.4, 135.9, 135.7 135.0, 134.8, 134.7, 134.3, 133.0, 131.0, 130.9, 129.6, 128.9, 127.6, 123.8, 123.1, 122.7.

HRMS ESI (m/z) calc'd for $C_{23}H_{16}N_3OCl_2$ $[M+H]^+$, 420.0665; found, 420.0665. deviation: –0.4 ppm.

Phenyl piperazine-derivative pyridyl derivative 98

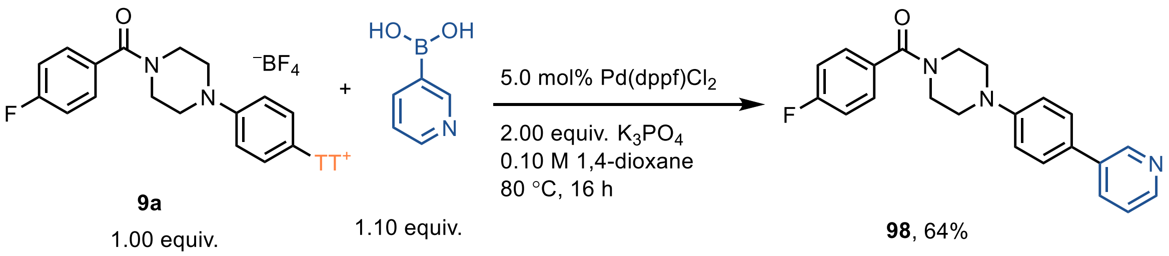

Under an ambient atmosphere, thianthrenium salt **9a** (58.6 mg, 0.100 mmol, 1.00 equiv.), K_3PO_4 (42.5 mg, 0.200 mmol, 2.00 equiv.), Pd(dppf)Cl_2 (3.7 mg, 5.0 µmol, 5.0 mol%) and pyridine-3-boronic acid

(13.5 mg, 0.110 mmol, 1.10 equiv.) were added to a 4-mL vial containing a Teflon-coated magnetic stirring bar. The vial was transferred to a nitrogen-filled glovebox and 1,4-dioxane (1.0 mL, *c* = 0.10 M) was added. The vial was sealed with a septum cap, and removed from the glovebox. The reaction mixture was stirred (600 rpm) at 80 °C for 16 h on a heating block. After that, the reaction mixture was cooled to room temperature and transferred to a separatory funnel using ethyl acetate (10 mL) and water (10 mL). The organic layer was separated from the aqueous layer, and the aqueous layer was further extracted with ethyl acetate (2 × 10 mL). The combined organic layers were dried over Na_2SO_4, filtered, and concentrated by rotary evaporation. The residue was purified by flash column chromatography on silica gel (hexanes / EtOAc = 1:1 (v/v) to EtOAc only) to afford 23.0 mg (64% yield) of the title compound **98** as a colorless solid.

NMR Spectroscopy:

^{1}H NMR (600 MHz, $CDCl_3$, 25 °C, δ): 8.81 (dd, *J* = 2.4, 0.9 Hz, 1H), 8.53 (dd, *J* = 4.8, 1.6 Hz, 1H), 7.83 (ddd, *J* = 7.9, 2.4, 1.6 Hz, 1H), 7.54 – 7.50 (m, 2H), 7.49 – 7.44 (m, 2H), 7.32 (ddd, *J* = 7.9, 4.8, 0.8 Hz, 1H), 7.12 (t, *J* = 8.6 Hz, 2H), 7.04 – 6.99 (m, 2H), 3.93 (s, 0H), 3.60 (m, 8H).

^{13}C NMR (151 MHz, $CDCl_3$, 25 °C, δ): 169.7, 163.7 (d, *J* = 250.4 Hz), 150.8, 148.0, 148.0, 136.2, 133.8, 131.6 (d, *J* = 3.5 Hz), 129.7, 129.6 (d, *J* = 8.3 Hz), 128.1, 123.6, 115.8 (d, *J* = 21.5 Hz), 49.4, 47.7, 42.3.

^{19}F NMR (565 MHz, $CDCl_3$, 25 °C, δ): –109.8.

HRMS ESI (m/z) calc'd for $C_{22}H_{21}N_3OF$ $[M+H]^+$, 362.1663; found, 362.1666. deviation: –0.7 ppm.

Sulfonylation of aryl thianthrenium salts

General procedure for sulfonylation of aryl thianthrenium salts

Under an ambient atmosphere, aryl thianthrenium salt (62.3 mg, 0.100 mmol, 1.00 equiv.), Cs_2CO_3 (0.150 mmol, 1.50 equiv.), $Pd_2(dba)_3$ (9.2 mg, 10 μmol, 10 mol%), Xantphos (12 mg, 20 μmol, 20 mol%) and phenyl(methyl)sulfinate sodium salt (0.110 mmol, 1.10 equiv.) were added to a 4-mL vial containing a magnetic stirring bar. The vial was transferred to a nitrogen-filled glovebox and 1,4-dioxane (1.0 mL, *c* = 0.10 M) was added. The vial was sealed with a septum cap, and removed from the glovebox. The reaction mixture was stirred (600 rpm) at 80 °C for 16 h on a heating block. After that, the reaction mixture was cooled to room temperature and transferred to a separatory funnel using ethyl acetate (2 mL) and water (2 mL). The organic layer was separated from the aqueous layer, and the aqueous layer was further extracted with ethyl acetate (2 × 2 mL). The combined organic layers were dried over Na_2SO_4, filtered, and concentrated by rotary evaporation. The residue was purified by flash column chromatography on silica gel.

(±)-Pyriproxyphen methyl sulfone derivative 99

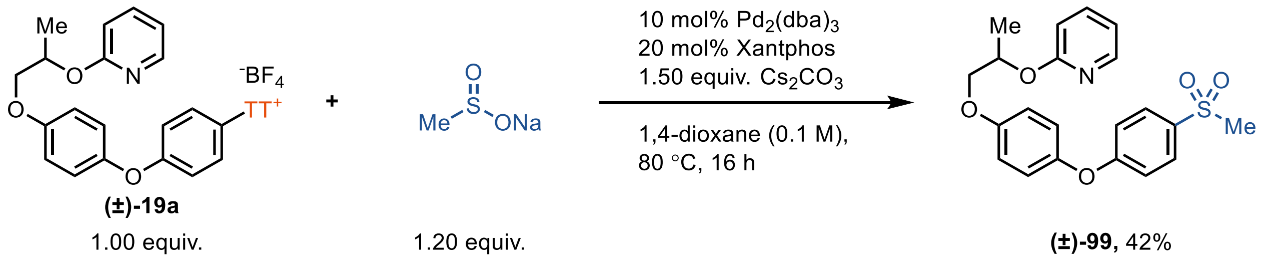

Under an ambient atmosphere, (±)-pyriproxyphen-derived thianthrenium salt **(±)-19a** (62.3 mg, 0.100 mmol, 1.00 equiv.), Cs_2CO_3 (48.9 mg, 0.150 mmol, 1.50 equiv.), $Pd_2(dba)_3$ (9.2 mg, 10 µmol, 10 mol%), Xantphos (12 mg, 20 µmol, 20 mol%) and methylsulfinate sodium salt (12.3 mg, 0.110 mmol, 1.10 equiv.) were added to a 4-mL vial containing a magnetic stirring bar. The vial was transferred to a nitrogen-filled glovebox and 1,4-dioxane (1.0 mL, *c* = 0.10 M) was added. The vial was sealed with a septum cap, and removed from the glovebox. The reaction mixture was stirred (600 rpm) at 80 °C for 16 h on a heating block. After that, the reaction mixture was cooled to room temperature and transferred to a separatory funnel using ethyl acetate (2 mL) and water (2 mL). The organic layer was separated from the aqueous layer, and the aqueous layer was further extracted with ethyl acetate (2 × 2 mL). The combined organic layers were dried over Na_2SO_4, filtered, and concentrated by rotary evaporation. The residue was purified by flash column chromatography on silica gel (hexanes / EtOAc = 10:1 to 1:1 (v/v)) to afford 16.6 mg (42% yield) of the title compound **(±)-99** as a colorless oil.

R*f* = 0.14 (hexanes / EtOAc = 5:1 (v/v))

NMR Spectroscopy:

^{1}H NMR (600 MHz, $CDCl_3$, 25 °C, δ): 8.15 (ddd, *J* = 5.1, 2.0, 0.8 Hz, 1H), 7.88 – 7.81 (m, 2H), 7.57 (ddd, *J* = 8.3, 7.1, 2.0 Hz, 1H), 7.05 – 6.93 (m, 6H), 6.87 (ddd, *J* = 7.1, 5.0, 1.0 Hz, 1H), 6.75 (dt, *J* = 8.4, 0.9 Hz, 1H), 5.60 (dt, *J* = 6.4, 5.0 Hz, 1H), 4.21 (dd, *J* = 9.9, 5.4 Hz, 1H), 4.10 (dd, *J* = 9.9, 4.9 Hz, 1H), 3.04 (s, 3H), 1.49 (d, *J* = 6.4 Hz, 3H).

^{13}C NMR (151 MHz, $CDCl_3$, 25 °C, δ): 163.5, 163.2, 156.5, 148.3, 146.9, 138.9, 133.7, 129.8, 121.9, 117.08, 118.0, 116.3, 111.8, 71.2, 69.3, 45.0, 17.1.

HRMS GC-ESI (m/z) calc'd for $C_{21}H_{22}NO_5S$ $[M+H]^+$, 400.1213; found, 400.1217. deviation: –0.9 ppm.

(±)-Pyriproxyphen phenyl sulfone derivative 100

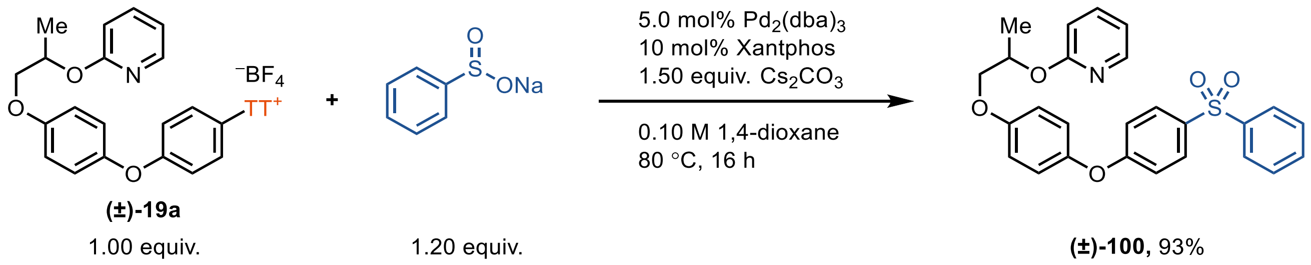

Under an ambient atmosphere, (±)-pyriproxyphen-derived thianthrenium salt **(±)-19a** (62.3 mg, 0.100 mmol, 1.00 equiv.), Cs_2CO_3 (48.9 mg, 0.150 mmol, 1.50 equiv.), $Pd_2(dba)_3$ (4.6 mg, 5.0 µmol, 5.0

mol%), Xantphos (5.8 mg, 10 μmol, 10 mol%) and phenylsulfinate sodium salt (19.7 mg, 0.120 mmol, 1.20 equiv.) were added to a 4-mL vial containing a magnetic stirring bar. The vial was transferred to a nitrogen-filled glovebox and 1,4-dioxane (1.0 mL, *c* = 0.10 M) was added. The vial was sealed with a septum cap, and removed from the glovebox. The reaction mixture was stirred (600 rpm) at 80 °C for 16 h on a heating block. After that, the reaction mixture was cooled to room temperature and transferred to a separatory funnel using ethyl acetate (2 mL) and water (2 mL). The organic layer was separated from the aqueous layer, and the aqueous layer was further extracted with ethyl acetate (2 × 2 mL). The combined organic layers were dried over Na_2SO_4, filtered, and concentrated by rotary evaporation. The residue was purified by flash column chromatography on silica gel (hexanes / EtOAc = 10:1 to 1:1 (v/v)) to afford 41.0 mg (88% yield) of the title compound **(±)-100** as a colorless oil.

R_f = 0.66 (hexanes / EtOAc = 2:1 (v/v))

NMR Spectroscopy:

^{1}H NMR (600 MHz, $CDCl_3$, 25 °C, δ): 8.15 (ddd, *J* = 5.1, 2.0, 0.8 Hz, 1H), 7.95 – 7.90 (m, 2H), 7.87 – 7.80 (m, 2H), 7.60 – 7.52 (m, 2H), 7.51 – 7.45 (m, 2H), 6.97 – 6.93 (m, 6H), 6.86 (ddd, *J* = 7.1, 5.0, 1.0 Hz, 1H), 6.74 (dt, *J* = 8.3, 0.9 Hz, 1H), 5.68 – 5.44 (m, 1H), 4.20 (dd, *J* = 9.8, 5.3 Hz, 1H), 4.08 (dd, *J* = 9.9, 4.8 Hz, 1H), 1.48 (d, *J* = 6.4 Hz, 3H).

^{13}C NMR (151 MHz, $CDCl_3$, 25 °C, δ): 163.2, 163.1, 156.4, 148.2, 146.9, 142.3, 138.9, 134.6, 133.1, 130.0, 129.4, 127.6, 121.9, 117.1, 117.0, 116.2, 111.8, 71.1, 69.3, 17.1.

HRMS GC-ESI (m/z) calc'd for $C_{26}H_{24}NO_5SNa$ $[M+Na]^+$, 484.1189; found, 484.1190. deviation: –0.1 ppm.

Procedure by using Schlenk line: To a a 4-mL borosilicate vial containing a Teflon-coated magnetic stirring bar were added (±)-pyriproxyphen-derived thianthrenium salt **(±)-19a** (62.3 mg, 0.100 mmol, 1.00 equiv.), Cs_2CO_3 (48.9 mg, 0.150 mmol, 1.50 equiv.), $Pd_2(dba)_3$ (4.6 mg, 5.0 μmol, 5.0 mol%), Xantphos (5.8 mg, 10 μmol, 10 mol%) and phenylsulfinate sodium salt (19.7 mg, 0.120 mmol, 1.20 equiv.) After attaching a Schlenk-line adapter, the vial was evacuated and backfilled with argon three times using a Schlenk line. Degassed 1,4-dioxane (1.0 mL, *c* = 0.10 M) was then added under Ar flow. The vial was sealed, and the reaction mixture was stirred vigorously (600 rpm) at 80 °C for 16 h on a heating block. After that, the reaction mixture was cooled to room temperature. 1,3,5-trimethoxybenzene (16.8 mg, 0.100 mmol, 1.00 equiv.) was added into the reaction mixture as an internal standard, followed by EtOAc (2.0 mL). An aliquot (ca. 0.5 mL) from the EtOAc phase was taken and the concentrated by rotary evaporation. The residue was dissolved in $CDCl_3$ (0.6 mL), and the ^{1}H NMR was recorded. The yield (96%) was determined from the ^{1}H NMR spectrum by integrating the signal of the internal standard at 6.11 ppm (s, 3H) and the product **100** at 6.74 ppm (dt, *J* = 8.3, 0.9 Hz, 1H).

Boscalid phenyl sulfone derivative 101

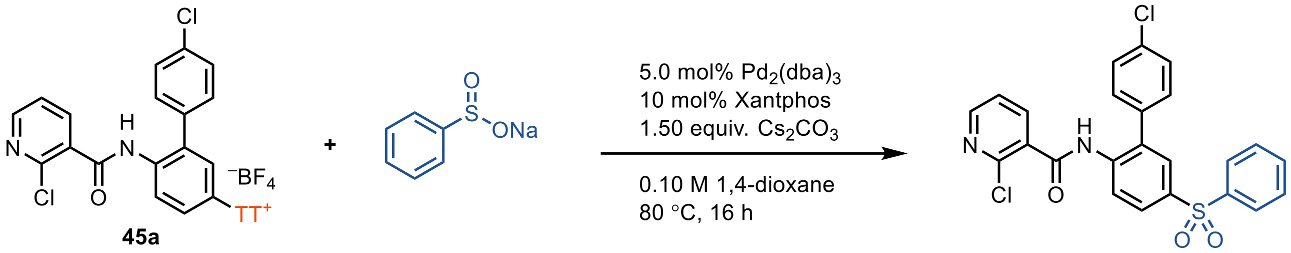

1.00 equiv. 1.20 equiv. **101,** 63%

Under an ambient atmosphere, boscalid-derived thianthrenium salt **45a** (64.5 mg, 0.100 mmol, 1.00 equiv.), Cs_2CO_3 (48.9 mg, 0.150 mmol, 1.50 equiv.), $Pd_2(dba)_3$ (4.6 mg, 5.0 μmol, 5.0 mol%), Xantphos (5.8 mg, 10 μmol, 10 mol%), and phenylsulfinate sodium salt (19.7 mg, 0.120 mmol, 1.20 equiv.) were added to a 4-mL vial containing a magnetic stirring bar. The vial was transferred to a nitrogen-filled glovebox and 1,4-dioxane (1.0 mL, *c* = 0.10 M) was added. The vial was sealed with a septum cap, and removed from the glovebox. The reaction mixture was stirred (600 rpm) at 80 °C for 16 h on a heating block. After that, the reaction mixture was cooled to room temperature and transferred to a separatory funnel using ethyl acetate (2 mL) and water (2 mL). The organic layer was separated from the aqueous layer, and the aqueous layer was further extracted with ethyl acetate (2 × 2 mL). The combined organic layers were dried over Na_2SO_4, filtered, and concentrated by rotary evaporation. The residue was purified by chromatography on silica gel (hexanes / EtOAc = 15:1 (v/v) to EtOAc only) to afford 30.4 mg (63% yield) of the title compound **101** as colorless solid.

R*f* = 0.23 (hexanes / EtOAc = 2:1 (v/v))

NMR Spectroscopy:

^{1}H NMR (500 MHz, $CDCl_3$, 25 °C, δ): 8.73 (d, *J* = 8.9 Hz, 1H), 8.54 – 8.39 (m, 2H), 8.24 – 8.11 (m, 1H), 8.01 (dd, *J* = 8.8, 2.4 Hz, 1H), 7.98 – 7.90 (m, 2H), 7.84 (d, *J* = 2.3 Hz, 1H), 7.63 – 7.44 (m, 5H), 7.37 (dd, *J* = 7.8, 4.7 Hz, 1H), 7.31 (d, *J* = 8.4 Hz, 2H).

^{13}C NMR (126 MHz, $CDCl_3$, 25 °C, δ): 162.7, 152.0, 146.6, 141.7, 140.8, 139.1, 137.4, 135.7, 134.3, 133.5, 132.2, 130.9, 130.3, 129.9, 129.5, 128.9, 127.8, 123.2, 121.6.

HRMS GC-ESI (m/z) calc'd for $C_{24}H_{16}N_2O_3SCl_2Na$ $[M+Na]^+$, 505.0151; found, 505.0156. deviation: –0.9 ppm.

(±)-Fluribuprofen methyl ester phenyl sulfone derivative 102

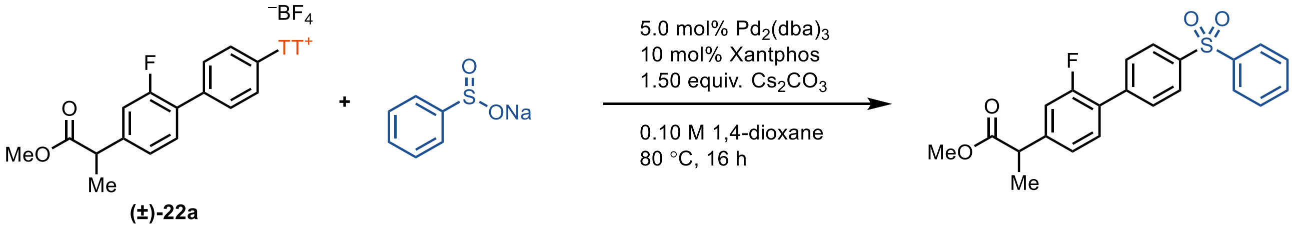

1.00 equiv. 1.20 equiv. **(±)-102,** 69%

Under an ambient atmosphere, (±)-flurbiprofen methyl ester-derived thianthrenium salt **(±)-22a** (56.0

mg, 0.100 mmol, 1.00 equiv.), Cs_2CO_3 (48.9 mg, 0.150 mmol, 1.50 equiv.), $Pd_2(dba)_3$ (4.6 mg, 5.0 µmol, 5.0 mol%), Xantphos (5.8 mg, 10 µmol, 10 mol%) and phenylsulfinate sodium salt (19.7 mg, 0.120 mmol, 1.20 equiv.) were added to a 4-mL vial containing a stirring bar. The vial was transferred to a nitrogen-filled glovebox and 1,4-dioxane (1.0 mL, *c* = 0.10 M) was added. The vial was sealed with a septum cap, and removed from the glovebox. The reaction mixture was stirred (600 rpm) at 80 °C for 16 h on a heating block. After that, the reaction mixture was cooled to room temperature and transferred to a separatory funnel using ethyl acetate (2 mL) and water (2 mL). The organic layer was separated from the aqueous layer, and the aqueous layer was further extracted with ethyl acetate (2 × 2 mL). The combined organic layers were dried over Na_2SO_4, filtered, and concentrated by rotary evaporation. The residue was purified by flash column chromatography on silica gel (hexanes / EtOAc = 20:1 to 1:1) to afford 27.6 mg (69% yield) of the title compound **(±)-102** as a colorless oil.

R_f = 0.64 (hexanes / EtOAc = 2:1 (v/v))

NMR Spectroscopy:

^{1}H NMR (500 MHz, $CDCl_3$, 25 °C, δ): 7.99 (td, *J* = 6.6, 1.8 Hz, 4H), 7.65 (dd, *J* = 8.6, 1.6 Hz, 2H), 7.60 – 7.50 (m, 3H), 7.36 (t, *J* = 8.0 Hz, 1H), 7.20 – 7.10 (m, 2H), 3.76 (q, *J* = 7.0 Hz, 1H), 3.69 (s, 3H), 1.53 (d, *J* = 7.2 Hz, 3H).

^{13}C NMR (126 MHz, $CDCl_3$, 25 °C, δ): 174.3, 159.7 (d, *J* = 249.7 Hz), 143.5 (d, *J* = 7.7 Hz), 141.7, 140.7 (d, *J* = 3.6 Hz), 133.4, 130.8 (d, *J* = 3.6 Hz), 129.8 (d, *J* = 3.0 Hz), 129.4, 127.8, 127.0, 125.9 (d, *J* = 13.1 Hz), 123.9 (d, *J* = 3.6 Hz), 115.6 (d, *J* = 23.2 Hz), 52.4, 45.1, 18.5.

^{19}F NMR (471 MHz, $CDCl_3$, 25 °C, δ): –117.1.

HRMS GC-ESI (m/z) calc'd for $C_{22}H_{19}O_4NaFS$ $[M+Na]^+$, 421.0880; found, 421.0878. deviation: +0.6 ppm.

Tianeptine intermediate sulfone derivative 103

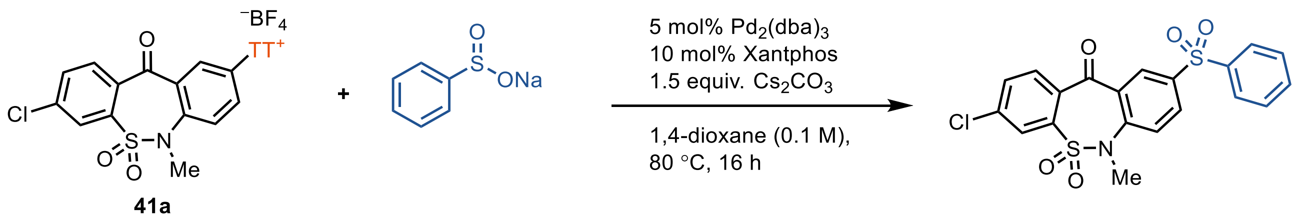

41a 1.00 equiv. 1.20 equiv. **103,** 72%

Under an ambient atmosphere, tianeptine intermediate-derived thianthrenium salt **41a** (61.0 mg, 0.100 mmol, 1.00 equiv.), Cs_2CO_3 (48.9 mg, 0.150 mmol, 1.50 equiv.), $Pd_2(dba)_3$ (4.6 mg, 5.0 µmol, 5.0 mol%), Xantphos (5.8 mg, 10 µmol, 10 mol%) and phenylsulfinate sodium salt (19.7 mg, 0.120 mmol, 1.20 equiv.) were added to a 4-mL vial containing a magnetic stirring bar. The vial was transferred to a nitrogen-filled glovebox and 1,4-dioxane (1.0 mL, *c* = 0.10 M) was added. The vial was sealed with a septum cap, and removed from the glovebox. The reaction mixture was stirred (600 rpm) at 80 °C for 16 h on a heating block. After that, the reaction mixture was cooled to room temperature and transferred to a separatory funnel using ethyl acetate (2 mL) and water (2 mL). The organic layer was

separated from the aqueous layer, and the aqueous layer was further extracted with ethyl acetate (2 × 2 mL). The combined organic layers were dried over Na_2SO_4, filtered, and concentrated by rotary evaporation. The residue was purified by flash column chromatography on silica gel (hexanes / EtOAc = 10:1 (v/v) to EtOAc only) to afford 32.1 mg (72% yield) of the title compound **103** as a colorless oil.

R*f* = 0.40 (hexanes / EtOAc = 2:1 (v/v))

NMR Spectroscopy:

^{1}H NMR (500 MHz, $CDCl_3$, 25 °C, δ): 8.84 (d, *J* = 2.3 Hz, 1H), 8.16 (dd, *J* = 8.6, 2.3 Hz, 1H), 8.04 – 8.00 (m, 2H), 7.91 (d, *J* = 2.1 Hz, 1H), 7.85 (d, *J* = 8.2 Hz, 1H), 7.71 (dd, *J* = 8.3, 2.1 Hz, 1H), 7.61 (t, *J* = 7.4 Hz, 1H), 7.55 (t, *J* = 7.6 Hz, 2H), 7.41 (d, *J* = 8.5 Hz, 1H), 3.44 (s, 3H).

^{13}C NMR (126 MHz, $CDCl_3$, 25 °C, δ): 188.3, 145.0, 140.9, 139.3, 139.1, 138.2, 134.0, 134.0, 133.9, 133.2, 133.2, 132.4, 130.6, 129.7, 128.0, 124.9, 124.1, 38.6.

HRMS GC-ESI (m/z) calc'd for $C_{20}H_{14}NO_5S_2ClNa$ [M+Na]$^+$, 469.9894; found, 469.9895. deviation: –0.1 ppm.

Halogenation and methoxylation of aryl thianthrenium salts

General procedure for halogenation of aryl thianthrenium salts

A 4-mL borosilicate vial containing a Teflon-coated magnetic stirring bar was charged with (±)-flurbiprofen aryl thianthrenium salt (0.100 mmol, 1.00 equiv.) and NaBr (for bromination, 20.6 mg, 0.200 mmol, 2.00 equiv.). The vial was introduced into a nitrogen-filled glovebox, $NiBr_2$·diglyme (3.5 mg, 10 µmol, 10 mol%) and TBACl (for chlorination, 55.6 mg, 0.200 mmol, 2.00 equiv.). were added to the vial. DMA (1.0 mL, *c* = 0.10 M) was added using a syringe. The reaction vial was taken out of the glovebox, and pempidine (54 µL, 47 mg, 0.30 mmol, 3.0 equiv.) was added by using a microsyringe via septum. The mixture was then transferred to a Penn photoreactor and irradiated at 450 nm (100% light intensity, 600 rpm stir rate, max fans (6800 rpm)). After 20 h, the mixture was diluted with EtOAc (2 mL) washed with brine (2 mL), and dried over Na_2SO_4. Upon filtration, the organic layer was concentrated by rotary evaporation and purified by flash column chromatography on silica gel.

General procedure for methoxylation of aryl thianthrenium salts

A 4-mL borosilicate vial containing a Teflon-coated magnetic stirring bar was charged with aryl thianthrenium salt (0.100 mmol, 1.00 equiv.) and quiniclidine (33.4 mg, 0.300 mmol, 3.00 equiv.). The vial was introduced into a nitrogen-filled glovebox and $NiCl_2$·glyme (2.2 mg, 10 µmol, 10 mol%). was added to the vial. DMA (1.0 mL, *c* = 0.10 M) was added using a syringe. The reaction vial was taken out of the glovebox, and MeOH (40 µL, 32 mg, 10 mmol, 10 equiv.) was added by using a microsyringe via septum. The mixture was then transferred to a Penn photoreactor and irradiated at 450 nm (100% light intensity, 600 rpm stir rate, max fans (6800 rpm)). After 16 h, the mixture was diluted with EtOAc (2 mL) washed with water (2 mL), and dried over Na_2SO_4. Upon filtration, the organic layer was concentrated by rotary evaporation and purified by flash column chromatography on silica gel.

Effect of base on product ratio for chlorination of aryl thianthrenium salts

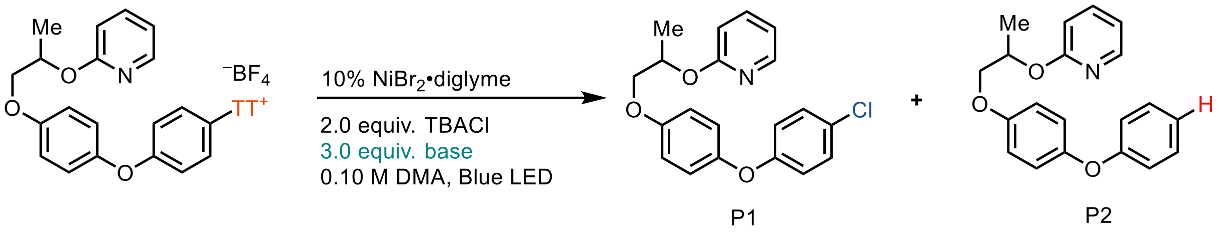

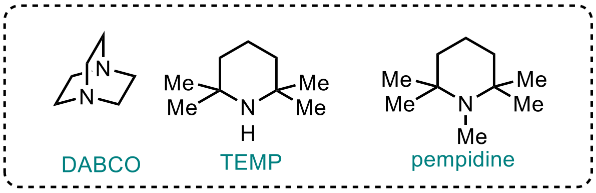

base	yield of P1	yield of P2
DABCO	56%	35%
TEMP	90%	8%
pempidine	99%	n.d.

Fig S13: Reactions were carried out on a 0.10 mmol scale. Yields were determined by ^{1}H NMR using 1,3,5-trimethoxybenzene (1.0 equiv.) as an internal standard.

Effect of water for chlorination of aryl thianthrenium salts

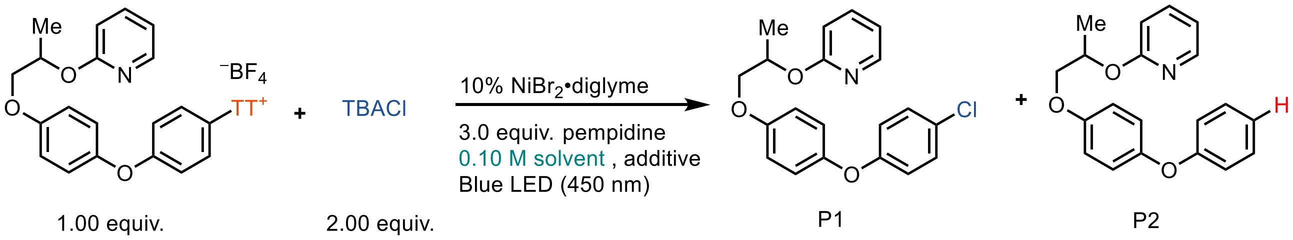

Table S11 Effect of water for chlorination of aryl thianthrenium salts:

Solvent	Additive	Yield of P1	Yield of P2
DMA[a]	None	99%	n.d.
DMA[b]	None	90%	10%
DMA[b,c]	None	n.d	40%
DMA[a]	0.5 equiv. H_2O	99%	n.d.
DMA[a]	1.5 equiv. H_2O	78%	12%
DMA[a]	2.5 equiv. H_2O	<40%	n.d.

Reaction were carried out on a 0.10 mmol scale. Yields were determined by ^{1}H NMR using 1,3,5-trimethoxybenzene (1.0 equiv.) as an internal standard. [a]anhydrous DMA: bottle was always stored in a nitrogen-fillled glovebox. [b]DMA bottle was always stored outside of the glovebox. [c]All the reagents were added under air and no vacuum-argon cycle performed.

Effect of irradiation source

Aryl-TT	*Nucleophile*	*395 nm Penn Photoreactor*	*420 nm Penn Photoreactor*	*450 nm Penn Photoreactor*	*456 nm Kessil Lamps*
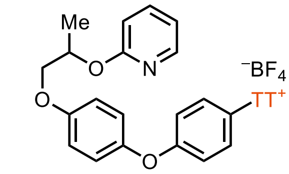	MeOH	**100%**	**100%**	**99%**	**20%**
	TBACl	**100%**	**75%**	**100%**	**100%**
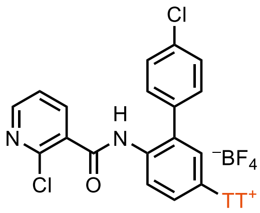	MeOH	**21%**	**n.d.**	**45%**	**65%**
	TBACl	**99%**	**n.d.**	**99%**	**99%**

yield > 70% **yield = 40%–70%** **yield < 40%**

Fig S14: Effect of the irraditation source. Reactions were carried out on a 0.10 mmol scale. Yields were determined by ^{1}H NMR using 1,3,5-trimethoxybenzene (1.0 equiv.) as an internal standard.

(±)-Flurbiprofen methyl ester chloride derivative 104

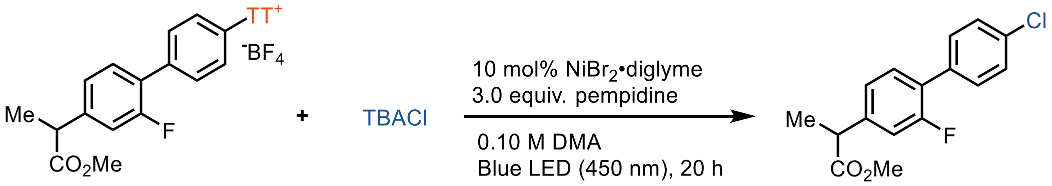

(±)-22a 1.00 equiv 2.00 equiv. **(±)-104**, 92%

A 4-mL borosilicate vial containing a Teflon-coated magnetic stirring bar was charged with (±)-flurbiprofen methyl ester-derived thianthrenium salt **(±)-22a** (56.0 mg, 0.100 mmol, 1.00 equiv.). The vial was introduced into a nitrogen-filled glovebox, $NiBr_2$·diglyme (3.5 mg, 10 μmol, 10 mol%) and TBACl (55.6 mg, 0.200 mmol, 2.00 equiv.). were added to the vial. DMA (1.0 mL, *c* = 0.10 M) was added using a syringe. The reaction vial was taken out of the glovebox, and pempidine (54 μL, 47 mg, 0.30 mmol, 3.0 equiv.) was added by using a microsyringe via septum. The mixture was then transferred to a Penn photoreactor and irradiated at 450 nm (100% light intensity, 600 rpm stir rate, max fans (6800 rpm)). After 20 h, the mixture was diluted with EtOAc (2 mL) washed with brine (2 mL), and dried over Na_2SO_4. Upon filtration, the organic layer was concentrated by rotary evaporation and purified by flash column chromatography on silica gel (hexanes / EtOAc = 10:1 (v/v)) to afford 26.9 mg (92% yield) of the title compound **(±)-104** as a colorless oil.

R*f* = 0.35 (hexanes / EtOAc = 10:1 (v/v))

NMR Spectroscopy:

^{1}H NMR (500 MHz, $CDCl_3$, 25 °C, δ): 7.47 (dd, *J* = 8.4, 1.6 Hz, 2H), 7.41 (d, *J* = 8.5 Hz, 2H), 7.36 (t, *J* = 8.0 Hz, 1H), 7.17 – 7.10 (m, 2H), 3.76 (q, *J* = 7.2 Hz, 1H), 3.70 (s, 3H), 1.54 (d, *J* = 7.2 Hz, 3H).

^{13}C NMR (126 MHz, $CDCl_3$, 25 °C, δ): 174.5, 159.8 (d, *J* = 248.6 Hz), 142.4 (d, *J* = 7.8 Hz), 134.0 (d, *J* = 14.3 Hz), 130.7 (d, *J* = 3.6 Hz), 130.3 (d, *J* = 3.6 Hz), 128.8, 126.8 (d, *J* = 13.1 Hz), 123.8 (d, *J* = 3.6 Hz), 115.5 (d, *J* = 23.2 Hz), 52.4, 45.1, 18.6.

^{19}F NMR (471 MHz, $CDCl_3$, 25 °C, δ): –117.4.

HRMS GC-EI (m/z) calc'd for $C_{16}H_{14}O_2FCl$ $[M]^+$, 292.0661; found, 292.0662. deviation: –0.5 ppm.

(±)-Flurbiprofen methyl ester bromide derivative 105

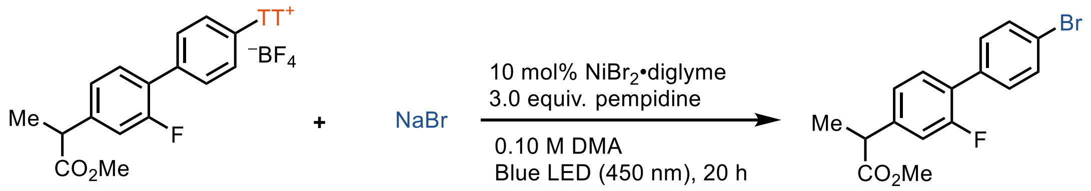

(±)-22a

1.00 equiv 2.00 equiv. **(±)-105**, 84%

A 4-mL borosilicate vial containing a Teflon-coated magnetic stirring bar was charged with (±)-flurbiprofen methyl ester-derived thianthrenium salt **(±)-22a** (56.0 mg, 0.100 mmol, 1.00 equiv.) and NaBr (20.6 mg, 0.200 mmol, 2.00 equiv.). The vial was introduced into a nitrogen-filled glovebox, and $NiBr_2$·diglyme (3.5 mg, 10 μmol, 10 mol%) was added to the vial. DMA (1.0 mL, *c* = 0.10 M) was added using a syringe. The reaction vial was taken out of the glovebox, and pempidine (54 μL, 47 mg, 0.30 mmol, 3.0 equiv.) was added by using a microsyringe via septum. The mixture was then transferred to a Penn photoreactor and irradiated at 450 nm (100% light intensity, 600 rpm stir rate, max fans (6800 rpm)). After 20 h, the mixture was diluted with EtOAc (2 mL) washed with brine (2 mL), and dried over Na_2SO_4. Upon filtration, the organic layer was concentrated by rotary evaporation and purified by flash column chromatography (hexanes / EtOAc = 40:1 to 20:1 (v/v)) on silica gel to afford 28.3 mg (84% yield) of the title compound **(±)-105** as a colorless oil.

R*f* = 0.33 (hexanes / ethyl acetate = 5:1 (v/v))

NMR Spectroscopy:

^{1}H NMR (500 MHz, $CDCl_3$, 25 °C, δ): 7.58 – 7.54 (m, 2H), 7.42 – 7.33 (m, 3H), 7.18 – 7.10 (m, 2H), 3.76 (q, *J* = 7.1 Hz, 1H), 3.70 (s, 3H), 1.54 (d, *J* = 7.2 Hz, 3H).

^{13}C NMR (126 MHz, $CDCl_3$, 25 °C, δ): 174.5, 159.7 (d, *J* = 248.6 Hz), 142.4 (d, *J* = 7.8 Hz), 134.5, 131.8, 130.6 (d, *J* = 3.6 Hz), 126.8 (d, *J* = 13.1 Hz), 123.8 (d, *J* = 3.6 Hz), 122.1, 115.5 (d, *J* = 23.2 Hz), 52.4, 45.1, 18.6.

^{19}F NMR (471 MHz, $CDCl_3$, 25 °C, δ): –117.4.

HRMS GC-EI (m/z) calc'd for $C_{16}H_{14}O_2FBr$ $[M]^+$, 336.0156; found, 336.0161. deviation: –1.5 ppm.

Procedure by using the Schlenk line: A 4-mL borosilicate vial containing a Teflon-coated magnetic stirring bar was used. (±)-flurbiprofen methyl ester-derived thianthrenium salt **(±)-22a** (56.0 mg, 0.100 mmol, 1.00 equiv.), NaBr (20.6 mg, 0.200 mmol, 2.00 equiv.) and $NiBr_2$·diglyme (3.5 mg, 10 µmol, 10 mol%) was added to the vial. After sealing with a Schlenk-line adapter, the vial was evacuated and backfilled with argon three times using a Schlenk-line. DMA (deoxygenated with Ar flow for 1 minutes, 1.0 mL, c = 0.10 M) was added using a syringe under Ar flow. The vial was sealed with a septum cap and pempidine (13 µL, 13 mg, 0.15 mmol, 1.5 equiv.) (54 µL, 47 mg, 0.30 mmol, 3.0 equiv.) was added by using a microsyringe via septum. The mixture was then transferred to a Penn photoreactor and irradiated at 450 nm (100% light intensity, 600 rpm stir rate, max fans (6800 rpm)). After 20 h, the mixture was cooled to 25 °C. 1,3,5-trimethoxybenzene (16.8 mg, 0.100 mmol, 1.00 equiv.) was added into the reaction mixture as an internal standard, followed by EtOAc (2.0 mL) and H_2O (2 mL). Layers were separated and an aliquot (ca. 1 mL) from the EtOAc phase was taken and the concentrated by rotary evaporation. The residue was dissolved in $CDCl_3$ (0.6 mL), and the ^{1}H NMR was recorded. The yield (95%) was determined from the ^{1}H NMR spectrum by integrating the signal of the internal standard at 6.11 ppm (s, 3H) and the product **105** at 7.58 – 7.54 (m, 2H).

Boscalid bromide derivative 106

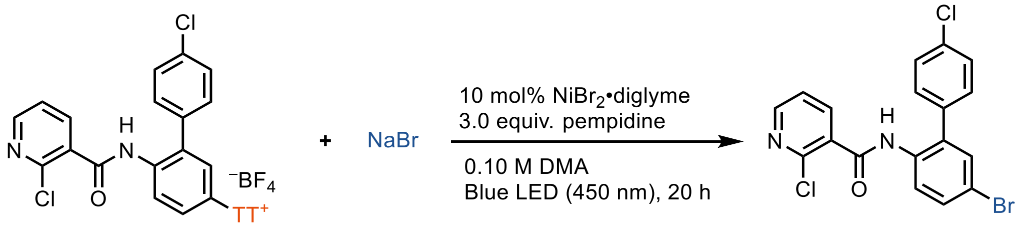

45a
1.00 equiv

2.00 equiv.

106, 87%

A 4-mL borosilicate vial containing a Teflon-coated magnetic stirring bar was charged with boscalid-derived thianthrenium salt **45a** (64.5 mg, 0.100 mmol, 1.00 equiv.) and NaBr (20.6 mg, 0.200 mmol, 2.00 equiv.).The vial was introduced into a nitrogen-filled glovebox and $NiBr_2$·diglyme (3.5 mg, 10 µmol, 10 mol%) was added to the vial. DMA (1.0 mL, c = 0.10 M) was added using a syringe. The reaction vial was taken out of the glovebox, and pempidine (54 µL, 47 mg, 0.30 mmol, 3.0 equiv.) was added by using a microsyringe via septum. The mixture was then transferred to a Penn photoreactor and irradiated at 450 nm (100% light intensity, 600 rpm stir rate, max fans (6800 rpm)). After 20 h, the mixture was diluted with EtOAc (2 mL) washed with brine (2 mL), and dried over Na_2SO_4. Upon filtration, the organic layer was concentrated by rotary evaporation and purified by flash column chromatography on silica gel (hexanes / EtOAc = 3:1 (v/v)) to afford 36.8 mg (87% yield) of the title compound **106** as a colorless oil.

R$_f$ = 0.55 (hexanes / EtOAc = 1:1 (v/v))

NMR Spectroscopy:

^{1}**H NMR** (500 MHz, $CDCl_3$, 25 °C, δ): 8.45 (dd, *J* = 4.8, 2.0 Hz, 1H), 8.36 (d, *J* = 8.8 Hz, 1H), 8.19 (s, 1H), 8.16 (dd, *J* = 7.7, 2.0 Hz, 1H), 7.56 (dd, *J* = 8.8, 2.4 Hz, 1H), 7.48 – 7.43 (m, 2H), 7.41 (d, *J* = 2.4 Hz, 1H), 7.38 – 7.29 (m, 3H).

^{13}**C NMR** (126 MHz, $CDCl_3$, 25 °C, δ): 162.5, 151.7, 146.7, 140.5, 135.2, 134.9, 134.0, 133.8, 133.0, 131.9, 130.8, 130.8, 129.7, 123.5, 123.1, 118.1.

HRMS ESI (m/z) calc'd for $C_{18}H_{11}N_2OBrCl_2Na$ $[M+Na]^+$, 442.9324; found, 442.9326. deviation: –0.4 ppm.

Fenofibrate chloride derivative 107

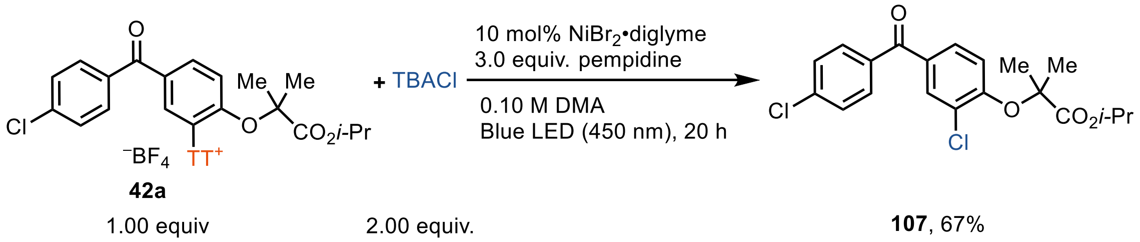

A 4-mL borosilicate vial containing a Teflon-coated magnetic stirring bar was charged with fenofibrate-derived thianthrenium salt **42a** (66.3 mg, 0.100 mmol, 1.00 equiv.). The vial was introduced into a nitrogen-filled glovebox. Then, $NiBr_2$·diglyme (3.5 mg, 10 μmol, 10 mol%) and TBACl (55.6 mg, 0.200 mmol, 1.00 equiv.) were added to the vial. DMA (1.0 mL, *c* = 0.10 M) was added using a syringe. The reaction vial was taken out of the glovebox, and pempidine (54 μL, 47 mg, 0.30 mmol, 3.0 equiv.) was added by using a microsyringe via septum. The mixture was then transferred to a Penn photoreactor and irradiated at 450 nm (100% light intensity, 600 rpm stir rate, max fans (6800 rpm)). After 16 h, the mixture was diluted with EtOAc (2 mL) washed with brine (2 mL), and dried over Na_2SO_4. Upon filtration, the organic layer was concentrated by rotary evaporation and purified by flash column chromatography on silica gel (hexanes / EtOAc = 10:1 to 1:1 (v/v)) to afford 26.3 mg (67% yield) of the title compound **107** as a colorless oil.

R$_f$ = 0.82 (hexanes / EtOAc = 1:1 (v/v))

NMR Spectroscopy:

^{1}**H NMR** (500 MHz, $CDCl_3$, 25 °C, δ): 7.86 (d, *J* = 2.2 Hz, 1H), 7.71 – 7.68 (m, 2H), 7.59 (dd, *J* = 8.6, 2.2 Hz, 1H), 7.48 – 7.45 (m, 2H), 6.86 (d, *J* = 8.6 Hz, 1H), 5.09 (hept, *J* = 6.3 Hz, 1H), 1.69 (s, 6H), 1.23 (d, *J* = 6.3 Hz, 6H).

^{13}**C NMR** (126 MHz, $CDCl_3$, 25 °C, δ): 193.3, 172.9, 155.6, 139.0, 135.9, 132.6, 131.3, 129.6, 128.9, 125.7, 117.0, 81.2, 69.7, 25.4, 21.7.

HRMS GC-EI (m/z) calc'd for $C_{20}H_{20}O_4Cl_2$ $[M]^+$, 394.0733; found, 394.0736. deviation: –0.6 ppm.

(±)-Pyriproxyphen methoxide derivative 108

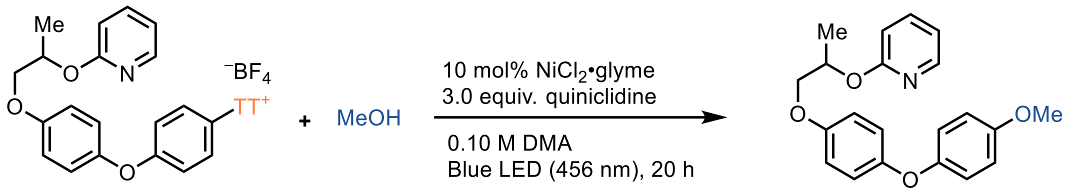

(±)-19a 1.00 equiv. 10 equiv. **(±)-108**, 75%

A 4-mL borosilicate vial containing a Teflon-coated magnetic stirring bar was charged with (±)-pyriproxyphen-derived thianthrenium salt **(±)-19a** (62.4 mg, 0.100 mmol, 1.00 equiv.) and quiniclidine (33.4 mg, 0.300 mmol, 3.00 equiv.). The vial was introduced into a nitrogen-filled glovebox and $NiCl_2$·glyme (2.2 mg, 10 µmol, 10 mol%). was added to the vial. DMA (1.0 mL, *c* = 0.10 M) was added using a syringe. The reaction vial was taken out of the glovebox, and MeOH (40 µL, 32 mg, 10 mmol, 10 equiv.) was added by using a microsyringe via septum. The mixture was then transferred to a Penn photoreactor and irradiated at 450 nm (100% light intensity, 600 rpm stir rate, max fans (6800 rpm)). After 16 h, the mixture was diluted with EtOAc washed with water, and dried over Na_2SO_4. Upon filtration, the organic layer was concentrated by rotary evaporation and purified by flash column chromatography on silica gel (hexanes / EtOAc = 10:1 (v/v)) to afford 21.3 mg (75% yield) of the title compound **(±)-108** as a colorless oil.

R*f* = 0.36 (hexanes / EtOAc = 10:1 (v/v)).

NMR Spectroscopy:

^{1}H NMR (500 MHz, $CDCl_3$, 25 °C, δ): 8.15 (ddd, *J* = 5.0, 2.0, 0.9 Hz, 1H), 7.56 (ddd, *J* = 8.4, 7.1, 2.0 Hz, 1H), 6.94 – 6.82 (m, 9H), 6.74 (dt, *J* = 8.4, 0.9 Hz, 1H), 5.58 (d, *J* = 6.5 Hz, 1H), 4.17 (dd, *J* = 9.8, 5.3 Hz, 1H), 4.06 (dd, *J* = 9.9, 4.8 Hz, 1H), 3.79 (s, 3H), 1.47 (d, *J* = 6.4 Hz, 3H).

^{13}C NMR (126 MHz, $CDCl_3$, 25 °C, δ): 163.3, 155.5, 154.8, 151.9, 151.7, 146.9, 138.8, 119.7, 119.6, 116.9, 115.9, 114.9, 111.8, 71.3, 69.4, 55.8, 17.1.

HRMS GC-EI (m/z) calc'd for $C_{21}H_{21}NO_4$ $[M]^+$, 351.1465; found, 351.1468. deviation: –0.8 ppm.

3,3-Diphenyl propyl acetate 109-S-I

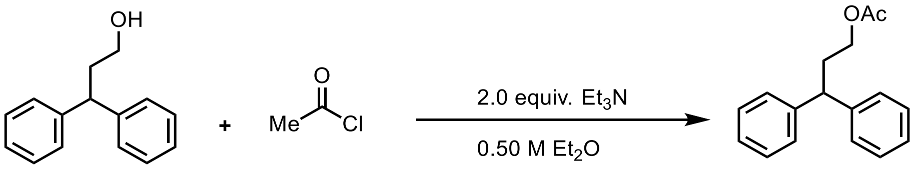

109-S-II 1.00 equiv. 2.0 equiv. **109-S-I**, 95%

To a solution of 3,3-diphenyl propanol **109-S-II** (1.00 g, 4.71 mmol, 1.00 equiv.) in Et_2O (*c* = 0.50 M, 20 mL), Et_3N (1.3 ml, 0.95 g, 9.4 mmol, 2.0 equiv.) was added dropwise at 25 °C. The reaction mixture was stirred for 2 h at 25 °C The resulting precipitate was filtered and washed with H_2O. The solid was

further purified by flash column chromatography on silica gel (hexanes / EtOAc = 4:1 (v/v)) to afford 1142 mg (95% yield) of the title compound **109-S-I** as a colorless oil.

NMR Spectroscopy:

^{1}H NMR (500 MHz, $CDCl_3$, 25 °C, δ): 7.36 – 7.16 (m, 10H), 4.17 – 3.95 (m, 3H), 2.49 – 2.36 (m, 2H), 2.05 (s, 3H).

^{13}C NMR (126 MHz, $CDCl_3$, 25 °C, δ): 171.1, 144.0, 128.6, 127.8, 126.4, 63.0, 47.7, 34.3, 21.0.

HRMS ESI (m/z) calc'd for $C_{17}H_{18}O_2Na$ [M+Na]$^+$, 277.1199; found, 277.1200. deviation: –0.3 ppm.

3,3-Diphenyl propyl acetate-derived thianthrenium salt 109-S

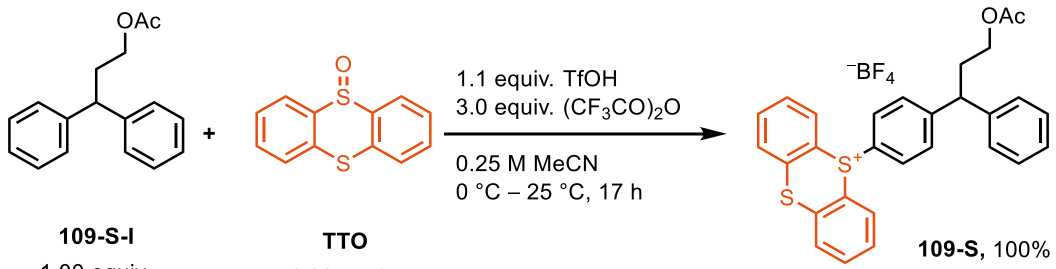

109-S-I 1.00 equiv. **TTO** 1.00 equiv. **109-S,** 100%

To a solution of 3,3-diphenyl propyl acetate (657 mg, 2.58 mmol, 1.00 equiv.) and TTO (thianthrene-*S*-oxide, 600 mg, 2.58 mmol, 1.00 equiv.) in MeCN (6.0 mL, *c* = 0.25 M), trifluoroacetic anhydride (1.1 mL, 1.6 g, 7.8 mmol, 3.0 equiv.) was added at 0 °C, under an ambient atmosphere followed by addition of TfOH (0.27 mL, 0.46 g, 3.1 mmol, 1.1 equiv.). The reaction mixture was stirred at 0 °C for 1 h, and then stirred at 25 °C for 16 h. The solvent was removed under reduced pressure by rotary evaporation and the residue was dissolved in DCM (10 mL). The resulting solution was washed with saturated aqueous $NaHCO_3$ solution (10 mL) and aqueous $NaBF_4$ solution (2 × 10 mL, 10% w/w). The residue was dissolved in DCM (5 mL) and Et_2O (50 mL) was added to obtain the product as white precipitate. The precipitate was filtered to afford 1.44 g of the title compound **109-S** as a colorless solid (100% yield).

NMR Spectroscopy:

^{1}H NMR (500 MHz, $(CD_3)_2SO$, 25 °C, δ): 8.54 (d, *J* = 7.9 Hz, 2H), 8.04 (d, *J* = 7.9 Hz, 2H), 7.91 (t, *J* = 7.7 Hz, 2H), 7.84 (t, *J* = 7.7 Hz, 2H), 7.53 (d, *J* = 8.3 Hz, 2H), 7.26 (d, *J* = 4.4 Hz, 4H), 7.16 (d, *J* = 8.4 Hz, 3H), 4.12 (t, *J* = 7.9 Hz, 1H), 3.83 (t, *J* = 6.6 Hz, 2H), 2.31 (q, *J* = 7.5 Hz, 2H), 1.87 (s, 3H).

^{13}C NMR (151 MHz, $(CD_3)_2SO$, 25 °C, δ): 170.7, 150.2, 143.5, 136.0, 135.8, 135.2, 130.7, 130.2, 130.1, 129.2, 128.8, 128.0, 127.1, 122.9, 119.6, 62.5, 47.1, 33.3, 21.0

^{19}F NMR (471 MHz, $CDCl_3$, 25 °C, δ): –148.2 (bs), –148.3 (bs).

HRMS ESI (m/z) calc'd for $C_{29}H_{25}O_2S_2$ [M]$^+$, 469.1291; found, 469.1295. deviation: –1.0 ppm.

3,3-Diphenyl propyl acetate methoxide derivative 109

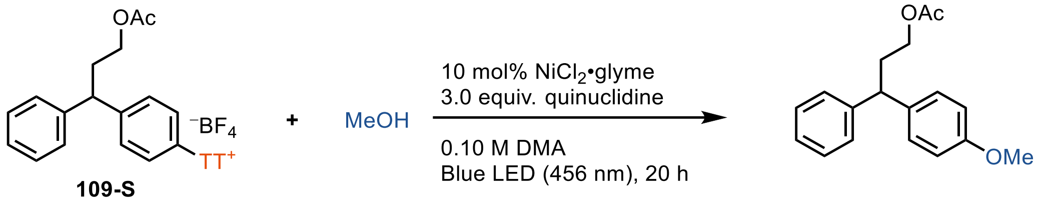

1.00 equiv 10 equiv. **109,** 61%

A 4-mL borosilicate vial containing a Teflon-coated magnetic stirring bar was charged with 3,3-diphenyl propyl acetate-derived thianthrenium salt **109-S** (55.6 mg, 0.100 mmol, 1.00 equiv.) and quiniclidine (33.4 mg, 0.300 mmol, 3.00 equiv.). The vial was introduced into a nitrogen-filled glovebox and $NiCl_2$·glyme (2.2 mg, 10 μmol, 10 mol%). was added to the vial. DMA (1.0 mL, *c* = 0.10 M) was added using a syringe. The reaction vial was taken out of the glovebox, and MeOH (40 μL, 32 mg, 10 mmol, 10 equiv.) was added by using a microsyringe via septum. The mixture was then transferred to a Penn photoreactor and irradiated at 450 nm (100% light intensity, 600 rpm stir rate, max fans (6800 rpm)). After 16 h, the mixture was diluted with EtOAc (2 mL) washed with water (2 mL), and dried over Na_2SO_4. Upon filtration, the organic layer was concentrated by rotary evaporation and purified by flash column chromatography on silica gel (hexanes / EtOAc = 10:1 (v/v)) to afford 17.3 mg (61% yield) of the title compound **109** as a colorless oil.

R*f* = 0.41 (hexanes / EtOAc = 10:1 (v/v)).

NMR Spectroscopy:

^{1}H NMR (600 MHz, $CDCl_3$, 25 °C, δ): 7.30 – 7.26 (m, 2H), 7.24 – 7.20 (m, 2H), 7.19 – 7.12 (m, 3H), 6.86 – 6.81 (m, 2H), 4.03 – 3.99 (m, 3H), 3.77 (s, 3H), 2.39 – 2.30 (m, 2H), 2.02 (d, *J* = 1.6 Hz, 3H).

^{13}C NMR (151 MHz, $CDCl_3$, 25 °C, δ): 171.2, 158.2, 144.5, 136.3, 128.8, 128.7, 127.8, 126.5, 114.1, 63.1, 55.4, 47.1, 34.5, 21.0.

HRMS GC-EI (m/z) calc'd for $C_{18}H_{20}O_3N$ $[M]^+$, 284.1407; found, 284.1407. deviation: 0.0 ppm.

(±)-Fenoprofen methyl ester methoxide derivative 110

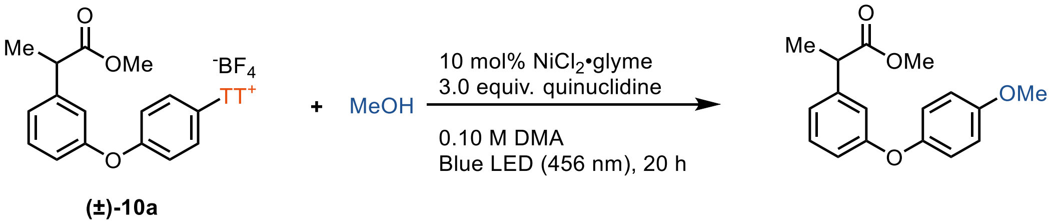

1.00 equiv 10 equiv. **110,** 65%

A 4-mL borosilicate vial containing a Teflon-coated magnetic stirring bar was charged with (±)-fenoprofen methyl ester-derived thianthrenium salt **(±)-10a** (55.8 mg, 0.100 mmol, 1.00 equiv.) and quiniclidine (33.4 mg, 0.300 mmol, 3.00 equiv.). The vial was introduced into a nitrogen-filled glovebox and $NiCl_2$·glyme (2.2 mg, 10 μmol, 10 mol%). was added to the vial. DMA (1.0 mL, *c* = 0.10 M) was

added using a syringe. The reaction vial was taken out of the glovebox, and MeOH (40 µL, 32 mg, 10 mmol, 10 equiv.) was added by using a microsyringe via septum. The mixture was then transferred to a Penn photoreactor and irradiated at 450 nm (100% light intensity, 600 rpm stir rate, max fans (6800 rpm)). After 16 h, the mixture was diluted with EtOAc (2 mL) washed with water (2 mL), and dried over Na_2SO_4. Upon filtration, the organic layer was concentrated by rotary evaporation and purified by flash column chromatography on silica gel (hexanes / EtOAc = 20:1 (v/v)) to afford 18.6 mg (65% yield) of the title compound **(±)-110** as a colorless oil.

R*f* = 0.43 (hexanes / EtOAc = 10:1 (v/v)).

NMR Spectroscopy:

^{1}H NMR (500 MHz, $CDCl_3$, 25 °C, δ): 7.23 (t, *J* = 7.9 Hz, 1H), 7.00 – 6.96 (m, 3H), 6.93 – 6.87 (m, 3H), 6.79 (ddd, *J* = 8.2, 2.5, 1.0 Hz, 1H), 3.81 (s, 3H), 3.66 (m, 4H), 1.47 (d, *J* = 7.2 Hz, 3H).

^{13}C NMR (151 MHz, $CDCl_3$, 25 °C, δ): 174.8, 158.8, 156.1, 150.0, 142.5, 129.8, 121.6, 121.0, 117.0, 116.1, 115.0, 55.8, 52.2, 45.4, 18.6.

HRMS GC-EI (m/z) calc'd for $C_{17}H_{18}O_4$ $[M]^+$, 286.1200; found, 286.1203. deviation: –1.2 ppm.

Phenoxybenzonitrile methoxide derivative 111

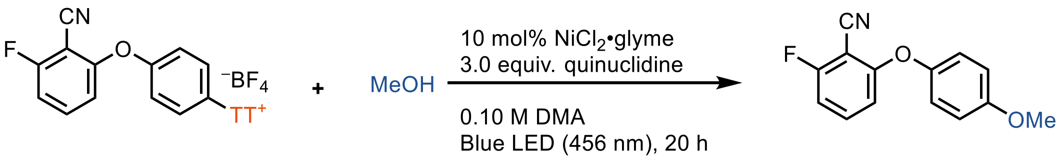

24a
1.00 equiv

10 equiv.

111, 76%

A 4-mL borosilicate vial containing a Teflon-coated magnetic stirring bar was charged with 2-fluoro-6-phenoxybenzonitrile-derived thianthrenium salt **24a** (51.5 mg, 0.100 mmol, 1.00 equiv.) and quinuclidine (33.4 mg, 0.300 mmol, 3.00 equiv.). The vial was introduced into a nitrogen-filled glovebox and $NiCl_2$·glyme (2.2 mg, 10 µmol, 10 mol%). was added to the vial. DMA (1.0 mL, *c* = 0.10 M) was added using a syringe. The reaction vial was taken out of the glovebox, and MeOH (40 µL, 32 mg, 10 mmol, 10 equiv.) was added by using a microsyringe via septum. The mixture was then transferred to a Penn photoreactor and irradiated at 450 nm (100% light intensity, 600 rpm stir rate, max fans (6800 rpm)). After 16 h, the mixture was diluted with EtOAc washed with water, and dried over Na_2SO_4. Upon filtration, the organic layer was concentrated by rotary evaporation and purified by flash column chromatography on silica gel (hexanes / EtOAc = 10:1 (v/v)) to afford 18.4 mg (76% yield) of the title compound **111** as a colorless oil.

R*f* = 0.21 (hexanes / EtOAc = 10:1 (v/v)).

NMR Spectroscopy:

^{1}H NMR (500 MHz, $CDCl_3$, 25 °C, δ): 7.38 (td, *J* = 8.5, 6.4 Hz, 1H), 7.08 – 7.00 (m, 2H), 6.97 – 6.90 (m, 2H), 6.83 (td, *J* = 8.4, 0.9 Hz, 1H), 6.53 (dt, *J* = 8.6, 0.9 Hz, 1H), 3.83 (s, 3H).

^{13}C NMR (126 MHz, $CDCl_3$, 25 °C, δ): 164.2 (d, *J* = 259.3 Hz), 162.2 (d, *J* = 4.2 Hz), 157.5, 147.7,

134.9 (d, J = 10.7 Hz), 122.0, 115.3, 111.4, 111.0 (d, J = 3.6 Hz), 109.2 (d, J = 19.3 Hz), 93.1 (d, J = 17.9 Hz), 55.8.

^{19}F NMR (471 MHz, $CDCl_3$, 25 °C, δ): –105.0.

HRMS GC-EI (m/z) calc'd for $C_{14}H_{10}NO_2FNa$ [M+Na]$^+$, 266.0588; found, 266.0590. deviation: –0.8 ppm.

Effect of counterianion on physical properties and reactivity of aryl thianthrenium salts

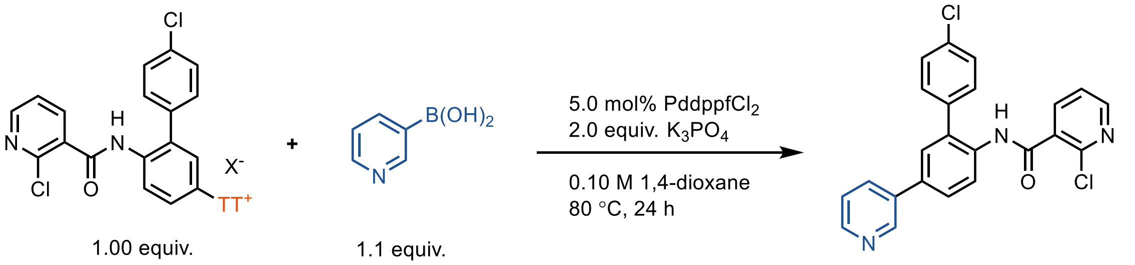

X^- (counterion)	physical state	Yield (%)
$^-$OTf	colorless solid	99
$^-BF_4$	colorless solid	99
$^-PF_6$	colorless solid	99

Fig S15: Reactions were carried out on a 0.10 mmol scale. Yields were determined by ^{1}H NMR using 1,3,5-trimethoxybenzene (1.0 equiv.) as an internal standard.

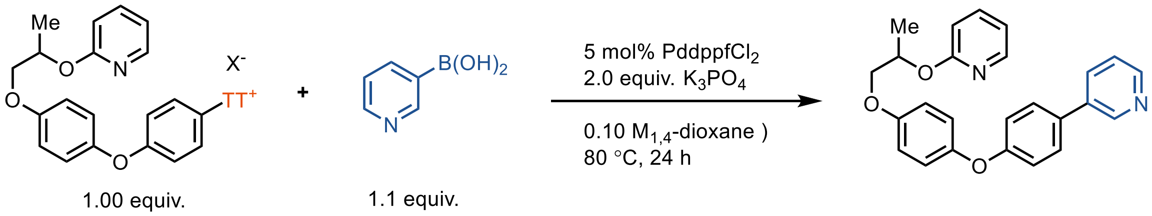

X^- (counterion)	physical state	Yield (%)
$^-$OTf	colorless solid	99
$^-BF_4$	colorless solid	99
$^-PF_6$	colorless solid	99

Fig S16: Reactions were carried out on a 0.10 mmol scale. Yields were determined by ^{1}H NMR using 1,3,5-trimethoxybenzene (1.0 equiv.) as an internal standard.

SPECTROSCOPIC DATA

^{1}H NMR of 2

$CDCl_3$, 500 MHz, 23 ºC.

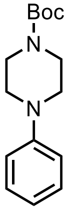

2

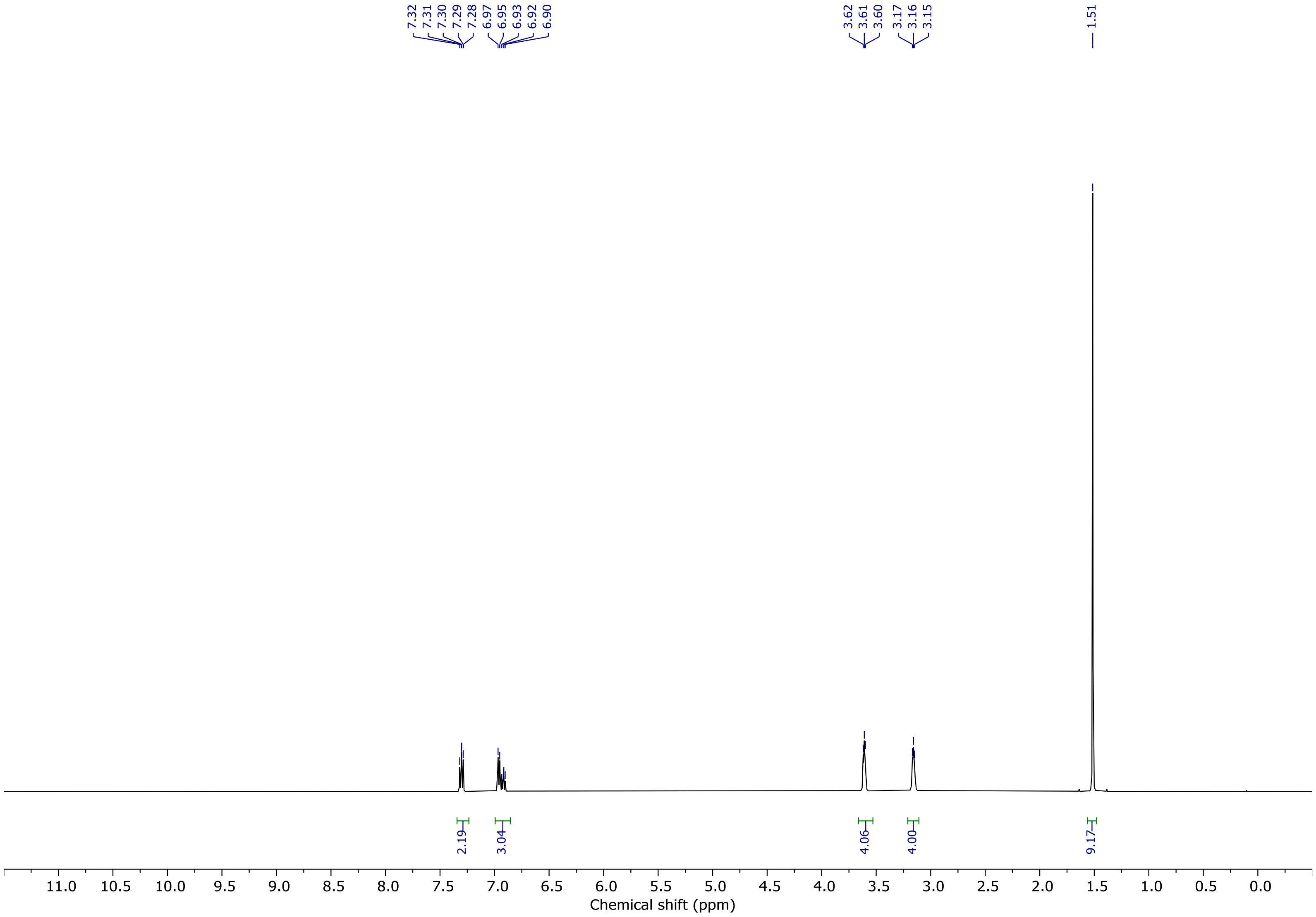

^{13}C NMR of 2

$CDCl_3$, 126 MHz, 23 ºC.

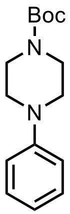

2

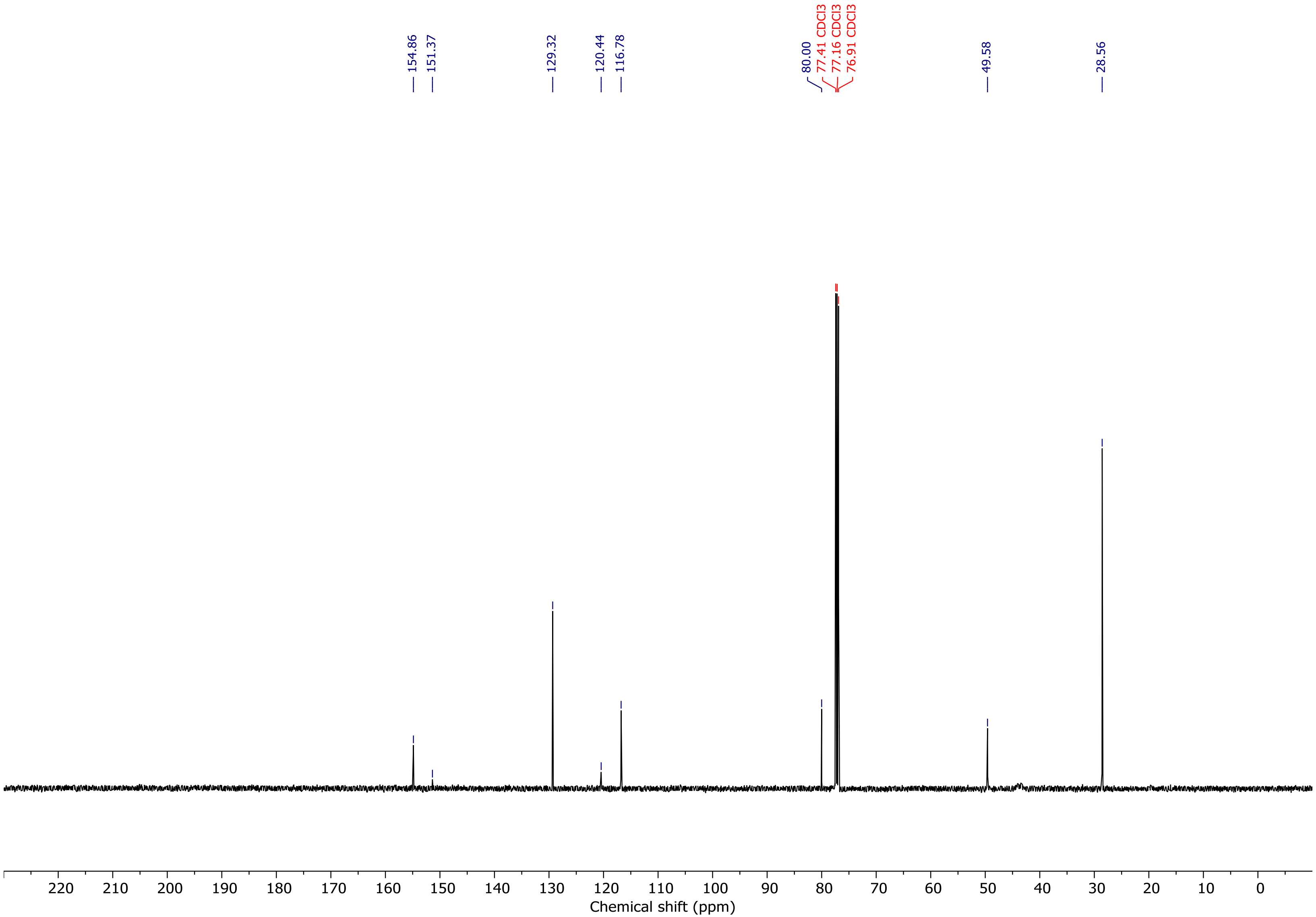

^{1}H NMR of 2a

$CDCl_3$, 500 MHz, 23 ºC.

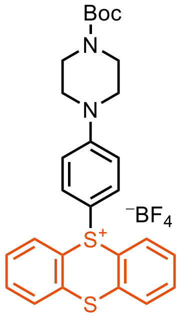

2a

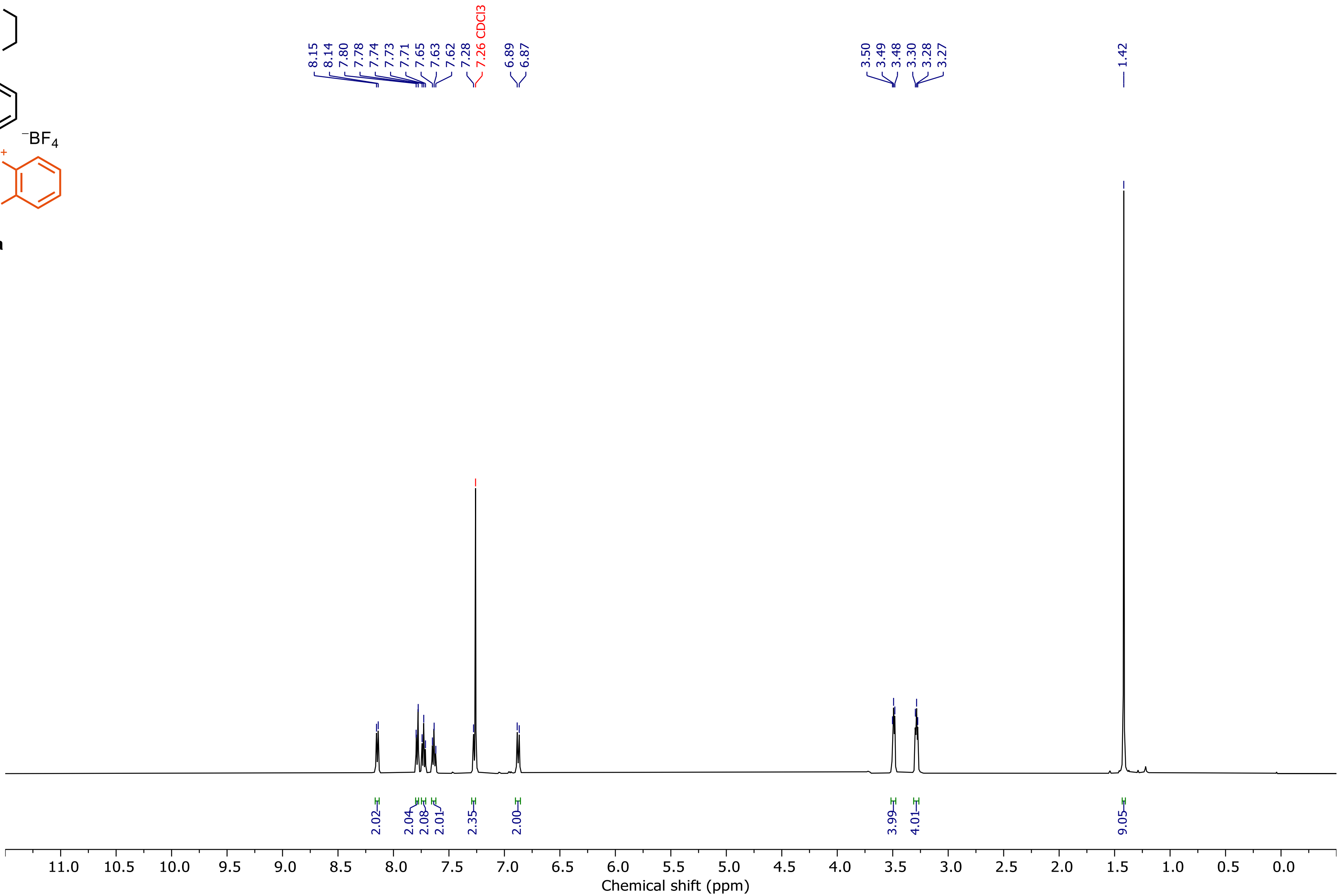

^{13}C NMR of 2a

$CDCl_3$, 126 MHz, 23 ºC.

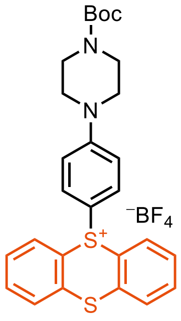

2a

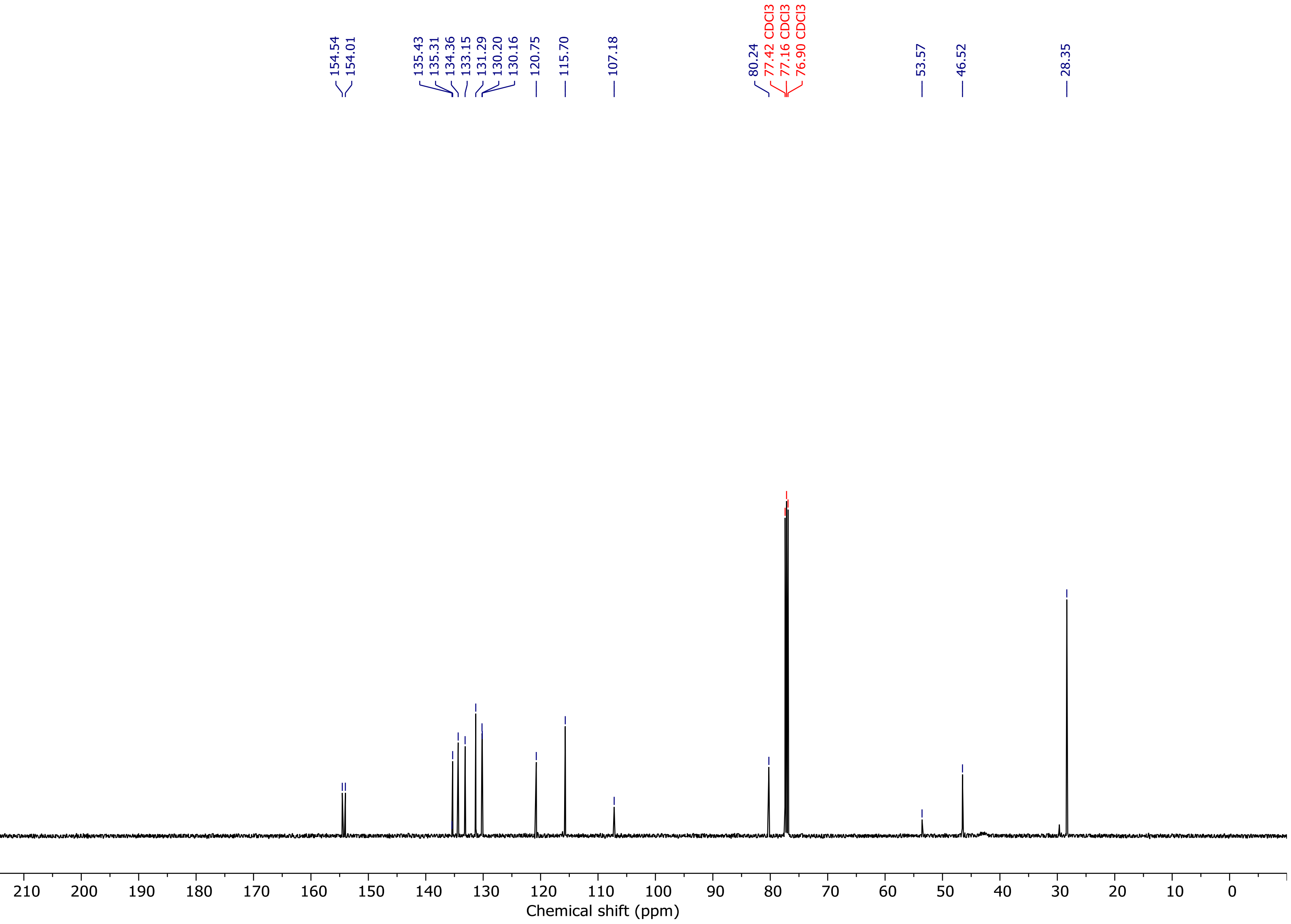

^{19}F NMR of 2a

$CDCl_3$, 471 MHz, 23 ºC.

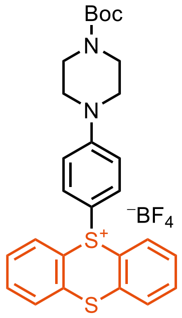

2a

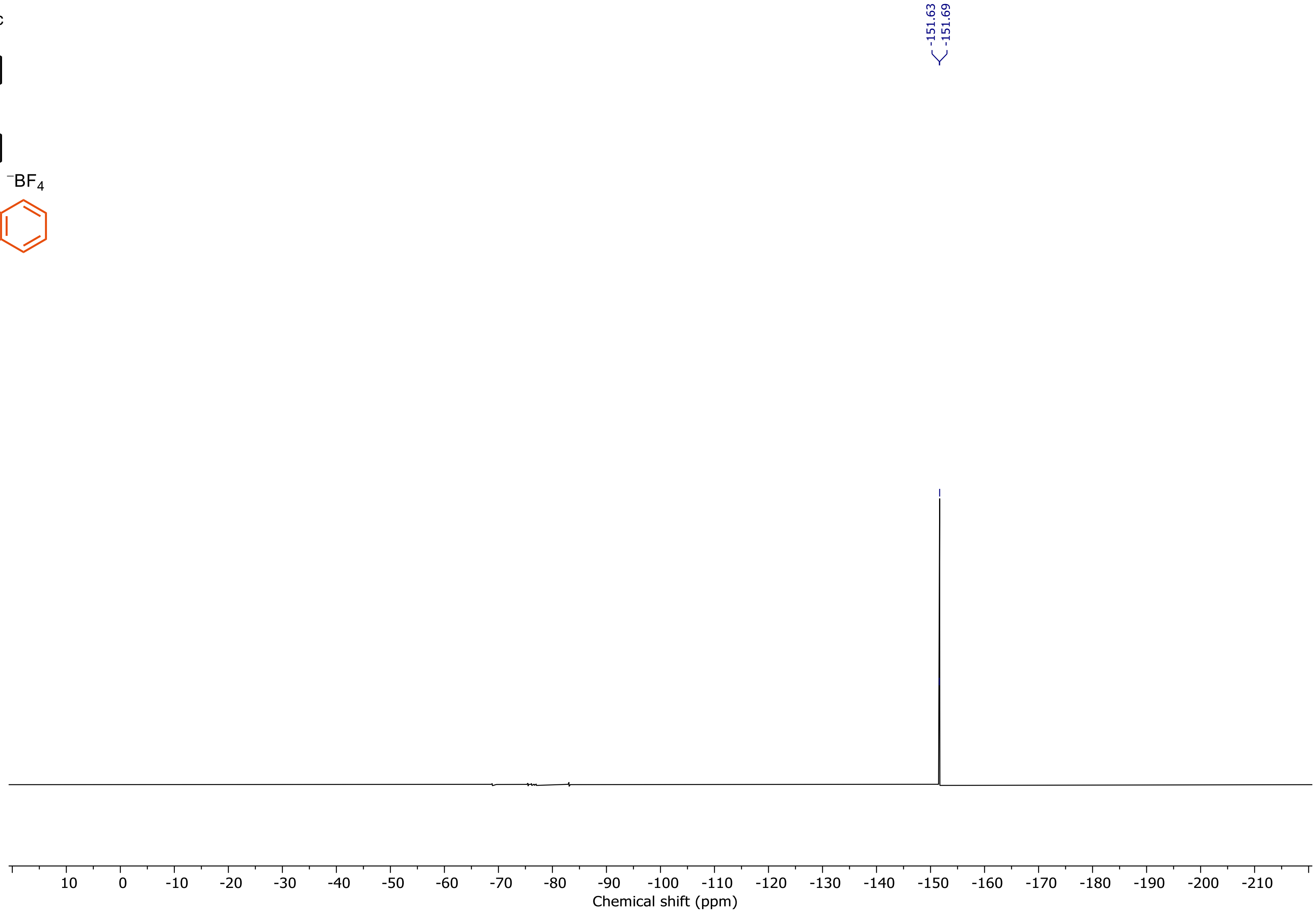

^{1}H NMR of 3

CDCl3, 500 MHz, 23 ºC.

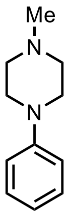

3

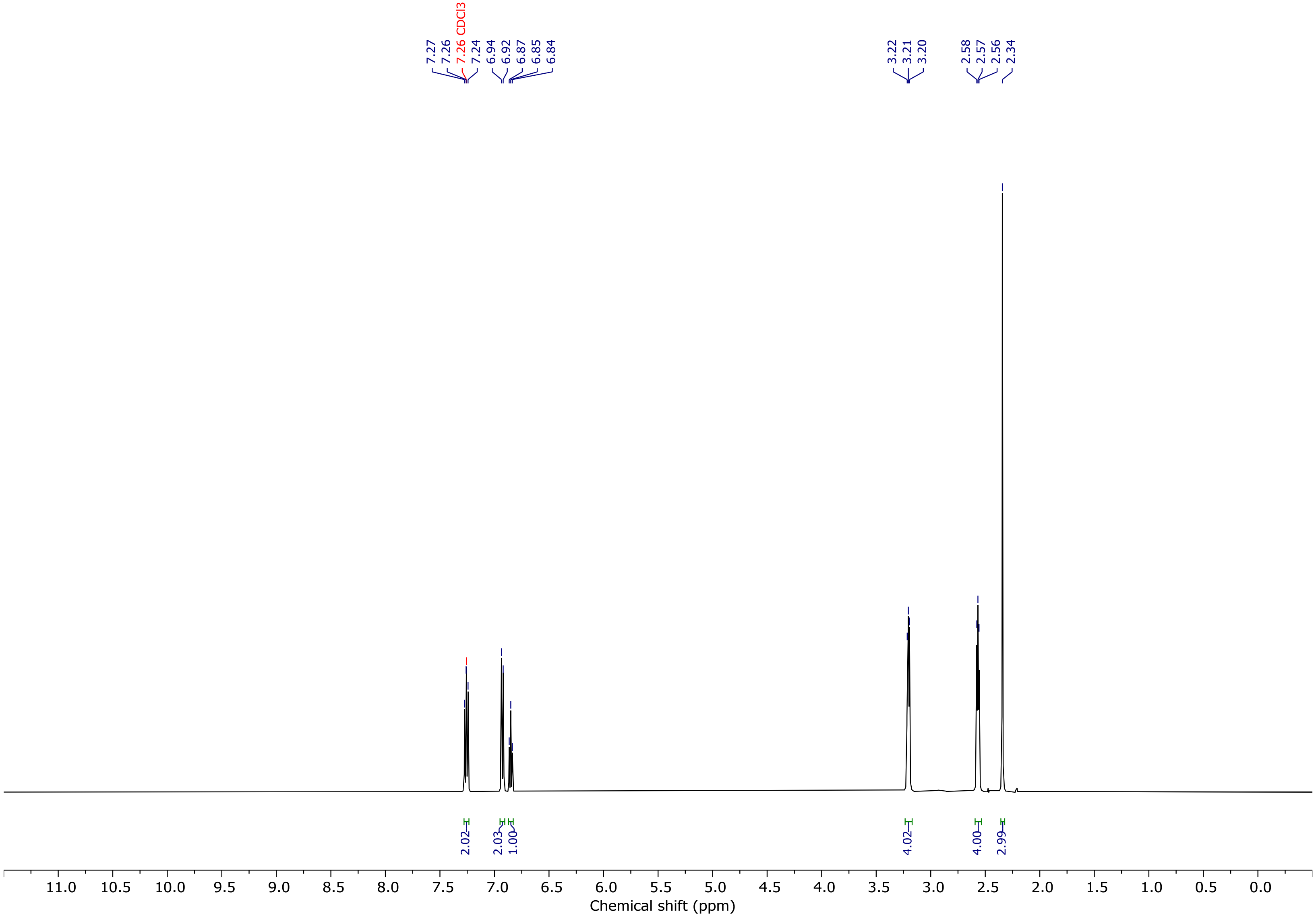

^{13}C NMR of 3

CDCl3, 126 MHz, 23 ºC.

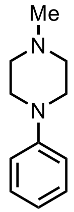

3

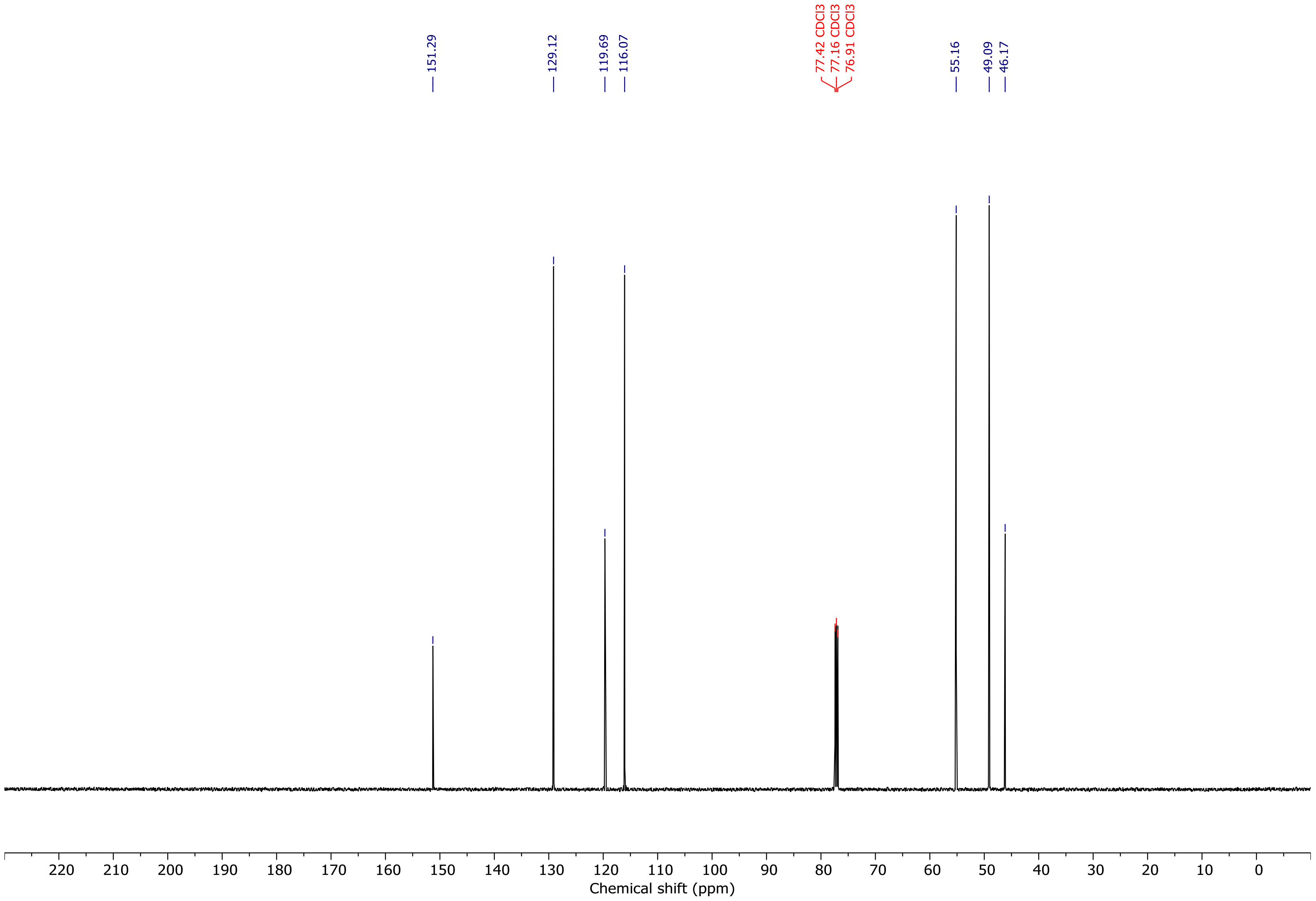

^{1}H NMR of 3a

CD_3CN, 500 MHz, 23 ºC.

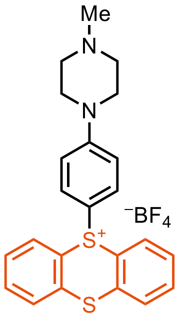

3a

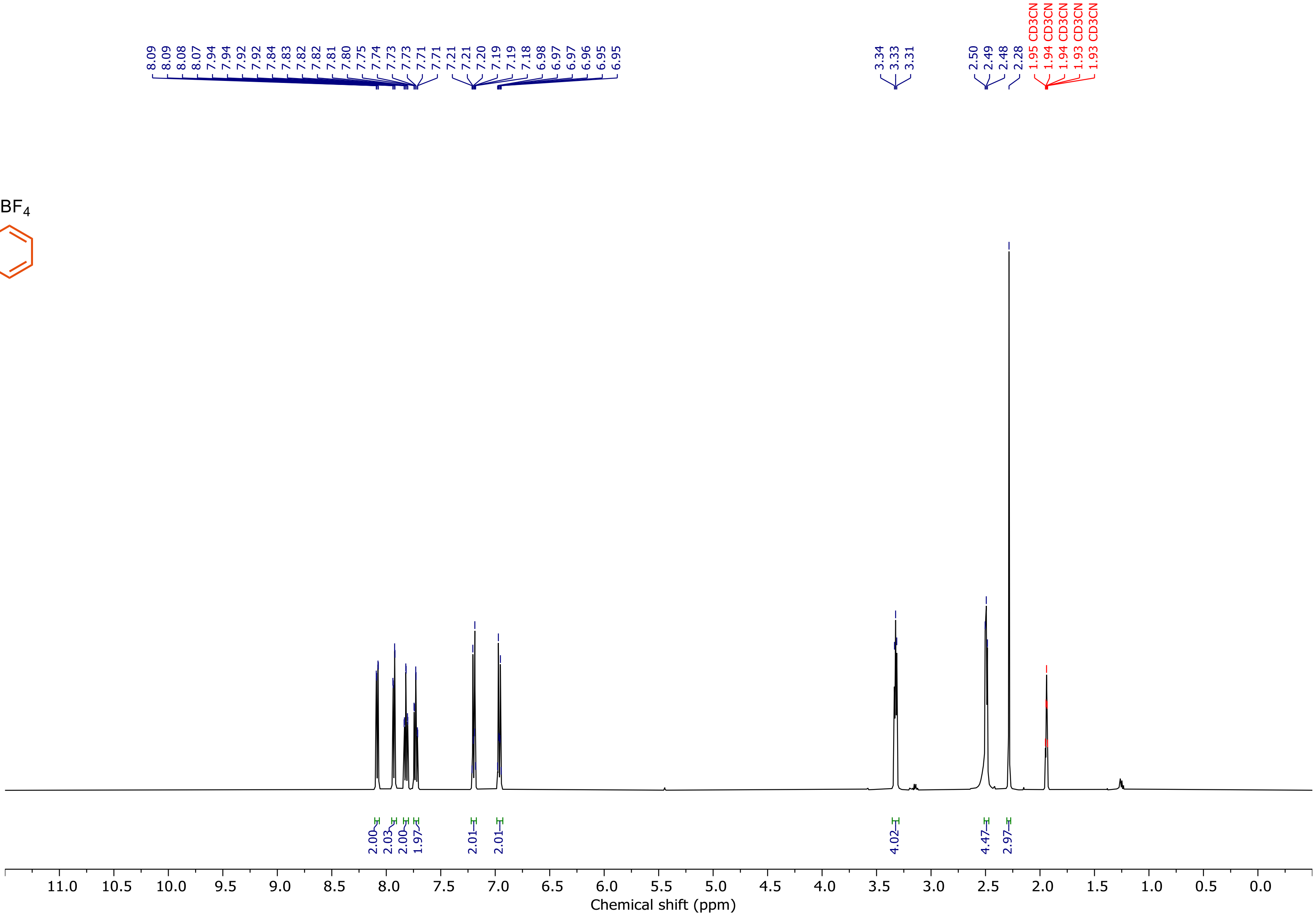

^{13}C NMR of 3a

CD_3CN, 126 MHz, 23 ºC.

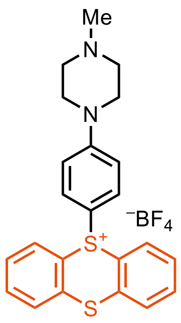

3a

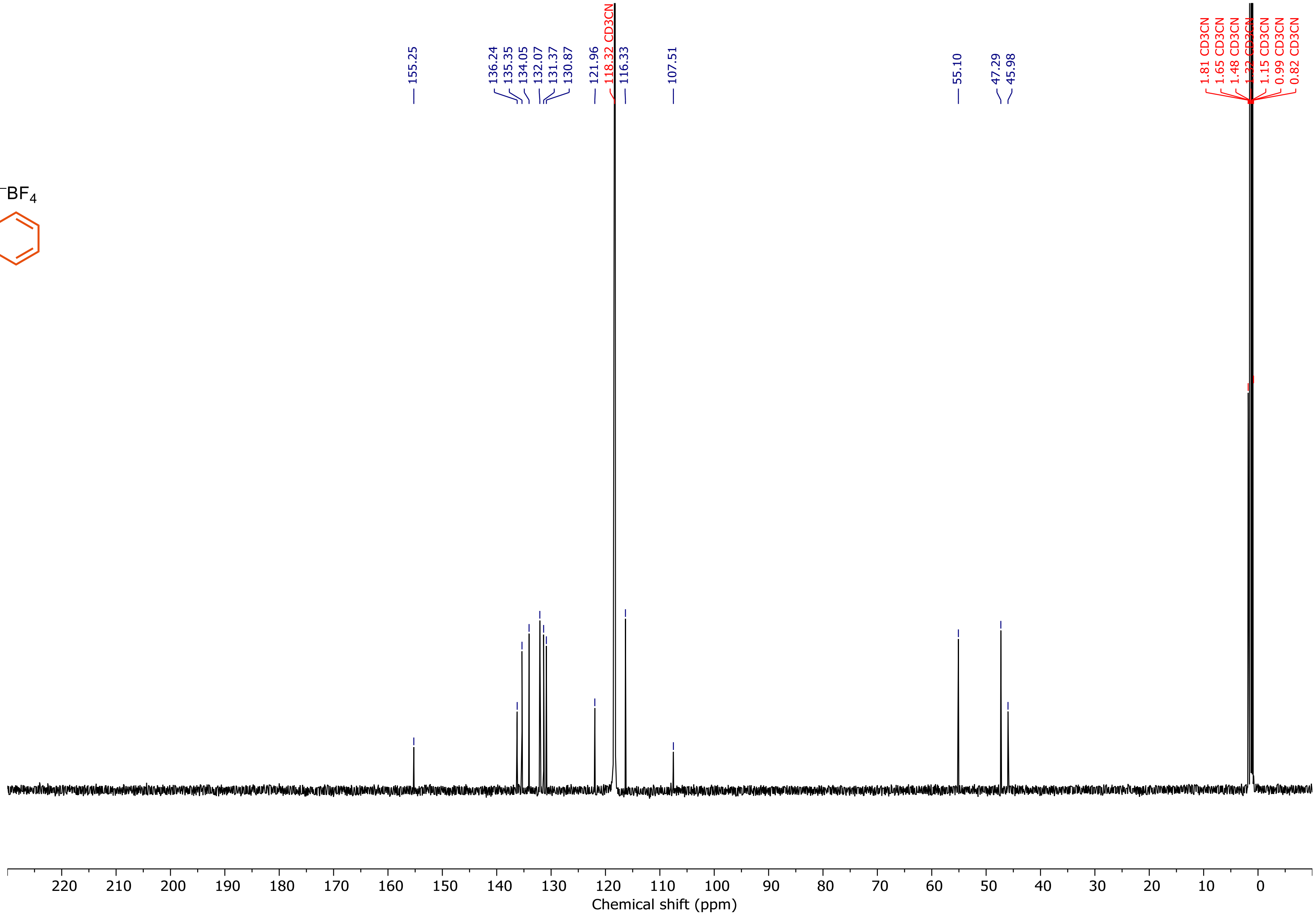

^{19}F NMR of 3a

CD_3CN, 471 MHz, 23 ºC.

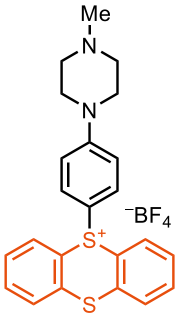

3a

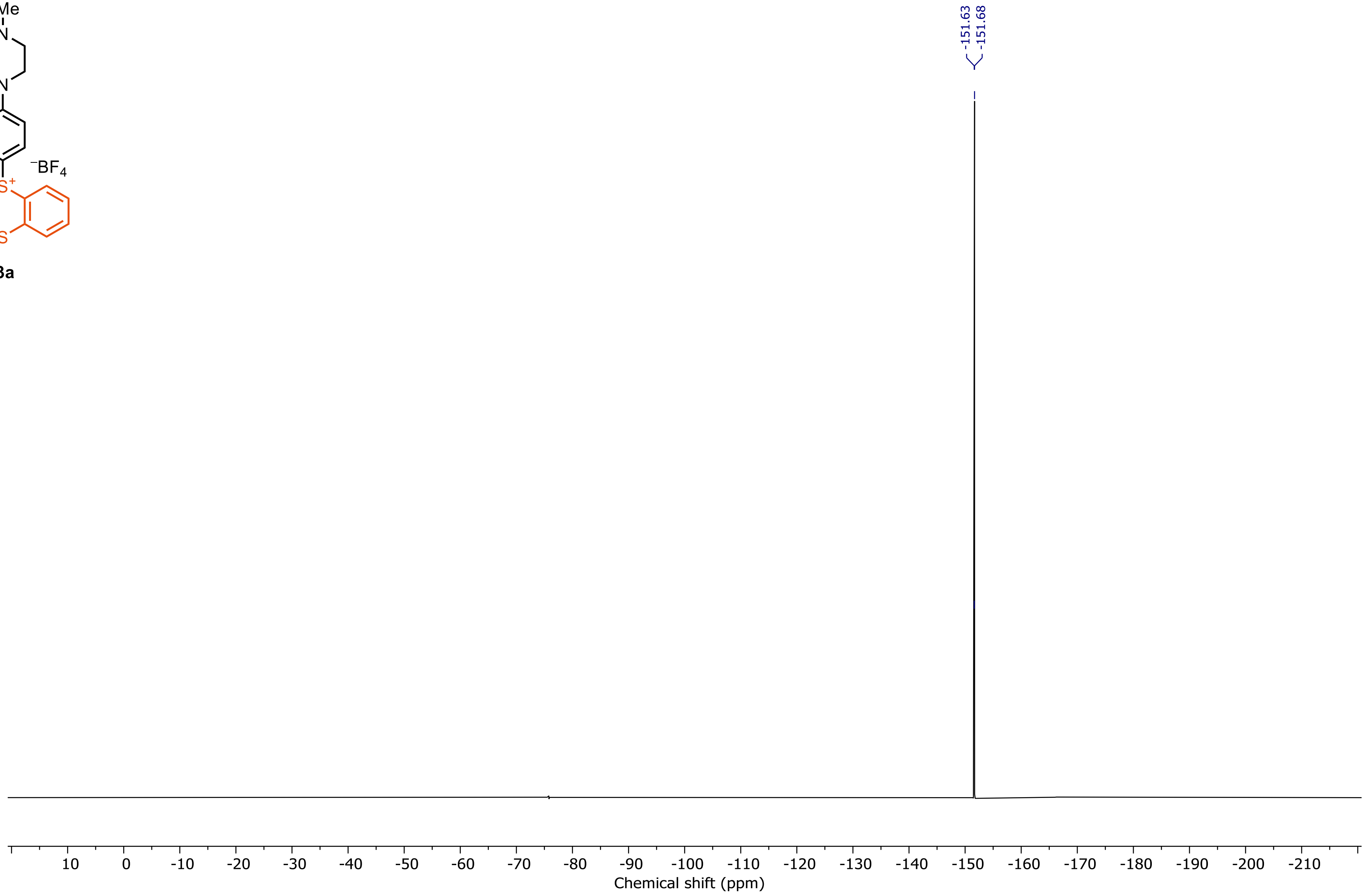

^{1}H NMR of 4a

CD_3CN, 600 MHz, 23 ºC.

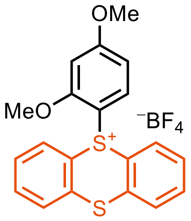

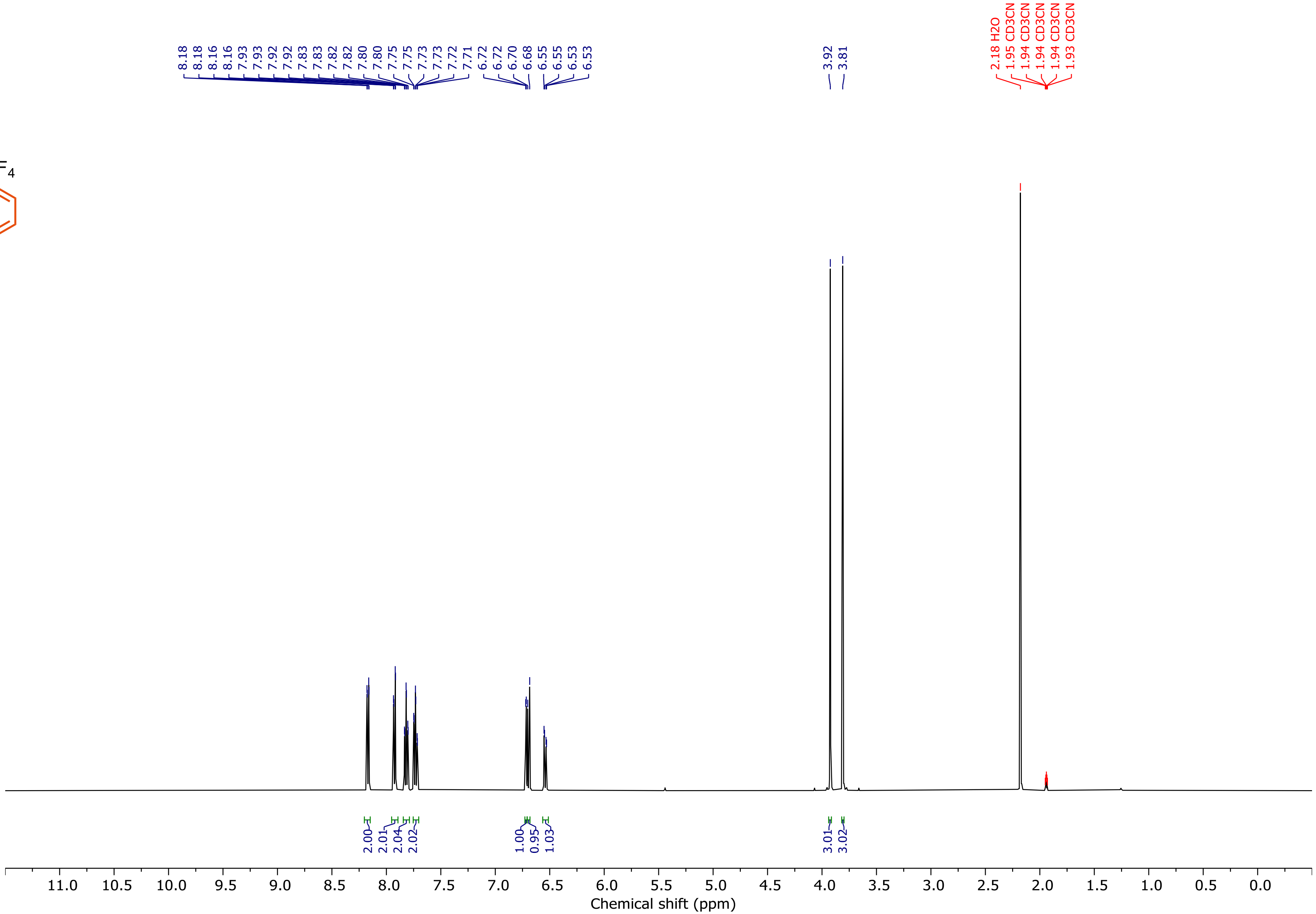

^{13}C NMR of 4a

CD_3CN, 151 MHz, 23 ºC.

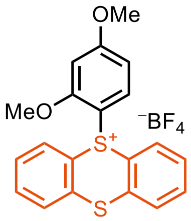

4a

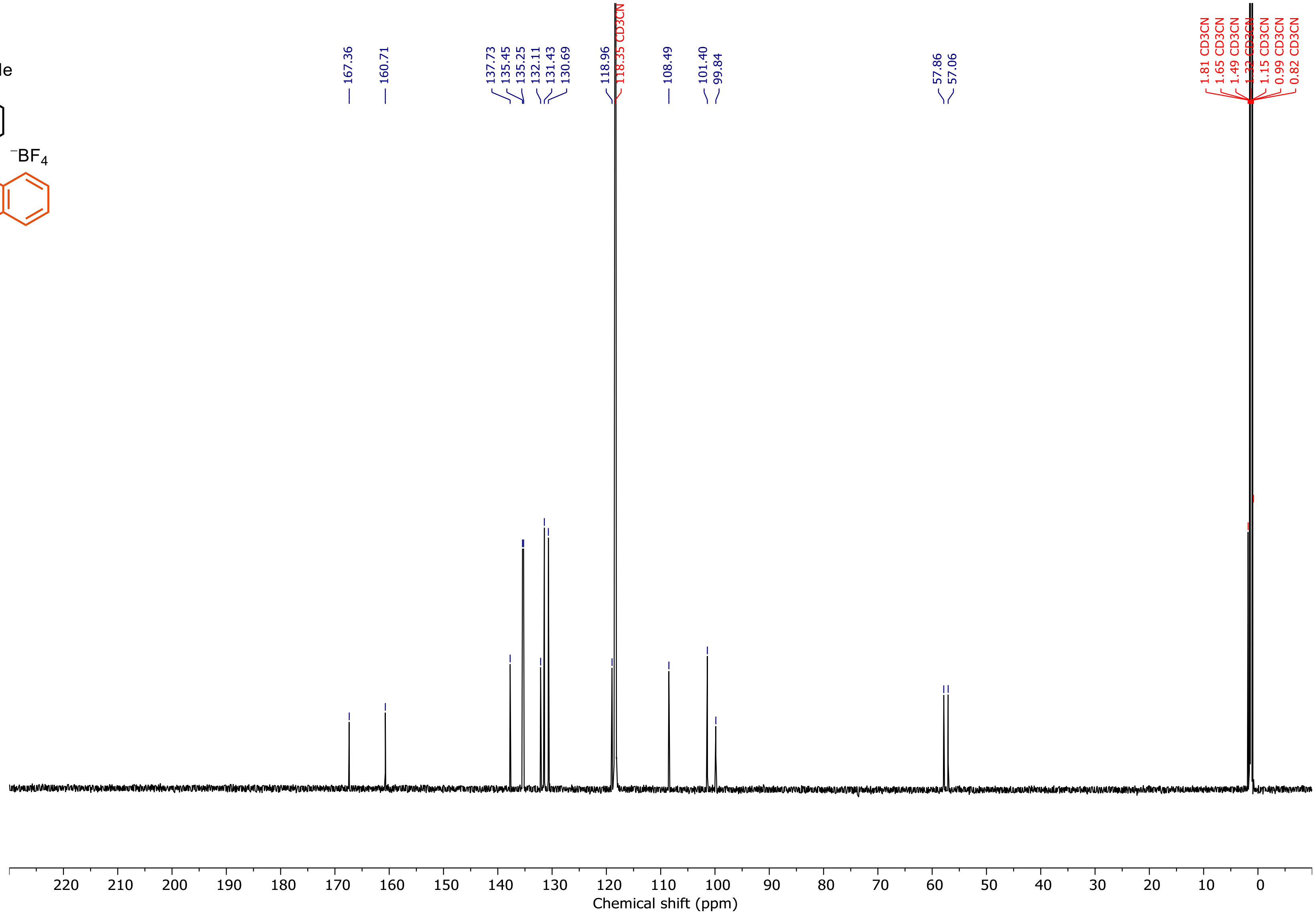

^{19}F NMR of 4a

CD_3CN, 565 MHz, 23 ºC.

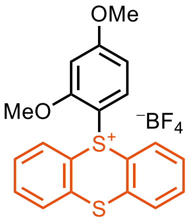

4a

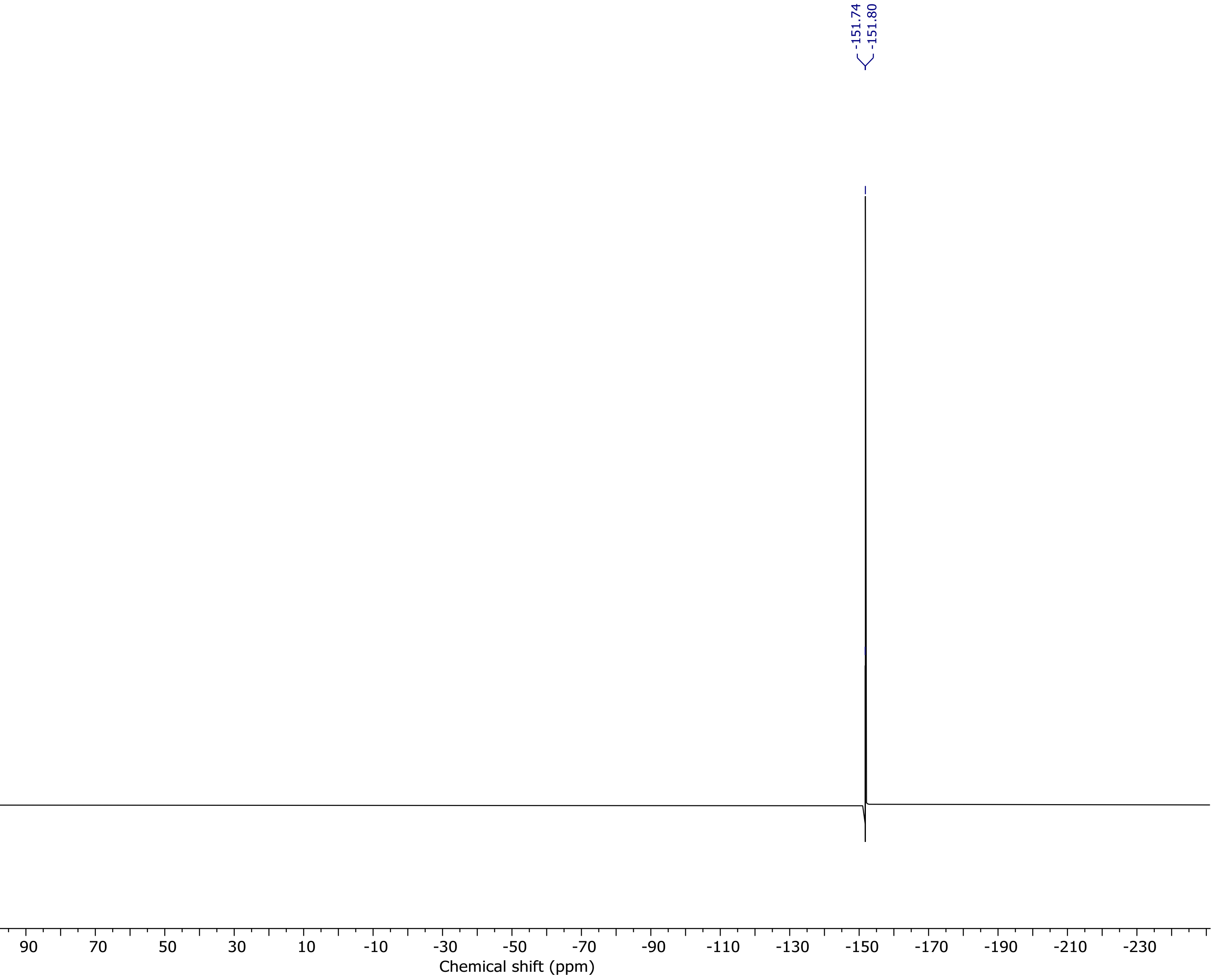

^{1}H NMR of 5a

CD_3CN, 600 MHz, 23 ºC.

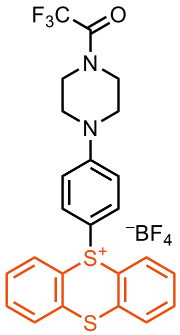

5a

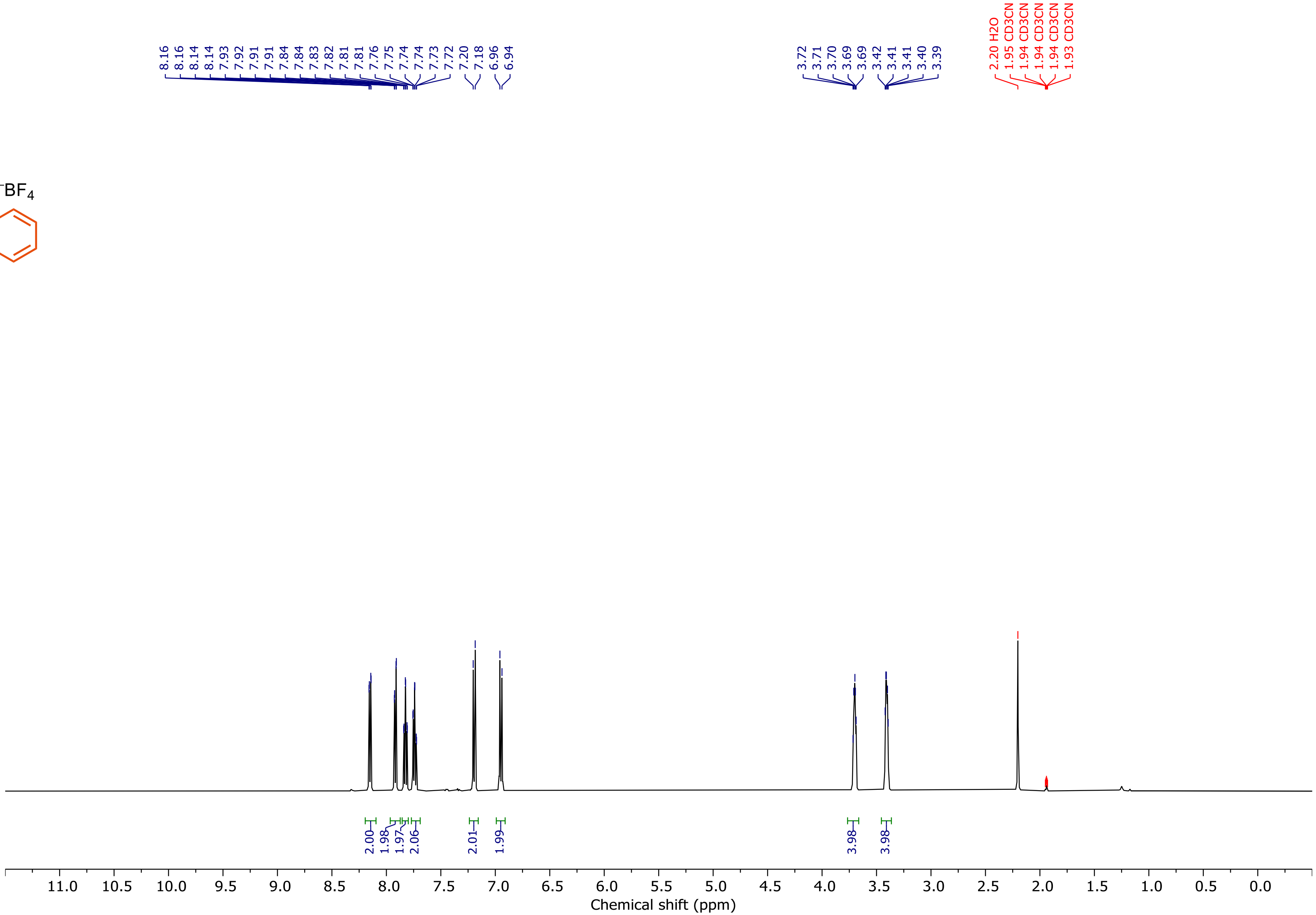

^{13}C NMR of 5a

CD_3CN, 151 MHz, 23 ºC.

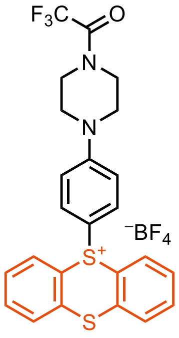

5a

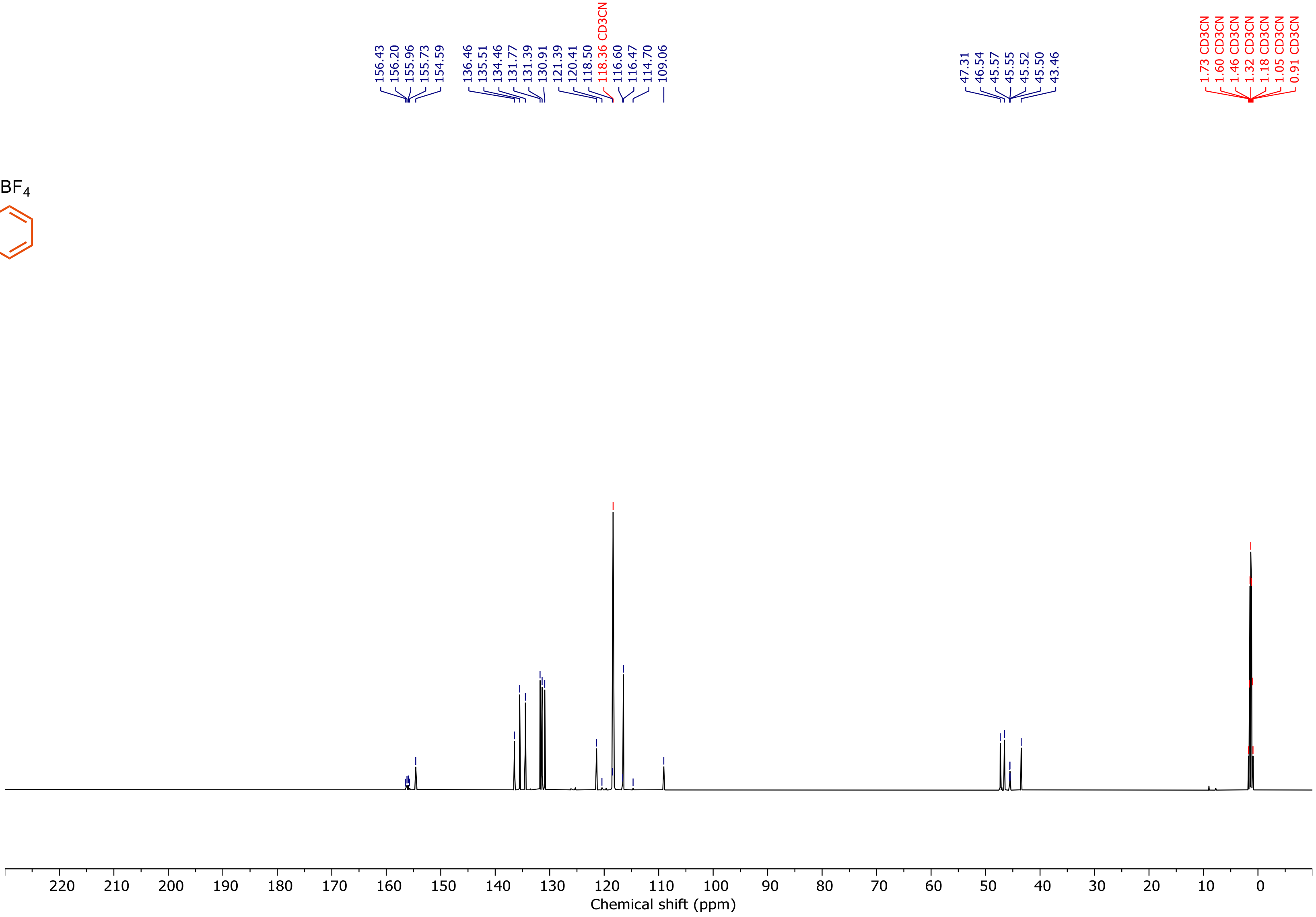

^{19}F NMR of 5a

CD_3CN, 565 MHz, 23 ºC.

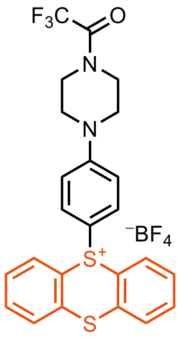

5a

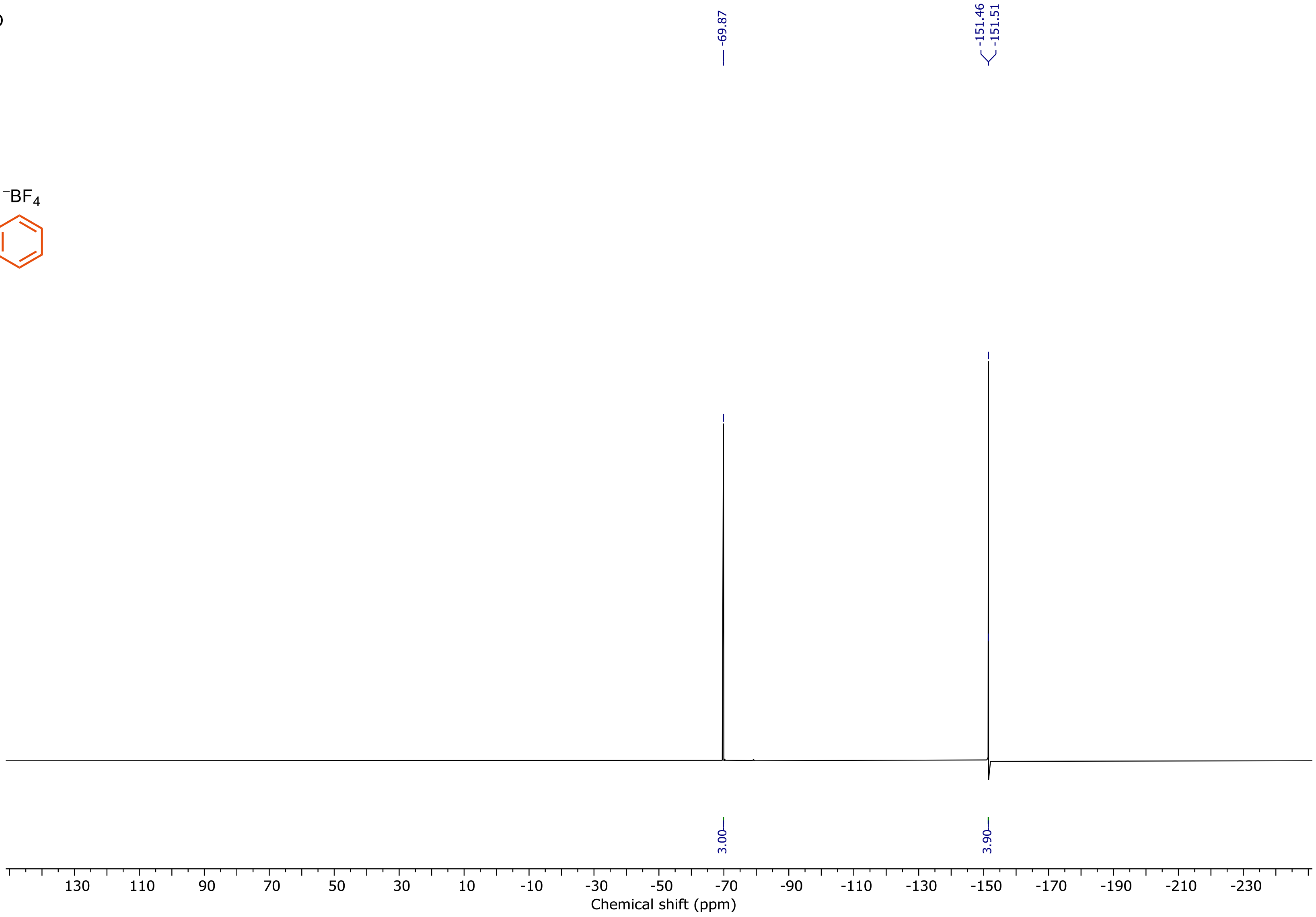

^{1}H NMR of 6a

CD_3CN, 600 MHz, 23 ºC.

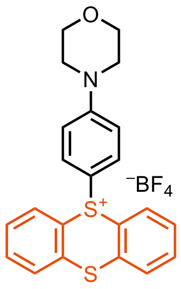

6a

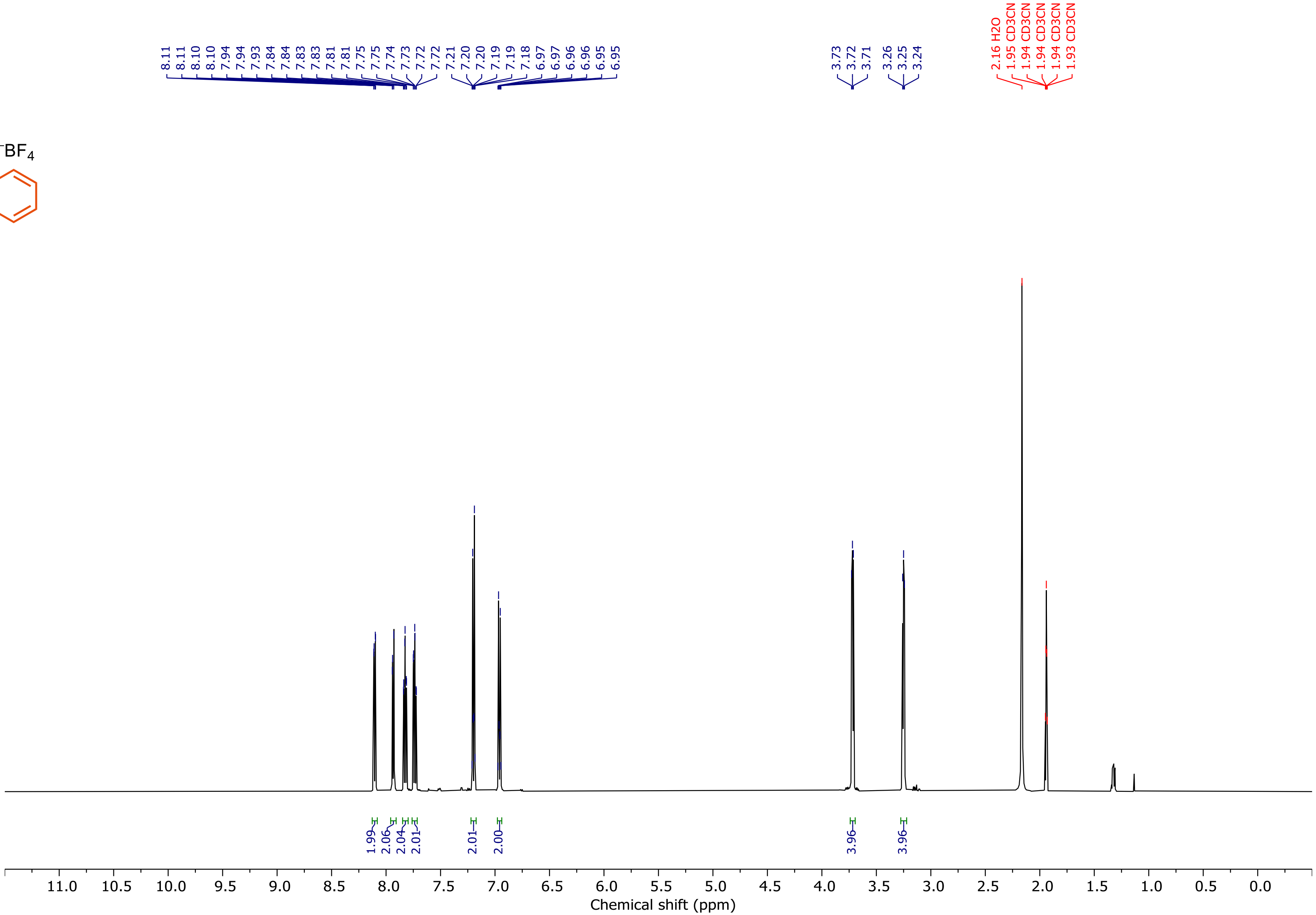

^{13}C NMR of 6a

CD_3CN, 151 MHz, 23 ºC.

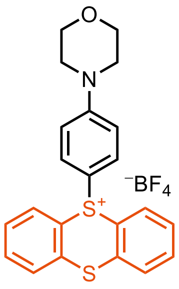

6a

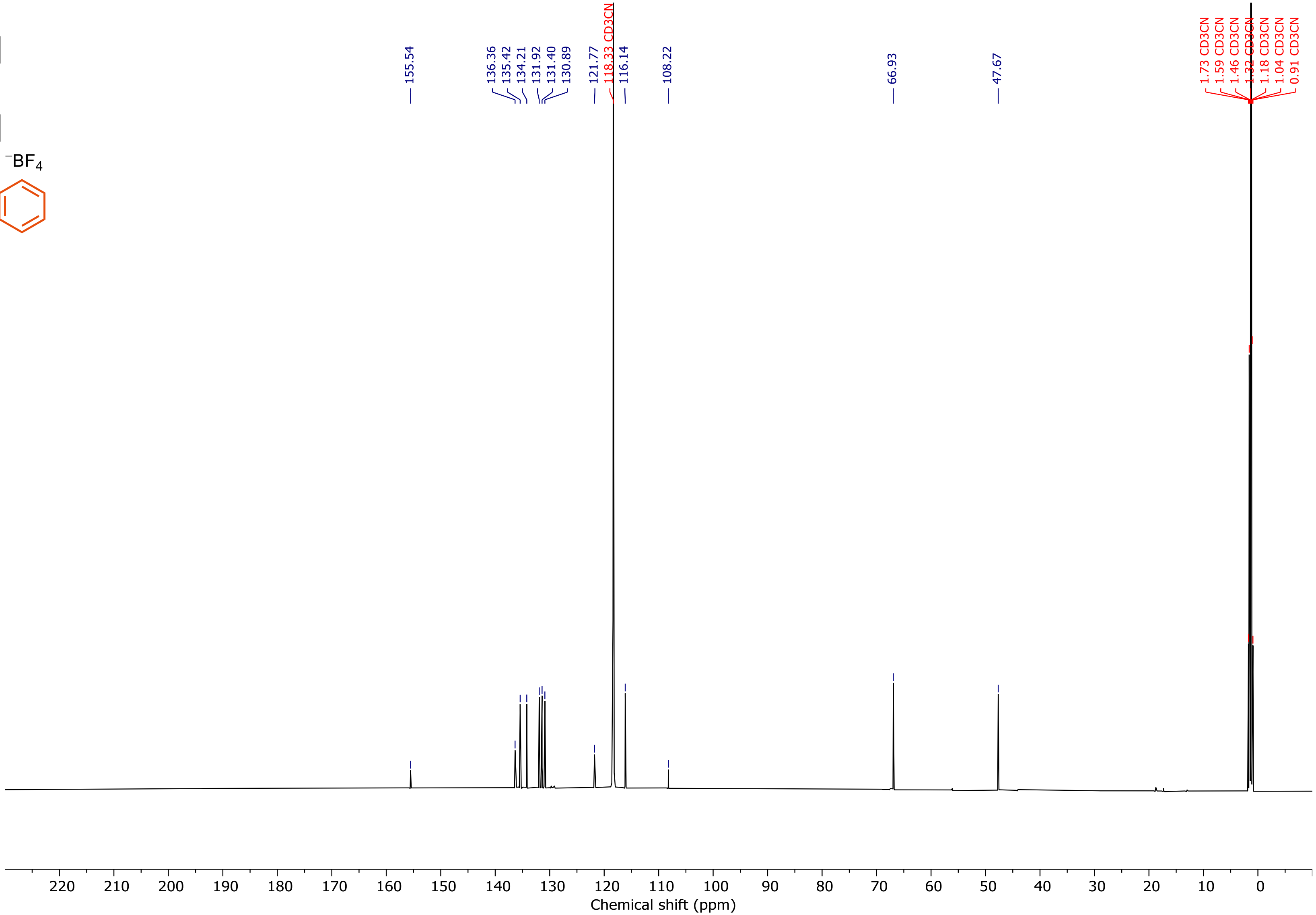

^{19}F NMR of 6a

CD_3CN, 565 MHz, 23 ºC.

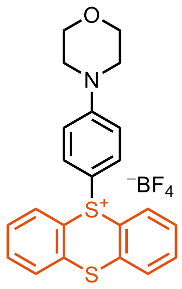

6a

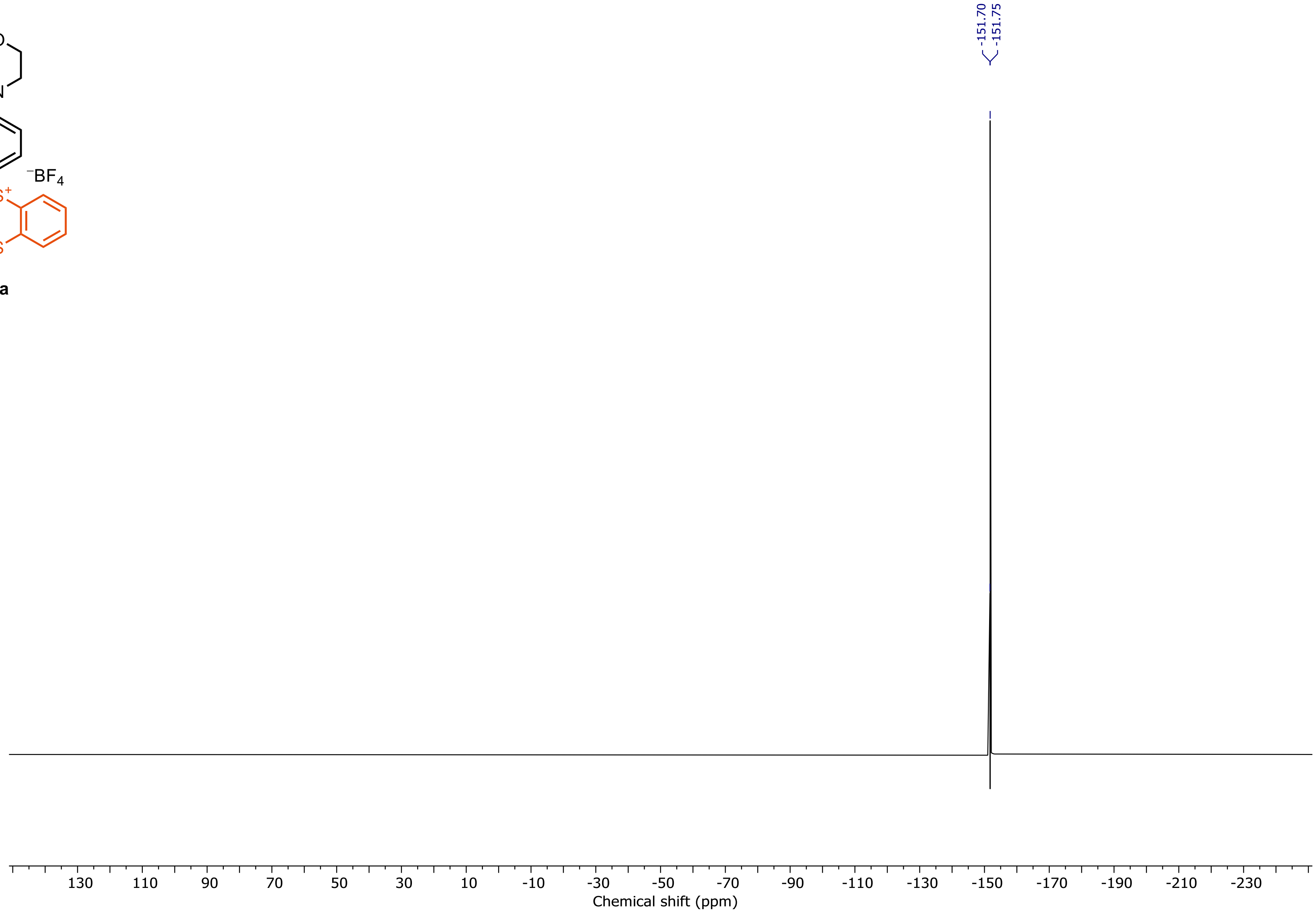

^{1}H NMR of 7a

CD_3CN, 500 MHz, 23 ºC.

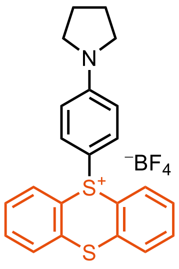

7a

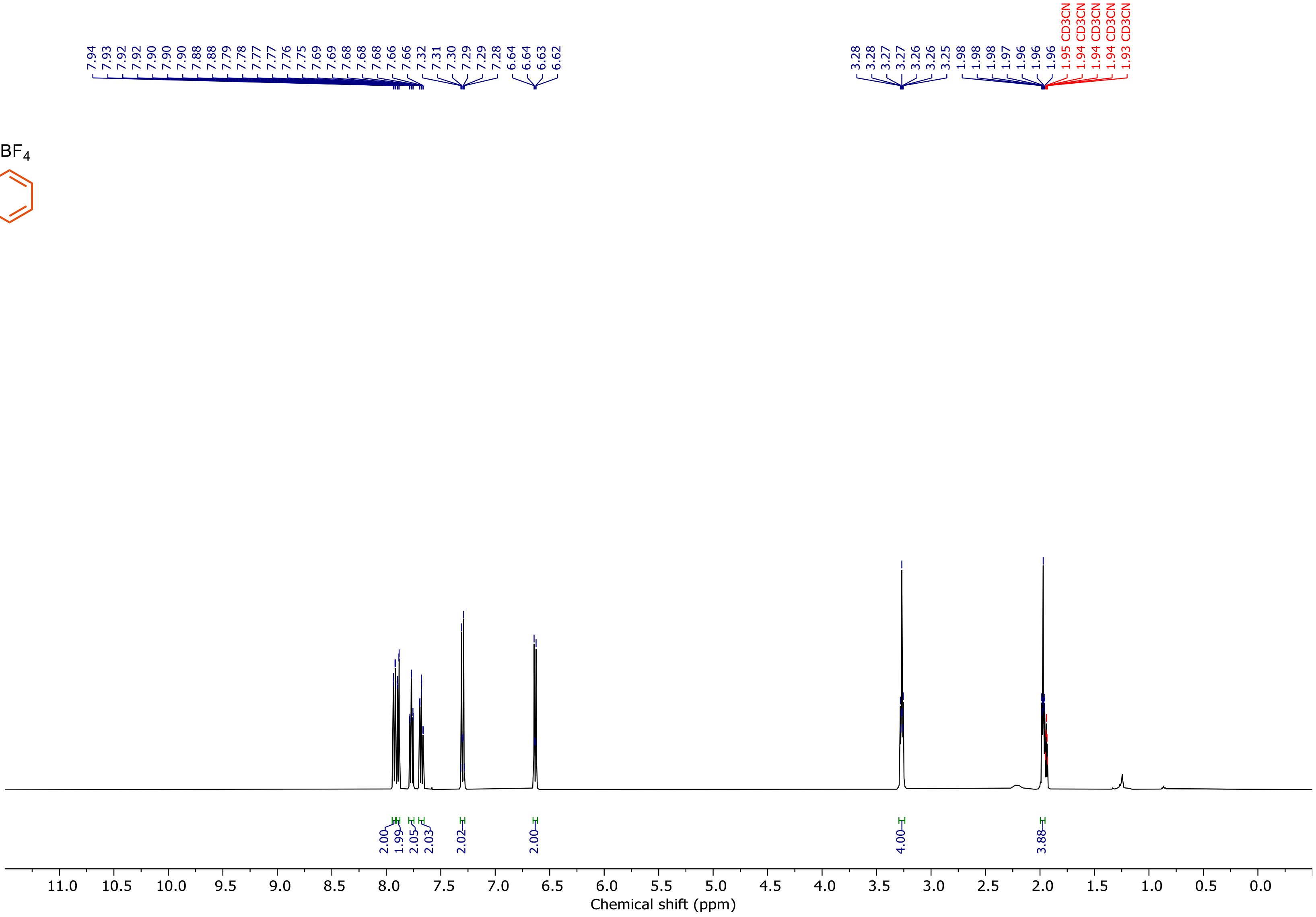

^{13}C NMR of 7a

CD_3CN, 126 MHz, 23 ºC.

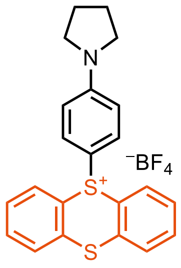

7a

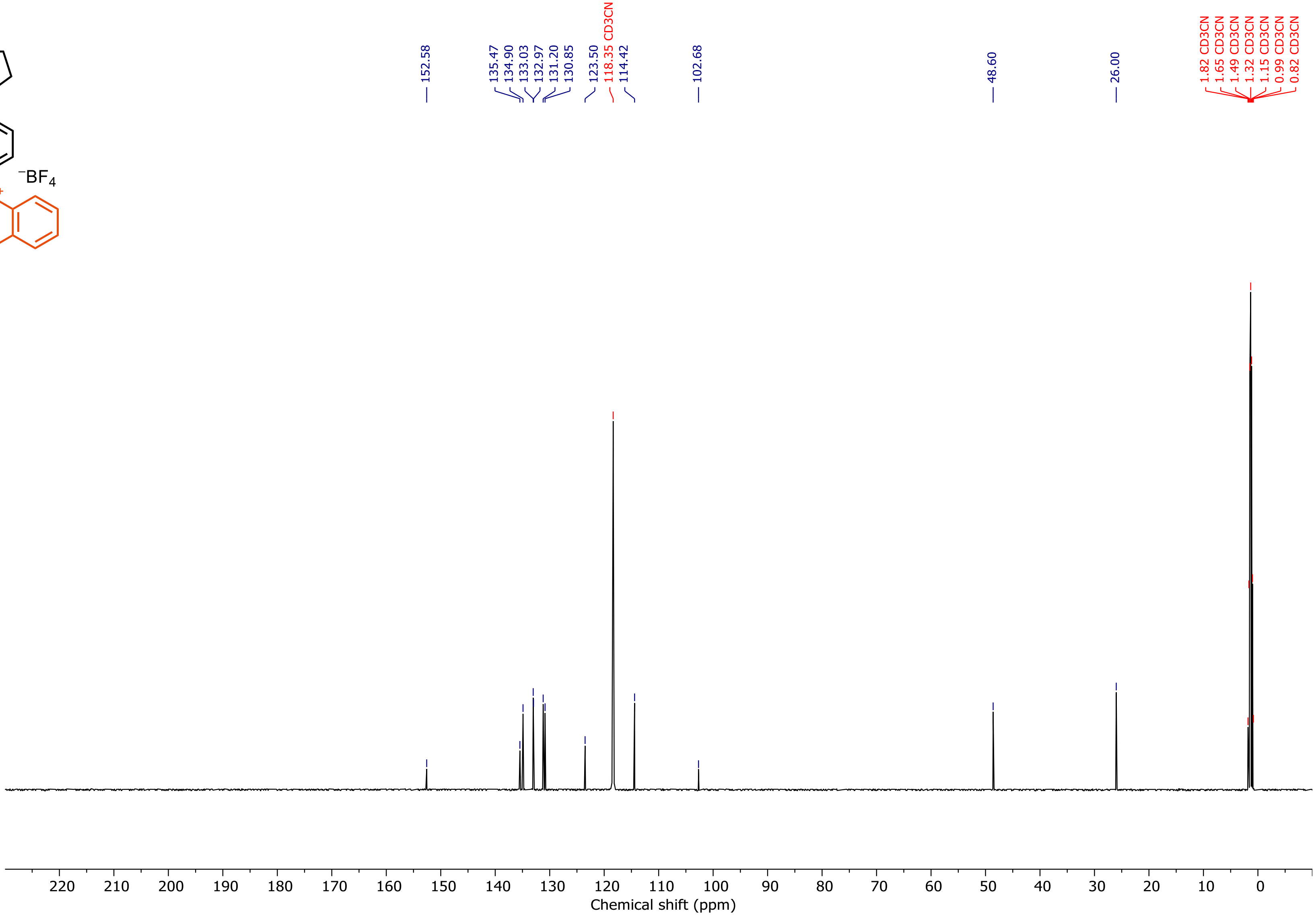

^{19}F NMR of 7a

CD_3CN, 471 MHz, 23 ºC.

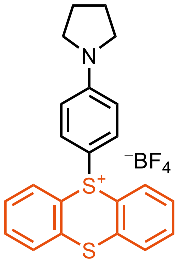

7a

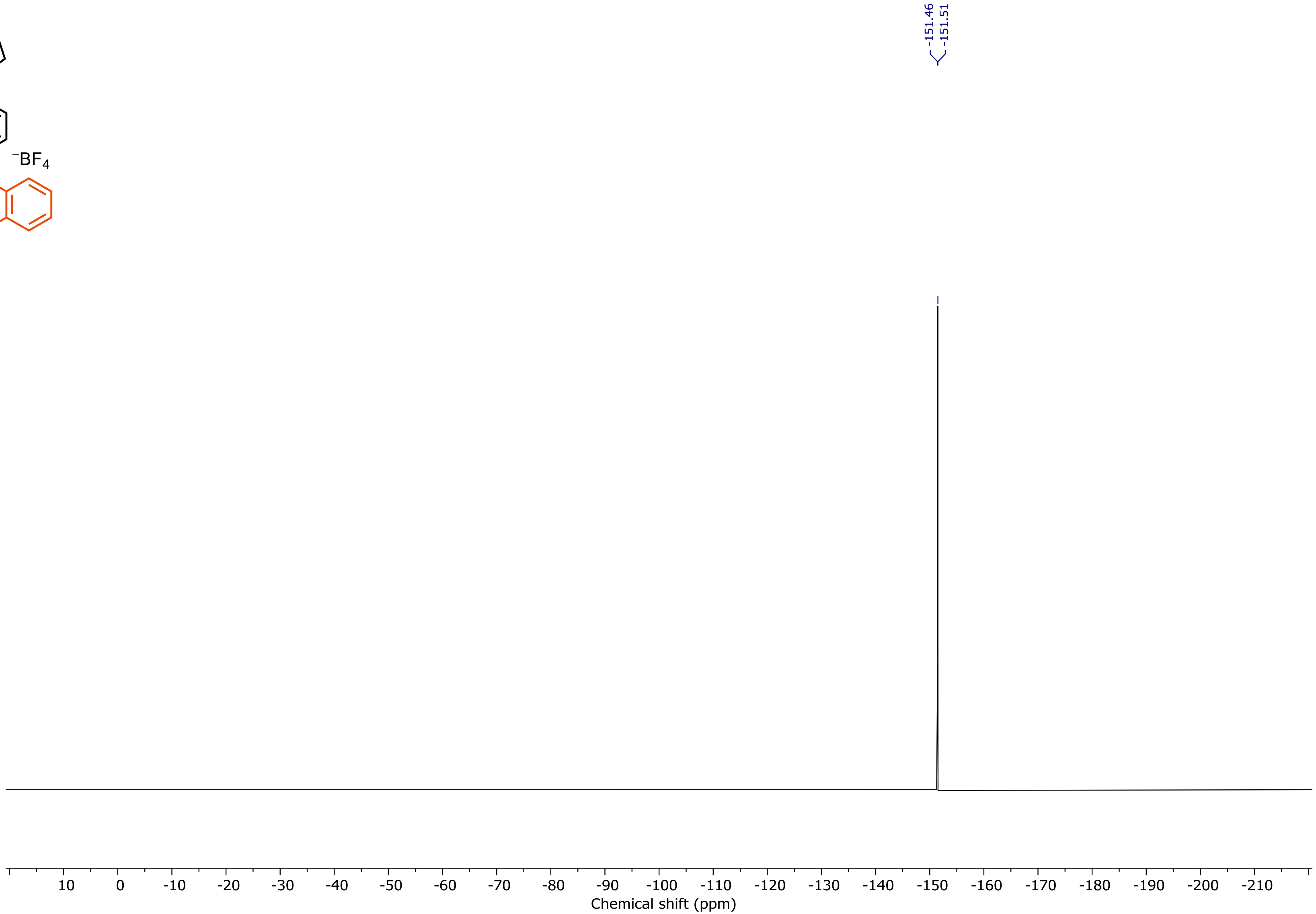

^{1}H NMR of 8a

CD_3CN, 500 MHz, 23 ºC.

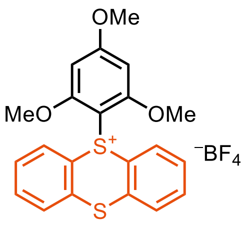

8a

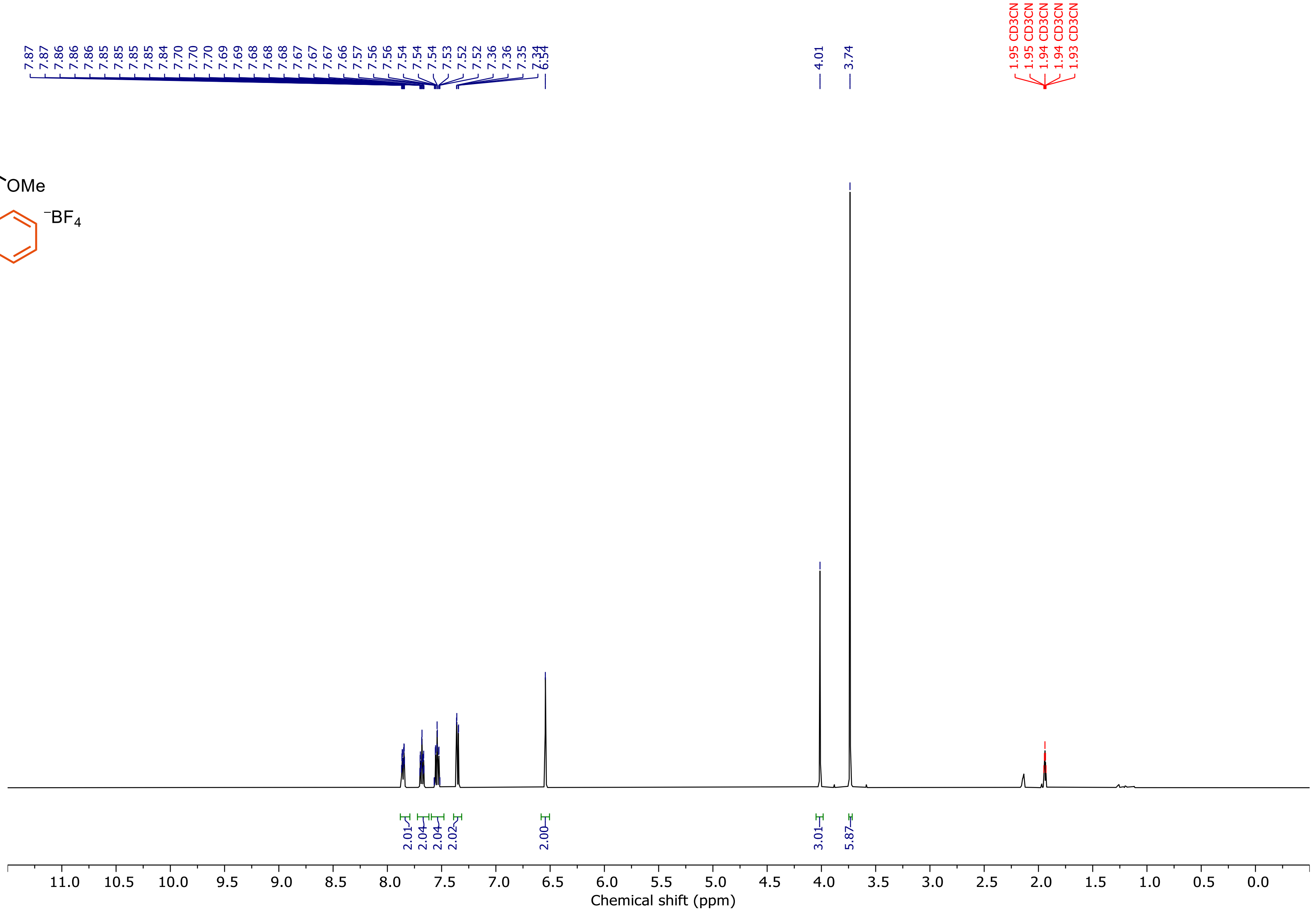

^{13}C NMR of 8a

CD_3CN, 126 MHz, 23 ºC.

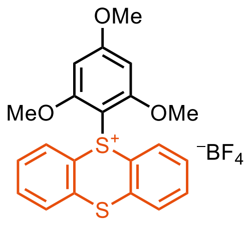

8a

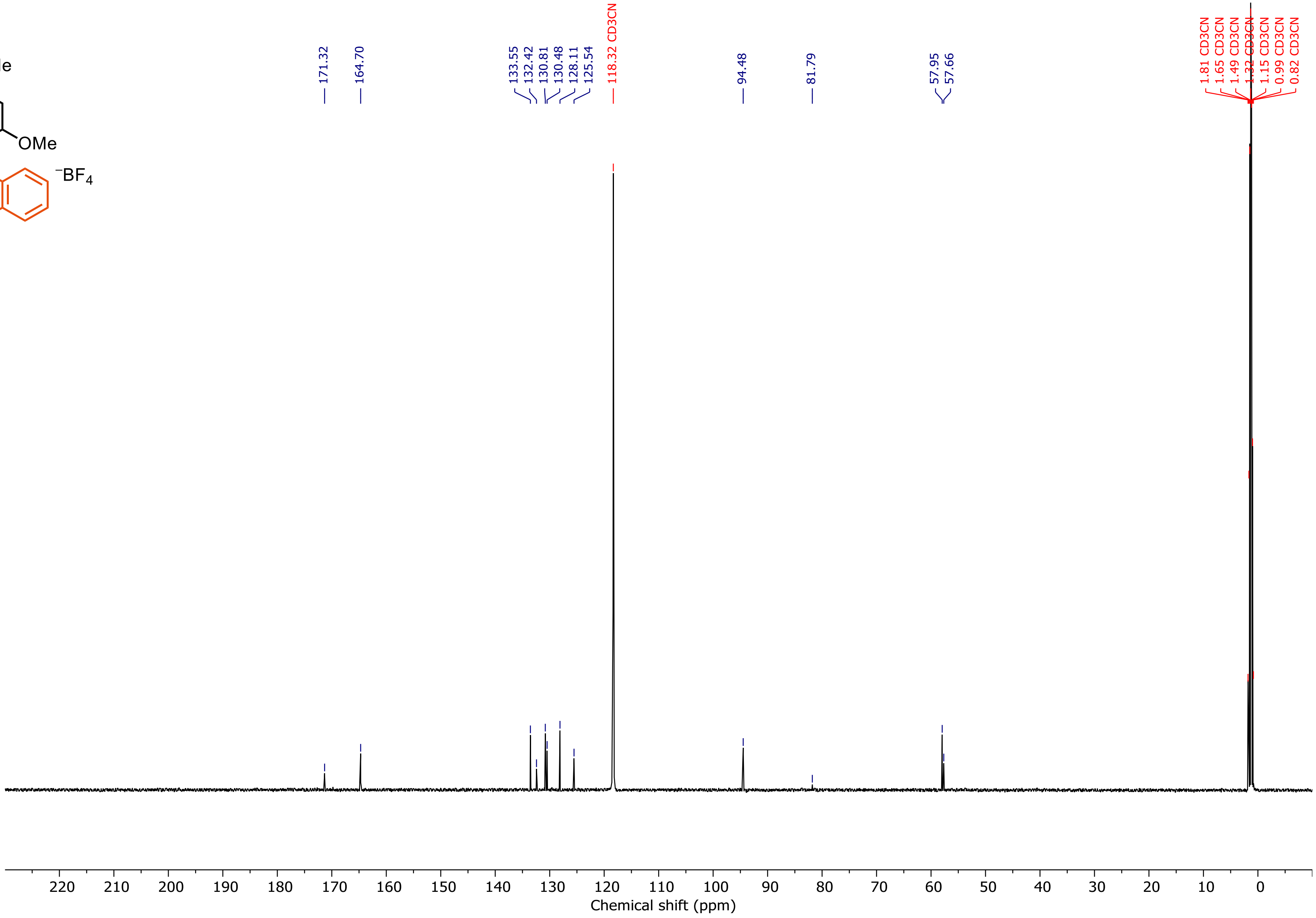

^{19}F NMR of 8a

CD_3CN, 471 MHz, 23 ºC.

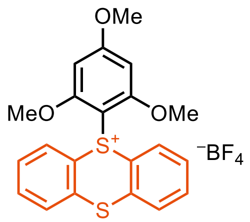

8a

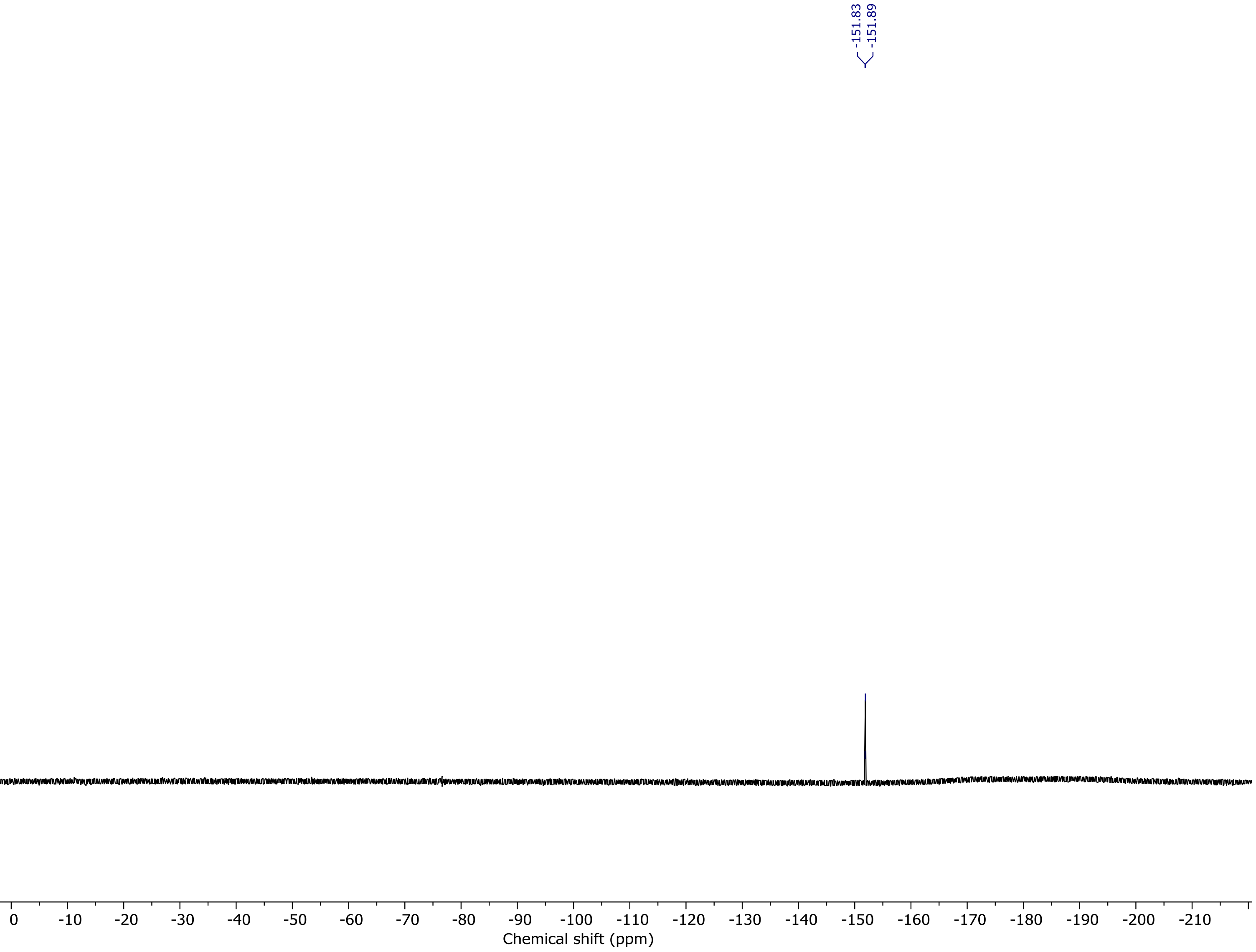

^{1}H NMR of 9

$CDCl_3$, 600 MHz, 23 ºC.

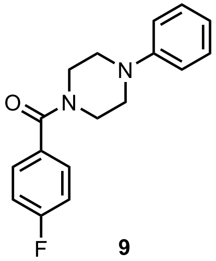

9

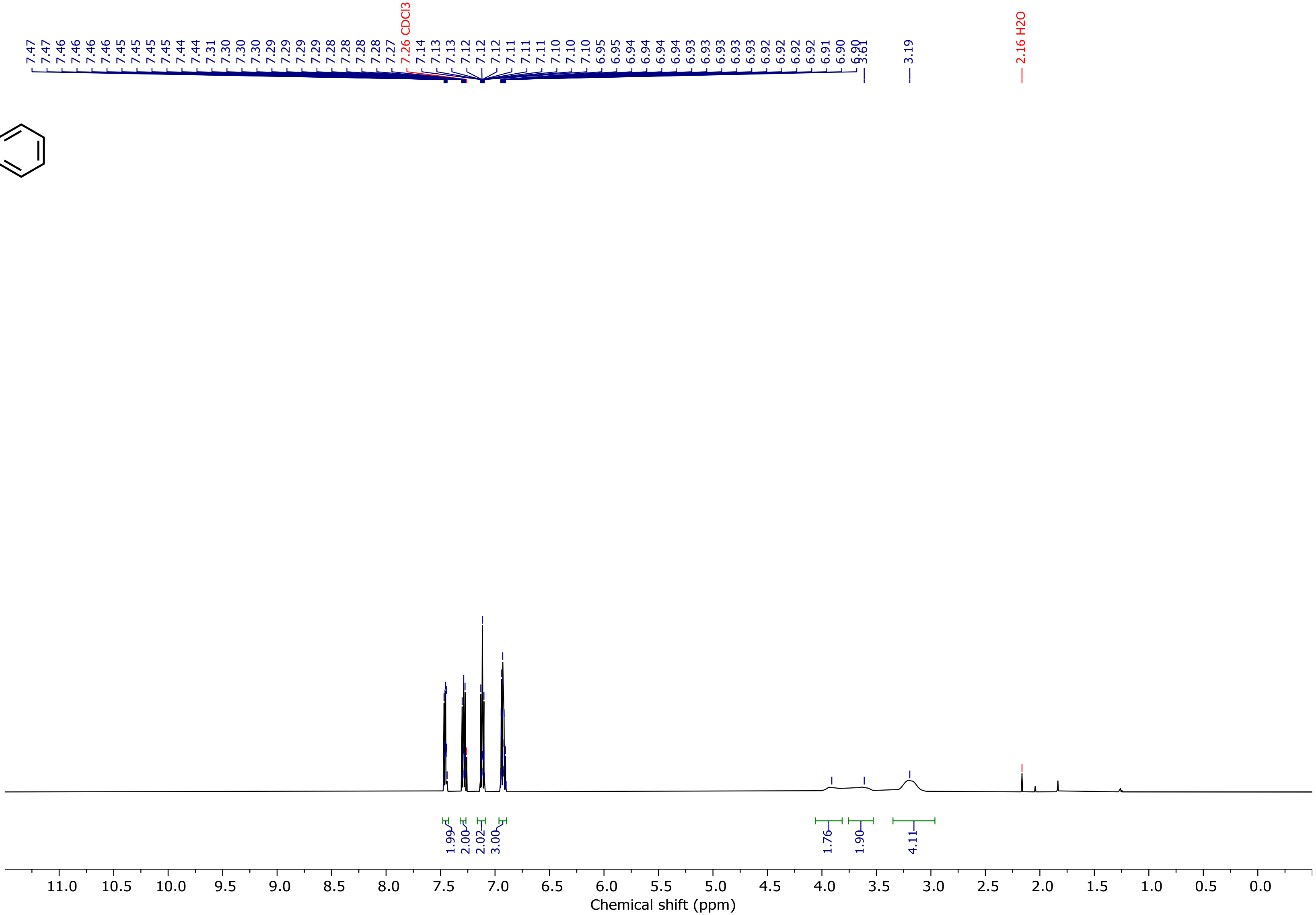

^{13}C NMR of 9

$CDCl_3$, 151 MHz, 23 ºC.

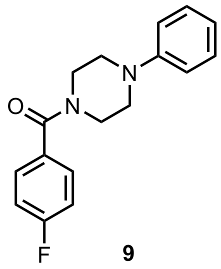

9

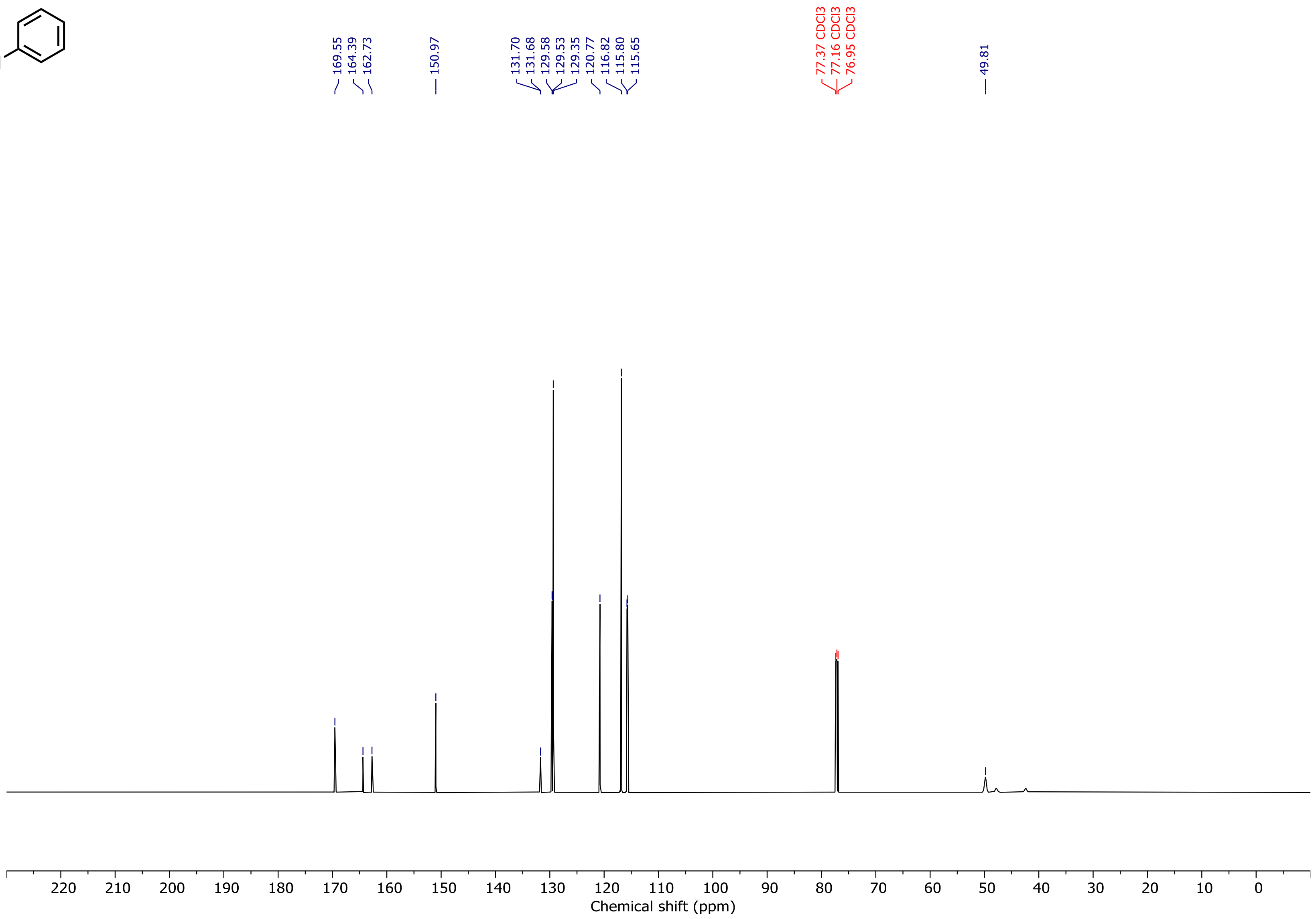

^{19}F NMR of 9

$CDCl_3$, 565 MHz, 23 ºC.

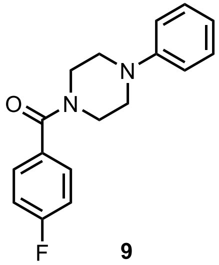

9

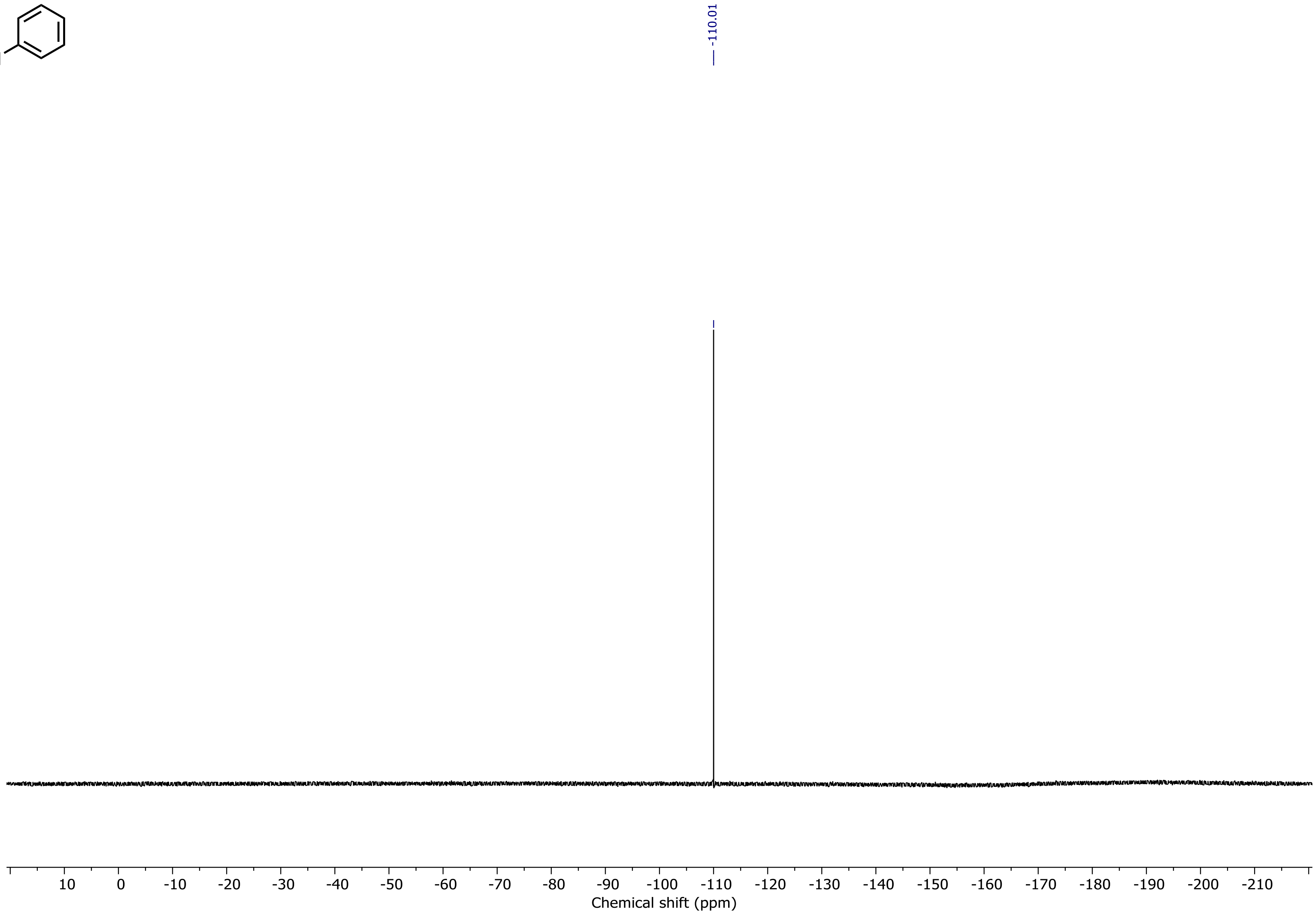

^{1}H NMR of 9a

$CDCl_3$, 600 MHz, 23 ºC.

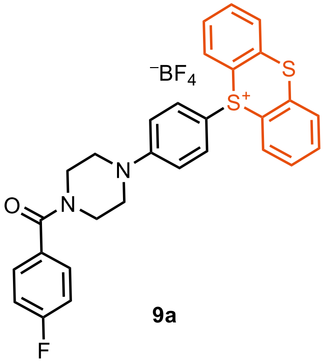

9a

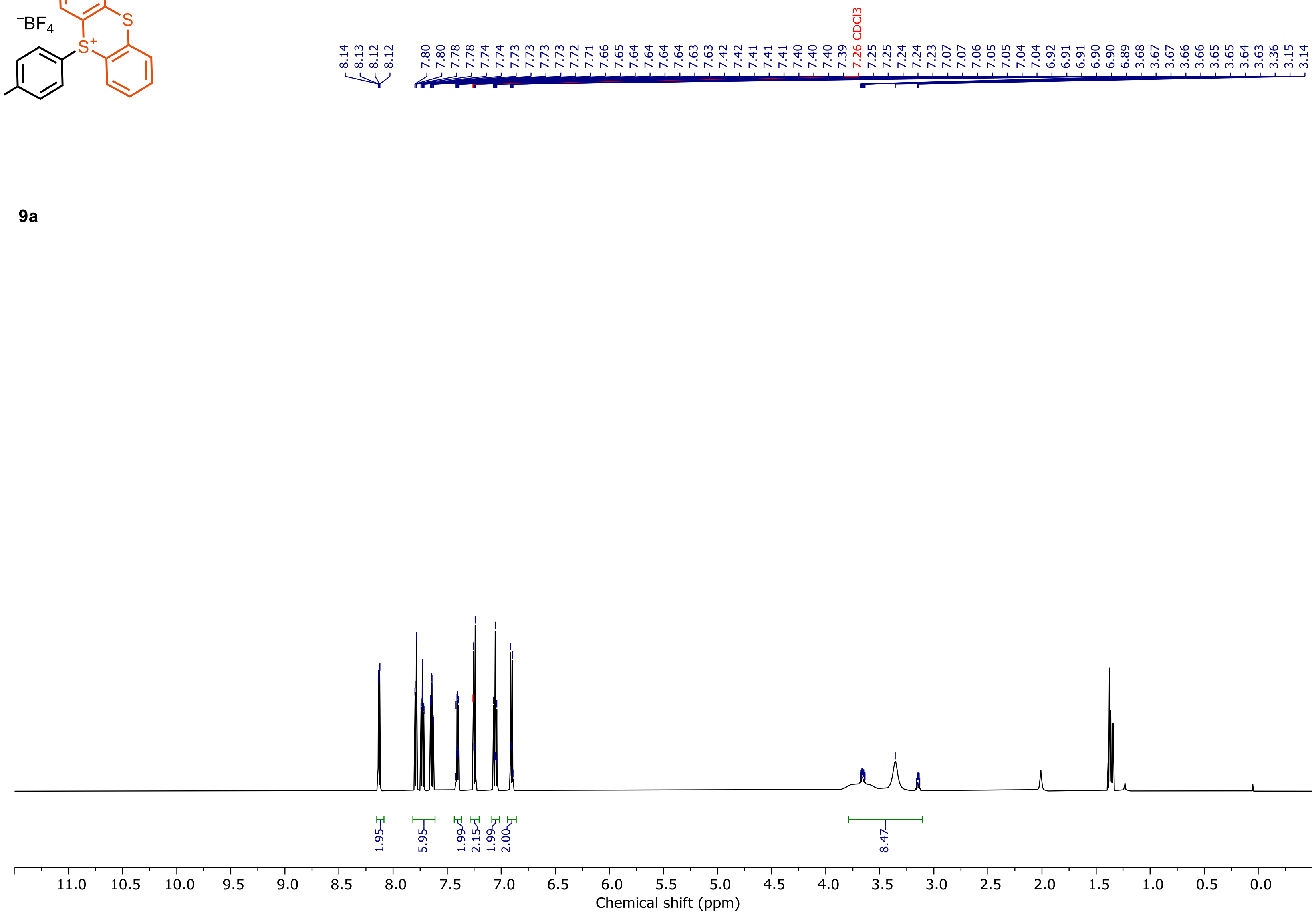

^{13}C NMR of 9a

$CDCl_3$, 151 MHz, 23 ºC.

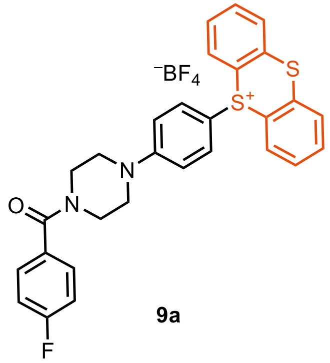

9a

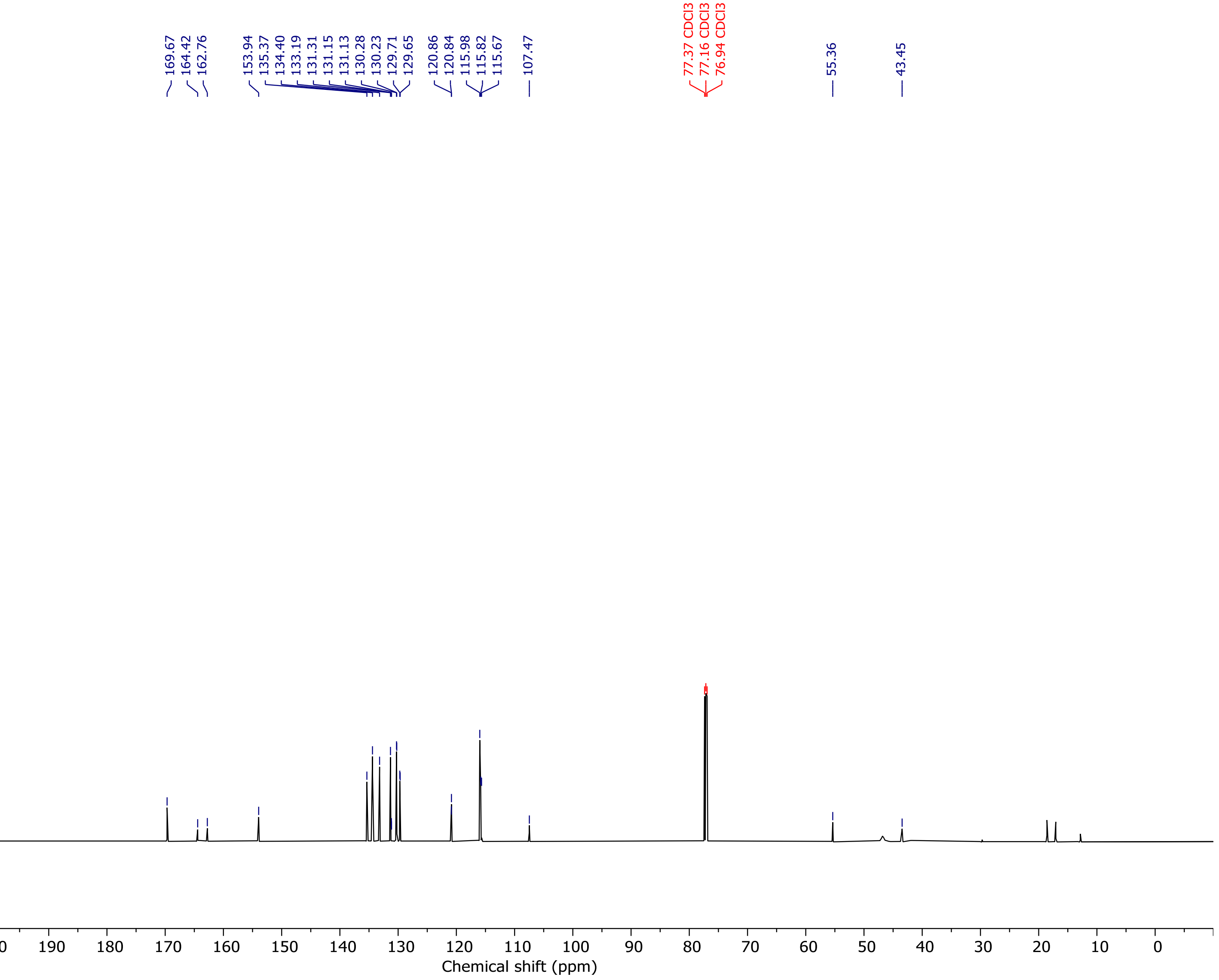

^{19}F NMR of 9a

$CDCl_3$, 565 MHz, 23 ºC.

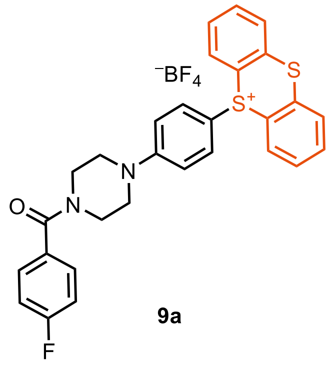

9a

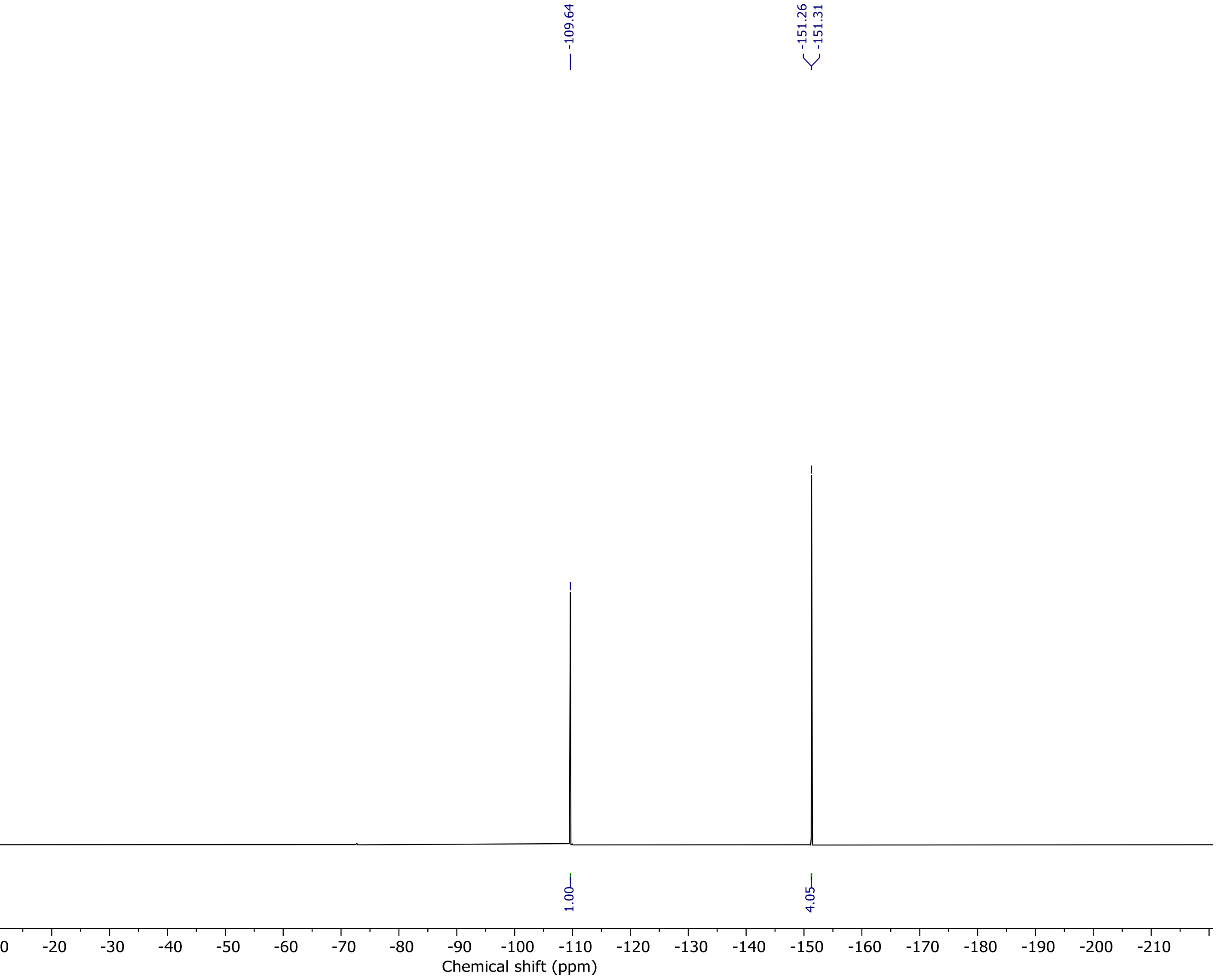

^{1}H NMR of 10a

CD_3CN, 500 MHz, 23 ºC.

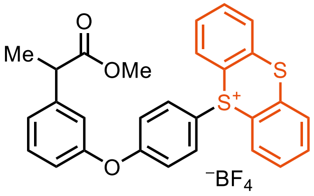

10a

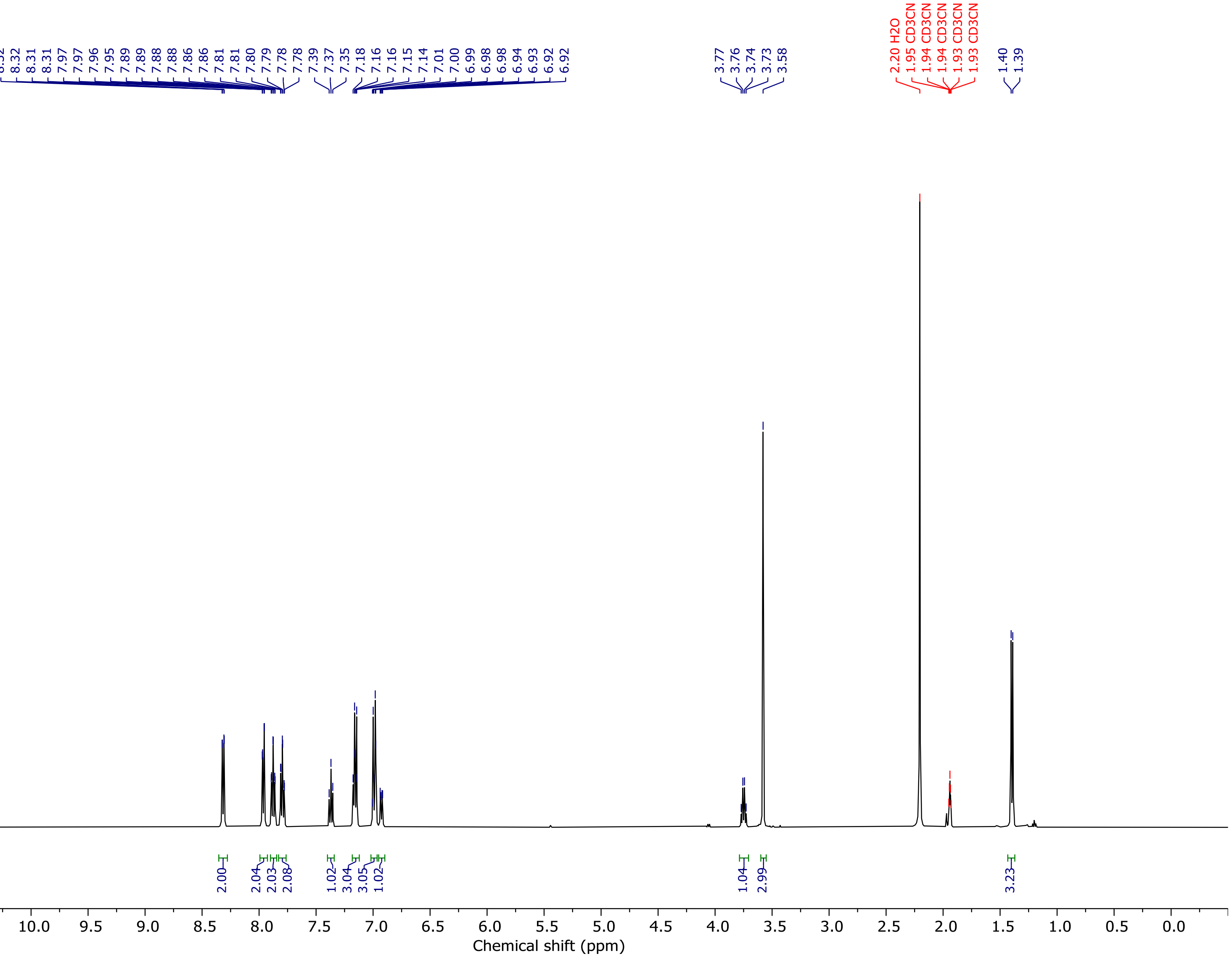

^{13}C NMR of 10a

CD_3CN, 126 MHz, 23 ºC.

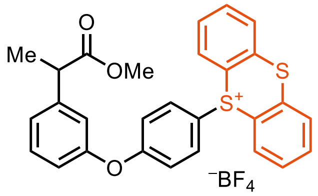

10a

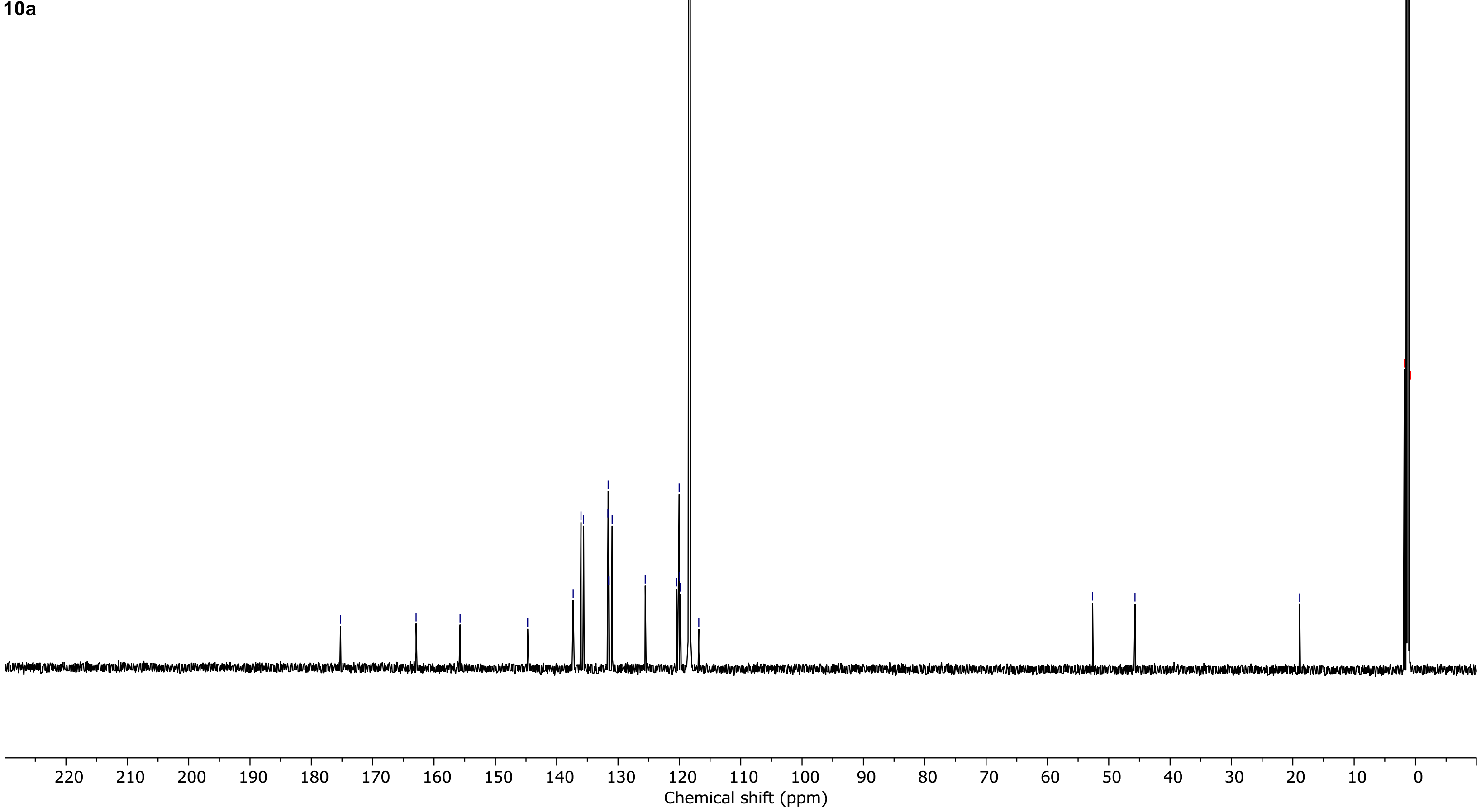

^{19}F NMR of 10a

CD_3CN, 471 MHz, 23 ºC.

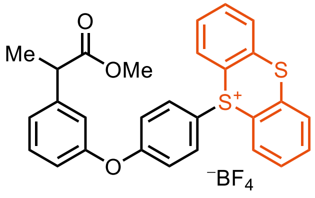

10a

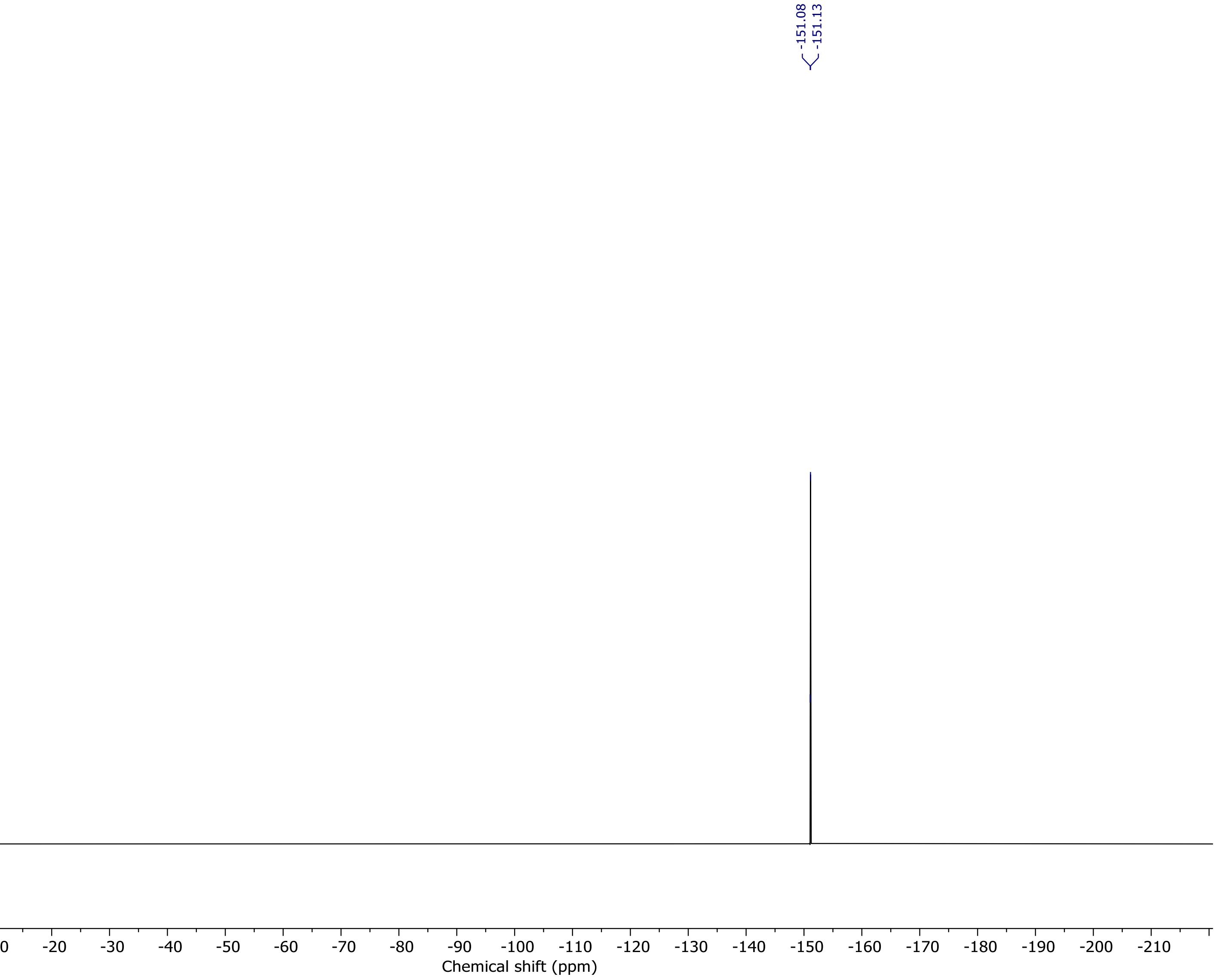

^{1}H NMR of 11a

CD_3CN, 500 MHz, 23 ºC.

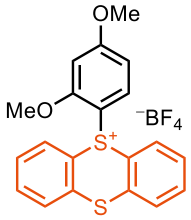

11a

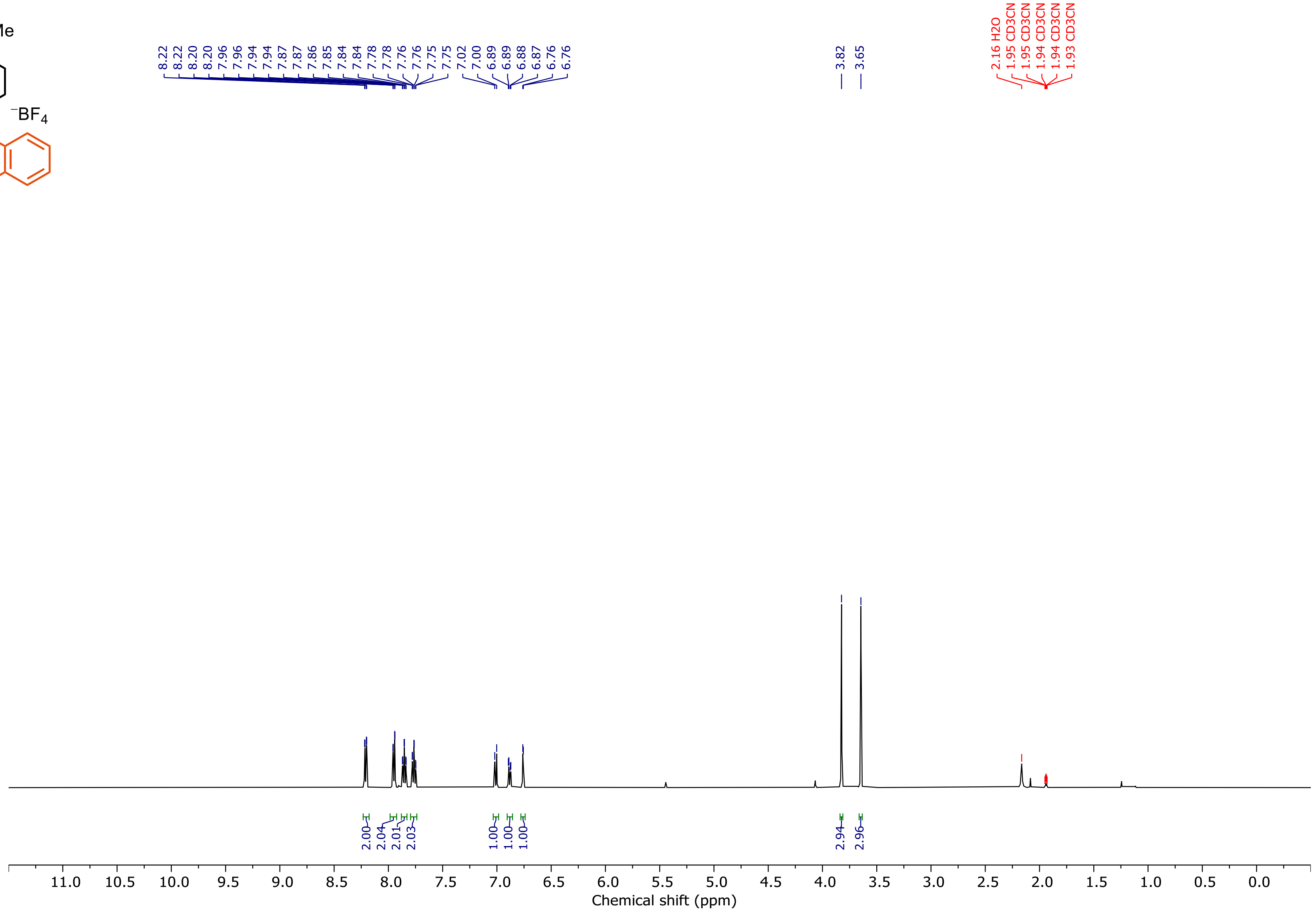

^{13}C NMR of 11a

CD_3CN, 126 MHz, 23 ºC.

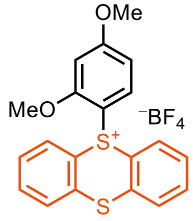

11a

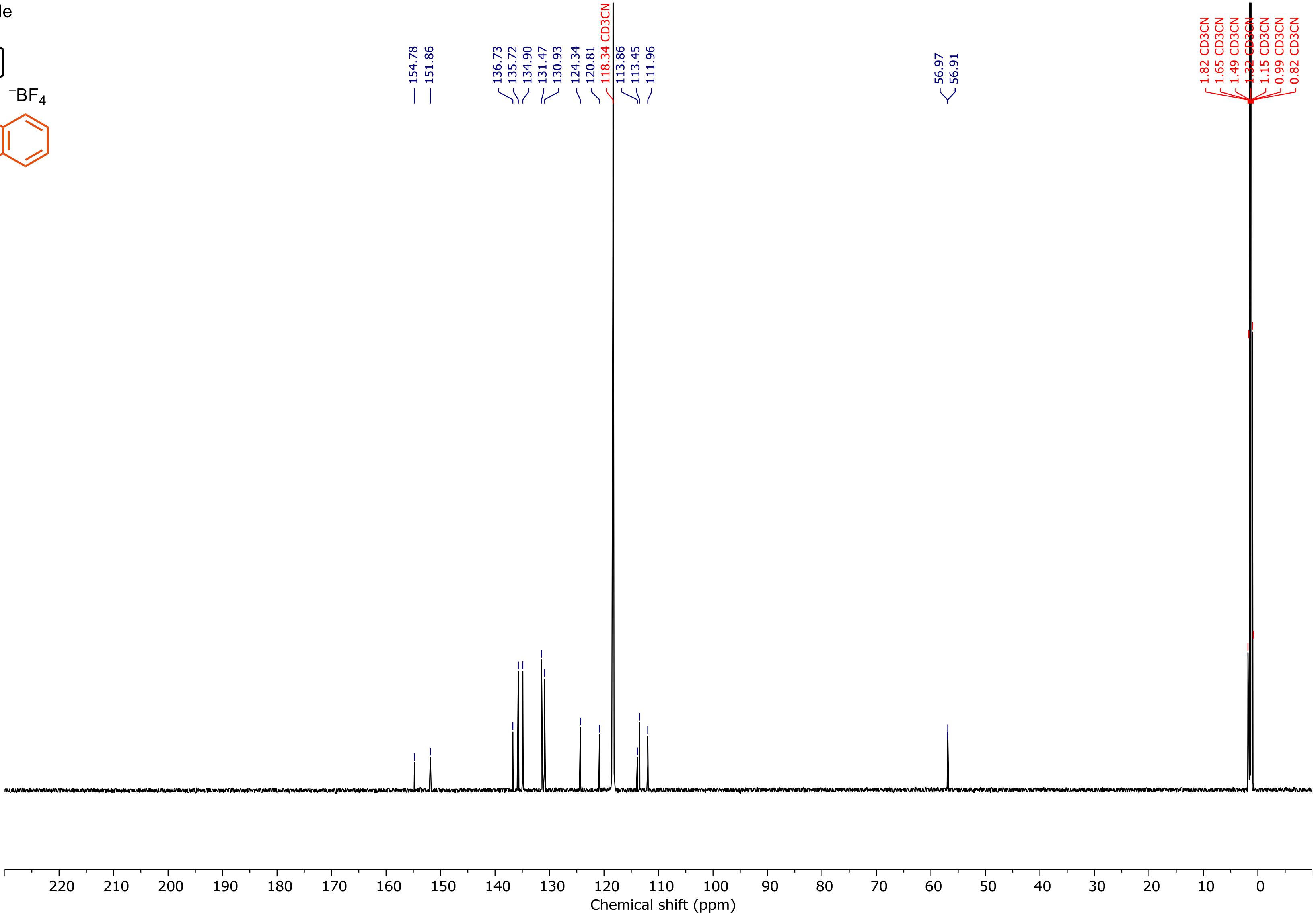

^{19}F NMR of 11a

CD_3CN, 471 MHz, 23 ºC.

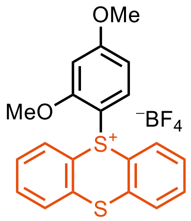

11a

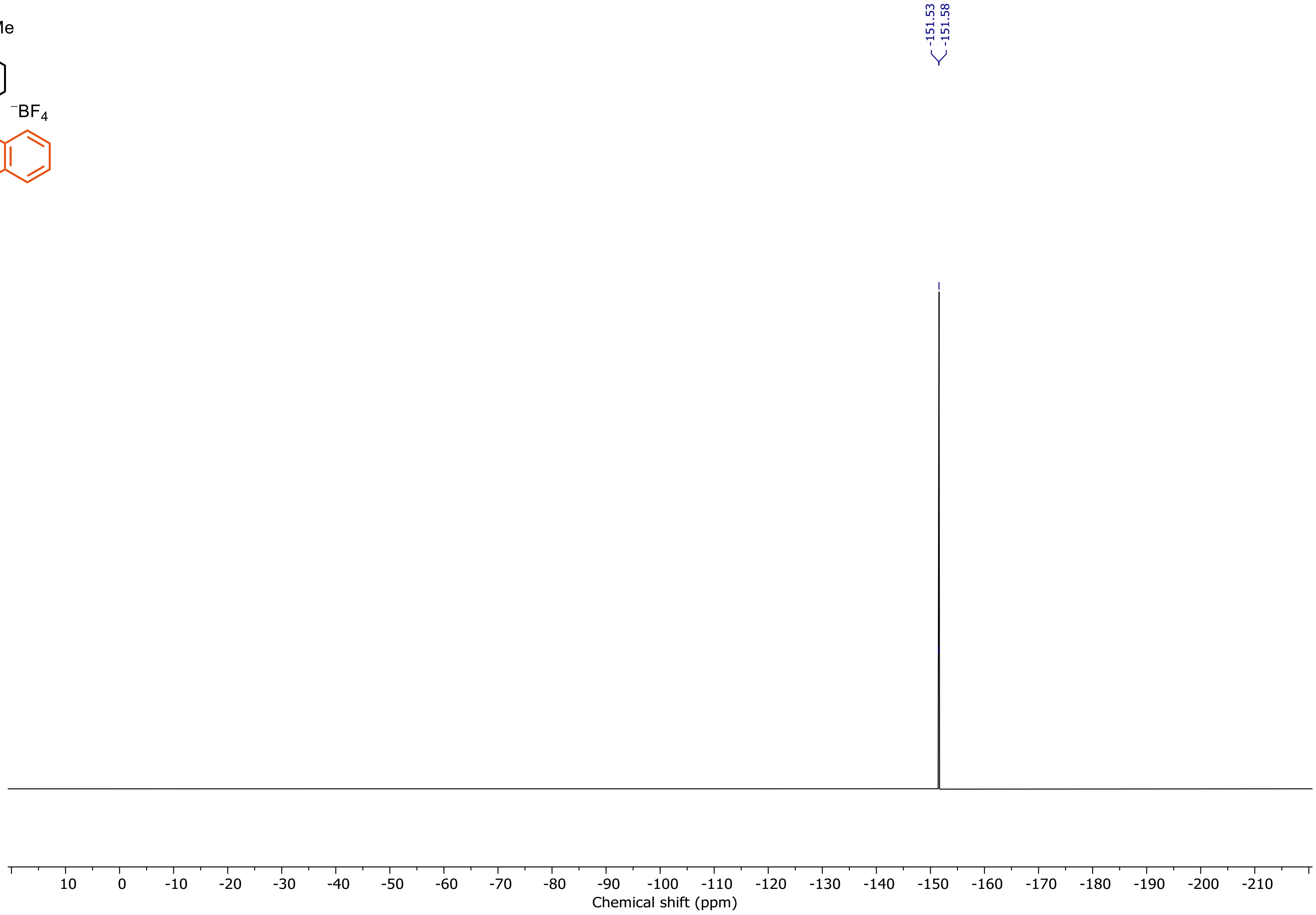

^{1}H NMR of 12-Ia

CD_3CN, 500 MHz, 23 ºC.

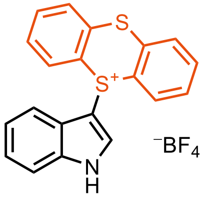

12-Ia

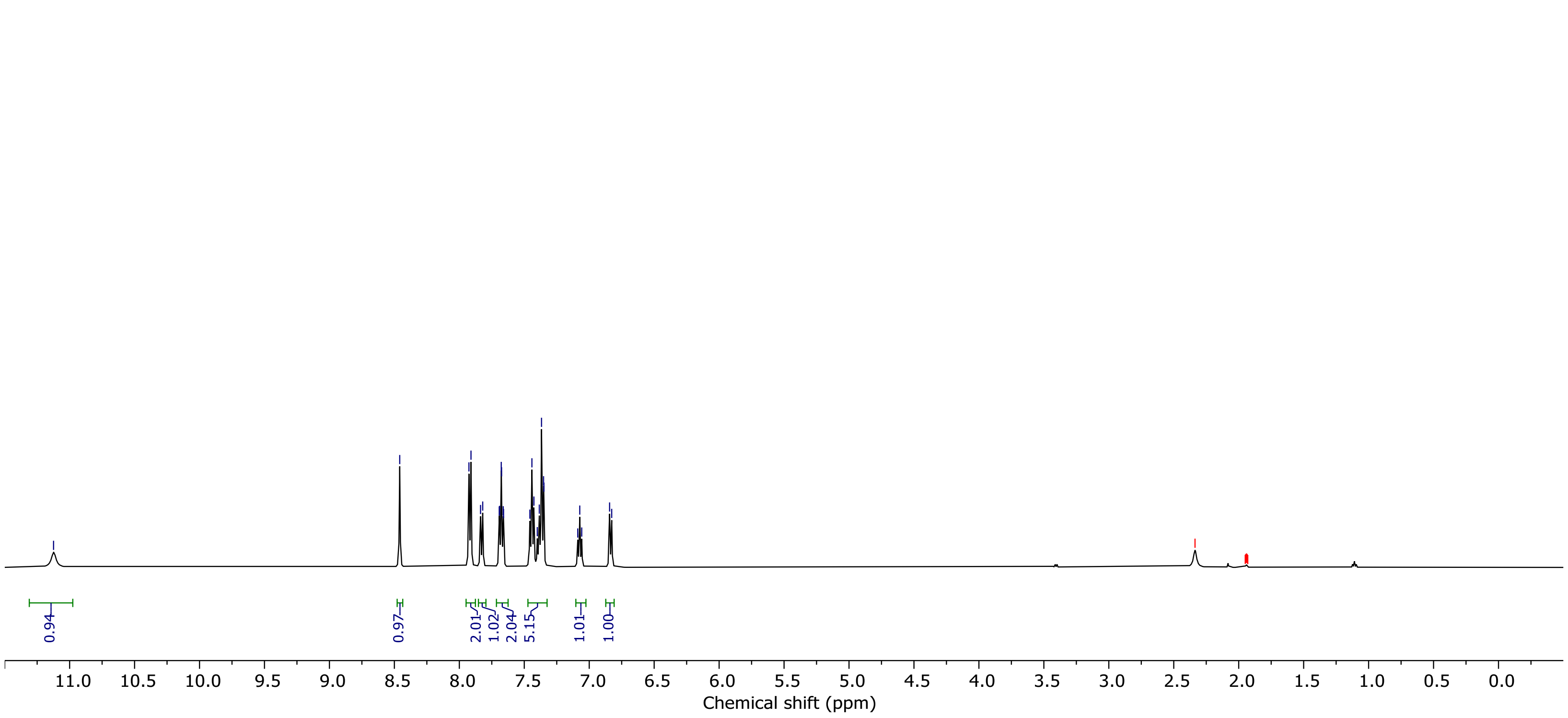

^{13}C NMR of 12-Ia

CD_3CN, 126 MHz, 23 ºC.

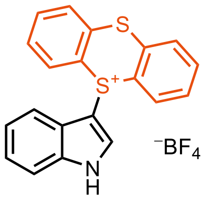

12-Ia

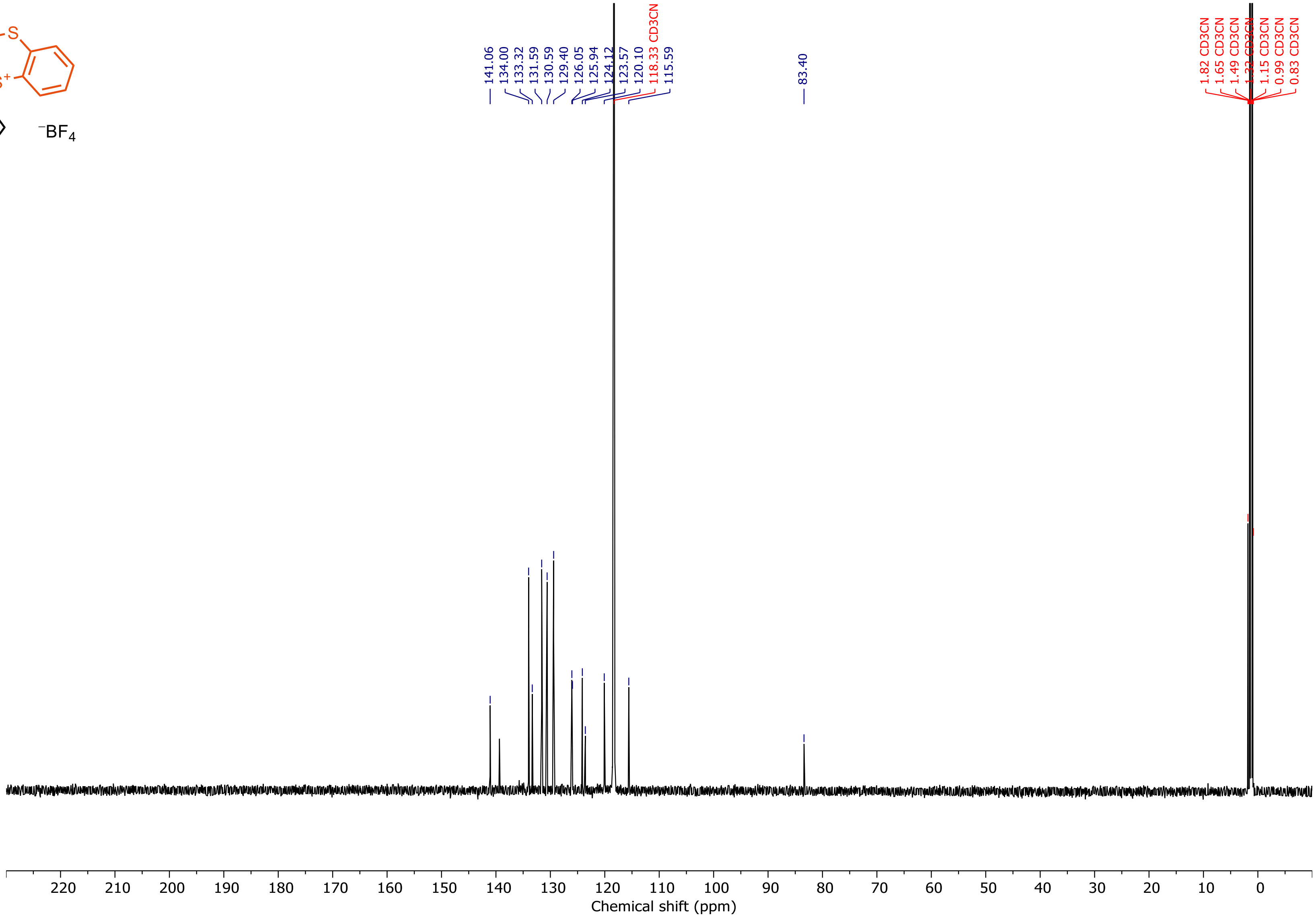

^{19}F NMR of 12-Ia

CD_3CN, 471 MHz, 23 ºC.

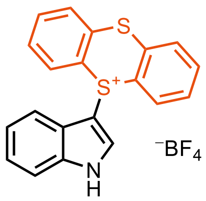

12-Ia

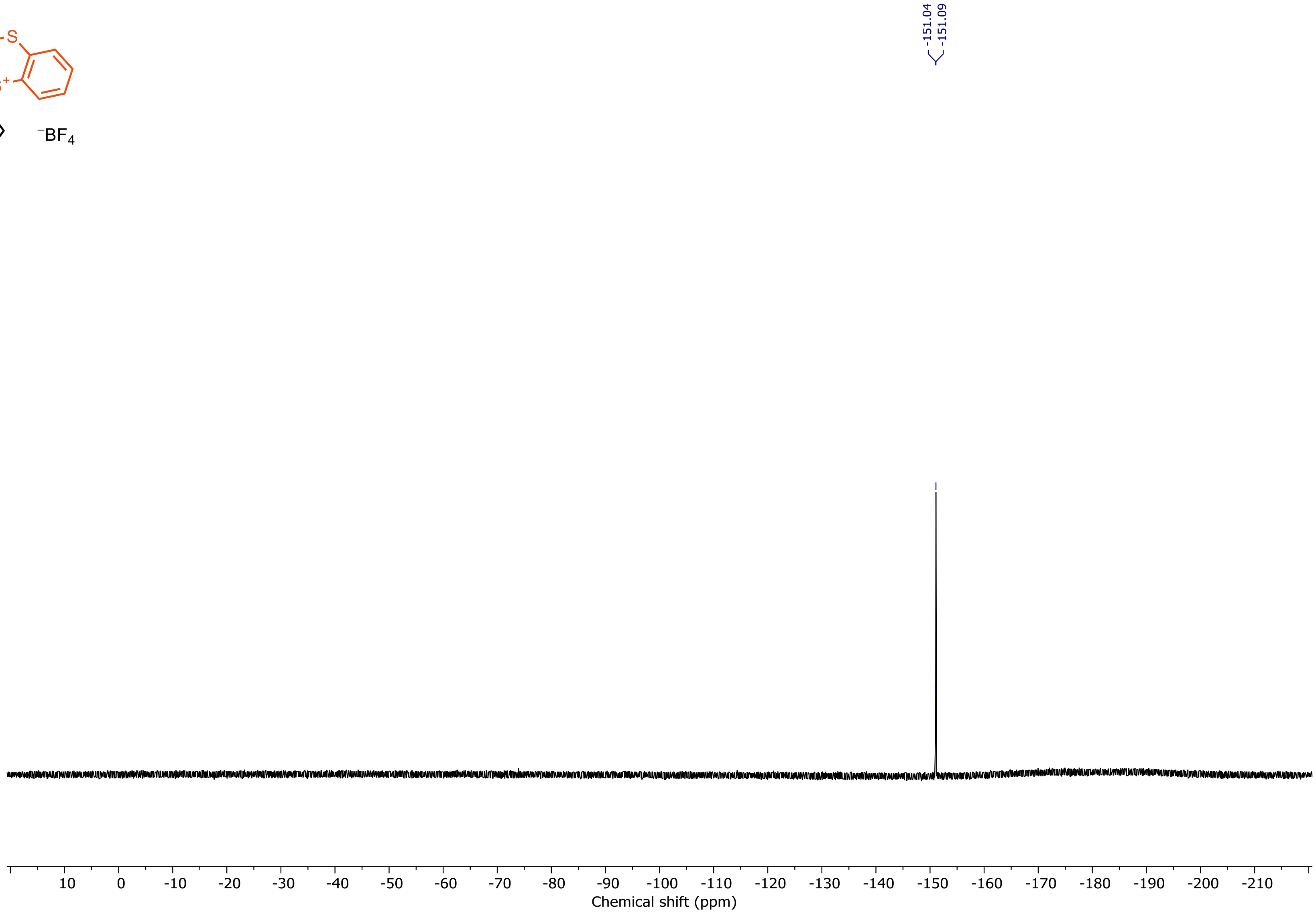

^{1}H NMR of 12-IIa

CDCl$_3$, 500 MHz, 23 ºC

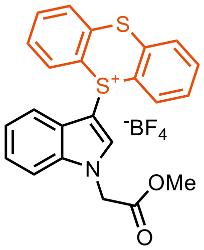

12-IIa

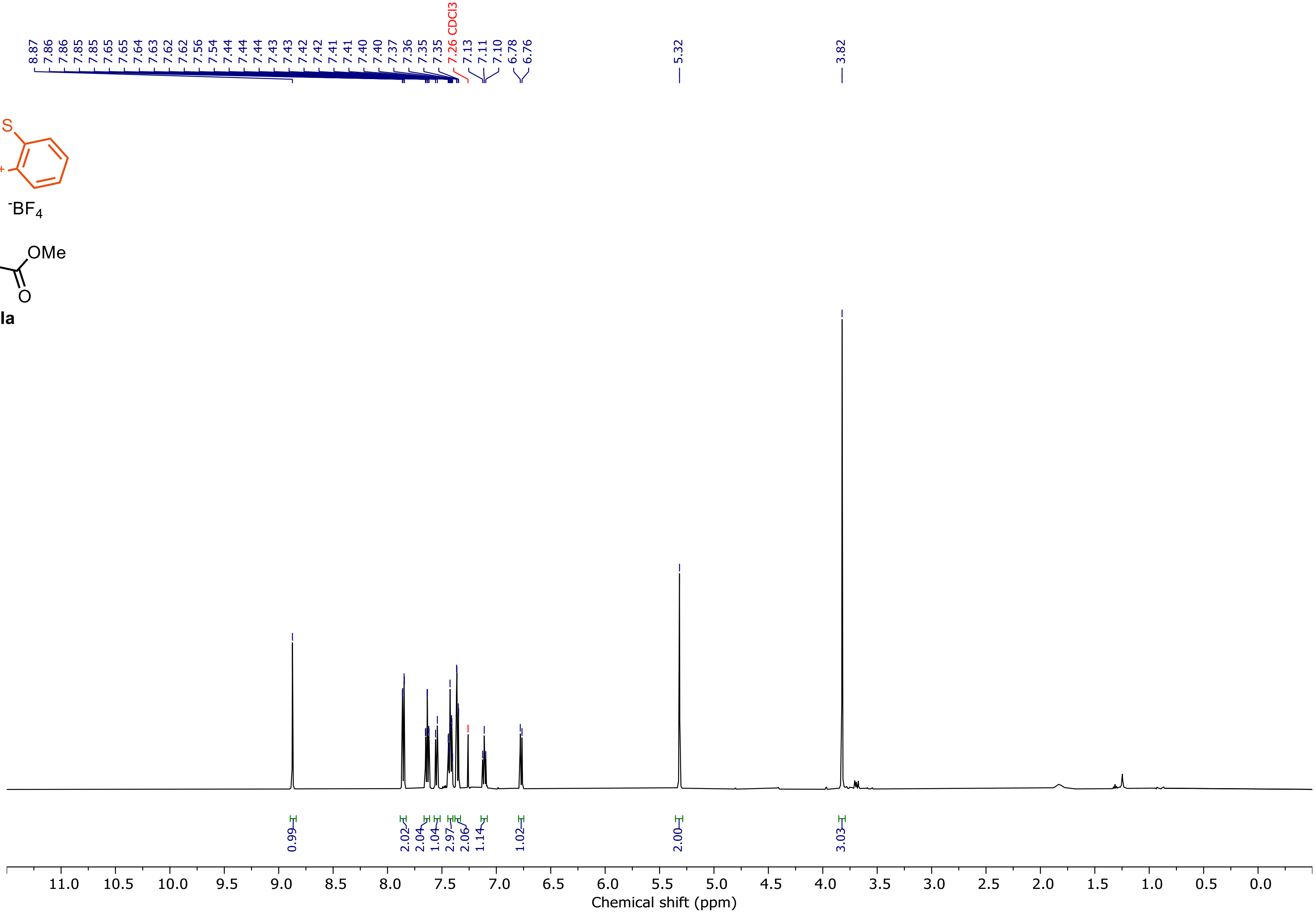

^{13}C NMR of 12-IIa

$CDCl_3$, 126 MHz, 23 ºC.

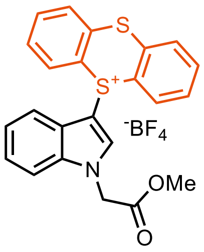
12-IIa

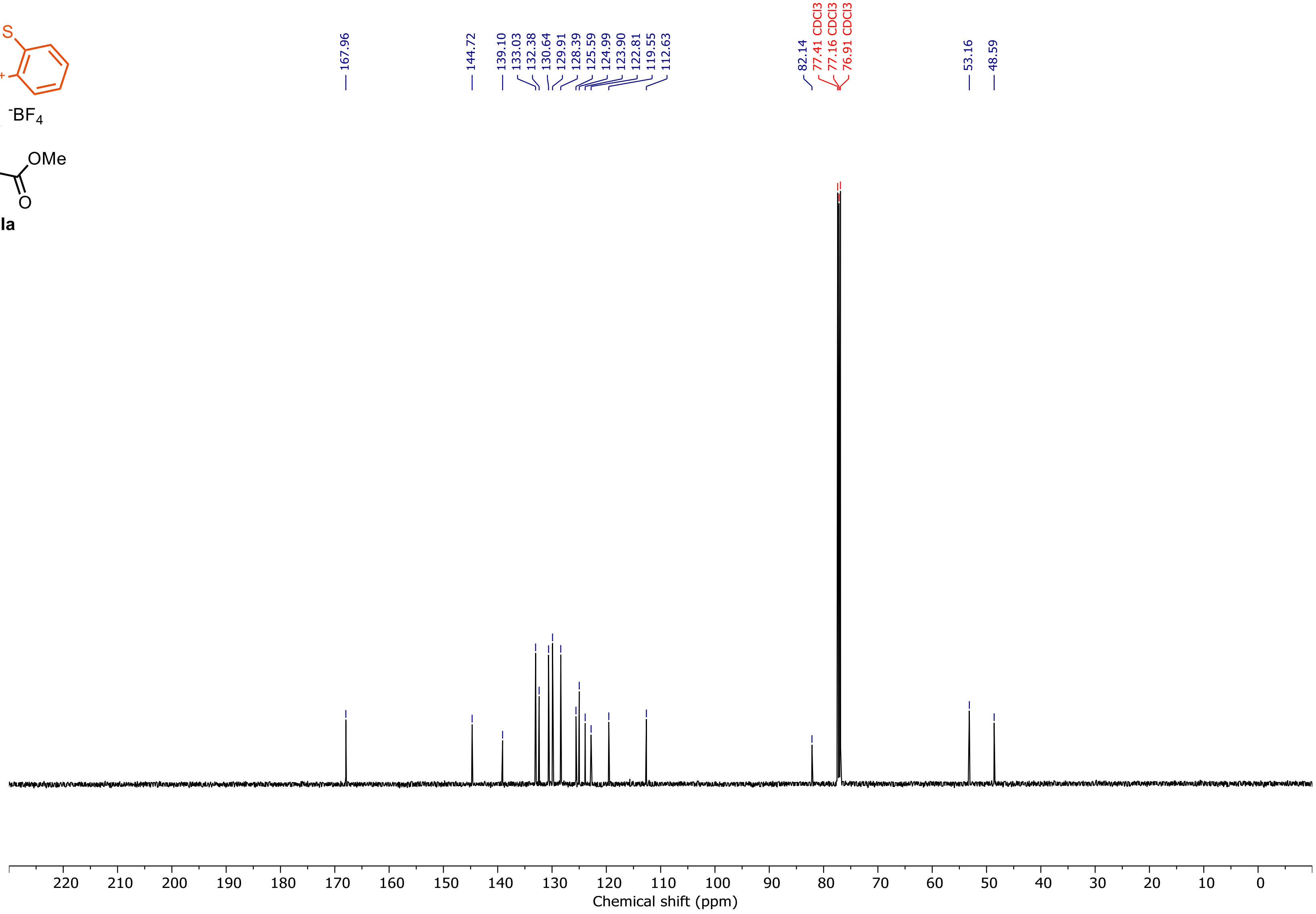

^{19}F NMR of 12-IIa

CDCl$_3$, 471 MHz, 23 ºC.

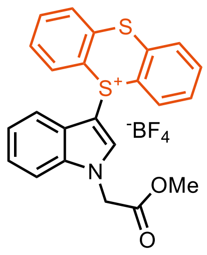

12-IIa

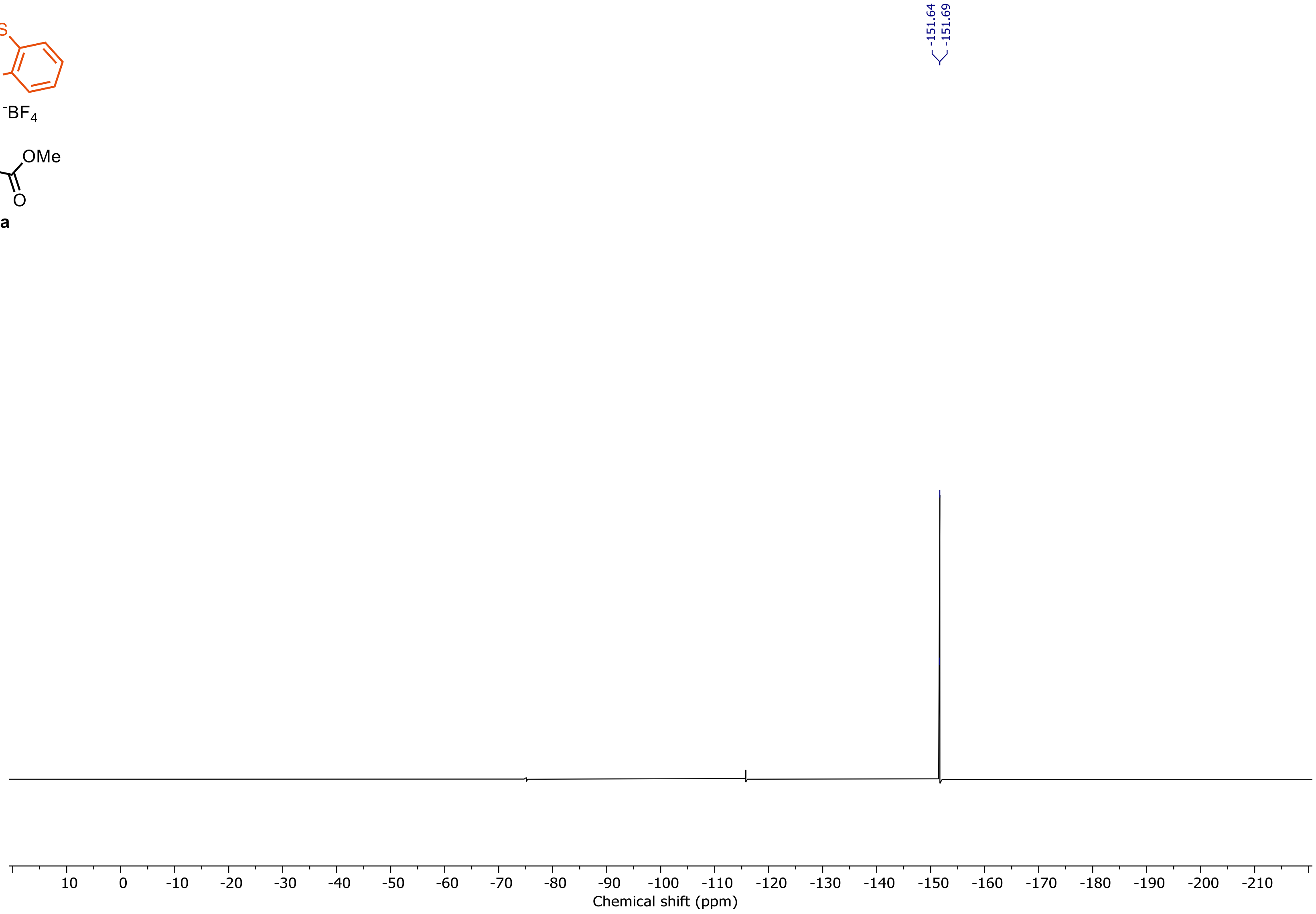

^{1}H NMR of 13a

CD_3CN, 600 MHz, 23 ºC

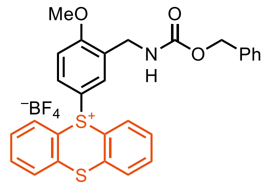

13a

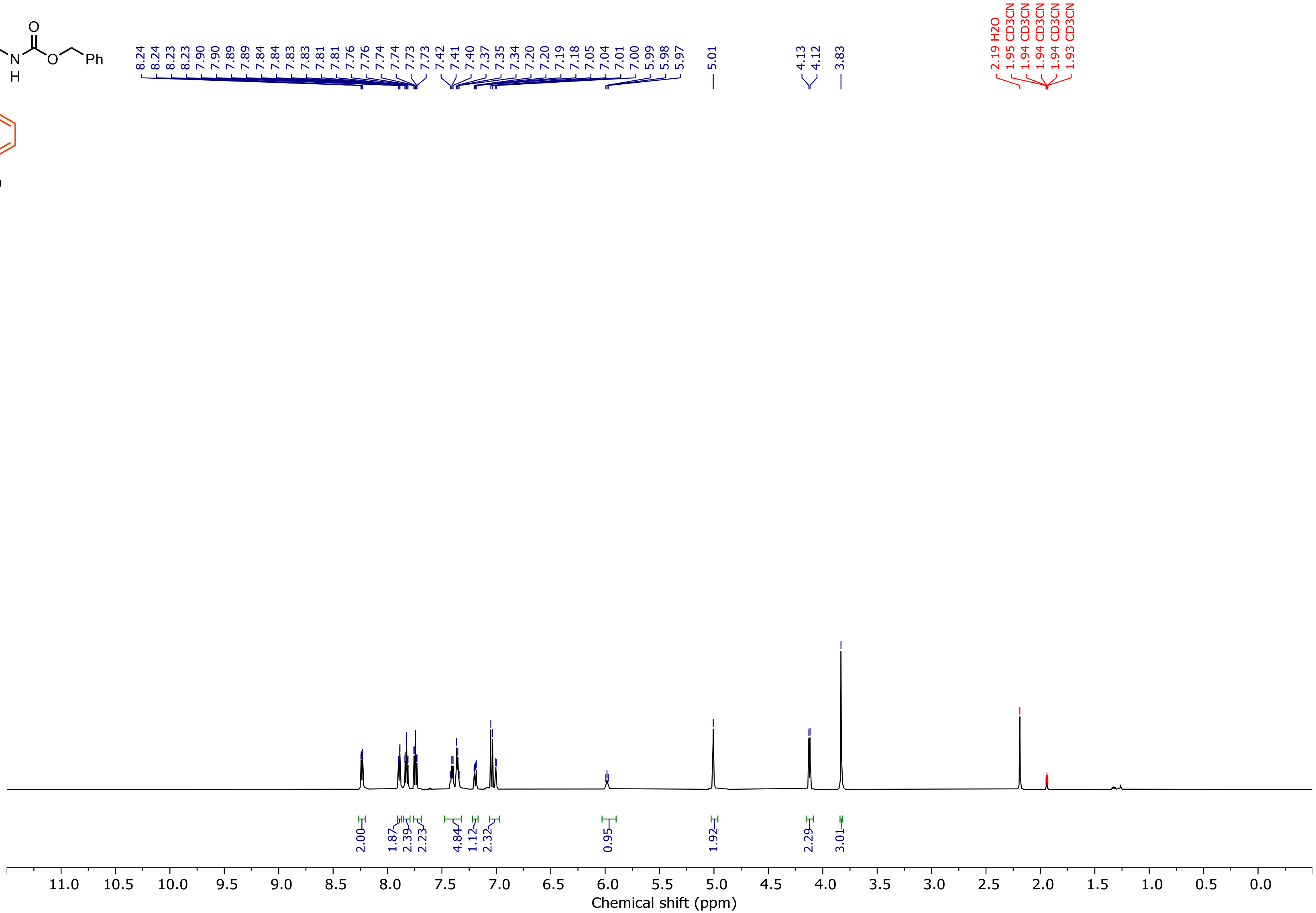

^{13}C NMR of 13a

CD_3CN, 151 MHz, 23 ºC

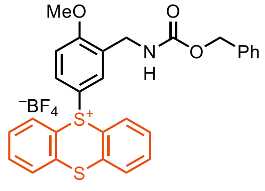

13a

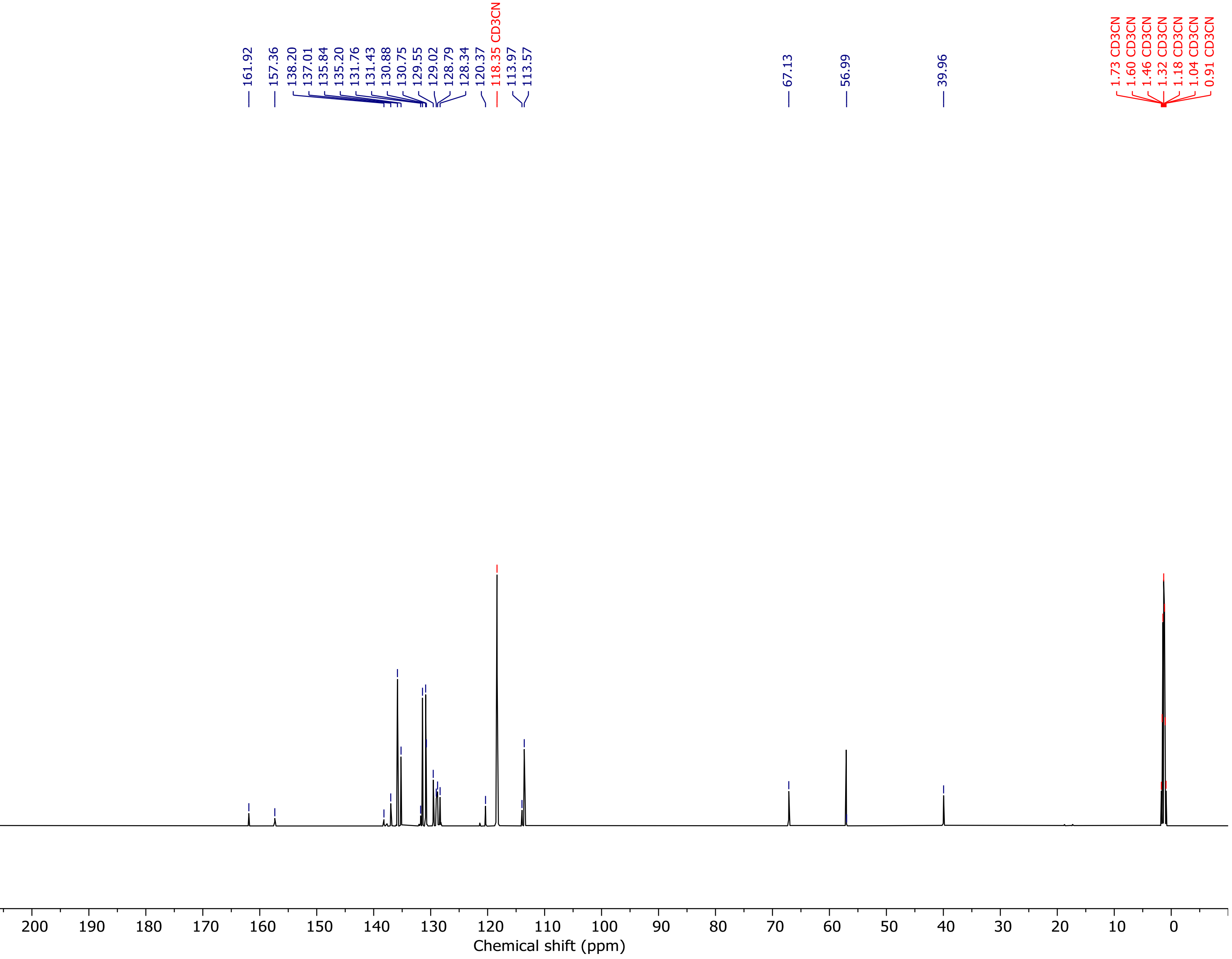

^{19}F NMR of 13a

CD_3CN, 576 MHz, 23 ºC

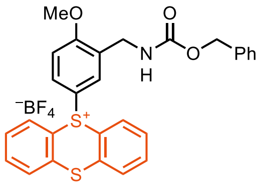

13a

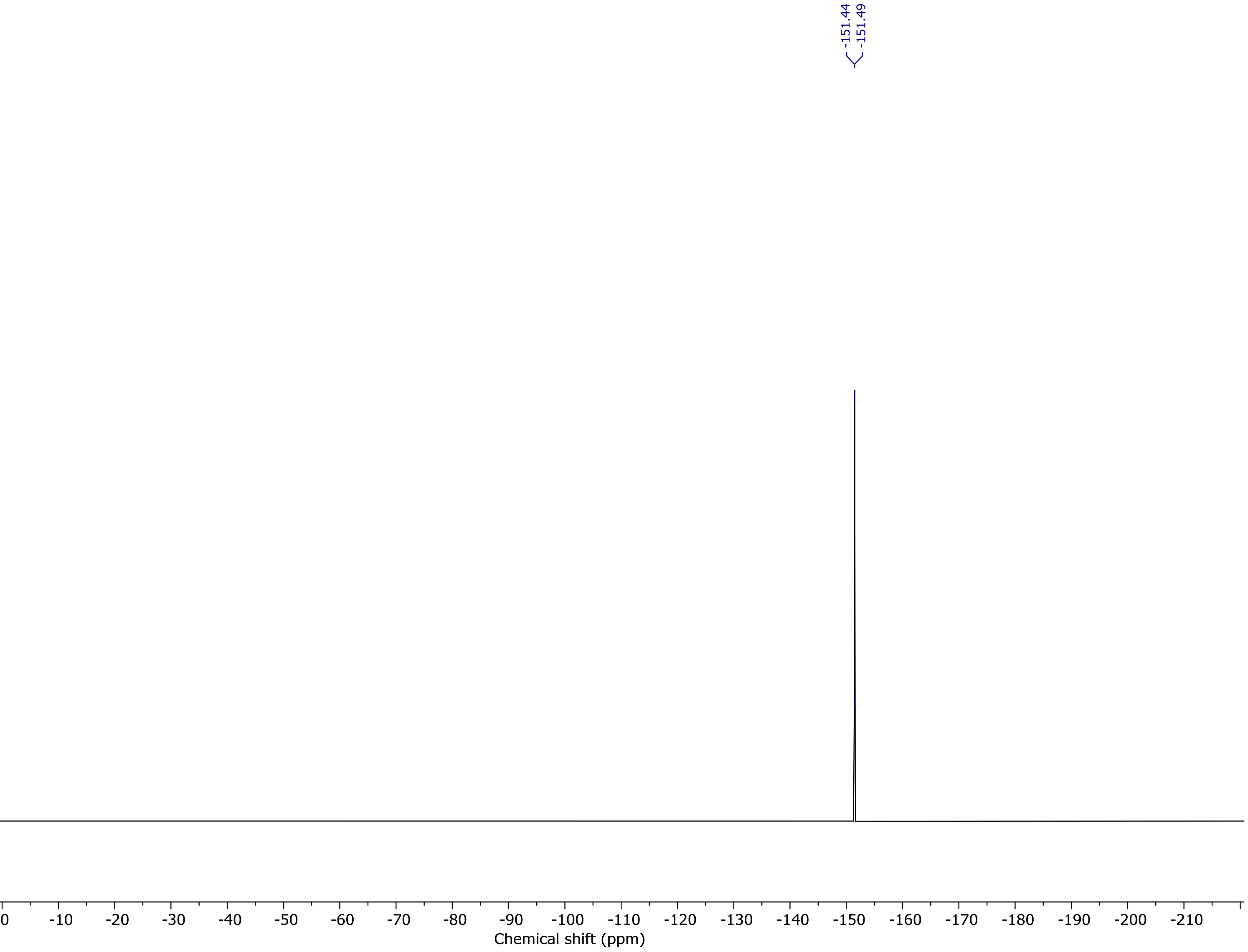

^{1}H NMR of 14a

CD_3CN, 500 MHz, 23 ºC.

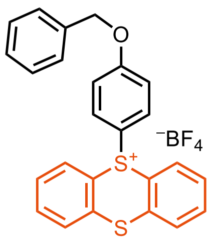

14a

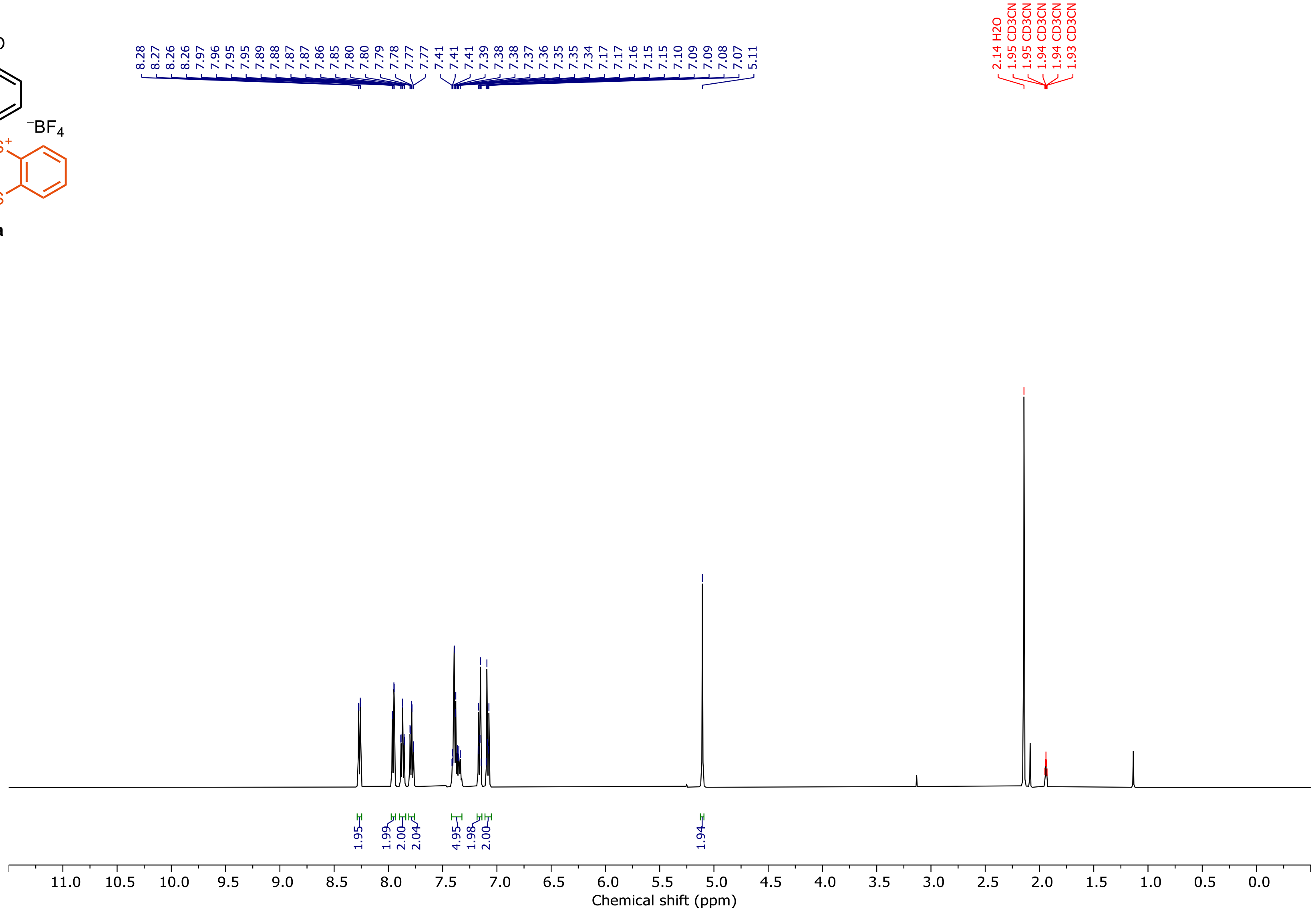

^{13}C NMR of 14a

CD_3CN, 126 MHz, 23 ºC.

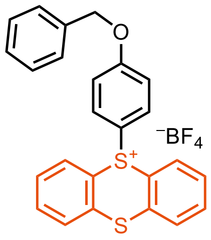

14a

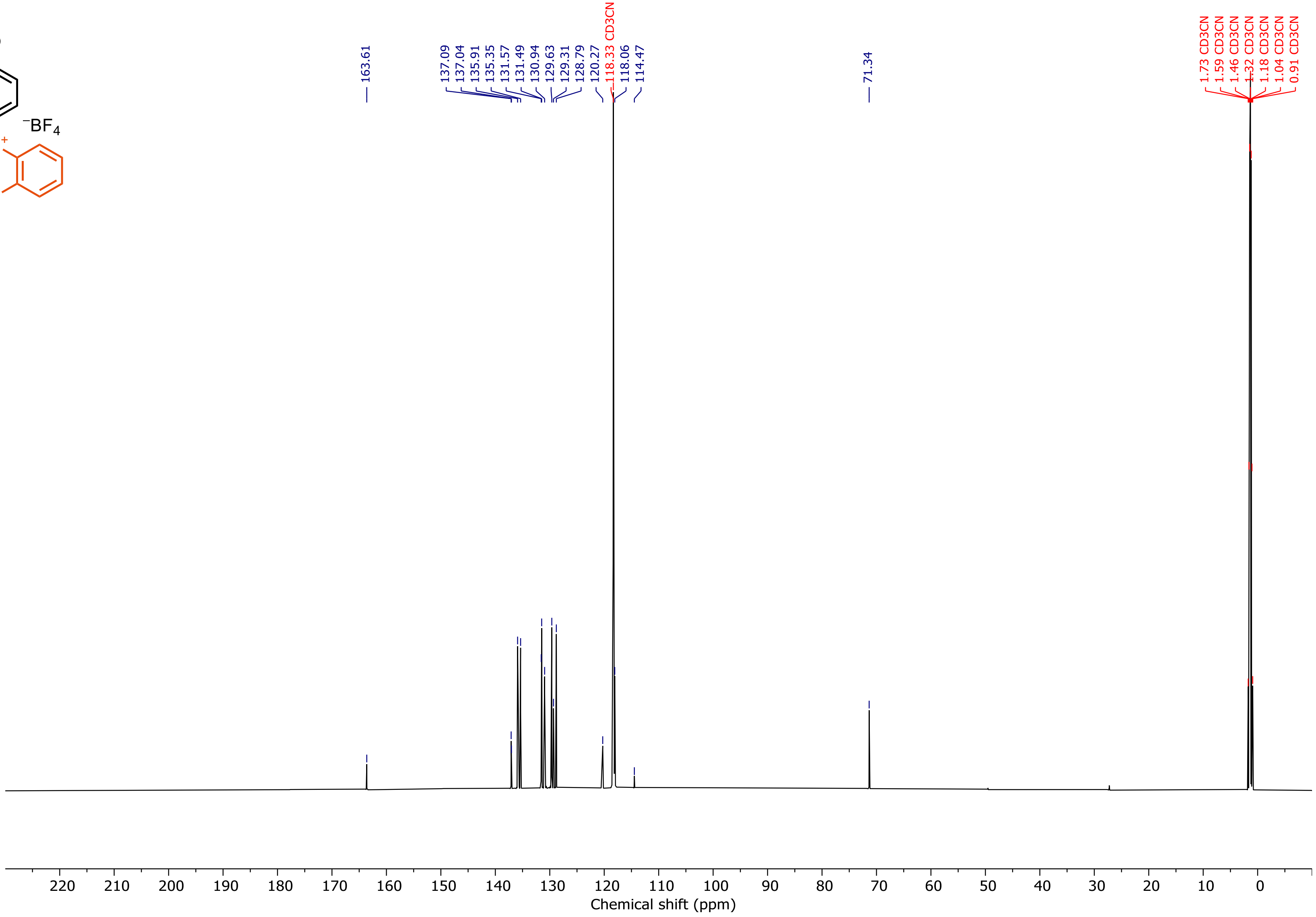

^{19}F NMR of 14a

CD_3CN, 471 MHz, 23 ºC.

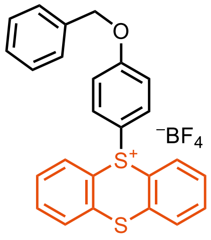

14a

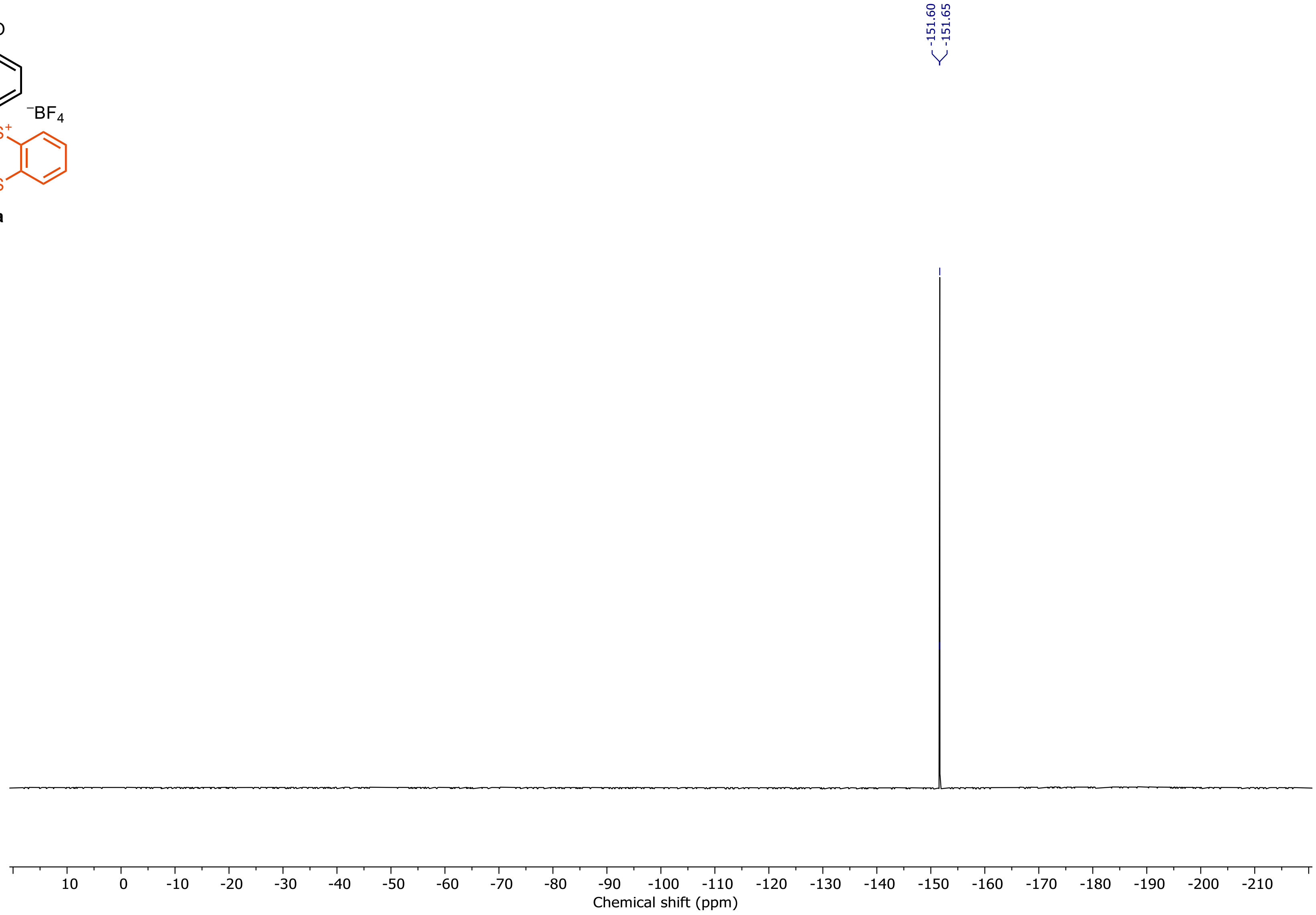

^{1}H NMR of 15a

CD_3CN, 500 MHz, 23 ºC.

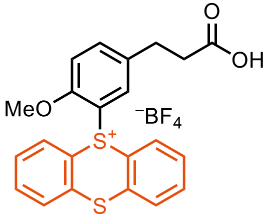

15a

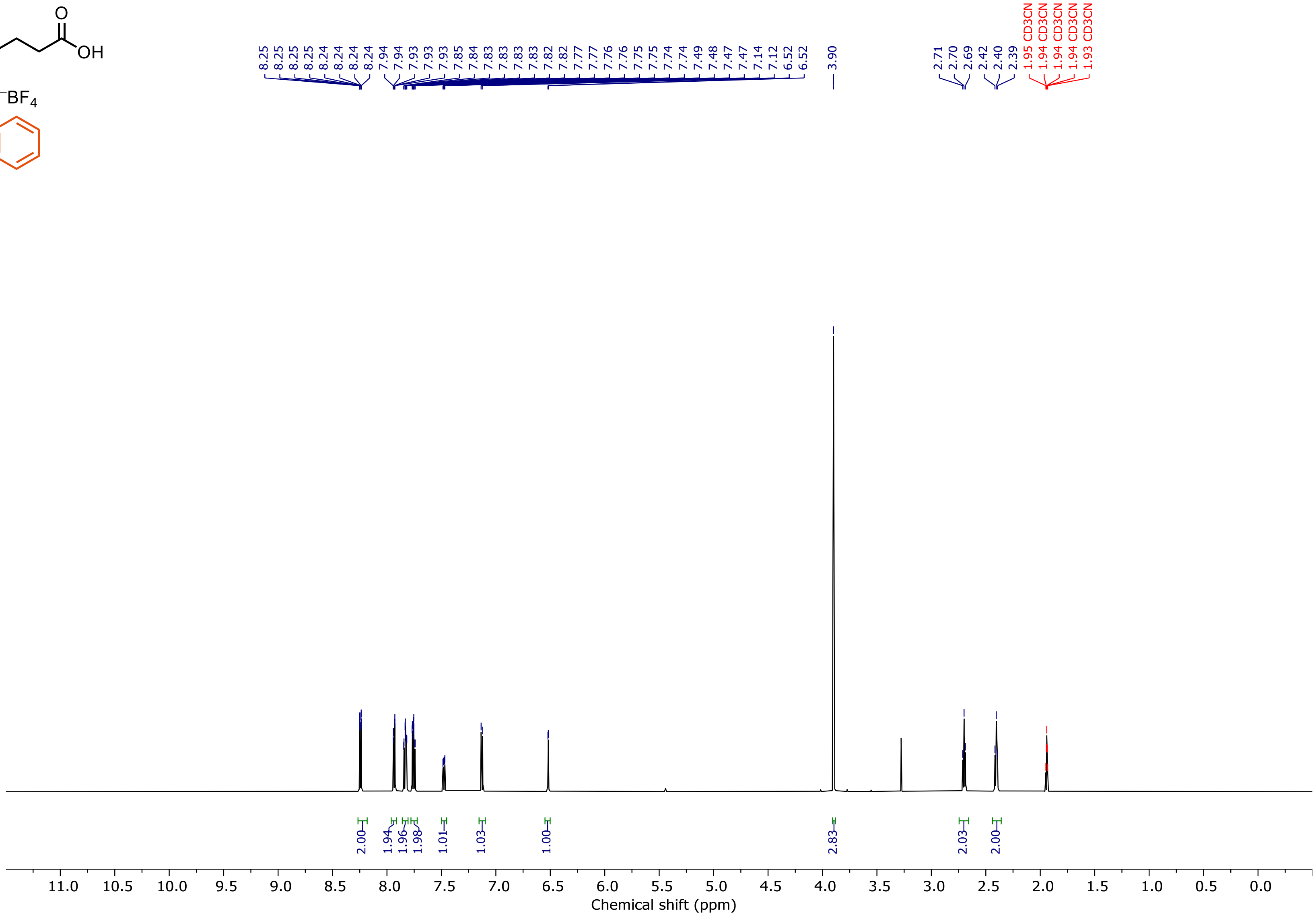

^{13}C NMR of 15a

CD_3CN, 126 MHz, 23 ºC.

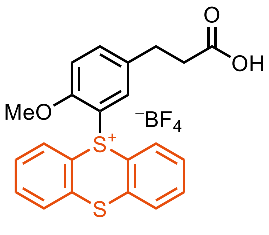

15a

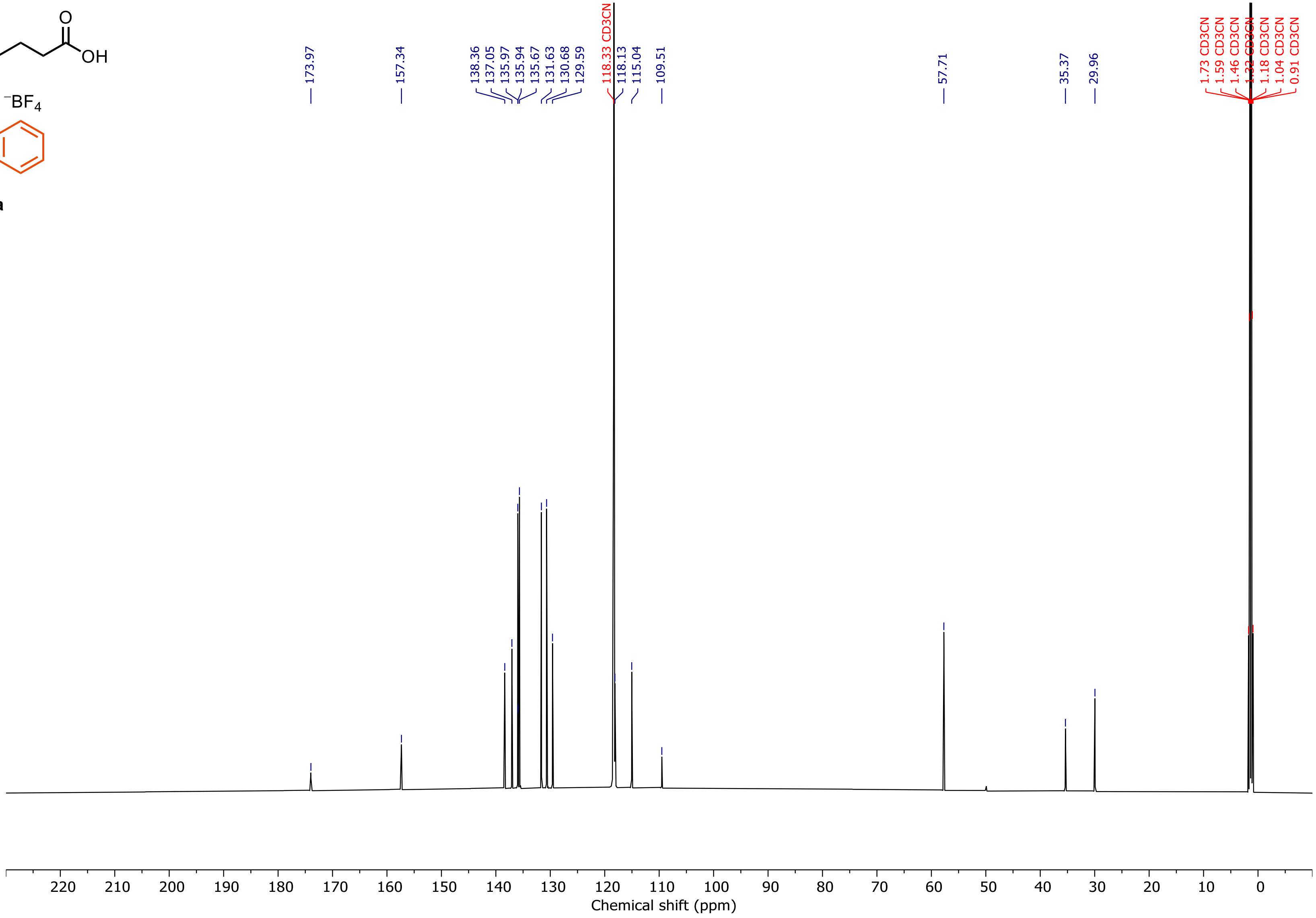

^{19}F NMR of 15a

CD_3CN, 471 MHz, 23 ºC.

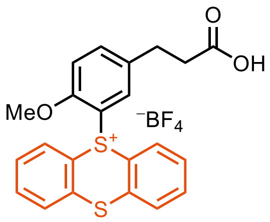

15a

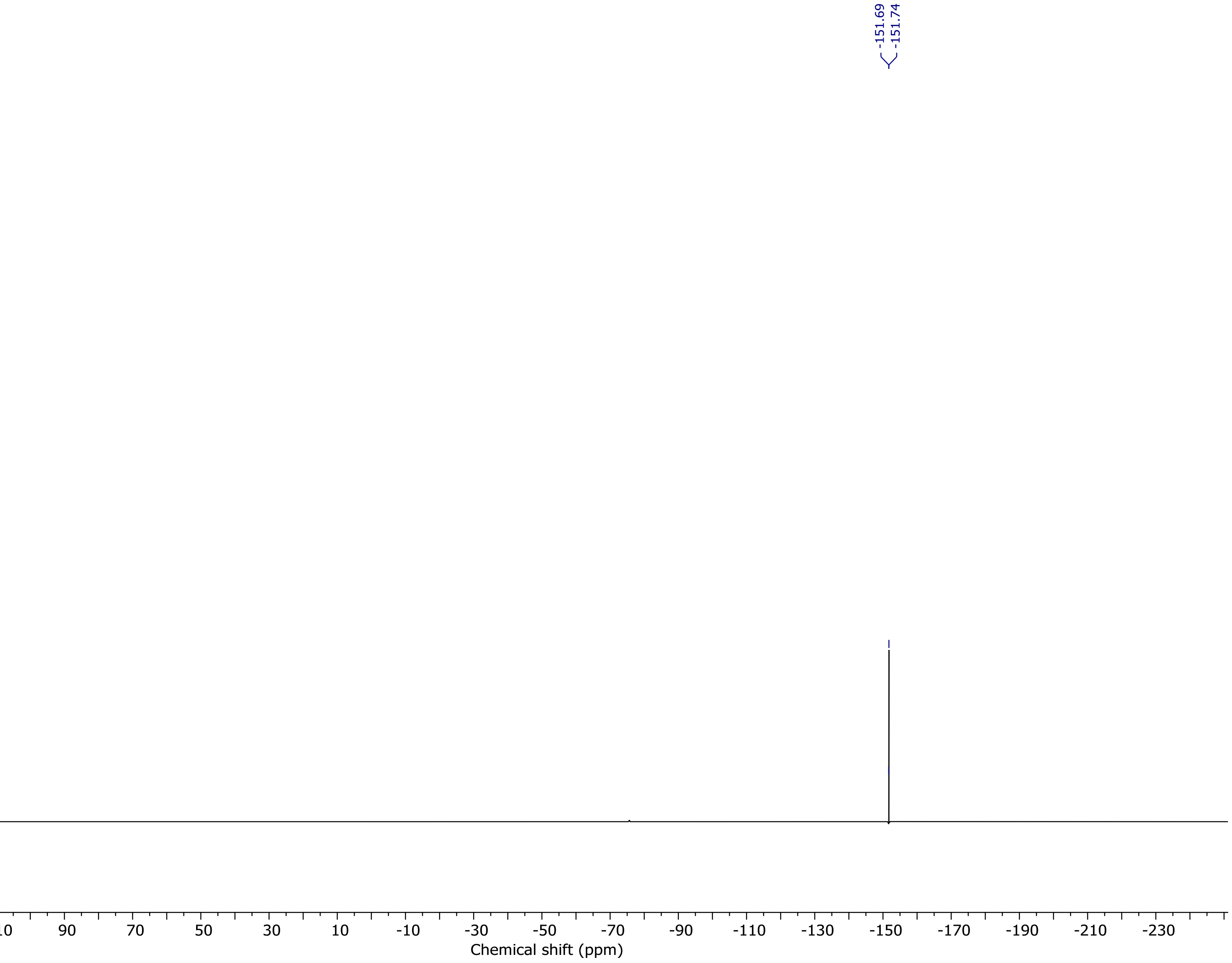

^{1}H NMR of 16a

CD_3CN, 500 MHz, 23 ºC.

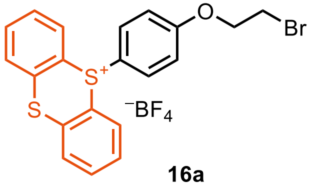

16a

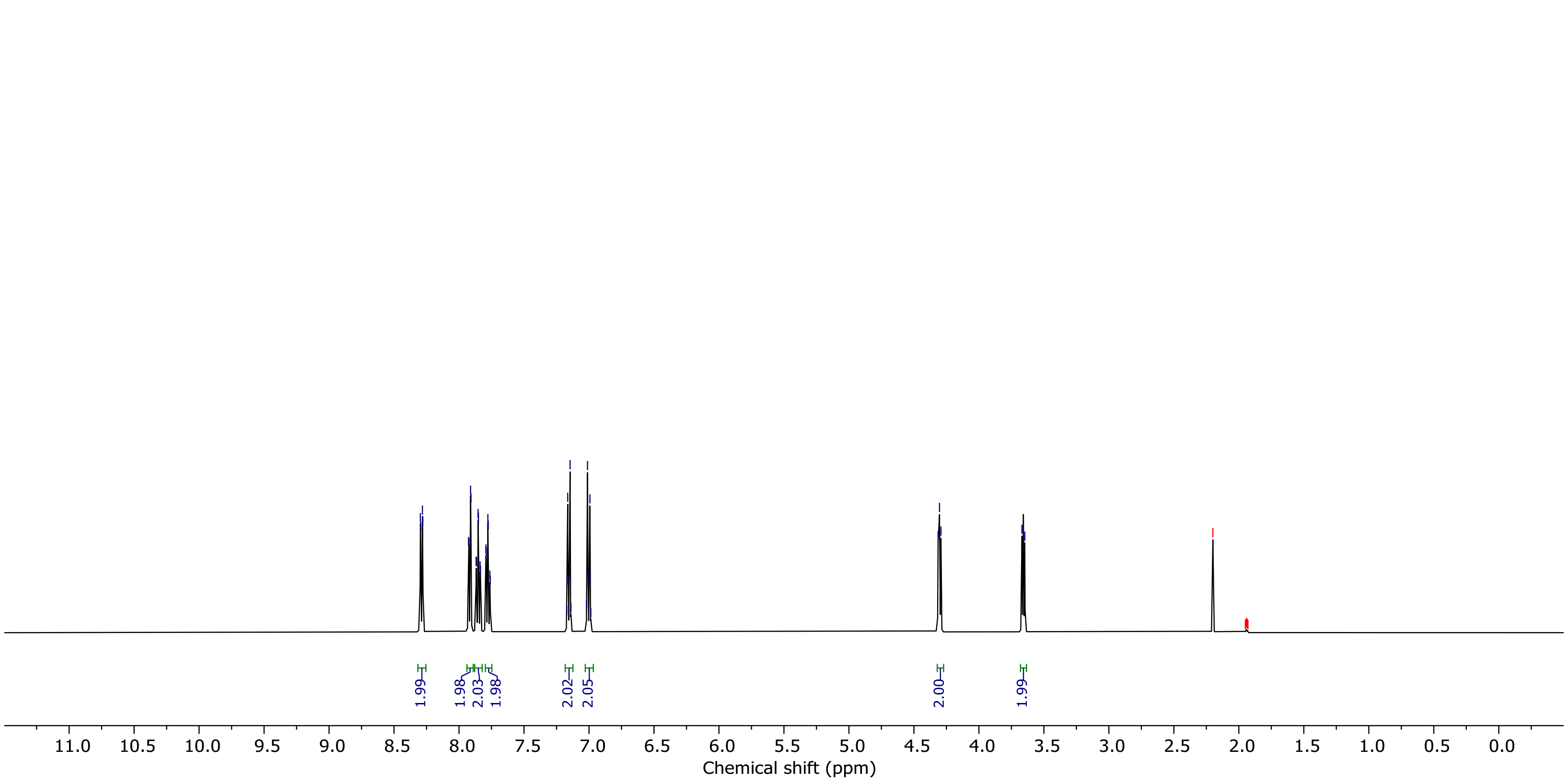

^{13}C NMR of 16a

CD_3CN, 126 MHz, 23 ºC.

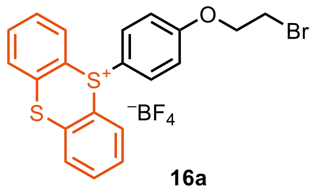

16a

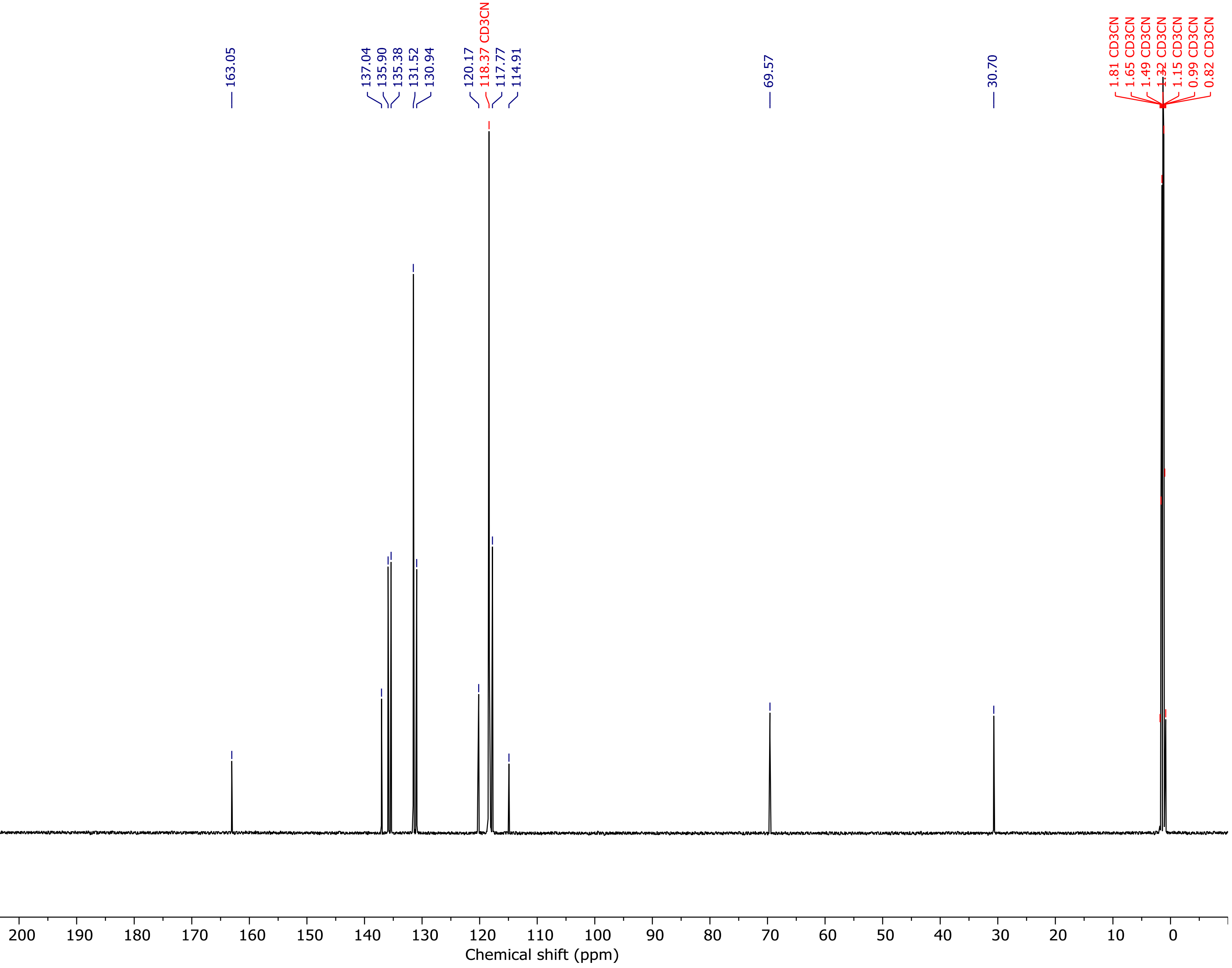

^{19}F NMR of 16a

CD_3CN, 471 MHz, 23 ºC.

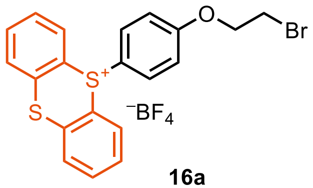

16a

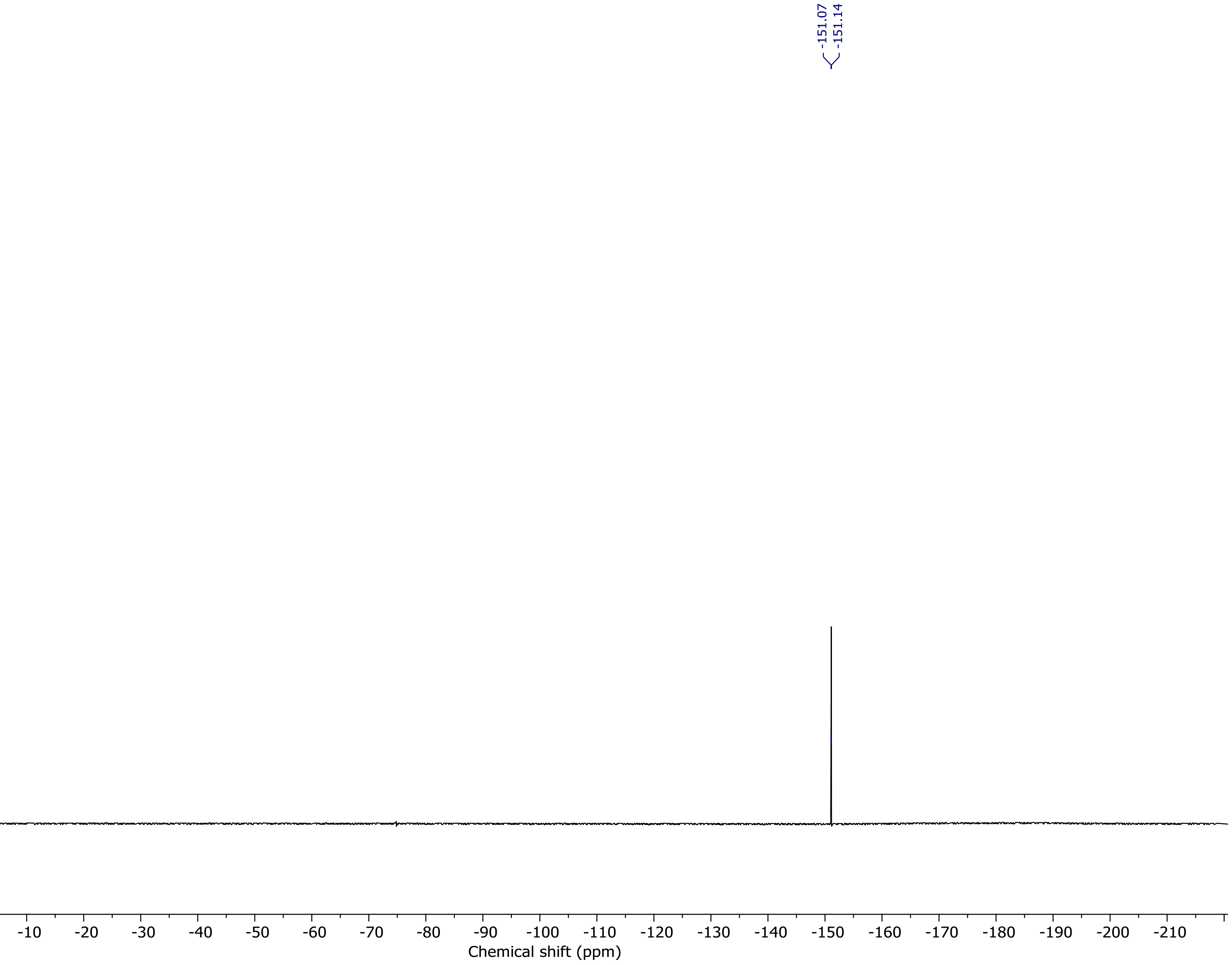

^{1}H NMR of 17a

$CDCl_3$, 400 MHz, 23 ºC.

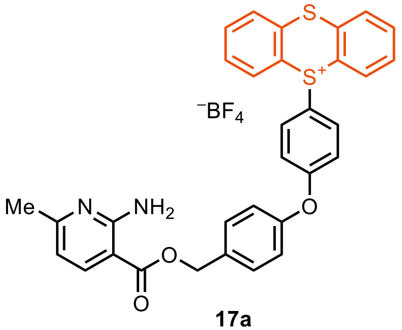

17a

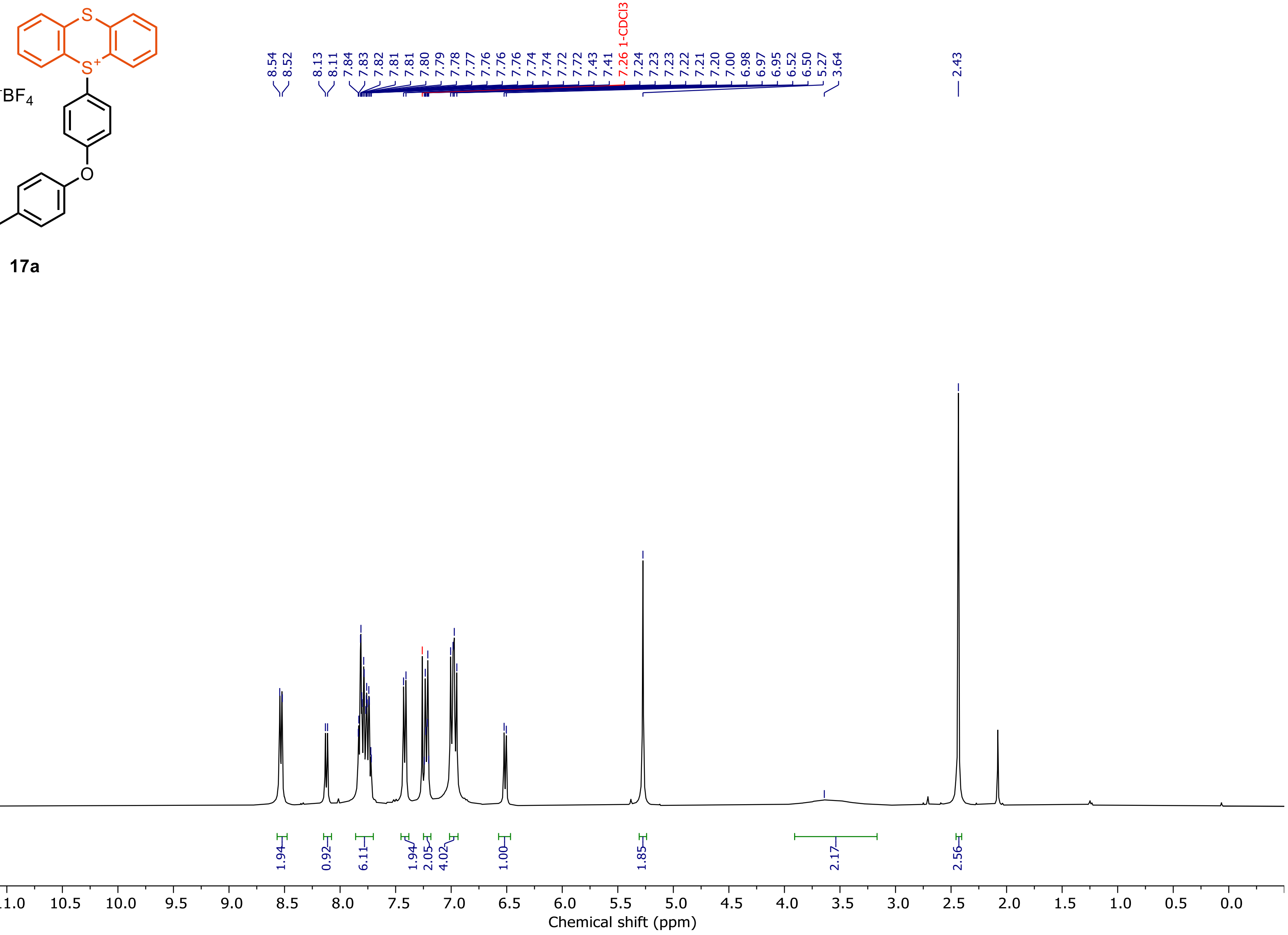

^{13}C NMR of 17a

$CDCl_3$, 101 MHz, 23 ºC.

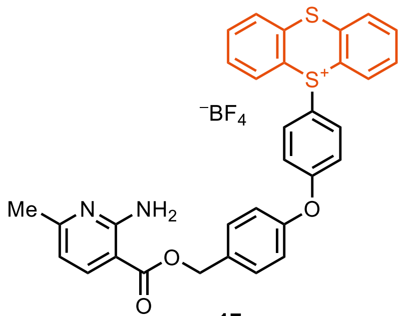

17a

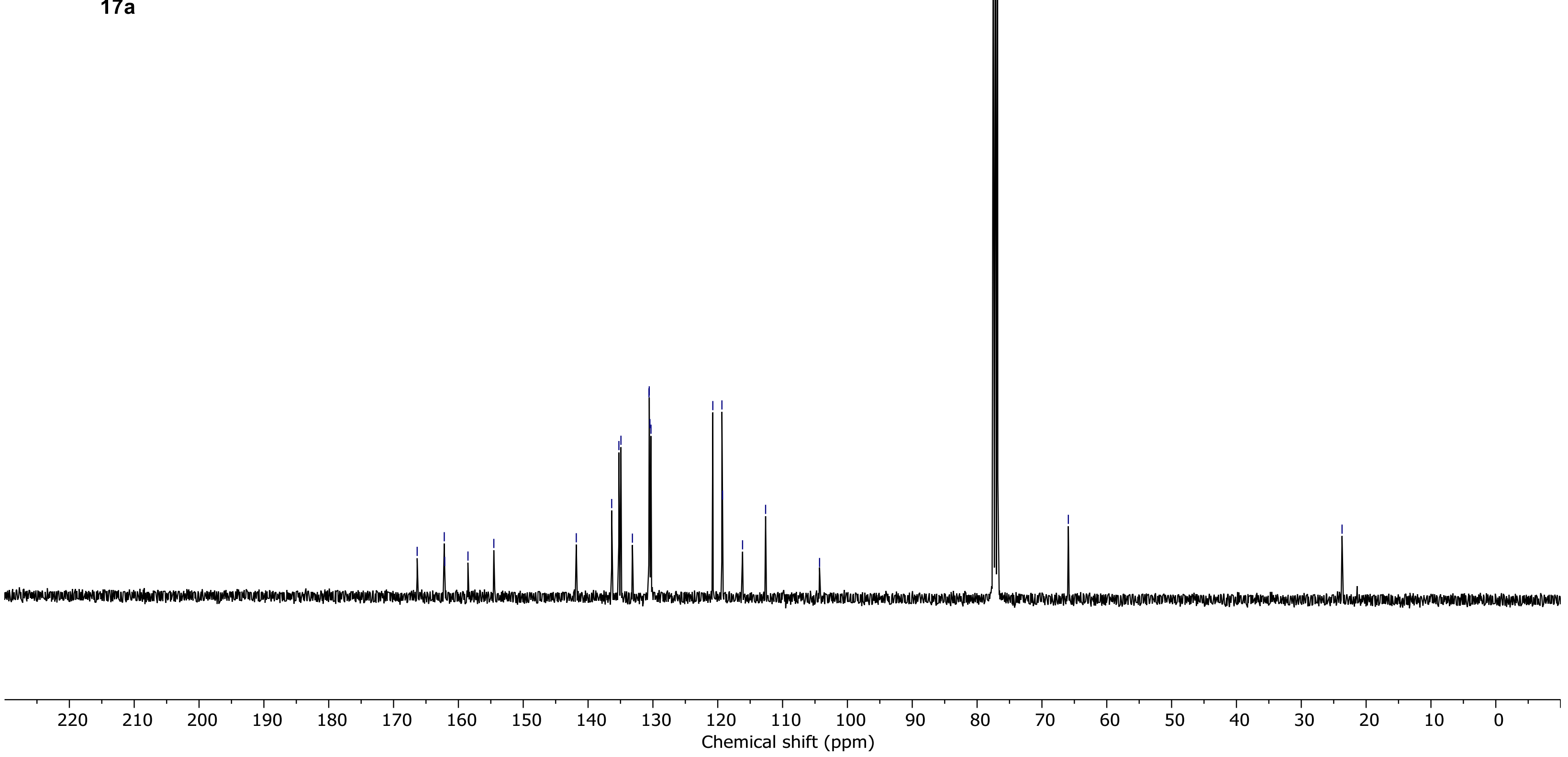

^{19}F NMR of 17a

$CDCl_3$, 377 MHz, 23 ºC.

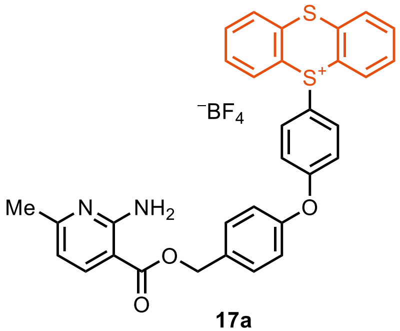

17a

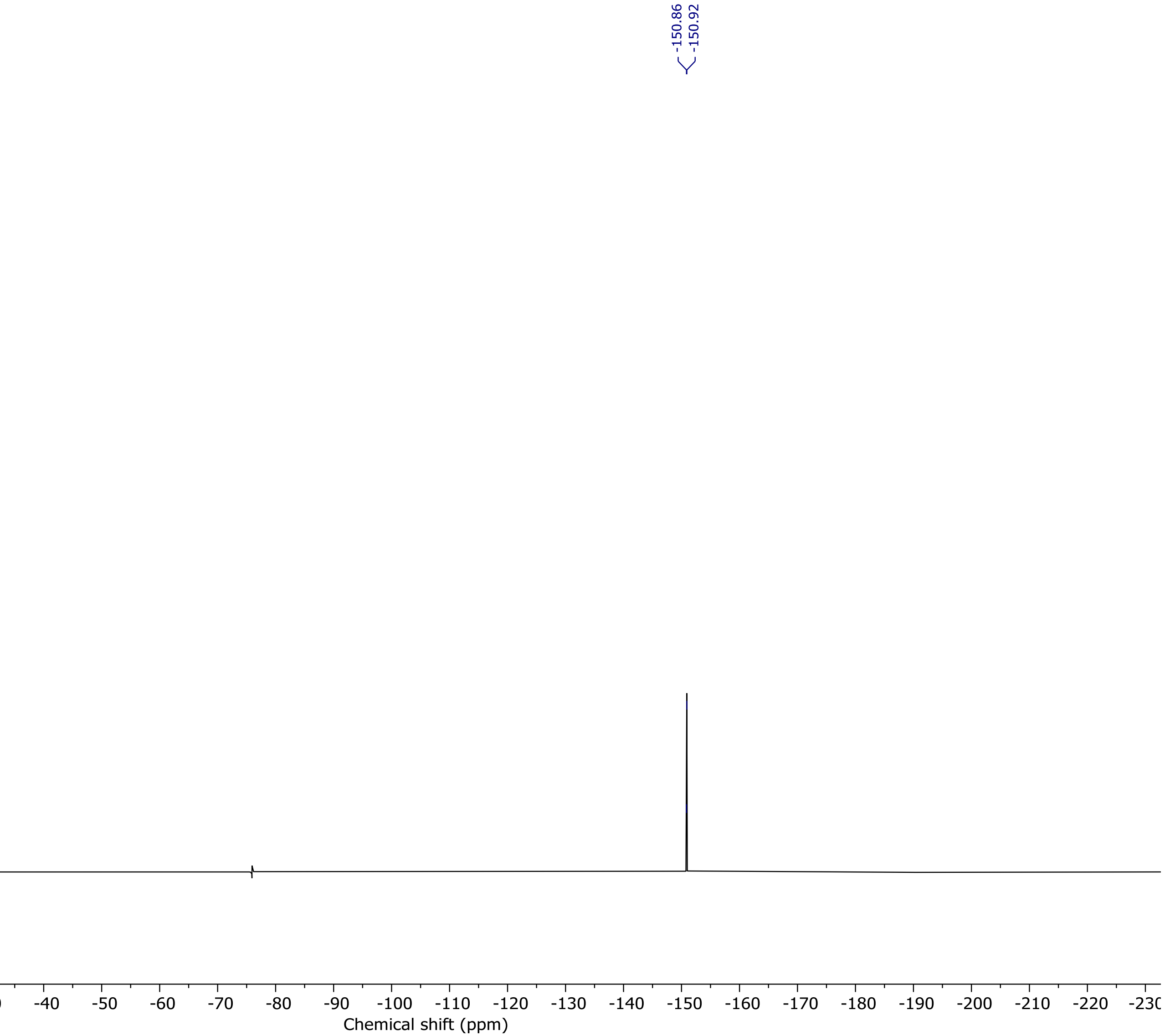

^{1}H NMR of 18a

$CDCl_3$, 500 MHz, 23 ºC.

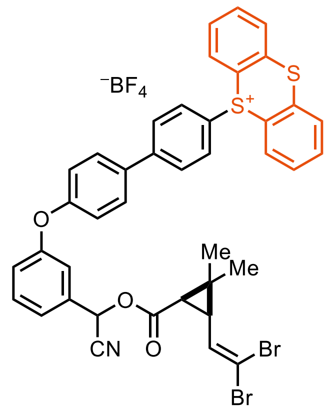

18a

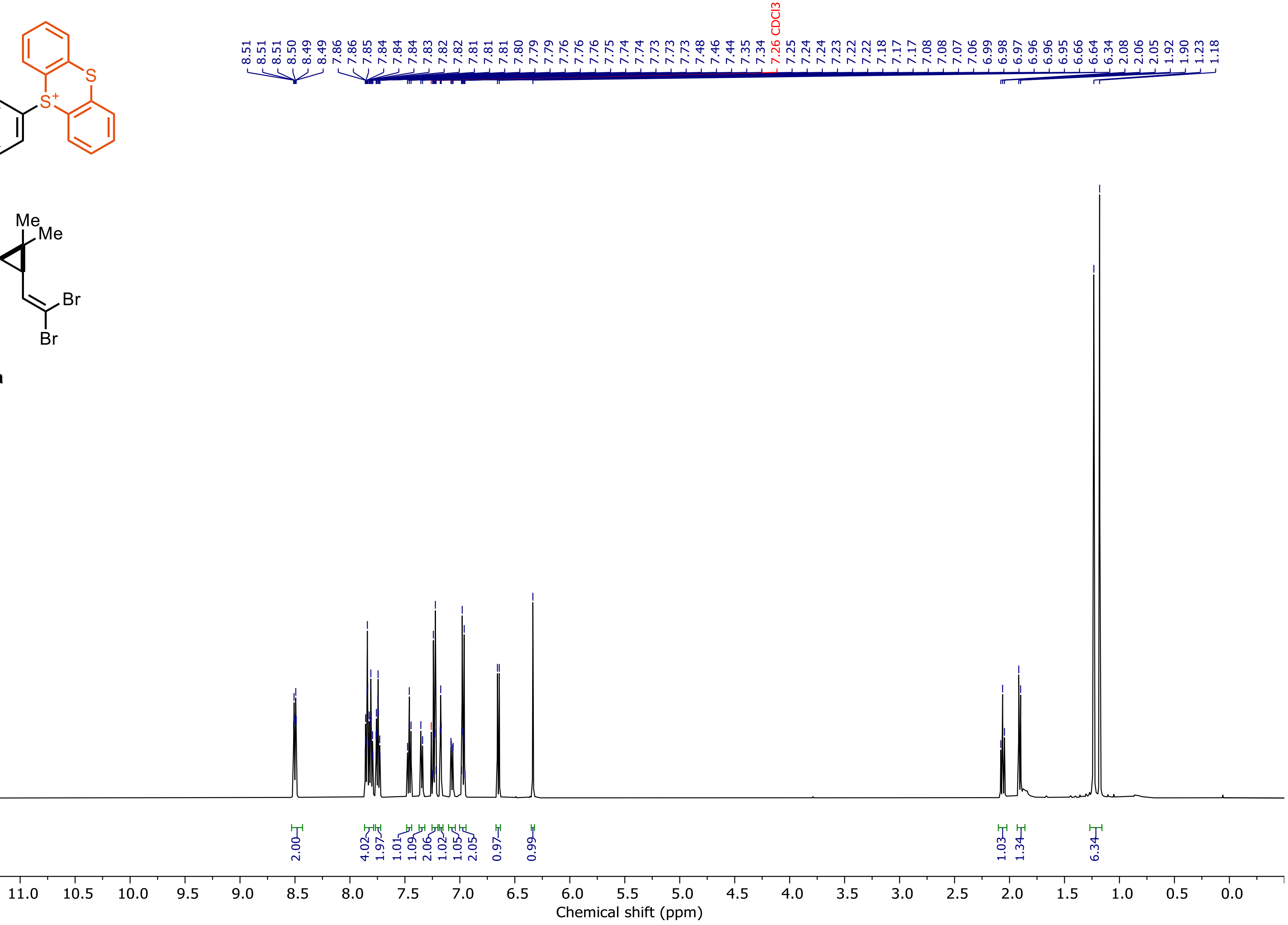

^{13}C NMR of 18a

$CDCl_3$, 126 MHz, 23 ºC.

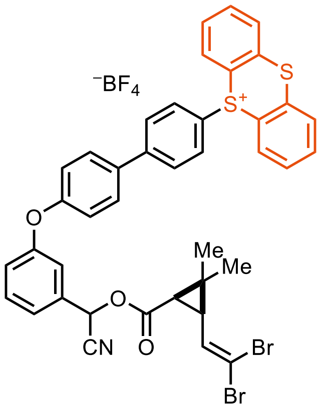

18a

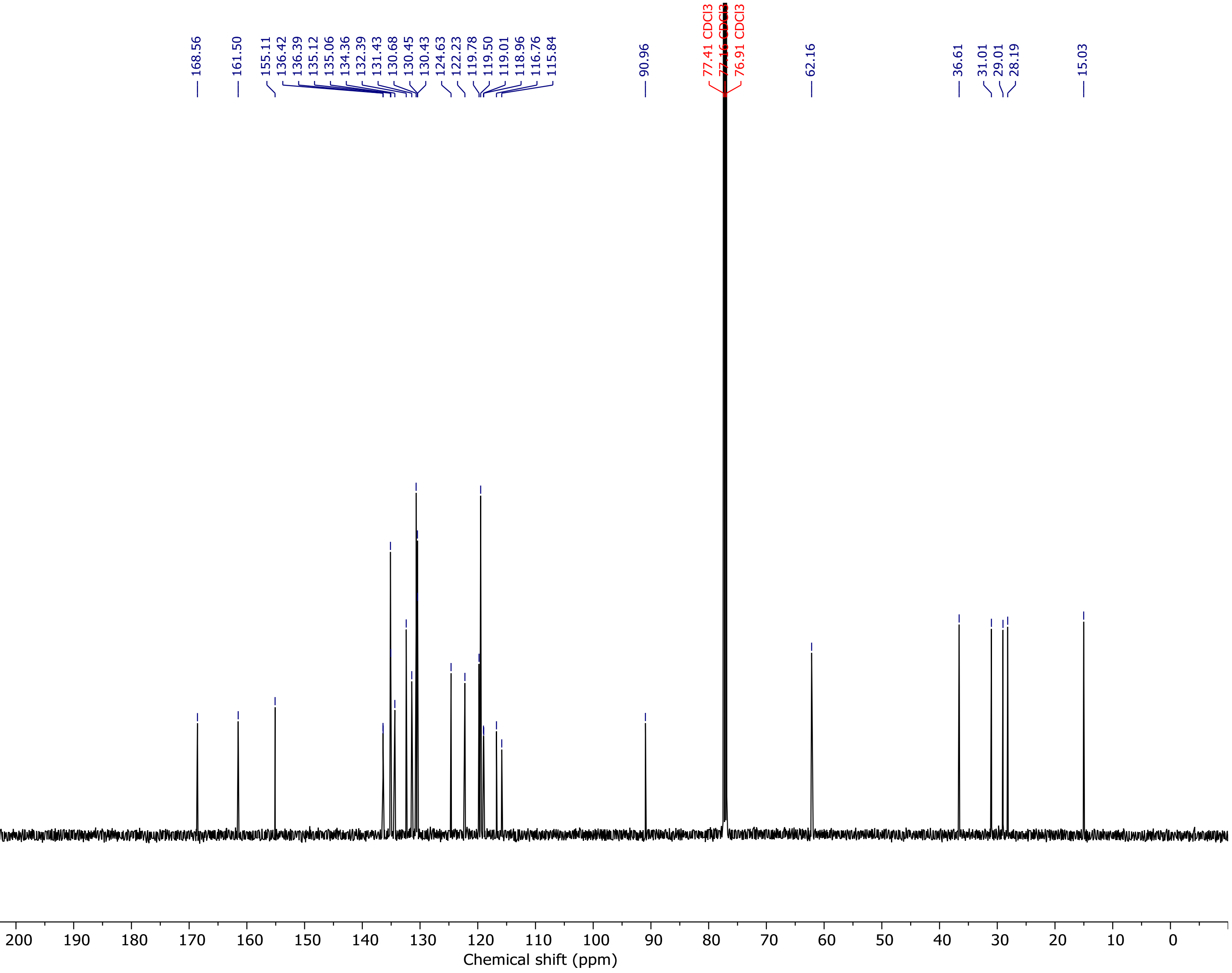

^{19}F NMR of 18a

$CDCl_3$, 471 MHz, 23 ºC.

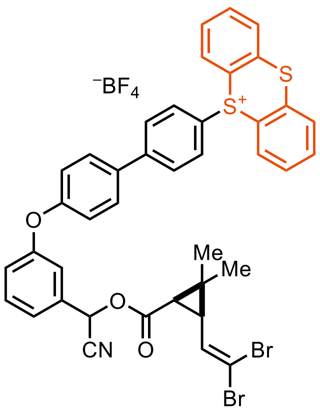

18a

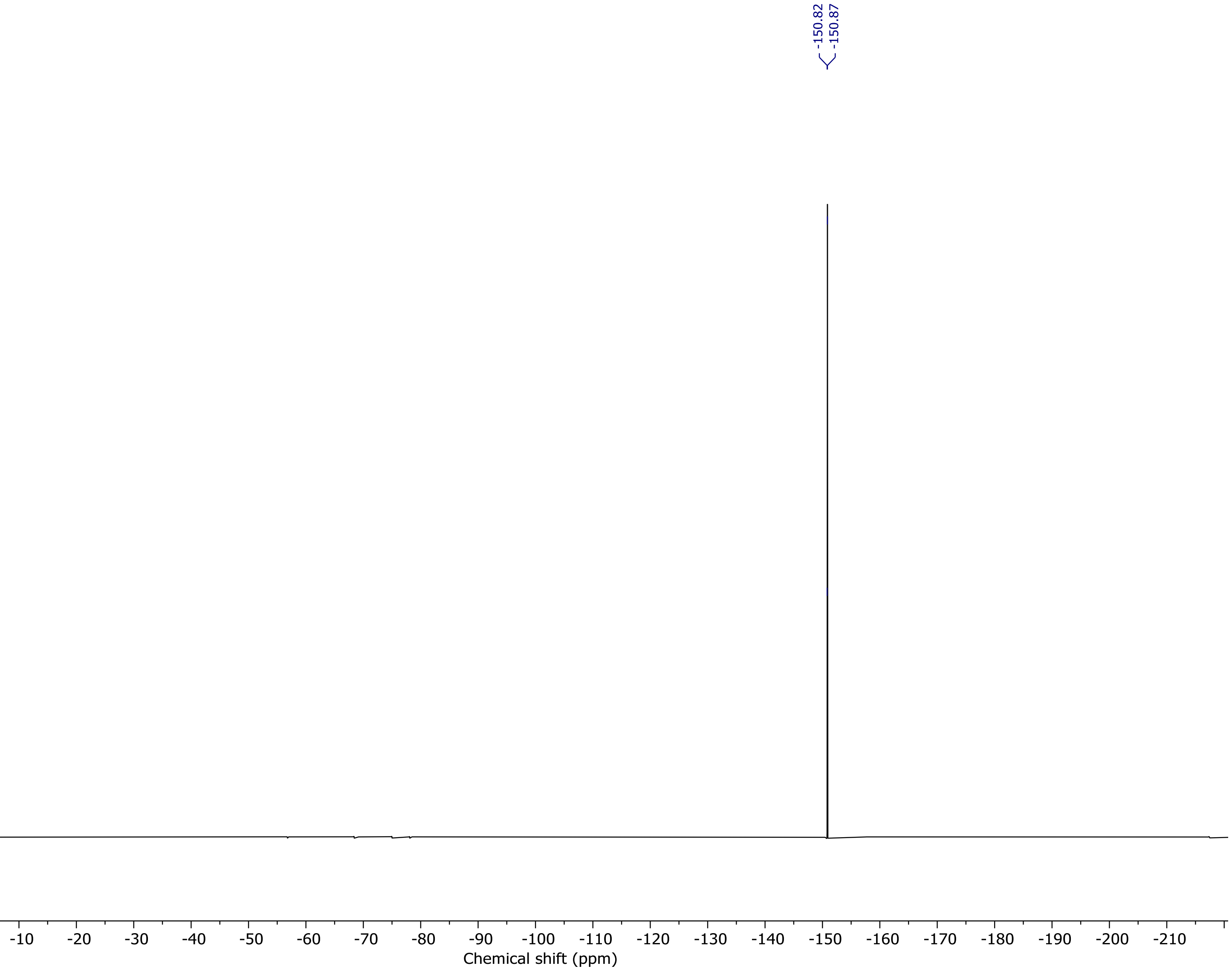

^{1}H NMR of 19a

CD_3CN, 500 MHz, 23 ºC.

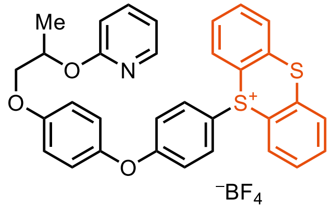

19a

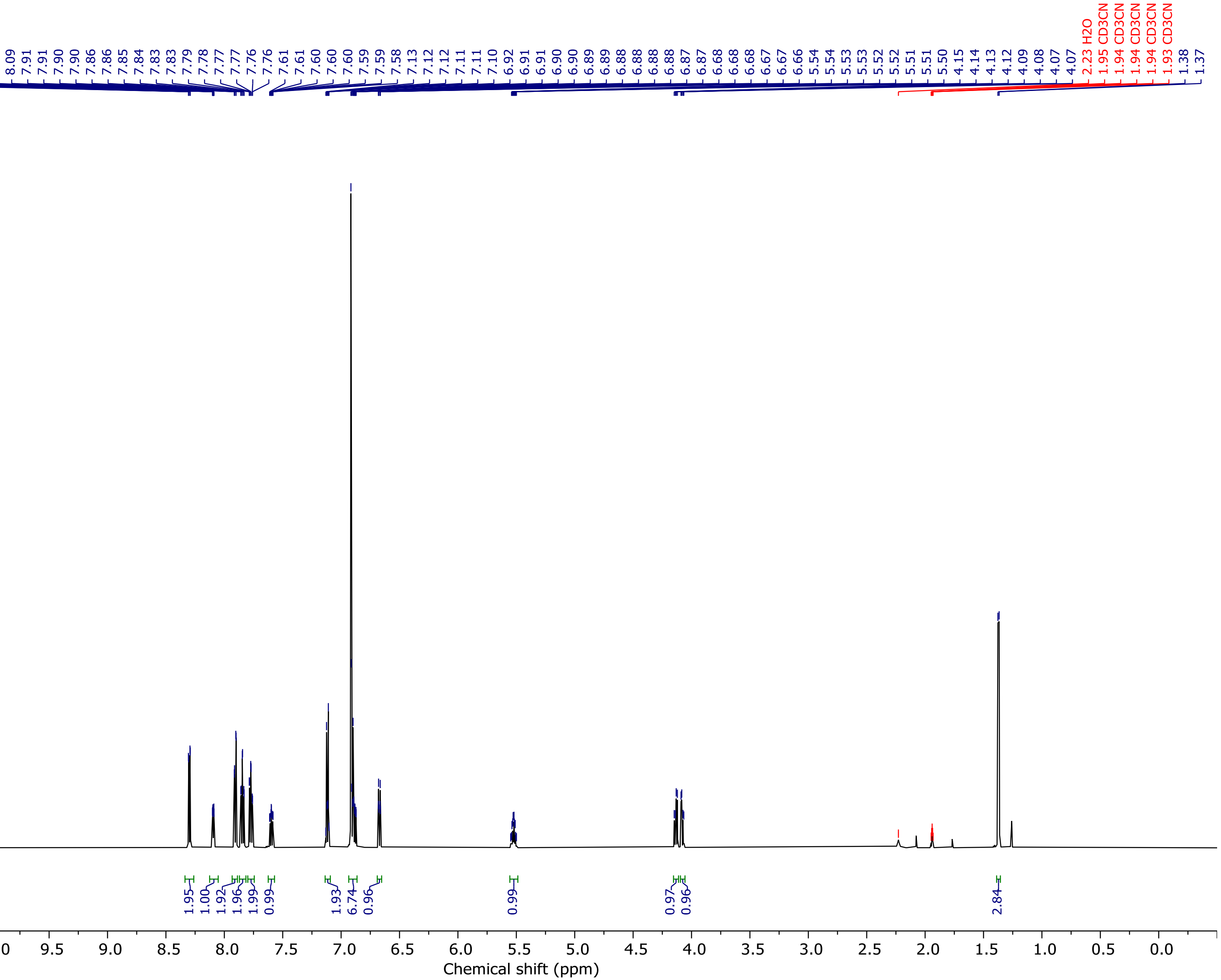

^{13}C NMR of 19a

CD_3CN, 126 MHz, 23 ºC.

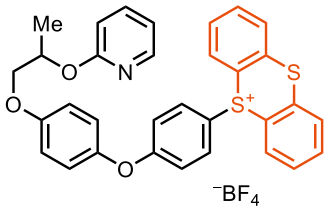

19a

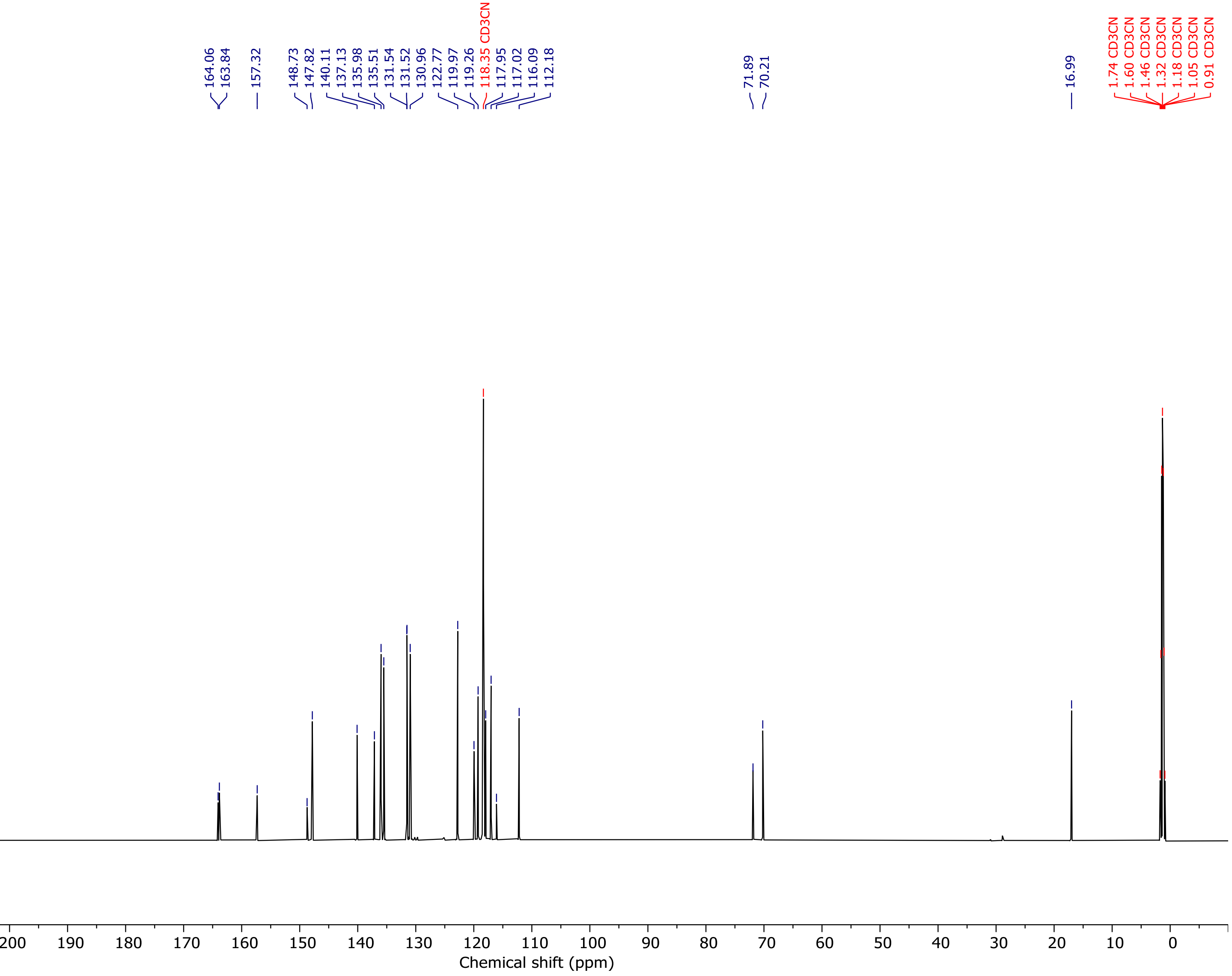

^{19}F NMR of 19a

CD_3CN, 471 MHz, 23 ºC.

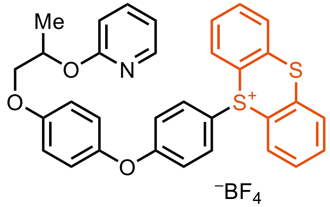

19a

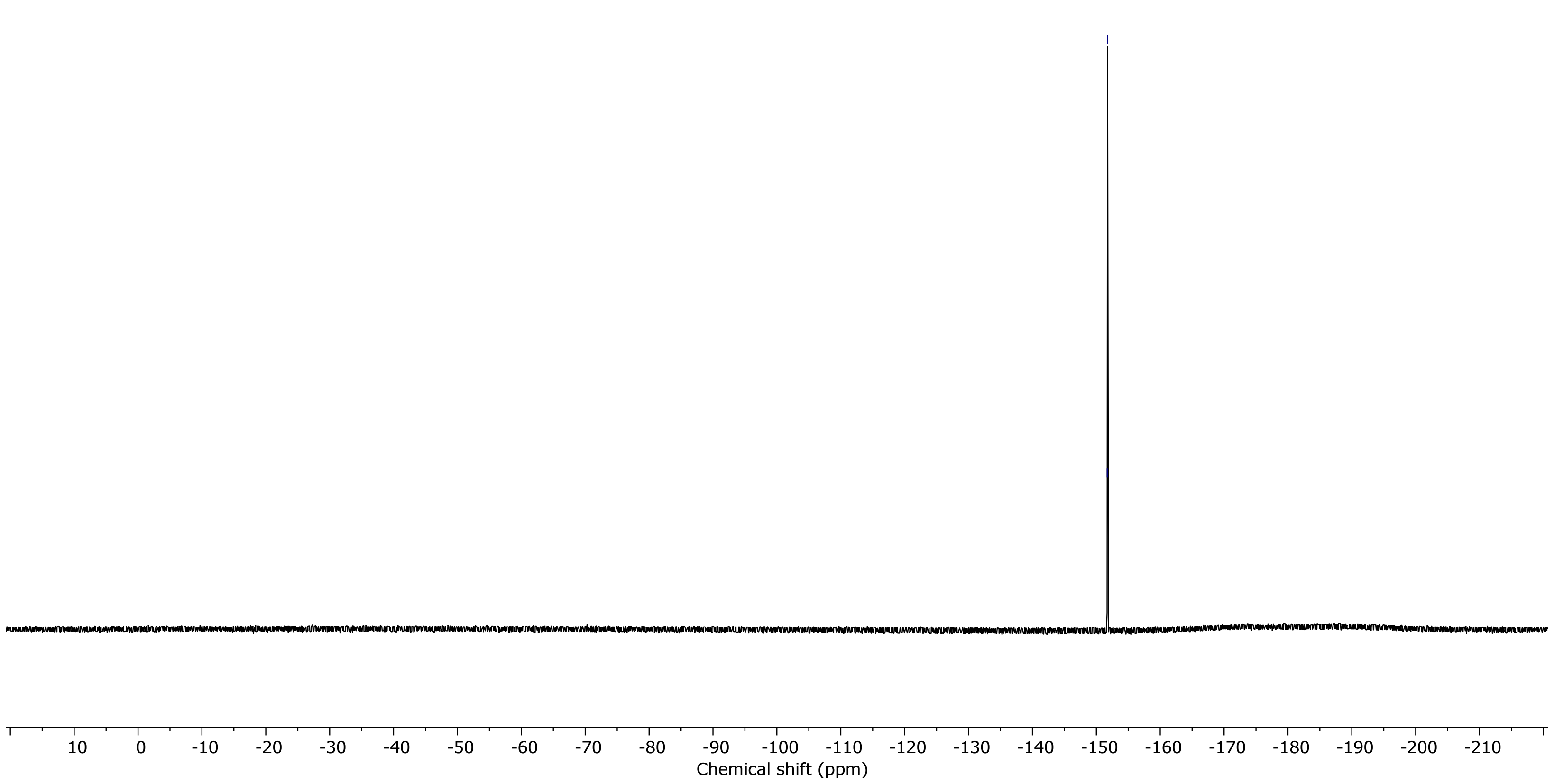

^{1}H NMR of 20a

$CDCl_3$, 600 MHz, 23 ºC.

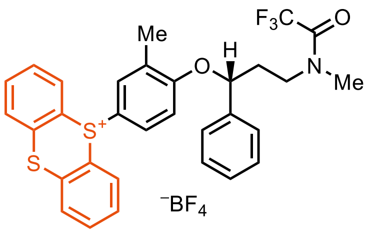

20a

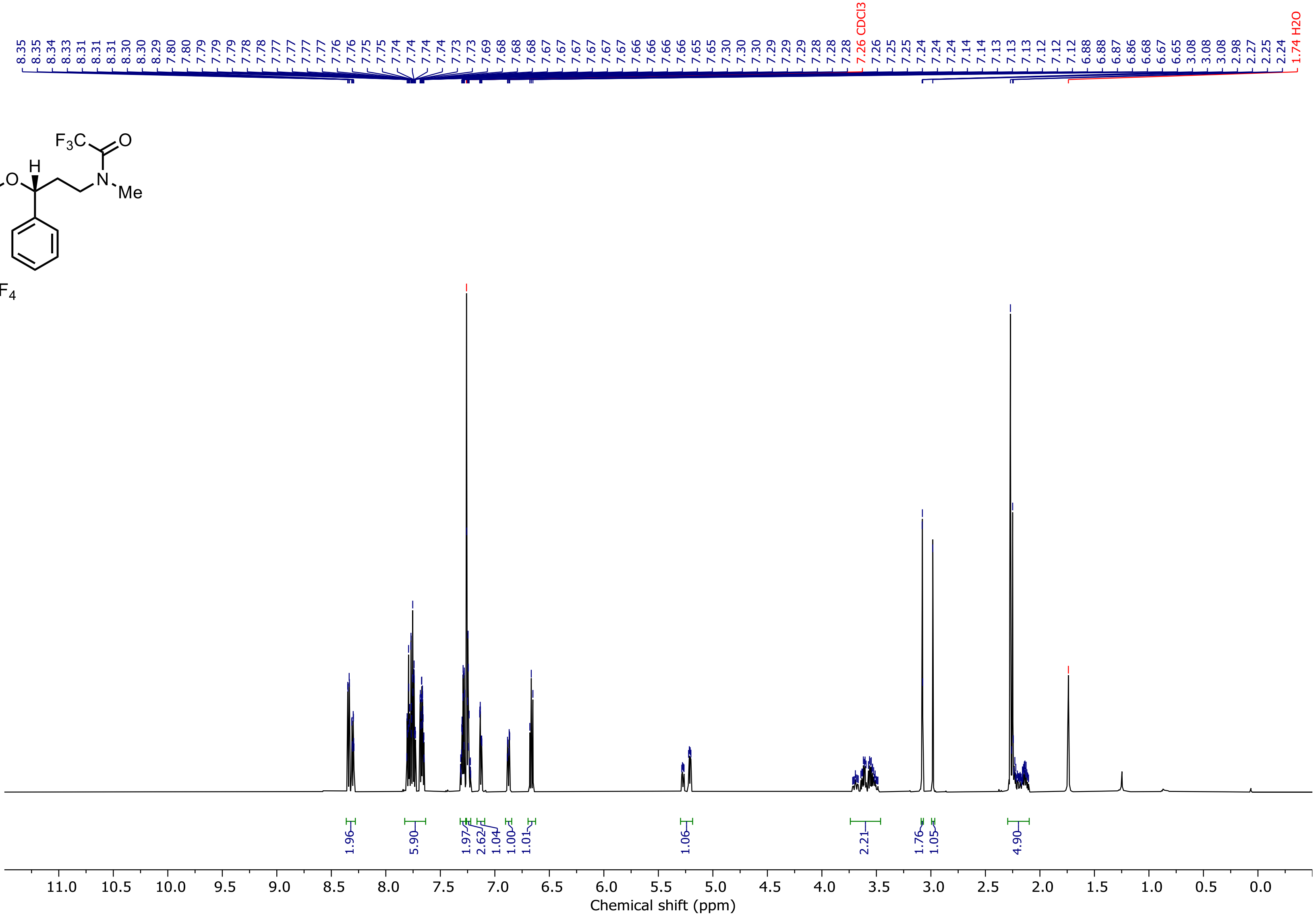

^{13}C NMR of 20a

$CDCl_3$, 151 MHz, 23 ºC.

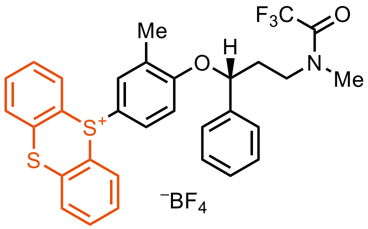

20a

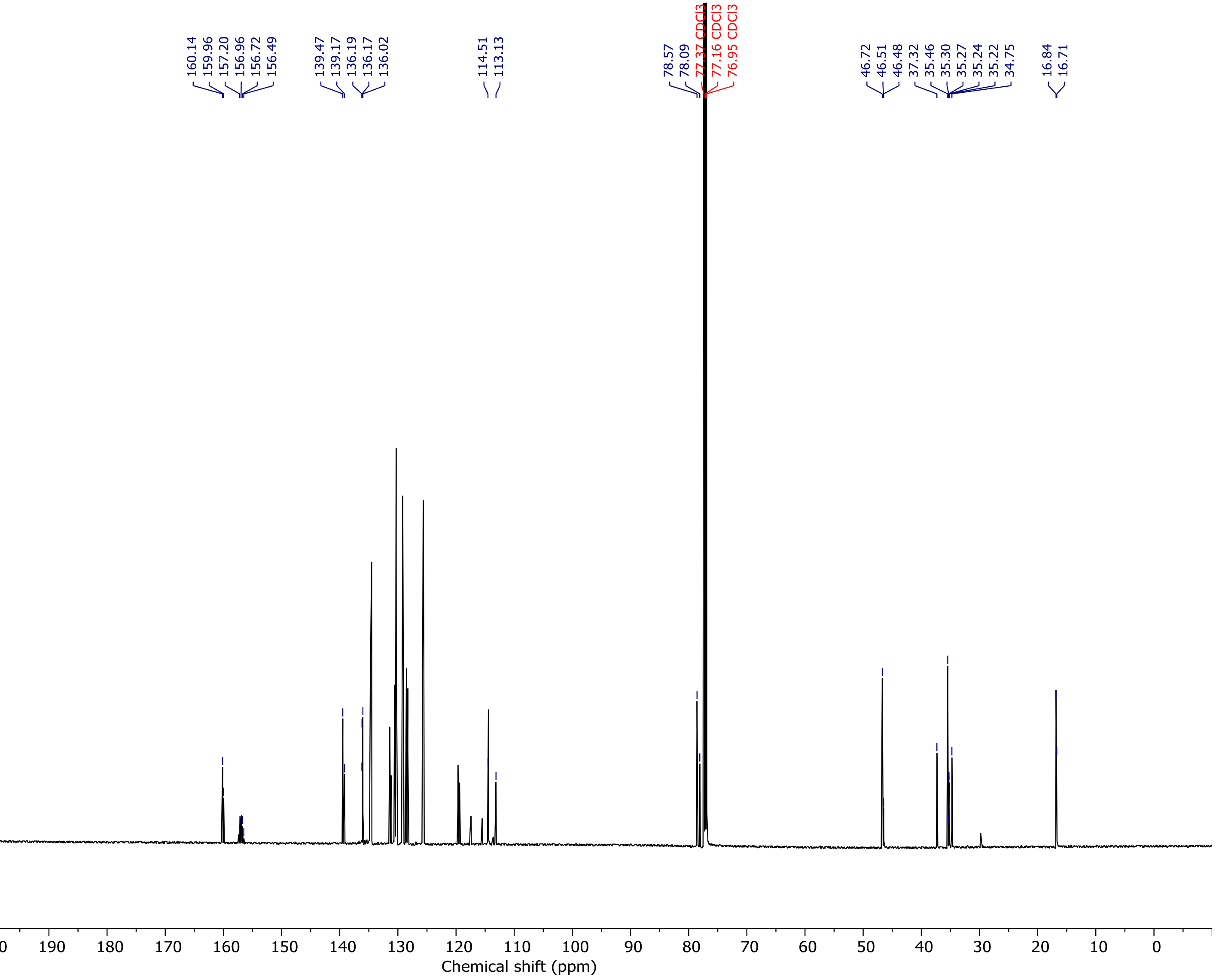

^{19}F NMR of 20a

$CDCl_3$, 565 MHz, 23 ºC.

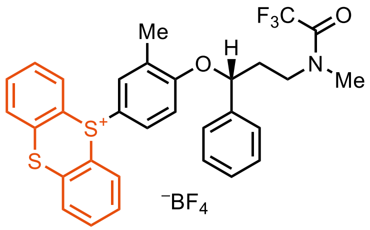

20a

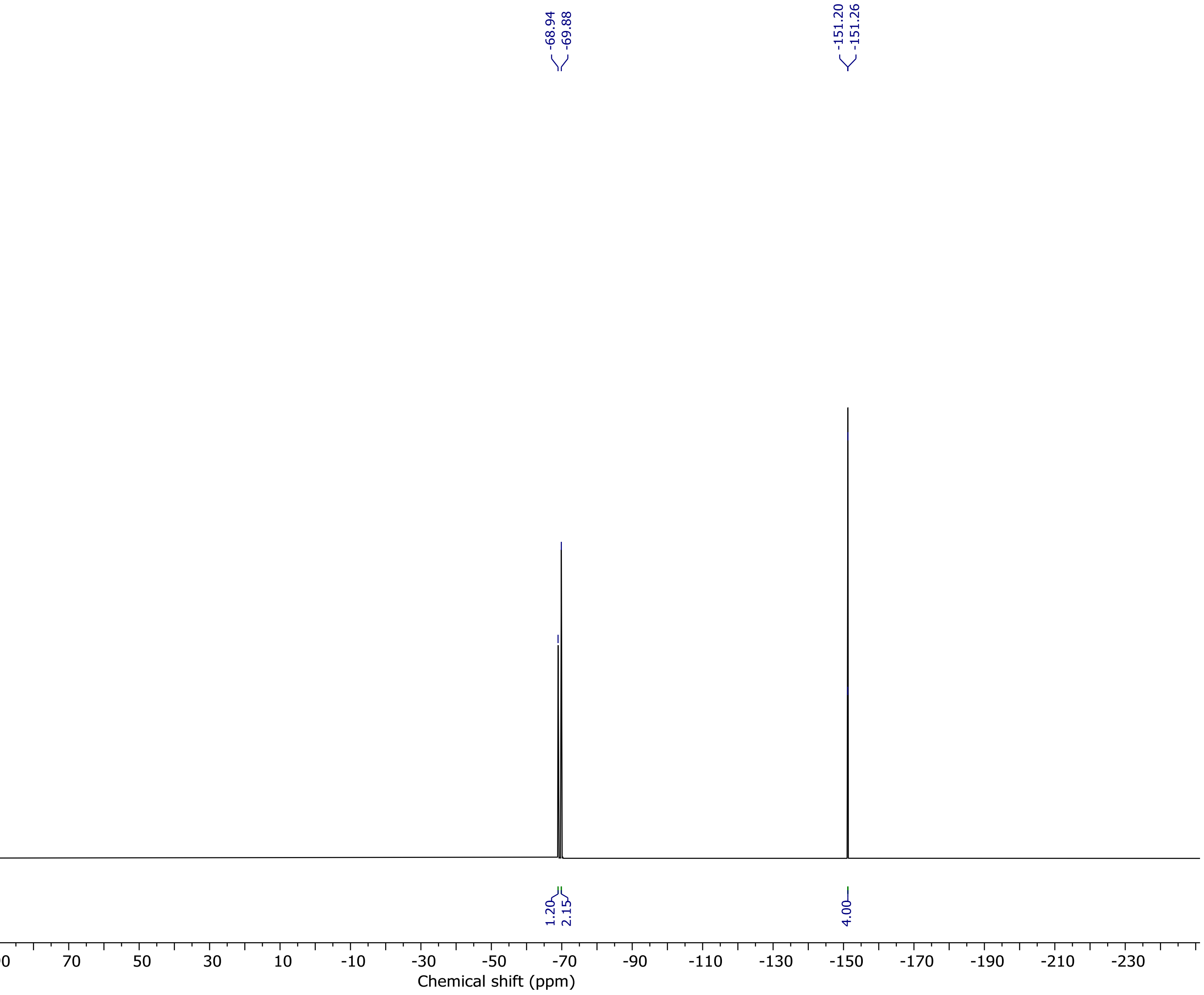

^{1}H NMR of 21a

CD_3CN, 600 MHz, 23 ºC.

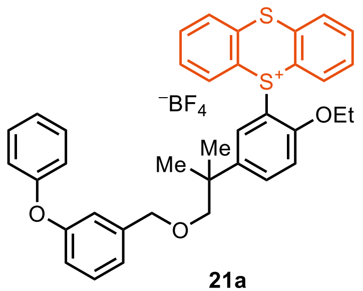

21a

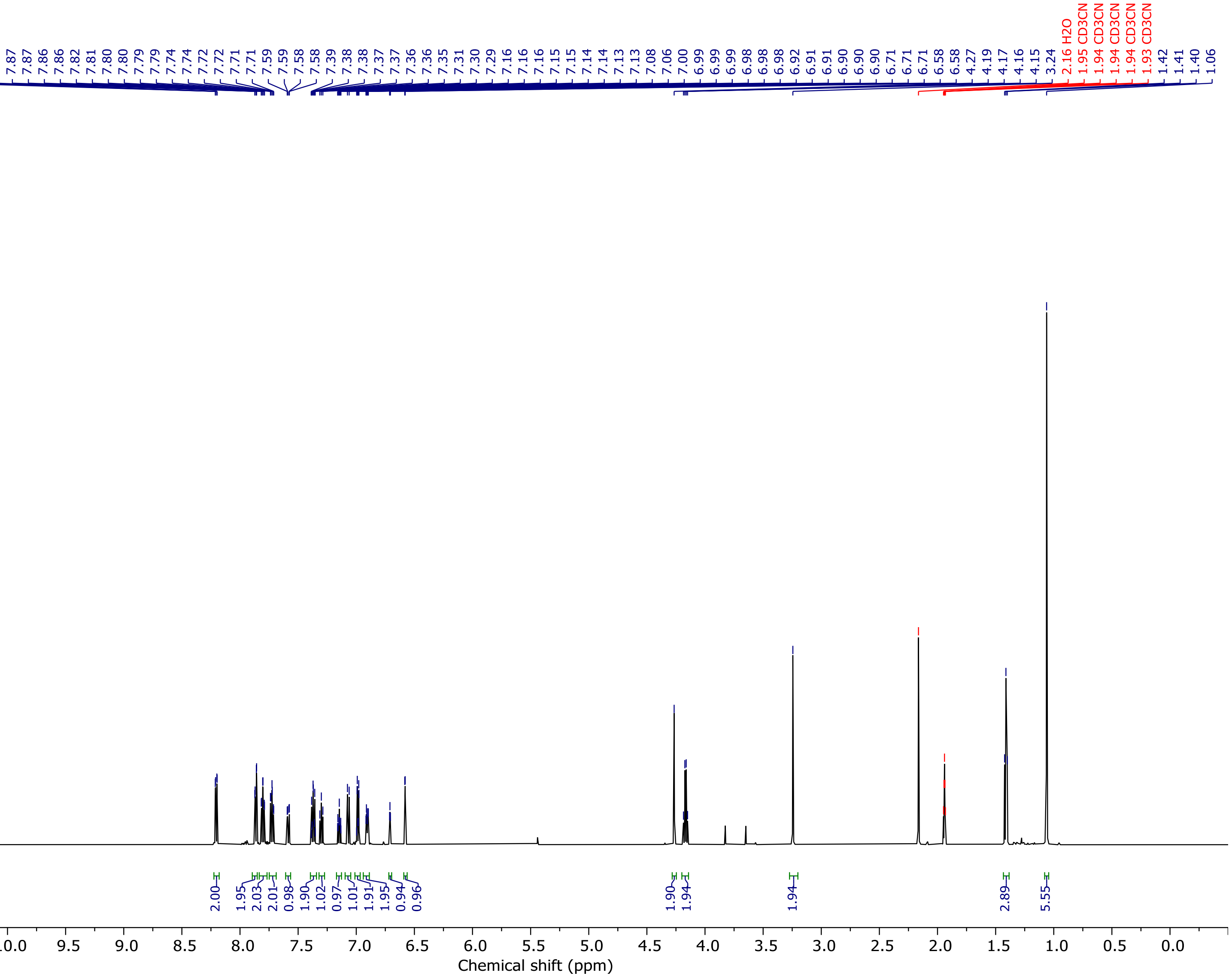

^{13}C NMR of 21a

CD_3CN, 151 MHz, 23 ºC.

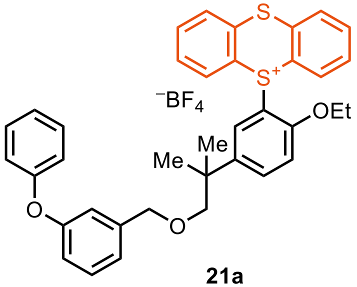

21a

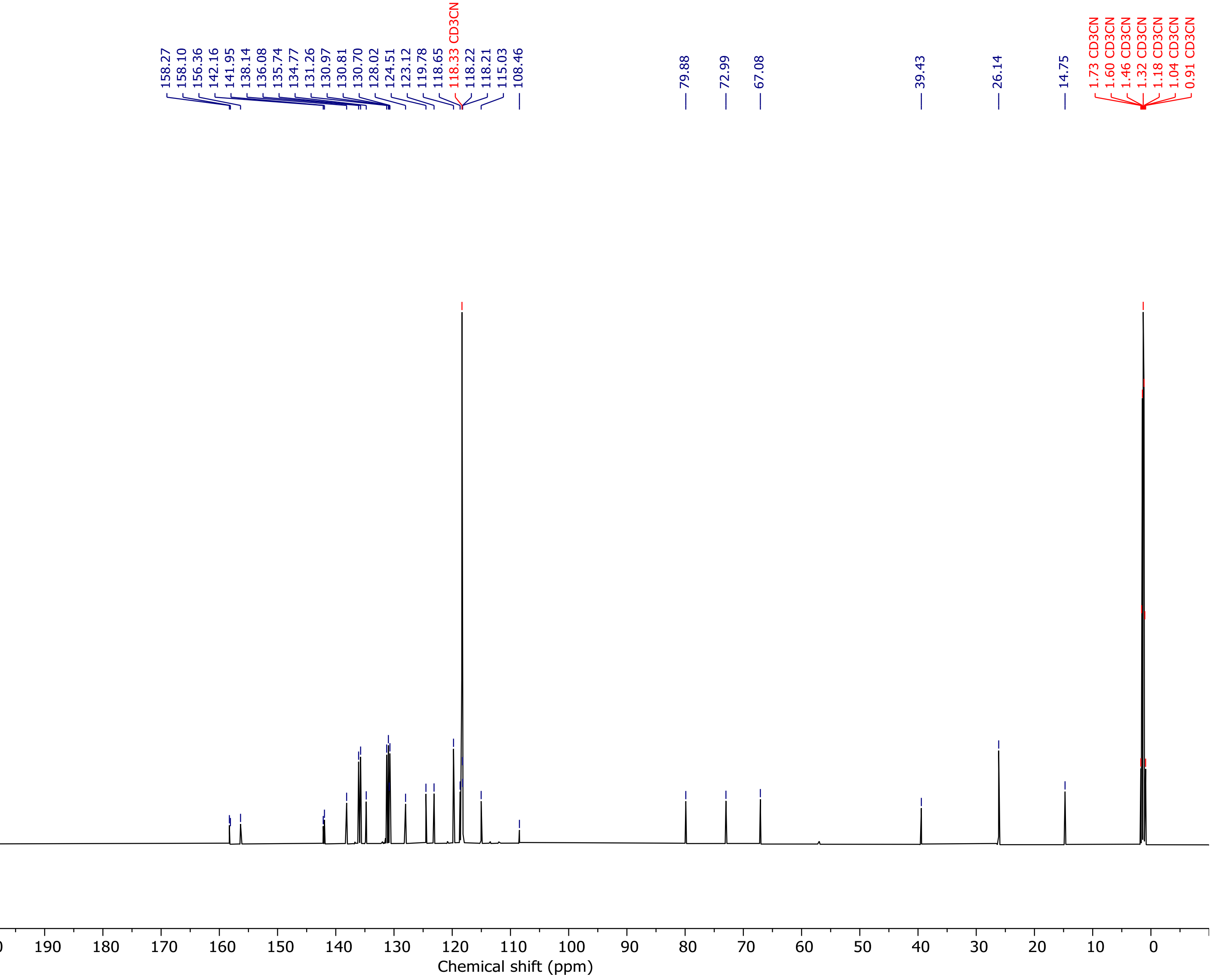

^{19}F NMR of 21a

CD_3CN, 565 MHz, 23 ºC.

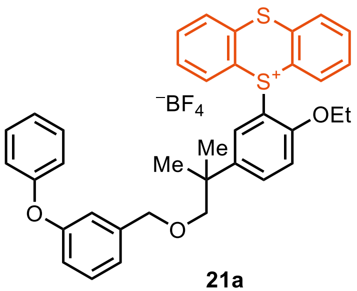

21a

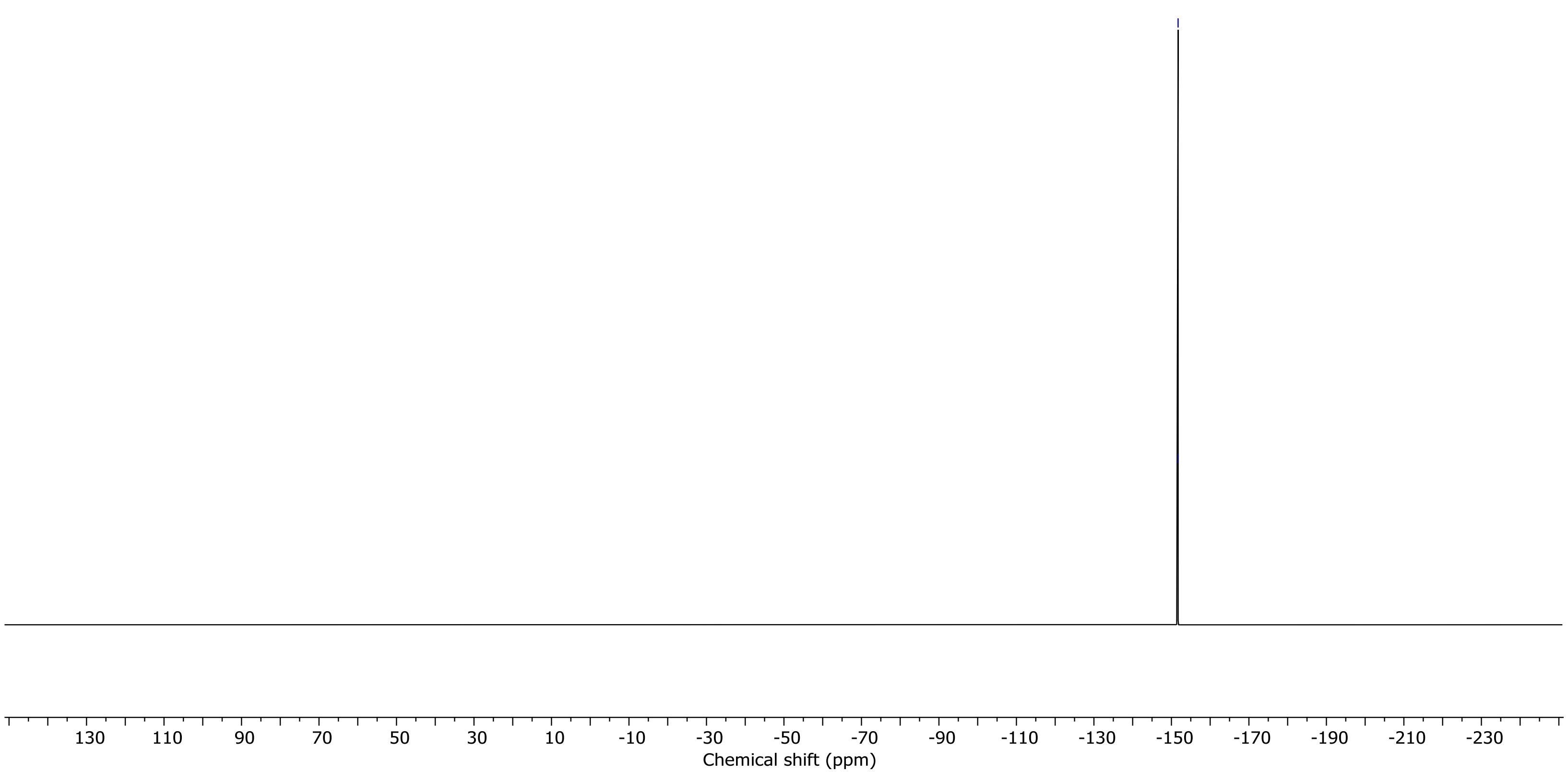

^{1}H NMR of 22a

$(CD_3)_2SO$, 500 MHz, 23 ºC.

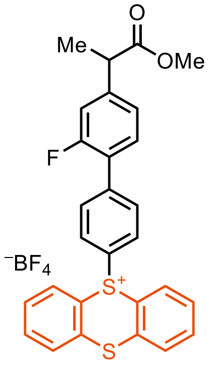

22a

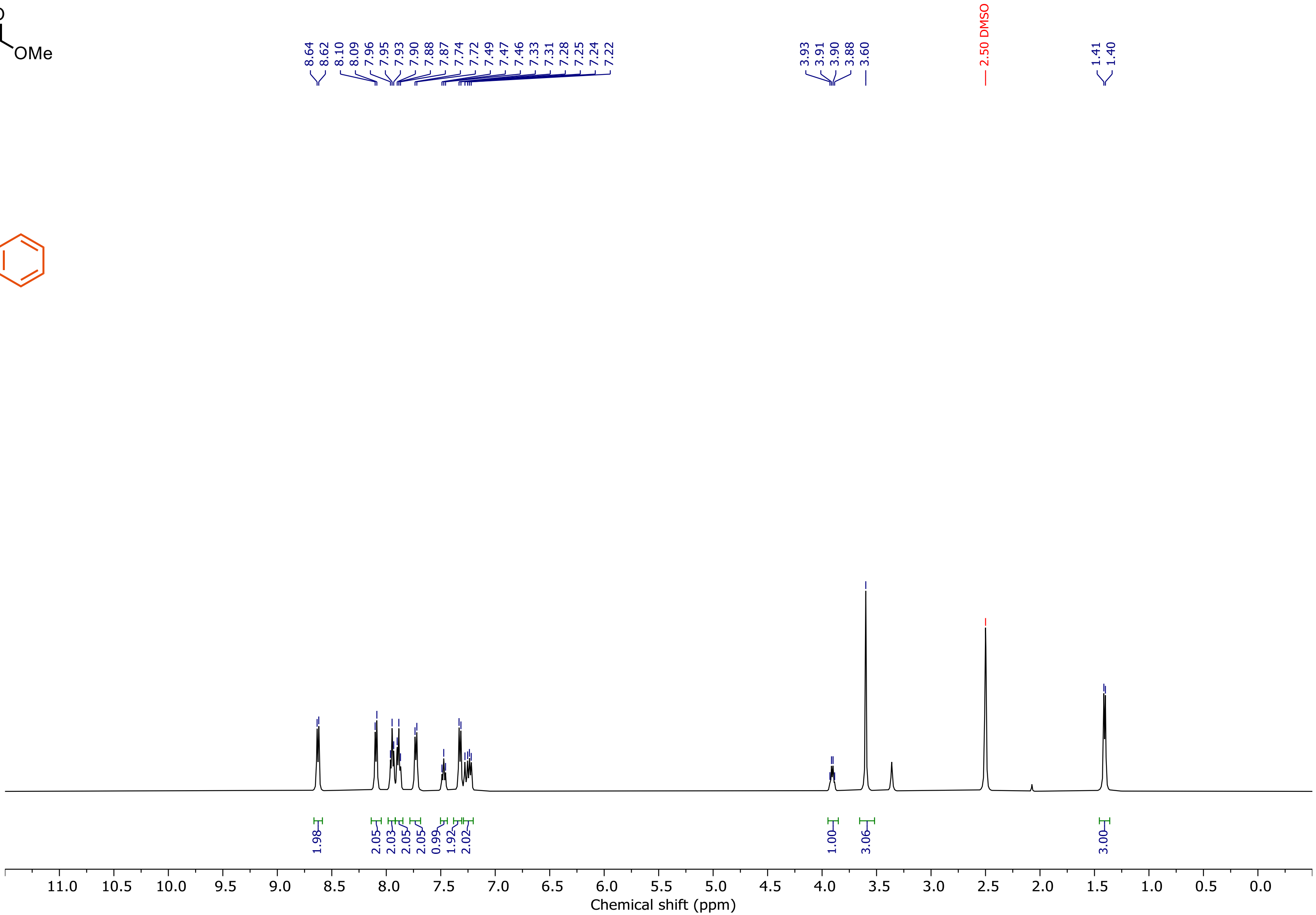

^{13}C NMR of 22a

$(CD_3)_2SO$, 126 MHz, 23 ºC.

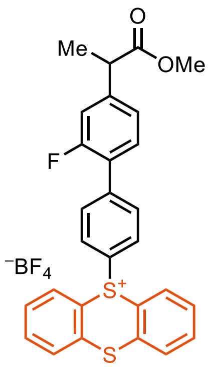

22a

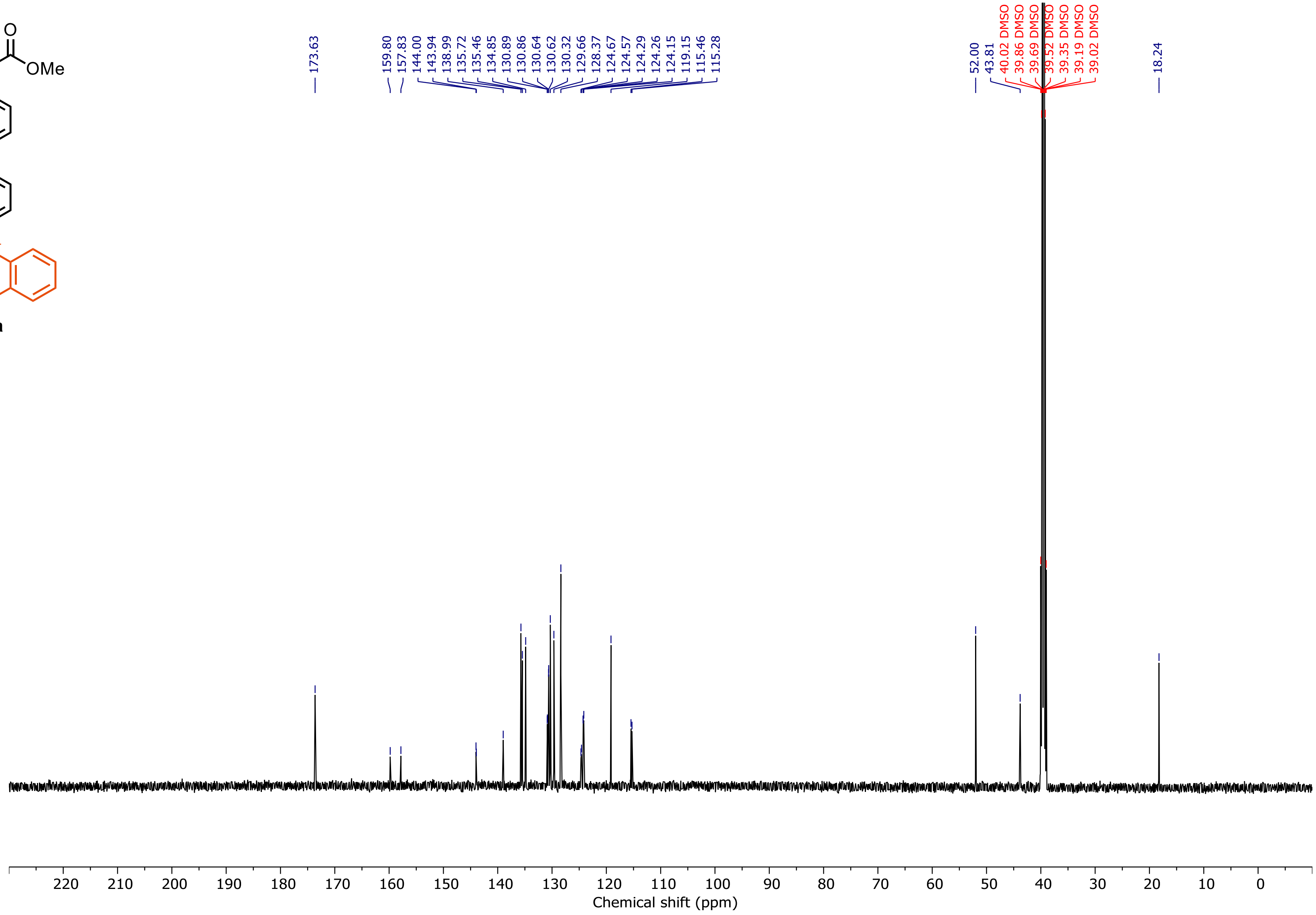

^{19}F NMR of 22a

$(CD_3)_2SO$, 471 MHz, 23 ºC.

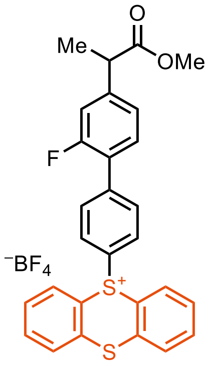

22a

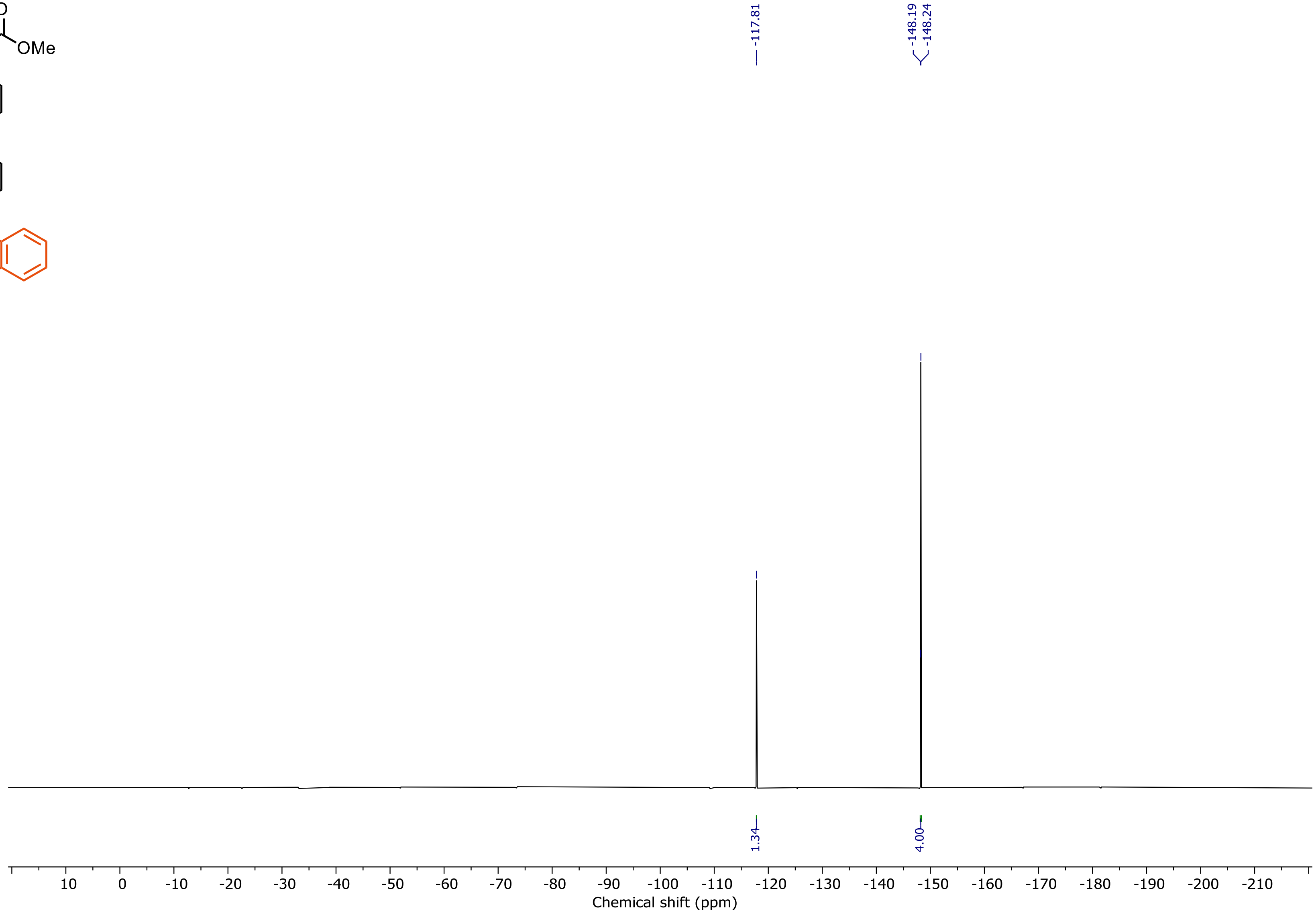

^{1}H NMR of 23a

CD_3CN, 600 MHz, 23 ºC.

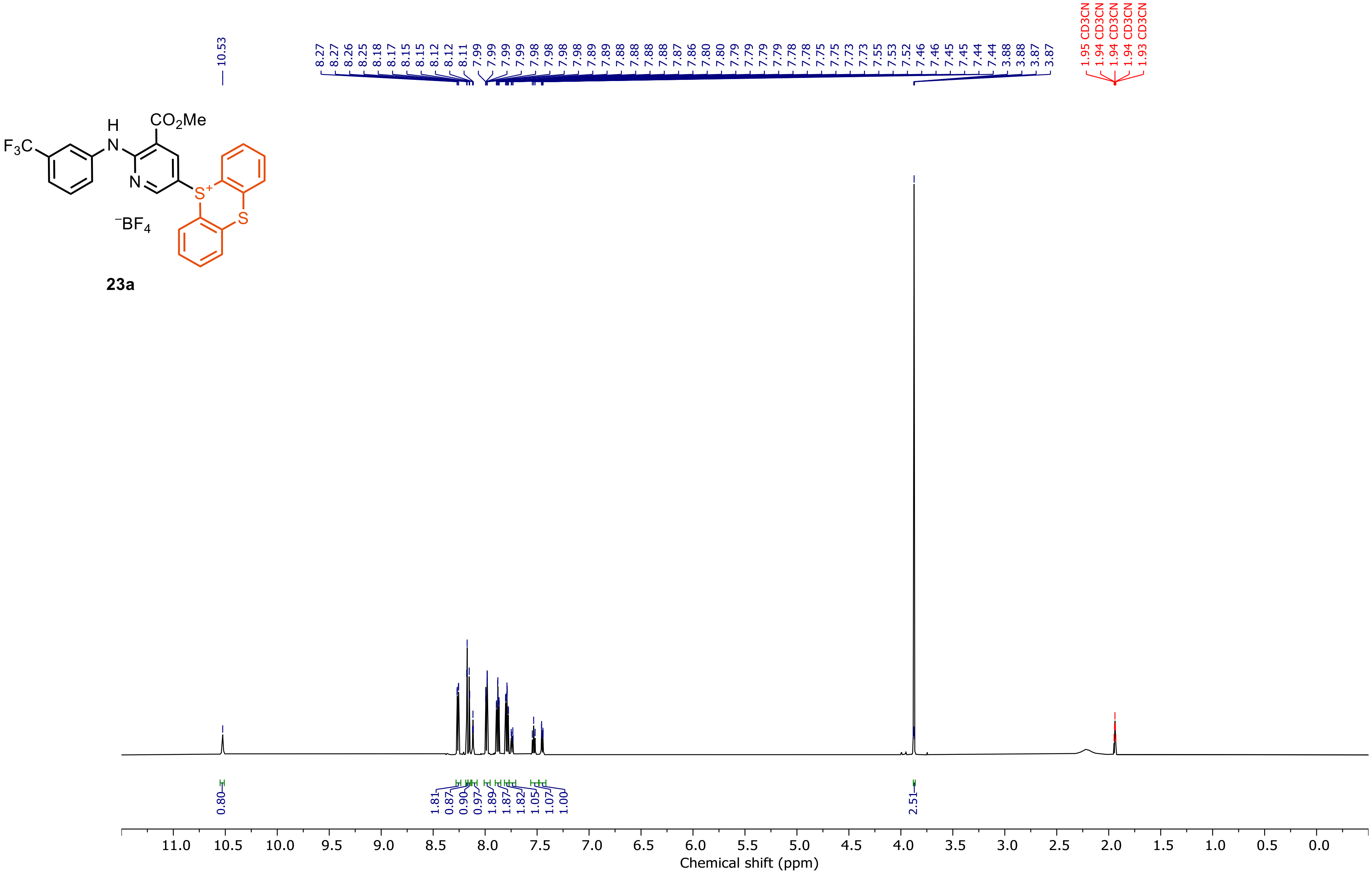

^{13}C NMR of 23a

CD_3CN, 151 MHz, 23 ºC.

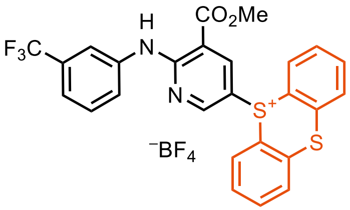

23a

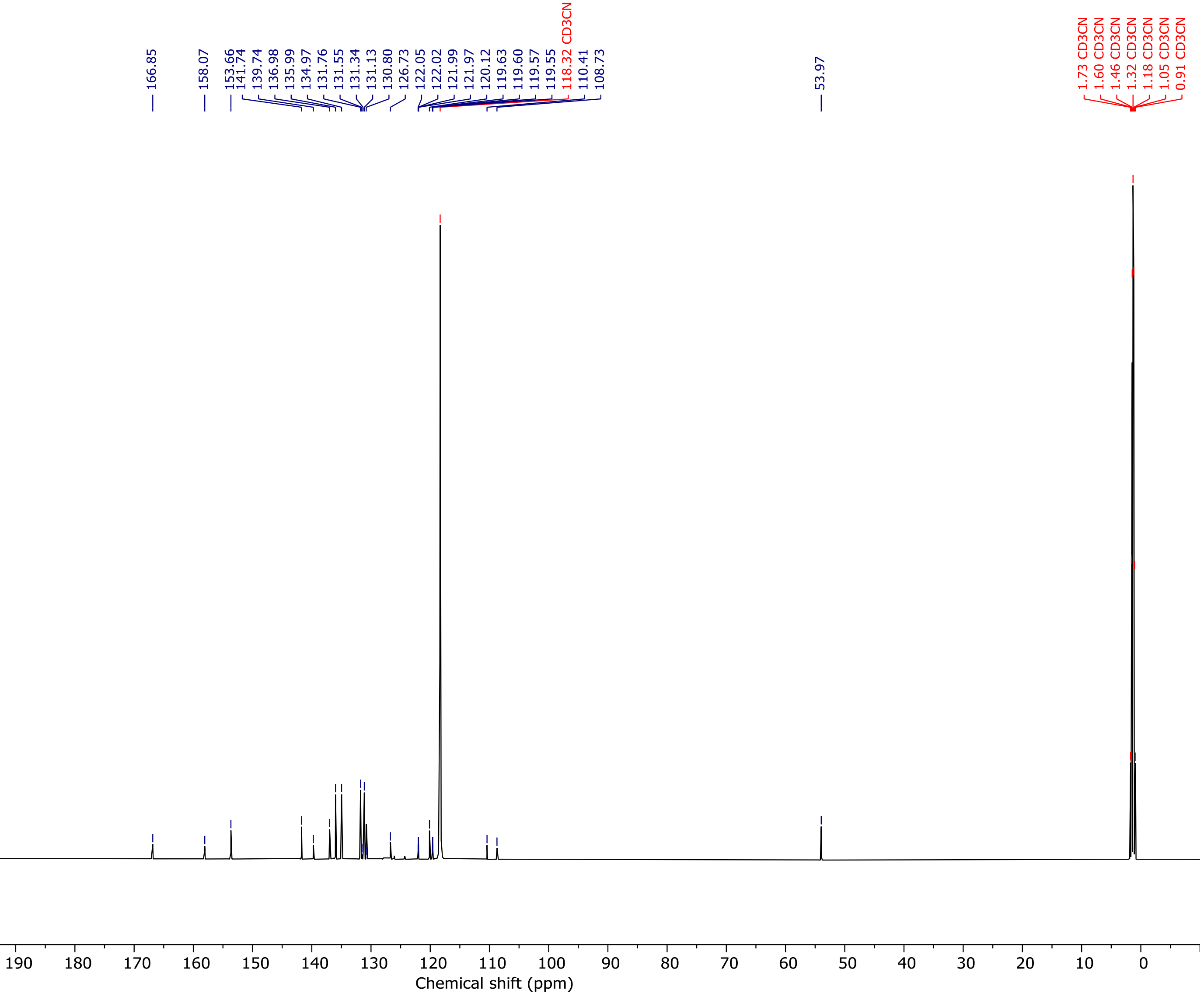

^{19}F NMR of 23a

CD_3CN, 565 MHz, 23 ºC.

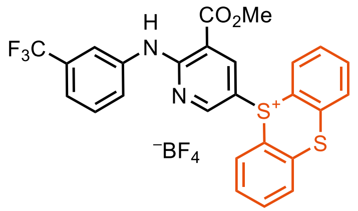

23a

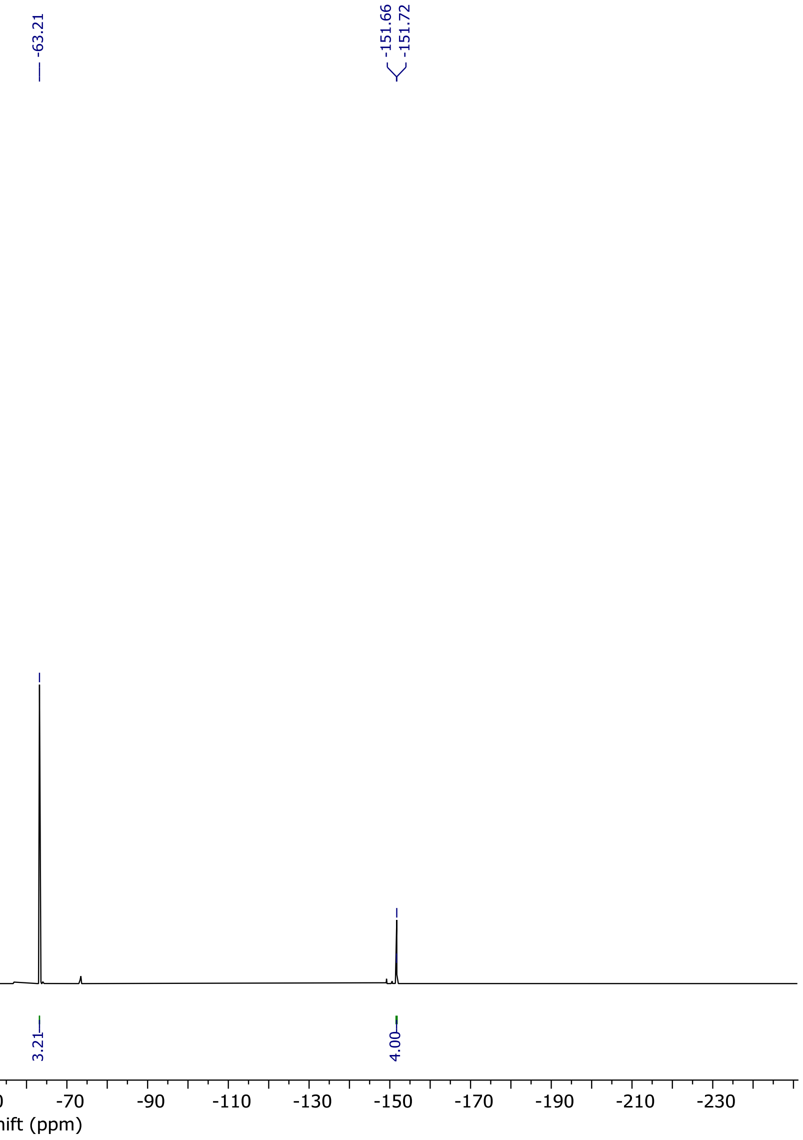

^{1}H NMR of 24a

CD_3CN, 500 MHz, 23 ºC.

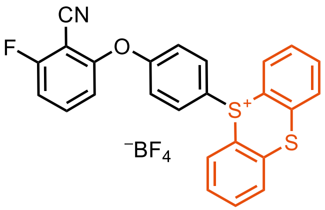

24a

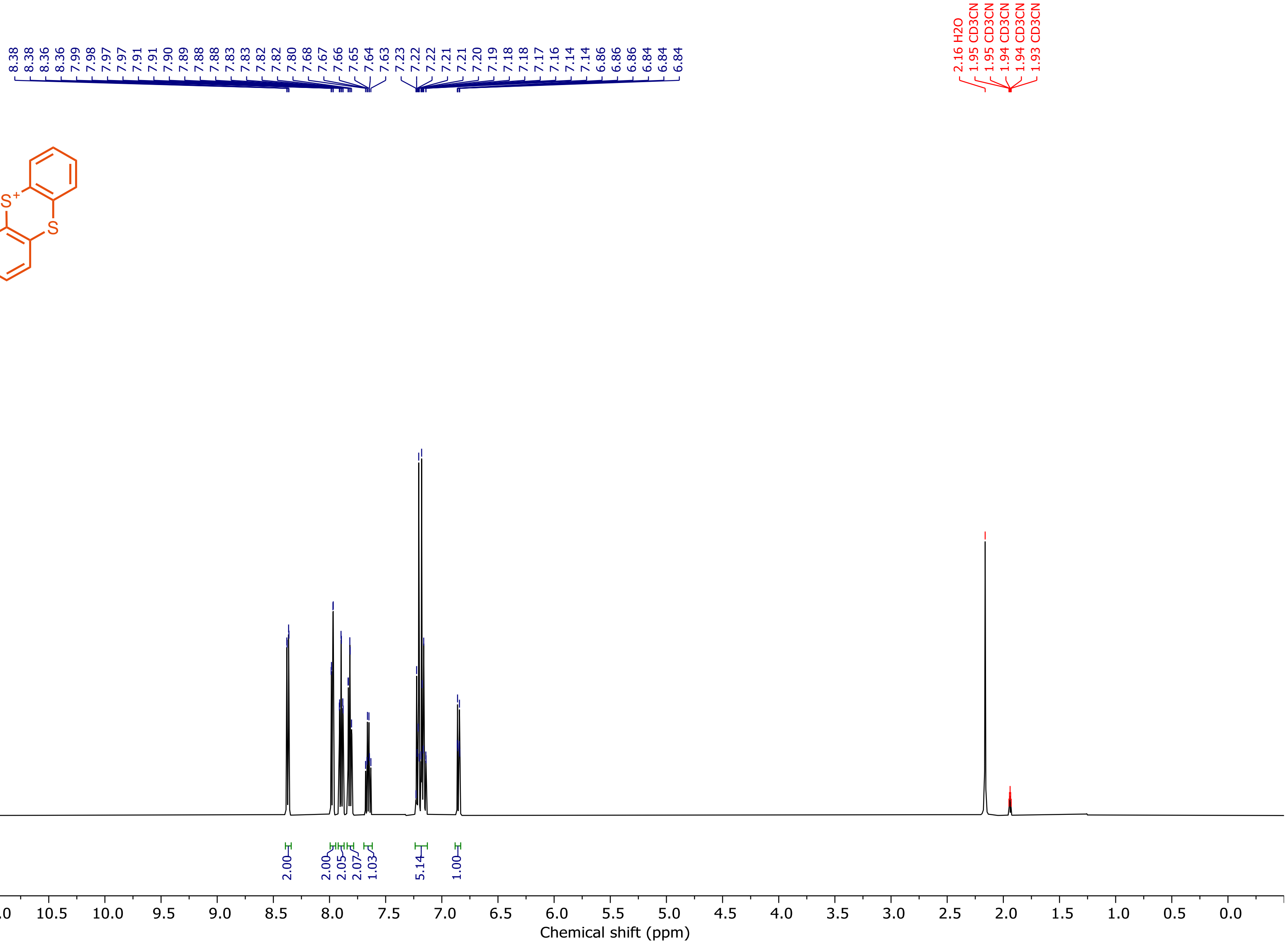

^{13}C NMR of 24a

CD_3CN, 126 MHz, 23 ºC.

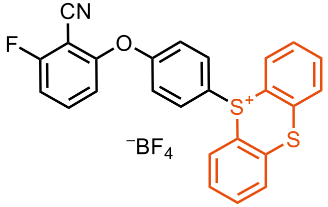

24a

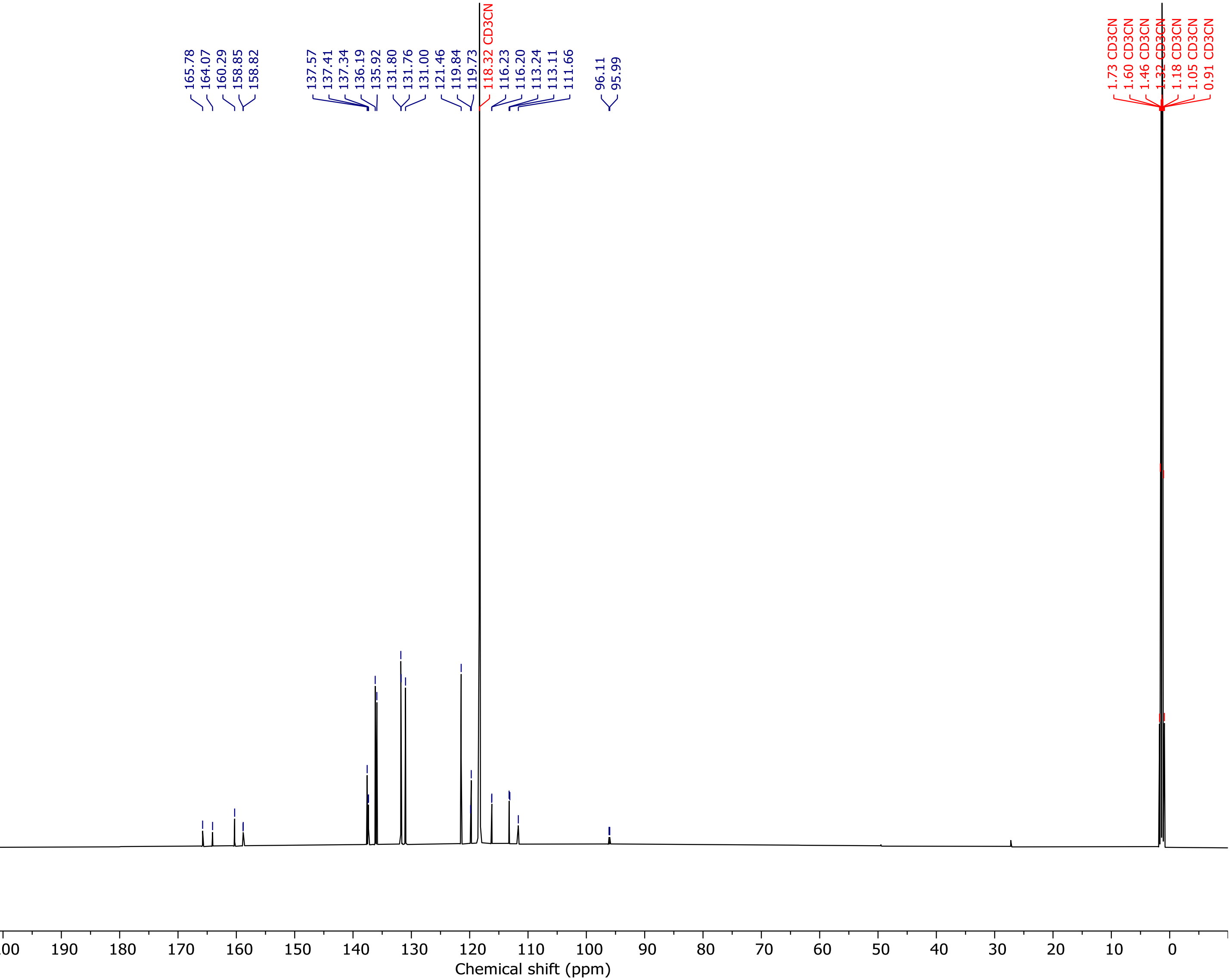

^{19}F NMR of 24a

CD_3CN, 471 MHz, 23 ºC.

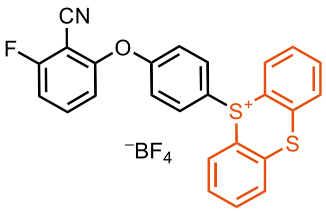

24a

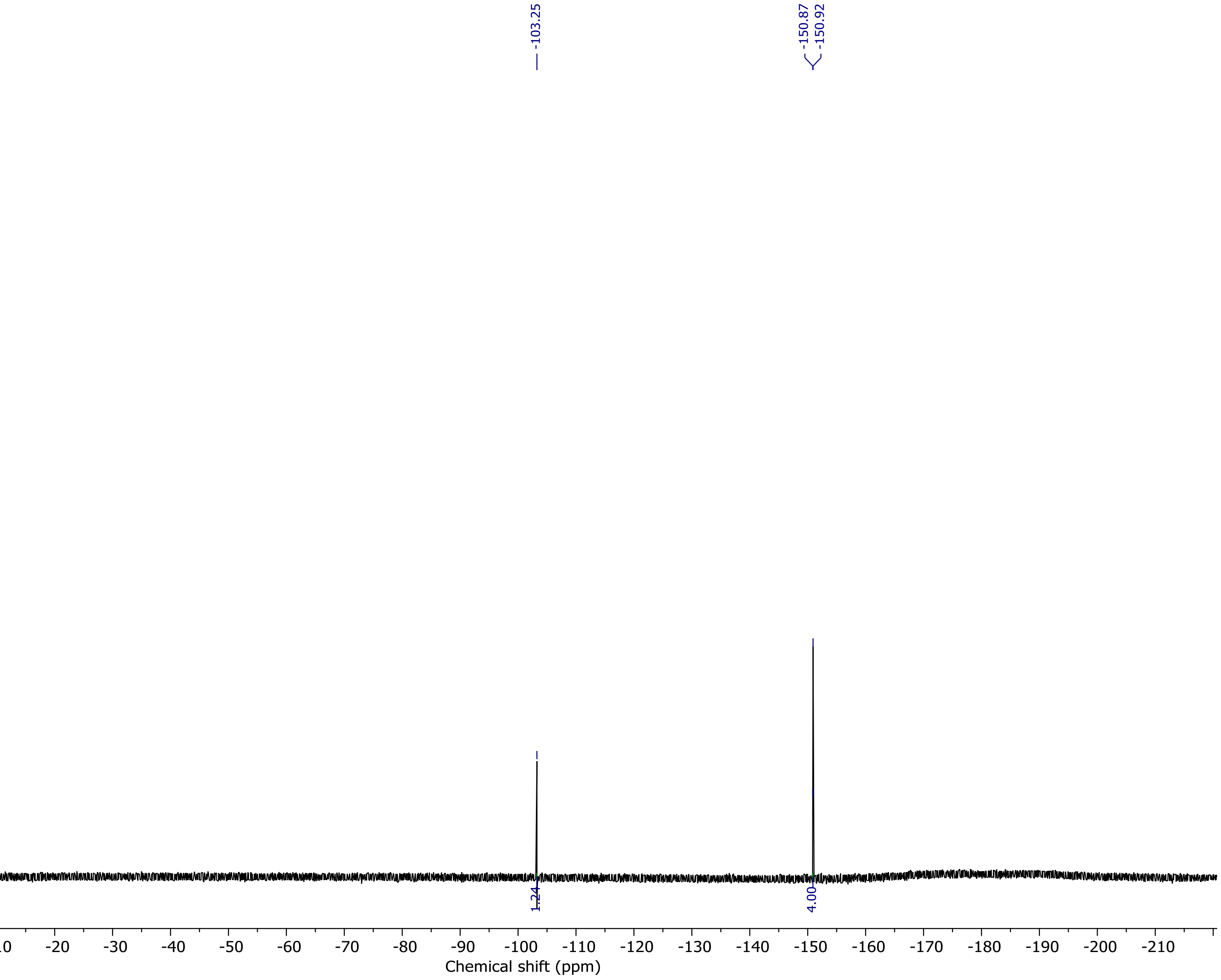

^{1}H NMR of 25a

$CDCl_3$, 500 MHz, 23 ºC.

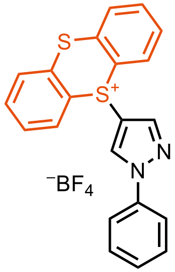

25a

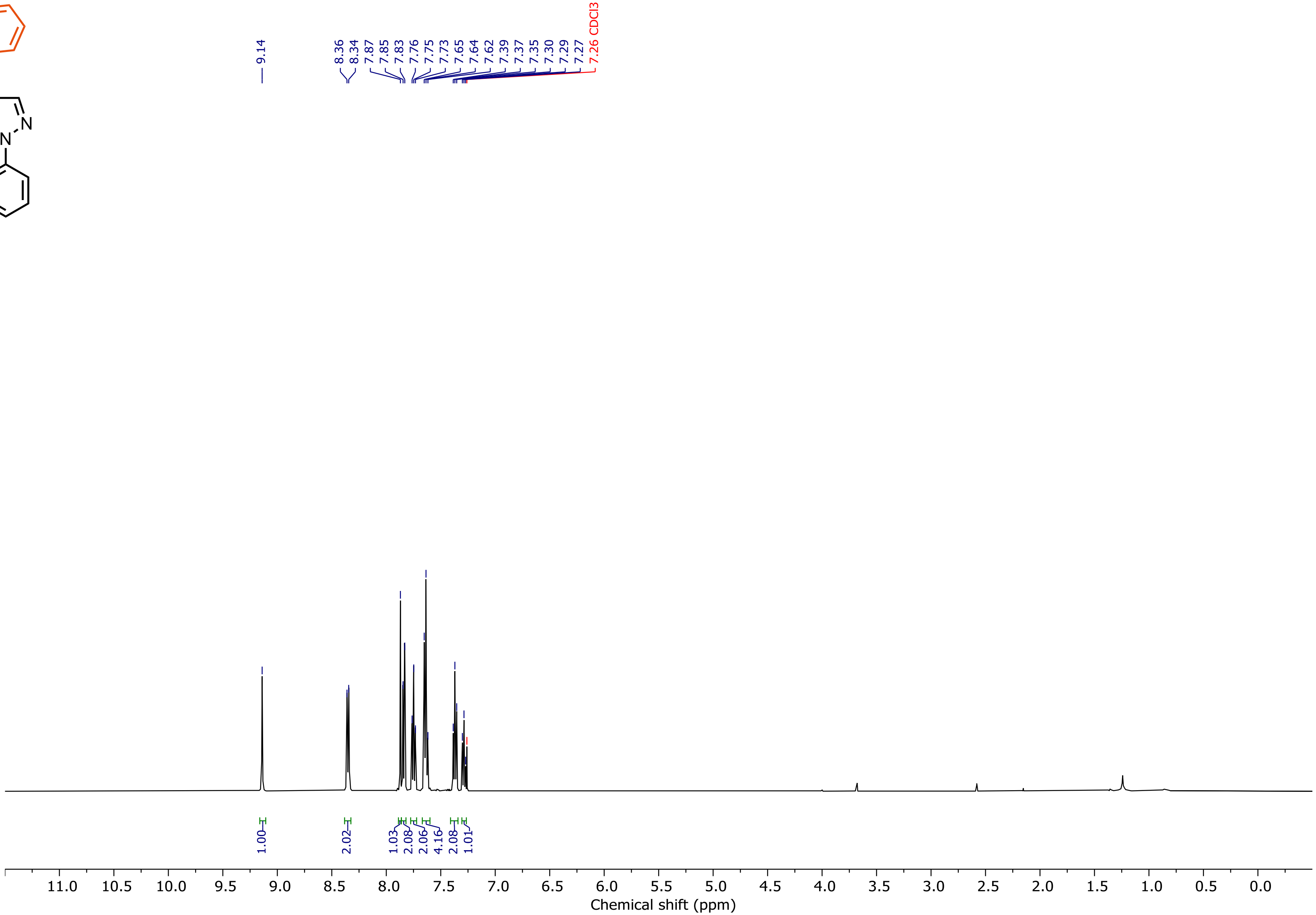

^{13}C NMR of 25a

$CDCl_3$, 126 MHz, 23 ºC.

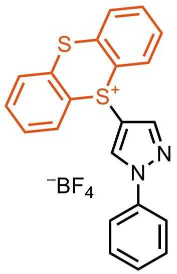

25a

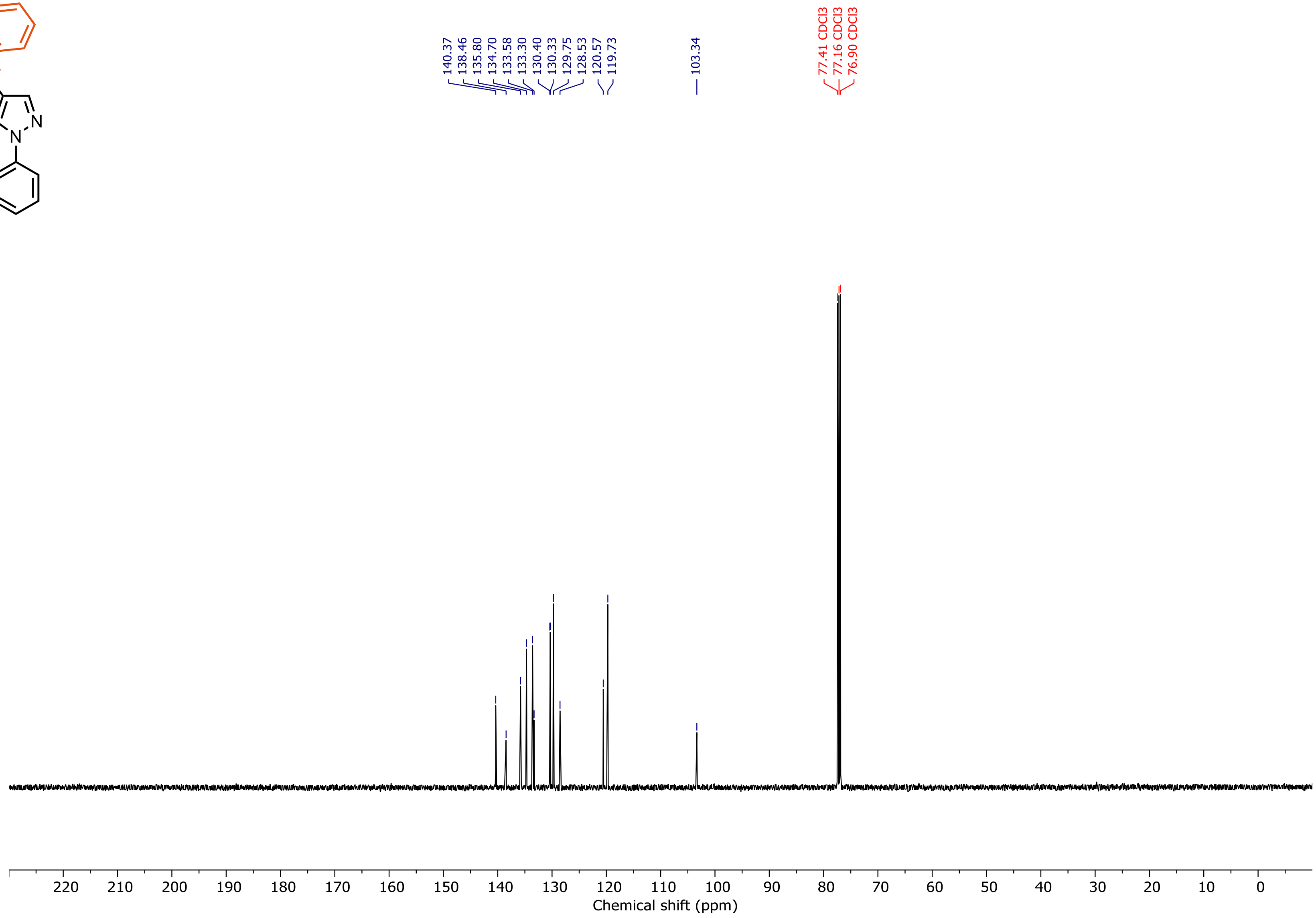

^{19}F NMR of 25a

$CDCl_3$, 471 MHz, 23 ºC.

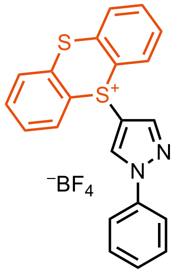

25a

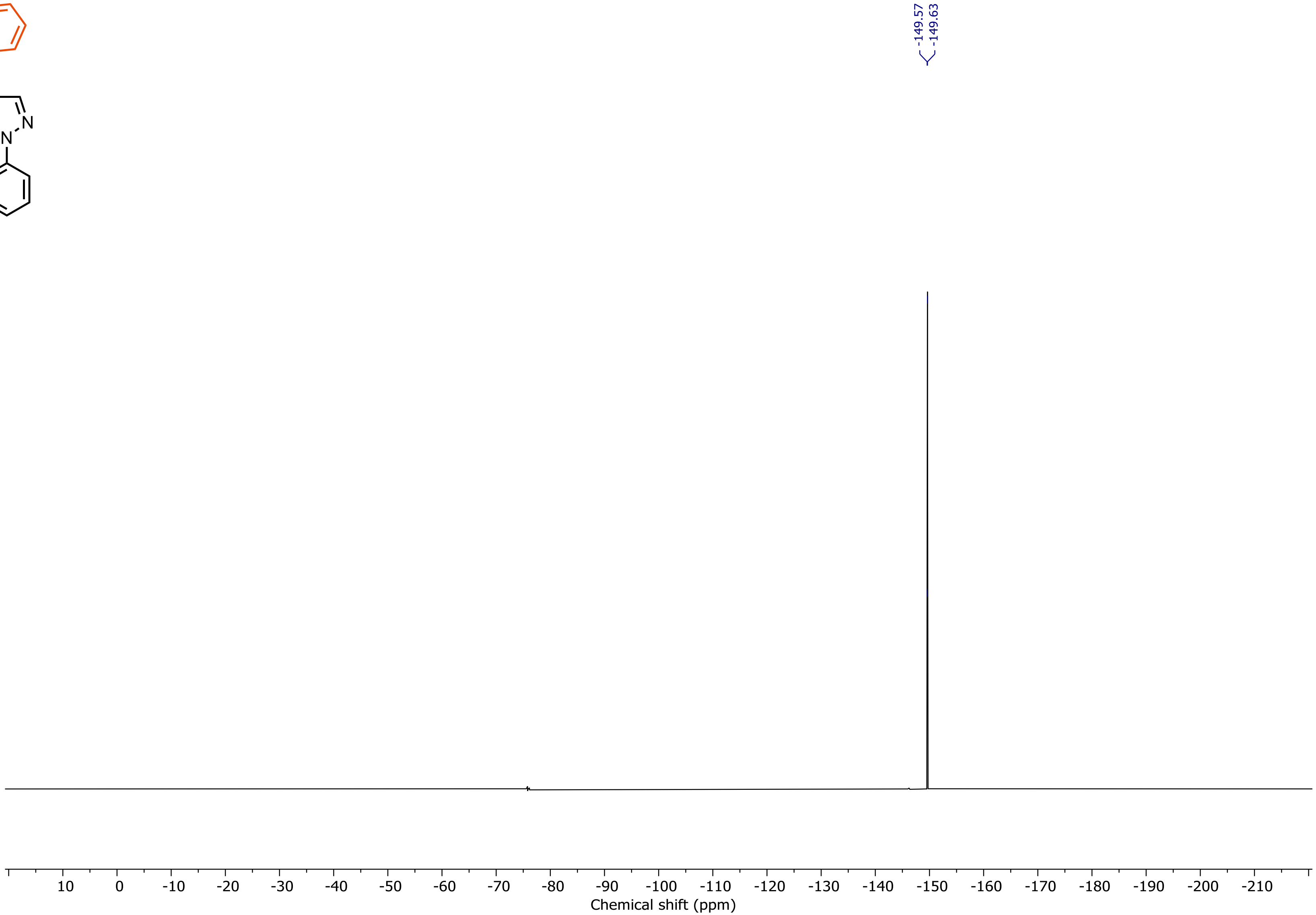

^{1}H NMR of 26a

CD_3CN, 500 MHz, 23 ºC.

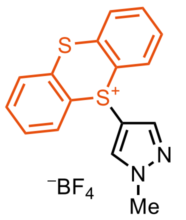

26a

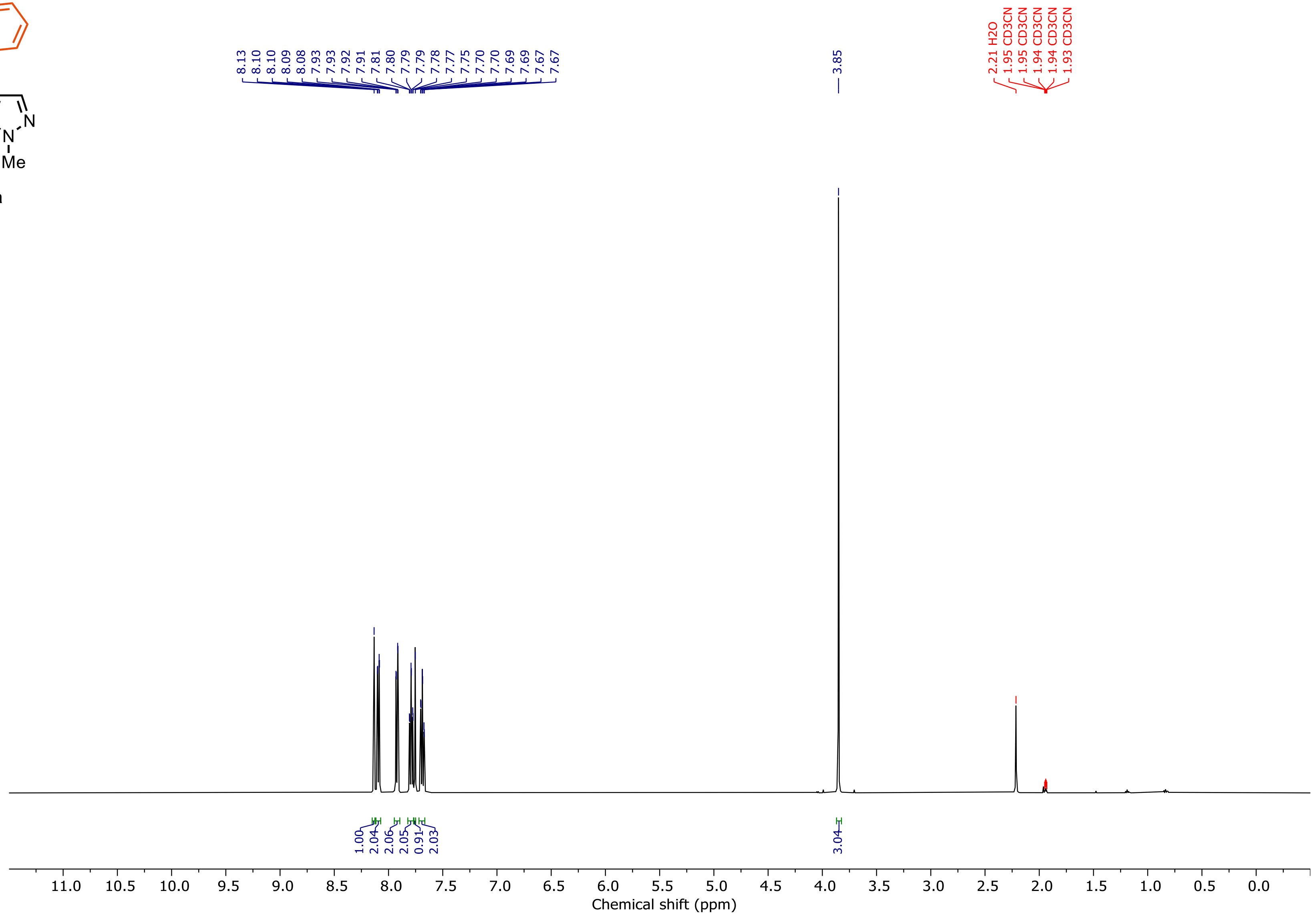

^{13}C NMR of 26a

CD_3CN, 151 MHz, 23 ºC.

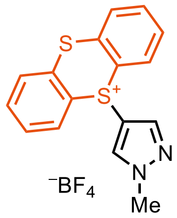

26a

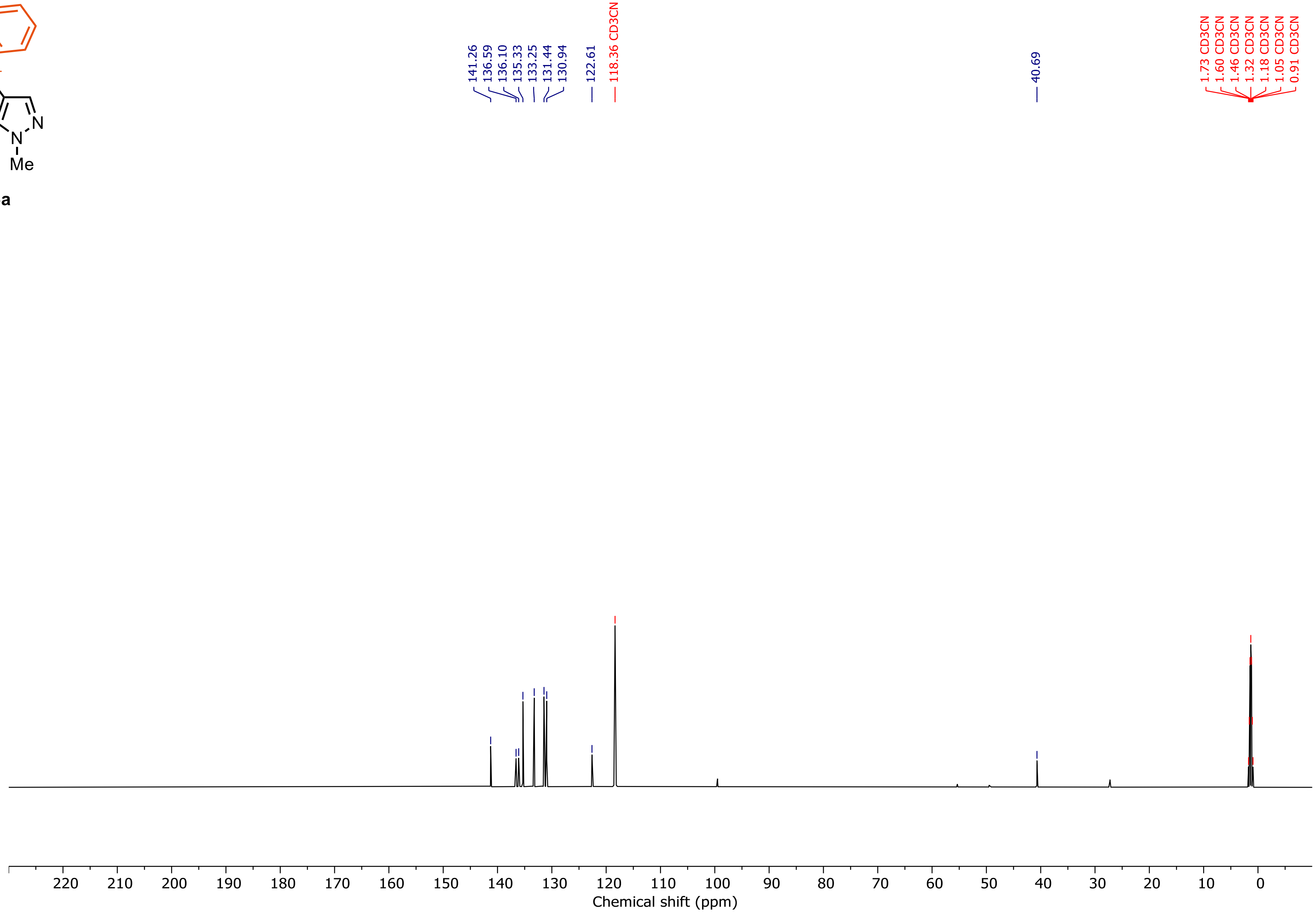

^{19}F NMR of 26a

CD_3CN, 471 MHz, 23 ºC.

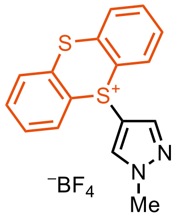

26a

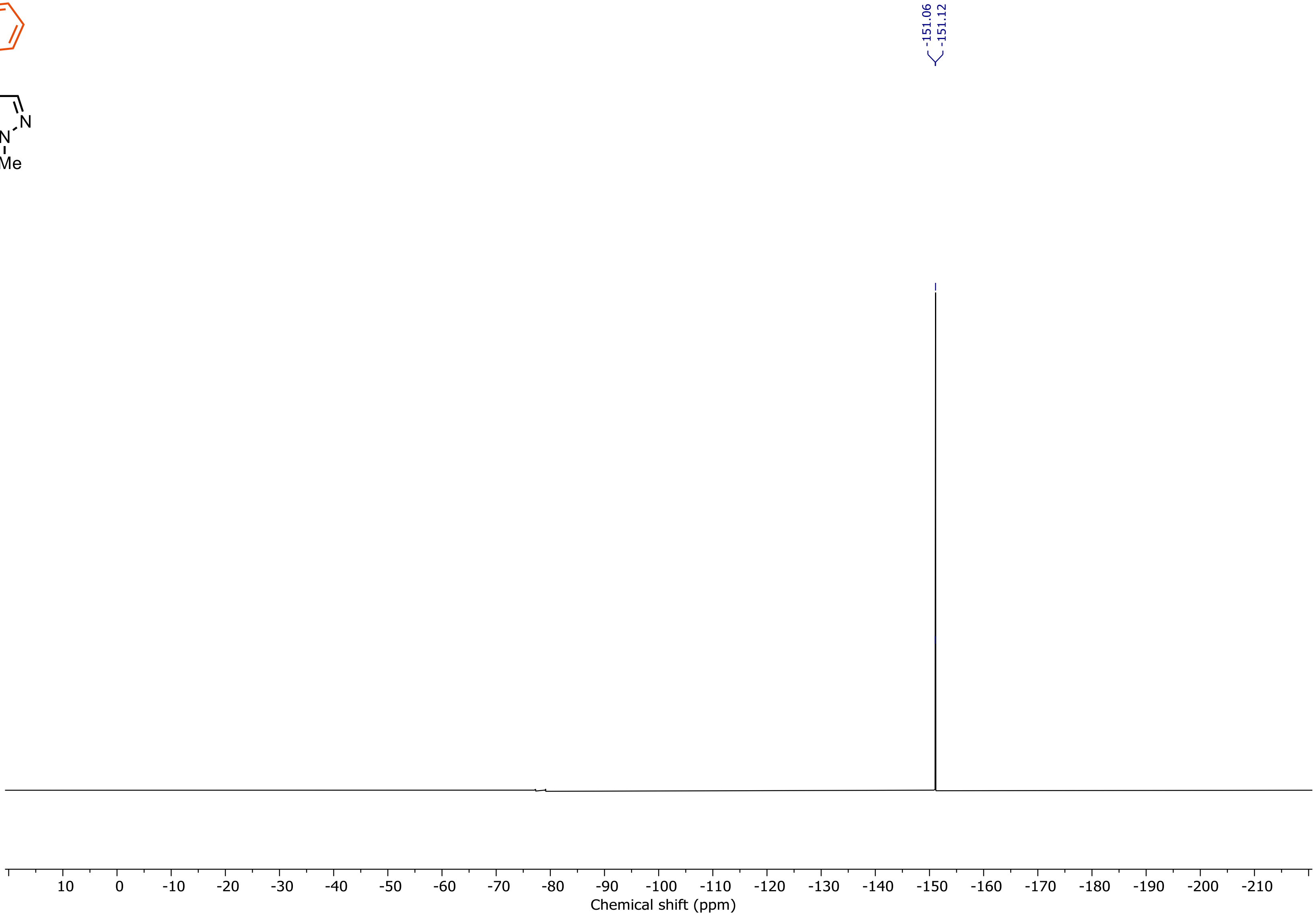

^{1}H NMR of 27

CDCl$_3$, 500 MHz, 23 ºC.

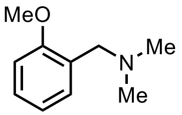

27

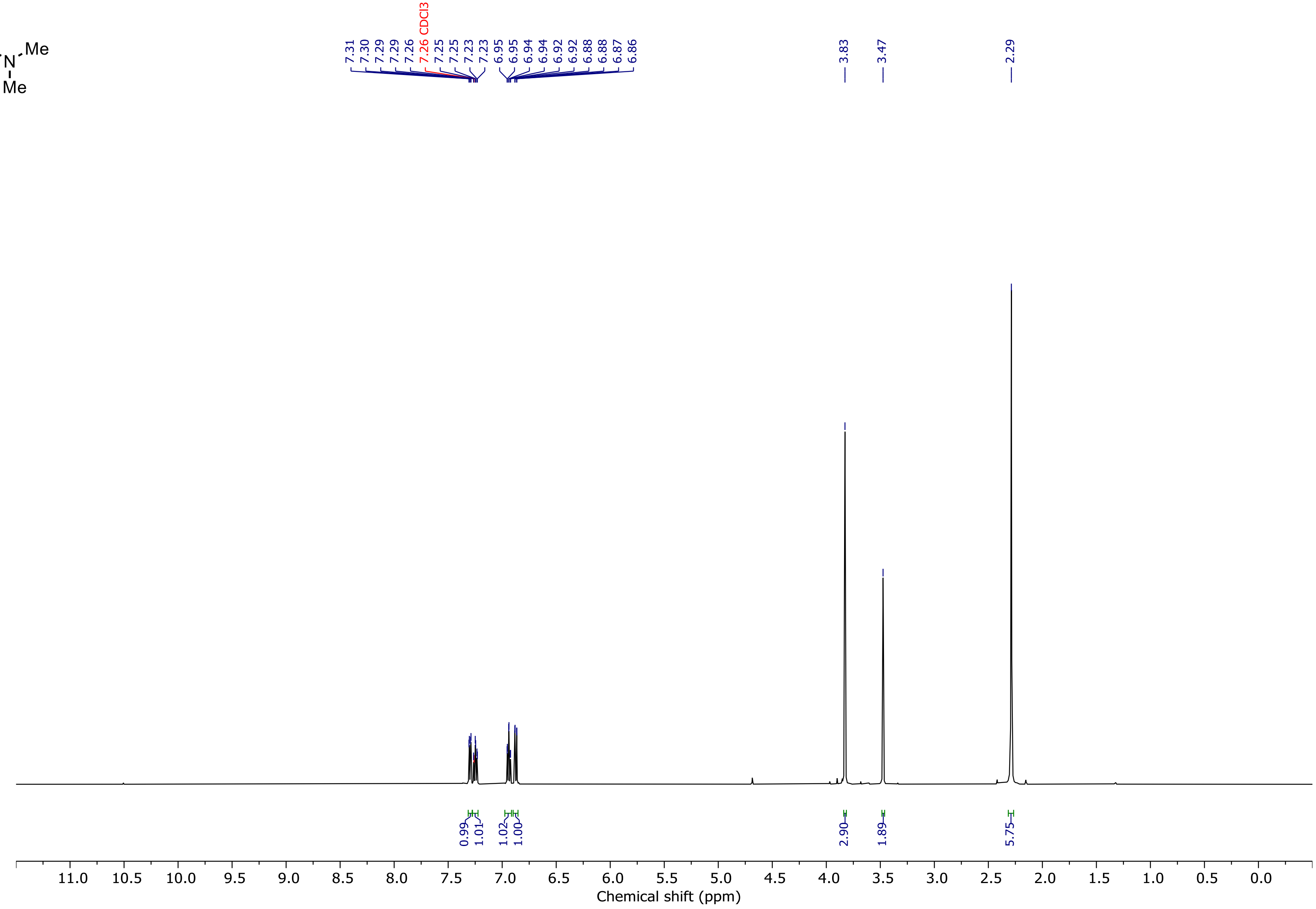

^{13}C NMR of 27

$CDCl_3$, 126 MHz, 23 ºC.

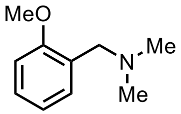

27

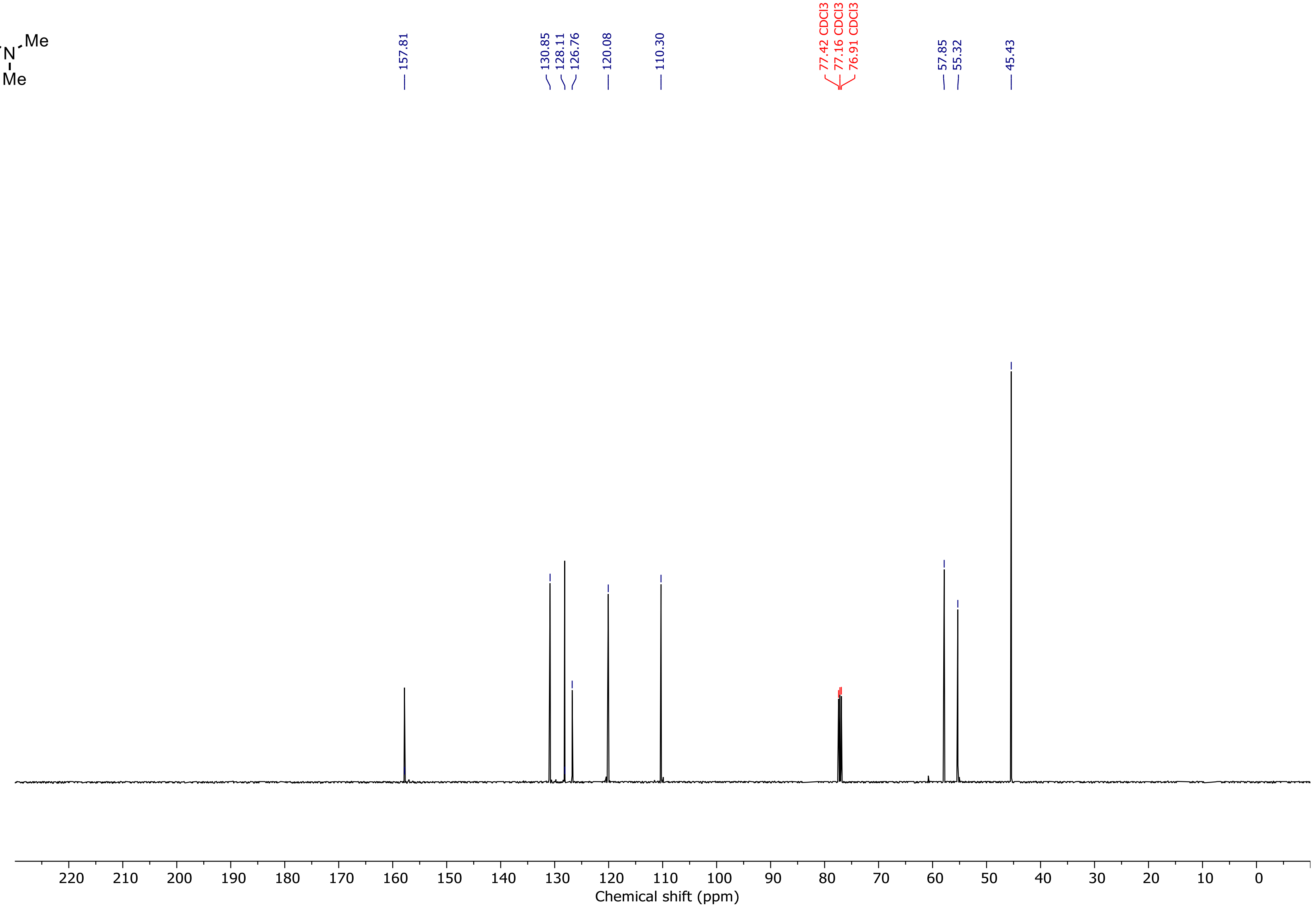

^{1}H NMR of 27a

CD_3CN, 500 MHz, 23 ºC.

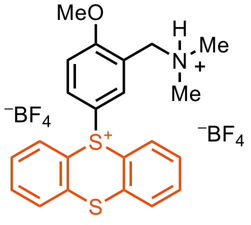

27a

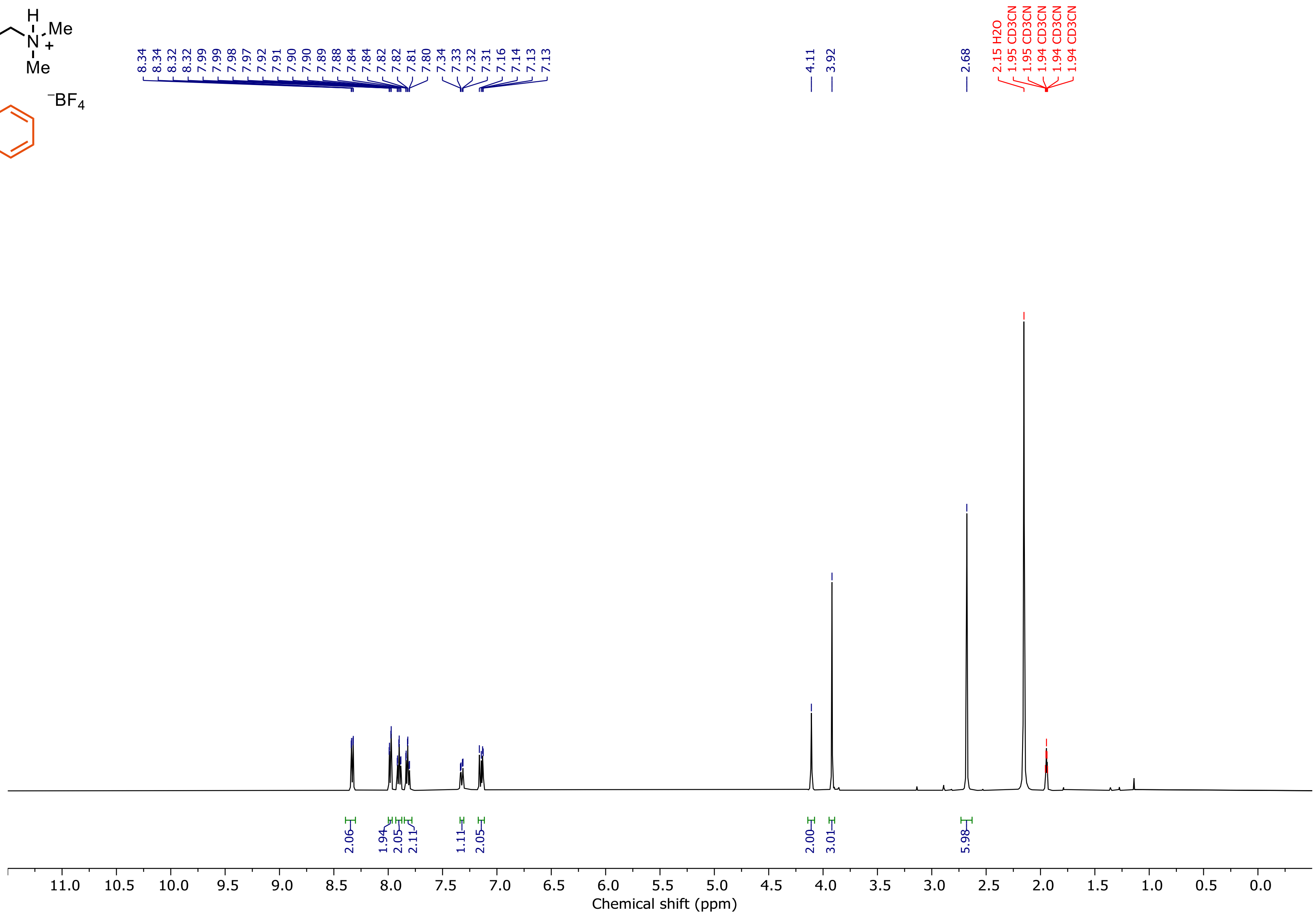

^{13}C NMR of 27a

CD_3CN, 126 MHz, 23 ºC.

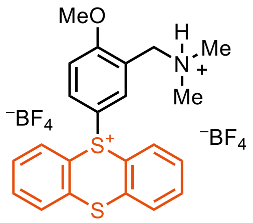

27a

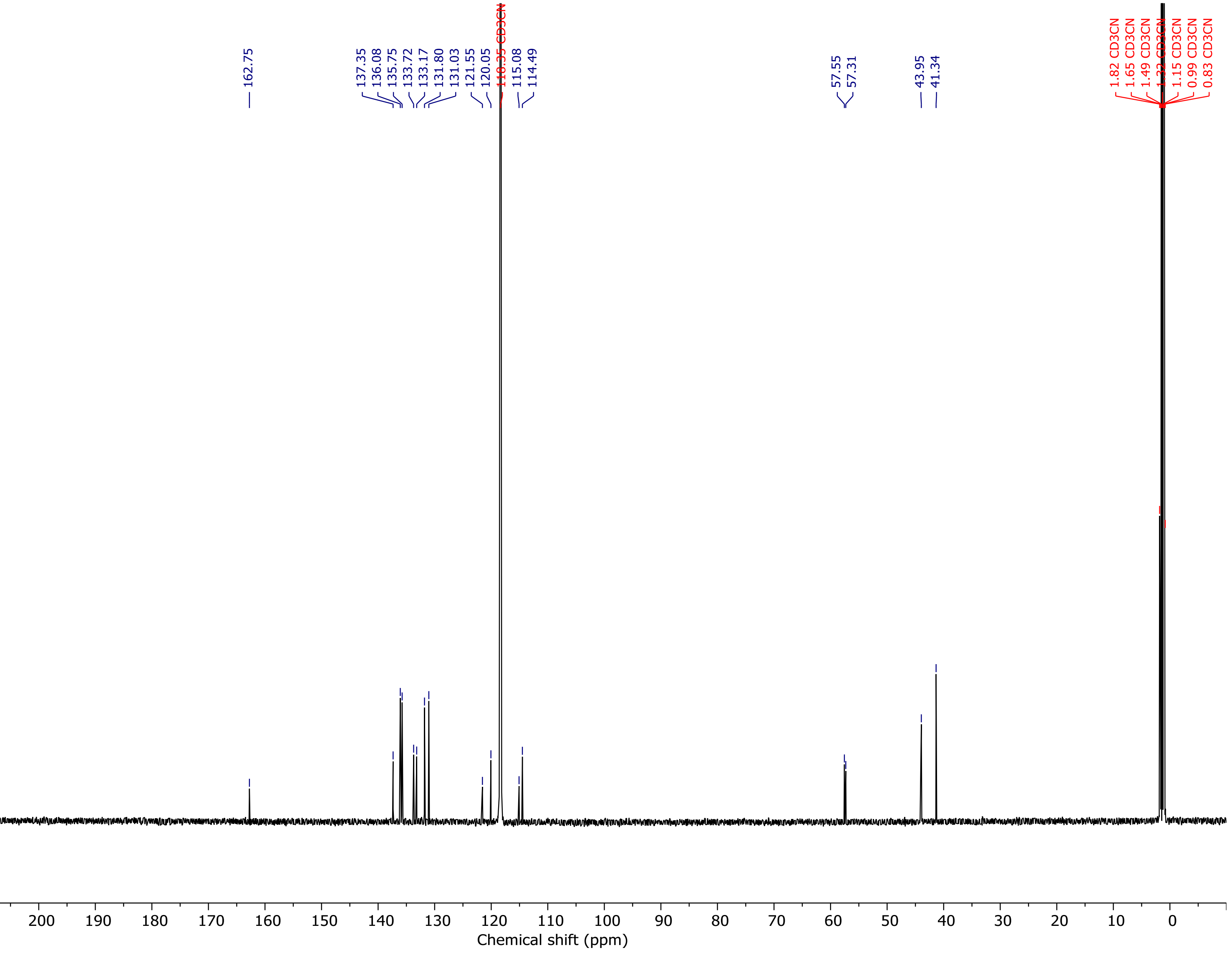

^{19}F NMR of 27a

CD_3CN, 471 MHz, 23 ºC.

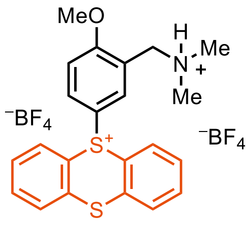

27a

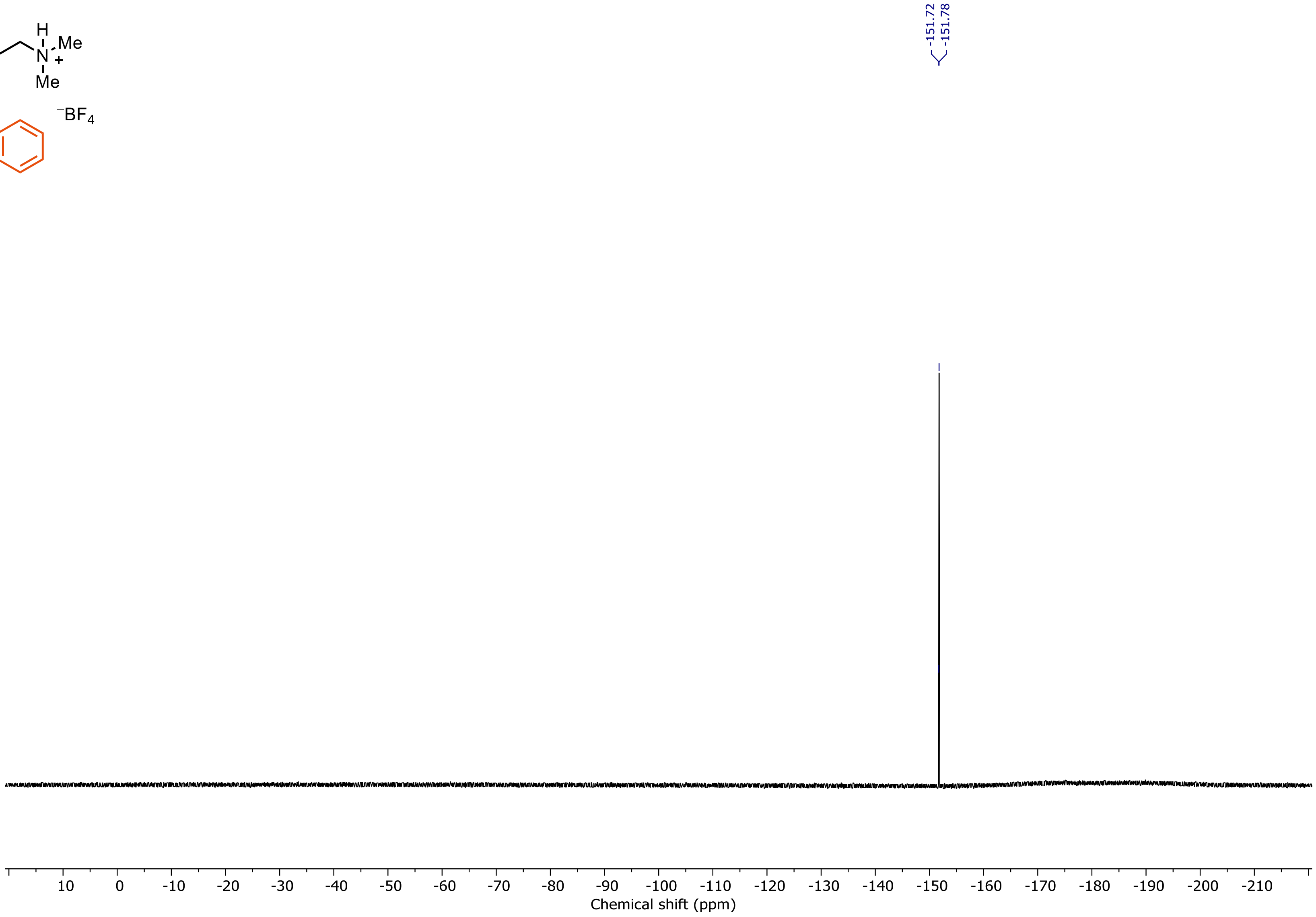

^{1}H NMR of 28

$CDCl_3$, 500 MHz, 23 ºC.

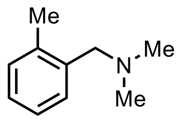

28

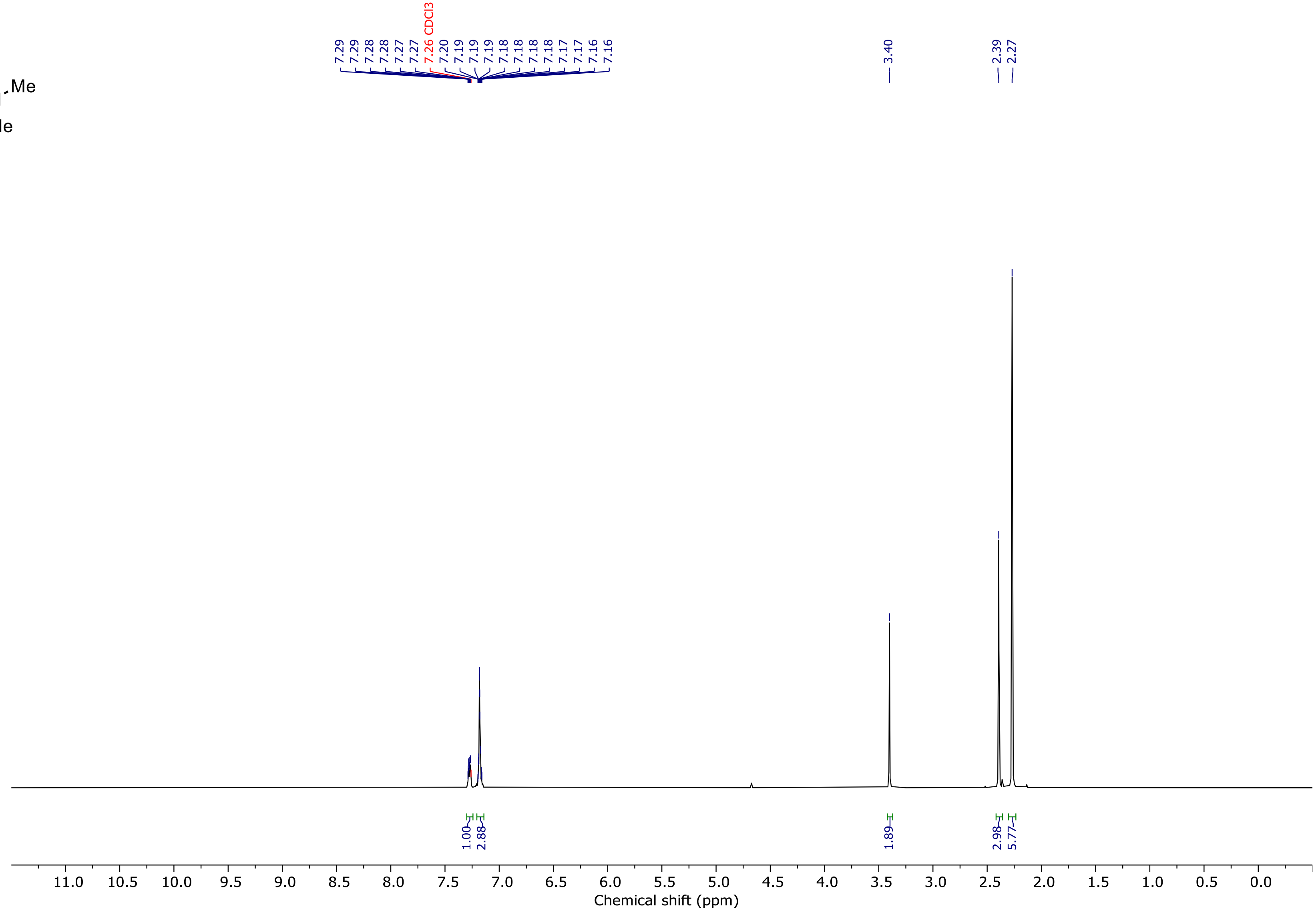

^{13}C NMR of 28

$CDCl_3$, 126 MHz, 23 ºC.

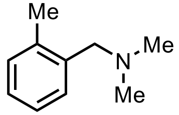

28

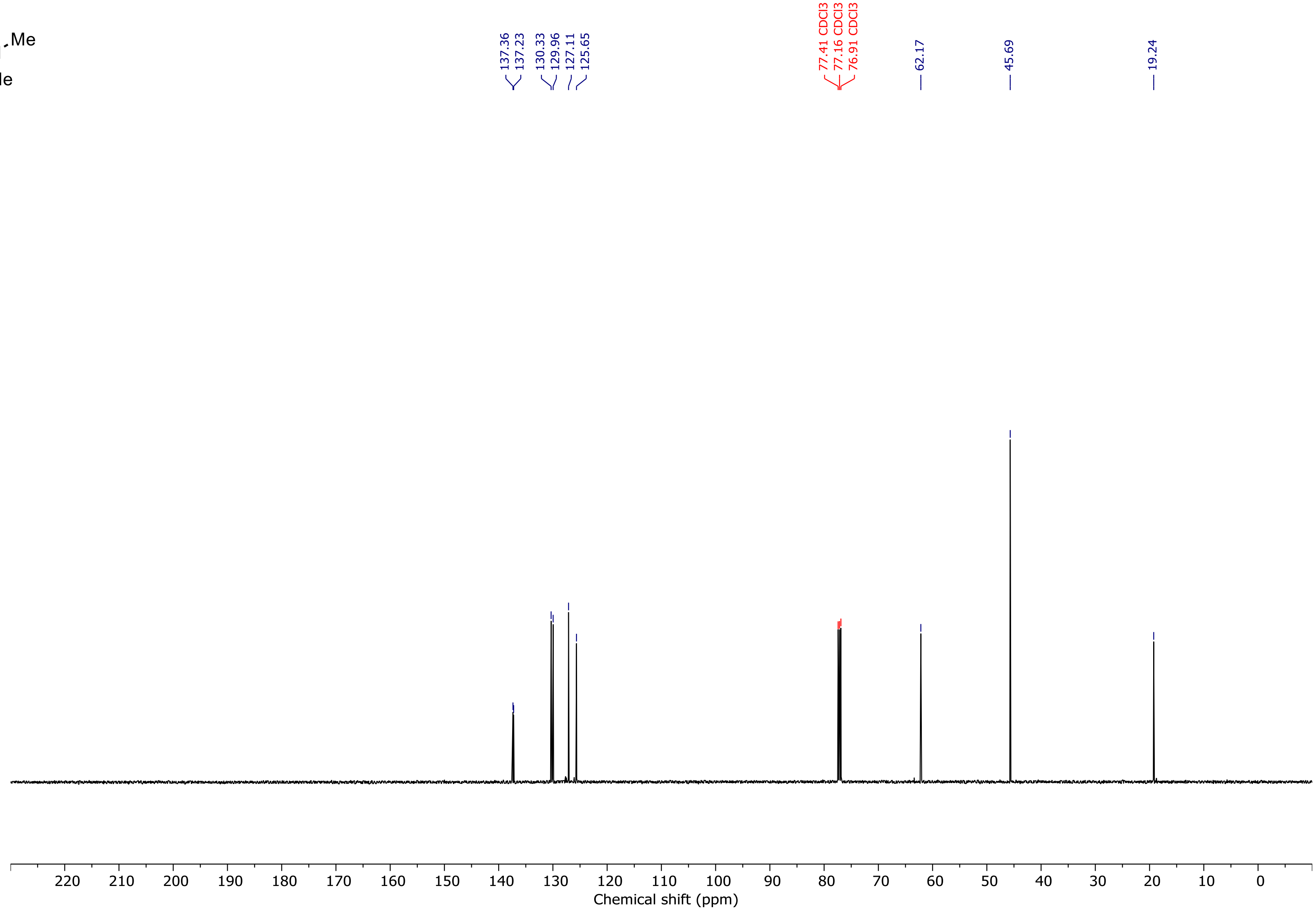

^{1}H NMR of 28a

$CDCl_3$, 500 MHz, 23 ºC.

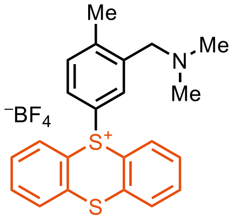

28a

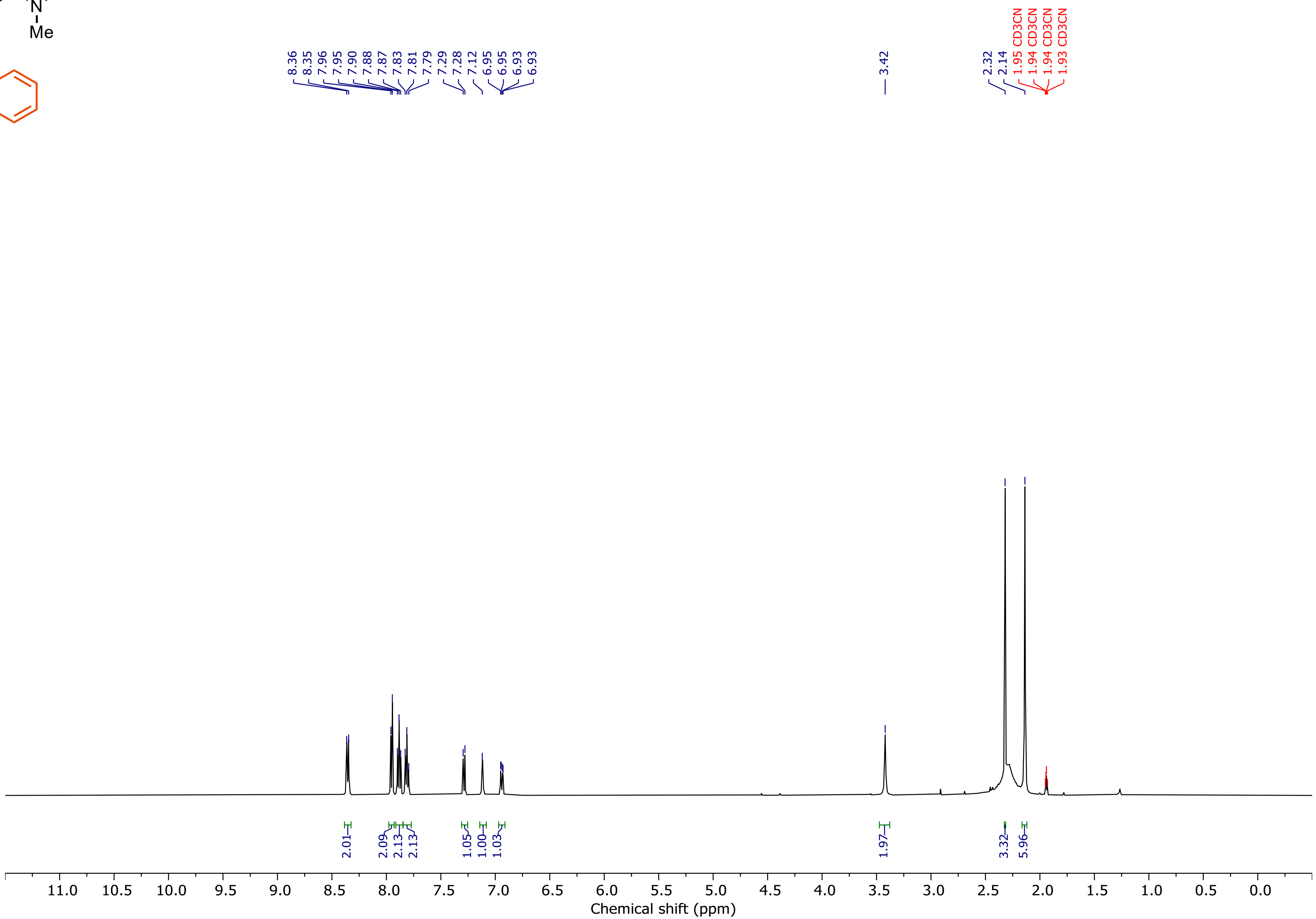

^{13}C NMR of 28a

$CDCl_3$, 126 MHz, 23 ºC.

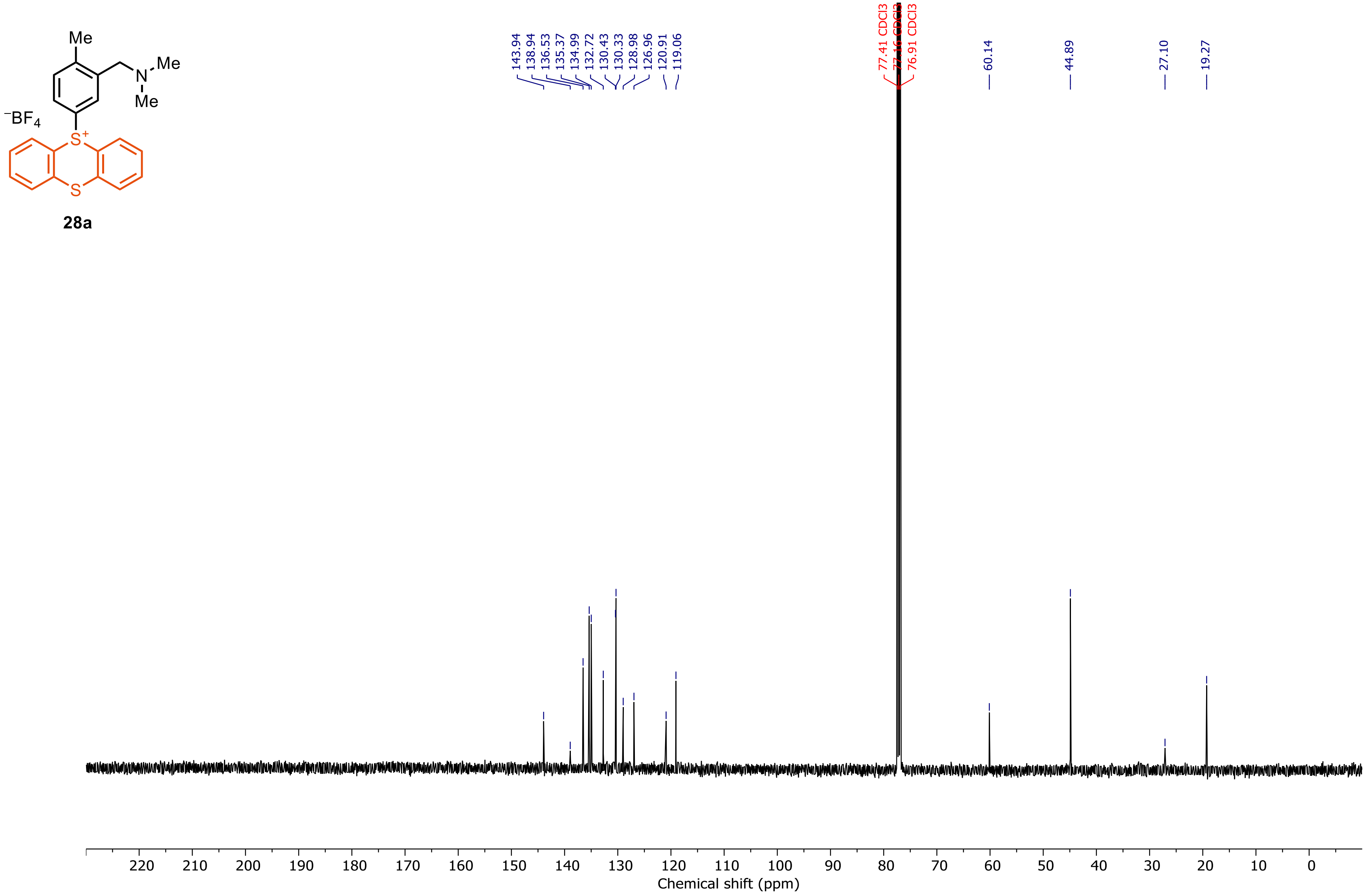

28a

^{19}F NMR of 28a

CDCl$_3$, 471 MHz, 23 ºC.

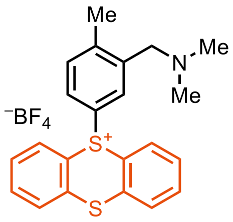

28a

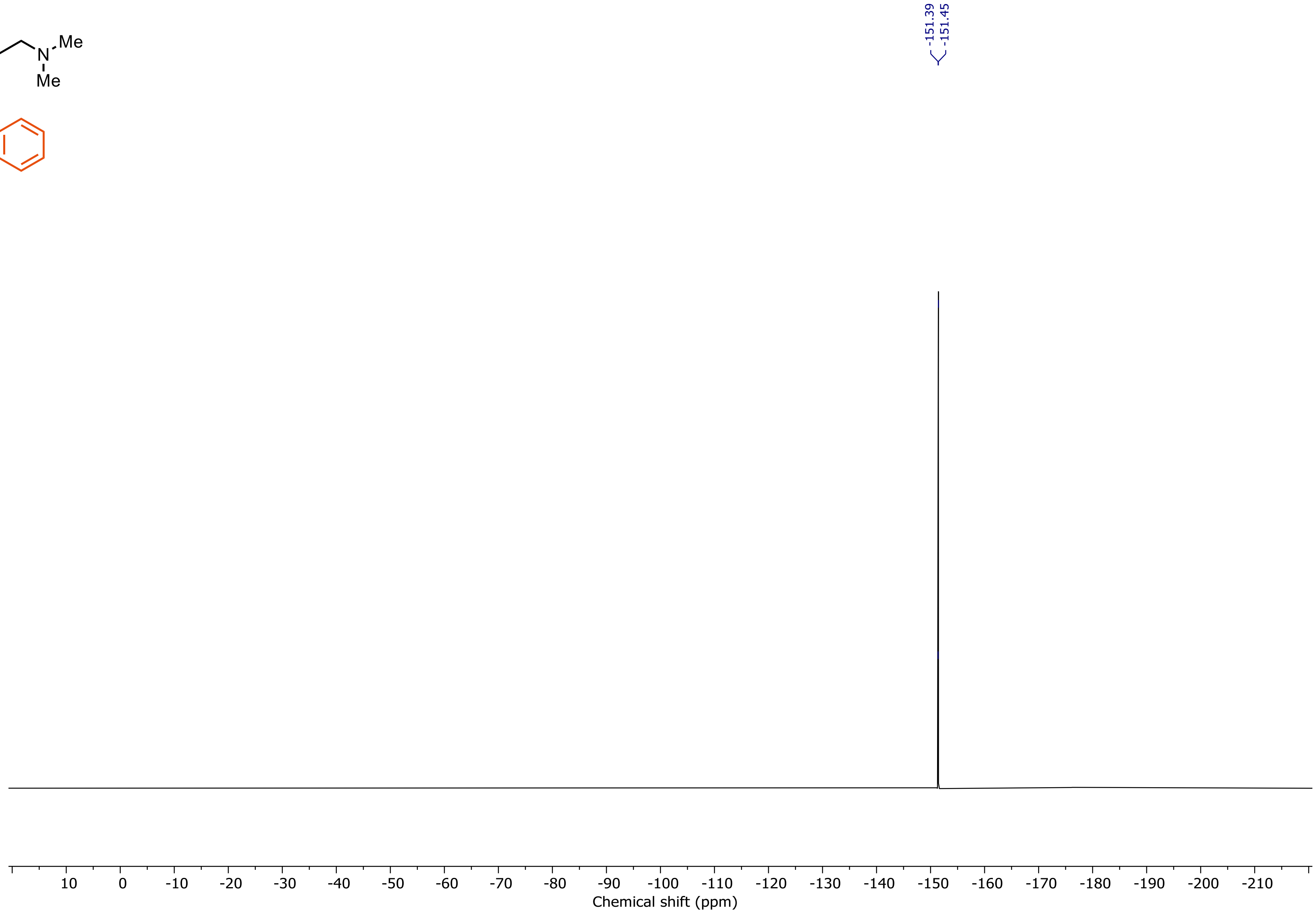

^{1}H NMR of 29

CD_3CN, 500 MHz, 23 ºC.

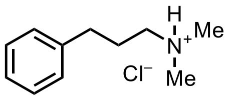

29

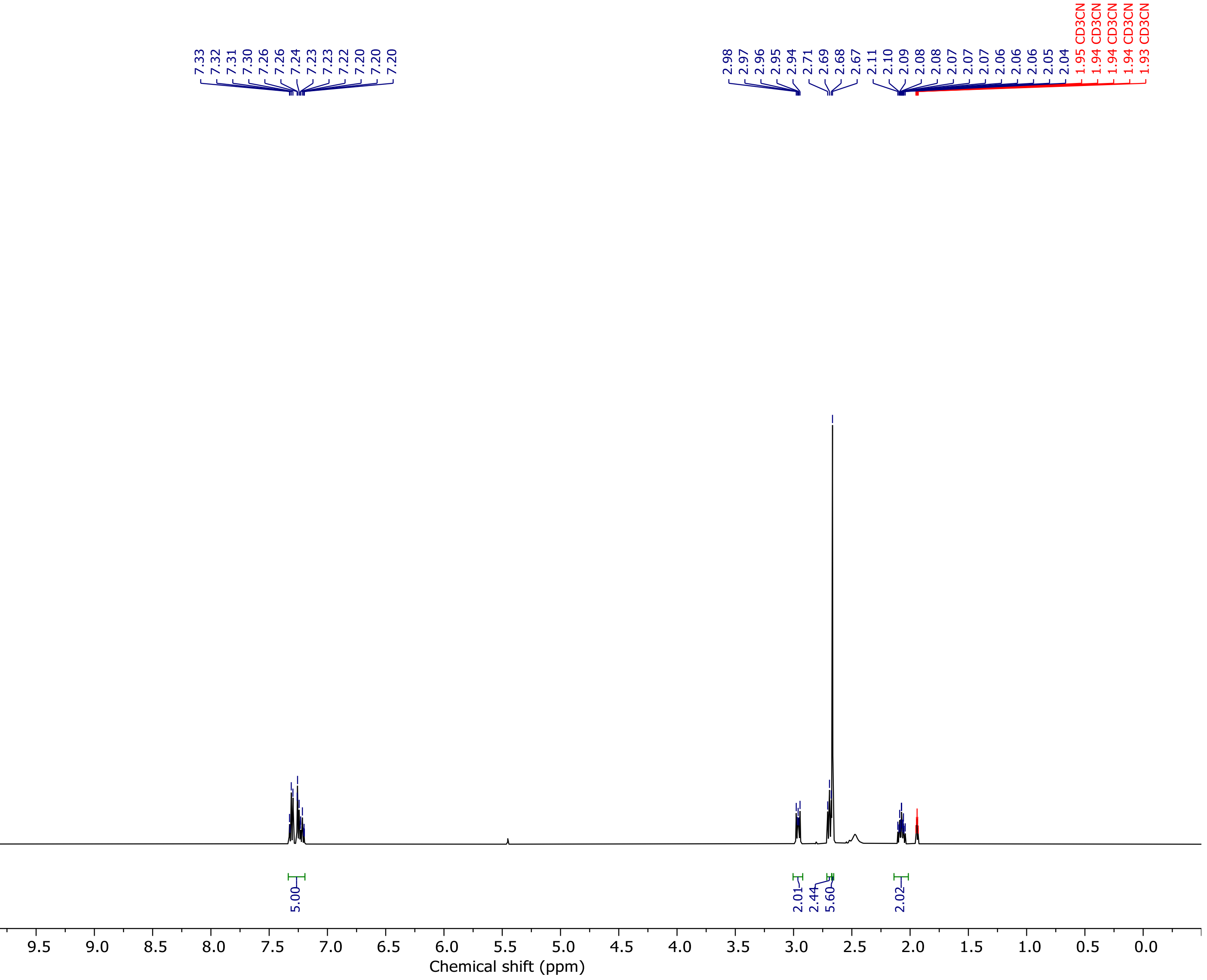

^{13}C NMR of 29

CD_3CN, 126 MHz, 23 ºC.

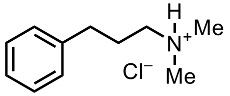

29

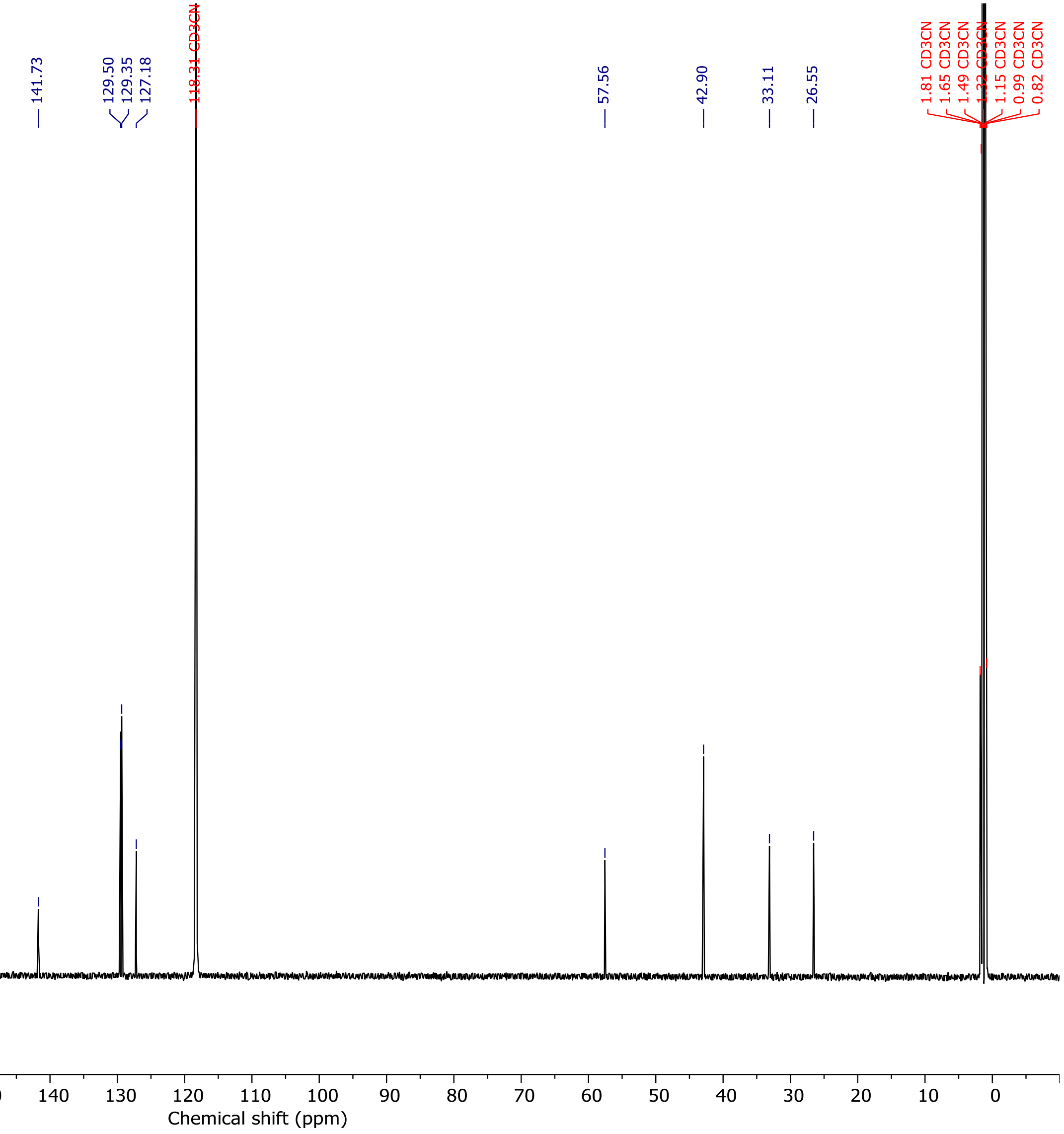

^{1}H NMR of 29a

CD_3CN, 500 MHz, 23 ºC.

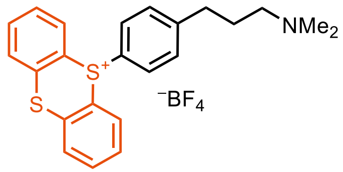

29a

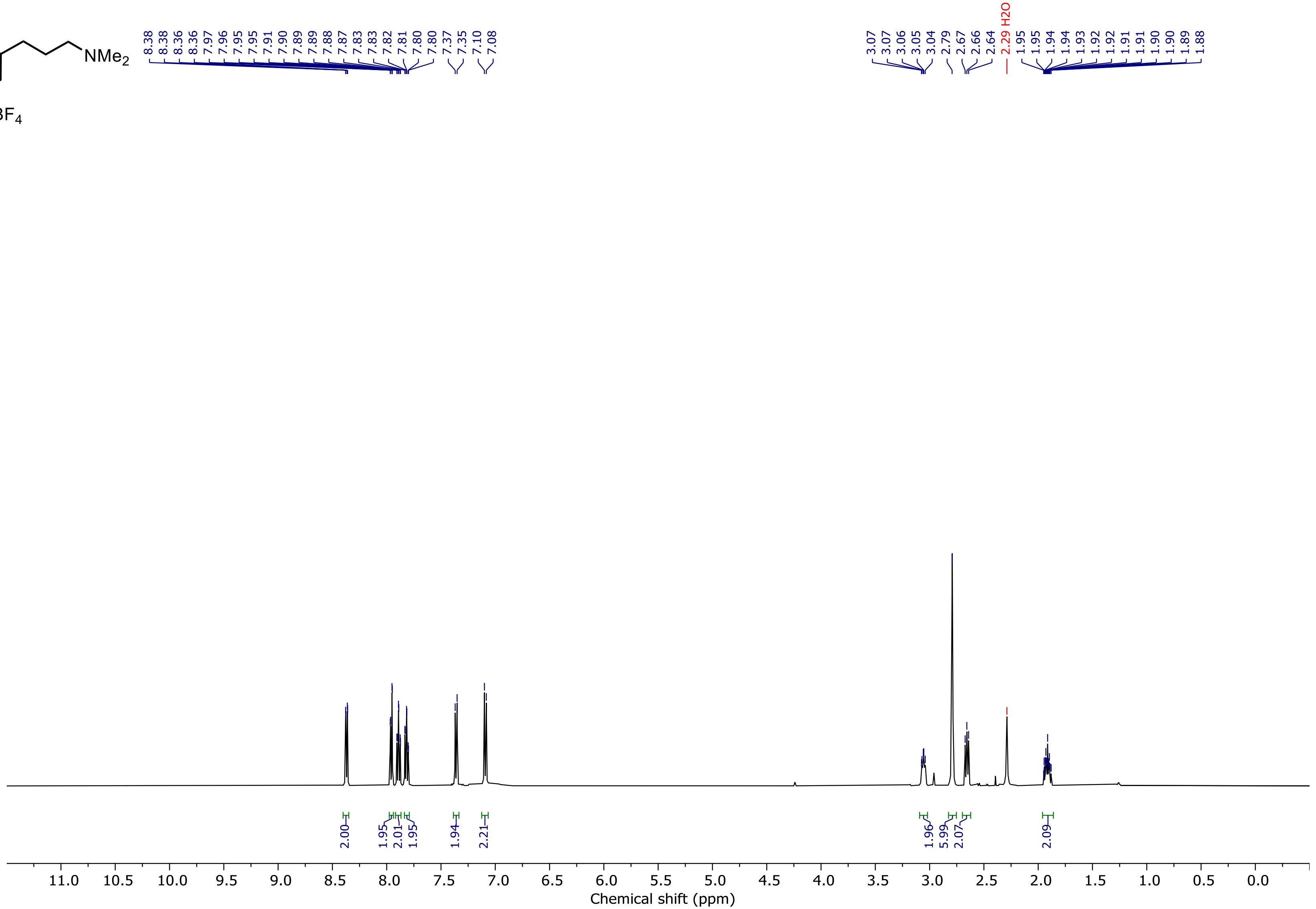

^{13}C NMR of 29a

CD_3CN, 126 MHz, 23 ºC.

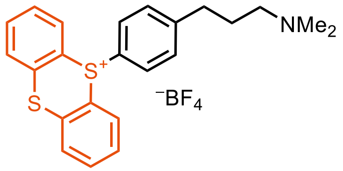

29a

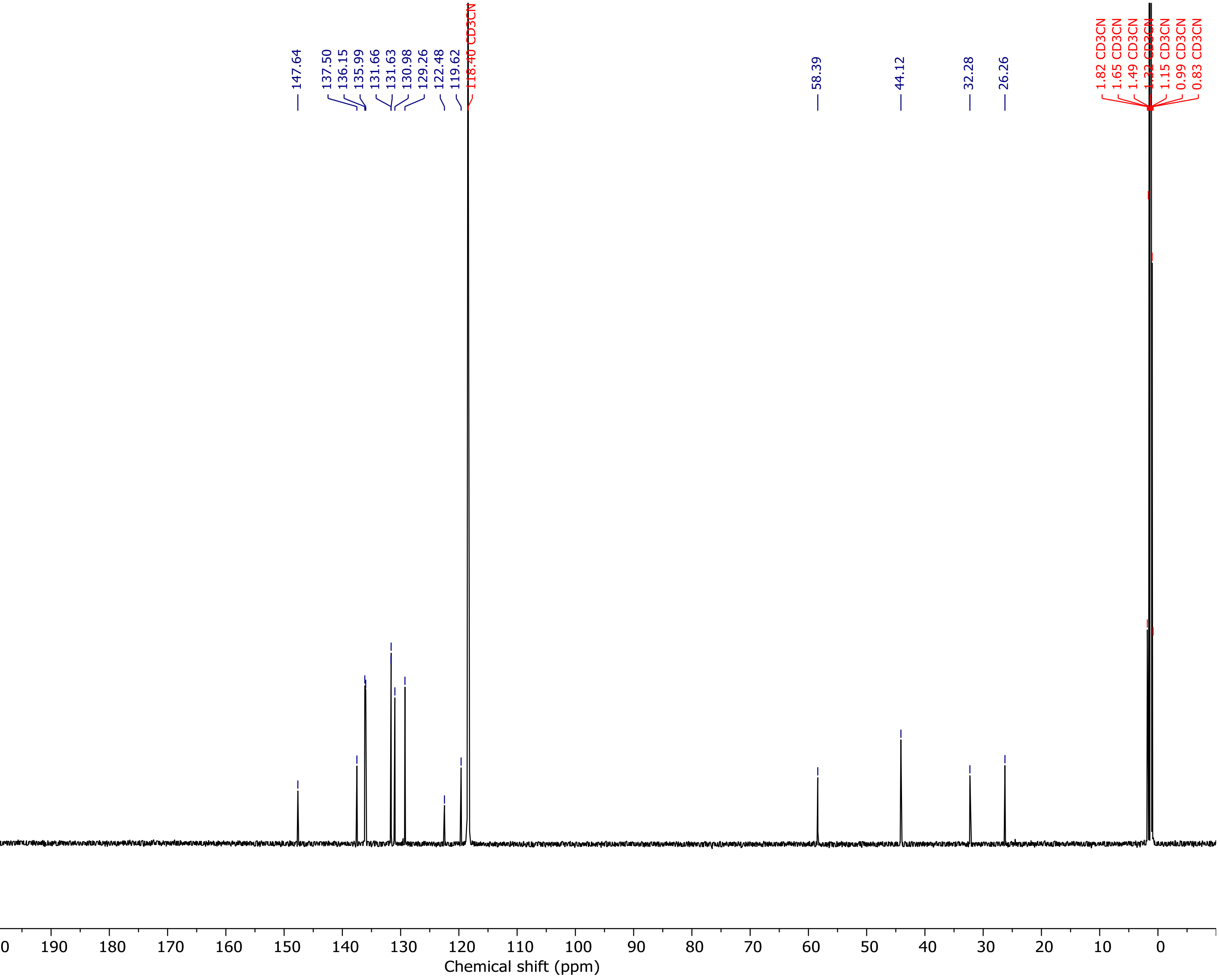

^{19}F NMR of 29a

CD_3CN, 471 MHz, 23 ºC.

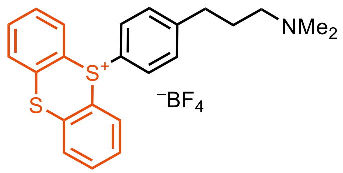

29a

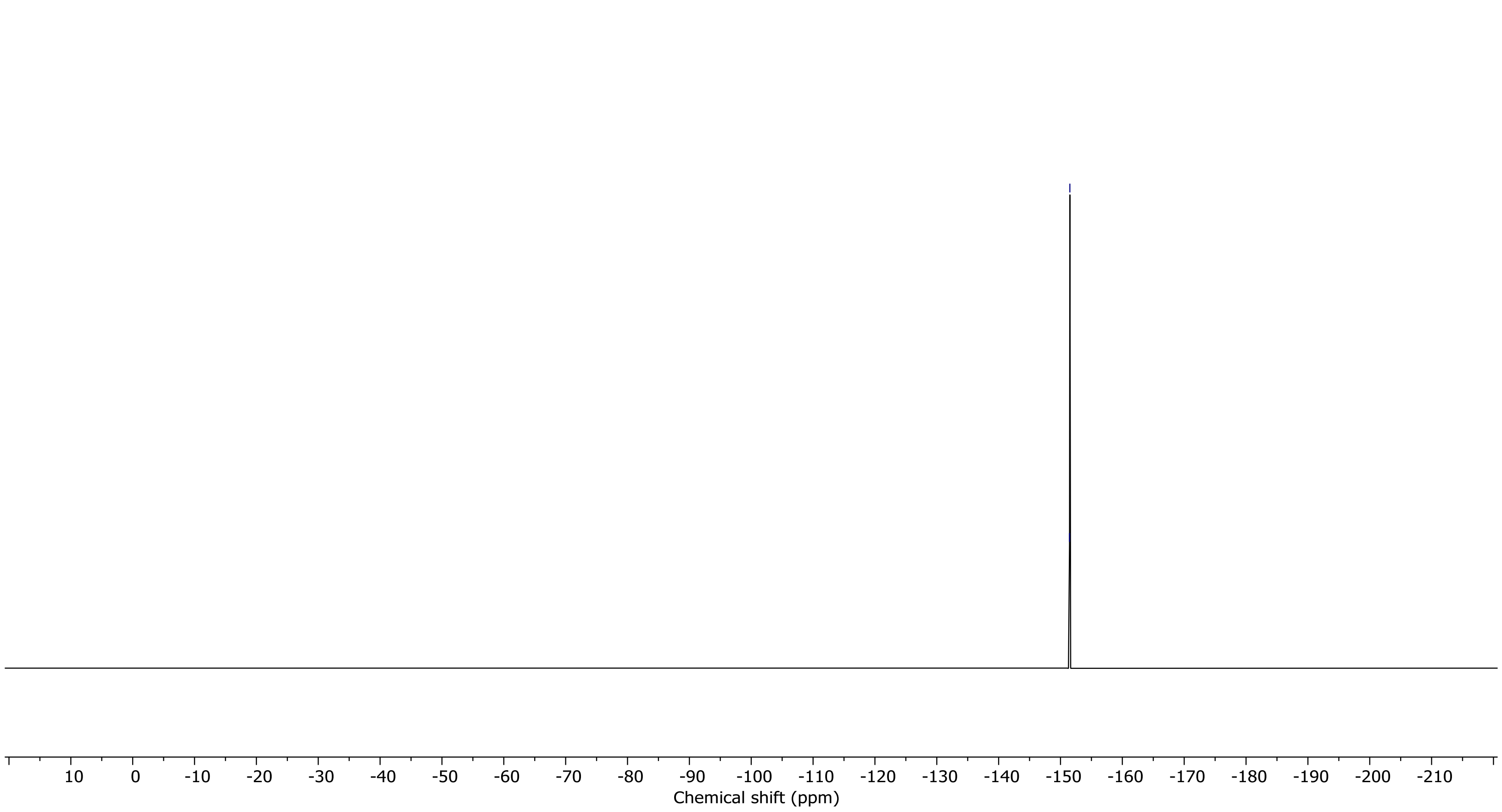

^{1}H NMR of 30

$CDCl_3$, 500 MHz, 23 ºC.

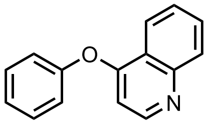

30

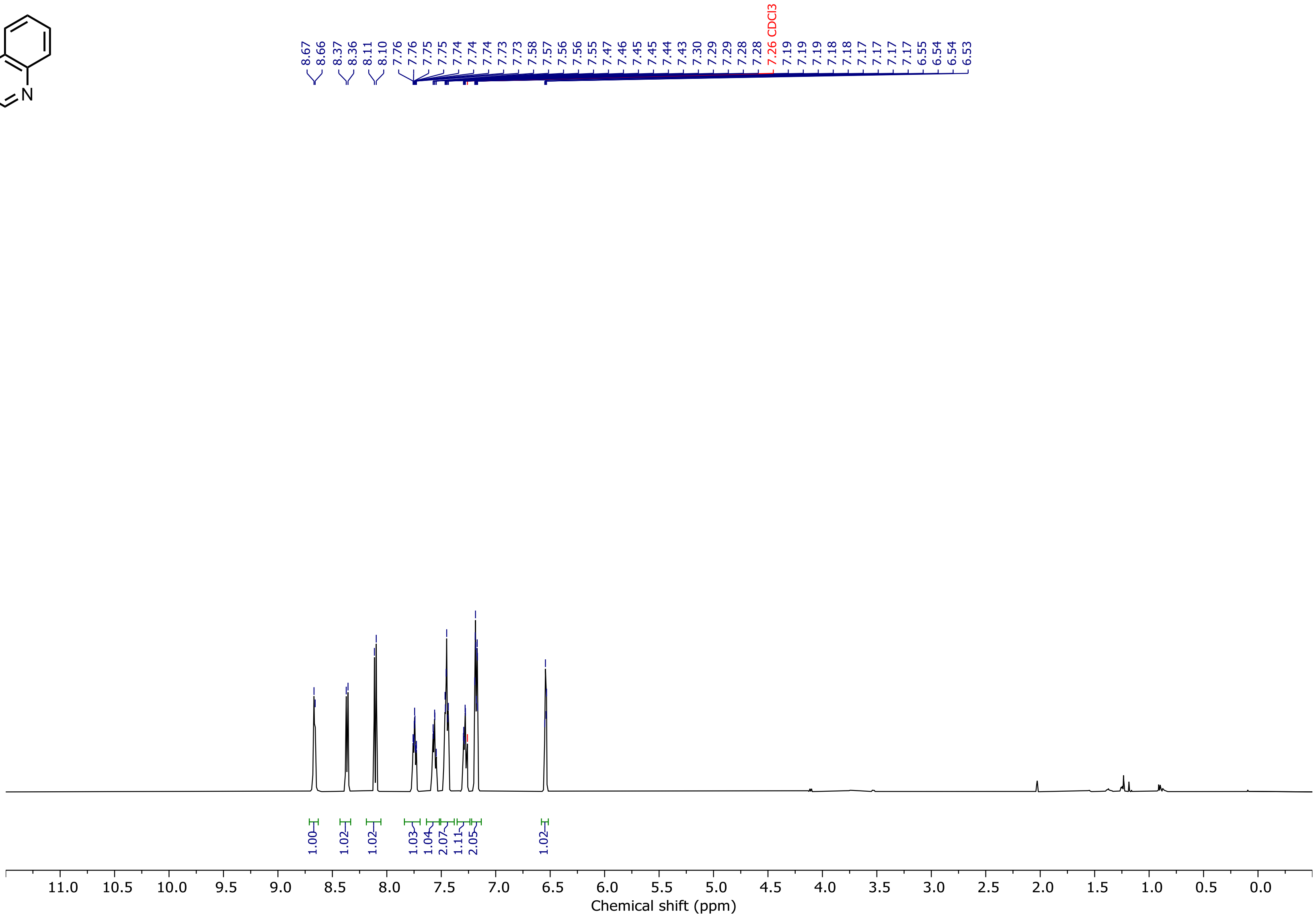

^{13}C NMR of 30

$CDCl_3$, 126 MHz, 23 ºC.

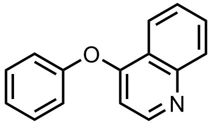

30

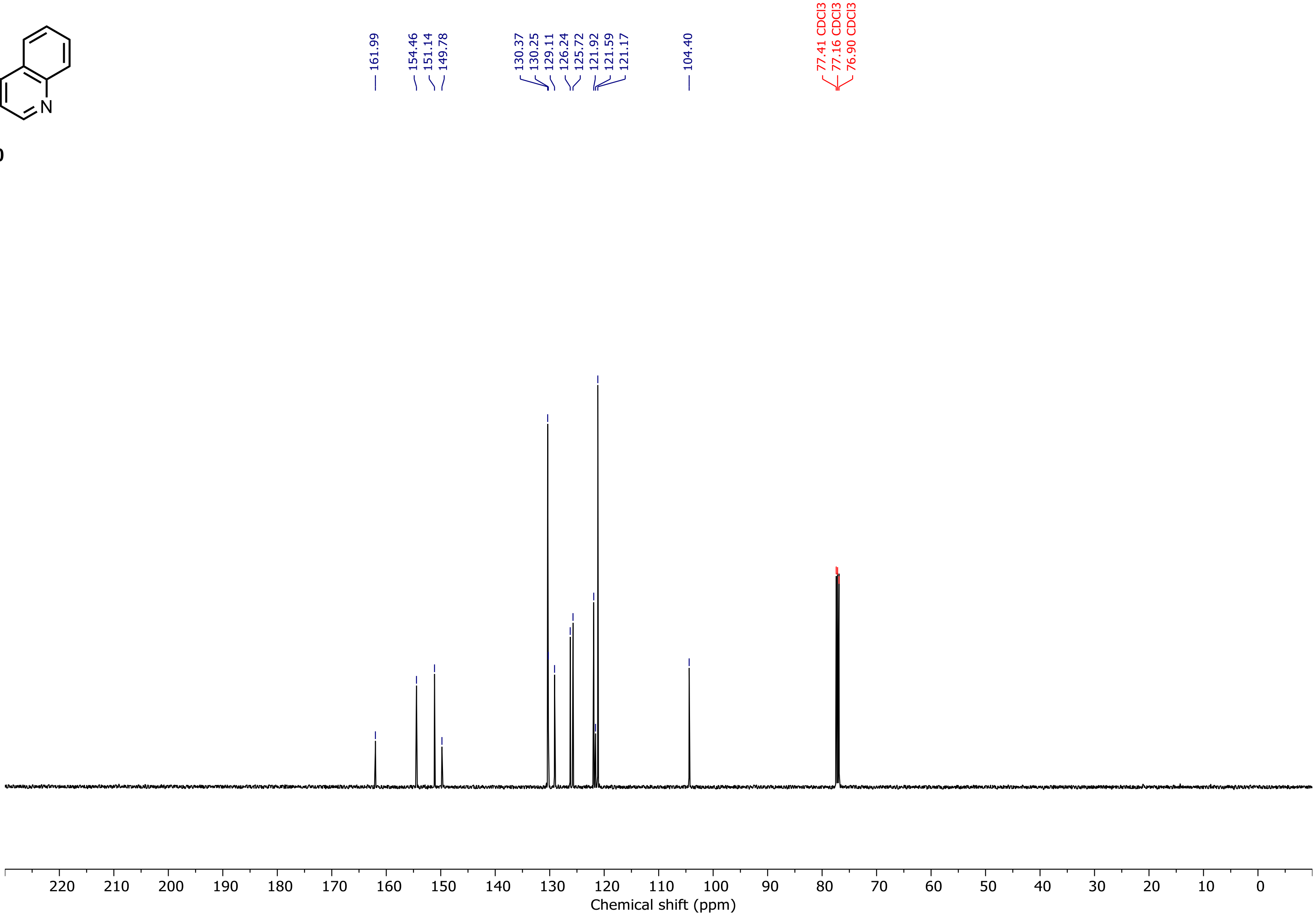

^{1}H NMR of 30a

CDCl$_3$, 600 MHz, 23 ºC.

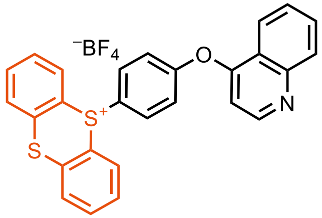

30a

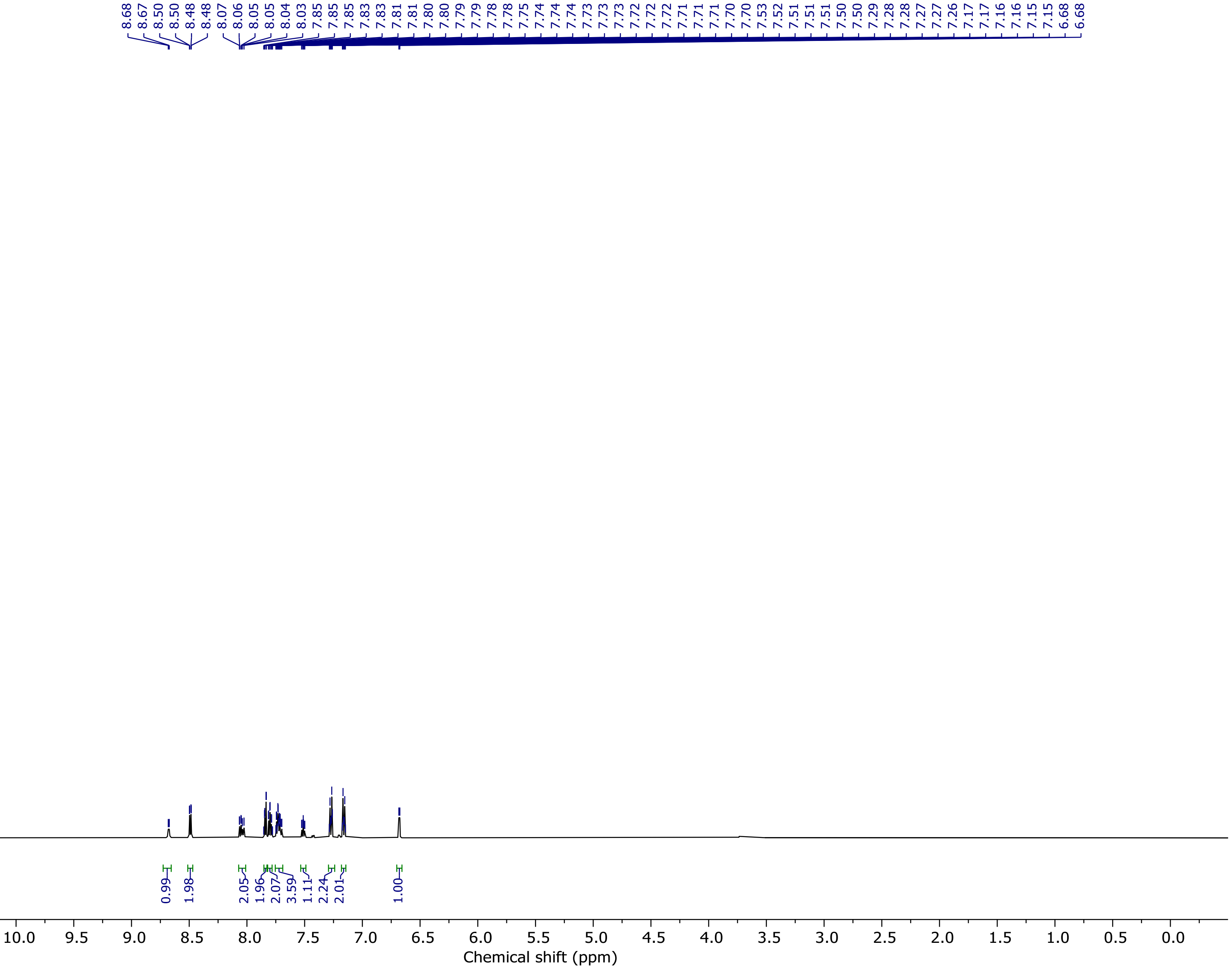

^{13}C NMR of 30a

$CDCl_3$, 151 MHz, 23 ºC.

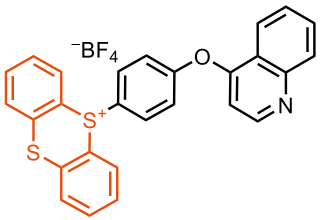

30a

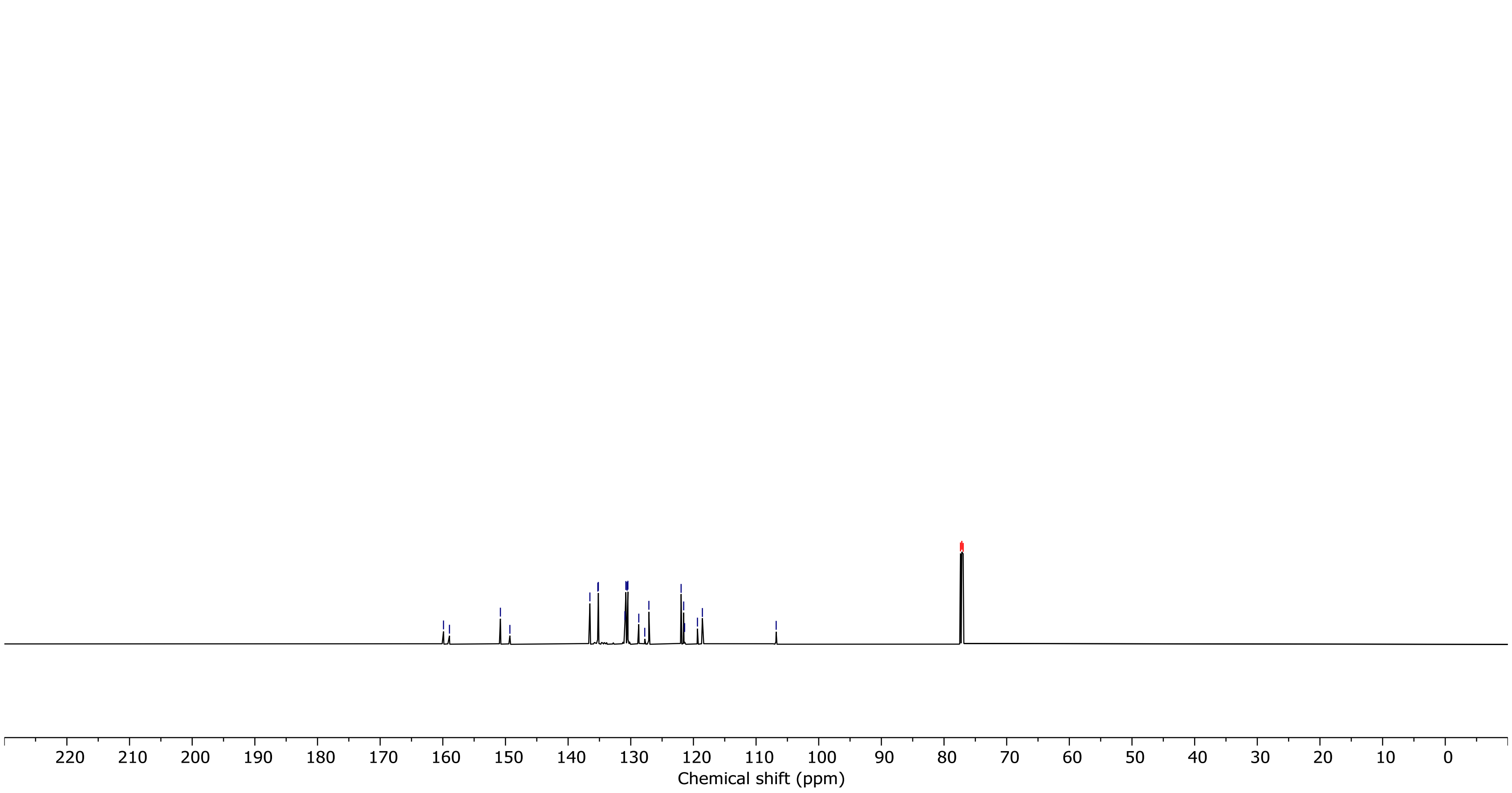

^{19}F NMR of 30a

$CDCl_3$, 565 MHz, 23 ºC.

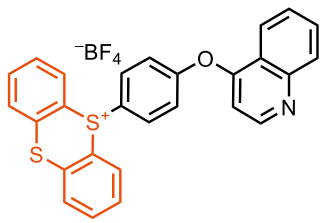

30a

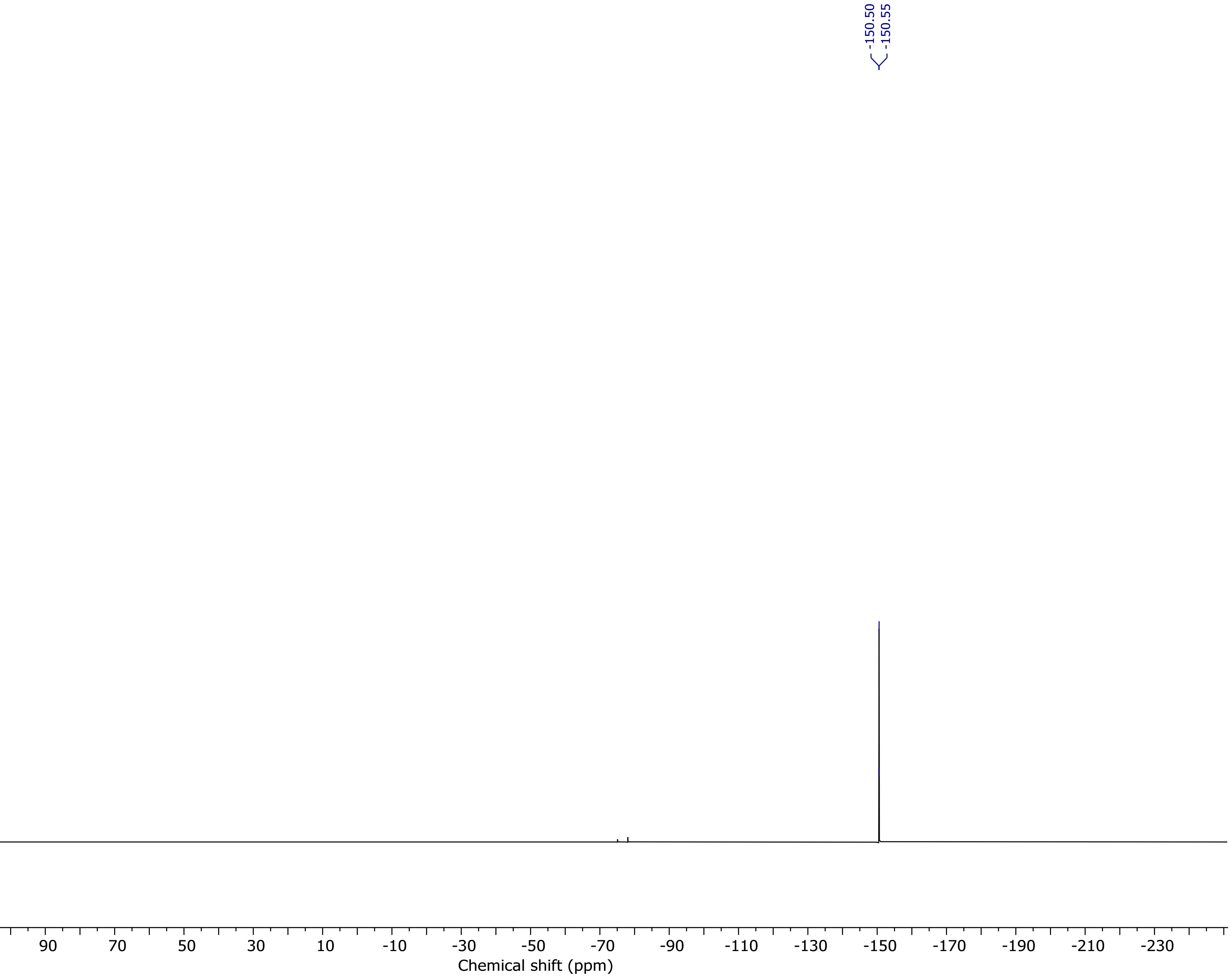

^{1}H NMR of 31a

$(CD_3)_2SO$, 500 MHz, 23 ºC.

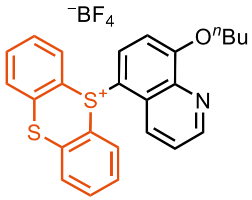

31a

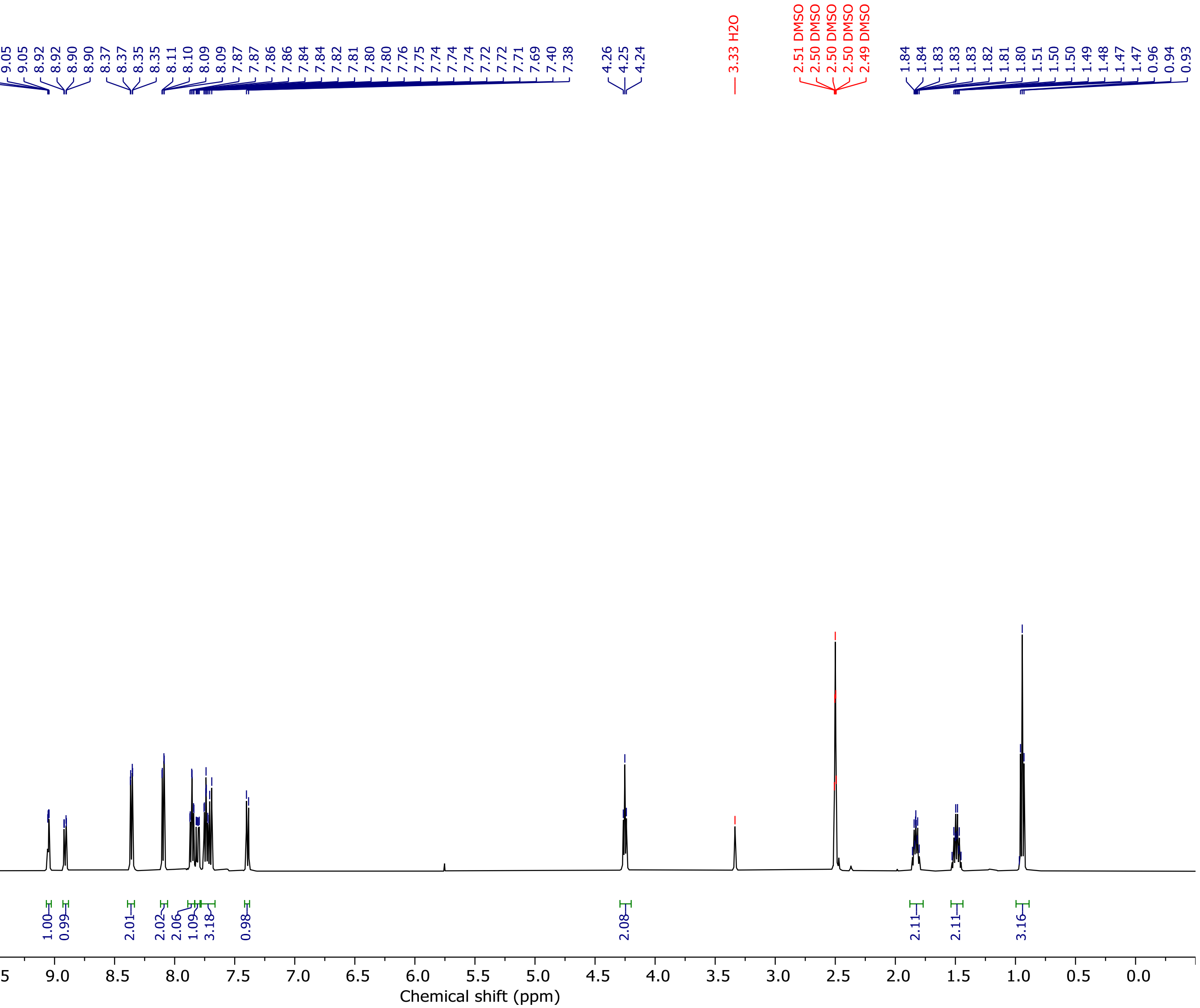

^{13}C NMR of 31a

$(CD_3)_2SO$, 126 MHz, 23 °C.

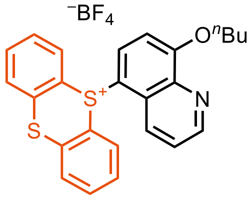

31a

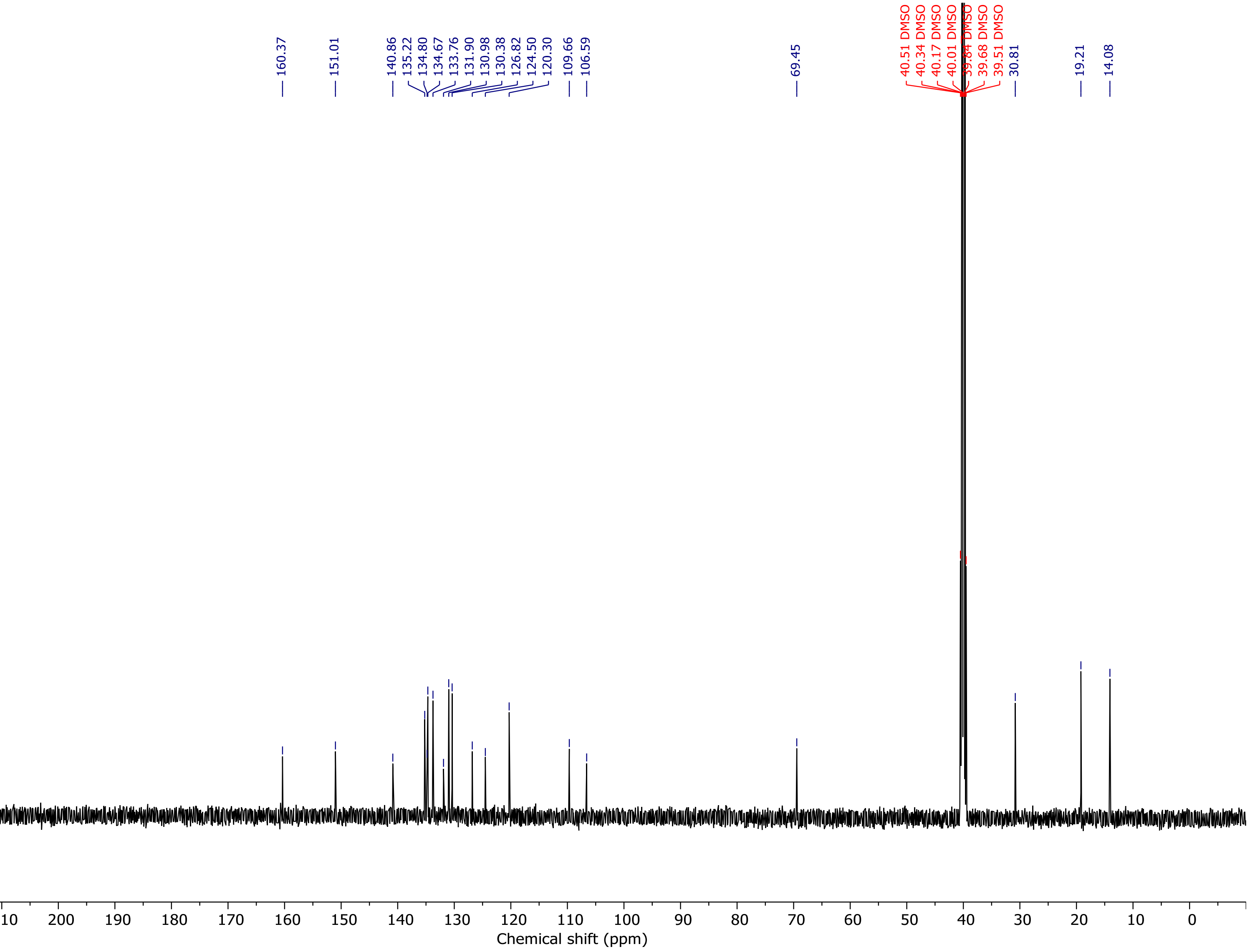

^{19}F NMR of 31a

$(CD_3)_2SO$, 470 MHz, 23 ºC.

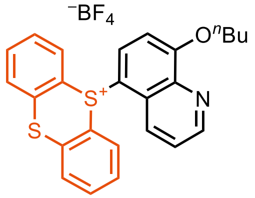

31a

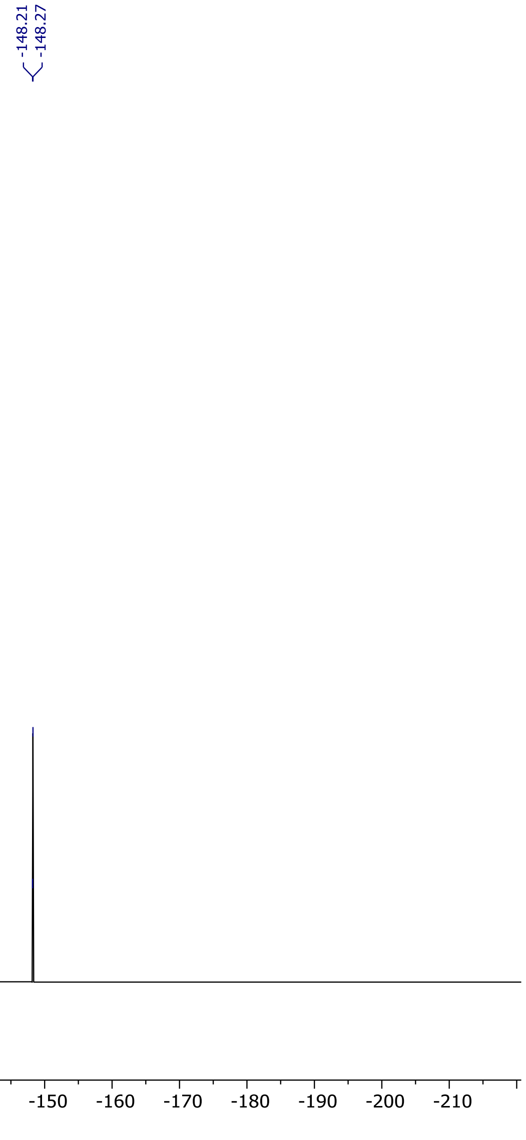

^{1}H NMR of 32a

CD_3CN, 500 MHz, 23 ºC.

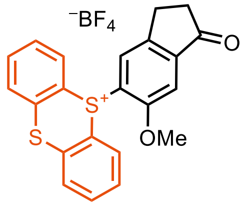

32a

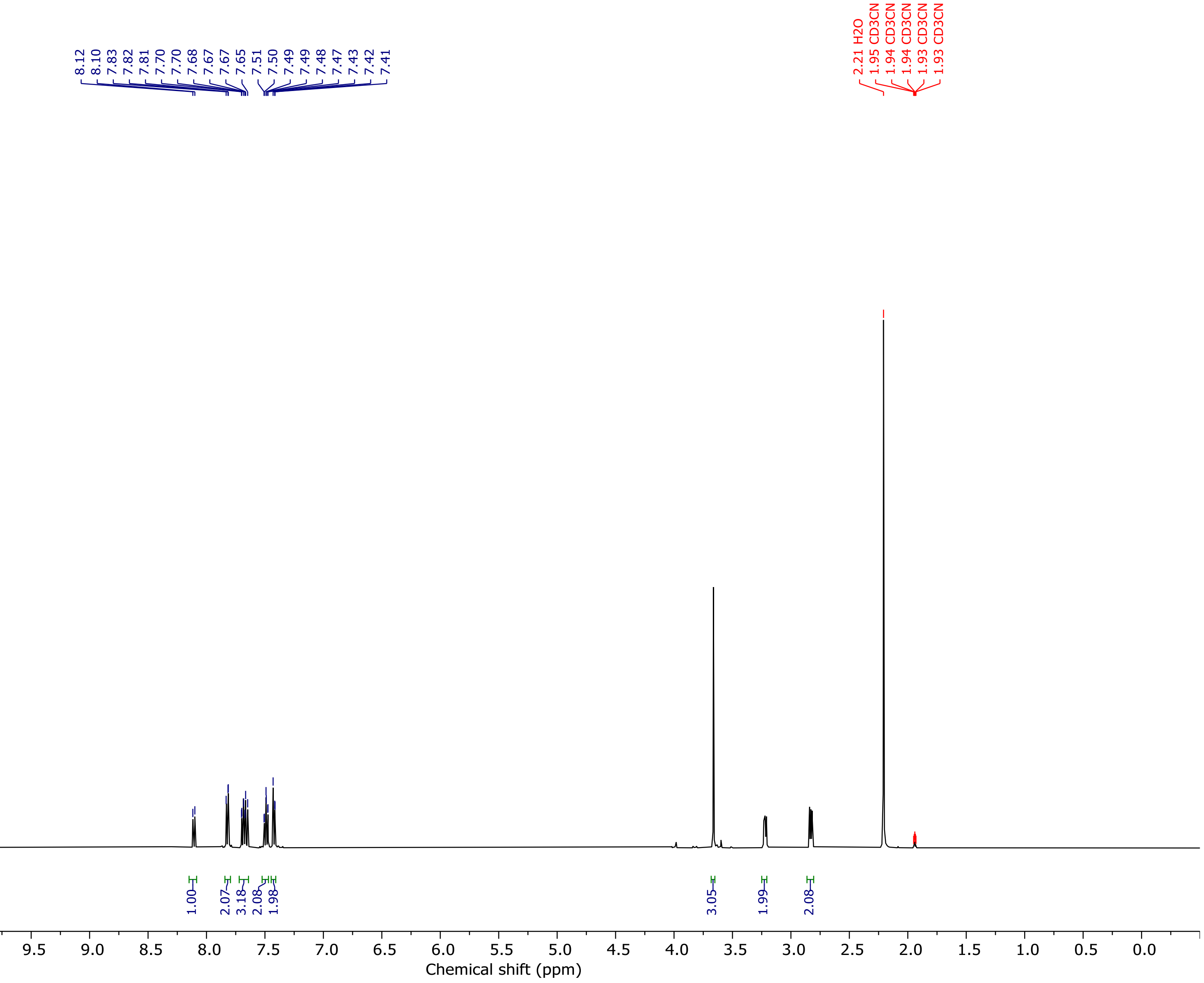

^{13}C NMR of 32a

CD_3CN, 126 MHz, 23 ºC.

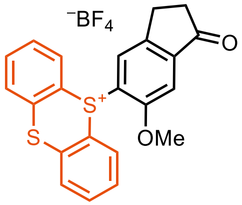

32a

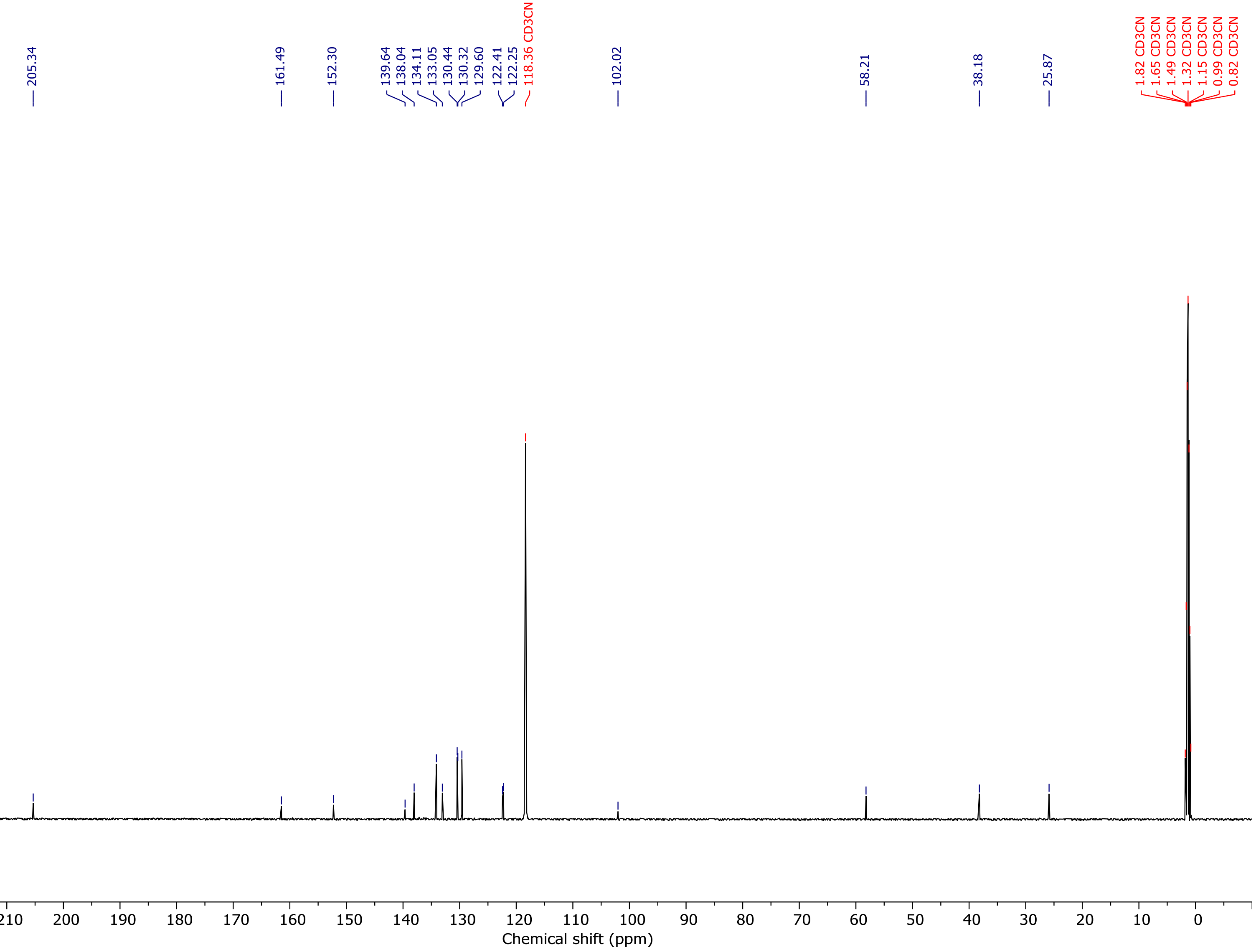

^{19}F NMR of 32a

CD_3CN, 470 MHz, 23 ºC.

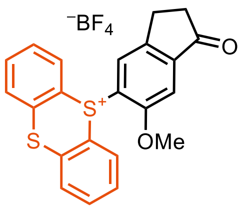

32a

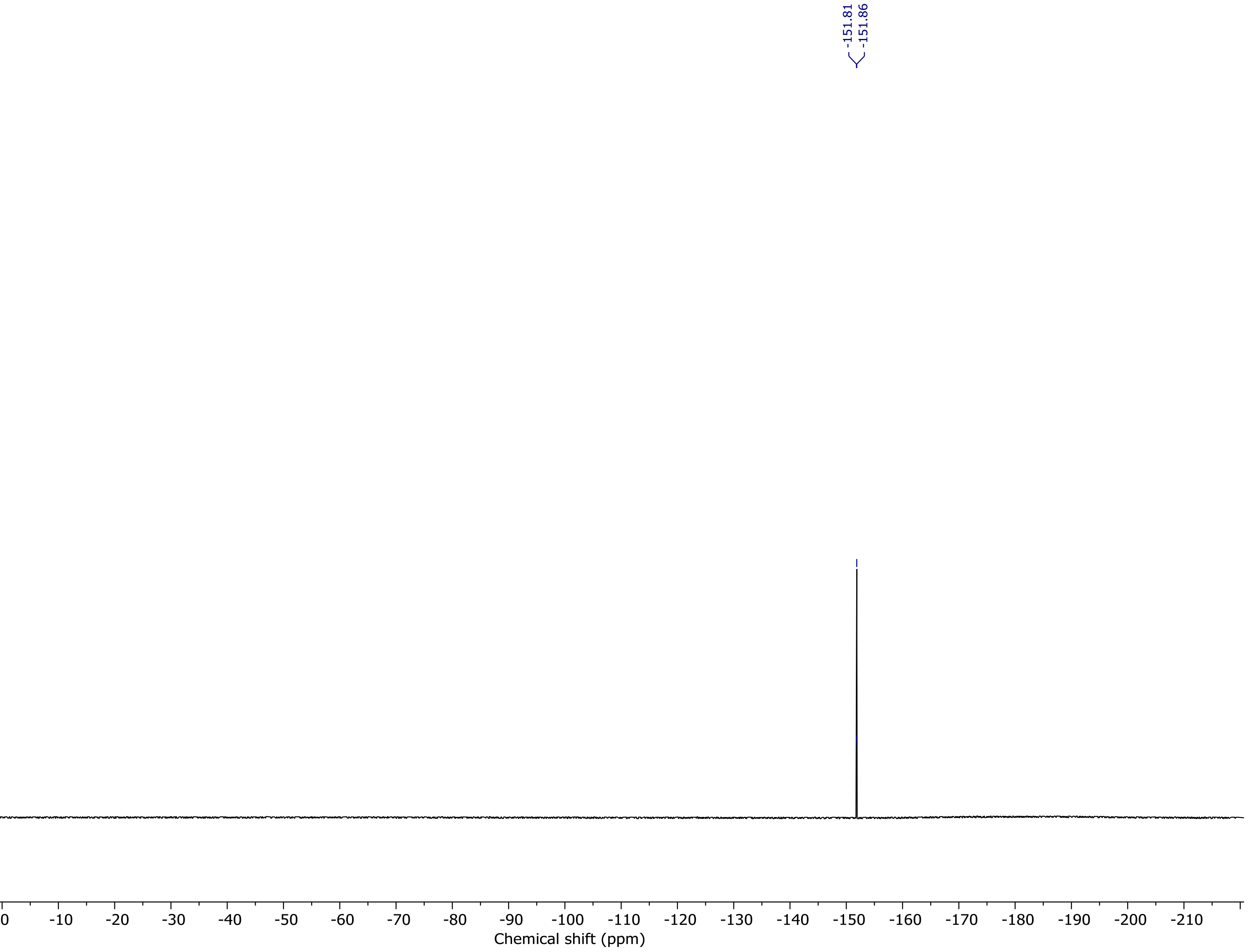

^{1}H NMR of 33a

CD_3CN, 600 MHz, 23 ºC.

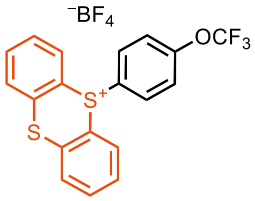

33a

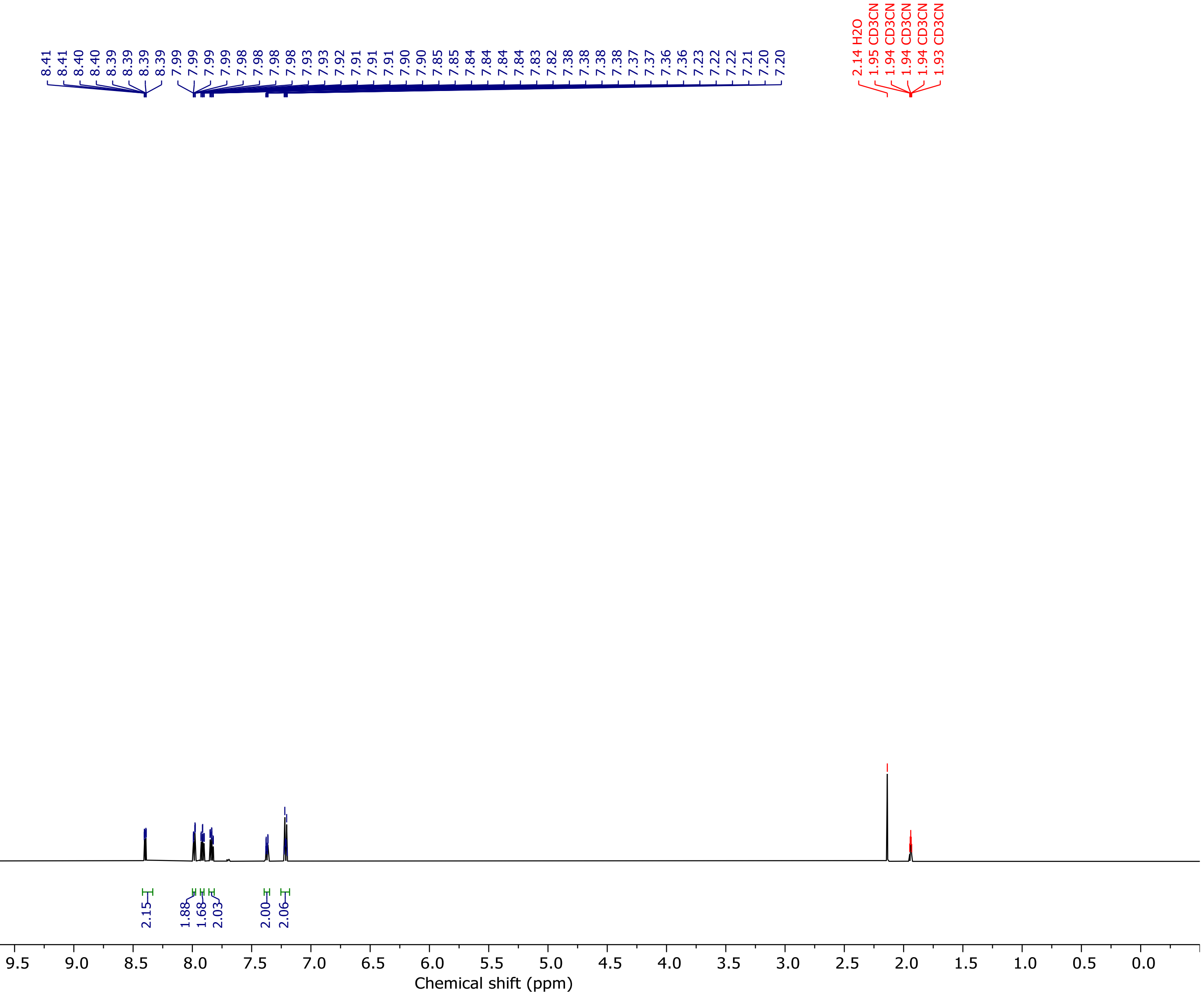

^{13}C NMR of 33a

CD_3CN, 151 MHz, 23 ºC.

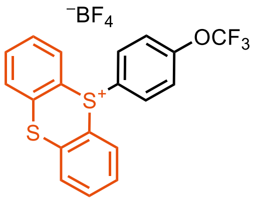

33a

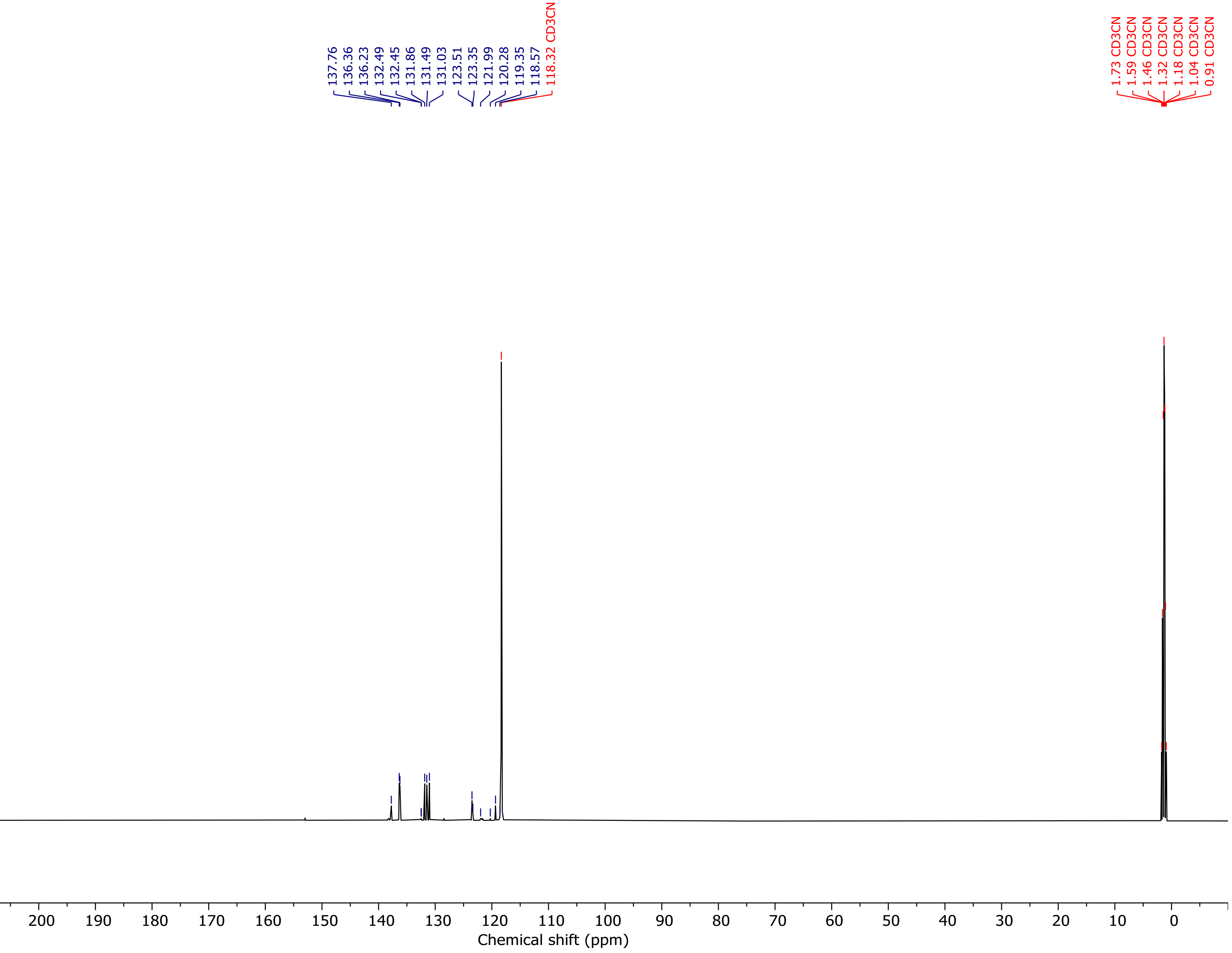

^{19}F NMR of 33a

CD_3CN, 565 MHz, 23 ºC.

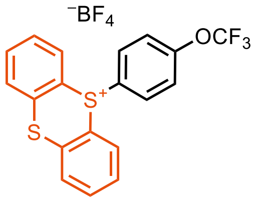

33a

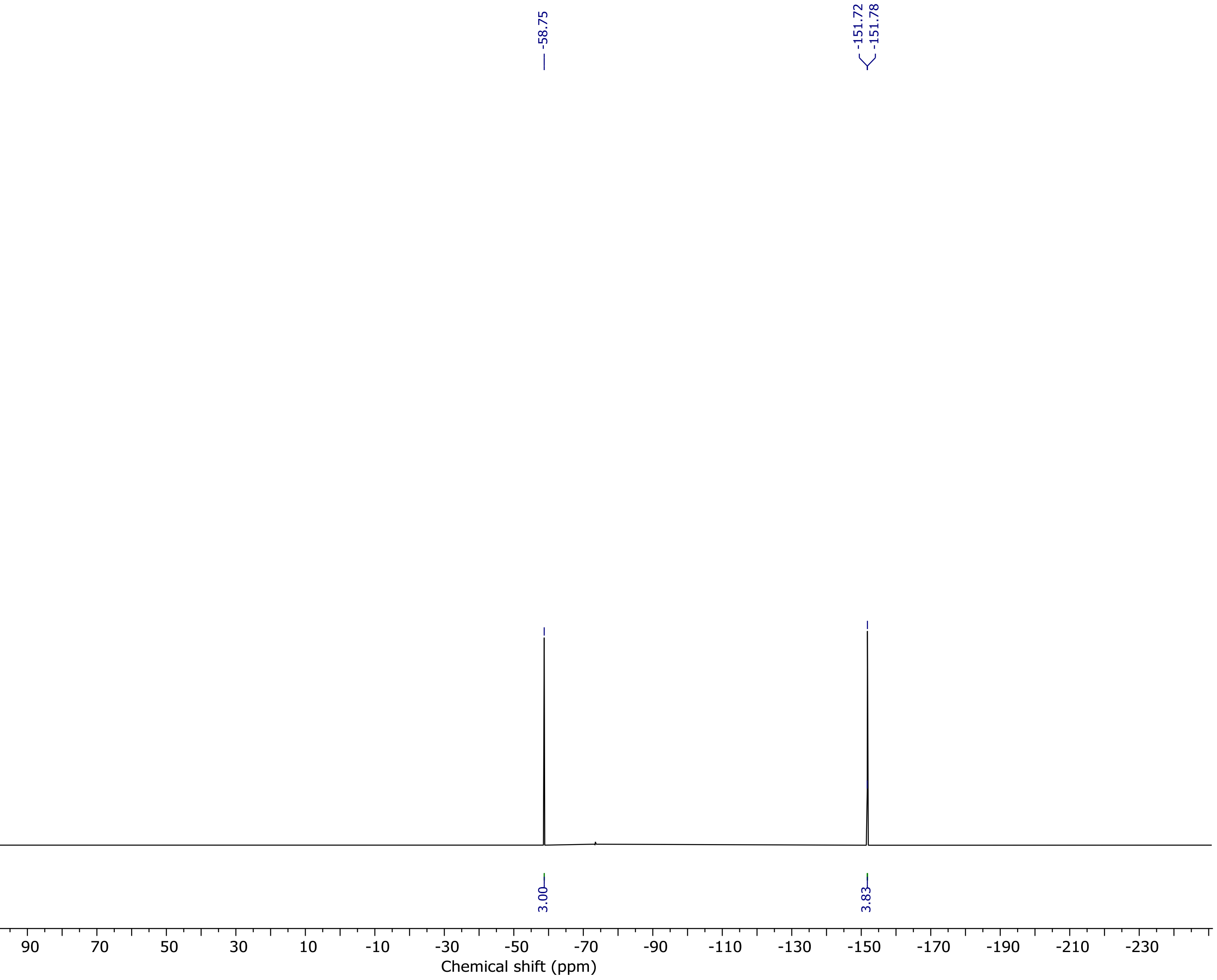

^{1}H NMR of 34a

CD_3CN, 300 MHz, 23 °C

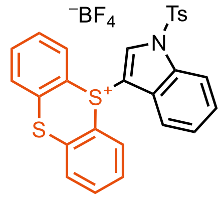

34a

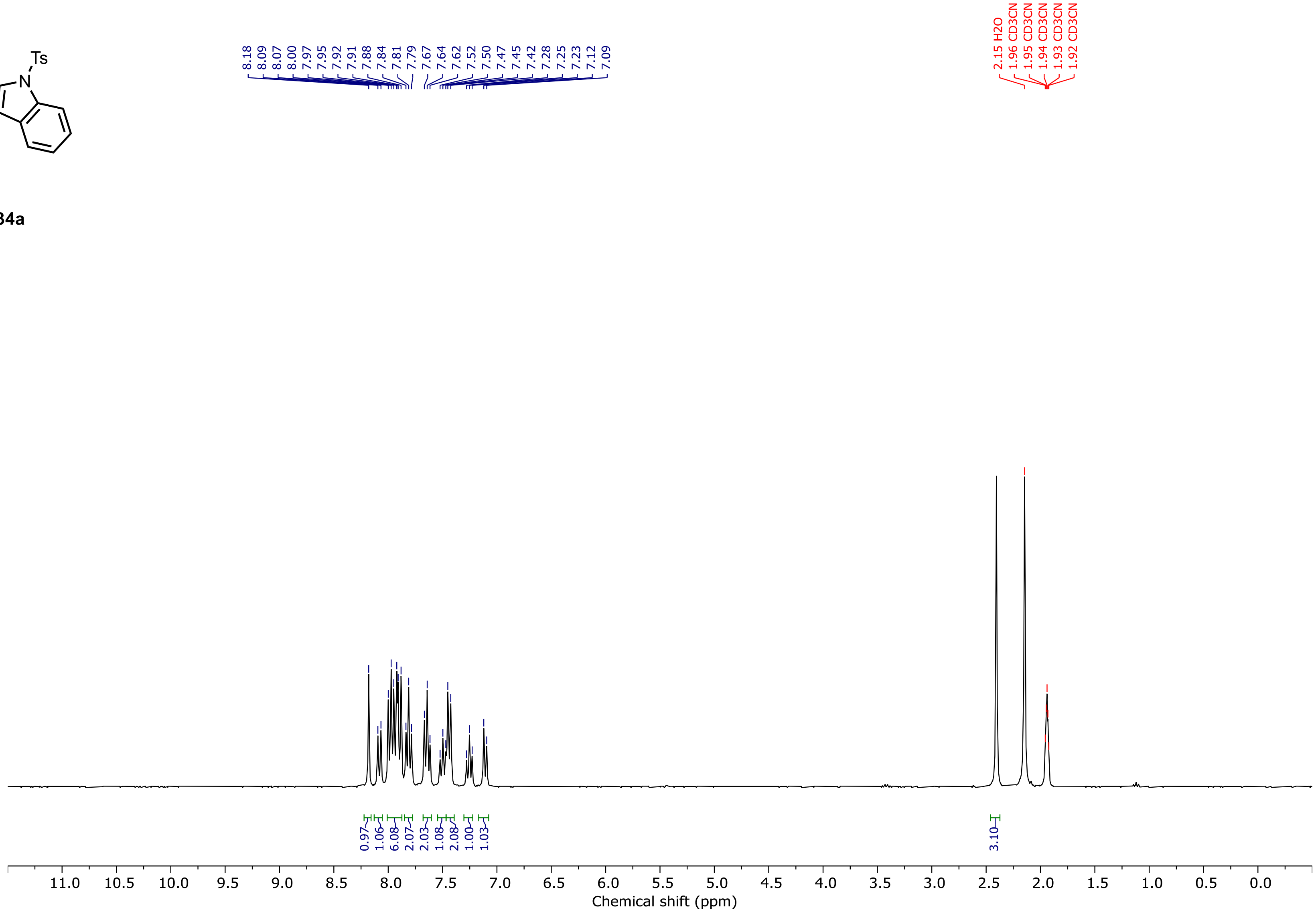

^{13}C NMR of 34a

$(CD_3)_2SO$, 76 MHz, 23 ºC

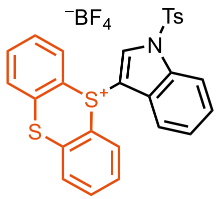

34a

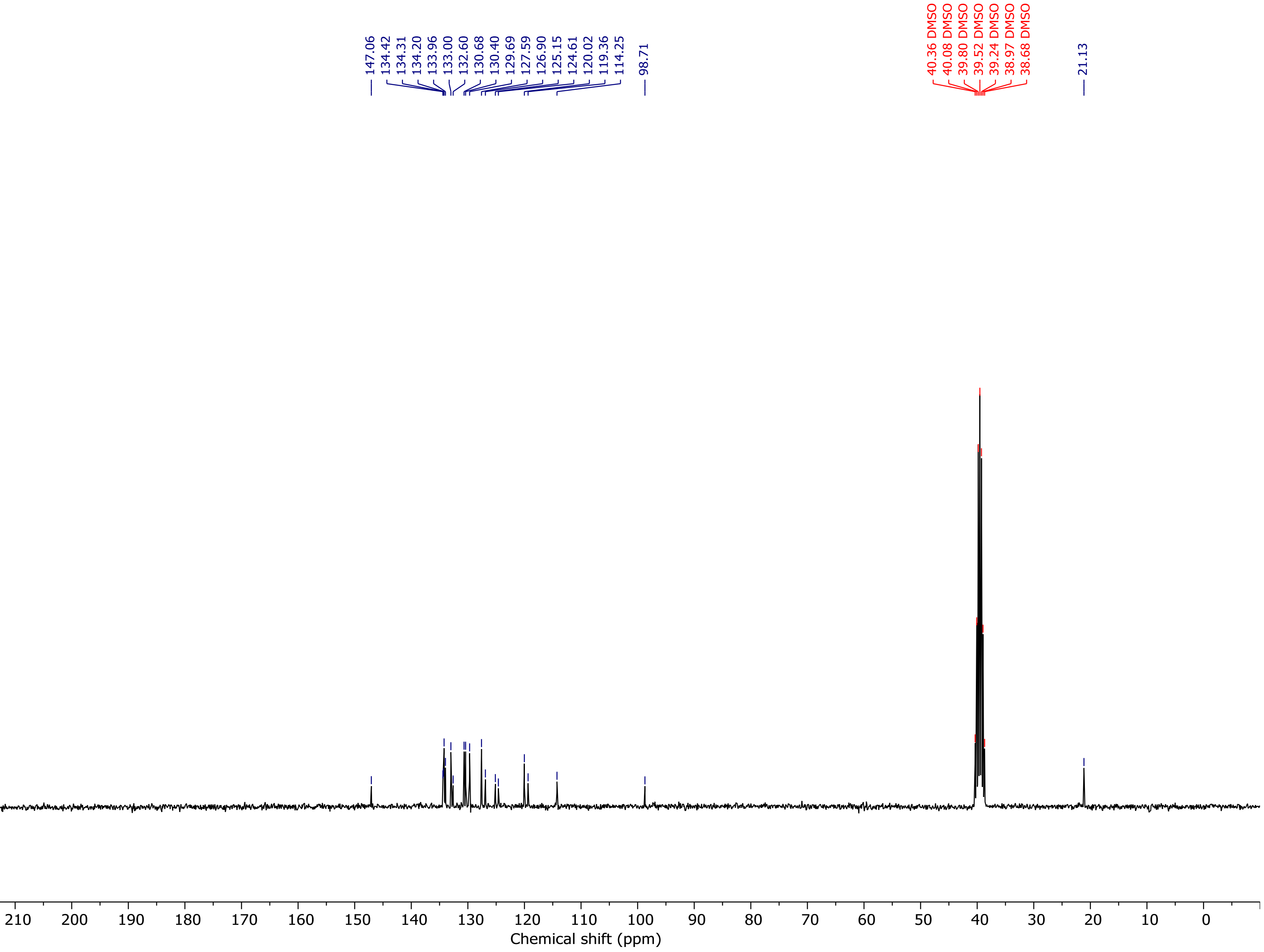

^{19}F NMR of 34a

CD_3CN, 282 MHz, 23 ºC.

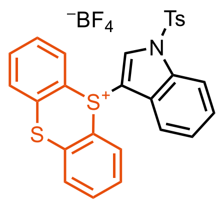

34a

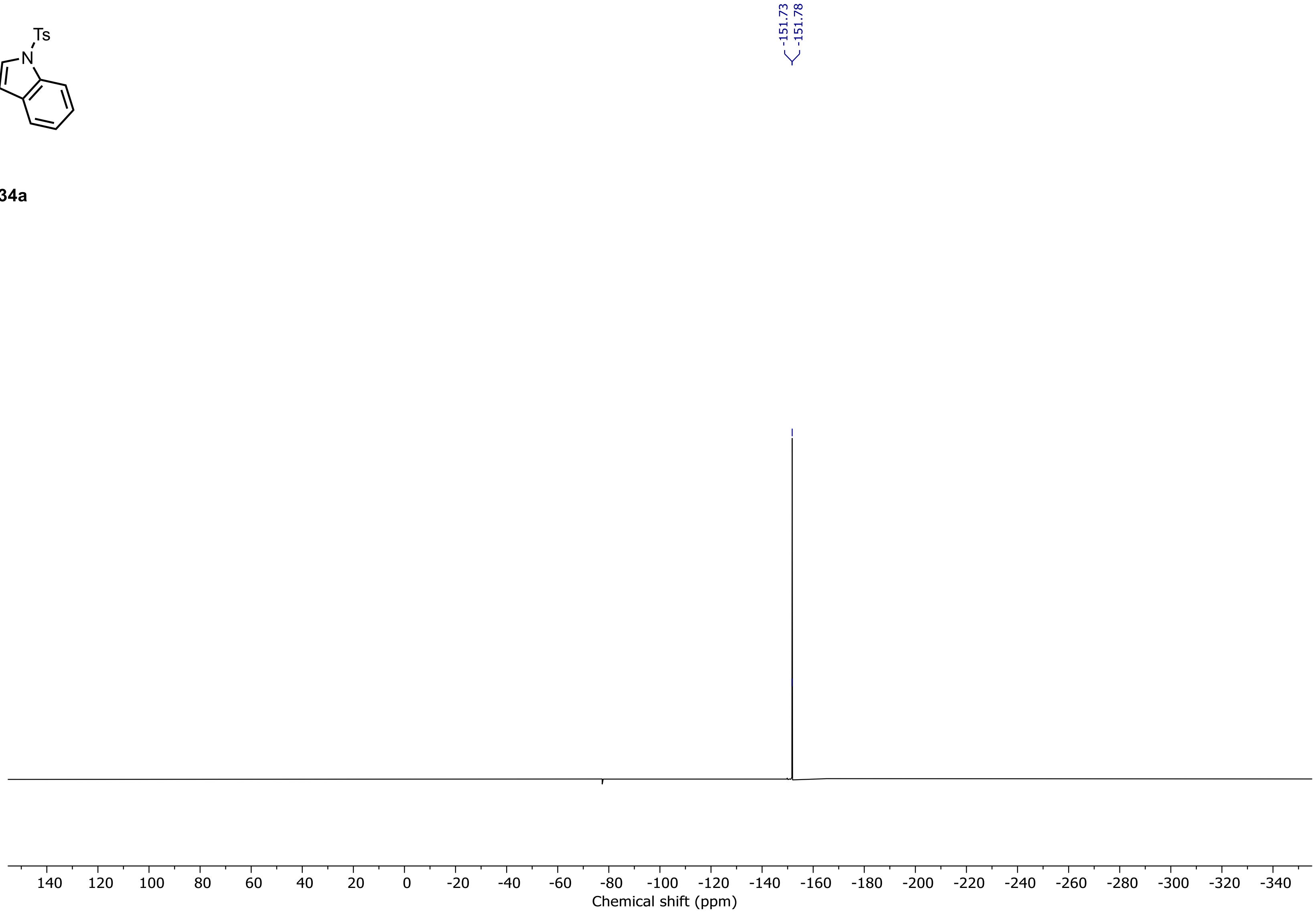

^{1}H NMR of 35a

CD_3CN, 600 MHz, 23 ºC

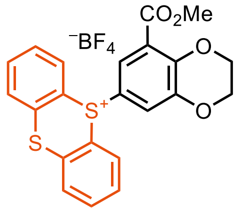

35a

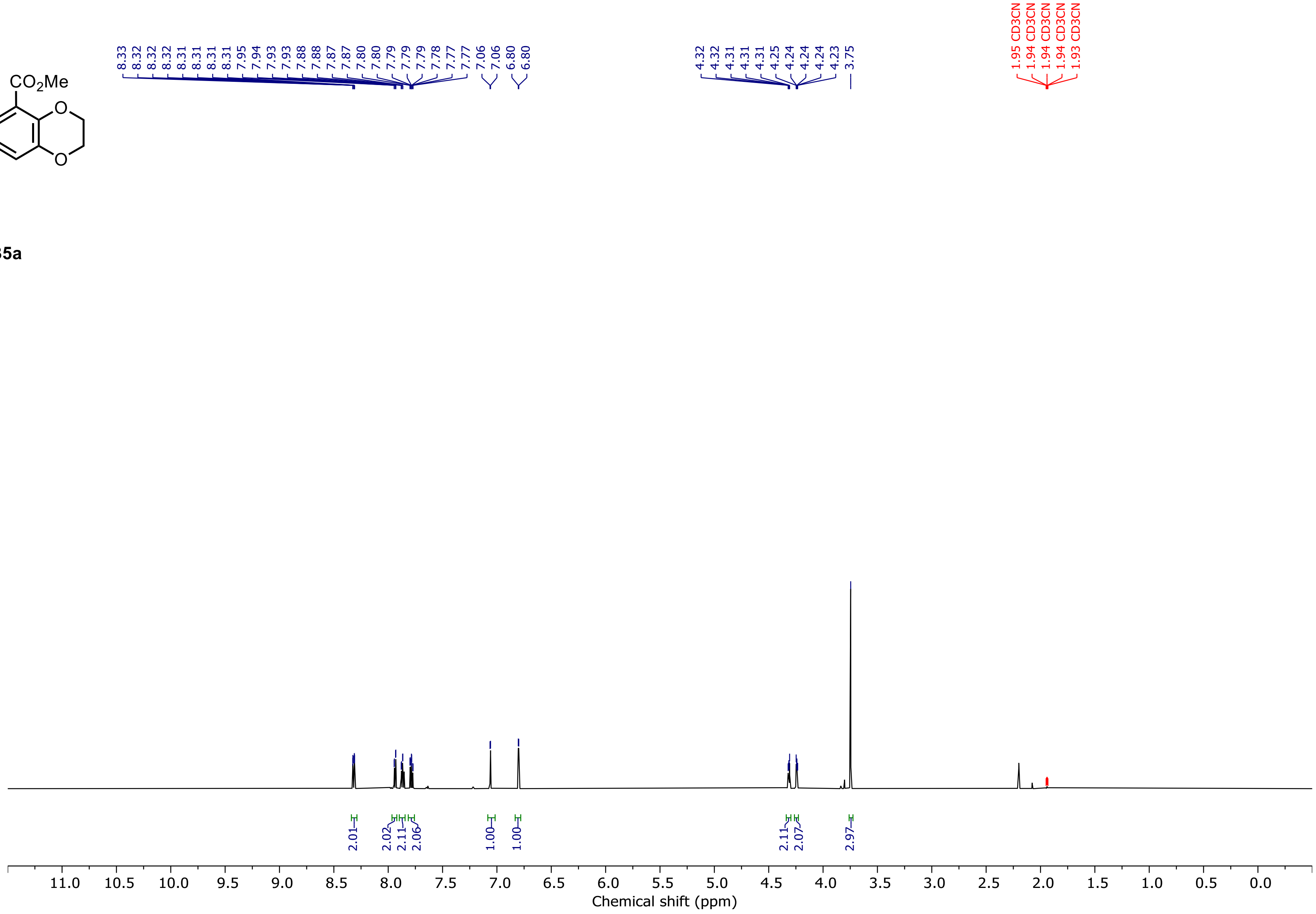

^{13}C NMR of 35a

CD_3CN, 151 MHz, 23 ºC

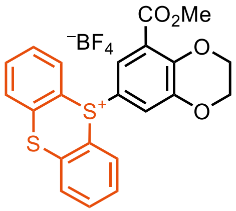

35a

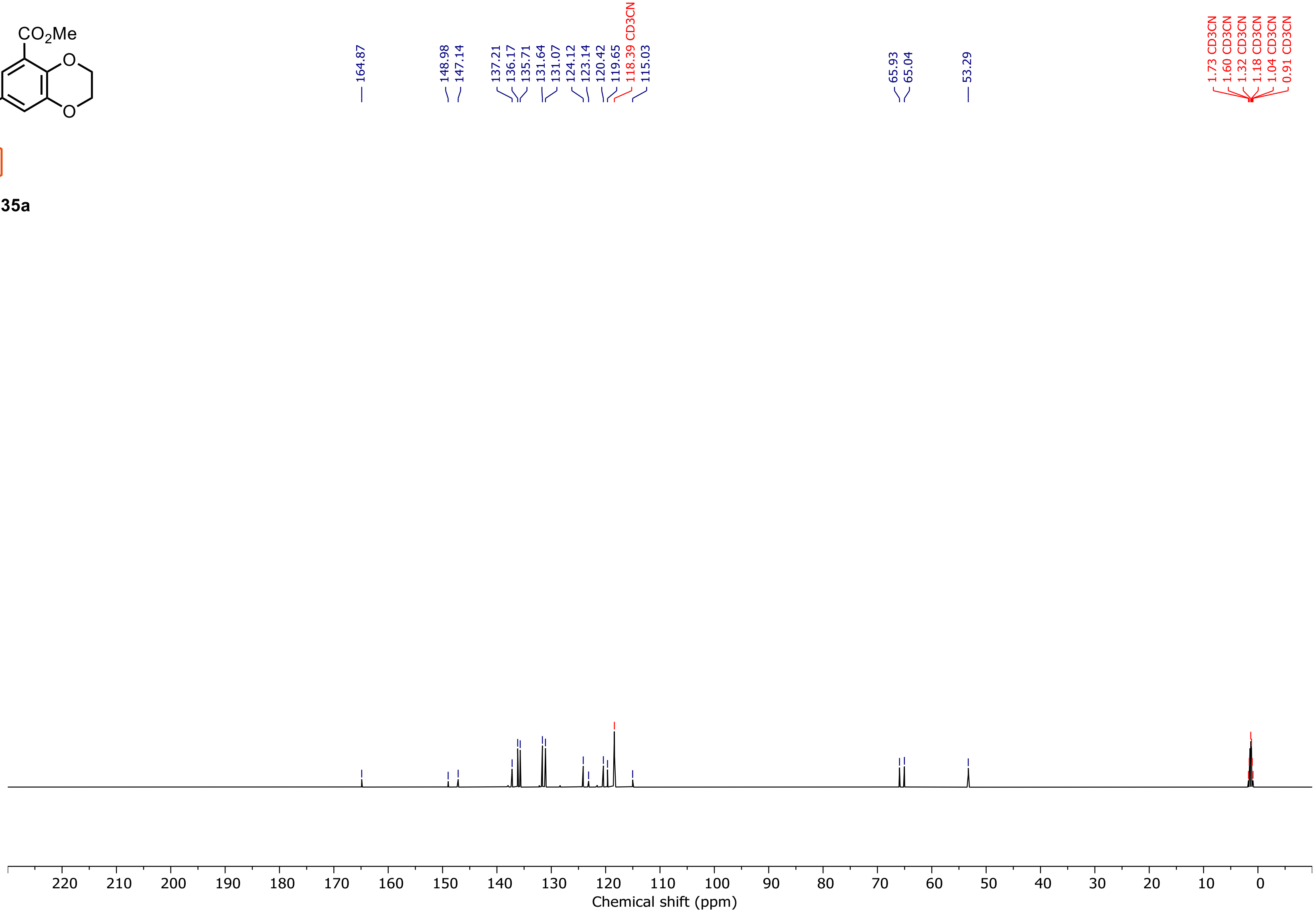

^{19}F NMR of 35a

CD_3CN, 565 MHz, 23 ºC

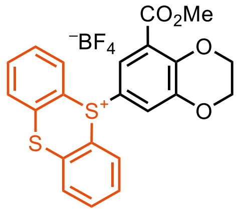

35a

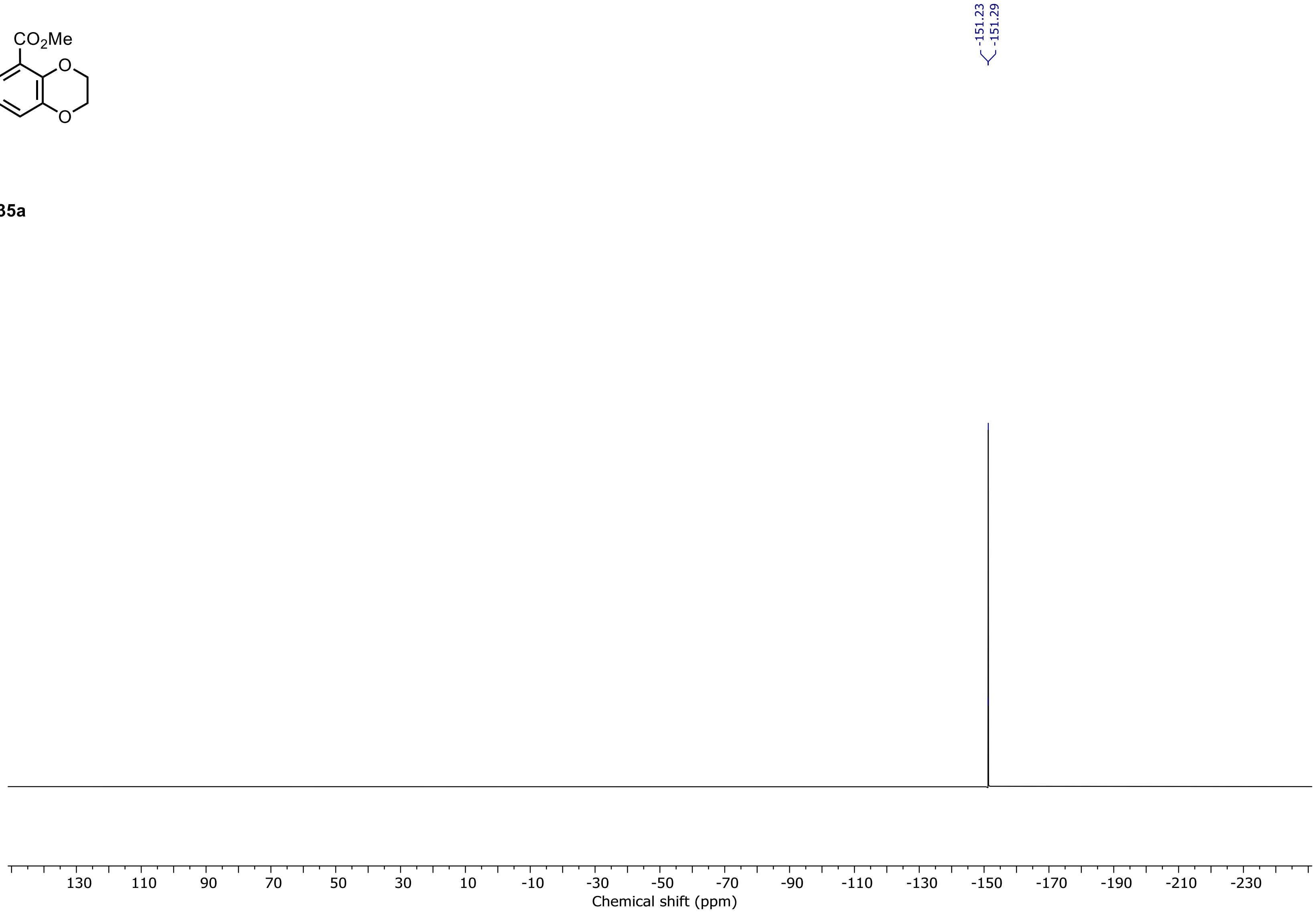

^{1}H NMR of 36a

CD_3CN, 600 MHz, 23 ºC

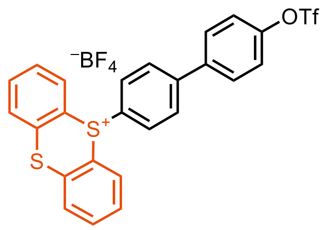

36a

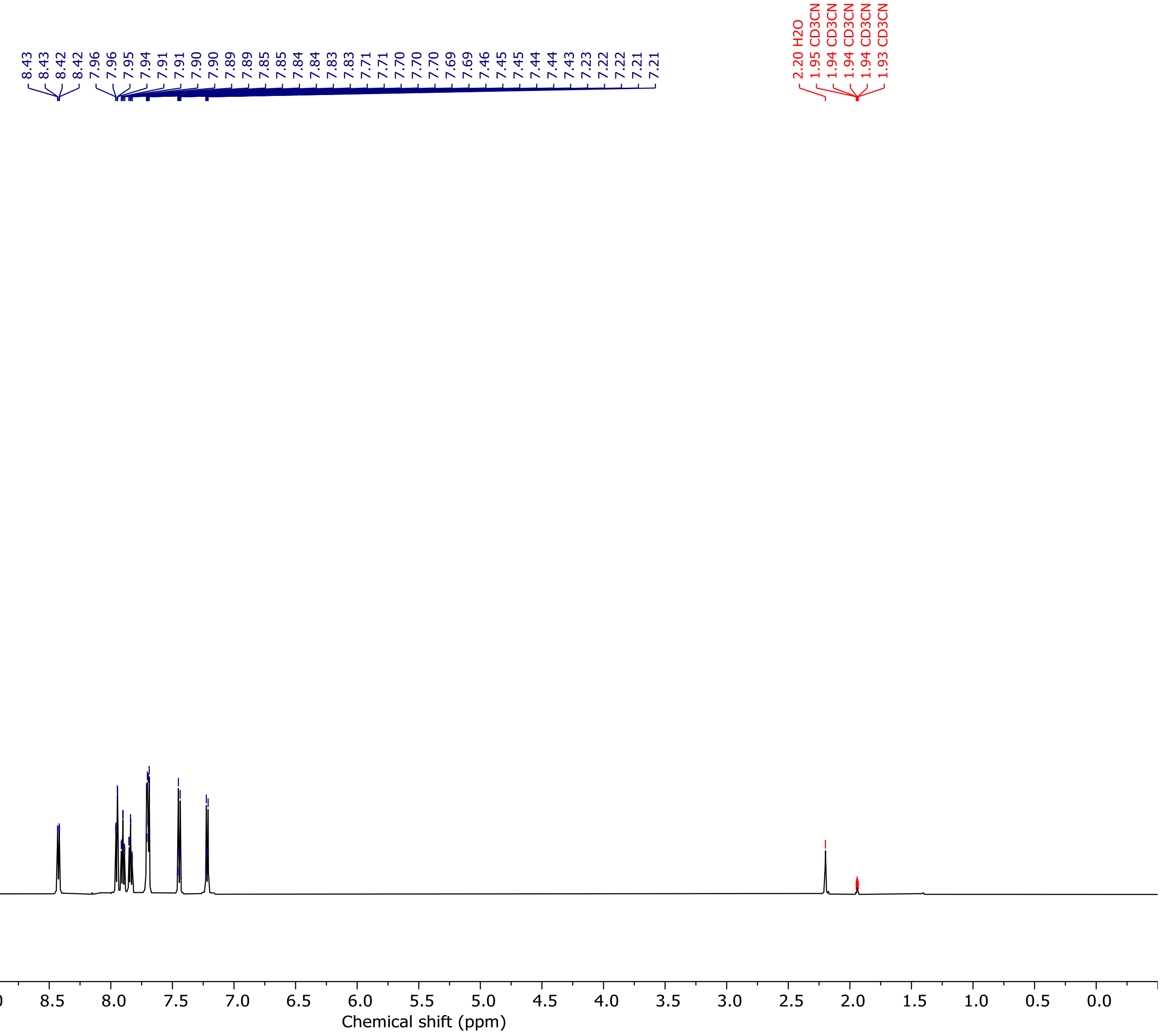

^{13}C NMR of 36a

CD_3CN, 151 MHz, 23 ºC

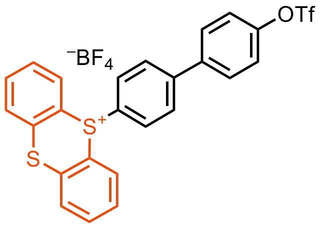

36a

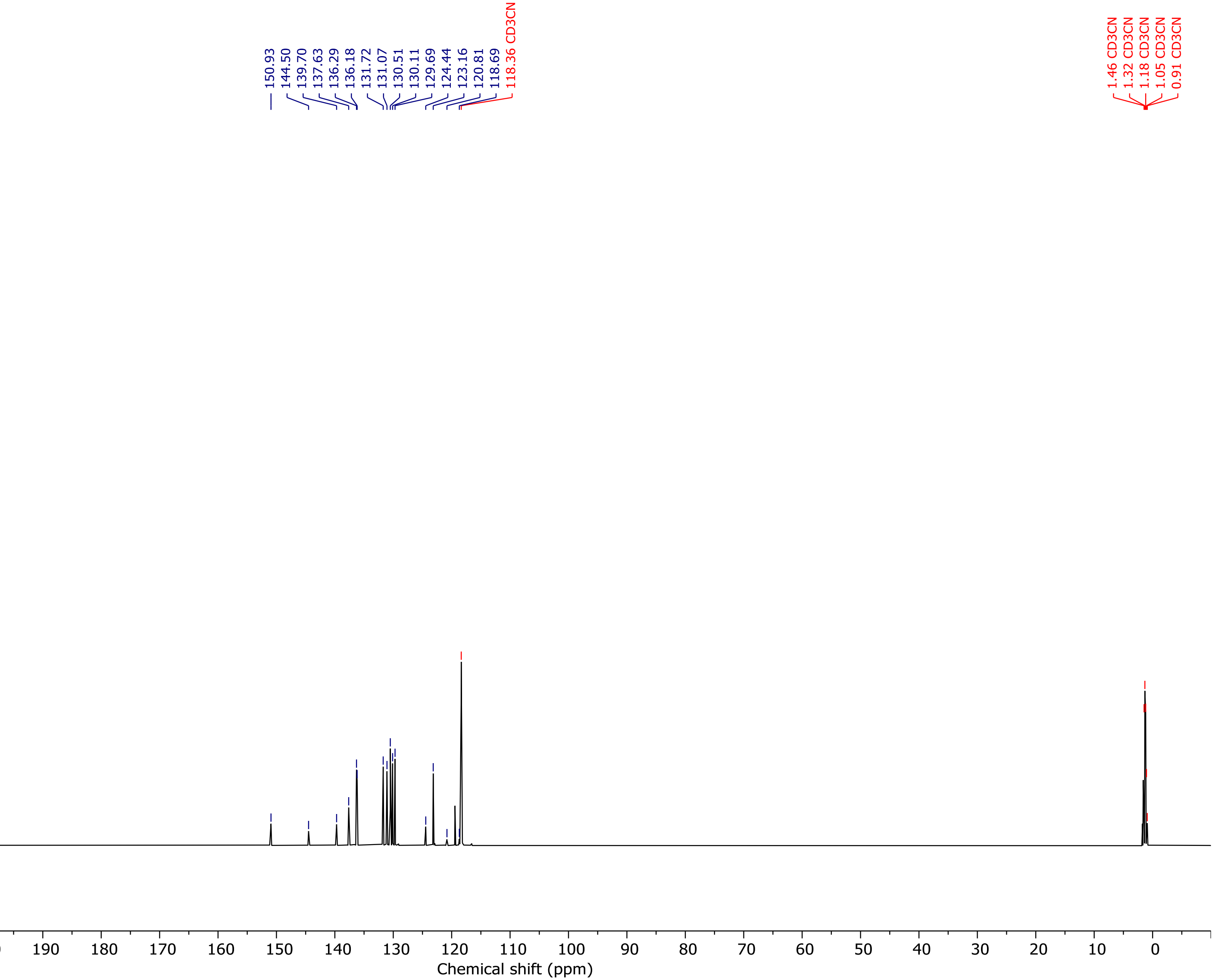

^{19}F NMR of 36a

CD_3CN, 565 MHz, 23 ºC

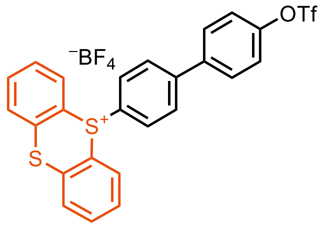

36a

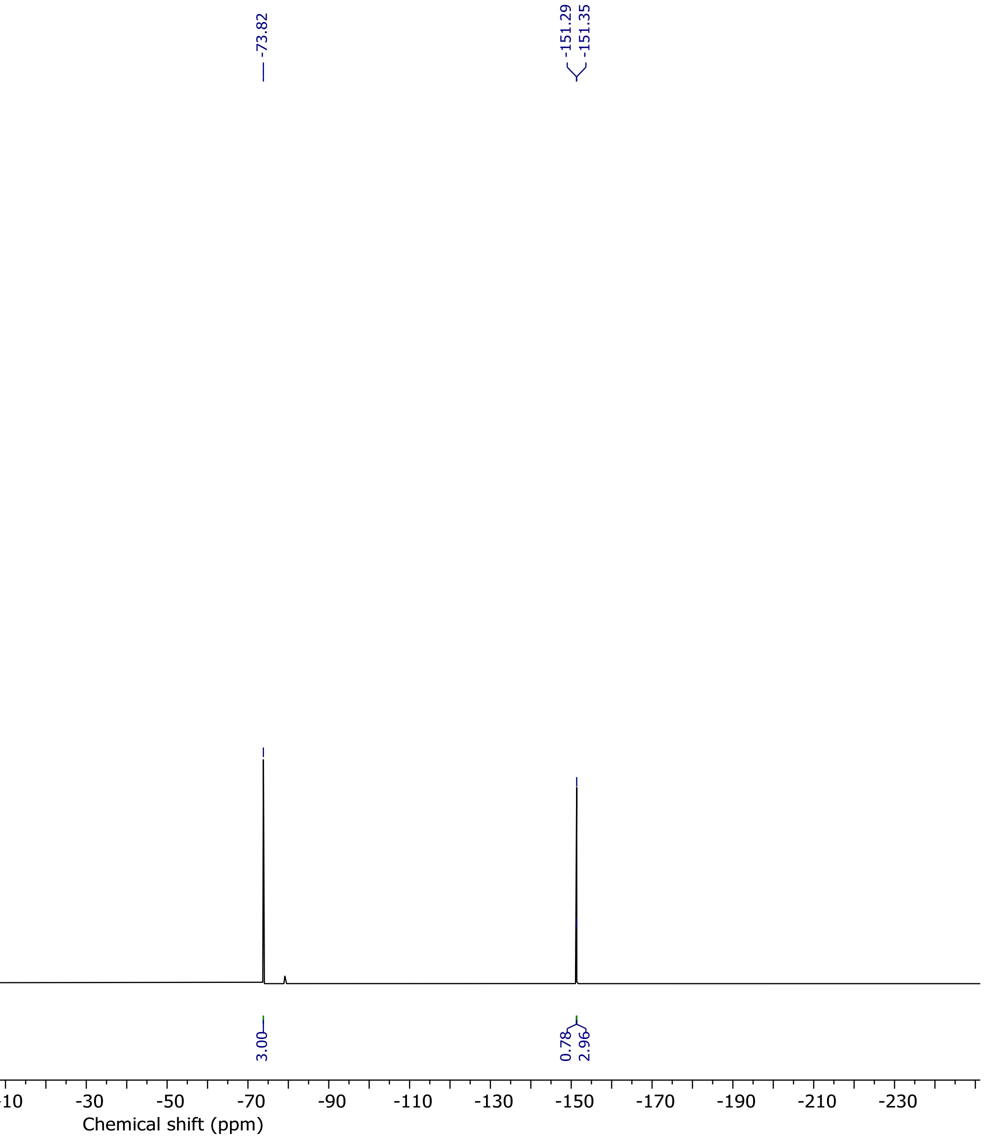

^{1}H NMR of 37a

CD_3CN, 600 MHz, 23 ºC

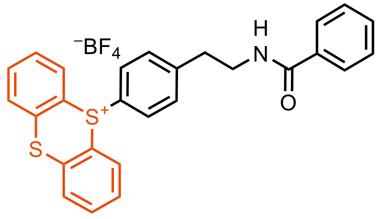

37a

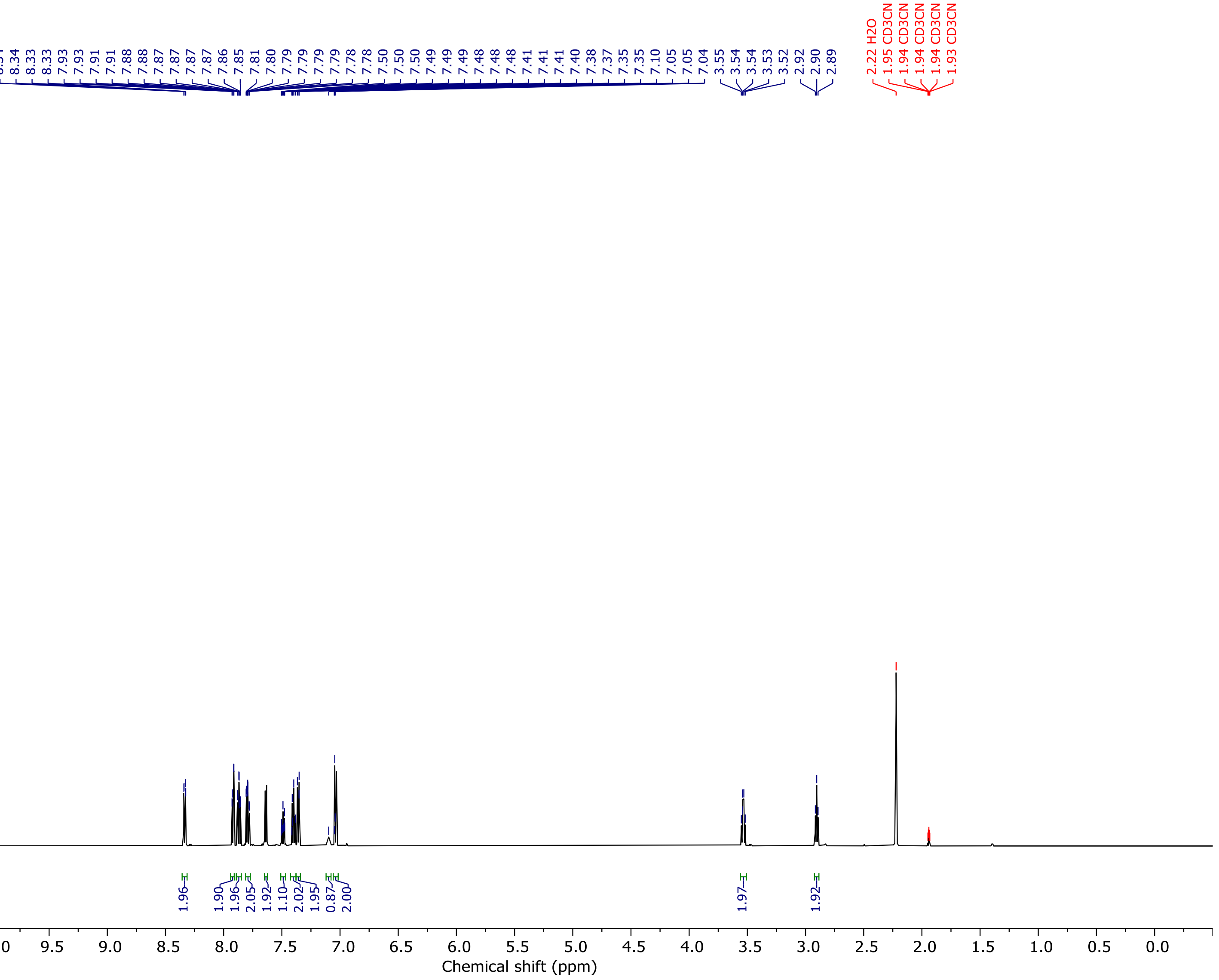

^{13}C NMR of 37a

CD_3CN, 151 MHz, 23 ºC.

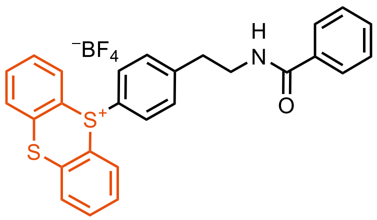

37a

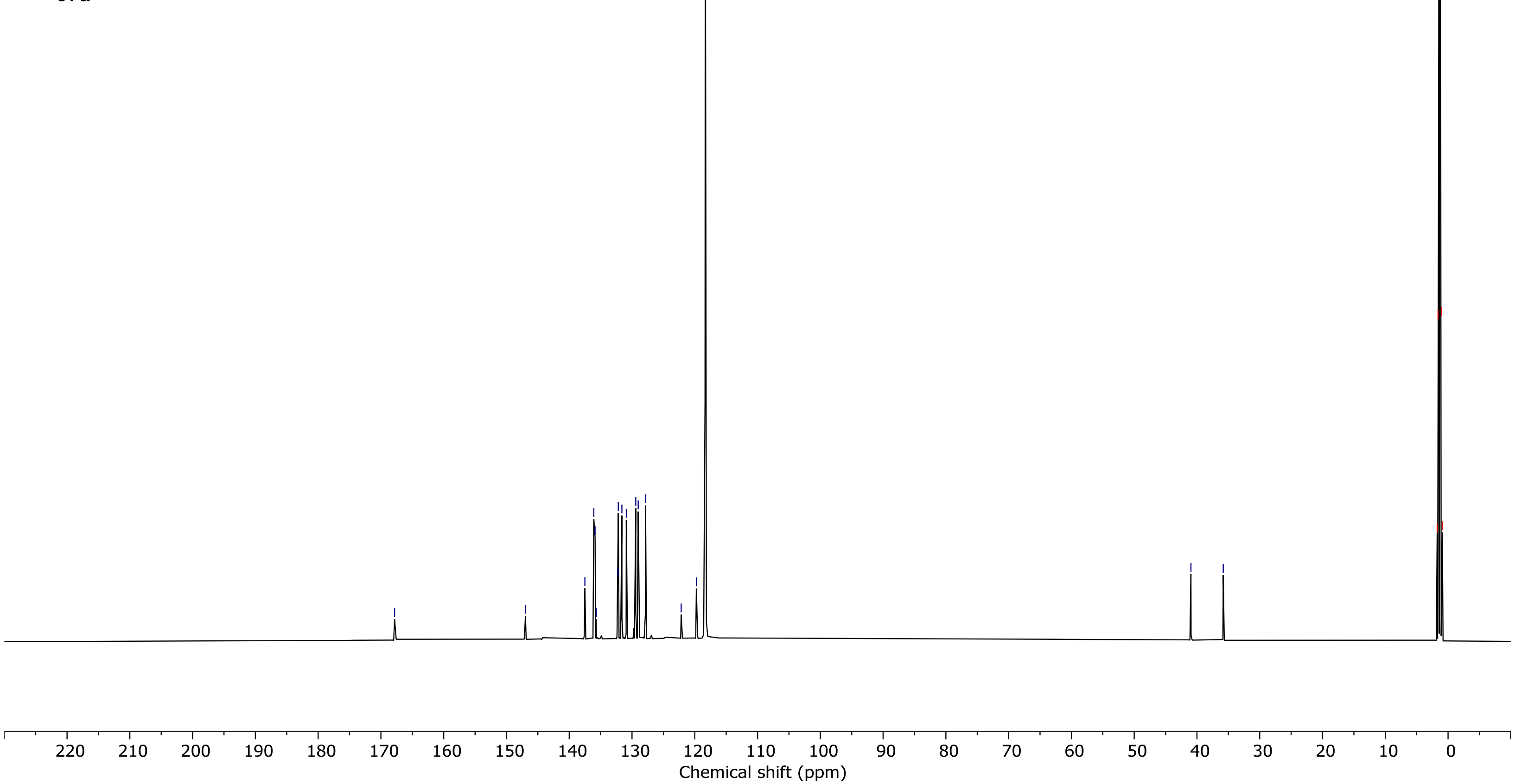

^{19}F NMR of 37a

CD_3CN, 565 MHz, 23 ºC.

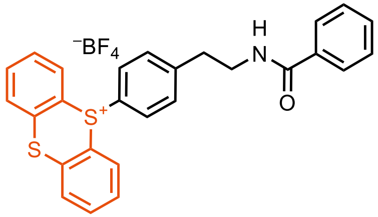

37a

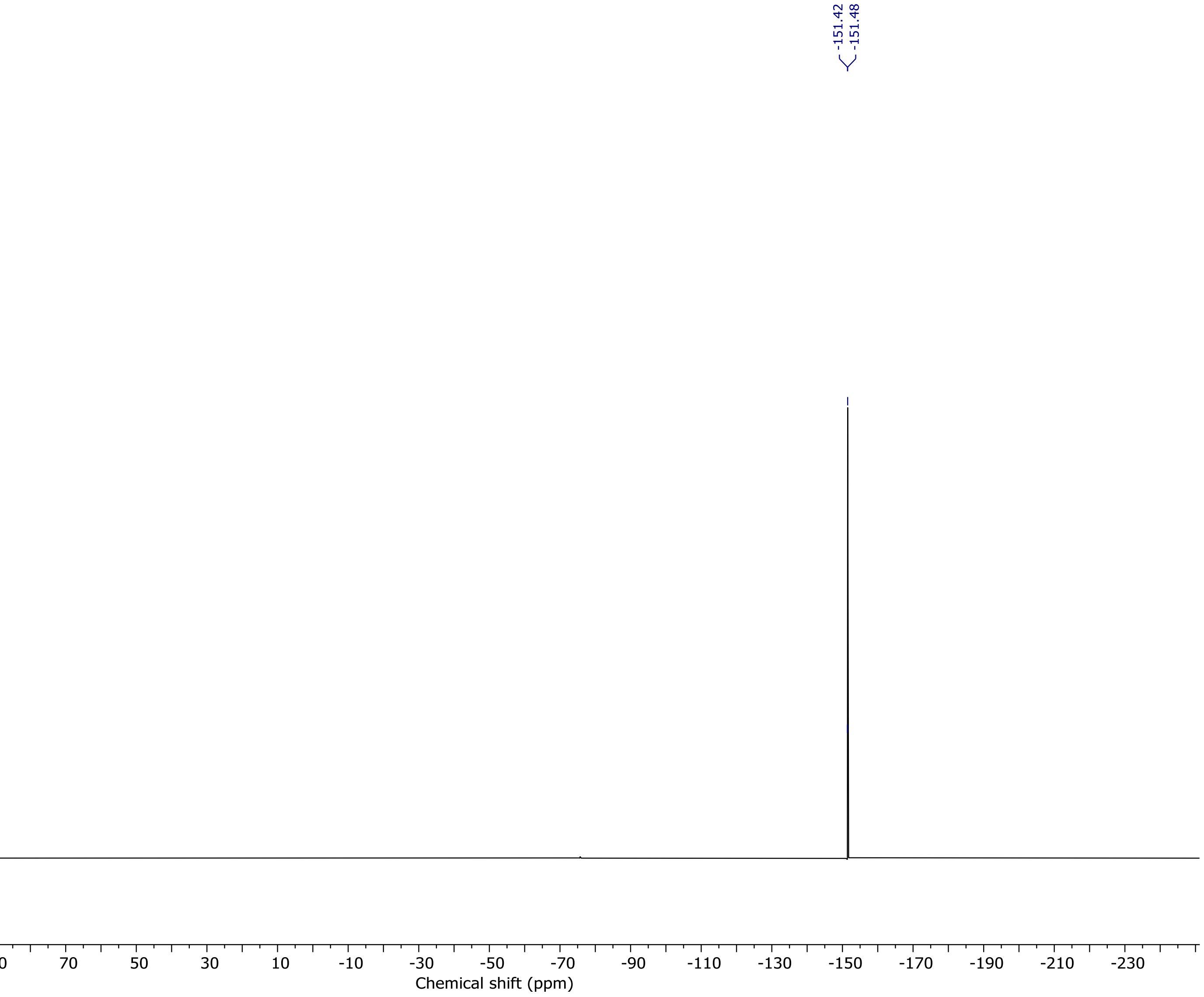

^{1}H NMR of 38a

CDCl$_3$, 300 MHz, 23 ºC.

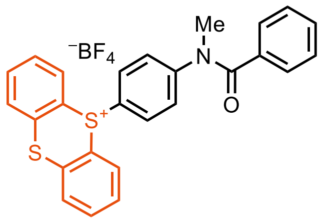

38a

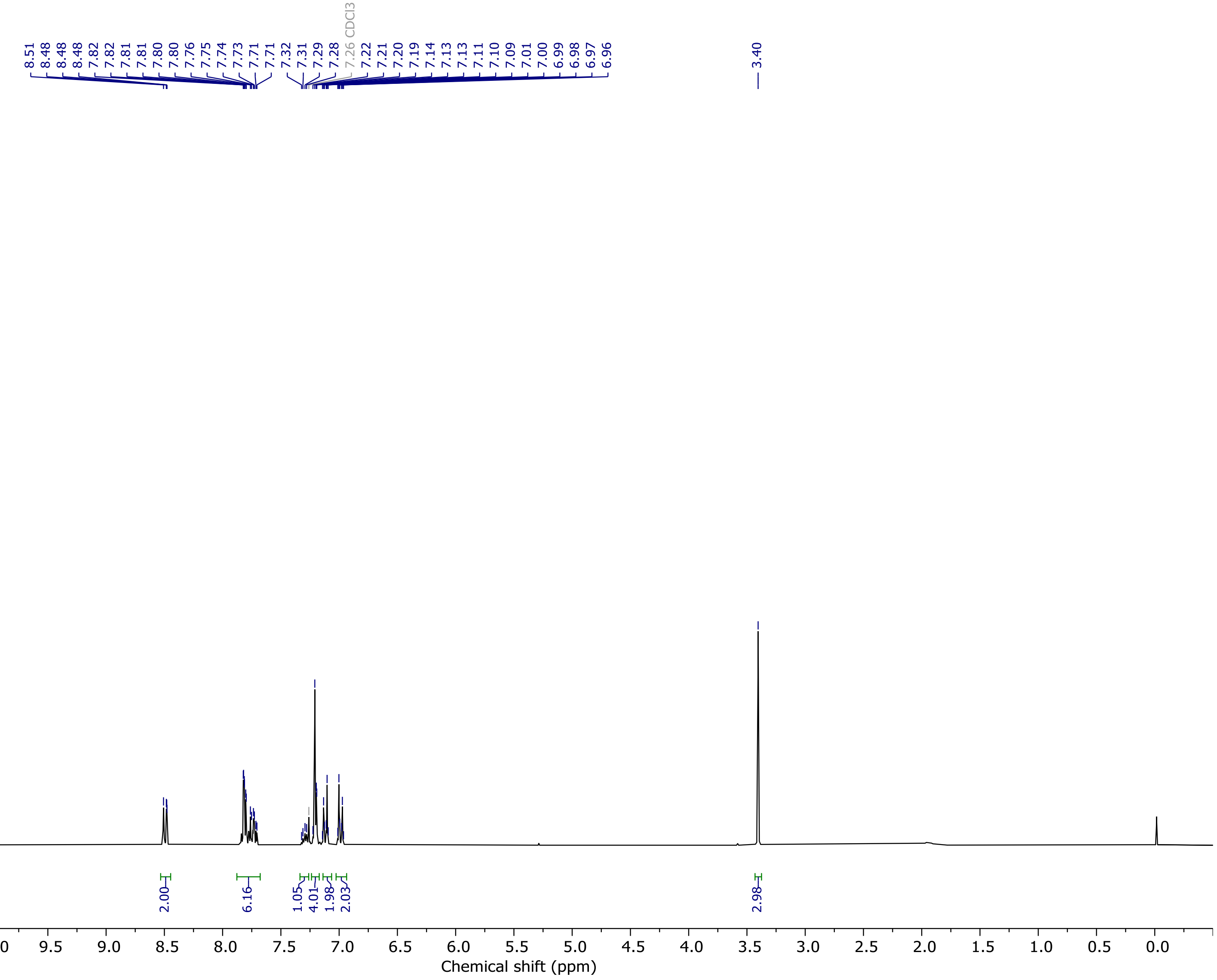

^{13}C NMR of 38a

$CDCl_3$, 76 MHz, 23 ºC.

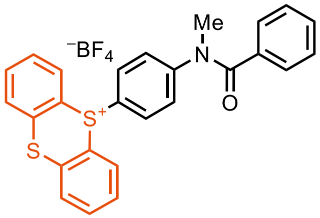

38a

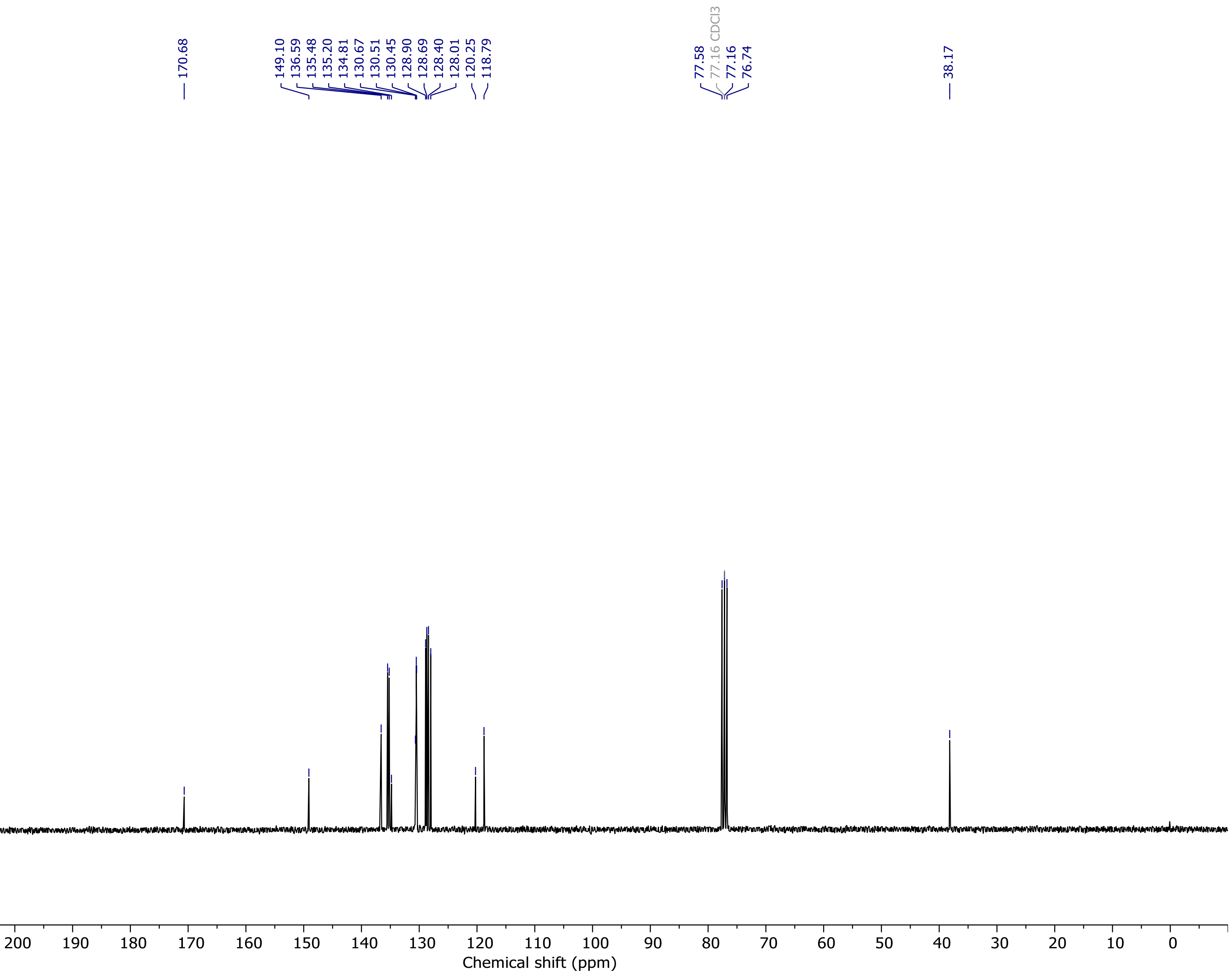

^{19}F NMR of 38a

$CDCl_3$, 282 MHz, 23 ºC.

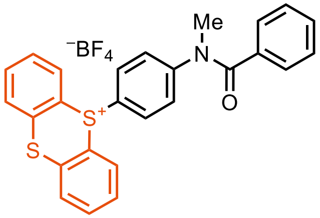

38a

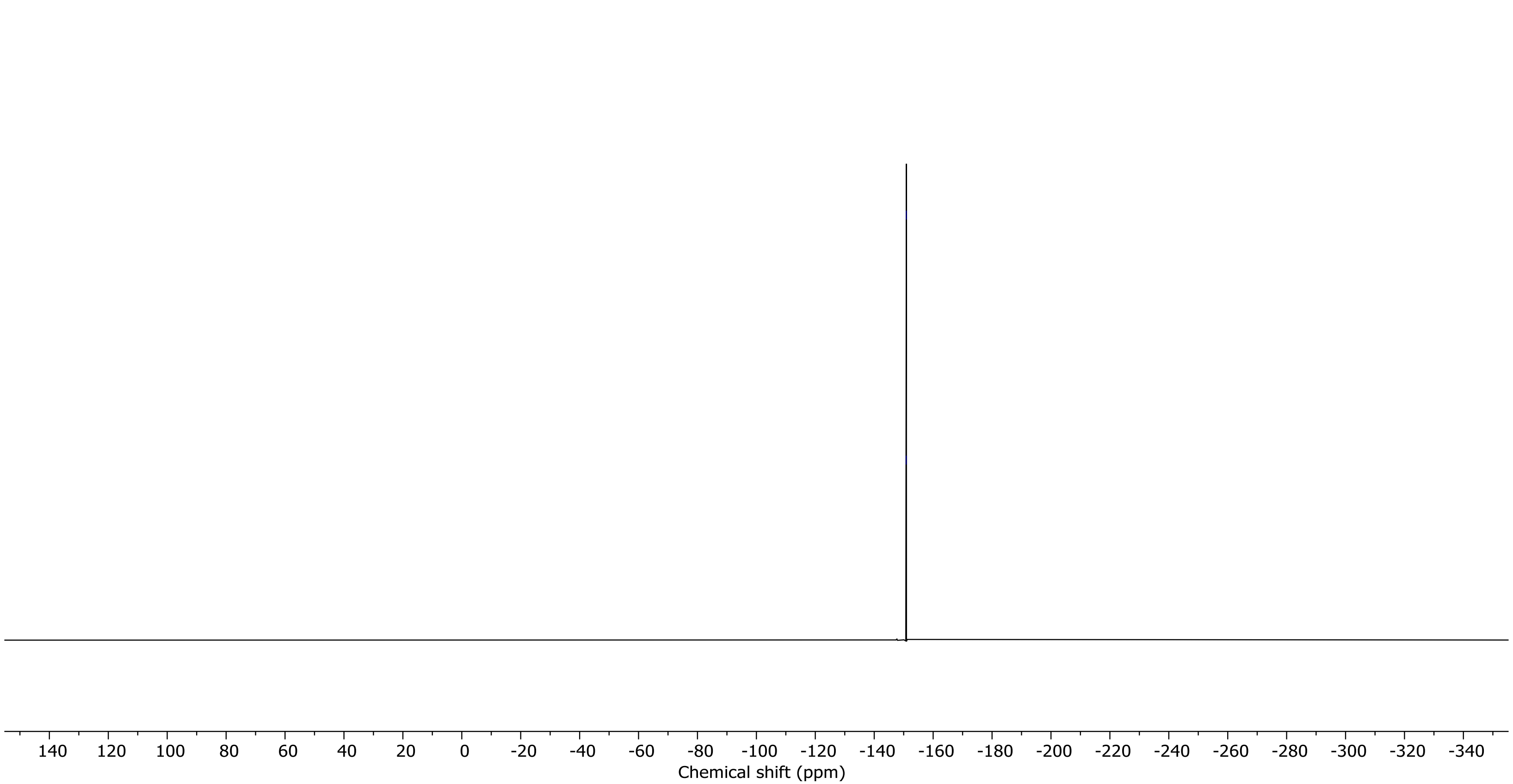

^{1}H NMR of 39a

$CDCl_3$, 500 MHz, 23 ºC.

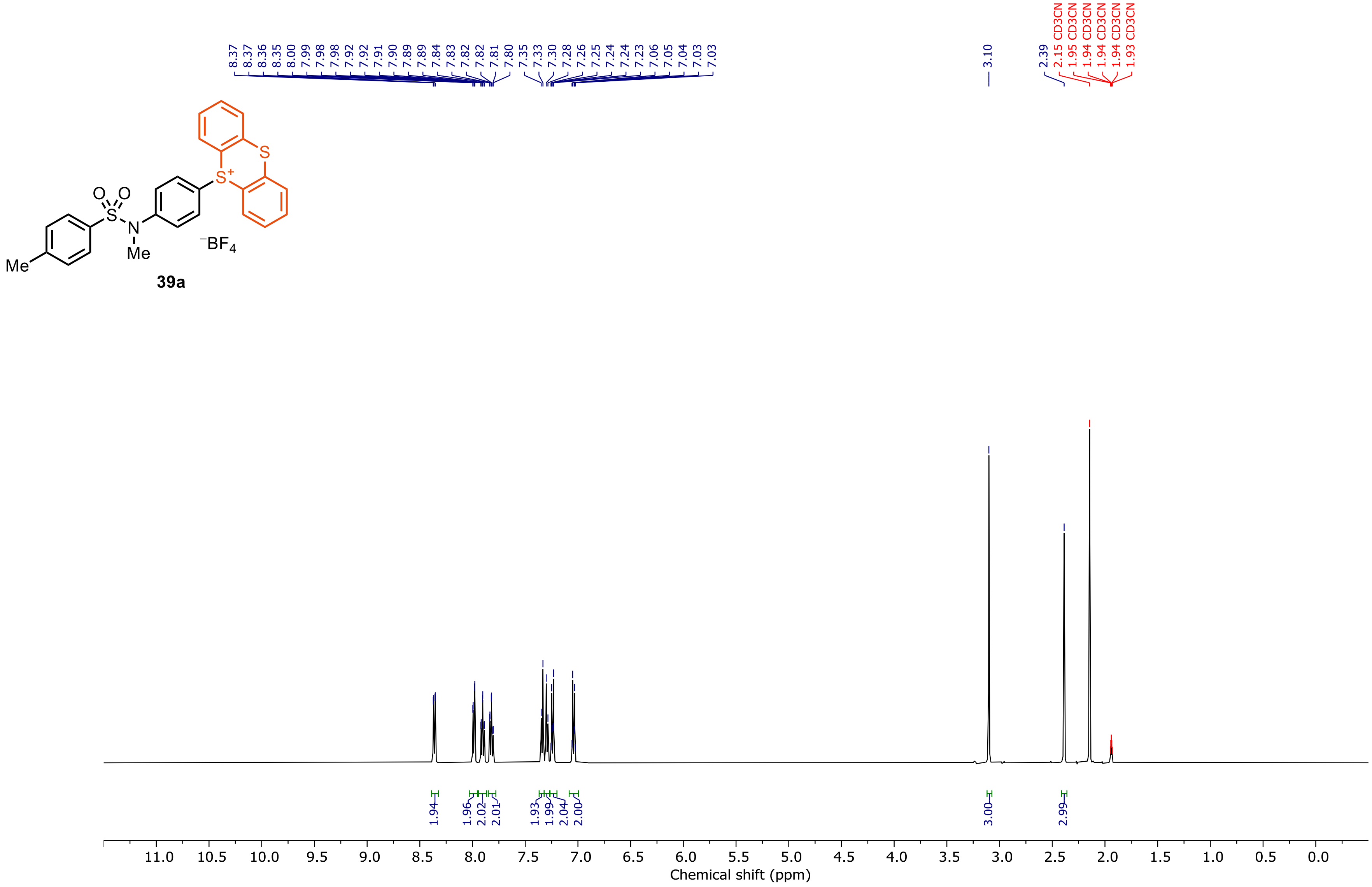

^{13}C NMR of 39a

$CDCl_3$, 126 MHz, 23 ºC.

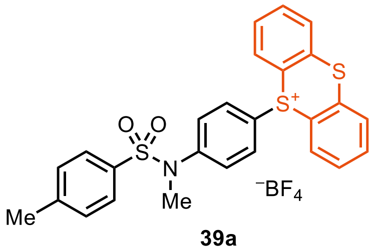

39a

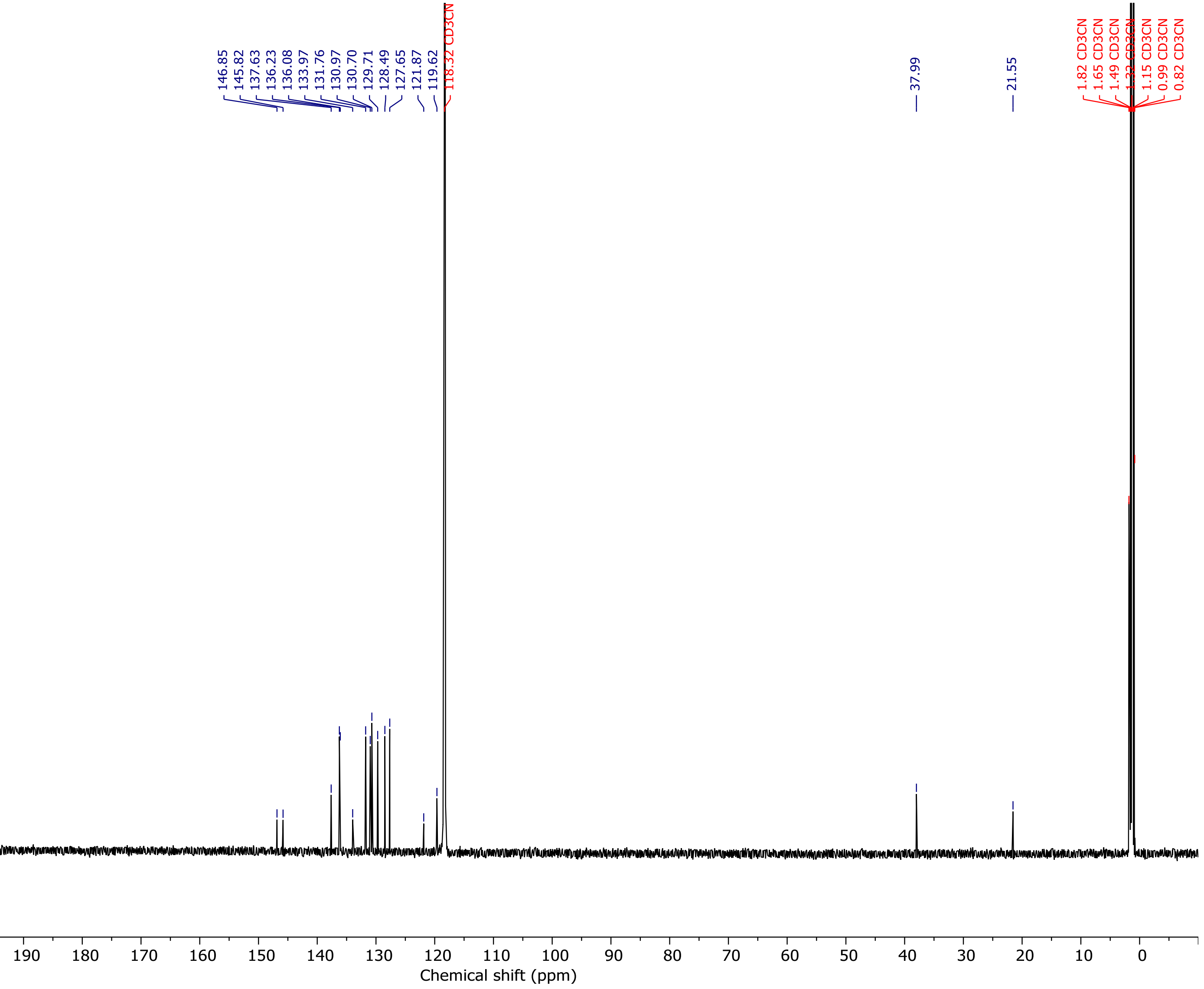

^{19}F NMR of 39a

$CDCl_3$, 471 MHz, 23 ºC.

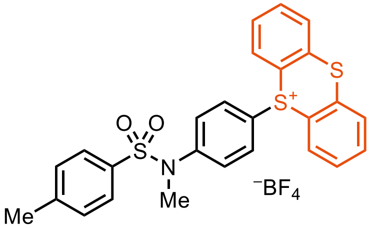

39a

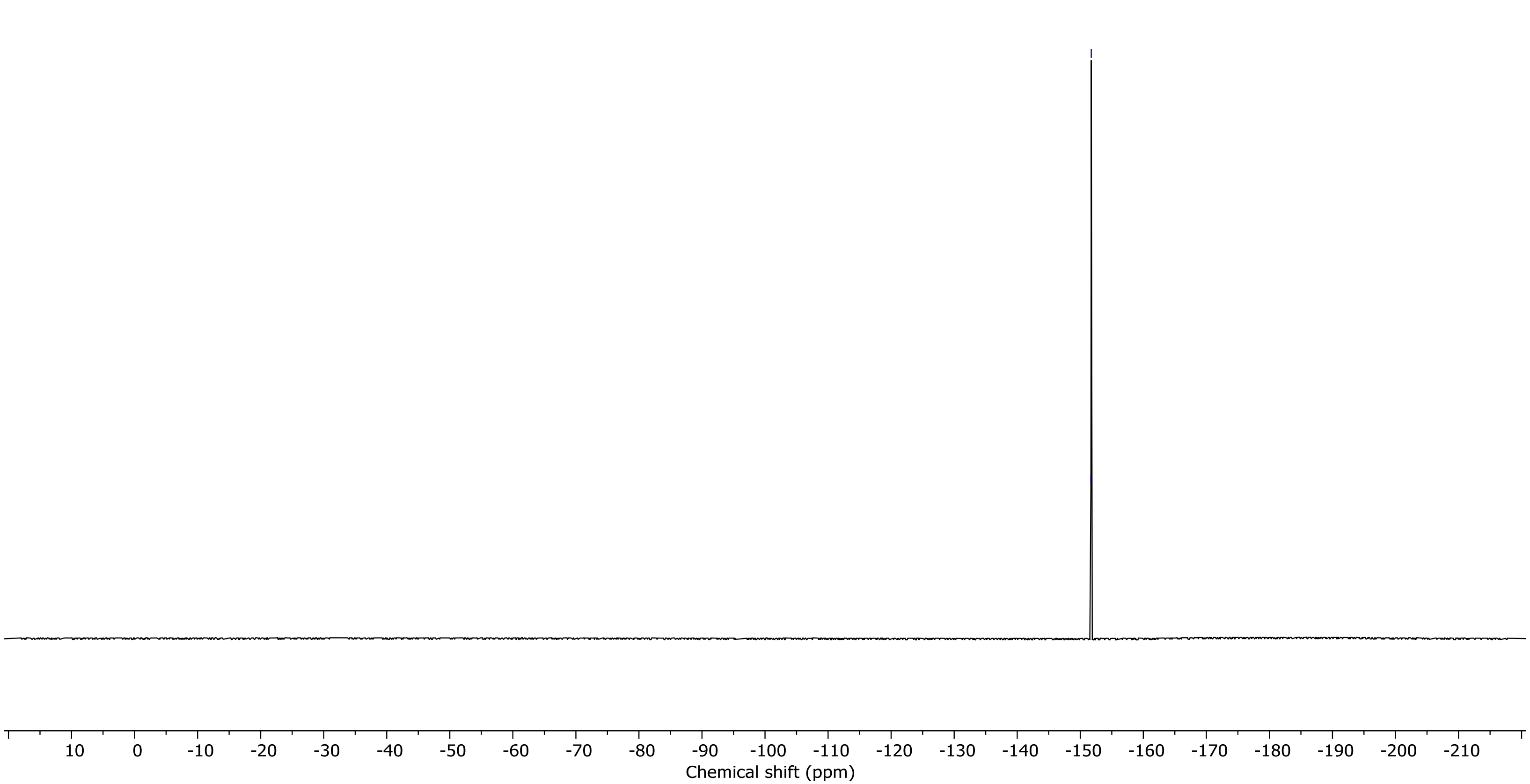

^{1}H NMR of 40

CDCl$_3$, 300 MHz, 23 ºC

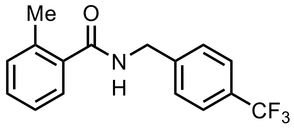

40

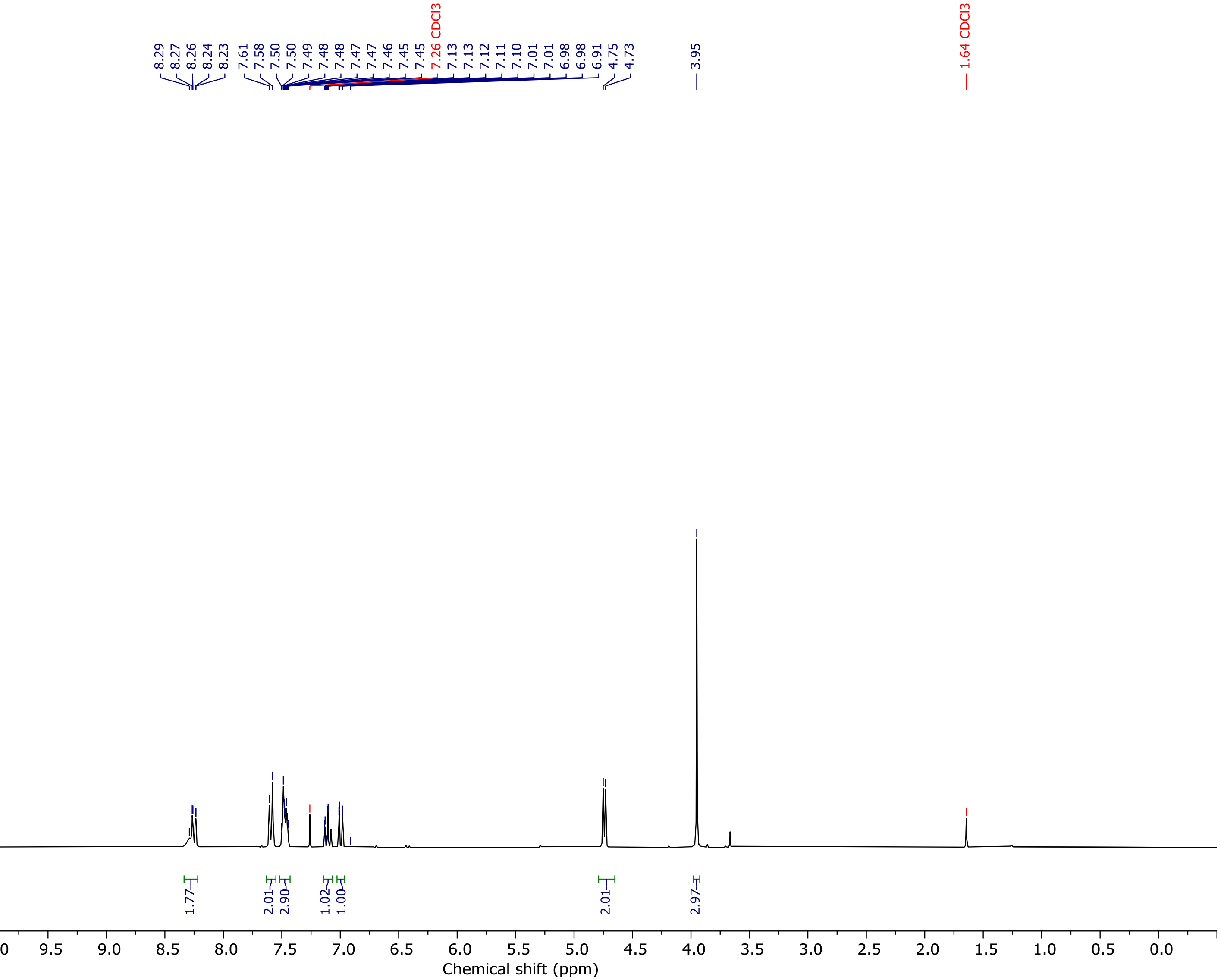

^{13}C NMR of 40

$CDCl_3$, 75 MHz, 23 ºC.

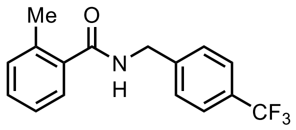

40

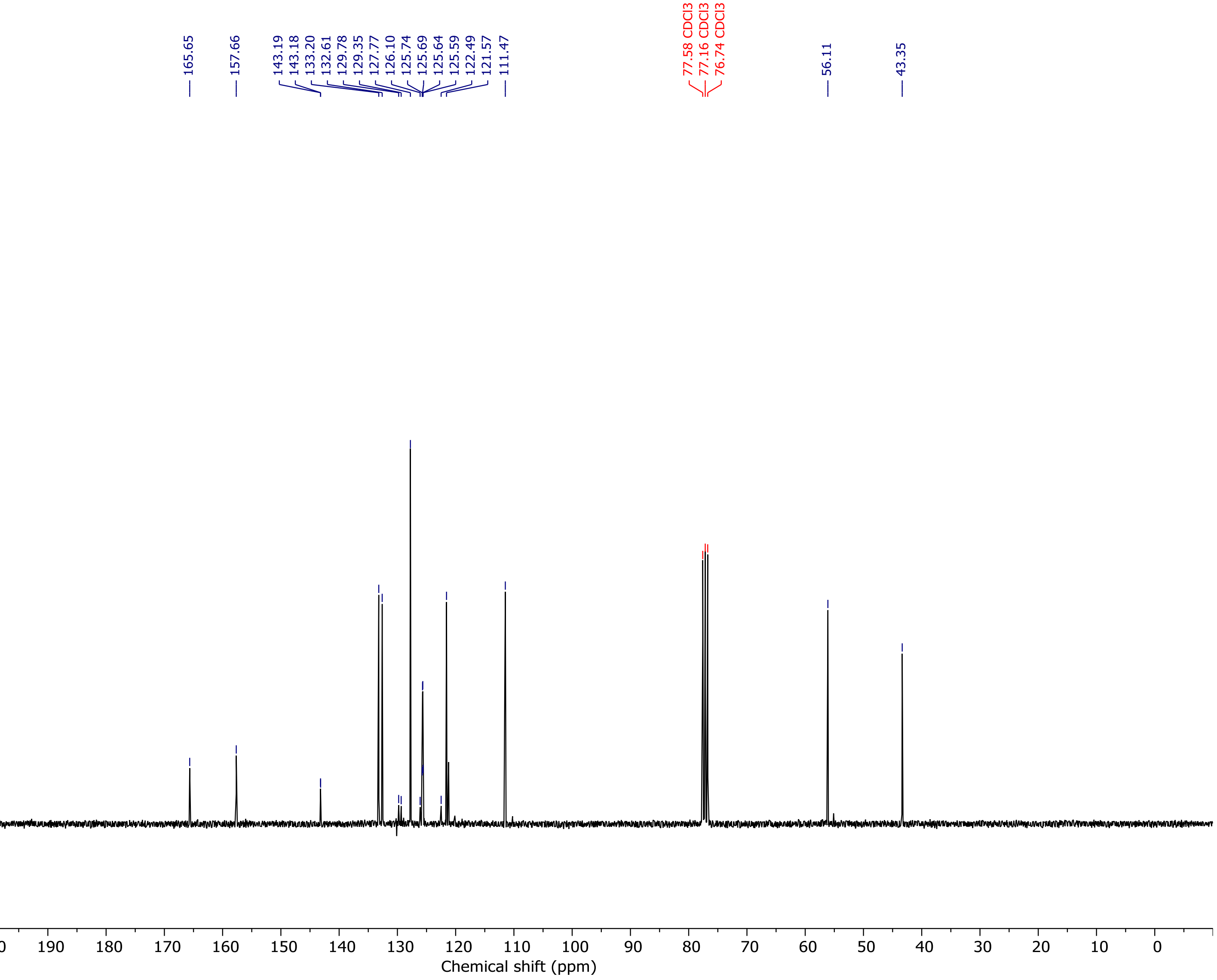

^{19}F NMR of 40

$CDCl_3$, 282 MHz, 23 ºC.

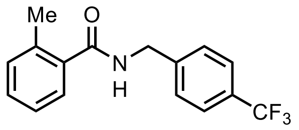

40

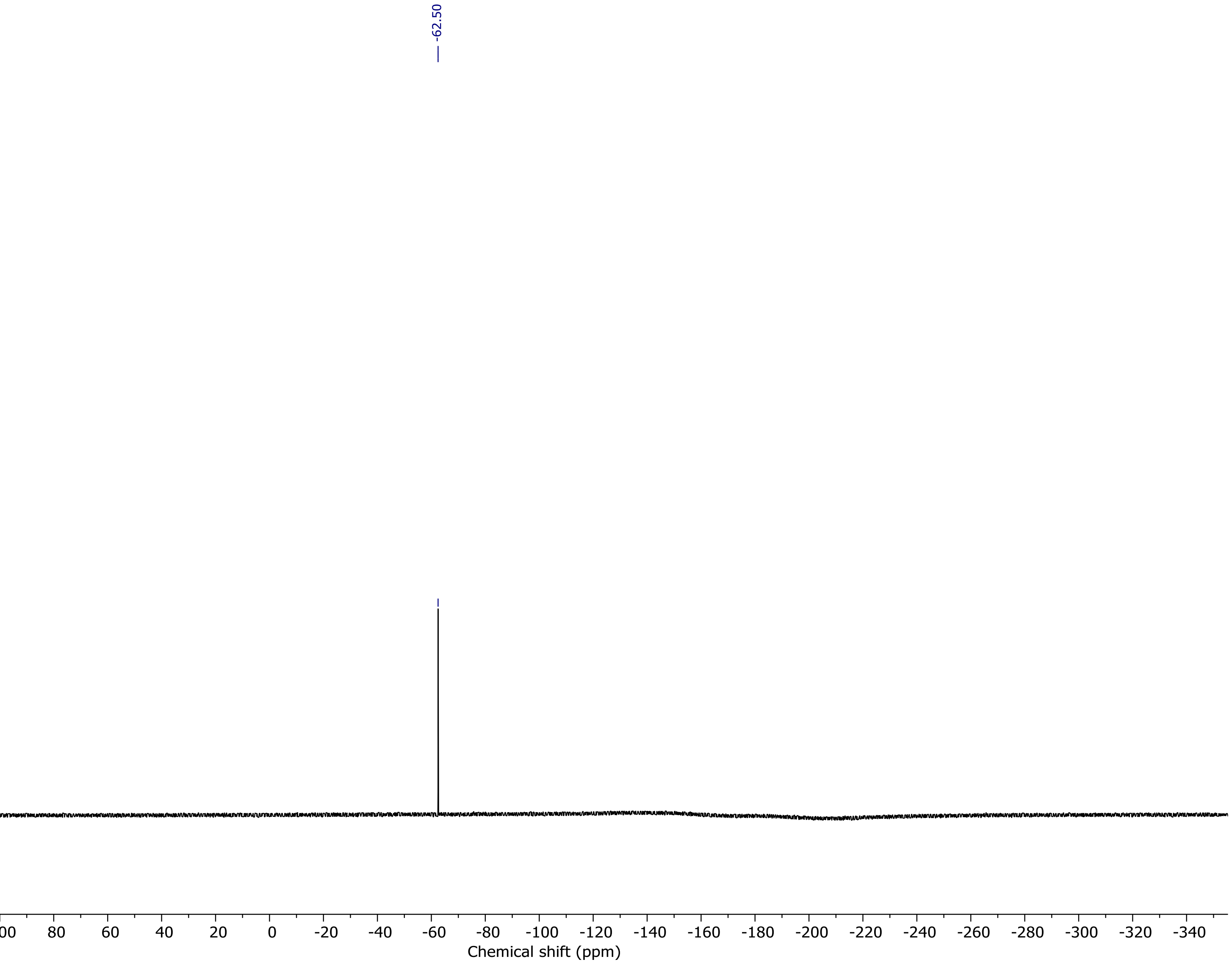

^{1}H NMR of 40a

CD_3CN, 600 MHz, 23 ºC.

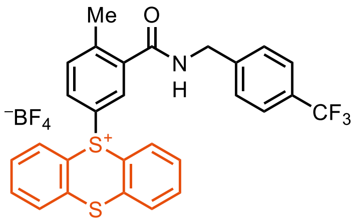

40a

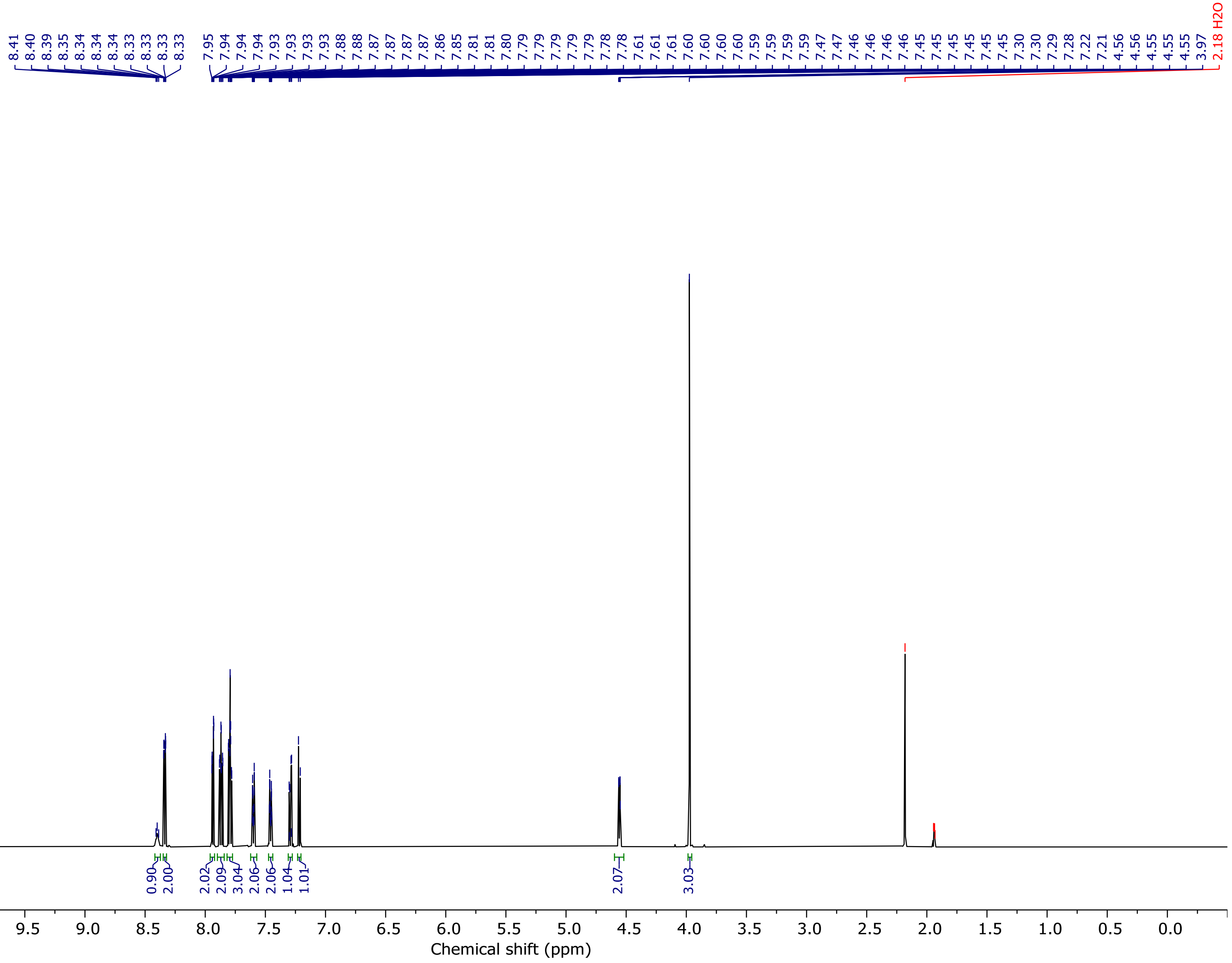

^{13}C NMR of 40a

CD_3CN, 151 MHz, 23 ºC.

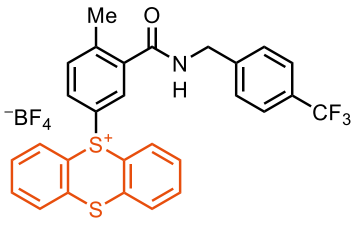

40a

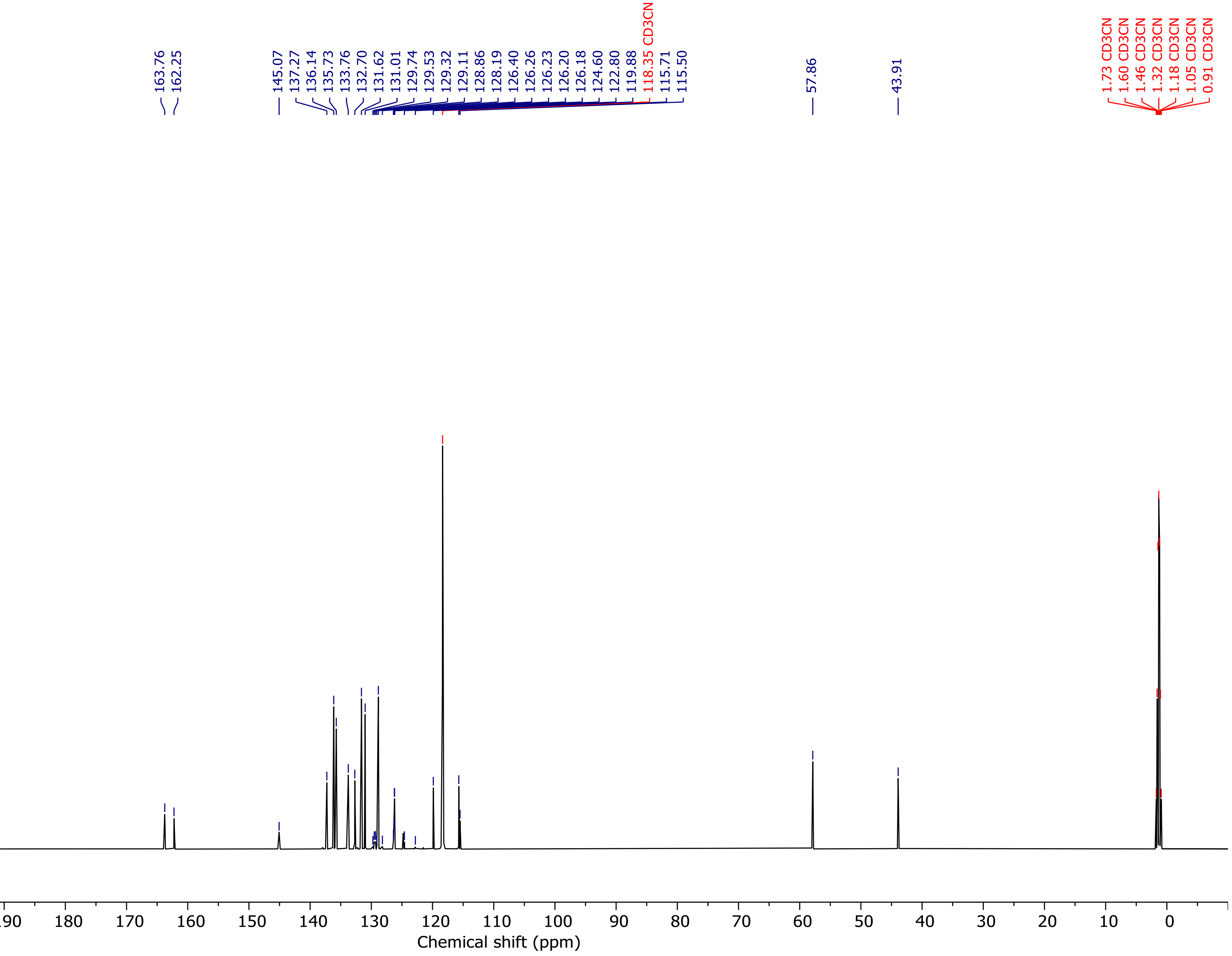

^{19}F NMR of 40a

CD_3CN, 565 MHz, 23 ºC.

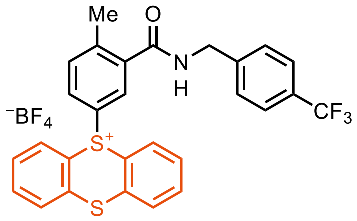

40a

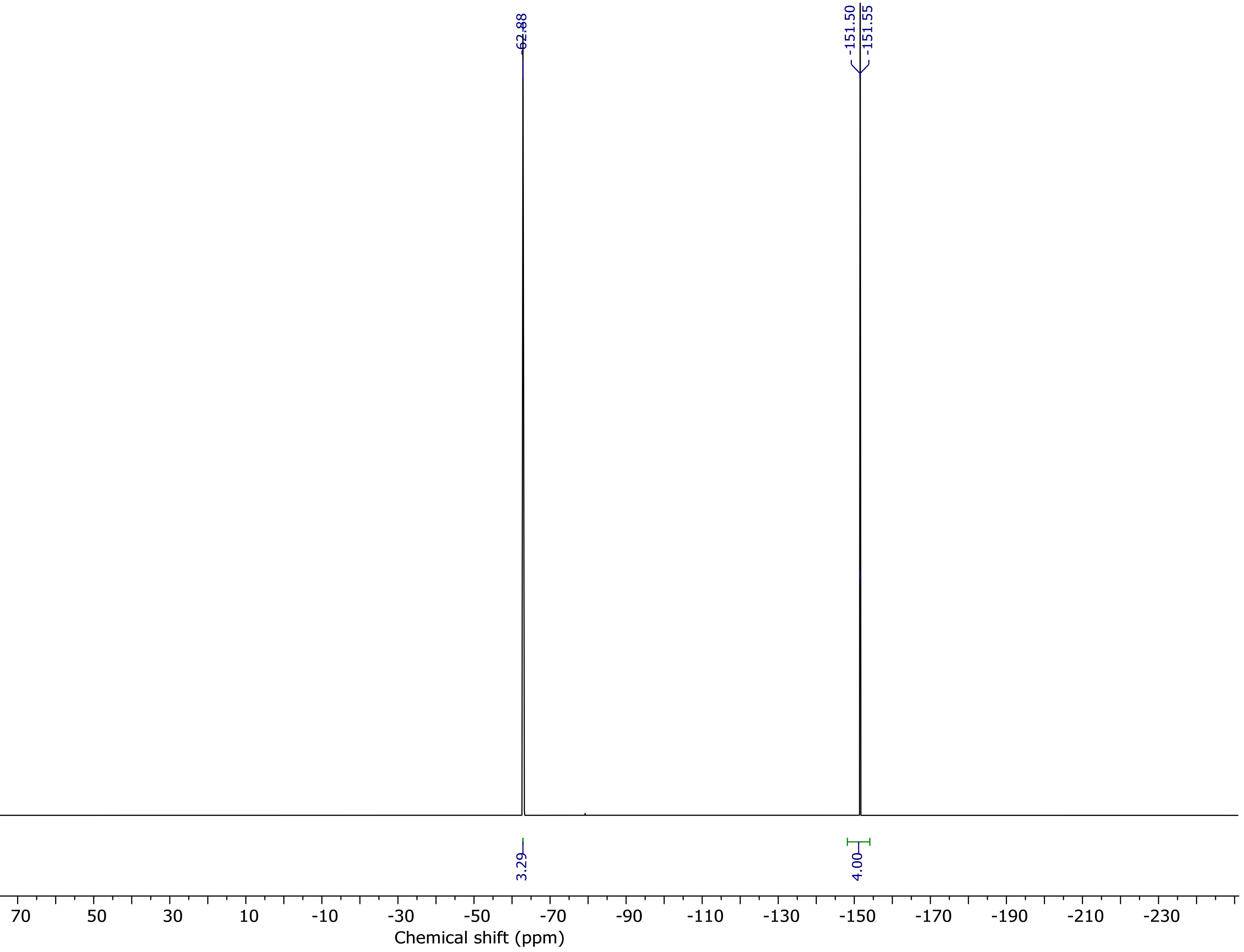

^{1}H NMR of 41a

CD_3CN, 600 MHz, 23 ºC.

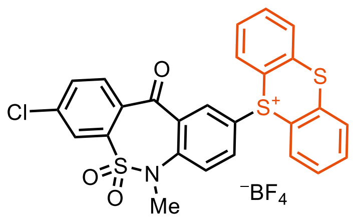

41a

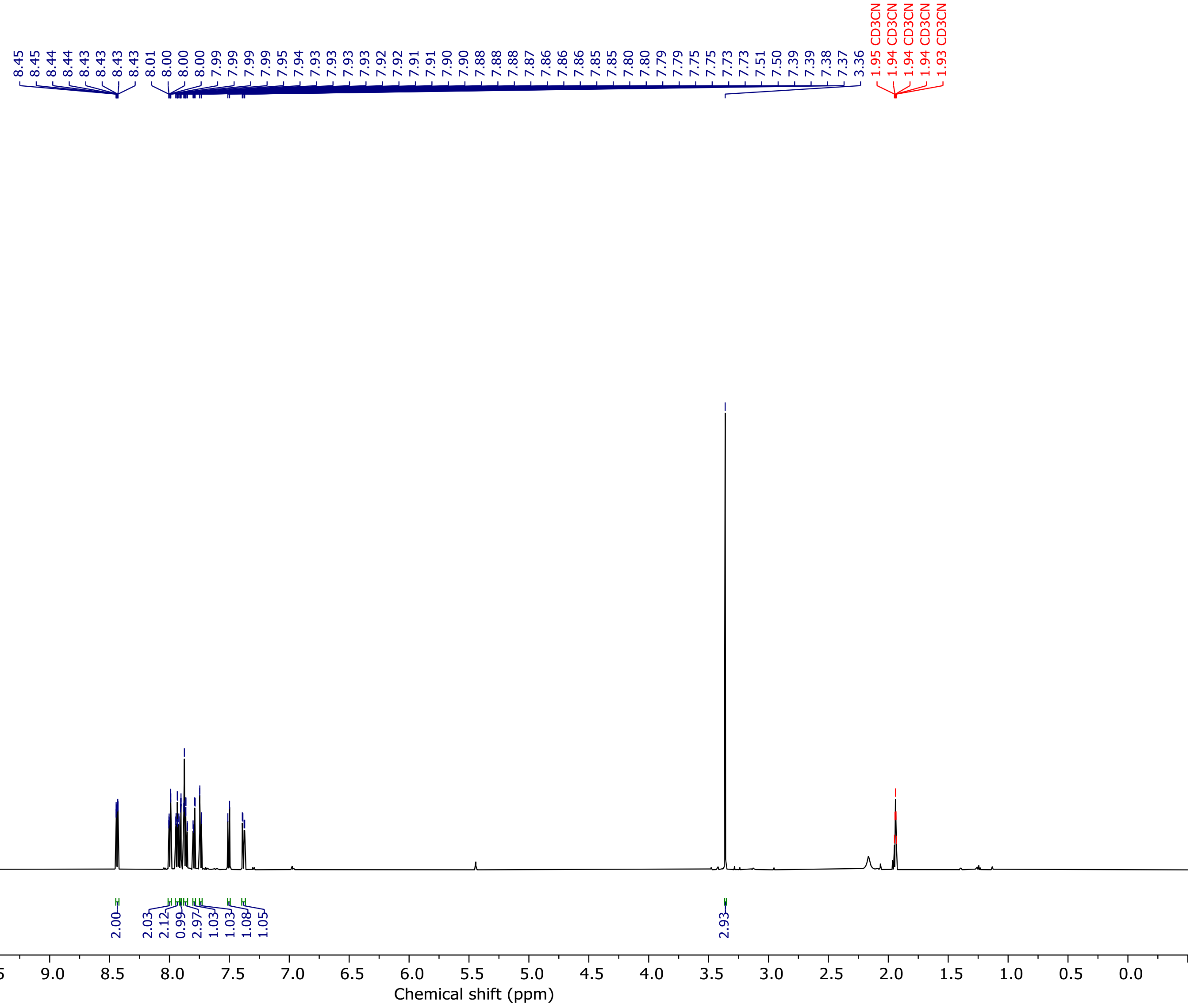

^{13}C NMR of 41a

CD_3CN, 151 MHz, 23 ºC.

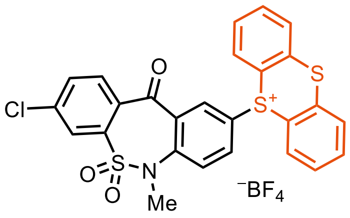

41a

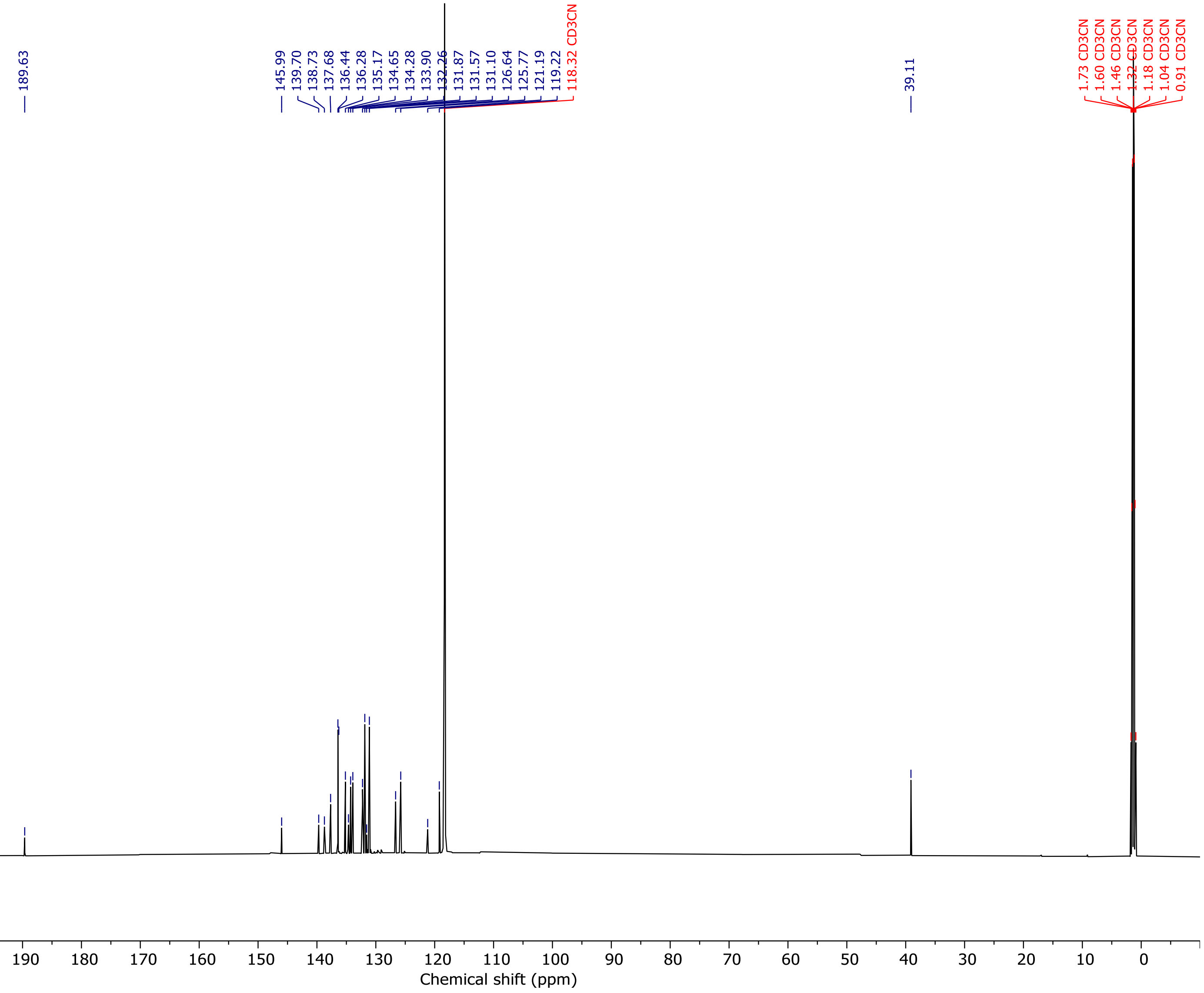

^{19}F NMR of 41a

CD_3CN, 565 MHz, 23 ºC.

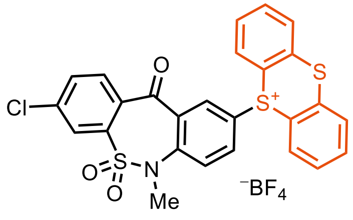

41a

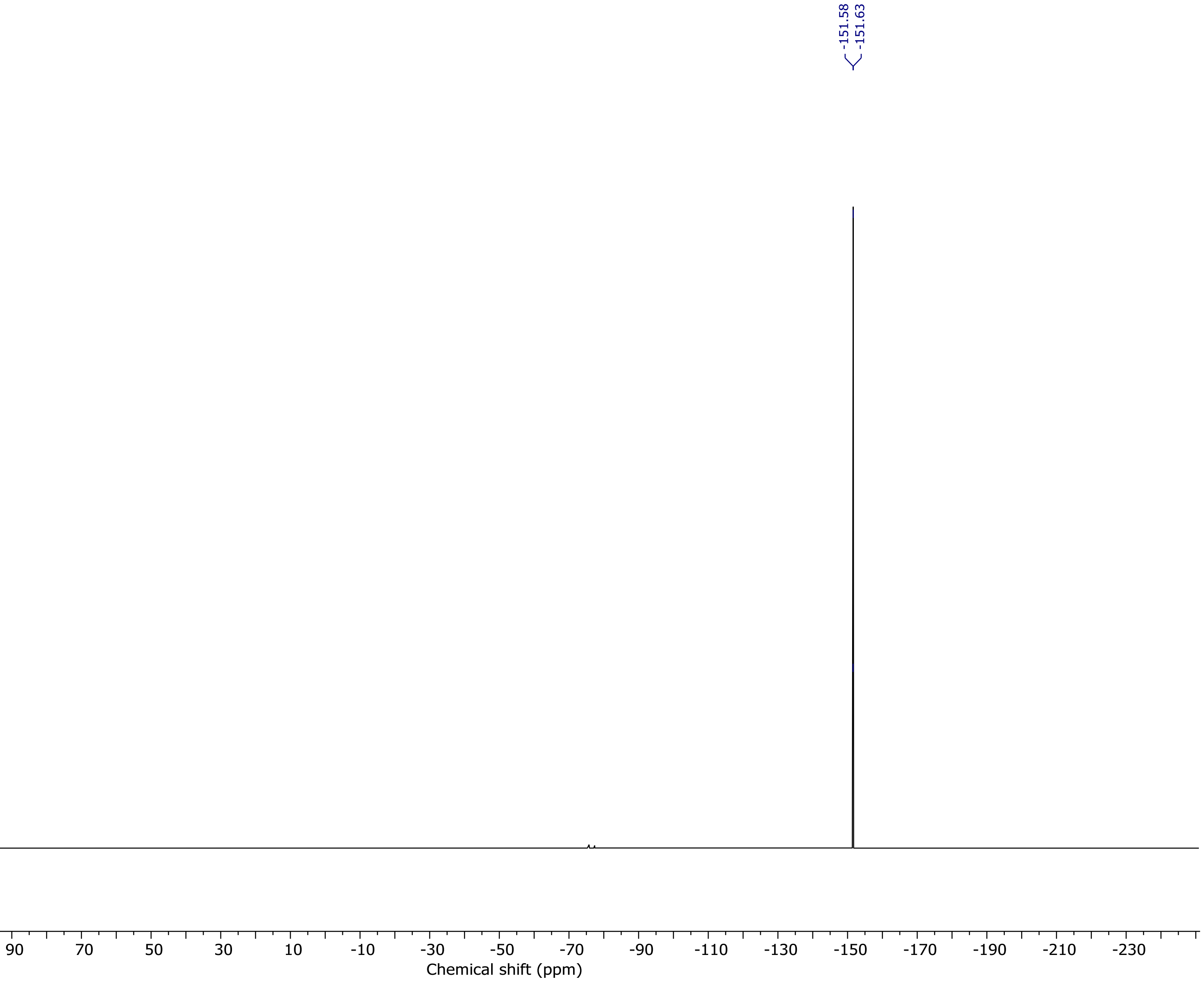

^{1}H NMR of 42a

CD_3CN, 600 MHz, 23 ºC.

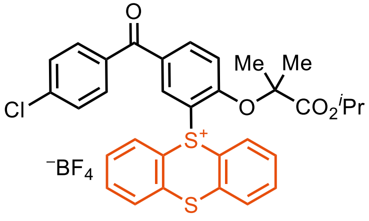

42a

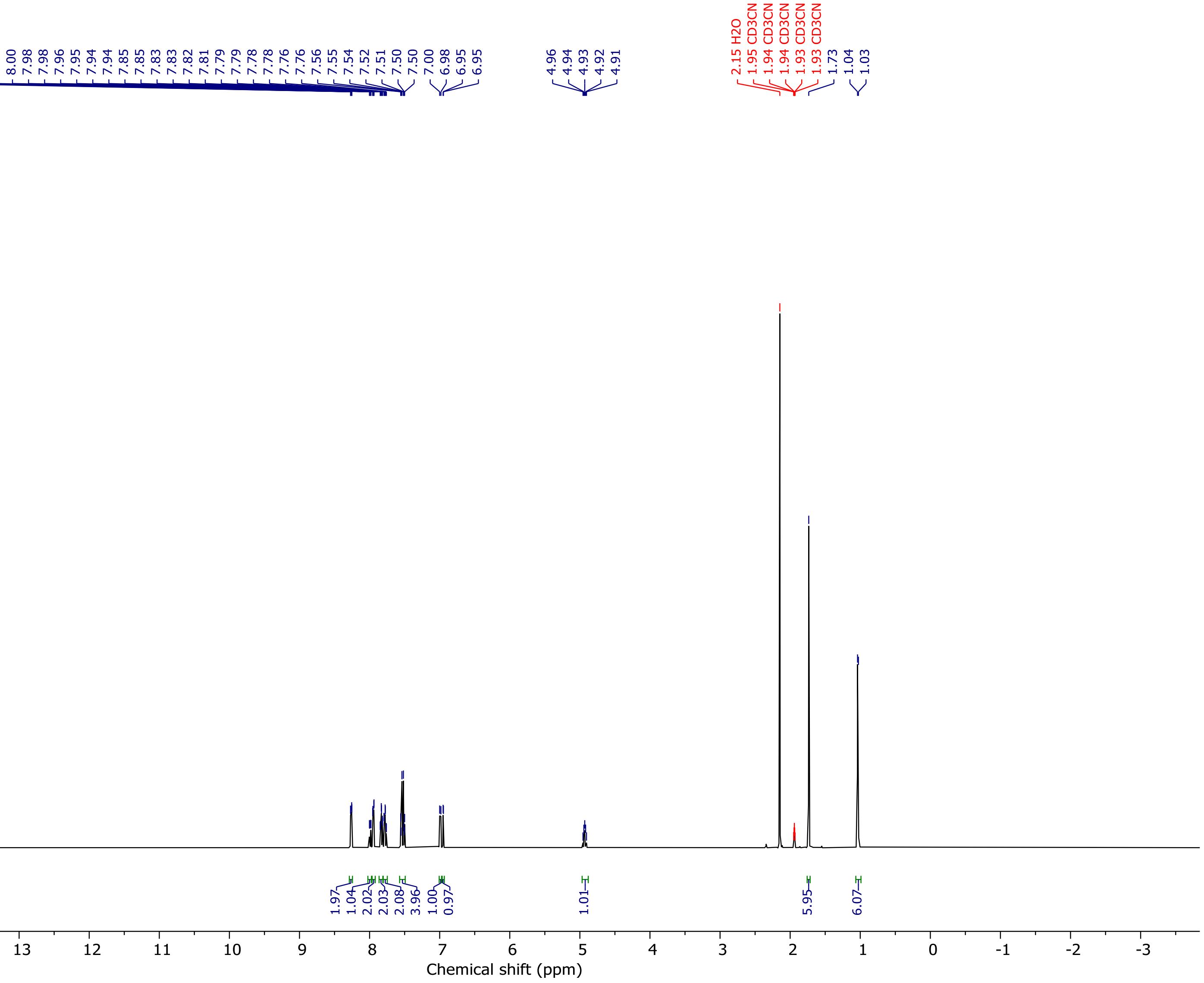

^{13}C NMR of 42a

CD$_3$CN, 151 MHz, 23 ºC.

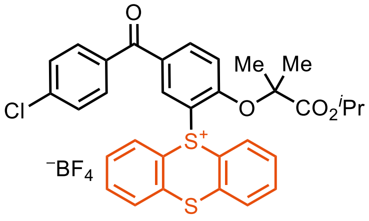

42a

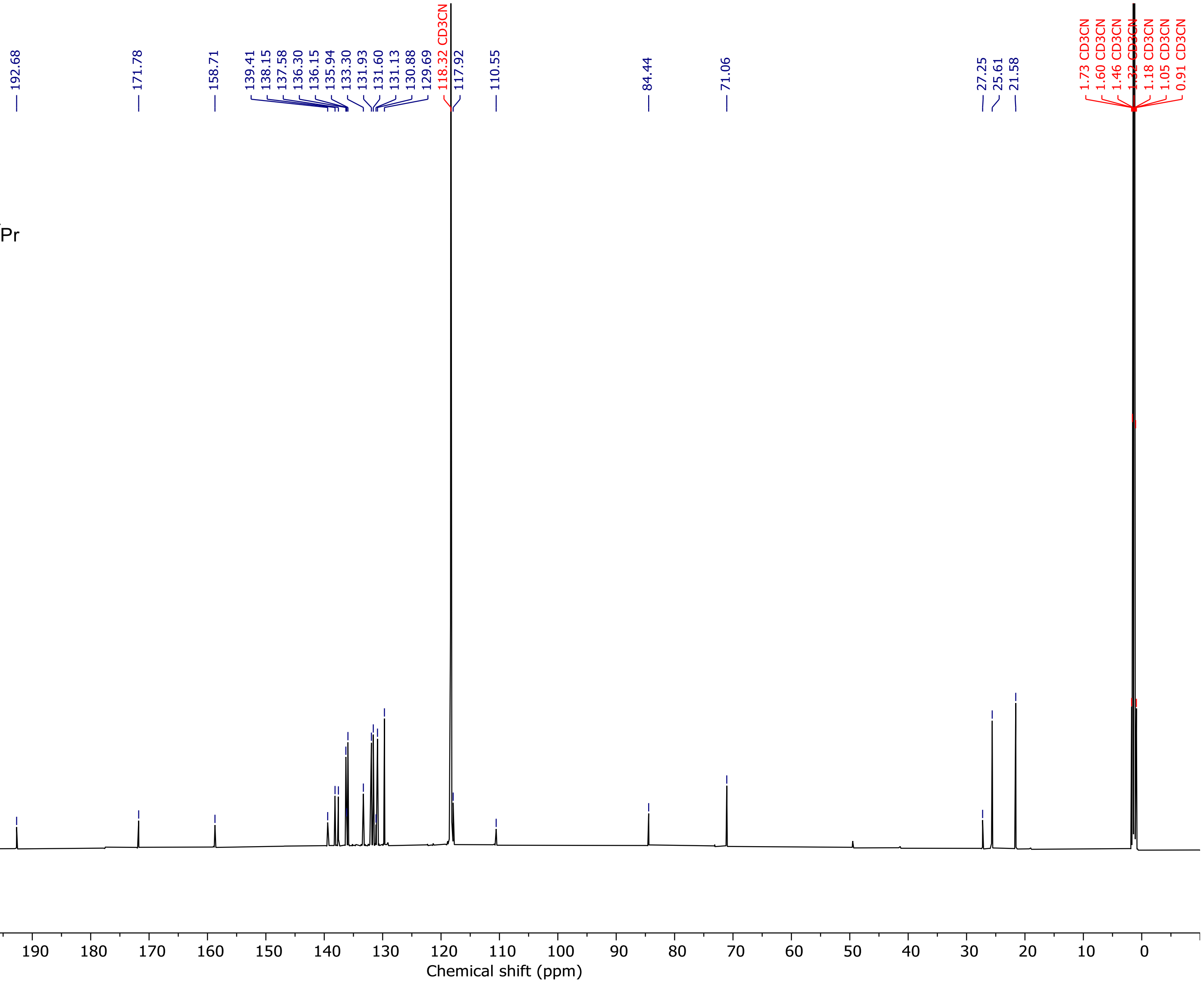

^{19}F NMR of 42a

CD_3CN, 565 MHz, 23 ºC.

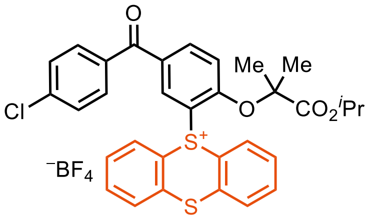

42a

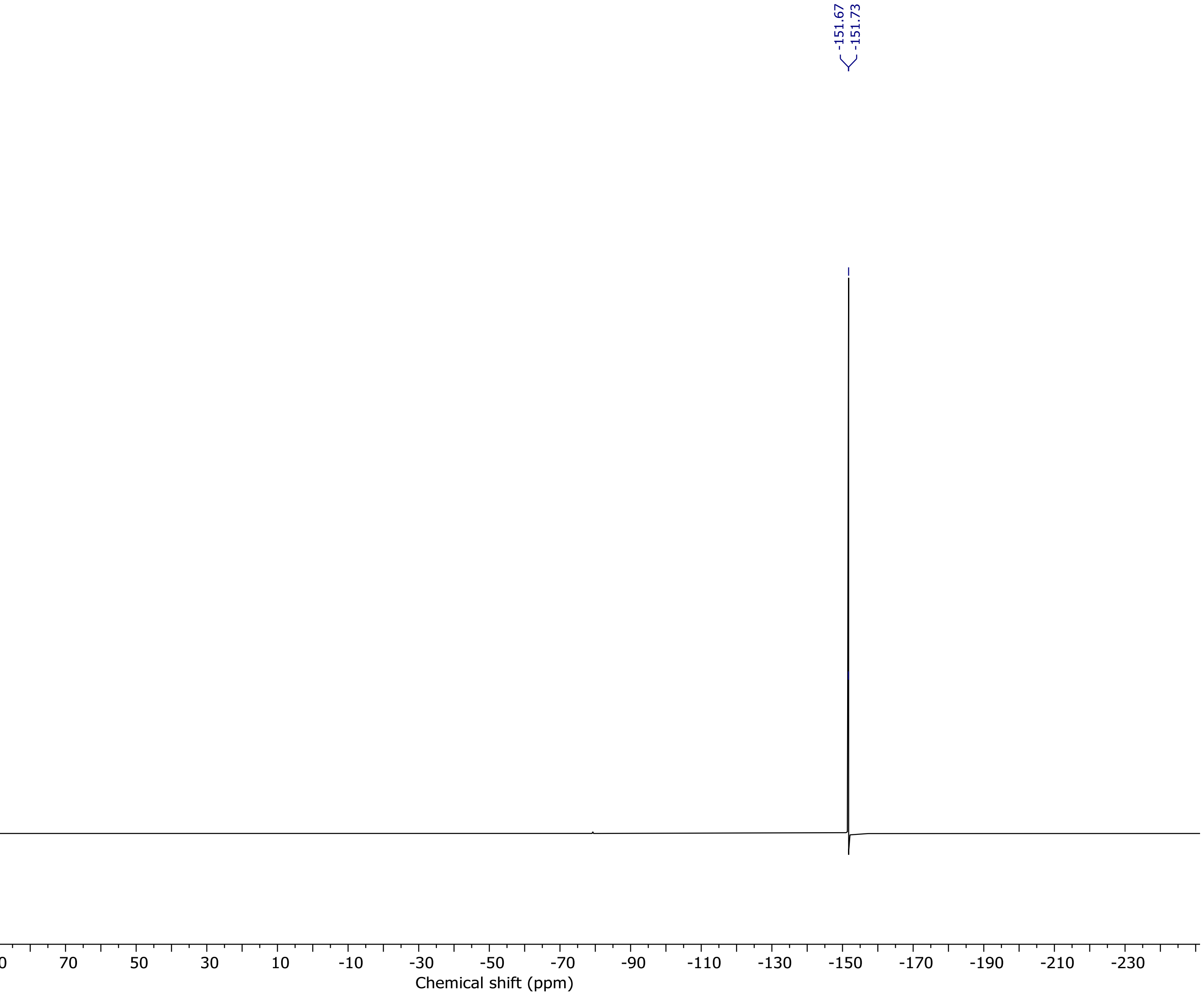

^{1}H NMR of 43a

CD_3CN, 600 MHz, 23 ºC.

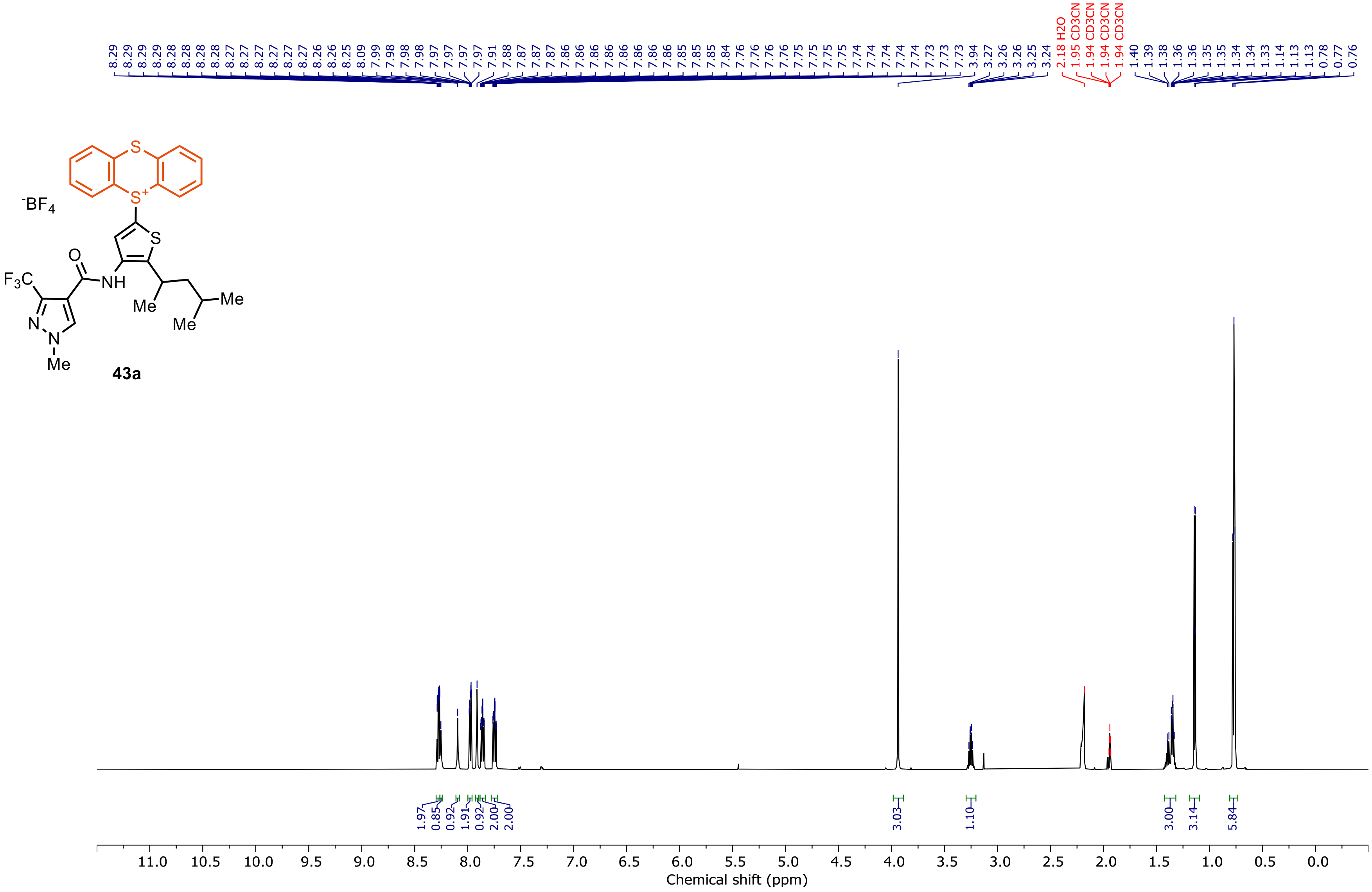

^{13}C NMR of 43a

CD_3CN, 151 MHz, 23 ºC.

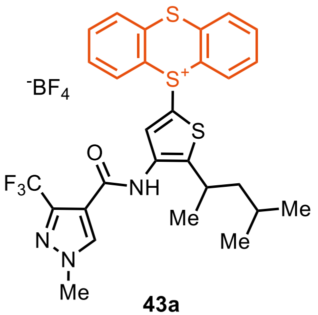

43a

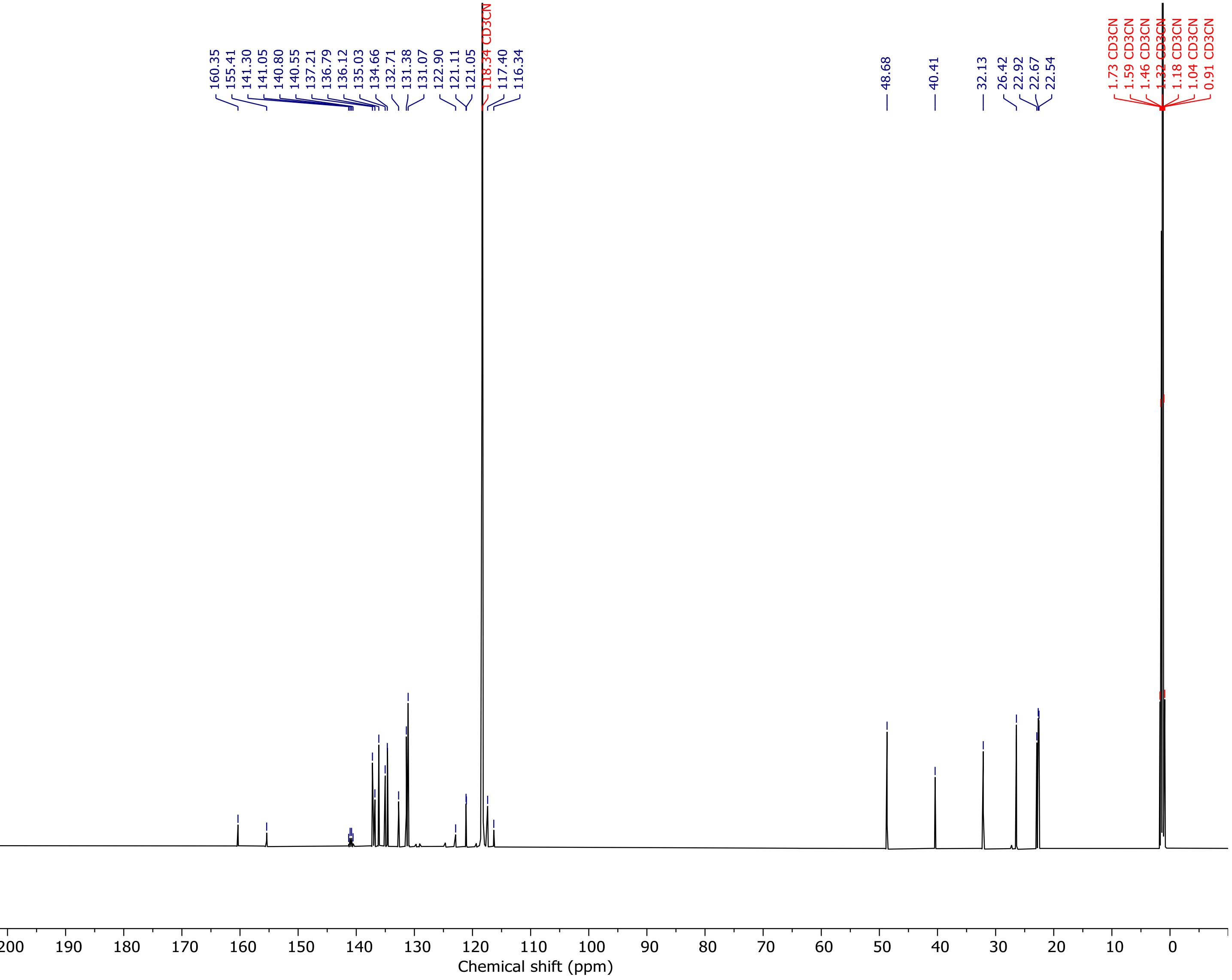

^{19}F NMR of 43a

CD_3CN, 565 MHz, 23 ºC.

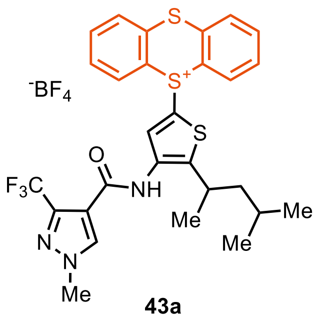

43a

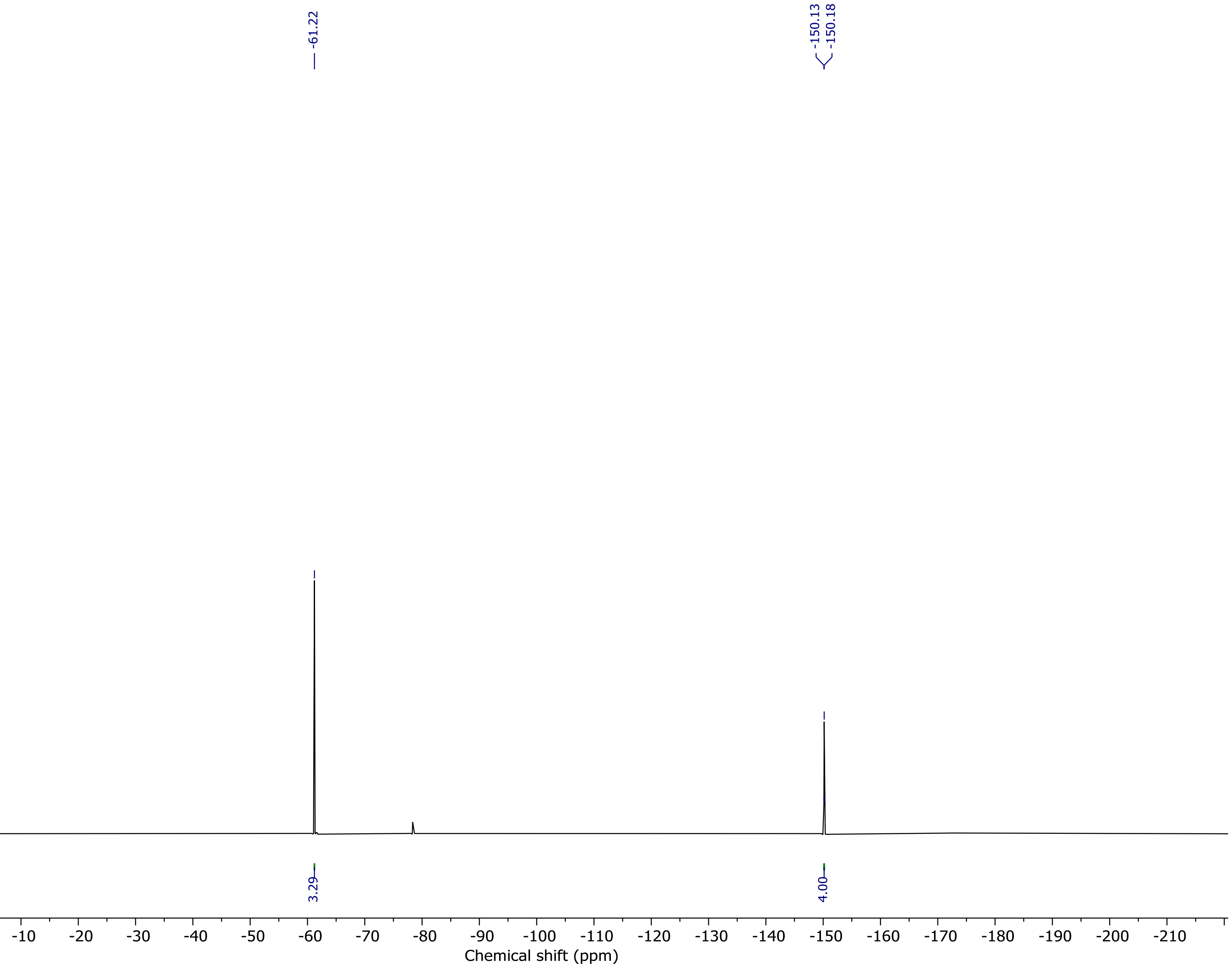

^{1}H NMR of 44a

$CDCl_3$, 600 MHz, 23 ºC.

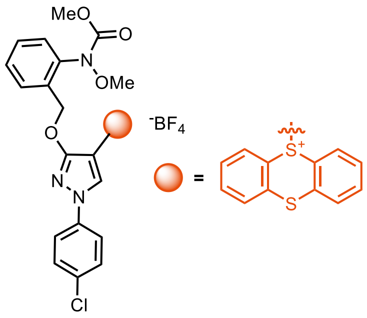

44a

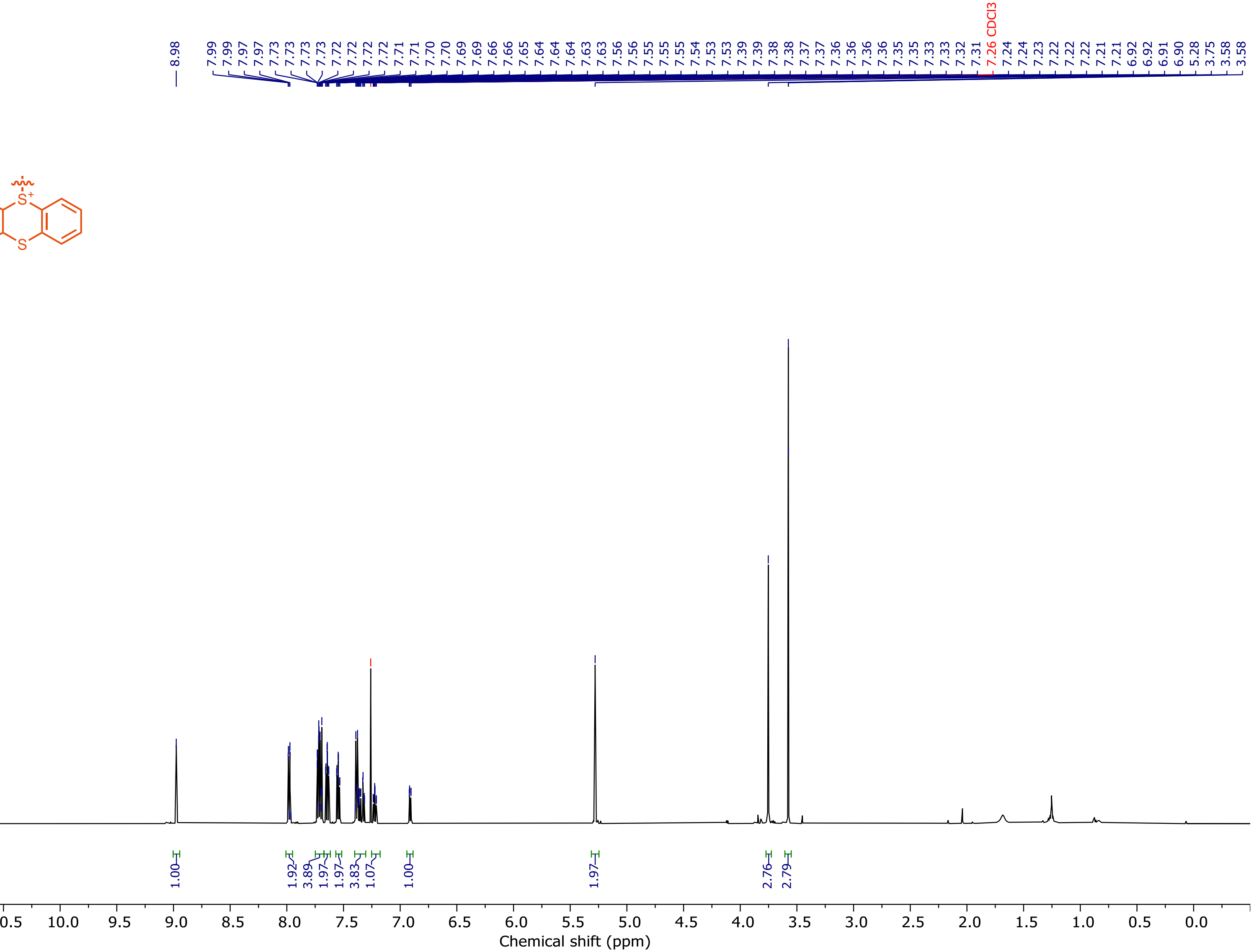

^{13}C NMR of 44a

$CDCl_3$, 151 MHz, 23 ºC.

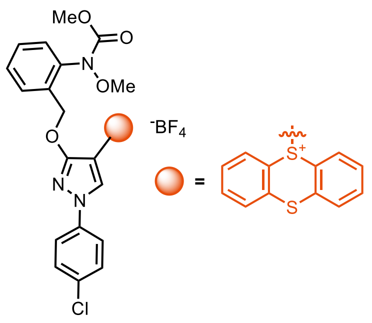

44a

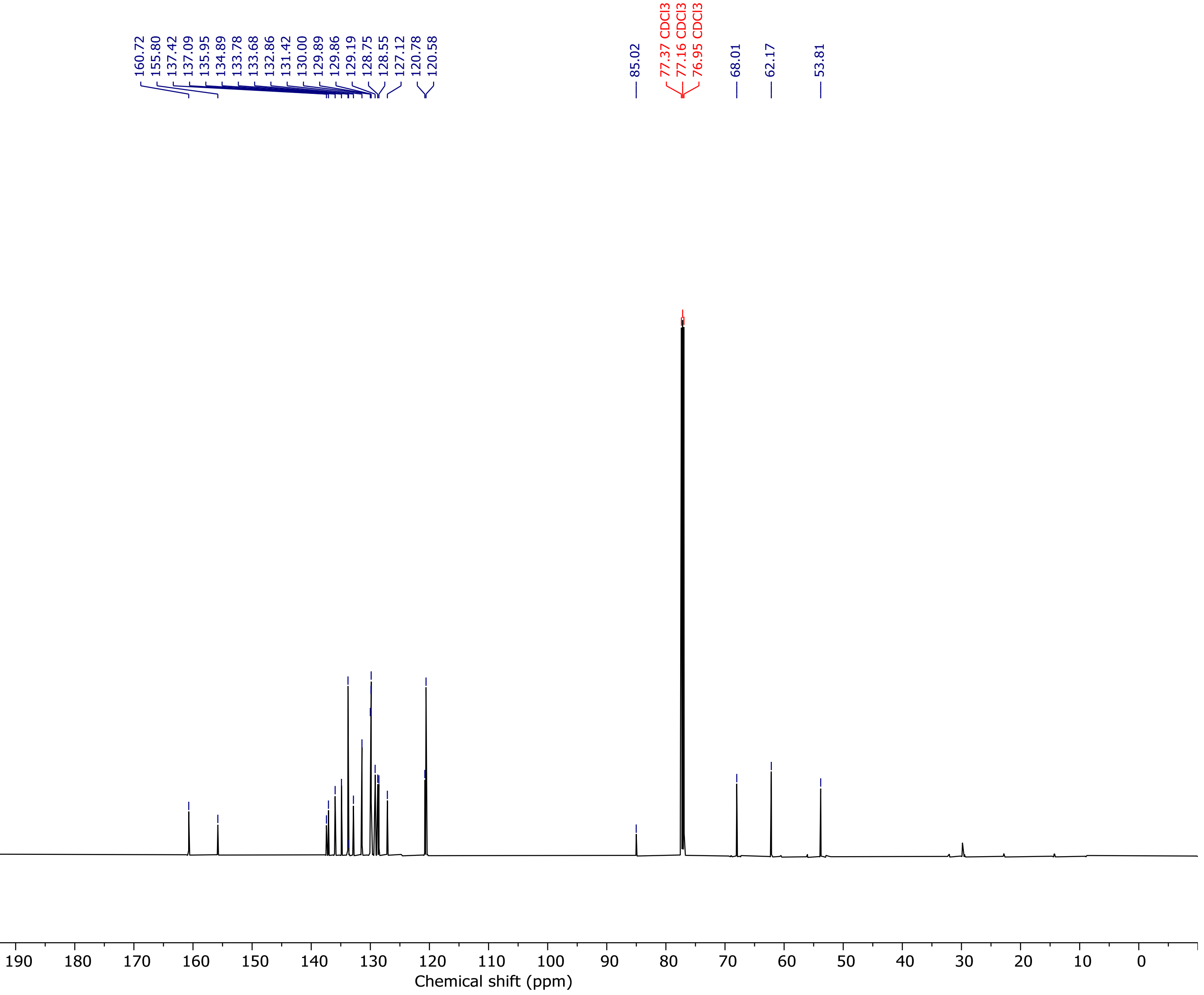

^{19}F NMR of 44a

CDCl$_3$, 565 MHz, 23 ºC.

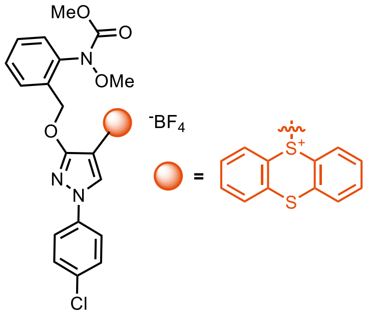

44a

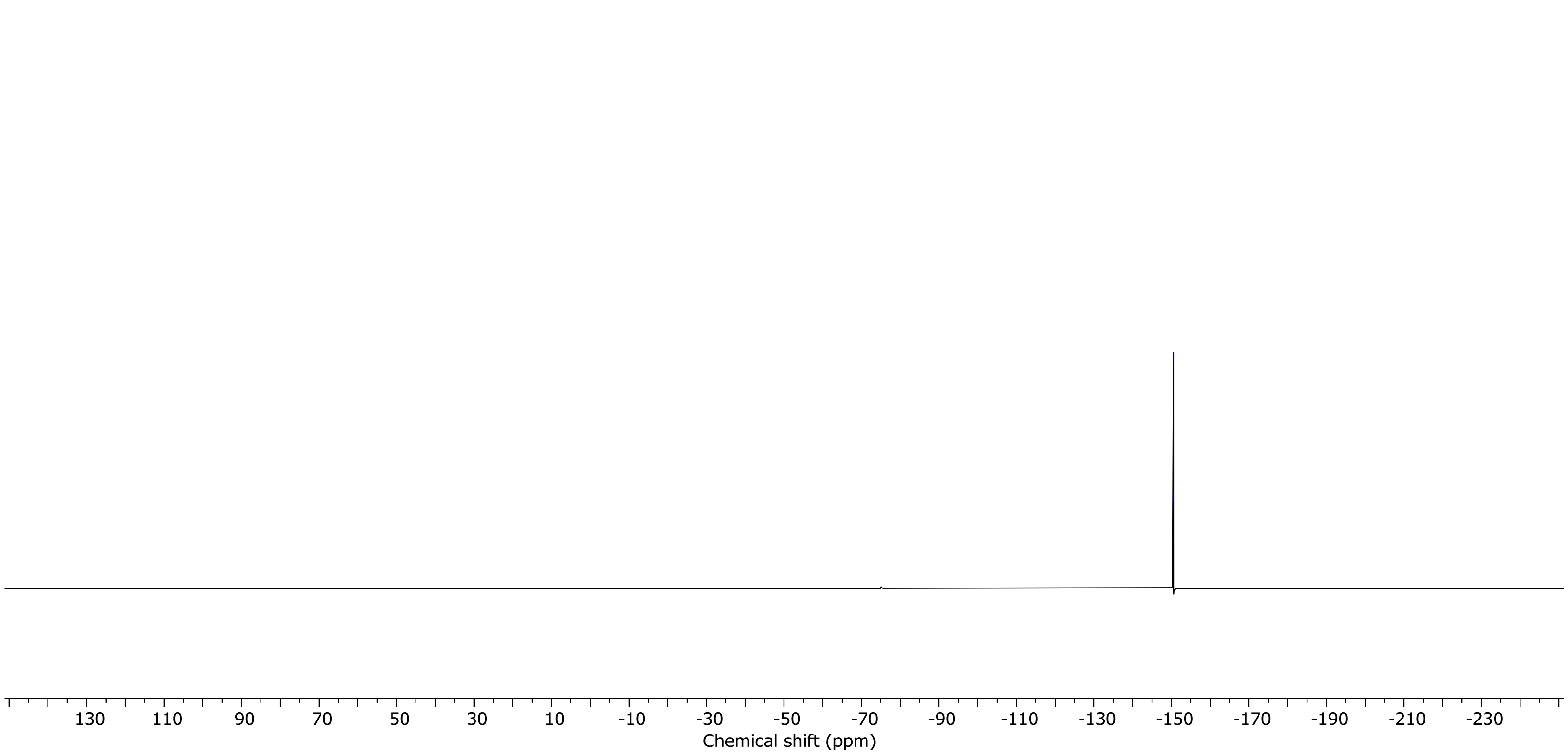

^{1}H NMR of 45a

CD_3CN, 500 MHz, 23 ºC.

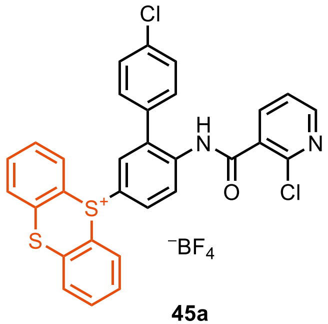

45a

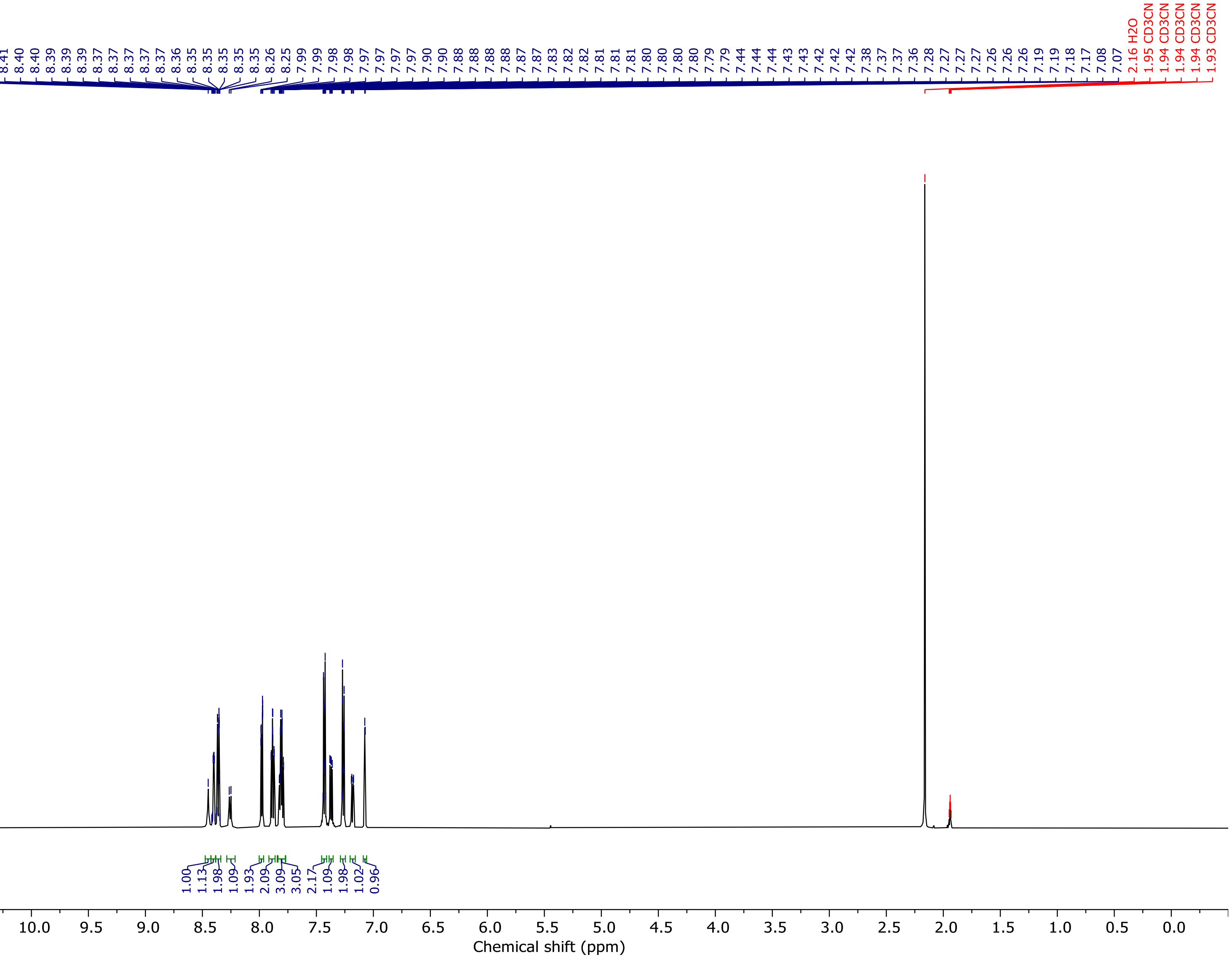

^{13}C NMR of 45a

CD_3CN, 126 MHz, 23 ºC.

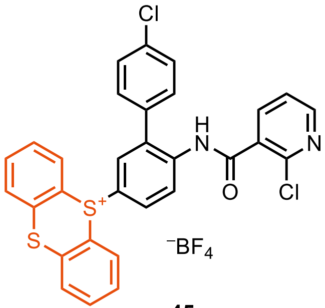

45a

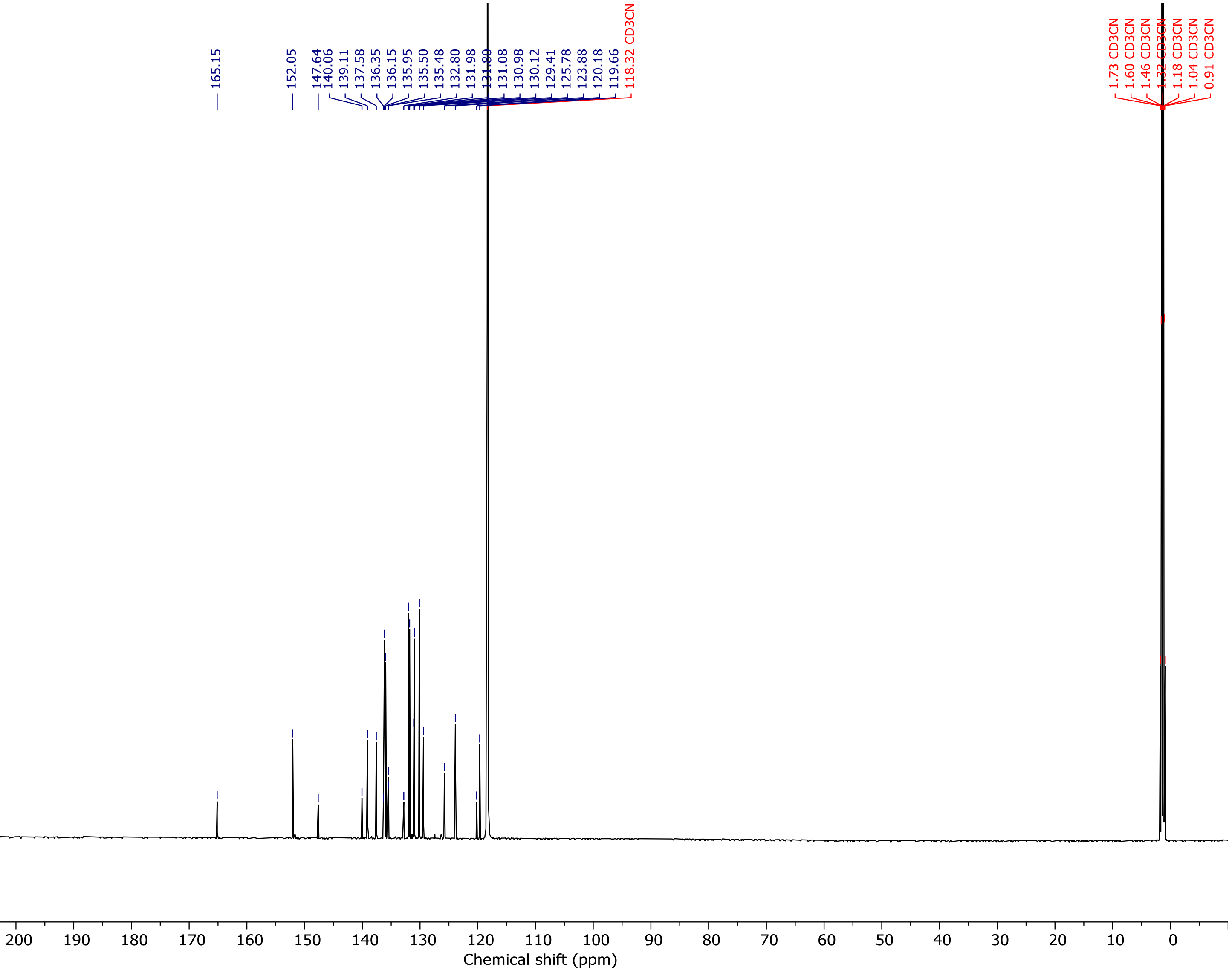

^{19}F NMR of 45a

CD_3CN, 471 MHz, 23 ºC.

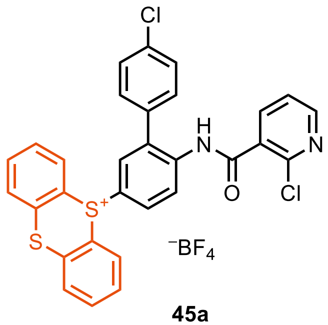

45a

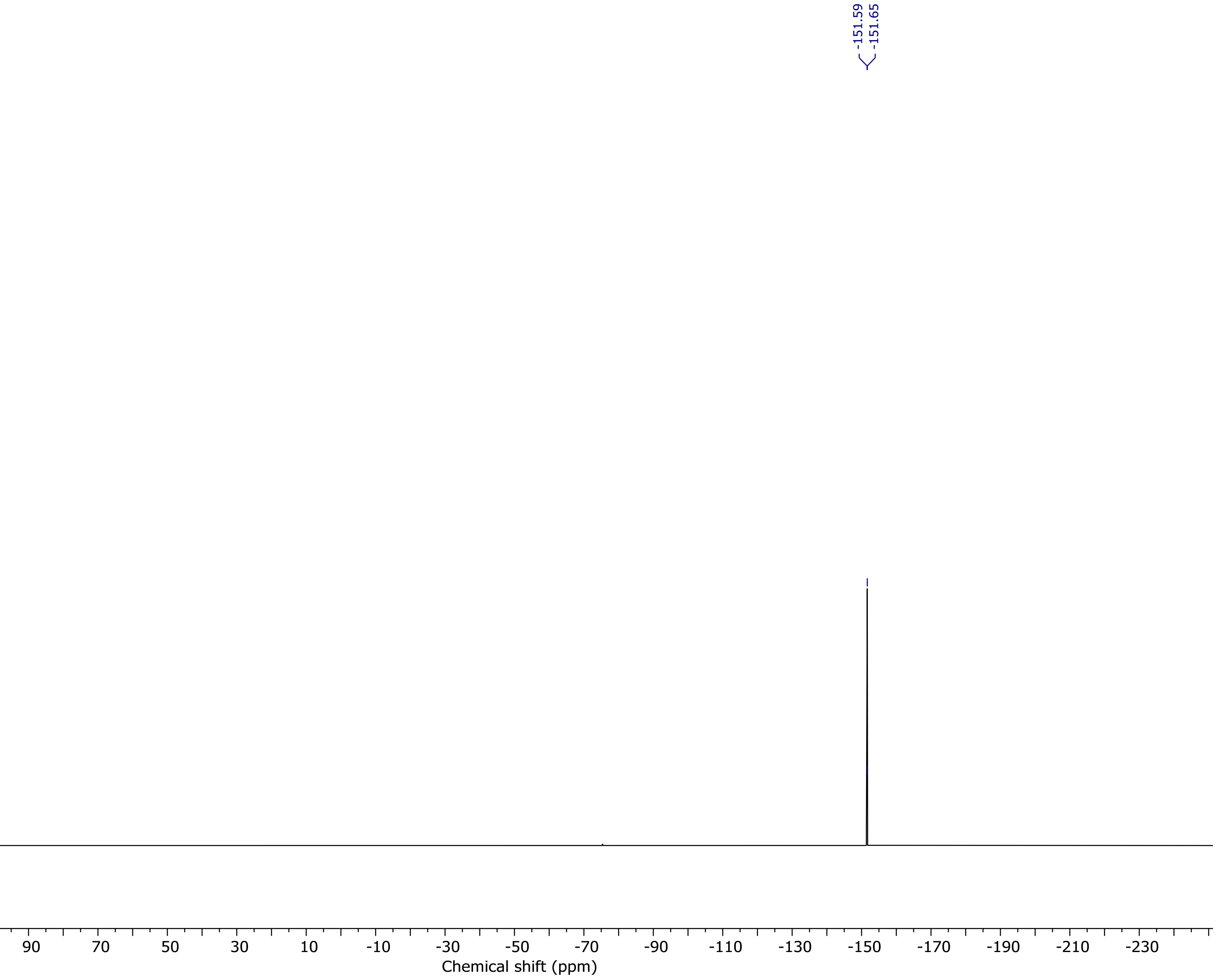

^{1}H NMR of 46a

CD_3CN, 600 MHz, 23 ºC.

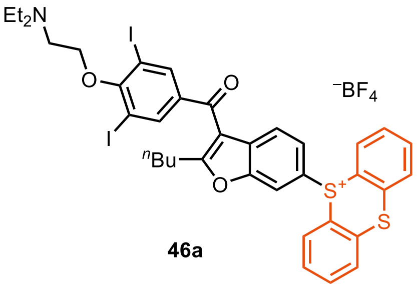

46a

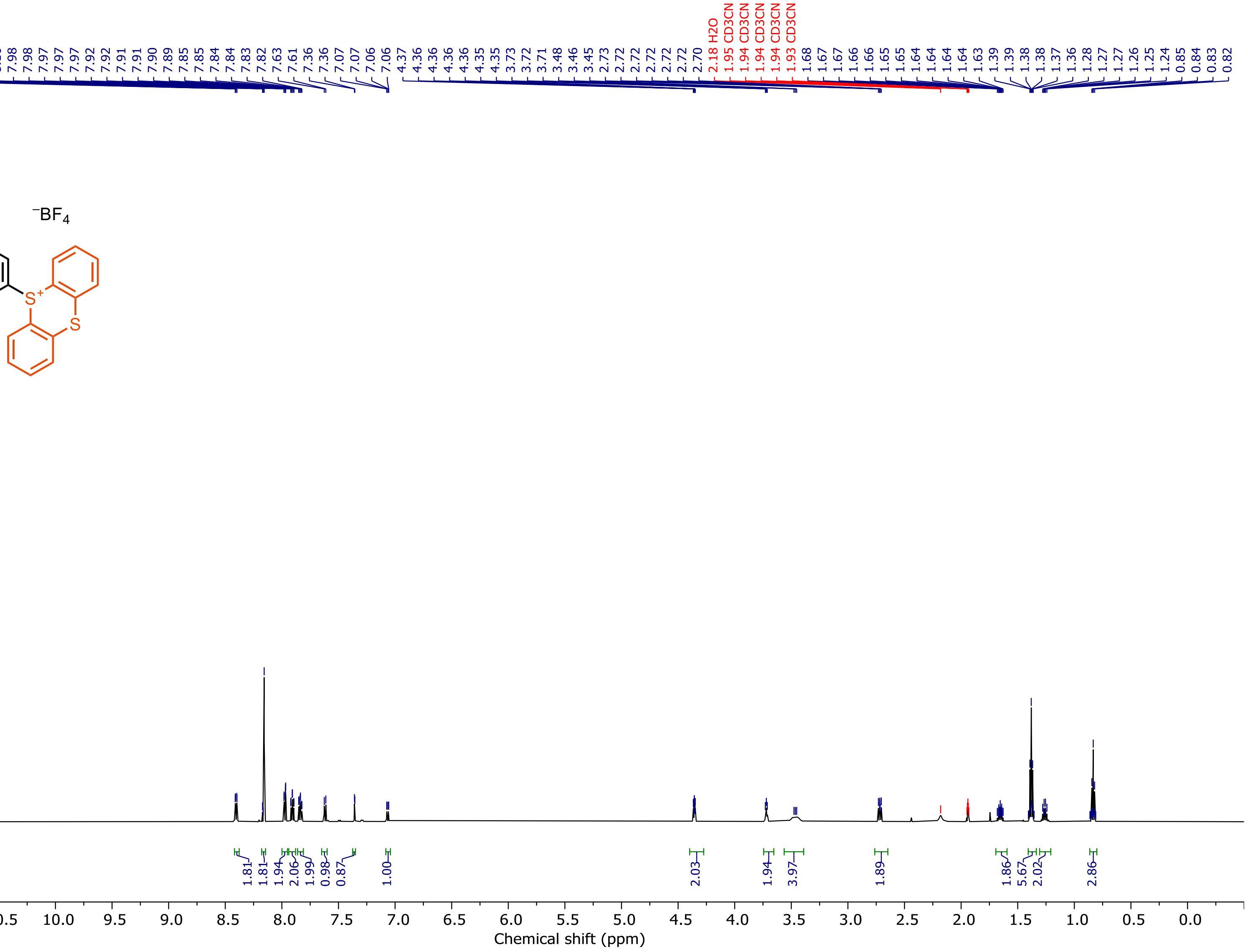

^{13}C NMR of 46a

CD_3CN, 151 MHz, 23 ºC.

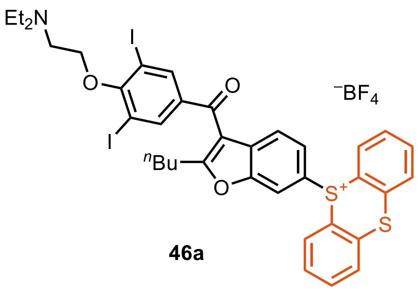

46a

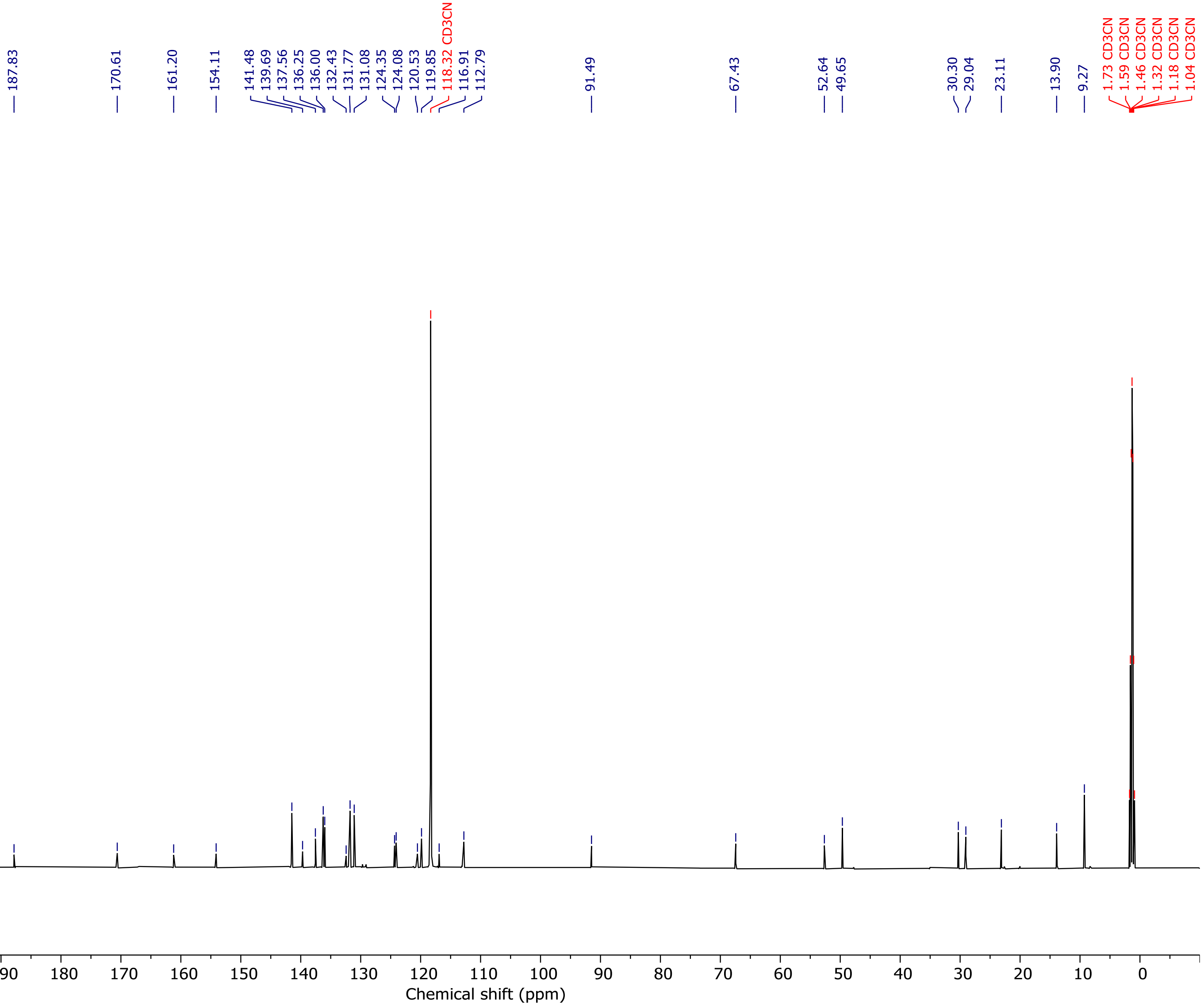

^{19}F NMR of 46a

CD_3CN, 565 MHz, 23 ºC.

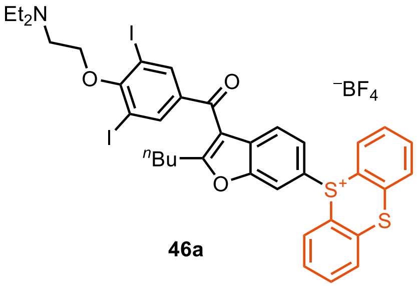

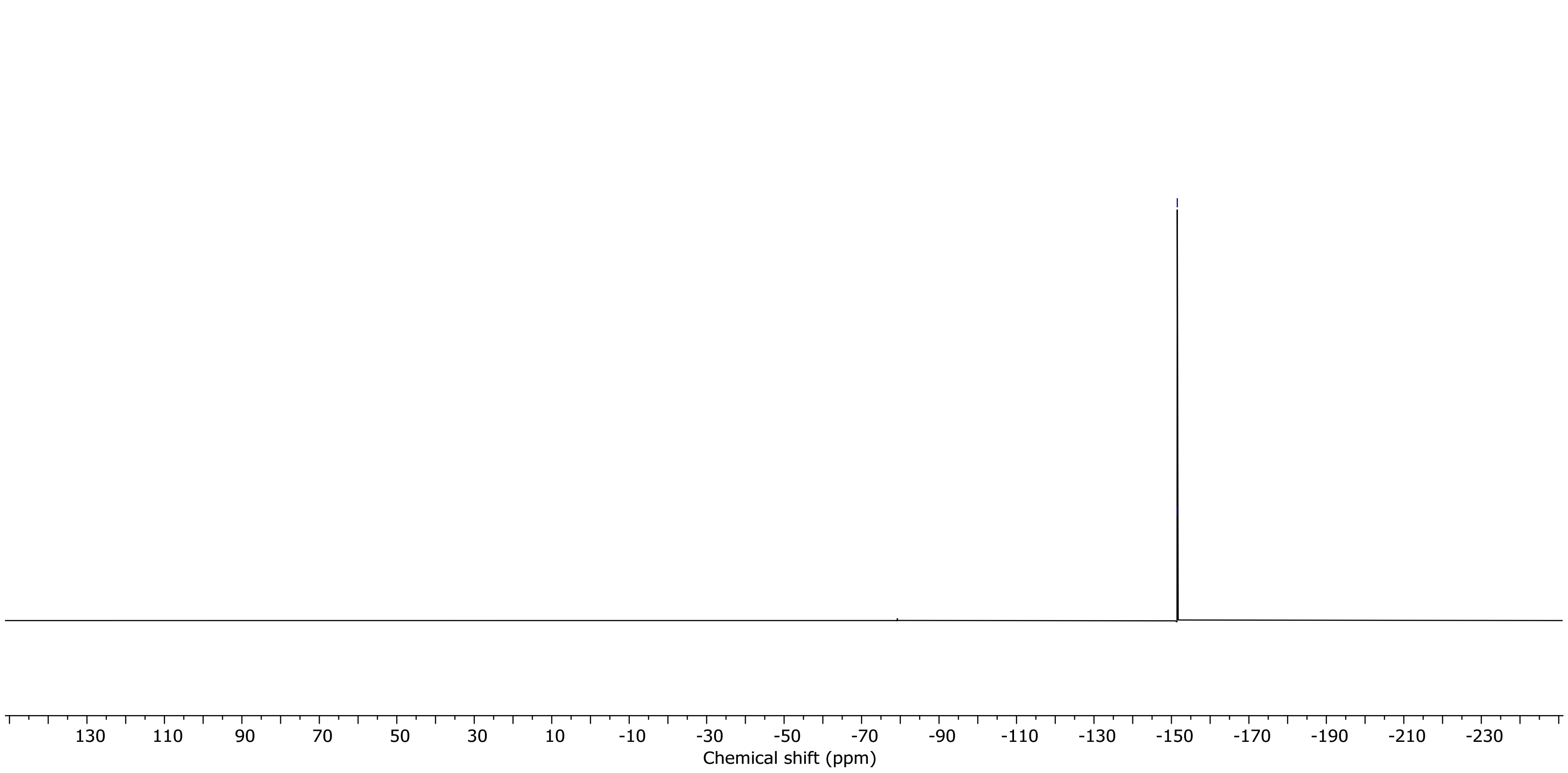

^{1}H NMR of 47a

CD_3CN, 300 MHz, 23 ºC.

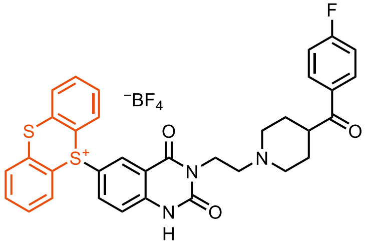

47a

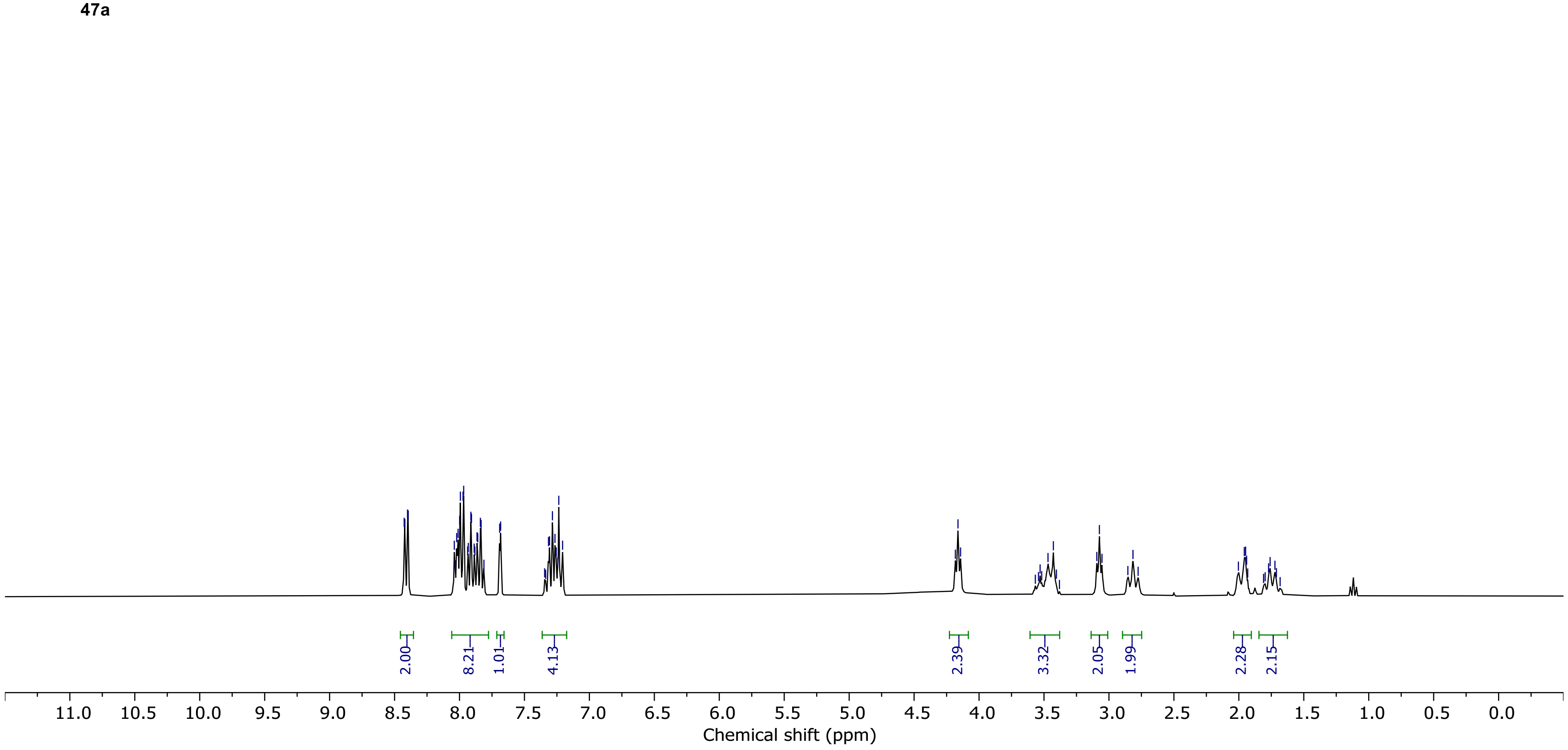

^{13}C NMR of 47a

CD_3CN, 75 MHz, 23 ºC.

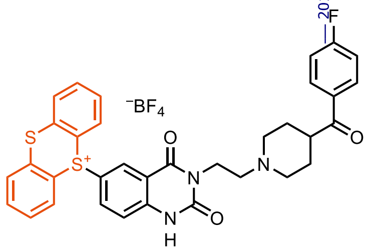

47a

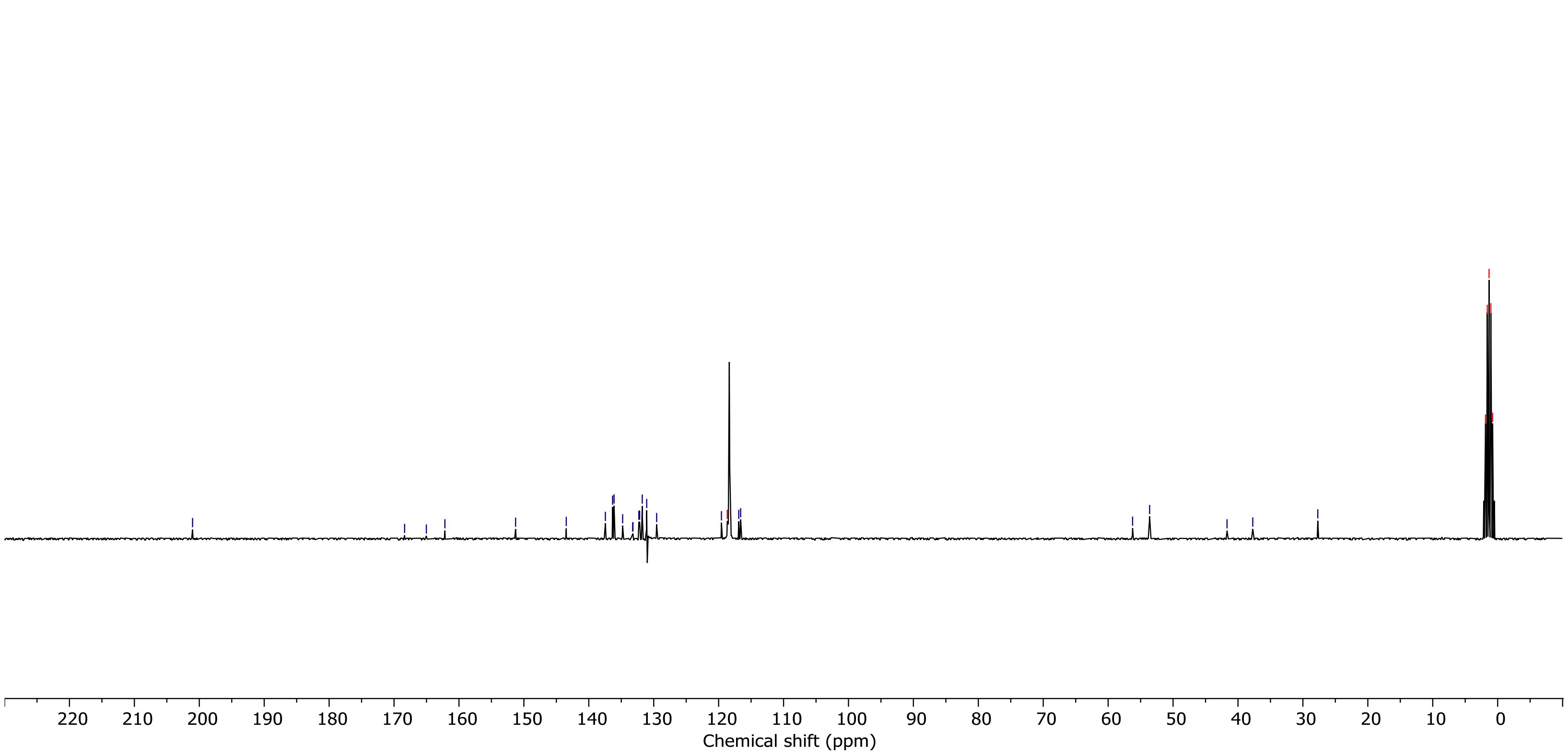

^{19}F NMR of 47a

CD_3CN, 828 MHz, 23 ºC.

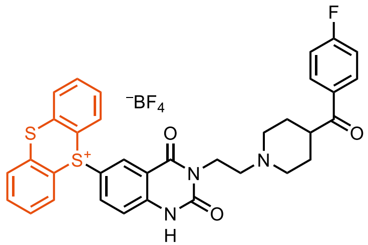

47a

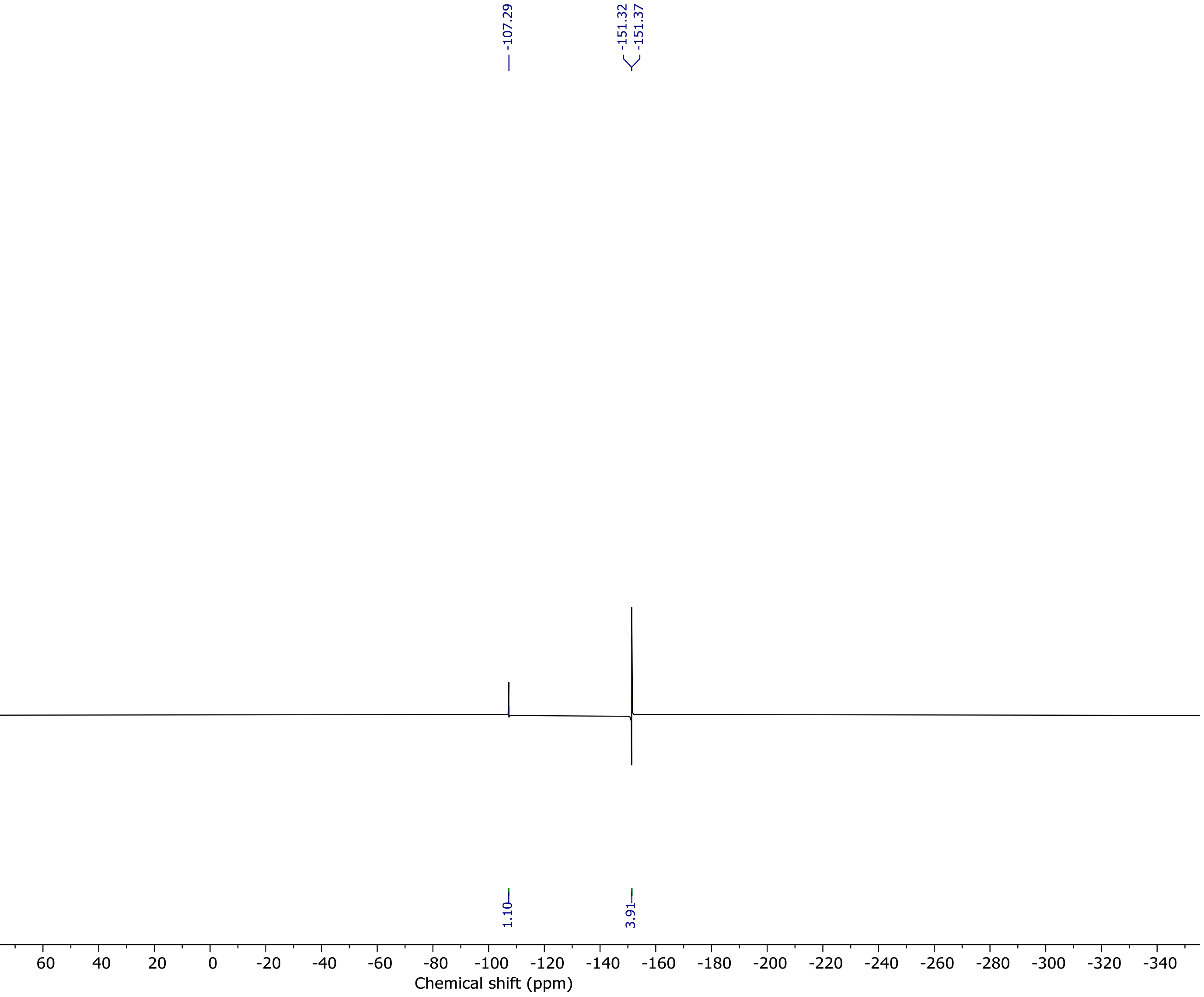

^{1}H NMR of 48-S-I

$CDCl_3$, 500 MHz, 23 ºC.

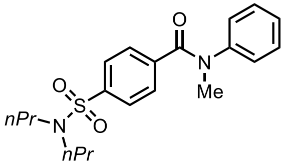

48-S-I

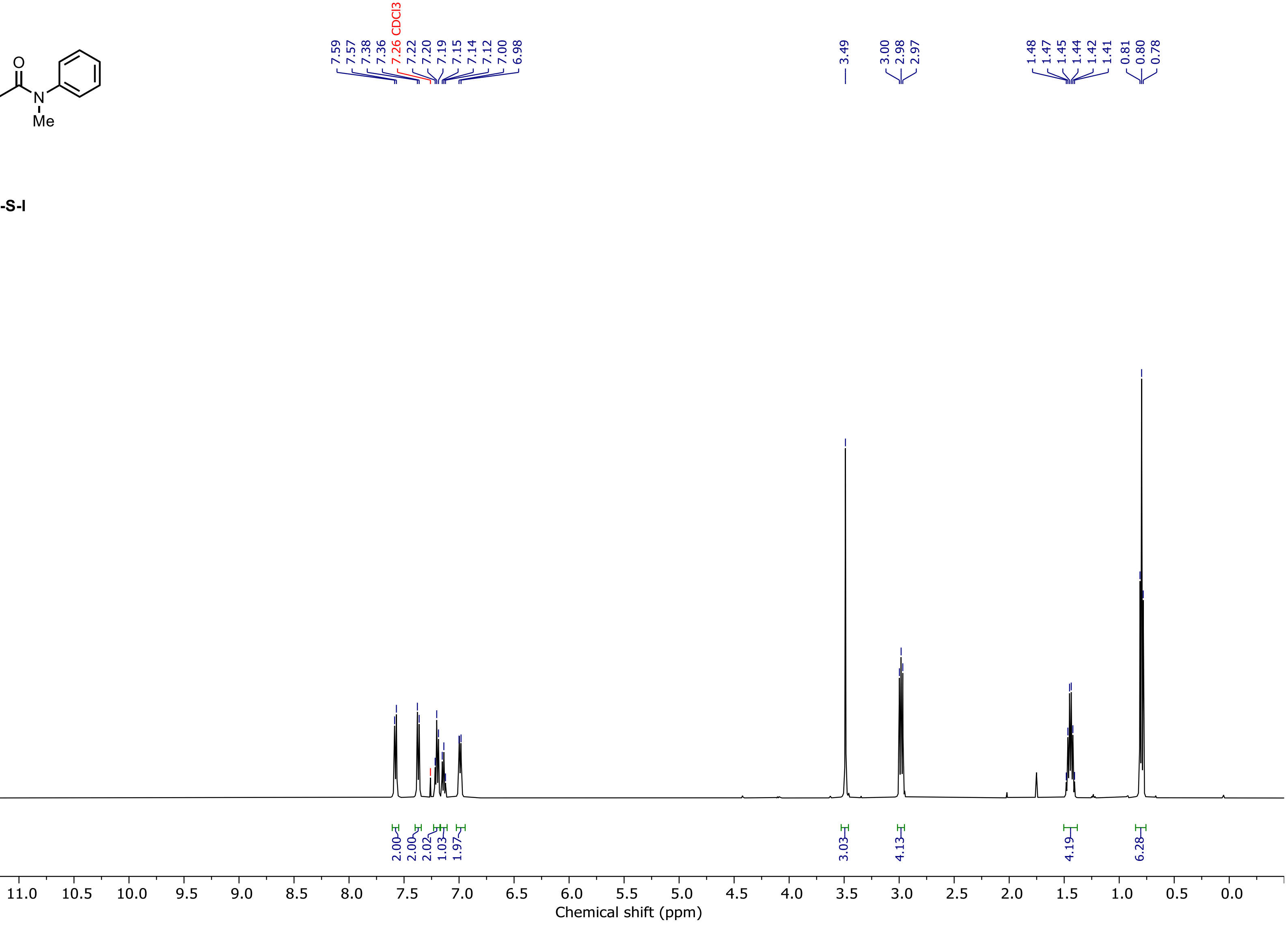

^{13}C NMR of 48-S-I

$CDCl_3$, 126 MHz, 23 ºC.

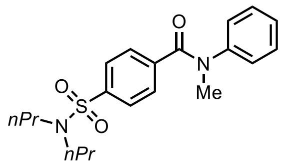

48-S-I

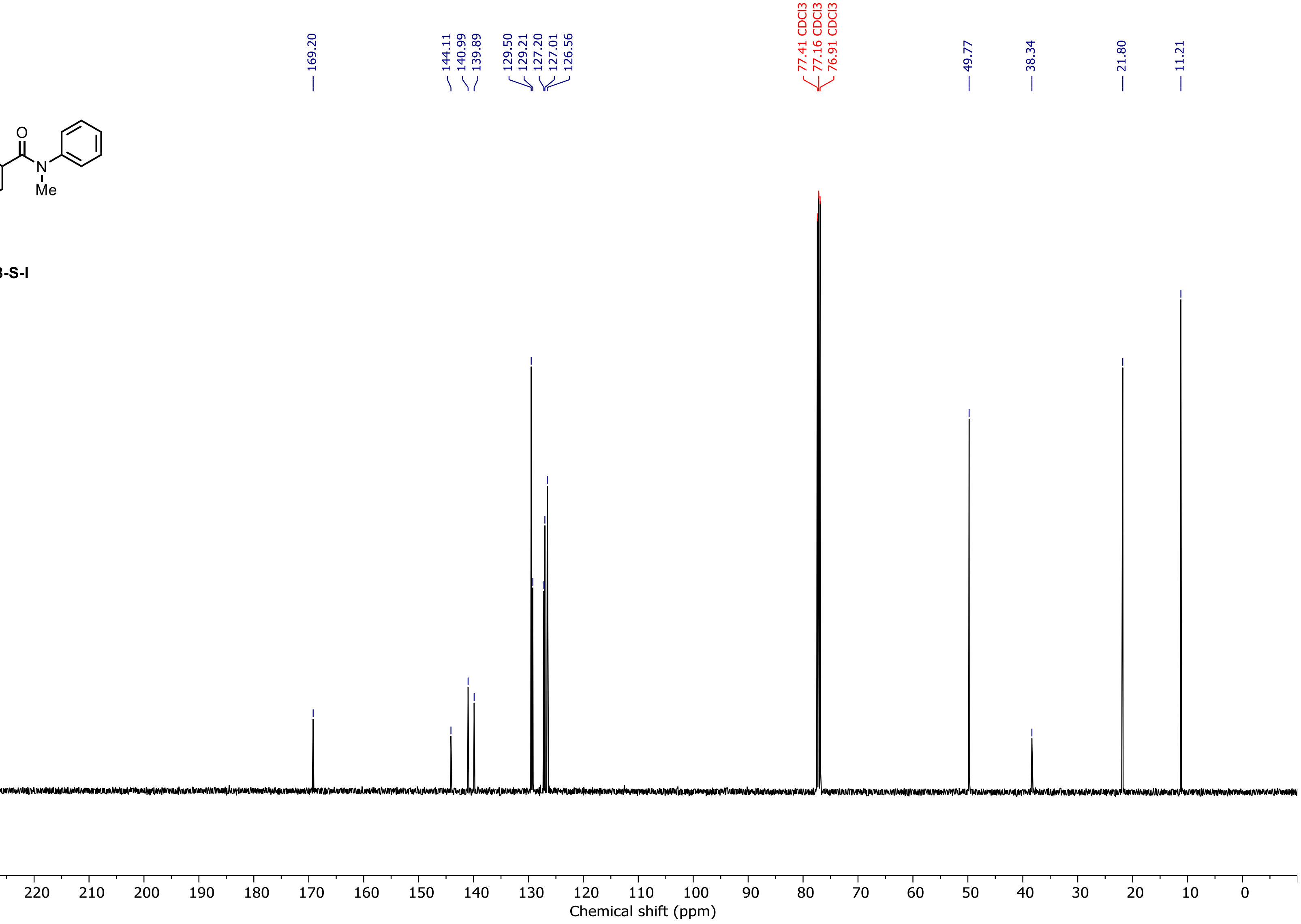

^{1}H NMR of 48

CDCl$_3$, 500 MHz, 23 ºC.

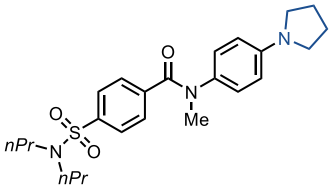

48

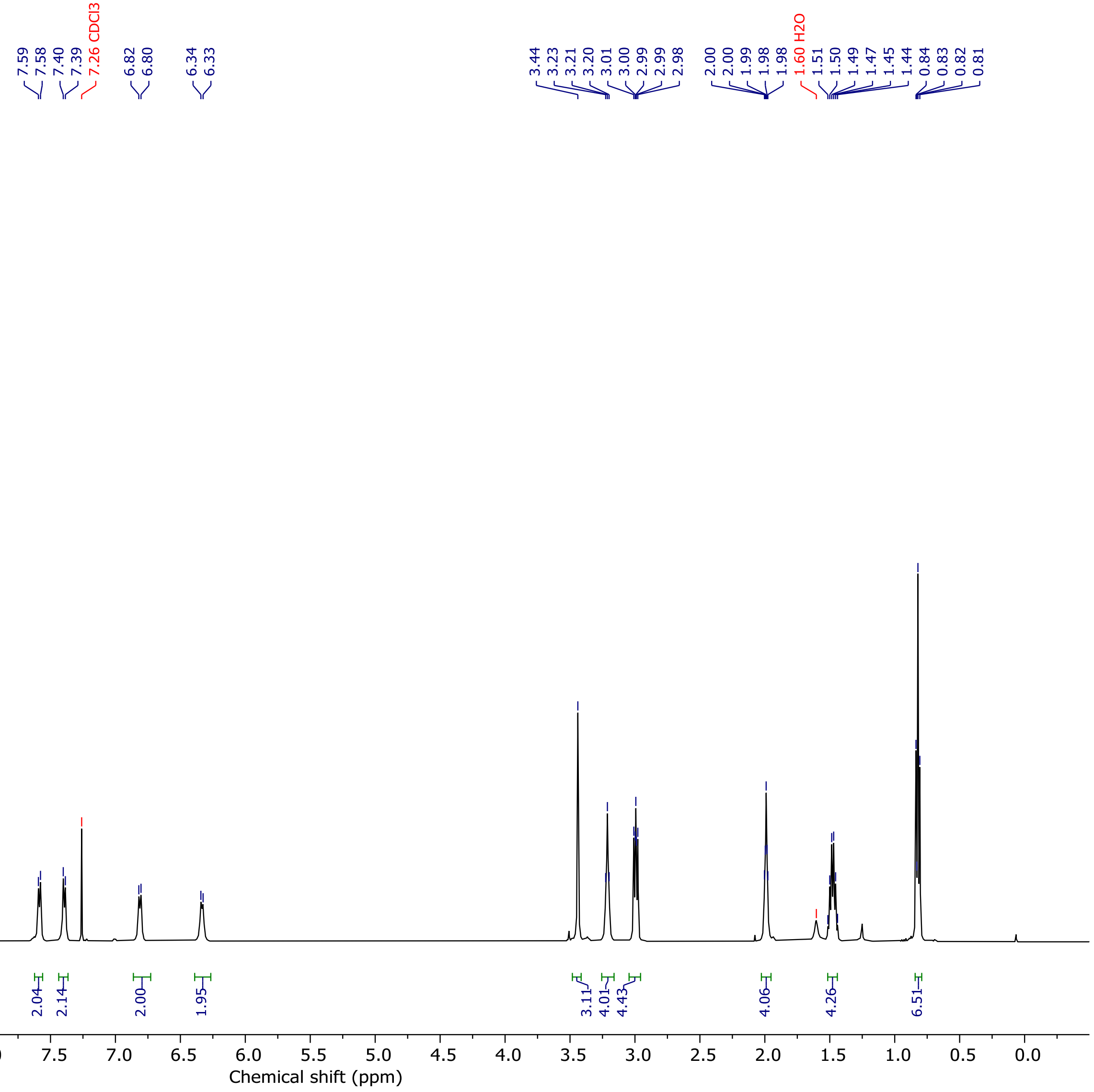

^{13}C NMR of 48

$CDCl_3$, 126 MHz, 23 ºC.

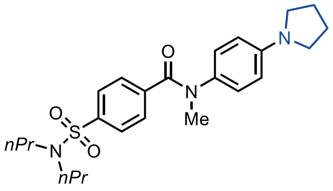

48

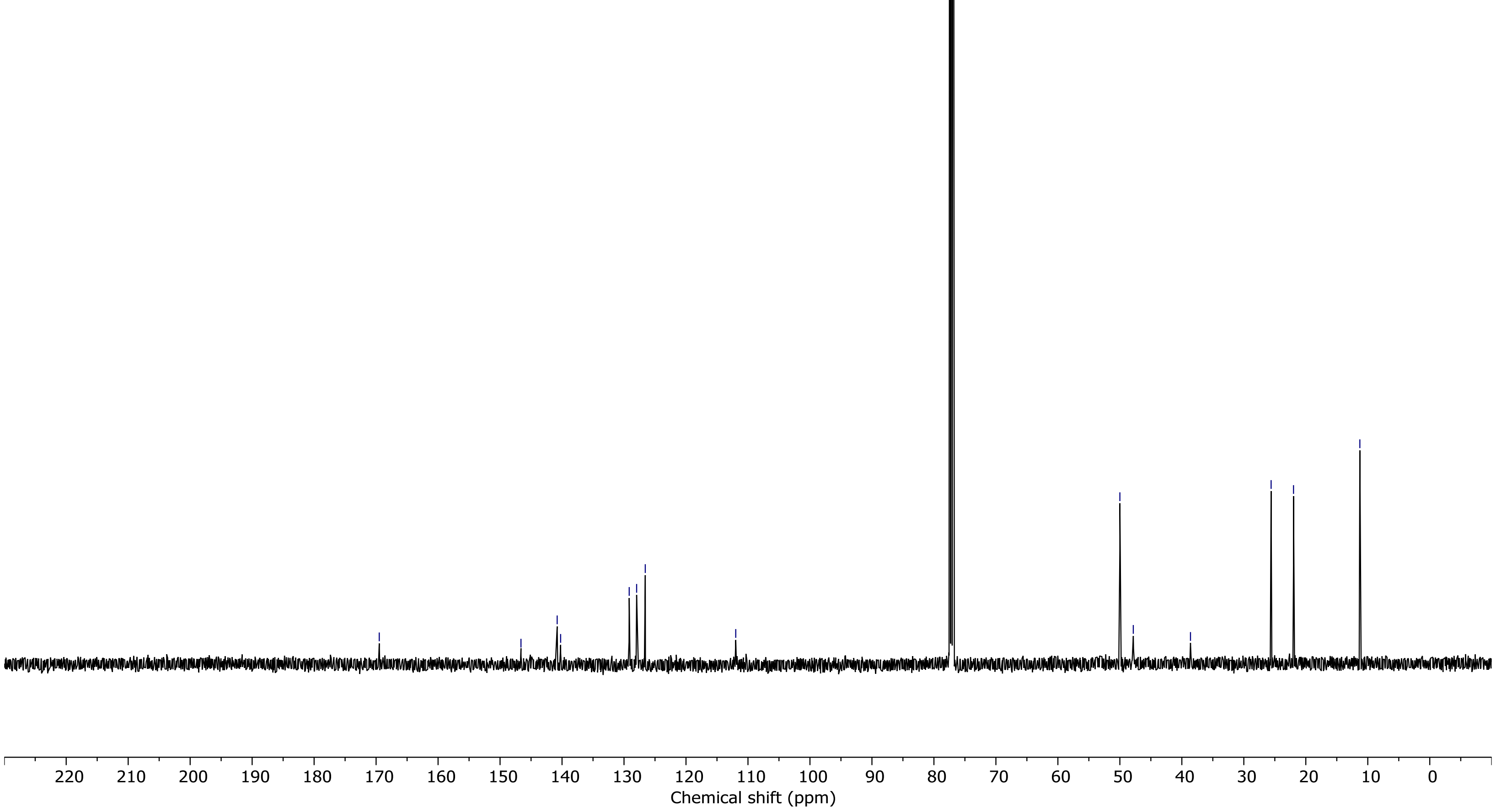

^{1}H NMR of 49

$CDCl_3$, 600 MHz, 23 ºC.

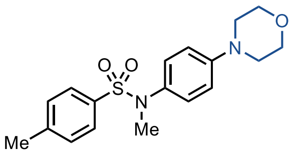

49

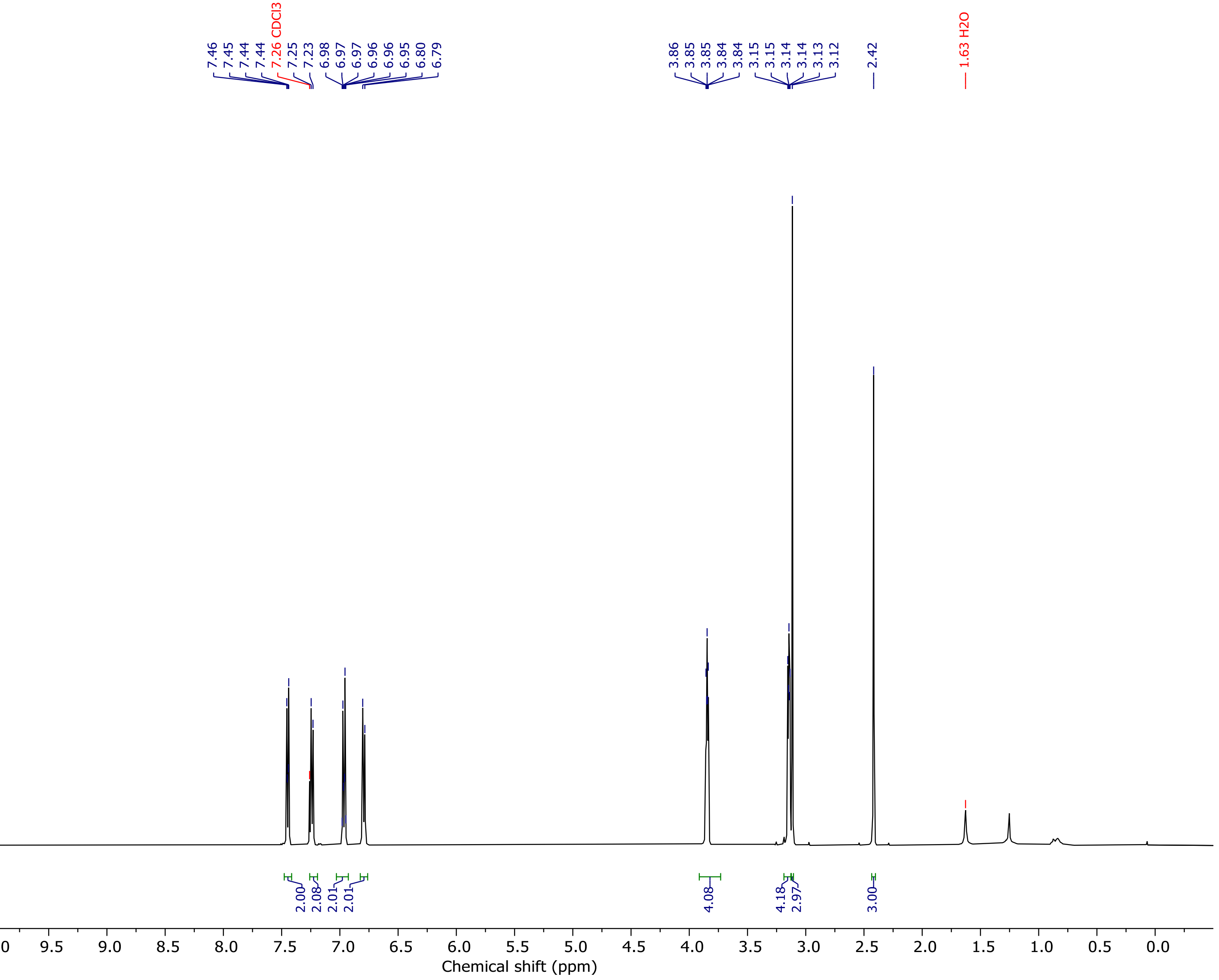

^{13}C NMR of 49

$CDCl_3$, 151 MHz, 23 ºC.

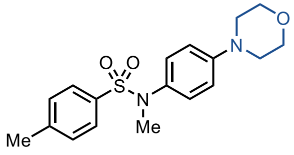

49

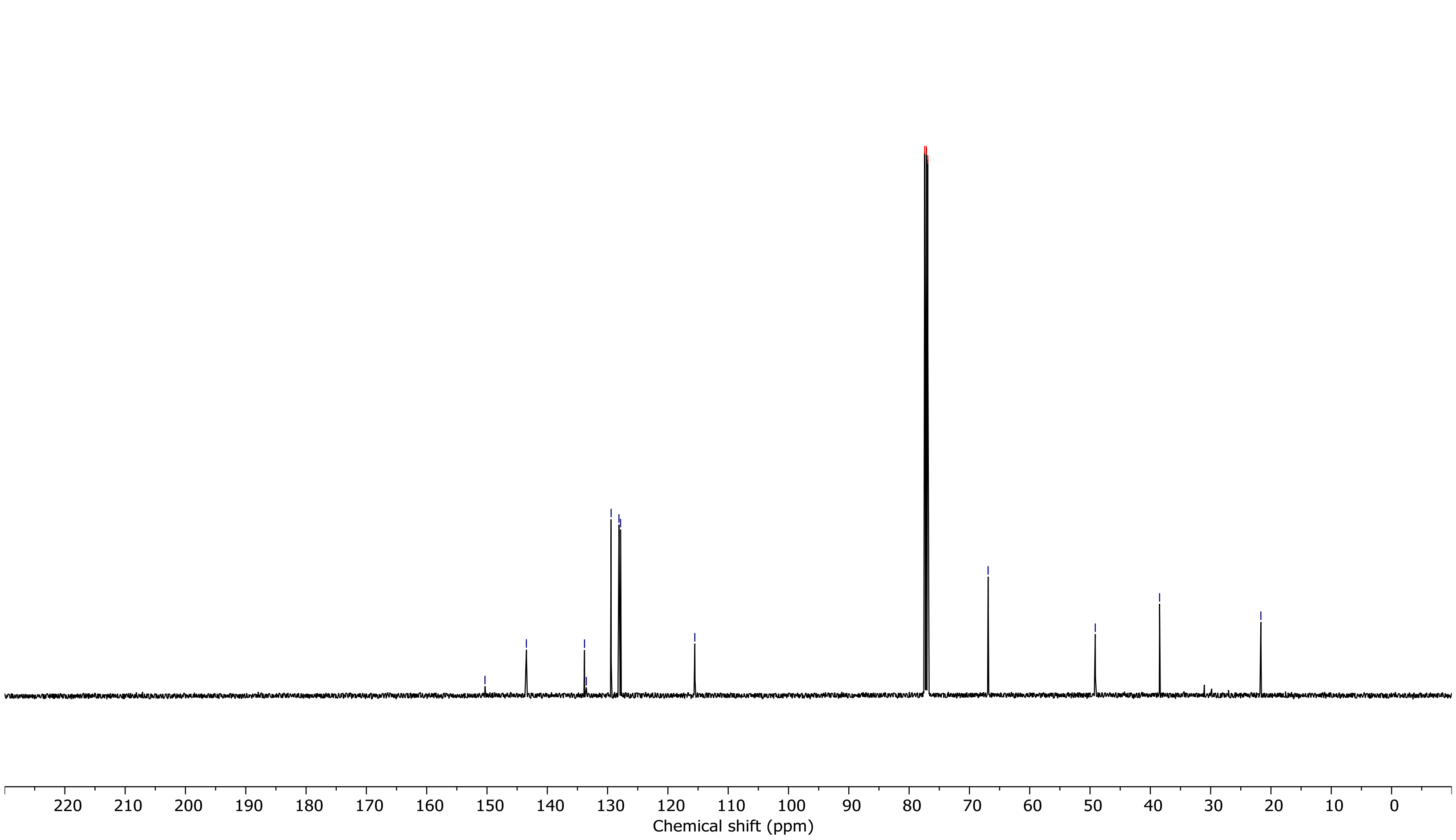

^{1}H NMR of 50

$(CD_3)_2SO$, 500 MHz, 23 ºC.

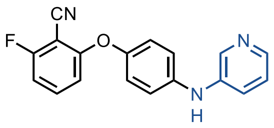

50

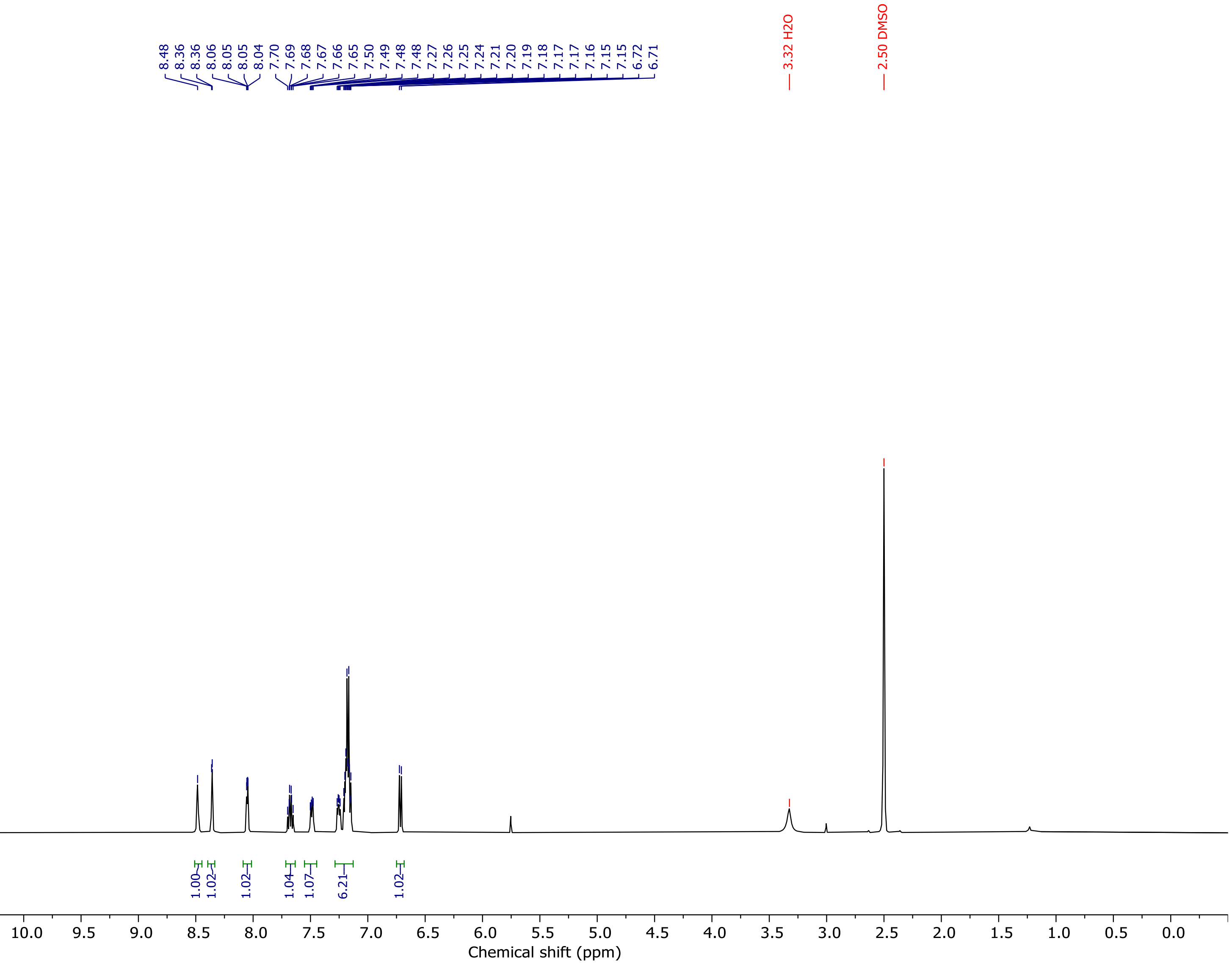

^{13}C NMR of 50

$(CD_3)_2SO$, 126 MHz, 23 ºC.

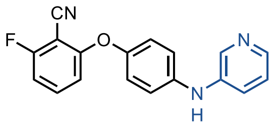

50

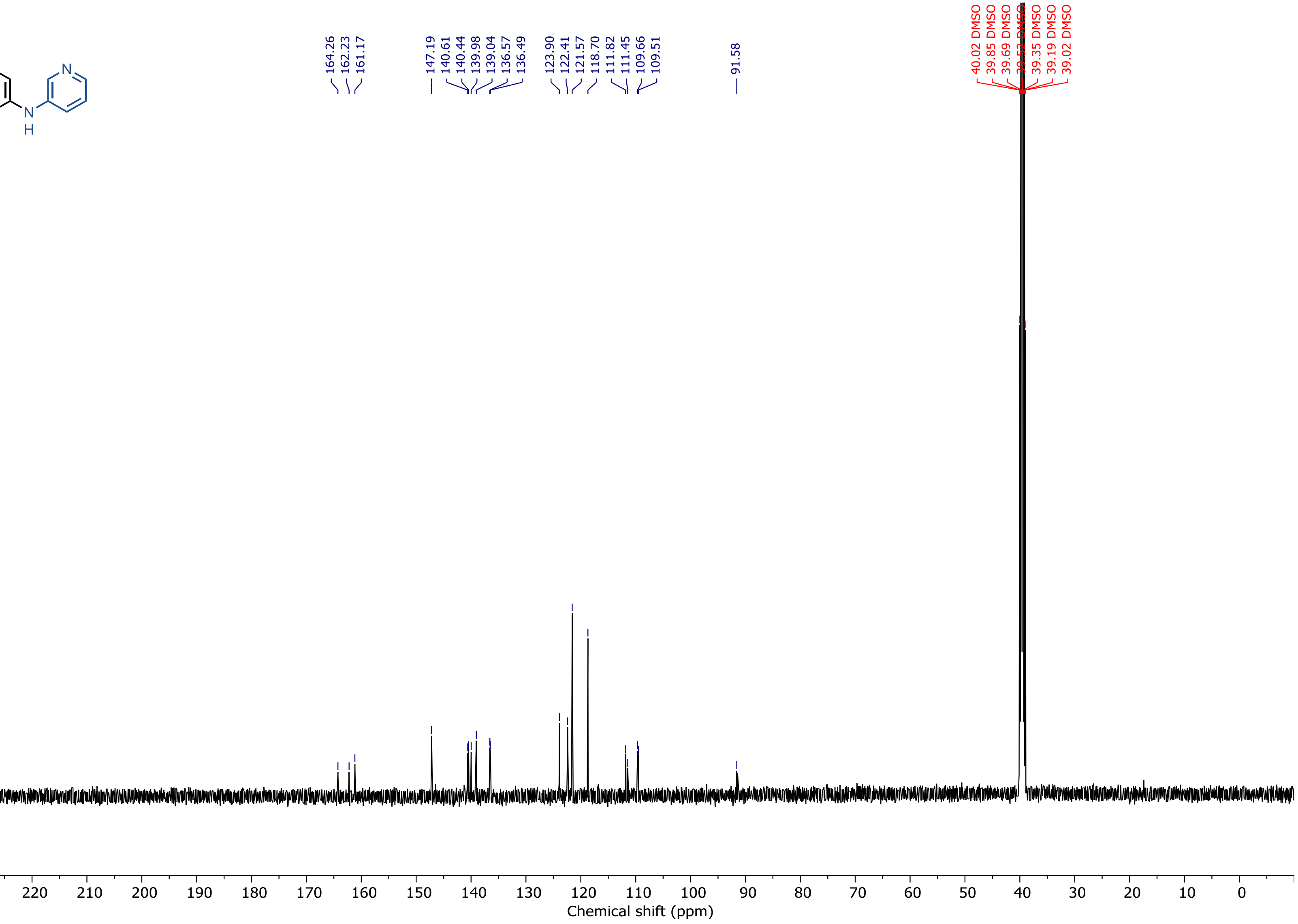

^{19}F NMR of 50

$(CD_3)_2SO$, 471 MHz, 23 ºC.

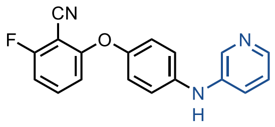

50

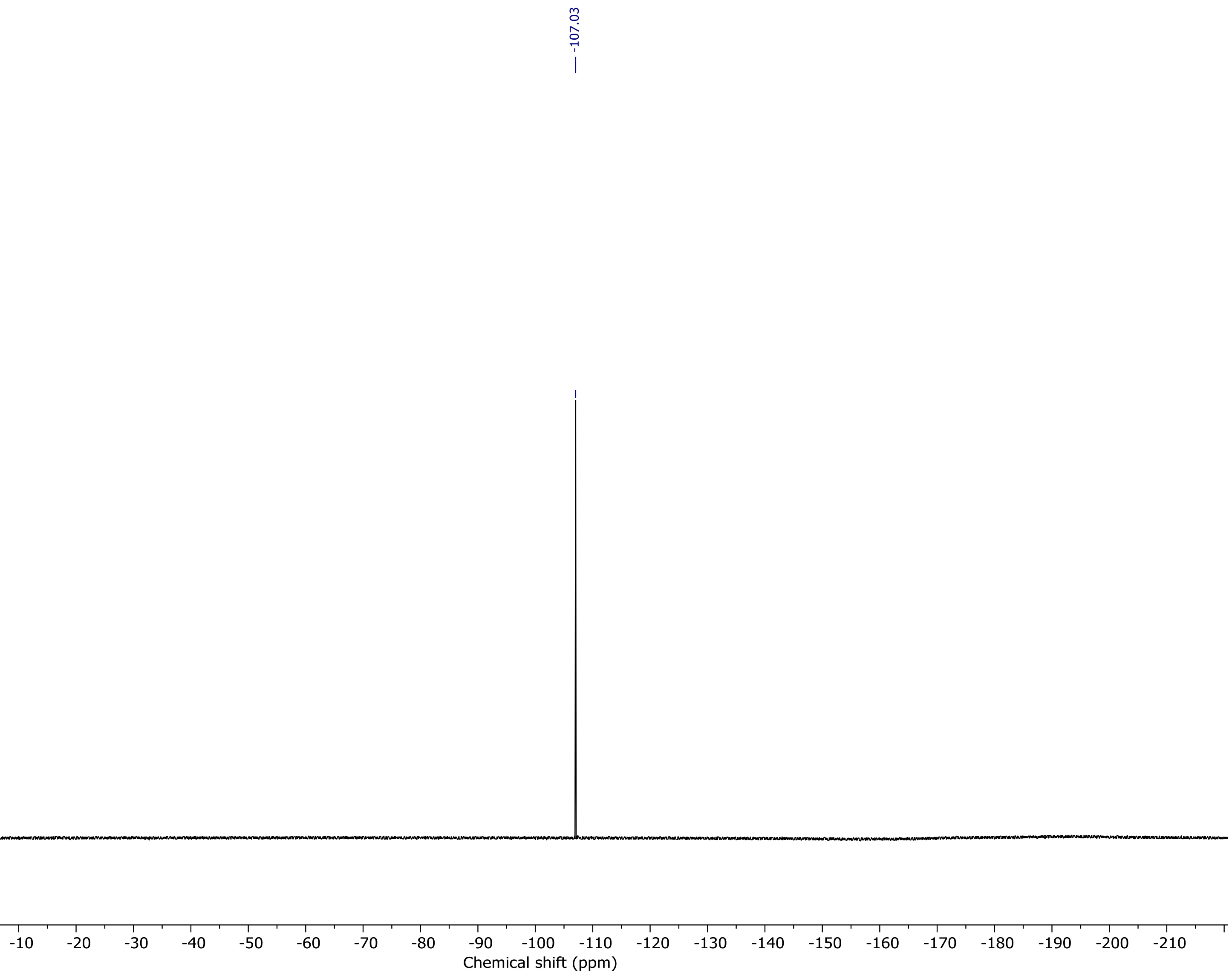

^{1}H NMR of 51

$CDCl_3$, 500 MHz, 23 ºC.

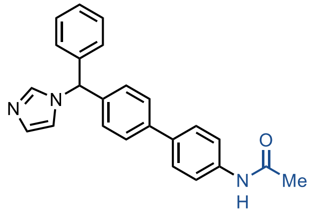

(±)-51

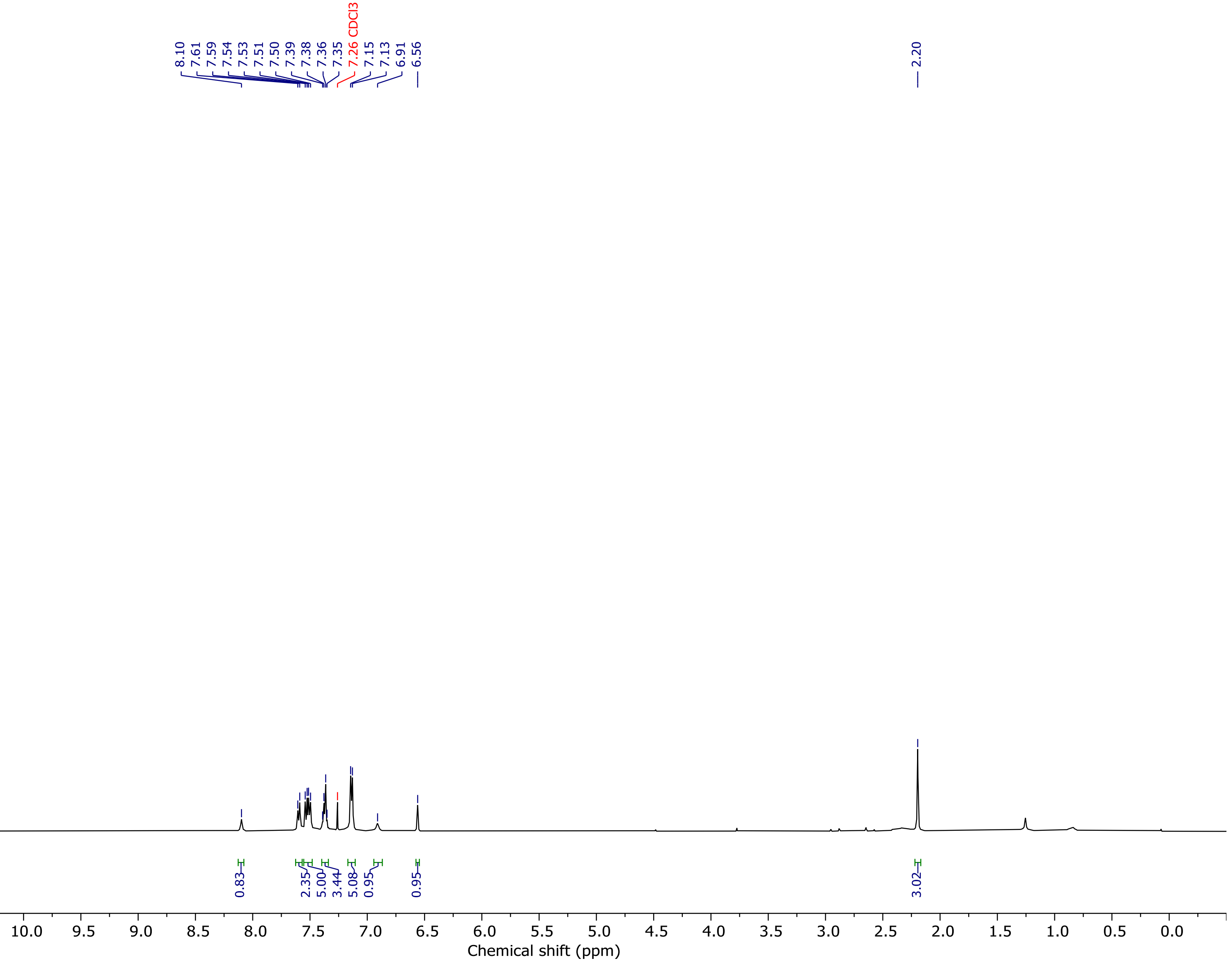

^{13}C NMR of 51

CDCl$_3$, 126 MHz, 23 ºC.

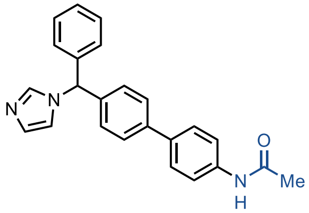

(±)-51

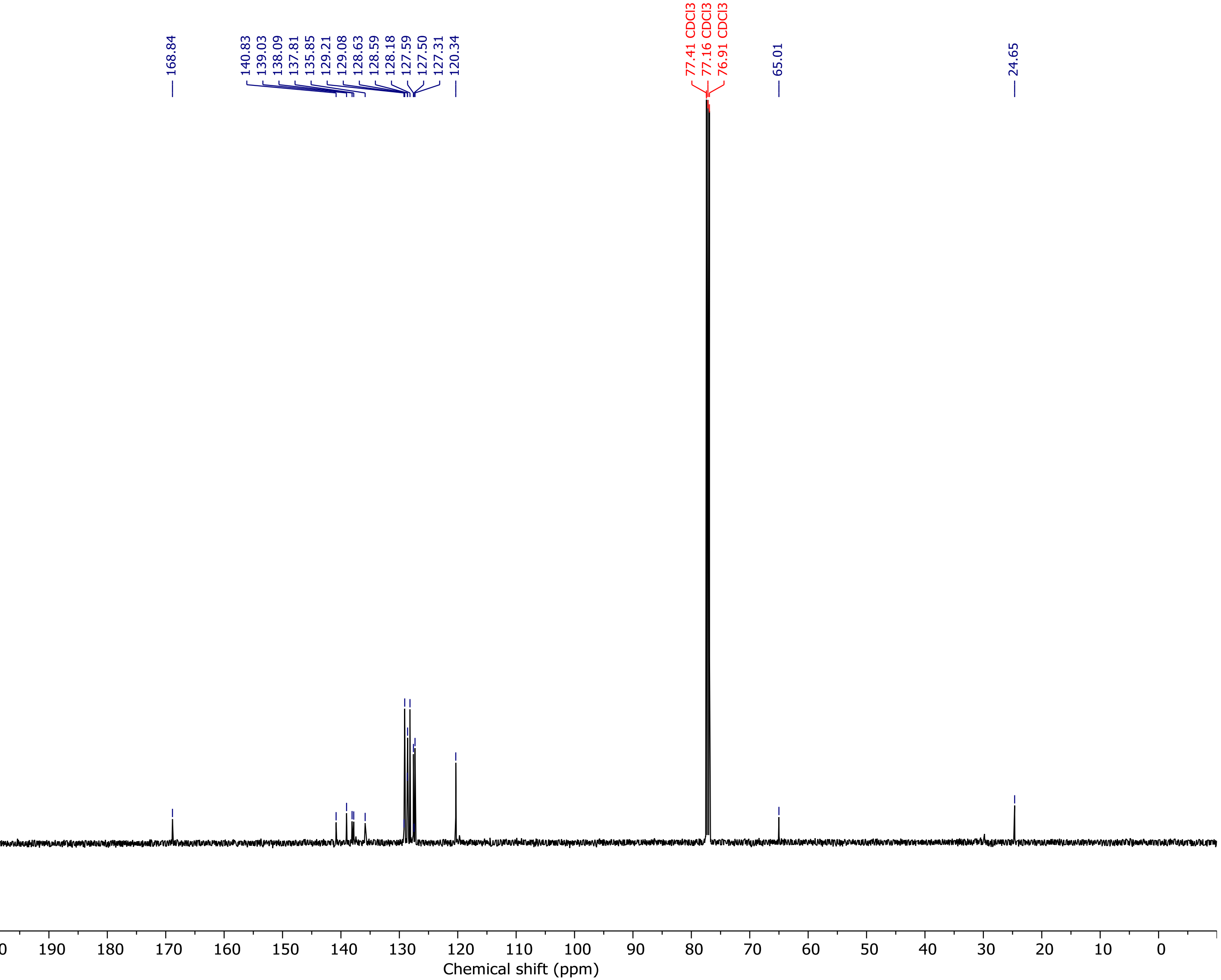

^{1}H NMR of 52

CDCl$_3$, 600 MHz, 23 ºC.

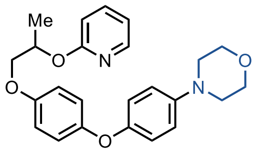

(±)-52

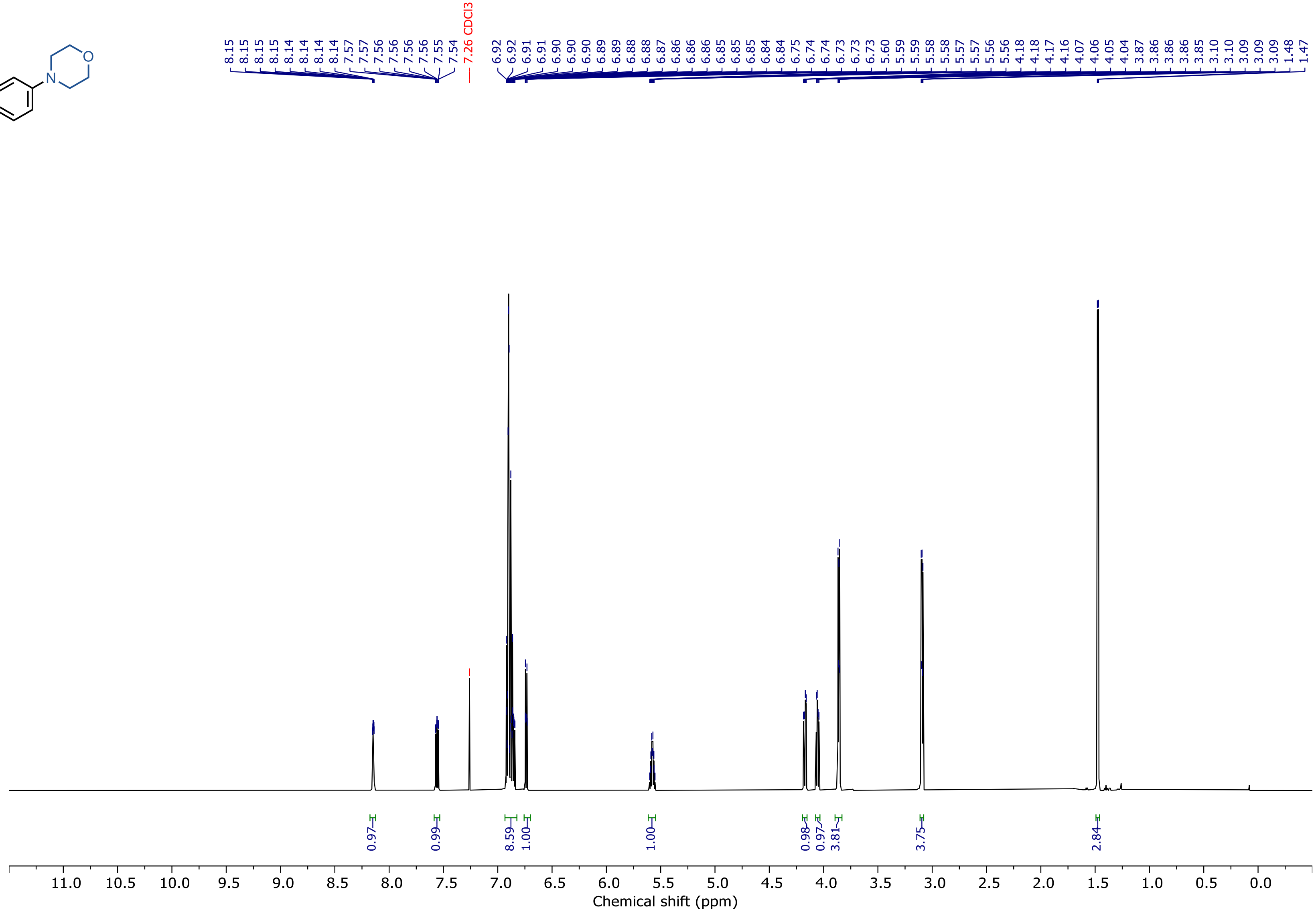

^{13}C NMR of 52

$CDCl_3$, 151 MHz, 23 ºC.

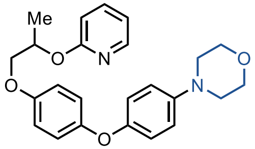

(±)-52

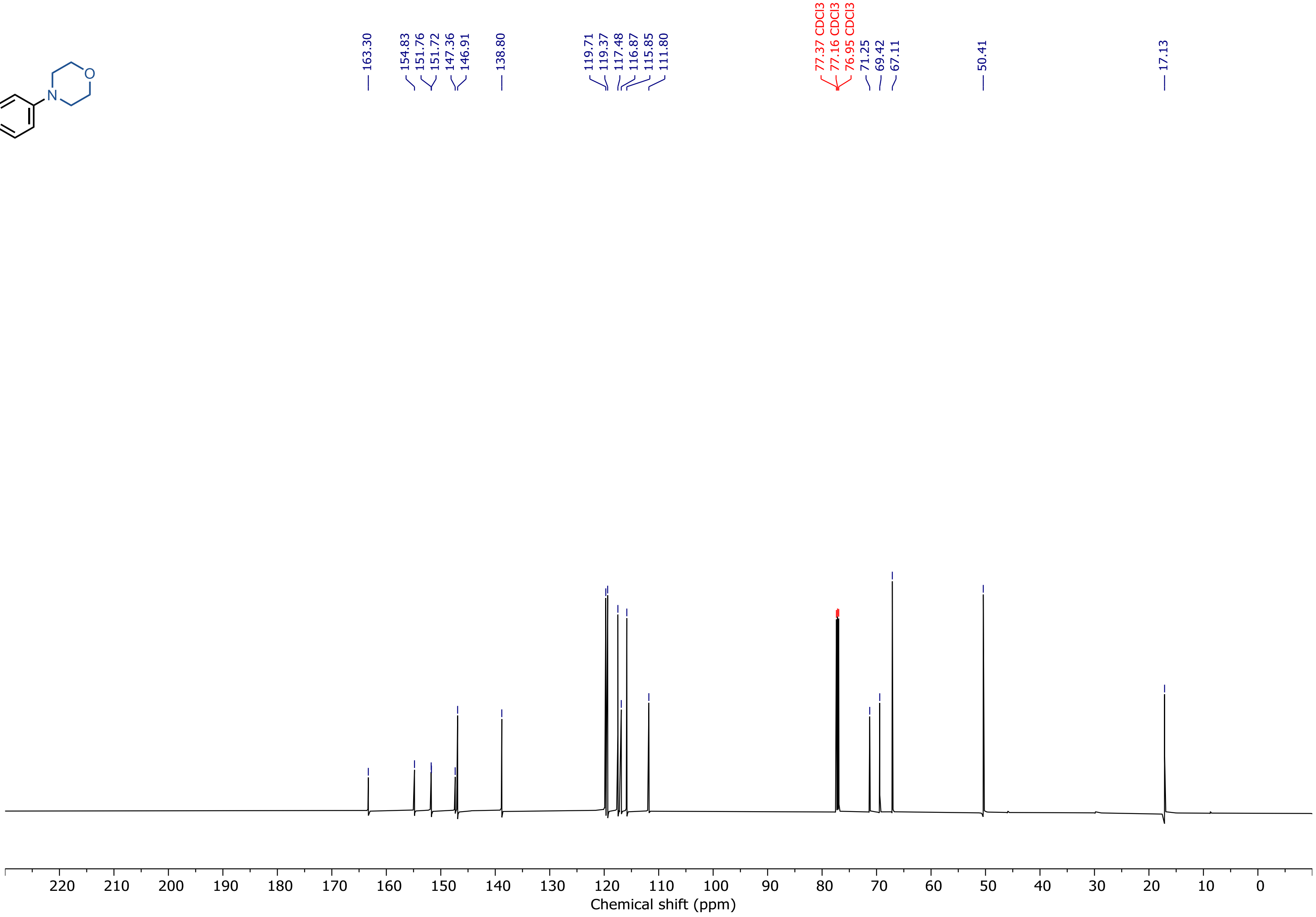

^{1}H NMR of 53

CDCl$_3$, 600 MHz, 23 ºC.

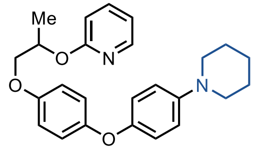

(±)-53

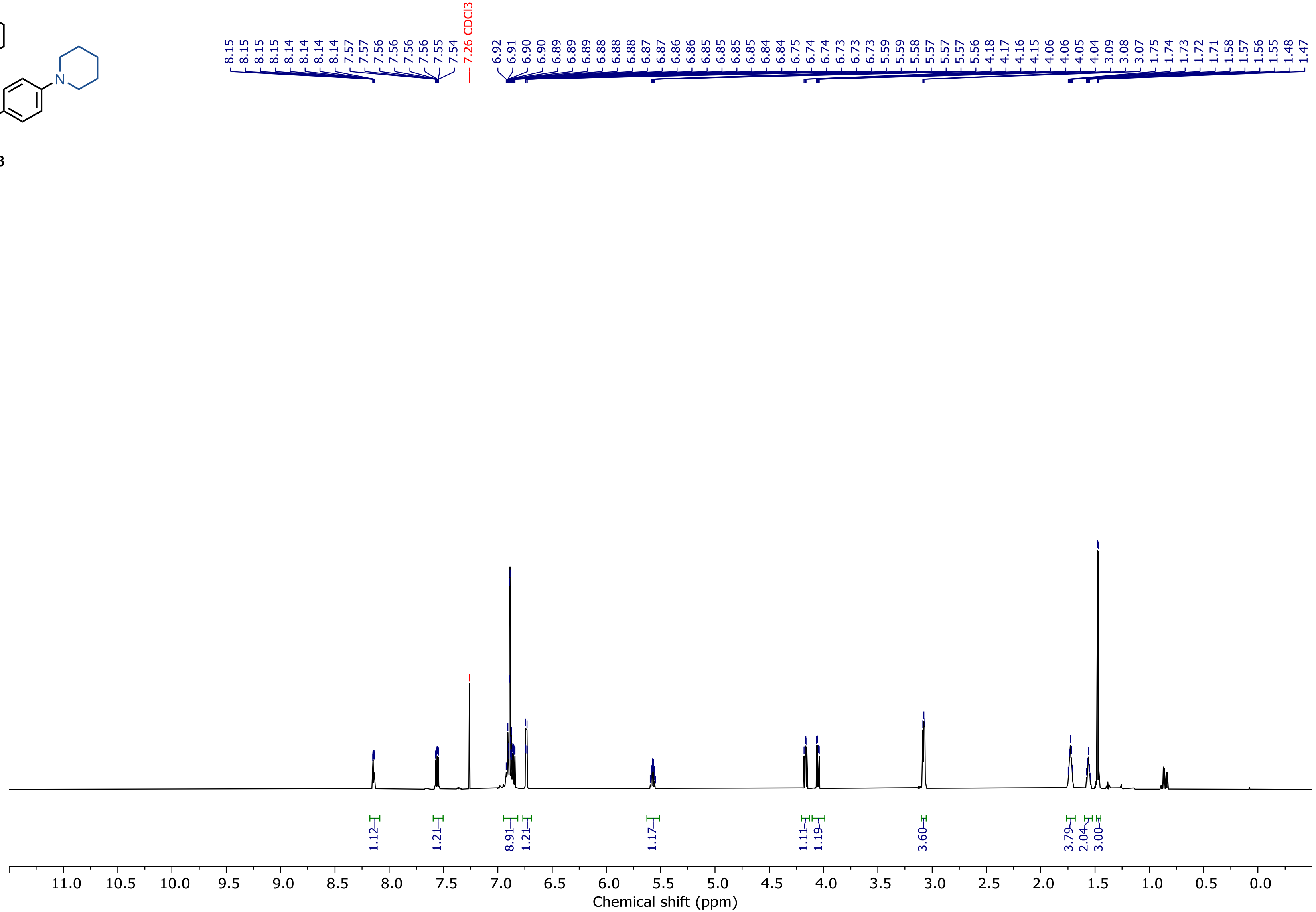

^{13}C NMR of 53

$CDCl_3$, 151 MHz, 23 ºC.

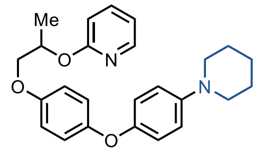

(±)-53

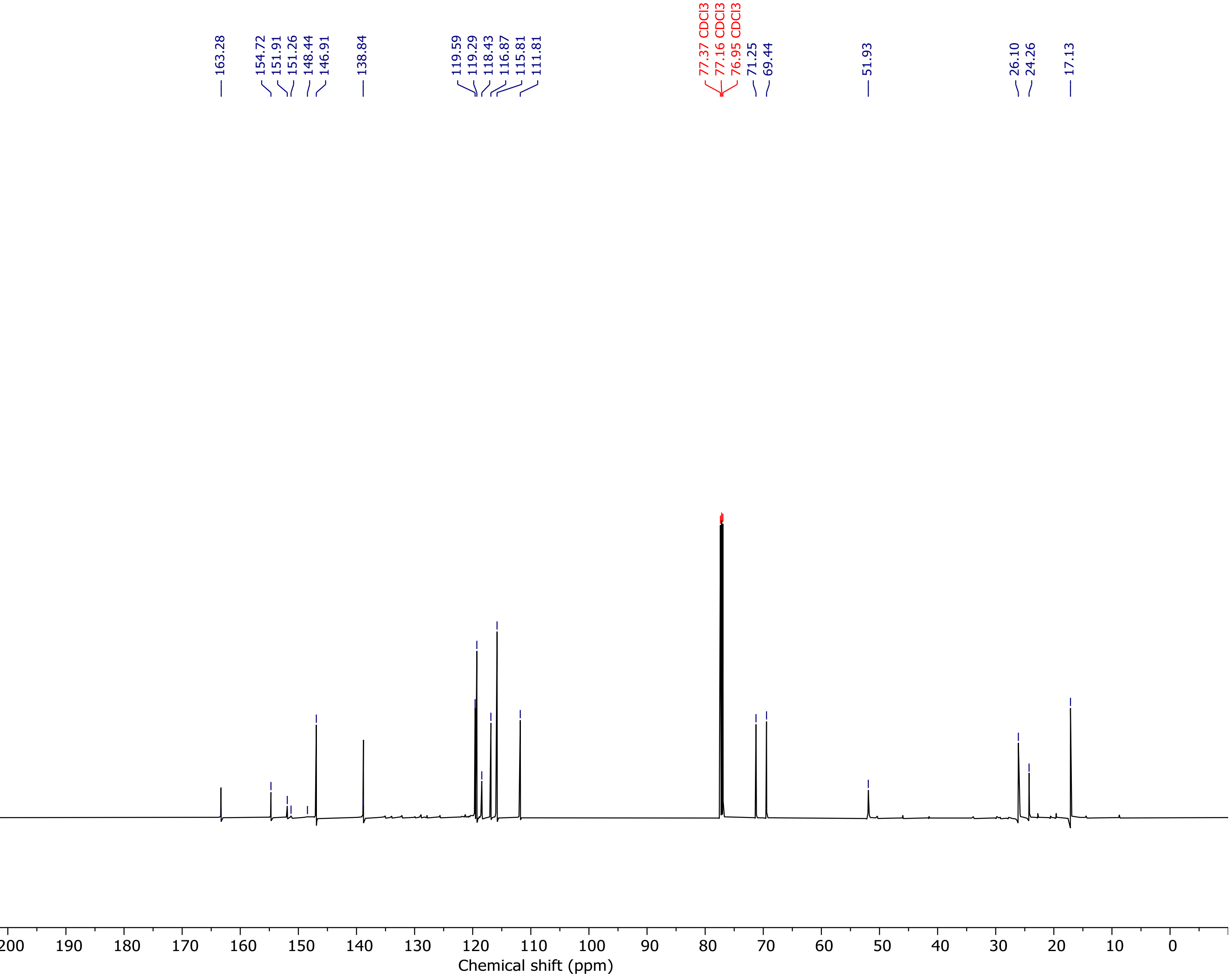

^{1}H NMR of 54

CDCl3, 600 MHz, 23 ºC.

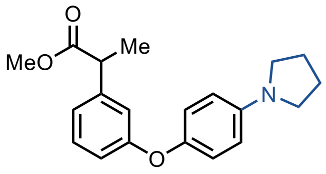

54

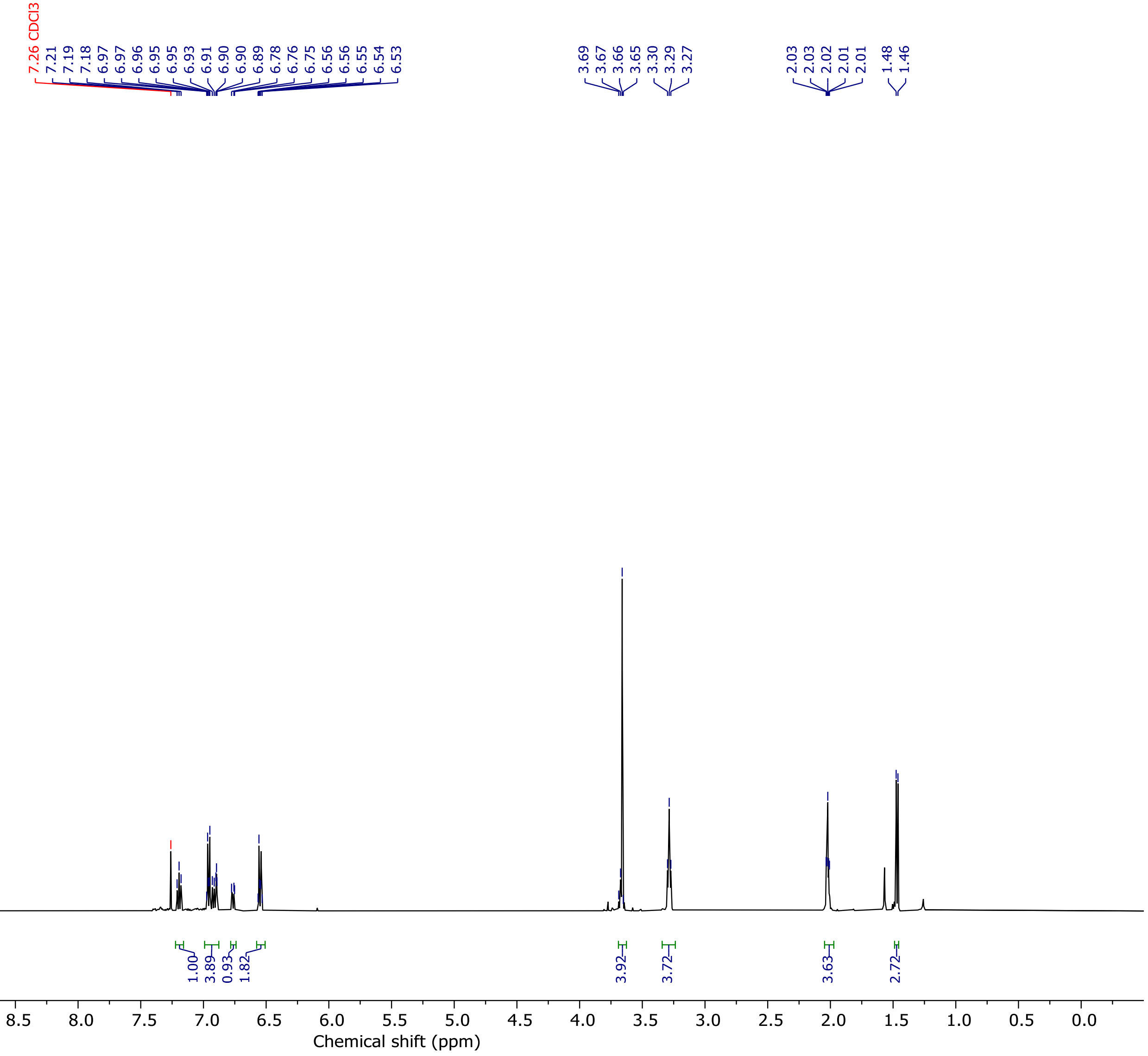

^{13}C NMR of 54

CDCl$_3$, 151 MHz, 23 ºC.

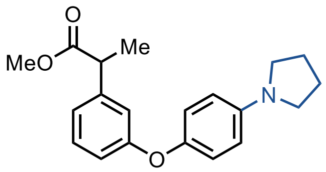

54

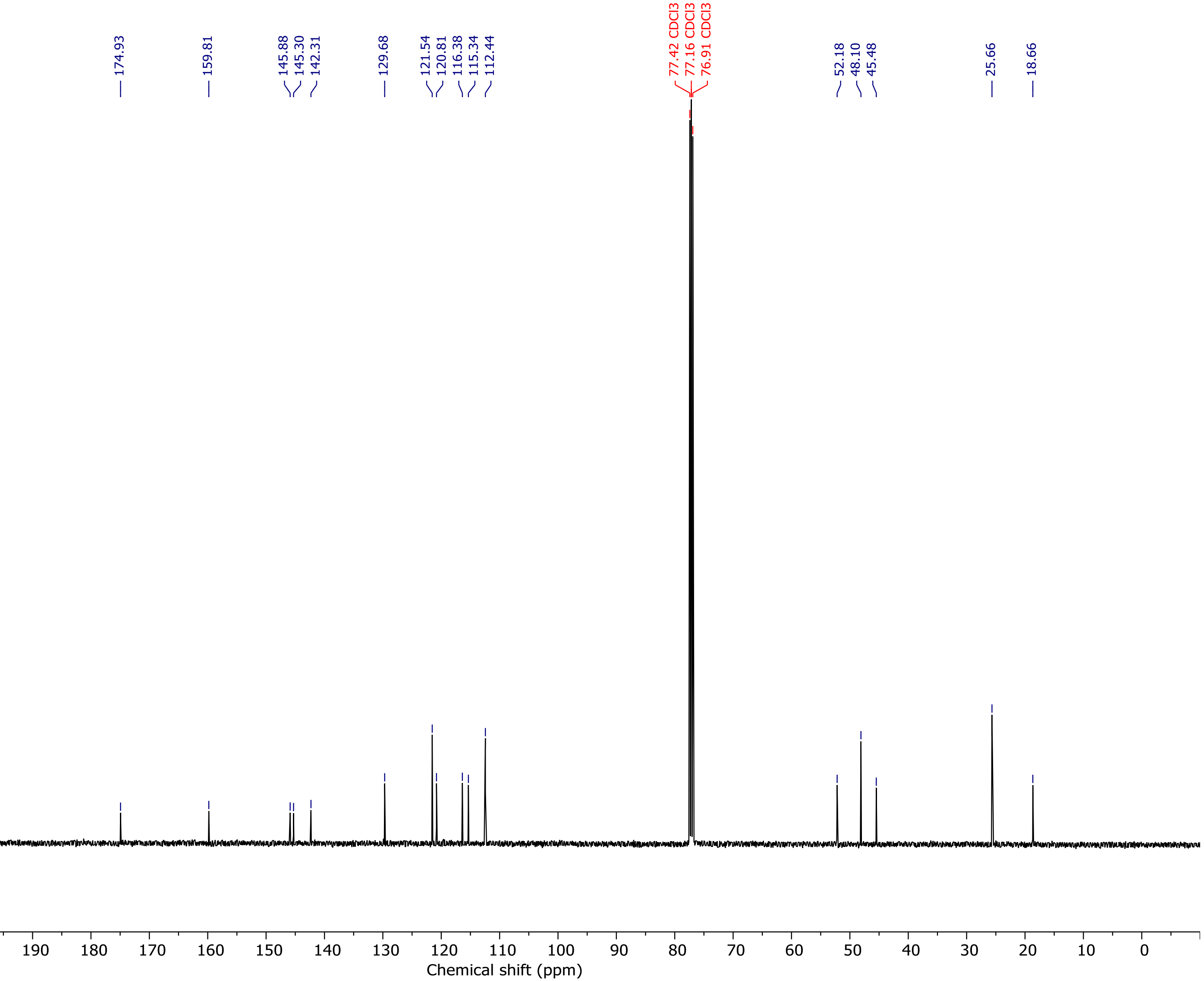

^{1}H NMR of 55

$CDCl_3$, 600 MHz, 23 ºC.

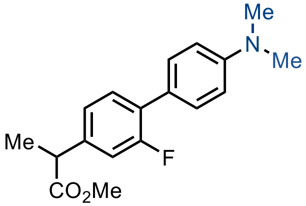

55

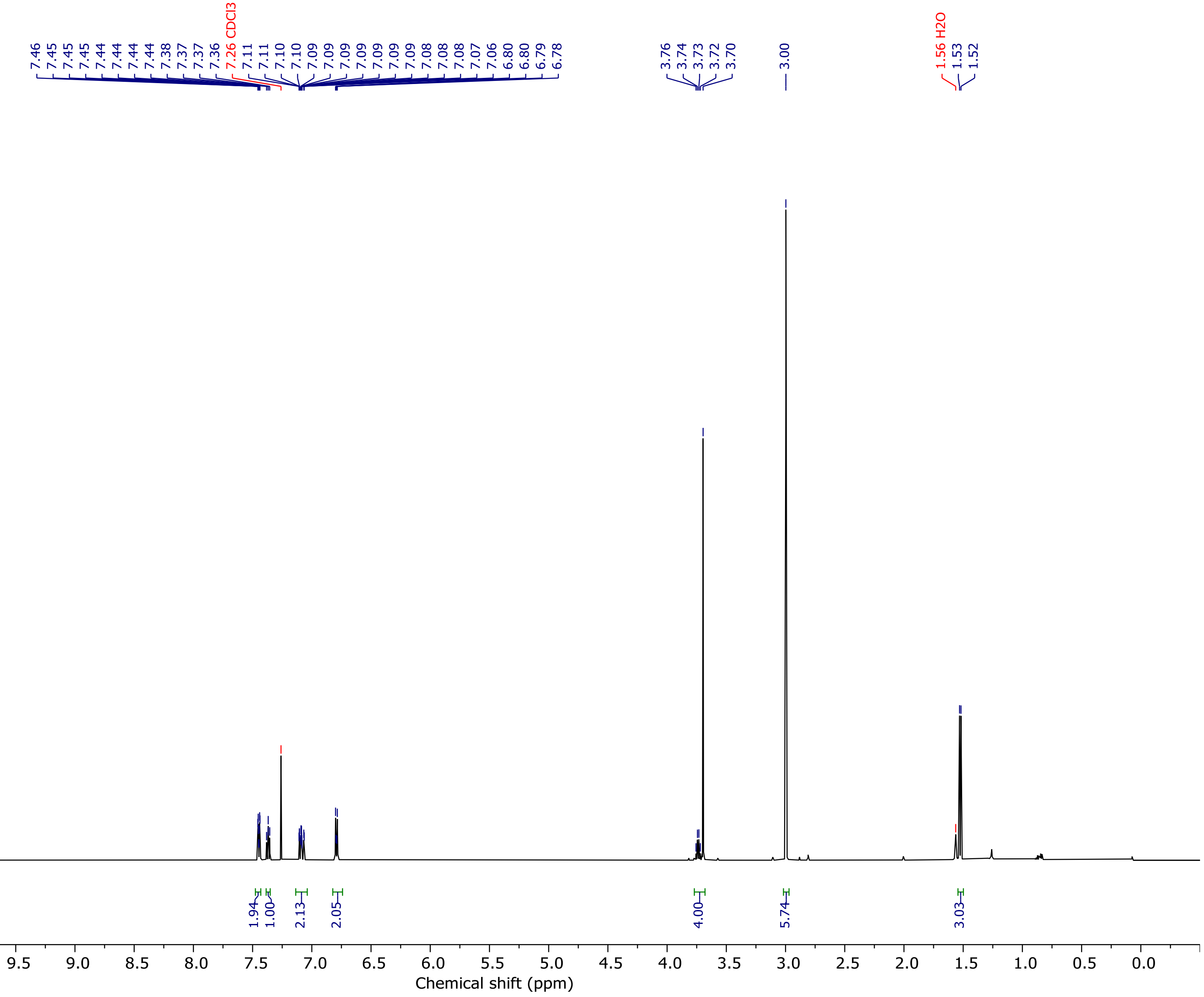

^{13}C NMR of 55

$CDCl_3$, 151 MHz, 23 ºC.

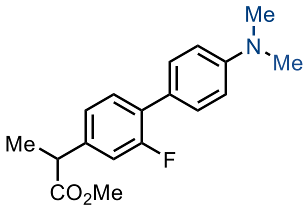

55

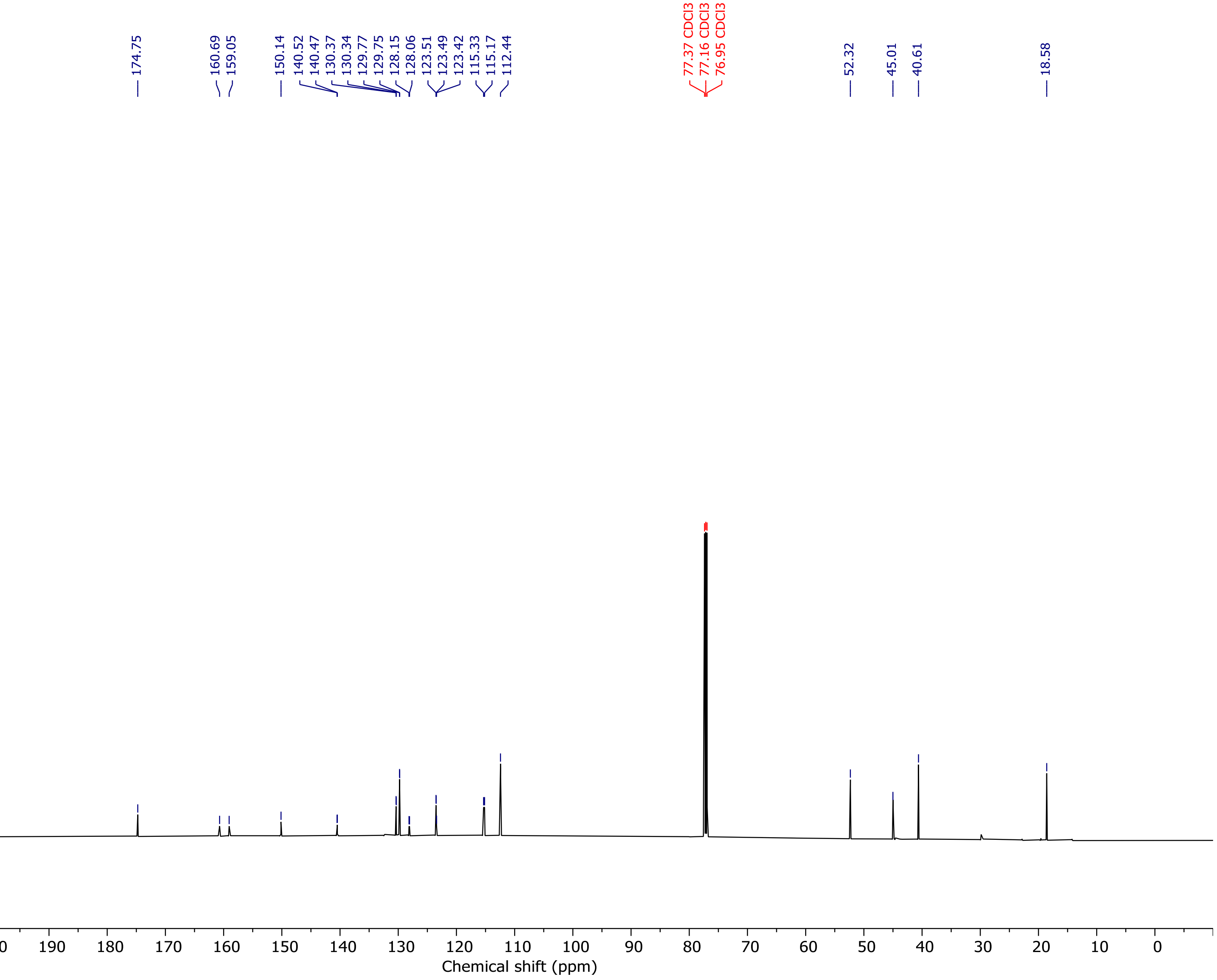

^{19}F NMR of 55

$CDCl_3$, 565 MHz, 23 ºC.

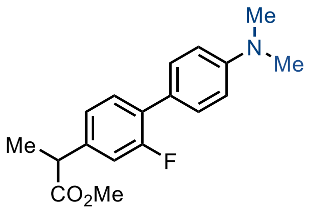

55

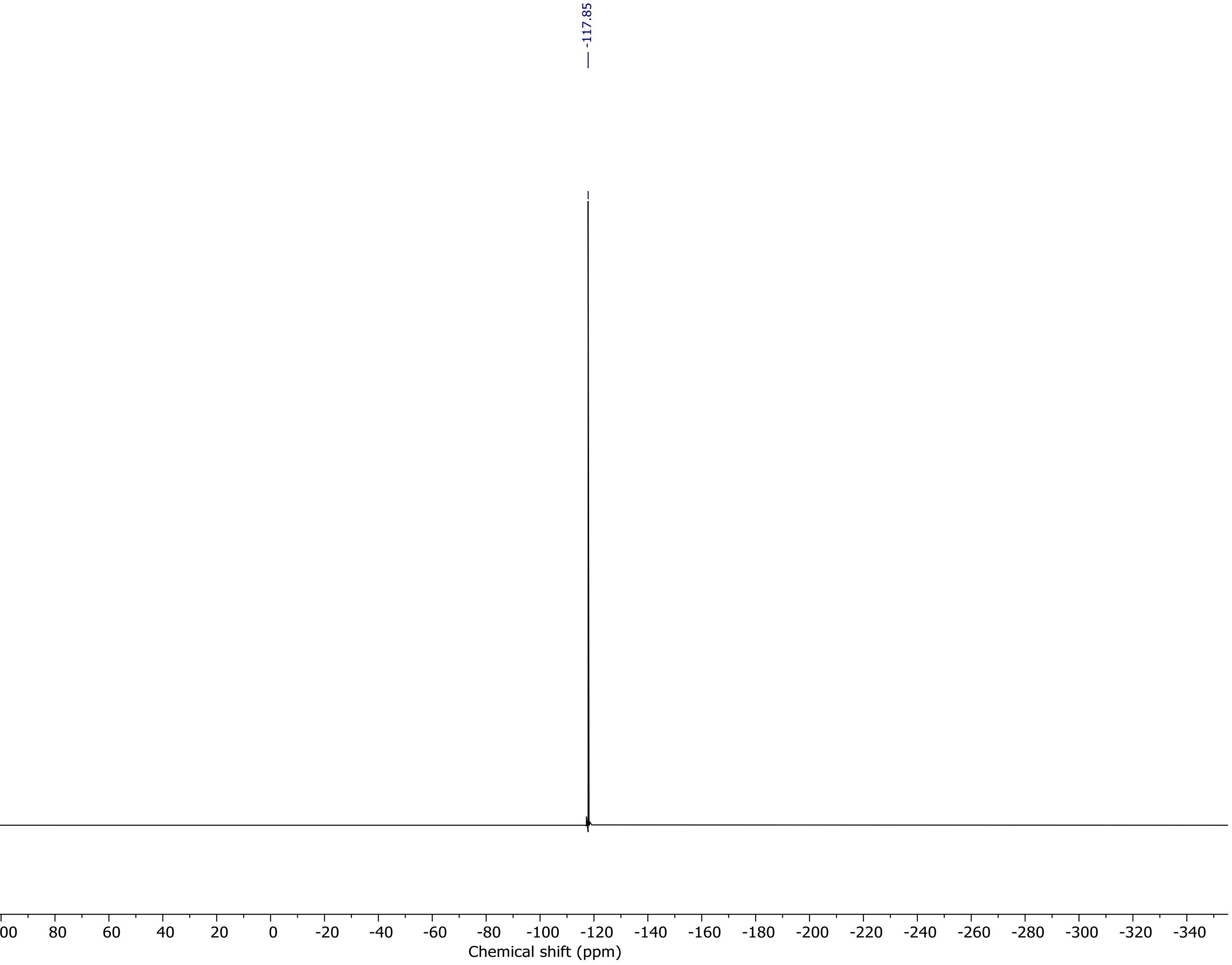

^{1}H NMR of 56

CDCl$_3$, 600 MHz, 23 ºC.

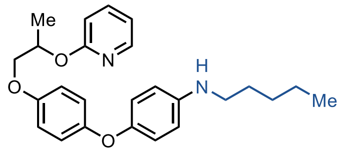

(±)-56

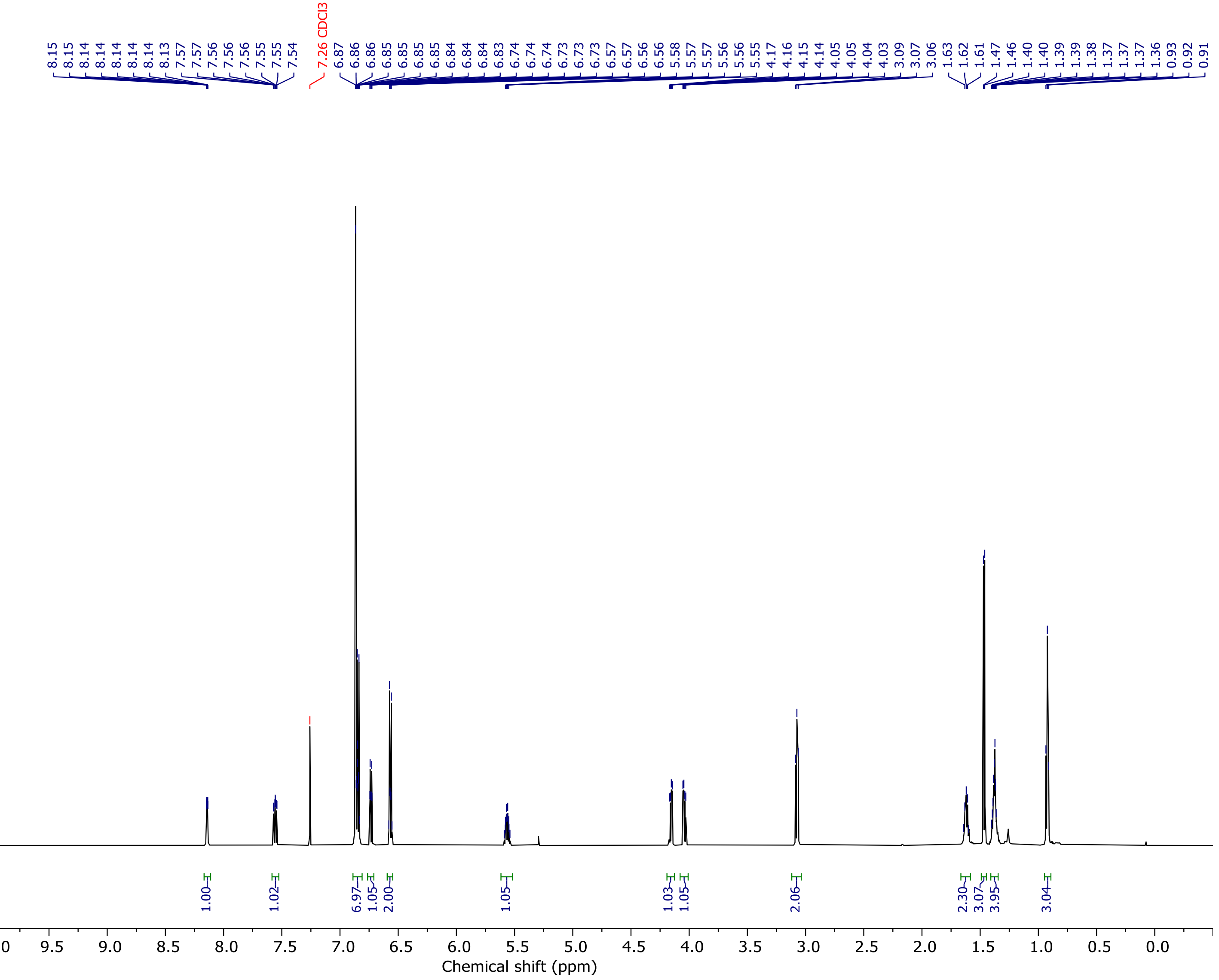

^{13}C NMR of 56

$CDCl_3$, 151 MHz, 23 ºC.

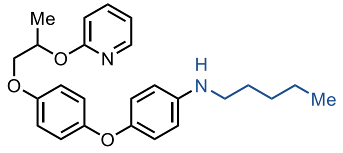

(±)-56

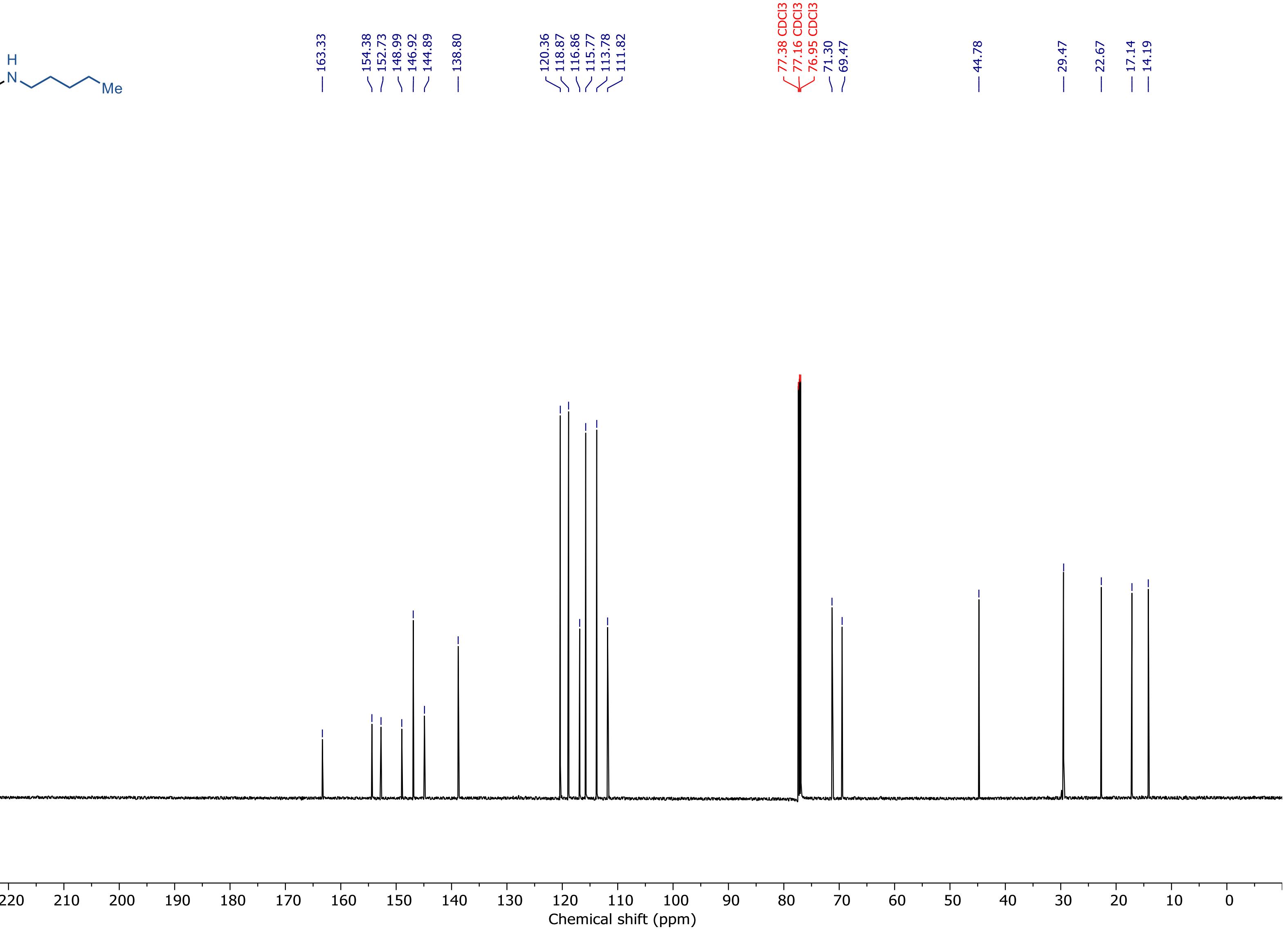

^{1}H NMR of 57

CDCl$_3$, 600 MHz, 23 ºC.

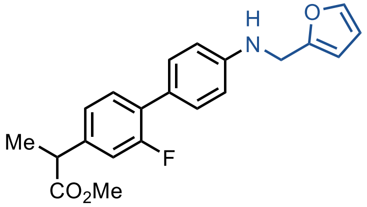

57

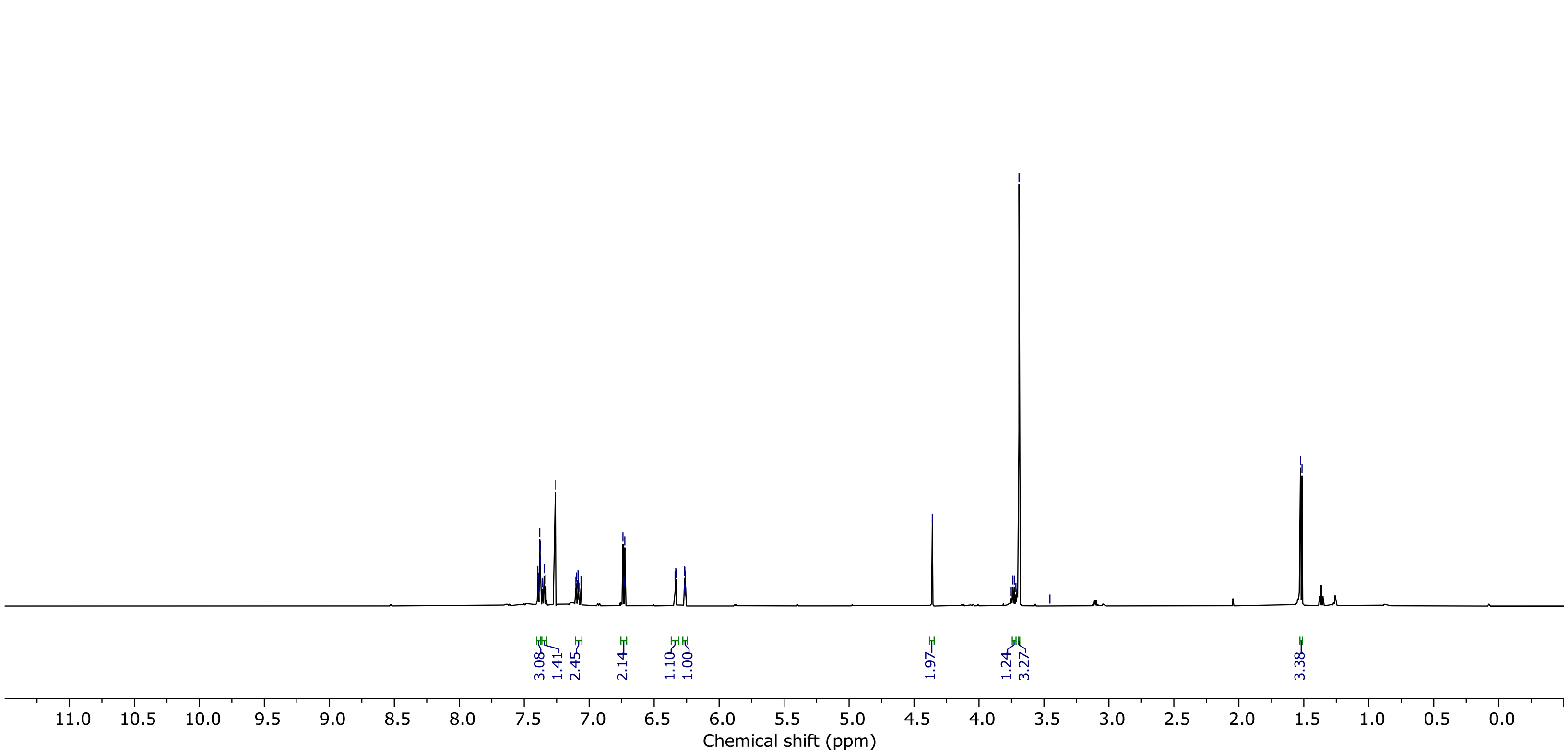

^{13}C NMR of 57

$CDCl_3$, 151 MHz, 23 ºC.

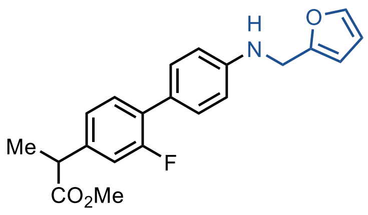

57

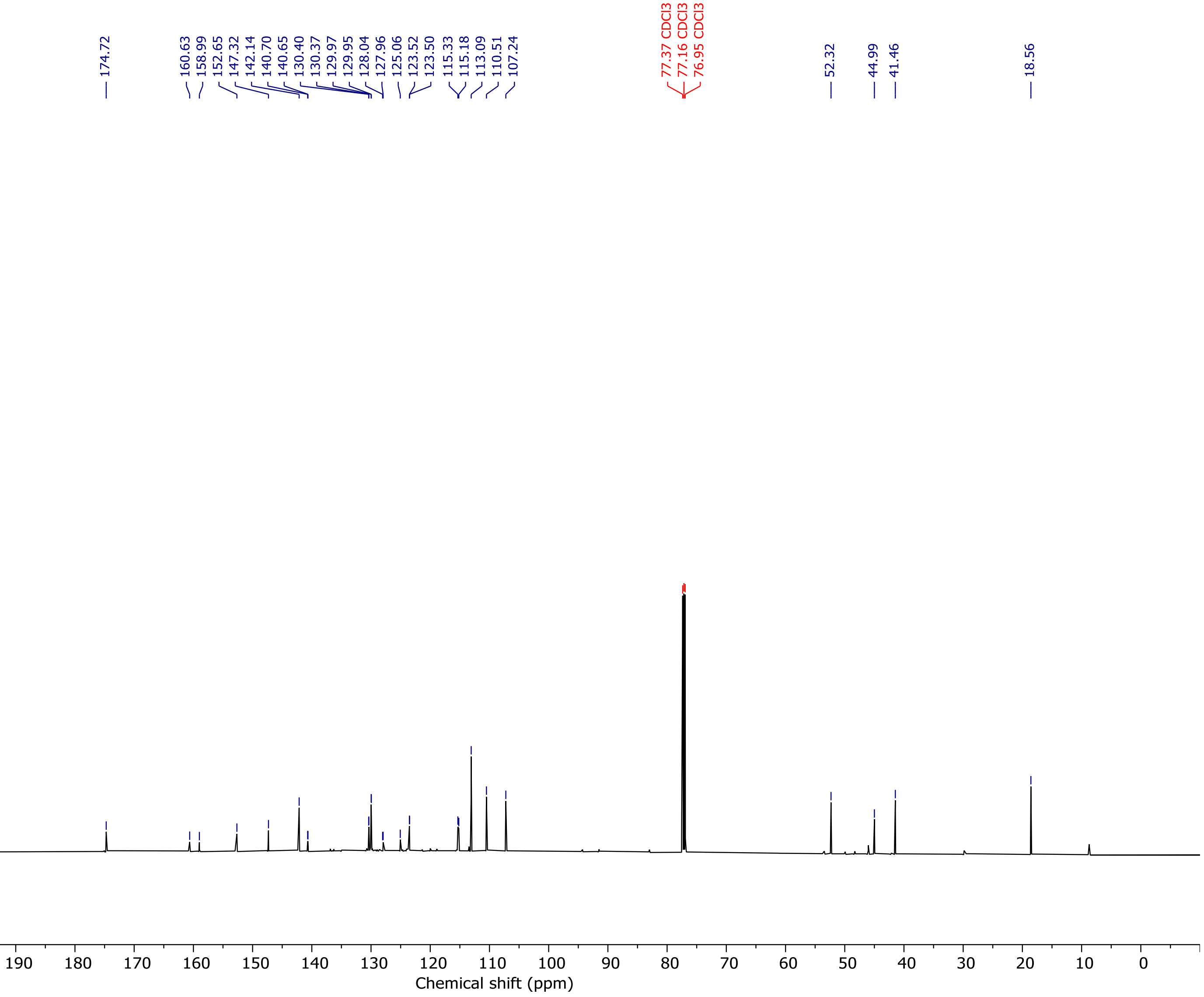

^{19}F NMR of 57

$CDCl_3$, 565 MHz, 23 ºC.

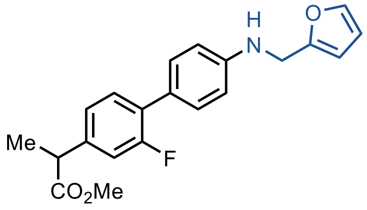

57

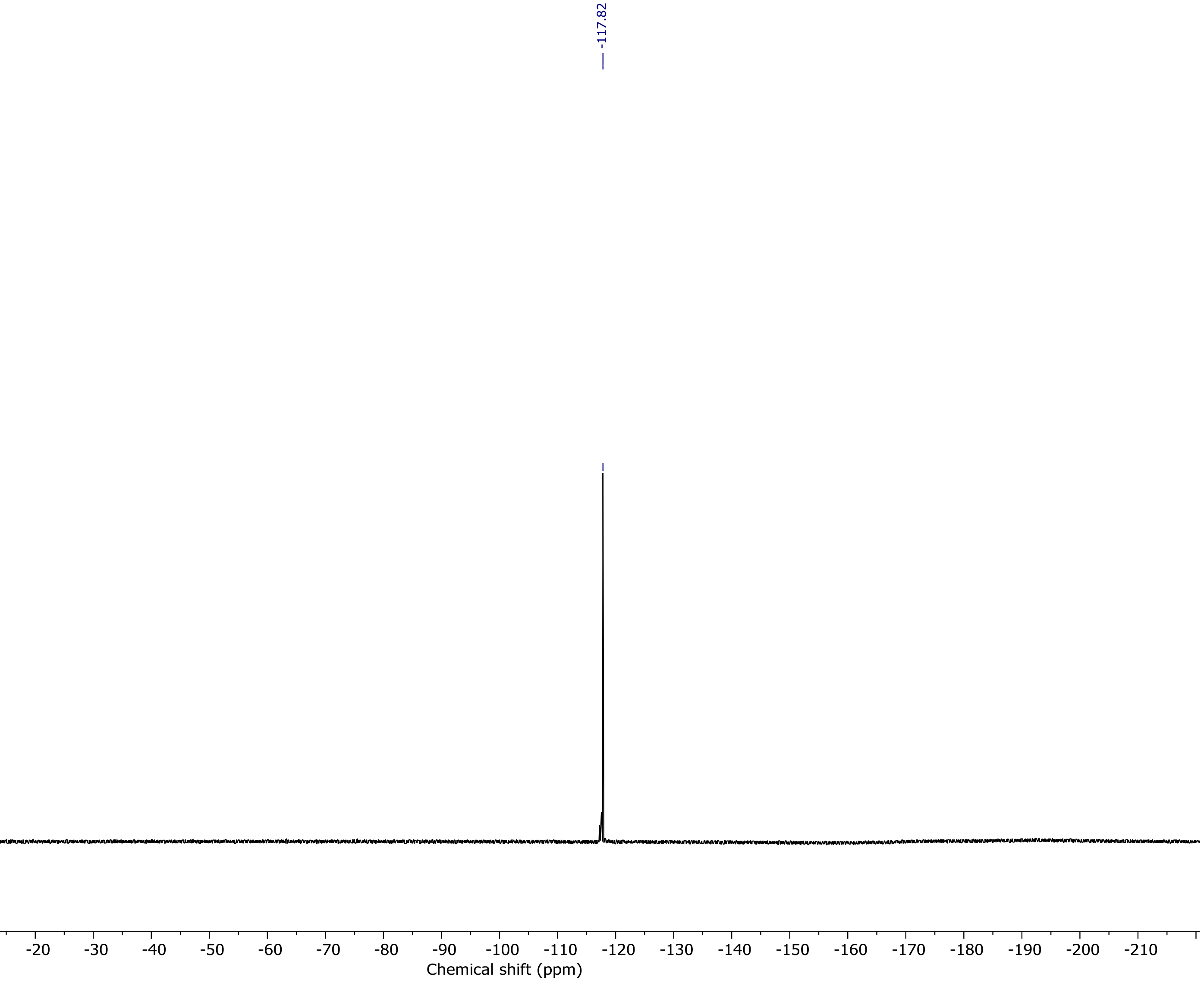

^{1}H NMR of 58

$CDCl_3$, 600 MHz, 23 ºC.

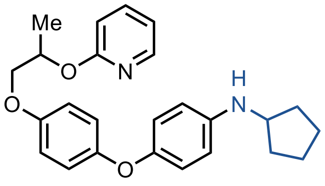

58

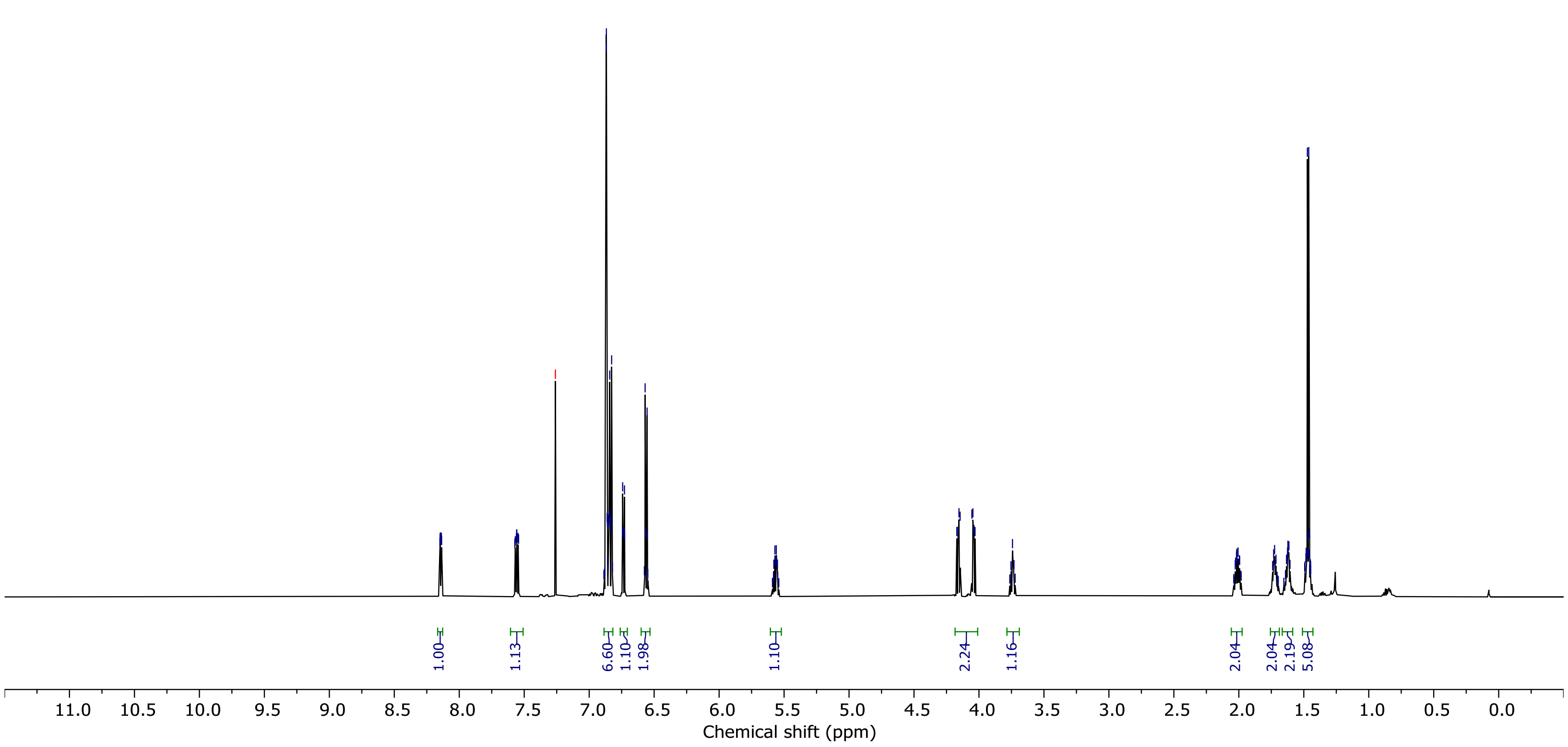

^{13}C NMR of 58

$CDCl_3$, 151 MHz, 23 ºC.

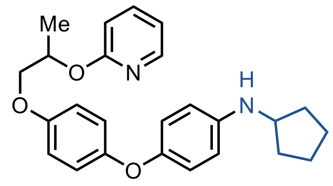

58

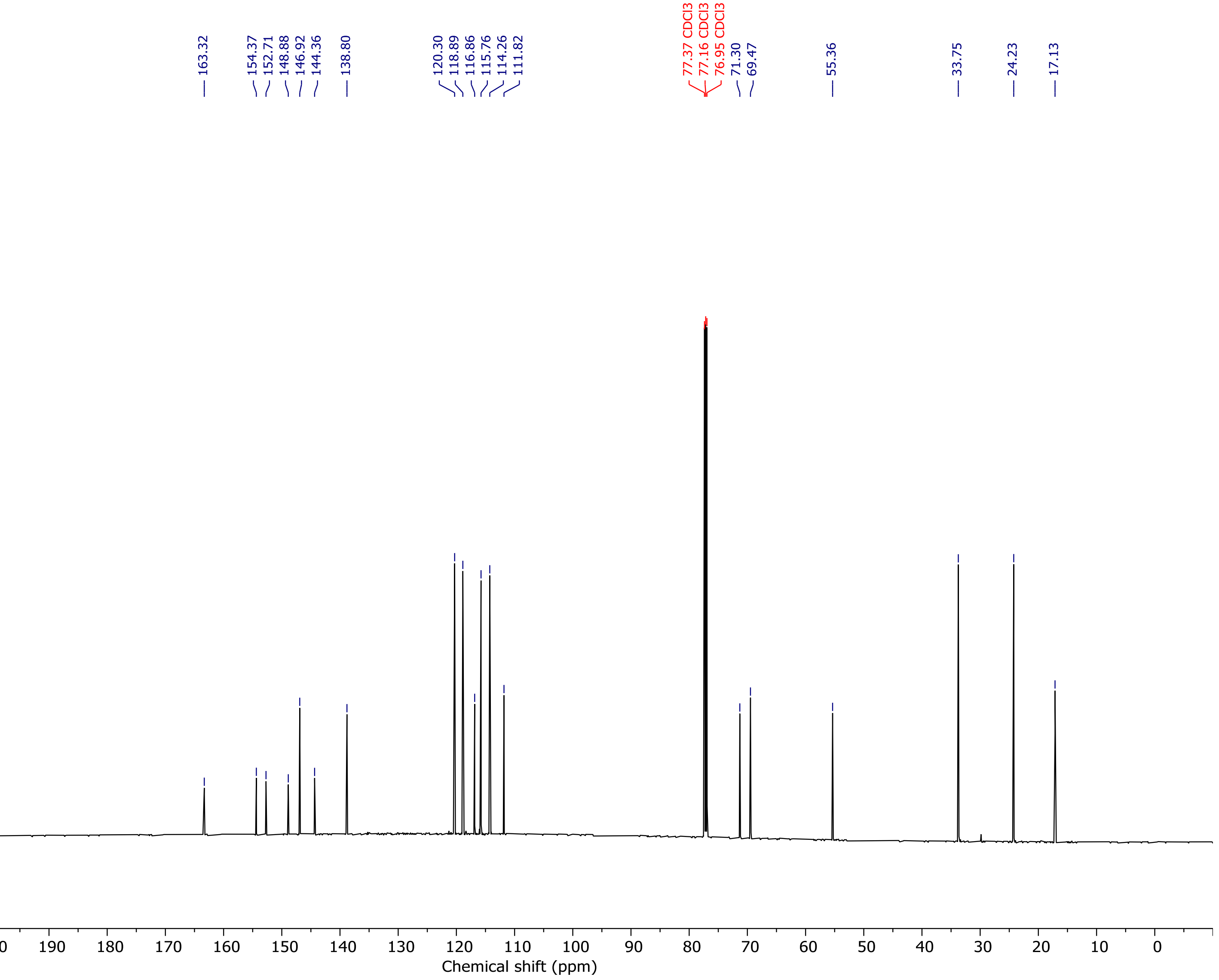

^{1}H NMR of 59

$CDCl_3$, 600 MHz, 23 ºC.

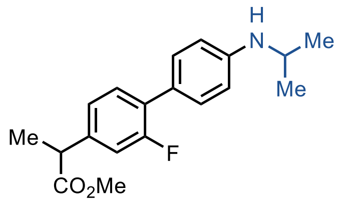

59

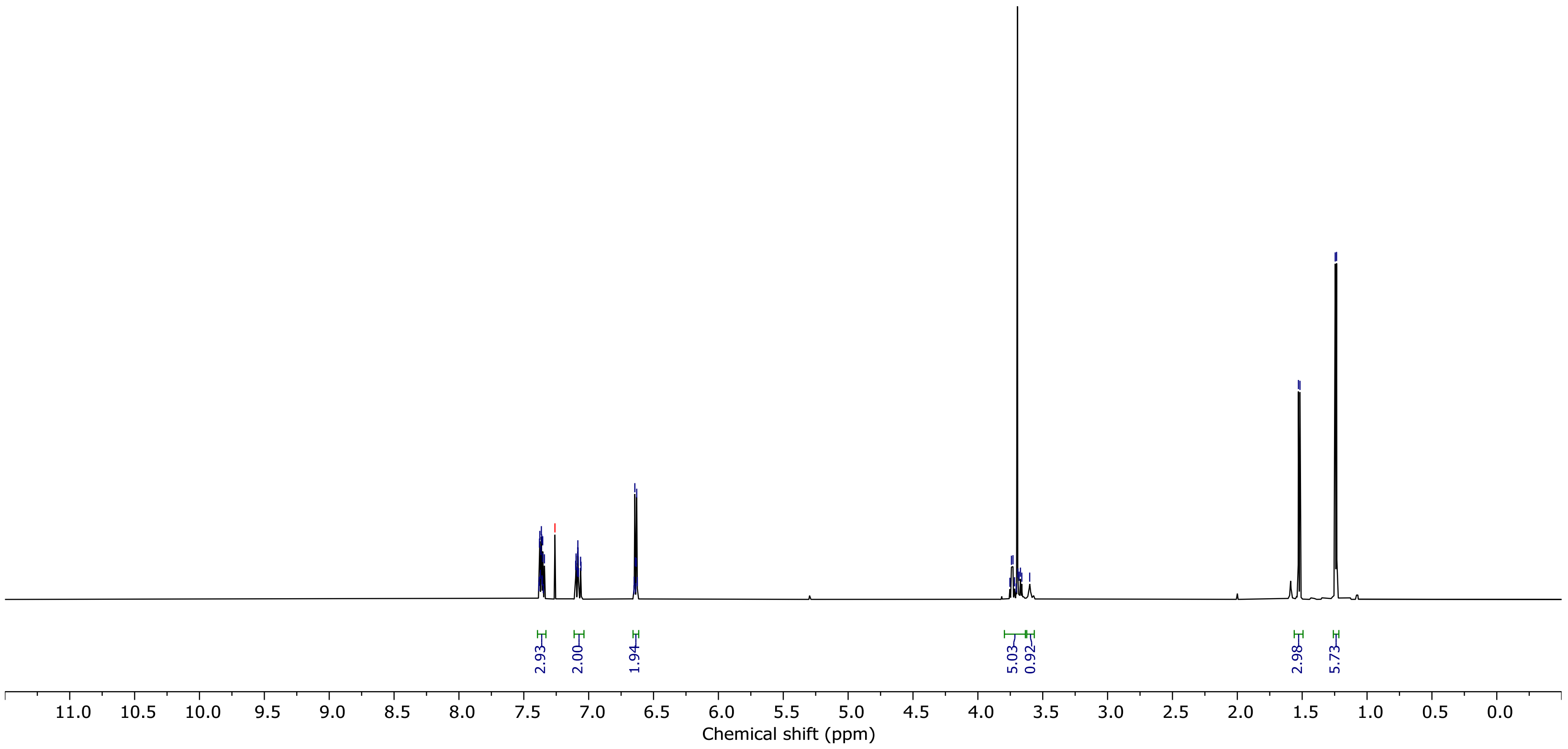

^{13}C NMR of 59

$CDCl_3$, 151 MHz, 23 ºC.

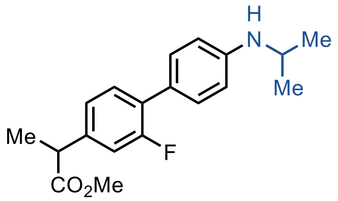

59

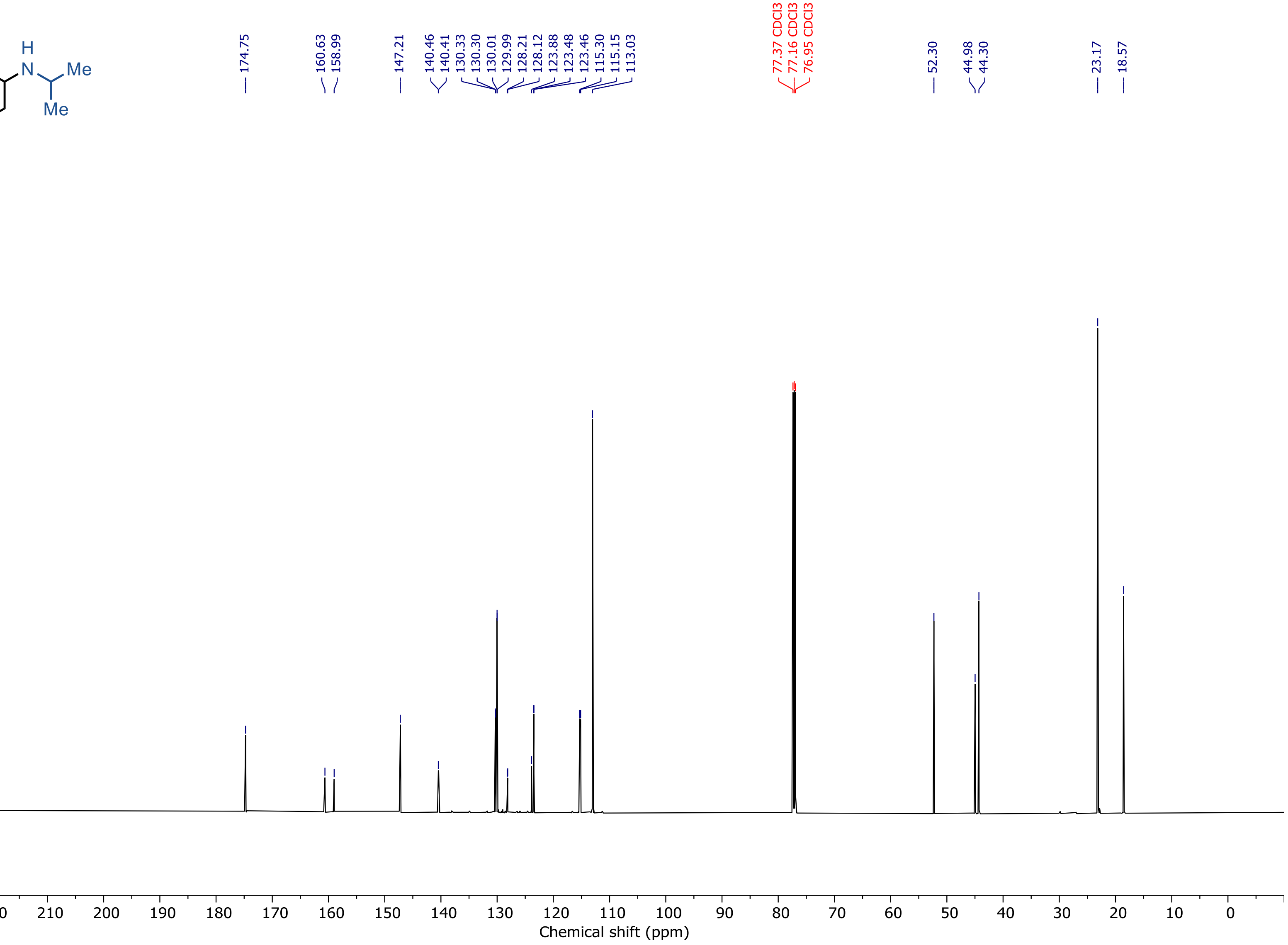

^{19}F NMR of 59

$CDCl_3$, 565 MHz, 23 ºC.

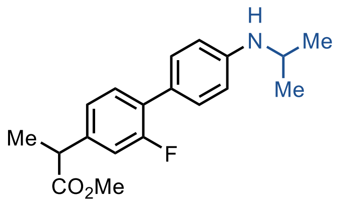

59

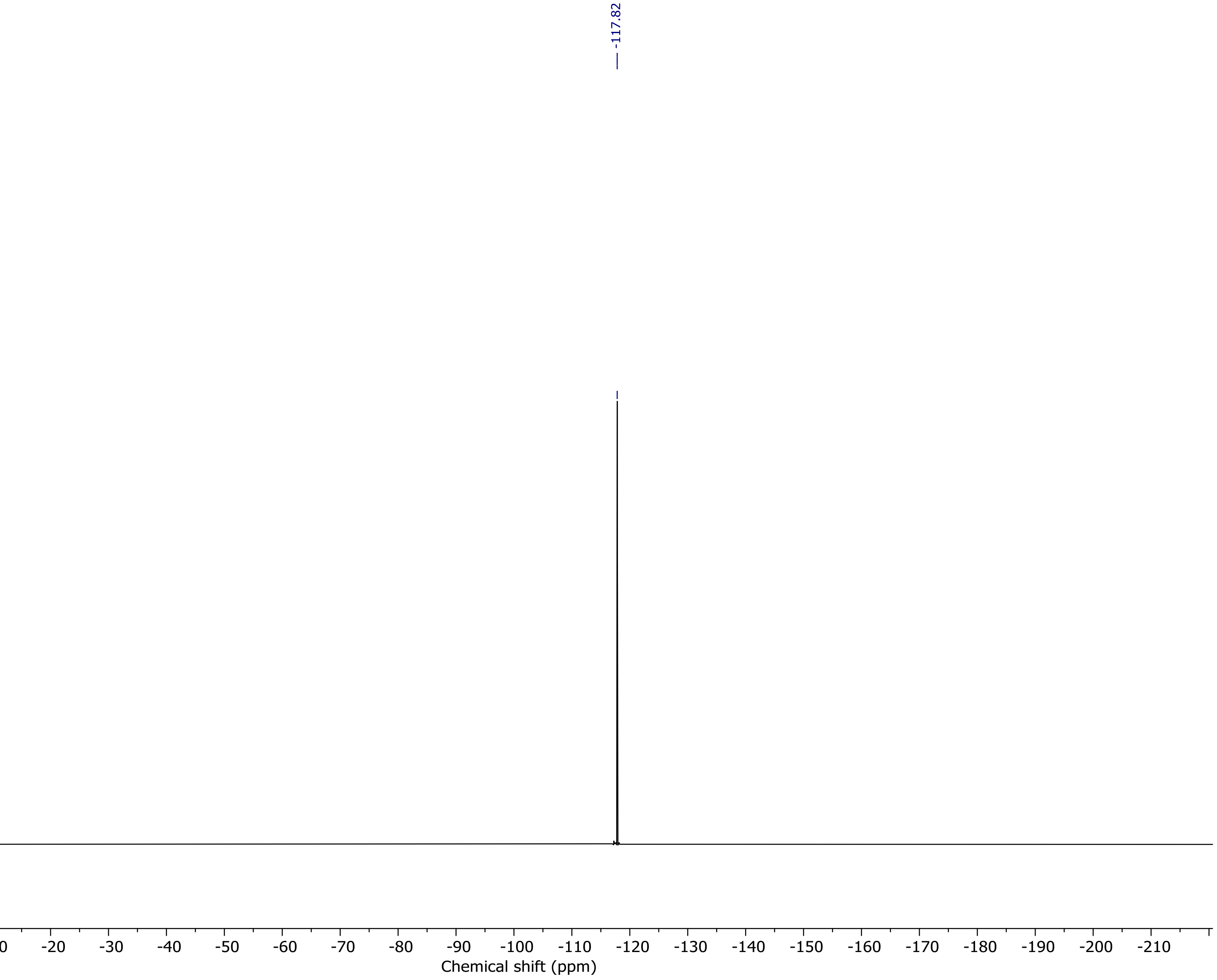

^{1}H NMR of 60

CDCl3, 600 MHz, 23 ºC.

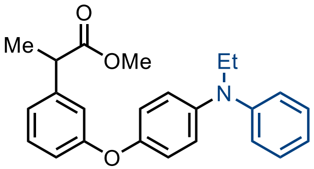

60

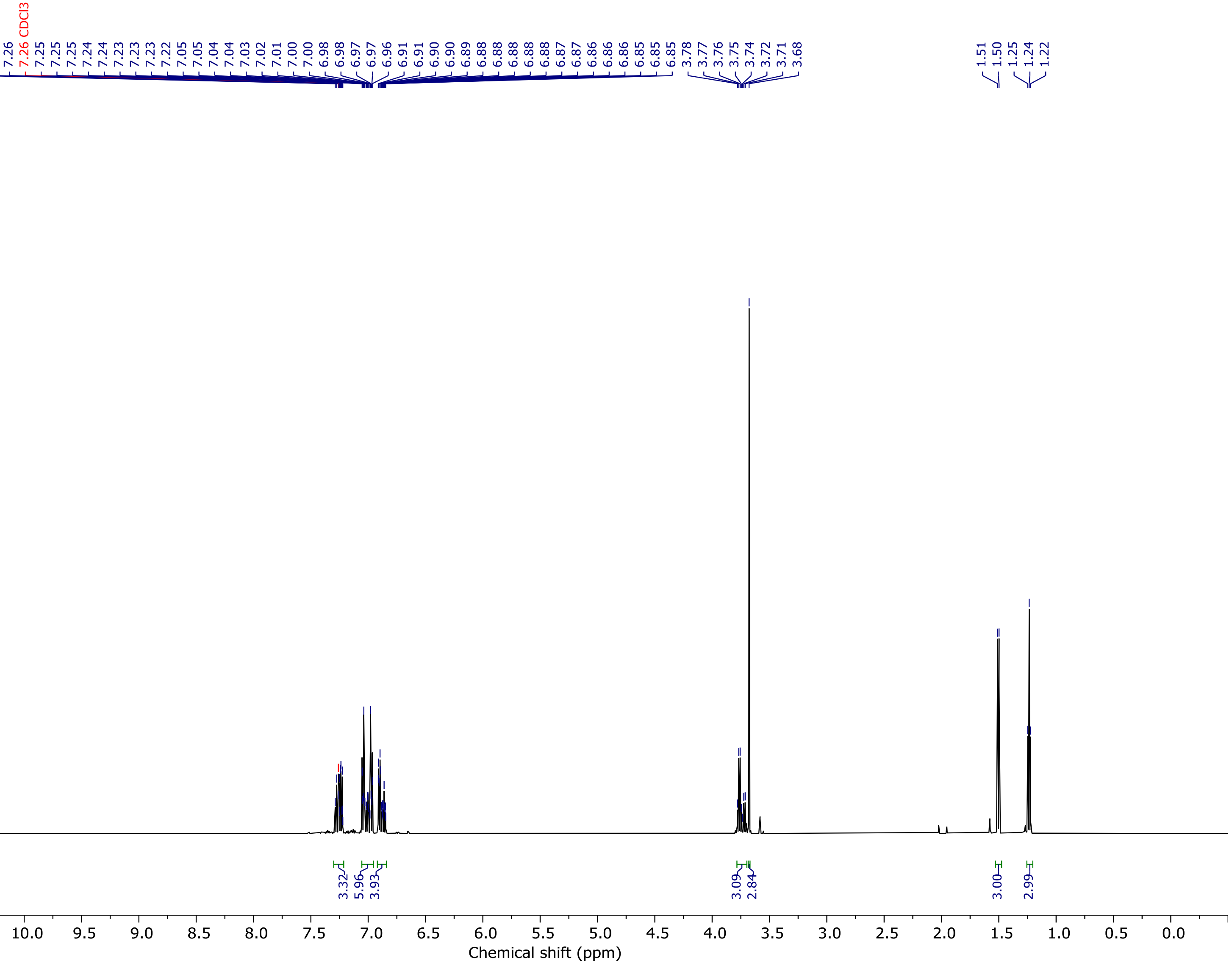

^{13}C NMR of 60

$CDCl_3$, 151 MHz, 23 ºC.

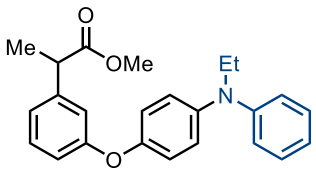

60

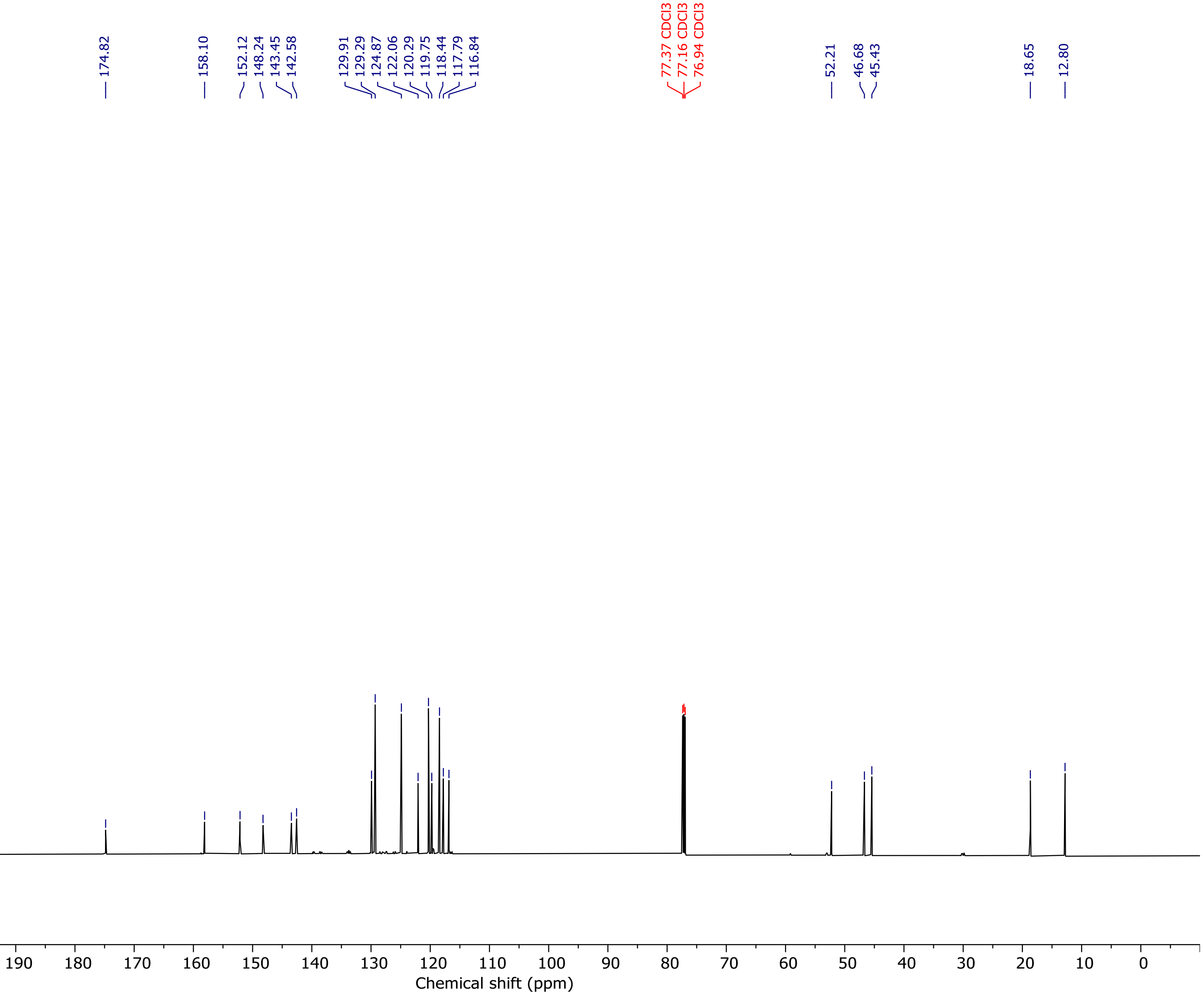

^{1}H NMR of 61

CDCl$_3$, 600 MHz, 23 ºC.

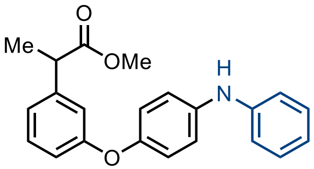

61

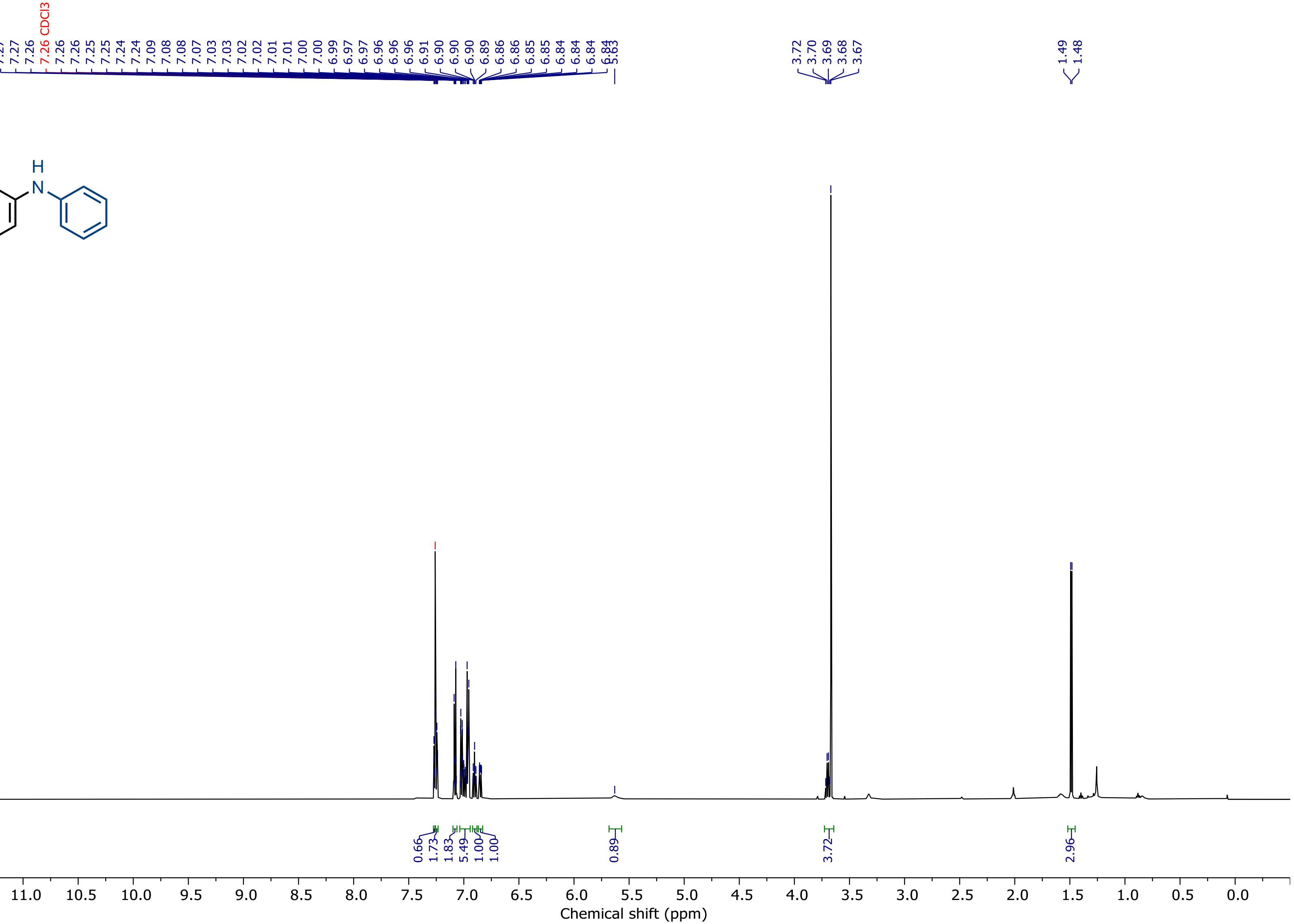

^{13}C NMR of 61

$CDCl_3$, 151 MHz, 23 ºC.

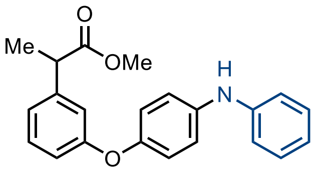

61

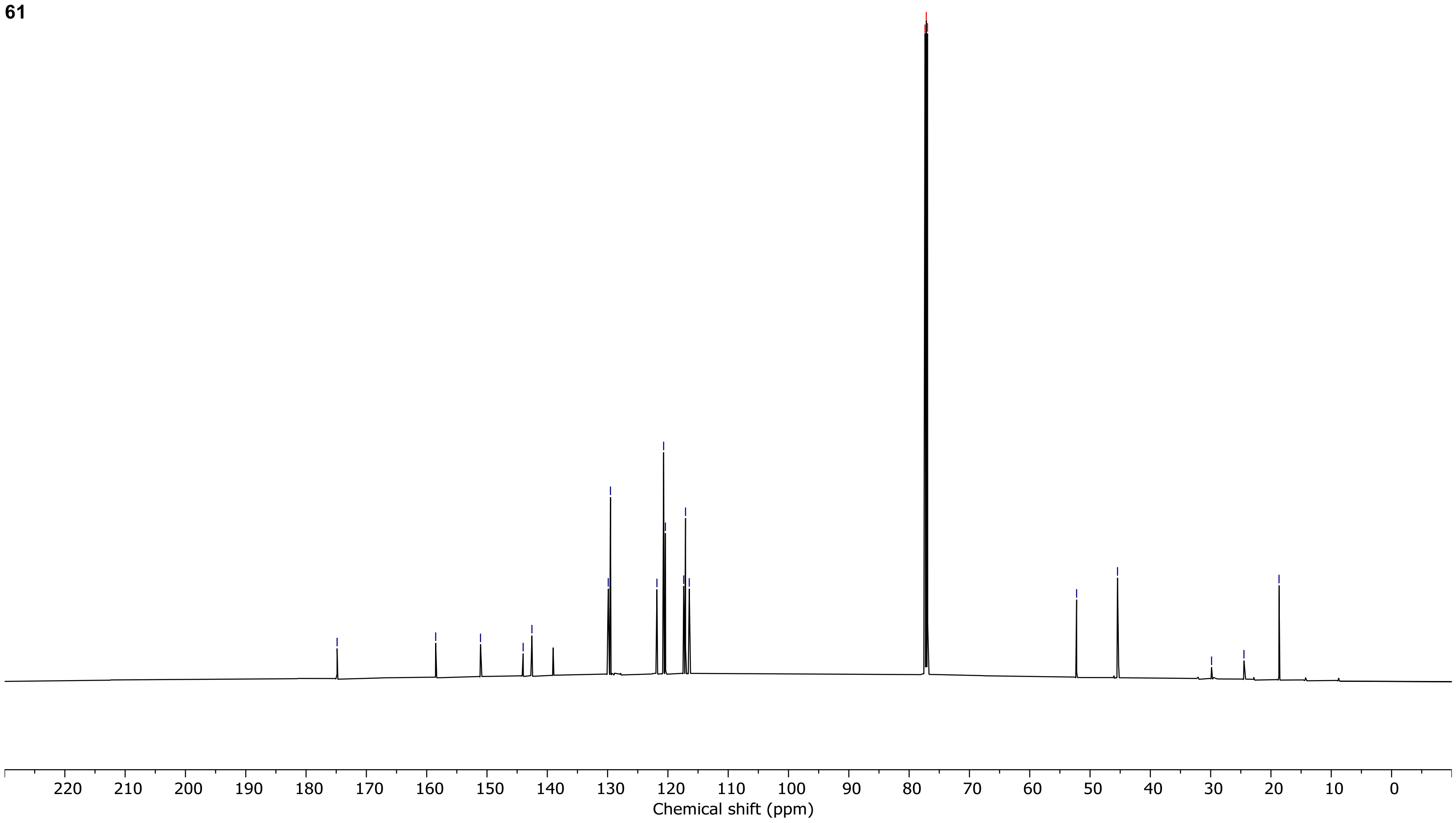

^{1}H NMR of 62

CDCl$_3$, 600 MHz, 23 ºC.

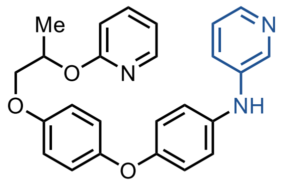

62

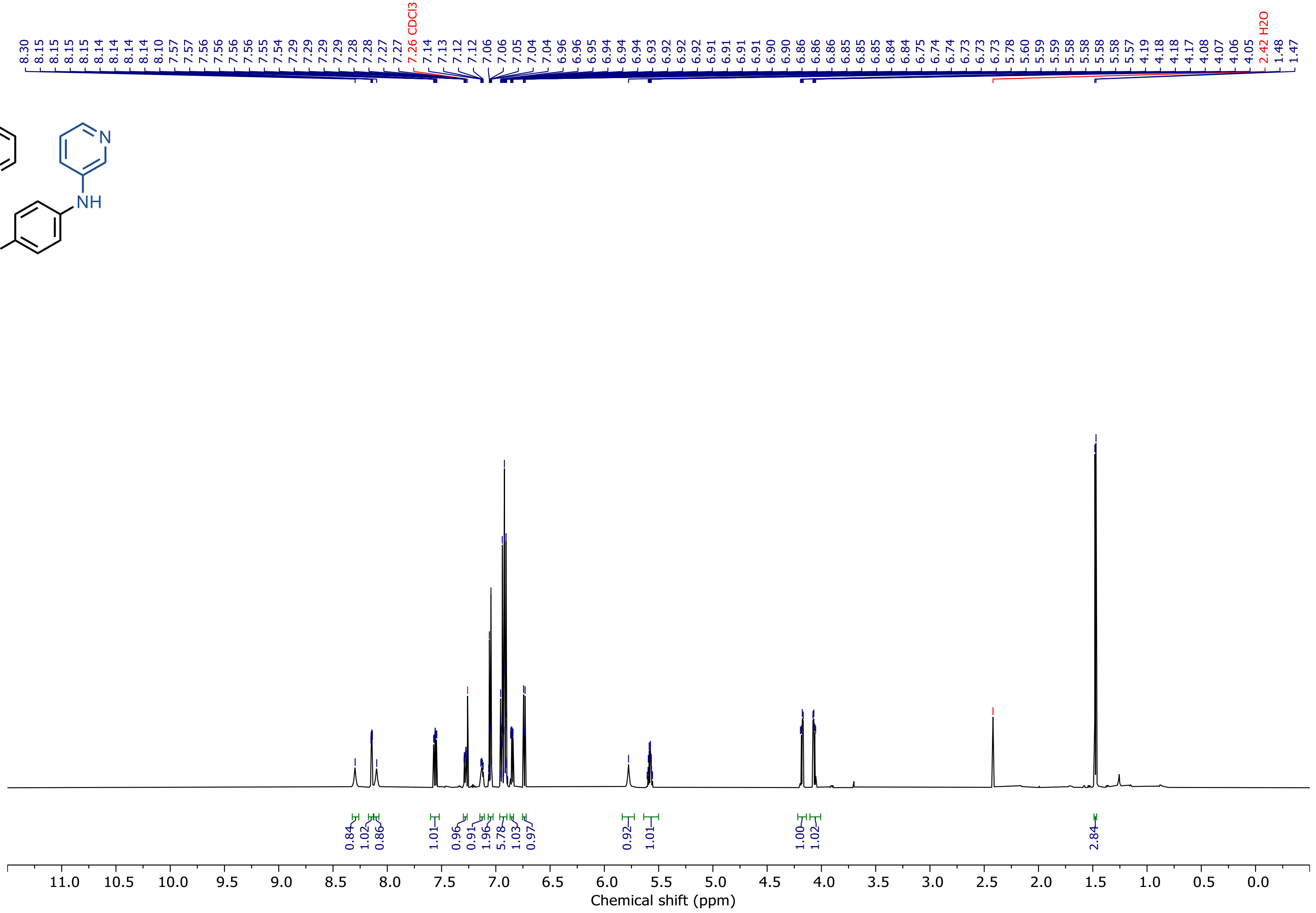

^{13}C NMR of 62

$CDCl_3$, 151 MHz, 23 ºC.

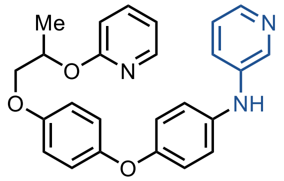

62

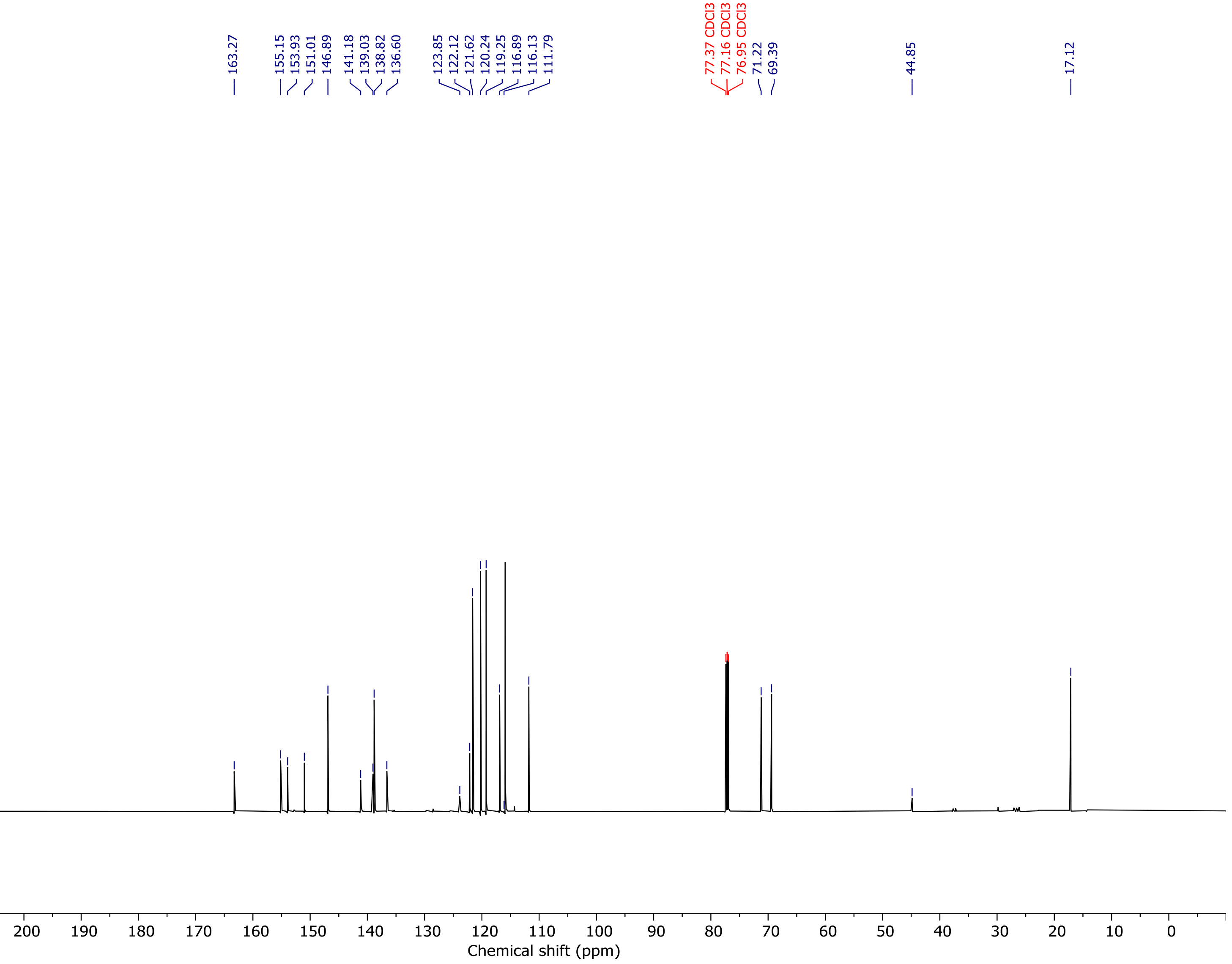

^{1}H NMR of 63

$CDCl_3$, 600 MHz, 23 ºC.

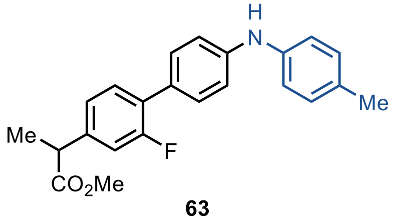

63

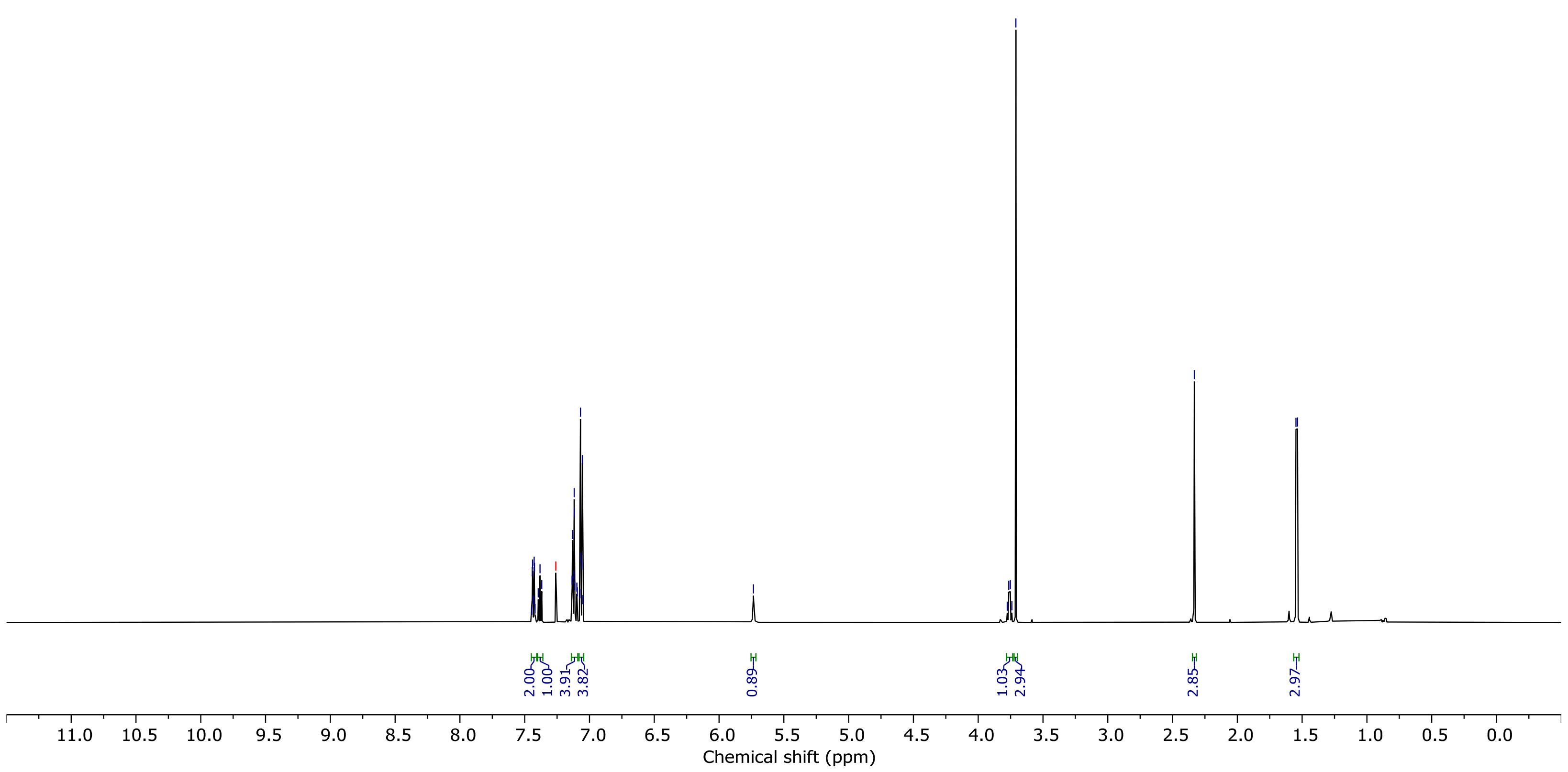

^{13}C NMR of 63

$CDCl_3$, 151 MHz, 23 ºC.

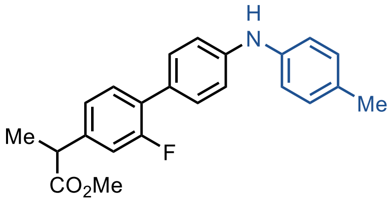
63

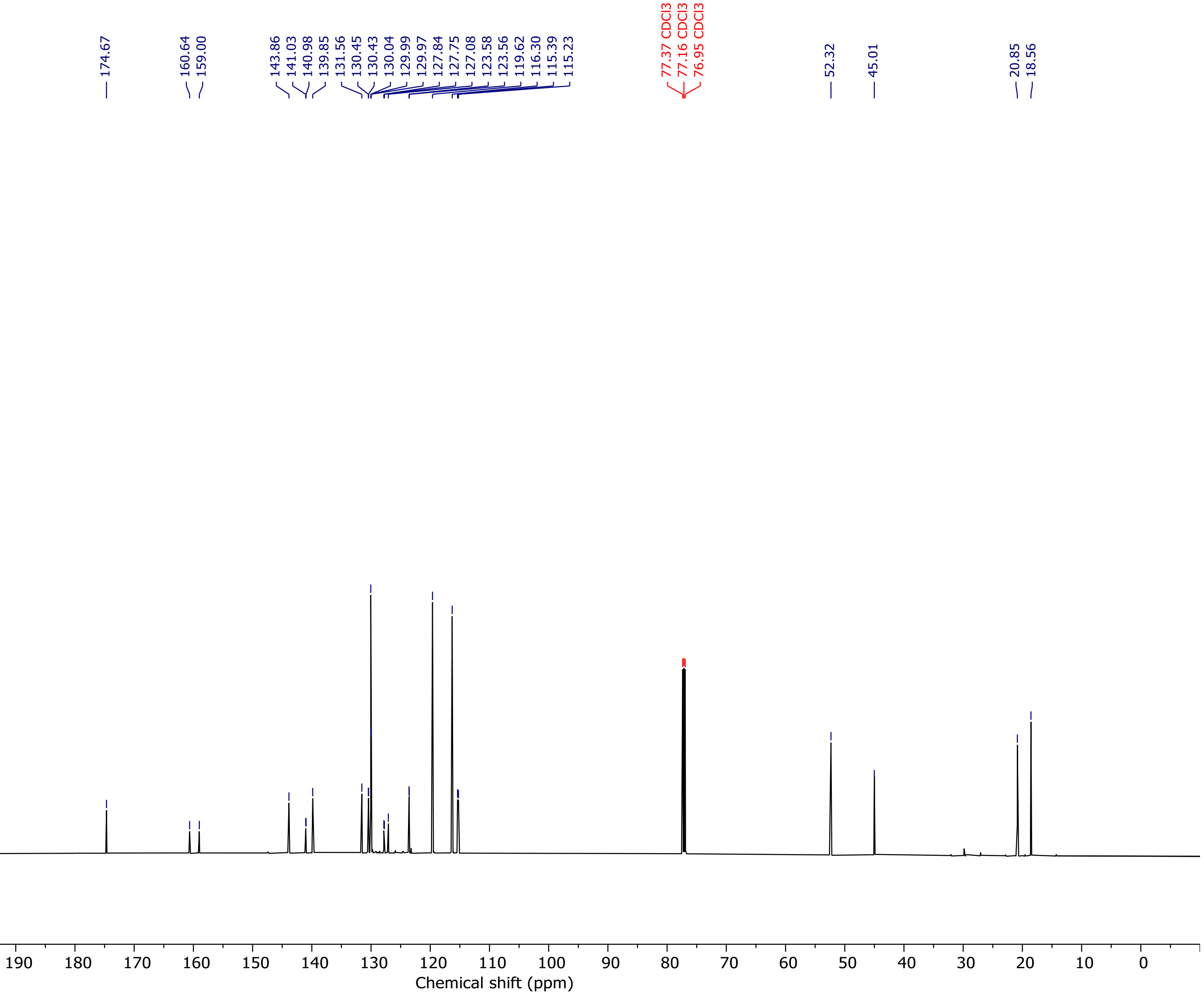

^{19}F NMR of 63

$CDCl_3$, 565 MHz, 23 ºC.

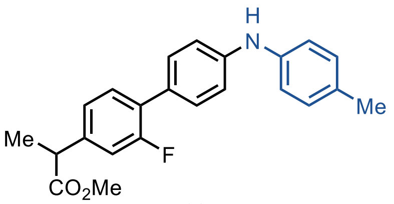

63

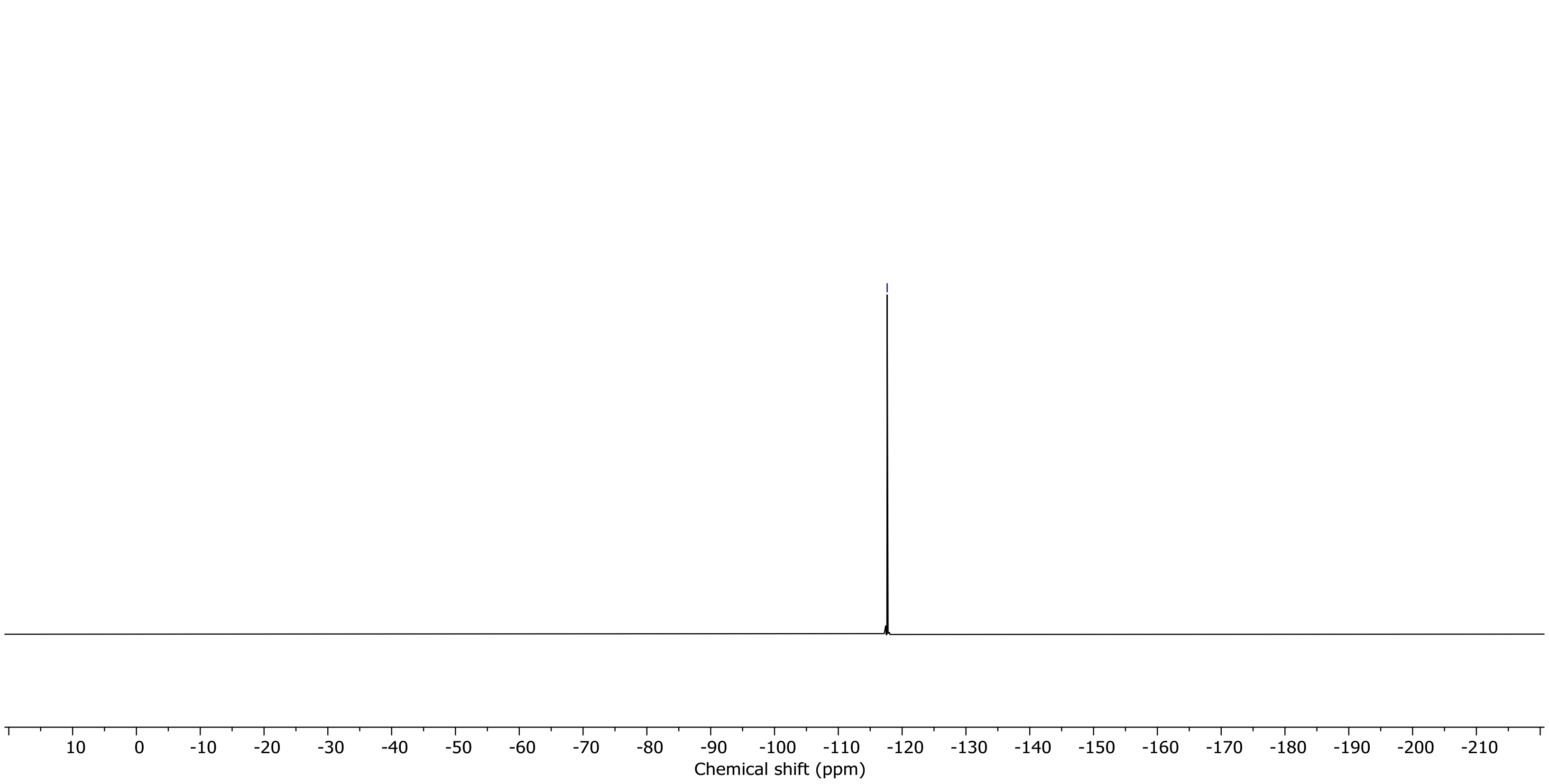

^{1}H NMR of 64

CDCl3, 600 MHz, 23 ºC.

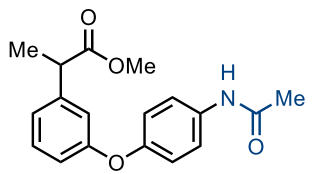

64

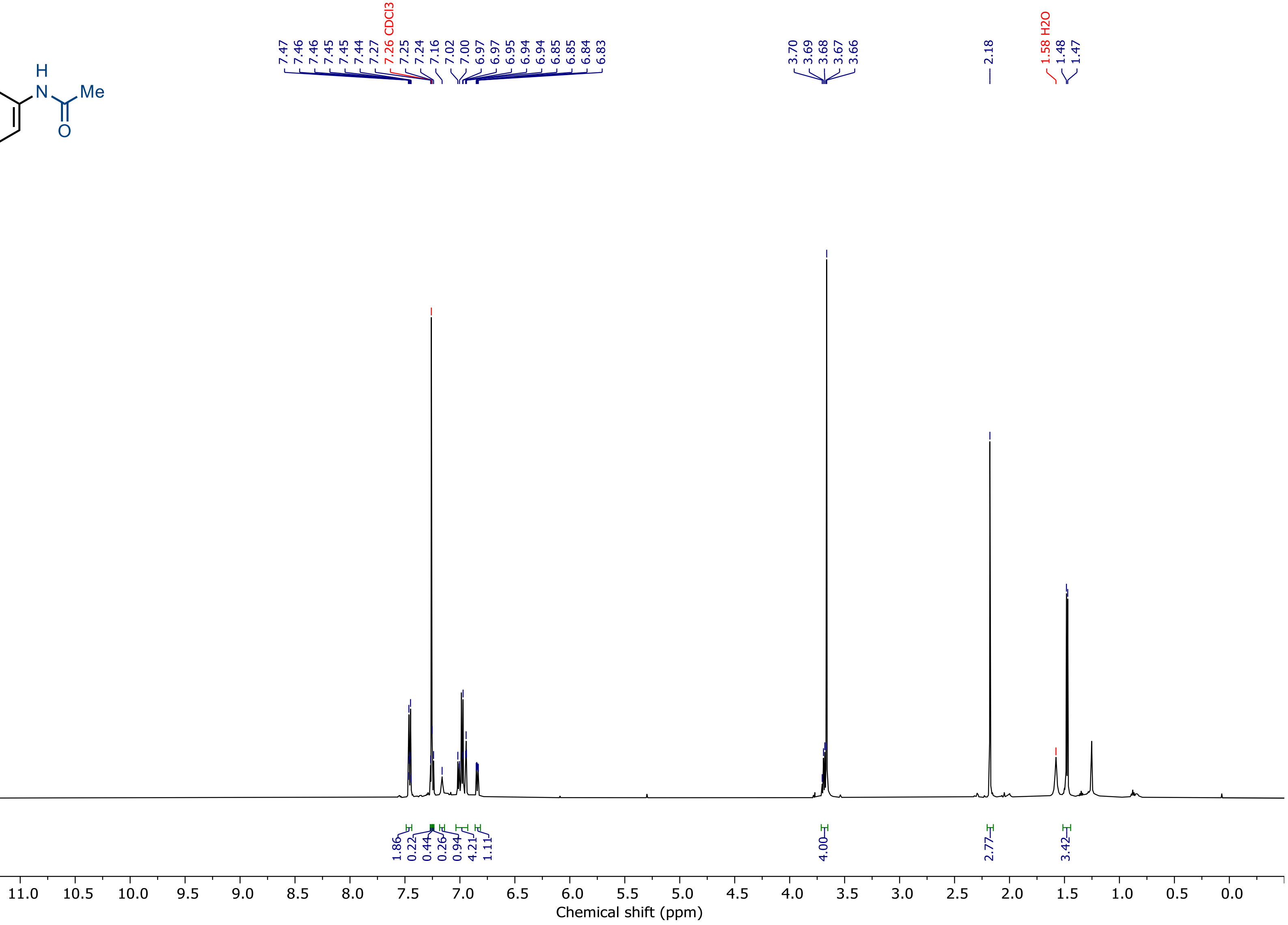

^{13}C NMR of 64

$CDCl_3$, 151 MHz, 23 ºC.

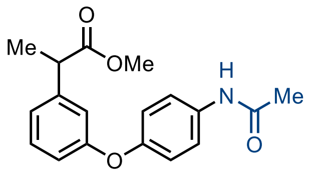

64

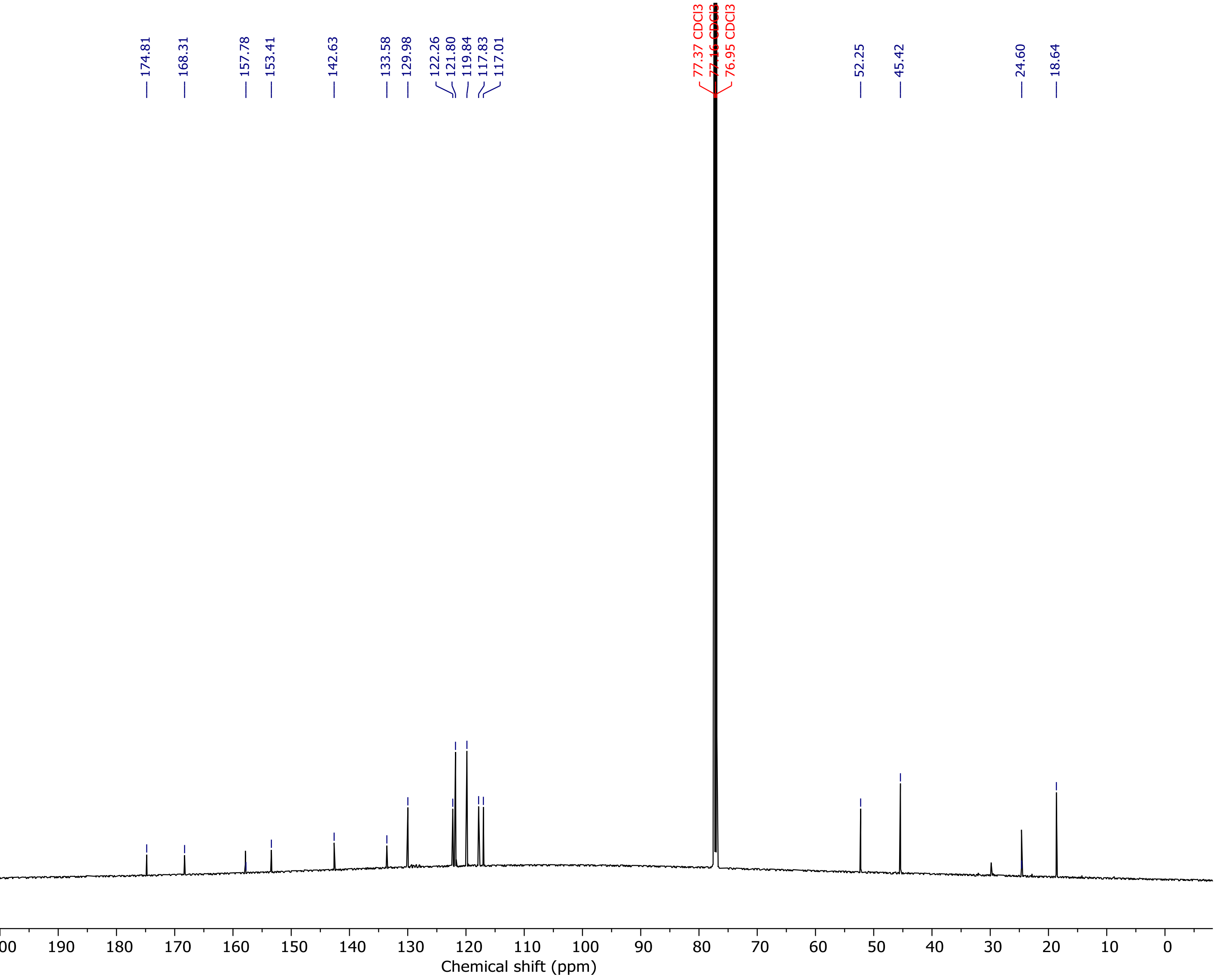

^{1}H NMR of 65

$CDCl_3$, 600 MHz, 23 ºC.

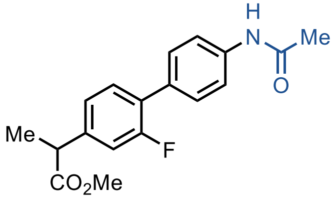

65

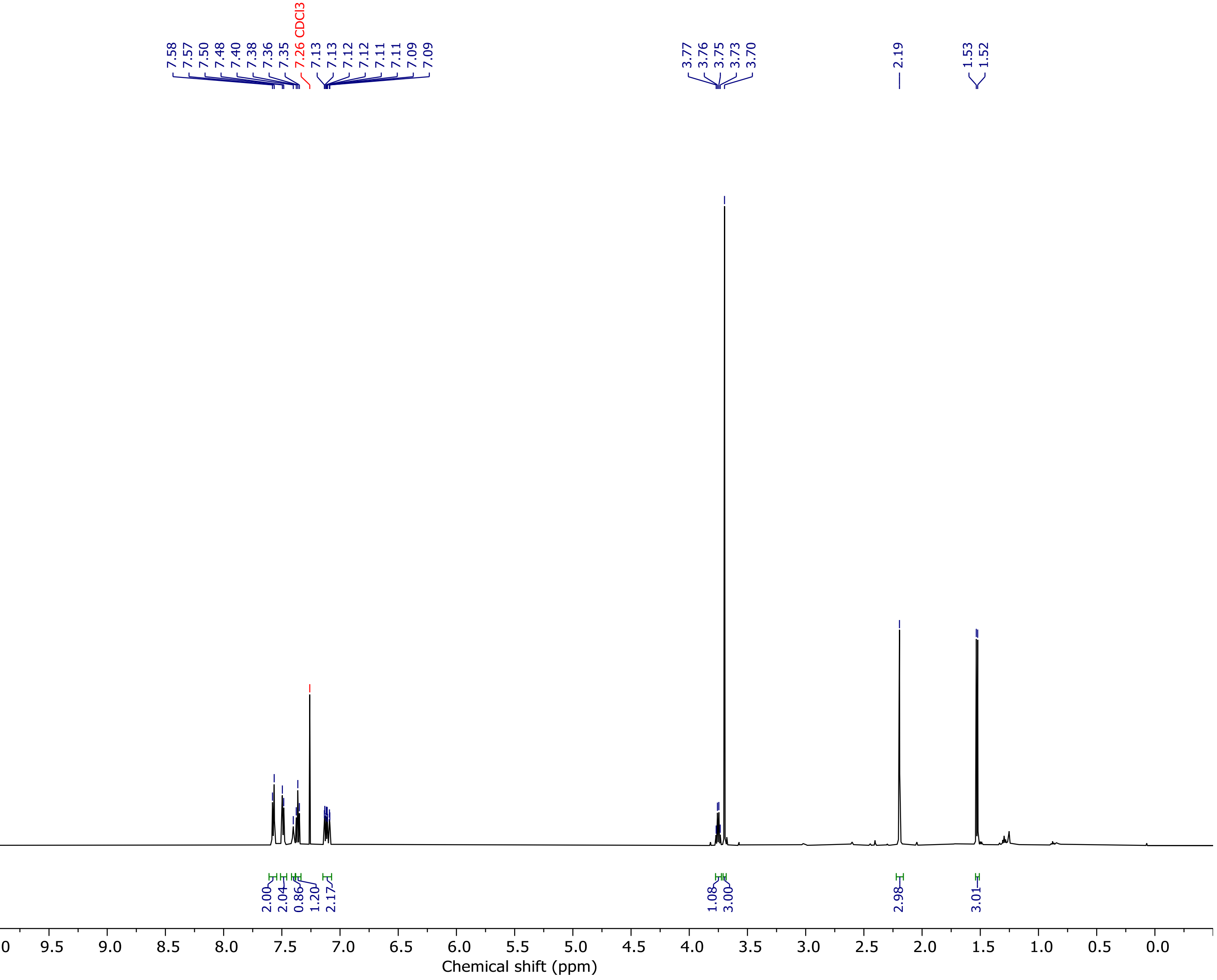

^{13}C NMR of 65

$CDCl_3$, 151 MHz, 23 ºC.

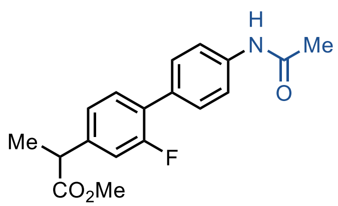

65

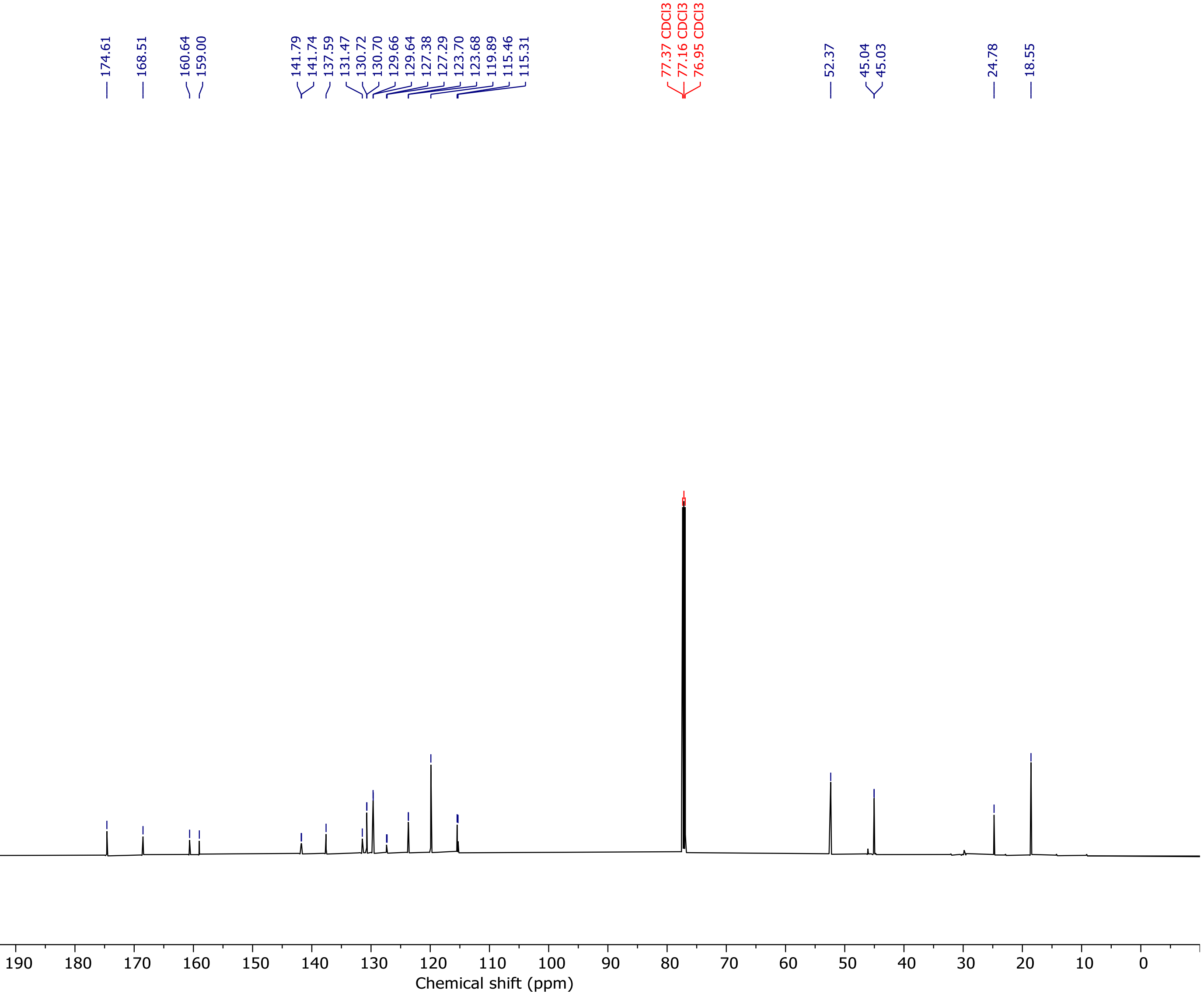

^{19}F NMR of 65

$CDCl_3$, 565 MHz, 23 ºC.

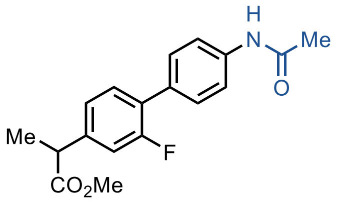

65

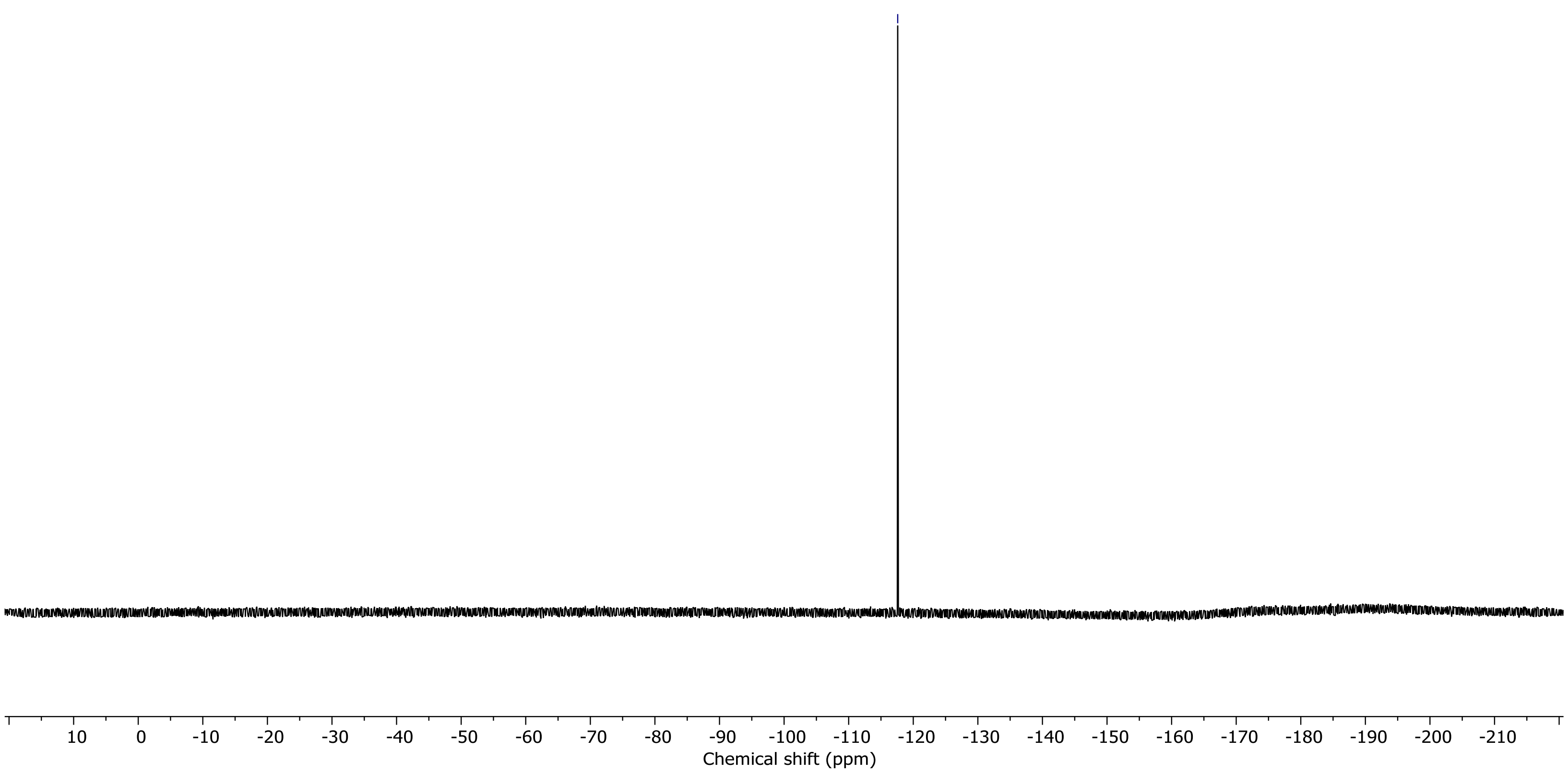

^{1}H NMR of 66

$CDCl_3$, 600 MHz, 23 ºC.

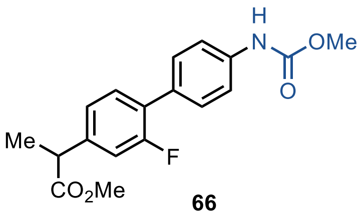

66

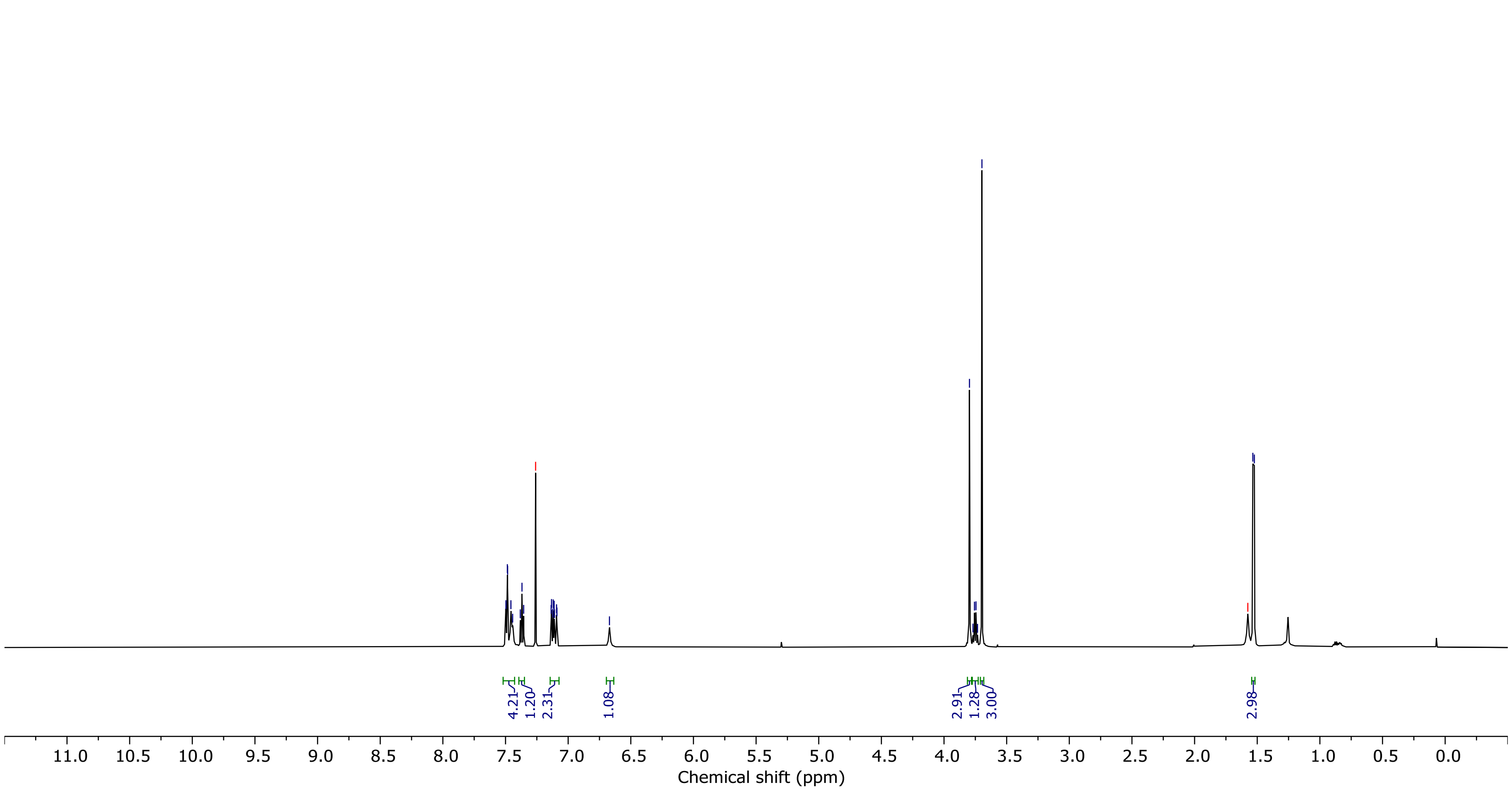

^{13}C NMR of 66

$CDCl_3$, 151 MHz, 23 ºC.

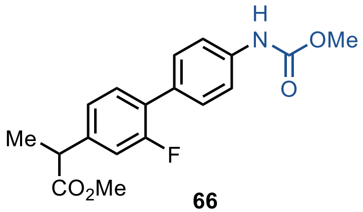

66

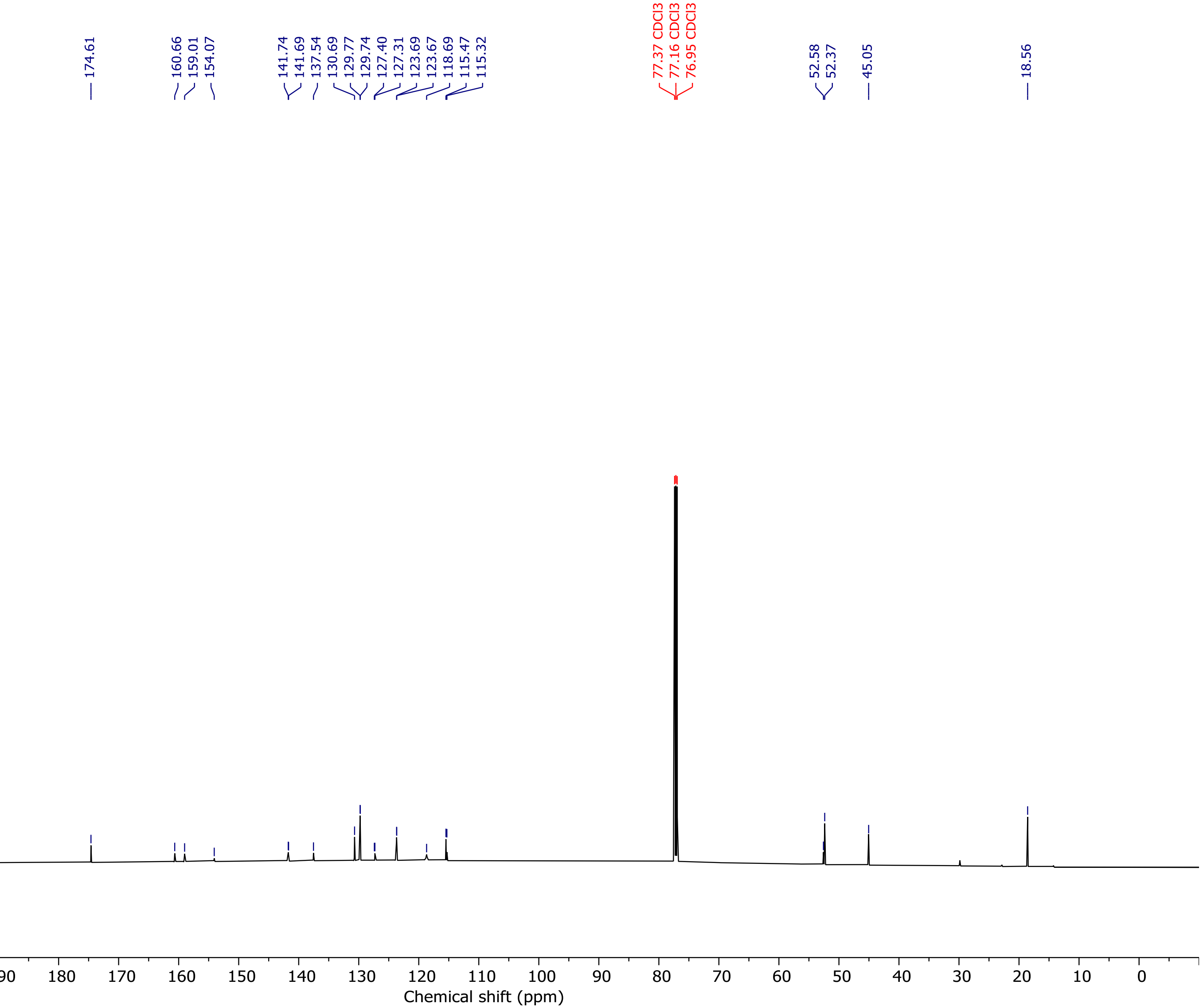

^{19}F NMR of 66

$CDCl_3$, 565 MHz, 23 ºC.

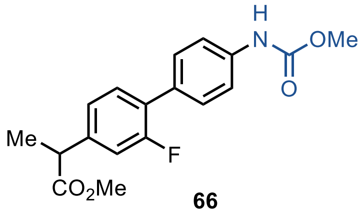

66

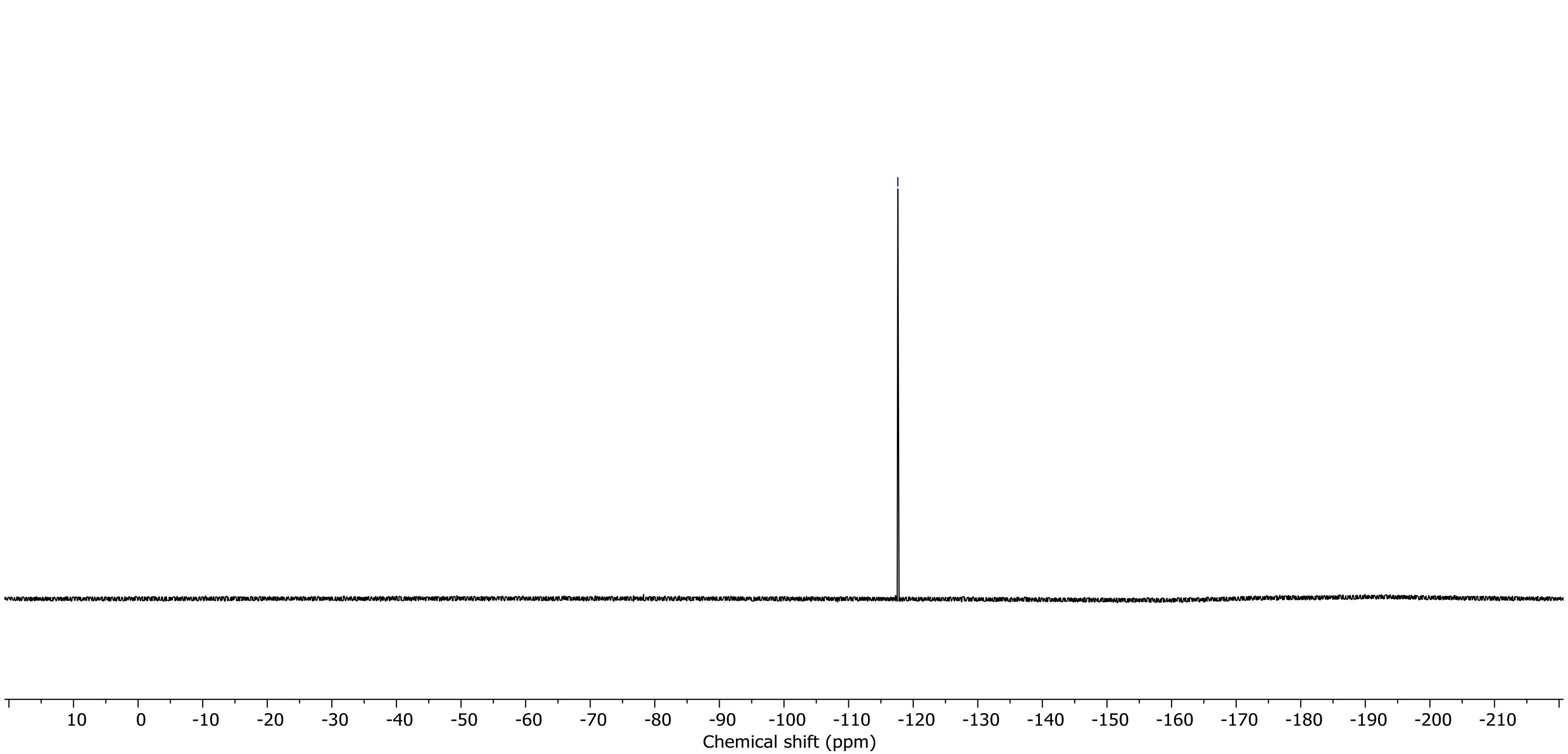

^{1}H NMR of 67

$CDCl_3$, 600 MHz, 23 ºC.

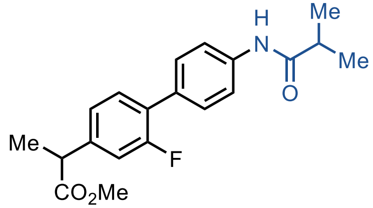

67

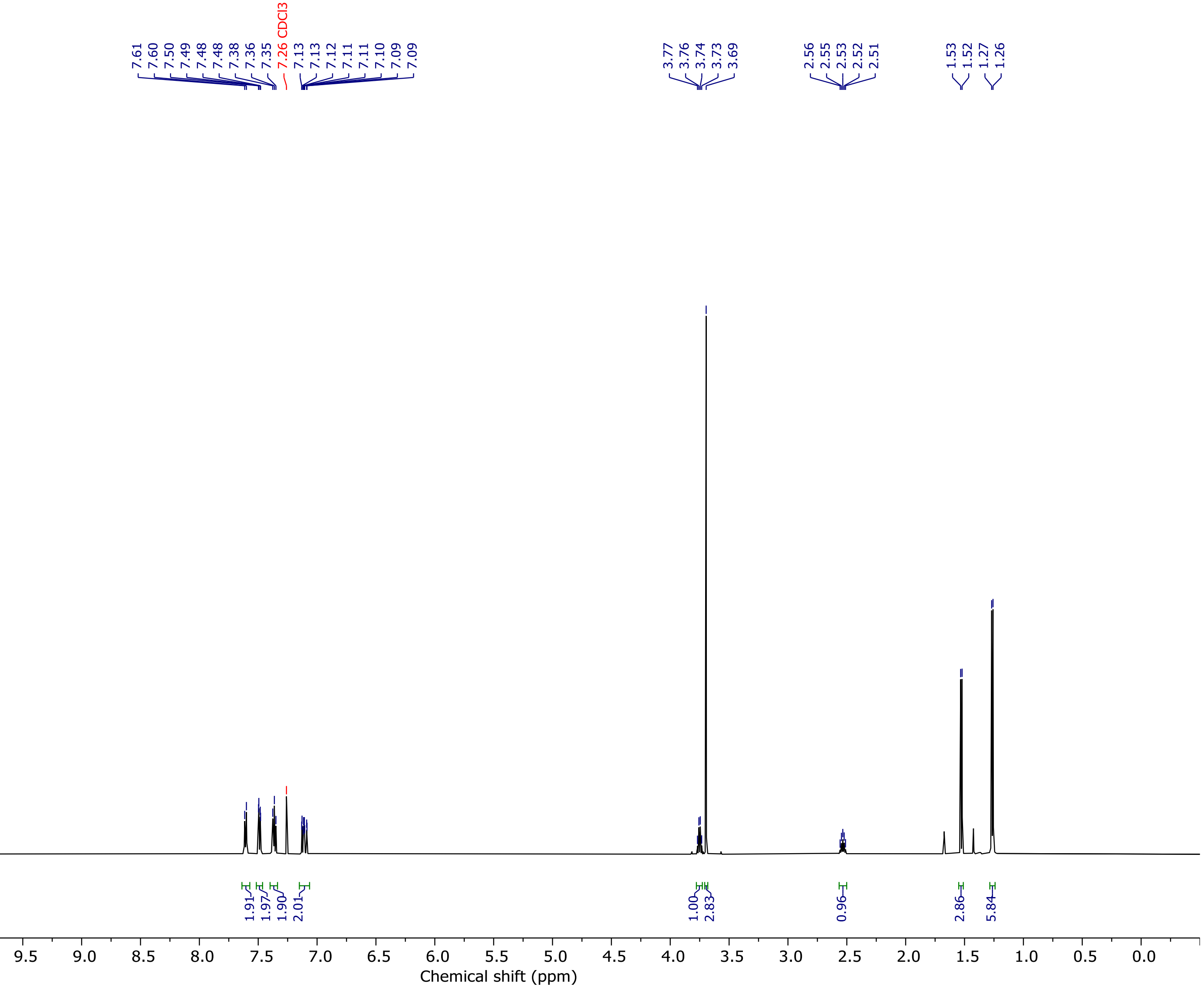

^{13}C NMR of 67

$CDCl_3$, 151 MHz, 23 ºC.

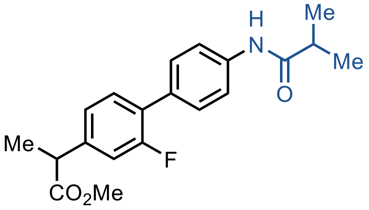

67

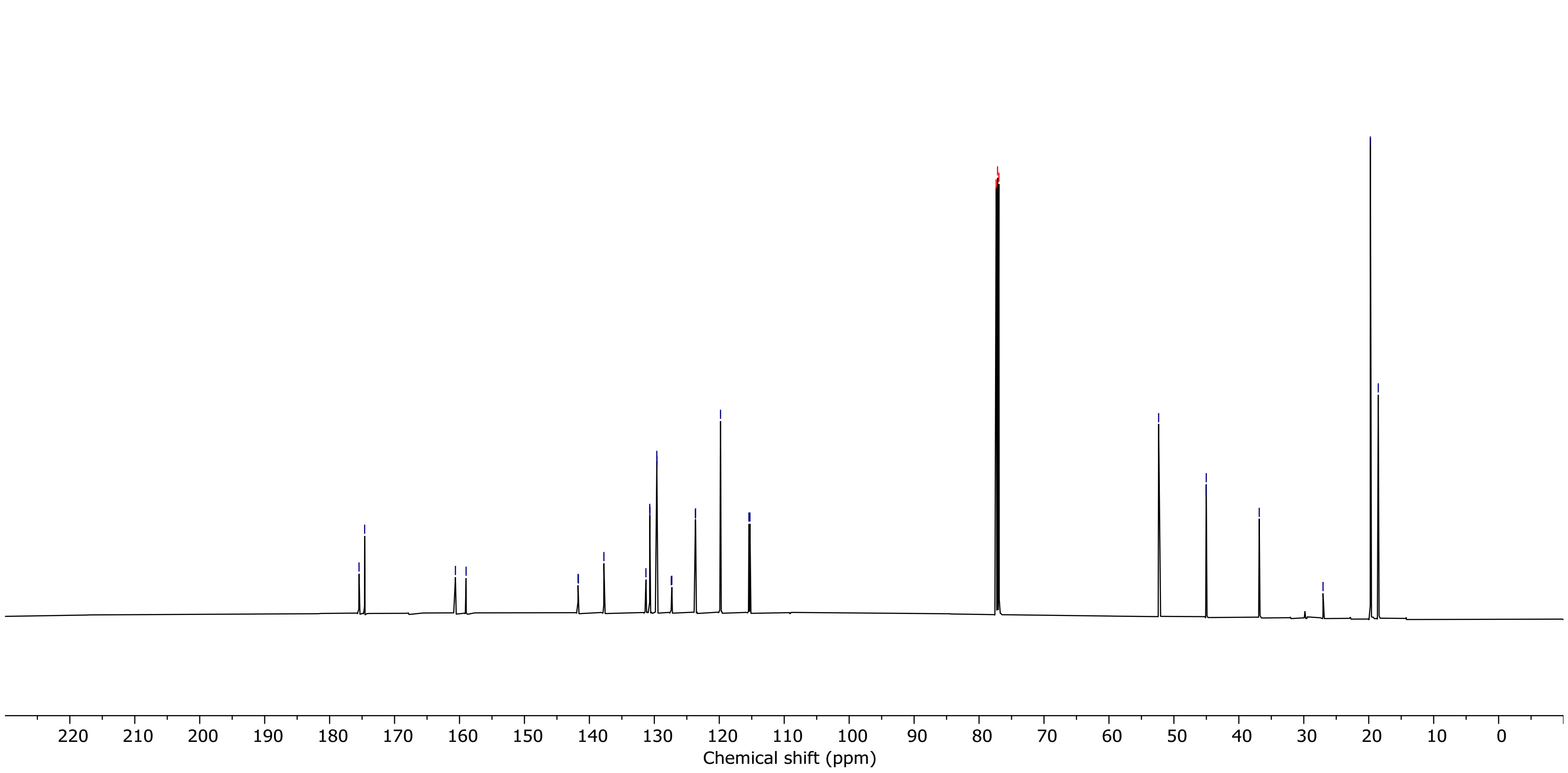

^{19}F NMR of 67

$CDCl_3$, 565 MHz, 23 ºC.

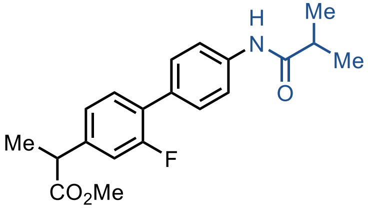

67

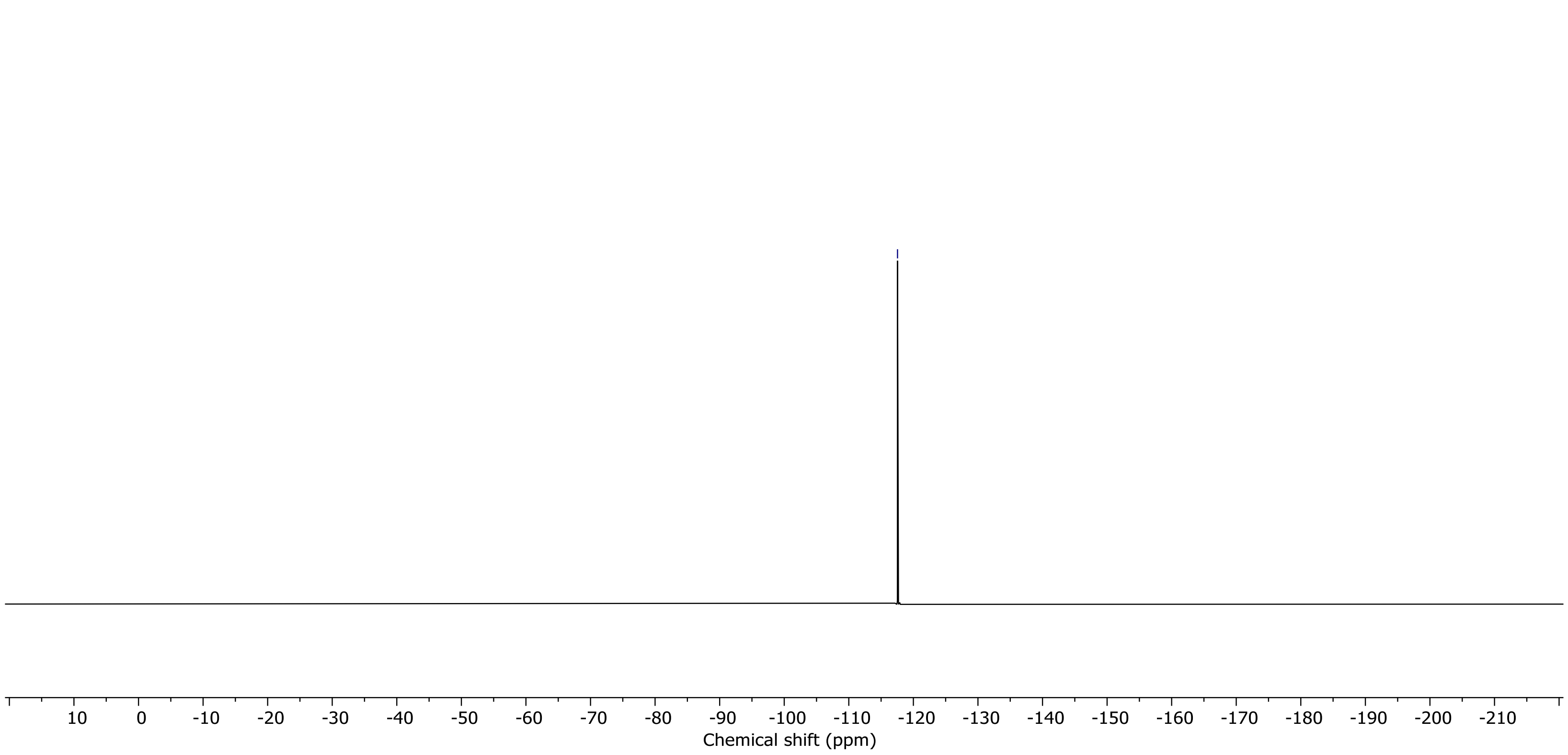

^{1}H NMR of 68

$CDCl_3$, 500 MHz, 23 ºC.

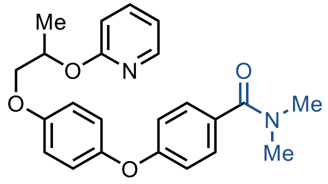

68

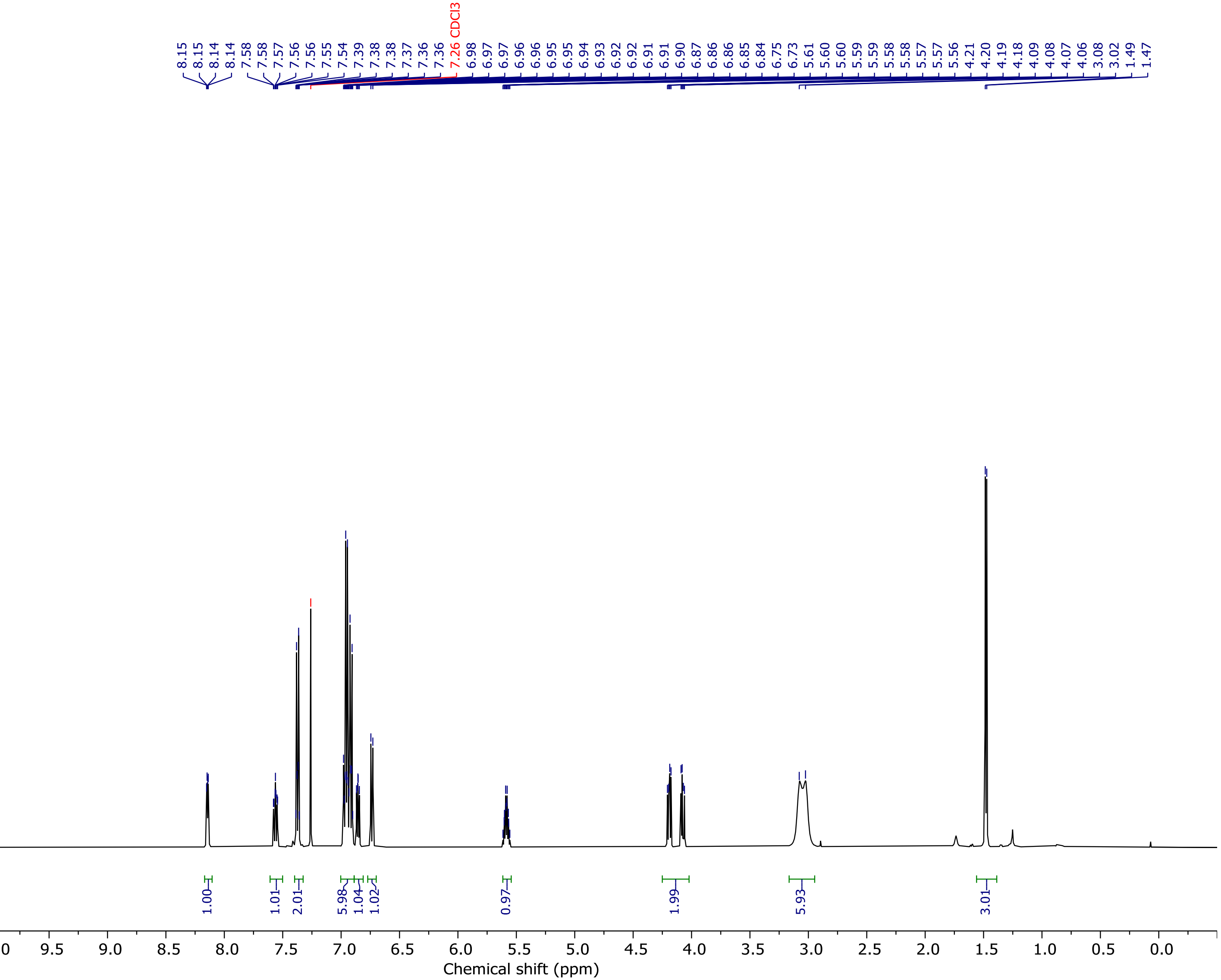

^{13}C NMR of 68

$CDCl_3$, 126 MHz, 23 ºC.

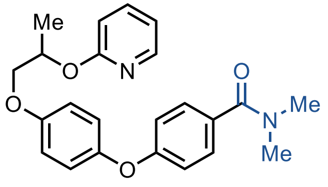

68

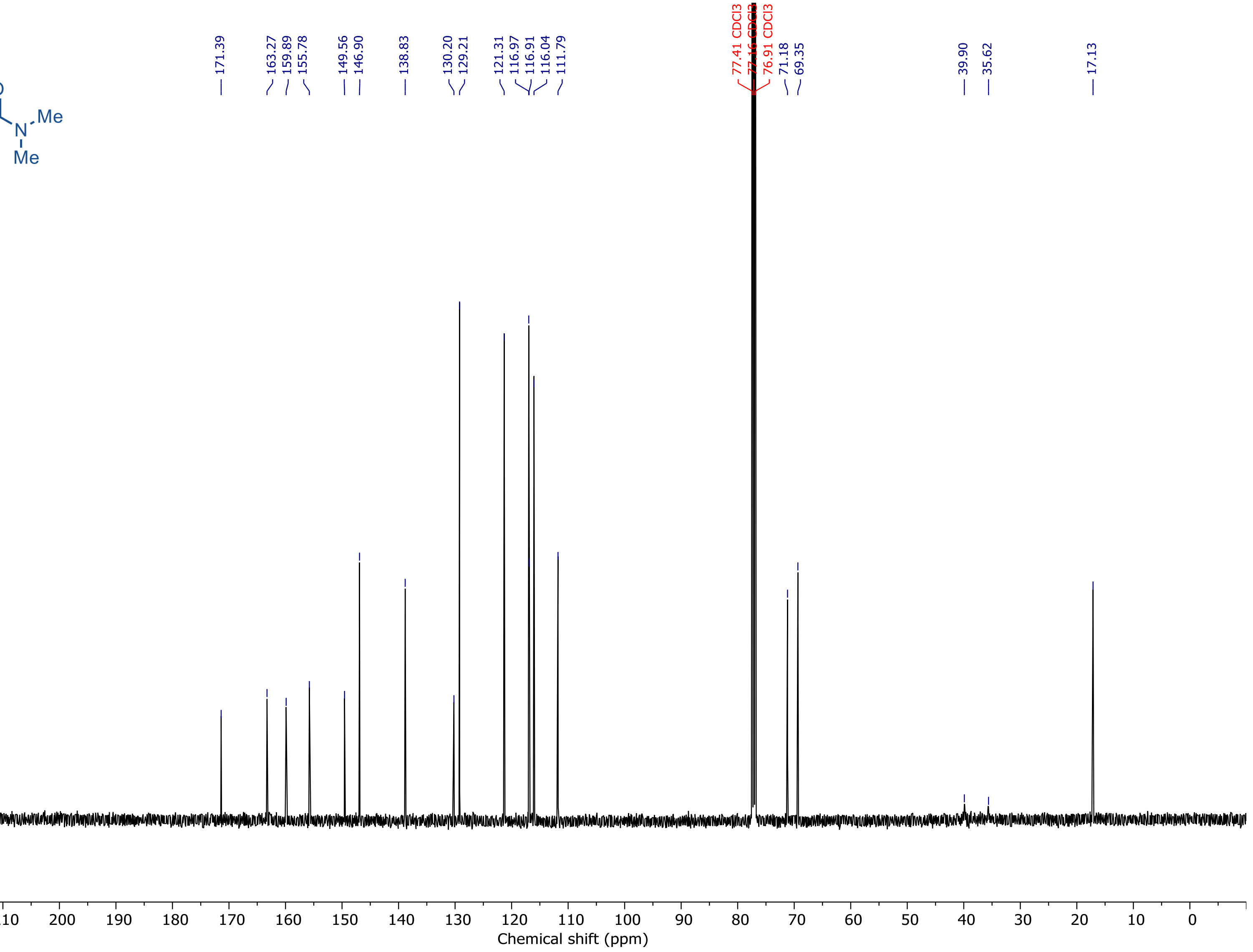

^{1}H NMR of 69

$CDCl_3$, 600 MHz, 23 ºC.

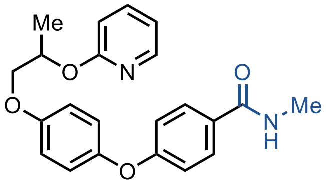

69

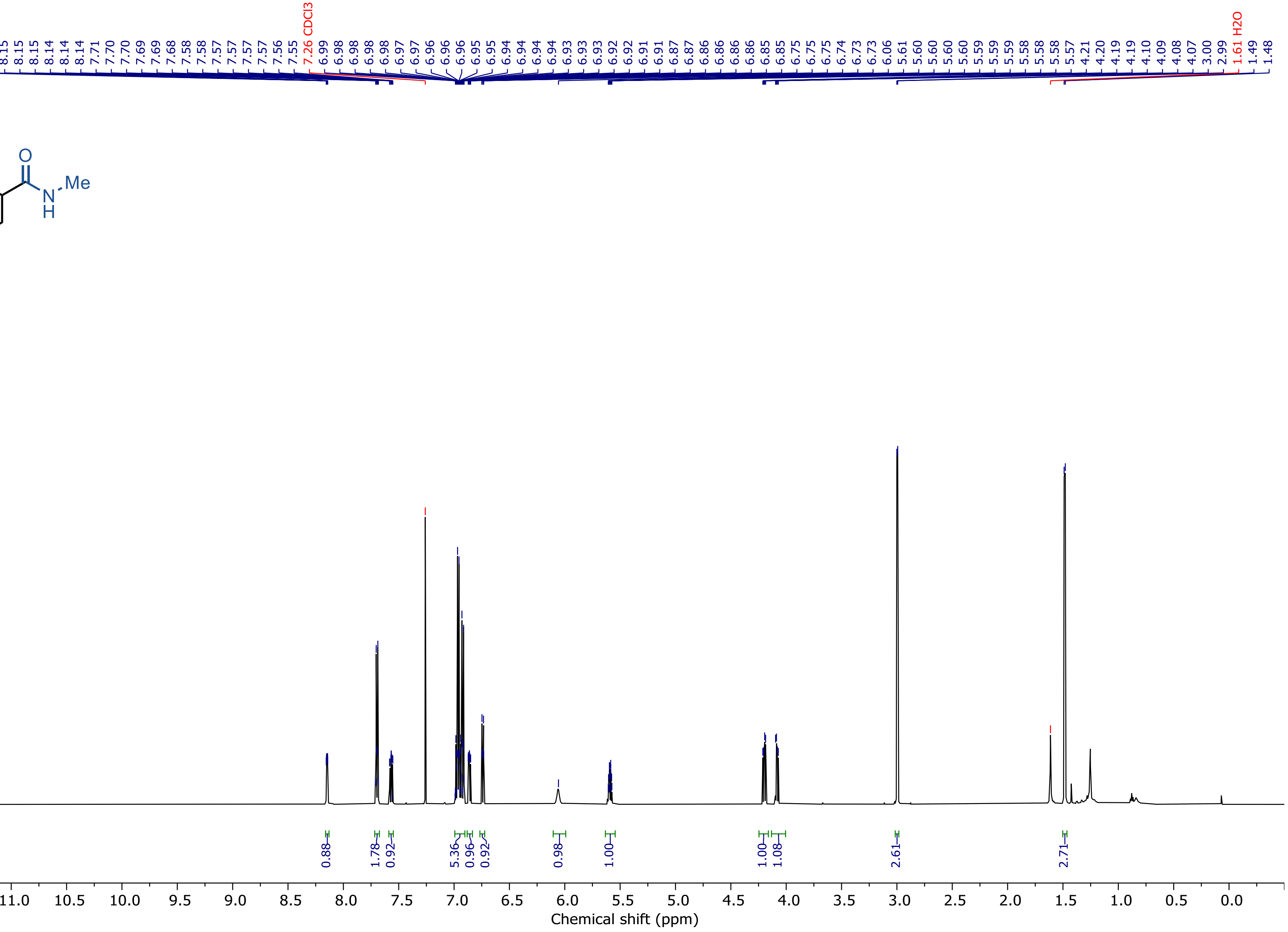

^{13}C NMR of 69

$CDCl_3$, 151 MHz, 23 ºC.

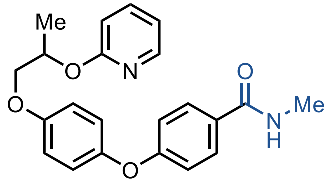

69

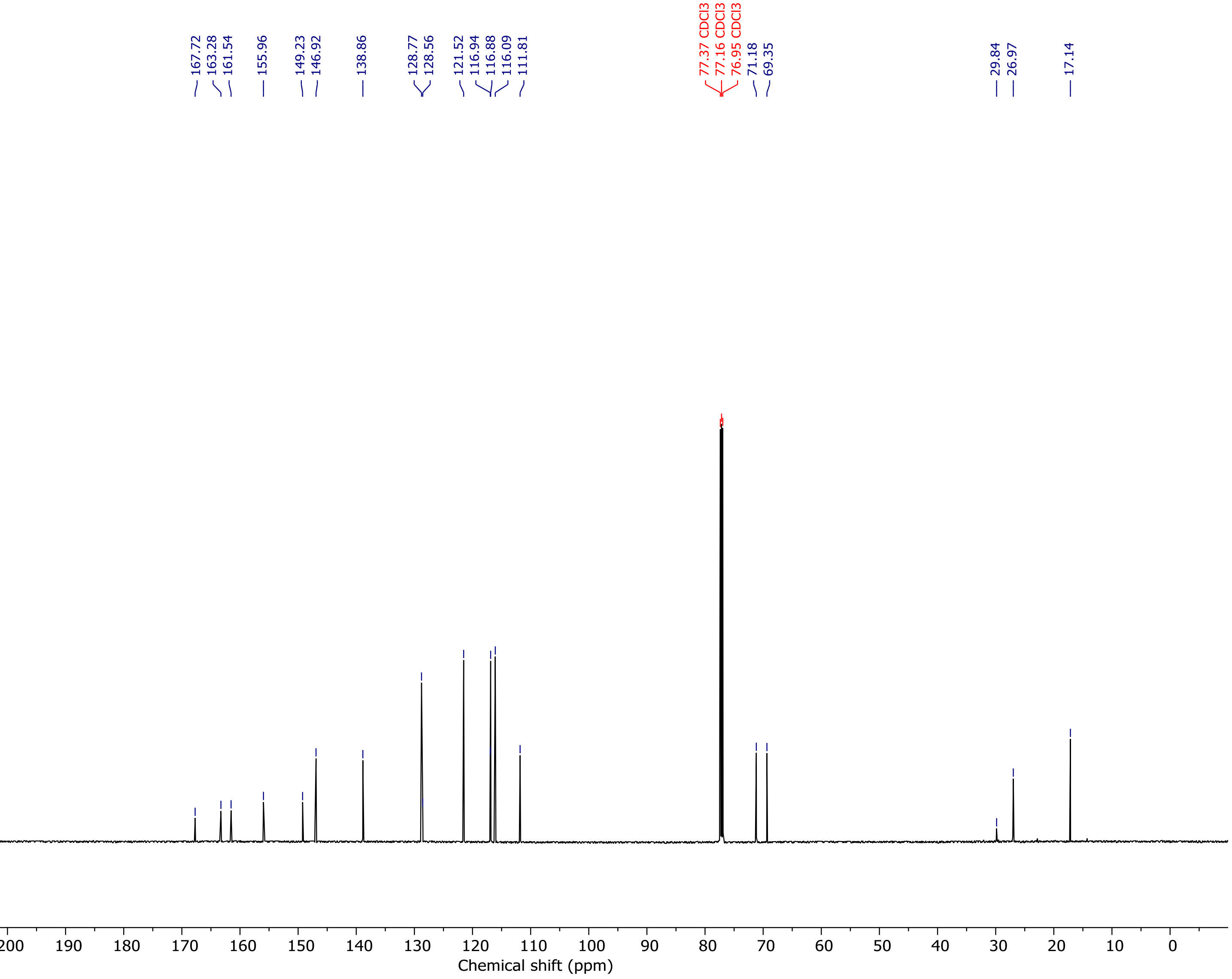

^{1}H NMR of 70

CDCl$_3$, 500 MHz, 23 ºC.

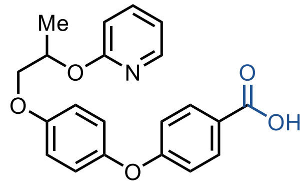

70

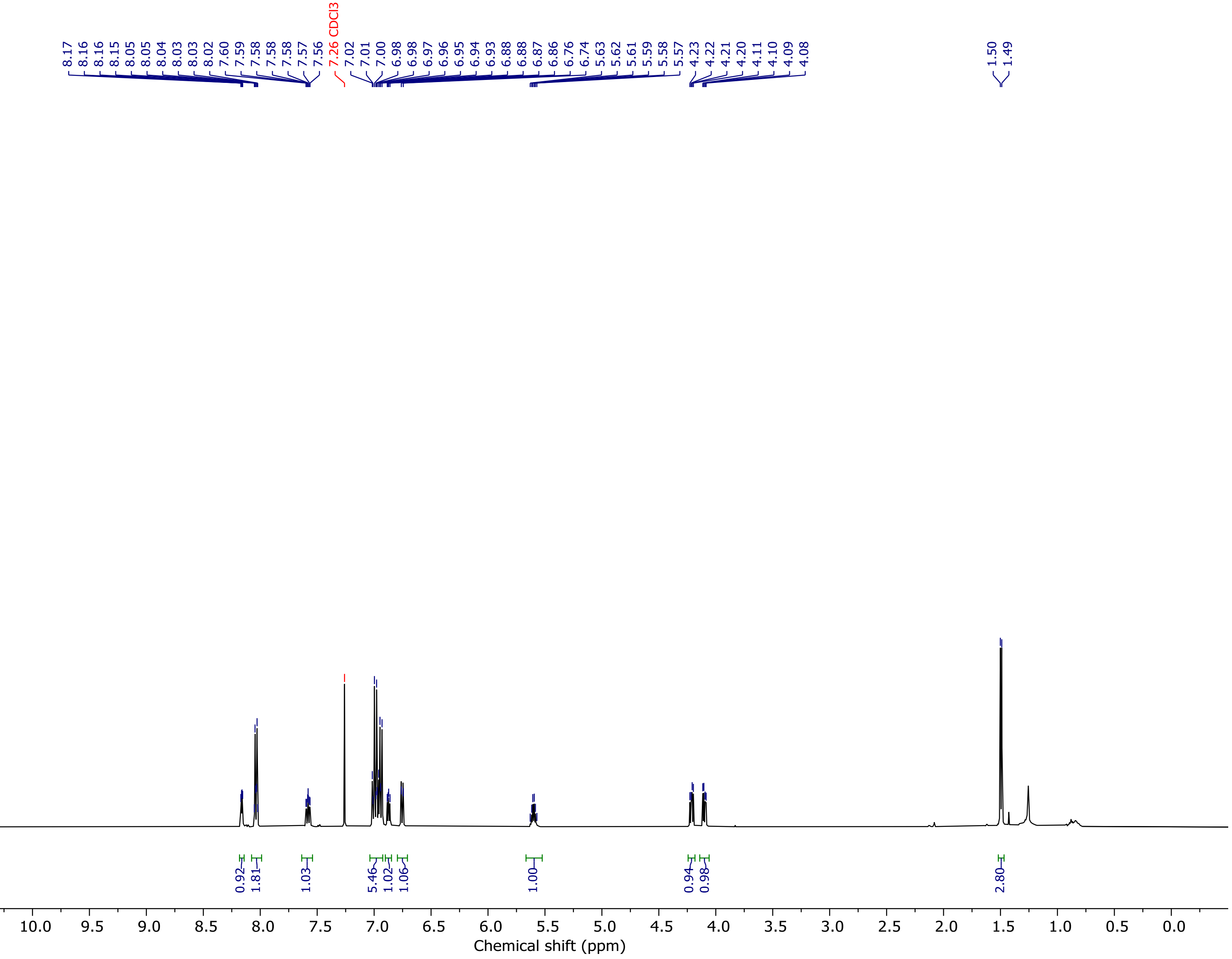

^{13}C NMR of 70

$CDCl_3$, 126 MHz, 23 ºC.

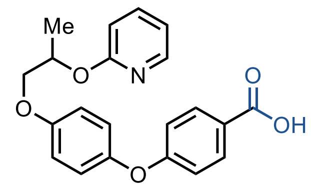

70

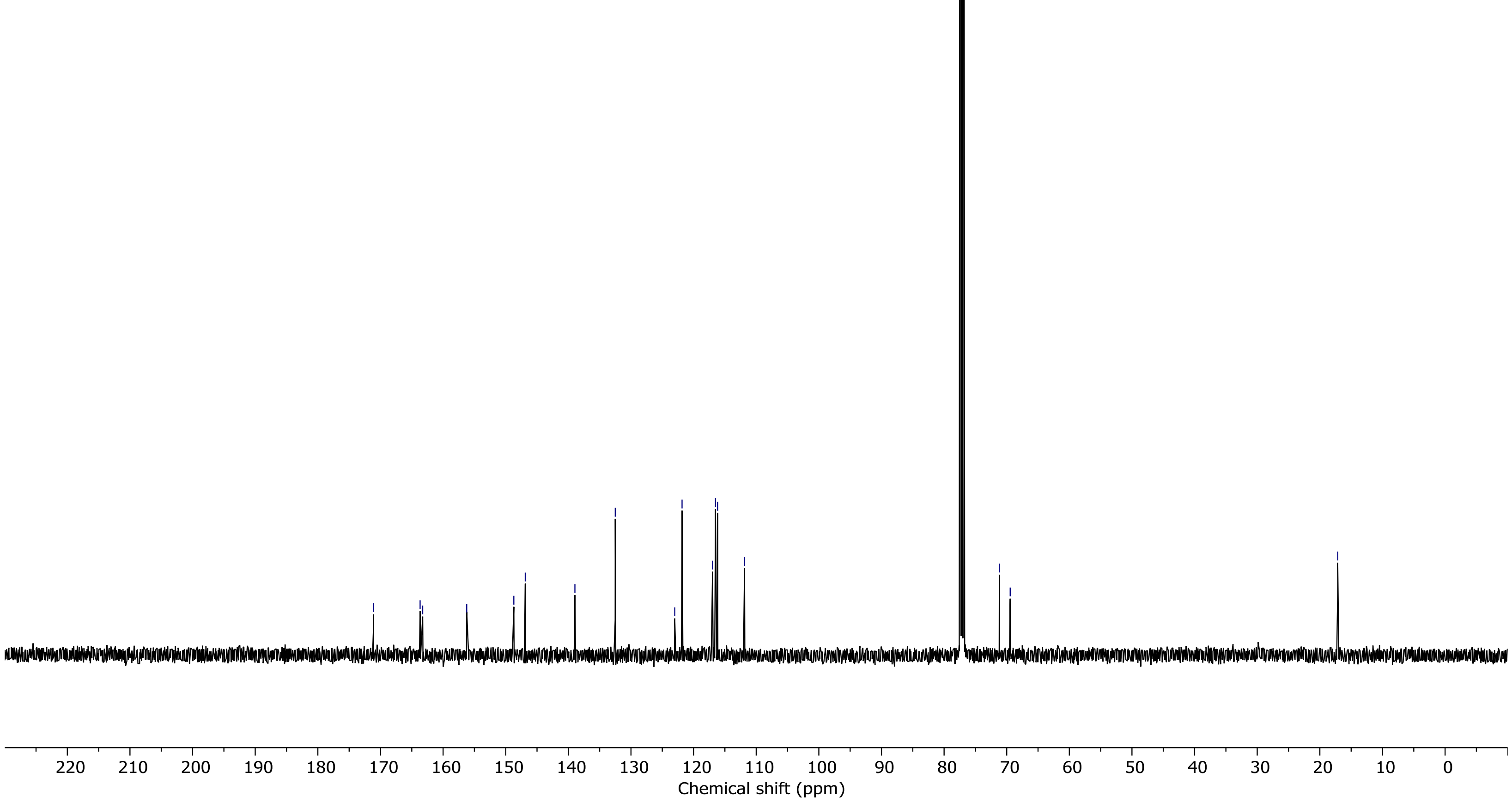

^{1}H NMR of 71

$CDCl_3$, 500 MHz, 23 ºC.

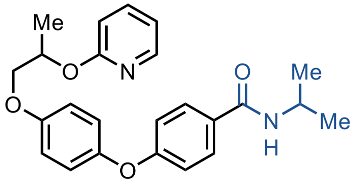

71

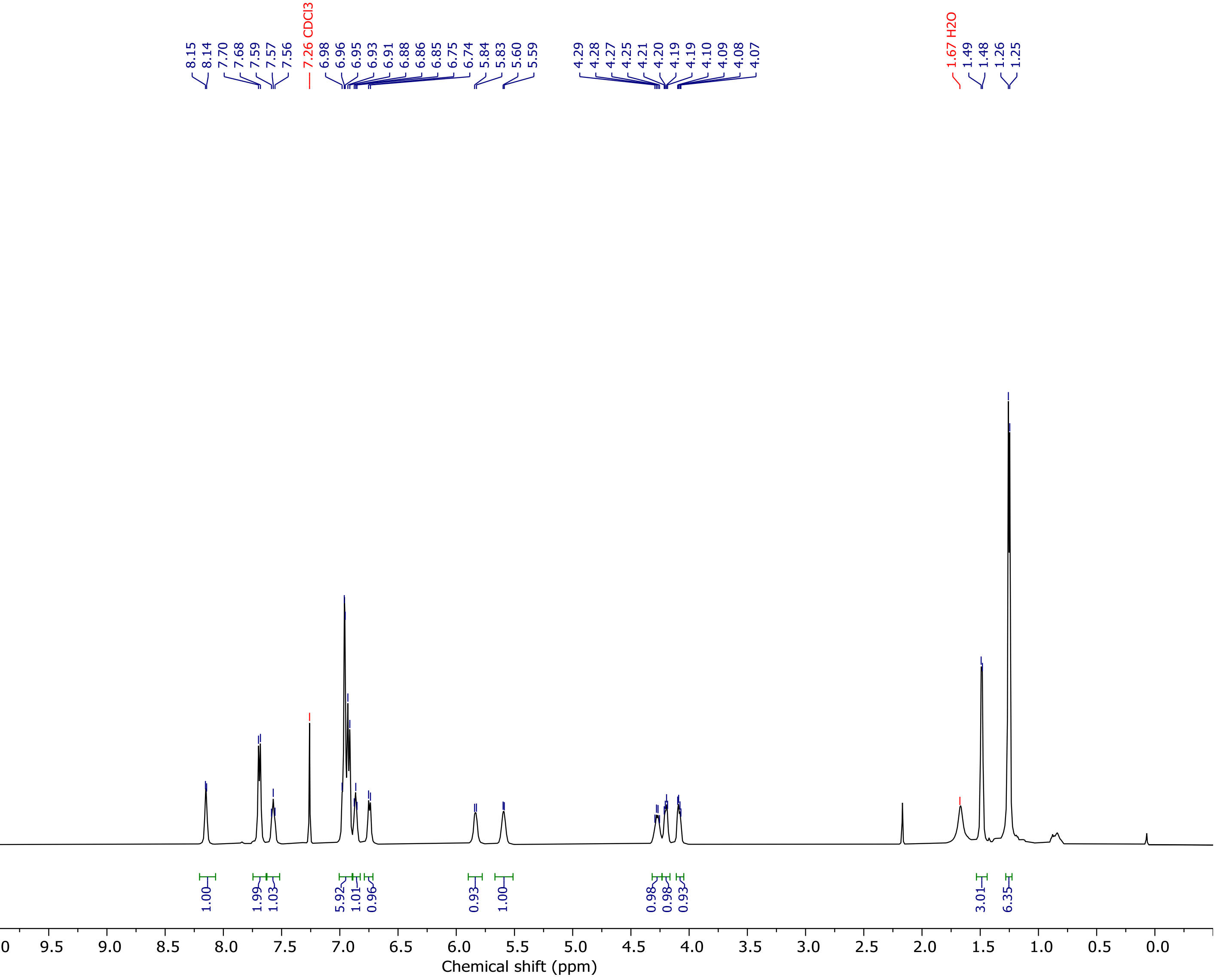

^{13}C NMR of 71

$CDCl_3$, 126 MHz, 23 ºC.

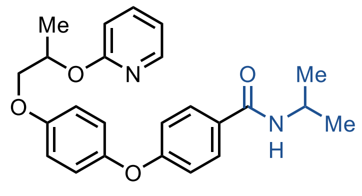

71

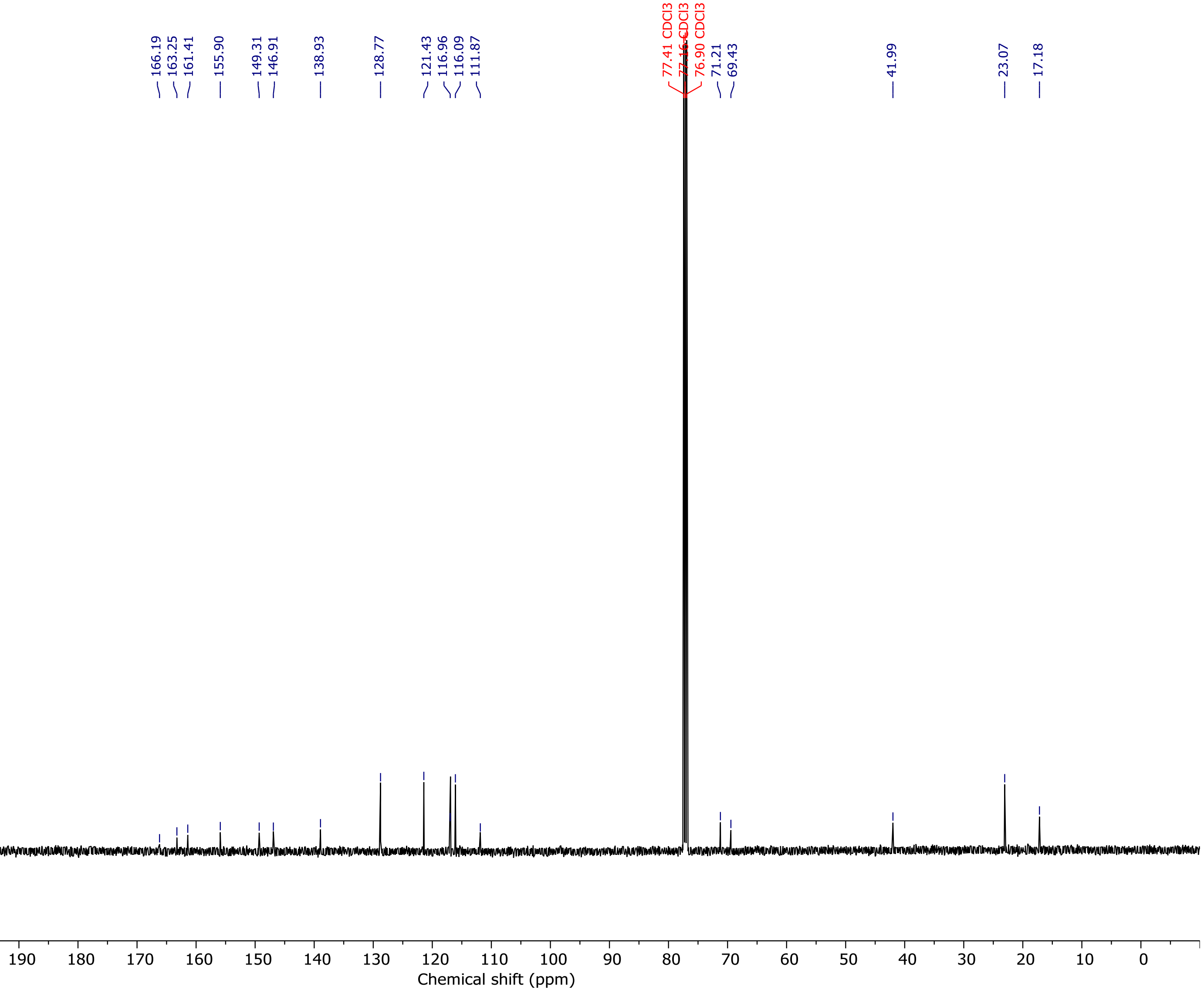

^{1}H NMR of 72

$CDCl_3$, 600 MHz, 23 ºC.

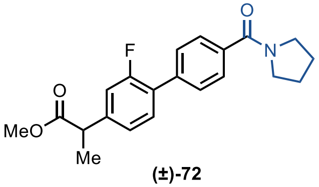

(±)-72

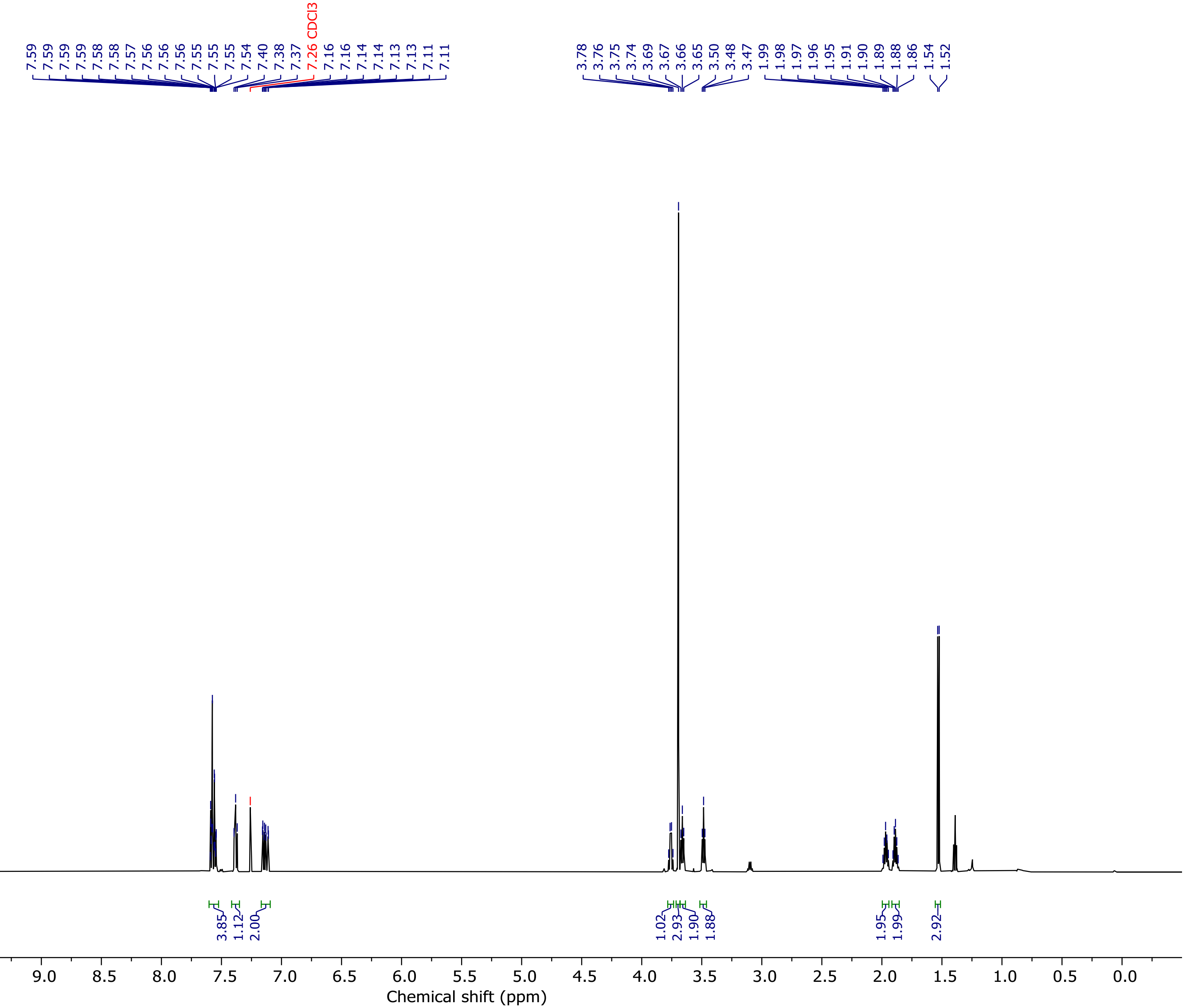

^{13}C NMR of 72

CDCl$_3$, 151 MHz, 23 ºC.

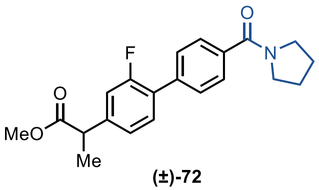

(±)-72

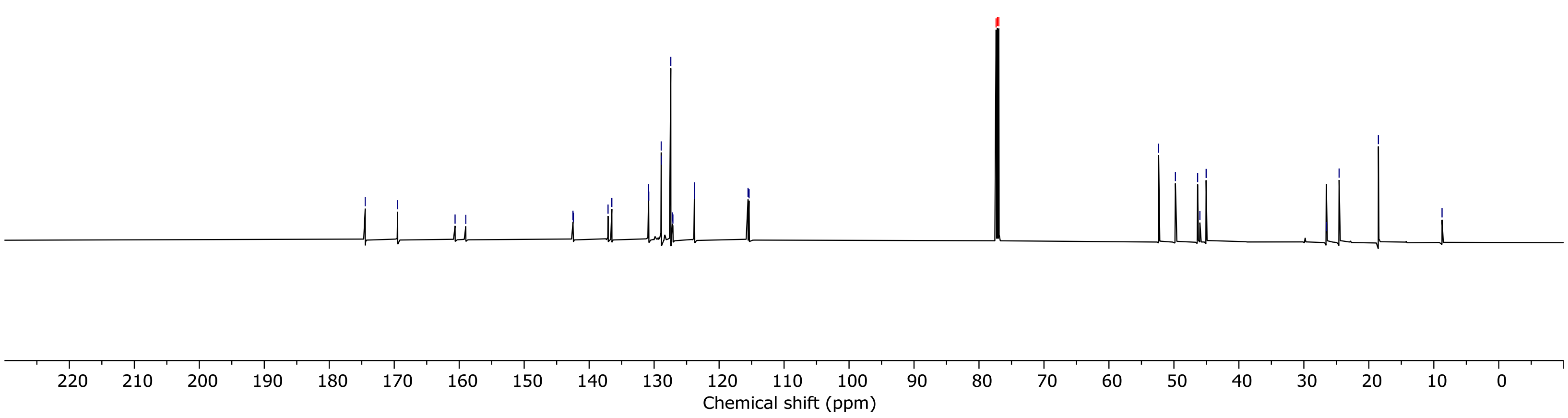

^{19}F NMR of 72

$CDCl_3$, 565 MHz, 23 ºC.

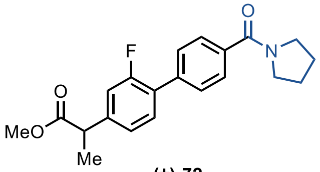

(±)-72

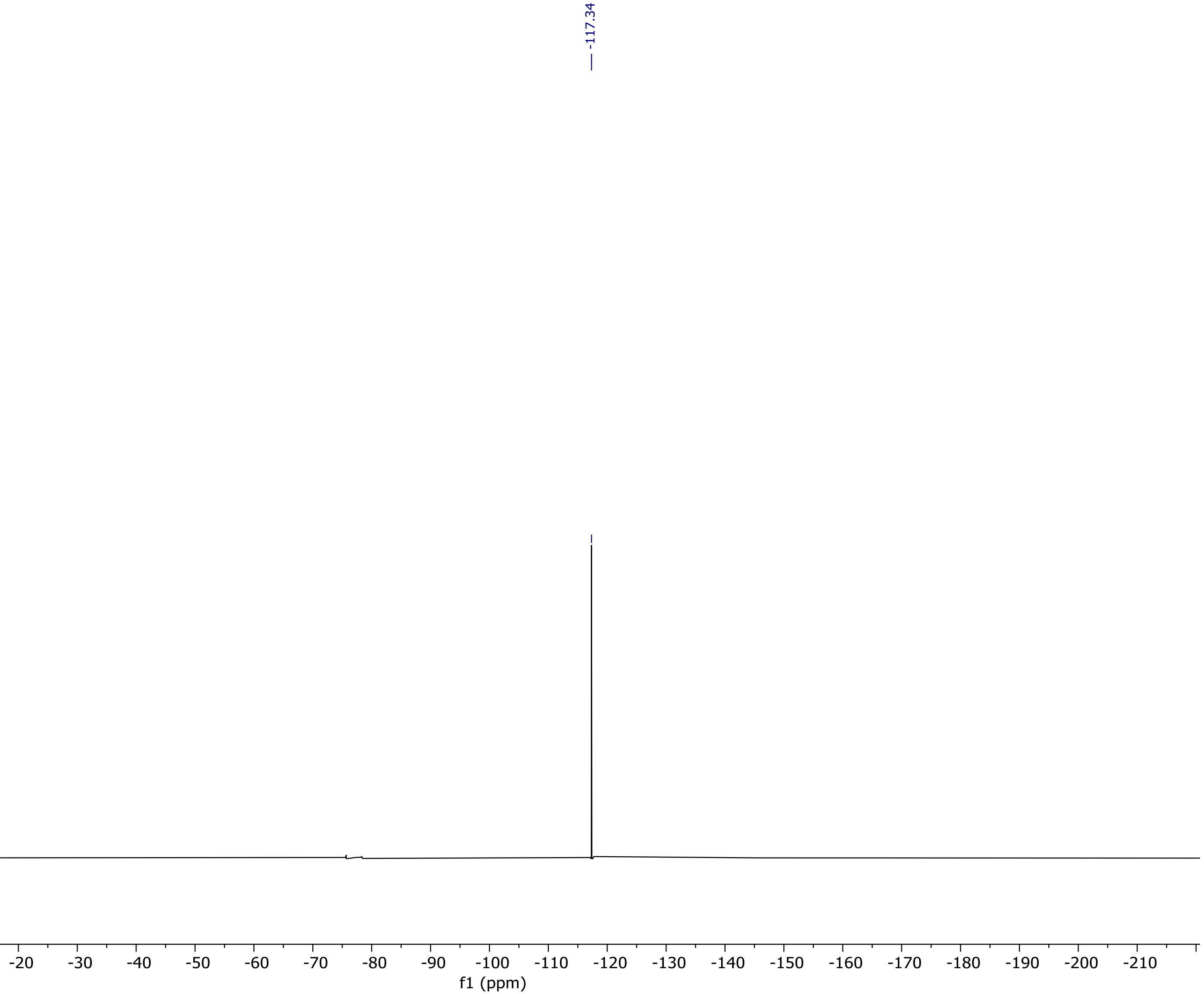

^{1}H NMR of 73

$CDCl_3$, 600 MHz, 23 ºC.

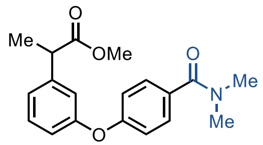

(±)-73

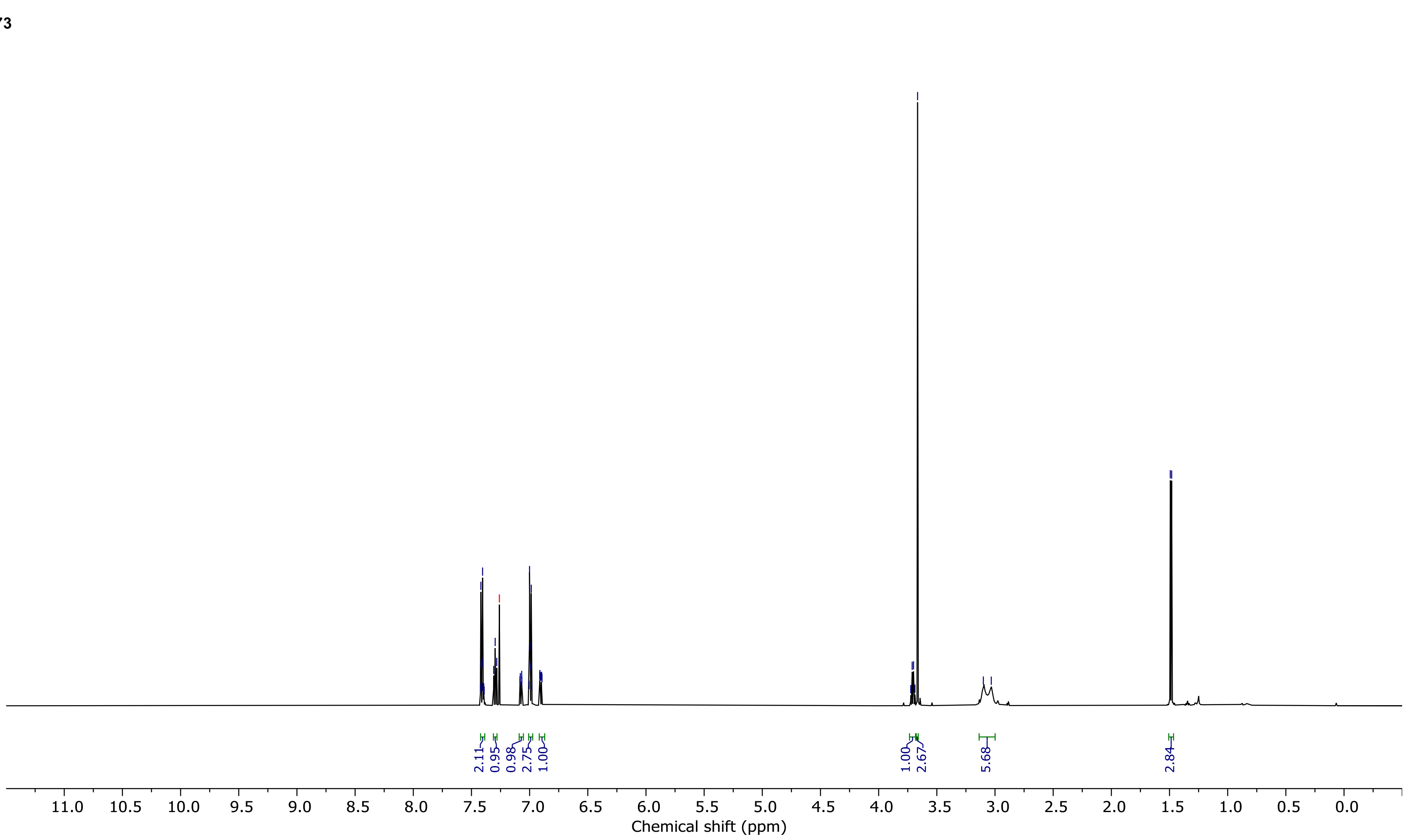

^{13}C NMR of 73

$CDCl_3$, 151 MHz, 23 ºC.

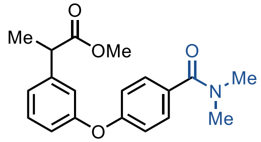

(±)-73

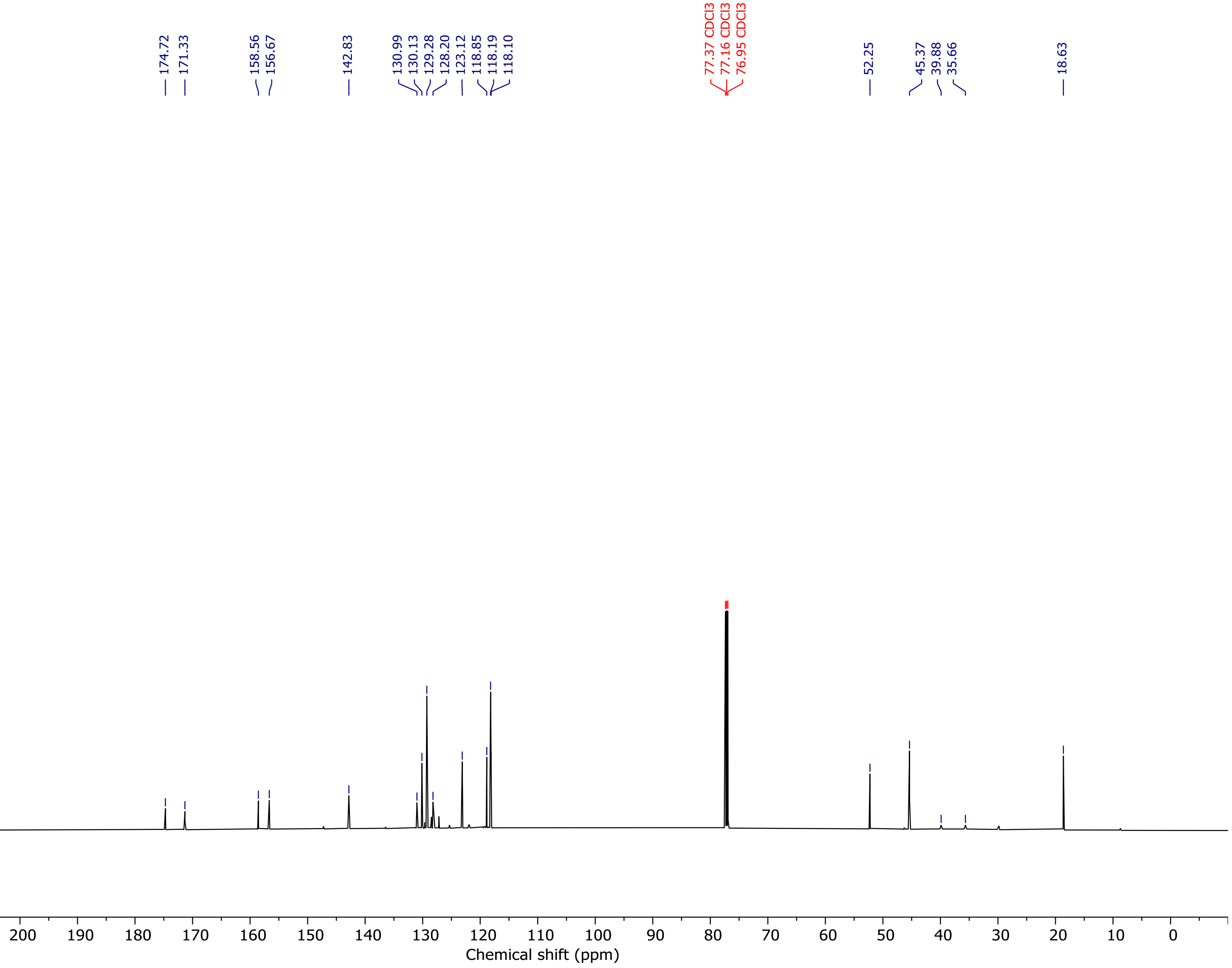

^{1}H NMR of 74

$CDCl_3$, 500 MHz, 23 ºC.

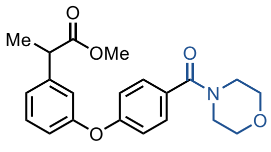

(±)-74

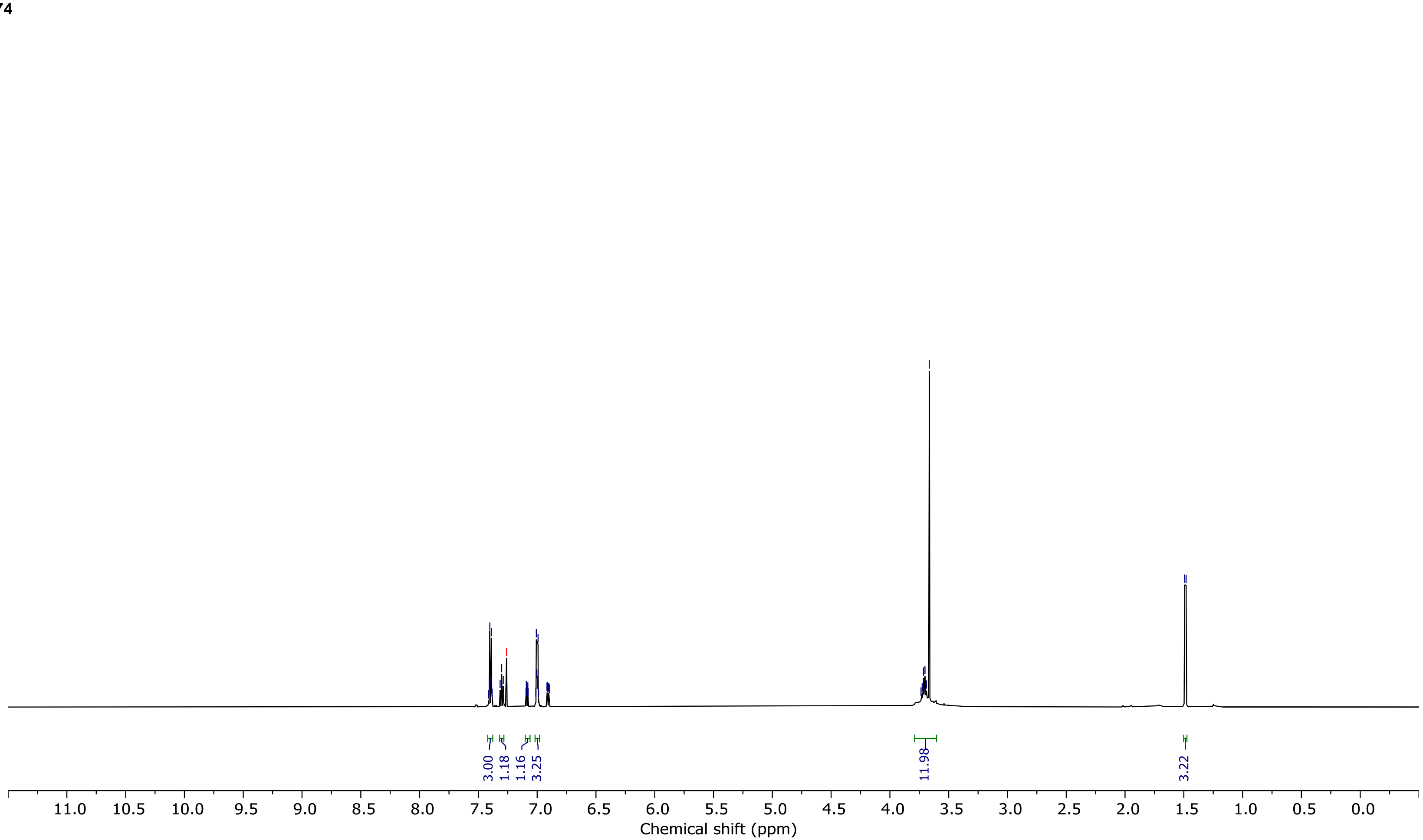

^{13}C NMR of 74

$CDCl_3$, 126 MHz, 23 ºC.

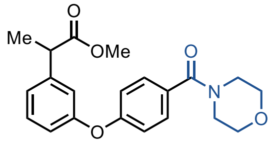

(±)-74

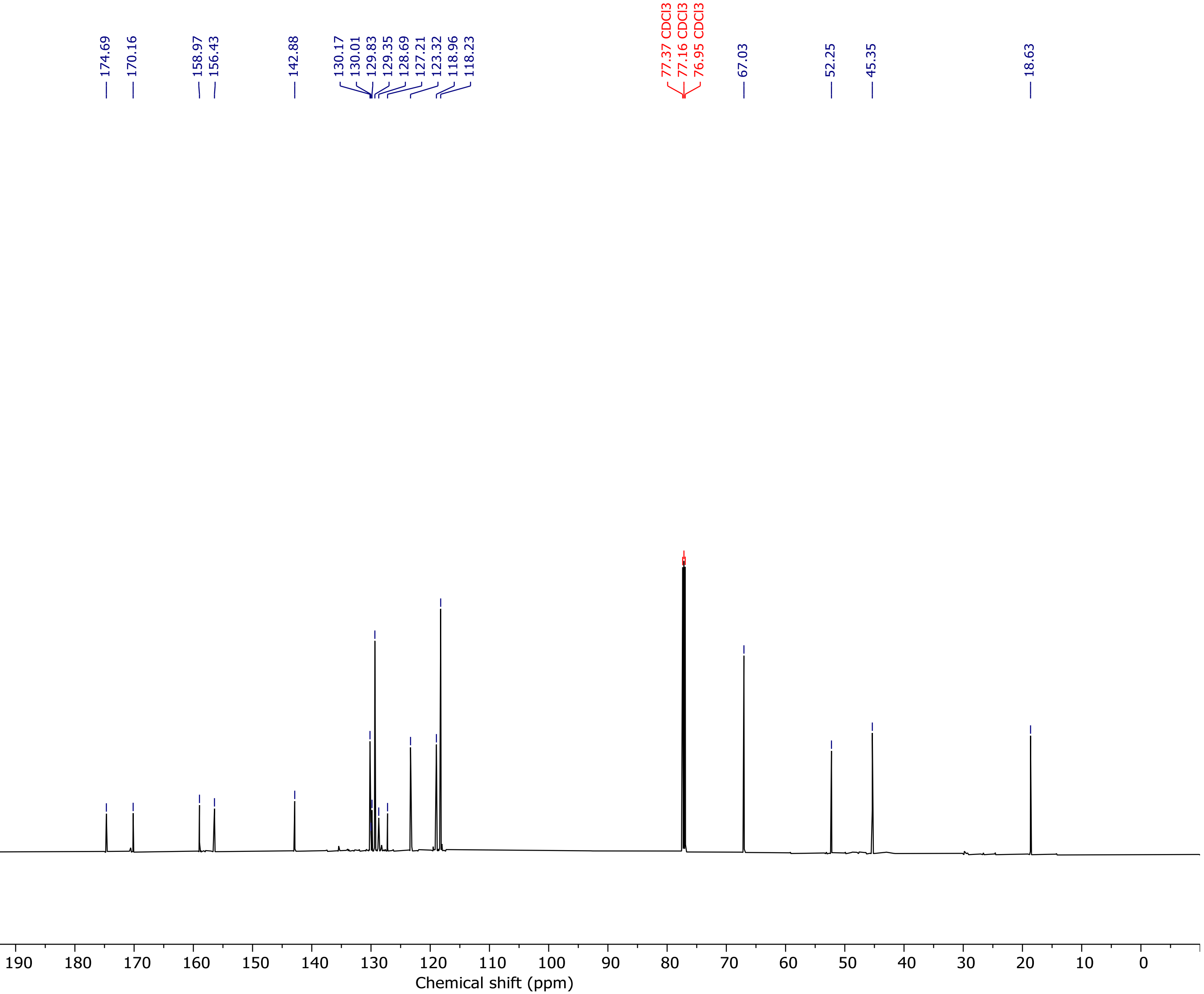

^{1}H NMR of 75

$CDCl_3$, 600 MHz, 23 ºC.

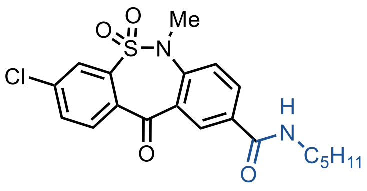

75

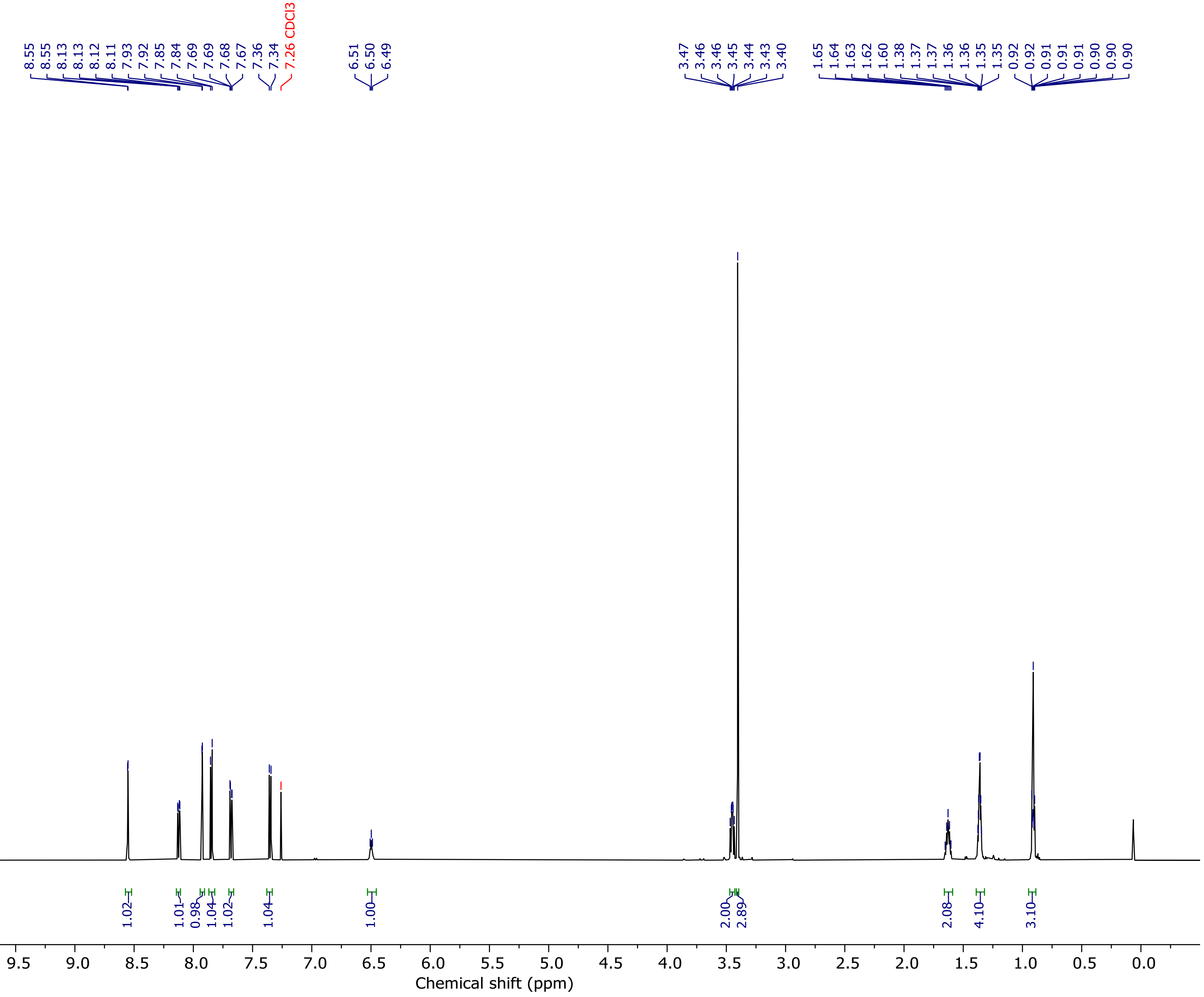

^{13}C NMR of 75

$CDCl_3$, 151 MHz, 23 ºC.

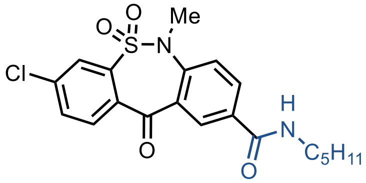

75

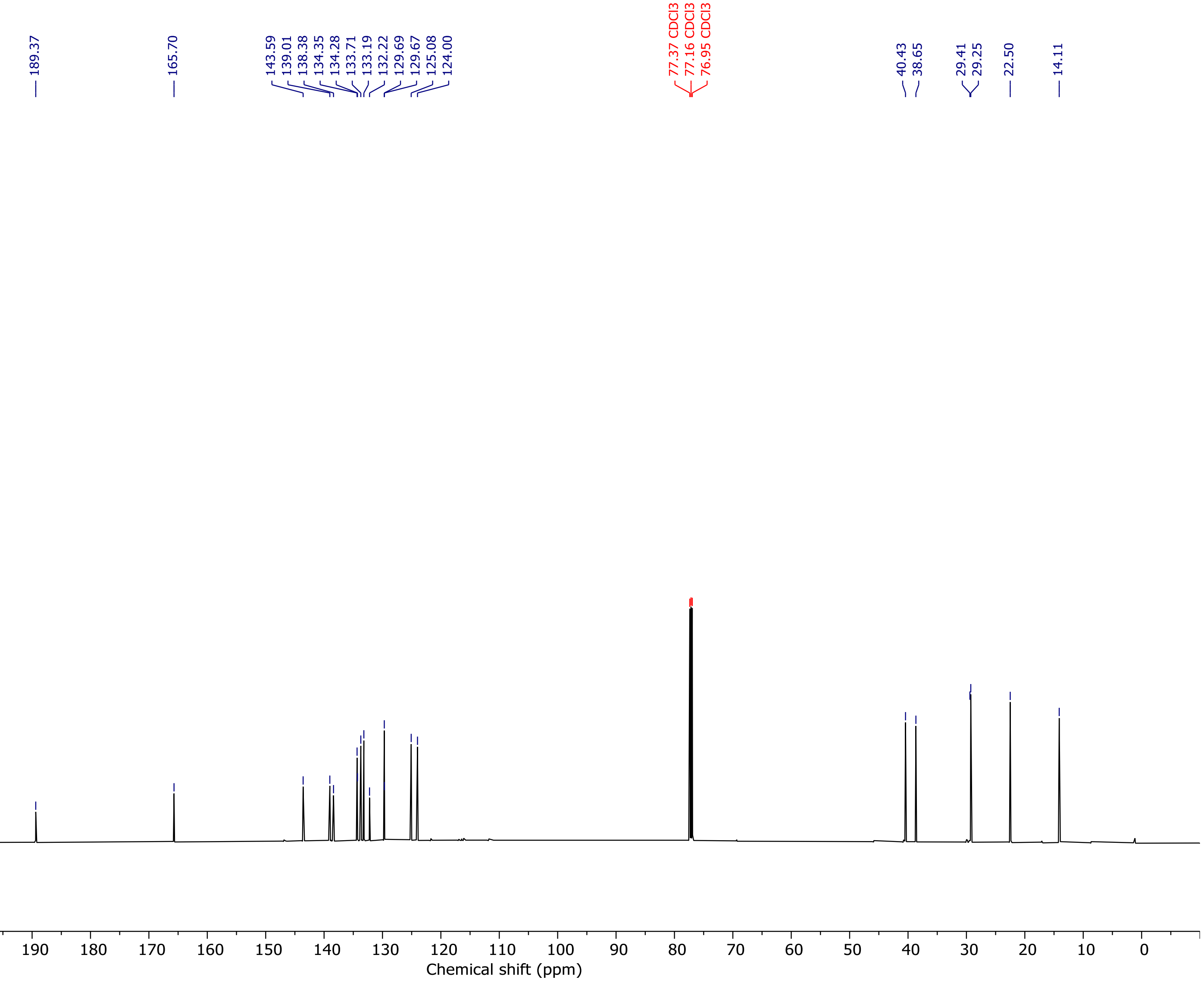

^{1}H NMR of 76

CDCl3, 500 MHz, 23 ºC.

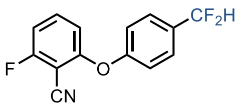

76

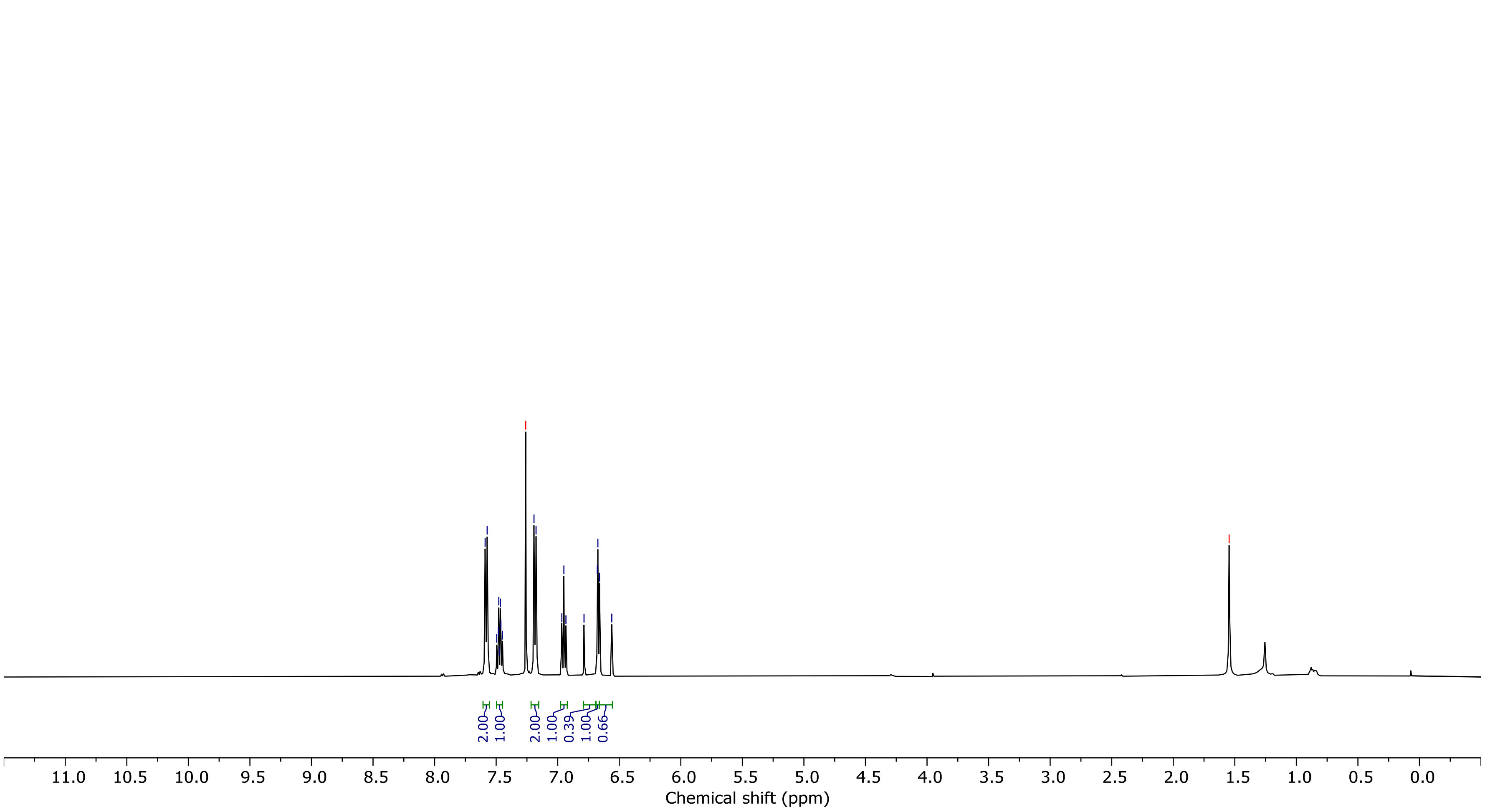

^{13}C NMR of 76

$CDCl_3$, 126 MHz, 23 ºC.

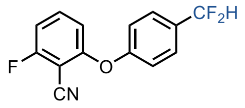

76

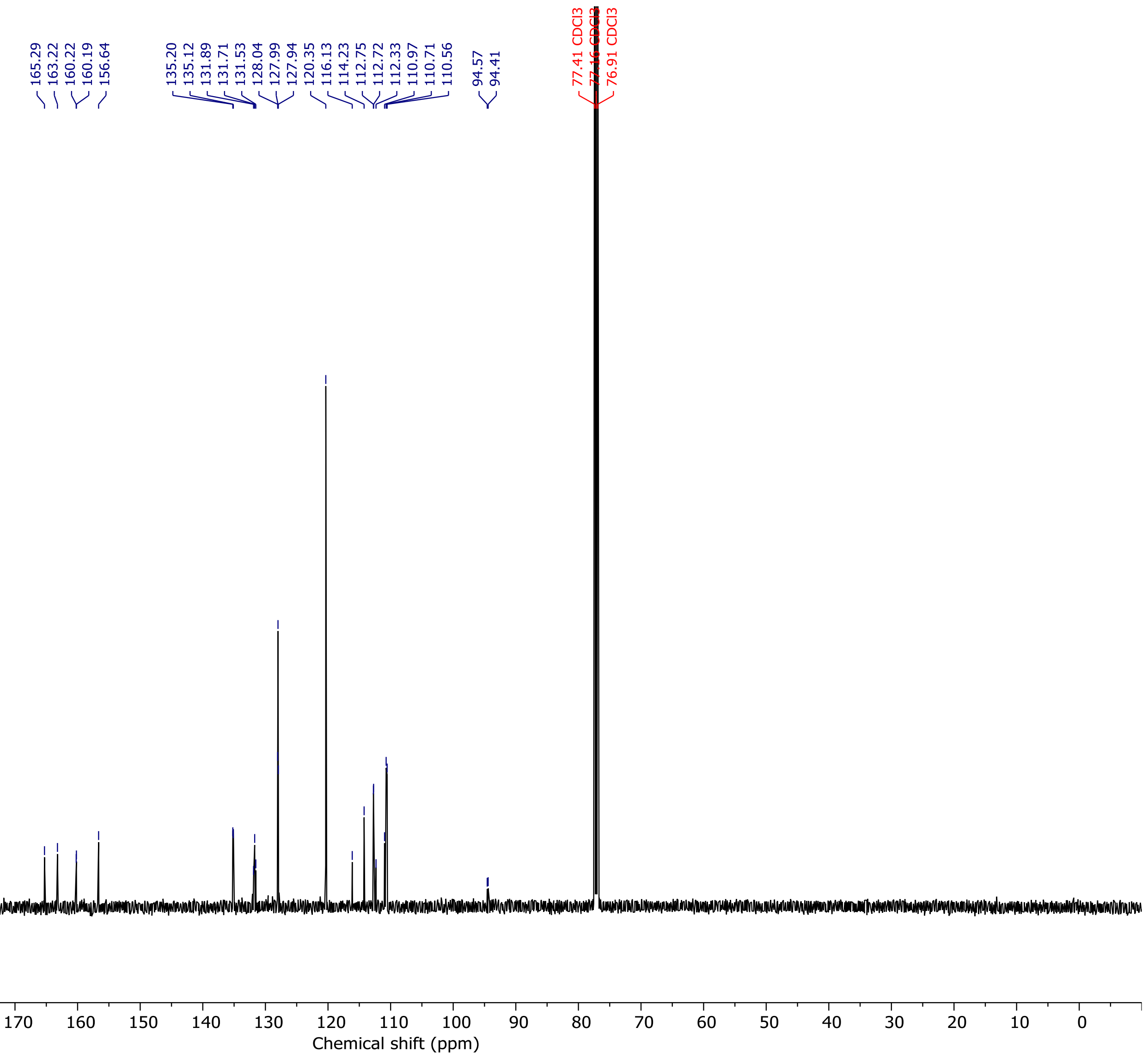

^{19}F NMR of 76

$CDCl_3$, 471 MHz, 23 ºC.

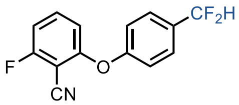

76

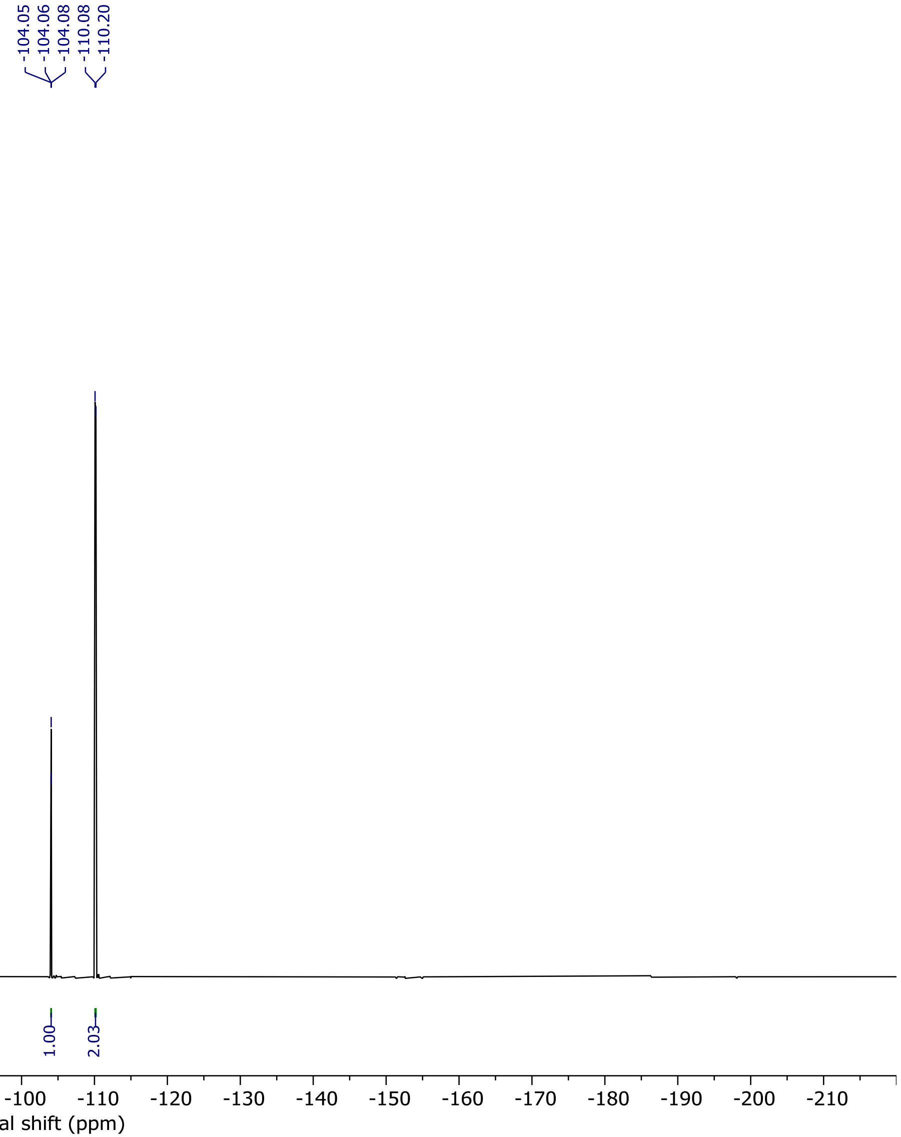

^{1}H NMR of 77

$CDCl_3$, 500 MHz, 23 ºC.

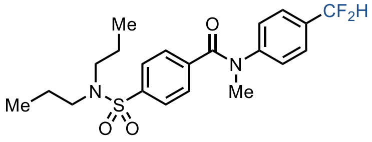

77

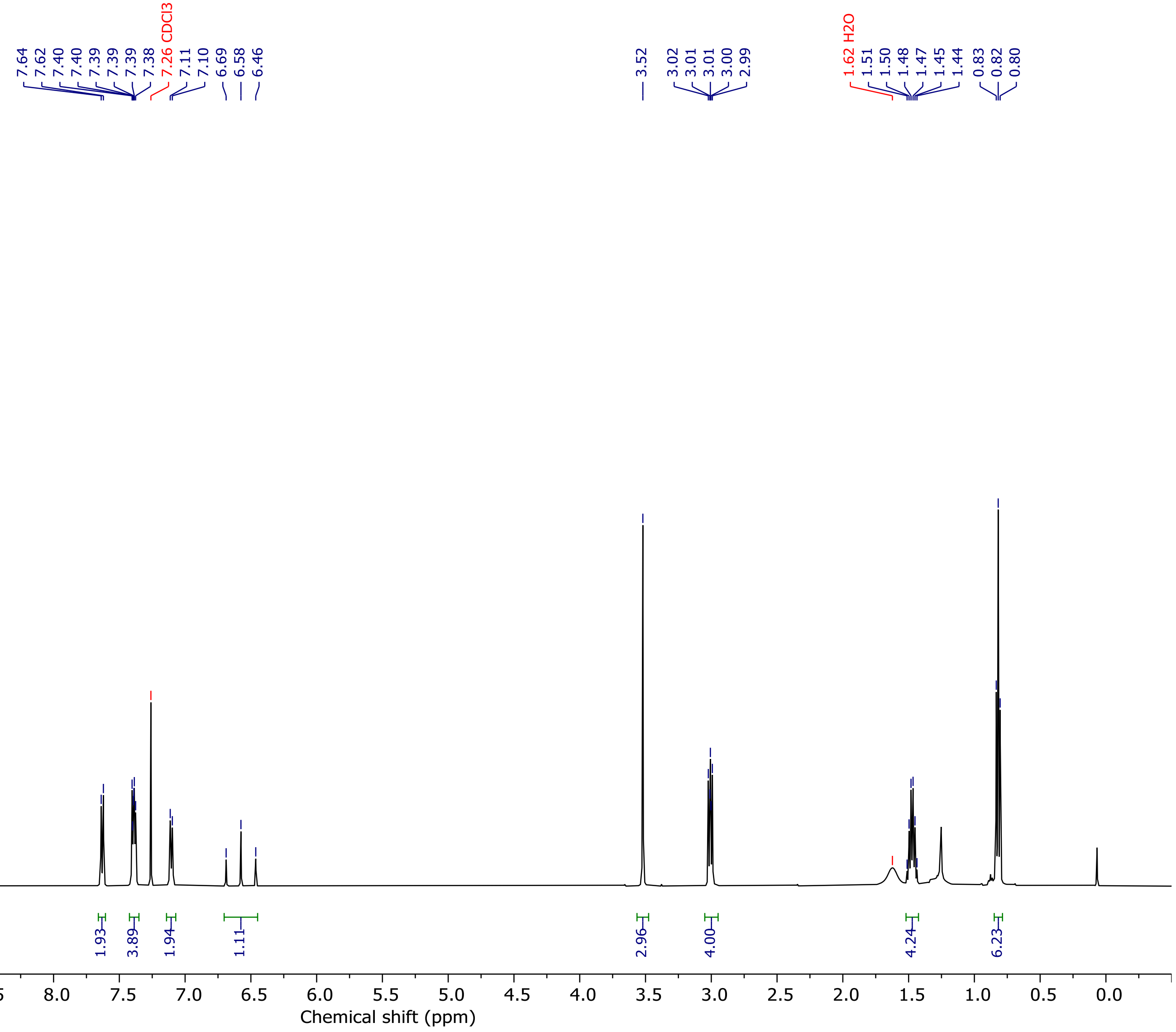

^{13}C NMR of 77

$CDCl_3$, 151 MHz, 23 ºC.

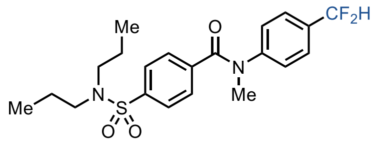

77

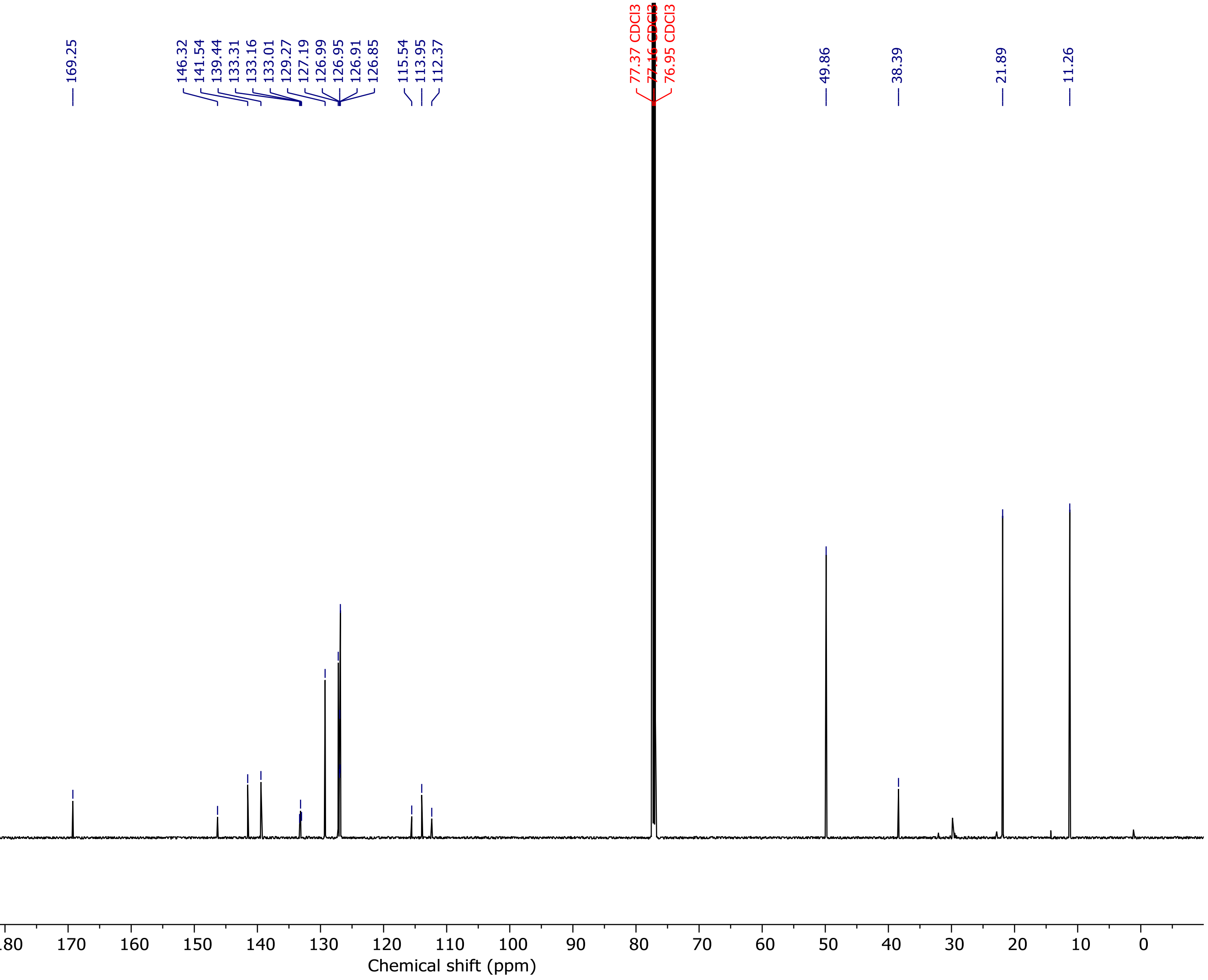

^{19}F NMR of 77

$CDCl_3$, 565 MHz, 23 ºC.

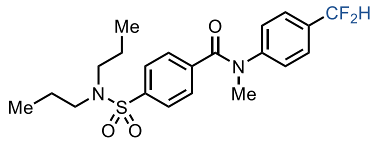

77

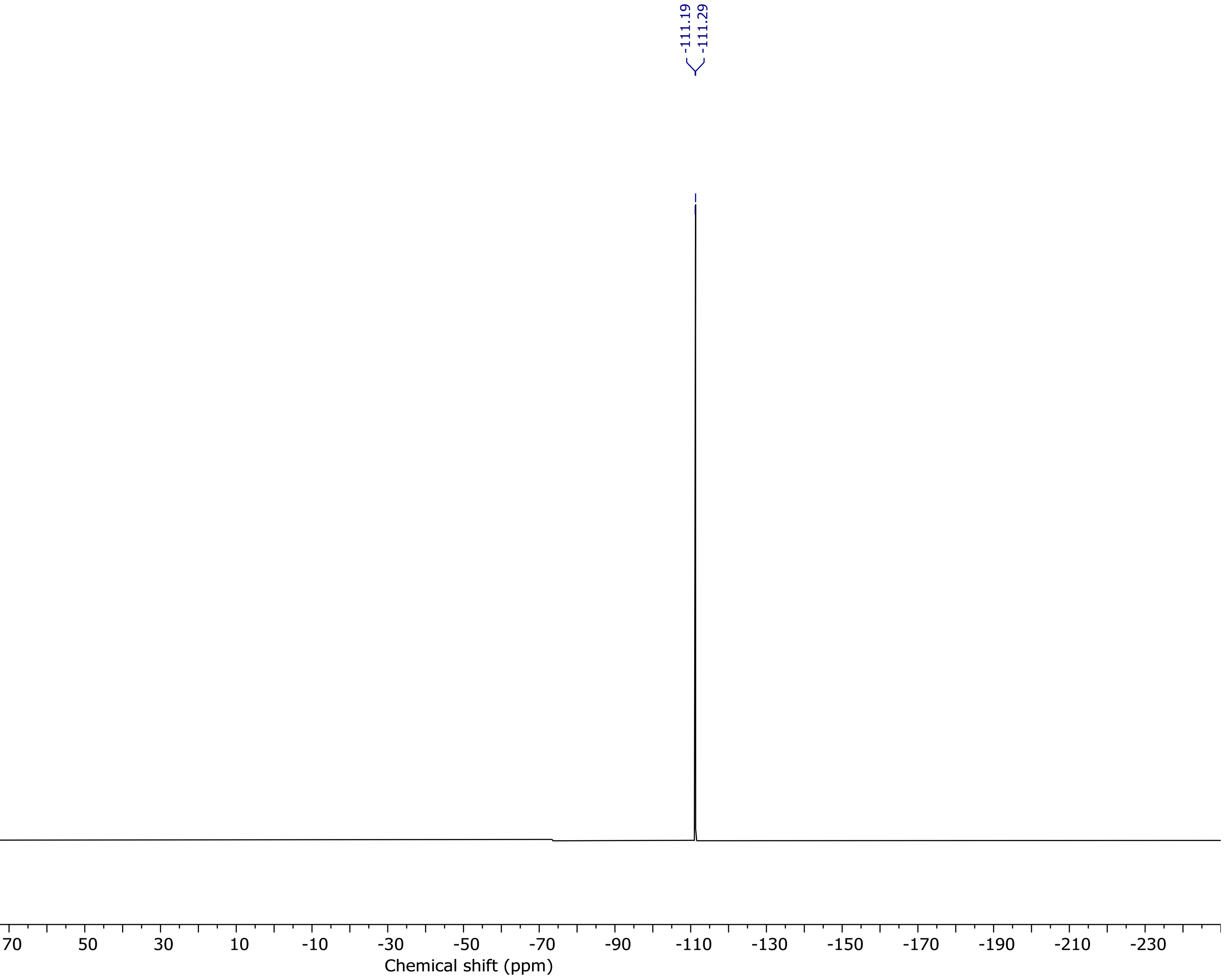

^{1}H NMR of 78

$CDCl_3$, 500 MHz, 23 ºC.

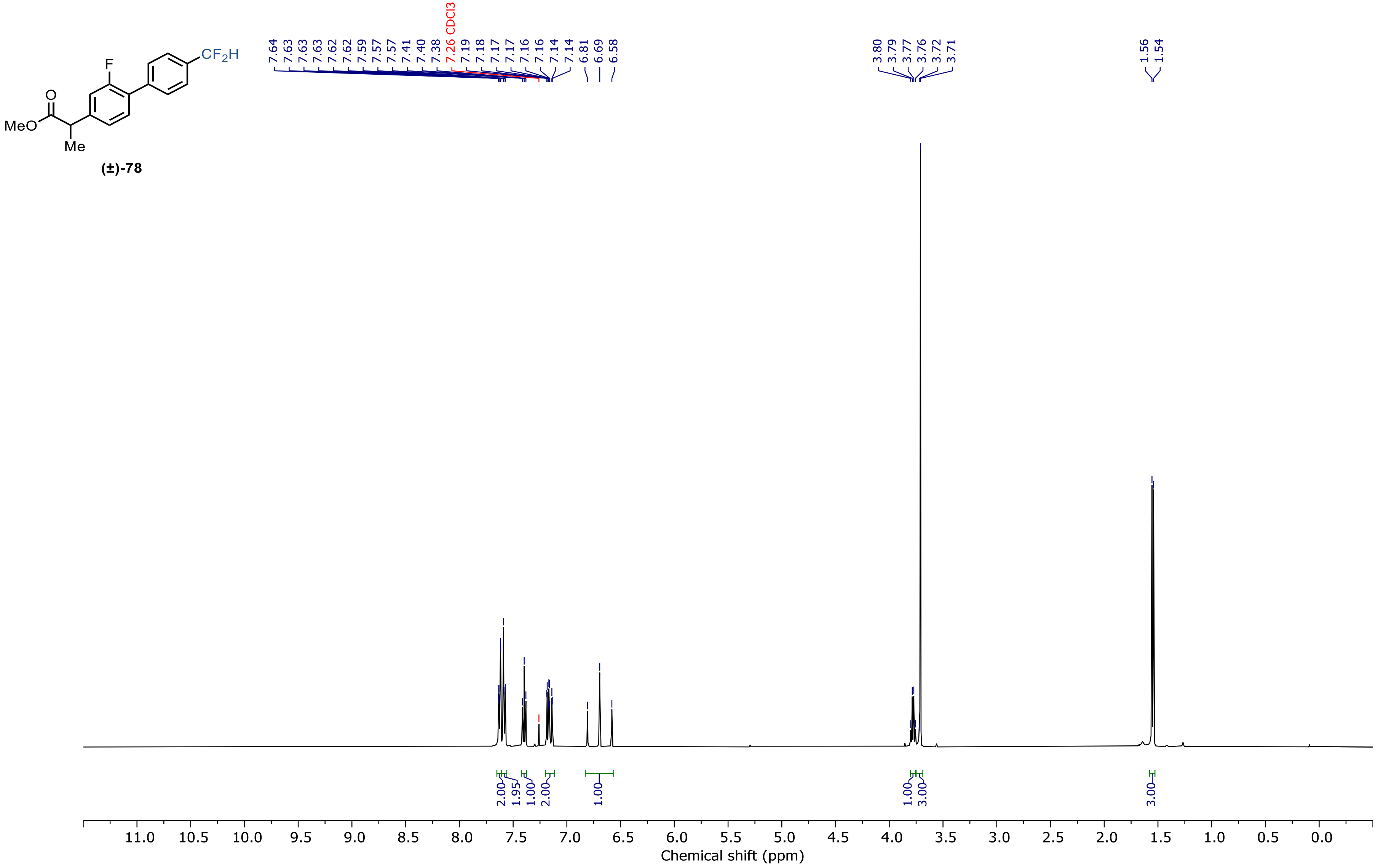

(±)-78

^{13}C NMR of 78

$CDCl_3$, 126 MHz, 23 ºC.

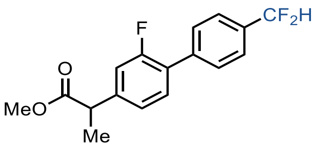

(±)-78

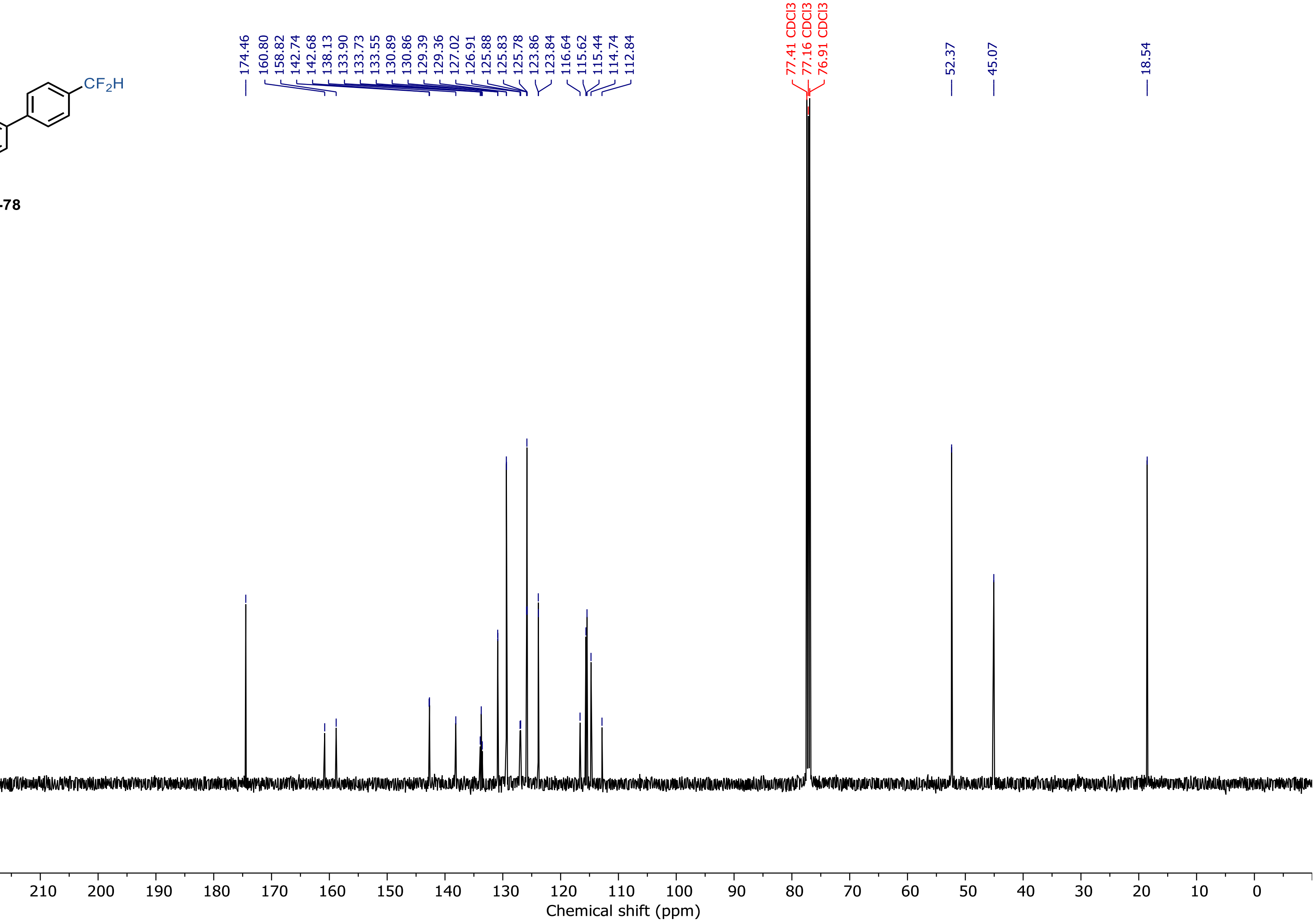

^{19}F NMR of 78

CDCl3, 471 MHz, 23 ºC.

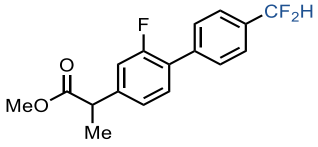

(±)-78

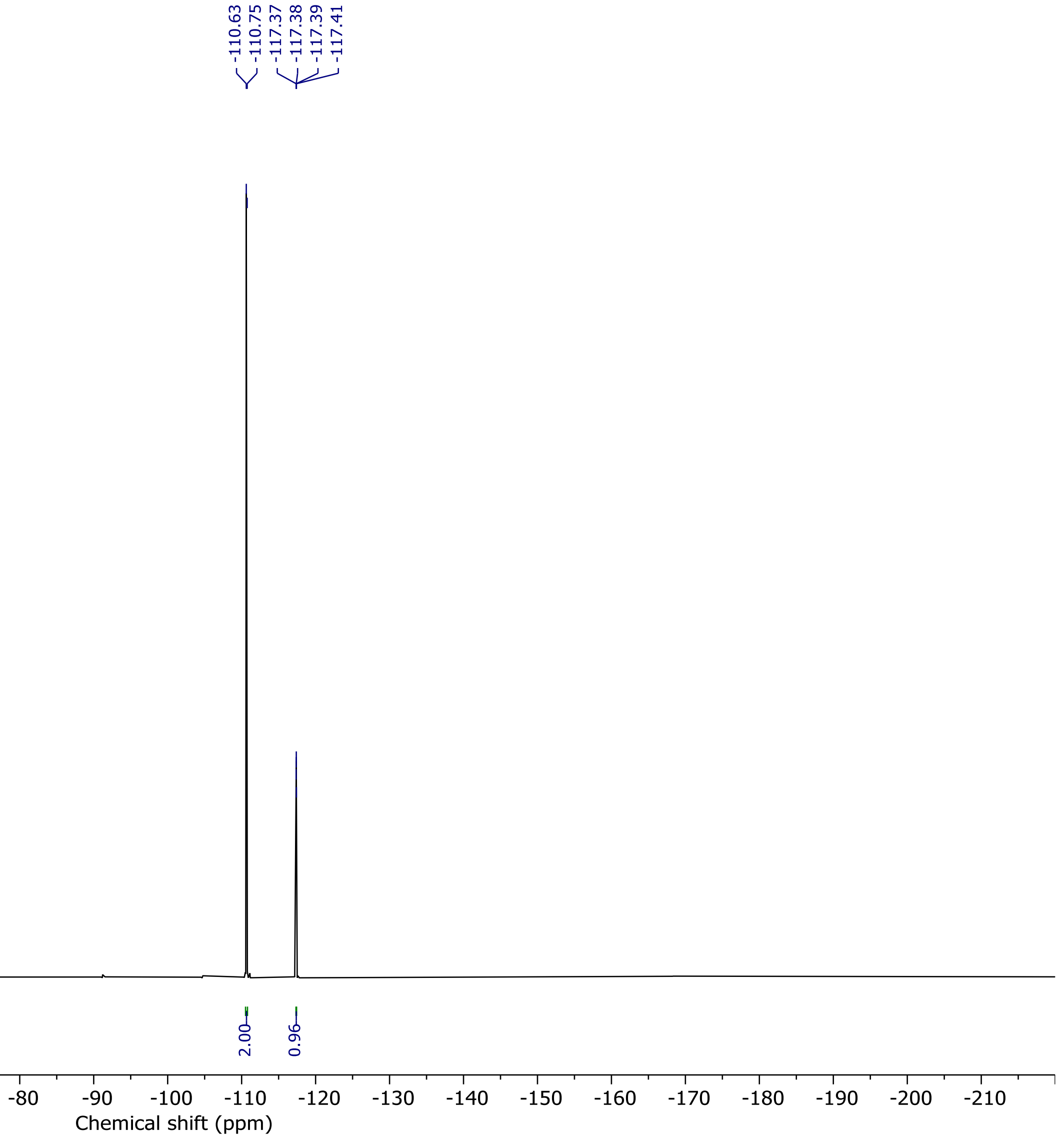

^{1}H NMR of 79

CDCl3, 500 MHz, 23 ºC.

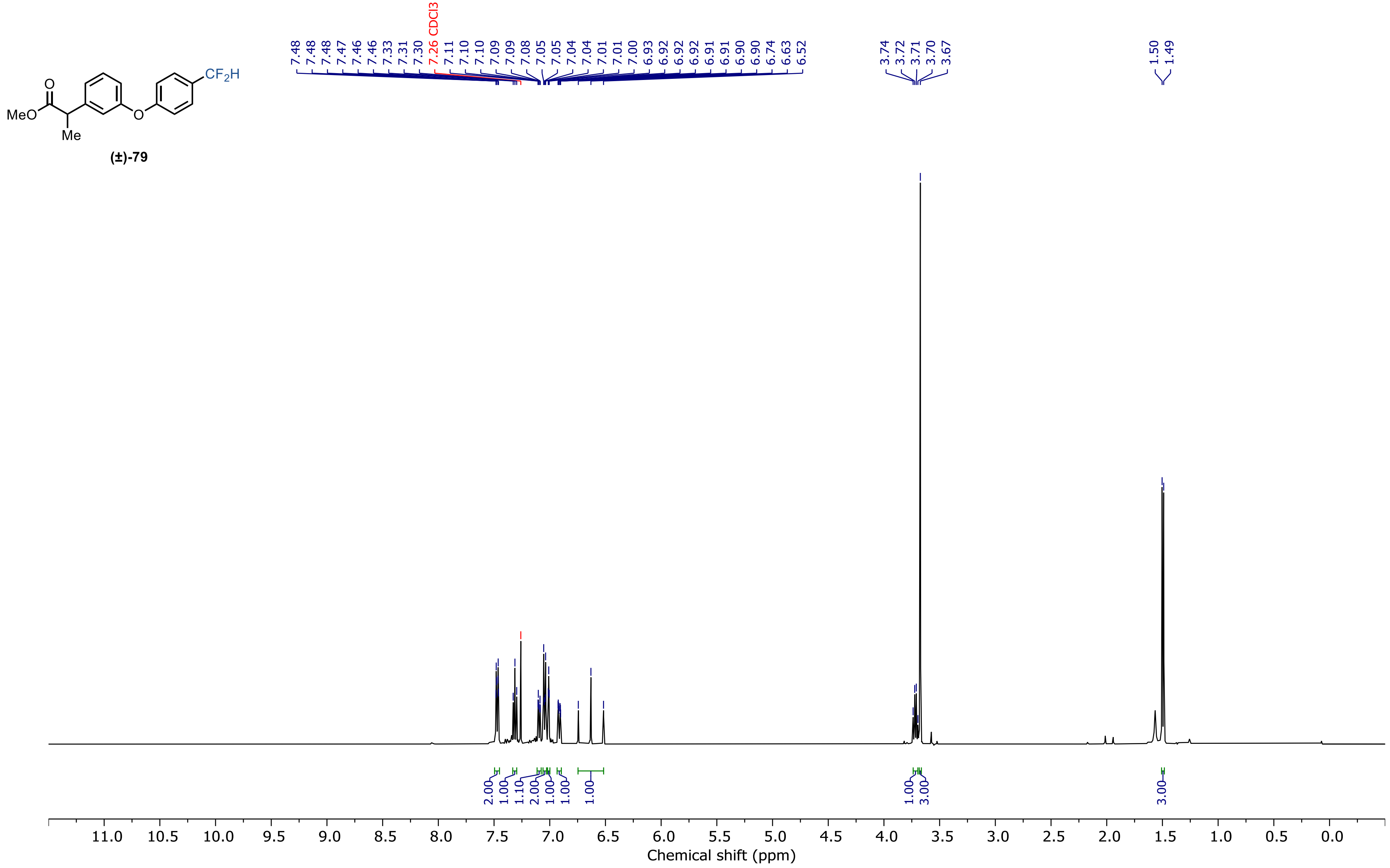

^{13}C NMR of 79

$CDCl_3$, 126 MHz, 23 ºC.

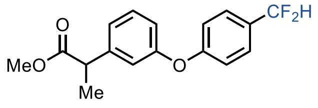

(±)-79

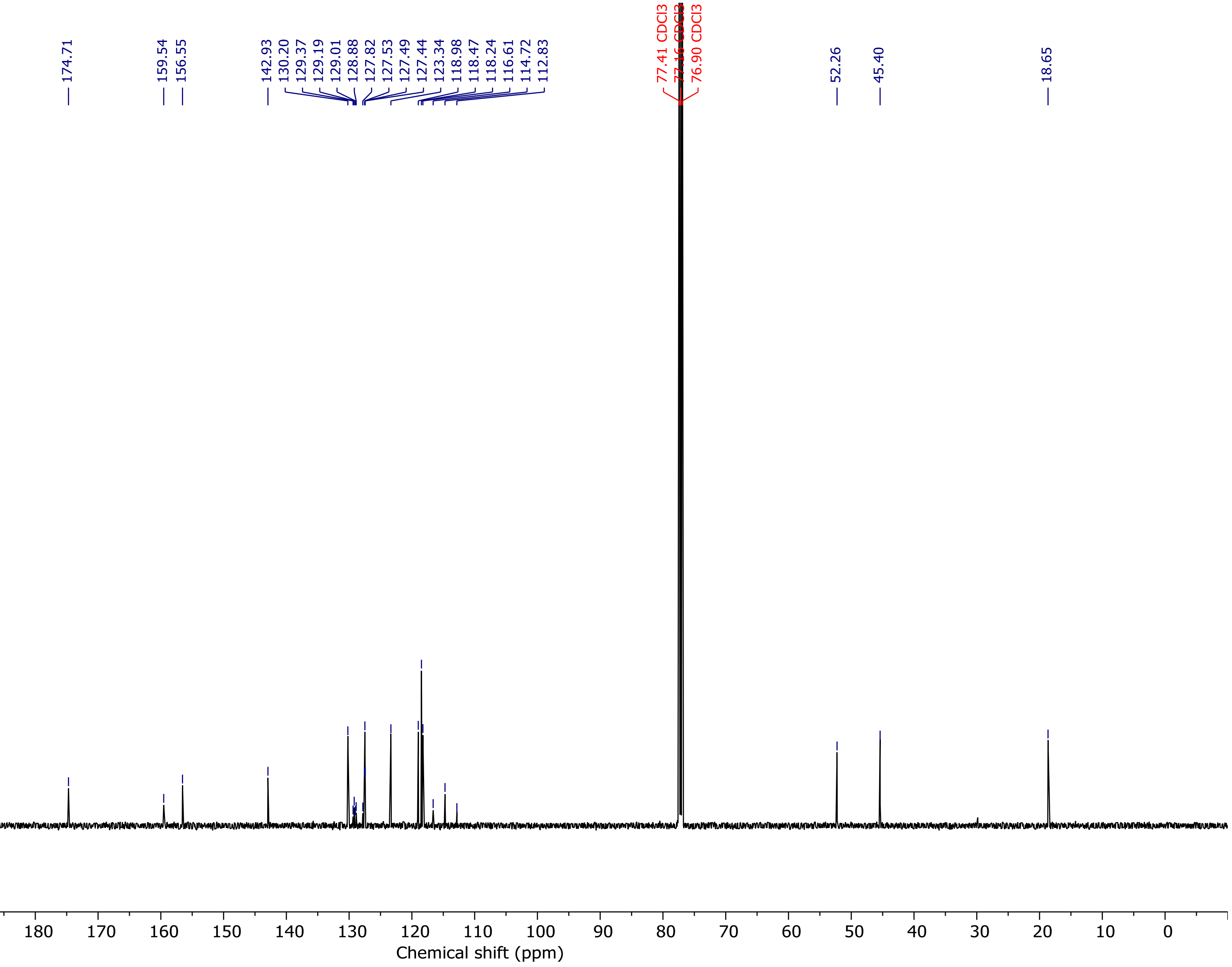

^{19}F NMR of 79

$CDCl_3$, 471

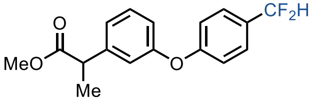

(±)-79

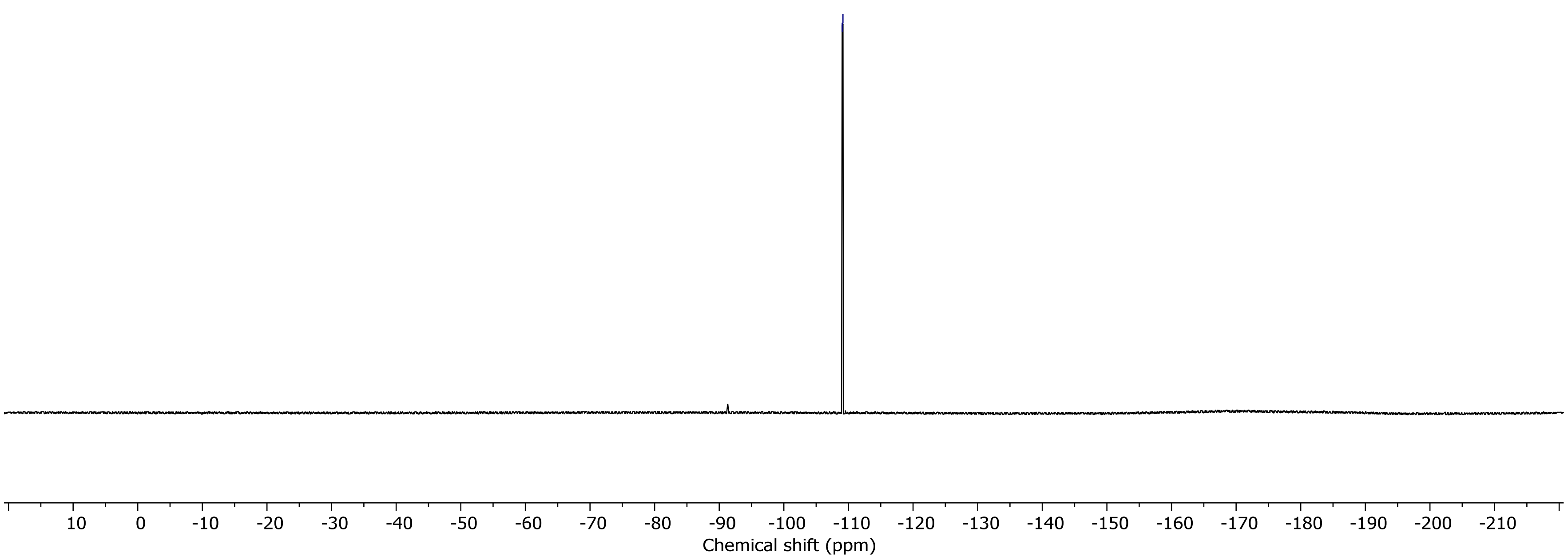

^{1}H NMR of 80

CDCl$_3$, 500 MHz, 23 ºC.

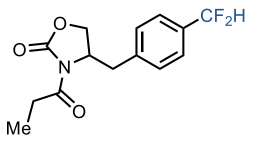

(±)-80

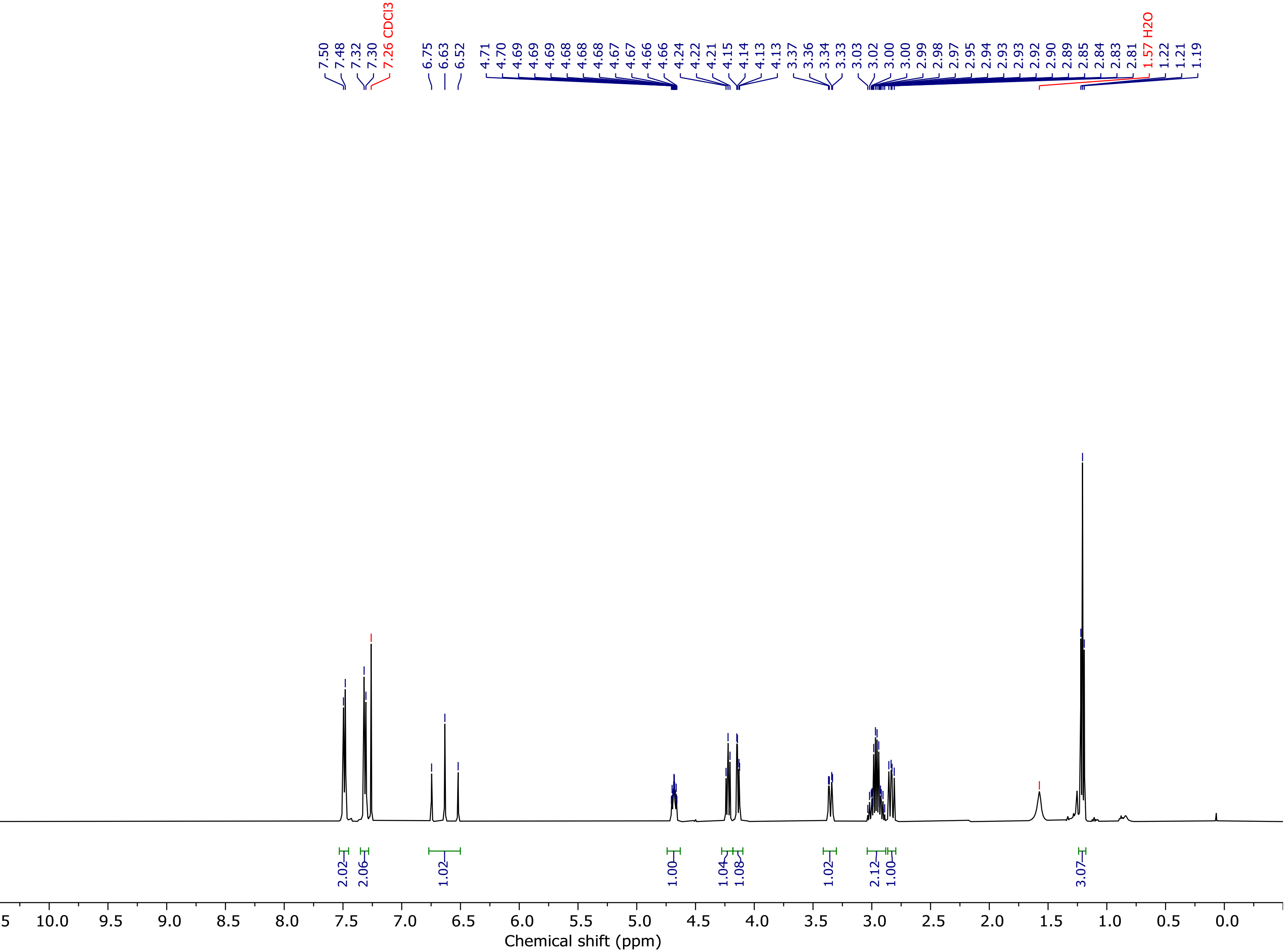

^{13}C NMR of 80

$CDCl_3$, 126 MHz, 23 ºC.

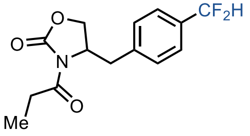

(±)-80

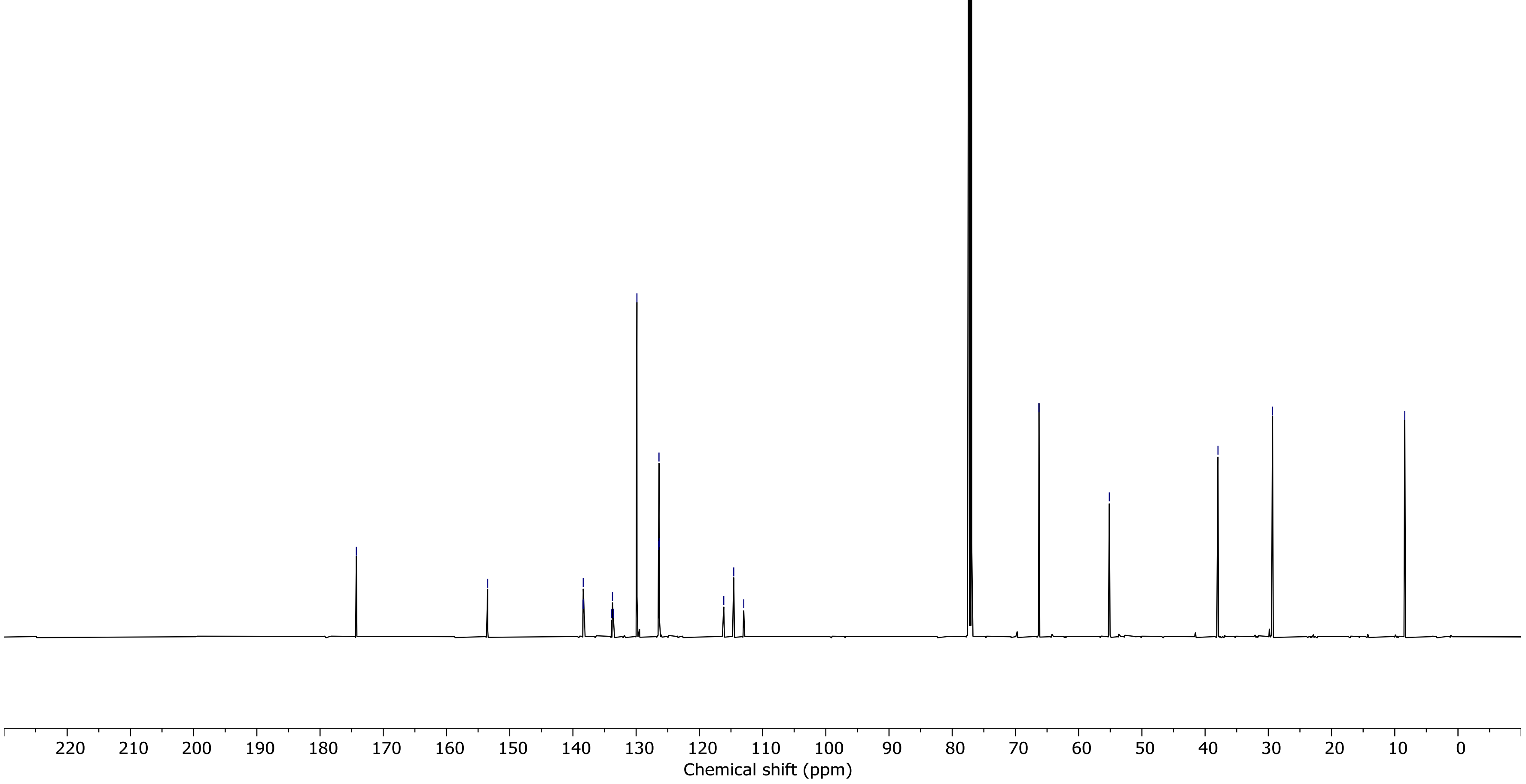

^{19}F NMR of 80

$CDCl_3$, 471 MHz, 23 ºC.

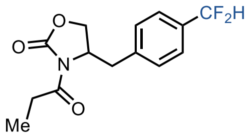

(±)-80

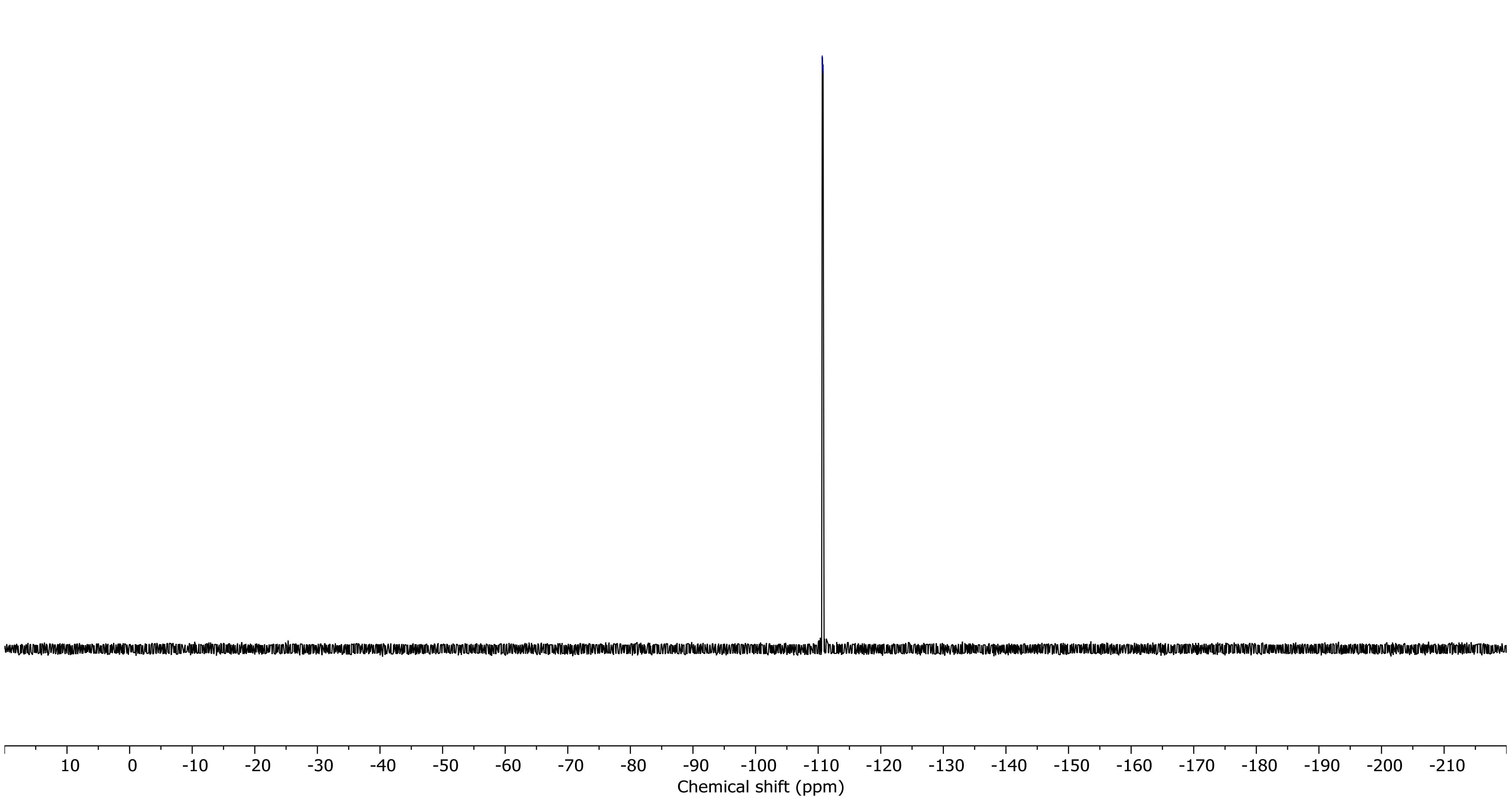

^{1}H NMR of 81

CDCl$_3$, 600 MHz, 23 ºC.

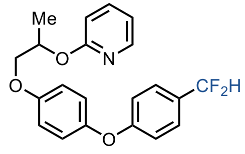

(±)-81

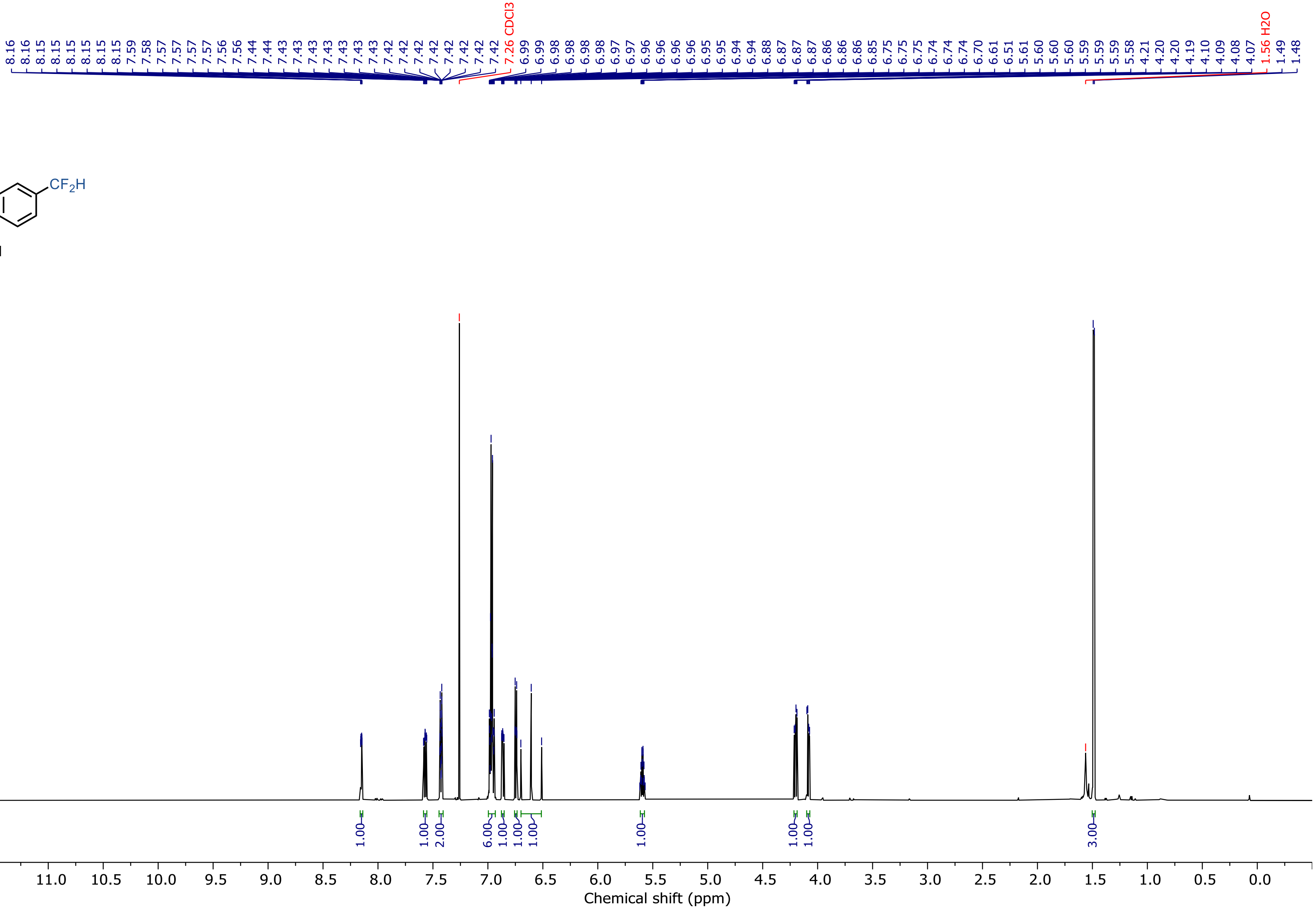

^{13}C NMR of 81

$CDCl_3$, 151 MHz, 23 ºC.

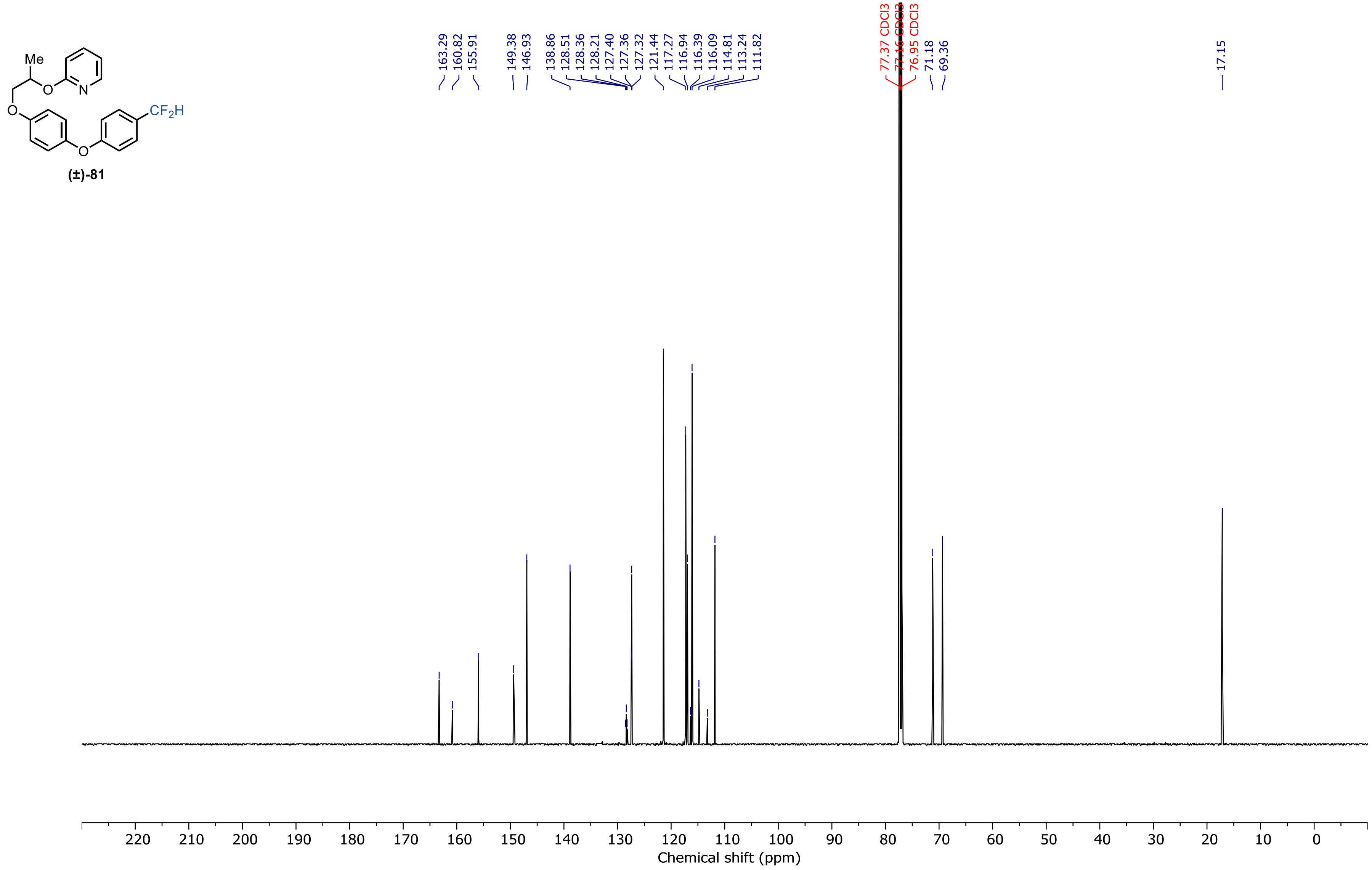

(±)-81

^{19}F NMR of 81

$CDCl_3$, 471 MHz, 23 ºC.

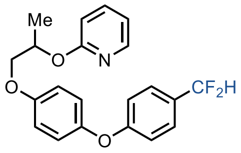

(±)-81

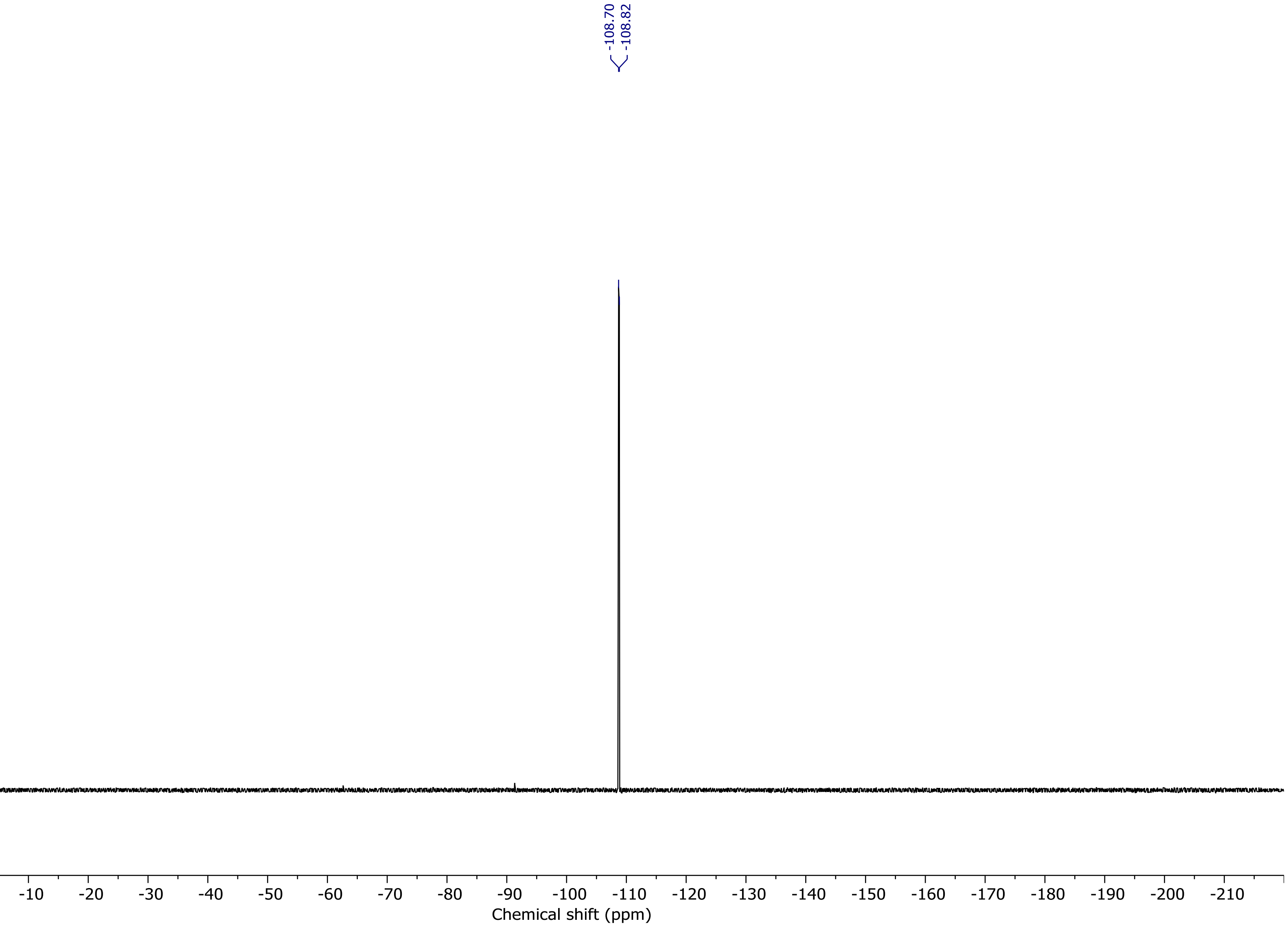

^{1}H NMR of 82

$CDCl_3$, 500 MHz, 23 ºC.

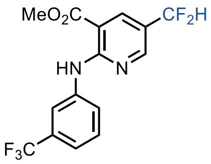

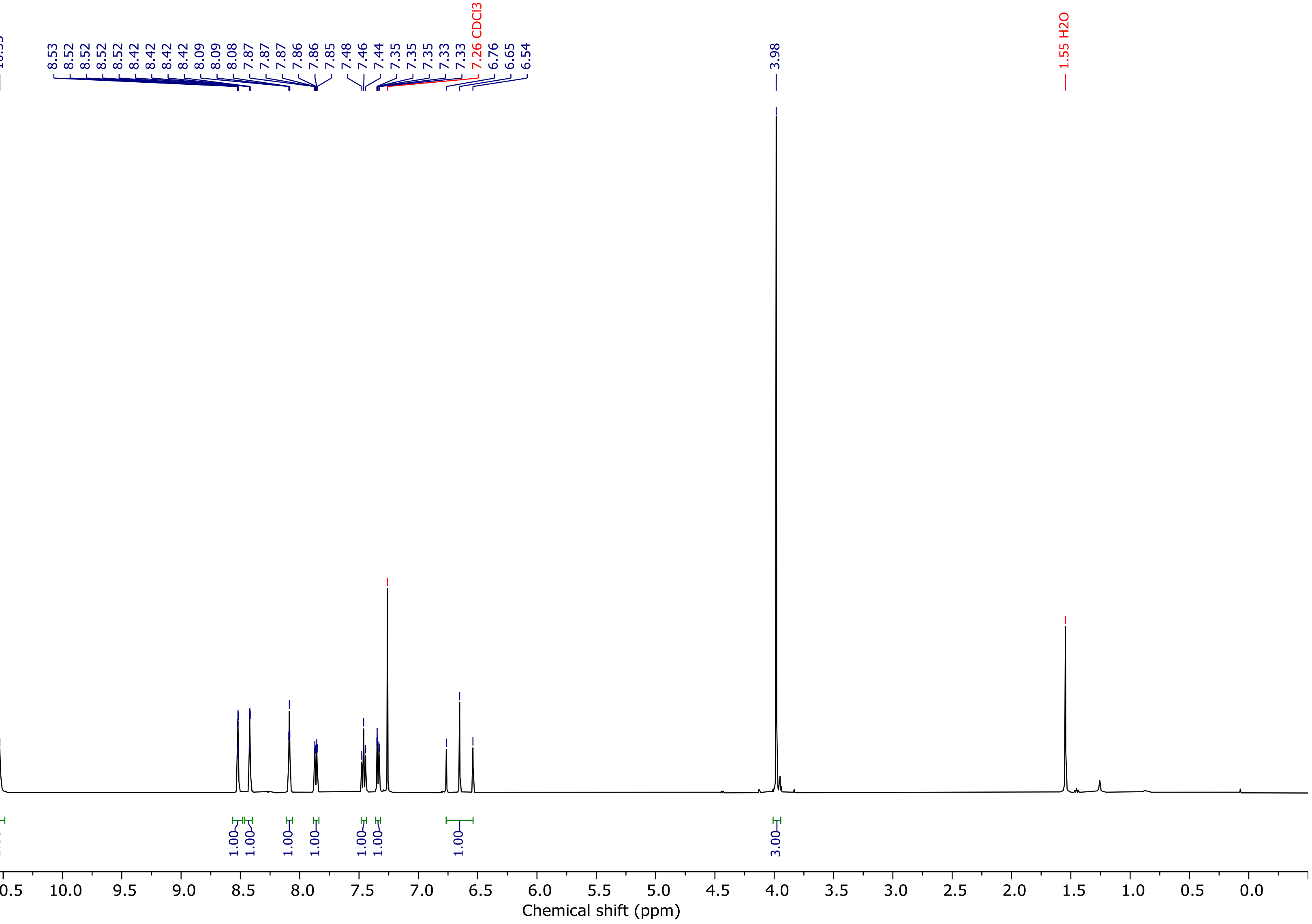

^{13}C NMR of 82

$CDCl_3$, 151 MHz, 23 ºC.

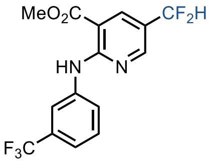

82

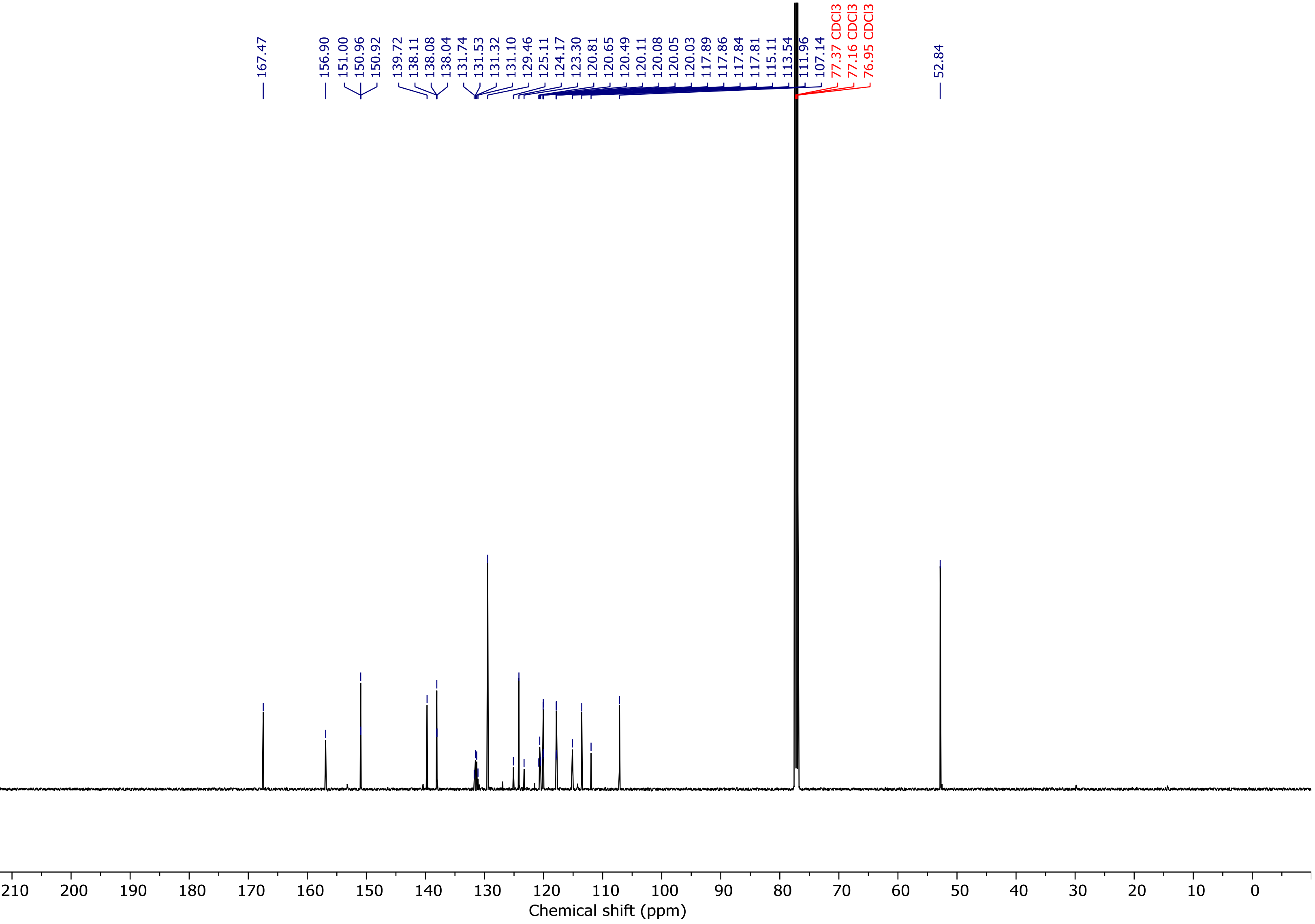

^{19}F NMR of 82

$CDCl_3$, 471 MHz, 23 ºC.

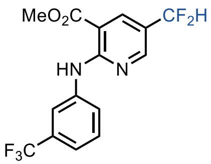

82

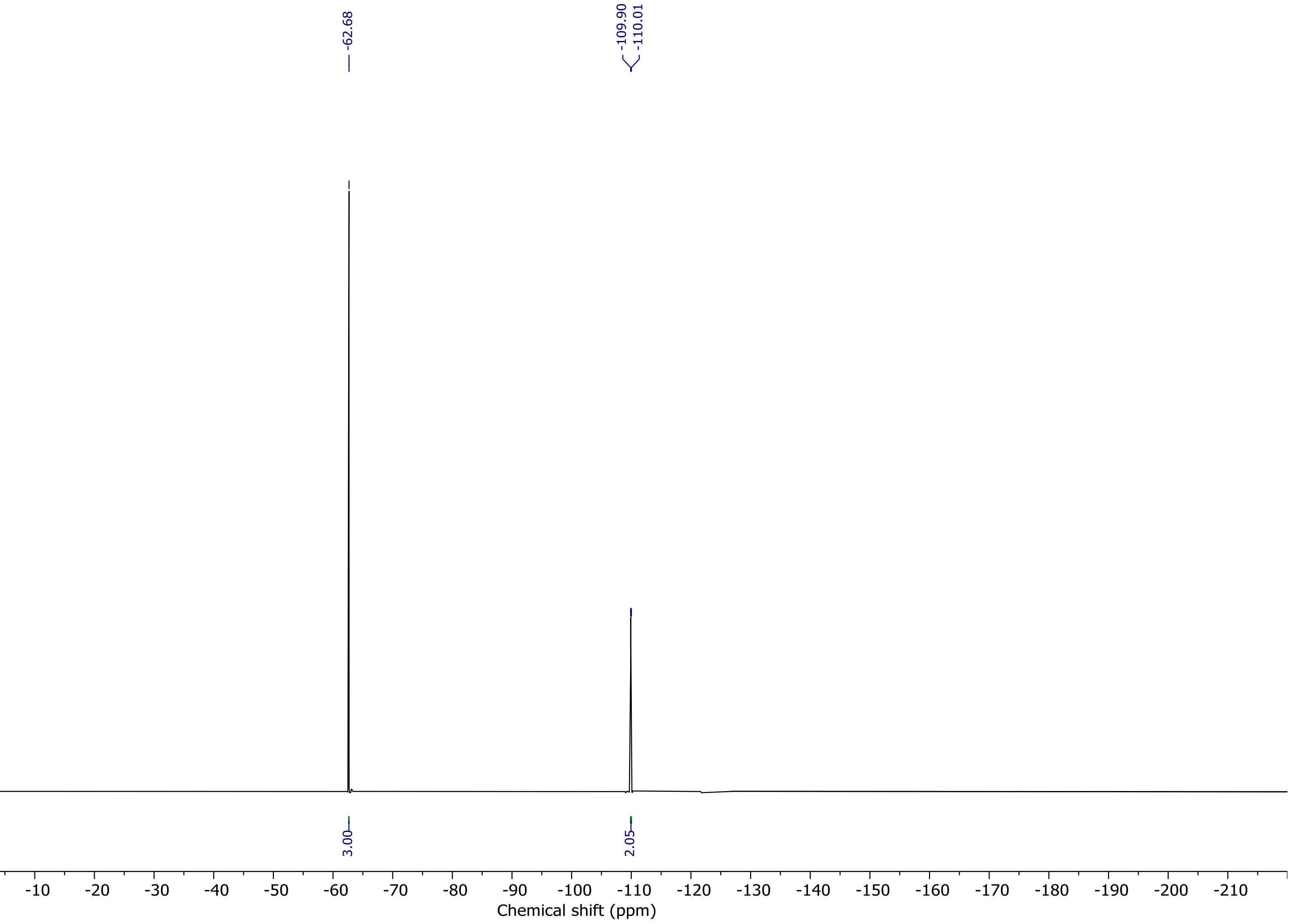

^{1}H NMR of 83

$CDCl_3$, 500 MHz, 23 ºC.

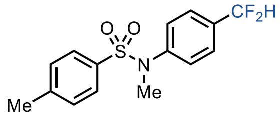

83

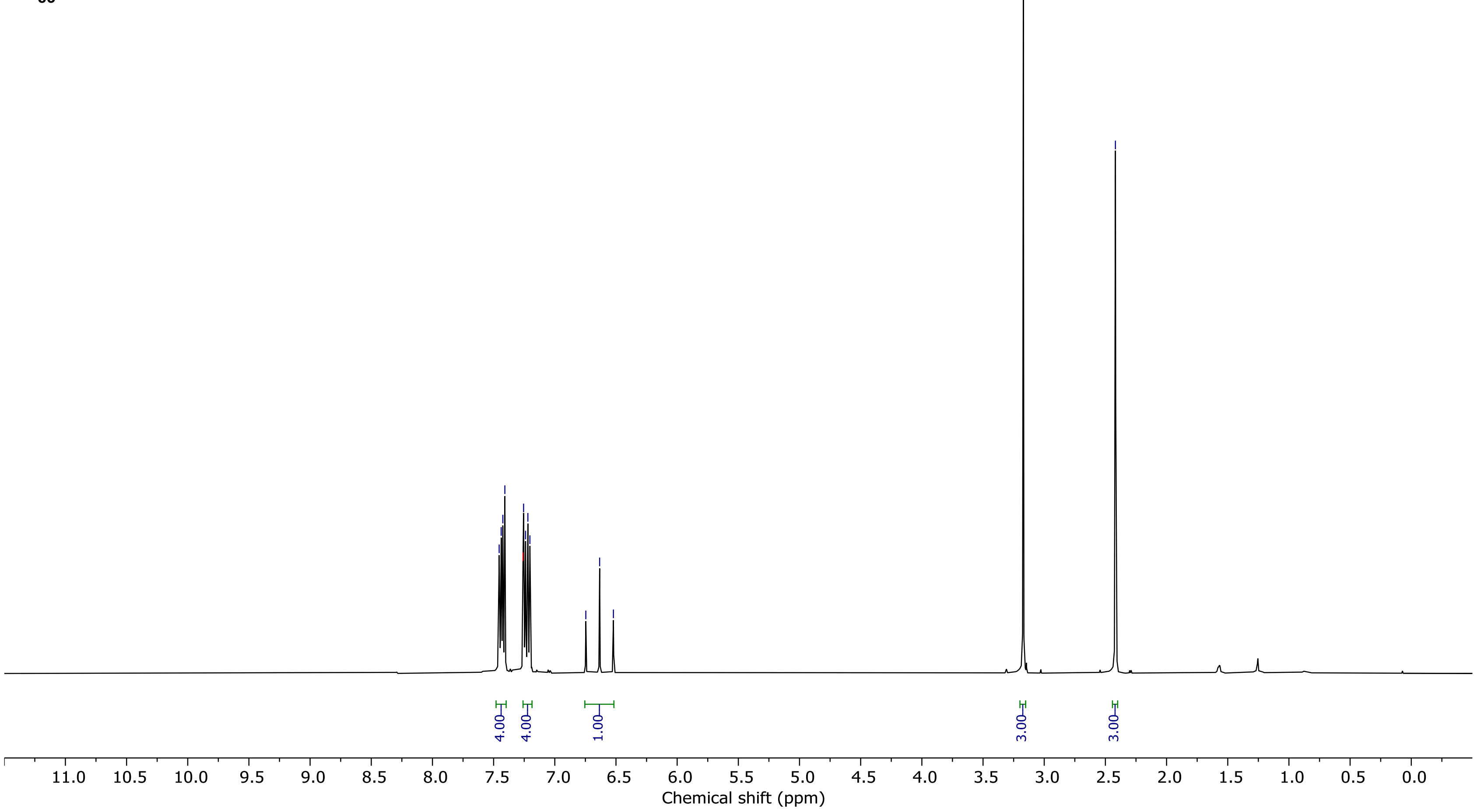

^{13}C NMR of 83

$CDCl_3$, 126 MHz, 23 ºC.

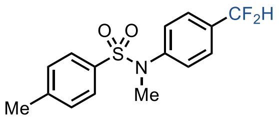

83

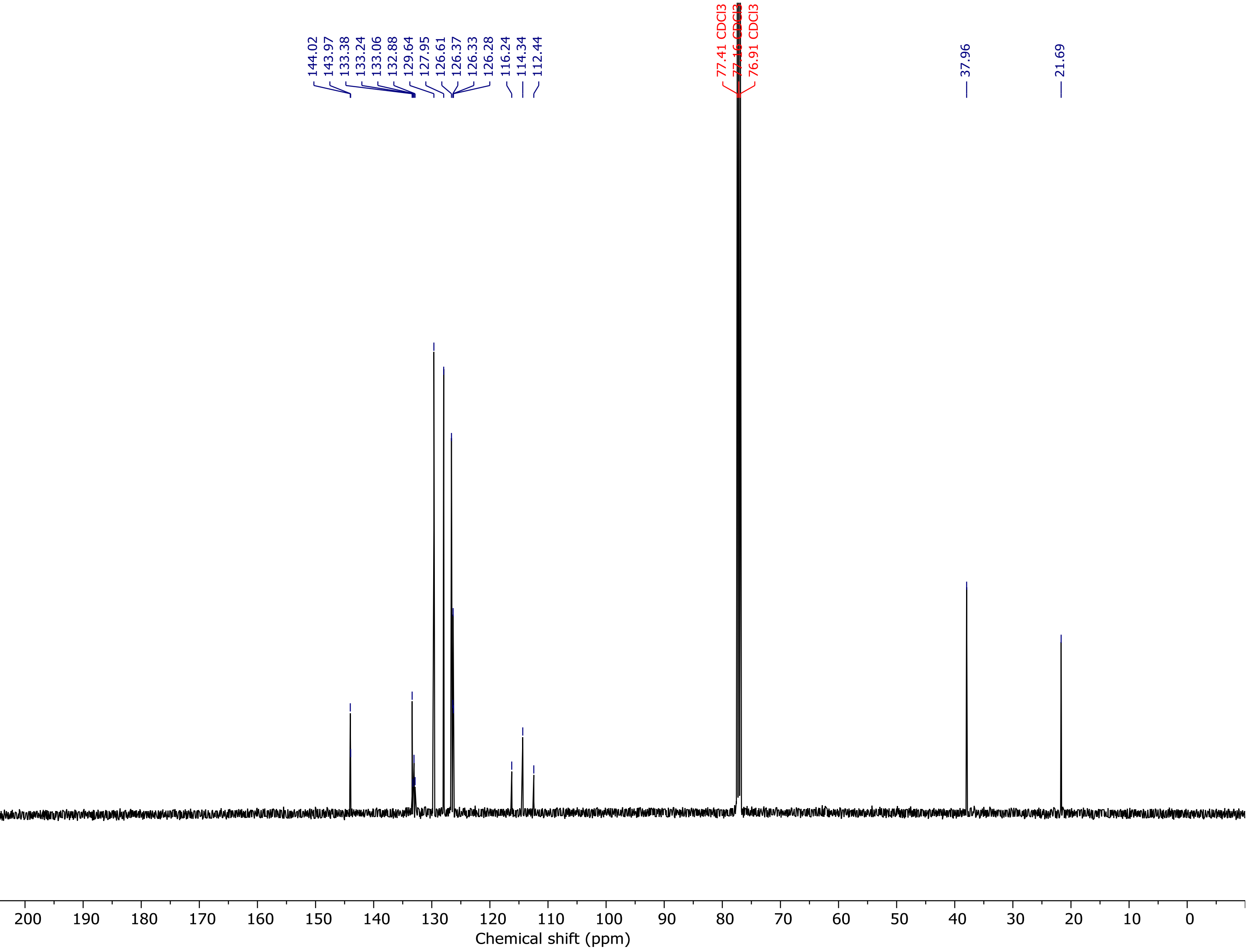

^{19}F NMR of 83

$CDCl_3$, 471 MHz, 23 ºC.

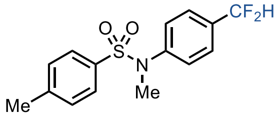

83

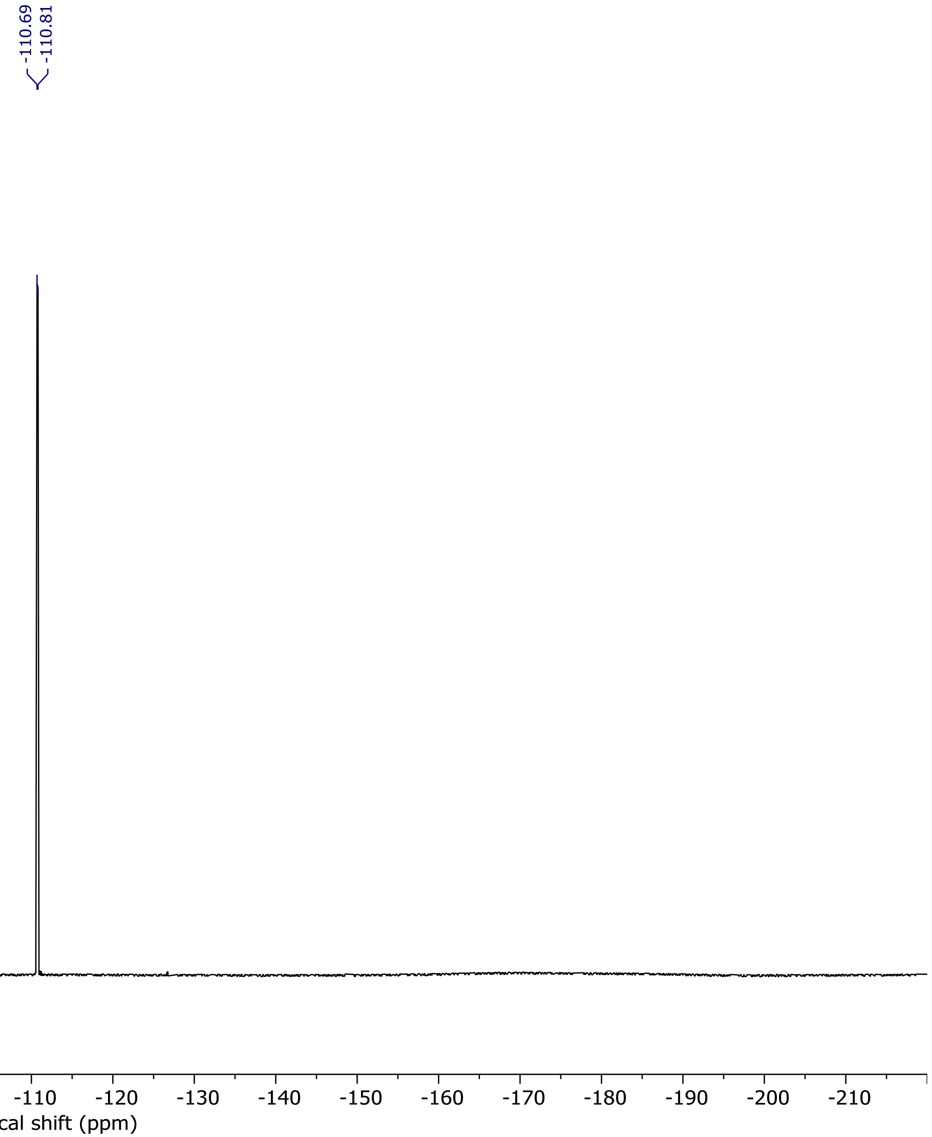

^{1}H NMR of 83a

$CDCl_3$, 500 MHz, 23 ºC.

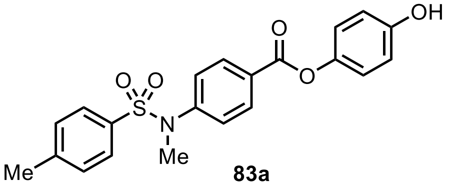

83a

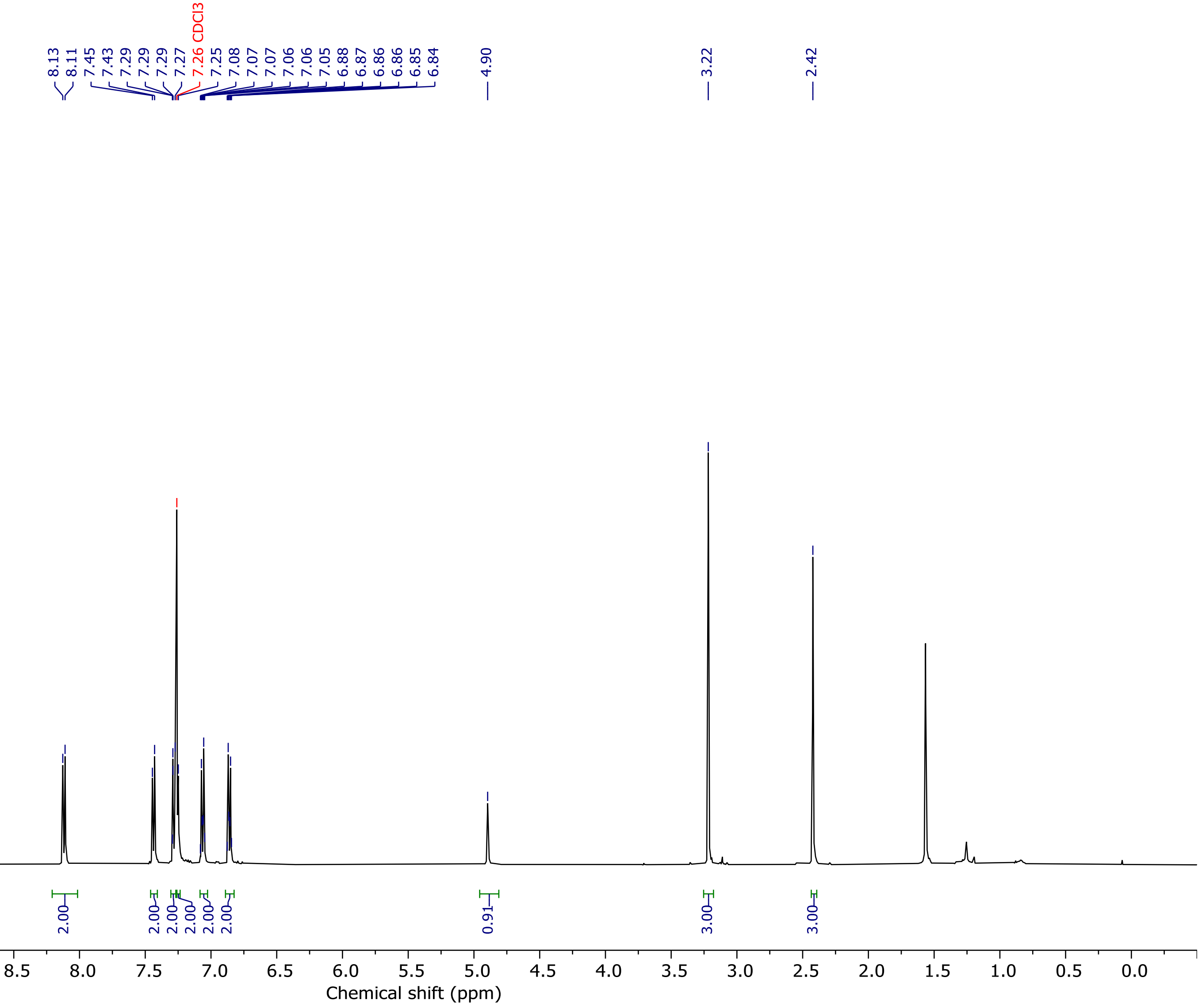

^{13}C NMR of 83a

$CDCl_3$, 151 MHz, 23 ºC.

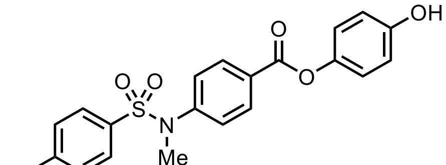

83a

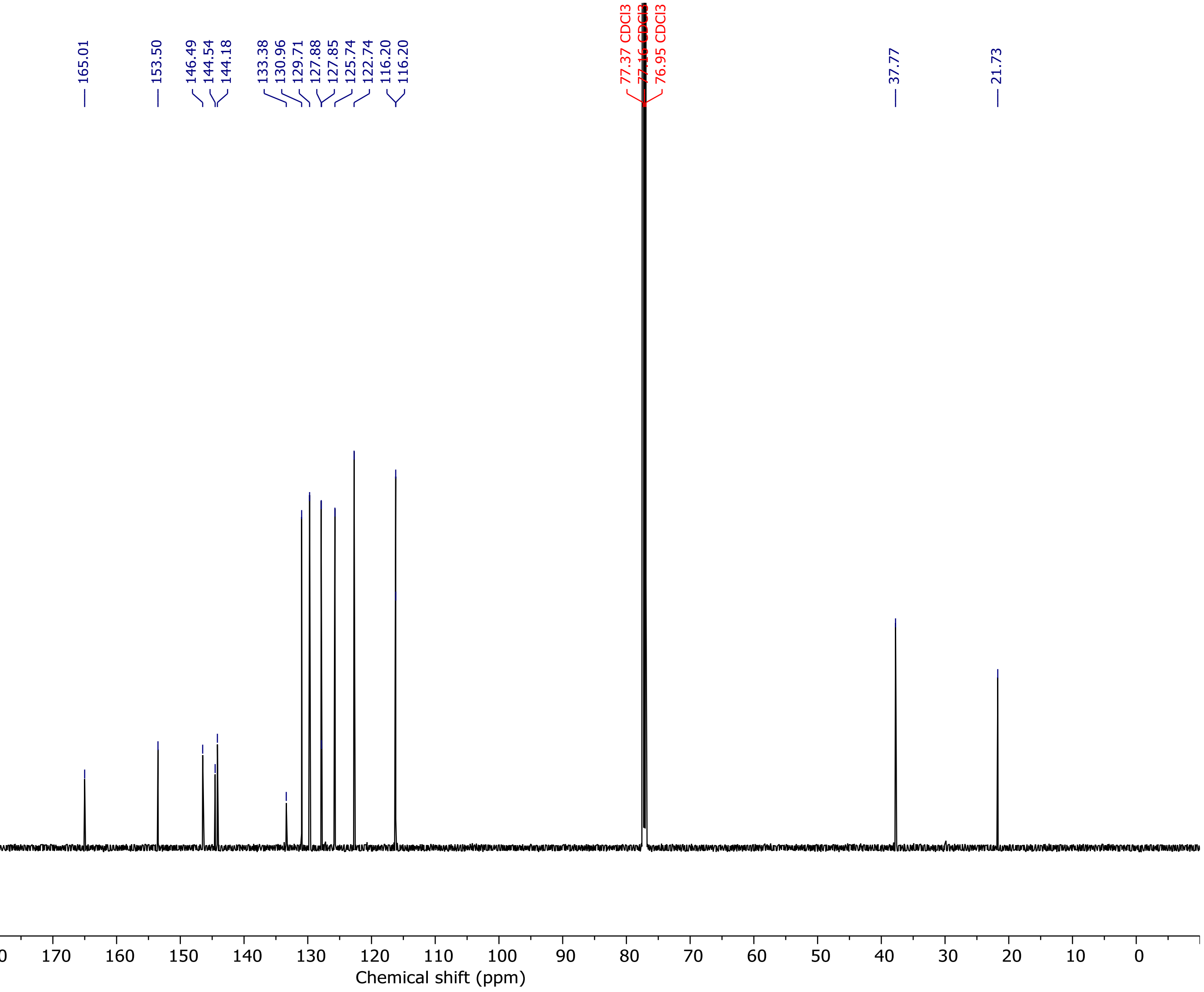

^{1}H NMR of 83b

$CDCl_3$, 500 MHz, 23 ºC.

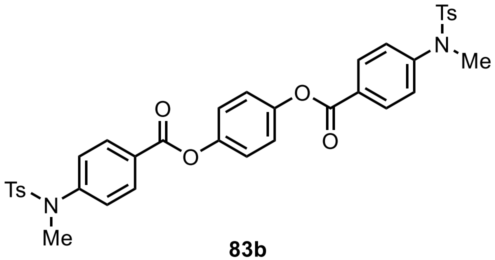

83b

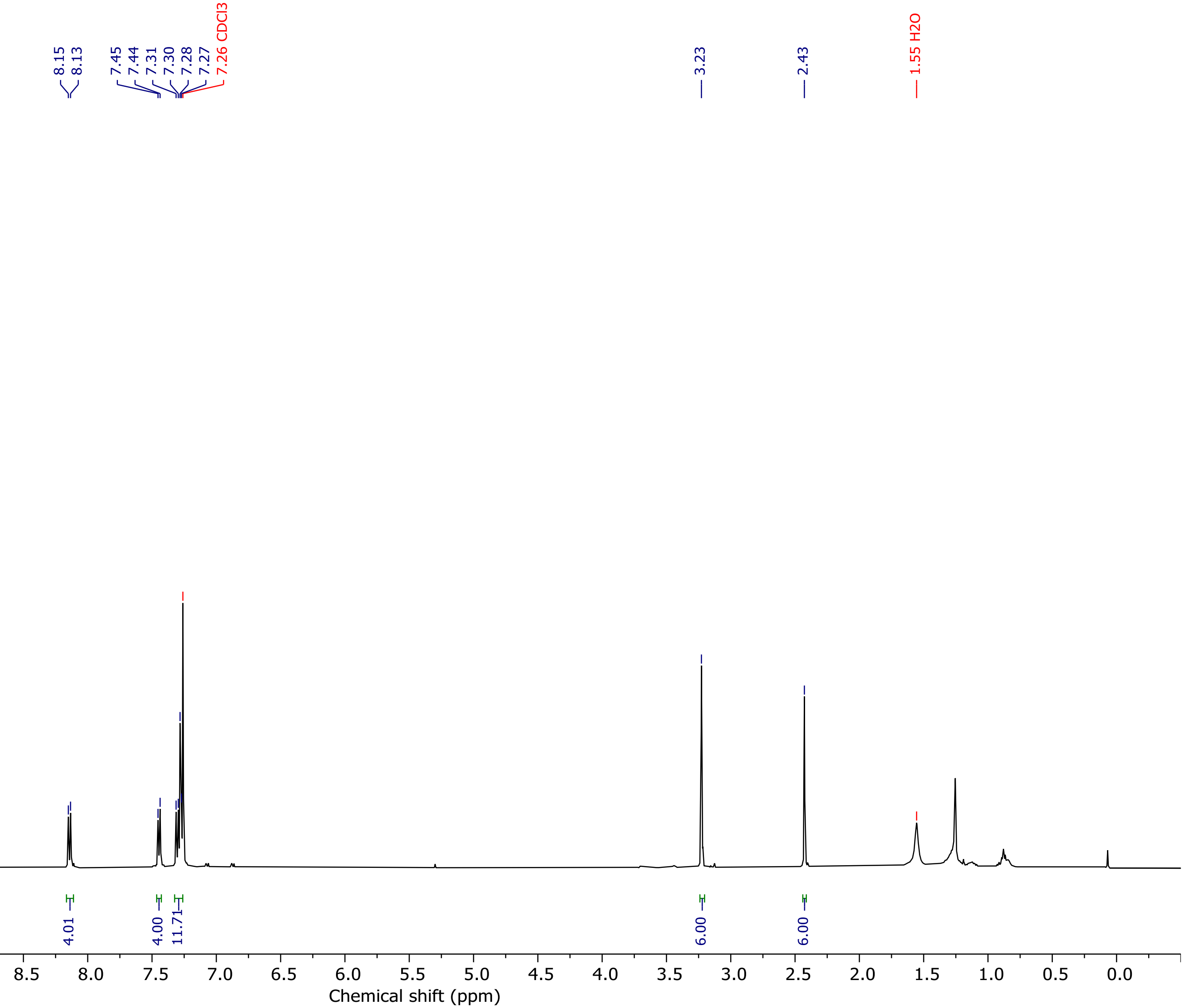

^{13}C NMR of 83b

$CDCl_3$, 151 MHz, 23 ºC.

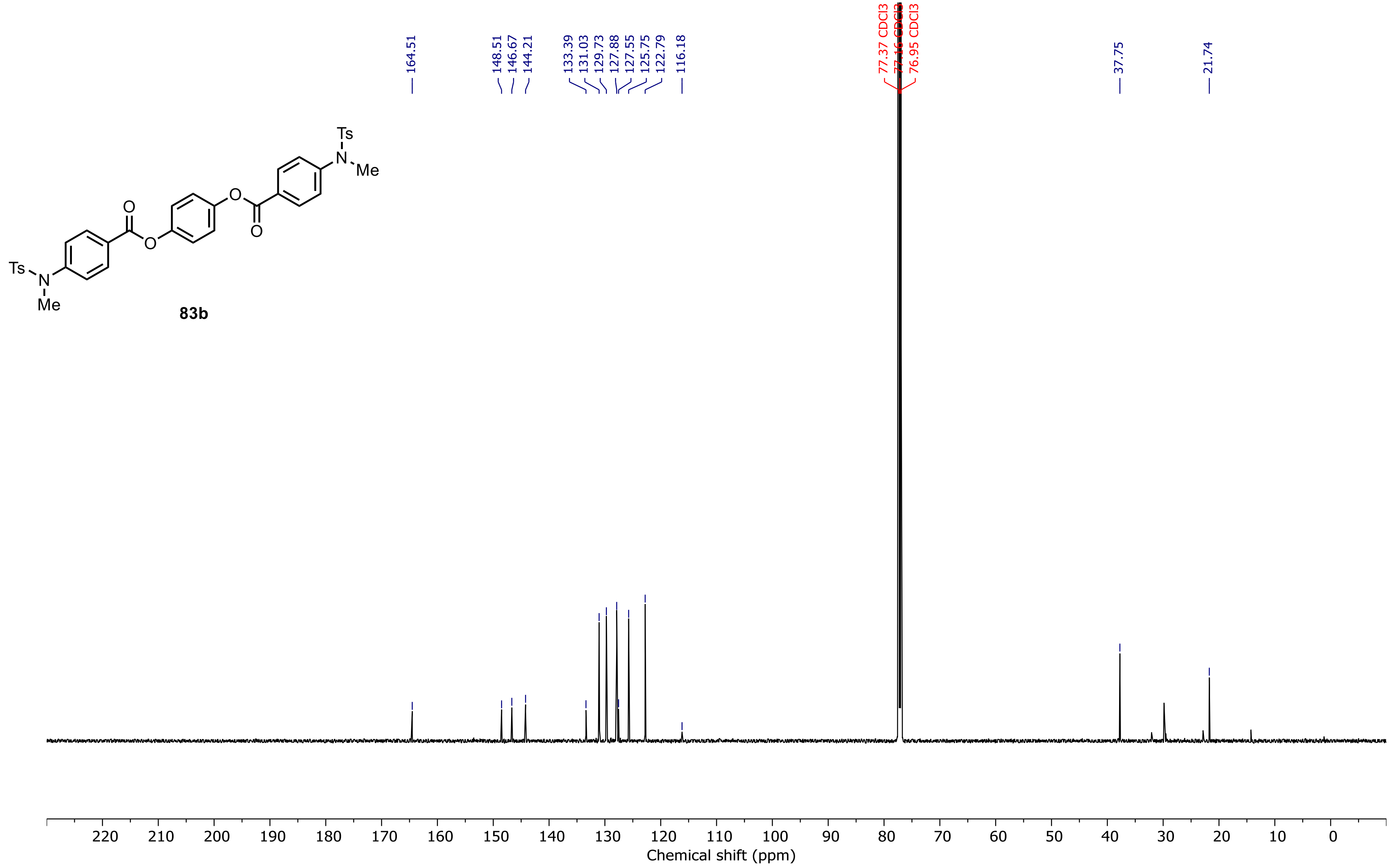

^{1}H NMR of 83c

$CDCl_3$, 500 MHz, 23 ºC.

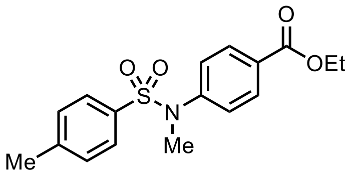

83c

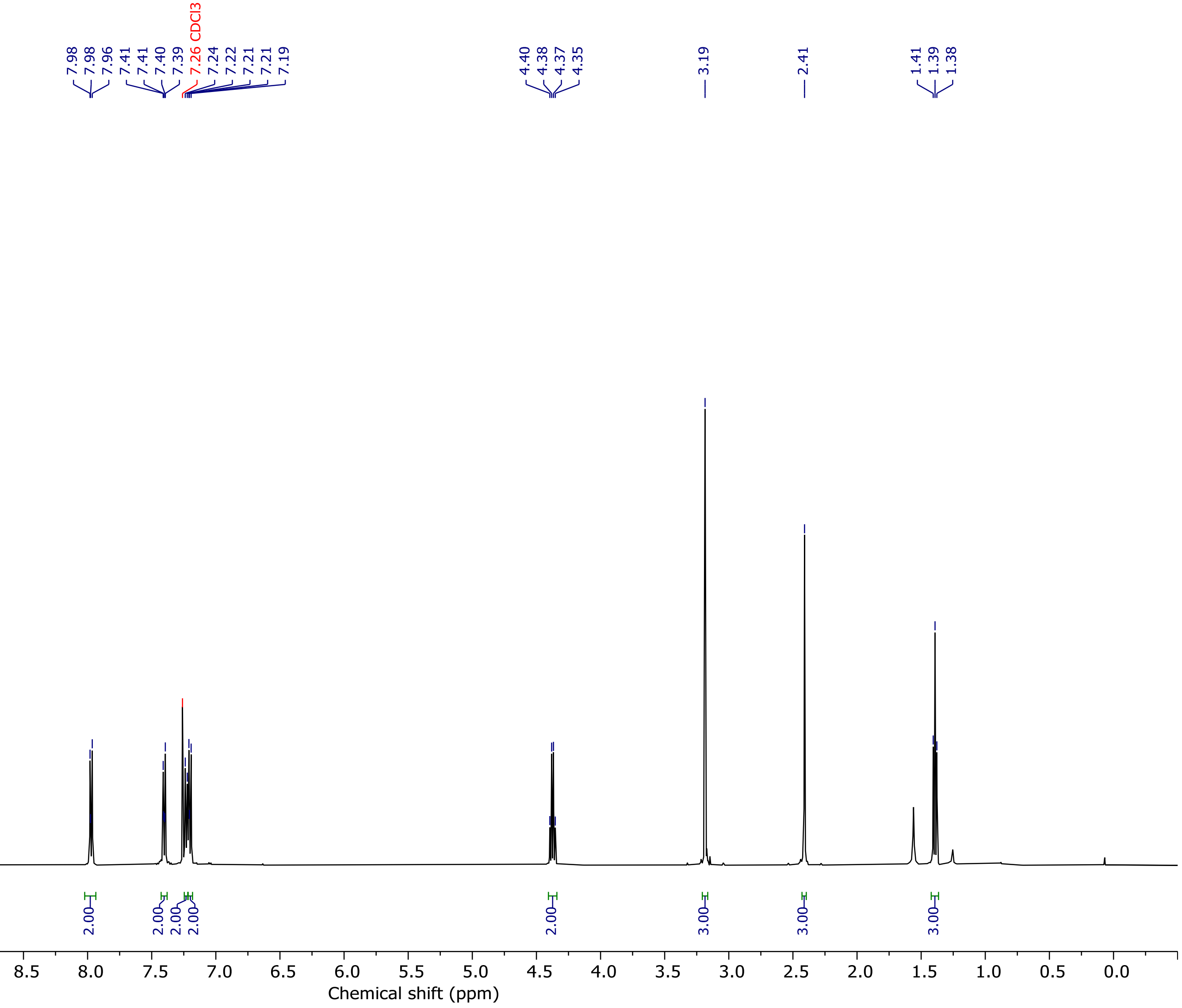

^{13}C NMR of 83c

$CDCl_3$, 151 MHz, 23 ºC.

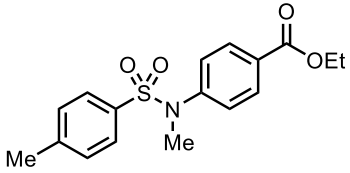

83c

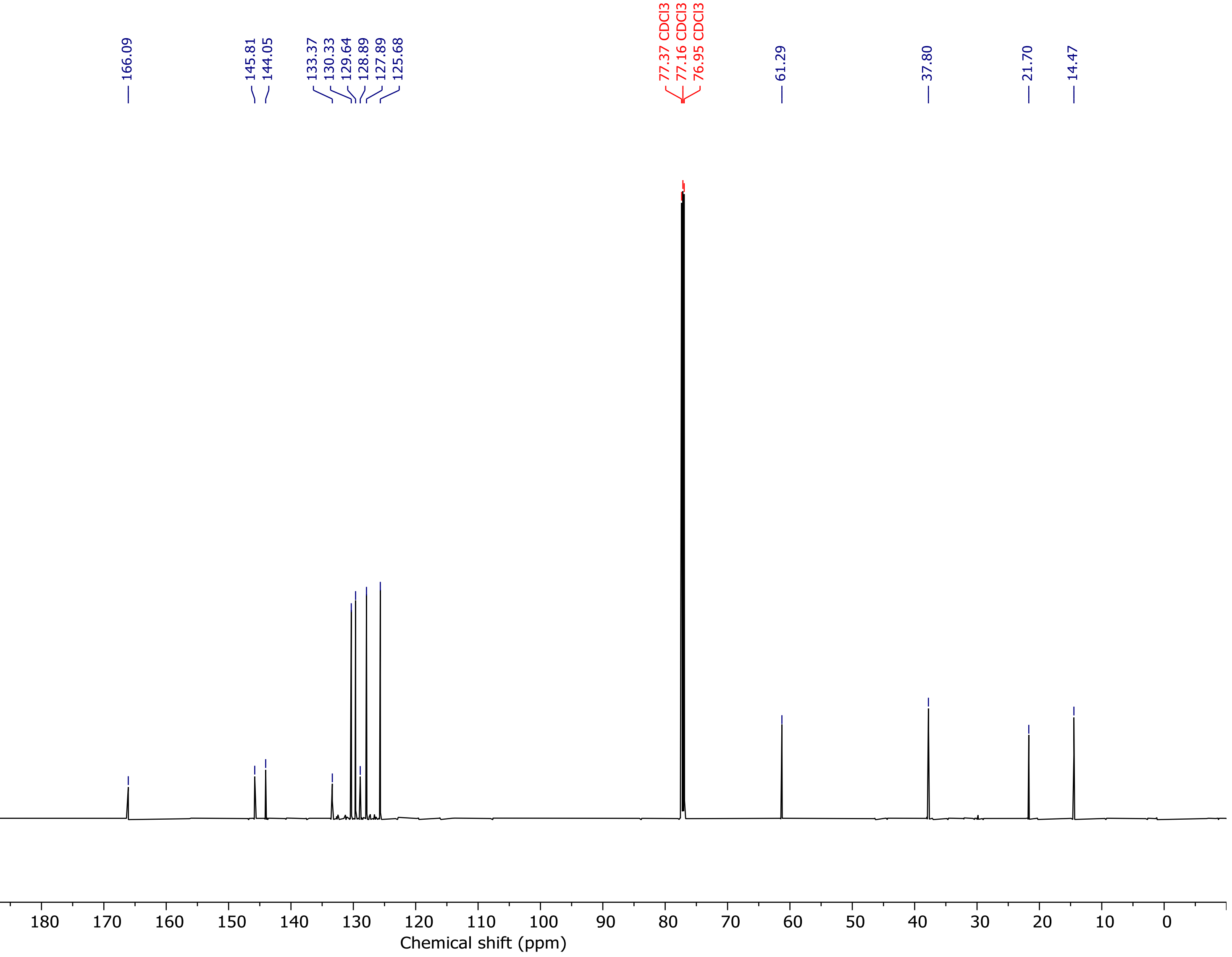

^{1}H NMR of 83d

$CDCl_3$, 500 MHz, 23 ºC.

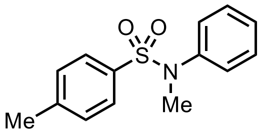

83d

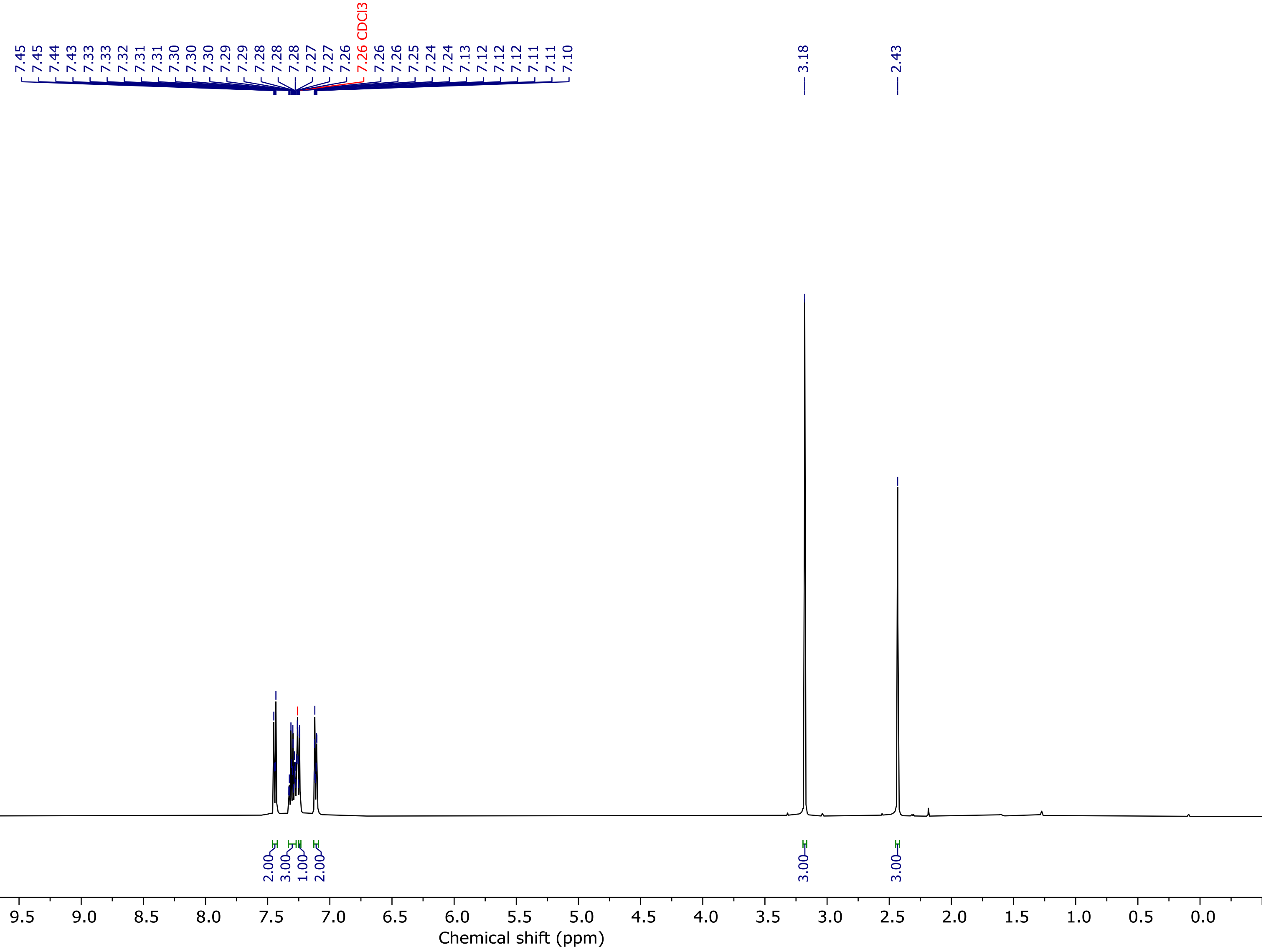

^{13}C NMR of 83d

$CDCl_3$, 126 MHz, 23 ºC.

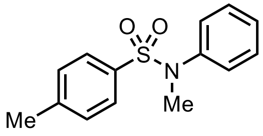

83d

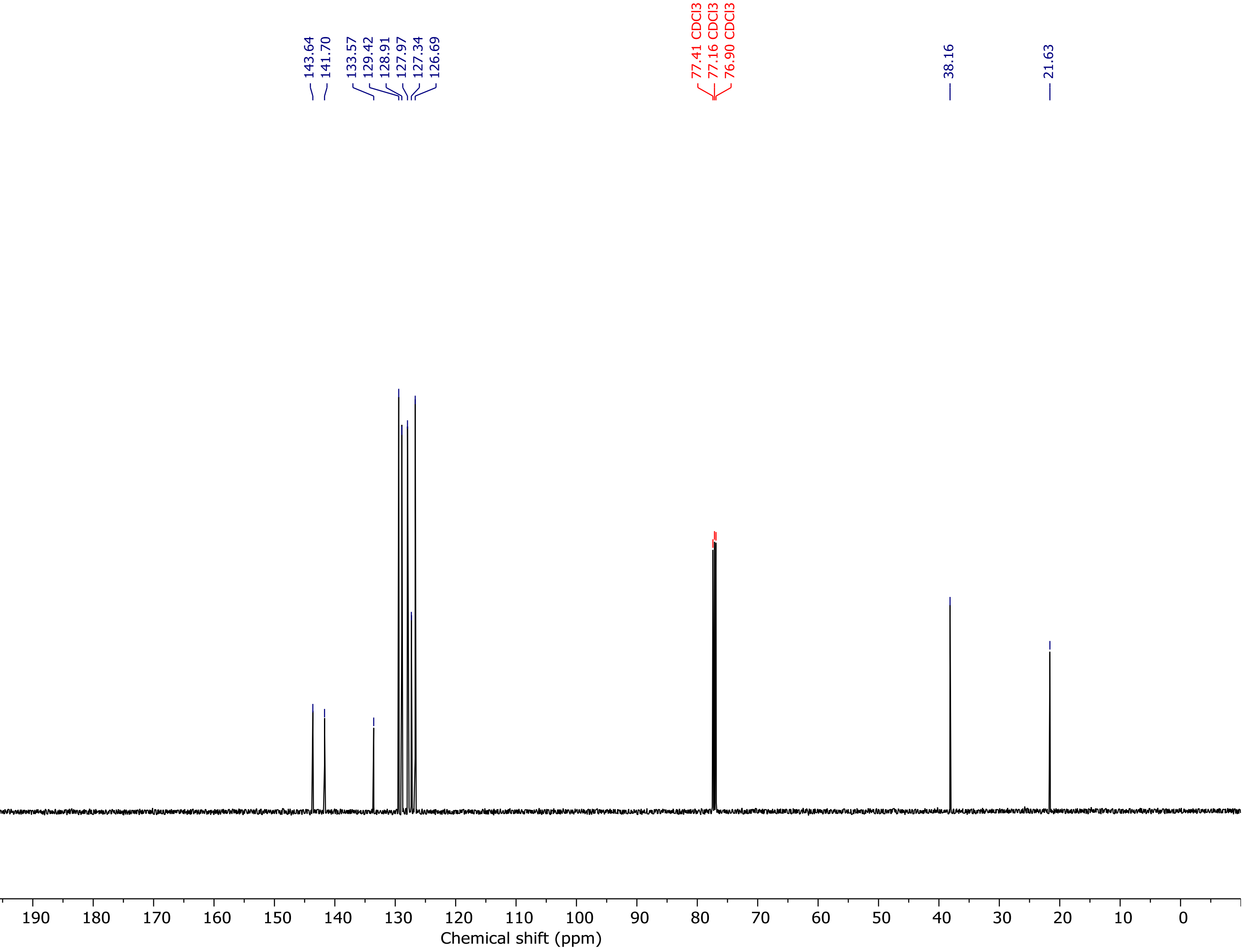

^{1}H NMR of 84

$CDCl_3$, 600 MHz, 23 ºC.

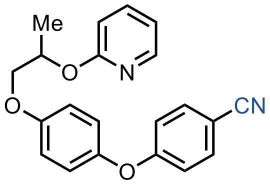

84

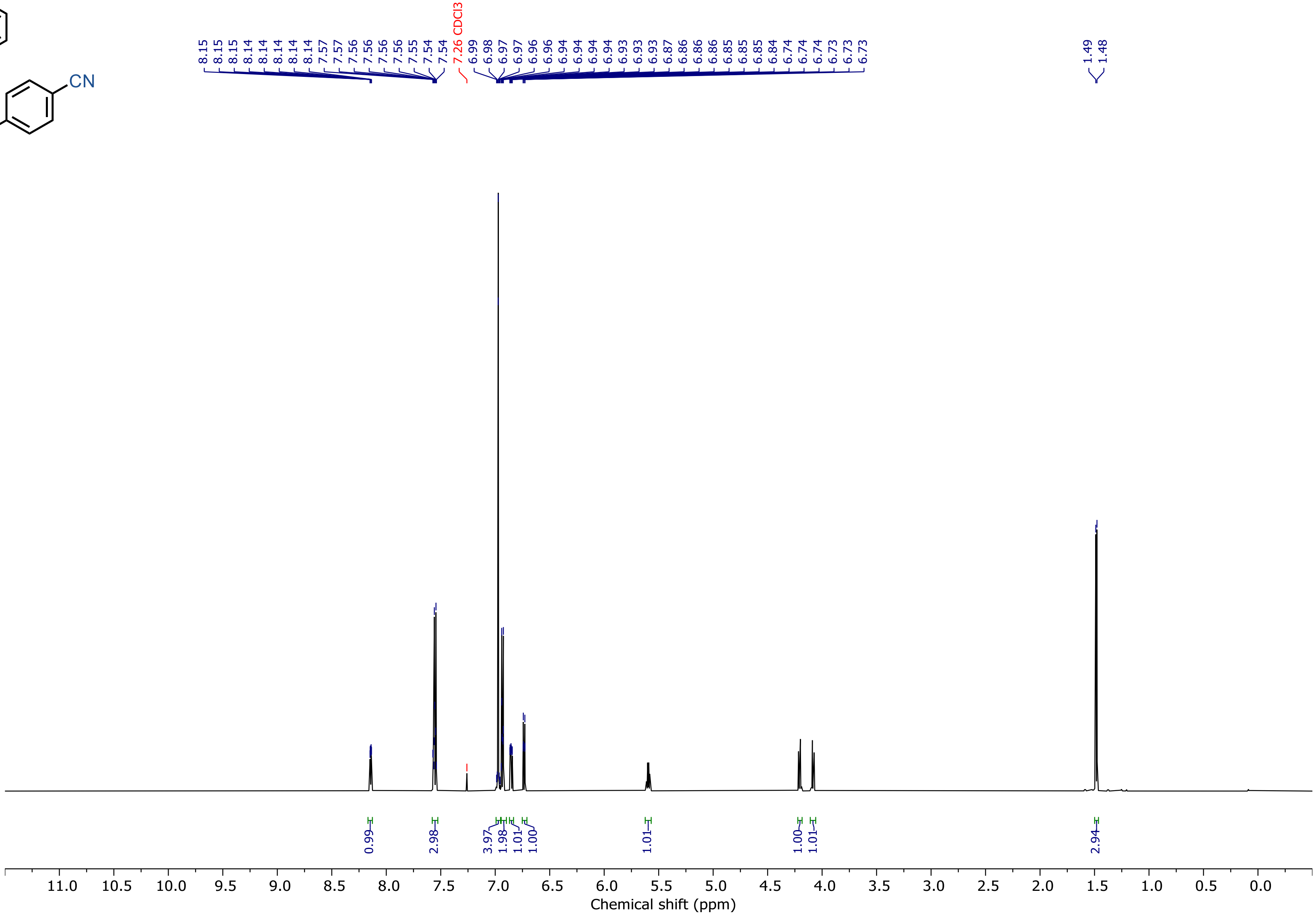

^{13}C NMR of 84

$CDCl_3$, 151 MHz, 23 ºC.

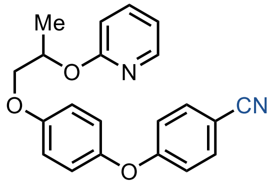

84

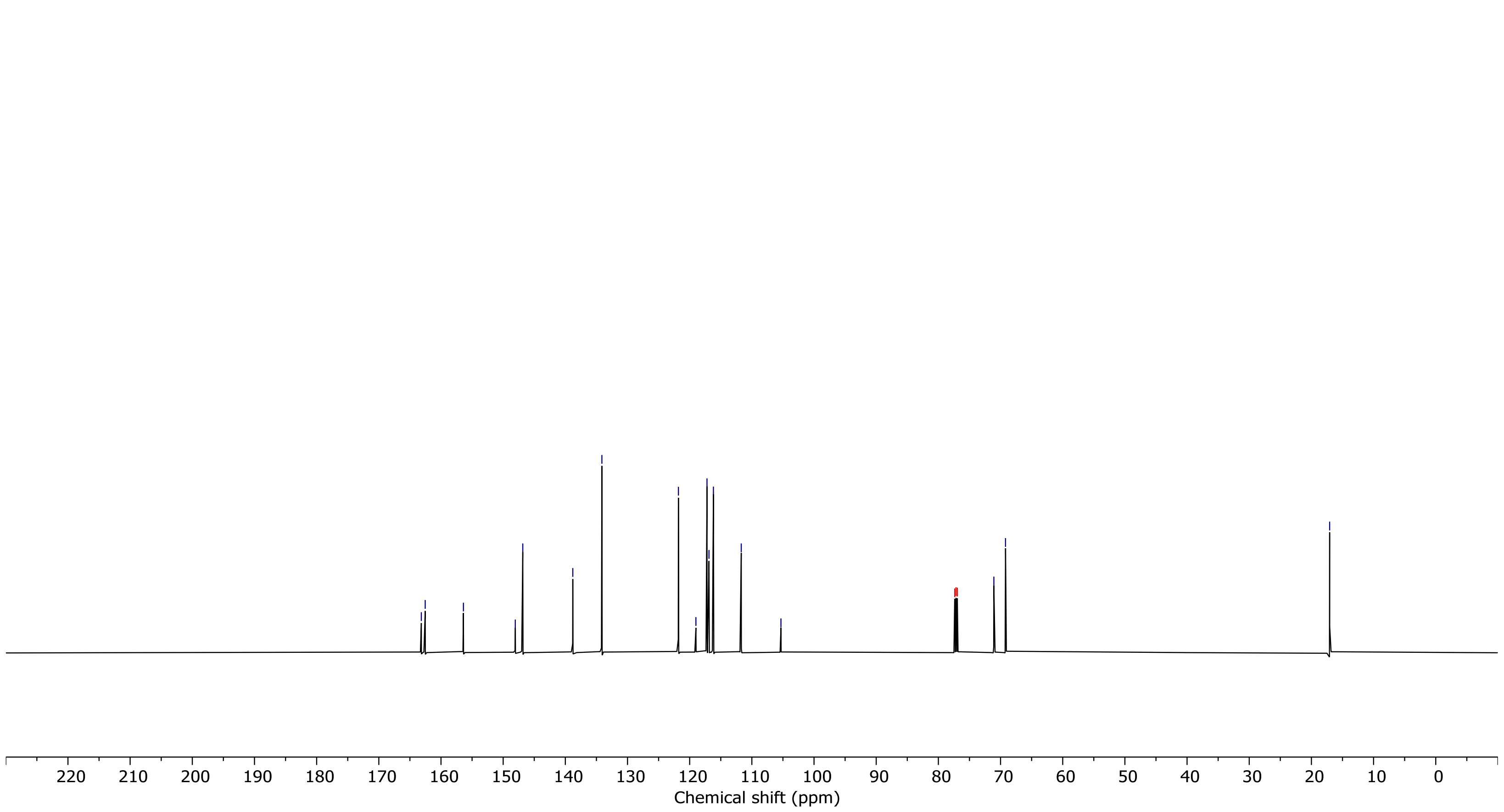

^{1}H NMR of 85

$CDCl_3$, 500 MHz, 23 ºC.

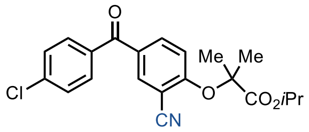

85

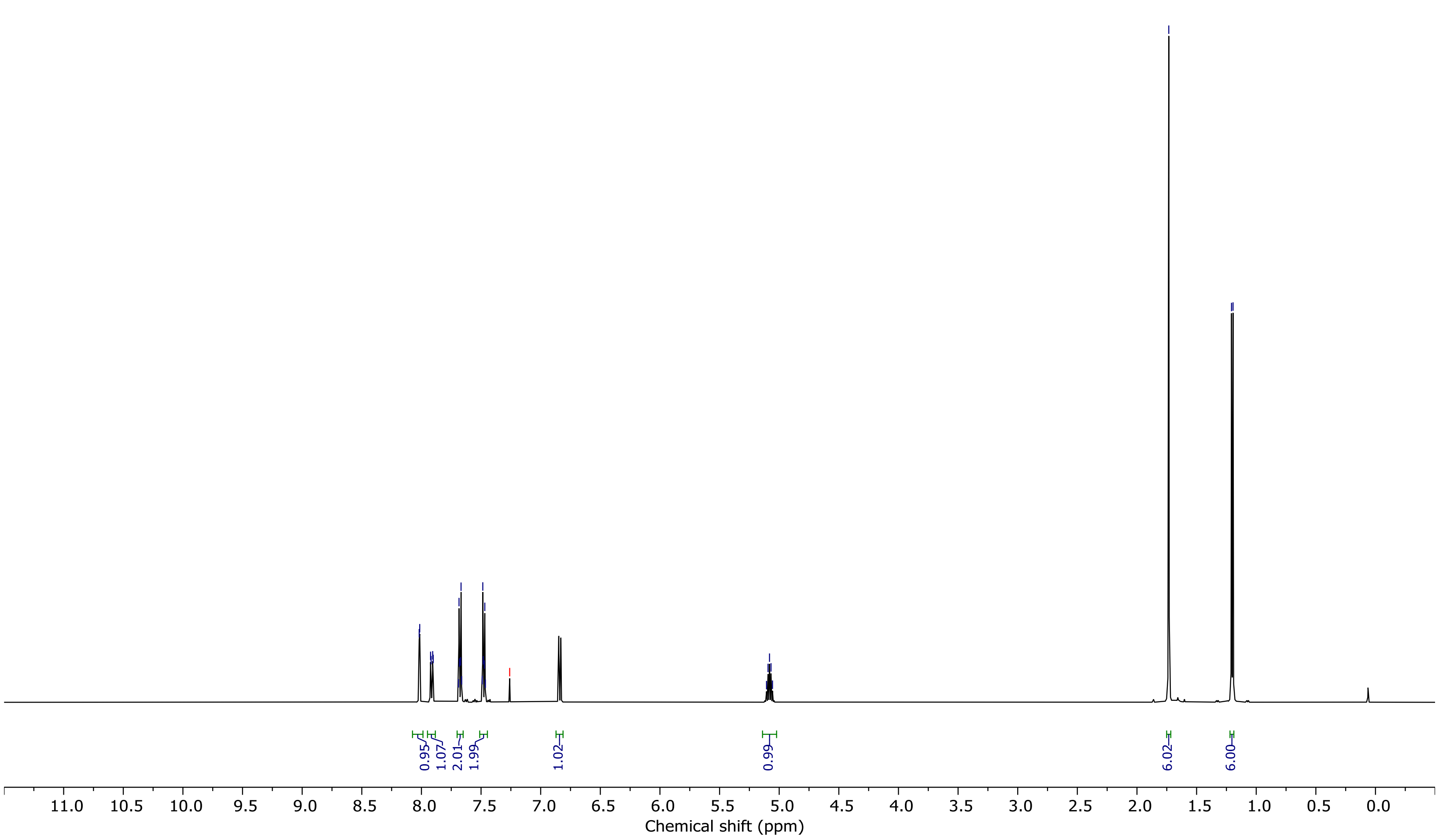

^{13}C NMR of 85

$CDCl_3$, 126 MHz, 23 ºC.

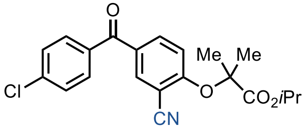

85

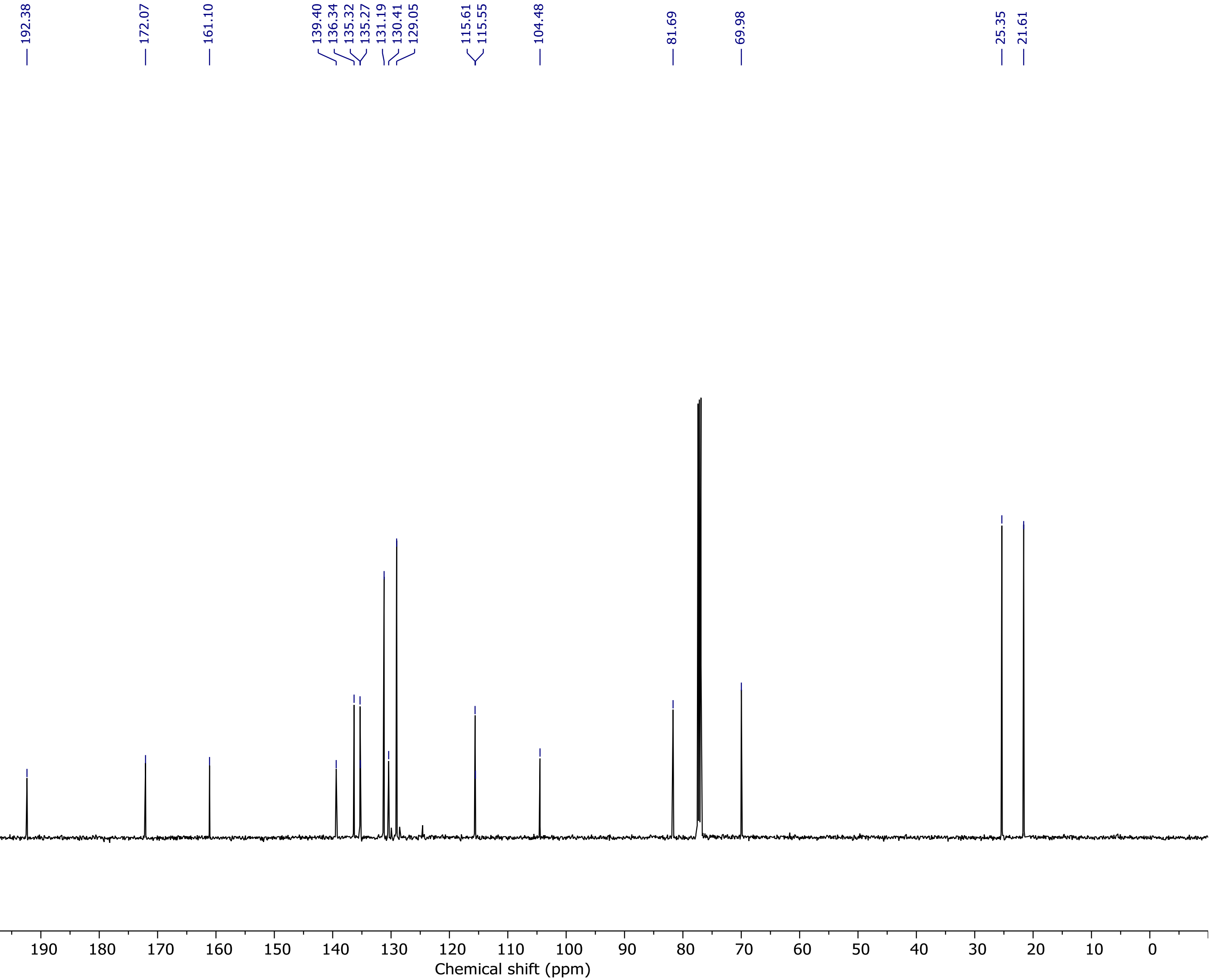

^{1}H NMR of 86

$CDCl_3$, 500 MHz, 23 ºC.

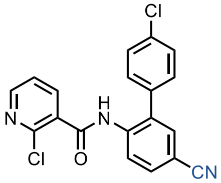

86

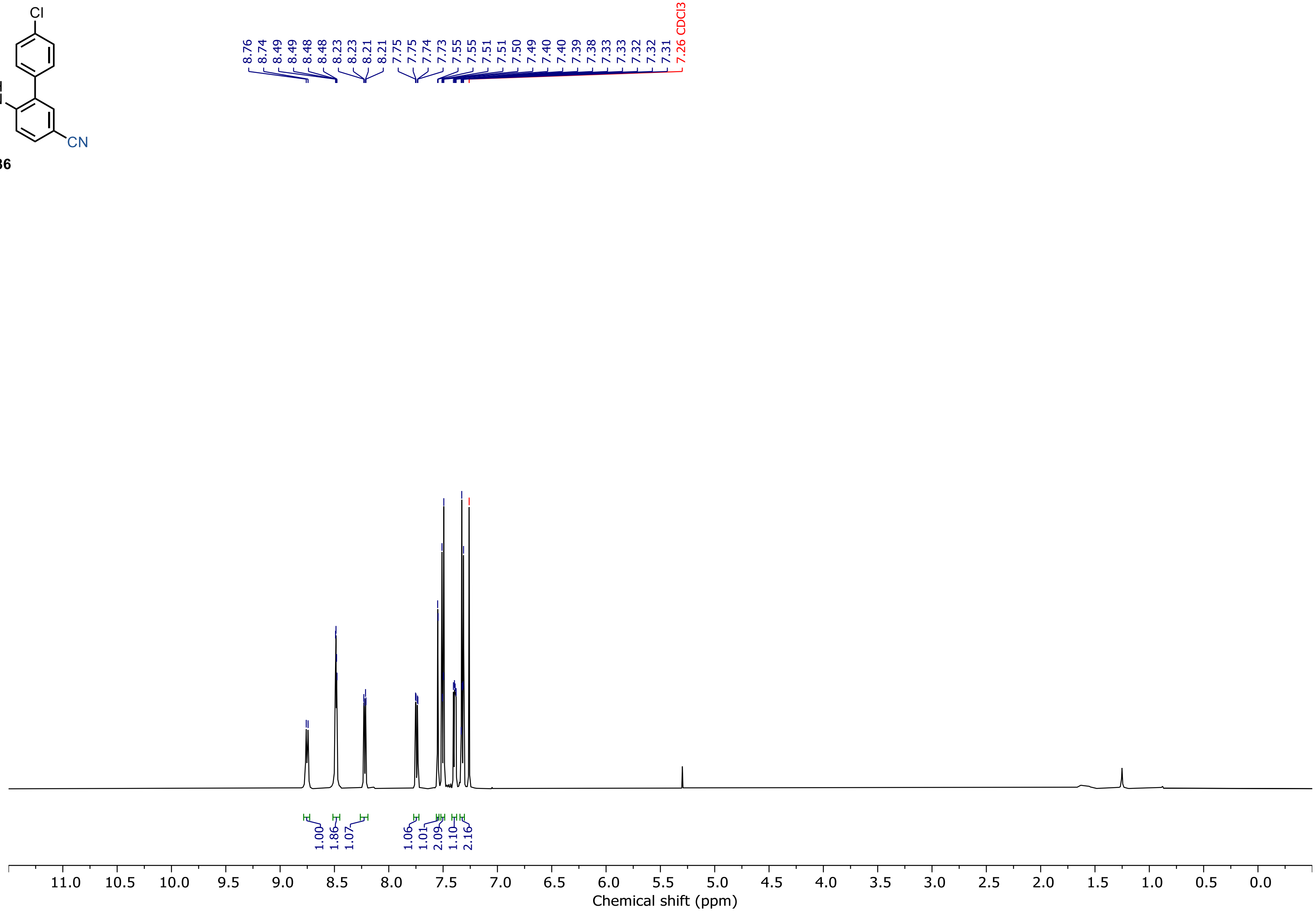

^{13}C NMR of 86

$CDCl_3$, 126 MHz, 23 ºC.

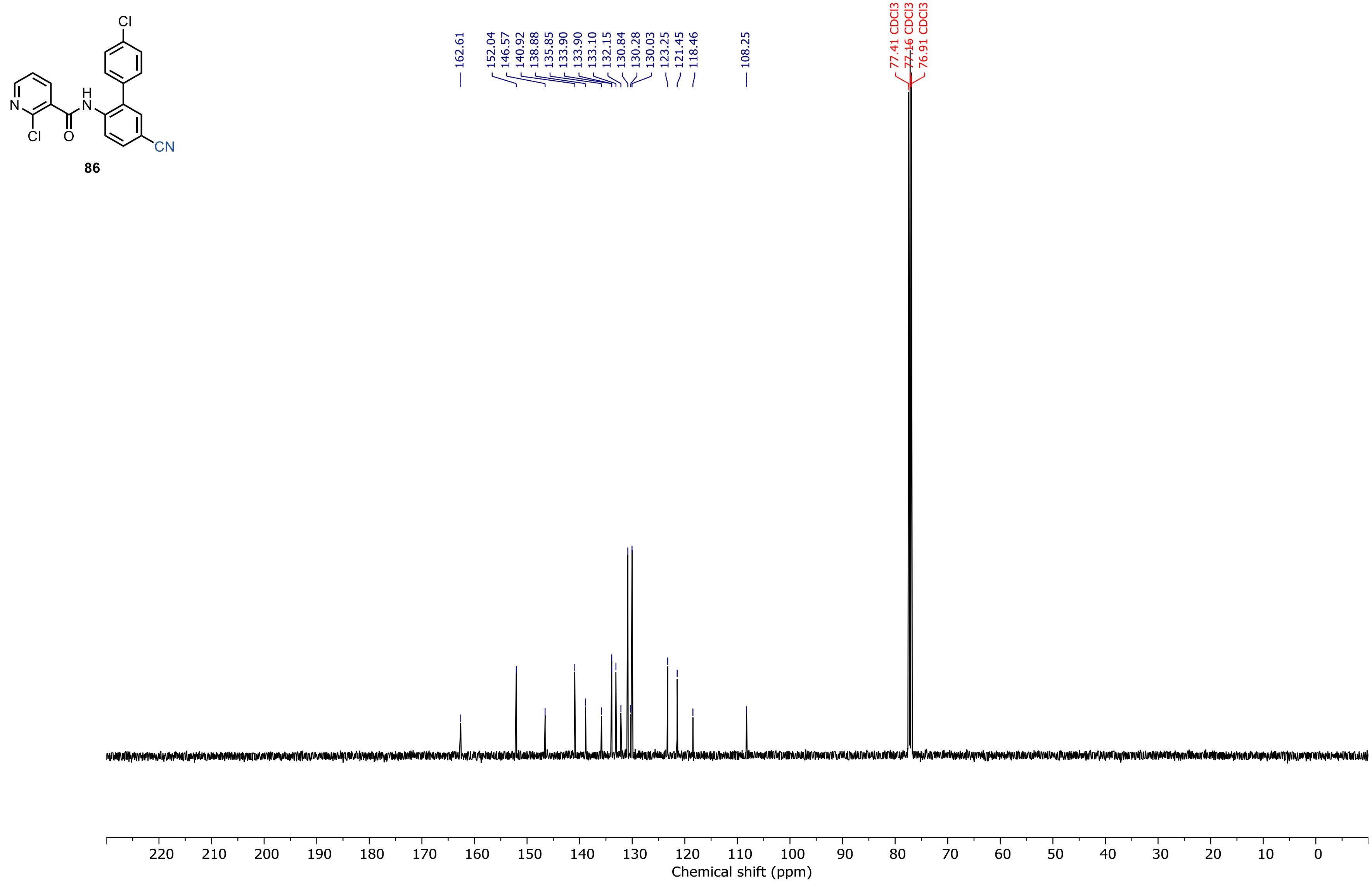

^{1}H NMR of 87

$CDCl_3$, 500 MHz, 23 ºC.

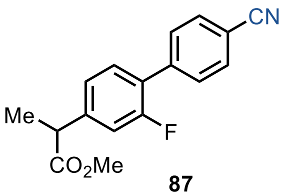

87

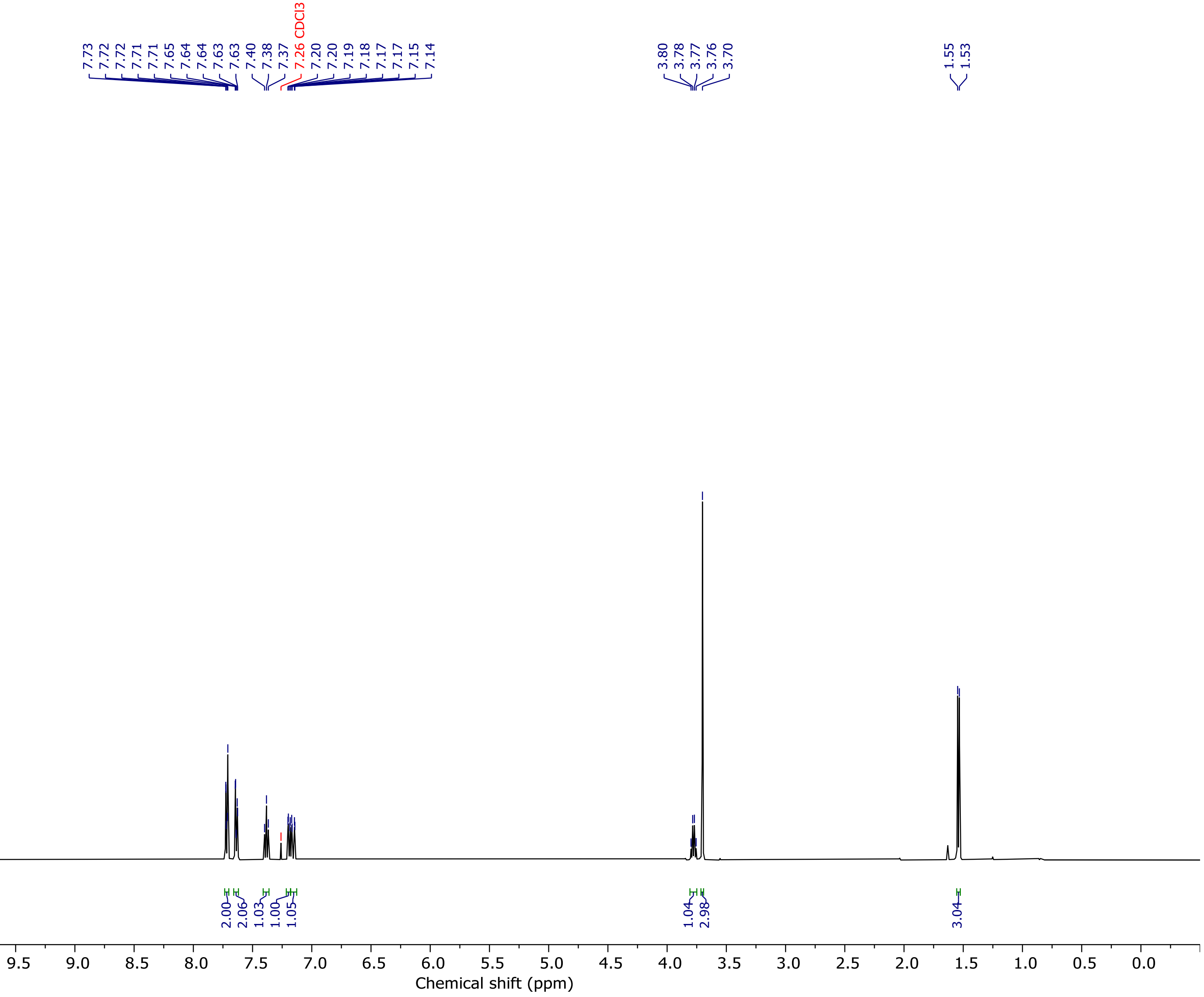

^{13}C NMR of 87

$CDCl_3$, 126 MHz, 23 ºC.

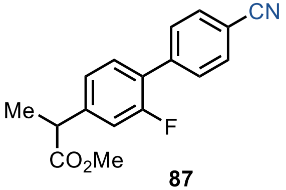

87

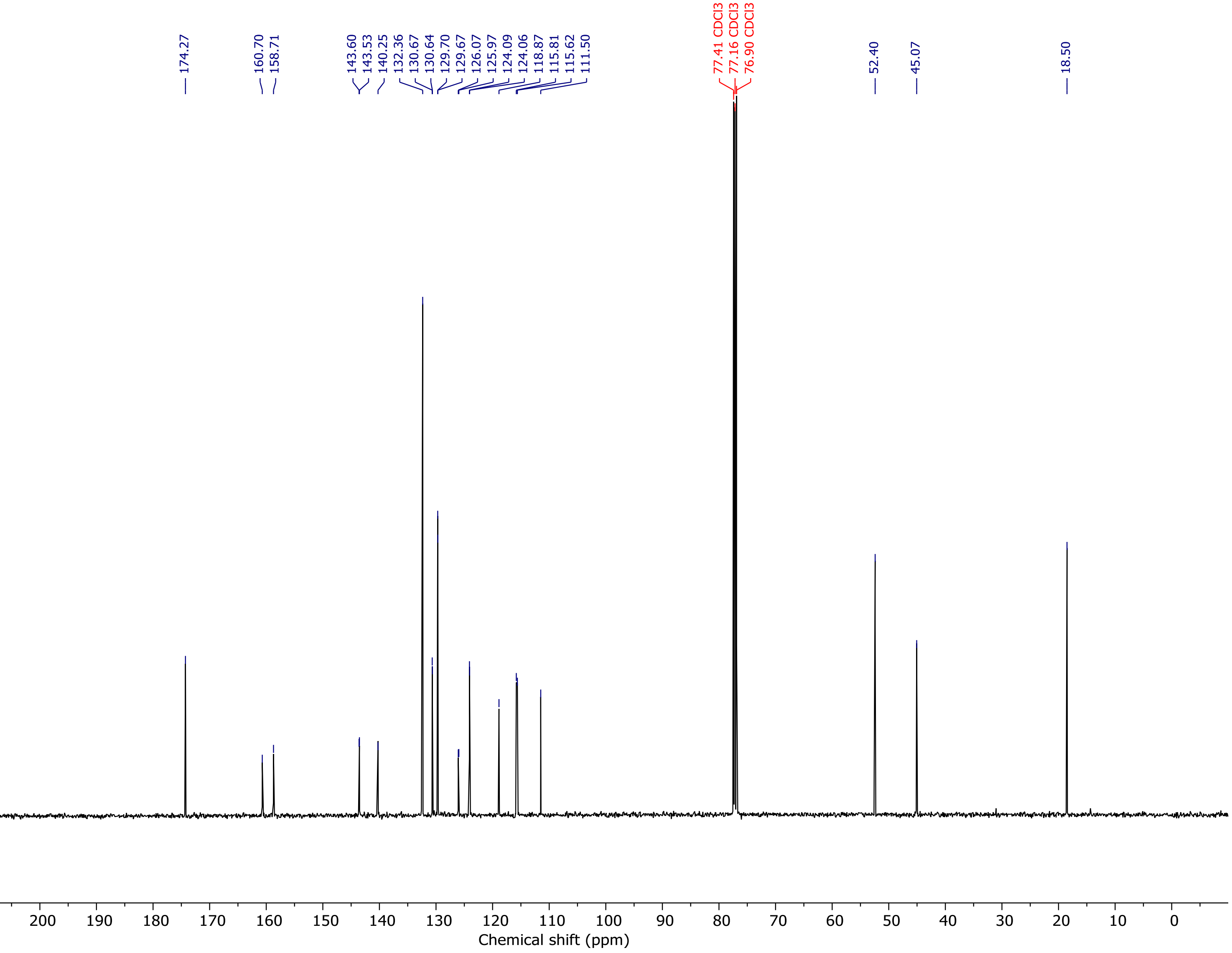

^{19}F NMR of 87

$CDCl_3$, 471 MHz, 23 ºC.

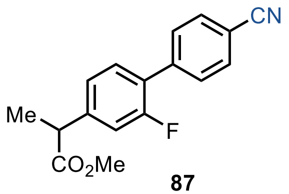

87

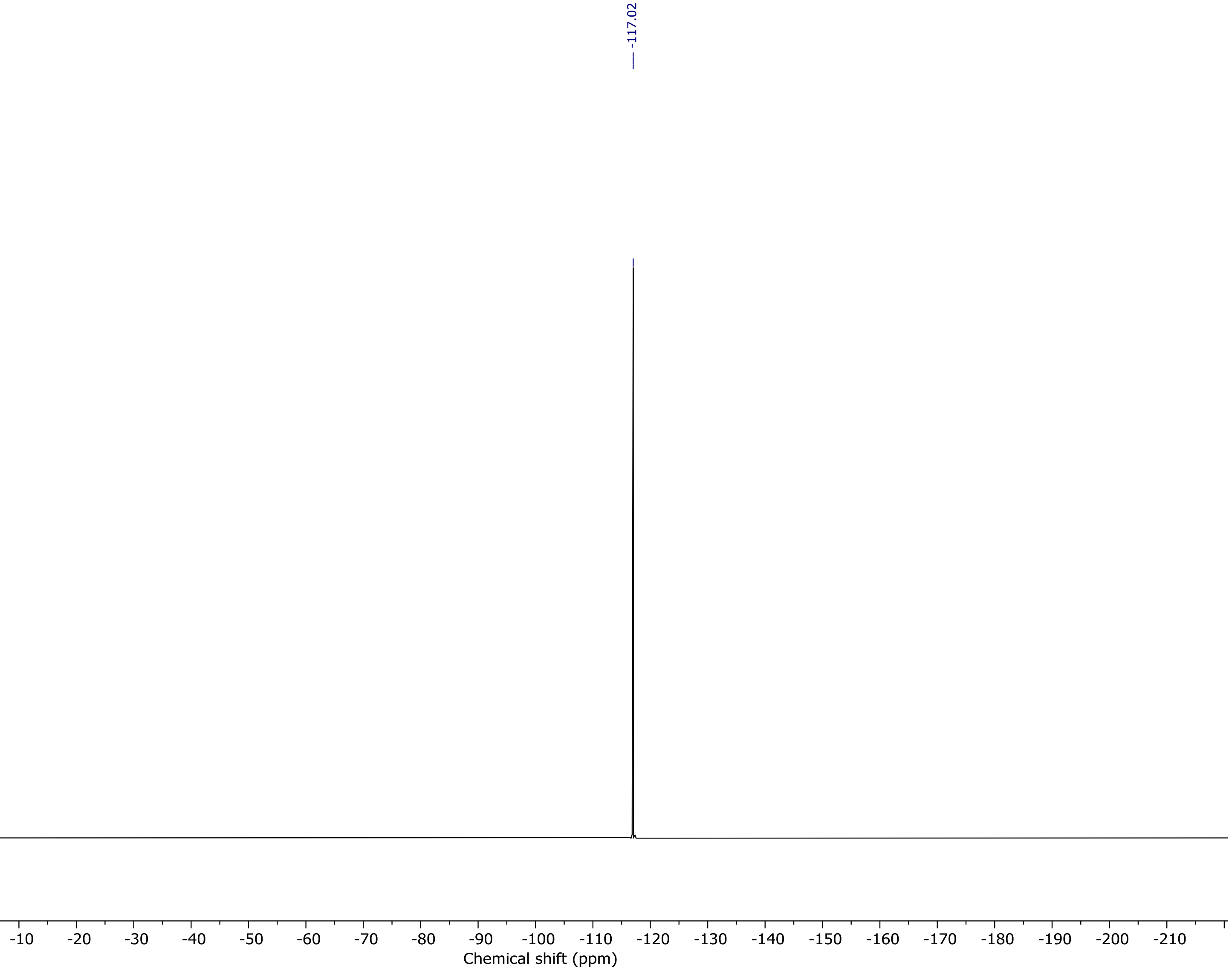

^{1}H NMR of 88

$CDCl_3$, 600 MHz, 23 ºC.

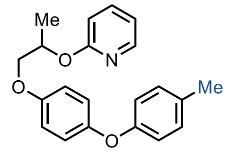

(±)-88

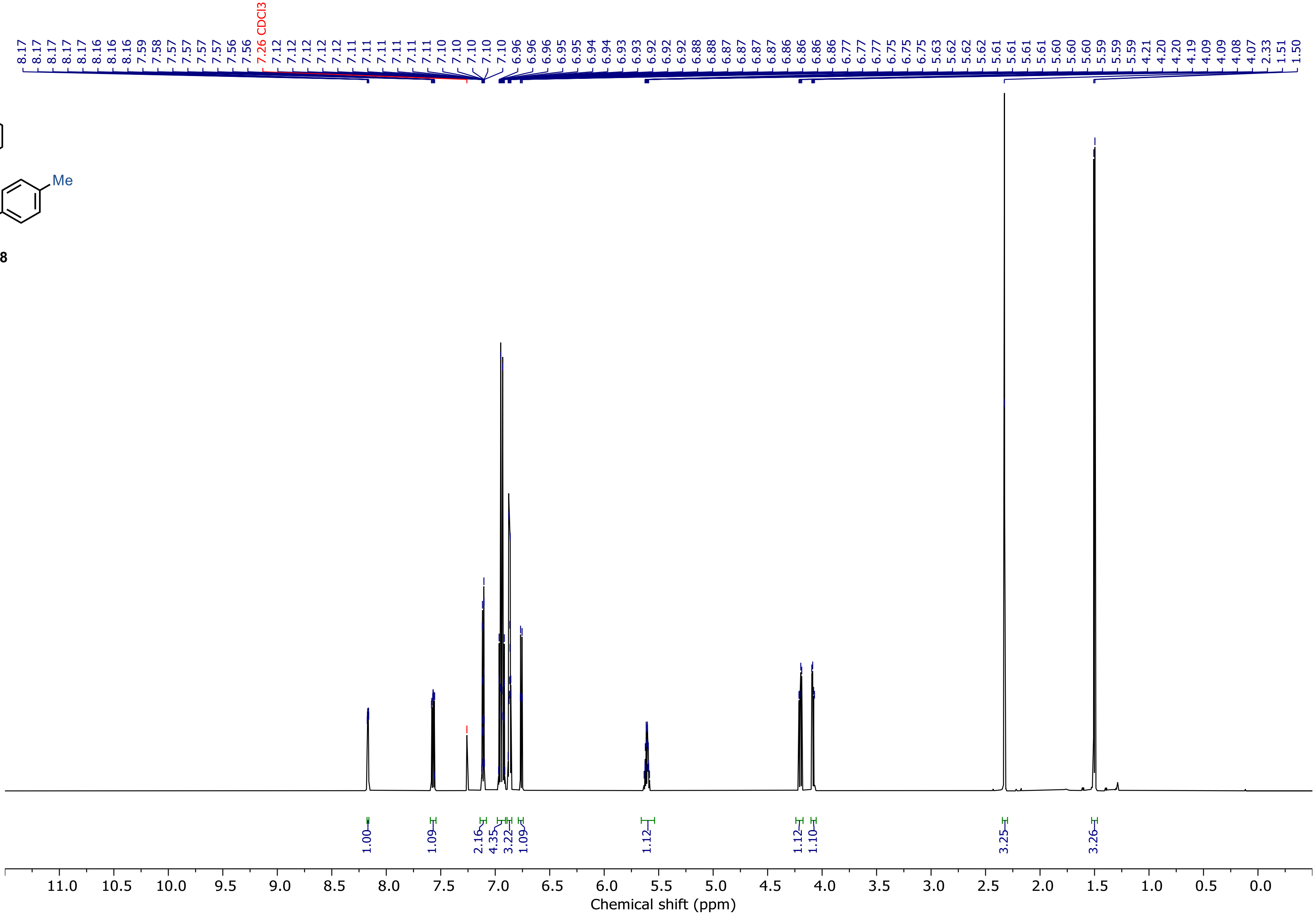

^{13}C NMR of 88

$CDCl_3$, 151 MHz, 23 ºC.

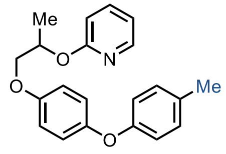

(±)-88

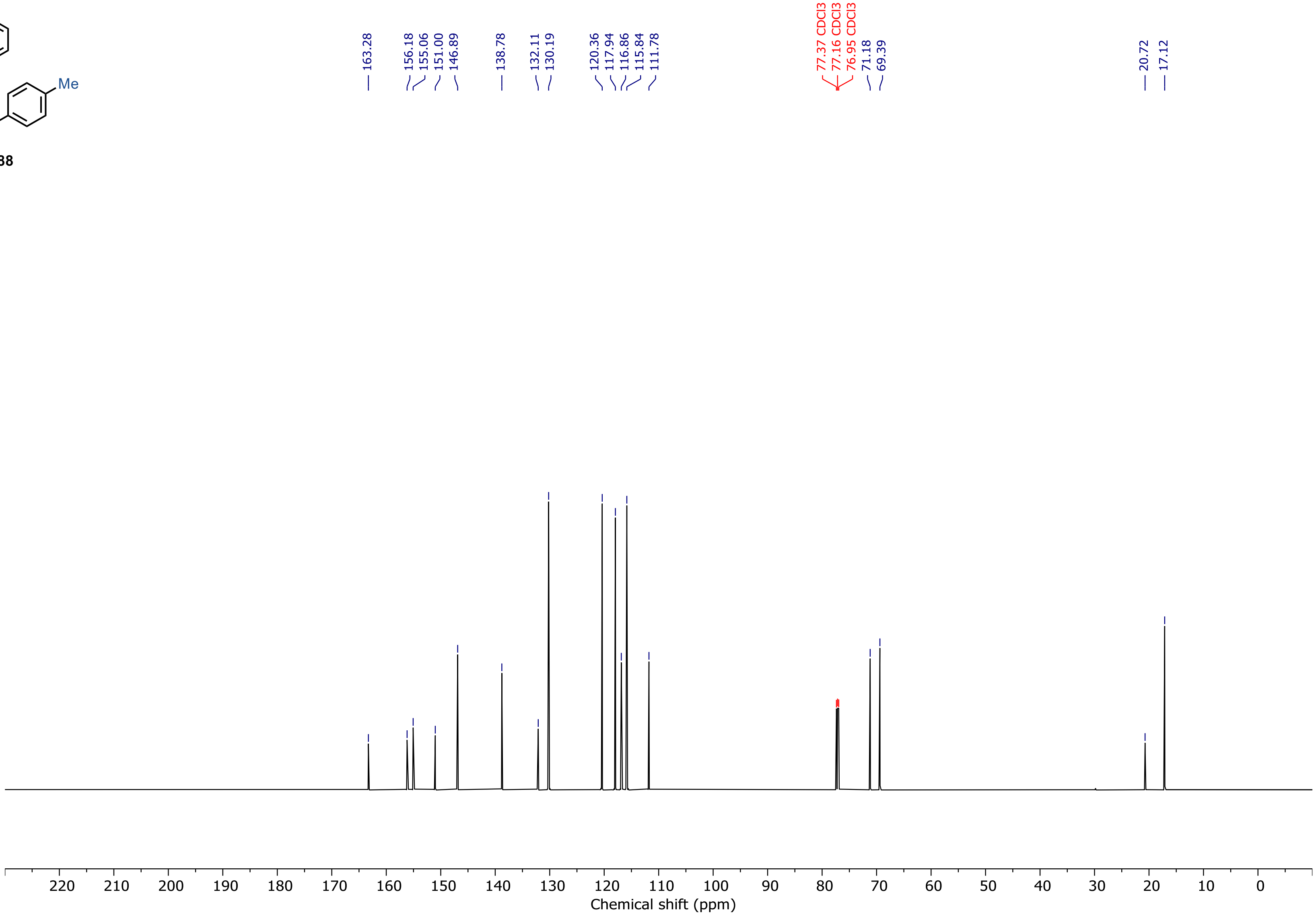

^{1}H NMR of 89

CDCl$_3$, 600 MHz, 23 ºC.

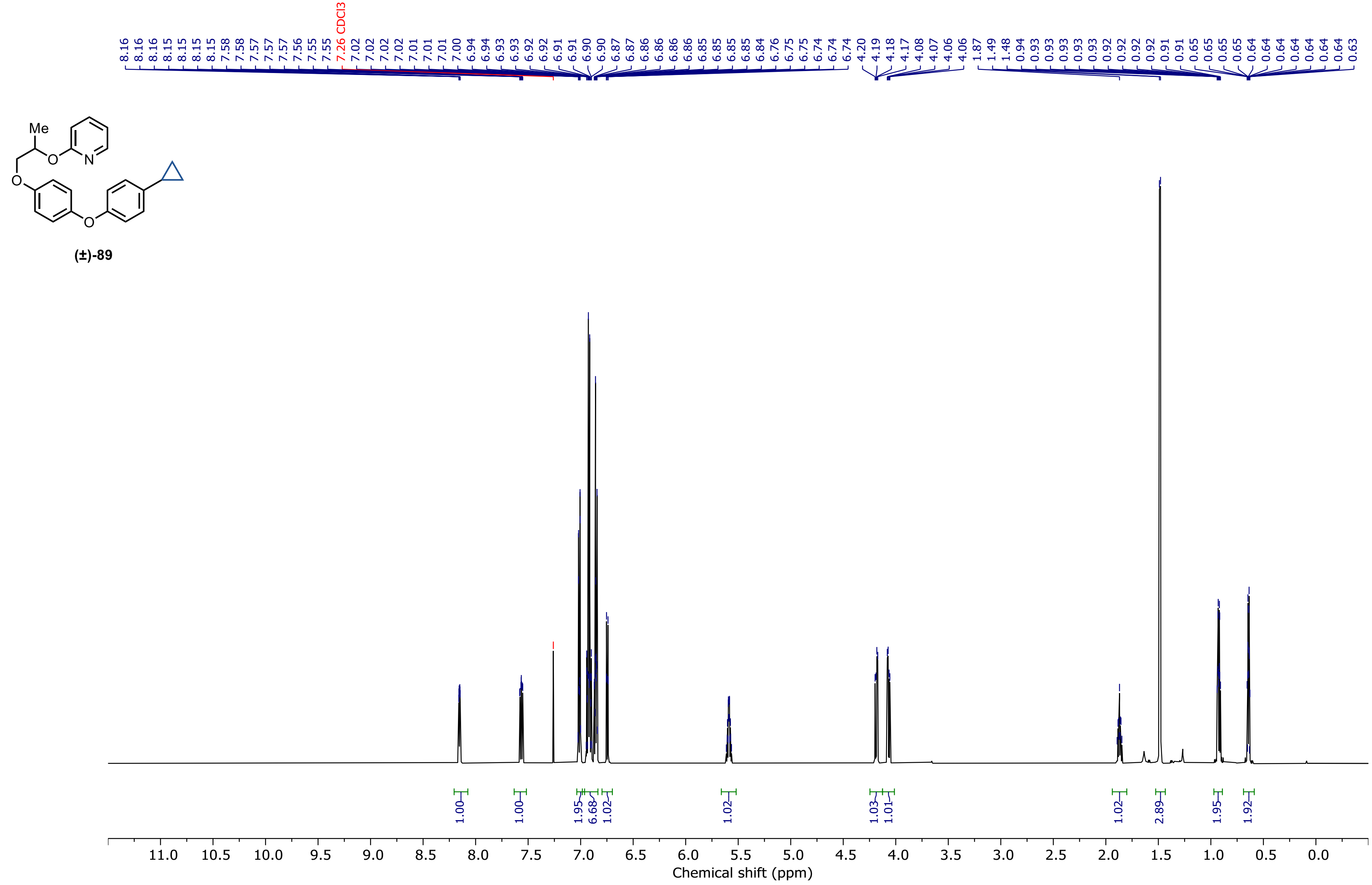

^{13}C NMR of 89

$CDCl_3$, 151 MHz, 23 ºC.

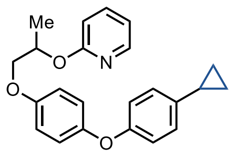

(±)-89

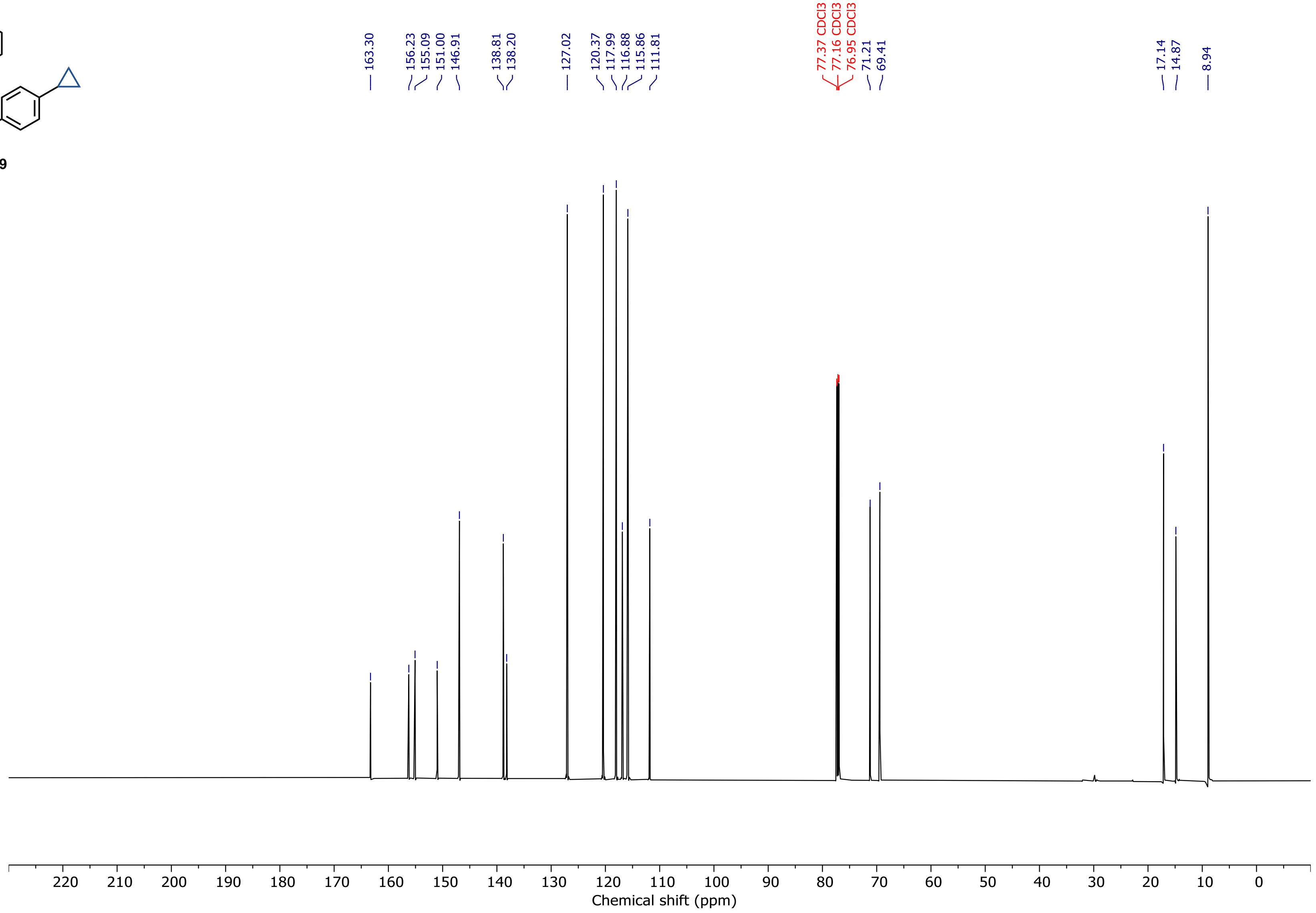

^{1}H NMR of 90

$CDCl_3$, 500 MHz, 23 ºC.

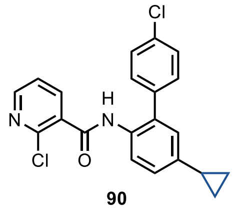

90

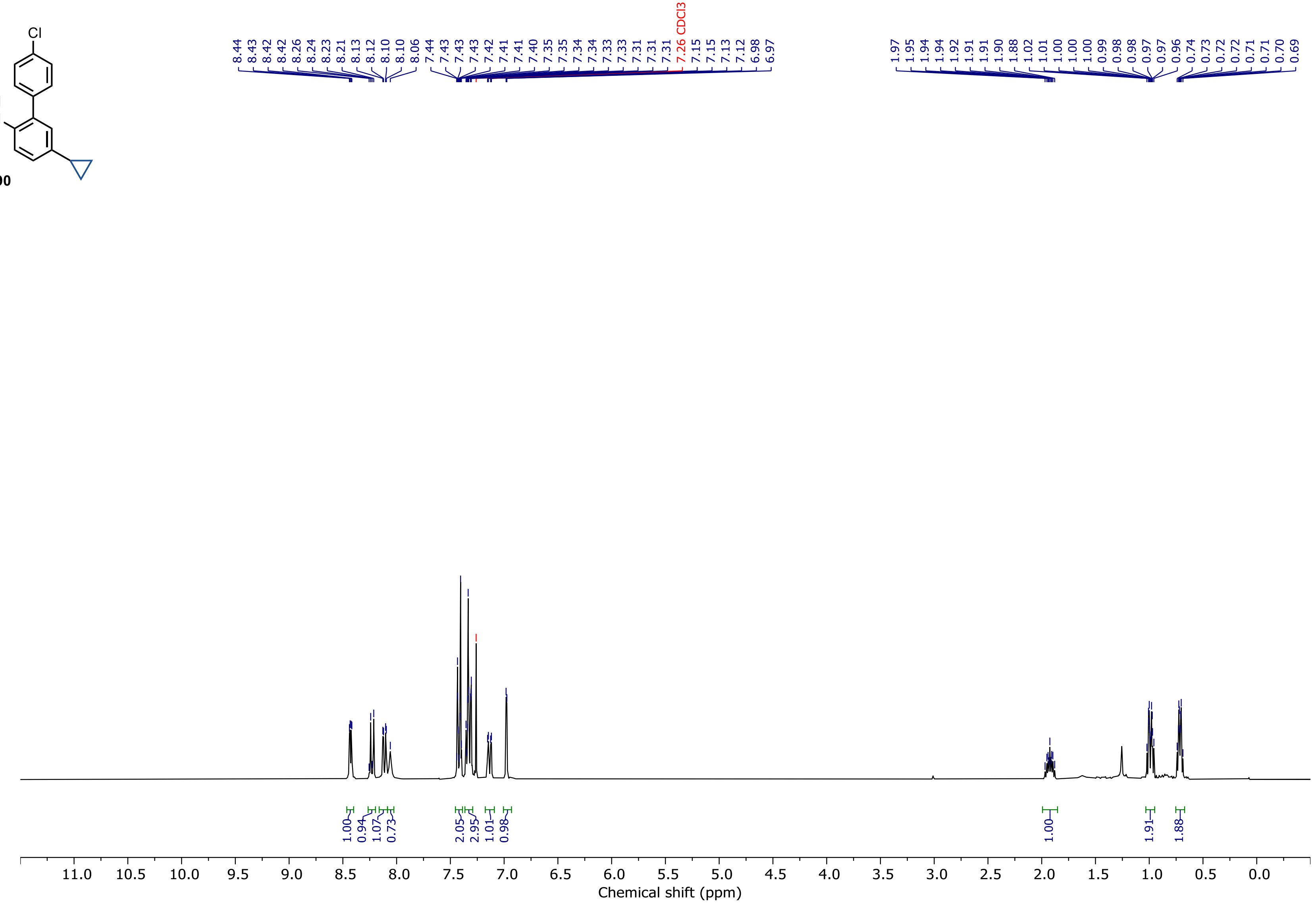

^{13}C NMR of 90

$CDCl_3$, 126 MHz, 23 ºC.

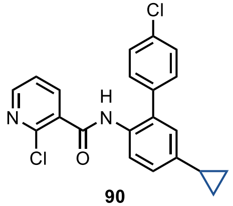

90

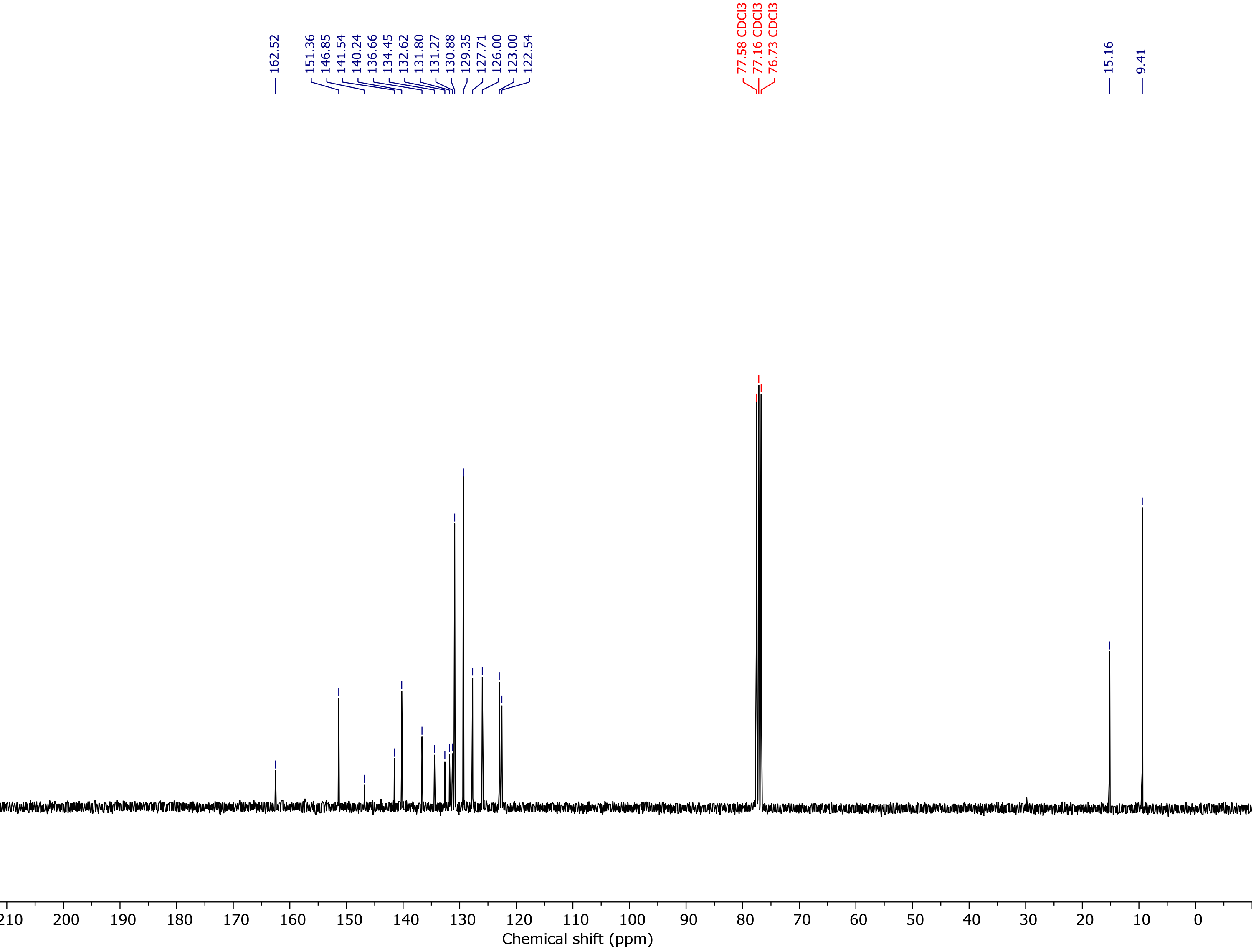

^{1}H NMR of 91

$CDCl_3$, 500 MHz, 23 ºC.

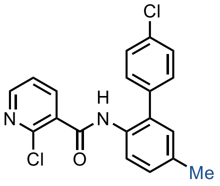

91

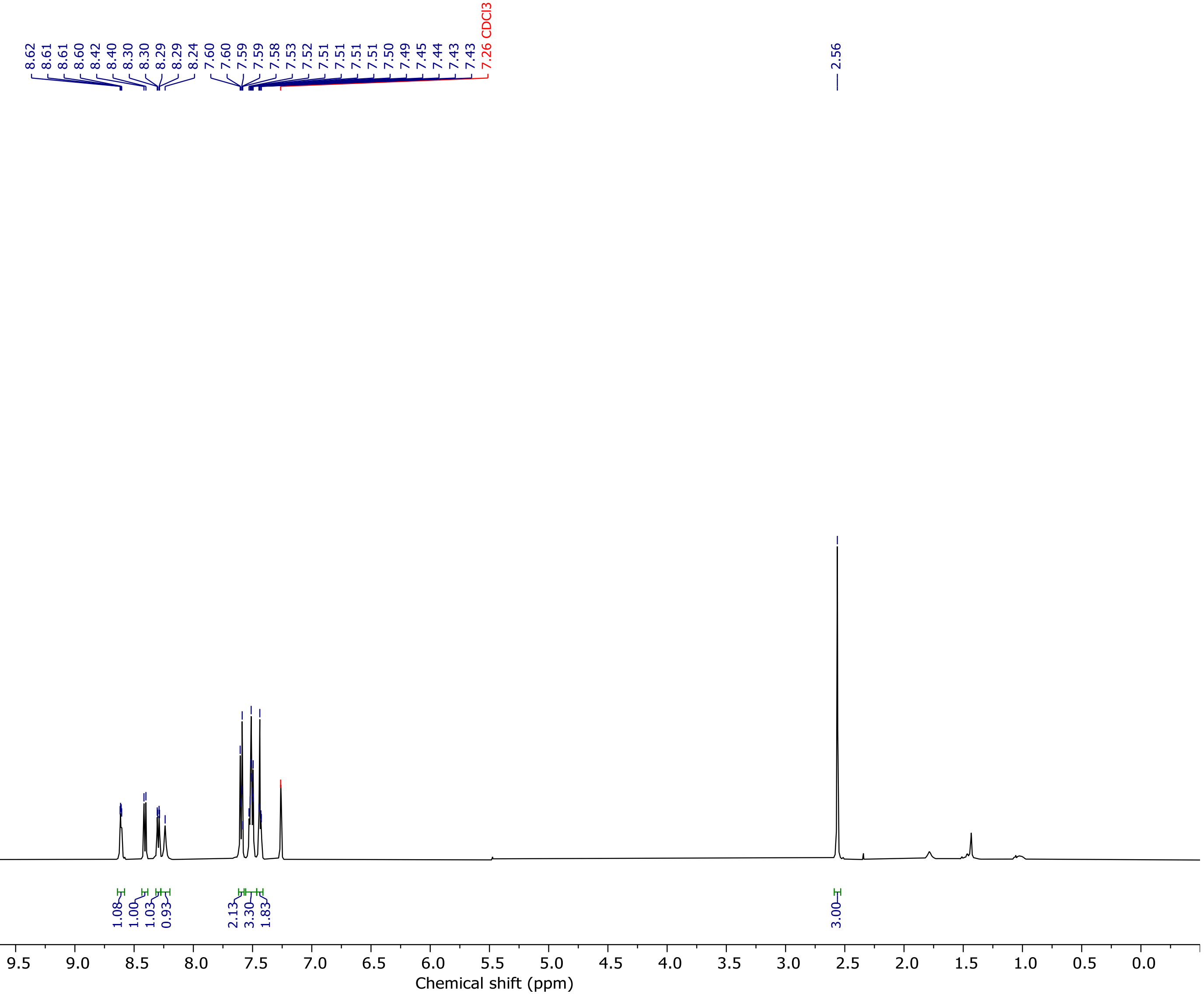

^{13}C NMR of 91

$CDCl_3$, 126 MHz, 23 ºC.

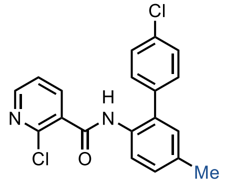

91

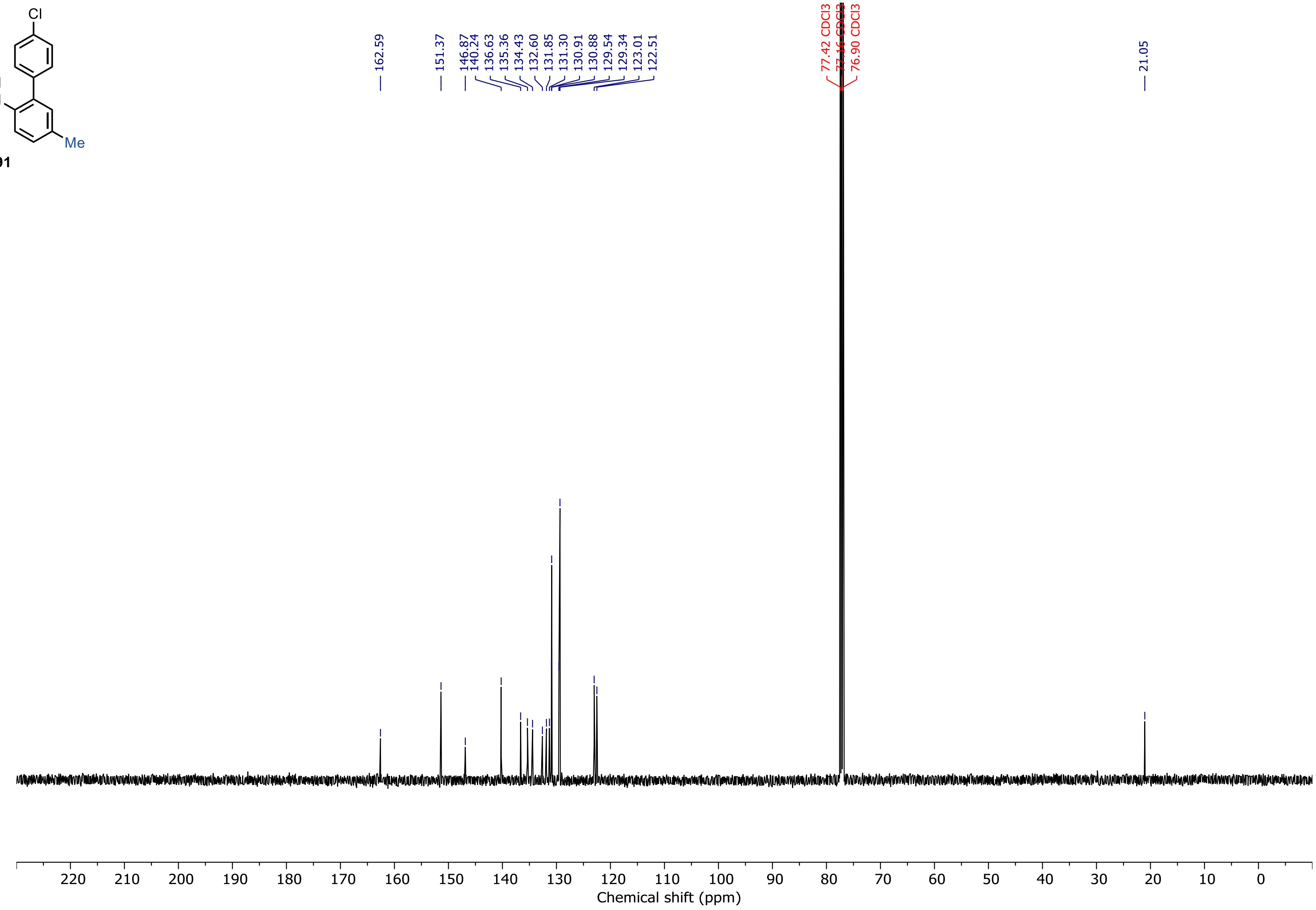

^{1}H NMR of 92

$CDCl_3$, 500 MHz, 23 °C.

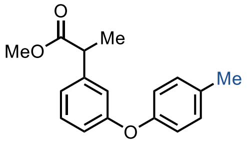

(±)-92

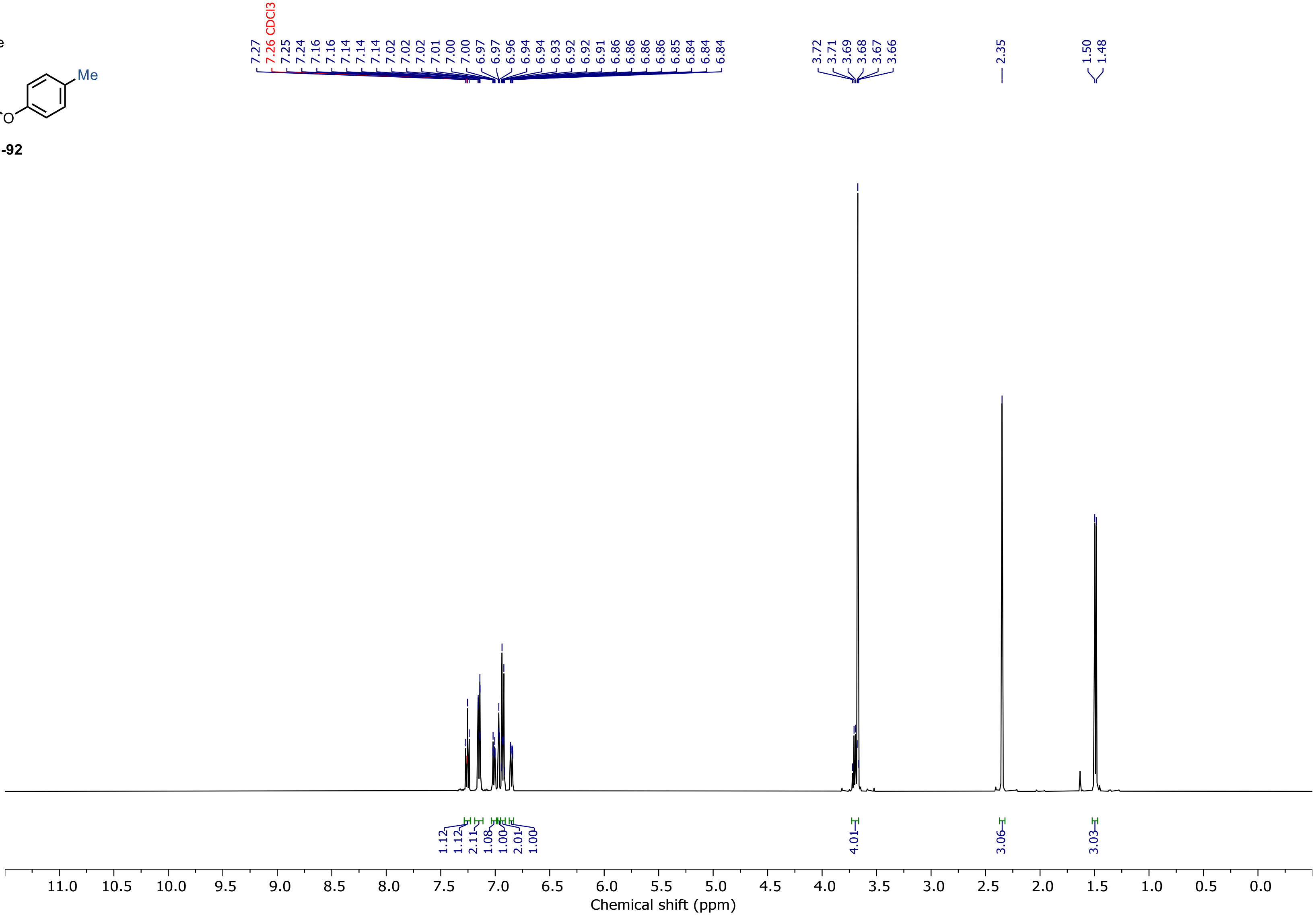

^{13}C NMR of 92

$CDCl_3$, 126 MHz, 23 ºC.

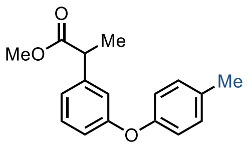

(±)-92

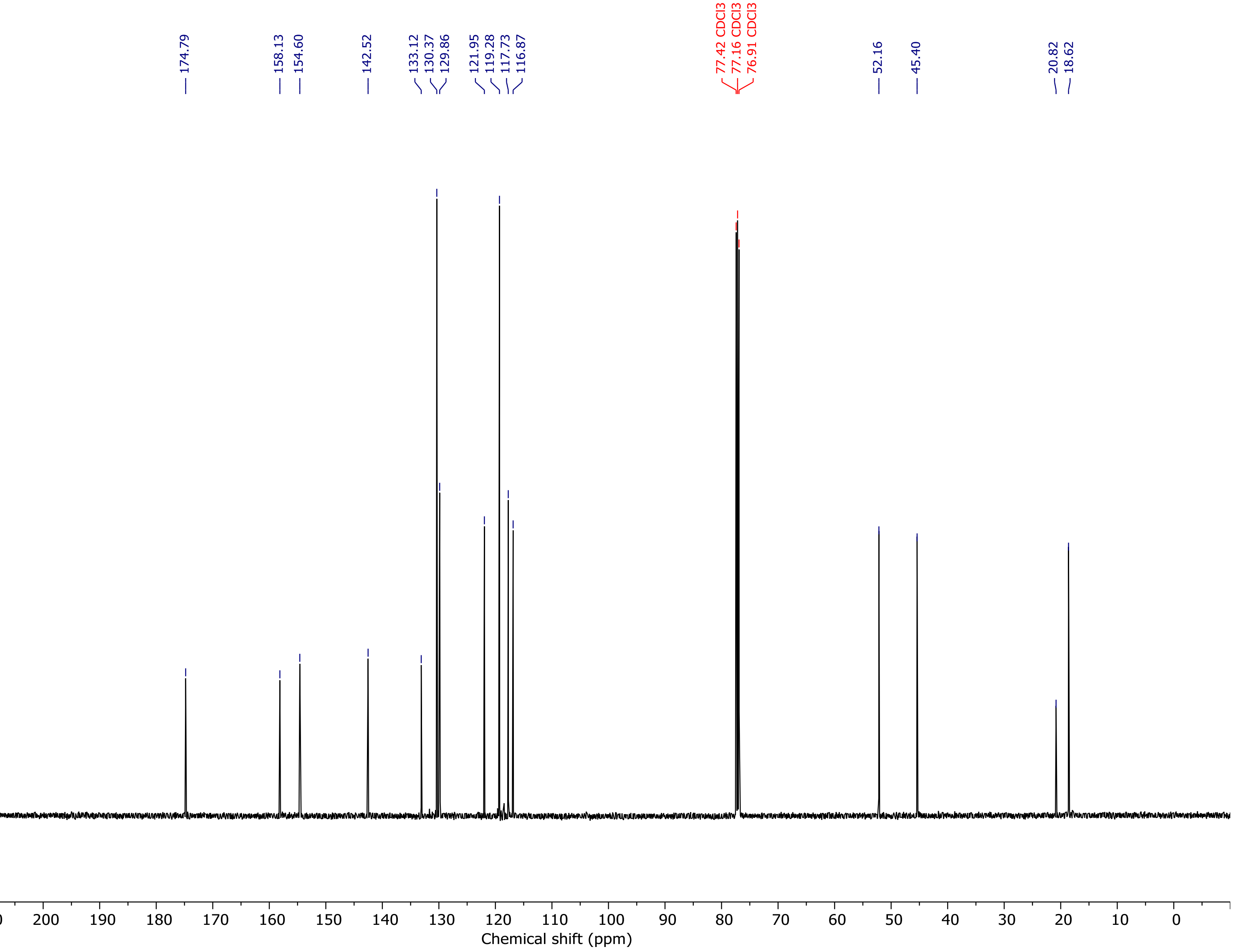

^{1}H NMR of 93

$CDCl_3$, 600 MHz, 23 ºC.

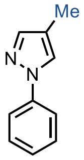

93

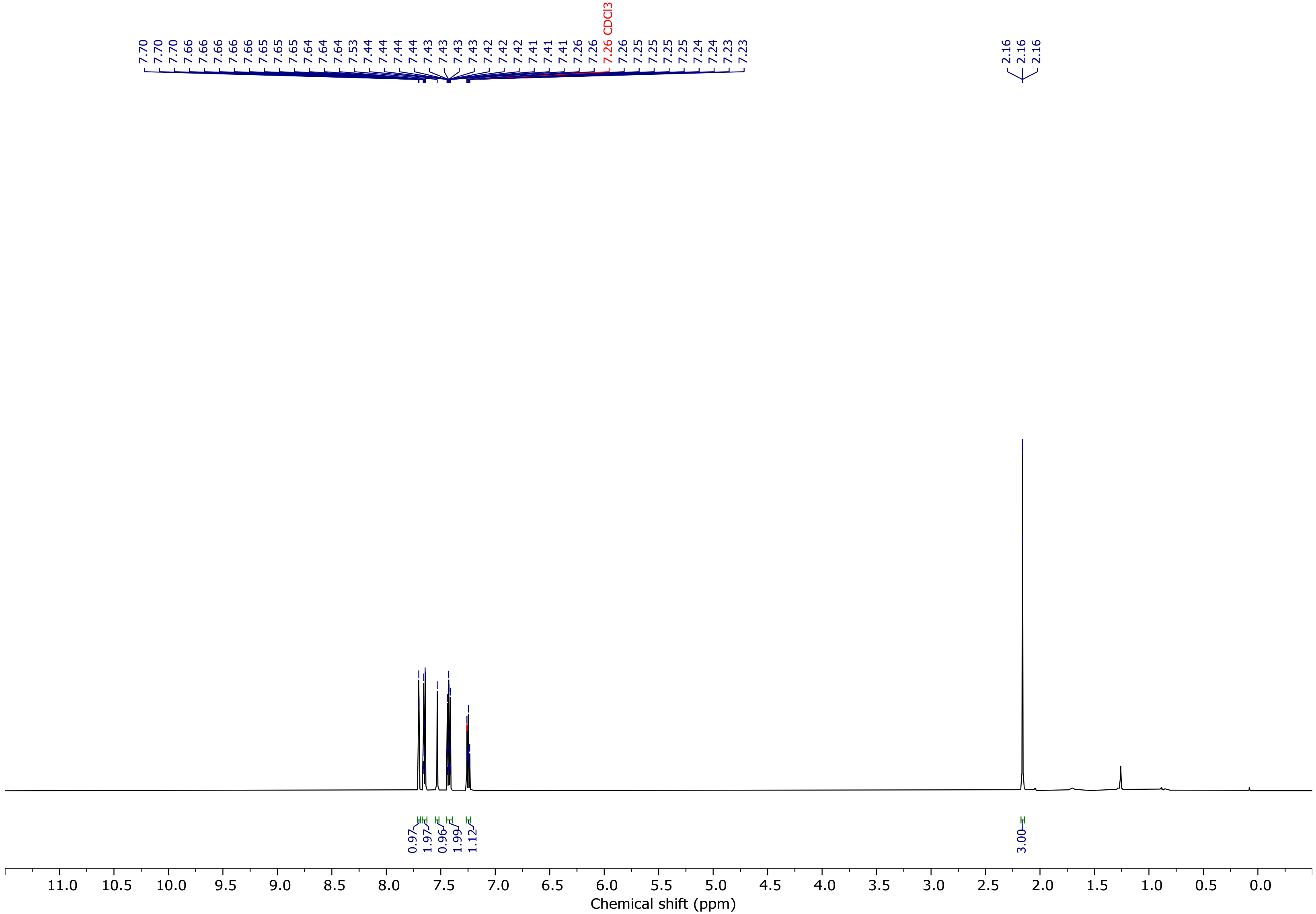

^{13}C NMR of 93

$CDCl_3$, 151 MHz, 23 ºC.

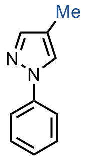

93

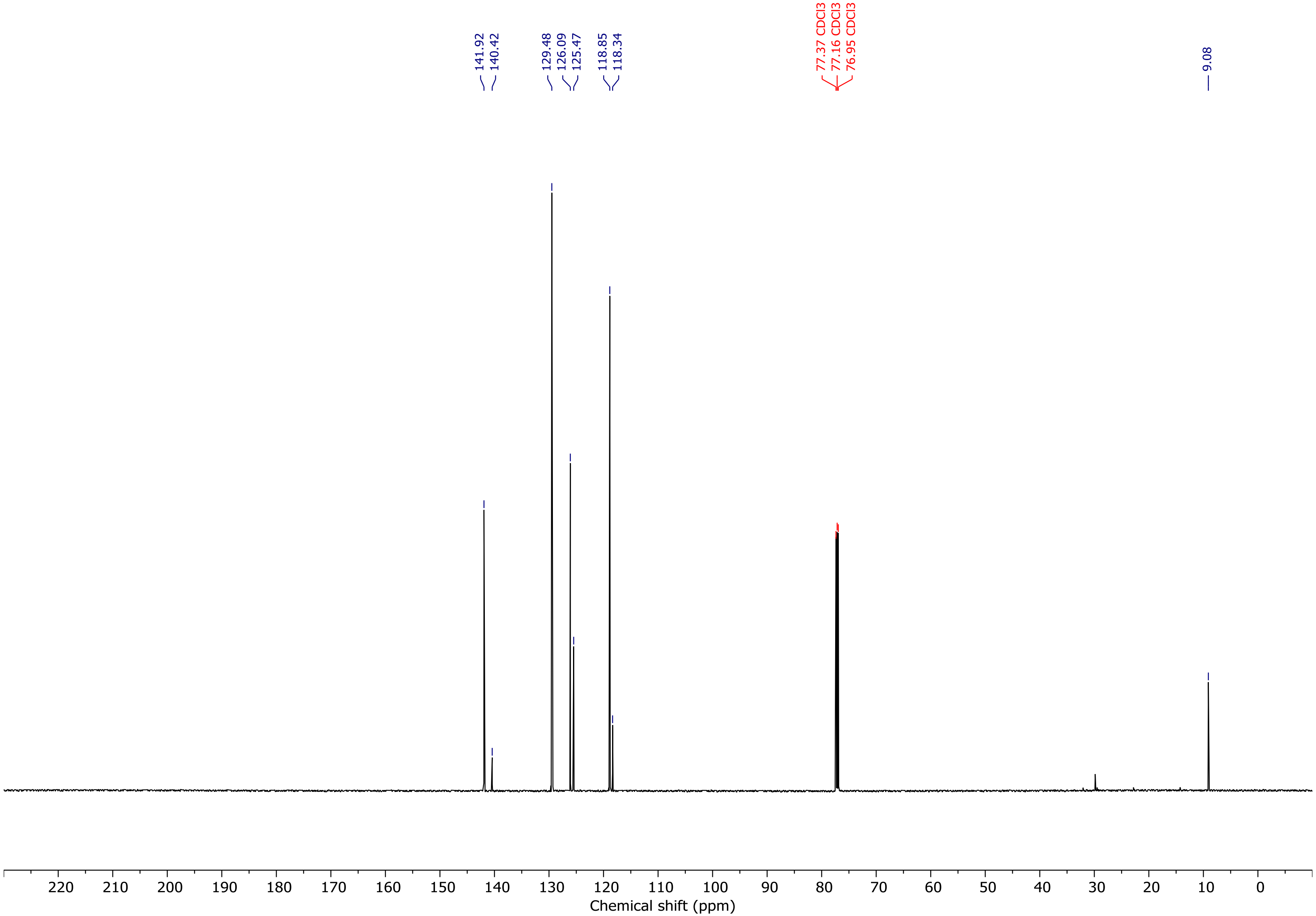

^{1}H NMR of 94

CDCl3, 600 MHz, 23 ºC.

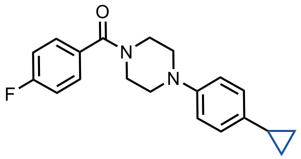

94

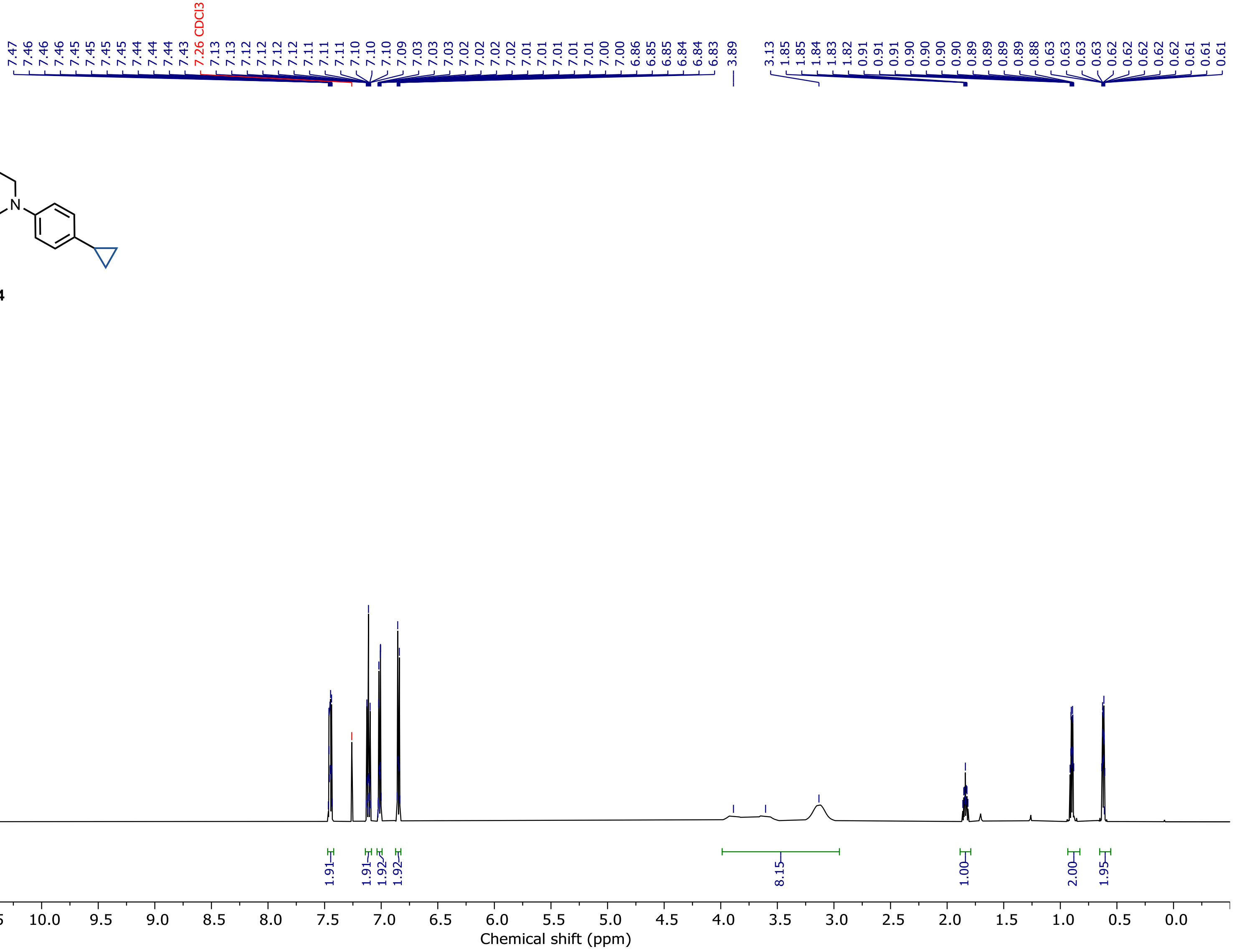

^{13}C NMR of 94

$CDCl_3$, 151 MHz, 23 ºC.

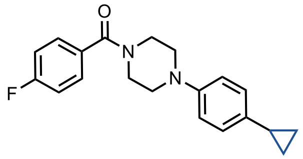

94

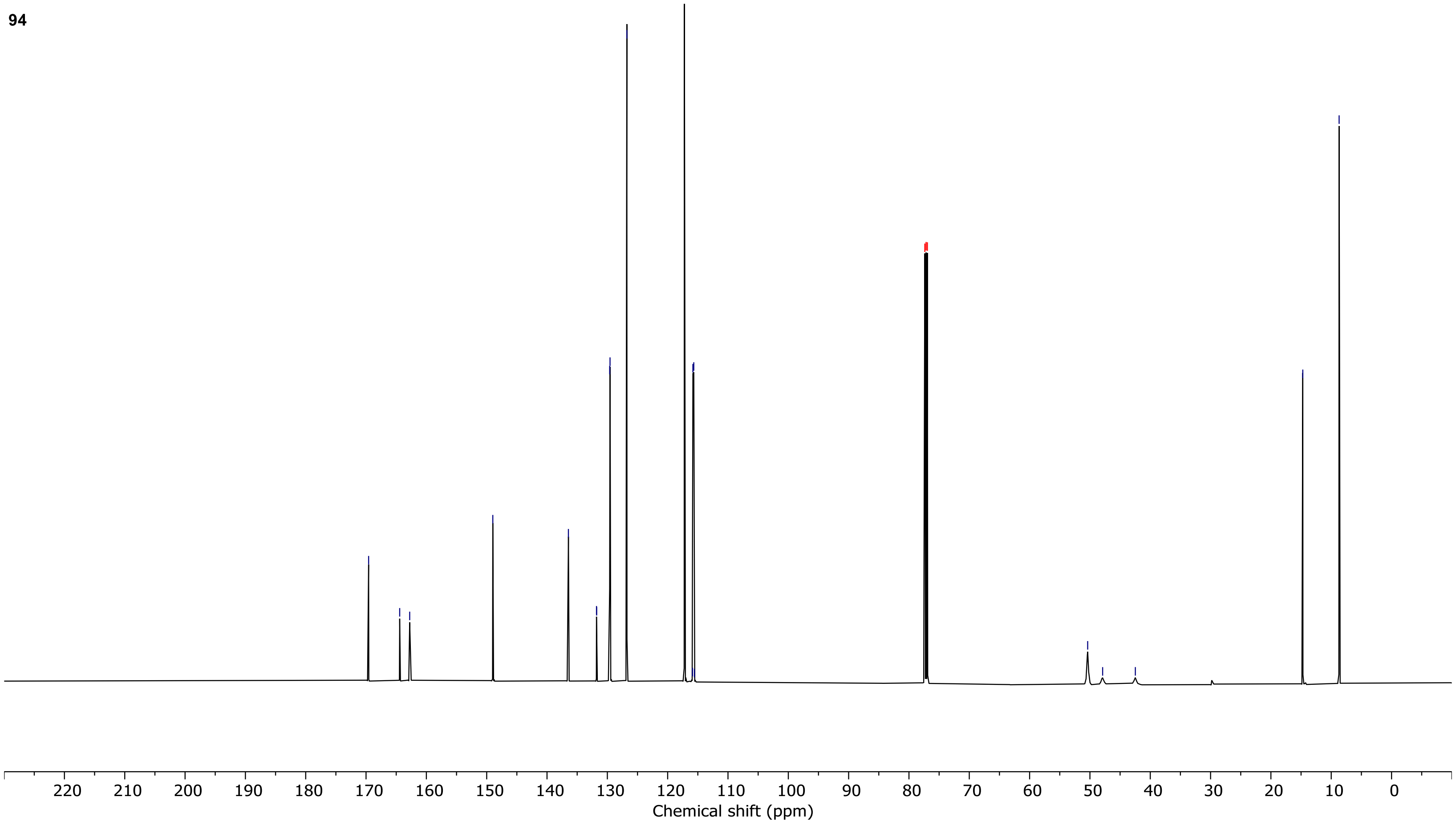

^{19}F NMR of 94

$CDCl_3$, 565 MHz, 23 ºC.

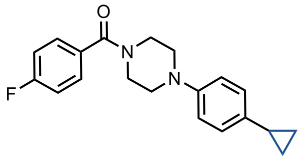

94

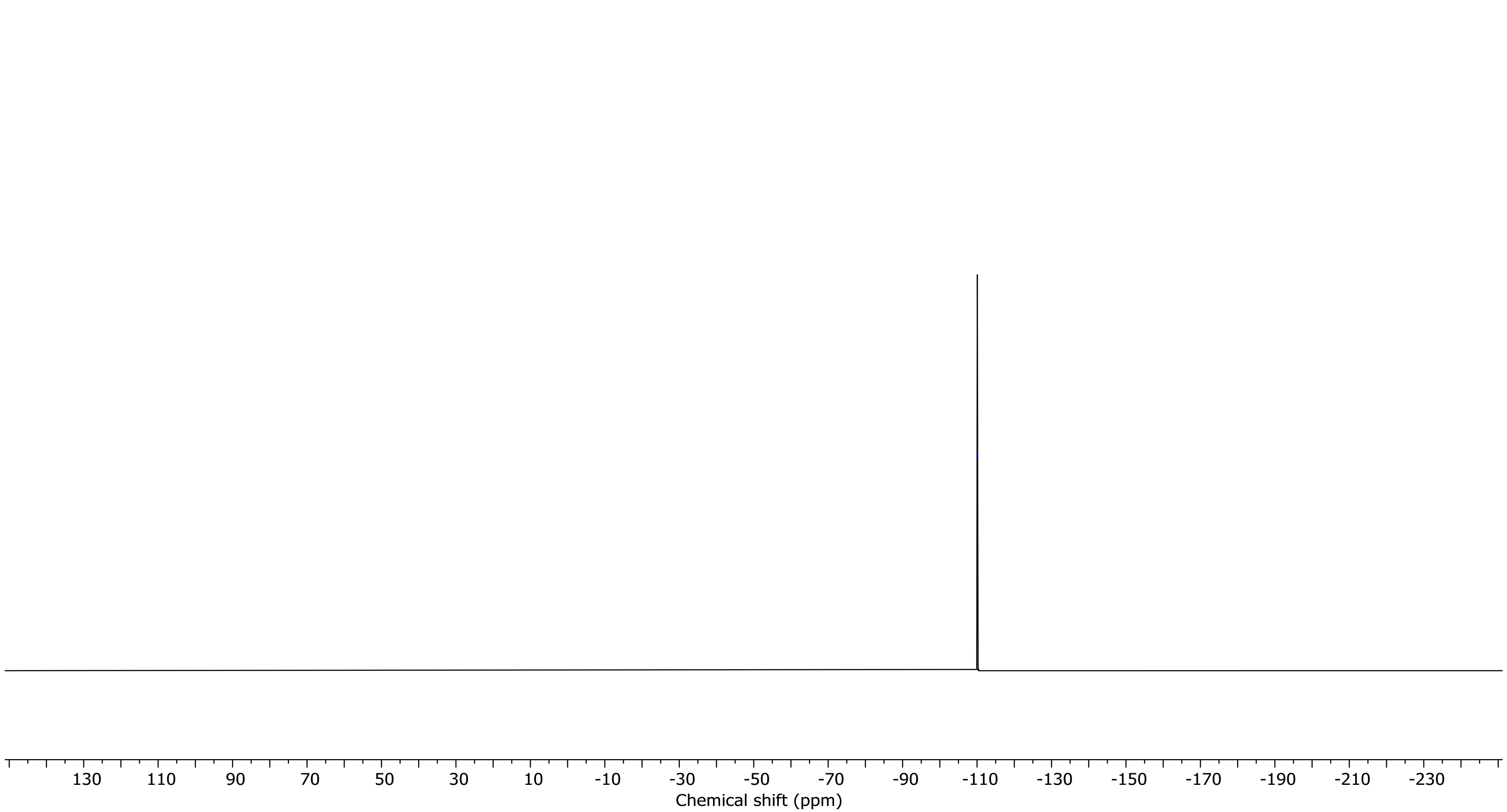

^{1}H NMR of 95

$CDCl_3$, 600 MHz, 23 ºC.

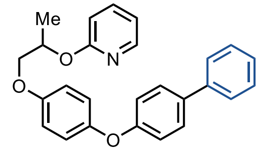

95

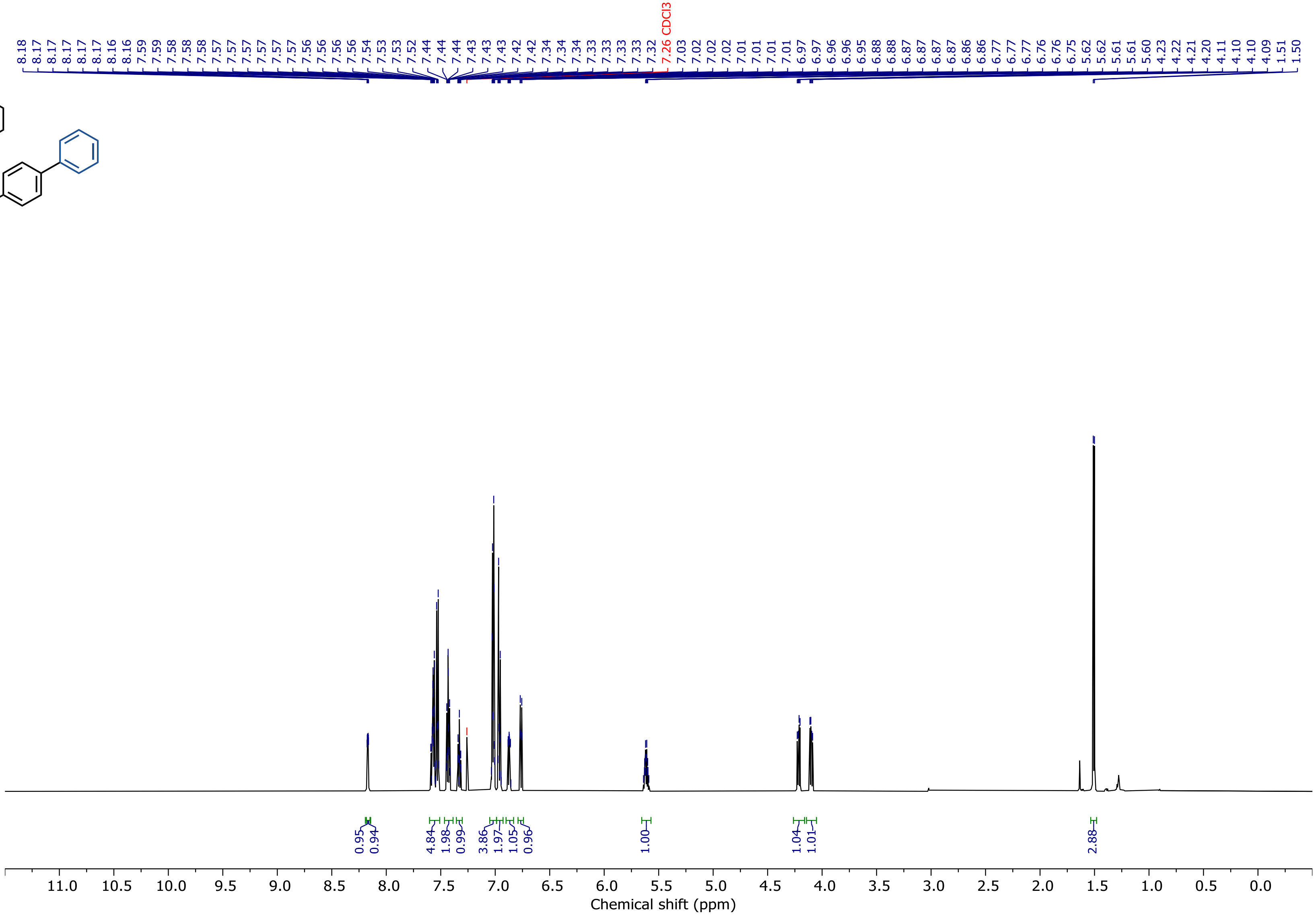

^{13}C NMR of 95

$CDCl_3$, 151 MHz, 23 ºC.

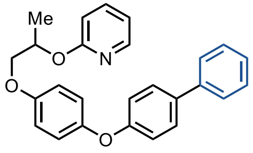

95

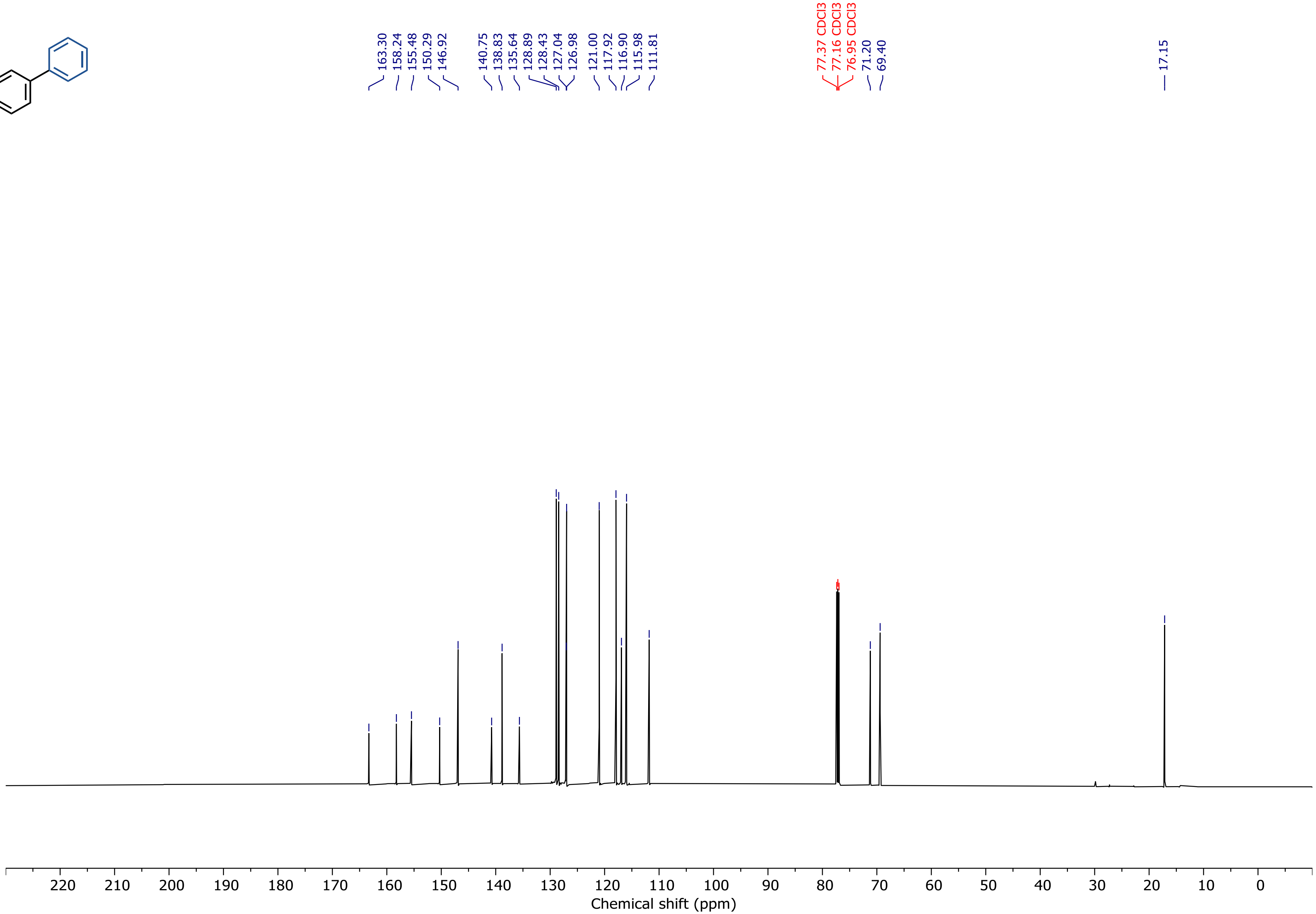

^{1}H NMR of 96

$CDCl_3$, 600 MHz, 23 ºC.

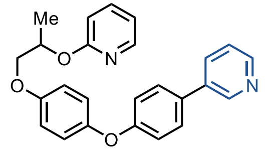

96

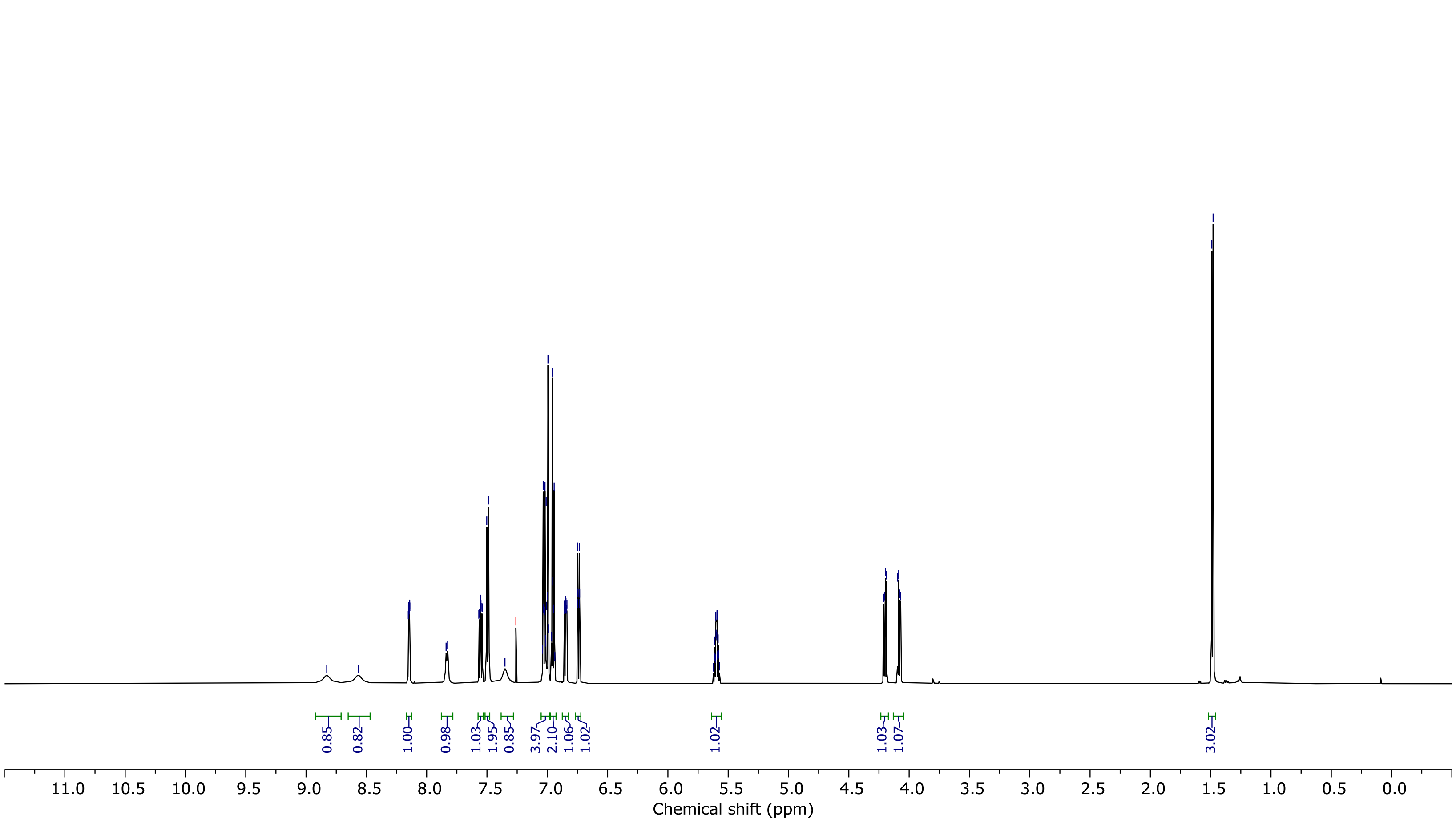

^{13}C NMR of 96

$CDCl_3$, 151 MHz, 23 ºC.

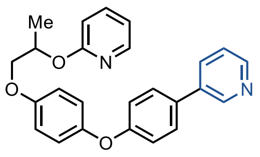

96

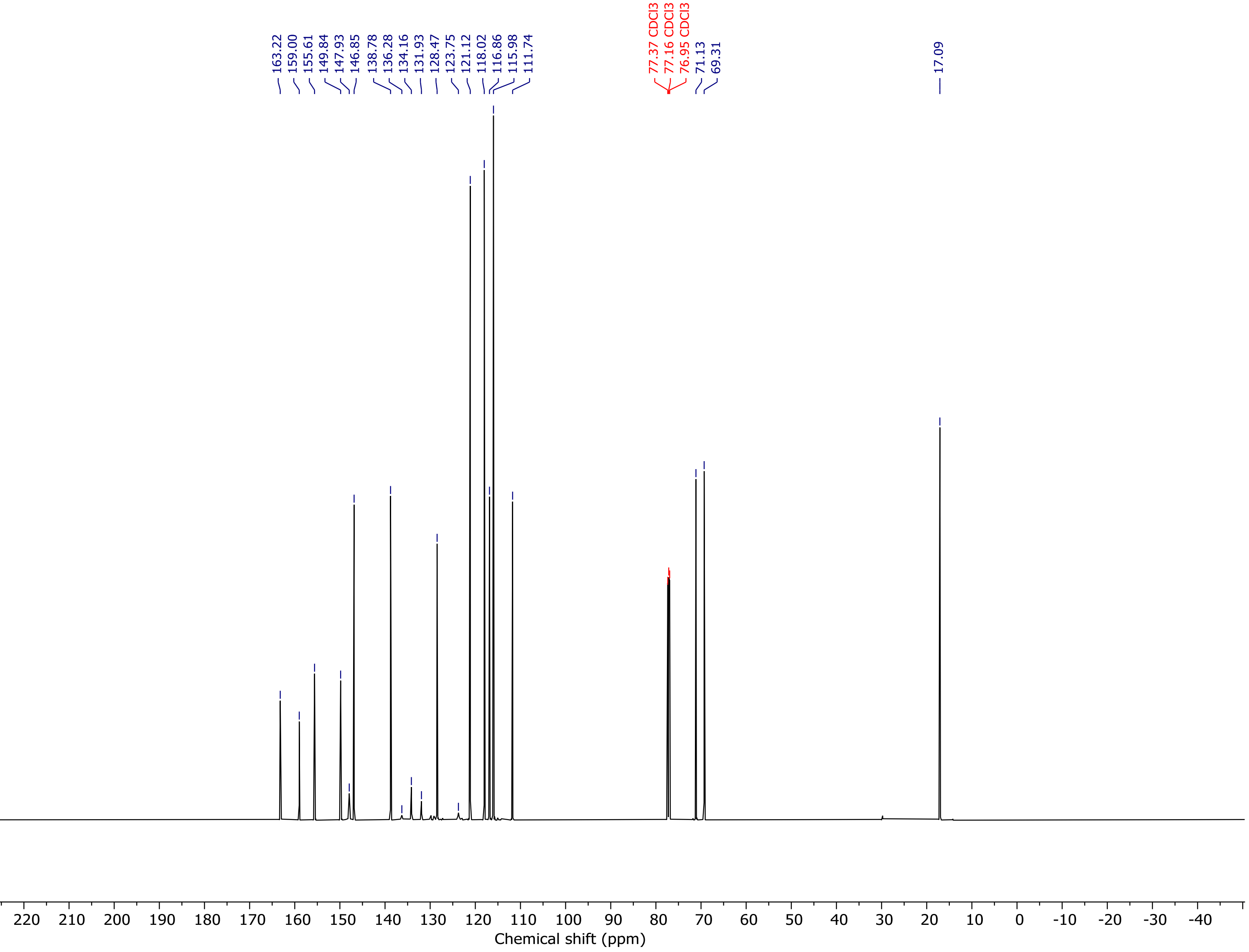

^{1}H NMR of 97

CDCl$_3$, 500 MHz, 23 ºC.

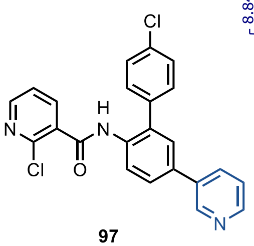

97

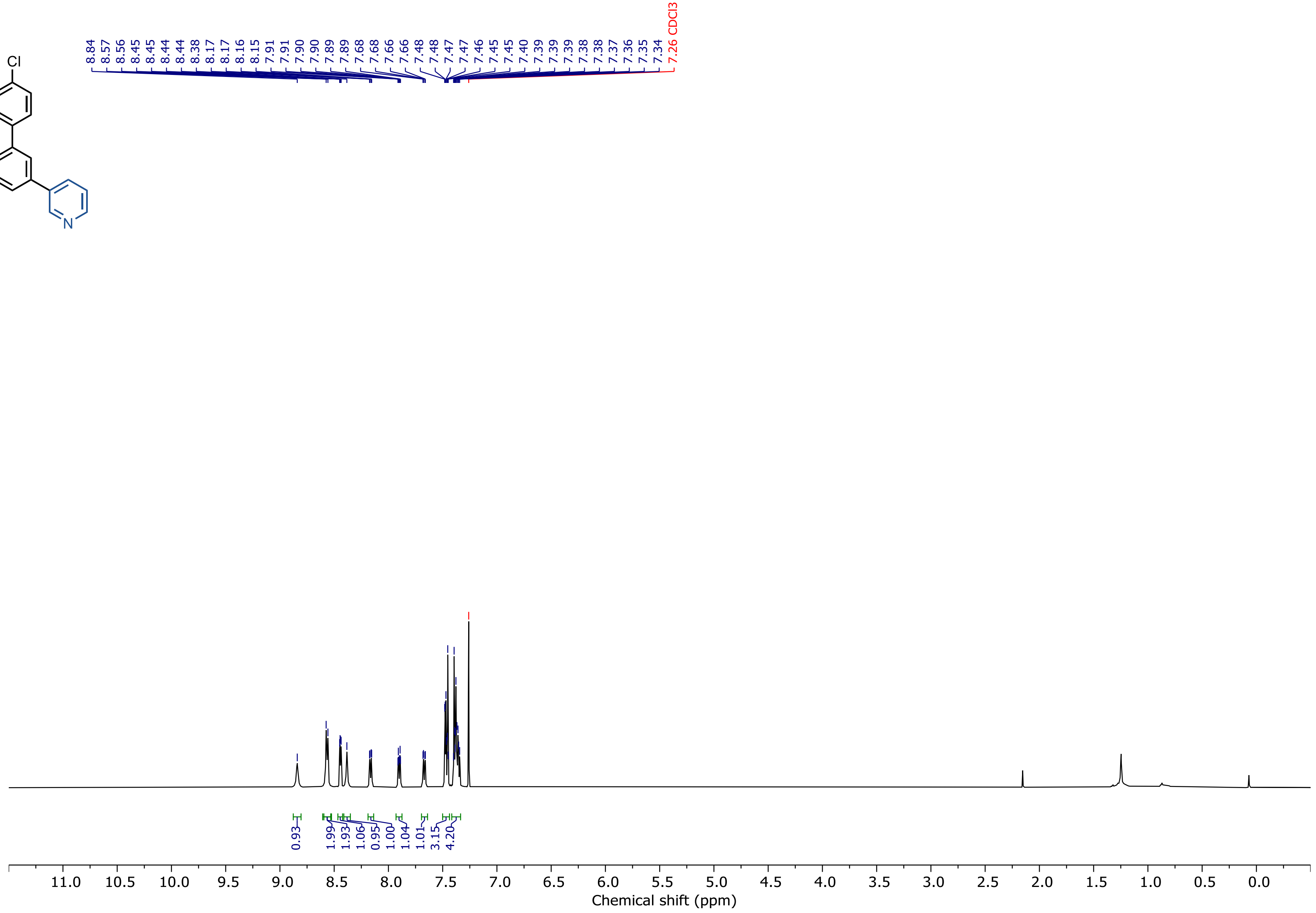

^{13}C NMR of 97

$CDCl_3$, 126 MHz, 23 ºC.

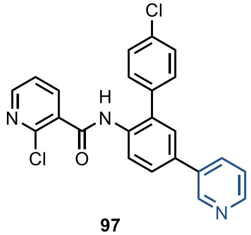

97

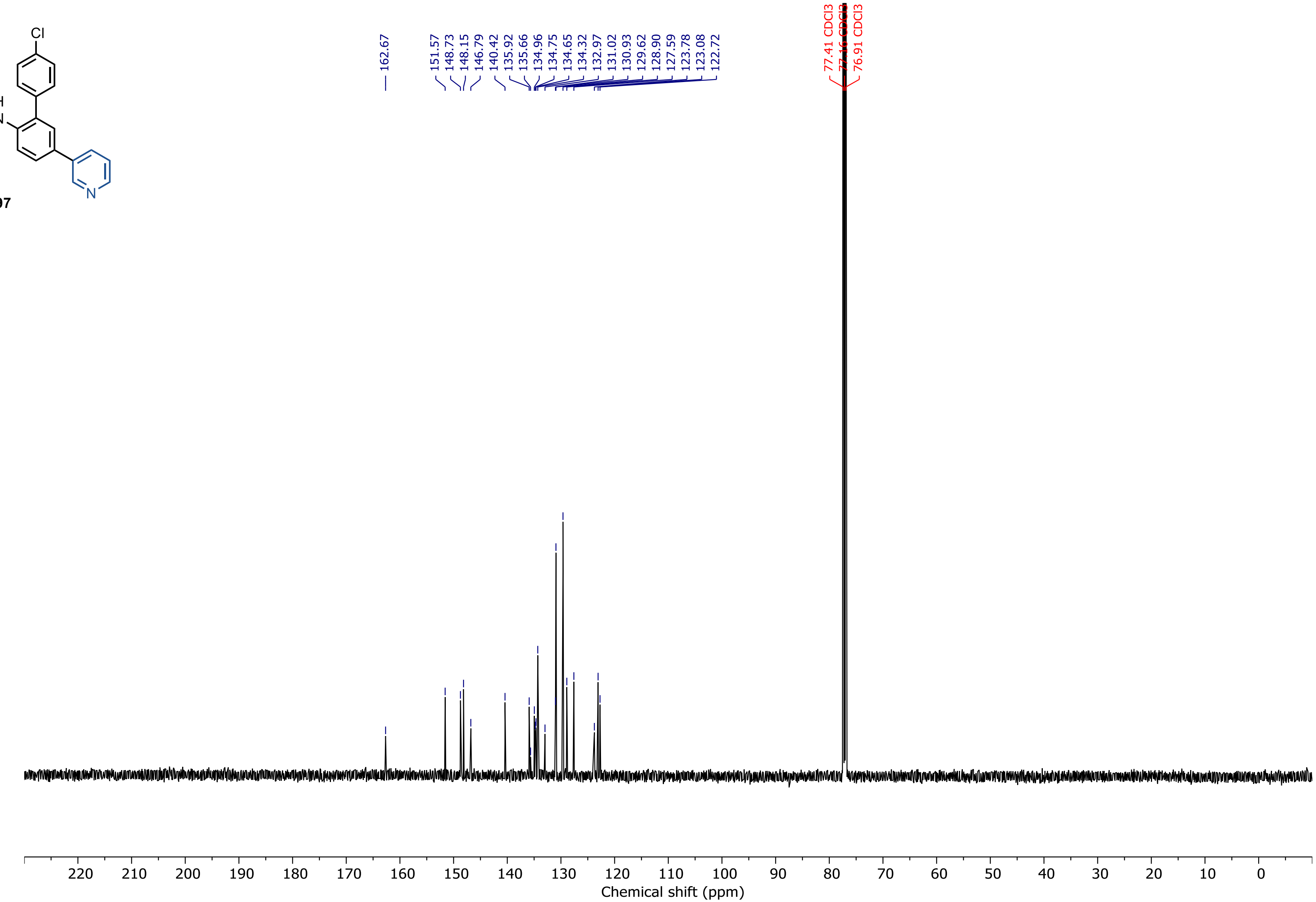

^{1}H NMR of 98

$CDCl_3$, 600 MHz, 23 ºC.

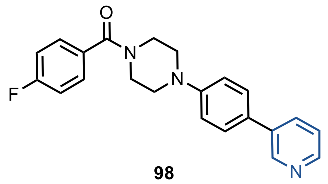

98

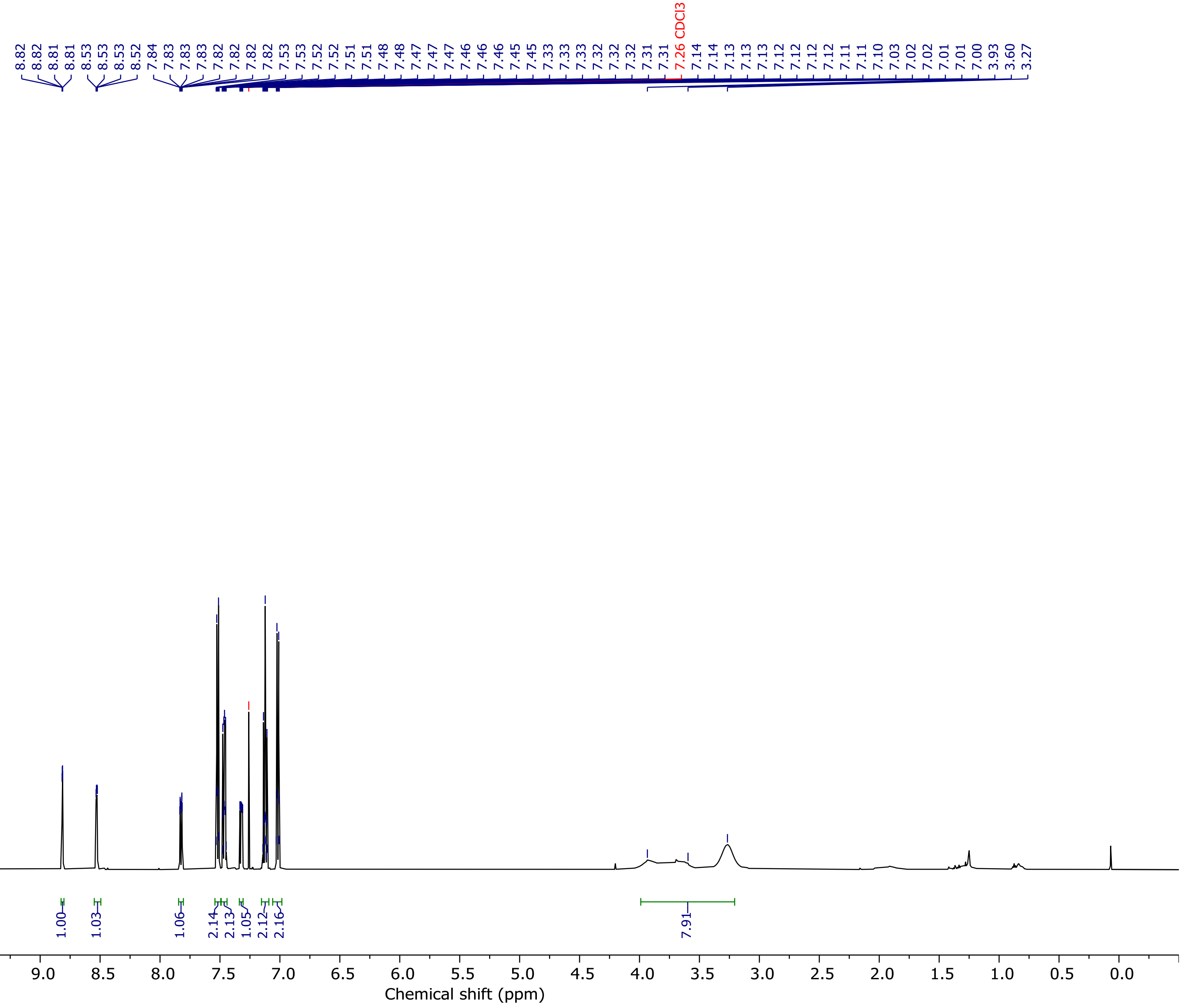

^{13}C NMR of 98

$CDCl_3$, 151 MHz, 23 ºC.

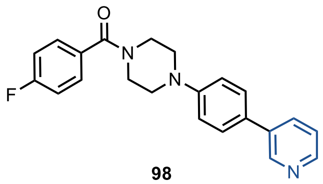

98

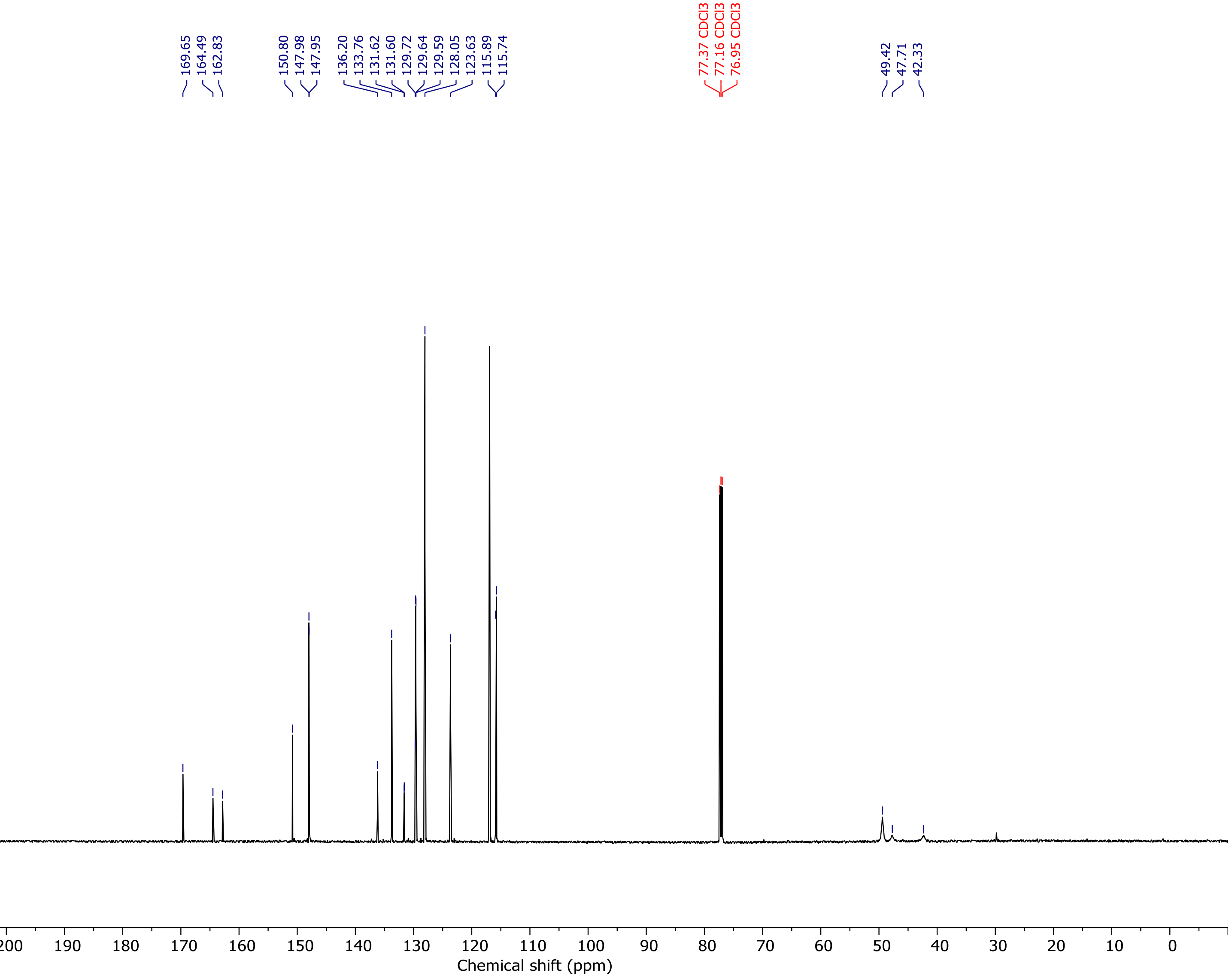

^{19}F NMR of 98

$CDCl_3$, 565 MHz, 23 ºC.

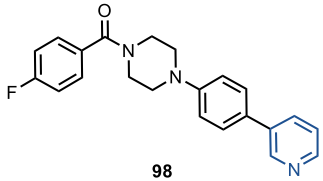

98

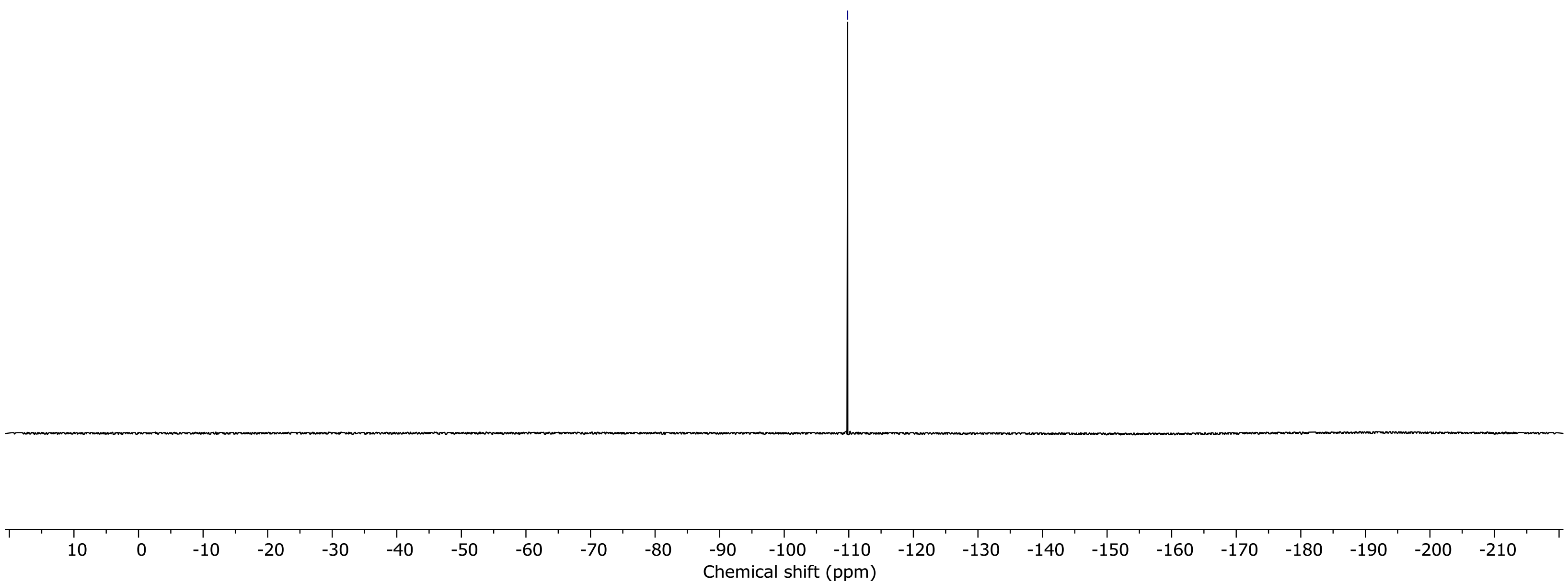

^{1}H NMR of 99

$CDCl_3$, 600 MHz, 23 ºC.

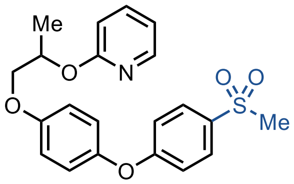

99

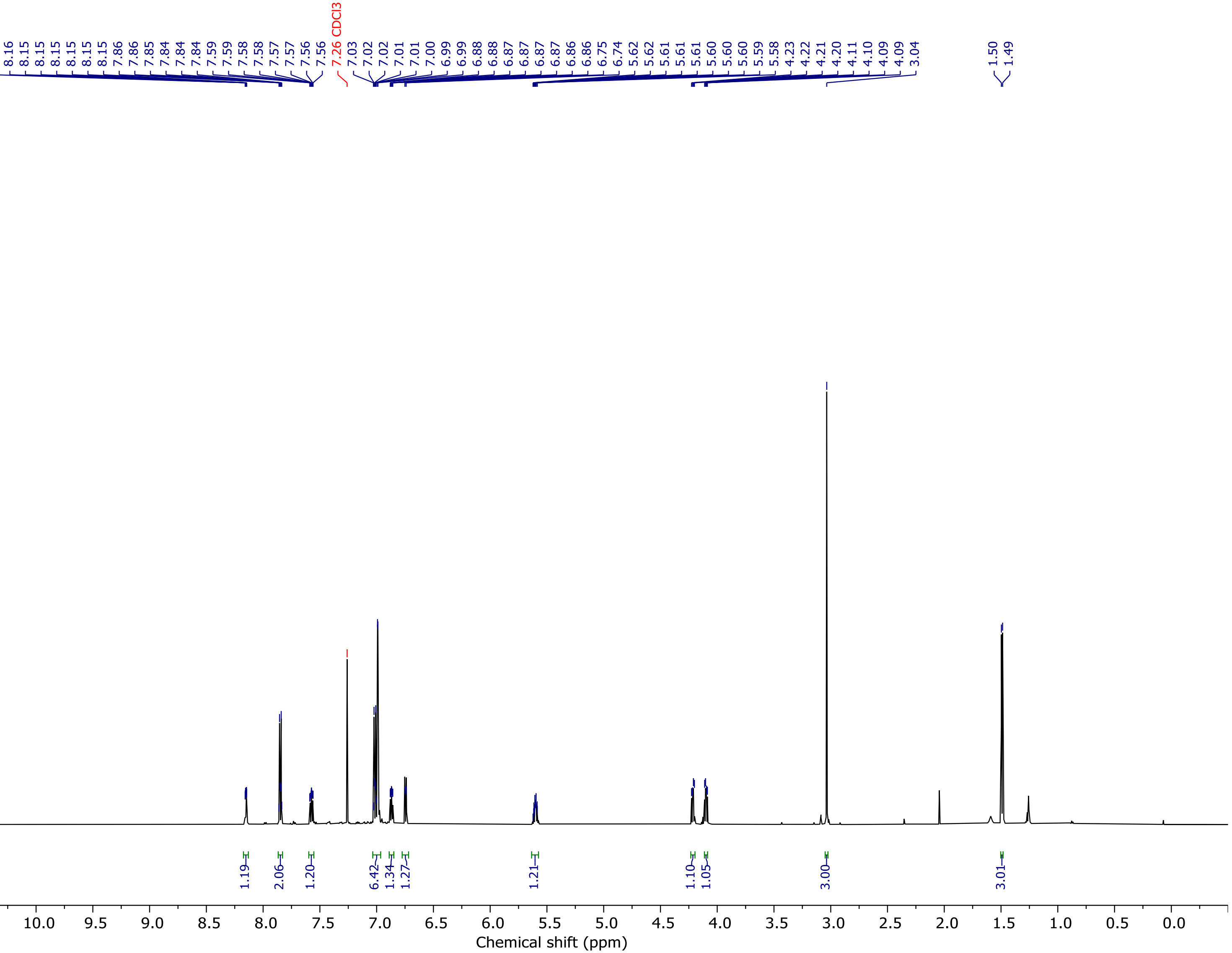

^{13}C NMR of 99

$CDCl_3$, 151 MHz, 23 ºC.

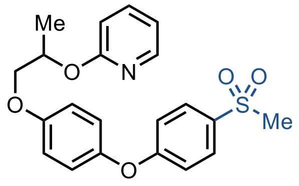

99

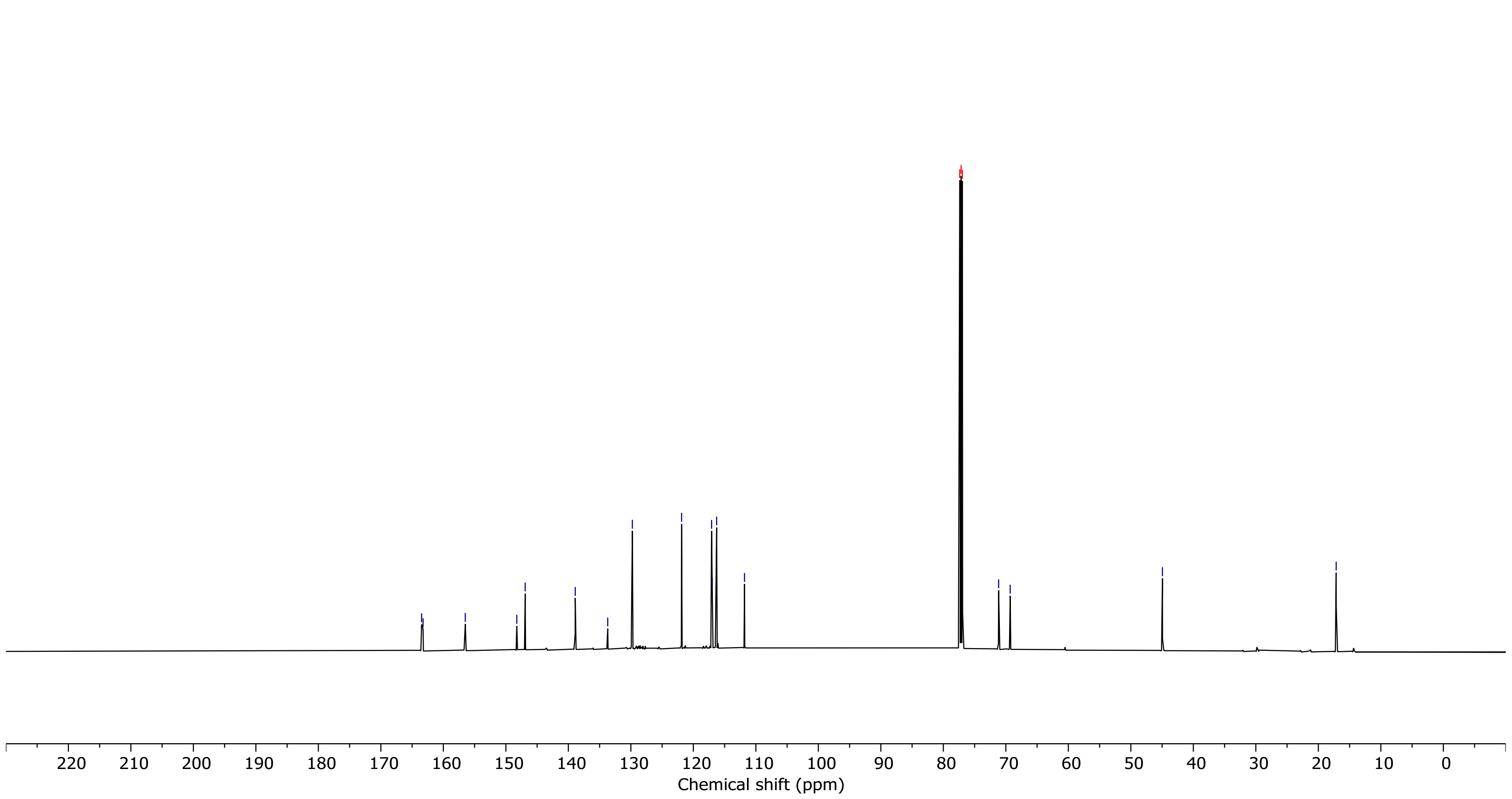

^{1}H NMR of 100

$CDCl_3$, 600 MHz, 23 ºC.

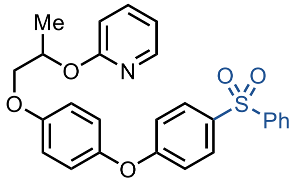

100

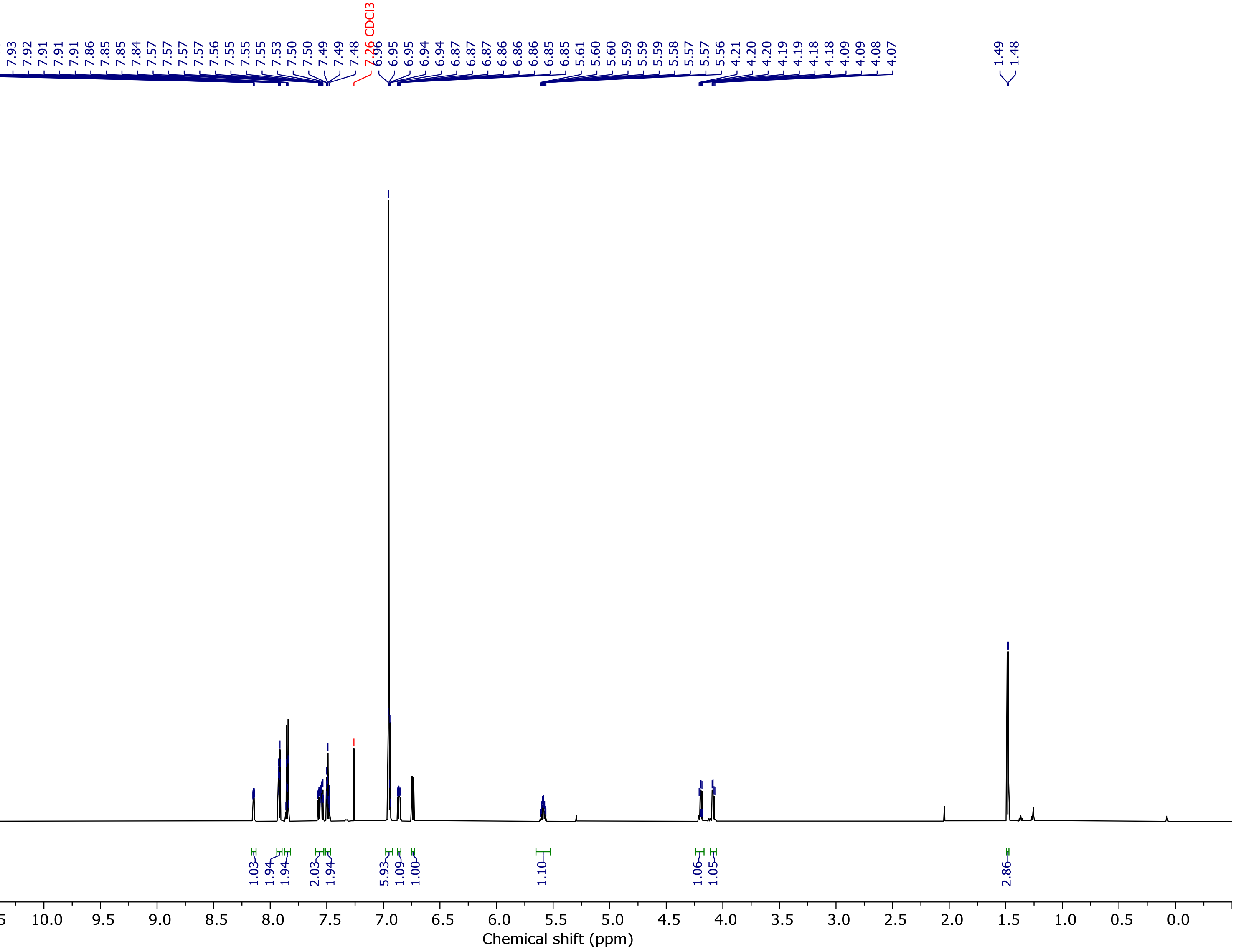

^{13}C NMR of 100

$CDCl_3$, 151 MHz, 23 ºC.

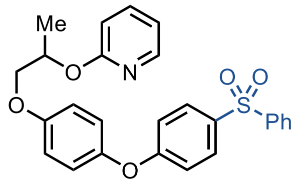

100

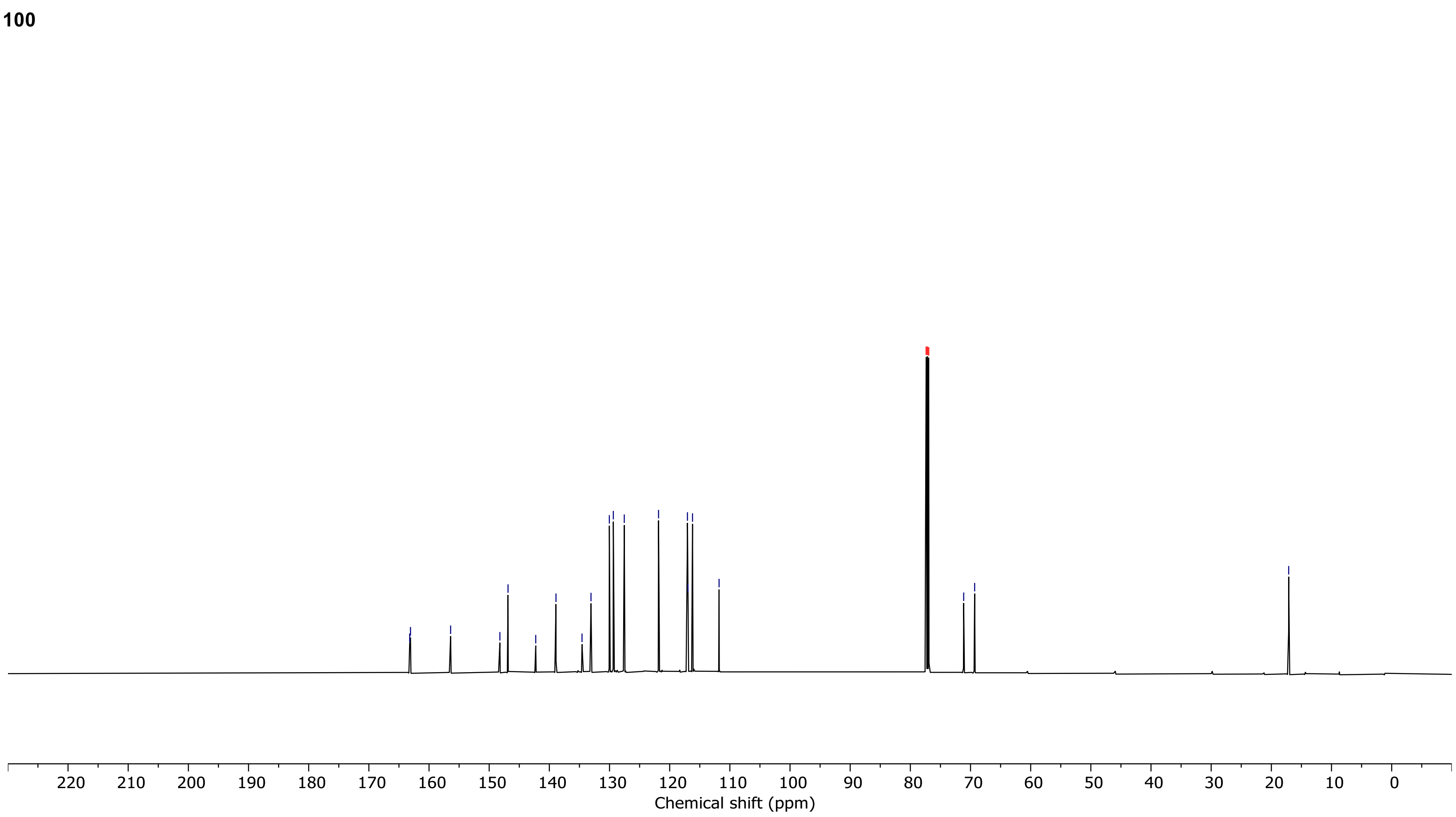

^{1}H NMR of 101

$CDCl_3$, 500 MHz, 23 ºC.

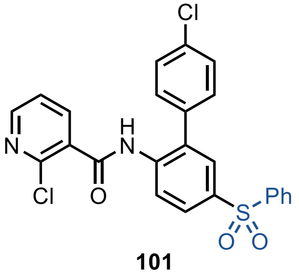

101

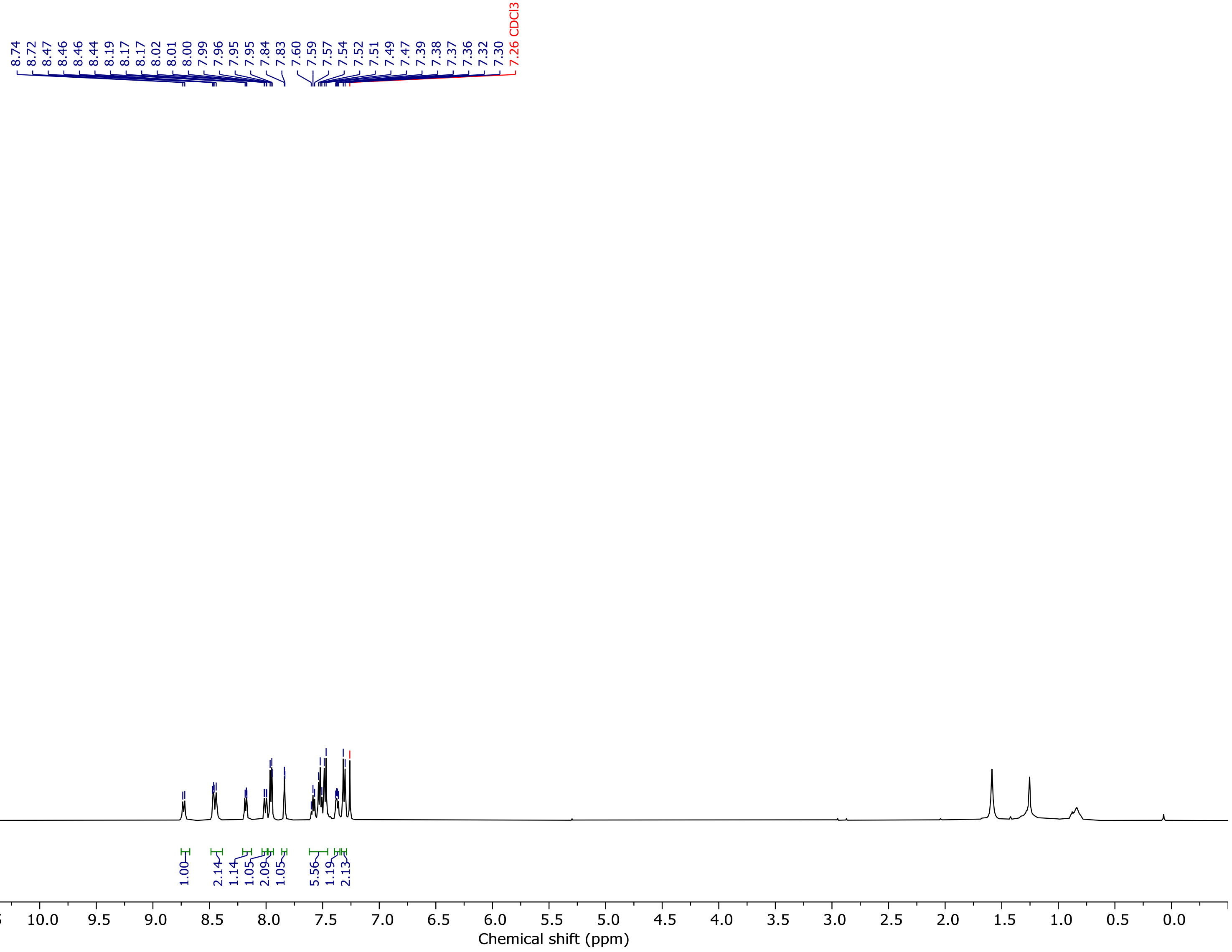

^{13}C NMR of 101

$CDCl_3$, 126 MHz, 23 ºC.

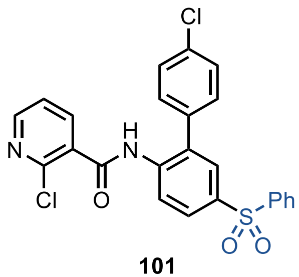

101

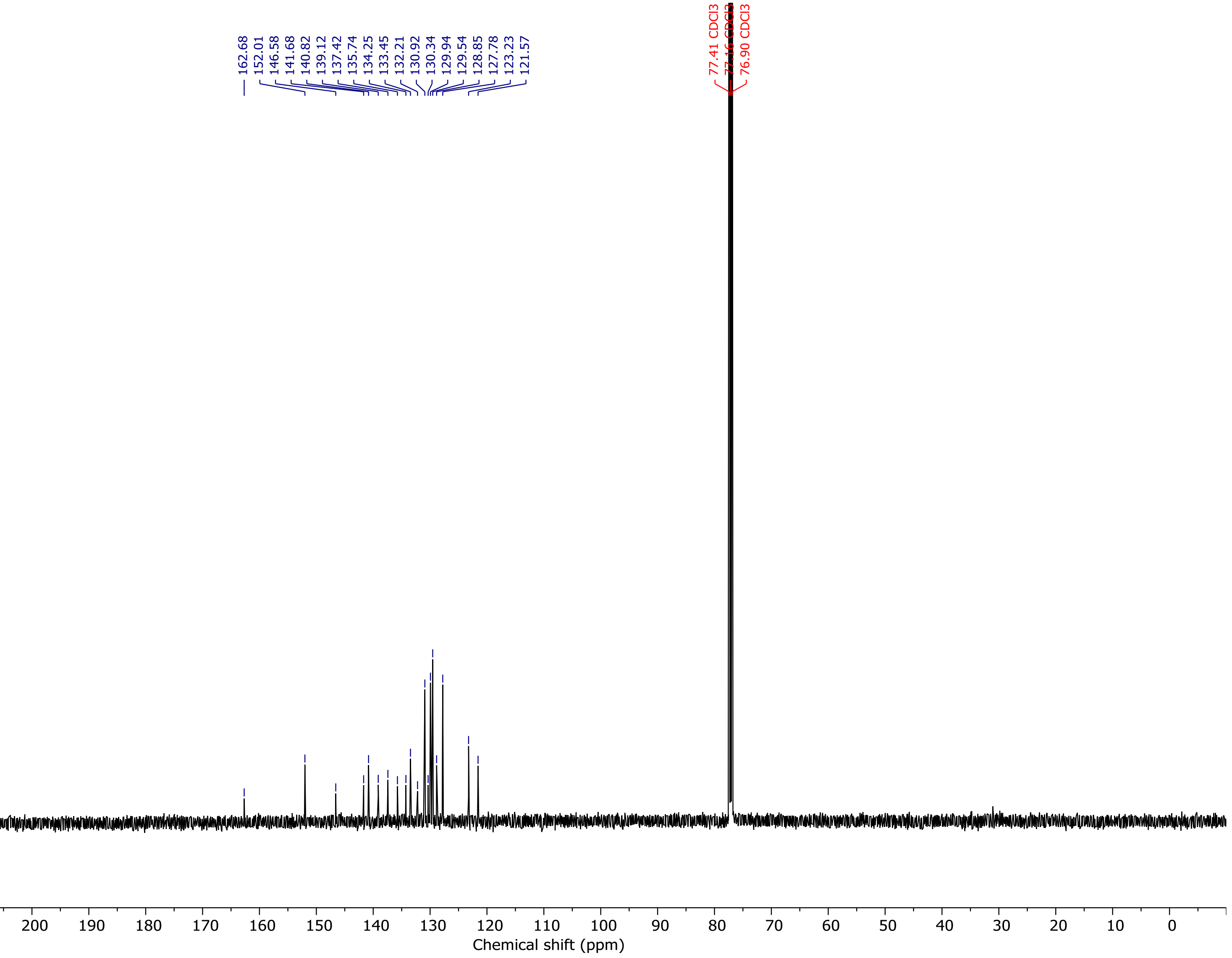

^{1}H NMR of 102

CDCl3, 500 MHz, 23 ºC.

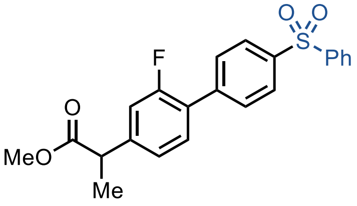

102

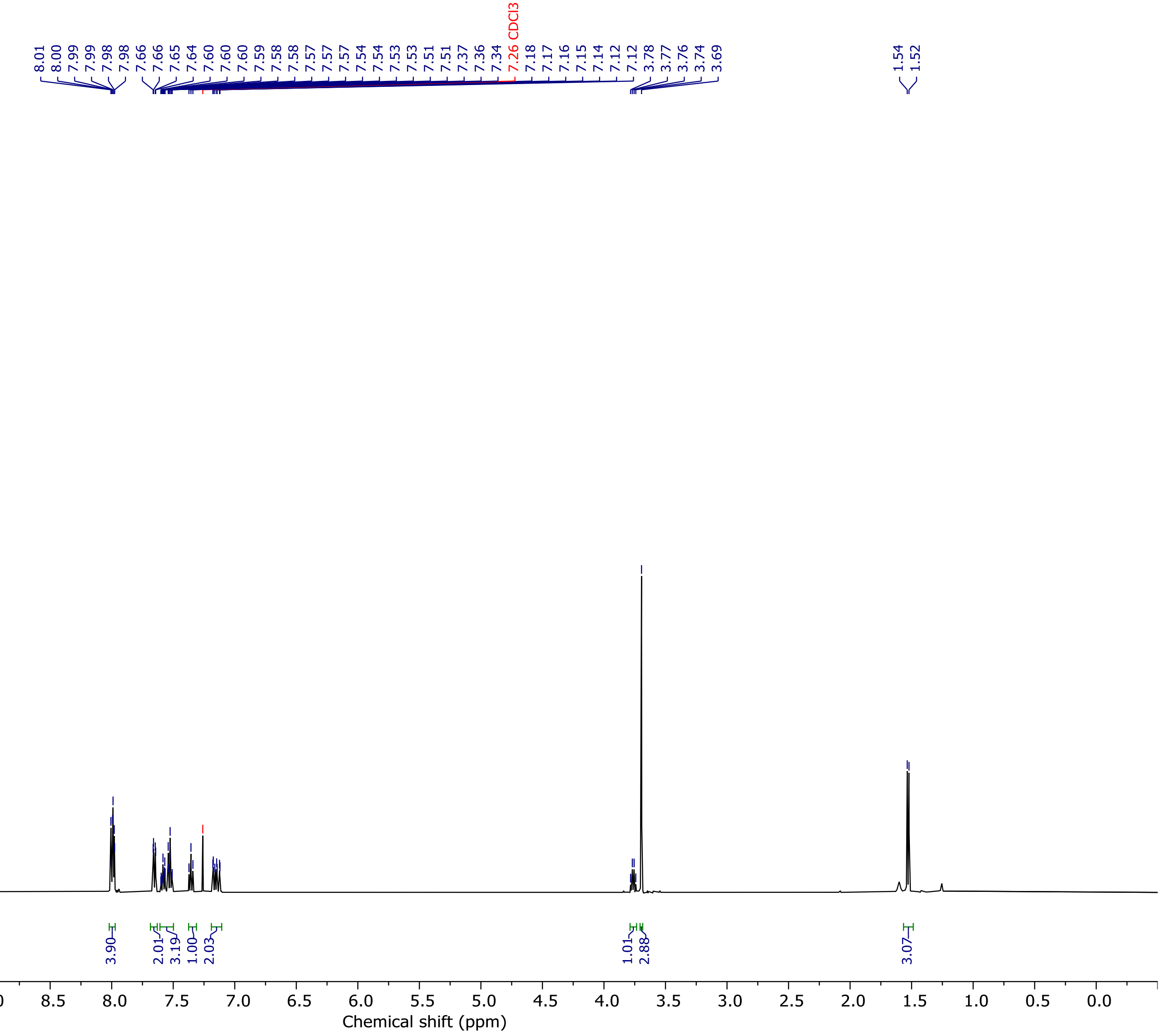

^{13}C NMR of 102

$CDCl_3$, 126 MHz, 23 ºC.

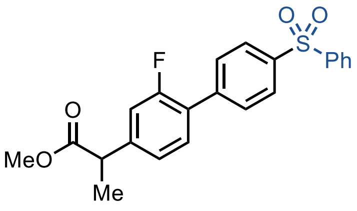

102

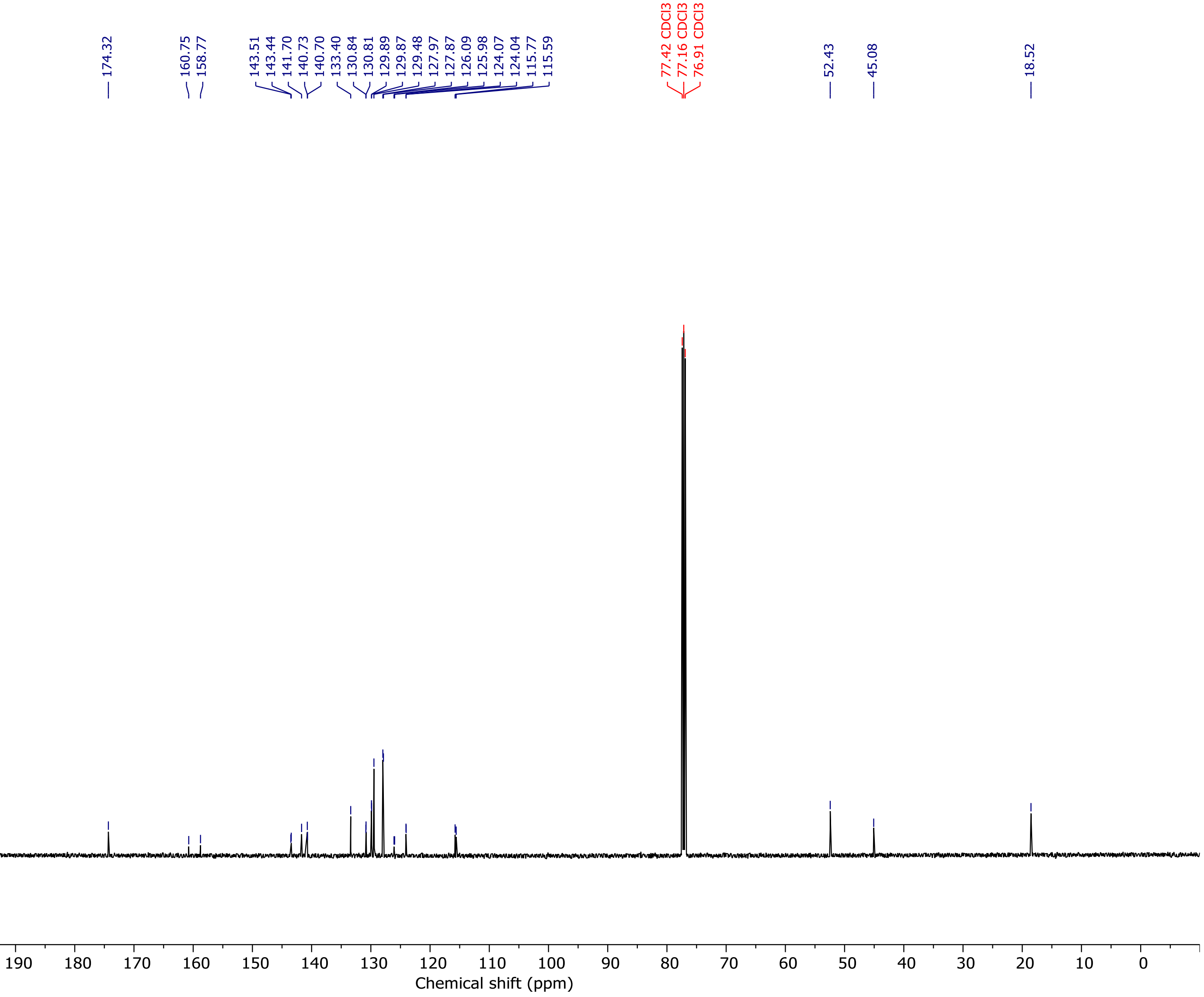

^{19}F NMR of 102

$CDCl_3$, 471 MHz, 23 ºC.

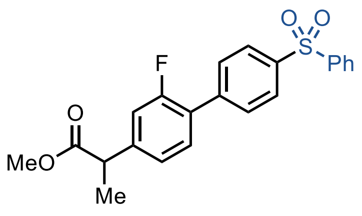

102

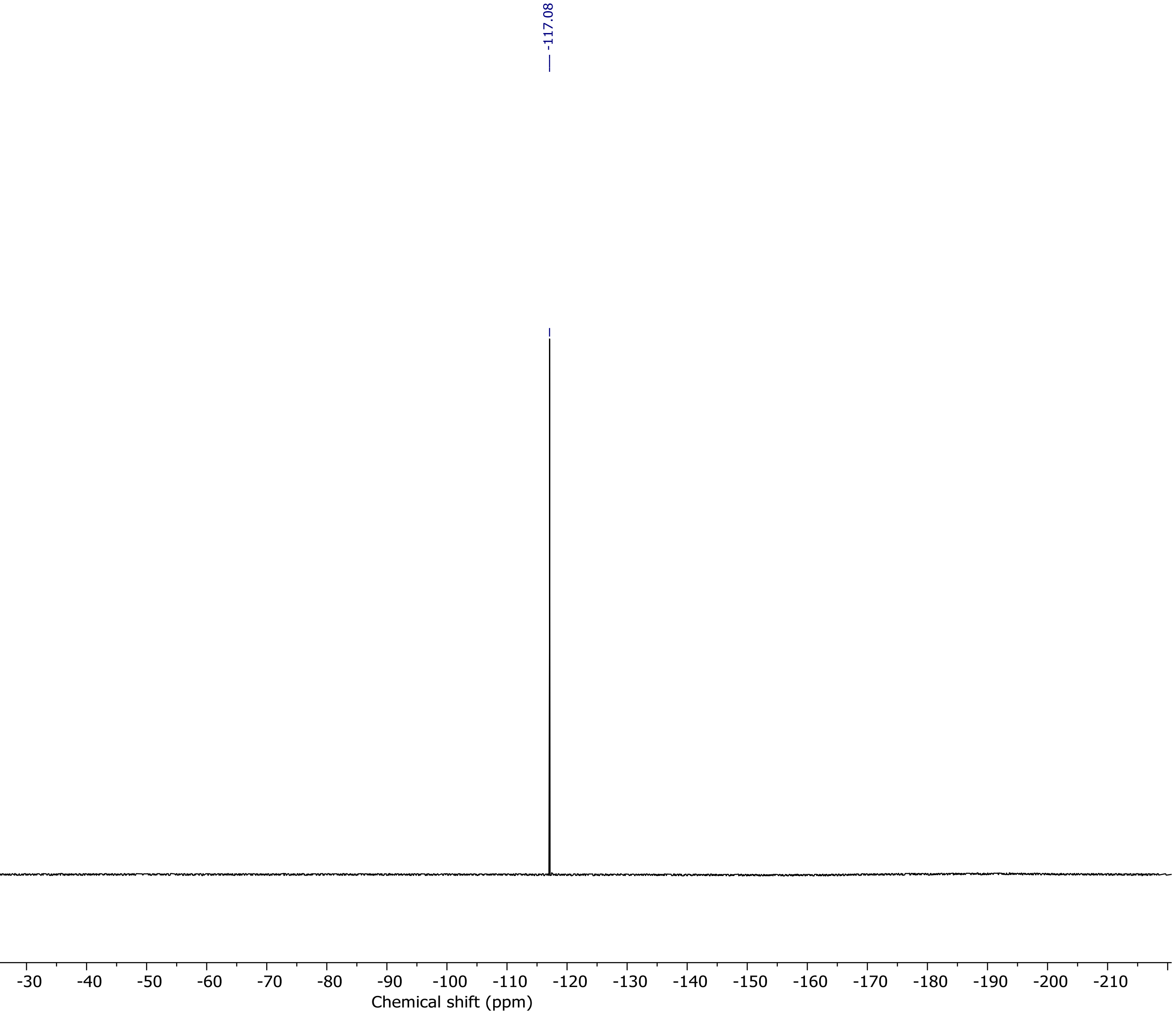

^{1}H NMR of 103

$CDCl_3$, 500 MHz, 23 ºC.

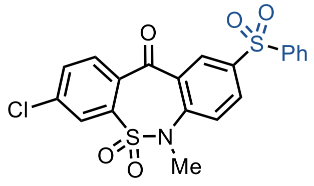

103

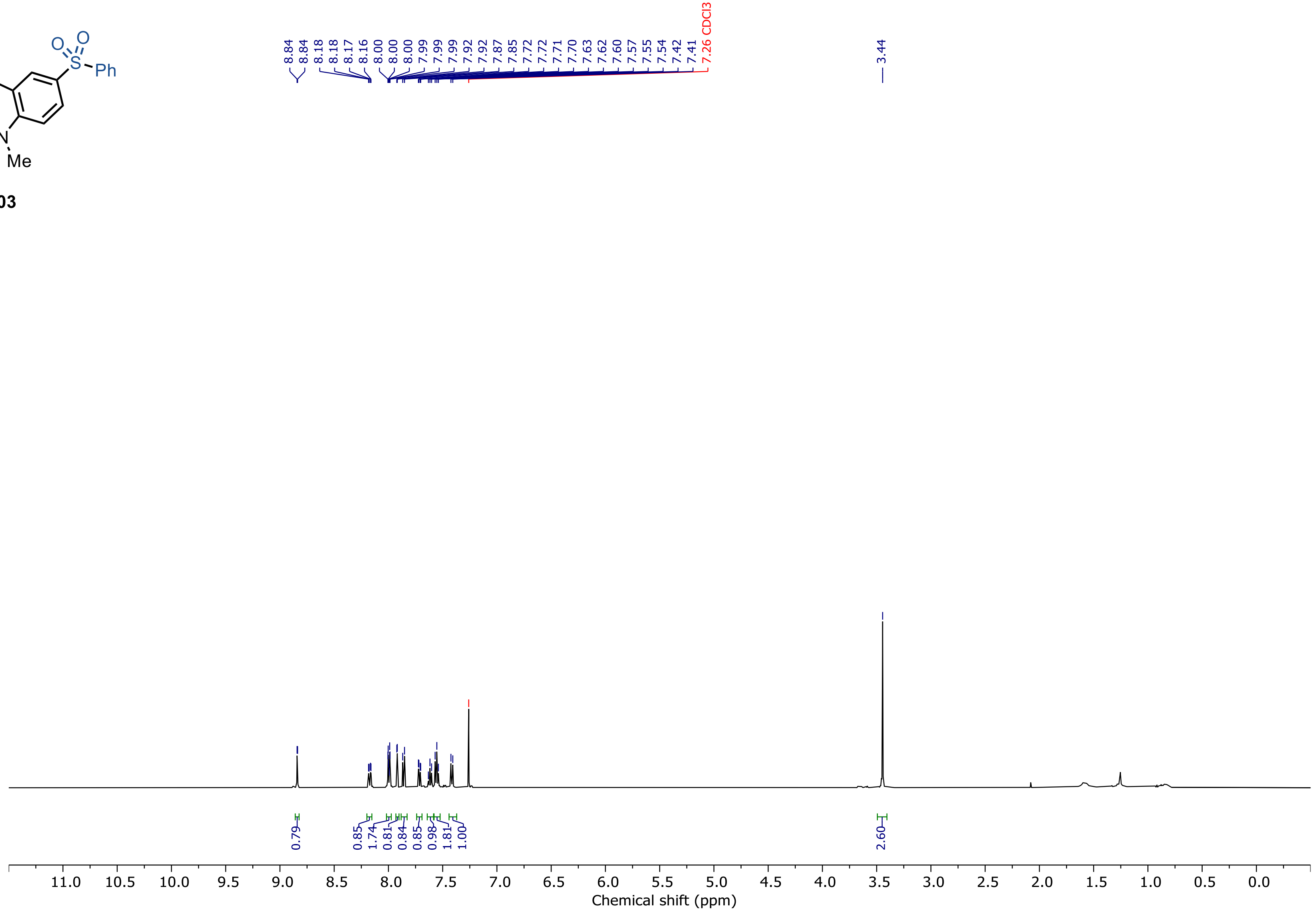

^{13}C NMR of 103

$CDCl_3$, 126 MHz, 23 ºC.

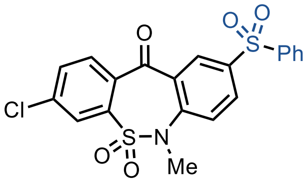

103

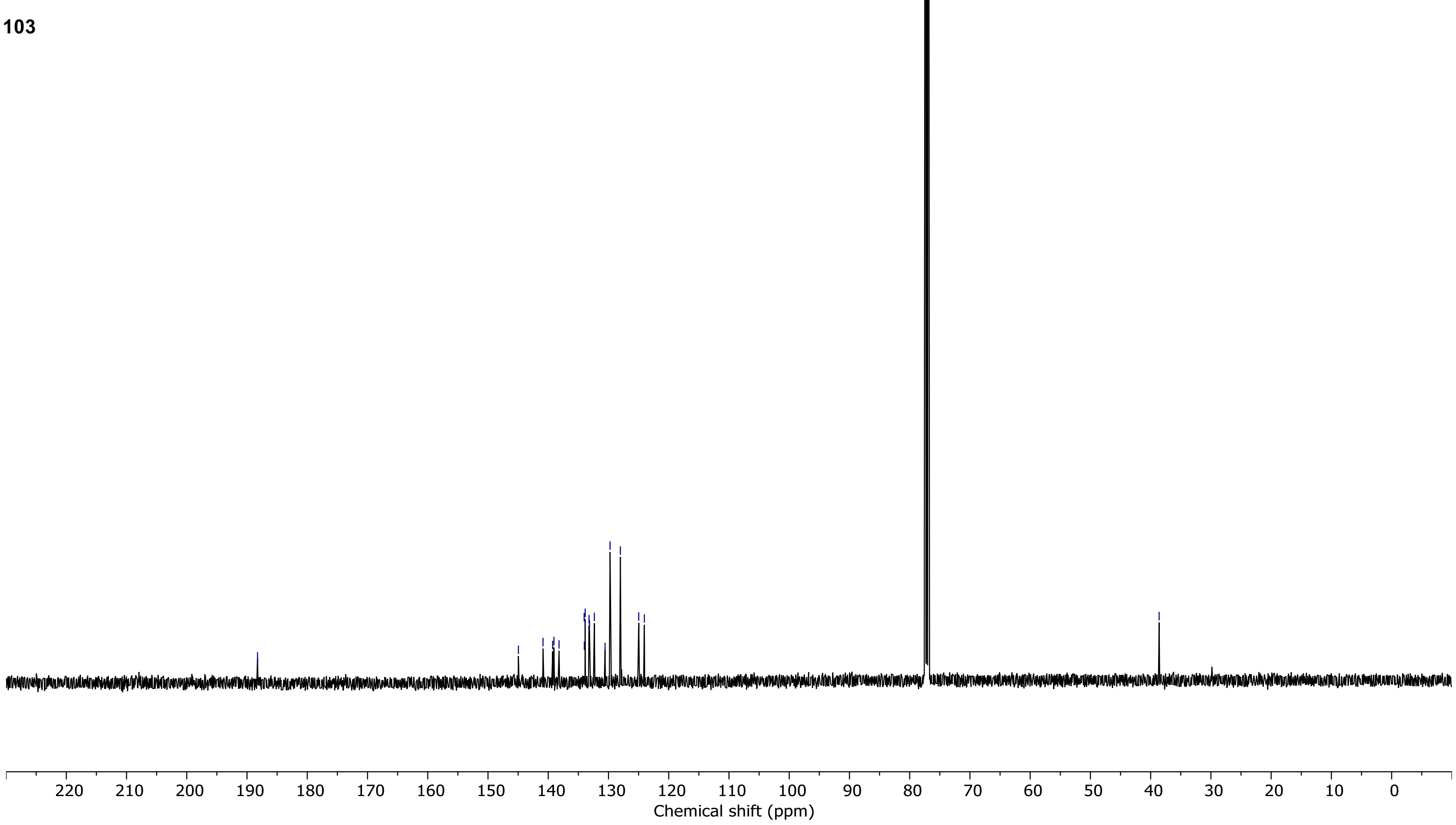

^{1}H NMR of 104

$CDCl_3$, 500 MHz, 23 ºC.

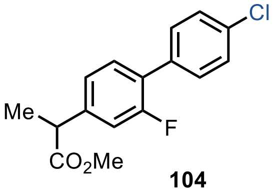

104

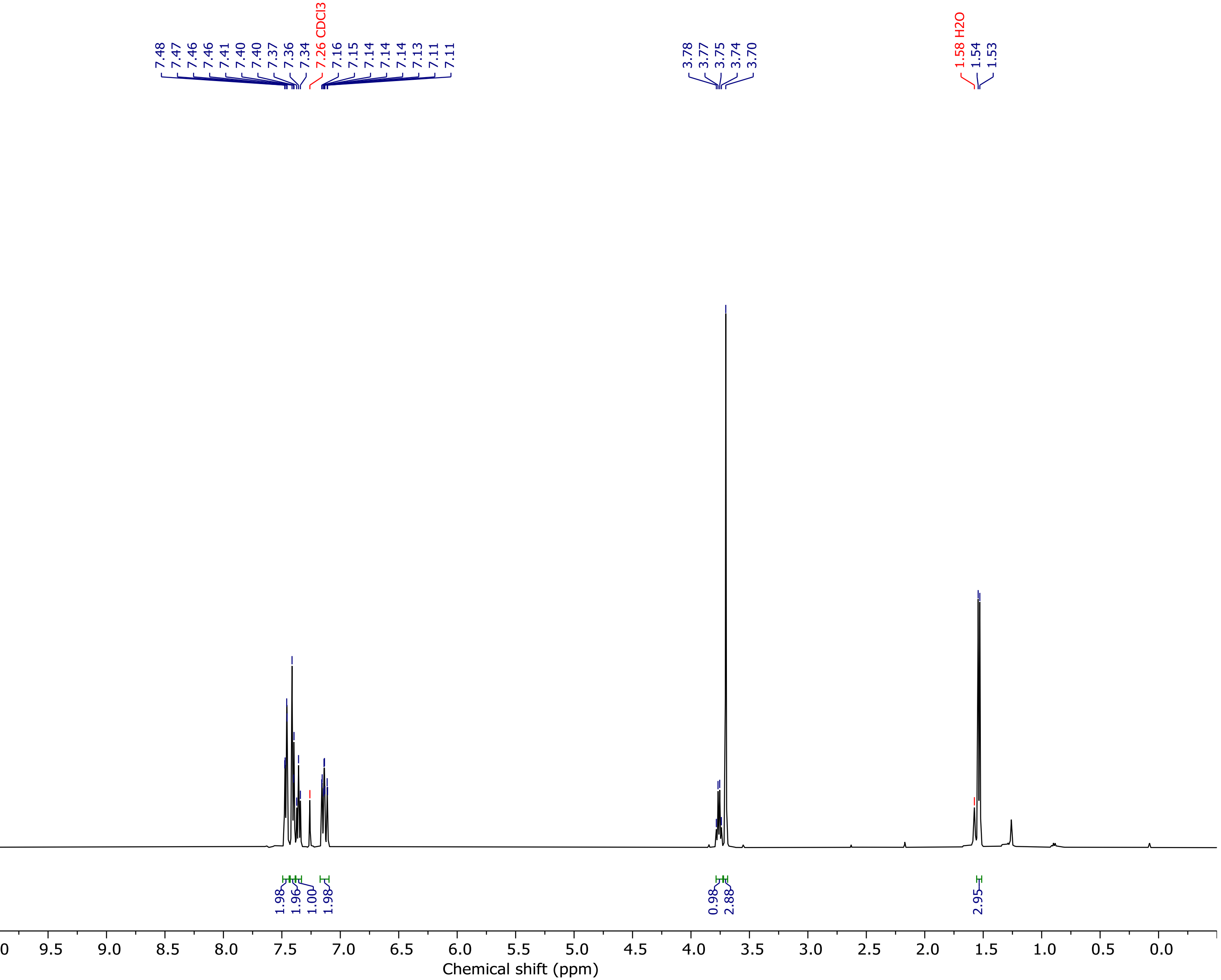

^{13}C NMR of 104

$CDCl_3$, 126 MHz, 23 ºC.

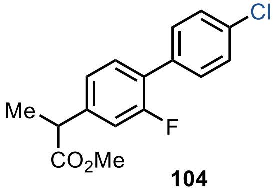

104

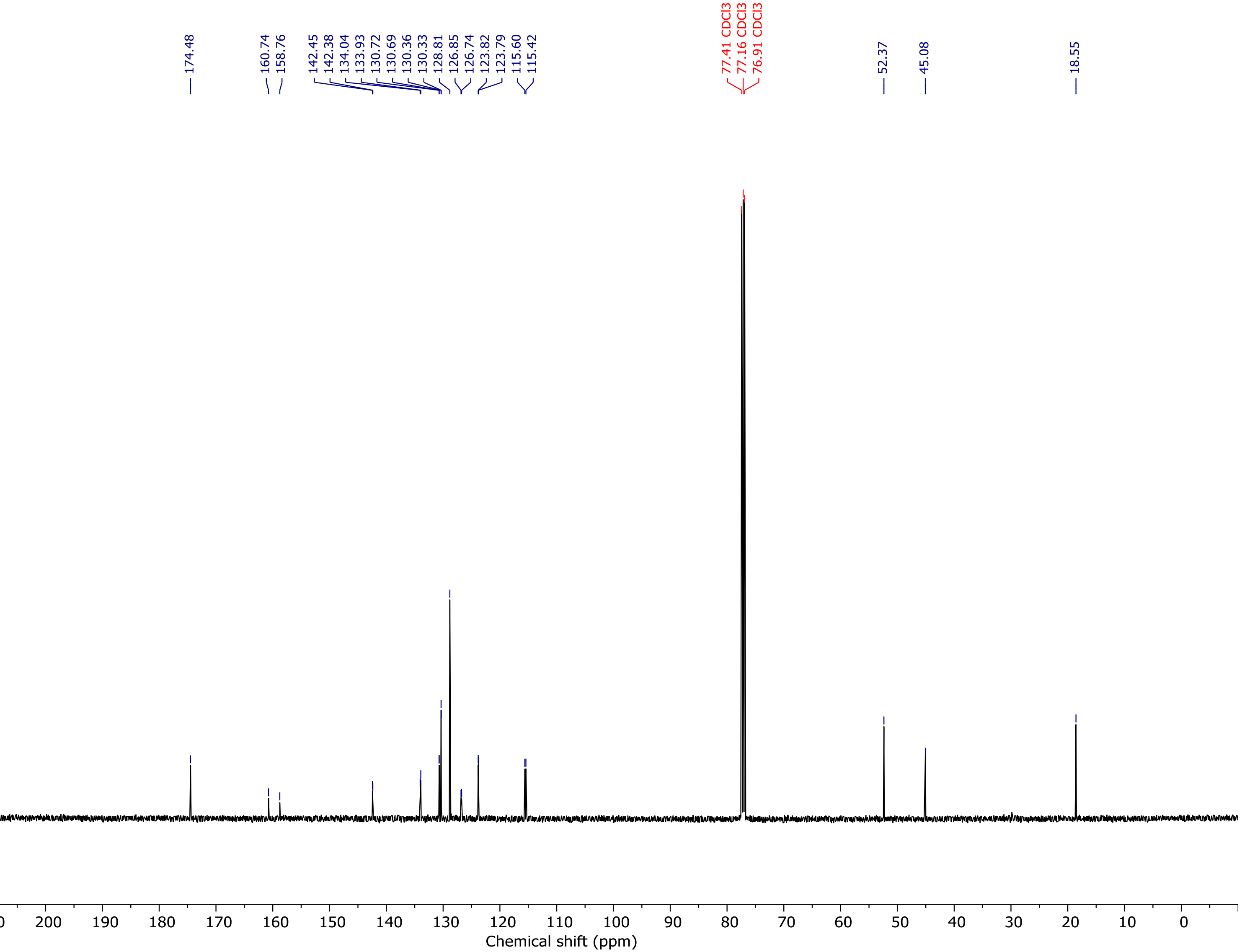

^{19}F NMR of 104

$CDCl_3$, 471 MHz, 23 ºC.

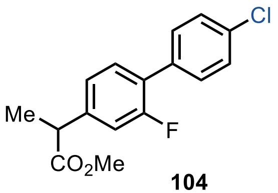

104

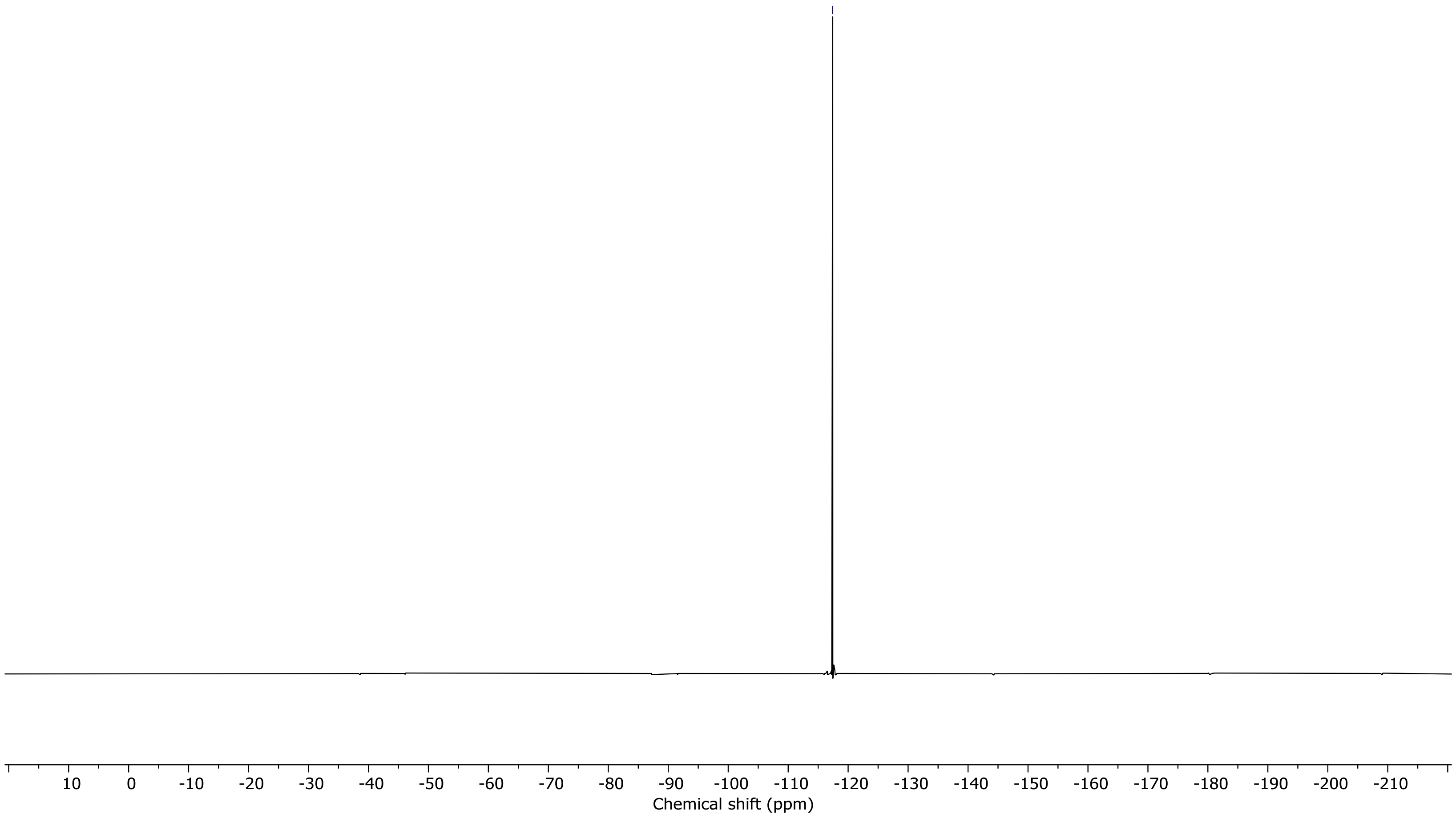

^{1}H NMR of 105

$CDCl_3$, 500 MHz, 23 ºC.

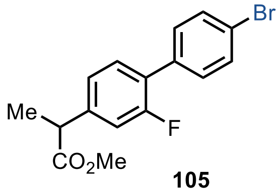

105

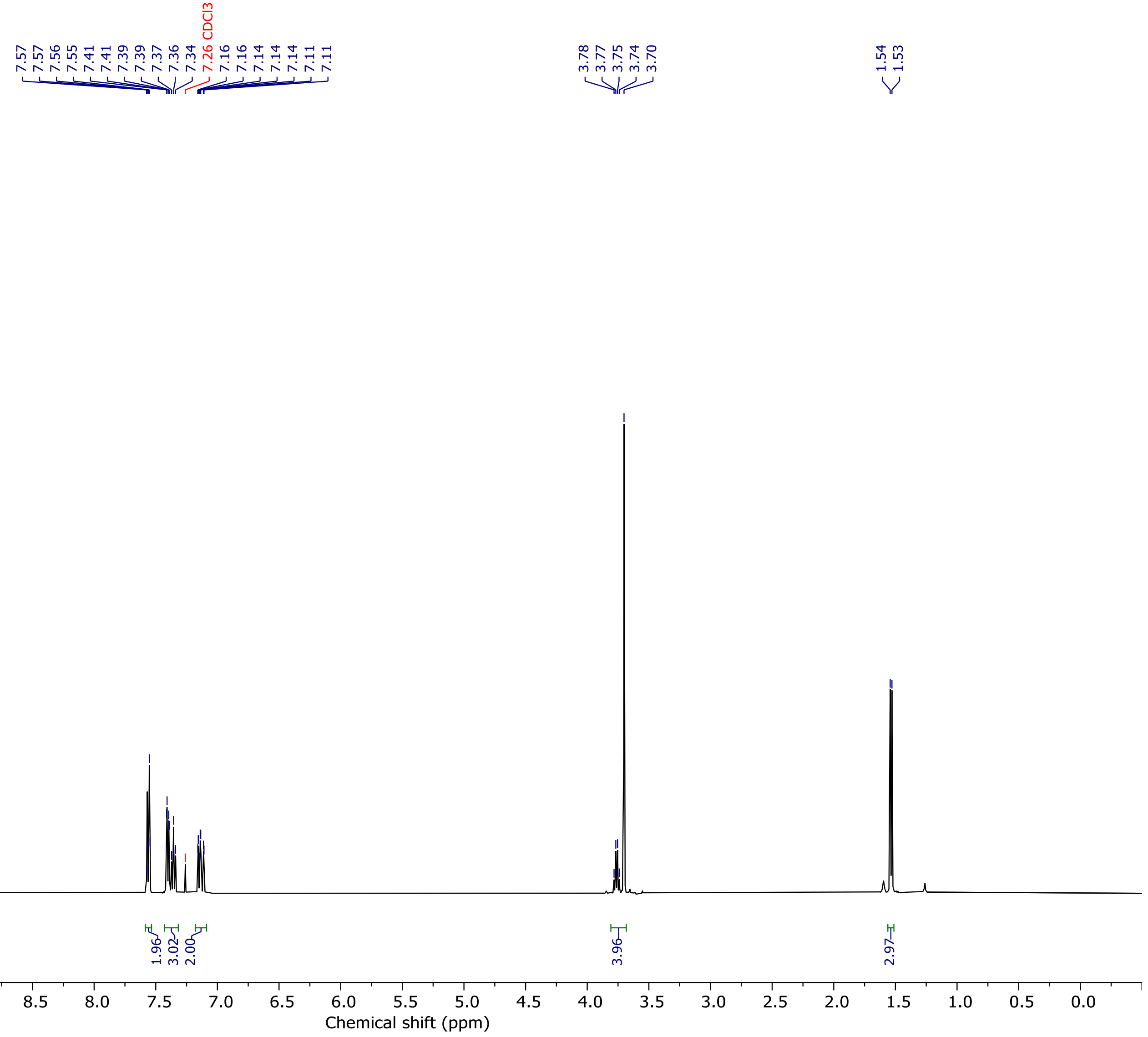

^{13}C NMR of 105

$CDCl_3$, 126 MHz, 23 ºC.

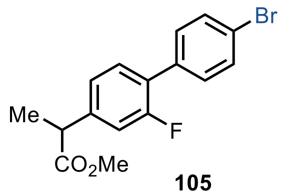

105

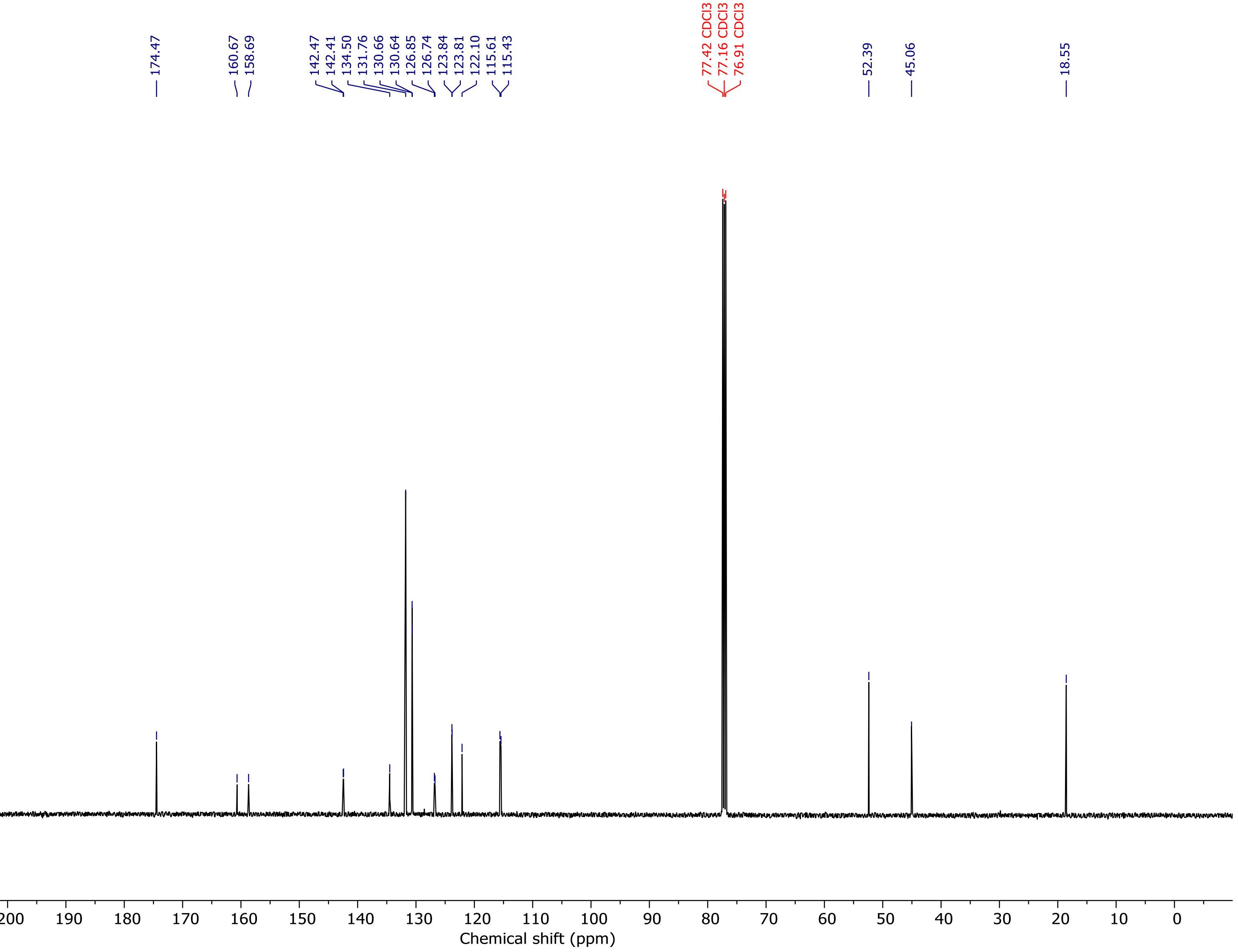

^{19}F NMR of 105

$CDCl_3$, 471 MHz, 23 ºC.

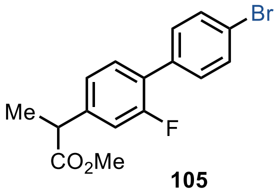

105

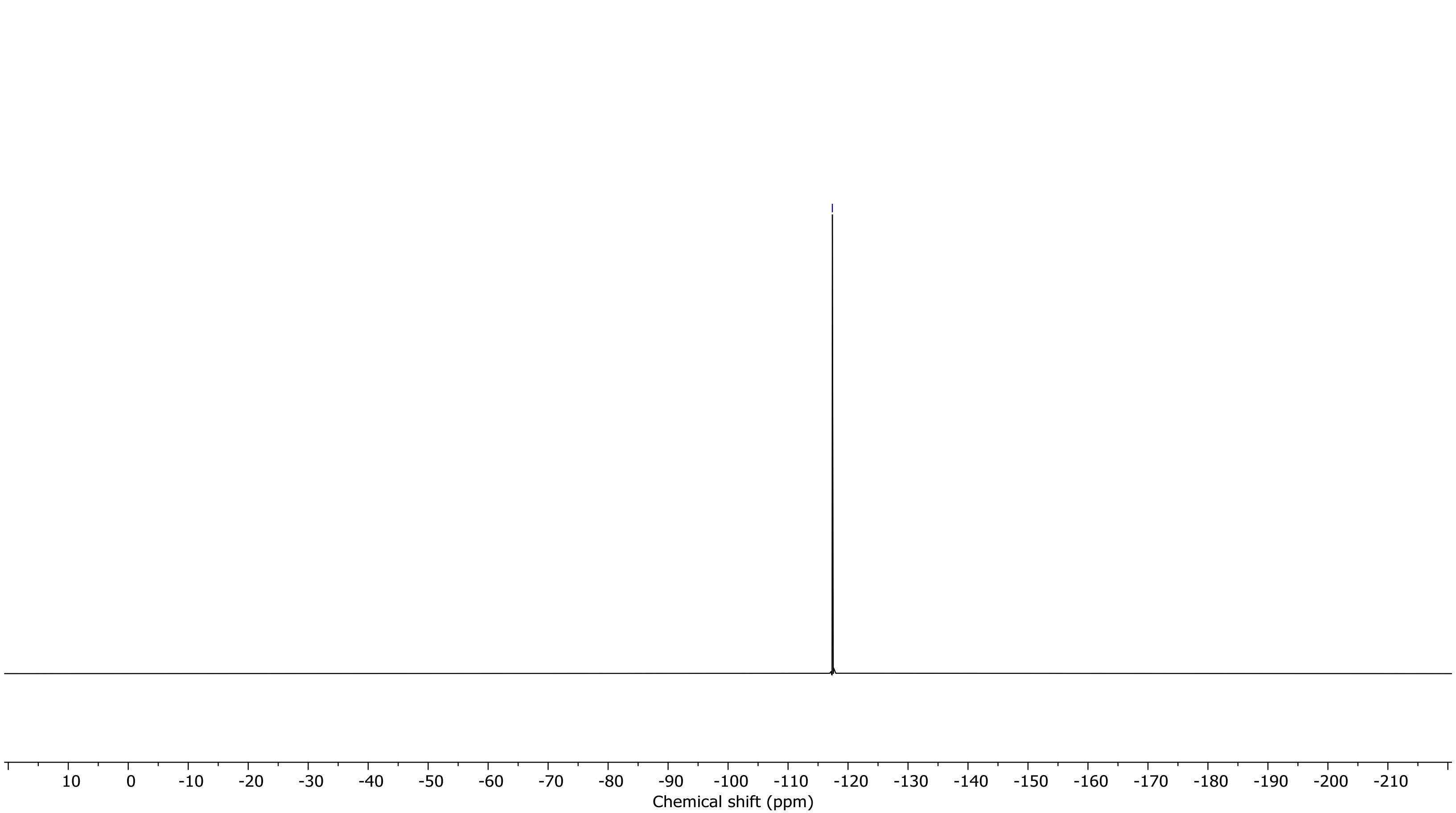

^{1}H NMR of 106

CDCl$_3$, 500 MHz, 23 ºC.

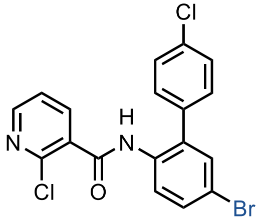

106

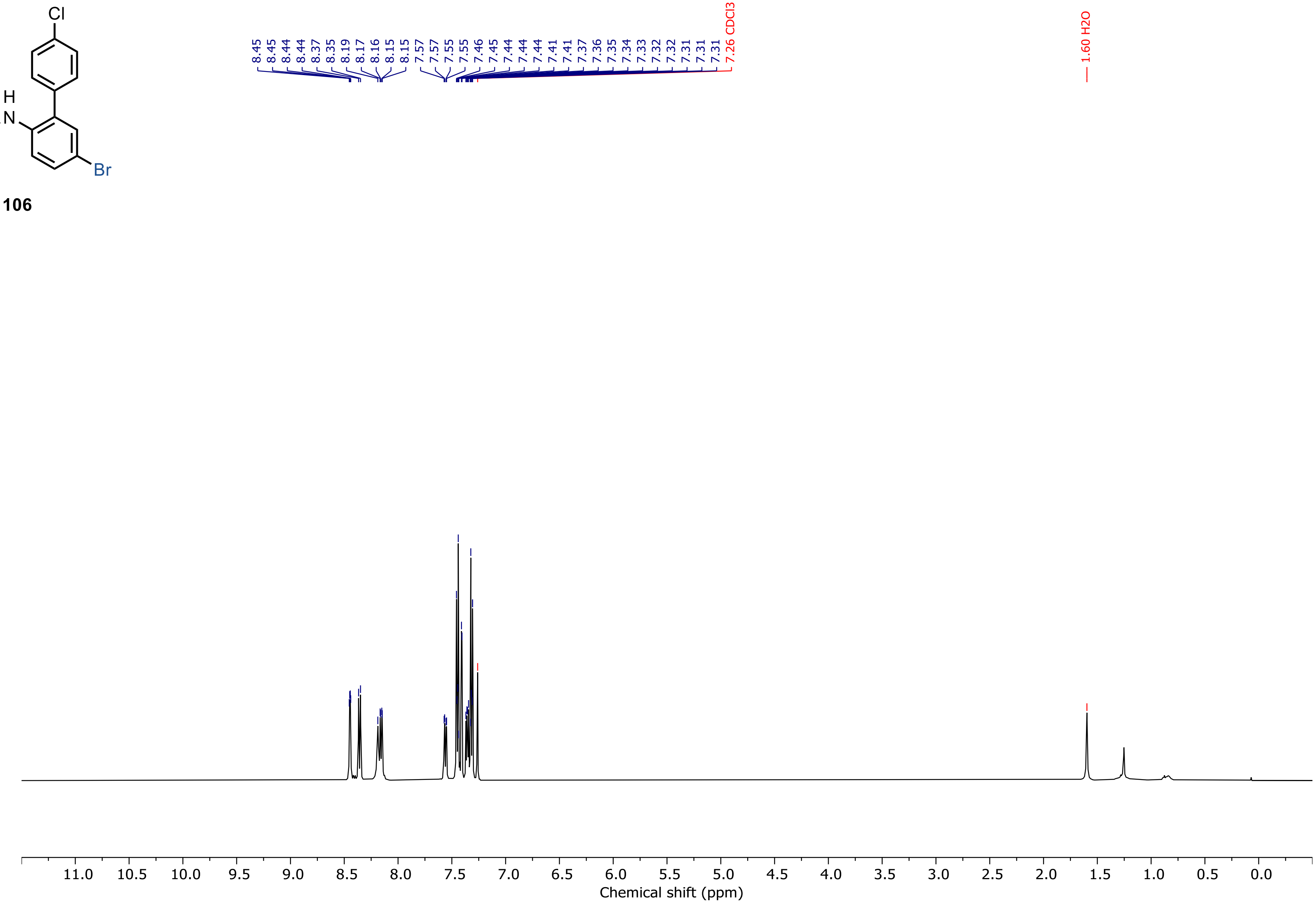

^{13}C NMR of 106

$CDCl_3$, 126 MHz, 23 ºC.

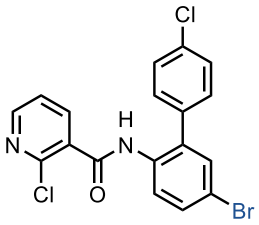

106

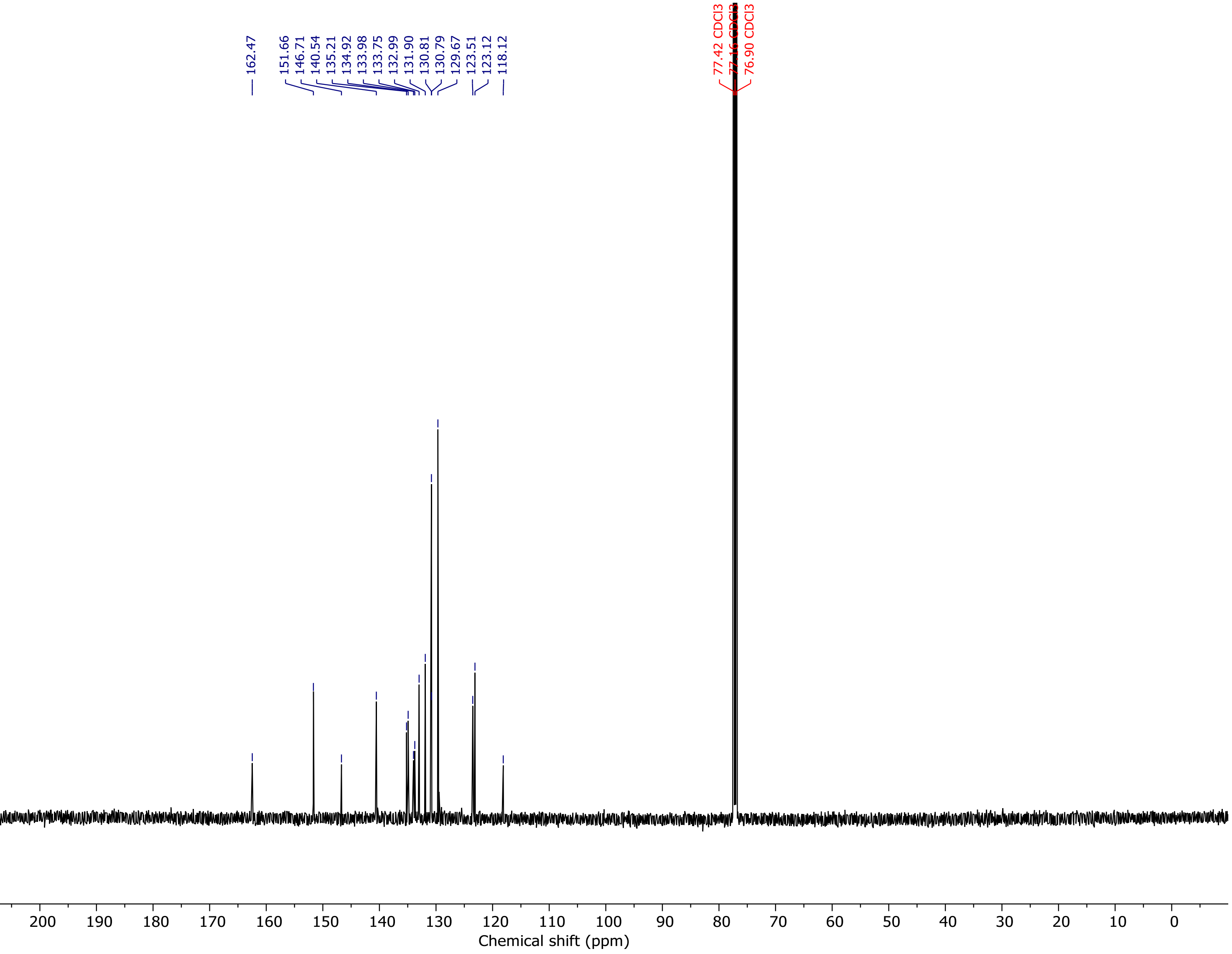

^{1}H NMR of 107

$CDCl_3$, 500 MHz, 23 ºC.

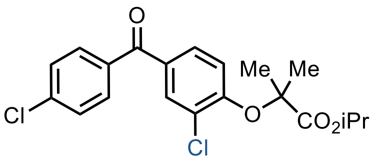

107

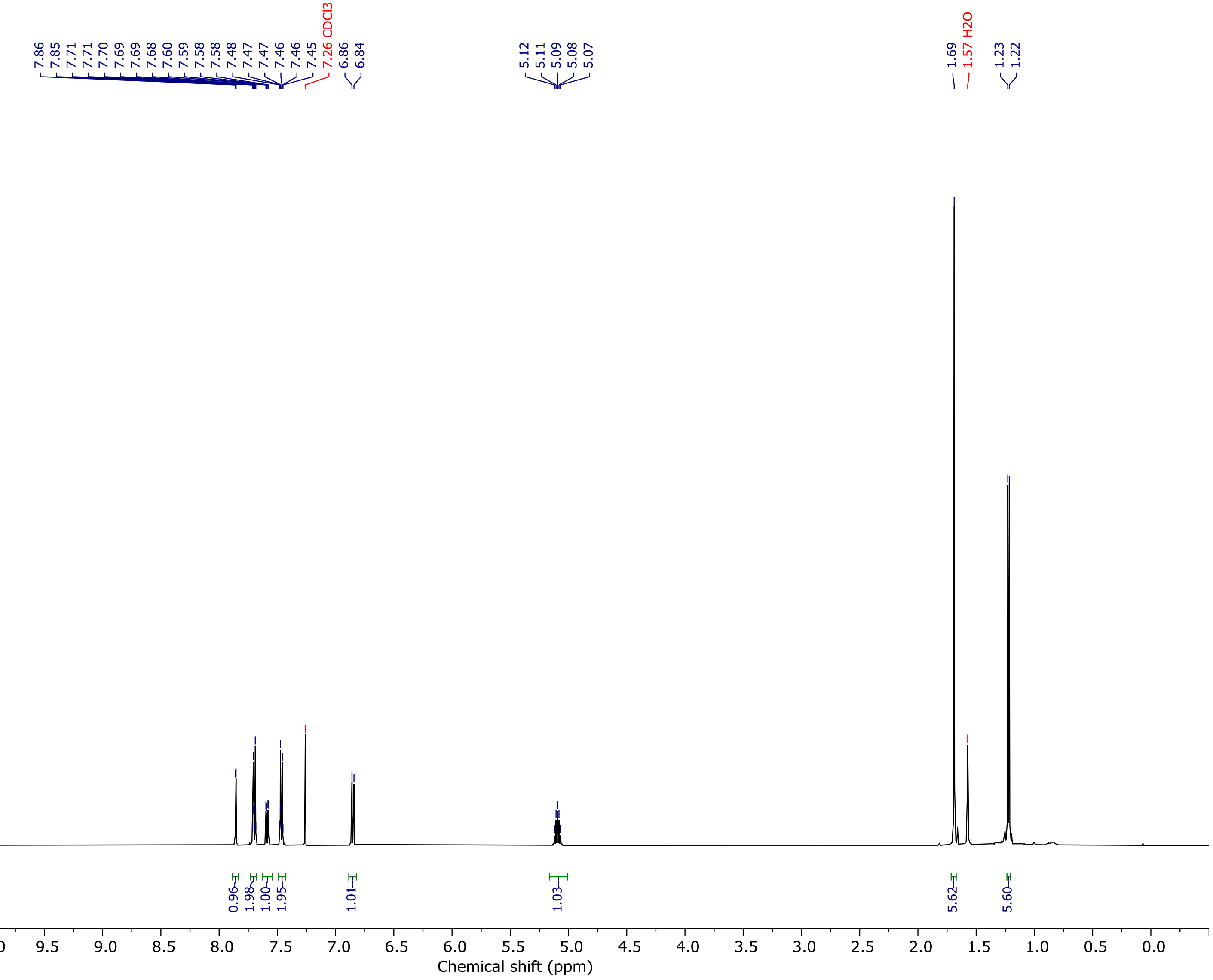

^{13}C NMR of 107

$CDCl_3$, 126 MHz, 23 ºC.

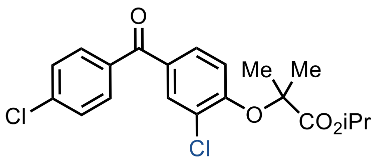

107

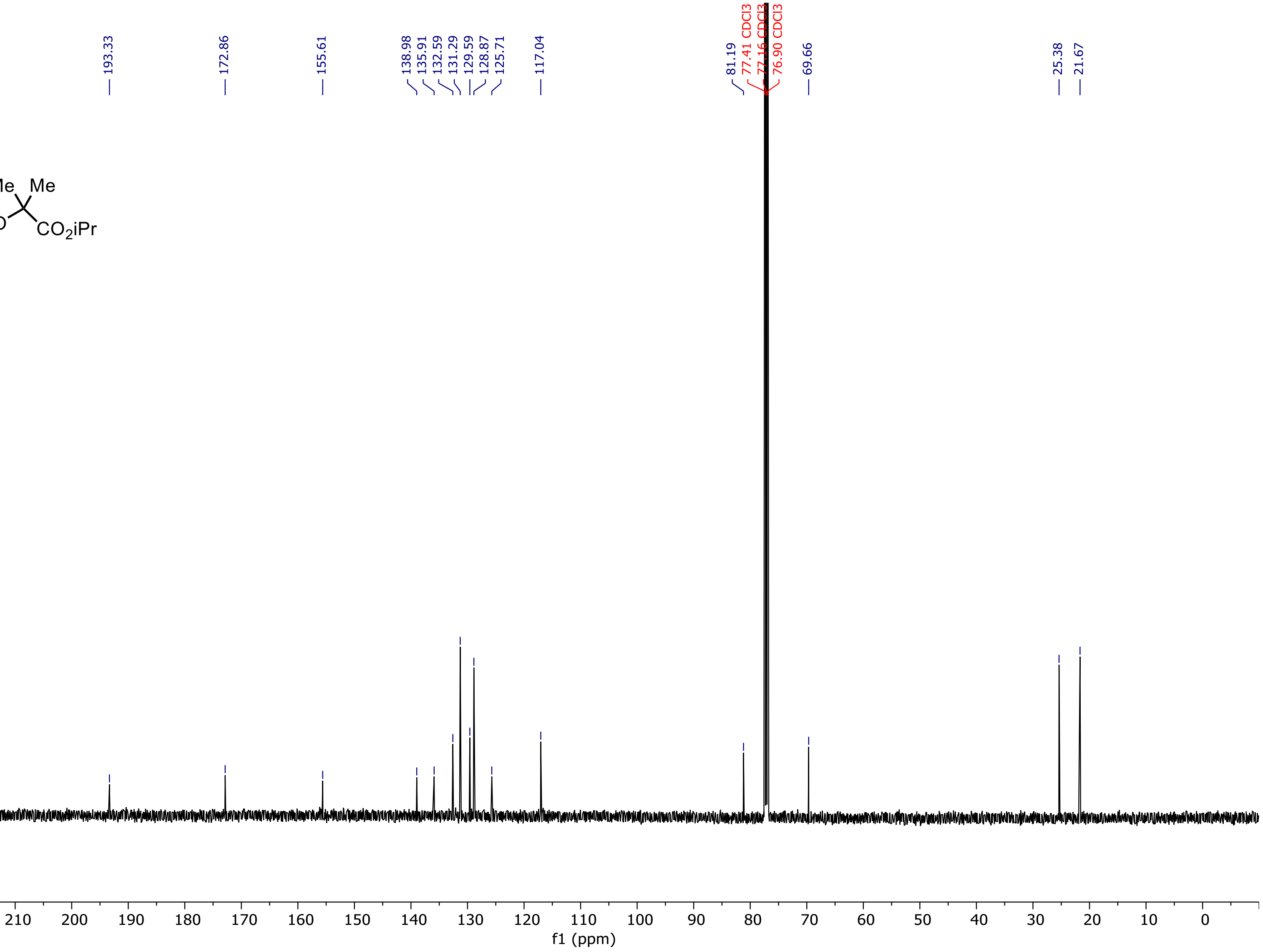

^{1}H NMR of 108

CDCl$_3$, 500 MHz, 23 ºC.

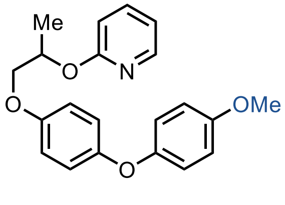

108

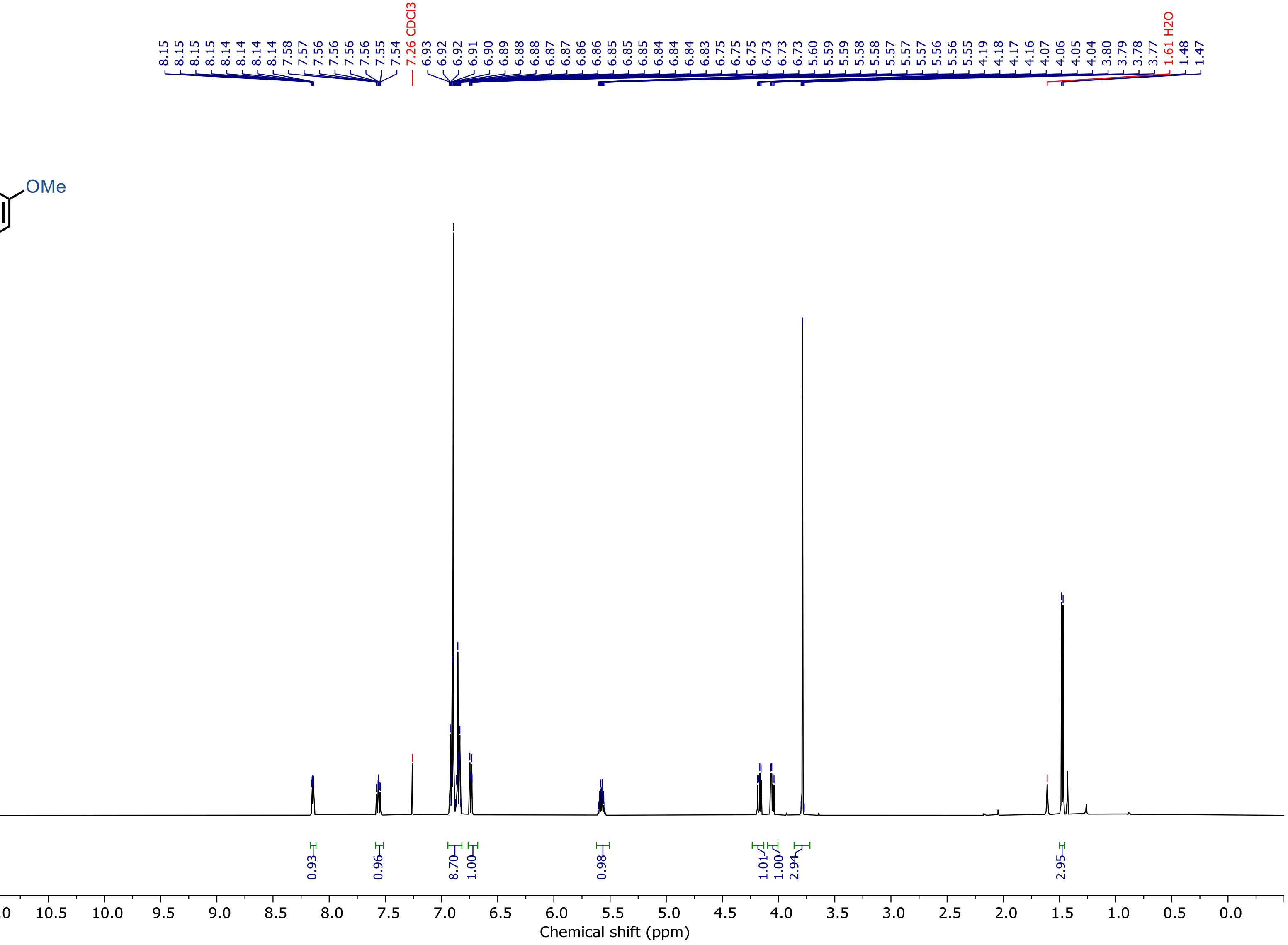

^{13}C NMR of 108

$CDCl_3$, 126 MHz, 23 ºC.

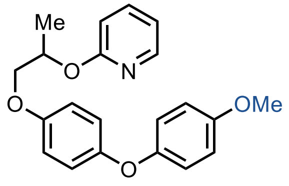

108

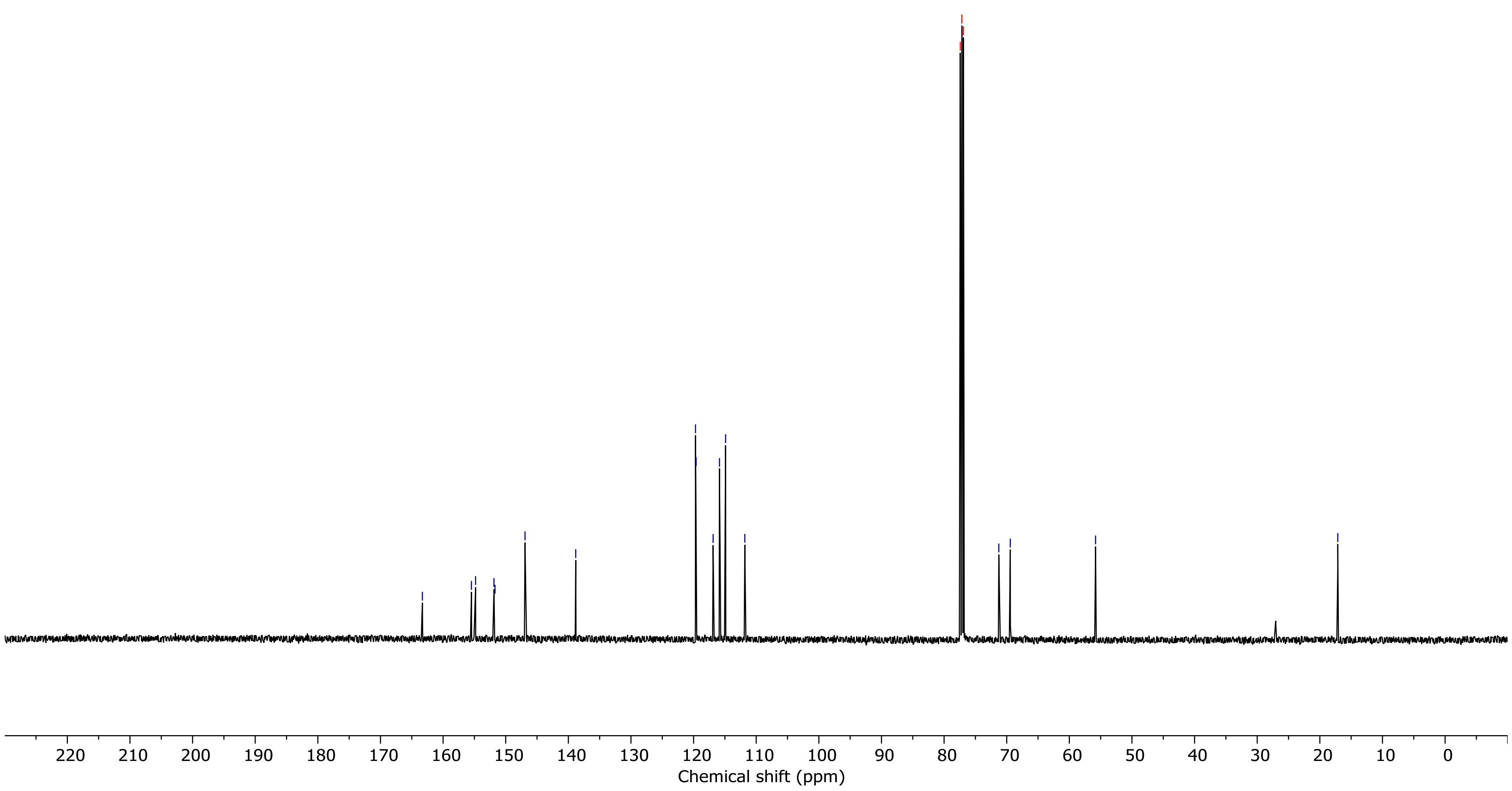

^{1}H NMR of 109

$CDCl_3$, 600 MHz, 23 ºC.

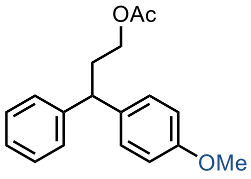

109

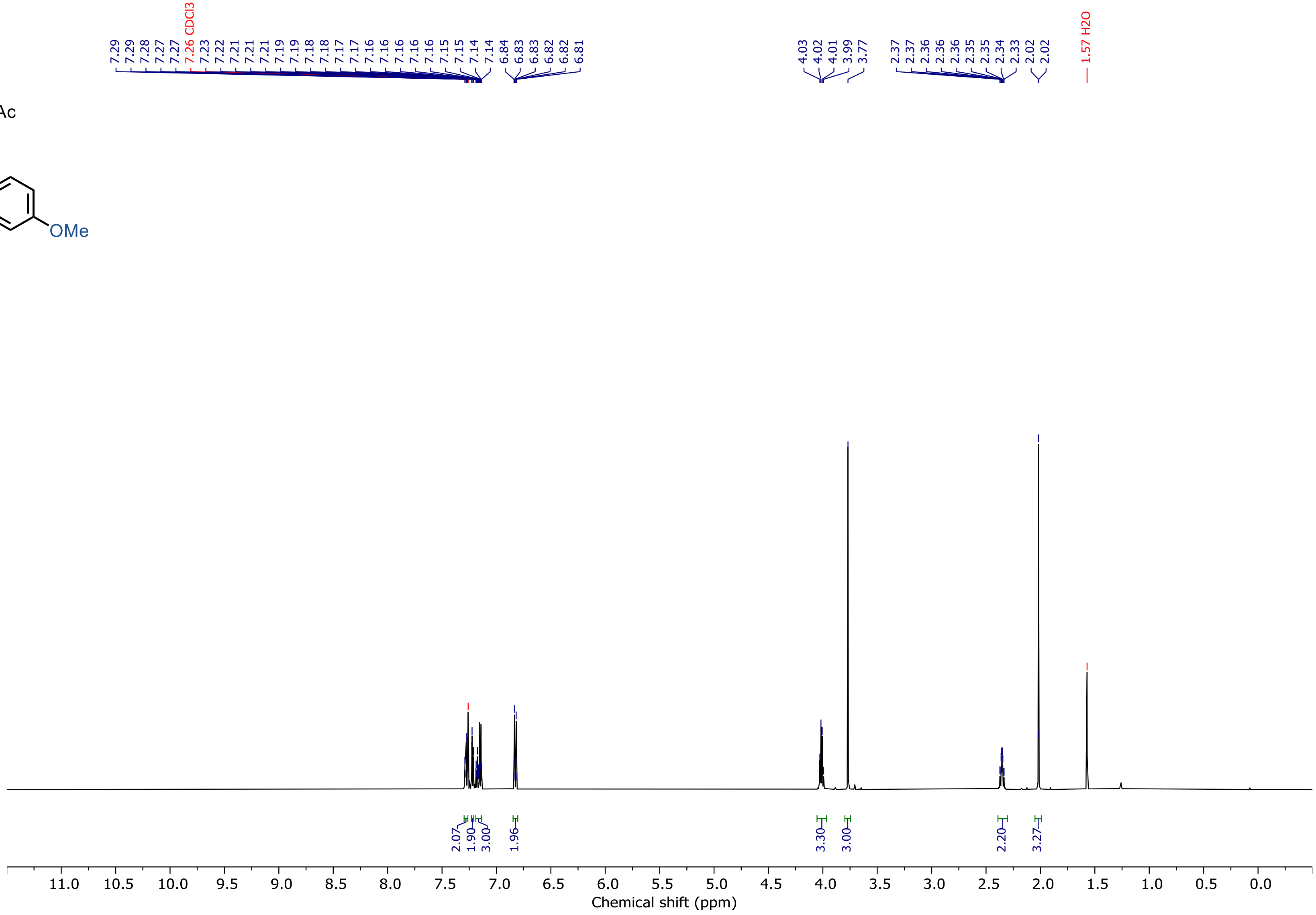

^{13}C NMR of 109

CDCl$_3$, 151 MHz, 23 ºC.

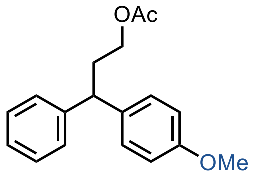

109

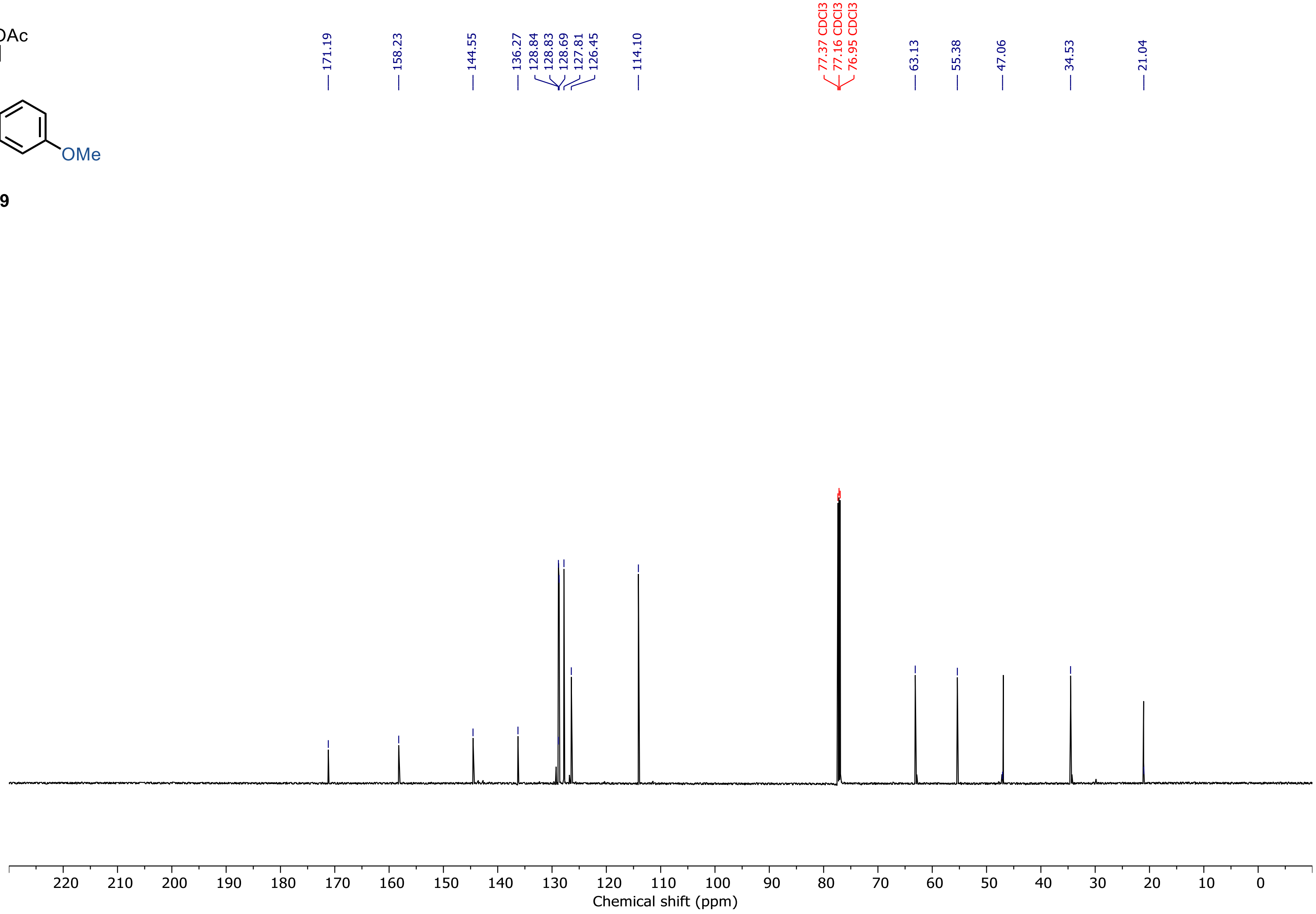

^{1}H NMR of 110

$CDCl_3$, 500 MHz, 23 ºC.

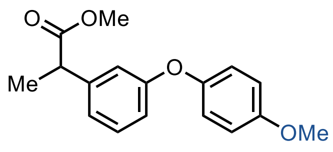

110

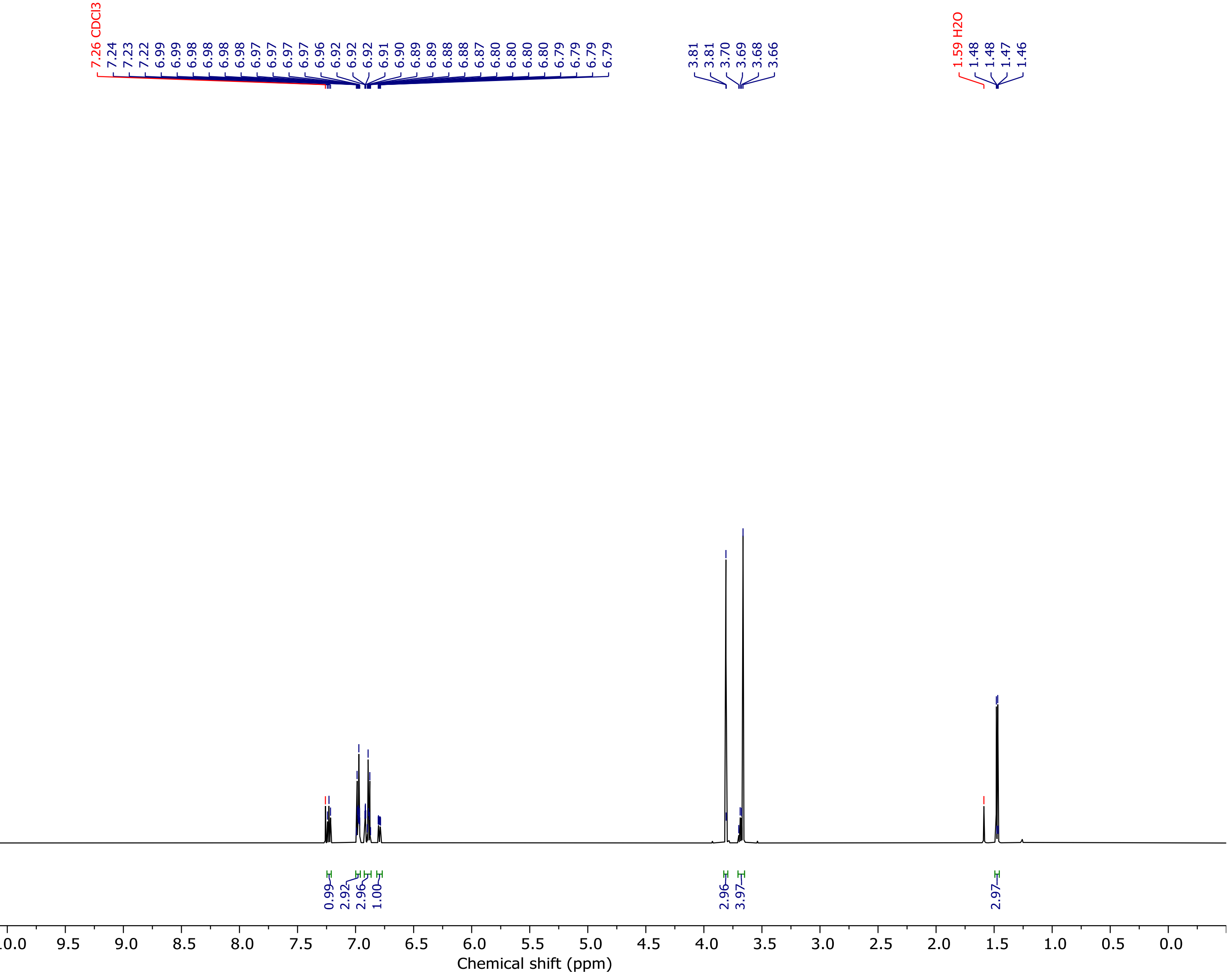

^{13}C NMR of 110

$CDCl_3$, 126 MHz, 23 ºC.

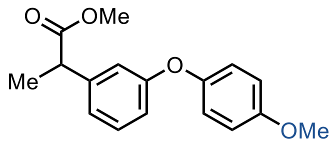

110

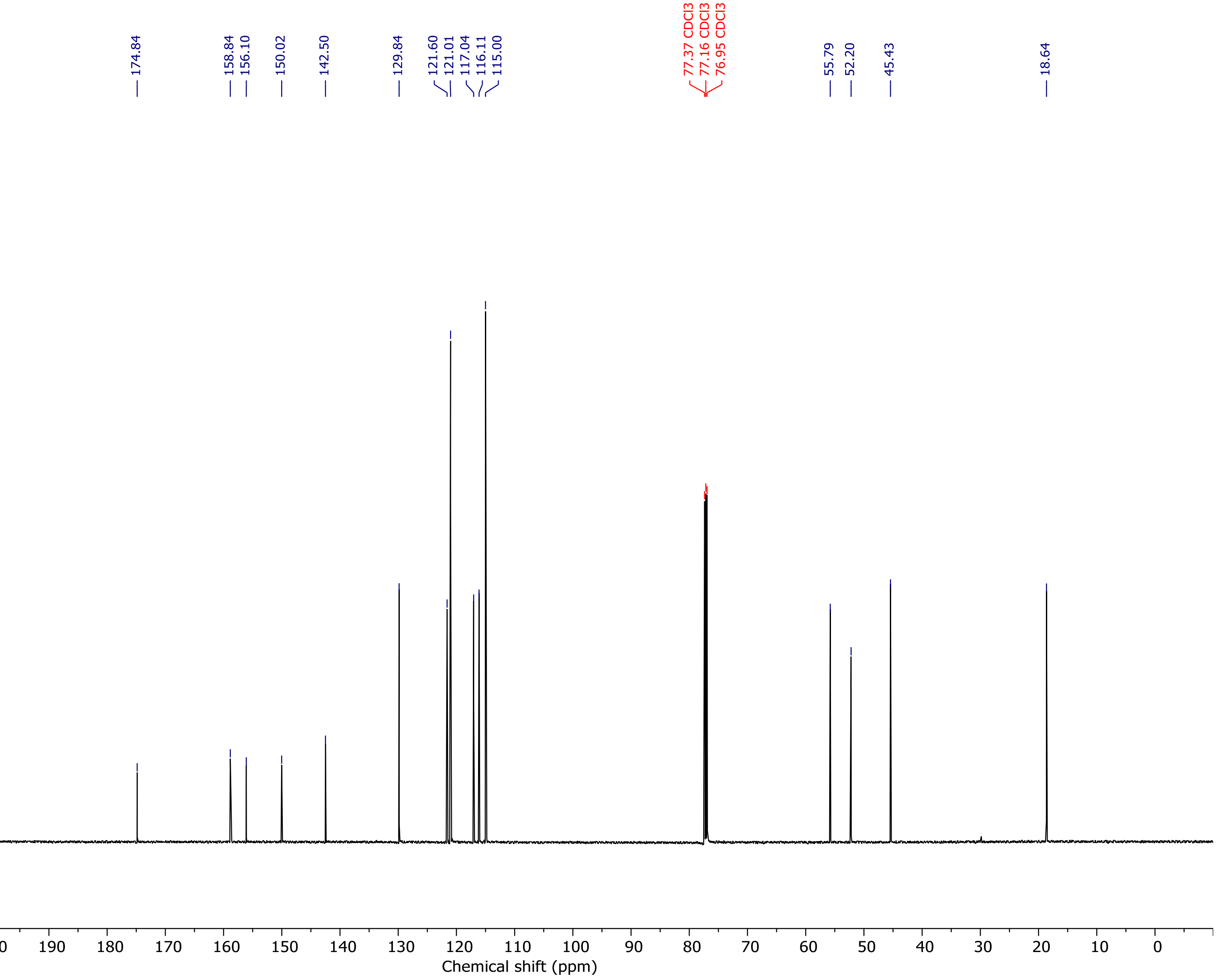

^{1}H NMR of 111

CDCl3, 500 MHz, 23 ºC.

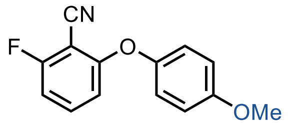

111

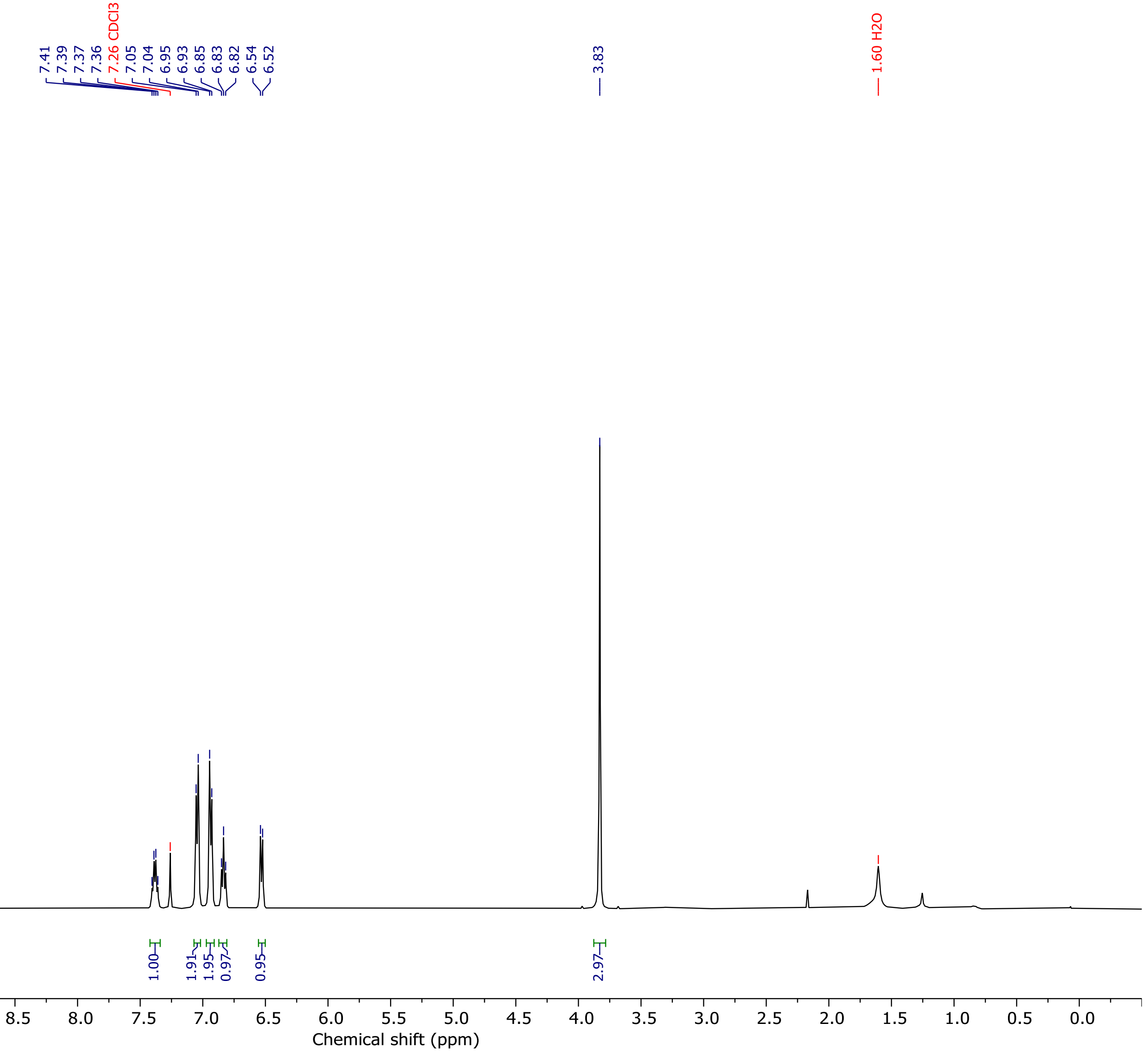

^{13}C NMR of 111

$CDCl_3$, 126 MHz, 23 ºC.

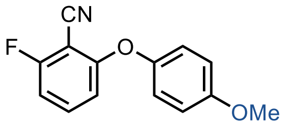

111

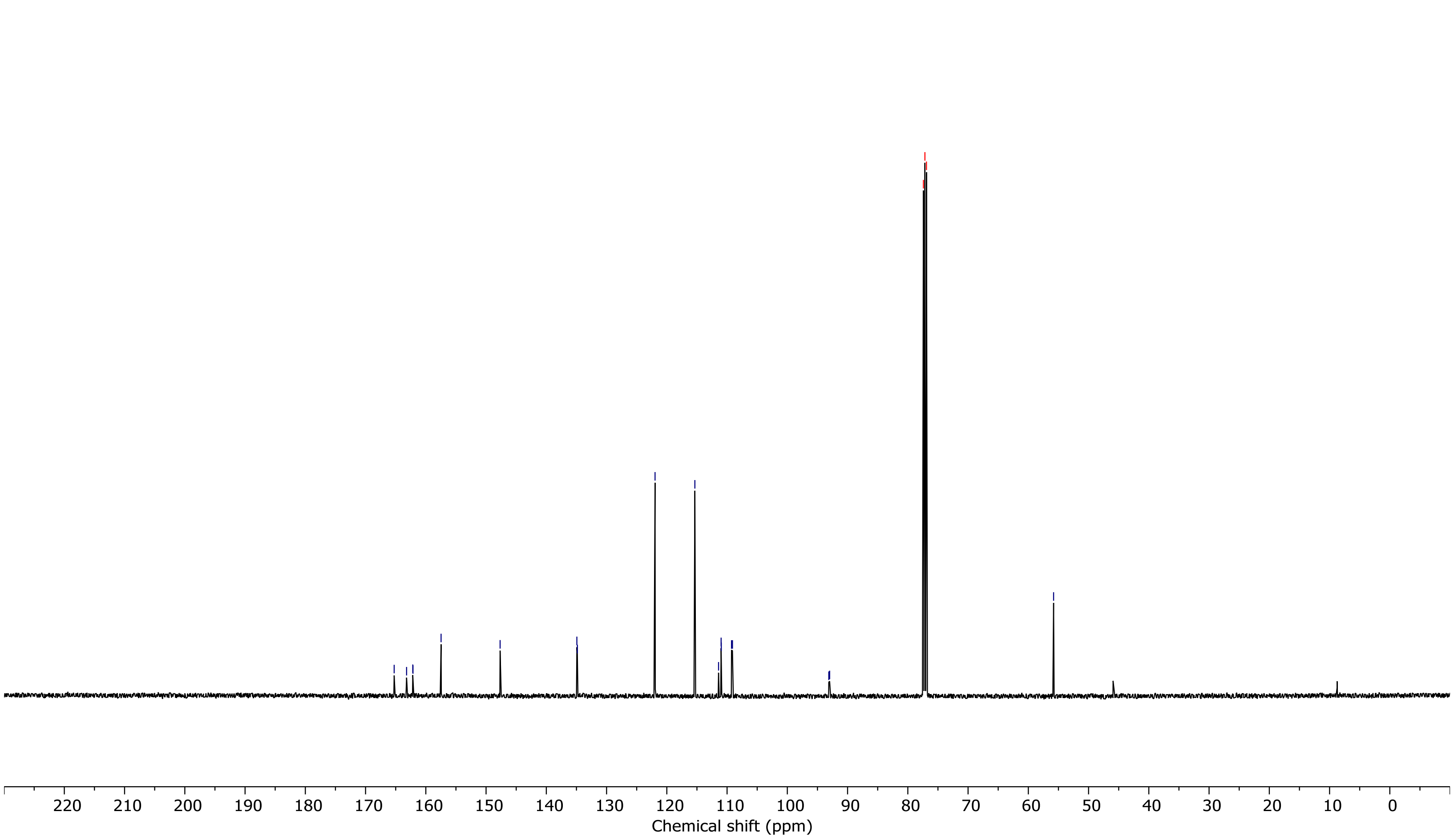

^{19}F NMR of 111

$CDCl_3$, 471 MHz, 23 ºC.

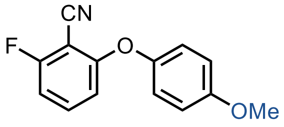

111

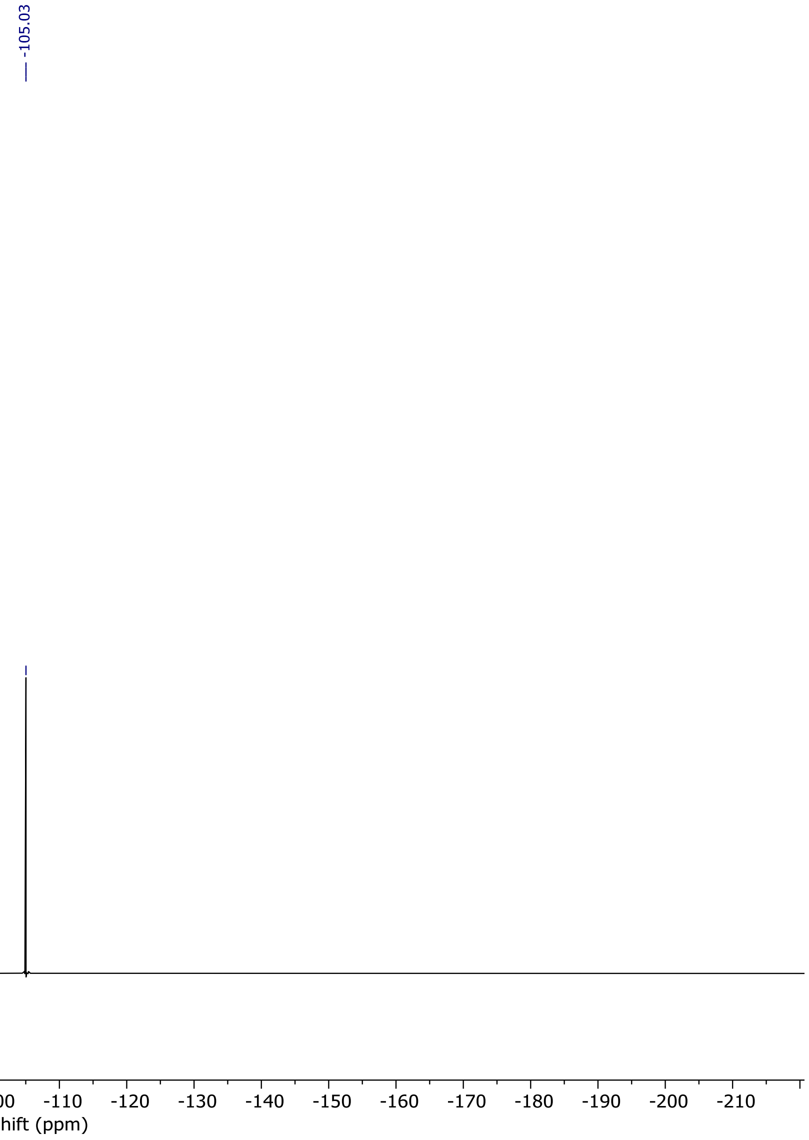

REFERENCES

1. Fulmer, G. R.; Miller, A. J. M.; Sherden, N. H.; Gottlieb, H. E.; Nudelman, A.; Stoltz, B. M.; Bercaw, J. E.; Goldberg, K. I., NMR Chemical Shifts of Trace Impurities: Common Laboratory Solvents, Organics, and Gases in Deuterated Solvents Relevant to the Organometallic Chemist. *Organometallics* **2010,** *29* (9), 2176–2179.
2. Ayotte, Y.; Woo, S.; LaPlante, S. R., Practical Considerations and Guidelines for Spectral Referencing for Fluorine NMR Ligand Screening. *ACS Omega* **2022,** *7* (15), 13155–13163.
3. Berger, F.; Plutschack, M. B.; Riegger, J.; Yu, W.; Speicher, S.; Ho, M.; Frank, N.; Ritter, T., Site-selective and versatile aromatic C−H functionalization by thianthrenation. *Nature* **2019,** *567* (7747), 223−228.
4. Serpier, F.; Pan, F.; Ham, W. S.; Jacq, J.; Genicot, C.; Ritter, T., Selective Methylation of Arenes: A Radical C−H Functionalization/Cross-Coupling Sequence. *Angew. Chem. Int. Ed.* **2018,** *130* (33), 10857–10861.
5. Turnu, F.; Luridiana, A.; Cocco, A.; Porcu, S.; Frongia, A.; Sarais, G.; Secci, F., Catalytic Tandem Friedel–Crafts Alkylation/C4–C3 Ring-Contraction Reaction: An Efficient Route for the Synthesis of Indolyl Cyclopropanecarbaldehydes and Ketones. *Org. Lett.* **2019,** *21* (18), 7329–7332.
6. Li, G.; Hu, J.; Zeng, R.; Shi, D.; Zhao, Y., Direct ortho-Selective C–H Functionalization of Carboxybenzyl-Protected Arylalkylamines via Ir(III)-Catalyzed C–H Activation. *Org. Lett.* **2018,** *20* (8), 2454–2458.
7. Börgel, J.; Tanwar, L.; Berger, F.; Ritter, T., Late-Stage Aromatic C–H Oxygenation. *J. Am. Chem. Soc.* **2018,** *140* (47), 16026–16031.
8. Mélin, L.; Abdullayev, S.; Fnaiche, A.; Vu, V.; González S., N.; Zeng, H.; Szewczyk, M. M.; Li, F.; Senisterra, G.; Allali-Hassani, A., Development of LM98, a Small-Molecule TEAD Inhibitor Derived from Flufenamic Acid. *ChemMedChem* **2021,** *16* (19), 2982–3002.
9. Criddle, D. N.; Meireles, A. V. P.; Macêdo, L. B.; Leal-Cardoso, J.; Scarparo, H. C.; Jaffar, M., Comparative inhibitory effects of niflumic acid and novel synthetic derivatives on the rat isolated stomach fundus. *J. Pharm. Pharmacol.* **2010,** *54* (2), 283–288.
10. Yan, F.; Bai, J.; Dong, Y.; Liu, S.; Li, C.; Du, C.; Li, Y., Catalytic Cyanation of C–N Bonds with CO_2/NH_3. *JACS Au* **2022,** *2* (11), 2522–2528.
11. Ravinder, B.; Rajeswar R., S.; Panasa R., A.; Bandichhor, R., Amide activation by TMSCl: reduction of amides to amines by $LiAlH_4$ under mild conditions. *Tetrahedron Lett.* **2013,** *54* (36), 4908–4913.
12. Ong, D. Y.; Yen, Z.; Yoshii, A.; Revillo I., J.; Takita, R.; Chiba, S., Controlled Reduction of Carboxamides to Alcohols

or Amines by Zinc Hydrides. *Angew. Chem. Int. Ed.* **2019,** *58* (15), 4992–4997.

13. Glennon, R. A.; Salley, J. J., Jr.; Steinsland, O. S.; Nelson, S., Synthesis and evaluation of novel alkylpiperazines as potential dopamine antagonists. *J. Med. Chem.* **1981,** *24* (6), 678–683.
14. Du, R.; Zhu, C.; Zhang, P.; Fan, R., Selective Hydrogenation of Aromatic Aminoketones by Pd/C Catalysis. *Synthetic Communications* **2008,** *38* (17), 2889-2897.
15. Shanahan, R. M.; Hickey, A.; Bateman, L. M.; Light, M. E.; McGlacken, G. P., One-Pot Cross-Coupling/C–H Functionalization Reactions: Quinoline as a Substrate and Ligand through N–Pd Interaction. *J. Org. Chem.* **2020,** *85* (4), 2585–2596.
16. Mastoor, S.; Faizi, S.; Saleem, R.; Siddiqui, B. S., NMR study of O and N, O-substituted 8-quinolinol derivatives. *Magn. Reson. Chem.* **2014,** *52* (3), 115–121.
17. Costanzo, P.; Cariati, L.; Desiderio, D.; Sgammato, R.; Lamberti, A.; Arcone, R.; Salerno, R.; Nardi, M.; Masullo, M.; Oliverio, M., Design, Synthesis, and Evaluation of Donepezil-Like Compounds as AChE and BACE-1 Inhibitors. *ACS Medicinal Chem. Lett.* **2016,** *7* (5), 470–475.
18. Suga, T.; Mizuno, H.; Takaya, J.; Iwasawa, N., Direct carboxylation of simple arenes with CO_2 through a rhodium-catalyzed C–H bond activation. *ChemComm.* **2014,** *50* (92), 14360–14363.
19. Ma, X.; Wang, S.; Tang, Z.; Huang, J.; Jia, T.; Zhao, X.; Zhao, D., Visible light-induced Mallory reaction of tertiary benzanilides via iminium intermediates. *Chem. Sci.* **2024,** *15* (41), 17210–17216.
20. Li, C.; Huang, Y.; Cao, S.; Luo, Y.; Zhang, Y.; Yang, G., A robust and facile method for desulfonation to amines. *Org. Chem. Front.* **2021,** *8* (22), 6182–6186.
21. Calderone, V.; Fiamingo, F. L.; Amato, G. I.; Livi, O.; Martelli, A.; Martinotti, E., New amido derivatives as potential BKCa potassium channel activators. XI. *Eur. J. Med. Chem.* **2008,** *43* (4), 792–799.
22. Ertl, P.; Rohde, B.; Selzer, P., Fast Calculation of Molecular Polar Surface Area as a Sum of Fragment-Based Contributions and Its Application to the Prediction of Drug Transport Properties. *J. Med. Chem.* **2000,** *43* (20), 3714–3717.
23. Chen, D.; Xu, L.; Ren, B.; Wang, Z.; Liu, C., Triflylpyridinium as Coupling Reagent for Rapid Amide and Ester Synthesis. *Org. Lett.* **2023,** *25* (24), 4571–4575.
24. Mrozowicz, M.; Chatterjee, S.; Mermigki, M. A.; Pantazis, D.; Ritter, T., Meta-Dimethylation of Arenes via Catellani Reaction from Aryl Thianthrenium Salts. *Angew. Chem. Int. Ed.* e202419472.
25. Sau, S.; Takizawa, S.; Kim, H. Y.; Oh, K., Visible Light-Induced Radical Cascade Functionalization of Quinoxalin-2(1H)-ones: Three-Component 1,2-Di(hetero)arylation Approach with Styrenes and Thianthrenium Salts. *Org.Lett.* **2024,** *26* (41), 8821–8826.
